# Clinical Relevance and Tumor Growth Suppression of Mitochondrial ROS Regulators along NADH:Ubiquinone Oxidoreductase Subunit B3 in Thyroid Cancer

**DOI:** 10.1155/2022/8038857

**Published:** 2022-01-17

**Authors:** Jiao Zhu, Xiaobo Zheng, Dan Lu, Yun Zheng, Jun Liu

**Affiliations:** ^1^Department of Otorhinolaryngology Head & Neck Surgery, West China Hospital, Sichuan University, Chengdu, China; ^2^Department of Critical Care Medicine, West China Hospital, Sichuan University, Chengdu, Sichuan, China

## Abstract

Mitochondrial reactive oxygen species (mitoROS) are a double-edged sword in cancer progression, connoting the ROS-dependent malignant transformation and the oxidative stress-induced cell death. However, the underlying role of mitoROS in thyroid cancer remains unclear. Here, we collected 35 prominent mitoROS regulators to stratify 510 thyroid cancer patients in TCGA cohort through consensus clustering. Three molecular subtypes (cluster 1/2/3) were identified, among which cluster 1 (mitoROS^low^) was preferentially associated with unfavorable prognosis. Individually, there were 12 regulators with a high expression that predicted a significantly favorable progression-free survival. The NADH:Ubiquinone Oxidoreductase Subunit B3 (NDUFB3) had a highest impact. NDUFB3 knockdown significantly reduced mitoROS levels in BCPAP and C643 cells. Bioinformatically, the consistency between NDUFB3 expression and cluster 1/2/3 was confirmed; lower expression of NUDFB3 was associated with a poor clinical outcome. Pathway analysis of differentially expressed genes in the NDUFB3^low^ and NDUFB3^high^ cohorts revealed a predominance of oxidative phosphorylation pathway changes. Consistently, mitochondrial functions, including oxygen consumption rate, ATP levels, complex I activity, mitoROS levels, and the expression of mitochondrially encoded NADH:Ubiquinone oxidoreductase core subunit 5, were significantly increased in NDUFB3-overexpressed BCPAP cells or C643 cells. The *in vivo* NDUFB3 overexpression and sideroxylin treatment significantly suppressed tumor growth and prolonged survival, concurrently elevating mitoROS levels ex vivo in mouse xenograft models. Conversely, NDUFB3 knockdown had the opposite effect. Together, these findings implicated the importance of mitoROS regulators in predicting clinical outcomes of patients with thyroid cancer. Our findings may pave the way for developing a mitoROS-based treatment for thyroid cancer patients.

## 1. Introduction

The incidence of thyroid cancer has rapidly increased in recent decades [1]. Histologically, differentiated thyroid cancer (DTC) has two subtypes: papillary thyroid cancer (PTC) and follicular thyroid cancer (FTC). DTC is the most frequent subtype of thyroid cancer, whereas the more rare subtypes are medullary thyroid cancer (MTC) and anaplastic thyroid cancer (ATC). Approved targeted treatments for DTC and ATC have prolonged progression-free survival (PFS); however, they are not curative and therefore reserved for patients with the progressive or symptomatic disease [2]. Thus, we must understand the molecular characteristics that can serve as potential prognostic indicators and drug targets.

Reactive oxygen species (ROS) are highly reactive oxygen-containing chemical species that have evolved as regulators of cancer signaling pathways. Mitochondria produce the majority of reactive oxygen species, named mitochondrial reactive oxygen species (mitoROS). An imbalance of mitoROS alters the intracellular redox homeostasis, triggers downstream pathways, and contributes to cancer development and progression [3]. The imbalance of ROS in thyroid cancer can be a risk factor for the clinical implication. Modulating ROS levels may reduce this risk and be therapeutically beneficial to thyroid cancer patients [4]. However, determining mitoROS or ROS levels in humans, especially the primary tumor location, is difficult to achieve to be the best of our knowledge. This problem can be addressed indirectly by determining the orchestrated expression levels of mitoROS regulators. Several ROS-associated gene sets have been collected from the Molecular Signatures Database (MSigDB), such as M5938 (hallmark reactive oxygen species pathway), but mitoROS regulators have not been completely defined. In the present study, we collected a mitoROS-related gene set including a total of 35 mitoROS regulators, each role of which can be classified as mitoROS generation or antioxidant networks. Although this gene set can be continuously updated or expanded, it can be used to determine mitoROS levels in thyroid cancer.

Generally, tumor cells have increased ROS levels compared with those normal cells [5]. As high ROS levels trigger apoptosis, treating cancer cells with doses of mitoROS-simulating agents may offer a cancer-specific therapy [5]. The effects of various kinds of antioxidants on tumor growth have yielded mixed results. For example, supplementation with N-acetyl-cysteine (NAC) or vitamin E accelerates tumor growth and increases mortality in a mouse model of K-Ras or B-Raf-driven lung cancer [6]. Some agents have an overall detrimental effect in clinical trials, increasing cancer-related mortality in patients [7]. These results indicate that the effects of general antioxidants are complex and require further deciphering of fundamental mechanisms. Antioxidant in clinical practice remains ineffective partly due to the variance in mitoROS levels across cancer types. Here, we first clarified the impact of mitoROS levels on the clinical prognosis of thyroid cancer before treatment with mitoROS modulators. We hypothesize that regulation of mitochondrial ROS based on the impact could suppress thyroid cancer growth.

## 2. Methods

### 2.1. Consensus Clustering and Clinical Information

Raw counts of RNA-sequencing data (level 3) and corresponding clinical information from 510 patients with thyroid cancer (Supplementary Table 1) were obtained from The Cancer Genome Atlas (TCGA) dataset (https://portal.gdc.cancer.gov/) in January 2020, in which the method of acquisition and application complied with the guidelines and policies. As these data were not collected from human biological specimens but were downloaded from TCGA database, which has already received ethics committee approval, this study did not require additional approval.

Using the R software package ConsensusClusterPlus (v1.54.0) for consistency analysis, the maximum number of clusters is 6, and 80% of the total sample is drawn 100 times, clusterAlg=“hc,” innerLinkage=“ward.D2.” The R software package pheatmap (v1.0.12) was used for clustering the heatmaps. The gene expression heatmap retained genes with an SD > 0.1. If the number of input genes is more than 1000, the top 25% of genes will be extracted after sorting the SD. All the above analysis methods and R package were implemented by the R Foundation for Statistical Computing (2020) version 4.0.3 and ggplot2 (v3.3.2). Statistical significance was set at *P* < 0.05. For more detailed information about TNM system, see https://www.cancer.gov/about-cancer/diagnosis-staging/staging.

### 2.2. PFS Analyses

For the comparison of the PFS among the three clusters after consensus clustering, the 510 thyroid cancer patients from The Cancer Genome Atlas (TCGA) database and tumoral RNA-seq data were downloaded from the Genomic Data Commons (GDC) data portal (TCGA). Statistical analyses were performed using the R software v4.0.3 (R Foundation for Statistical Computing, Vienna, Austria). Statistical significance was set at *P* < 0.05.

To assess the impact of each gene expression among 35 mitoROS regulators on PFS in thyroid cancer patients, raw counts of RNA-sequencing data (level 3) and corresponding clinical information from 510 patients with thyroid cancer were obtained from The Cancer Genome Atlas (TCGA) dataset (https://portal.gdc.cancer.gov/) in January 2020, in which the method of acquisition and application complied with the guidelines and policies. The Kaplan–Meier survival analysis with log-rank test was also used to compare the survival difference between the above two groups or more groups. TimeROC (v 0.4) analysis was performed to compare the predictive accuracy of each gene and the risk score. The least absolute shrinkage and selection operator (LASSO) regression algorithm for feature selection, using 10-fold cross-validation, uses the R software package glmnet (v 4.1-1).

For Kaplan–Meier curves, *P* values and hazard ratios (HR) with 95% confidence intervals (CIs) were generated by log-rank tests and univariate Cox proportional hazards regression. All analytical methods above and R packages were performed using the R software version 4.0.3 (the R Foundation for Statistical Computing, 2020). *P* < 0.05 was considered as statistically significant.

### 2.3. Cell Culture and Reagents

Human anaplastic thyroid carcinoma (C643 cells) and human papillary thyroid carcinoma (BCPAP cells) were a gift from the Zhu Lab (Department of Thyroid Surgery, West China Hospital). The two cell lines were cultured in T25 tissue culture flasks or 6 wells in RPMI-1640 medium (Gibco) supplemented with 10% fetal bovine serum (Gibco) and incubated in a humidified incubator containing 5% CO_2_ at the temperature of 37°C. Each cell line was authenticated using STR profiling and tested negative for mycoplasma contamination.

### 2.4. mitoROS Regulator Expression

To obtain the exact expression values of 35 mitoROS regulators in 510 thyroid cancer patients that were divided into three groups by consensus clustering, tumoral RNA-seq data were downloaded from the Genomic Data Commons (GDC) data portal (TCGA). All the above analysis methods and R package were implemented by the R Foundation for Statistical Computing (2020) version 4.0.3.

### 2.5. Differential Gene Expression Analysis

To investigate the possible signaling pathway caused by differential NUDFB3 expression, two groups were selected based on the NDUFB3 expression levels from the 510 thyroid cancer samples in TCGA cohort: higher (top 25%) and lower (bottom 25%) expression. Next, the limma package (version: 3.40.2) of the R software was used to study the differential expression of mRNAs. The adjusted *P* value was analyzed to correct for false-positive results in TCGA or GTEx. “Adjusted *P* < 0.05 and Log (Fold Change) > 1 or Log (Fold Change) < −1” were defined as the thresholds for the screening of differential expression of mRNAs.

To further confirm the underlying function of the potential targets, the data were analyzed using functional enrichment. Gene Ontology (GO) is a widely used tool for annotating genes with functions, especially molecular function (MF), biological pathways (BPs), and cellular components (CCs). Kyoto Encyclopedia of Genes and Genomes (KEGG) enrichment analysis is a practical resource for the analytical study of gene functions and associated high-level genome functional information. To better understand the carcinogenesis of mRNA, the ClusterProfiler package (version: 3.18.0) in R was employed to analyze the GO function of potential targets and enrich the KEGG pathway.

Gene set enrichment analysis (GSEA) was performed using the GSEA software (http://software.broadinstitute.org/gsea/index.jsp) and GSEA online tool (https://onlinetoolweb.com/gsea-online-tool) based on a matrix of all genes.

### 2.6. Western Blot

BCPAP cells or C643 cells were harvested and lysed with RIPA buffer (Millipore). Immunoblotting was performed in a standard fashion. The following antibodies were used: *β*-actin (1 : 1000; cat #AF5003; Beyotime, China), NDUFB3 (1 : 500; cat #SC-393351; Santa Cruz, CA), MT-ND1 (1 : 1000, cat #ab181848; Abcam), MT-ND2 (1 : 1000, cat #PA5-37185; Invitrogen), MT-ND3 (1 : 1000, cat #ab192306; Abcam), MT-ND4 (1 : 1000, cat #ab219822; Abcam), MT-ND4L (1 : 1000, cat #PA5-103953; Invitrogen), MT-ND5 (1 : 1000, cat #ab230509; Abcam), MT-ND6 (1 : 1000, cat #PA5-109993; Invitrogen), GPX1 (1 : 1000, cat #ab22604; Abcam), MnSOD (1 : 1000, cat #ab68155; Abcam), CuMnSOD (1 : 1000, cat #ab13498; Abcam), PRDX1 (1 : 1000, cat #NBP1-82558; Novus), PRDX3 (1 : 1000, cat #NBP2-67043; Novus), PRDX6 (1 : 1000, cat #H00009588-M01; Novus), and *α*-tubulin (1 : 1000; cat# AF0001; Beyotime, China).

### 2.7. qRT-PCR

Total RNA was isolated using TRIZOL reagent (Life technologies). The cDNA was collected using a PrimeScript RT reagent kit (TAKARA). Quantitative RT-PCR analysis was performed using a BIO-RAD CFX96 Real-Time PCR System (Bio-Rad) with SYBR Green qPCR Master Mix for all the target genes (TAKARA). Primers for ACTB were used as the internal controls. All primer sequences used are as shown in Supplementary Table 2.

### 2.8. Flow Cytometry

For mitoROS, BCPAP cells or C643 cells with 40%-50% confluence in 6 wells carrying NDUFB3 overexpression or knockdown were cultured for 48 h before trypsin digestion. In contrast, xenograft tumors were digested into a single-cell suspension for mitoROS-FACS analysis. The experiments for mitochondrial ROS or mitochondrial mass were carried out using MitoSOX or MitoTracker staining according to the manufacturer's protocol (Thermo Fisher, cat #M36008 and #M22425). Flow analysis was performed on a FACS LSR Fortessa (BD Biosciences), and the data were analyzed using the FlowJo 10.7 software.

### 2.9. Xenograft Models

We used two mouse models for the xenograft studies. For one of the xenograft models, 5 × 10^6^ BCPAP cells or C643 cells carrying NDUFB3 overexpression or control vector were xenografted into the subcutaneous region of NOD/SCID mice (NOD/SCID mice were purchased from the Nanjing Biomedical Research Institute of Nanjing University (Nanjing, China)). For another xenograft model, 5 × 10^6^ BCPAP cells and C643 cells were inoculated into the dorsal tissues of NOD/SCID mice. All mice were maintained under specific pathogen-free conditions at the animal facilities of Sichuan University. All animal experiments were approved by the Ethical and Animal Welfare Committee of Sichuan University.

All mice were randomly grouped. Eleven days postinoculation, mice bearing tumors were treated with sideroxylin (5 mg/kg; MCE; cat #HY-N1306) dissolved in 0.5% DMSO with 40% PEG300, 5% Tween 80, and normal saline or vehicle (0.5% DMSO, 40% PEG300, 5% Tween 80, and normal saline) via intraperitoneal injection every three days. Tumor volumes were monitored 3 times/week to observe dynamic developments in tumor growth in the two models five days postinoculation. The tumor volume was calculated using the following formula: 0.52 × length × width^2^. Mice with tumor volumes exceeding 3000 mm^3^ were euthanized. Then, the tumors were dissected out and further digested into a single-cell suspension for FACS analysis. For survival curves, tumor-bearing mice were euthanized at the endpoint. Mice with 25% weight loss were considered moribund and euthanized. The tumor-bearing mice in the treatment groups were analyzed using the Mantel-Cox log-rank test.

### 2.10. Plasmid and Lentivirus Production

Plasmids encoding for scrambled shRNA and NDUFB3 shRNA were purchased from HANBiO (http://www.hanbio.net). The shRNA sequence targeting human NDUFB3 was 5′-UGGCU UUGCA AAGAG UGUUU C-3′. For NDUFB3 overexpression, the full-length CDS of human NUDFB3 was inserted into pWPXLd vector (Addgene #12258). Lentivirus was packaged in 293T cells using psPAX2 and VsvG, followed by viral transduction to BCPAP cells or C643 cells with 5 *μ*g/*μ*l polybrene (HANBiO).

### 2.11. ATP Determination

The ATP concentrations were determined using an ATP Determination Kit (Molecular Probes, cat #A22066). For the kit, a standard curve was drawn and the ATP concentration of all samples to be tested was normalized to the total protein content evaluated by the Bradford assay (Beyotime, cat #P0006).

### 2.12. Extracellular Flux Analysis

OCR measurements were performed using the Mito Stress kit (Seahorse, Agilent, cat #103015-100) with 2 *μ*M oligomycin, 2 *μ*M FCCP, and 0.5 *μ*M Retenone/Antimycin A in an XF24 Extracellular Flux Analyzer (Seahorse, Agilent). Briefly, each group of cells was seeded in an XF culture dish at 12000 cells per pore one day before the test. On the day of testing, the pretreated medium was used to replace the previous medium quickly and put into the machine for testing.

### 2.13. Complex I Activity

Complex I activity was determined using the mitochondrial complex I activity colorimetric assay kit (BioVision, cat #K968). Specifically, mitochondria were isolated from BCPAP cells and C643 cells (Miltenyi Biotec, cat #130-094-532); then, a 5 *μ*g sample was reacted with complex I assay buffer, decylubiquinone, and complex I dye, with or without rotenone addition. OD600 was measured in kinetic mode at 30 s intervals for up to 5 min at room temperature. Mitochondrial complex I activity was calculated using a standard curve.

### 2.14. Mitochondrial DNA Copy Number

Mitochondrial DNA copy number was quantified using the Human mitochondrial to nuclear DNA ratio kit (Takara, cat #7246) according to the manufacturer's instructions and was then calculated relative to genomic DNA copy number using the 2-*ΔΔ*Ct method.

### 2.15. Determination Content of GSH

BCPAP cells or C643 cells were treated with or without L-BSO (50 *μ*M; MedChemExpress, cat #HY-106376A) for 48 h before collection. GSH detection was performed according to the manufacturer's instructions (Beyotime, cat #S0053). Briefly, cells were washed with 1× PBS and collected, resuspended in 3 times the volume of protein removal reagent M solution. Cell samples were treated with two rapid freeze–thaw cycles using liquid nitrogen and 37°C water bath. The corresponding detection reagents were added to an appropriate amount of the cell sample. After 25 min, GSH was detected using a microplate analyzer at an absorbance of 412 nm. The GSH content was calculated according to the standard curve.

### 2.16. Determination Activity of Catalase

BCPAP cells or C643 cells were treated with or without EGCG (50 *μ*M; MedChemExpress, cat #HY-13653) for 24 h before collection. Catalase activity was determined according to the manufacturer's instructions (Beyotime, cat #S0051). Briefly, cells were lysed in lysis buffer (20 mM Tris–HCl, pH 7.4, 0.5% NP-40, 250 mM NaCl, 3 mM EGTA, and 3 mM EDTA) supplemented with protease inhibitors (Beyotime, cat #P1049) and incubated on ice for 30 min. The cell lysis samples were mixed with the corresponding test solutions. After 20 min, catalase activity was detected using a microplate analyzer at an absorbance of 240 nm.

### 2.17. Statistical Analysis

All data are presented as the mean ± standard deviation. Two-tailed unpaired Mann–Whitney *t*-tests were used to compare the values between the two groups. Comparisons of the values obtained with three or more groups were analyzed using two-way analysis of variance, and Bonferroni post hoc test was used to determine these differences using the GraphPad software 8.0. Survival analyses were performed by drawing Kaplan–Meier curves, and the differences between subgroups were analyzed using the log-rank test. *P* < 0.05 was considered to indicate a statistically significant difference.

## 3. Results

### 3.1. mitoROS Regulators Are Consistent with Clinical Features of Thyroid Cancers

**Figure 1:**
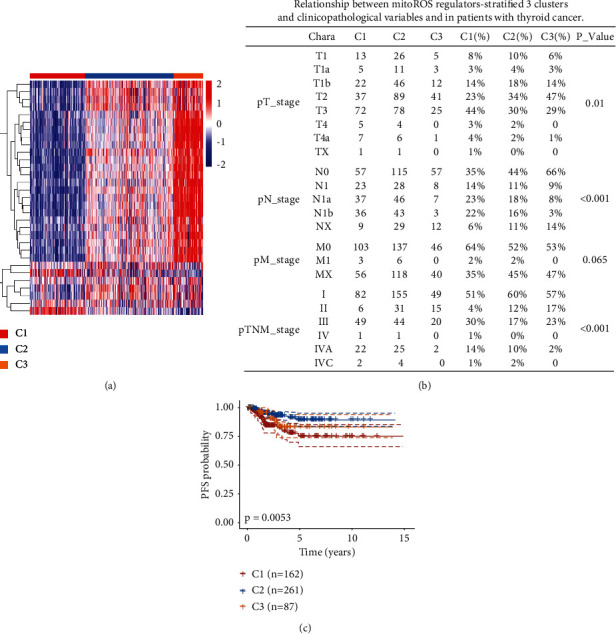
mitoROS regulators are associated with clinical outcomes in thyroid cancer. (a) Heatmap depicting consensus clustering solution (*k* = 3) for 35 mitoROS regulators in 510 thyroid cancer samples; red represents high expression, and blue represents low expression. (b) Relationship between mitoROS regulator-stratified 3 clusters and clinicopathological variables and in thyroid cancer patients. The unpaired *t*-test was used to analyze statistical assessments. The association between mitoROS levels and clinical characteristic variables was analyzed using the Pearson chi-squared test or Fisher's exact test. (c) Kaplan–Meier survival analysis of the three groups based on mitoROS regulator-dependent consensus clustering. PFS analysis was statistically evaluated using the log-rank test.

mitoROS play a critical role in tumorigenesis and are integral to the regulation of diverse signaling networks that drive tumor cell proliferation, malignant progression, and survival [3]. Therefore, we collected a mitoROS-related gene subset as a self-defined gene set containing 35 genes to stratify thyroid cancers (Supplementary Table 3). To obtain clinical information from patients with thyroid cancer, we downloaded omics datasets generated by TCGA public database. The following inclusion criteria were used: (1) simultaneously available information on mRNA expression values and PFS and (2) histologically confirmed 510 thyroid cancers containing 395 patients with papillary thyroid cancer and 106 patients with follicular thyroid cancer plus 9 patients with undetermined thyroid cancer. Lastly, the patients with the corresponding clinicopathological information, including age, gender, race, TNM stage, and grade, were enrolled for further analysis. Based on the relative mRNA expression levels of the patients with thyroid cancers in TCGA datasets, the clustering performance of each *k* value displayed the corresponding similarity by a proportion of ambiguous clustering as shown in Supplementary Figure 1A-1H. To better reflect the effect of mitoROS on the clinical prognosis of thyroid cancer in the follow-up analysis, the *k* = 3 was identified with optimal clustering stability from *k* = 2 to 6 (Figure 1(a)). Thus, the 510 samples were clustered into three subtypes, namely, cluster 1 (mitoROS^low^; C1; *n* = 162), cluster 2 (mitoROS^Int^; C2; *n* = 261), and cluster 3 (mitoROS^high^; C3; *n* = 87) (Supplementary Table 1). Next, we compared the clinicopathological features of the three groups (Supplementary Table 4). C1 was preferentially associated with a higher percentage of patients in the advanced stage of the TNM system (Figure 1(b)). Consistently, the PFS probability of the C1 was lower than that of the C2 and C3 (Figure 1(c) and Supplementary Table 5). Notably, C2 (mitoROS^Int^) had the highest survival rate among the three, whereas the PFS probability of C3 (mitoROS^high^) was situated between C1 and C2, indicating that moderate mitoROS level was associated with a favorable prognosis than both higher and lower levels; however, lower mitoROS levels were relatively common features of adverse prognosis in thyroid cancer. Together, these findings suggest that clustering subtypes defined by mitoROS regulators may be closely related to the clinical significance of patients with thyroid cancer.

### 3.2. High Expression of mitoROS Regulators Predicts Better Patient Outcomes

**Figure 2:**
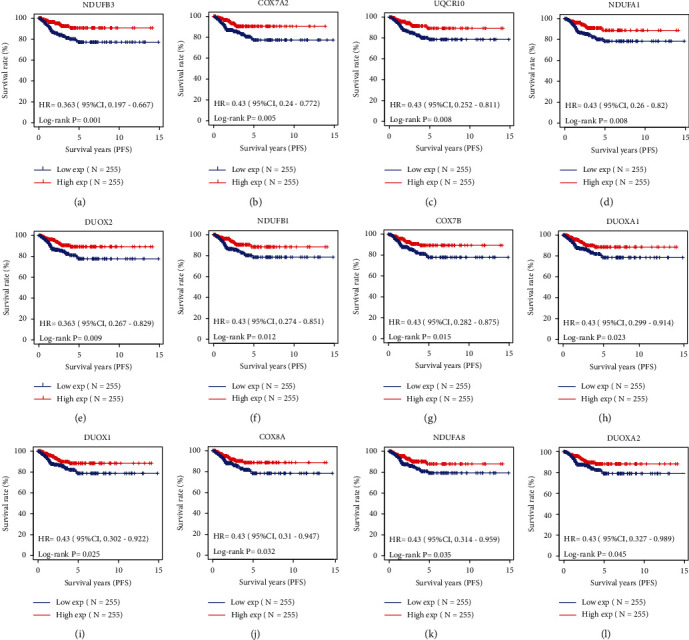
Higher expression of mitoROS regulators has significantly better PFS. (a–l) Kaplan–Meier survival analysis of 12 mitoROS regulators. The cutoff point was defined as the median baseline value. The order from (a) to (l) is from small to large according to the *P* value (*P* value is less than 0.05). PFS analysis was statistically evaluated using the log-rank test.

We then analyzed the relationship between each mitoROS regulator expression and survival of the 510 patients with the thyroid cancer. Thereafter, patients were grouped into “high” (*n* = 255) or “low” (*n* = 255) signature expression groups based on the median expression of each regulator. PFS Kaplan–Meier curves for each regulator in patients with thyroid cancer are shown; *P* values were calculated by log-rank tests comparing the two Kaplan–Meier curves. Not every regulator caused a significant difference in PFS (Supplementary Table 6). Twelve regulators among of them significantly predicted better PFS probability (Figures 2(a)–2(l)), including NADH:Ubiquinone Oxidoreductase Subunit B3 (NDUFB3), Cytochrome C Oxidase Subunit 7A2 (COX7A2), Ubiquinol-Cytochrome C Reductase (UQCR10), NADH:Ubiquinone Oxidoreductase Subunit A1 (NDUFA1), Dual Oxidase 2 (DUOX2), NADH:Ubiquinone Oxidoreductase Subunit B1 (NDUFB1), Cytochrome C Oxidase Subunit 7B (COX7B), Dual Oxidase Maturation Factor 1 (DUOXA1), Dual Oxidase 1 (DUOX1), Cytochrome C Oxidase Subunit 8A (COX8A), NADH:Ubiquinone Oxidoreductase Subunit A8 (NDUFA8), and Dual Oxidase Maturation Factor 2 (DUOXA2). These data suggest the possibility that modulating one of these regulators might slow cell growth or tumor progression in thyroid cancers.

### 3.3. NDUFB3 Correlates with mitoROS Level and Clinical Features in Thyroid Cancers

**Figure 3:**
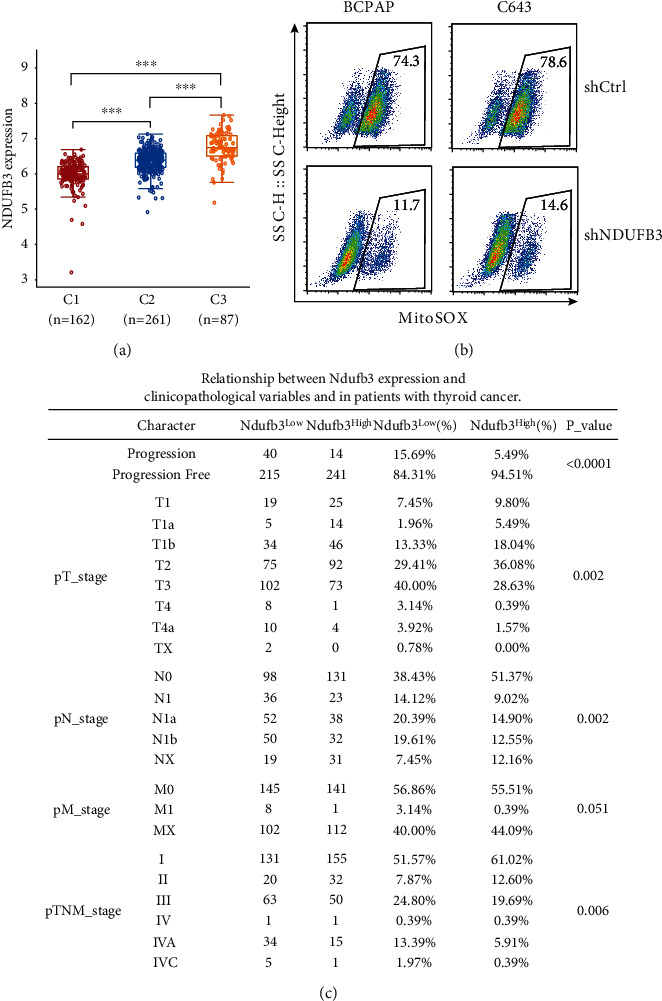
The mitoROS and clinicopathological relevance of NDUFB3 expression in thyroid tumors. (a) The expression distribution of NDUFB3 gene in the three indicated clusters based on mitoROS regulator-dependent consensus clustering. Each data point in a dot plot represents a single patient. Box plots depict the median (horizontal line in each box), the 25th percentile (bottom of each box), and the 75th percentile (top of each box). NDUFB3 gene expression was analyzed for significance using the unpaired Student *t*-test. (b) Representative flow cytometry analysis of NDUFB3 shRNA and control shRNA showing respective mitoROS levels in BCPAP and C643 cells. (c) Relationship between NDUFB3 expression and clinicopathological variables and in patients with thyroid cancer. The unpaired *t*-test was used to analyze statistical assessments. The association between NDUFB3 expression and clinical characteristic variables was analyzed using the Pearson chi-squared test or Fisher's exact test.

Notably, NDUFB3, which is reported as an accessory subunit of the mitochondrial complex I [8], ranks #1 according to log-rank *P* values in the list (Supplementary Table 6). Consequently, we attempted to preliminarily investigate the connections between NDUFB3 expression and mitoROS levels, as well as clinicopathological features in thyroid cancer. The NDUFB3 expression along with that of the other regulators was investigated in the clusters grouped by the three mitoROS regulators (Supplementary Table 7). We found that NDUFB3 expression progressively increased from C1 to C3 (Figure 3(a)), and that the cases grouped by NDUFB3 expression overlapped well with the cluster 1/2/3 (Supplementary Figure 2). This revealed that the NDUFB3-expressing population represented the population of mitoROS regulators in thyroid cancers, and that its expression may be positively correlated with mitoROS levels. This scenario was validated experimentally. We performed lentivirus-mediated shRNA knockdown targeting human NDUFB3 in both BCPAP (papillary thyroid cancer cells) and C643 (anaplastic thyroid cancer cells) cell lines. NDUFB3 protein expression levels upon knockdown were confirmed using western blotting (Supplementary Figure 3). The mitoSOX assay confirmed diminished mitoROS generation in NDUFB3 knockdown thyroid cancer cells (Figure 3(b)).

To corroborate whether NDUFB3 was the only subunit of complex I that changes in NDUFB3-knocked down cells, we determined the gene or protein expressions of the 45 subunits included in the human mitochondrial complex I [9–11]. The results showed that there were no significant changes for either the nuclear or mitochondrial DNA-encoded genes at the transcriptional or protein levels, respectively, upon NDUFB3 knockdown (Supplementary Figure 4A-4B). Except for MT-ND5, an antiporter-like subunit with four transmembrane helices bound to NDUFB3 along with NDUFB6, NDUFB2, and NDUFB8 [12, 13], the expression of which is significantly downregulated in cells with NDUFB3 knockdown. Thus, this regulation suggests that NDUFB3 may depend on MT-ND5 to indirectly affect mitoROS generation, or that NDUFB3 affects mitoROS generation in both direct and NDFUB3/MT-ND5 axis pathways.

We next investigated the relationship between NDUFB3 expression and the clinicopathological characteristics of the 510 patients with thyroid tumors, as listed in Supplementary Table 8. Specifically, low expression of NDUFB3 (top 50%) was generally associated with a higher percentage of advanced TNM stage (Figure 3(c)), highlighting that NDUFB3 could be used as a prognostic biomarker for thyroid cancers and implying the possibility that highly expressed NDUFB3 can hinder thyroid cancer progression.

### 3.4. NDUFB3 Promotes Mitochondrial Respiration and mitoROS Generation

**Figure 4:**
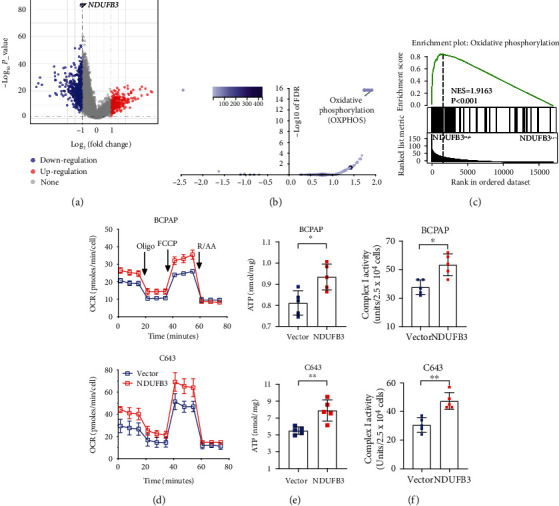
The role of NDUFB3 overexpression in mitochondrial respiration and mitoROS generation. (a) Volcano plots were constructed using fold-change values and adjusted *P* (NDUFB3^low^ versus NDUFB3^high^). The red point in the plot represents the overexpressed mRNAs, and the blue point indicates the downregulated mRNAs with statistical significance. The plot of NDUFB3 is shown on the top, as indicated. (b) Volcano plots were constructed using enrichment score and FDR. The number of genes enriched in each KEGG pathway is shown in the top-left corner. In the enrichment result, *P* < 0.05 or FDR < 0.05 is considered to be enriched to a meaningful pathway. (c) GSEA histogram for the gene set “OXPHOS.” Real-time OCR tracing of BCPAP cells or C643 cells stably overexpressing NDUFB3 or empty vector control (*n* = 3). (d) Total cellular ATP concentration in BCPAP cells or C643 cells stably overexpressing NDUFB3 or empty vector control (*n* = 5). Statistical differences were determined by a two-tailed unpaired Mann–Whitney *t*-test. ^∗^*P* < 0.05; ^∗∗^*P* < 0.01. (e) Complex I activity was determined in mitochondria isolated from BCPAP cells or C643 cells overexpressing NDFUB3 or empty vector control (*n* = 5). Statistical differences were determined using a two-tailed unpaired Mann–Whitney *t*-test. ^∗^*P* < 0.05; ^∗∗^*P* < 0.01. (f) Representative flow cytometry analysis of NDUFB3 overexpression or its empty vector control showing respective mitoROS levels in BCPAP or C643 cells.

Given the critical role of NDUFB3 in mitoROS and thyroid cancer clinicopathology, we sought to investigate the possible signaling pathway caused by its differential expression in thyroid cancers. The next two groups were selected based on the NDUFB3 expression levels from the 510 thyroid cancer samples in TCGA cohort: higher (top 25%) and lower (bottom 25%) expression. Hierarchical clustering demonstrated that the distinct clustering of the two population separated by NDUFB3 expression segregates from each other (*n* = 127; Supplementary Figure 5). The volcano plot showed gene expression changes by comparing the two groups, 247 upregulated genes and 418 downregulated genes (NDUFB3^low^ versus NDUFB3^high^, *P* < 0.05; log_2_FC > 1 or log_2_FC < −1; Figure 4(a) and supplementary Table 9). The three GO categories, biological process (GO-BP), molecular function (GO-MF), and cellular component (GO-CC), were tested independently. GO-CC analyses revealed that the markedly regulated genes were focused on mitochondrion (Supplementary Figure 6). GSEA with the KEGG pathway gene sets revealed enrichment of differentially expressed genes containing 18 significantly changed signaling pathways (Figure 4(b), Supplementary Figure 7, and supplementary Table 10). Strikingly, the top-ranking gene set in this enrichment analysis was mitochondrial oxidative phosphorylation (OXPHOS) according to NES (NES = 1.9163; Figures 4(b) and 4(c) and supplementary Table 11). Mitochondrial OXPHOS sustains organelle function and plays a central role in aerobic respiration [14]. The mitochondrial localization of NDUFB3 was roughly confirmed in BCPAP cells using MitoTracker staining (Supplementary Figure 8), consistent with previous GO-CC analyses.

Based on the above findings, we hypothesized that overexpression of NDUFB3 may cause favorable mitochondrial functions. Thus, we infected thyroid cancer cells with lentivirus encoding a human NDFUB3 cDNA or a control noncoding lentivirus and confirmed the overexpression of NDUFB3 using western blotting (Supplementary Figure 9). Consistent with the NDUFB3 knockdown, further investigation of human complex I subunits showed that no significant changes were observed for the nuclear and mitochondrial DNA-encoded genes upon overexpressing NDUFB3, except for MT-ND5 (Supplementary Figure 4C-4D). This further validated the critical role of NDUFB3 in the regulation of MT-ND5 expression. We then measured the respiratory capacity of BCPAP cells and C643 cells overexpressing human NDUFB3 using the seahorse system. Seahorse curves showed that the basal and maximum oxygen consumption rates (OCRs) were increased in the NDUFB3-overexpressed tumor cells (Figure 4(d)). Although no significant difference was detected in mitochondrial DNA copy number and mitochondrial mass (Supplementary Figure 10), overexpression of NUDFB3 significantly increased ATP production, complex I activity, and mitoROS levels in BCPAP and C643 cells (Figures 4(e) and 4(f)). Thus, NDUFB3 expression partially enhanced mitochondrial respiration and increased mitoROS production.

ROS levels depend on ROS producers and ROS consumers. Next, we examined cells that overexpressed NDUFB3 what happens with antioxidant mechanisms. The mitoROS levels were tightly controlled by robust scavenger antioxidant enzymes including MnSOD (Mn Superoxide Dismutase, also named SOD2), CuZnSOD (Cu/Zn Superoxide Dismutase, also named SOD1), GPX1 (Glutathione Peroxidase 1, GPX1), and PRDX (Peroxiredoxin) [15–18]. The immunoblots of these antioxidant enzymes showed that overexpression of NDUFB3 remarkably increased the protein levels of MnSOD, but not those of GPX1 or other PRDX family members including PRDX1, PRDX3, and PRDX6 (Supplementary Figure 11A). Further investigation of MnSOD at the transcriptional level demonstrated that overexpression of NDUFB3 exerted no significant effect on the mRNA expression of MnSOD (Supplementary Figure 11B), suggesting that NUDFB3 regulates MnSOD at the protein level. We assessed the two main cellular antioxidants, GSH levels, and catalase activity. The results showed that overexpression of NDUFB3 exerted significant effect on neither the level of GSH nor the activity of catalase in both BCPAP and C643 cells (Supplementary Figure 11C-11D). These findings highlighted a critical antioxidant role of MnSOD during the increase of mitoROS derived from overexpression of NDUFB3 in the thyroid cancer cells, as expected. Based on the cellular localization of NUDFB3, mitoROS upon its overexpression was generated mainly from complex I, which produces superoxide in the mitochondrial matrix. Alternatively, complex III produces and directs it to the matrix and intermembrane space at approximately equal rates under deenergized conditions [19]. In relation, MnSOD converts the superoxide radical to hydrogen peroxide in the mitochondrial matrix, whereas CuZnSOD converts superoxide radicals into the intermembrane space or cytosol [20]. Therefore, NDUFB3-overexpressed cells may depend on MnSOD as a ROS consumer to maintain redox balance.

### 3.5. NDUFB3 and Sideroxylin Limit *In Vivo* Thyroid Cancer Growth

**Figure 5:**
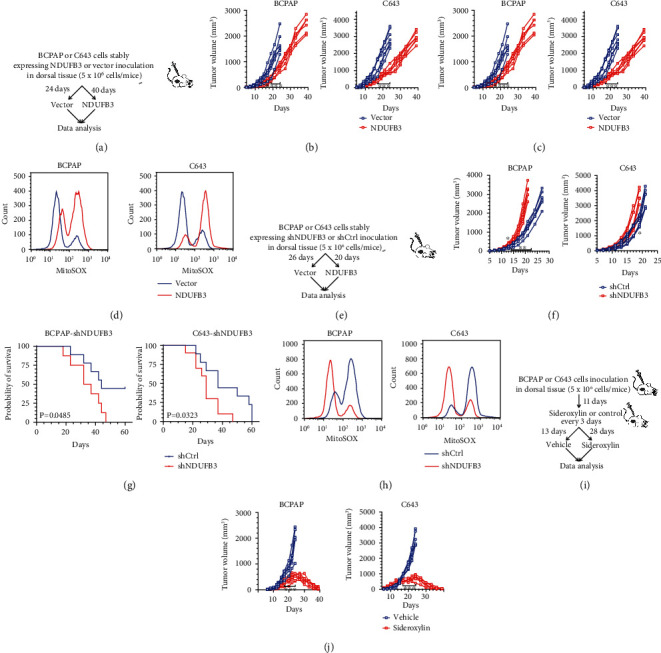
Overexpression of NDUFB3 or sideroxylin suppresses thyroid tumor growth in vivo. (a) A sketch map showing mice bearing thyroid tumors derived from BCPAP or C643 cells stably overexpressing NDUFB3 or its empty control vector in dorsal tissues. (b) Tumor volumes over the indicated times were analyzed. Mice were euthanized as their tumor volumes reached approximately 3000 mm^3^. Each line represents a mouse, and each plot on the line represents a different time point. *P* values represent two-way ANOVA and post hoc Tuckey's tests for tumor growth. ^∗^*P* < 0.05; ^∗∗^*P* < 0.01; ^∗∗∗^*P* < 0.001. (c) Survival curves of tumor-bearing mice overexpressing NDUFB3 or empty vector controls. Analysis was statistically evaluated using the log-rank test. (d) Representative flow cytometry analysis of single-cell suspensions from resected tumor masses (vector versus NDUFB3). (e) A sketch map showing mice bearing thyroid tumors derived from BCPAP or C643 cells stably expressing shRNA against NDUFB3 or its empty control vector in dorsal tissues. (d) Tumor volumes over the indicated times were analyzed. Mice were euthanized as their tumor volumes reached approximately 3000 mm^3^. Each line represents a mouse, and each plot on the line represents a different time point. *P* values represent two-way ANOVA and post hoc Tuckey's tests for tumor growth. ^∗^*P* < 0.05; ^∗∗^*P* < 0.01. (e) The survival curves of tumor-bearing mice with NDUFB3 knockdown or its empty vector controls. Analysis was statistically evaluated using the log-rank test. (f) Representative flow cytometry analysis of single-cell suspensions from resected tumor masses (shNDUFB3 versus shCtrl). (g) A sketch map showing that mice bearing thyroid tumors were treated with sideroxylin or vehicle after BCPAP cells or C643 cells were inoculated into the dorsal tissues of mice. (h) Tumor volumes over the indicated times were analyzed. Mice were euthanized as their tumor volumes reached approximately 3000 mm^3^. Each line represents a mouse, and each plot on the line represents a different time point. *P* values represent two-way ANOVA and post hoc Tuckey's tests for tumor growth. ^∗^*P* < 0.05; ^∗∗^*P* < 0.01; ^∗∗∗^*P* < 0.001. (i) Survival curves of tumor-bearing mice treated with sideroxylin or vehicle. Analysis was statistically evaluated using the log-rank test. (j) Representative flow cytometry analysis of single-cell suspensions from resected tumor masses (vehicle versus sideroxylin).

We subsequently hypothesized that NDUFB3 overexpression would be capable of slowing tumor growth *in vivo*. In a mouse xenograft model (Figure 5(a)), the tumor volumes in the NDUFB3-overexpressed tumor cells were delayed for approximately 10 days (Figure 5(b)). The survival rate was also significantly prolonged in the NDUFB3 overexpression group (Figure 5(c)). To determine mitoROS levels, tumor-derived single-cell suspensions were stained with mitoSOX. The results showed that NDUFB3 increased ex vivo mitoROS levels (Figure 5(d)). In contrast, when the tumor cells with NDUFB3 knockdown were inoculated in mice (Figure 5(e)), significantly accelerated tumor growth and shortened survival times were observed compared to mice in the empty vector group (Figures 5(f) and 5(g)). Consistent with the *in vitro* experiment, *ex vivo* mitoSOX staining of these tumor cells showed a diminished mitoROS level in NDUFB3 knockdown tumor cells (Figure 5(h)). Together, these data confirmed the relationship between low NDUFB3 expression and its association with a higher percentage of advanced TNM stage (Figure 3(c)), as well as highlight the negative regulatory role of NDUFB3 in tumor growth and the potential involvement of mitoROS in NDUFB3-modulated tumor growth.

Next, we assessed whether there was a mitoROS inducer that specifically augmented mitoROS production could diminish tumor growth and showcase an inhibitory effect on tumor growth that exceeded NDUFB3 overexpression. Sideroxylin suppresses ovarian tumor cell proliferation accompanied by mitochondrial dysfunction and ROS generation [21]. We hypothesized that sideroxylin exerts antitumor effects. Using a BCPAP or C643-bearing mouse model (Figure 5(i)), we found that intraperitoneal injection of sideroxylin effectively suppressed tumor growth and prolonged the survival of the mice (Figures 5(j) and 5(k)). We also noted that the time of statistically significant difference in tumor volume between the sideroxylin and control groups was from day 17. At the end of 39 days, *in vivo* tumor growth was almost eliminated. Furthermore, we determined mitoROS levels in tumor-derived single-cell suspensions using mitoSOX dye after sideroxylin treatment. The results showed that sideroxylin-treated cells had higher mitoROS levels than the control cells (Figure 5(l)). Together, these findings uncovered an important clue that increasing mitoROS levels by sideroxylin treatment or NDUFB3 overexpression could effectively suppress thyroid cancer growth.

## 4. Discussion

The current consensus is that mitoROS amplify the tumorigenic phenotype and accelerate the accumulation of additional mutations that lead to proliferative or metastatic behaviors in cancer cells [3]. However, it is worth noting that ROS might act as a double-edged sword [22], and mitoROS is no exception. At low concentrations, ROS promote cancer cell survival by activating growth factors and mitogen-activated protein kinases (MAPKs) that further activate cell cycle progression. At high concentrations, ROS produce oxidative stress that activates programmed cell death or apoptosis [23]. Here, we recognized that this occurs in thyroid cancer. Our analysis based on 510 patients with thyroid cancer in TCGA data suggests that moderate mitoROS was beneficial to PFS in thyroid cancer patients. However, the patients with the most adverse survival were those with low levels of mitoROS (Figure 1(c)). In this sense, our study emphasized the association between mitoROS levels and tumor type, helpful in developing treatment strategies based on regulating mitoROS levels. An important limitation in our study was the lack of the correlation between mitoROS levels and the pathological subtypes of thyroid cancer.

NDUFB3 is a nuclear-encoded complex I subunit gene. Mutations in this gene cause complex I deficiency and lethal infantile mitochondrial disease [24]. A recurrent homozygous NDUFB3 mutation (c.64T>C, p.Trp22Arg) was associated with markers of mitochondrial dysfunction, short stature, and distinctive facial appearance, including a prominent forehead, smooth philtrum, and deep-set eyes [25]. NDUFB3 homozygous pathogenic variant was also found in a patient with a mitochondrial disorder and with a prior diagnosis of nonalcoholic steatohepatitis [26]. NDUFB3 may play an important role in promoting the pathological process of recurrent pregnancy loss (RPL), as NDUFB3 was significantly increased in decidual cells from RPL patients, and overexpression of NDUFB3 decreased the mitochondrial membrane potential and inhibited cell vitality and oxidative stress in decimal cells [27]. *In vitro* knockdown of NDUFB3 in nasopharyngeal carcinoma cells exhibited significant reductions in cisplatin-induced mitoROS production [28], demonstrating that it positively correlated with mitoROS production consistent with our results. In addition, NDUFB3 mRNA is decreased in healthy young men fed a high-fat diet (HFD) and in mice with HFD [29]. However, in the field of tumor pathology, NDUFB3 has only been associated with nasopharyngeal carcinoma. Its function and role in other tumor types remain unknown. Our study showed that a prosurvival function following NDUFB3 overexpression in thyroid cancer cell-derived xenograft tumors can be observed. These findings suggest that high expression of mitoROS regulators along NDUFB3 could be a promising therapeutic target in patients with thyroid cancers. Future studies will explore the roles of the other 11 regulators shown in Figure 2 in the prognosis and therapy of thyroid cancer.

We also showed that NDUFB3 in thyroid cancer cells upregulated MT-ND5 subunit, increased complex I activity and ATP levels, enhanced mitoROS generation, and improved mitochondrial respiration, despite no changes in mitochondrial number (Figures 4(d)–4(g); Supplementary Figures 4 and Figure 10). These data promoted us to investigate how NDUFB3 improves mitochondrial function. NDUFB3 is methylated by METTL9, and that the latter's enzymatic activity promotes complex I-mediated respiration as NDUFB3 contains several methylhistidines within a stretch of alternating histidines in its N-terminal region [8]. In terms of the enhanced mitochondrial functions by overexpression of NDUFB3, combining our data with the report can provide a hypothetic explanation that in the tumor microenvironment or upon endogenous stimuli, NDUFB3 methylated by METTL9 actively enhances complex I activity by upregulating MT-ND5 subunits, thus increasing ATP levels, enhancing mitoROS generation, and improving mitochondrial function. This hypothesis warrants further study in the future. Notably, the range of the improved mitochondrial functions was limited such as the unchanged mitochondrial number and the 43 unvaried complex I subunits in our study (Supplement Figures 4 and 10). In addition, a 4.2 Å resolution single-particle electron cryomicroscopy demonstrated that NDUFB3, along with other 17 supernumerary transmembrane helices (TMHs), established a cage around the core membrane domain in complex I from *Bos taurus*. Among them, NDUFB3, NDUFB6, NDUFB2, and NDUFB8 are bound to MT-ND5 [12]. Knowledge of the structures of the complex I further implies that the effect of NDUFB3 on the improvement of mitochondrial functions may be due to the NDUFB3-MT-ND5 binding pattern in complex I. However, additional investigation is required to validate these hypotheses and elucidate the molecular mechanisms involved in the improvement.

Sideroxylin, a C-methylated flavone, is a novel therapeutic agent used to combat the proliferation of ovarian cancer cells through the induction of mitochondrial dysfunction, including an increase in ROS generation and the activation of PI3K and MAPK signal transduction [21]. Sideroxylin also showed an antiproliferative effect against MDA-MB-231 and MCF-7 cell lines with IC50 values of 36.9 and 14.7 *μ*M, respectively [30]. In addition, sideroxylin exhibited potent anti-inflammatory activity by downregulating nitric oxide and TNF-*α* production, suppressing phosphorylation of ERK, c-Jun, limiting the phosphorylation of STAT-1 and STAT-3 in response to LPS and IFN-*γ* activation in RAW 264.7, and inhibiting NF-*κ*B activation by preventing the translocation of the p65 subunit into the nucleus [31]. Based on these findings on sideroxylin, known to elevate the intracellular ROS levels, which are causal factors for mitochondrial dysfunction and antitumor cell proliferation, we sought to apply it to the antitumoral aspect. As expected, sideroxylin not only exerts an effective mitoROS stimulator but also shows a strong antitumoral effect on *in vivo* thyroid cancer despite the unclear mechanism.

Collectively, we demonstrated the role of mitoROS regulation in clinical relevance and tumor growth suppression in thyroid cancer, both bioinformatically and experimentally. The expression of mitoROS regulators represented by NDUFB3 was associated with the clinical features and PFS of patients with thyroid cancers. NDUFB3 overexpression and treatment with sideroxylin effectively increased mitoROS and limited *in vivo* tumor growth. The bioinformatics techniques combined with laboratory experiments provide a classical and practical example of using emerging technology. Another highlight of this work is that moderate mitoROS is beneficial to the survival of thyroid cancer patients. Thus, direct antitumor therapy with antioxidants is unlikely to have a good therapeutic impact. Our findings demonstrate that determining a correlation between mitoROS regulators and survival before ROS modulation is integral for better patient outcomes.

## Data Availability

The original data will be available upon request.

## Supplementary Materials

Supplementary 1 Supplementary Figure 1: consensus clusters by mitoROS regulators in TCGA cohort. Supplementary Figure 2: TCGA patient cases grouped by NDUFB3 expression overlap well with the cluster 1/2/3. Supplementary Figure 3: immunoblots indicating NDUFB3 protein expression in BCPAP or C643 cells expressing shRNA targeting human NDUFB3 (shNDUFB3) or empty vector (shCtrl). Supplementary Figure 4: hierarchical clustering analysis of mRNAs, which were differentially expressed between NDUFB3low and NDUFB3high. Supplementary Figure 5: GO analysis of differentially regulated genes in NDUFB3low vs. NUDFB3high thyroid cancer tissues. Supplementary Figure 6: bar chart showing pathway analysis by GO Slim summary for the differentiated genes comparing NDUFB3high with NDUFB3low. Supplementary Figure 7: NDUFB3 colocalizes with mitochondrial in C643 cell lines. Supplementary Figure 8: immunoblots indicating NDUFB3 protein expression in BCPAP or C643 cells expressing human NDUFB3 cDNA or empty vector.

Supplementary 2 Supplementary Table 2: a table showing primer sequences and production lengths of human complex I subunit genes and MnSOD. Supplementary Table 3: a table showing the names and corresponding reference PubMed IDs of 35 mitoROS regulators. Supplementary Table 1: a table showing the cluster to which each TCGA sample ID belongs to the postconsensus clustering of 510 diagnosed thyroid cancer patients. Supplementary Table 4: a table showing the detailed clinical information of each TCGA sample within each cluster. Supplementary Table 5: a table showing the PFS duration of each TCGA sample within each cluster. Supplementary Table 6: a table showing the effect of high (50%) or low (50%) expression of each of the 35 mitoSOX regulators on PFS of 510 thyroid cancer patients in TCGA cohort. Supplementary Table 7: a table showing the exact expression value of each of the 35 mitoROS regulators for 510 TCGA samples. Supplementary Table 8: a table showing the detailed clinical information of each of 510 thyroid cancer samples divided into NDUFB3high and NDUFB3low. Supplementary Table 9: a table showing the log2FC, average expression values, and statistical parameters of 16961 genes based on the comparison of NDUFB3low (25%, n = 127) with NDFFB3high (25%, n = 127). Supplementary Table 10: a table showing statistical parameters of each of the 18 enriched KEGG pathways based on the comparison of NDUFB3low with NDFFB3high. Supplementary Table 11: a table showing the score of each of the 80 genes in the OXPHOS pathway based on the comparison of NDUFB3low with NDFFB3high.
