# Supplementary material for: Clinical Relevance and Tumor Growth Suppression of Mitochondrial ROS Regulators along NADH:Ubiquinone Oxidoreductase Subunit B3 in Thyroid Cancer
Source: Oxid Med Cell Longev. 2022 Jan 17;2022:8038857. doi: 10.1155/2022/8038857 (PMC8787455; doi:10.1155/2022/8038857)
Supplement: Supplementary 1 — Supplementary Figure 1: consensus clusters by mitoROS regulators in TCGA cohort. Supplementary Figure 2: TCGA patient cases grouped by NDUFB3 expression overlap well with the cluster 1/2/3. Supplementary Figure 3: immunoblots indicating NDUFB3 protein expression in BCPAP or C643 cells expressing shRNA targeting human NDUFB3 (shNDUFB3) or empty vector (shCtrl). Supplementary Figure 4: hierarchical clustering analysis of mRNAs, which were differentially expressed between NDUFB3low and NDUFB3high. Supplementary Figure 5: GO analysis of differentially regulated genes in NDUFB3low vs. NUDFB3high thyroid cancer tissues. Supplementary Figure 6: bar chart showing pathway analysis by GO Slim summary for the differentiated genes comparing NDUFB3high with NDUFB3low. Supplementary Figure 7: NDUFB3 colocalizes with mitochondrial in C643 cell lines. Supplementary Figure 8: immunoblots indicating NDUFB3 protein expression in BCPAP or C643 cells expressing human NDUFB3 cDNA or empty vector. [file 8038857.f1.docx]

**Supplementary Figs**

**Clinical Relevance and Tumor Growth Suppression of Mitochondrial ROS Regulators along NADH:Ubiquinone Oxidoreductase Subunit B3 in Thyroid Cancer**

Jiao Zhu, Xiaobo Zheng, Dan Lu, Yun Zheng and Jun Liu

**Figure S1**. Related to Fig 1. Consensus clusters by mitoROS regulators in TCGA cohort. (A) Consensus clustering cumulative distribution function (CDF) for k=2 to 6. (B) Relative change in area under the CDF curve for k=2 to 6. (C) Tracking plot for k=2 to 6. (D-H) Heatmap depicting consensus clustering solution (k = 2 to 6) for genes regulating mitoROS in 510 patients with thyroid tumors in TCGA cohort.


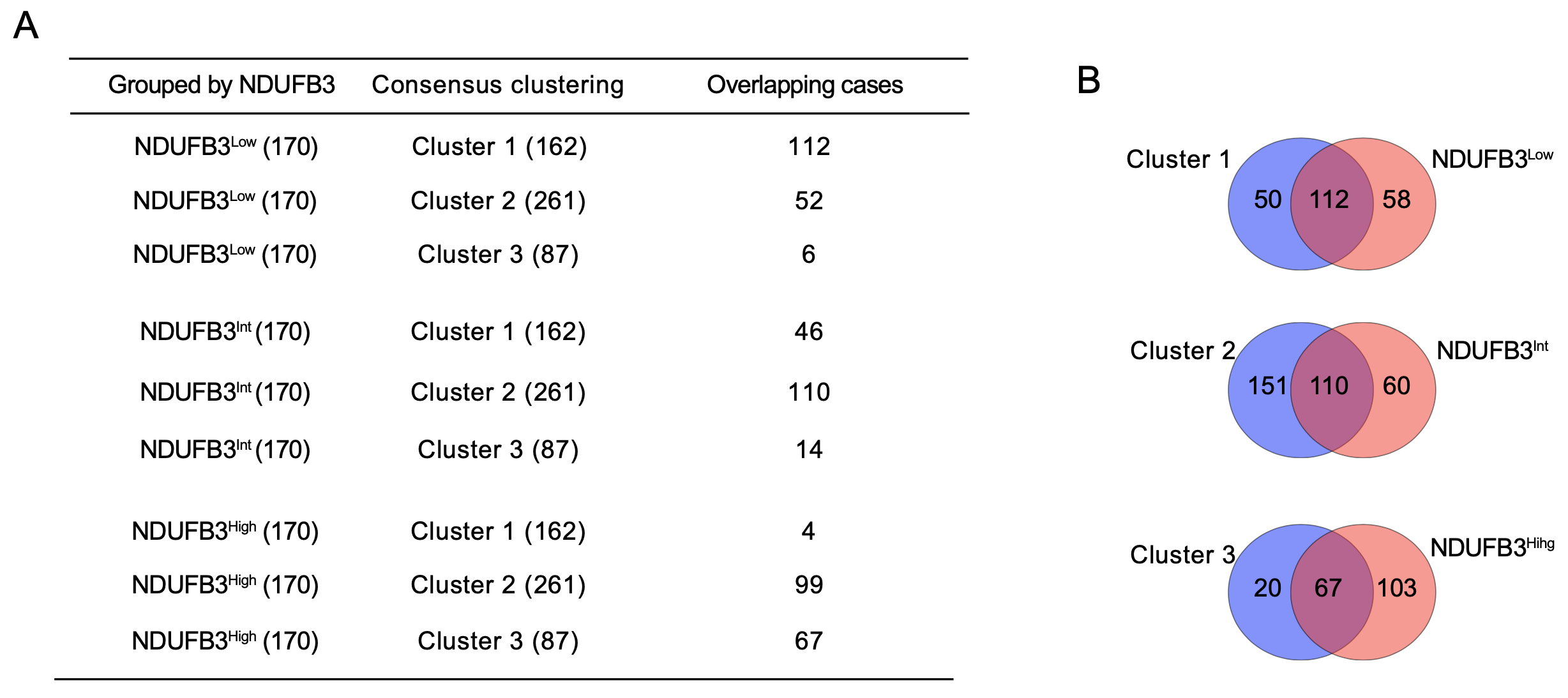


**Figure S2**. Related to Fig 3. TCGA patient cases grouped by NDUFB3 expression overlap well with the cluster 1/2/3. (A) Table showing low, medium and high expression of NDUFB3 corresponded to the number of overlapping cases of cluster1 / 2 / 3, respectively. (B) Venn diagram showing the overlap NDUFB3^Low^ and cluster 1, NDUFB3^Int^ and cluster 2, as well as NDUFB3^High^ and cluster 3.


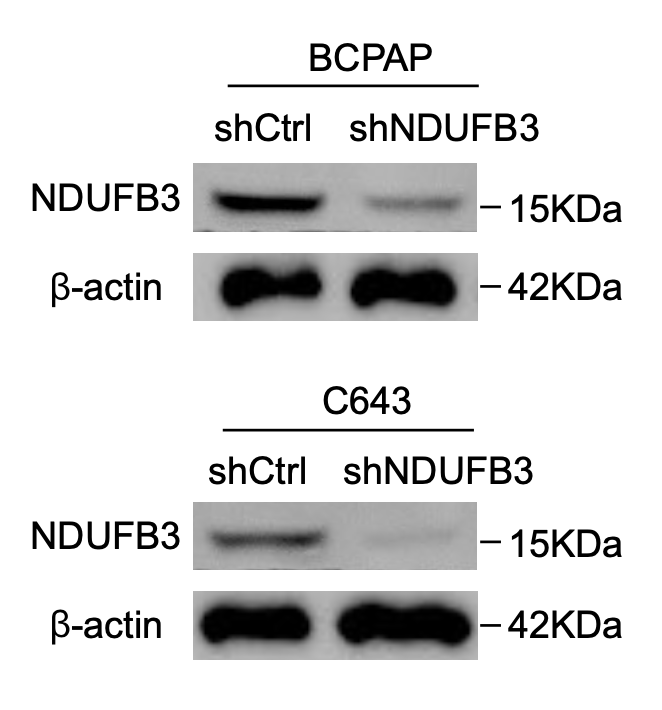


**Figure S3**. Related to Fig 3. Immunoblots indicating NDUFB3 protein expression in BCPAP or C643 cells expressing shRNA targeting human NDUFB3 (shNDUFB3) or empty vector (shCtrl).

**Figure S4**. Related to Fig 3 and Fig4. The expression changes of human complex I subunits. (A and C) qRT-PCR analyses of nuclear-encoded complex I subunits in NDUFB3-knockdown (A) or overexpressed (C) C643 cells. (B and D) Immunoblots indicating the protein expression levels of the mitochondrial DNA-encoded complex I subunits in NDUFB3-knockdown (B) or overexpressed (D) C643 cells.


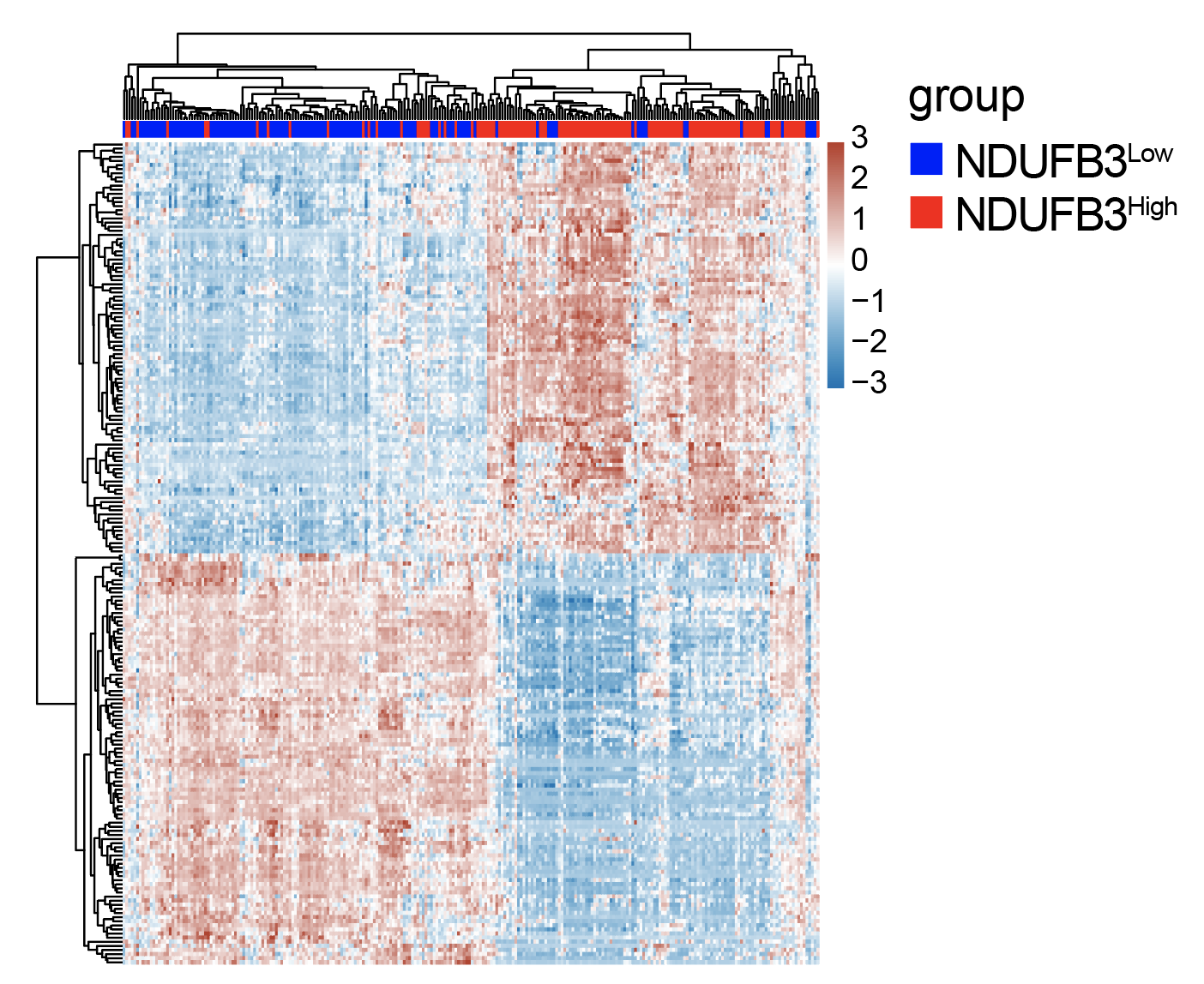


**Figure S5**: Related to Fig 4. Hierarchical clustering analysis of mRNAs, which were differentially expressed between NDUFB3^low^ and NDUFB3^high^.


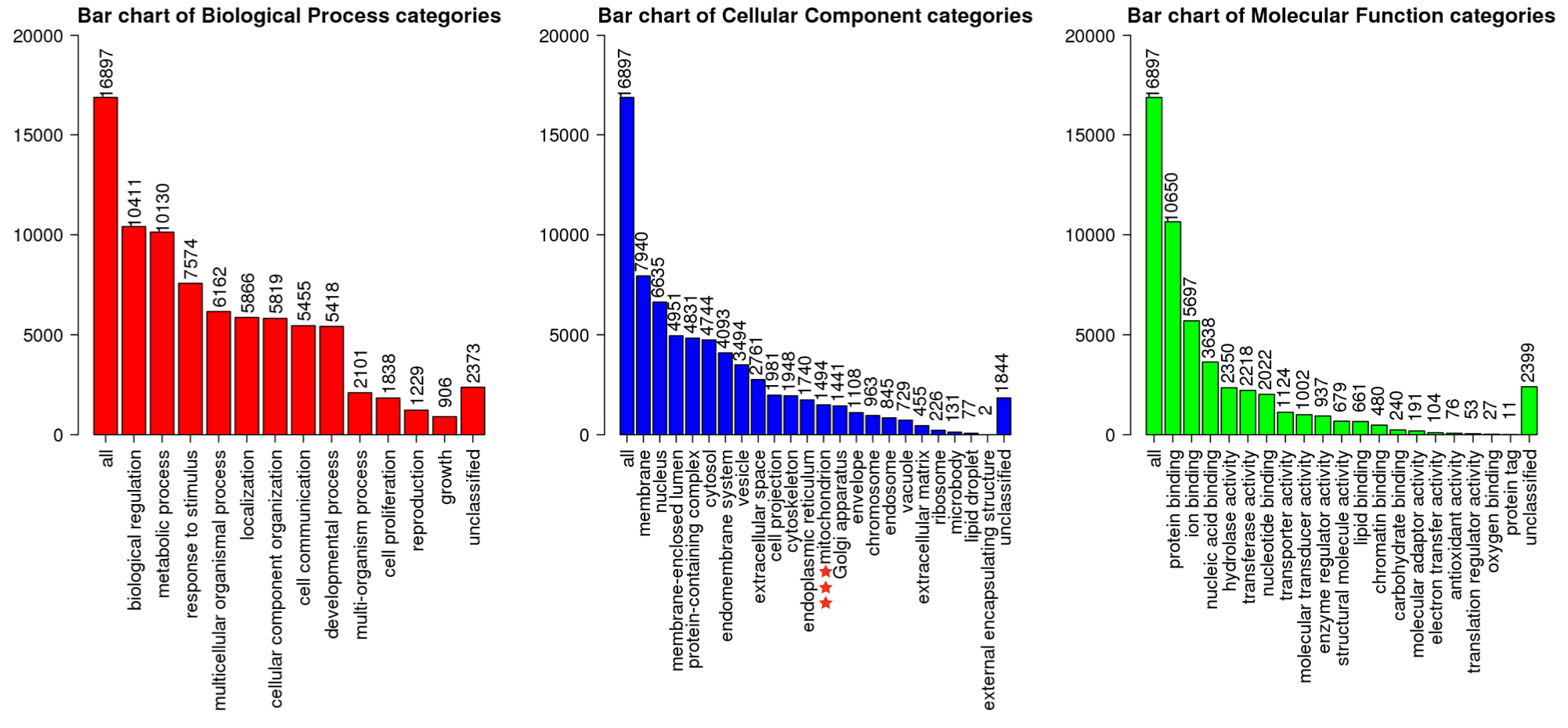


**Figure S6**: Related to Fig 4. GO analysis of differentially regulated genes in NDUFB3^Low^ vs NUDFB3^High^ thyroid cancer tissues. Genes belonging to the cellular component GO term “mitochondrion” are highlighted with three red stars.


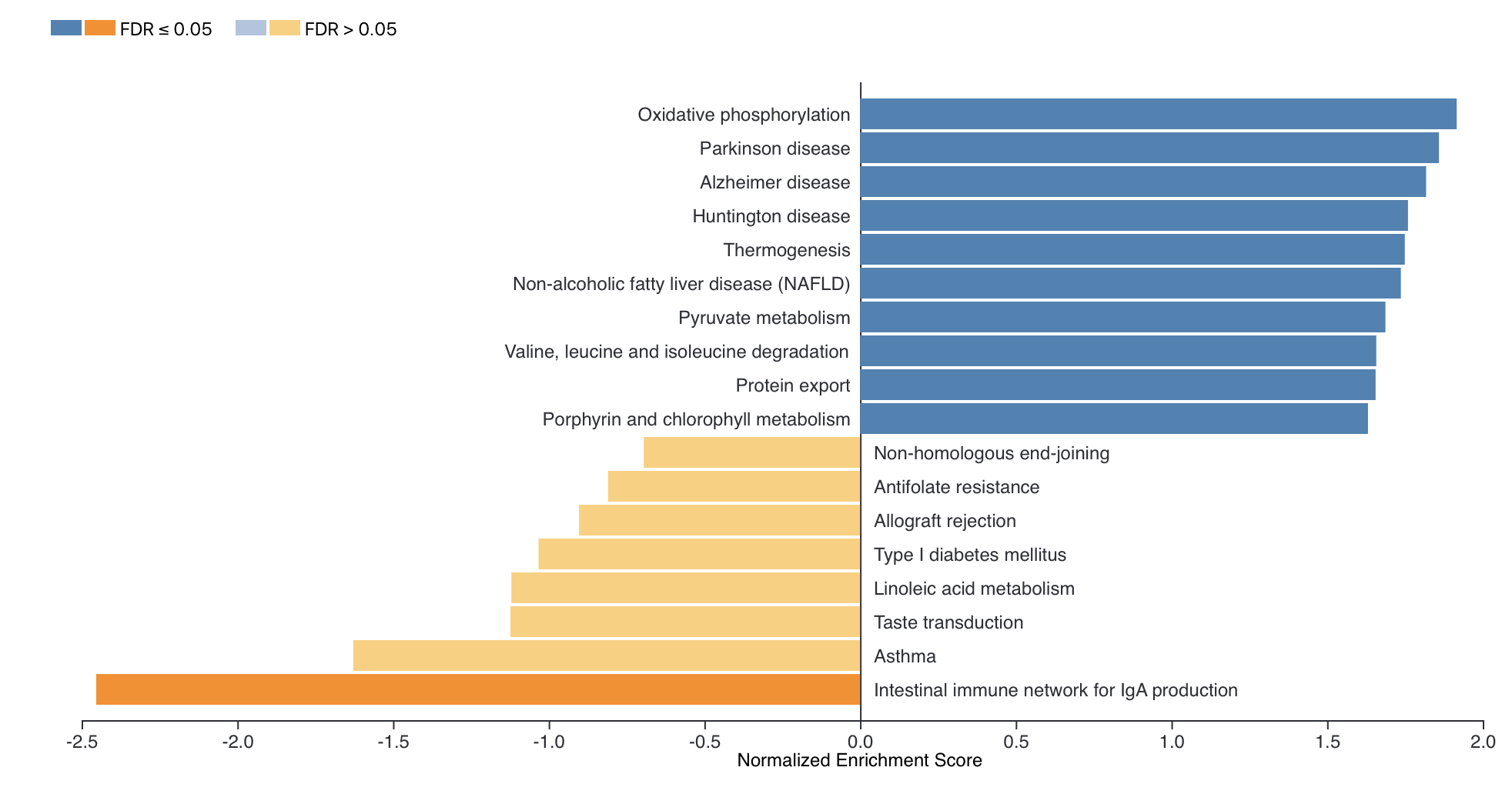


**Figure S7**: Related to Fig 4. Bar chart showing pathway analysis by GO Slim summary for the differentiated genes comparing NDUFB3^high^ with NDUFB3^low^.


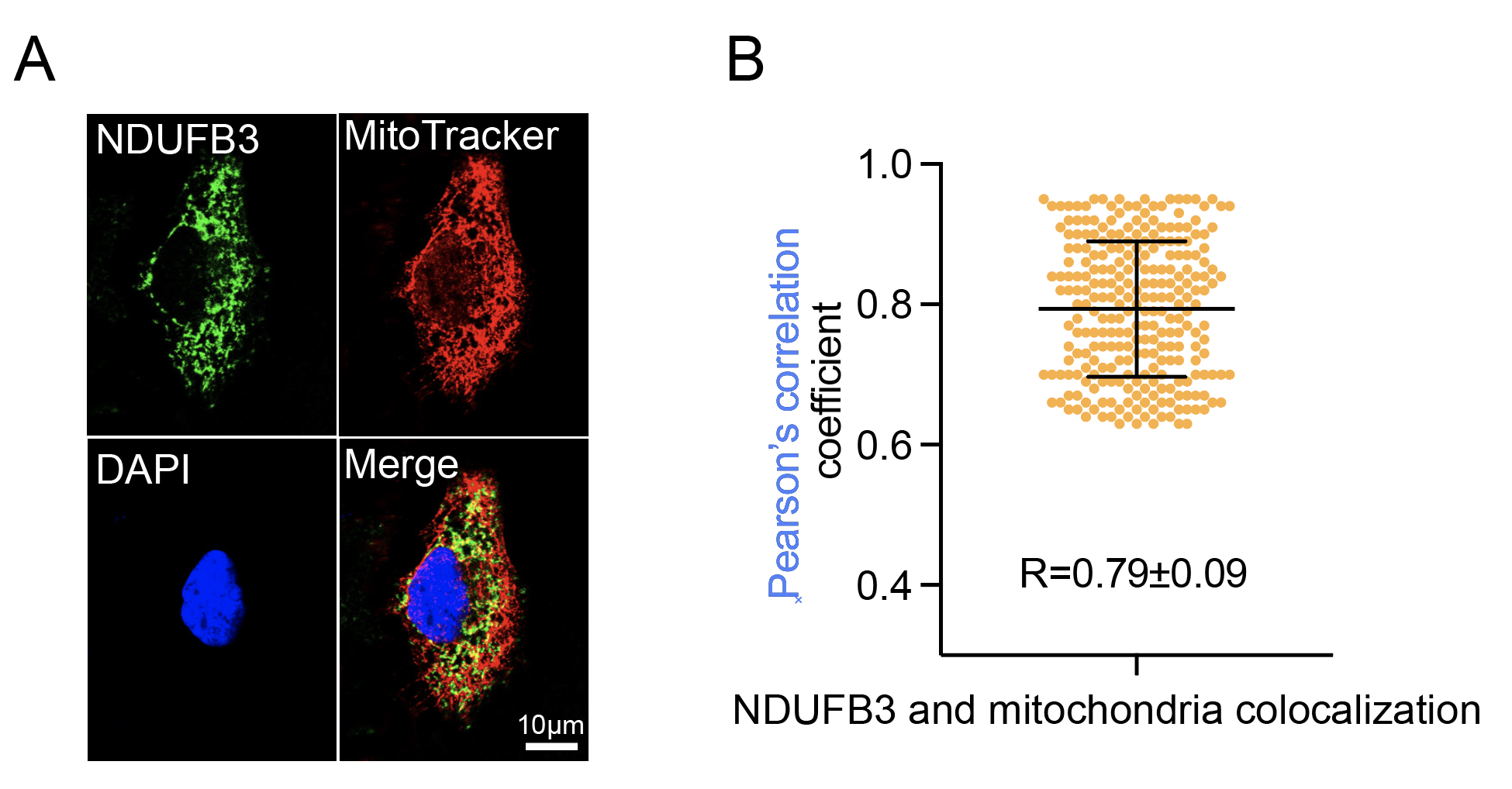


**Figure S8**: Related to Fig 4. NDUFB3 colocalizes with mitochondrial in C643 cell lines. (A) Representative immunofluorescence image showing endogenous NDUFB3 and mitochondria colocalization in C643 cells. Scale bars indicate 10 μm. (B) Quantification of NDUFB3 and mitochondria colocalization data performed using GraphPad Prism with assessment of 264 regions of interest on more than 10 cells for each experimental group with experiments repeated at least three times.


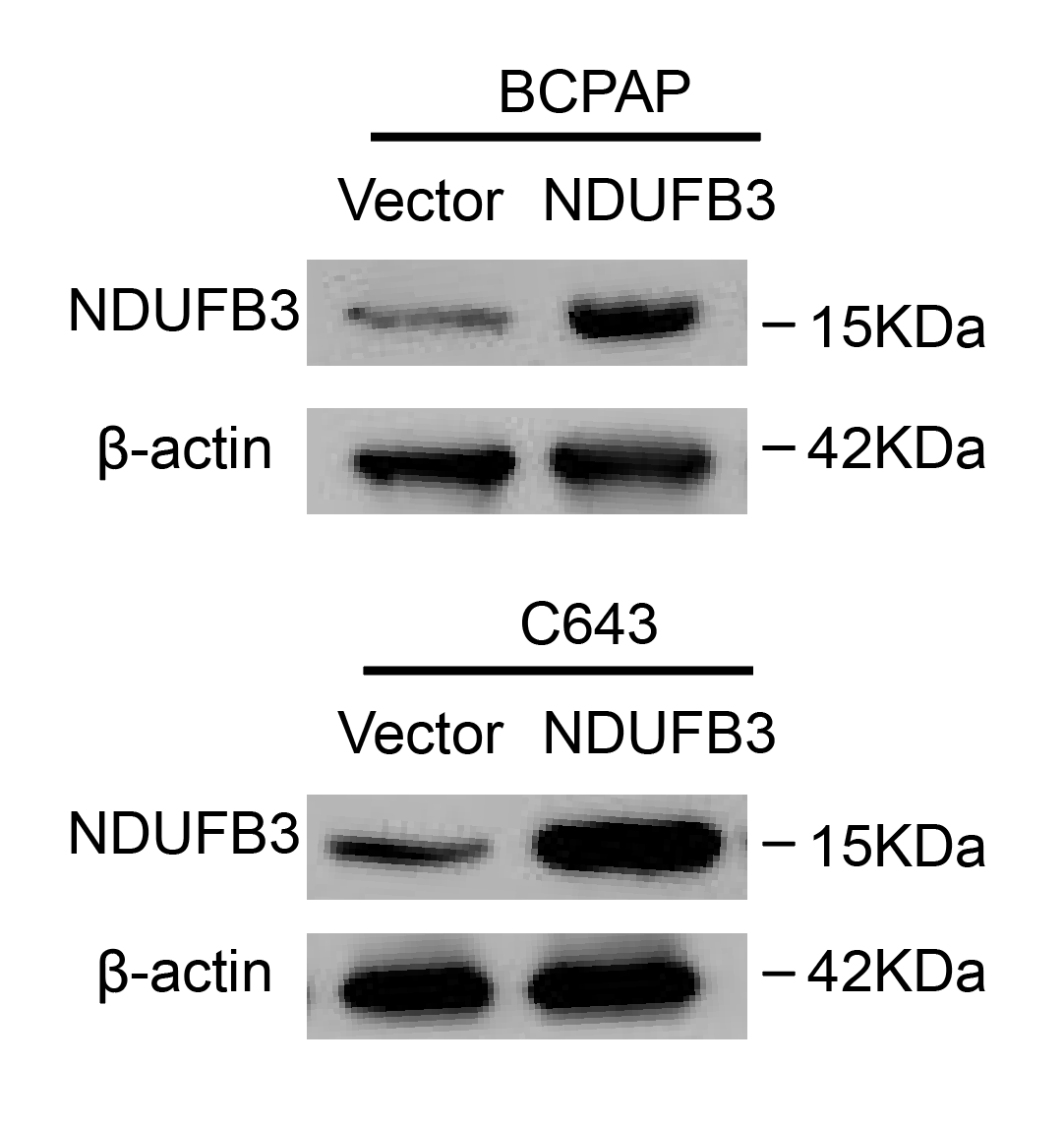


**Figure S9**: Related to Fig 4. Immunoblots indicating NDUFB3 protein expression in BCPAP or C643 cells expressing human NDUFB3 cDNA or empty vector.

**Figure S10**: Related to Fig 4. The effects of NDUFB3 overexpression on mtDNA copy number and mitochondrial mass. (A) mtDNA copy number was determined by qRT-PCR in the indicated cells. (B) Mitochondrial mass was determined by flow cytometry post MitoTracker staining in the indicated cells. mtDNA, mitochondrial DNA. Statistical differences were determined by a two-tailed unpaired Mann–Whitney t-test.

**Figure S11**: Related to Fig 4. The effects of NDUFB3 overexpression on the antioxidant system. (A) Immunoblots and quantification indicating the expression of the indicated antioxidant enzymes in C643 cells over-expressing NDUFB3 or empty vector. (B) MnSOD mRNA expression was determined by qRT-PCR in BCPAP and C643 cells over-expressing NDUFB3 or empty vector. (C-D) Comparison of GSH levels (C) and catalase activity (D) between NDUFB3-overexpression and empty vector in BCPAP and C643 cells. L-BSO, L-Buthionine sulfoximine; EGCG, epigallocatechol gallate. They are effective inhibitors of GSH levels and catalase activity, respectively, and are used as positive controls here. Statistical differences were determined by a two-tailed unpaired Mann–Whitney t-test. *P<0.05, **P<0.01.
