# Supplementary material for: Clinical Relevance and Tumor Growth Suppression of Mitochondrial ROS Regulators along NADH:Ubiquinone Oxidoreductase Subunit B3 in Thyroid Cancer
Source: Oxid Med Cell Longev. 2022 Jan 17;2022:8038857. doi: 10.1155/2022/8038857 (PMC8787455; doi:10.1155/2022/8038857)
Supplement: Supplementary 2 — Supplementary Table 2: a table showing primer sequences and production lengths of human complex I subunit genes and MnSOD. Supplementary Table 3: a table showing the names and corresponding reference PubMed IDs of 35 mitoROS regulators. Supplementary Table 1: a table showing the cluster to which each TCGA sample ID belongs to the postconsensus clustering of 510 diagnosed thyroid cancer patients. Supplementary Table 4: a table showing the detailed clinical information of each TCGA sample within each cluster. Supplementary Table 5: a table showing the PFS duration of each TCGA sample within each cluster. Supplementary Table 6: a table showing the effect of high (50%) or low (50%) expression of each of the 35 mitoSOX regulators on PFS of 510 thyroid cancer patients in TCGA cohort. Supplementary Table 7: a table showing the exact expression value of each of the 35 mitoROS regulators for 510 TCGA samples. Supplementary Table 8: a table showing the detailed clinical information of each of 510 thyroid cancer samples divided into NDUFB3high and NDUFB3low. Supplementary Table 9: a table showing the log2FC, average expression values, and statistical parameters of 16961 genes based on the comparison of NDUFB3low (25%, n = 127) with NDFFB3high (25%, n = 127). Supplementary Table 10: a table showing statistical parameters of each of the 18 enriched KEGG pathways based on the comparison of NDUFB3low with NDFFB3high. Supplementary Table 11: a table showing the score of each of the 80 genes in the OXPHOS pathway based on the comparison of NDUFB3low with NDFFB3high. [file 8038857.f2.zip › Supplementary table 1.pdf]

TCGA _ID	Cluster
TCGA-DE-A4MD-06	C1
TCGA-EM-A2CS-06	C2
TCGA-EM-A2P1-06	C1
TCGA-EM-A3FQ-06	C3
TCGA-EM-A3SU-06	C1
TCGA-J8-A3O2-06	C2
TCGA-J8-A3YH-06	C2
TCGA-J8-A4HW-06	C2
TCGA-4C-A93U-01	C1
TCGA-BJ-A0YZ-01	C3
TCGA-BJ-A0Z0-01	C2
TCGA-BJ-A0Z2-01	C2
TCGA-BJ-A0Z3-01	C2
TCGA-BJ-A0Z5-01	C1
TCGA-BJ-A0Z9-01	C1
TCGA-BJ-A0ZA-01	C2
TCGA-BJ-A0ZB-01	C1
TCGA-BJ-A0ZC-01	C2
TCGA-BJ-A0ZE-01	C2
TCGA-BJ-A0ZF-01	C3
TCGA-BJ-A0ZG-01	C2
TCGA-BJ-A0ZH-01	C1
TCGA-BJ-A0ZJ-01	C2
TCGA-BJ-A18Y-01	C2
TCGA-BJ-A18Z-01	C1
TCGA-BJ-A190-01	C2
TCGA-BJ-A191-01	C2
TCGA-BJ-A192-01	C3
TCGA-BJ-A28R-01	C1
TCGA-BJ-A28S-01	C2
TCGA-BJ-A28T-01	C1
TCGA-BJ-A28V-01	C3
TCGA-BJ-A28W-01	C3
TCGA-BJ-A28X-01	C1
TCGA-BJ-A28Z-01	C1
TCGA-BJ-A290-01	C2
TCGA-BJ-A291-01	C3
TCGA-BJ-A2N7-01	C2
TCGA-BJ-A2N8-01	C3

TCGA-BJ-A2N9-01	C3
TCGA-BJ-A2NA-01	C1
TCGA-BJ-A2P4-01	C3
TCGA-BJ-A3EZ-01	C1
TCGA-BJ-A3F0-01	C3
TCGA-BJ-A3PR-01	C1
TCGA-BJ-A3PT-01	C3
TCGA-BJ-A3PU-01	C3
TCGA-BJ-A45C-01	C2
TCGA-BJ-A45D-01	C3
TCGA-BJ-A45E-01	C1
TCGA-BJ-A45F-01	C2
TCGA-BJ-A45G-01	C2
TCGA-BJ-A45H-01	C2
TCGA-BJ-A45I-01	C1
TCGA-BJ-A45J-01	C2
TCGA-BJ-A45K-01	C3
TCGA-BJ-A4O8-01	C1
TCGA-BJ-A4O9-01	C2
TCGA-CE-A13K-01	C2
TCGA-CE-A27D-01	C2
TCGA-CE-A3MD-01	C3
TCGA-CE-A3ME-01	C2
TCGA-CE-A481-01	C2
TCGA-CE-A482-01	C2
TCGA-CE-A483-01	C2
TCGA-CE-A484-01	C3
TCGA-CE-A485-01	C2
TCGA-DE-A0XZ-01	C2
TCGA-DE-A0Y2-01	C1
TCGA-DE-A0Y3-01	C1
TCGA-DE-A2OL-01	C3
TCGA-DE-A3KN-01	C2
TCGA-DE-A4M8-01	C1
TCGA-DE-A4M9-01	C2
TCGA-DE-A4MA-01	C3
TCGA-DE-A4MB-01	C1
TCGA-DE-A4MC-01	C1
TCGA-DE-A4MD-01	C1
TCGA-DE-A69J-01	C2

TCGA-DE-A69K-01	C2
TCGA-DE-A7U5-01	C1
TCGA-DJ-A13L-01	C1
TCGA-DJ-A13M-01	C3
TCGA-DJ-A13O-01	C2
TCGA-DJ-A13P-01	C2
TCGA-DJ-A13R-01	C1
TCGA-DJ-A13S-01	C2
TCGA-DJ-A13T-01	C2
TCGA-DJ-A13U-01	C1
TCGA-DJ-A13V-01	C2
TCGA-DJ-A13W-01	C1
TCGA-DJ-A13X-01	C2
TCGA-DJ-A1QD-01	C1
TCGA-DJ-A1QE-01	C1
TCGA-DJ-A1QF-01	C1
TCGA-DJ-A1QG-01	C1
TCGA-DJ-A1QH-01	C2
TCGA-DJ-A1QI-01	C2
TCGA-DJ-A1QL-01	C3
TCGA-DJ-A1QM-01	C2
TCGA-DJ-A1QN-01	C2
TCGA-DJ-A1QO-01	C1
TCGA-DJ-A1QQ-01	C1
TCGA-DJ-A2PN-01	C2
TCGA-DJ-A2PO-01	C1
TCGA-DJ-A2PP-01	C2
TCGA-DJ-A2PQ-01	C2
TCGA-DJ-A2PR-01	C1
TCGA-DJ-A2PS-01	C2
TCGA-DJ-A2PT-01	C3
TCGA-DJ-A2PU-01	C2
TCGA-DJ-A2PV-01	C1
TCGA-DJ-A2PW-01	C2
TCGA-DJ-A2PX-01	C2
TCGA-DJ-A2PY-01	C1
TCGA-DJ-A2PZ-01	C1
TCGA-DJ-A2Q0-01	C2
TCGA-DJ-A2Q1-01	C1
TCGA-DJ-A2Q2-01	C2

TCGA-DJ-A2Q3-01	C1
TCGA-DJ-A2Q4-01	C1
TCGA-DJ-A2Q5-01	C2
TCGA-DJ-A2Q6-01	C2
TCGA-DJ-A2Q7-01	C2
TCGA-DJ-A2Q9-01	C1
TCGA-DJ-A2QA-01	C3
TCGA-DJ-A2QB-01	C2
TCGA-DJ-A2QC-01	C3
TCGA-DJ-A3UK-01	C2
TCGA-DJ-A3UM-01	C2
TCGA-DJ-A3UN-01	C2
TCGA-DJ-A3UO-01	C2
TCGA-DJ-A3UP-01	C2
TCGA-DJ-A3UQ-01	C1
TCGA-DJ-A3UR-01	C2
TCGA-DJ-A3US-01	C2
TCGA-DJ-A3UT-01	C3
TCGA-DJ-A3UU-01	C2
TCGA-DJ-A3UV-01	C2
TCGA-DJ-A3UW-01	C1
TCGA-DJ-A3UX-01	C2
TCGA-DJ-A3UY-01	C1
TCGA-DJ-A3UZ-01	C2
TCGA-DJ-A3V0-01	C2
TCGA-DJ-A3V2-01	C2
TCGA-DJ-A3V3-01	C2
TCGA-DJ-A3V4-01	C2
TCGA-DJ-A3V5-01	C1
TCGA-DJ-A3V6-01	C1
TCGA-DJ-A3V7-01	C2
TCGA-DJ-A3V8-01	C2
TCGA-DJ-A3V9-01	C2
TCGA-DJ-A3VA-01	C2
TCGA-DJ-A3VB-01	C1
TCGA-DJ-A3VD-01	C1
TCGA-DJ-A3VE-01	C1
TCGA-DJ-A3VF-01	C1
TCGA-DJ-A3VG-01	C3
TCGA-DJ-A3VI-01	C1

TCGA-DJ-A3VJ-01	C2
TCGA-DJ-A3VK-01	C3
TCGA-DJ-A3VL-01	C3
TCGA-DJ-A3VM-01	C2
TCGA-DJ-A4UL-01	C3
TCGA-DJ-A4UP-01	C2
TCGA-DJ-A4UQ-01	C2
TCGA-DJ-A4UR-01	C2
TCGA-DJ-A4UT-01	C1
TCGA-DJ-A4UW-01	C2
TCGA-DJ-A4V0-01	C2
TCGA-DJ-A4V2-01	C1
TCGA-DJ-A4V4-01	C1
TCGA-DJ-A4V5-01	C2
TCGA-DO-A1JZ-01	C2
TCGA-DO-A1K0-01	C1
TCGA-DO-A2HM-01	C1
TCGA-E3-A3DY-01	C2
TCGA-E3-A3DZ-01	C2
TCGA-E3-A3E0-01	C2
TCGA-E3-A3E1-01	C2
TCGA-E3-A3E2-01	C2
TCGA-E3-A3E3-01	C2
TCGA-E3-A3E5-01	C2
TCGA-E8-A242-01	C1
TCGA-E8-A2EA-01	C1
TCGA-E8-A2JQ-01	C1
TCGA-E8-A3X7-01	C2
TCGA-E8-A413-01	C1
TCGA-E8-A414-01	C1
TCGA-E8-A415-01	C2
TCGA-E8-A416-01	C2
TCGA-E8-A417-01	C1
TCGA-E8-A418-01	C2
TCGA-E8-A419-01	C1
TCGA-E8-A432-01	C3
TCGA-E8-A433-01	C3
TCGA-E8-A434-01	C2
TCGA-E8-A436-01	C1
TCGA-E8-A437-01	C2

TCGA-E8-A438-01	C2
TCGA-E8-A44K-01	C1
TCGA-E8-A44M-01	C2
TCGA-EL-A3CL-01	C2
TCGA-EL-A3CM-01	C1
TCGA-EL-A3CN-01	C1
TCGA-EL-A3CO-01	C1
TCGA-EL-A3CP-01	C2
TCGA-EL-A3CR-01	C2
TCGA-EL-A3CS-01	C1
TCGA-EL-A3CT-01	C1
TCGA-EL-A3CU-01	C1
TCGA-EL-A3CV-01	C1
TCGA-EL-A3CW-01	C2
TCGA-EL-A3CX-01	C3
TCGA-EL-A3CY-01	C1
TCGA-EL-A3CZ-01	C2
TCGA-EL-A3D0-01	C1
TCGA-EL-A3D1-01	C3
TCGA-EL-A3D4-01	C3
TCGA-EL-A3D5-01	C2
TCGA-EL-A3D6-01	C1
TCGA-EL-A3GO-01	C1
TCGA-EL-A3GP-01	C1
TCGA-EL-A3GQ-01	C1
TCGA-EL-A3GR-01	C1
TCGA-EL-A3GS-01	C1
TCGA-EL-A3GU-01	C1
TCGA-EL-A3GV-01	C1
TCGA-EL-A3GW-01	C2
TCGA-EL-A3GX-01	C1
TCGA-EL-A3GY-01	C1
TCGA-EL-A3GZ-01	C2
TCGA-EL-A3H1-01	C3
TCGA-EL-A3H2-01	C3
TCGA-EL-A3H3-01	C2
TCGA-EL-A3H4-01	C1
TCGA-EL-A3H5-01	C1
TCGA-EL-A3H7-01	C1
TCGA-EL-A3H8-01	C2

TCGA-EL-A3MW-01	C2
TCGA-EL-A3MX-01	C2
TCGA-EL-A3MY-01	C1
TCGA-EL-A3MZ-01	C1
TCGA-EL-A3N2-01	C1
TCGA-EL-A3N3-01	C1
TCGA-EL-A3T0-01	C2
TCGA-EL-A3T1-01	C1
TCGA-EL-A3T2-01	C2
TCGA-EL-A3T3-01	C1
TCGA-EL-A3T6-01	C1
TCGA-EL-A3T7-01	C2
TCGA-EL-A3T8-01	C2
TCGA-EL-A3T9-01	C1
TCGA-EL-A3TA-01	C2
TCGA-EL-A3TB-01	C2
TCGA-EL-A3ZG-01	C2
TCGA-EL-A3ZH-01	C2
TCGA-EL-A3ZK-01	C1
TCGA-EL-A3ZL-01	C2
TCGA-EL-A3ZM-01	C3
TCGA-EL-A3ZN-01	C3
TCGA-EL-A3ZO-01	C1
TCGA-EL-A3ZP-01	C2
TCGA-EL-A3ZQ-01	C3
TCGA-EL-A3ZR-01	C3
TCGA-EL-A3ZS-01	C2
TCGA-EL-A3ZT-01	C1
TCGA-EL-A4JV-01	C2
TCGA-EL-A4JW-01	C1
TCGA-EL-A4JX-01	C1
TCGA-EL-A4JZ-01	C2
TCGA-EL-A4K0-01	C2
TCGA-EL-A4K1-01	C1
TCGA-EL-A4K2-01	C2
TCGA-EL-A4K4-01	C1
TCGA-EL-A4K6-01	C2
TCGA-EL-A4K7-01	C3
TCGA-EL-A4K9-01	C2
TCGA-EL-A4KD-01	C2

TCGA-EL-A4KG-01	C2
TCGA-EL-A4KH-01	C3
TCGA-EL-A4KI-01	C3
TCGA-EM-A1CS-01	C2
TCGA-EM-A1CT-01	C1
TCGA-EM-A1CU-01	C2
TCGA-EM-A1CV-01	C2
TCGA-EM-A1CW-01	C2
TCGA-EM-A1YA-01	C2
TCGA-EM-A1YB-01	C2
TCGA-EM-A1YC-01	C2
TCGA-EM-A1YD-01	C2
TCGA-EM-A1YE-01	C3
TCGA-EM-A22I-01	C1
TCGA-EM-A22J-01	C2
TCGA-EM-A22K-01	C2
TCGA-EM-A22L-01	C2
TCGA-EM-A22M-01	C1
TCGA-EM-A22N-01	C2
TCGA-EM-A22O-01	C2
TCGA-EM-A22P-01	C2
TCGA-EM-A22Q-01	C3
TCGA-EM-A2CJ-01	C2
TCGA-EM-A2CK-01	C2
TCGA-EM-A2CL-01	C2
TCGA-EM-A2CM-01	C2
TCGA-EM-A2CN-01	C2
TCGA-EM-A2CO-01	C2
TCGA-EM-A2CP-01	C1
TCGA-EM-A2CQ-01	C2
TCGA-EM-A2CR-01	C3
TCGA-EM-A2CS-01	C2
TCGA-EM-A2CT-01	C3
TCGA-EM-A2CU-01	C2
TCGA-EM-A2OV-01	C2
TCGA-EM-A2OW-01	C2
TCGA-EM-A2OX-01	C2
TCGA-EM-A2OY-01	C2
TCGA-EM-A2OZ-01	C2
TCGA-EM-A2P0-01	C2

TCGA-EM-A2P1-01	C1
TCGA-EM-A2P2-01	C2
TCGA-EM-A2P3-01	C2
TCGA-EM-A3AI-01	C3
TCGA-EM-A3AJ-01	C3
TCGA-EM-A3AK-01	C2
TCGA-EM-A3AL-01	C3
TCGA-EM-A3AN-01	C2
TCGA-EM-A3AO-01	C2
TCGA-EM-A3AP-01	C2
TCGA-EM-A3AQ-01	C3
TCGA-EM-A3AR-01	C2
TCGA-EM-A3FJ-01	C2
TCGA-EM-A3FK-01	C1
TCGA-EM-A3FL-01	C3
TCGA-EM-A3FM-01	C1
TCGA-EM-A3FN-01	C3
TCGA-EM-A3FO-01	C2
TCGA-EM-A3FP-01	C3
TCGA-EM-A3FQ-01	C2
TCGA-EM-A3FR-01	C2
TCGA-EM-A3O3-01	C1
TCGA-EM-A3O6-01	C3
TCGA-EM-A3O7-01	C2
TCGA-EM-A3O8-01	C2
TCGA-EM-A3OA-01	C3
TCGA-EM-A3OB-01	C3
TCGA-EM-A3ST-01	C3
TCGA-EM-A3SU-01	C3
TCGA-EM-A3SX-01	C2
TCGA-EM-A3SY-01	C2
TCGA-EM-A3SZ-01	C1
TCGA-EM-A4FF-01	C1
TCGA-EM-A4FH-01	C3
TCGA-EM-A4FK-01	C3
TCGA-EM-A4FM-01	C1
TCGA-EM-A4FN-01	C2
TCGA-EM-A4FO-01	C2
TCGA-EM-A4FQ-01	C1
TCGA-EM-A4FR-01	C2

TCGA-EM-A4FU-01	C2
TCGA-EM-A4FV-01	C2
TCGA-EM-A4G1-01	C2
TCGA-ET-A25G-01	C1
TCGA-ET-A25I-01	C2
TCGA-ET-A25K-01	C2
TCGA-ET-A25L-01	C1
TCGA-ET-A25M-01	C2
TCGA-ET-A25N-01	C1
TCGA-ET-A25O-01	C2
TCGA-ET-A25P-01	C2
TCGA-ET-A25R-01	C1
TCGA-ET-A2MX-01	C2
TCGA-ET-A2MY-01	C2
TCGA-ET-A2MZ-01	C2
TCGA-ET-A2N0-01	C3
TCGA-ET-A2N3-01	C3
TCGA-ET-A2N4-01	C2
TCGA-ET-A2N5-01	C3
TCGA-ET-A39I-01	C2
TCGA-ET-A39J-01	C2
TCGA-ET-A39K-01	C3
TCGA-ET-A39L-01	C2
TCGA-ET-A39M-01	C2
TCGA-ET-A39N-01	C3
TCGA-ET-A39O-01	C1
TCGA-ET-A39P-01	C2
TCGA-ET-A39R-01	C2
TCGA-ET-A39S-01	C2
TCGA-ET-A39T-01	C2
TCGA-ET-A3BN-01	C1
TCGA-ET-A3BO-01	C1
TCGA-ET-A3BP-01	C2
TCGA-ET-A3BQ-01	C2
TCGA-ET-A3BS-01	C1
TCGA-ET-A3BT-01	C2
TCGA-ET-A3BU-01	C2
TCGA-ET-A3BV-01	C1
TCGA-ET-A3BW-01	C2
TCGA-ET-A3BX-01	C1

TCGA-ET-A3DO-01	C1
TCGA-ET-A3DP-01	C2
TCGA-ET-A3DQ-01	C2
TCGA-ET-A3DR-01	C2
TCGA-ET-A3DS-01	C2
TCGA-ET-A3DU-01	C1
TCGA-ET-A3DV-01	C3
TCGA-ET-A3DW-01	C1
TCGA-ET-A40P-01	C3
TCGA-ET-A40Q-01	C1
TCGA-ET-A40R-01	C3
TCGA-ET-A40S-01	C2
TCGA-ET-A40T-01	C2
TCGA-ET-A4KN-01	C3
TCGA-ET-A4KQ-01	C2
TCGA-FE-A22Z-01	C1
TCGA-FE-A230-01	C1
TCGA-FE-A231-01	C2
TCGA-FE-A232-01	C2
TCGA-FE-A233-01	C1
TCGA-FE-A234-01	C1
TCGA-FE-A235-01	C3
TCGA-FE-A236-01	C2
TCGA-FE-A237-01	C2
TCGA-FE-A238-01	C2
TCGA-FE-A239-01	C3
TCGA-FE-A23A-01	C1
TCGA-FE-A3PA-01	C2
TCGA-FE-A3PB-01	C1
TCGA-FE-A3PC-01	C2
TCGA-FE-A3PD-01	C3
TCGA-FK-A3S3-01	C2
TCGA-FK-A3SB-01	C2
TCGA-FK-A3SD-01	C3
TCGA-FK-A3SE-01	C2
TCGA-FK-A3SG-01	C2
TCGA-FK-A3SH-01	C3
TCGA-FK-A4UB-01	C2
TCGA-FY-A2QD-01	C3
TCGA-FY-A3BL-01	C1

TCGA-FY-A3I4-01	C2
TCGA-FY-A3I5-01	C2
TCGA-FY-A3NM-01	C3
TCGA-FY-A3NN-01	C1
TCGA-FY-A3NP-01	C3
TCGA-FY-A3ON-01	C2
TCGA-FY-A3R6-01	C2
TCGA-FY-A3R7-01	C2
TCGA-FY-A3R8-01	C1
TCGA-FY-A3R9-01	C2
TCGA-FY-A3RA-01	C2
TCGA-FY-A3TY-01	C1
TCGA-FY-A3W9-01	C2
TCGA-FY-A3WA-01	C2
TCGA-FY-A3YR-01	C1
TCGA-FY-A40K-01	C2
TCGA-FY-A40L-01	C1
TCGA-FY-A40M-01	C2
TCGA-FY-A40N-01	C2
TCGA-FY-A4B0-01	C3
TCGA-FY-A4B3-01	C1
TCGA-FY-A4B4-01	C2
TCGA-FY-A76V-01	C2
TCGA-GE-A2C6-01	C2
TCGA-H2-A26U-01	C1
TCGA-H2-A2K9-01	C2
TCGA-H2-A3RH-01	C3
TCGA-H2-A3RI-01	C2
TCGA-H2-A421-01	C1
TCGA-H2-A422-01	C1
TCGA-IM-A3EB-01	C1
TCGA-IM-A3ED-01	C1
TCGA-IM-A3U2-01	C1
TCGA-IM-A3U3-01	C2
TCGA-IM-A41Y-01	C2
TCGA-IM-A41Z-01	C3
TCGA-IM-A420-01	C1
TCGA-IM-A4EB-01	C1
TCGA-J8-A3NZ-01	C2
TCGA-J8-A3O0-01	C2

TCGA-J8-A3O1-01	C1
TCGA-J8-A3O2-01	C2
TCGA-J8-A3YD-01	C2
TCGA-J8-A3YE-01	C2
TCGA-J8-A3YF-01	C1
TCGA-J8-A3YG-01	C1
TCGA-J8-A3YH-01	C2
TCGA-J8-A42S-01	C2
TCGA-J8-A4HW-01	C2
TCGA-J8-A4HY-01	C2
TCGA-KS-A41F-01	C3
TCGA-KS-A41I-01	C1
TCGA-KS-A41J-01	C3
TCGA-KS-A41L-01	C3
TCGA-KS-A4I1-01	C3
TCGA-KS-A4I3-01	C2
TCGA-KS-A4I5-01	C2
TCGA-KS-A4I7-01	C2
TCGA-KS-A4I9-01	C1
TCGA-KS-A4IB-01	C1
TCGA-KS-A4IC-01	C2
TCGA-KS-A4ID-01	C1
TCGA-L6-A4EP-01	C3
TCGA-L6-A4EQ-01	C2
TCGA-L6-A4ET-01	C2
TCGA-L6-A4EU-01	C1
TCGA-MK-A4N6-01	C1
TCGA-MK-A4N7-01	C2
TCGA-MK-A4N9-01	C2
TCGA-MK-A84Z-01	C3
TCGA-QD-A8IV-01	C2
