# Supplementary material for: Clinical Relevance and Tumor Growth Suppression of Mitochondrial ROS Regulators along NADH:Ubiquinone Oxidoreductase Subunit B3 in Thyroid Cancer
Source: Oxid Med Cell Longev. 2022 Jan 17;2022:8038857. doi: 10.1155/2022/8038857 (PMC8787455; doi:10.1155/2022/8038857)
Supplement: Supplementary 2 — Supplementary Table 2: a table showing primer sequences and production lengths of human complex I subunit genes and MnSOD. Supplementary Table 3: a table showing the names and corresponding reference PubMed IDs of 35 mitoROS regulators. Supplementary Table 1: a table showing the cluster to which each TCGA sample ID belongs to the postconsensus clustering of 510 diagnosed thyroid cancer patients. Supplementary Table 4: a table showing the detailed clinical information of each TCGA sample within each cluster. Supplementary Table 5: a table showing the PFS duration of each TCGA sample within each cluster. Supplementary Table 6: a table showing the effect of high (50%) or low (50%) expression of each of the 35 mitoSOX regulators on PFS of 510 thyroid cancer patients in TCGA cohort. Supplementary Table 7: a table showing the exact expression value of each of the 35 mitoROS regulators for 510 TCGA samples. Supplementary Table 8: a table showing the detailed clinical information of each of 510 thyroid cancer samples divided into NDUFB3high and NDUFB3low. Supplementary Table 9: a table showing the log2FC, average expression values, and statistical parameters of 16961 genes based on the comparison of NDUFB3low (25%, n = 127) with NDFFB3high (25%, n = 127). Supplementary Table 10: a table showing statistical parameters of each of the 18 enriched KEGG pathways based on the comparison of NDUFB3low with NDFFB3high. Supplementary Table 11: a table showing the score of each of the 80 genes in the OXPHOS pathway based on the comparison of NDUFB3low with NDFFB3high. [file 8038857.f2.zip › Supplementary table 10.pdf]

Gene Set	Description	Size	Leading Edge Number	ES	NES	P Value	**FDR**
hsa00190	Oxidative phosphorylation	117	80	0.83948	1.9163	0	0
hsa05012	Parkinson disease	125	74	0.81209	1.8594	0	0
hsa05010	Alzheimer disease	160	74	0.79204	1.8181	0	0
hsa05016	Huntington disease	183	80	0.76288	1.7599	0	0
hsa04714	Thermogenesis	209	93	0.75683	1.7496	0	0
hsa04932	Non-alcoholic fatty liver disease (NAFLD)	139	72	0.75801	1.737	0	0
hsa04672	Intestinal immune network for IgA production	44	44	-0.33246	-2.4531	0	0
hsa00620	Pyruvate metabolism	38	19	0.76776	1.6875	0	0.00027933
hsa03060	Protein export	23	14	0.77657	1.6559	0	0.0010863
hsa00860	Porphyrin and chlorophyll metabolism	25	13	0.76702	1.6314	0	0.0012221
hsa00280	Valine, leucine and isoleucine degradation	47	38	0.74749	1.6581	0	0.0012221
hsa05310	Asthma	25	25	-0.33209	-1.6275	0	0.25007
hsa00591	Linoleic acid metabolism	27	14	-0.26293	-1.1196	0.28571	0.76754
hsa04940	Type I diabetes mellitus	38	38	-0.17979	-1.0323	0.2	0.8031
hsa04742	Taste transduction	58	23	-0.14344	-1.1227	0	1
hsa05330	Allograft rejection	34	33	-0.22172	-0.90238	0.6	1
hsa01523	Antifolate resistance	31	31	-0.1624	-0.80932	0.71429	1
hsa03450	Non-homologous end-joining	13	13	-0.21565	-0.6944	0.82857	1
