# Supplementary material for: Clinical Relevance and Tumor Growth Suppression of Mitochondrial ROS Regulators along NADH:Ubiquinone Oxidoreductase Subunit B3 in Thyroid Cancer
Source: Oxid Med Cell Longev. 2022 Jan 17;2022:8038857. doi: 10.1155/2022/8038857 (PMC8787455; doi:10.1155/2022/8038857)
Supplement: Supplementary 2 — Supplementary Table 2: a table showing primer sequences and production lengths of human complex I subunit genes and MnSOD. Supplementary Table 3: a table showing the names and corresponding reference PubMed IDs of 35 mitoROS regulators. Supplementary Table 1: a table showing the cluster to which each TCGA sample ID belongs to the postconsensus clustering of 510 diagnosed thyroid cancer patients. Supplementary Table 4: a table showing the detailed clinical information of each TCGA sample within each cluster. Supplementary Table 5: a table showing the PFS duration of each TCGA sample within each cluster. Supplementary Table 6: a table showing the effect of high (50%) or low (50%) expression of each of the 35 mitoSOX regulators on PFS of 510 thyroid cancer patients in TCGA cohort. Supplementary Table 7: a table showing the exact expression value of each of the 35 mitoROS regulators for 510 TCGA samples. Supplementary Table 8: a table showing the detailed clinical information of each of 510 thyroid cancer samples divided into NDUFB3high and NDUFB3low. Supplementary Table 9: a table showing the log2FC, average expression values, and statistical parameters of 16961 genes based on the comparison of NDUFB3low (25%, n = 127) with NDFFB3high (25%, n = 127). Supplementary Table 10: a table showing statistical parameters of each of the 18 enriched KEGG pathways based on the comparison of NDUFB3low with NDFFB3high. Supplementary Table 11: a table showing the score of each of the 80 genes in the OXPHOS pathway based on the comparison of NDUFB3low with NDFFB3high. [file 8038857.f2.zip › Supplementary table 11.pdf]

User ID	**Score**	Gene Symbol	Gene Name	Entrez Gene ID
NDUFB3	179.3918	NDUFB3	NADH:ubiquinone oxidoreductase subunit B3	4709
ATP5PF	129.0456	ATP5PF	ATP synthase peripheral stalk subunit F6	522
NDUFB6	126.7139	NDUFB6	NADH:ubiquinone oxidoreductase subunit B6	4712
NDUFA12	120.2904	NDUFA12	NADH:ubiquinone oxidoreductase subunit A12	55967
COX7A2	113.3525	COX7A2	cytochrome c oxidase subunit 7A2	1347
NDUFB2	107.1703	NDUFB2	NADH:ubiquinone oxidoreductase subunit B2	4708
ATP5MC3	101.7195	ATP5MC3	ATP synthase membrane subunit c locus 3	518
NDUFA8	99.311	NDUFA8	NADH:ubiquinone oxidoreductase subunit A8	4702
UQCRH	98.6369	UQCRH	ubiquinol-cytochrome c reductase hinge protein	7388
ATP5F1C	98.3918	ATP5F1C	ATP synthase F1 subunit gamma	509
ATP5PD	97.9057	ATP5PD	ATP synthase peripheral stalk subunit d	10476
COX7B	94.7072	COX7B	cytochrome c oxidase subunit 7B	1349
ATP5MC1	93.2733	ATP5MC1	ATP synthase membrane subunit c locus 1	516
ATP5MG	93.2615	ATP5MG	ATP synthase membrane subunit g	10632
NDUFA1	93.1269	NDUFA1	NADH:ubiquinone oxidoreductase subunit A1	4694
COX8A	92.0201	COX8A	cytochrome c oxidase subunit 8A	1351
COX6C	91.9781	COX6C	cytochrome c oxidase subunit 6C	1345
NDUFS3	87.7395	NDUFS3	NADH:ubiquinone oxidoreductase core subunit S3	4722
NDUFA6	85.5989	NDUFA6	NADH:ubiquinone oxidoreductase subunit A6	4700
UQCR10	83.8508	UQCR10	ubiquinol-cytochrome c reductase, complex III subunit X	29796
NDUFB5	82.7434	NDUFB5	NADH:ubiquinone oxidoreductase subunit B5	4711
CYC1	81.2944	CYC1	cytochrome c1	1537
COX5A	80.5208	COX5A	cytochrome c oxidase subunit 5A	9377
ATP5F1E	78.9978	ATP5F1E	ATP synthase F1 subunit epsilon	514
NDUFC1	78.845	NDUFC1	NADH:ubiquinone oxidoreductase subunit C1	4717
NDUFB1	77.0647	NDUFB1	NADH:ubiquinone oxidoreductase subunit B1	4707
COX7C	76.794	COX7C	cytochrome c oxidase subunit 7C	1350
ATP5PO	76.6956	ATP5PO	ATP synthase peripheral stalk subunit OSCP	539
NDUFA5	76.6682	NDUFA5	NADH:ubiquinone oxidoreductase subunit A5	4698
UQCRQ	76.4465	UQCRQ	ubiquinol-cytochrome c reductase complex III subunit VII	27089
ATP5PB	76.3743	ATP5PB	ATP synthase peripheral stalk-membrane subunit b	515
NDUFC2	76.0013	NDUFC2	NADH:ubiquinone oxidoreductase subunit C2	4718
NDUFA4	73.4072	NDUFA4	NDUFA4, mitochondrial complex associated	4697
COX6A1	72.9782	COX6A1	cytochrome c oxidase subunit 6A1	1337
NDUFS4	72.1132	NDUFS4	NADH:ubiquinone oxidoreductase subunit S4	4724
NDUFB4	72.0865	NDUFB4	NADH:ubiquinone oxidoreductase subunit B4	4710
ATP5MF	70.6501	ATP5MF	ATP synthase membrane subunit f	9551

COX17	70.4861	COX17	cytochrome c oxidase copper chaperone COX17	10063
UQCRB	66.6475	UQCRB	ubiquinol-cytochrome c reductase binding protein	7381
NDUFS2	66.476	NDUFS2	NADH:ubiquinone oxidoreductase core subunit S2	4720
ATP5ME	65.714	ATP5ME	ATP synthase membrane subunit e	521
ATP5F1A	63.6938	ATP5F1A	ATP synthase F1 subunit alpha	498
UQCR11	63.5823	UQCR11	ubiquinol-cytochrome c reductase, complex III subunit XI	10975
NDUFAB1	60.482	NDUFAB1	NADH:ubiquinone oxidoreductase subunit AB1	4706
SDHD	59.8125	SDHD	succinate dehydrogenase complex subunit D	6392
SDHB	59.4534	SDHB	succinate dehydrogenase complex iron sulfur subunit B	6390
NDUFV3	59.406	NDUFV3	NADH:ubiquinone oxidoreductase subunit V3	4731
NDUFA10	58.0709	NDUFA10	NADH:ubiquinone oxidoreductase subunit A10	4705
COX4I1	56.5988	COX4I1	cytochrome c oxidase subunit 4I1	1327
UQCRFS1	56.4965	UQCRFS1	ubiquinol-cytochrome c reductase, Rieske iron-sulfur polypeptide 1	7386
ATP6V0B	55.7696	ATP6V0B	ATPase H+ transporting V0 subunit b	533
NDUFA9	55.748	NDUFA9	NADH:ubiquinone oxidoreductase subunit A9	4704
NDUFS6	55.3265	NDUFS6	NADH:ubiquinone oxidoreductase subunit S6	4726
NDUFB11	54.2217	NDUFB11	NADH:ubiquinone oxidoreductase subunit B11	54539
NDUFA2	53.831	NDUFA2	NADH:ubiquinone oxidoreductase subunit A2	4695
ATP6V0E1	52.3143	ATP6V0E1	ATPase H+ transporting V0 subunit e1	8992
COX5B	51.0715	COX5B	cytochrome c oxidase subunit 5B	1329
ATP5F1B	50.9523	ATP5F1B	ATP synthase F1 subunit beta	506
PPA2	48.321	PPA2	pyrophosphatase (inorganic) 2	27068
NDUFB7	45.4367	NDUFB7	NADH:ubiquinone oxidoreductase subunit B7	4713
ATP6V1D	45.1474	ATP6V1D	ATPase H+ transporting V1 subunit D	51382
NDUFB10	44.7972	NDUFB10	NADH:ubiquinone oxidoreductase subunit B10	4716
ATP6V0D1	44.1765	ATP6V0D1	ATPase H+ transporting V0 subunit d1	9114
UQCRC1	43.8835	UQCRC1	ubiquinol-cytochrome c reductase core protein 1	7384
ATP6V1G2	43.0584	ATP6V1G2	ATPase H+ transporting V1 subunit G2	534
ATP5MC2	42.9193	ATP5MC2	ATP synthase membrane subunit c locus 2	517
UQCRHL	42.7714	UQCRHL	ubiquinol-cytochrome c reductase hinge protein like	440567
NDUFB9	42.4705	NDUFB9	NADH:ubiquinone oxidoreductase subunit B9	4715
NDUFV1	41.7466	NDUFV1	NADH:ubiquinone oxidoreductase core subunit V1	4723
ATP6V1H	40.6885	ATP6V1H	ATPase H+ transporting V1 subunit H	51606
COX6B1	40.5669	COX6B1	cytochrome c oxidase subunit 6B1	1340
ATP6V0E2	40.1169	ATP6V0E2	ATPase H+ transporting V0 subunit e2	155066
LHPP	38.9279	LHPP	phospholysine phosphohistidine inorganic pyrophosphate phosphatase	64077
UQCRC2	38.7465	UQCRC2	ubiquinol-cytochrome c reductase core protein 2	7385
COX10	38.3561	COX10	cytochrome c oxidase assembly factor heme A:farnesyltransferase COX10	1352

NDUFS7	37.7786	NDUFS7	NADH:ubiquinone oxidoreductase core subunit S7	374291
COX7A2L	35.5097	COX7A2L	cytochrome c oxidase subunit 7A2 like	9167
NDUFV2	33.3902	NDUFV2	NADH:ubiquinone oxidoreductase core subunit V2	4729
NDUFB8	32.9587	NDUFB8	NADH:ubiquinone oxidoreductase subunit B8	4714
NDUFA3	32.4565	NDUFA3	NADH:ubiquinone oxidoreductase subunit A3	4696
