# Supplementary material for: Clinical Relevance and Tumor Growth Suppression of Mitochondrial ROS Regulators along NADH:Ubiquinone Oxidoreductase Subunit B3 in Thyroid Cancer
Source: Oxid Med Cell Longev. 2022 Jan 17;2022:8038857. doi: 10.1155/2022/8038857 (PMC8787455; doi:10.1155/2022/8038857)
Supplement: Supplementary 2 — Supplementary Table 2: a table showing primer sequences and production lengths of human complex I subunit genes and MnSOD. Supplementary Table 3: a table showing the names and corresponding reference PubMed IDs of 35 mitoROS regulators. Supplementary Table 1: a table showing the cluster to which each TCGA sample ID belongs to the postconsensus clustering of 510 diagnosed thyroid cancer patients. Supplementary Table 4: a table showing the detailed clinical information of each TCGA sample within each cluster. Supplementary Table 5: a table showing the PFS duration of each TCGA sample within each cluster. Supplementary Table 6: a table showing the effect of high (50%) or low (50%) expression of each of the 35 mitoSOX regulators on PFS of 510 thyroid cancer patients in TCGA cohort. Supplementary Table 7: a table showing the exact expression value of each of the 35 mitoROS regulators for 510 TCGA samples. Supplementary Table 8: a table showing the detailed clinical information of each of 510 thyroid cancer samples divided into NDUFB3high and NDUFB3low. Supplementary Table 9: a table showing the log2FC, average expression values, and statistical parameters of 16961 genes based on the comparison of NDUFB3low (25%, n = 127) with NDFFB3high (25%, n = 127). Supplementary Table 10: a table showing statistical parameters of each of the 18 enriched KEGG pathways based on the comparison of NDUFB3low with NDFFB3high. Supplementary Table 11: a table showing the score of each of the 80 genes in the OXPHOS pathway based on the comparison of NDUFB3low with NDFFB3high. [file 8038857.f2.zip › Supplementary table 2.pdf]

| Gene name | Forward primer | Reverse primer | Production length |
|---|---|---|---|
| NDUFV1 | ATGAAGGTGACAGCGTGAGG | TTCTTGGGTGCTGTCCGC | 100bp |
| NDUFV2 | TACGGCTGGAGTTTCAGAACTA[illegible] | CTGCTGGCATGCATTTGGAG | 214bp |
| NDUFV3 | AAAAAGCCAGCCCCAGTGC | AGGATGCAGTGAAGACGGTG | 209bp |
| NDUFS1 | ACCGTCCTCCAAGTTGTAGCTG | ACAGCACGCTTCCCCTCTAA | 230bp |
| NDUFS2 | TAGGATCACGGTCCTGGGAG | CCTGGAAACCAGCCACTAGG | 111bp |
| NDUFS3 | GTGGAGTTGGCCCAAGAGTT | GCTTCTTGTCTCCGGCTTCA | 112bp |
| NDUFS4 | GGATCTTTGTTCCTGCTCGC | TAGCTCCATCGTTGATGCCC | 128bp |
| NDUFS5 | GCATCGGCCATGCCTTTCTT | CGACCAGCCATCTTGTAGGG | 104bp |
| NDUFS6 | GTTCAGACAGCACCACCACT | CACCAGGAATACCCTTCGCA | 124bp |
| NDUFS7 | AGTGGCCTTGTCCTTTCTCAC | CGCAGGTTCAGAACAAAGGG | 96bp |
| NDUFS8 | GAAGTTGCTCAACAACGGGG | TCGGAGTGGTTTTATTGGGCA | 122bp |
| NDUFA1 | GTGGTTCGAGATTCTCCCCG | TTTCCTTGCCCCCGTTAGTG | 104bp |
| NDUFA2 | CGAATCCCGACCTACCCATC | AAGAGAGAGAGGGAGGTGTC | 113bp |
| NDUFA3 | CATCTGGGATGAGACCTAGCG | CTCAGCCTAGAAGTCGCTCC | 114bp |
| NDUFA4 | AGATCATCGGTCAGGCCAAG | GGACCCAGTTTGTTCCAGGG | 163bp |
| NDUFA5 | TGTGCTGAAGAAGAGGCTAAGA | GCCAGCTTCTCATTTGTAATC[illegible] | 117bp |
| NDUFA6 | GGATCAGGGCAGCACTAACA | GAACAGCTGTGCACCTAAGC | 176bp |
| NDUFA7 | CATCATGTCGTCGCAGAAGG | GAGGACAGCTCCCACCTCTT | 120bp |
| NDUFA8 | TTTGACGAGTGTGTGCTGGA | TCCACCAGGGCTTGAGTTTC | 144bp |
| NDUFA9 | CCTGTGGCTGGCTACTCTTC | GCAAAGACCCTTGCCTGGTA | 125bp |
| NDUFA10 | CTGCGATTACTCTGCCGGG | CCCTCCCCCGATCTTAAAGC | 176bp |
| NDUFA11 | GATGTCCACGCGTGTTCCAG | TGAGGGGCCCTTATTCCCAT | 287bp |
| NDUFA12 | TTTTTGGCCGTCACCGATGG | AAGCCAACGATGCCATTCAG | 107bp |
| NDUFA13 | ATACTGCGAGTATGGCGGC | GTGCCCGTAGATCAGGGTTC | 152bp |
| NDUFB1 | ACTTCAGATTGTGCGGGACC | GGCAGTTAGCCGTTCATCAC | 100bp |
| NDUFB2 | GTGCTGGGTCACTTTCCGTA | GGCTCTTGTACAGAGTGAGG[illegible] | 115bp |
| NDUFB3 | TGCTGTCAGGCAGAAGAACAG | CTTAGCCCTTTTGCAGCCAG | 159bp |
| NDUFB4 | GGATTTGGGCCCCTCATCTT | TCACTCTGACAGCAGATTCCC | 279bp |
| NDUFB5 | TTTCCGAAGGCTGCTGGTCA | AAGGACGGCCATTGTTCTTTC | 156bp |
| NDUFB6 | GAGGAAAATGGTCCATGGGGT | TCTGGACTTCTTTTCAACTAT[illegible] | 139bp |
| NDUFB7 | CCGGGTAGGAGCTAGGTGA | GTAGTCTGGCGGGAAGGTTG | 143bp |
| NDUFB8 | CTGGGTCTGACCAGAGGCTA | CTCATAGTGAACCACCCGCT | 207bp |

| | | | |
|---|---|---|---|
| NDUFB9 | GTAATGGCGTTCTTGGCGTC | CGGGCTCTCATCAAACAAGC | 141bp |
| NDUFB10 | AAAGCGTTCGACCTCATCGT | ACTCAGTGATGTCTGGCACG | 127bp |
| NDUFB11 | GACAAGGACCCCGTTTTGGA | CCATCCCACGCTCTTGGAC | 140bp |
| NDUFAB1 | TCGCGCAGGTTCCTGGTAG | GTCCTGGATGCCCTCTAACG | 83bp |
| NDUFC1 | AACTGACTACAAGGAGAAGGCT[illegible] | GGCTCTCGCACGTAGAACTT | 275bp |
| NDUFC2 | TCGCCAGCTTCTATATATTACGG | TCAGTGAAACTGGAGCAAGC | 219bp |
| MnSOD | CCACTGCTGGGGATTGATGT | CATTAGGGTATGATCAGCATA | 170bp |
