# Supplementary material for: Clinical Relevance and Tumor Growth Suppression of Mitochondrial ROS Regulators along NADH:Ubiquinone Oxidoreductase Subunit B3 in Thyroid Cancer
Source: Oxid Med Cell Longev. 2022 Jan 17;2022:8038857. doi: 10.1155/2022/8038857 (PMC8787455; doi:10.1155/2022/8038857)
Supplement: Supplementary 2 — Supplementary Table 2: a table showing primer sequences and production lengths of human complex I subunit genes and MnSOD. Supplementary Table 3: a table showing the names and corresponding reference PubMed IDs of 35 mitoROS regulators. Supplementary Table 1: a table showing the cluster to which each TCGA sample ID belongs to the postconsensus clustering of 510 diagnosed thyroid cancer patients. Supplementary Table 4: a table showing the detailed clinical information of each TCGA sample within each cluster. Supplementary Table 5: a table showing the PFS duration of each TCGA sample within each cluster. Supplementary Table 6: a table showing the effect of high (50%) or low (50%) expression of each of the 35 mitoSOX regulators on PFS of 510 thyroid cancer patients in TCGA cohort. Supplementary Table 7: a table showing the exact expression value of each of the 35 mitoROS regulators for 510 TCGA samples. Supplementary Table 8: a table showing the detailed clinical information of each of 510 thyroid cancer samples divided into NDUFB3high and NDUFB3low. Supplementary Table 9: a table showing the log2FC, average expression values, and statistical parameters of 16961 genes based on the comparison of NDUFB3low (25%, n = 127) with NDFFB3high (25%, n = 127). Supplementary Table 10: a table showing statistical parameters of each of the 18 enriched KEGG pathways based on the comparison of NDUFB3low with NDFFB3high. Supplementary Table 11: a table showing the score of each of the 80 genes in the OXPHOS pathway based on the comparison of NDUFB3low with NDFFB3high. [file 8038857.f2.zip › Supplementary table 3.pdf]

Gene	Entry	Entry name	Protein names	Pubmed_ID
ATP6V1E1	P36543	VATE1_HUMAN	V-type proton ATPase subunit E 1	PMID: 25704631
COX15	Q7KZN9	COX15_HUMAN	Cytochrome c oxidase assembly prote...	PMID: 25704631
COX5A	P20674	COX5A_HUMAN	Cytochrome c oxidase subunit 5A, mi...	PMID: 25704631
COX6B1	P14854	CX6B1_HUMAN	Cytochrome c oxidase subunit 6B1	PMID: 25704631
COX6C	P09669	COX6C_HUMAN	Cytochrome c oxidase subunit 6C	PMID: 25704631
COX7A2	P14406	CX7A2_HUMAN	Cytochrome c oxidase subunit 7A2, m...	PMID: 25704631
COX7b	P24311	COX7B_HUMAN	Cytochrome c oxidase subunit 7B, mi...	PMID: 25704631
COX8A	P10176	COX8A_HUMAN	Cytochrome c oxidase subunit 8A, mi...	PMID: 25704631
CYC1	P08574	CY1_HUMAN	Cytochrome c1, heme protein, mitoch...	PMID: 25704631
DLAT	P10515	ODP2_HUMAN	Dihydrolipoyllysine-residue acetylt...	PMID: 25704631
DUOX1	Q9NRD9	DUOX1_HUMAN	Dual oxidase 1	PMID: 30367082
DUOX2	Q9NRD8	DUOX2_HUMAN	Dual oxidase 2	PMID: 30367082
DUOXA1	Q1HG43	DOXA1_HUMAN	Dual oxidase maturation factor 1	PMID: 30367082
DUOXA2	Q1HG44	DOXA2_HUMAN	Dual oxidase maturation factor 2	PMID: 30367082
ETFB	P38117	ETFB_HUMAN	Electron transfer flavoprotein subu...	PMID: 25704631
NDUFA1	O15239	NDUA1_HUMAN	NADH dehydrogenase [ubiquinone] 1 a...	PMID: 25704631
NDUFA2	O43678	NDUA2_HUMAN	NADH dehydrogenase [ubiquinone] 1 a...	PMID: 25704631
NDUFA8	P51970	NDUA8_HUMAN	NADH dehydrogenase [ubiquinone] 1 a...	PMID: 25704631
NDUFB1	O75438	NDUB1_HUMAN	NADH dehydrogenase [ubiquinone] 1 b...	PMID: 25704631
NDUFB10	O96000	NDUBA_HUMAN	NADH dehydrogenase [ubiquinone] 1 b...	PMID: 25704631
NDUFB2	O95178	NDUB2_HUMAN	NADH dehydrogenase [ubiquinone] 1 b...	PMID: 25704631
NDUFB3	O43676	NDUB3_HUMAN	NADH dehydrogenase [ubiquinone] 1 b...	PMID: 25704631
NDUFB7	P17568	NDUB7_HUMAN	NADH dehydrogenase [ubiquinone] 1 b...	PMID: 25704631
NOX4	Q9NPH5	NOX4_HUMAN	NADPH oxidase 4	PMID: 30367082
PDC	P20941	PHOS_HUMAN	Phosducin	PMID: 25704631
ROMO1	P60602	ROMO1_HUMAN	Reactive oxygen species modulator 1	PMID:32825500
SCO1	O75880	SCO1_HUMAN	Protein SCO1 homolog, mitochondrial	PMID: 25704631
SLC25A5	P05141	ADT2_HUMAN	ADP/ATP translocase 2	PMID: 25704631
SOD2	P04179	SODM_HUMAN	Superoxide dismutase [Mn], mitochon...	PMID: 28445781
SUCLG2	Q96I99	SUCB2_HUMAN	Succinate--CoA ligase [GDP-forming]...	PMID: 25704631
SURF1	Q15526	SURF1_HUMAN	Surfeit locus protein 1	PMID: 25704631
UCP2	P55851	UCP2_HUMAN	Mitochondrial uncoupling protein 2	PMID: 25704631
UQCC3	Q6UW78	UQCC3_HUMAN	Ubiquinol-cytochrome-c reductase co...	PMID: 33147459
UQCR10	Q9UDW1	QCR9_HUMAN	Cytochrome b-c1 complex subunit 9	PMID: 25704631
UQCRQ	O14949	QCR8_HUMAN	Cytochrome b-c1 complex subunit 8	PMID: 25704631
