# Supplementary material for: Clinical Relevance and Tumor Growth Suppression of Mitochondrial ROS Regulators along NADH:Ubiquinone Oxidoreductase Subunit B3 in Thyroid Cancer
Source: Oxid Med Cell Longev. 2022 Jan 17;2022:8038857. doi: 10.1155/2022/8038857 (PMC8787455; doi:10.1155/2022/8038857)
Supplement: Supplementary 2 — Supplementary Table 2: a table showing primer sequences and production lengths of human complex I subunit genes and MnSOD. Supplementary Table 3: a table showing the names and corresponding reference PubMed IDs of 35 mitoROS regulators. Supplementary Table 1: a table showing the cluster to which each TCGA sample ID belongs to the postconsensus clustering of 510 diagnosed thyroid cancer patients. Supplementary Table 4: a table showing the detailed clinical information of each TCGA sample within each cluster. Supplementary Table 5: a table showing the PFS duration of each TCGA sample within each cluster. Supplementary Table 6: a table showing the effect of high (50%) or low (50%) expression of each of the 35 mitoSOX regulators on PFS of 510 thyroid cancer patients in TCGA cohort. Supplementary Table 7: a table showing the exact expression value of each of the 35 mitoROS regulators for 510 TCGA samples. Supplementary Table 8: a table showing the detailed clinical information of each of 510 thyroid cancer samples divided into NDUFB3high and NDUFB3low. Supplementary Table 9: a table showing the log2FC, average expression values, and statistical parameters of 16961 genes based on the comparison of NDUFB3low (25%, n = 127) with NDFFB3high (25%, n = 127). Supplementary Table 10: a table showing statistical parameters of each of the 18 enriched KEGG pathways based on the comparison of NDUFB3low with NDFFB3high. Supplementary Table 11: a table showing the score of each of the 80 genes in the OXPHOS pathway based on the comparison of NDUFB3low with NDFFB3high. [file 8038857.f2.zip › Supplementary table 4.pdf]

TCGA_ID	Age	Sex	Race	T	N	M	Stage	Grade	newTumor	Smoking	Radiation	Neoadjuvant	Therapy	OS	OS.time	DSS	DSS.time	DFI	DFI.time	PFI	PFI.time	Groups/Cluster
TCGA-DE-A4MD	71	MALE	WHITE	T3	N1	M0	IV	NA	Recurrence	NA	YES	No	NA	0	764	0	764	NA	NA	1	267	C1
TCGA-EM-A2P1	33	MALE	NA	T2	N1	NA	I	NA	Recurrence	NA	NA	No	NA	0	531	0	531	1	363	1	363	C1
TCGA-EM-A3SU	43	FEMALE	NA	T2	N1	NA	I	NA	NA	NA	YES	No	NA	0	911	0	911	0	911	0	911	C1
TCGA-4C-A93U	74	FEMALE	BLACK	T4	N1	M0	IV	NA	NA	NA	NO	No	NA	0	129	0	129	NA	NA	0	129	C1
TCGA-BJ-A0Z5	58	MALE	WHITE	T3	N1	M0	III	NA	NA	NA	NA	No	NA	0	448	0	448	NA	NA	0	448	C1
TCGA-BJ-A0Z9	57	FEMALE	WHITE	T3	N0	NA	III	NA	NA	NA	NA	No	NA	0	1257	0	1257	NA	NA	0	1257	C1
TCGA-BJ-A0ZB	66	MALE	WHITE	T3	N1	M0	IV	NA	NA	NA	NA	No	NA	0	122	0	122	NA	NA	0	122	C1
TCGA-BJ-A0ZH	52	FEMALE	WHITE	T2	N1	NA	IV	NA	NA	NA	NA	No	NA	0	460	0	460	NA	NA	0	460	C1
TCGA-BJ-A18Z	58	MALE	WHITE	T3	N1	NA	IV	NA	NA	NA	NA	No	NA	0	1263	0	1263	NA	NA	0	1263	C1
TCGA-BJ-A28R	38	FEMALE	WHITE	T1	N0	M0	I	NA	NA	NA	NA	No	NA	0	1282	0	1282	NA	NA	0	1282	C1
TCGA-BJ-A28T	34	FEMALE	WHITE	T1	N1	M0	I	NA	NA	NA	NA	No	NA	0	1081	0	1081	NA	NA	0	1081	C1
TCGA-BJ-A28X	32	FEMALE	WHITE	T3	N1	M0	I	NA	NA	NA	YES	No	NA	0	942	0	942	1	494	1	494	C1
TCGA-BJ-A28Z	46	FEMALE	WHITE	T3	N1	M0	III	NA	NA	NA	NA	No	NA	0	337	0	337	NA	NA	0	337	C1
TCGA-BJ-A2NA	77	MALE	WHITE	T3	N0	M0	III	NA	Metastasis	NA	NA	No	NA	0	577	0	577	NA	NA	1	127	C1
TCGA-BJ-A3EZ	51	MALE	WHITE	T3	N1	M0	IV	NA	NA	NA	NA	No	NA	0	487	0	487	NA	NA	0	487	C1
TCGA-BJ-A3PR	69	FEMALE	WHITE	T1	N0	M0	I	NA	NA	NA	NO	No	NA	0	1107	0	1107	0	1107	0	1107	C1
TCGA-BJ-A45E	46	FEMALE	WHITE	T1	N0	M0	I	NA	NA	NA	NO	No	NA	0	639	0	639	NA	NA	0	639	C1
TCGA-BJ-A45I	51	FEMALE	WHITE	T3	N0	M0	III	NA	NA	NA	YES	No	NA	0	470	0	470	NA	NA	1	110	C1
TCGA-BJ-A4O8	47	MALE	WHITE	T3	N1	M0	III	NA	NA	NA	YES	No	NA	0	591	0	591	0	591	0	591	C1
TCGA-DE-A0Y2	30	FEMALE	WHITE	T2	N1	M0	I	NA	NA	NA	NA	No	NA	0	658	0	658	0	658	0	658	C1
TCGA-DE-A0Y3	60	FEMALE	ASIAN	T4	N1	M1	IV	NA	Metastasis	NA	NA	No	NA	0	1526	0	1526	NA	NA	1	30	C1
TCGA-DE-A4M8	61	FEMALE	WHITE	T2	N1	M0	III	NA	NA	NA	YES	No	NA	0	564	0	564	0	564	0	564	C1
TCGA-DE-A4MB	79	FEMALE	WHITE	T3	N1	M0	IV	NA	NA	NA	YES	No	NA	0	352	0	352	NA	NA	0	352	C1
TCGA-DE-A4MC	43	FEMALE	AMERICAN INDIAN	T3	N1	M0	I	NA	Recurrence	NA	YES	No	NA	0	913	0	913	1	332	1	332	C1
TCGA-DE-A4MD.1	71	MALE	WHITE	T3	N1	M0	IV	NA	Recurrence	NA	YES	No	NA	0	764	0	764	NA	NA	1	267	C1
TCGA-DE-A7U5	36	FEMALE	BLACK	T2	N0	M0	I	NA	NA	NA	NO	No	NA	0	84	0	84	NA	NA	0	84	C1
TCGA-DJ-A13L	85	MALE	WHITE	T1	N0	M0	I	NA	NA	NA	NO	No	NA	0	303	0	303	0	303	0	303	C1
TCGA-DJ-A13R	50	MALE	WHITE	T3	N0	M0	III	NA	NA	NA	NA	No	NA	0	565	0	565	0	565	0	565	C1
TCGA-DJ-A13U	60	MALE	WHITE	T3	N0	M0	III	NA	NA	NA	NA	No	NA	0	1590	0	1590	0	1590	0	1590	C1
TCGA-DJ-A13W	45	FEMALE	WHITE	T1	N0	M0	I	NA	NA	NA	NA	No	NA	0	1593	0	1593	0	1593	0	1593	C1
TCGA-DJ-A1QD	20	FEMALE	WHITE	T2	N1	M0	I	NA	NA	NA	NA	No	NA	0	1462	0	1462	0	1462	0	1462	C1
TCGA-DJ-A1QE	62	FEMALE	WHITE	T3	N0	M0	III	NA	NA	NA	NA	No	NA	0	4134	0	4134	0	4134	0	4134	C1
TCGA-DJ-A1QF	61	FEMALE	WHITE	T2	N0	M0	II	NA	NA	NA	NA	No	NA	0	931	0	931	0	931	0	931	C1
TCGA-DJ-A1QG	62	MALE	WHITE	T1	N0	M0	I	NA	NA	NA	NA	No	NA	0	1368	0	1368	0	1368	0	1368	C1
TCGA-DJ-A1QO	69	MALE	WHITE	T3	N0	M0	III	NA	Primary	NA	NA	No	NA	0	1442	0	1442	0	1442	1	1062	C1
TCGA-DJ-A1QQ	43	MALE	ASIAN	T3	N0	M0	I	NA	NA	NA	NA	No	NA	0	534	0	534	0	534	0	534	C1
TCGA-DJ-A2PO	54	MALE	WHITE	T1	N0	M0	I	NA	NA	NA	NA	No	NA	0	243	0	243	0	243	0	243	C1
TCGA-DJ-A2PR	27	MALE	ASIAN	T3	N1	M0	I	NA	NA	NA	NA	No	NA	0	835	0	835	0	835	0	835	C1
TCGA-DJ-A2PV	53	FEMALE	WHITE	T2	N0	M0	II	NA	NA	NA	NA	No	NA	0	973	0	973	0	973	0	973	C1
TCGA-DJ-A2PY	46	FEMALE	WHITE	T3	N1	M0	III	NA	NA	NA	NA	No	NA	0	267	0	267	0	267	0	267	C1
TCGA-DJ-A2PZ	63	MALE	WHITE	T3	N0	M0	III	NA	NA	NA	NA	No	NA	0	256	0	256	0	256	0	256	C1
TCGA-DJ-A2Q1	44	FEMALE	WHITE	T1	N1	M0	I	NA	NA	NA	NA	No	NA	0	1148	0	1148	0	1148	0	1148	C1
TCGA-DJ-A2Q3	61	FEMALE	ASIAN	T3	N1	M0	III	NA	Primary	NA	NA	No	NA	0	1224	0	1224	0	1224	1	385	C1
TCGA-DJ-A2Q4	53	MALE	WHITE	T1	N1	M0	III	NA	NA	NA	NA	No	NA	0	1032	0	1032	0	1032	0	1032	C1
TCGA-DJ-A2Q9	65	FEMALE	WHITE	T1	N1	M0	III	NA	NA	NA	NA	No	NA	0	1092	0	1092	0	1092	0	1092	C1
TCGA-DJ-A3UQ	45	FEMALE	ASIAN	T3	N1	M0	III	NA	NA	NA	YES	No	NA	0	595	0	595	0	595	0	595	C1
TCGA-DJ-A3UW	50	FEMALE	WHITE	T1	N0	M0	I	NA	NA	NA	NO	No	NA	0	1812	0	1812	0	1812	0	1812	C1
TCGA-DJ-A3UY	32	FEMALE	WHITE	T1	N0	M0	I	NA	NA	NA	NO	No	NA	0	1091	0	1091	0	1091	0	1091	C1
TCGA-DJ-A3V5	76	FEMALE	WHITE	T3	N0	M0	III	NA	NA	NA	YES	No	NA	0	584	0	584	NA	NA	0	584	C1
TCGA-DJ-A3V6	54	MALE	WHITE	T3	N0	M0	III	NA	NA	NA	YES	Yes	NA	0	818	0	818	0	818	0	818	C1
TCGA-DJ-A3VB	52	MALE	NA	T3	N1	M0	III	NA	NA	NA	YES	No	NA	0	697	0	697	0	697	0	697	C1
TCGA-DJ-A3VD	32	FEMALE	WHITE	T1	N1	M0	I	NA	NA	NA	YES	No	NA	0	1238	0	1238	0	1238	0	1238	C1
TCGA-DJ-A3VE	50	MALE	WHITE	T1	N0	M0	I	NA	NA	NA	NO	No	NA	0	577	0	577	0	577	0	577	C1
TCGA-DJ-A3VF	60	FEMALE	WHITE	T3	N1	M0	IV	NA	NA	NA	YES	No	NA	0	292	0	292	NA	NA	0	292	C1
TCGA-DJ-A3VI	52	FEMALE	WHITE	T1	N1	M0	III	NA	NA	NA	NO	No	NA	0	95	0	95	0	95	0	95	C1
TCGA-DJ-A4UT	32	FEMALE	WHITE	T1	N0	M0	I	NA	NA	NA	NO	No	NA	0	823	0	823	0	823	0	823	C1
TCGA-DJ-A4V2	38	FEMALE	NA	T2	N0	M0	I	NA	NA	NA	NO	No	NA	0	95	0	95	0	95	0	95	C1

TCGA-DJ-A4V4	48	FEMALE	WHITE	T1	N0	M0	I	NA	NA	NA	NO	No	NA	0	481	0	481	0	481	0	481	C1
TCGA-DO-A1K0	30	FEMALE	WHITE	T3	N1	NA	I	NA	Recurrence	NA	NA	No	Hormone Therapy	0	655	0	655	1	577	1	577	C1
TCGA-DO-A2HM	49	MALE	WHITE	T1	N1	NA	IV	NA	NA	NA	NA	No	NA	0	71	0	71	NA	NA	0	71	C1
TCGA-E8-A242	56	FEMALE	WHITE	T3	N0	NA	III	NA	NA	NA	NA	No	Hormone Therapy	0	950	0	950	NA	NA	0	950	C1
TCGA-E8-A2EA	52	FEMALE	WHITE	T1	N0	M0	I	NA	NA	NA	NA	No	NA	0	1085	0	1085	0	1085	0	1085	C1
TCGA-E8-A2JQ	18	FEMALE	WHITE	T3	N1	M0	I	NA	NA	NA	NA	No	Hormone Therapy	0	356	0	356	NA	NA	0	356	C1
TCGA-E8-A413	38	FEMALE	ASIAN	T1	N0	M0	I	NA	NA	NA	NA	No	NA	0	912	0	912	0	912	0	912	C1
TCGA-E8-A414	45	FEMALE	ASIAN	T3	N0	M0	III	NA	NA	NA	NA	No	NA	0	958	0	958	0	958	0	958	C1
TCGA-E8-A417	38	FEMALE	ASIAN	T2	N1	M0	I	NA	NA	NA	NA	No	NA	0	938	0	938	0	938	0	938	C1
TCGA-E8-A419	30	FEMALE	WHITE	T2	N1	M0	I	NA	NA	NA	NA	No	NA	0	774	0	774	0	774	0	774	C1
TCGA-E8-A436	53	FEMALE	ASIAN	T2	N1	M0	IV	NA	NA	NA	NA	No	NA	0	455	0	455	NA	NA	0	455	C1
TCGA-E8-A44K	50	FEMALE	ASIAN	T1	N0	M0	I	NA	NA	NA	NO	No	NA	0	943	0	943	0	943	0	943	C1
TCGA-EL-A3CM	64	FEMALE	WHITE	T3	N1	M0	III	NA	NA	NA	YES	No	NA	1	813	NA	813	NA	NA	0	813	C1
TCGA-EL-A3CN	47	FEMALE	WHITE	T3	N0	M0	II	NA	NA	NA	YES	No	NA	0	4731	0	4731	NA	NA	0	4731	C1
TCGA-EL-A3CO	88	MALE	WHITE	T3	N1	M0	III	NA	NA	NA	YES	No	NA	1	2973	NA	2973	NA	NA	0	2973	C1
TCGA-EL-A3CS	62	FEMALE	WHITE	T4	N0	M0	IV	NA	NA	NA	YES	Yes	NA	1	1385	1	1385	NA	NA	1	1385	C1
TCGA-EL-A3CT	78	FEMALE	WHITE	T4	N1	M0	IV	NA	NA	NA	NO	No	NA	1	1854	NA	1854	NA	NA	0	1854	C1
TCGA-EL-A3CU	70	FEMALE	WHITE	T1	N1	M0	III	NA	NA	NA	YES	No	NA	0	4780	0	4780	0	4780	0	4780	C1
TCGA-EL-A3CV	40	MALE	WHITE	T3	N1	M0	I	NA	NA	NA	YES	No	NA	0	1154	0	1154	NA	NA	0	1154	C1
TCGA-EL-A3CY	28	MALE	WHITE	NA	N1	M0	I	NA	NA	NA	NO	Yes	NA	0	127	0	127	0	127	0	127	C1
TCGA-EL-A3D0	61	MALE	WHITE	T4	N1	M0	III	NA	NA	NA	YES	No	NA	0	4254	0	4254	NA	NA	0	4254	C1
TCGA-EL-A3D6	51	FEMALE	WHITE	T3	N1	M0	III	NA	NA	NA	YES	No	NA	0	3296	0	3296	NA	NA	0	3296	C1
TCGA-EL-A3GO	31	FEMALE	WHITE	T2	N0	M0	I	NA	Recurrence	NA	NO	No	NA	0	1973	0	1973	1	6	1	6	C1
TCGA-EL-A3GP	70	MALE	BLACK	T3	N1	M0	III	NA	NA	NA	NO	No	NA	0	1506	0	1506	0	1506	0	1506	C1
TCGA-EL-A3GQ	72	FEMALE	WHITE	T1	N0	M0	I	NA	NA	NA	NO	No	NA	0	1528	0	1528	0	1528	0	1528	C1
TCGA-EL-A3GR	31	FEMALE	WHITE	T3	NA	M0	I	NA	NA	NA	YES	No	NA	0	3501	0	3501	0	3501	0	3501	C1
TCGA-EL-A3GS	37	FEMALE	WHITE	T3	N1	M0	I	NA	NA	NA	YES	No	NA	0	1989	0	1989	0	1989	0	1989	C1
TCGA-EL-A3GU	72	FEMALE	WHITE	T3	N1	M0	III	NA	Metastasis	NA	NO	No	NA	0	4137	0	4137	1	544	1	544	C1
TCGA-EL-A3GV	65	FEMALE	WHITE	T3	N0	M0	III	NA	NA	NA	YES	No	NA	0	1532	0	1532	0	1532	0	1532	C1
TCGA-EL-A3GX	41	FEMALE	WHITE	T2	N1	M0	I	NA	Recurrence	NA	YES	No	NA	0	4494	0	4494	1	439	1	439	C1
TCGA-EL-A3GY	40	FEMALE	WHITE	T1	N1	M1	II	NA	NA	NA	YES	No	NA	0	3076	0	3076	0	3076	0	3076	C1
TCGA-EL-A3H4	46	FEMALE	BLACK	T4	N1	M0	IV	NA	NA	NA	YES	No	NA	0	1512	0	1512	NA	NA	0	1512	C1
TCGA-EL-A3H5	57	FEMALE	WHITE	T4	N1	M0	IV	NA	NA	NA	YES	No	NA	0	2542	0	2542	NA	NA	0	2542	C1
TCGA-EL-A3H7	36	FEMALE	WHITE	T3	N1	M0	I	NA	NA	NA	NO	No	NA	0	4228	0	4228	0	4228	0	4228	C1
TCGA-EL-A3MY	81	MALE	WHITE	T4	NA	M1	IV	NA	NA	NA	YES	No	NA	1	533	1	533	NA	NA	1	533	C1
TCGA-EL-A3MZ	66	MALE	WHITE	T4	N1	NA	III	NA	NA	NA	YES	No	NA	1	1597	0	1597	NA	NA	0	1597	C1
TCGA-EL-A3N2	24	FEMALE	WHITE	T2	N1	M0	I	NA	NA	NA	YES	No	NA	0	902	0	902	0	902	0	902	C1
TCGA-EL-A3N3	53	FEMALE	WHITE	T3	NA	M0	II	NA	NA	NA	YES	No	NA	0	5423	0	5423	0	5423	0	5423	C1
TCGA-EL-A3T1	38	FEMALE	BLACK	T2	N0	M0	I	NA	NA	NA	NO	No	NA	0	3941	0	3941	0	3941	0	3941	C1
TCGA-EL-A3T3	63	MALE	WHITE	T2	N0	M0	II	NA	NA	NA	YES	No	NA	0	4017	0	4017	0	4017	0	4017	C1
TCGA-EL-A3T6	34	FEMALE	WHITE	T3	N0	M0	I	NA	NA	NA	YES	No	NA	0	2988	0	2988	0	2988	0	2988	C1
TCGA-EL-A3T9	69	FEMALE	WHITE	T4	N1	NA	IV	NA	NA	NA	NA	No	NA	1	174	1	174	NA	NA	1	174	C1
TCGA-EL-A3ZK	41	FEMALE	WHITE	T2	N1	M0	I	NA	NA	NA	YES	No	NA	0	1575	0	1575	0	1575	0	1575	C1
TCGA-EL-A3ZO	79	FEMALE	WHITE	T3	N1	M0	III	NA	NA	NA	YES	No	NA	0	2211	0	2211	0	2211	0	2211	C1
TCGA-EL-A3ZT	35	MALE	WHITE	T3	N0	M0	I	NA	NA	NA	YES	No	NA	0	1808	0	1808	0	1808	0	1808	C1
TCGA-EL-A4JW	38	FEMALE	WHITE	T1	N0	M0	I	NA	NA	NA	YES	No	NA	0	1056	0	1056	0	1056	0	1056	C1
TCGA-EL-A4JX	31	FEMALE	BLACK	T3	N0	NA	I	NA	NA	NA	NO	No	NA	0	1398	0	1398	0	1398	0	1398	C1
TCGA-EL-A4K1	68	FEMALE	WHITE	T3	N1	M0	III	NA	NA	NA	NO	No	NA	0	1046	0	1046	0	1046	0	1046	C1
TCGA-EL-A4K4	33	FEMALE	WHITE	T3	N1	M0	I	NA	NA	NA	YES	No	NA	0	1623	0	1623	0	1623	0	1623	C1
TCGA-EM-A1CT	76	MALE	NA	T1	N1	NA	IV	NA	NA	NA	NA	No	NA	0	553	0	553	NA	NA	0	553	C1
TCGA-EM-A22I	52	FEMALE	NA	T3	N0	NA	III	NA	NA	NA	NA	No	NA	0	448	0	448	NA	NA	0	448	C1
TCGA-EM-A22M	52	MALE	ASIAN	T1	N0	NA	I	NA	NA	NA	NA	No	NA	0	442	0	442	0	442	0	442	C1
TCGA-EM-A2CP	26	FEMALE	NA	T2	N0	NA	I	NA	NA	NA	NA	No	NA	0	91	0	91	0	91	0	91	C1
TCGA-EM-A2P1.1	33	MALE	NA	T2	N1	NA	I	NA	Recurrence	NA	NA	No	NA	0	531	0	531	1	363	1	363	C1
TCGA-EM-A3FK	30	FEMALE	NA	T1	N1	NA	I	NA	NA	NA	NA	No	NA	0	723	0	723	0	723	0	723	C1
TCGA-EM-A3FM	57	MALE	NA	T2	N1	NA	IV	NA	Recurrence	NA	NA	No	NA	0	639	0	639	NA	NA	1	514	C1
TCGA-EM-A3O3	83	FEMALE	NA	T2	N1	NA	III	NA	NA	NA	YES	No	NA	0	441	0	441	0	441	0	441	C1
TCGA-EM-A3SZ	42	FEMALE	NA	T2	N0	NA	I	NA	NA	NA	NO	No	NA	0	389	0	389	0	389	0	389	C1

TCGA-EM-A4FF	40	FEMALE	NA	T1	N1	NA	I	NA	Recurrence	NA	YES	No	NA	0	2573	0	2573	NA	NA	1	1785	C1
TCGA-EM-A4FM	59	FEMALE	NA	T3	N1	NA	IV	NA	NA	NA	YES	No	NA	0	162	0	162	NA	NA	0	162	C1
TCGA-EM-A4FQ	42	FEMALE	NA	T2	N1	NA	I	NA	NA	NA	YES	No	NA	0	683	0	683	0	683	0	683	C1
TCGA-ET-A25G	56	FEMALE	WHITE	T3	N0	NA	III	NA	NA	NA	NA	No	NA	0	2492	0	2492	0	2492	0	2492	C1
TCGA-ET-A25L	52	FEMALE	WHITE	T3	N1	NA	IV	NA	NA	NA	NA	No	NA	0	1721	0	1721	NA	NA	0	1721	C1
TCGA-ET-A25N	28	FEMALE	WHITE	T2	N1	NA	I	NA	NA	NA	NA	No	NA	0	1928	0	1928	0	1928	0	1928	C1
TCGA-ET-A25R	51	FEMALE	WHITE	T3	NA	NA	III	NA	NA	NA	NA	No	NA	0	1930	0	1930	0	1930	0	1930	C1
TCGA-ET-A39O	39	MALE	WHITE	T1	N0	NA	I	NA	NA	NA	NA	No	NA	0	3059	0	3059	0	3059	0	3059	C1
TCGA-ET-A3BN	33	FEMALE	ASIAN	T2	NA	NA	I	NA	NA	NA	NA	No	NA	0	859	0	859	0	859	0	859	C1
TCGA-ET-A3BO	44	FEMALE	WHITE	T3	N1	NA	I	NA	NA	NA	NA	No	NA	0	1533	0	1533	NA	NA	0	1533	C1
TCGA-ET-A3BS	42	MALE	ASIAN	T3	N1	NA	I	NA	NA	NA	NA	No	NA	0	176	0	176	0	176	0	176	C1
TCGA-ET-A3BV	47	FEMALE	WHITE	T2	N1	NA	III	NA	Recurrence	NA	NA	No	NA	0	2294	0	2294	1	1204	1	1204	C1
TCGA-ET-A3BX	24	MALE	WHITE	T3	N1	NA	I	NA	NA	NA	NA	No	NA	0	1596	0	1596	0	1596	0	1596	C1
TCGA-ET-A3DO	33	FEMALE	WHITE	T2	NA	NA	I	NA	Recurrence	NA	NA	No	NA	0	3552	0	3552	1	570	1	570	C1
TCGA-ET-A3DU	21	FEMALE	WHITE	T1	N1	NA	I	NA	NA	NA	NA	No	NA	0	1911	0	1911	NA	NA	0	1911	C1
TCGA-ET-A3DW	64	MALE	ASIAN	T2	N1	NA	III	NA	NA	NA	NA	No	NA	0	752	0	752	0	752	0	752	C1
TCGA-ET-A40Q	38	MALE	WHITE	T3	N1	NA	I	NA	Metastasis	NA	YES	No	NA	0	1407	0	1407	1	218	1	218	C1
TCGA-FE-A22Z	61	FEMALE	WHITE	T4	N1	NA	IV	NA	Recurrence	NA	NA	No	NA	0	2243	0	2243	NA	NA	1	1215	C1
TCGA-FE-A230	30	FEMALE	WHITE	T3	N1	NA	I	NA	Recurrence	NA	NA	No	NA	0	2173	0	2173	1	526	1	526	C1
TCGA-FE-A233	18	FEMALE	WHITE	T2	NA	NA	I	NA	Recurrence	NA	NA	No	NA	0	751	0	751	1	567	1	567	C1
TCGA-FE-A234	26	FEMALE	WHITE	T2	N1	NA	I	NA	Recurrence	NA	NA	No	NA	0	2075	0	2075	1	483	1	483	C1
TCGA-FE-A23A	27	FEMALE	WHITE	T2	N0	NA	I	NA	NA	NA	NA	No	NA	0	2489	0	2489	0	2489	0	2489	C1
TCGA-FE-A3PB	33	FEMALE	WHITE	T3	N1	NA	I	NA	NA	NA	NA	No	NA	0	2590	0	2590	0	2590	0	2590	C1
TCGA-FY-A3BL	29	MALE	WHITE	T1	NA	M0	I	NA	NA	NA	NA	No	NA	0	621	0	621	0	621	0	621	C1
TCGA-FY-A3NN	48	FEMALE	NA	T1	N1	NA	III	NA	NA	NA	NA	No	NA	0	0	0	0	0	0	0	0	C1
TCGA-FY-A3R8	62	FEMALE	WHITE	T3	NA	NA	III	NA	NA	NA	YES	No	Hormone Therapy	0	477	0	477	0	477	0	477	C1
TCGA-FY-A3TY	61	FEMALE	WHITE	T3	N0	NA	III	NA	NA	NA	NA	No	NA	0	709	0	709	0	709	0	709	C1
TCGA-FY-A3YR	64	FEMALE	WHITE	T3	N1	NA	III	NA	NA	NA	YES	No	Hormone Therapy	0	528	0	528	0	528	0	528	C1
TCGA-FY-A40L	55	FEMALE	WHITE	T4	N1	NA	IV	NA	NA	NA	YES	No	Hormone Therapy	0	726	0	726	NA	NA	0	726	C1
TCGA-FY-A4B3	51	MALE	WHITE	T3	N1	NA	III	NA	NA	NA	NA	No	NA	0	427	0	427	NA	NA	0	427	C1
TCGA-H2-A26U	54	FEMALE	WHITE	T3	N0	NA	III	NA	NA	NA	NA	No	NA	0	517	0	517	NA	NA	0	517	C1
TCGA-H2-A421	34	FEMALE	WHITE	T1	N1	M0	I	NA	NA	NA	YES	No	NA	0	610	0	610	NA	NA	0	610	C1
TCGA-H2-A422	40	FEMALE	WHITE	T2	N0	NA	I	NA	NA	NA	YES	No	NA	0	566	0	566	NA	NA	0	566	C1
TCGA-IM-A3EB	32	FEMALE	WHITE	T3	N1	NA	I	NA	NA	NA	NA	No	NA	0	1488	0	1488	0	1488	0	1488	C1
TCGA-IM-A3ED	58	FEMALE	WHITE	T1	N0	NA	I	NA	NA	NA	NA	No	Hormone Therapy	0	1442	0	1442	0	1442	0	1442	C1
TCGA-IM-A3U2	89	FEMALE	WHITE	T3	N1	NA	IV	NA	NA	NA	NA	No	NA	0	563	0	563	NA	NA	0	563	C1
TCGA-IM-A420	39	FEMALE	WHITE	T2	N1	NA	I	NA	NA	NA	NO	No	NA	0	250	0	250	0	250	0	250	C1
TCGA-IM-A4EB	37	MALE	WHITE	T3	N1	NA	I	NA	NA	NA	YES	No	NA	0	780	0	780	0	780	0	780	C1
TCGA-J8-A3O1	33	FEMALE	WHITE	T3	N1	M0	I	NA	NA	NA	NA	No	NA	0	827	0	827	0	827	0	827	C1
TCGA-J8-A3YF	83	MALE	WHITE	T1	N1	M0	III	NA	NA	NA	YES	No	NA	0	751	0	751	0	751	0	751	C1
TCGA-J8-A3YG	54	FEMALE	WHITE	T2	N1	M0	III	NA	NA	NA	YES	No	NA	0	678	0	678	0	678	0	678	C1
TCGA-KS-A41I	47	FEMALE	WHITE	T1	N0	M0	I	NA	NA	NA	YES	No	Hormone Therapy	0	486	0	486	0	486	0	486	C1
TCGA-KS-A4I9	46	FEMALE	BLACK	T1	N0	M0	I	NA	NA	NA	YES	No	Hormone Therapy	0	2658	0	2658	0	2658	0	2658	C1
TCGA-KS-A4IB	41	FEMALE	ASIAN	T2	N1	M0	I	NA	NA	NA	YES	No	Hormone Therapy	0	2577	0	2577	0	2577	0	2577	C1
TCGA-KS-A4ID	55	FEMALE	WHITE	T1	N0	M0	I	NA	NA	NA	NO	No	Hormone Therapy	0	1011	0	1011	0	1011	0	1011	C1
TCGA-L6-A4EU	58	FEMALE	BLACK	T3	N1	M0	III	NA	NA	NA	YES	No	NA	0	289	0	289	NA	NA	0	289	C1
TCGA-MK-A4N6	35	MALE	WHITE	T3	N1	M0	I	NA	NA	NA	YES	No	Hormone Therapy	0	1098	0	1098	0	1098	0	1098	C1
TCGA-EM-A2CS	51	FEMALE	NA	T3	N1	NA	IV	NA	NA	NA	NA	No	NA	0	841	0	841	NA	NA	0	841	C2
TCGA-J8-A3O2	39	MALE	WHITE	T3	N1	M0	I	NA	NA	NA	NA	No	NA	0	342	0	342	NA	NA	0	342	C2
TCGA-J8-A3YH	39	MALE	WHITE	T3	N1	M0	I	NA	NA	NA	YES	No	NA	0	175	0	175	0	175	0	175	C2
TCGA-J8-A4HW	59	FEMALE	WHITE	T2	N1	M0	III	NA	NA	NA	YES	No	NA	0	79	0	79	0	79	0	79	C2
TCGA-BJ-A0Z0	55	MALE	NA	T2	N0	NA	II	NA	NA	NA	NA	No	NA	0	419	0	419	0	419	0	419	C2
TCGA-BJ-A0Z2	57	MALE	BLACK	T2	N0	M1	IV	NA	Metastasis	NA	NA	No	NA	0	1514	0	1514	NA	NA	1	158	C2
TCGA-BJ-A0Z3	33	FEMALE	WHITE	T1	N0	M0	I	NA	NA	NA	NA	No	NA	0	497	0	497	NA	NA	0	497	C2
TCGA-BJ-A0ZA	67	FEMALE	WHITE	T2	N0	M0	II	NA	NA	NA	NA	No	NA	0	574	0	574	0	574	0	574	C2
TCGA-BJ-A0ZC	55	MALE	WHITE	T1	N0	M0	I	NA	NA	NA	NA	No	NA	0	352	0	352	0	352	0	352	C2
TCGA-BJ-A0ZE	63	FEMALE	WHITE	T3	N0	M0	III	NA	NA	NA	NA	No	NA	0	509	0	509	NA	NA	0	509	C2
TCGA-BJ-A0ZG	80	MALE	WHITE	T1	N0	M0	I	NA	NA	NA	NA	No	NA	0	775	0	775	NA	NA	0	775	C2

TCGA-BJ-A0ZJ	36	MALE	WHITE	T2	N1	M0	I	NA	NA	NA	NA	No	NA	0	1280	0	1280	0	1280	0	1280	C2
TCGA-BJ-A18Y	29	MALE	WHITE	T1	N0	NA	I	NA	NA	NA	NA	No	NA	0	1563	0	1563	0	1563	0	1563	C2
TCGA-BJ-A190	55	MALE	WHITE	T2	N0	NA	II	NA	NA	NA	NA	No	NA	0	626	0	626	0	626	0	626	C2
TCGA-BJ-A191	49	FEMALE	WHITE	T1	N0	M0	I	NA	NA	NA	NA	No	NA	0	147	0	147	0	147	0	147	C2
TCGA-BJ-A28S	79	MALE	WHITE	T2	N0	M0	II	NA	NA	NA	NA	No	NA	0	640	0	640	NA	NA	0	640	C2
TCGA-BJ-A290	70	MALE	WHITE	T3	N1	M0	III	NA	Metastasis	NA	NA	No	NA	0	919	0	919	NA	NA	1	329	C2
TCGA-BJ-A2N7	30	FEMALE	WHITE	T3	N0	M0	I	NA	NA	NA	YES	No	Hormone Therapy	0	559	0	559	0	559	0	559	C2
TCGA-BJ-A45C	78	MALE	BLACK	T3	N0	M0	III	NA	NA	NA	YES	No	NA	1	243	1	243	NA	NA	1	243	C2
TCGA-BJ-A45F	59	FEMALE	WHITE	T1	N0	M0	I	NA	NA	NA	YES	No	NA	0	105	0	105	NA	NA	0	105	C2
TCGA-BJ-A45G	48	FEMALE	WHITE	T2	N1	M0	III	NA	NA	NA	YES	No	NA	0	725	0	725	NA	NA	0	725	C2
TCGA-BJ-A45H	45	MALE	WHITE	T3	N0	M0	III	NA	NA	NA	YES	No	NA	0	865	0	865	NA	NA	0	865	C2
TCGA-BJ-A45J	39	FEMALE	WHITE	T1	N1	M0	I	NA	NA	NA	YES	No	NA	0	658	0	658	NA	NA	0	658	C2
TCGA-BJ-A4O9	51	FEMALE	WHITE	T2	N0	M0	II	NA	NA	NA	NO	No	NA	0	714	0	714	NA	NA	0	714	C2
TCGA-CE-A13K	30	FEMALE	ASIAN	T3	N1	M0	I	NA	NA	NA	NA	No	NA	0	385	0	385	0	385	0	385	C2
TCGA-CE-A27D	28	FEMALE	ASIAN	T2	N1	M0	I	NA	NA	NA	NA	No	NA	0	374	0	374	0	374	0	374	C2
TCGA-CE-A3ME	51	FEMALE	ASIAN	T2	N0	M0	II	NA	NA	NA	NA	No	NA	0	379	0	379	0	379	0	379	C2
TCGA-CE-A481	41	FEMALE	ASIAN	T1	N0	M0	I	NA	NA	NA	NA	No	Chemotherapy	0	346	0	346	0	346	0	346	C2
TCGA-CE-A482	27	FEMALE	ASIAN	T1	N1	M0	I	NA	NA	NA	NO	No	NA	0	325	0	325	0	325	0	325	C2
TCGA-CE-A483	34	FEMALE	ASIAN	T2	N1	M0	I	NA	NA	NA	NO	No	NA	0	457	0	457	0	457	0	457	C2
TCGA-CE-A485	32	MALE	ASIAN	T2	N1	M0	I	NA	NA	NA	NO	No	NA	0	385	0	385	0	385	0	385	C2
TCGA-DE-A0XZ	65	FEMALE	WHITE	T1	N1	M0	III	NA	NA	NA	NA	No	NA	0	1090	0	1090	1	204	1	204	C2
TCGA-DE-A3KN	49	FEMALE	WHITE	T3	N1	M0	IV	NA	NA	NA	NA	No	NA	0	831	0	831	NA	NA	0	831	C2
TCGA-DE-A4M9	28	MALE	WHITE	T2	NA	M0	I	NA	NA	NA	YES	No	NA	0	573	0	573	NA	NA	0	573	C2
TCGA-DE-A69J	34	FEMALE	BLACK	T2	NA	M0	I	NA	NA	NA	YES	No	NA	0	447	0	447	0	447	0	447	C2
TCGA-DE-A69K	58	FEMALE	BLACK	T3	N0	M0	III	NA	Primary	NA	YES	No	NA	0	434	0	434	NA	NA	1	434	C2
TCGA-DJ-A13O	56	MALE	WHITE	T1	N0	M0	I	NA	NA	NA	NA	No	NA	0	1161	0	1161	0	1161	0	1161	C2
TCGA-DJ-A13P	52	FEMALE	WHITE	T2	N0	M0	II	NA	NA	NA	NA	No	NA	0	5150	0	5150	0	5150	0	5150	C2
TCGA-DJ-A13S	19	FEMALE	WHITE	T1	N0	M0	I	NA	NA	NA	NA	No	NA	0	265	0	265	0	265	0	265	C2
TCGA-DJ-A13T	37	FEMALE	WHITE	T2	N0	M0	I	NA	NA	NA	NA	No	NA	0	1709	0	1709	0	1709	0	1709	C2
TCGA-DJ-A13V	21	FEMALE	NA	T3	N1	M0	I	NA	NA	NA	NA	No	NA	0	1313	0	1313	0	1313	0	1313	C2
TCGA-DJ-A13X	51	FEMALE	NA	T3	N0	M0	III	NA	NA	NA	NA	No	NA	0	253	0	253	0	253	0	253	C2
TCGA-DJ-A1QH	58	FEMALE	WHITE	T3	N1	M0	III	NA	NA	NA	NA	Yes	NA	0	1293	0	1293	NA	NA	0	1293	C2
TCGA-DJ-A1QI	63	FEMALE	WHITE	T2	N0	M0	II	NA	NA	NA	NA	No	NA	0	455	0	455	0	455	0	455	C2
TCGA-DJ-A1QM	42	MALE	WHITE	T1	N0	M0	I	NA	NA	NA	NA	No	NA	0	183	0	183	0	183	0	183	C2
TCGA-DJ-A1QN	42	FEMALE	NA	T1	N0	M0	I	NA	NA	NA	NA	No	NA	0	1295	0	1295	0	1295	0	1295	C2
TCGA-DJ-A2PN	70	FEMALE	WHITE	T1	N0	M0	I	NA	NA	NA	NA	No	NA	0	307	0	307	0	307	0	307	C2
TCGA-DJ-A2PP	47	MALE	NA	T2	N0	M0	II	NA	NA	NA	NA	No	NA	0	930	0	930	0	930	0	930	C2
TCGA-DJ-A2PQ	26	MALE	WHITE	T2	N1	M0	I	NA	NA	NA	NA	No	NA	0	1077	0	1077	0	1077	0	1077	C2
TCGA-DJ-A2PS	40	FEMALE	WHITE	T3	N1	M0	I	NA	NA	NA	NA	No	NA	0	797	0	797	0	797	0	797	C2
TCGA-DJ-A2PU	52	FEMALE	WHITE	T2	N0	M0	II	NA	NA	NA	NA	No	NA	0	1033	0	1033	0	1033	0	1033	C2
TCGA-DJ-A2PW	65	MALE	WHITE	T3	N1	M0	III	NA	NA	NA	NA	No	NA	0	1060	0	1060	0	1060	0	1060	C2
TCGA-DJ-A2PX	55	FEMALE	WHITE	T1	N0	M0	I	NA	NA	NA	NA	No	NA	0	881	0	881	0	881	0	881	C2
TCGA-DJ-A2Q0	57	FEMALE	WHITE	T2	N0	M0	II	NA	NA	NA	NA	No	NA	0	1045	0	1045	0	1045	0	1045	C2
TCGA-DJ-A2Q2	57	FEMALE	WHITE	T3	N0	M0	III	NA	NA	NA	NA	No	NA	0	1337	0	1337	0	1337	0	1337	C2
TCGA-DJ-A2Q5	51	MALE	WHITE	T2	N1	M0	IV	NA	NA	NA	NA	No	NA	0	1140	0	1140	NA	NA	0	1140	C2
TCGA-DJ-A2Q6	38	FEMALE	WHITE	T1	N1	M0	I	NA	NA	NA	NA	No	NA	0	1109	0	1109	0	1109	0	1109	C2
TCGA-DJ-A2Q7	52	FEMALE	ASIAN	T2	N1	M0	III	NA	NA	NA	NA	No	NA	0	1068	0	1068	0	1068	0	1068	C2
TCGA-DJ-A2QB	53	FEMALE	ASIAN	T3	N1	M0	IV	NA	NA	NA	NA	No	NA	0	1070	0	1070	NA	NA	0	1070	C2
TCGA-DJ-A3UK	35	FEMALE	WHITE	T1	N0	M0	I	NA	NA	NA	NO	No	NA	0	1123	0	1123	0	1123	0	1123	C2
TCGA-DJ-A3UM	45	FEMALE	ASIAN	T1	N0	M0	I	NA	NA	NA	NO	No	NA	0	944	0	944	0	944	0	944	C2
TCGA-DJ-A3UN	51	FEMALE	WHITE	T1	N0	M0	I	NA	NA	NA	NO	No	NA	0	986	0	986	0	986	0	986	C2
TCGA-DJ-A3UO	63	MALE	WHITE	T3	N1	M0	IV	NA	Primary	NA	YES	No	NA	0	957	0	957	NA	NA	1	641	C2
TCGA-DJ-A3UP	38	FEMALE	NA	T2	N0	M0	I	NA	NA	NA	YES	No	NA	0	499	0	499	0	499	0	499	C2
TCGA-DJ-A3UR	51	FEMALE	WHITE	T3	N1	M0	III	NA	NA	NA	NO	No	NA	0	1105	0	1105	0	1105	0	1105	C2
TCGA-DJ-A3US	37	FEMALE	WHITE	T1	N0	M0	I	NA	NA	NA	NO	No	NA	0	998	0	998	0	998	0	998	C2
TCGA-DJ-A3UU	49	FEMALE	WHITE	T3	N0	M0	III	NA	NA	NA	YES	No	NA	0	713	0	713	NA	NA	0	713	C2
TCGA-DJ-A3UV	18	FEMALE	WHITE	T1	N0	M0	I	NA	NA	NA	NO	No	NA	0	1466	0	1466	0	1466	0	1466	C2
TCGA-DJ-A3UX	46	FEMALE	WHITE	T1	N0	M0	I	NA	NA	NA	NO	No	NA	0	345	0	345	0	345	0	345	C2

TCGA-DJ-A3UZ	70	FEMALE	WHITE	T3	N0	M0	III	NA	NA	NA	NO	No	NA	0	1246	0	1246	0	1246	0	1246	C2
TCGA-DJ-A3V0	56	MALE	NA	T4	N1	M0	IV	NA	NA	NA	YES	No	NA	0	1125	0	1125	NA	NA	0	1125	C2
TCGA-DJ-A3V2	44	FEMALE	WHITE	T2	N1	M0	I	NA	NA	NA	NO	No	NA	0	615	0	615	0	615	0	615	C2
TCGA-DJ-A3V3	57	FEMALE	ASIAN	T2	N0	M0	II	NA	NA	NA	NO	No	NA	0	931	0	931	0	931	0	931	C2
TCGA-DJ-A3V4	60	FEMALE	WHITE	T3	N1	M0	III	NA	NA	NA	YES	No	NA	0	1294	0	1294	0	1294	0	1294	C2
TCGA-DJ-A3V7	44	FEMALE	WHITE	T3	N0	M0	III	NA	NA	NA	YES	No	NA	0	706	0	706	0	706	0	706	C2
TCGA-DJ-A3V8	28	FEMALE	WHITE	T3	N1	M0	I	NA	NA	NA	YES	No	NA	0	647	0	647	0	647	0	647	C2
TCGA-DJ-A3V9	50	FEMALE	WHITE	T2	N1	M0	IV	NA	NA	NA	YES	No	NA	0	623	0	623	NA	NA	0	623	C2
TCGA-DJ-A3VA	39	FEMALE	NA	T1	N1	M0	I	NA	NA	NA	NO	No	NA	0	755	0	755	0	755	0	755	C2
TCGA-DJ-A3VJ	22	MALE	WHITE	T3	N1	M0	I	NA	NA	NA	YES	No	NA	0	1108	0	1108	0	1108	0	1108	C2
TCGA-DJ-A3VM	68	FEMALE	WHITE	T2	N0	M0	II	NA	NA	NA	YES	No	NA	0	840	0	840	0	840	0	840	C2
TCGA-DJ-A4UP	15	FEMALE	WHITE	T1	N1	M0	I	NA	NA	NA	YES	No	NA	0	916	0	916	0	916	0	916	C2
TCGA-DJ-A4UQ	60	MALE	ASIAN	T3	N1	M0	IV	NA	NA	NA	YES	No	NA	0	1005	0	1005	NA	NA	0	1005	C2
TCGA-DJ-A4UR	36	FEMALE	ASIAN	T3	N1	M1	II	NA	Metastasis	NA	YES	No	NA	0	1058	0	1058	1	113	1	113	C2
TCGA-DJ-A4UW	34	FEMALE	ASIAN	T1	N1	M0	I	NA	NA	NA	YES	No	NA	0	819	0	819	NA	NA	0	819	C2
TCGA-DJ-A4V0	36	FEMALE	ASIAN	T2	N0	M0	I	NA	NA	NA	NO	No	NA	0	1081	0	1081	0	1081	0	1081	C2
TCGA-DJ-A4V5	55	MALE	WHITE	T3	N1	M0	IV	NA	NA	NA	YES	No	NA	0	1076	0	1076	NA	NA	0	1076	C2
TCGA-DO-A1JZ	23	FEMALE	BLACK	T2	NA	NA	I	NA	NA	NA	NA	No	Hormone Therapy	0	1670	0	1670	NA	NA	0	1670	C2
TCGA-E3-A3DY	37	MALE	WHITE	T3	N1	M0	I	NA	NA	NA	NA	No	Hormone Therapy	0	2815	0	2815	0	2815	0	2815	C2
TCGA-E3-A3DZ	55	FEMALE	WHITE	T3	N0	M0	III	NA	NA	NA	NA	No	Hormone Therapy	0	1436	0	1436	0	1436	0	1436	C2
TCGA-E3-A3E0	34	FEMALE	WHITE	T2	N0	NA	I	NA	NA	NA	NA	No	Hormone Therapy	0	2019	0	2019	0	2019	0	2019	C2
TCGA-E3-A3E1	46	FEMALE	WHITE	T1	N0	M0	I	NA	NA	NA	NA	No	Hormone Therapy	0	2449	0	2449	0	2449	0	2449	C2
TCGA-E3-A3E2	34	FEMALE	WHITE	T1	N1	NA	I	NA	NA	NA	NA	No	Hormone Therapy	0	1922	0	1922	0	1922	0	1922	C2
TCGA-E3-A3E3	49	FEMALE	WHITE	T2	N0	M0	II	NA	Metastasis	NA	NA	No	Hormone Therapy	0	1595	0	1595	1	445	1	445	C2
TCGA-E3-A3E5	57	MALE	WHITE	T4	N1	NA	IV	NA	NA	NA	NA	No	Hormone Therapy	0	1215	0	1215	NA	NA	0	1215	C2
TCGA-E8-A3X7	57	FEMALE	WHITE	T4	N0	M0	IV	NA	NA	NA	NA	No	NA	0	951	0	951	NA	NA	0	951	C2
TCGA-E8-A415	39	FEMALE	ASIAN	T1	N1	M0	I	NA	NA	NA	NA	No	NA	0	944	0	944	0	944	0	944	C2
TCGA-E8-A416	51	FEMALE	ASIAN	T1	N0	M0	I	NA	NA	NA	NA	No	NA	0	952	0	952	0	952	0	952	C2
TCGA-E8-A418	75	FEMALE	WHITE	T4	N0	M0	IV	NA	NA	NA	NA	No	NA	0	92	0	92	NA	NA	0	92	C2
TCGA-E8-A434	56	FEMALE	ASIAN	T1	N0	M0	I	NA	NA	NA	NA	No	NA	0	953	0	953	0	953	0	953	C2
TCGA-E8-A437	57	FEMALE	WHITE	NA	NA	M0	I	NA	NA	NA	NA	No	NA	0	1213	0	1213	NA	NA	0	1213	C2
TCGA-E8-A438	23	FEMALE	WHITE	T1	N0	M0	I	NA	NA	NA	NA	No	NA	0	1019	0	1019	0	1019	0	1019	C2
TCGA-E8-A44M	23	FEMALE	ASIAN	T1	N0	M0	I	NA	NA	NA	NO	No	NA	0	917	0	917	0	917	0	917	C2
TCGA-EL-A3CL	70	FEMALE	WHITE	T2	NA	M0	II	NA	NA	NA	NO	No	NA	1	963	NA	963	0	963	0	963	C2
TCGA-EL-A3CP	50	FEMALE	BLACK	T1	NA	M0		NA	NA	NA	YES	No	NA	0	4831	0	4831	NA	NA	0	4831	C2
TCGA-EL-A3CR	78	FEMALE	WHITE	T3	N1	M0	III	NA	NA	NA	NO	No	NA	1	233	NA	233	NA	NA	0	233	C2
TCGA-EL-A3CW	87	FEMALE	WHITE	T2	N1	M0	III	NA	NA	NA	NO	No	NA	1	1734	NA	1734	0	1734	0	1734	C2
TCGA-EL-A3CZ	37	FEMALE	WHITE	T1	N1	M0	I	NA	Recurrence	NA	YES	No	NA	0	2418	0	2418	NA	NA	1	1426	C2
TCGA-EL-A3D5	44	FEMALE	WHITE	T3	N1	M0	I	NA	NA	NA	YES	No	NA	0	1737	0	1737	NA	NA	0	1737	C2
TCGA-EL-A3GW	37	FEMALE	WHITE	T2	N0	M0	I	NA	NA	NA	YES	No	NA	0	1527	0	1527	0	1527	0	1527	C2
TCGA-EL-A3GZ	34	FEMALE	WHITE	T1	N0	M0	I	NA	NA	NA	YES	No	NA	0	4258	0	4258	0	4258	0	4258	C2
TCGA-EL-A3H3	19	FEMALE	WHITE	T2	N1	M0	I	NA	NA	NA	YES	No	NA	0	1319	0	1319	0	1319	0	1319	C2
TCGA-EL-A3H8	35	FEMALE	WHITE	T3	N1	M0	I	NA	NA	NA	NA	No	NA	0	4577	0	4577	0	4577	0	4577	C2
TCGA-EL-A3MW	55	FEMALE	WHITE	T2	N0	M0	II	NA	NA	NA	YES	No	NA	0	3305	0	3305	0	3305	0	3305	C2
TCGA-EL-A3MX	66	FEMALE	WHITE	T4	N1	M1	IV	NA	Metastasis	NA	YES	No	NA	1	1753	1	1753	NA	NA	1	1708	C2
TCGA-EL-A3T0	45	FEMALE	WHITE	T3	N1	M0	III	NA	NA	NA	YES	No	NA	0	4335	0	4335	0	4335	0	4335	C2
TCGA-EL-A3T2	55	FEMALE	WHITE	T2	N1	M0	III	NA	Recurrence	NA	YES	No	NA	0	988	0	988	1	903	1	903	C2
TCGA-EL-A3T7	47	FEMALE	WHITE	T2	N0	M0	II	NA	NA	NA	NO	No	NA	0	1256	0	1256	0	1256	0	1256	C2
TCGA-EL-A3T8	36	MALE	ASIAN	T2	N0	M0	I	NA	NA	NA	YES	No	NA	0	3269	0	3269	0	3269	0	3269	C2
TCGA-EL-A3TA	42	MALE	WHITE	T2	N0	M0	I	NA	NA	NA	YES	No	NA	0	2035	0	2035	0	2035	0	2035	C2
TCGA-EL-A3TB	47	FEMALE	WHITE	T3	N1	M0	III	NA	NA	NA	YES	No	NA	0	2519	0	2519	0	2519	0	2519	C2
TCGA-EL-A3ZG	15	MALE	ASIAN	T3	N0	M0	I	NA	NA	NA	YES	No	NA	0	2724	0	2724	0	2724	0	2724	C2
TCGA-EL-A3ZH	42	FEMALE	WHITE	T3	N1	M0	I	NA	NA	NA	YES	No	NA	0	2773	0	2773	0	2773	0	2773	C2
TCGA-EL-A3ZL	34	FEMALE	WHITE	T1	N1	M0	I	NA	NA	NA	YES	No	NA	0	1895	0	1895	0	1895	0	1895	C2
TCGA-EL-A3ZP	19	MALE	WHITE	T4	N1	M0	I	NA	NA	NA	YES	No	NA	0	1992	0	1992	0	1992	0	1992	C2
TCGA-EL-A3ZS	22	FEMALE	WHITE	T1	N1	M0	I	NA	NA	NA	YES	No	NA	0	215	0	215	0	215	0	215	C2
TCGA-EL-A4JV	41	FEMALE	ASIAN	T2	N0	M0	I	NA	NA	NA	YES	No	NA	0	1748	0	1748	0	1748	0	1748	C2
TCGA-EL-A4JZ	55	FEMALE	WHITE	T3	N1	M0	III	NA	NA	NA	NO	No	NA	0	1856	0	1856	0	1856	0	1856	C2

TCGA-EL-A4K0	54	FEMALE	WHITE	T1	N0	M0	I	NA	NA	NA	NO	No	NA	0	944	0	944	NA	NA	0	944	C2
TCGA-EL-A4K2	43	FEMALE	WHITE	T1	N0	M0	I	NA	NA	NA	NO	No	NA	0	928	0	928	0	928	0	928	C2
TCGA-EL-A4K6	75	MALE	WHITE	T4	N1	NA	IV	NA	Metastasis	NA	YES	No	NA	0	1343	0	1343	NA	NA	1	874	C2
TCGA-EL-A4K9	68	MALE	WHITE	T2	N0	M0	II	NA	NA	NA	YES	No	NA	0	1455	0	1455	0	1455	0	1455	C2
TCGA-EL-A4KD	41	MALE	WHITE	T3	N1	M0	I	NA	NA	NA	YES	No	NA	0	1326	0	1326	0	1326	0	1326	C2
TCGA-EL-A4KG	35	FEMALE	WHITE	T3	N0	M0	I	NA	NA	NA	NO	No	NA	0	739	0	739	0	739	0	739	C2
TCGA-EM-A1CS	55	FEMALE	NA	T1	N0	NA	I	NA	NA	NA	NA	No	NA	0	817	0	817	0	817	0	817	C2
TCGA-EM-A1CU	31	MALE	NA	T3	N1	M0	I	NA	NA	NA	NA	No	NA	0	636	0	636	NA	NA	0	636	C2
TCGA-EM-A1CV	32	FEMALE	NA	T1	N0	NA	I	NA	NA	NA	NA	No	NA	0	1475	0	1475	NA	NA	0	1475	C2
TCGA-EM-A1CW	39	FEMALE	NA	T3	N0	NA	I	NA	NA	NA	NA	No	NA	0	549	0	549	0	549	0	549	C2
TCGA-EM-A1YA	74	FEMALE	NA	T2	NA	NA	II	NA	NA	NA	NA	No	NA	0	602	0	602	0	602	0	602	C2
TCGA-EM-A1YB	73	FEMALE	NA	T2	NA	NA	II	NA	NA	NA	NA	No	NA	0	592	0	592	0	592	0	592	C2
TCGA-EM-A1YC	71	FEMALE	NA	T3	N0	NA	III	NA	NA	NA	NA	No	NA	0	2391	0	2391	0	2391	0	2391	C2
TCGA-EM-A1YD	53	FEMALE	NA	T2	N0	NA	II	NA	NA	NA	NA	No	NA	0	1734	0	1734	0	1734	0	1734	C2
TCGA-EM-A22J	56	FEMALE	NA	T2	N0	NA	II	NA	NA	NA	NA	No	NA	0	441	0	441	NA	NA	0	441	C2
TCGA-EM-A22K	46	FEMALE	NA	T1	N1	NA	IV	NA	NA	NA	NA	No	NA	0	479	0	479	NA	NA	0	479	C2
TCGA-EM-A22L	33	FEMALE	NA	T2	N0	NA	I	NA	NA	NA	NA	No	NA	0	318	0	318	0	318	0	318	C2
TCGA-EM-A22N	68	FEMALE	NA	T1	N0	NA	I	NA	NA	NA	NA	No	NA	0	324	0	324	NA	NA	0	324	C2
TCGA-EM-A22O	75	MALE	ASIAN	T4	N1	NA	IV	NA	NA	NA	NA	No	NA	0	1344	0	1344	NA	NA	0	1344	C2
TCGA-EM-A22P	64	MALE	NA	T1	N1	NA	IV	NA	NA	NA	NA	No	NA	0	1256	0	1256	NA	NA	0	1256	C2
TCGA-EM-A2CJ	49	FEMALE	NA	T1	NA	NA	I	NA	NA	NA	NA	No	NA	0	1447	0	1447	0	1447	0	1447	C2
TCGA-EM-A2CK	28	MALE	NA	T2	N1	NA	I	NA	NA	NA	NA	No	NA	0	544	0	544	0	544	0	544	C2
TCGA-EM-A2CL	31	MALE	NA	T2	NA	NA	I	NA	NA	NA	NA	No	NA	0	401	0	401	0	401	0	401	C2
TCGA-EM-A2CM	62	FEMALE	NA	T3	N0	NA	III	NA	NA	NA	NA	No	NA	0	1253	0	1253	0	1253	0	1253	C2
TCGA-EM-A2CN	62	MALE	NA	T3	N0	M1	IV	NA	NA	NA	NA	No	NA	0	715	0	715	NA	NA	0	715	C2
TCGA-EM-A2CO	77	FEMALE	NA	T2	NA	NA	II	NA	NA	NA	NA	No	NA	0	184	0	184	0	184	0	184	C2
TCGA-EM-A2CQ	40	FEMALE	NA	T1	N1	NA	I	NA	NA	NA	NA	No	NA	0	374	0	374	0	374	0	374	C2
TCGA-EM-A2CS.1	51	FEMALE	NA	T3	N1	NA	IV	NA	NA	NA	NA	No	NA	0	841	0	841	NA	NA	0	841	C2
TCGA-EM-A2CU	47	FEMALE	NA	T2	N1	NA	III	NA	NA	NA	NA	No	NA	0	400	0	400	0	400	0	400	C2
TCGA-EM-A2OV	64	FEMALE	ASIAN	T2	N0	NA	II	NA	NA	NA	NA	No	NA	0	674	0	674	0	674	0	674	C2
TCGA-EM-A2OW	37	FEMALE	NA	T1	NA	NA	I	NA	NA	NA	NA	No	NA	0	1227	0	1227	0	1227	0	1227	C2
TCGA-EM-A2OX	46	MALE	NA	T1	N1	NA	IV	NA	NA	NA	NA	No	NA	0	600	0	600	NA	NA	0	600	C2
TCGA-EM-A2OY	46	FEMALE	NA	T2	N0	NA	II	NA	NA	NA	NA	No	NA	0	983	0	983	0	983	0	983	C2
TCGA-EM-A2OZ	66	MALE	NA	T1	N0	NA	I	NA	NA	NA	NA	No	NA	0	1033	0	1033	0	1033	0	1033	C2
TCGA-EM-A2P0	33	MALE	NA	T2	N1	NA	I	NA	NA	NA	NA	No	NA	0	535	0	535	0	535	0	535	C2
TCGA-EM-A2P2	46	MALE	NA	T3	N0	NA	III	NA	NA	NA	NA	No	NA	0	526	0	526	0	526	0	526	C2
TCGA-EM-A2P3	47	FEMALE	NA	T1	NA	NA	I	NA	NA	NA	NA	No	NA	0	444	0	444	0	444	0	444	C2
TCGA-EM-A3AK	62	FEMALE	NA	T1	N0	NA	I	NA	NA	NA	NA	No	NA	0	477	0	477	0	477	0	477	C2
TCGA-EM-A3AN	36	FEMALE	NA	T1	N1	NA	I	NA	NA	NA	NA	No	NA	0	1006	0	1006	NA	NA	0	1006	C2
TCGA-EM-A3AO	61	MALE	NA	T2	N1	NA	IV	NA	NA	NA	NA	No	NA	0	1354	0	1354	NA	NA	0	1354	C2
TCGA-EM-A3AP	31	FEMALE	NA	T2	NA	NA	I	NA	NA	NA	NA	No	NA	0	254	0	254	0	254	0	254	C2
TCGA-EM-A3AR	41	MALE	NA	T1	N1	NA	I	NA	NA	NA	NA	No	NA	0	1133	0	1133	0	1133	0	1133	C2
TCGA-EM-A3FJ	24	FEMALE	NA	T1	N1	NA	I	NA	NA	NA	NA	No	NA	0	850	0	850	0	850	0	850	C2
TCGA-EM-A3FO	40	MALE	NA	T3	N0	NA	I	NA	NA	NA	NA	No	NA	0	532	0	532	0	532	0	532	C2
TCGA-EM-A3FQ	19	FEMALE	NA	T3	N1	NA	I	NA	NA	NA	NA	No	NA	0	492	0	492	0	492	0	492	C2
TCGA-EM-A3FR	55	FEMALE	NA	T2	N1	NA	III	NA	NA	NA	NA	No	NA	0	457	0	457	0	457	0	457	C2
TCGA-EM-A3O7	46	FEMALE	NA	T3	NA	NA	III	NA	NA	NA	YES	No	NA	0	223	0	223	0	223	0	223	C2
TCGA-EM-A3O8	33	FEMALE	NA	T1	N0	NA	I	NA	NA	NA	NO	No	NA	0	381	0	381	0	381	0	381	C2
TCGA-EM-A3SX	28	FEMALE	NA	T3	N1	NA	I	NA	NA	NA	NO	No	NA	0	908	0	908	NA	NA	0	908	C2
TCGA-EM-A3SY	36	MALE	NA	T1	NA	NA	I	NA	NA	NA	NO	No	NA	0	411	0	411	0	411	0	411	C2
TCGA-EM-A4FN	49	FEMALE	NA	T1	N1	NA	IV	NA	NA	NA	YES	No	NA	0	802	0	802	NA	NA	0	802	C2
TCGA-EM-A4FO	72	MALE	NA	T1	N0	NA	I	NA	NA	NA	YES	No	NA	0	802	0	802	0	802	0	802	C2
TCGA-EM-A4FR	28	MALE	NA	T3	N1	NA	I	NA	NA	NA	YES	No	NA	0	655	0	655	0	655	0	655	C2
TCGA-EM-A4FU	35	MALE	NA	T3	NA	NA	I	NA	Recurrence	NA	YES	No	NA	0	1840	0	1840	1	781	1	781	C2
TCGA-EM-A4FV	64	FEMALE	NA	T1	N0	NA	I	NA	NA	NA	NO	No	NA	0	744	0	744	0	744	0	744	C2
TCGA-EM-A4G1	26	FEMALE	NA	T1	N0	NA	I	NA	NA	NA	NO	No	NA	0	416	0	416	0	416	0	416	C2
TCGA-ET-A25I	37	FEMALE	WHITE	T2	N0	NA	I	NA	NA	NA	NA	No	NA	0	2485	0	2485	0	2485	0	2485	C2
TCGA-ET-A25K	36	FEMALE	WHITE	T2	N1	NA	I	NA	NA	NA	NA	No	NA	0	2142	0	2142	0	2142	0	2142	C2

TCGA-ET-A25M	33	MALE	WHITE	T2	N1	NA	I	NA	NA	NA	NA	No	NA	0	1831	0	1831	0	1831	0	1831	C2
TCGA-ET-A25O	35	FEMALE	BLACK	T2	N1	NA	I	NA	NA	NA	NA	No	NA	0	1986	0	1986	0	1986	0	1986	C2
TCGA-ET-A25P	24	FEMALE	ASIAN	T1	N0	NA	I	NA	NA	NA	NA	No	NA	0	559	0	559	0	559	0	559	C2
TCGA-ET-A2MX	27	MALE	WHITE	T2	N1	NA	I	NA	NA	NA	YES	No	NA	0	2689	0	2689	0	2689	0	2689	C2
TCGA-ET-A2MY	72	FEMALE	BLACK	T1	NA	NA	I	NA	NA	NA	NA	No	NA	0	1787	0	1787	0	1787	0	1787	C2
TCGA-ET-A2MZ	39	MALE	WHITE	T1	N0	NA	I	NA	NA	NA	NA	No	NA	0	1133	0	1133	NA	NA	0	1133	C2
TCGA-ET-A2N4	53	FEMALE	WHITE	T3	N0	NA	III	NA	NA	NA	NA	No	NA	0	81	0	81	NA	NA	0	81	C2
TCGA-ET-A39I	83	FEMALE	WHITE	T1	NA	NA	I	NA	NA	NA	NA	No	NA	1	1011	0	1011	0	1011	0	1011	C2
TCGA-ET-A39J	23	FEMALE	WHITE	T2	NA	NA	I	NA	NA	NA	NA	No	NA	0	3227	0	3227	0	3227	0	3227	C2
TCGA-ET-A39L	20	FEMALE	NA	T3	N0	NA	I	NA	NA	NA	NA	No	NA	0	3299	0	3299	0	3299	0	3299	C2
TCGA-ET-A39M	34	MALE	WHITE	T2	NA	NA	I	NA	NA	NA	NA	No	NA	0	2582	0	2582	0	2582	0	2582	C2
TCGA-ET-A39P	73	FEMALE	BLACK	T3	N0	NA	III	NA	NA	NA	NA	No	NA	0	2900	0	2900	NA	NA	0	2900	C2
TCGA-ET-A39R	24	FEMALE	WHITE	T1	N0	NA	I	NA	NA	NA	NA	No	NA	0	2863	0	2863	0	2863	0	2863	C2
TCGA-ET-A39S	27	FEMALE	WHITE	T1	N0	NA	I	NA	NA	NA	NA	No	NA	0	1676	0	1676	0	1676	0	1676	C2
TCGA-ET-A39T	55	FEMALE	WHITE	T3	N0	NA	III	NA	NA	NA	NA	No	NA	0	2987	0	2987	0	2987	0	2987	C2
TCGA-ET-A3BP	34	FEMALE	WHITE	T2	N1	NA	I	NA	NA	NA	NA	No	NA	0	2406	0	2406	0	2406	0	2406	C2
TCGA-ET-A3BQ	27	FEMALE	WHITE	T2	N0	NA	I	NA	NA	NA	NA	No	NA	0	2331	0	2331	0	2331	0	2331	C2
TCGA-ET-A3BT	60	FEMALE	WHITE	T2	N1	NA	IV	NA	Recurrence	NA	NA	No	NA	0	2665	0	2665	NA	NA	1	267	C2
TCGA-ET-A3BU	28	MALE	WHITE	T1	N1	NA	I	NA	NA	NA	NA	No	NA	0	2367	0	2367	0	2367	0	2367	C2
TCGA-ET-A3BW	30	FEMALE	WHITE	T2	NA	NA	I	NA	NA	NA	NA	No	NA	0	1419	0	1419	0	1419	0	1419	C2
TCGA-ET-A3DP	43	FEMALE	BLACK	T2	NA	NA	I	NA	NA	NA	NA	No	NA	0	2877	0	2877	0	2877	0	2877	C2
TCGA-ET-A3DQ	43	FEMALE	WHITE	T1	N0	NA	I	NA	NA	NA	NA	No	NA	0	2581	0	2581	0	2581	0	2581	C2
TCGA-ET-A3DR	42	FEMALE	WHITE	T1	N1	NA	I	NA	NA	NA	NA	No	NA	0	2451	0	2451	0	2451	0	2451	C2
TCGA-ET-A3DS	33	FEMALE	WHITE	T1	N0	NA	I	NA	NA	NA	NA	No	NA	0	2033	0	2033	0	2033	0	2033	C2
TCGA-ET-A40S	62	MALE	WHITE	T2	N0	NA	II	NA	NA	NA	YES	No	NA	0	1223	0	1223	NA	NA	0	1223	C2
TCGA-ET-A40T	26	FEMALE	WHITE	T2	N1	NA	I	NA	NA	NA	NA	No	NA	0	1140	0	1140	NA	NA	0	1140	C2
TCGA-ET-A4KQ	45	FEMALE	WHITE	T2	NA	NA	II	NA	NA	NA	NO	No	NA	0	955	0	955	0	955	0	955	C2
TCGA-FE-A231	70	MALE	WHITE	T3	N1	NA	IV	NA	NA	NA	NA	No	NA	0	961	0	961	NA	NA	0	961	C2
TCGA-FE-A232	44	FEMALE	WHITE	T3	NA	NA	I	NA	NA	NA	NA	No	NA	0	2424	0	2424	0	2424	0	2424	C2
TCGA-FE-A236	33	MALE	WHITE	T2	N1	NA	I	NA	NA	NA	NA	No	NA	0	2031	0	2031	0	2031	0	2031	C2
TCGA-FE-A237	19	FEMALE	WHITE	T3	N1	NA	I	NA	Recurrence	NA	NA	No	NA	0	3431	0	3431	1	492	1	492	C2
TCGA-FE-A238	36	FEMALE	WHITE	T3	N0	NA	I	NA	Metastasis	NA	NA	No	NA	0	1215	0	1215	1	431	1	431	C2
TCGA-FE-A3PA	16	MALE	WHITE	T4	N0	M1	II	NA	NA	NA	NA	No	NA	0	3235	0	3235	NA	NA	0	3235	C2
TCGA-FE-A3PC	37	FEMALE	WHITE	T3	N1	NA	I	NA	NA	NA	NA	No	NA	0	2606	0	2606	0	2606	0	2606	C2
TCGA-FK-A3S3	43	FEMALE	BLACK	T3	N1	NA	I	NA	NA	NA	YES	No	NA	0	2162	0	2162	0	2162	0	2162	C2
TCGA-FK-A3SB	28	FEMALE	ASIAN	T3	N1	NA	I	NA	NA	NA	YES	No	NA	0	125	0	125	0	125	0	125	C2
TCGA-FK-A3SE	31	FEMALE	ASIAN	T2	N1	NA	I	NA	NA	NA	YES	No	NA	0	670	0	670	0	670	0	670	C2
TCGA-FK-A3SG	21	FEMALE	WHITE	T2	N1	NA	I	NA	NA	NA	NA	No	NA	0	28	0	28	0	28	0	28	C2
TCGA-FK-A4UB	51	MALE	WHITE	T3	N1	NA	III	NA	NA	NA	YES	No	NA	0	3531	0	3531	NA	NA	0	3531	C2
TCGA-FY-A3I4	34	FEMALE	WHITE	T2	N0	M0	I	NA	NA	NA	NA	No	NA	0	542	0	542	0	542	0	542	C2
TCGA-FY-A3I5	64	FEMALE	WHITE	T3	N0	NA	III	NA	NA	NA	NA	No	NA	0	238	0	238	0	238	0	238	C2
TCGA-FY-A3ON	20	MALE	WHITE	T2	N1	NA	I	NA	NA	NA	NA	No	Hormone Therapy	0	595	0	595	0	595	0	595	C2
TCGA-FY-A3R6	57	FEMALE	WHITE	T2	N1	NA	IV	NA	NA	NA	NA	No	NA	0	423	0	423	NA	NA	0	423	C2
TCGA-FY-A3R7	50	FEMALE	WHITE	T3	N1	NA	III	NA	NA	NA	NA	No	NA	0	495	0	495	NA	NA	0	495	C2
TCGA-FY-A3R9	66	FEMALE	WHITE	T1	N0	NA	I	NA	NA	NA	NA	No	Hormone Therapy	0	504	0	504	0	504	0	504	C2
TCGA-FY-A3RA	21	FEMALE	WHITE	T2	NA	NA	I	NA	NA	NA	YES	No	NA	0	550	0	550	0	550	0	550	C2
TCGA-FY-A3W9	66	FEMALE	WHITE	T2	N0	NA	II	NA	NA	NA	NA	No	Hormone Therapy	0	658	0	658	NA	NA	0	658	C2
TCGA-FY-A3WA	52	FEMALE	WHITE	T1	NA	NA	I	NA	NA	NA	NA	No	NA	0	519	0	519	0	519	0	519	C2
TCGA-FY-A40K	46	FEMALE	WHITE	T1	N0	NA	I	NA	NA	NA	YES	No	Hormone Therapy	0	461	0	461	0	461	0	461	C2
TCGA-FY-A40M	51	FEMALE	WHITE	T1	N0	NA	I	NA	NA	NA	YES	No	Hormone Therapy	0	684	0	684	0	684	0	684	C2
TCGA-FY-A40N	55	FEMALE	WHITE	T1	N0	NA	I	NA	NA	NA	NA	No	Hormone Therapy	0	579	0	579	0	579	0	579	C2
TCGA-FY-A4B4	62	FEMALE	WHITE	T1	N1	NA	III	NA	NA	NA	NA	No	Hormone Therapy	0	657	0	657	NA	NA	0	657	C2
TCGA-FY-A76V	54	MALE	BLACK	T2	NA	NA	II	NA	NA	NA	NA	No	NA	0	51	0	51	0	51	0	51	C2
TCGA-GE-A2C6	33	FEMALE	WHITE	T2	N1	NA	I	NA	NA	NA	NA	No	NA	0	381	0	381	NA	NA	0	381	C2
TCGA-H2-A2K9	25	MALE	WHITE	T2	N1	NA	I	NA	NA	NA	NA	No	NA	0	659	0	659	NA	NA	0	659	C2
TCGA-H2-A3RI	29	FEMALE	WHITE	T3	N0	NA	I	NA	Recurrence	NA	NA	No	NA	0	335	0	335	NA	NA	1	279	C2
TCGA-IM-A3U3	55	FEMALE	WHITE	T1	N0	NA	I	NA	NA	NA	NA	No	NA	0	514	0	514	NA	NA	0	514	C2
TCGA-IM-A41Y	42	FEMALE	WHITE	T3	N1	NA	I	NA	NA	NA	NO	No	Hormone Therapy	0	664	0	664	0	664	0	664	C2

TCGA-J8-A3NZ	52	FEMALE	WHITE	T3	N1	M0	III	NA	NA	NA	NA	No	NA	0	524	0	524	0	524	0	524	C2
TCGA-J8-A3O0	38	MALE	WHITE	T2	N0	M0	I	NA	NA	NA	NA	No	NA	0	1313	0	1313	0	1313	0	1313	C2
TCGA-J8-A3O2.1	39	MALE	WHITE	T3	N1	M0	I	NA	NA	NA	NA	No	NA	0	342	0	342	NA	NA	0	342	C2
TCGA-J8-A3YD	47	FEMALE	WHITE	T1	N1	M0	III	NA	NA	NA	YES	No	NA	0	968	0	968	0	968	0	968	C2
TCGA-J8-A3YE	31	FEMALE	WHITE	T3	N1	M0	I	NA	NA	NA	YES	No	NA	0	847	0	847	0	847	0	847	C2
TCGA-J8-A3YH.1	39	MALE	WHITE	T3	N1	M0	I	NA	NA	NA	YES	No	NA	0	175	0	175	0	175	0	175	C2
TCGA-J8-A42S	45	MALE	WHITE	T1	N1	NA	III	NA	NA	NA	NO	No	NA	0	75	0	75	0	75	0	75	C2
TCGA-J8-A4HW.1	59	FEMALE	WHITE	T2	N1	M0	III	NA	NA	NA	YES	No	NA	0	79	0	79	0	79	0	79	C2
TCGA-J8-A4HY	68	FEMALE	WHITE	T4	N1	NA	IV	NA	NA	NA	NO	No	NA	0	6	0	6	NA	NA	0	6	C2
TCGA-KS-A4I3	41	MALE	ASIAN	T3	N1	M0	I	NA	NA	NA	YES	No	Hormone Therapy	0	1329	0	1329	NA	NA	0	1329	C2
TCGA-KS-A4I5	49	FEMALE	WHITE	T1	N1	M0	IV	NA	NA	NA	YES	No	Hormone Therapy	0	567	0	567	NA	NA	0	567	C2
TCGA-KS-A4I7	61	FEMALE	WHITE	T1	N0	M0	I	NA	NA	NA	YES	No	Hormone Therapy	0	987	0	987	0	987	0	987	C2
TCGA-KS-A4IC	45	FEMALE	WHITE	T1	N1	M0	III	NA	NA	NA	YES	No	Hormone Therapy	0	1073	0	1073	NA	NA	0	1073	C2
TCGA-L6-A4EQ	47	MALE	WHITE	T2	N1	M0	III	NA	NA	NA	YES	No	NA	0	1228	0	1228	0	1228	0	1228	C2
TCGA-L6-A4ET	49	FEMALE	WHITE	T3	NA	M1	IV	NA	NA	NA	YES	No	NA	0	664	0	664	NA	NA	0	664	C2
TCGA-MK-A4N7	20	FEMALE	WHITE	T2	N0	M0	I	NA	NA	NA	YES	No	Hormone Therapy	0	987	0	987	0	987	0	987	C2
TCGA-MK-A4N9	41	FEMALE	WHITE	T3	N1	M0	I	NA	NA	NA	YES	No	Hormone Therapy	0	919	0	919	0	919	0	919	C2
TCGA-QD-A8IV	50	FEMALE	BLACK	T3	N1	NA	IV	NA	NA	NA	NO	No	NA	0	392	0	392	NA	NA	0	392	C2
TCGA-EM-A3FQ1	19	FEMALE	NA	T3	N1	NA	I	NA	NA	NA	NA	No	NA	0	492	0	492	0	492	0	492	C3
TCGA-BJ-A0YZ	65	MALE	WHITE	T2	N0	M0	II	NA	NA	NA	NA	No	NA	0	754	0	754	0	754	0	754	C3
TCGA-BJ-A0ZF	54	FEMALE	WHITE	T2	N0	M0	II	NA	Recurrence	NA	NA	No	NA	0	1217	0	1217	1	735	1	735	C3
TCGA-BJ-A192	54	FEMALE	WHITE	T3	NA	M0	III	NA	NA	NA	NA	No	NA	0	1349	0	1349	NA	NA	0	1349	C3
TCGA-BJ-A28V	80	FEMALE	WHITE	T3	N0	NA	III	NA	NA	NA	NA	No	NA	0	987	0	987	NA	NA	0	987	C3
TCGA-BJ-A28W	32	FEMALE	WHITE	T2	N0	M0	I	NA	NA	NA	NA	No	NA	0	344	0	344	NA	NA	0	344	C3
TCGA-BJ-A291	56	FEMALE	WHITE	T1	N0	M0	I	NA	NA	NA	NA	No	NA	0	696	0	696	NA	NA	0	696	C3
TCGA-BJ-A2N8	30	FEMALE	WHITE	T1	N0	M0	I	NA	NA	NA	NA	No	NA	0	504	0	504	NA	NA	0	504	C3
TCGA-BJ-A2N9	42	FEMALE	WHITE	T2	N0	M0	I	NA	Metastasis	NA	NA	No	NA	0	623	0	623	NA	NA	1	225	C3
TCGA-BJ-A2P4	29	FEMALE	WHITE	T2	N0	M0	I	NA	NA	NA	NA	No	NA	0	390	0	390	0	390	0	390	C3
TCGA-BJ-A3F0	64	FEMALE	WHITE	T1	N0	M0	I	NA	NA	NA	NA	No	NA	0	424	0	424	0	424	0	424	C3
TCGA-BJ-A3PT	51	FEMALE	WHITE	T3	N0	M0	III	NA	NA	NA	YES	No	NA	0	709	0	709	NA	NA	0	709	C3
TCGA-BJ-A3PU	52	MALE	WHITE	T3	N1	M0	III	NA	Metastasis	NA	YES	No	NA	0	567	0	567	1	174	1	174	C3
TCGA-BJ-A45D	36	MALE	WHITE	T2	N0	M0	I	NA	NA	NA	NO	No	NA	0	331	0	331	NA	NA	0	331	C3
TCGA-BJ-A45K	33	MALE	WHITE	T2	N0	M0	I	NA	NA	NA	NO	No	NA	0	705	0	705	NA	NA	0	705	C3
TCGA-CE-A3MD	31	MALE	ASIAN	T2	N1	M0	I	NA	NA	NA	NA	No	NA	0	389	0	389	0	389	0	389	C3
TCGA-CE-A484	37	FEMALE	ASIAN	T2	N1	M0	I	NA	NA	NA	NO	No	NA	0	378	0	378	0	378	0	378	C3
TCGA-DE-A2OL	44	FEMALE	WHITE	T2	NA	NA	I	NA	NA	NA	NA	No	NA	0	1145	0	1145	0	1145	0	1145	C3
TCGA-DE-A4MA	52	FEMALE	BLACK	T3	N1	M0	III	NA	NA	NA	YES	No	NA	0	916	0	916	0	916	0	916	C3
TCGA-DJ-A13M	28	FEMALE	WHITE	T1	N0	M0	I	NA	NA	NA	NA	No	NA	0	1484	0	1484	0	1484	0	1484	C3
TCGA-DJ-A1QL	70	MALE	NA	T2	N0	M0	II	NA	NA	NA	NA	No	NA	0	535	0	535	0	535	0	535	C3
TCGA-DJ-A2PT	70	FEMALE	WHITE	T3	N0	M0	III	NA	NA	NA	NA	No	NA	0	1181	0	1181	0	1181	0	1181	C3
TCGA-DJ-A2QA	56	FEMALE	ASIAN	T3	N0	M0	III	NA	NA	NA	NA	No	NA	0	1020	0	1020	0	1020	0	1020	C3
TCGA-DJ-A2QC	70	FEMALE	NA	T2	N0	M0	II	NA	NA	NA	NA	No	NA	0	1215	0	1215	0	1215	0	1215	C3
TCGA-DJ-A3UT	43	FEMALE	WHITE	T1	N0	M0	I	NA	NA	NA	NO	No	NA	0	1105	0	1105	0	1105	0	1105	C3
TCGA-DJ-A3VG	40	MALE	WHITE	T1	N0	M0	I	NA	NA	NA	NO	No	NA	0	1085	0	1085	0	1085	0	1085	C3
TCGA-DJ-A3VK	63	MALE	WHITE	T3	N0	M0	III	NA	NA	NA	NO	No	NA	0	753	0	753	0	753	0	753	C3
TCGA-DJ-A3VL	38	MALE	WHITE	T1	N0	M0	I	NA	Primary	NA	NO	No	NA	0	713	0	713	0	713	1	411	C3
TCGA-DJ-A4UL	68	FEMALE	WHITE	T1	N0	M0	I	NA	NA	NA	NO	No	NA	0	939	0	939	0	939	0	939	C3
TCGA-E8-A432	57	FEMALE	ASIAN	T2	N0	M0	II	NA	Recurrence	NA	NA	No	NA	0	931	0	931	1	720	1	720	C3
TCGA-E8-A433	25	FEMALE	ASIAN	T2	N0	M0	I	NA	NA	NA	NO	No	NA	0	951	0	951	0	951	0	951	C3
TCGA-EL-A3CX	22	FEMALE	WHITE	T2	N0	M0	I	NA	NA	NA	NO	No	NA	0	3243	0	3243	0	3243	0	3243	C3
TCGA-EL-A3D1	39	MALE	ASIAN	T2	N0	M0	I	NA	NA	NA	YES	No	NA	0	2997	0	2997	0	2997	0	2997	C3
TCGA-EL-A3D4	62	MALE	BLACK	T3	N1	M0	IV	NA	NA	NA	YES	No	NA	0	2116	0	2116	NA	NA	0	2116	C3
TCGA-EL-A3H1	66	FEMALE	BLACK	T1	N0	M0	I	NA	NA	NA	YES	No	NA	1	1500	0	1500	0	1500	0	1500	C3
TCGA-EL-A3H2	58	MALE	WHITE	T2	N0	NA	II	NA	NA	NA	YES	Yes	NA	1	1019	1	1019	NA	NA	1	1019	C3
TCGA-EL-A3ZM	60	MALE	NA	T4	N1	M0	IV	NA	NA	NA	YES	No	NA	0	1096	0	1096	NA	NA	0	1096	C3
TCGA-EL-A3ZN	28	FEMALE	WHITE	T3	N1	M0	I	NA	NA	NA	NO	No	NA	0	1614	0	1614	0	1614	0	1614	C3
TCGA-EL-A3ZQ	78	FEMALE	WHITE	T2	N0	M0	II	NA	NA	NA	YES	No	NA	0	1862	0	1862	0	1862	0	1862	C3
TCGA-EL-A3ZR	46	FEMALE	WHITE	T2	N0	M0	II	NA	NA	NA	YES	No	NA	0	1830	0	1830	0	1830	0	1830	C3

TCGA-EL-A4K7	74	MALE	WHITE	T2	N1	M0	III	NA	NA	NA	NO	No	NA	0	1251	0	1251	0	1251	0	1251	C3
TCGA-EL-A4KH	36	FEMALE	WHITE	T2	N0	M0	I	NA	NA	NA	YES	No	NA	0	823	0	823	0	823	0	823	C3
TCGA-EL-A4KI	63	MALE	WHITE	T3	N0	NA	III	NA	Metastasis	NA	YES	No	NA	0	727	0	727	1	265	1	265	C3
TCGA-EM-A1YE	47	FEMALE	NA	T2	N0	NA	II	NA	NA	NA	NA	No	NA	0	1645	0	1645	0	1645	0	1645	C3
TCGA-EM-A22Q	36	MALE	NA	T3	N0	NA	I	NA	NA	NA	NA	No	NA	0	433	0	433	0	433	0	433	C3
TCGA-EM-A2CR	46	FEMALE	NA	T2	N0	NA	II	NA	NA	NA	NA	No	NA	0	415	0	415	0	415	0	415	C3
TCGA-EM-A2CT	20	FEMALE	NA	T1	N0	NA	I	NA	NA	NA	NA	No	NA	0	1474	0	1474	0	1474	0	1474	C3
TCGA-EM-A3AI	67	FEMALE	WHITE	T2	NA	NA	II	NA	NA	NA	NA	No	NA	0	685	0	685	0	685	0	685	C3
TCGA-EM-A3AJ	39	MALE	NA	T3	N1	NA	I	NA	NA	NA	NA	No	NA	0	941	0	941	NA	NA	0	941	C3
TCGA-EM-A3AL	45	FEMALE	NA	T1	N0	NA	I	NA	NA	NA	NA	No	NA	0	430	0	430	0	430	0	430	C3
TCGA-EM-A3AQ	85	FEMALE	NA	T1	N0	NA	I	NA	NA	NA	NA	No	NA	0	904	0	904	0	904	0	904	C3
TCGA-EM-A3FL	63	FEMALE	NA	T1	NA	NA	I	NA	NA	NA	NA	No	NA	0	1441	0	1441	0	1441	0	1441	C3
TCGA-EM-A3FN	42	FEMALE	NA	T3	N0	NA	I	NA	NA	NA	NA	No	NA	0	367	0	367	0	367	0	367	C3
TCGA-EM-A3FP	71	FEMALE	NA	T2	N0	NA	II	NA	NA	NA	NA	No	NA	0	529	0	529	0	529	0	529	C3
TCGA-EM-A3O6	54	FEMALE	NA	T2	NA	NA	II	NA	NA	NA	YES	No	NA	0	458	0	458	0	458	0	458	C3
TCGA-EM-A3OA	55	FEMALE	NA	T2	N0	NA	II	NA	NA	NA	NO	No	NA	0	822	0	822	0	822	0	822	C3
TCGA-EM-A3OB	33	FEMALE	NA	T2	N0	NA	I	NA	NA	NA	NO	No	NA	0	1176	0	1176	0	1176	0	1176	C3
TCGA-EM-A3ST	62	FEMALE	NA	T3	N0	NA	III	NA	Primary	NA	YES	No	NA	0	1182	0	1182	1	966	1	966	C3
TCGA-EM-A3SU1	43	FEMALE	NA	T2	N1	NA	I	NA	NA	NA	YES	No	NA	0	911	0	911	0	911	0	911	C3
TCGA-EM-A4FH	69	MALE	NA	T3	NA	NA	III	NA	NA	NA	YES	No	NA	0	892	0	892	0	892	0	892	C3
TCGA-EM-A4FK	33	FEMALE	NA	T2	NA	NA	I	NA	NA	NA	NO	No	NA	0	649	0	649	0	649	0	649	C3
TCGA-ET-A2N0	51	FEMALE	WHITE	T1	N1	NA	III	NA	NA	NA	NA	No	NA	0	1600	0	1600	0	1600	0	1600	C3
TCGA-ET-A2N3	34	FEMALE	WHITE	T2	NA	NA	I	NA	NA	NA	YES	No	NA	0	2535	0	2535	NA	NA	0	2535	C3
TCGA-ET-A2N5	46	FEMALE	BLACK	T3	NA	NA	III	NA	NA	NA	NA	No	NA	0	2460	0	2460	NA	NA	0	2460	C3
TCGA-ET-A39K	48	FEMALE	WHITE	T3	N1	NA	III	NA	Recurrence	NA	NA	No	NA	0	3267	0	3267	NA	NA	1	711	C3
TCGA-ET-A39N	35	FEMALE	WHITE	T3	NA	NA	I	NA	NA	NA	NA	No	NA	0	3751	0	3751	0	3751	0	3751	C3
TCGA-ET-A3DV	68	FEMALE	WHITE	T3	N0	NA	II	NA	NA	NA	NA	No	NA	0	5068	0	5068	0	5068	0	5068	C3
TCGA-ET-A40P	32	FEMALE	WHITE	T2	N0	NA	I	NA	NA	NA	YES	No	NA	0	1338	0	1338	0	1338	0	1338	C3
TCGA-ET-A40R	24	FEMALE	WHITE	T2	N1	NA	I	NA	NA	NA	YES	No	NA	0	1350	0	1350	NA	NA	0	1350	C3
TCGA-ET-A4KN	51	FEMALE	WHITE	T3	N0	NA	III	NA	NA	NA	YES	No	NA	0	1009	0	1009	0	1009	0	1009	C3
TCGA-FE-A235	26	FEMALE	WHITE	T2	N1	NA	I	NA	NA	NA	NA	No	NA	0	3571	0	3571	0	3571	0	3571	C3
TCGA-FE-A239	82	MALE	WHITE	T3	N0	NA	III	NA	Recurrence	NA	NA	No	NA	1	1023	1	1023	1	933	1	933	C3
TCGA-FE-A3PD	17	FEMALE	WHITE	T2	N0	NA	I	NA	NA	NA	NA	No	NA	0	2396	0	2396	0	2396	0	2396	C3
TCGA-FK-A3SD	61	FEMALE	WHITE	T1	N0	M0	I	NA	NA	NA	NO	No	NA	0	32	0	32	0	32	0	32	C3
TCGA-FK-A3SH	50	FEMALE	WHITE	T2	N1	NA	III	NA	NA	NA	YES	No	NA	0	1463	0	1463	0	1463	0	1463	C3
TCGA-FY-A2QD	61	FEMALE	BLACK	T1	N0	NA		NA	NA	NA	NA	No	NA	0	38	0	38	NA	NA	0	38	C3
TCGA-FY-A3NM	48	FEMALE	WHITE	T3	N0	NA	III	NA	NA	NA	NA	No	NA	0	885	0	885	0	885	0	885	C3
TCGA-FY-A3NP	69	MALE	WHITE	T1	N0	M0	I	NA	NA	NA	NA	No	NA	0	491	0	491	0	491	0	491	C3
TCGA-FY-A4B0	76	MALE	WHITE	T1	NA	NA	I	NA	NA	NA	NA	No	Hormone Therapy	0	420	0	420	NA	NA	0	420	C3
TCGA-H2-A3RH	41	FEMALE	WHITE	T1	N0	M0	I	NA	NA	NA	NA	No	NA	0	379	0	379	NA	NA	0	379	C3
TCGA-IM-A41Z	37	FEMALE	WHITE	T1	N0	NA	I	NA	NA	NA	YES	No	Hormone Therapy	0	946	0	946	0	946	0	946	C3
TCGA-KS-A41F	37	FEMALE	WHITE	T2	N1	M0	I	NA	NA	NA	YES	No	Hormone Therapy	0	4367	0	4367	0	4367	0	4367	C3
TCGA-KS-A41J	28	FEMALE	WHITE	T2	N1	M0	I	NA	NA	NA	YES	No	Hormone Therapy	0	477	0	477	0	477	0	477	C3
TCGA-KS-A41L	39	FEMALE	WHITE	T2	N0	M0	I	NA	NA	NA	YES	No	Hormone Therapy	0	3463	0	3463	0	3463	0	3463	C3
TCGA-KS-A4I1	37	FEMALE	ASIAN	T2	N0	M0	I	NA	NA	NA	YES	No	Hormone Therapy	0	812	0	812	NA	NA	0	812	C3
TCGA-L6-A4EP	41	FEMALE	ASIAN	T2	N0	M0	I	NA	NA	NA	YES	No	NA	0	1016	0	1016	0	1016	0	1016	C3
TCGA-MK-A84Z	74	MALE	BLACK	T3	NA	NA	III	NA	NA	NA	NO	No	NA	0	728	0	728	NA	NA	0	728	C3
