# Supplementary material for: Clinical Relevance and Tumor Growth Suppression of Mitochondrial ROS Regulators along NADH:Ubiquinone Oxidoreductase Subunit B3 in Thyroid Cancer
Source: Oxid Med Cell Longev. 2022 Jan 17;2022:8038857. doi: 10.1155/2022/8038857 (PMC8787455; doi:10.1155/2022/8038857)
Supplement: Supplementary 2 — Supplementary Table 2: a table showing primer sequences and production lengths of human complex I subunit genes and MnSOD. Supplementary Table 3: a table showing the names and corresponding reference PubMed IDs of 35 mitoROS regulators. Supplementary Table 1: a table showing the cluster to which each TCGA sample ID belongs to the postconsensus clustering of 510 diagnosed thyroid cancer patients. Supplementary Table 4: a table showing the detailed clinical information of each TCGA sample within each cluster. Supplementary Table 5: a table showing the PFS duration of each TCGA sample within each cluster. Supplementary Table 6: a table showing the effect of high (50%) or low (50%) expression of each of the 35 mitoSOX regulators on PFS of 510 thyroid cancer patients in TCGA cohort. Supplementary Table 7: a table showing the exact expression value of each of the 35 mitoROS regulators for 510 TCGA samples. Supplementary Table 8: a table showing the detailed clinical information of each of 510 thyroid cancer samples divided into NDUFB3high and NDUFB3low. Supplementary Table 9: a table showing the log2FC, average expression values, and statistical parameters of 16961 genes based on the comparison of NDUFB3low (25%, n = 127) with NDFFB3high (25%, n = 127). Supplementary Table 10: a table showing statistical parameters of each of the 18 enriched KEGG pathways based on the comparison of NDUFB3low with NDFFB3high. Supplementary Table 11: a table showing the score of each of the 80 genes in the OXPHOS pathway based on the comparison of NDUFB3low with NDFFB3high. [file 8038857.f2.zip › Supplementary table 5.pdf]

rownames(datas)	Type	values	times	status
TCGA-DE-A4MD-06	G1(n=162)	7.97139278	0.73150685	1
TCGA-EM-A2P1-06	G1(n=162)	6.90678153	0.99452055	1
TCGA-EM-A3SU-06	G1(n=162)	7.68570632	2.49589041	0
TCGA-4C-A93U-01	G1(n=162)	8.00555675	0.35342466	0
TCGA-BJ-A0Z5-01	G1(n=162)	7.71498331	1.22739726	0
TCGA-BJ-A0Z9-01	G1(n=162)	6.75896298	3.44383562	0
TCGA-BJ-A0ZB-01	G1(n=162)	7.12461719	0.33424658	0
TCGA-BJ-A0ZH-01	G1(n=162)	7.78365472	1.26027397	0
TCGA-BJ-A18Z-01	G1(n=162)	7.53251254	3.46027397	0
TCGA-BJ-A28R-01	G1(n=162)	7.40879496	3.51232877	0
TCGA-BJ-A28T-01	G1(n=162)	6.84891776	2.96164384	0
TCGA-BJ-A28X-01	G1(n=162)	7.0393964	1.35342466	1
TCGA-BJ-A28Z-01	G1(n=162)	7.3692515	0.92328767	0
TCGA-BJ-A2NA-01	G1(n=162)	7.67778853	0.34794521	1
TCGA-BJ-A3EZ-01	G1(n=162)	7.64538215	1.33424658	0
TCGA-BJ-A3PR-01	G1(n=162)	7.82273728	3.03287671	0
TCGA-BJ-A45E-01	G1(n=162)	6.70895883	1.75068493	0
TCGA-BJ-A45I-01	G1(n=162)	7.73688728	0.30136986	1
TCGA-BJ-A4O8-01	G1(n=162)	7.64090231	1.61917808	0
TCGA-DE-A0Y2-01	G1(n=162)	6.83932625	1.80273973	0
TCGA-DE-A0Y3-01	G1(n=162)	6.84353145	0.08219178	1
TCGA-DE-A4M8-01	G1(n=162)	7.32187188	1.54520548	0
TCGA-DE-A4MB-01	G1(n=162)	7.61106944	0.96438356	0
TCGA-DE-A4MC-01	G1(n=162)	7.66740185	0.90958904	1
TCGA-DE-A4MD-01	G1(n=162)	7.22643428	0.73150685	1
TCGA-DE-A7U5-01	G1(n=162)	7.41197319	0.23013699	0
TCGA-DJ-A13L-01	G1(n=162)	8.09047274	0.83013699	0
TCGA-DJ-A13R-01	G1(n=162)	7.21991168	1.54794521	0
TCGA-DJ-A13U-01	G1(n=162)	6.87962385	4.35616438	0
TCGA-DJ-A13W-01	G1(n=162)	7.11955536	4.36438356	0
TCGA-DJ-A1QD-01	G1(n=162)	7.19918587	4.00547945	0
TCGA-DJ-A1QE-01	G1(n=162)	7.27383615	11.3260274	0
TCGA-DJ-A1QF-01	G1(n=162)	7.02833557	2.55068493	0
TCGA-DJ-A1QG-01	G1(n=162)	7.21523712	3.74794521	0
TCGA-DJ-A1QO-01	G1(n=162)	7.46352211	2.90958904	1
TCGA-DJ-A1QQ-01	G1(n=162)	7.29678151	1.4630137	0
TCGA-DJ-A2PO-01	G1(n=162)	7.35525805	0.66575342	0
TCGA-DJ-A2PR-01	G1(n=162)	7.25500755	2.28767123	0
TCGA-DJ-A2PV-01	G1(n=162)	7.2222617	2.66575342	0

TCGA-DJ-A2PY-01	G1(n=162)	7.27711998	0.73150685	0
TCGA-DJ-A2PZ-01	G1(n=162)	7.35714437	0.70136986	0
TCGA-DJ-A2Q1-01	G1(n=162)	7.85413005	3.14520548	0
TCGA-DJ-A2Q3-01	G1(n=162)	7.28735649	1.05479452	1
TCGA-DJ-A2Q4-01	G1(n=162)	7.67955578	2.82739726	0
TCGA-DJ-A2Q9-01	G1(n=162)	7.11722503	2.99178082	0
TCGA-DJ-A3UQ-01	G1(n=162)	7.72105457	1.63013699	0
TCGA-DJ-A3UW-01	G1(n=162)	7.56143037	4.96438356	0
TCGA-DJ-A3UY-01	G1(n=162)	7.29353756	2.9890411	0
TCGA-DJ-A3V5-01	G1(n=162)	7.50630364	1.6	0
TCGA-DJ-A3V6-01	G1(n=162)	7.9080497	2.24109589	0
TCGA-DJ-A3VB-01	G1(n=162)	8.21521516	1.90958904	0
TCGA-DJ-A3VD-01	G1(n=162)	7.21008251	3.39178082	0
TCGA-DJ-A3VE-01	G1(n=162)	7.38736889	1.58082192	0
TCGA-DJ-A3VF-01	G1(n=162)	7.20609951	0.8	0
TCGA-DJ-A3VI-01	G1(n=162)	7.77223867	0.26027397	0
TCGA-DJ-A4UT-01	G1(n=162)	7.86200584	2.25479452	0
TCGA-DJ-A4V2-01	G1(n=162)	7.55457594	0.26027397	0
TCGA-DJ-A4V4-01	G1(n=162)	7.798992	1.31780822	0
TCGA-DO-A1K0-01	G1(n=162)	6.81117896	1.58082192	1
TCGA-DO-A2HM-01	G1(n=162)	7.43650621	0.19452055	0
TCGA-E8-A242-01	G1(n=162)	7.58930696	2.60273973	0
TCGA-E8-A2EA-01	G1(n=162)	7.89293319	2.97260274	0
TCGA-E8-A2JQ-01	G1(n=162)	7.5956286	0.97534247	0
TCGA-E8-A413-01	G1(n=162)	7.55835207	2.49863014	0
TCGA-E8-A414-01	G1(n=162)	7.30157654	2.62465753	0
TCGA-E8-A417-01	G1(n=162)	7.25972818	2.56986301	0
TCGA-E8-A419-01	G1(n=162)	7.55111029	2.12054795	0
TCGA-E8-A436-01	G1(n=162)	7.4383603	1.24657534	0
TCGA-E8-A44K-01	G1(n=162)	7.19823403	2.58356164	0
TCGA-EL-A3CM-01	G1(n=162)	6.9375393	2.22739726	0
TCGA-EL-A3CN-01	G1(n=162)	7.13452532	12.9616438	0
TCGA-EL-A3CO-01	G1(n=162)	6.96164772	8.14520548	0
TCGA-EL-A3CS-01	G1(n=162)	7.02673998	3.79452055	1
TCGA-EL-A3CT-01	G1(n=162)	6.92659603	5.07945205	0
TCGA-EL-A3CU-01	G1(n=162)	6.98796812	13.0958904	0
TCGA-EL-A3CV-01	G1(n=162)	6.86112121	3.16164384	0
TCGA-EL-A3CY-01	G1(n=162)	6.86333994	0.34794521	0
TCGA-EL-A3D0-01	G1(n=162)	7.37738319	11.6547945	0
TCGA-EL-A3D6-01	G1(n=162)	7.34742755	9.03013699	0

TCGA-EL-A3GO-01	G1(n=162)	6.70923196	0.01643836	1
TCGA-EL-A3GP-01	G1(n=162)	7.36167173	4.1260274	0
TCGA-EL-A3GQ-01	G1(n=162)	6.74001227	4.18630137	0
TCGA-EL-A3GR-01	G1(n=162)	7.46357412	9.59178082	0
TCGA-EL-A3GS-01	G1(n=162)	7.48521704	5.44931507	0
TCGA-EL-A3GU-01	G1(n=162)	7.742693	1.49041096	1
TCGA-EL-A3GV-01	G1(n=162)	7.79134809	4.19726027	0
TCGA-EL-A3GX-01	G1(n=162)	7.61594873	1.20273973	1
TCGA-EL-A3GY-01	G1(n=162)	7.59849773	8.42739726	0
TCGA-EL-A3H4-01	G1(n=162)	6.92900675	4.14246575	0
TCGA-EL-A3H5-01	G1(n=162)	6.24683317	6.96438356	0
TCGA-EL-A3H7-01	G1(n=162)	7.51001664	11.5835616	0
TCGA-EL-A3MY-01	G1(n=162)	7.67196978	1.46027397	1
TCGA-EL-A3MZ-01	G1(n=162)	8.14185033	4.37534247	0
TCGA-EL-A3N2-01	G1(n=162)	7.31431702	2.47123288	0
TCGA-EL-A3N3-01	G1(n=162)	7.20828607	14.8575342	0
TCGA-EL-A3T1-01	G1(n=162)	7.67448954	10.7972603	0
TCGA-EL-A3T3-01	G1(n=162)	7.85776238	11.0054795	0
TCGA-EL-A3T6-01	G1(n=162)	7.25920071	8.18630137	0
TCGA-EL-A3T9-01	G1(n=162)	8.68079391	0.47671233	1
TCGA-EL-A3ZK-01	G1(n=162)	7.28207261	4.31506849	0
TCGA-EL-A3ZO-01	G1(n=162)	8.22336752	6.05753425	0
TCGA-EL-A3ZT-01	G1(n=162)	7.72658687	4.95342466	0
TCGA-EL-A4JW-01	G1(n=162)	7.39579401	2.89315068	0
TCGA-EL-A4JX-01	G1(n=162)	6.89225986	3.83013699	0
TCGA-EL-A4K1-01	G1(n=162)	7.48230568	2.86575342	0
TCGA-EL-A4K4-01	G1(n=162)	7.50571759	4.44657534	0
TCGA-EM-A1CT-01	G1(n=162)	6.5936289	1.51506849	0
TCGA-EM-A22I-01	G1(n=162)	7.03857291	1.22739726	0
TCGA-EM-A22M-01	G1(n=162)	8.31999462	1.2109589	0
TCGA-EM-A2CP-01	G1(n=162)	6.86999418	0.24931507	0
TCGA-EM-A2P1-01	G1(n=162)	6.60105434	0.99452055	1
TCGA-EM-A3FK-01	G1(n=162)	7.81742533	1.98082192	0
TCGA-EM-A3FM-01	G1(n=162)	8.21824222	1.40821918	1
TCGA-EM-A3O3-01	G1(n=162)	8.75736922	1.20821918	0
TCGA-EM-A3SZ-01	G1(n=162)	7.82349972	1.06575342	0
TCGA-EM-A4FF-01	G1(n=162)	7.52864028	4.89041096	1
TCGA-EM-A4FM-01	G1(n=162)	7.87453268	0.44383562	0
TCGA-EM-A4FQ-01	G1(n=162)	7.95663021	1.87123288	0
TCGA-ET-A25G-01	G1(n=162)	7.48636722	6.82739726	0

TCGA-ET-A25L-01	G1(n=162)	7.77916183	4.71506849	0
TCGA-ET-A25N-01	G1(n=162)	7.14358958	5.28219178	0
TCGA-ET-A25R-01	G1(n=162)	7.1846901	5.28767123	0
TCGA-ET-A39O-01	G1(n=162)	7.49133644	8.38082192	0
TCGA-ET-A3BN-01	G1(n=162)	7.00175739	2.35342466	0
TCGA-ET-A3BO-01	G1(n=162)	7.15027493	4.2	0
TCGA-ET-A3BS-01	G1(n=162)	7.4575401	0.48219178	0
TCGA-ET-A3BV-01	G1(n=162)	7.96529576	3.29863014	1
TCGA-ET-A3BX-01	G1(n=162)	7.30822849	4.37260274	0
TCGA-ET-A3DO-01	G1(n=162)	7.2402381	1.56164384	1
TCGA-ET-A3DU-01	G1(n=162)	7.67731515	5.23561644	0
TCGA-ET-A3DW-01	G1(n=162)	7.55751058	2.06027397	0
TCGA-ET-A40Q-01	G1(n=162)	7.89796298	0.59726027	1
TCGA-FE-A22Z-01	G1(n=162)	7.54861387	3.32876712	1
TCGA-FE-A230-01	G1(n=162)	6.94627615	1.44109589	1
TCGA-FE-A233-01	G1(n=162)	7.13220083	1.55342466	1
TCGA-FE-A234-01	G1(n=162)	7.86216136	1.32328767	1
TCGA-FE-A23A-01	G1(n=162)	7.35992254	6.81917808	0
TCGA-FE-A3PB-01	G1(n=162)	7.89902971	7.09589041	0
TCGA-FY-A3BL-01	G1(n=162)	7.37882732	1.70136986	0
TCGA-FY-A3NN-01	G1(n=162)	7.92072658	0	0
TCGA-FY-A3R8-01	G1(n=162)	7.88250467	1.30684932	0
TCGA-FY-A3TY-01	G1(n=162)	7.64190634	1.94246575	0
TCGA-FY-A3YR-01	G1(n=162)	7.56525871	1.44657534	0
TCGA-FY-A40L-01	G1(n=162)	7.53285578	1.9890411	0
TCGA-FY-A4B3-01	G1(n=162)	7.66607932	1.16986301	0
TCGA-H2-A26U-01	G1(n=162)	7.62689604	1.41643836	0
TCGA-H2-A421-01	G1(n=162)	7.82201263	1.67123288	0
TCGA-H2-A422-01	G1(n=162)	7.00405816	1.55068493	0
TCGA-IM-A3EB-01	G1(n=162)	7.88792929	4.07671233	0
TCGA-IM-A3ED-01	G1(n=162)	7.10967184	3.95068493	0
TCGA-IM-A3U2-01	G1(n=162)	7.9885698	1.54246575	0
TCGA-IM-A420-01	G1(n=162)	7.40692012	0.68493151	0
TCGA-IM-A4EB-01	G1(n=162)	7.77065624	2.1369863	0
TCGA-J8-A3O1-01	G1(n=162)	7.2231621	2.26575342	0
TCGA-J8-A3YF-01	G1(n=162)	7.92569445	2.05753425	0
TCGA-J8-A3YG-01	G1(n=162)	7.5176005	1.85753425	0
TCGA-KS-A41I-01	G1(n=162)	7.74502773	1.33150685	0
TCGA-KS-A4I9-01	G1(n=162)	7.70695478	7.28219178	0
TCGA-KS-A4IB-01	G1(n=162)	7.7054898	7.06027397	0

TCGA-KS-A4ID-01	G1(n=162)	7.58813183	2.76986301	0
TCGA-L6-A4EU-01	G1(n=162)	8.21164025	0.79178082	0
TCGA-MK-A4N6-01	G1(n=162)	7.43922178	3.00821918	0
TCGA-EM-A2CS-06	G2(n=261)	8.51931387	2.30410959	0
TCGA-J8-A3O2-06	G2(n=261)	8.00126025	0.9369863	0
TCGA-J8-A3YH-06	G2(n=261)	8.35713986	0.47945205	0
TCGA-J8-A4HW-06	G2(n=261)	7.78110773	0.21643836	0
TCGA-BJ-A0Z0-01	G2(n=261)	7.58806415	1.14794521	0
TCGA-BJ-A0Z2-01	G2(n=261)	7.93886571	0.43287671	1
TCGA-BJ-A0Z3-01	G2(n=261)	7.87365213	1.36164384	0
TCGA-BJ-A0ZA-01	G2(n=261)	7.35309463	1.57260274	0
TCGA-BJ-A0ZC-01	G2(n=261)	7.25033556	0.96438356	0
TCGA-BJ-A0ZE-01	G2(n=261)	7.80219678	1.39452055	0
TCGA-BJ-A0ZG-01	G2(n=261)	7.36182797	2.12328767	0
TCGA-BJ-A0ZJ-01	G2(n=261)	7.72712961	3.50684932	0
TCGA-BJ-A18Y-01	G2(n=261)	7.83709507	4.28219178	0
TCGA-BJ-A190-01	G2(n=261)	7.23724922	1.71506849	0
TCGA-BJ-A191-01	G2(n=261)	7.753194	0.40273973	0
TCGA-BJ-A28S-01	G2(n=261)	7.82704693	1.75342466	0
TCGA-BJ-A290-01	G2(n=261)	8.17915563	0.90136986	1
TCGA-BJ-A2N7-01	G2(n=261)	7.6980198	1.53150685	0
TCGA-BJ-A45C-01	G2(n=261)	8.17787181	0.66575342	1
TCGA-BJ-A45F-01	G2(n=261)	7.20316074	0.28767123	0
TCGA-BJ-A45G-01	G2(n=261)	7.99230869	1.98630137	0
TCGA-BJ-A45H-01	G2(n=261)	8.10590274	2.36986301	0
TCGA-BJ-A45J-01	G2(n=261)	8.37112754	1.80273973	0
TCGA-BJ-A4O9-01	G2(n=261)	7.91426207	1.95616438	0
TCGA-CE-A13K-01	G2(n=261)	8.00725404	1.05479452	0
TCGA-CE-A27D-01	G2(n=261)	7.9555186	1.02465753	0
TCGA-CE-A3ME-01	G2(n=261)	7.62463979	1.03835616	0
TCGA-CE-A481-01	G2(n=261)	8.04823704	0.94794521	0
TCGA-CE-A482-01	G2(n=261)	8.15885117	0.89041096	0
TCGA-CE-A483-01	G2(n=261)	7.48463168	1.25205479	0
TCGA-CE-A485-01	G2(n=261)	7.62164541	1.05479452	0
TCGA-DE-A0XZ-01	G2(n=261)	7.64427239	0.55890411	1
TCGA-DE-A3KN-01	G2(n=261)	8.31572803	2.27671233	0
TCGA-DE-A4M9-01	G2(n=261)	7.86908461	1.56986301	0
TCGA-DE-A69J-01	G2(n=261)	8.23067424	1.22465753	0
TCGA-DE-A69K-01	G2(n=261)	8.41334111	1.1890411	1
TCGA-DJ-A13O-01	G2(n=261)	7.85768043	3.18082192	0

TCGA-DJ-A13P-01	G2(n=261)	8.00463295	14.109589	0
TCGA-DJ-A13S-01	G2(n=261)	7.5080284	0.7260274	0
TCGA-DJ-A13T-01	G2(n=261)	7.74616723	4.68219178	0
TCGA-DJ-A13V-01	G2(n=261)	7.59068599	3.59726027	0
TCGA-DJ-A13X-01	G2(n=261)	7.63814494	0.69315068	0
TCGA-DJ-A1QH-01	G2(n=261)	7.69933523	3.54246575	0
TCGA-DJ-A1QI-01	G2(n=261)	8.01449262	1.24657534	0
TCGA-DJ-A1QM-01	G2(n=261)	7.29373697	0.50136986	0
TCGA-DJ-A1QN-01	G2(n=261)	8.3318972	3.54794521	0
TCGA-DJ-A2PN-01	G2(n=261)	7.40790708	0.84109589	0
TCGA-DJ-A2PP-01	G2(n=261)	7.13954713	2.54794521	0
TCGA-DJ-A2PQ-01	G2(n=261)	8.00451008	2.95068493	0
TCGA-DJ-A2PS-01	G2(n=261)	7.52706277	2.18356164	0
TCGA-DJ-A2PU-01	G2(n=261)	7.72924608	2.83013699	0
TCGA-DJ-A2PW-01	G2(n=261)	7.63766355	2.90410959	0
TCGA-DJ-A2PX-01	G2(n=261)	7.69840268	2.41369863	0
TCGA-DJ-A2Q0-01	G2(n=261)	7.11171374	2.8630137	0
TCGA-DJ-A2Q2-01	G2(n=261)	7.86160899	3.6630137	0
TCGA-DJ-A2Q5-01	G2(n=261)	7.56467254	3.12328767	0
TCGA-DJ-A2Q6-01	G2(n=261)	7.41913472	3.03835616	0
TCGA-DJ-A2Q7-01	G2(n=261)	8.12854165	2.9260274	0
TCGA-DJ-A2QB-01	G2(n=261)	7.249125	2.93150685	0
TCGA-DJ-A3UK-01	G2(n=261)	7.84581518	3.07671233	0
TCGA-DJ-A3UM-01	G2(n=261)	7.73988661	2.58630137	0
TCGA-DJ-A3UN-01	G2(n=261)	8.15490421	2.70136986	0
TCGA-DJ-A3UO-01	G2(n=261)	8.18918968	1.75616438	1
TCGA-DJ-A3UP-01	G2(n=261)	8.13877236	1.36712329	0
TCGA-DJ-A3UR-01	G2(n=261)	8.31232365	3.02739726	0
TCGA-DJ-A3US-01	G2(n=261)	7.79883436	2.73424658	0
TCGA-DJ-A3UU-01	G2(n=261)	7.73136367	1.95342466	0
TCGA-DJ-A3UV-01	G2(n=261)	8.09630772	4.01643836	0
TCGA-DJ-A3UX-01	G2(n=261)	7.56810358	0.94520548	0
TCGA-DJ-A3UZ-01	G2(n=261)	7.8598256	3.41369863	0
TCGA-DJ-A3V0-01	G2(n=261)	8.07023425	3.08219178	0
TCGA-DJ-A3V2-01	G2(n=261)	8.19008225	1.68493151	0
TCGA-DJ-A3V3-01	G2(n=261)	8.29850701	2.55068493	0
TCGA-DJ-A3V4-01	G2(n=261)	7.6054934	3.54520548	0
TCGA-DJ-A3V7-01	G2(n=261)	8.18901002	1.93424658	0
TCGA-DJ-A3V8-01	G2(n=261)	8.0282521	1.77260274	0
TCGA-DJ-A3V9-01	G2(n=261)	7.93578493	1.70684932	0

TCGA-DJ-A3VA-01	G2(n=261)	8.02440924	2.06849315	0
TCGA-DJ-A3VJ-01	G2(n=261)	8.53599618	3.03561644	0
TCGA-DJ-A3VM-01	G2(n=261)	7.30933567	2.30136986	0
TCGA-DJ-A4UP-01	G2(n=261)	8.10978716	2.50958904	0
TCGA-DJ-A4UQ-01	G2(n=261)	7.94472676	2.75342466	0
TCGA-DJ-A4UR-01	G2(n=261)	8.35007042	0.30958904	1
TCGA-DJ-A4UW-01	G2(n=261)	8.08232424	2.24383562	0
TCGA-DJ-A4V0-01	G2(n=261)	7.27011852	2.96164384	0
TCGA-DJ-A4V5-01	G2(n=261)	8.12895091	2.94794521	0
TCGA-DO-A1JZ-01	G2(n=261)	7.45816499	4.57534247	0
TCGA-E3-A3DY-01	G2(n=261)	7.75223661	7.71232877	0
TCGA-E3-A3DZ-01	G2(n=261)	7.90786206	3.93424658	0
TCGA-E3-A3E0-01	G2(n=261)	8.3289763	5.53150685	0
TCGA-E3-A3E1-01	G2(n=261)	7.73210997	6.70958904	0
TCGA-E3-A3E2-01	G2(n=261)	8.3690707	5.26575342	0
TCGA-E3-A3E3-01	G2(n=261)	7.53905244	1.21917808	1
TCGA-E3-A3E5-01	G2(n=261)	8.3298843	3.32876712	0
TCGA-E8-A3X7-01	G2(n=261)	8.19736991	2.60547945	0
TCGA-E8-A415-01	G2(n=261)	7.82718109	2.58630137	0
TCGA-E8-A416-01	G2(n=261)	7.72317469	2.60821918	0
TCGA-E8-A418-01	G2(n=261)	8.58733854	0.25205479	0
TCGA-E8-A434-01	G2(n=261)	7.81226906	2.6109589	0
TCGA-E8-A437-01	G2(n=261)	8.19681792	3.32328767	0
TCGA-E8-A438-01	G2(n=261)	8.93646614	2.79178082	0
TCGA-E8-A44M-01	G2(n=261)	7.54306806	2.51232877	0
TCGA-EL-A3CL-01	G2(n=261)	7.81033178	2.63835616	0
TCGA-EL-A3CP-01	G2(n=261)	7.47702742	13.2356164	0
TCGA-EL-A3CR-01	G2(n=261)	8.45722453	0.63835616	0
TCGA-EL-A3CW-01	G2(n=261)	7.17043997	4.75068493	0
TCGA-EL-A3CZ-01	G2(n=261)	7.02048466	3.90684932	1
TCGA-EL-A3D5-01	G2(n=261)	7.83768734	4.75890411	0
TCGA-EL-A3GW-01	G2(n=261)	8.20415918	4.18356164	0
TCGA-EL-A3GZ-01	G2(n=261)	8.19696817	11.6657534	0
TCGA-EL-A3H3-01	G2(n=261)	8.04248598	3.61369863	0
TCGA-EL-A3H8-01	G2(n=261)	7.58006753	12.539726	0
TCGA-EL-A3MW-01	G2(n=261)	8.07541129	9.05479452	0
TCGA-EL-A3MX-01	G2(n=261)	9.22372167	4.67945205	1
TCGA-EL-A3T0-01	G2(n=261)	8.04522246	11.8767123	0
TCGA-EL-A3T2-01	G2(n=261)	7.28705461	2.4739726	1
TCGA-EL-A3T7-01	G2(n=261)	7.80875182	3.44109589	0

TCGA-EL-A3T8-01	G2(n=261)	7.84330516	8.95616438	0
TCGA-EL-A3TA-01	G2(n=261)	8.36226427	5.57534247	0
TCGA-EL-A3TB-01	G2(n=261)	8.27881254	6.90136986	0
TCGA-EL-A3ZG-01	G2(n=261)	8.36245668	7.4630137	0
TCGA-EL-A3ZH-01	G2(n=261)	8.10075209	7.59726027	0
TCGA-EL-A3ZL-01	G2(n=261)	8.02692826	5.19178082	0
TCGA-EL-A3ZP-01	G2(n=261)	8.1869435	5.45753425	0
TCGA-EL-A3ZS-01	G2(n=261)	7.79254931	0.5890411	0
TCGA-EL-A4JV-01	G2(n=261)	8.20117235	4.7890411	0
TCGA-EL-A4JZ-01	G2(n=261)	8.372465	5.08493151	0
TCGA-EL-A4K0-01	G2(n=261)	7.45433949	2.58630137	0
TCGA-EL-A4K2-01	G2(n=261)	7.65373001	2.54246575	0
TCGA-EL-A4K6-01	G2(n=261)	8.59316918	2.39452055	1
TCGA-EL-A4K9-01	G2(n=261)	8.02815435	3.98630137	0
TCGA-EL-A4KD-01	G2(n=261)	8.15415969	3.63287671	0
TCGA-EL-A4KG-01	G2(n=261)	8.27228031	2.02465753	0
TCGA-EM-A1CS-01	G2(n=261)	7.30661766	2.23835616	0
TCGA-EM-A1CU-01	G2(n=261)	7.67810778	1.74246575	0
TCGA-EM-A1CV-01	G2(n=261)	7.46651836	4.04109589	0
TCGA-EM-A1CW-01	G2(n=261)	7.38375243	1.50410959	0
TCGA-EM-A1YA-01	G2(n=261)	7.02289575	1.64931507	0
TCGA-EM-A1YB-01	G2(n=261)	6.41606108	1.62191781	0
TCGA-EM-A1YC-01	G2(n=261)	7.59202544	6.55068493	0
TCGA-EM-A1YD-01	G2(n=261)	7.24182976	4.75068493	0
TCGA-EM-A22J-01	G2(n=261)	6.86635998	1.20821918	0
TCGA-EM-A22K-01	G2(n=261)	8.03464988	1.31232877	0
TCGA-EM-A22L-01	G2(n=261)	7.77848347	0.87123288	0
TCGA-EM-A22N-01	G2(n=261)	6.2117002	0.88767123	0
TCGA-EM-A22O-01	G2(n=261)	7.97591198	3.68219178	0
TCGA-EM-A22P-01	G2(n=261)	7.56346302	3.44109589	0
TCGA-EM-A2CJ-01	G2(n=261)	7.92276106	3.96438356	0
TCGA-EM-A2CK-01	G2(n=261)	7.98052454	1.49041096	0
TCGA-EM-A2CL-01	G2(n=261)	7.10864342	1.09863014	0
TCGA-EM-A2CM-01	G2(n=261)	7.36498865	3.43287671	0
TCGA-EM-A2CN-01	G2(n=261)	8.41305171	1.95890411	0
TCGA-EM-A2CO-01	G2(n=261)	6.90711569	0.50410959	0
TCGA-EM-A2CQ-01	G2(n=261)	6.93993493	1.02465753	0
TCGA-EM-A2CS-01	G2(n=261)	7.67453959	2.30410959	0
TCGA-EM-A2CU-01	G2(n=261)	8.37729003	1.09589041	0
TCGA-EM-A2OV-01	G2(n=261)	6.69571588	1.84657534	0

TCGA-EM-A2OW-01	G2(n=261)	6.85347923	3.36164384	0
TCGA-EM-A2OX-01	G2(n=261)	8.57844247	1.64383562	0
TCGA-EM-A2OY-01	G2(n=261)	7.44305433	2.69315068	0
TCGA-EM-A2OZ-01	G2(n=261)	7.91399597	2.83013699	0
TCGA-EM-A2P0-01	G2(n=261)	7.67093416	1.46575342	0
TCGA-EM-A2P2-01	G2(n=261)	8.44533432	1.44109589	0
TCGA-EM-A2P3-01	G2(n=261)	7.84357692	1.21643836	0
TCGA-EM-A3AK-01	G2(n=261)	7.77257658	1.30684932	0
TCGA-EM-A3AN-01	G2(n=261)	8.36982875	2.75616438	0
TCGA-EM-A3AO-01	G2(n=261)	7.93827866	3.70958904	0
TCGA-EM-A3AP-01	G2(n=261)	7.69790348	0.69589041	0
TCGA-EM-A3AR-01	G2(n=261)	7.67478215	3.10410959	0
TCGA-EM-A3FJ-01	G2(n=261)	8.76933601	2.32876712	0
TCGA-EM-A3FO-01	G2(n=261)	8.26200781	1.45753425	0
TCGA-EM-A3FQ-01	G2(n=261)	8.24353111	1.34794521	0
TCGA-EM-A3FR-01	G2(n=261)	8.19580514	1.25205479	0
TCGA-EM-A3O7-01	G2(n=261)	8.42260231	0.6109589	0
TCGA-EM-A3O8-01	G2(n=261)	7.92405139	1.04383562	0
TCGA-EM-A3SX-01	G2(n=261)	7.95122949	2.48767123	0
TCGA-EM-A3SY-01	G2(n=261)	7.4516066	1.1260274	0
TCGA-EM-A4FN-01	G2(n=261)	8.41373358	2.19726027	0
TCGA-EM-A4FO-01	G2(n=261)	7.88450265	2.19726027	0
TCGA-EM-A4FR-01	G2(n=261)	8.86728625	1.79452055	0
TCGA-EM-A4FU-01	G2(n=261)	8.02542424	2.13972603	1
TCGA-EM-A4FV-01	G2(n=261)	7.97348374	2.03835616	0
TCGA-EM-A4G1-01	G2(n=261)	7.59102626	1.13972603	0
TCGA-ET-A25I-01	G2(n=261)	7.30725194	6.80821918	0
TCGA-ET-A25K-01	G2(n=261)	7.70311759	5.86849315	0
TCGA-ET-A25M-01	G2(n=261)	7.57031946	5.01643836	0
TCGA-ET-A25O-01	G2(n=261)	7.63612425	5.44109589	0
TCGA-ET-A25P-01	G2(n=261)	7.97874373	1.53150685	0
TCGA-ET-A2MX-01	G2(n=261)	7.52654587	7.36712329	0
TCGA-ET-A2MY-01	G2(n=261)	7.51943547	4.89589041	0
TCGA-ET-A2MZ-01	G2(n=261)	7.79969994	3.10410959	0
TCGA-ET-A2N4-01	G2(n=261)	8.28310199	0.22191781	0
TCGA-ET-A39I-01	G2(n=261)	7.59033095	2.76986301	0
TCGA-ET-A39J-01	G2(n=261)	7.65169502	8.84109589	0
TCGA-ET-A39L-01	G2(n=261)	7.70140261	9.03835616	0
TCGA-ET-A39M-01	G2(n=261)	8.57726397	7.0739726	0
TCGA-ET-A39P-01	G2(n=261)	7.83602401	7.94520548	0

TCGA-ET-A39R-01	G2(n=261)	7.89562772	7.84383562	0
TCGA-ET-A39S-01	G2(n=261)	8.03379056	4.59178082	0
TCGA-ET-A39T-01	G2(n=261)	7.73946219	8.18356164	0
TCGA-ET-A3BP-01	G2(n=261)	8.2155432	6.59178082	0
TCGA-ET-A3BQ-01	G2(n=261)	7.91447048	6.38630137	0
TCGA-ET-A3BT-01	G2(n=261)	7.76196631	0.73150685	1
TCGA-ET-A3BU-01	G2(n=261)	7.7068349	6.48493151	0
TCGA-ET-A3BW-01	G2(n=261)	7.58248723	3.88767123	0
TCGA-ET-A3DP-01	G2(n=261)	7.98808236	7.88219178	0
TCGA-ET-A3DQ-01	G2(n=261)	7.46202386	7.07123288	0
TCGA-ET-A3DR-01	G2(n=261)	7.53086672	6.71506849	0
TCGA-ET-A3DS-01	G2(n=261)	7.03248334	5.56986301	0
TCGA-ET-A40S-01	G2(n=261)	8.38703181	3.35068493	0
TCGA-ET-A40T-01	G2(n=261)	8.20463544	3.12328767	0
TCGA-ET-A4KQ-01	G2(n=261)	7.73531171	2.61643836	0
TCGA-FE-A231-01	G2(n=261)	7.75834688	2.63287671	0
TCGA-FE-A232-01	G2(n=261)	8.23144868	6.64109589	0
TCGA-FE-A236-01	G2(n=261)	7.42024806	5.56438356	0
TCGA-FE-A237-01	G2(n=261)	7.74433272	1.34794521	1
TCGA-FE-A238-01	G2(n=261)	7.84800899	1.18082192	1
TCGA-FE-A3PA-01	G2(n=261)	8.97688733	8.8630137	0
TCGA-FE-A3PC-01	G2(n=261)	8.44179052	7.13972603	0
TCGA-FK-A3S3-01	G2(n=261)	8.28511671	5.92328767	0
TCGA-FK-A3SB-01	G2(n=261)	8.08402677	0.34246575	0
TCGA-FK-A3SE-01	G2(n=261)	7.9201871	1.83561644	0
TCGA-FK-A3SG-01	G2(n=261)	9.00762205	0.07671233	0
TCGA-FK-A4UB-01	G2(n=261)	8.45612147	9.6739726	0
TCGA-FY-A3I4-01	G2(n=261)	7.8006036	1.48493151	0
TCGA-FY-A3I5-01	G2(n=261)	7.77545702	0.65205479	0
TCGA-FY-A3ON-01	G2(n=261)	7.92851752	1.63013699	0
TCGA-FY-A3R6-01	G2(n=261)	7.7962251	1.15890411	0
TCGA-FY-A3R7-01	G2(n=261)	7.81535651	1.35616438	0
TCGA-FY-A3R9-01	G2(n=261)	7.30328039	1.38082192	0
TCGA-FY-A3RA-01	G2(n=261)	7.80927245	1.50684932	0
TCGA-FY-A3W9-01	G2(n=261)	8.3446337	1.80273973	0
TCGA-FY-A3WA-01	G2(n=261)	7.24458462	1.42191781	0
TCGA-FY-A40K-01	G2(n=261)	7.59428416	1.2630137	0
TCGA-FY-A40M-01	G2(n=261)	8.39484501	1.8739726	0
TCGA-FY-A40N-01	G2(n=261)	8.06924261	1.58630137	0
TCGA-FY-A4B4-01	G2(n=261)	7.80936395	1.8	0

TCGA-FY-A76V-01	G2(n=261)	8.03998689	0.13972603	0
TCGA-GE-A2C6-01	G2(n=261)	7.53329716	1.04383562	0
TCGA-H2-A2K9-01	G2(n=261)	7.85915181	1.80547945	0
TCGA-H2-A3RI-01	G2(n=261)	7.73448955	0.76438356	1
TCGA-IM-A3U3-01	G2(n=261)	7.90573604	1.40821918	0
TCGA-IM-A41Y-01	G2(n=261)	8.00438027	1.81917808	0
TCGA-J8-A3NZ-01	G2(n=261)	8.14559184	1.43561644	0
TCGA-J8-A3O0-01	G2(n=261)	8.28473221	3.59726027	0
TCGA-J8-A3O2-01	G2(n=261)	8.08551158	0.9369863	0
TCGA-J8-A3YD-01	G2(n=261)	7.83322271	2.65205479	0
TCGA-J8-A3YE-01	G2(n=261)	8.19775868	2.32054795	0
TCGA-J8-A3YH-01	G2(n=261)	8.39277737	0.47945205	0
TCGA-J8-A42S-01	G2(n=261)	8.36597291	0.20547945	0
TCGA-J8-A4HW-01	G2(n=261)	7.80971682	0.21643836	0
TCGA-J8-A4HY-01	G2(n=261)	8.0887761	0.01643836	0
TCGA-KS-A4I3-01	G2(n=261)	7.67927363	3.64109589	0
TCGA-KS-A4I5-01	G2(n=261)	8.2130904	1.55342466	0
TCGA-KS-A4I7-01	G2(n=261)	8.56969203	2.70410959	0
TCGA-KS-A4IC-01	G2(n=261)	7.93355652	2.93972603	0
TCGA-L6-A4EQ-01	G2(n=261)	8.27834997	3.36438356	0
TCGA-L6-A4ET-01	G2(n=261)	8.28827986	1.81917808	0
TCGA-MK-A4N7-01	G2(n=261)	7.88803946	2.70410959	0
TCGA-MK-A4N9-01	G2(n=261)	8.08608519	2.51780822	0
TCGA-QD-A8IV-01	G2(n=261)	7.7951833	1.0739726	0
TCGA-EM-A3FQ-06	G3(n=87)	9.88108249	1.34794521	0
TCGA-BJ-A0YZ-01	G3(n=87)	7.88843865	2.06575342	0
TCGA-BJ-A0ZF-01	G3(n=87)	7.9631379	2.01369863	1
TCGA-BJ-A192-01	G3(n=87)	7.58617969	3.69589041	0
TCGA-BJ-A28V-01	G3(n=87)	7.91709269	2.70410959	0
TCGA-BJ-A28W-01	G3(n=87)	8.61453162	0.94246575	0
TCGA-BJ-A291-01	G3(n=87)	8.28636172	1.90684932	0
TCGA-BJ-A2N8-01	G3(n=87)	8.84292437	1.38082192	0
TCGA-BJ-A2N9-01	G3(n=87)	8.17727831	0.61643836	1
TCGA-BJ-A2P4-01	G3(n=87)	9.18645331	1.06849315	0
TCGA-BJ-A3F0-01	G3(n=87)	8.3275551	1.16164384	0
TCGA-BJ-A3PT-01	G3(n=87)	8.53219942	1.94246575	0
TCGA-BJ-A3PU-01	G3(n=87)	8.85923695	0.47671233	1
TCGA-BJ-A45D-01	G3(n=87)	9.54256901	0.90684932	0
TCGA-BJ-A45K-01	G3(n=87)	8.43918833	1.93150685	0
TCGA-CE-A3MD-01	G3(n=87)	8.27194472	1.06575342	0

TCGA-CE-A484-01	G3(n=87)	8.85808361	1.03561644	0
TCGA-DE-A2OL-01	G3(n=87)	8.08245339	3.1369863	0
TCGA-DE-A4MA-01	G3(n=87)	9.31019792	2.50958904	0
TCGA-DJ-A13M-01	G3(n=87)	8.98151423	4.06575342	0
TCGA-DJ-A1QL-01	G3(n=87)	8.38169527	1.46575342	0
TCGA-DJ-A2PT-01	G3(n=87)	8.3146671	3.23561644	0
TCGA-DJ-A2QA-01	G3(n=87)	9.38813848	2.79452055	0
TCGA-DJ-A2QC-01	G3(n=87)	9.12820257	3.32876712	0
TCGA-DJ-A3UT-01	G3(n=87)	8.32227412	3.02739726	0
TCGA-DJ-A3VG-01	G3(n=87)	8.49377178	2.97260274	0
TCGA-DJ-A3VK-01	G3(n=87)	8.35688328	2.0630137	0
TCGA-DJ-A3VL-01	G3(n=87)	8.88322461	1.1260274	1
TCGA-DJ-A4UL-01	G3(n=87)	9.07755426	2.57260274	0
TCGA-E8-A432-01	G3(n=87)	8.74692746	1.97260274	1
TCGA-E8-A433-01	G3(n=87)	8.62271201	2.60547945	0
TCGA-EL-A3CX-01	G3(n=87)	8.61335921	8.88493151	0
TCGA-EL-A3D1-01	G3(n=87)	9.32321568	8.2109589	0
TCGA-EL-A3D4-01	G3(n=87)	8.81493077	5.79726027	0
TCGA-EL-A3H1-01	G3(n=87)	8.22770747	4.10958904	0
TCGA-EL-A3H2-01	G3(n=87)	7.86979694	2.79178082	1
TCGA-EL-A3ZM-01	G3(n=87)	8.647907	3.00273973	0
TCGA-EL-A3ZN-01	G3(n=87)	8.68957003	4.42191781	0
TCGA-EL-A3ZQ-01	G3(n=87)	10.4160214	5.10136986	0
TCGA-EL-A3ZR-01	G3(n=87)	8.57863821	5.01369863	0
TCGA-EL-A4K7-01	G3(n=87)	9.40351359	3.42739726	0
TCGA-EL-A4KH-01	G3(n=87)	9.41828095	2.25479452	0
TCGA-EL-A4KI-01	G3(n=87)	10.2201545	0.7260274	1
TCGA-EM-A1YE-01	G3(n=87)	8.44180851	4.50684932	0
TCGA-EM-A22Q-01	G3(n=87)	10.3105495	1.18630137	0
TCGA-EM-A2CR-01	G3(n=87)	9.62273432	1.1369863	0
TCGA-EM-A2CT-01	G3(n=87)	7.65564106	4.03835616	0
TCGA-EM-A3AI-01	G3(n=87)	8.49824835	1.87671233	0
TCGA-EM-A3AJ-01	G3(n=87)	7.82927257	2.57808219	0
TCGA-EM-A3AL-01	G3(n=87)	8.50353413	1.17808219	0
TCGA-EM-A3AQ-01	G3(n=87)	8.65690071	2.47671233	0
TCGA-EM-A3FL-01	G3(n=87)	7.63534651	3.94794521	0
TCGA-EM-A3FN-01	G3(n=87)	8.61358021	1.00547945	0
TCGA-EM-A3FP-01	G3(n=87)	8.49363918	1.44931507	0
TCGA-EM-A3O6-01	G3(n=87)	8.19400793	1.25479452	0
TCGA-EM-A3OA-01	G3(n=87)	8.07105277	2.25205479	0

TCGA-EM-A3OB-01	G3(n=87)	8.59996196	3.22191781	0
TCGA-EM-A3ST-01	G3(n=87)	10.0058674	2.64657534	1
TCGA-EM-A3SU-01	G3(n=87)	8.66223284	2.49589041	0
TCGA-EM-A4FH-01	G3(n=87)	8.1955834	2.44383562	0
TCGA-EM-A4FK-01	G3(n=87)	8.01750761	1.77808219	0
TCGA-ET-A2N0-01	G3(n=87)	9.51483927	4.38356164	0
TCGA-ET-A2N3-01	G3(n=87)	9.16419096	6.94520548	0
TCGA-ET-A2N5-01	G3(n=87)	8.1921452	6.73972603	0
TCGA-ET-A39K-01	G3(n=87)	8.91007845	1.94794521	1
TCGA-ET-A39N-01	G3(n=87)	9.00276857	10.2767123	0
TCGA-ET-A3DV-01	G3(n=87)	8.46025053	13.8849315	0
TCGA-ET-A40P-01	G3(n=87)	9.36505589	3.66575342	0
TCGA-ET-A40R-01	G3(n=87)	9.01577183	3.69863014	0
TCGA-ET-A4KN-01	G3(n=87)	10.1940462	2.76438356	0
TCGA-FE-A235-01	G3(n=87)	8.82571961	9.78356164	0
TCGA-FE-A239-01	G3(n=87)	8.02039306	2.55616438	1
TCGA-FE-A3PD-01	G3(n=87)	8.73178545	6.56438356	0
TCGA-FK-A3SD-01	G3(n=87)	8.48934219	0.08767123	0
TCGA-FK-A3SH-01	G3(n=87)	9.25287702	4.00821918	0
TCGA-FY-A2QD-01	G3(n=87)	7.55885083	0.10410959	0
TCGA-FY-A3NM-01	G3(n=87)	7.44042184	2.42465753	0
TCGA-FY-A3NP-01	G3(n=87)	7.75934889	1.34520548	0
TCGA-FY-A4B0-01	G3(n=87)	7.91490612	1.15068493	0
TCGA-H2-A3RH-01	G3(n=87)	8.61446863	1.03835616	0
TCGA-IM-A41Z-01	G3(n=87)	8.08404843	2.59178082	0
TCGA-KS-A41F-01	G3(n=87)	10.4140994	11.9643836	0
TCGA-KS-A41J-01	G3(n=87)	8.48435776	1.30684932	0
TCGA-KS-A41L-01	G3(n=87)	8.49976965	9.48767123	0
TCGA-KS-A4I1-01	G3(n=87)	9.29928324	2.22465753	0
TCGA-L6-A4EP-01	G3(n=87)	9.09375717	2.78356164	0
TCGA-MK-A84Z-01	G3(n=87)	8.53718949	1.99452055	0
