# Supplementary material for: Clinical Relevance and Tumor Growth Suppression of Mitochondrial ROS Regulators along NADH:Ubiquinone Oxidoreductase Subunit B3 in Thyroid Cancer
Source: Oxid Med Cell Longev. 2022 Jan 17;2022:8038857. doi: 10.1155/2022/8038857 (PMC8787455; doi:10.1155/2022/8038857)
Supplement: Supplementary 2 — Supplementary Table 2: a table showing primer sequences and production lengths of human complex I subunit genes and MnSOD. Supplementary Table 3: a table showing the names and corresponding reference PubMed IDs of 35 mitoROS regulators. Supplementary Table 1: a table showing the cluster to which each TCGA sample ID belongs to the postconsensus clustering of 510 diagnosed thyroid cancer patients. Supplementary Table 4: a table showing the detailed clinical information of each TCGA sample within each cluster. Supplementary Table 5: a table showing the PFS duration of each TCGA sample within each cluster. Supplementary Table 6: a table showing the effect of high (50%) or low (50%) expression of each of the 35 mitoSOX regulators on PFS of 510 thyroid cancer patients in TCGA cohort. Supplementary Table 7: a table showing the exact expression value of each of the 35 mitoROS regulators for 510 TCGA samples. Supplementary Table 8: a table showing the detailed clinical information of each of 510 thyroid cancer samples divided into NDUFB3high and NDUFB3low. Supplementary Table 9: a table showing the log2FC, average expression values, and statistical parameters of 16961 genes based on the comparison of NDUFB3low (25%, n = 127) with NDFFB3high (25%, n = 127). Supplementary Table 10: a table showing statistical parameters of each of the 18 enriched KEGG pathways based on the comparison of NDUFB3low with NDFFB3high. Supplementary Table 11: a table showing the score of each of the 80 genes in the OXPHOS pathway based on the comparison of NDUFB3low with NDFFB3high. [file 8038857.f2.zip › Supplementary table 6.pdf]

Genes	p.value	HR	Low 95%CI	High 95%CI
NDUFB3	0.00110791	0.36293878	0.19739074	0.667328959
COX7A2	0.00470963	0.43038684	0.23985321	0.772275802
UQCR10	0.00775129	0.45192639	0.25186681	0.810894693
NDUFA1	0.00839306	0.46177398	0.25995888	0.820265168
DUOX2	0.00903735	0.47060493	0.26723901	0.82873005
NDUFB1	0.01178286	0.48321153	0.27436737	0.85102462
COX7B	0.01549202	0.49715568	0.28232458	0.875459651
DUOXA1	0.02277827	0.52257009	0.29891464	0.913570171
DUOX1	0.02481018	0.52746053	0.30170416	0.922143759
COX8A	0.03157644	0.54191112	0.30998712	0.947354402
NDUFA8	0.03509952	0.5483565	0.31358796	0.958885215
DUOXA2	0.0454468	0.56897966	0.32745784	0.988639804
SURF1	0.05852521	0.5864667	0.33738752	1.019430684
COX6C	0.06778146	0.59754426	0.34386951	1.038356525
ETFB	0.07867168	0.61198486	0.35403142	1.057887654
UQCC3	0.11057898	0.64276874	0.37348845	1.106196582
NDUFA2	0.16255197	0.67919733	0.39464982	1.168907184
UQCRQ	0.17255846	0.68532445	0.39819576	1.179494258
SCO1	0.18297226	0.69144251	0.40170355	1.1901631
PDC	0.18857156	1.43623359	0.83724702	2.463749492
NDUFB7	0.18981799	0.69524234	0.40378368	1.197081362
UCP2	0.19739064	0.69973175	0.40658299	1.204242489
DLAT	0.20560668	0.70575421	0.41143123	1.210625198
COX6B1	0.23080612	0.7189079	0.41902713	1.233401195
NDUFB2	0.24765991	0.72568822	0.42137283	1.249780135
COX5A	0.27734777	0.741503	0.43227743	1.271930183
NOX4	0.30073453	0.75298005	0.43996537	1.288689965
COX15	0.36472174	0.78012237	0.45601677	1.334580112
CYC1	0.48672809	0.8265192	0.48314094	1.413943481
ATP6V1E1	0.62095231	0.87374321	0.5117496	1.491798323
NDUFB10	0.66956346	0.8902648	0.52200066	1.518334108
ROMO1	0.75732812	0.91926033	0.53892119	1.568020646
SUCLG2	0.833488	1.05892	0.62096262	1.805763365
SLC25A5	0.95595166	1.01515149	0.59537039	1.730909976
SOD2	0.99090975	0.99690274	0.58464579	1.69985842
