# Supplementary material for: Clinical Relevance and Tumor Growth Suppression of Mitochondrial ROS Regulators along NADH:Ubiquinone Oxidoreductase Subunit B3 in Thyroid Cancer
Source: Oxid Med Cell Longev. 2022 Jan 17;2022:8038857. doi: 10.1155/2022/8038857 (PMC8787455; doi:10.1155/2022/8038857)
Supplement: Supplementary 2 — Supplementary Table 2: a table showing primer sequences and production lengths of human complex I subunit genes and MnSOD. Supplementary Table 3: a table showing the names and corresponding reference PubMed IDs of 35 mitoROS regulators. Supplementary Table 1: a table showing the cluster to which each TCGA sample ID belongs to the postconsensus clustering of 510 diagnosed thyroid cancer patients. Supplementary Table 4: a table showing the detailed clinical information of each TCGA sample within each cluster. Supplementary Table 5: a table showing the PFS duration of each TCGA sample within each cluster. Supplementary Table 6: a table showing the effect of high (50%) or low (50%) expression of each of the 35 mitoSOX regulators on PFS of 510 thyroid cancer patients in TCGA cohort. Supplementary Table 7: a table showing the exact expression value of each of the 35 mitoROS regulators for 510 TCGA samples. Supplementary Table 8: a table showing the detailed clinical information of each of 510 thyroid cancer samples divided into NDUFB3high and NDUFB3low. Supplementary Table 9: a table showing the log2FC, average expression values, and statistical parameters of 16961 genes based on the comparison of NDUFB3low (25%, n = 127) with NDFFB3high (25%, n = 127). Supplementary Table 10: a table showing statistical parameters of each of the 18 enriched KEGG pathways based on the comparison of NDUFB3low with NDFFB3high. Supplementary Table 11: a table showing the score of each of the 80 genes in the OXPHOS pathway based on the comparison of NDUFB3low with NDFFB3high. [file 8038857.f2.zip › Supplementary table 7.pdf]

rownames (datas.final)	Type	Values	Group
1	NDUFA1	7.839642267	Tumor_G1
2	NDUFA2	6.901279668	Tumor_G1
3	NDUFA8	6.916634304	Tumor_G1
4	NDUFB1	5.573372018	Tumor_G1
5	NDUFB10	7.880803727	Tumor_G1
6	NDUFB2	6.213885747	Tumor_G1
7	NDUFB3	5.671802455	Tumor_G1
8	NDUFB7	8.533851314	Tumor_G1
9	CYC1	6.865884646	Tumor_G1
10	ETFB	4.855080708	Tumor_G1
11	UQCR10	7.343539248	Tumor_G1
12	UQCRQ	7.494016406	Tumor_G1
13	UQCC3	3.729757746	Tumor_G1
14	COX15	4.123425224	Tumor_G1
15	COX5A	7.196844323	Tumor_G1
16	COX6B1	8.767707718	Tumor_G1
17	COX6C	6.436890183	Tumor_G1
18	COX7A2	6.517135913	Tumor_G1
19	COX7B	5.550401568	Tumor_G1
20	COX8A	8.373855639	Tumor_G1
21	SCO1	2.262540645	Tumor_G1
22	SURF1	5.698555039	Tumor_G1
23	ATP6V1E1	6.623781043	Tumor_G1
24	DLAT	3.550448541	Tumor_G1
25	PDC	0.041433796	Tumor_G1
26	SUCLG2	5.772053758	Tumor_G1
27	SLC25A5	7.273443051	Tumor_G1
28	UCP2	6.53327483	Tumor_G1
29	SOD2	4.153575997	Tumor_G1
30	NOX4	1.749118155	Tumor_G1
31	DUOX1	4.809375138	Tumor_G1
32	DUOXA1	4.428201051	Tumor_G1
33	DUOX2	4.061957503	Tumor_G1
34	DUOXA2	3.090086568	Tumor_G1
35	NDUFA1	7.851994698	Tumor_G1
36	NDUFA2	5.846170977	Tumor_G1
37	NDUFA8	6.46015489	Tumor_G1
38	NDUFB1	5.247018435	Tumor_G1

39	NDUFB10	6.74537787	Tumor_G1
40	NDUFB2	5.246339475	Tumor_G1
41	NDUFB3	6.043989643	Tumor_G1
42	NDUFB7	7.636328234	Tumor_G1
43	CYC1	5.764751244	Tumor_G1
44	ETFB	3.931052804	Tumor_G1
45	UQCR10	7.213560224	Tumor_G1
46	UQCRQ	6.663102122	Tumor_G1
47	UQCC3	2.933540287	Tumor_G1
48	COX15	4.401701007	Tumor_G1
49	COX5A	6.716531043	Tumor_G1
50	COX6B1	8.453808286	Tumor_G1
51	COX6C	5.908869744	Tumor_G1
52	COX7A2	6.67024768	Tumor_G1
53	COX7B	6.173962391	Tumor_G1
54	COX8A	7.986817421	Tumor_G1
55	SCO1	2.885830032	Tumor_G1
56	SURF1	5.530933262	Tumor_G1
57	ATP6V1E1	6.874852963	Tumor_G1
58	DLAT	4.882187031	Tumor_G1
59	PDC	0	Tumor_G1
60	SUCLG2	5.882569357	Tumor_G1
61	SLC25A5	7.836826072	Tumor_G1
62	UCP2	7.56332733	Tumor_G1
63	SOD2	5.81752386	Tumor_G1
64	NOX4	3.062036088	Tumor_G1
65	DUOX1	4.909123357	Tumor_G1
66	DUOXA1	5.188041246	Tumor_G1
67	DUOX2	3.795347978	Tumor_G1
68	DUOXA2	1.735844072	Tumor_G1
69	NDUFA1	7.794803336	Tumor_G1
70	NDUFA2	6.677111084	Tumor_G1
71	NDUFA8	6.674515029	Tumor_G1
72	NDUFB1	5.479799873	Tumor_G1
73	NDUFB10	7.345611123	Tumor_G1
74	NDUFB2	6.138629914	Tumor_G1
75	NDUFB3	6.242337072	Tumor_G1
76	NDUFB7	8.414870107	Tumor_G1
77	CYC1	6.639078562	Tumor_G1
78	ETFB	4.865690706	Tumor_G1

79	UQCR10	7.411999669	Tumor_G1
80	UQCRQ	7.151716163	Tumor_G1
81	UQCC3	4.297420791	Tumor_G1
82	COX15	4.495081471	Tumor_G1
83	COX5A	6.996684409	Tumor_G1
84	COX6B1	8.58877593	Tumor_G1
85	COX6C	6.231336336	Tumor_G1
86	COX7A2	6.713842924	Tumor_G1
87	COX7B	5.953148322	Tumor_G1
88	COX8A	9.366409733	Tumor_G1
89	SCO1	3.048278404	Tumor_G1
90	SURF1	6.291880307	Tumor_G1
91	ATP6V1E1	6.91845911	Tumor_G1
92	DLAT	4.737358762	Tumor_G1
93	PDC	0.041474902	Tumor_G1
94	SUCLG2	5.631739699	Tumor_G1
95	SLC25A5	8.373267826	Tumor_G1
96	UCP2	8.490550393	Tumor_G1
97	SOD2	5.130912688	Tumor_G1
98	NOX4	2.319356383	Tumor_G1
99	DUOX1	5.803336533	Tumor_G1
100	DUOXA1	6.150442711	Tumor_G1
101	DUOX2	5.322676094	Tumor_G1
102	DUOXA2	2.815541904	Tumor_G1
103	NDUFA1	7.967265091	Tumor_G1
104	NDUFA2	6.67140129	Tumor_G1
105	NDUFA8	6.075305251	Tumor_G1
106	NDUFB1	5.589816967	Tumor_G1
107	NDUFB10	7.44936714	Tumor_G1
108	NDUFB2	6.136160482	Tumor_G1
109	NDUFB3	6.29178362	Tumor_G1
110	NDUFB7	8.424591188	Tumor_G1
111	CYC1	6.536539287	Tumor_G1
112	ETFB	4.442281594	Tumor_G1
113	UQCR10	6.535504168	Tumor_G1
114	UQCRQ	7.209786315	Tumor_G1
115	UQCC3	3.825644595	Tumor_G1
116	COX15	4.790739423	Tumor_G1
117	COX5A	6.870525972	Tumor_G1
118	COX6B1	8.45013027	Tumor_G1

119	COX6C	6.158072641	Tumor_G1
120	COX7A2	6.657533655	Tumor_G1
121	COX7B	6.094497828	Tumor_G1
122	COX8A	9.18066684	Tumor_G1
123	SCO1	2.99965329	Tumor_G1
124	SURF1	5.57037345	Tumor_G1
125	ATP6V1E1	6.363573116	Tumor_G1
126	DLAT	5.099968773	Tumor_G1
127	PDC	0.271461649	Tumor_G1
128	SUCLG2	6.602474411	Tumor_G1
129	SLC25A5	7.768873844	Tumor_G1
130	UCP2	8.386691077	Tumor_G1
131	SOD2	5.286516401	Tumor_G1
132	NOX4	2.685764682	Tumor_G1
133	DUOX1	6.382116529	Tumor_G1
134	DUOXA1	5.989649472	Tumor_G1
135	DUOX2	6.131466015	Tumor_G1
136	DUOXA2	4.055705906	Tumor_G1
137	NDUFA1	8.004634348	Tumor_G1
138	NDUFA2	6.600127366	Tumor_G1
139	NDUFA8	6.696704364	Tumor_G1
140	NDUFB1	5.328072799	Tumor_G1
141	NDUFB10	7.330024797	Tumor_G1
142	NDUFB2	6.52426214	Tumor_G1
143	NDUFB3	5.686250862	Tumor_G1
144	NDUFB7	8.456376429	Tumor_G1
145	CYC1	6.795252487	Tumor_G1
146	ETFB	5.237591417	Tumor_G1
147	UQCR10	7.2593286	Tumor_G1
148	UQCRQ	7.191499153	Tumor_G1
149	UQCC3	4.108116464	Tumor_G1
150	COX15	4.583525754	Tumor_G1
151	COX5A	6.913465461	Tumor_G1
152	COX6B1	8.517802889	Tumor_G1
153	COX6C	6.205166902	Tumor_G1
154	COX7A2	6.558785733	Tumor_G1
155	COX7B	5.695585267	Tumor_G1
156	COX8A	9.217164772	Tumor_G1
157	SCO1	2.639550249	Tumor_G1
158	SURF1	5.717692126	Tumor_G1

159	ATP6V1E1	6.754756875	Tumor_G1
160	DLAT	4.67211474	Tumor_G1
161	PDC	0.050059037	Tumor_G1
162	SUCLG2	5.676310515	Tumor_G1
163	SLC25A5	7.520030665	Tumor_G1
164	UCP2	8.590283209	Tumor_G1
165	SOD2	4.233612447	Tumor_G1
166	NOX4	1.342301188	Tumor_G1
167	DUOX1	6.54850748	Tumor_G1
168	DUOXA1	6.271124274	Tumor_G1
169	DUOX2	6.685013602	Tumor_G1
170	DUOXA2	5.291646675	Tumor_G1
171	NDUFA1	7.190236517	Tumor_G1
172	NDUFA2	6.400169044	Tumor_G1
173	NDUFA8	6.99468819	Tumor_G1
174	NDUFB1	4.907330584	Tumor_G1
175	NDUFB10	7.483086653	Tumor_G1
176	NDUFB2	5.694420208	Tumor_G1
177	NDUFB3	5.490325476	Tumor_G1
178	NDUFB7	8.140366147	Tumor_G1
179	CYC1	6.873109736	Tumor_G1
180	ETFB	4.502087459	Tumor_G1
181	UQCR10	7.1742007	Tumor_G1
182	UQCRQ	6.761371029	Tumor_G1
183	UQCC3	4.001140981	Tumor_G1
184	COX15	4.828494251	Tumor_G1
185	COX5A	7.099574207	Tumor_G1
186	COX6B1	8.41608775	Tumor_G1
187	COX6C	5.572804246	Tumor_G1
188	COX7A2	6.545868264	Tumor_G1
189	COX7B	5.590791947	Tumor_G1
190	COX8A	9.163720518	Tumor_G1
191	SCO1	3.420494164	Tumor_G1
192	SURF1	5.844895297	Tumor_G1
193	ATP6V1E1	7.381482638	Tumor_G1
194	DLAT	5.535582149	Tumor_G1
195	PDC	0.293879882	Tumor_G1
196	SUCLG2	6.140797858	Tumor_G1
197	SLC25A5	8.308047264	Tumor_G1
198	UCP2	6.767444946	Tumor_G1

199	SOD2	6.045143562	Tumor_G1
200	NOX4	0.882567059	Tumor_G1
201	DUOX1	4.579629124	Tumor_G1
202	DUOXA1	5.341025073	Tumor_G1
203	DUOX2	4.053508083	Tumor_G1
204	DUOXA2	2.24220868	Tumor_G1
205	NDUFA1	7.400122294	Tumor_G1
206	NDUFA2	6.419334364	Tumor_G1
207	NDUFA8	6.587938727	Tumor_G1
208	NDUFB1	5.146268886	Tumor_G1
209	NDUFB10	7.14524372	Tumor_G1
210	NDUFB2	6.183566963	Tumor_G1
211	NDUFB3	5.80270173	Tumor_G1
212	NDUFB7	8.257626821	Tumor_G1
213	CYC1	6.609475625	Tumor_G1
214	ETFB	4.54737233	Tumor_G1
215	UQCR10	7.052401301	Tumor_G1
216	UQCRQ	6.790220575	Tumor_G1
217	UQCC3	4.09754765	Tumor_G1
218	COX15	4.742404324	Tumor_G1
219	COX5A	6.759539621	Tumor_G1
220	COX6B1	8.193539964	Tumor_G1
221	COX6C	5.96086095	Tumor_G1
222	COX7A2	6.410631878	Tumor_G1
223	COX7B	5.60481183	Tumor_G1
224	COX8A	9.185419722	Tumor_G1
225	SCO1	2.913863108	Tumor_G1
226	SURF1	6.131737063	Tumor_G1
227	ATP6V1E1	6.838169395	Tumor_G1
228	DLAT	4.942393446	Tumor_G1
229	PDC	0.146419071	Tumor_G1
230	SUCLG2	6.061996054	Tumor_G1
231	SLC25A5	7.950108705	Tumor_G1
232	UCP2	8.196949622	Tumor_G1
233	SOD2	4.446696013	Tumor_G1
234	NOX4	1.332982066	Tumor_G1
235	DUOX1	6.819125739	Tumor_G1
236	DUOXA1	6.330415085	Tumor_G1
237	DUOX2	6.939899724	Tumor_G1
238	DUOXA2	4.403329139	Tumor_G1

239	NDUFA1	7.886413508	Tumor_G1
240	NDUFA2	6.912049618	Tumor_G1
241	NDUFA8	7.02543975	Tumor_G1
242	NDUFB1	5.632284396	Tumor_G1
243	NDUFB10	7.514431202	Tumor_G1
244	NDUFB2	6.567939479	Tumor_G1
245	NDUFB3	5.856226054	Tumor_G1
246	NDUFB7	8.690610622	Tumor_G1
247	CYC1	6.762981738	Tumor_G1
248	ETFB	4.873200628	Tumor_G1
249	UQCR10	7.329832886	Tumor_G1
250	UQCRQ	7.32774817	Tumor_G1
251	UQCC3	4.333164804	Tumor_G1
252	COX15	4.400114698	Tumor_G1
253	COX5A	7.134508245	Tumor_G1
254	COX6B1	8.868899344	Tumor_G1
255	COX6C	6.516528287	Tumor_G1
256	COX7A2	6.728253716	Tumor_G1
257	COX7B	5.989158251	Tumor_G1
258	COX8A	9.26835094	Tumor_G1
259	SCO1	2.569430751	Tumor_G1
260	SURF1	5.908059915	Tumor_G1
261	ATP6V1E1	6.762323674	Tumor_G1
262	DLAT	4.688501738	Tumor_G1
263	PDC	0.117772893	Tumor_G1
264	SUCLG2	5.964194719	Tumor_G1
265	SLC25A5	7.685138715	Tumor_G1
266	UCP2	8.799554236	Tumor_G1
267	SOD2	4.529994453	Tumor_G1
268	NOX4	2.17789101	Tumor_G1
269	DUOX1	5.81738736	Tumor_G1
270	DUOXA1	6.016055663	Tumor_G1
271	DUOX2	6.539343505	Tumor_G1
272	DUOXA2	4.465518854	Tumor_G1
273	NDUFA1	7.670975957	Tumor_G1
274	NDUFA2	6.519379832	Tumor_G1
275	NDUFA8	6.910868641	Tumor_G1
276	NDUFB1	5.412473974	Tumor_G1
277	NDUFB10	7.522109094	Tumor_G1
278	NDUFB2	6.161458494	Tumor_G1

279	NDUFB3	5.883533649	Tumor_G1
280	NDUFB7	8.479040629	Tumor_G1
281	CYC1	6.529553765	Tumor_G1
282	ETFB	4.955210048	Tumor_G1
283	UQCR10	7.478886292	Tumor_G1
284	UQCRQ	7.157820881	Tumor_G1
285	UQCC3	4.286024338	Tumor_G1
286	COX15	4.611362101	Tumor_G1
287	COX5A	6.940884144	Tumor_G1
288	COX6B1	8.523853408	Tumor_G1
289	COX6C	6.307434598	Tumor_G1
290	COX7A2	6.645451861	Tumor_G1
291	COX7B	5.878614695	Tumor_G1
292	COX8A	9.143188503	Tumor_G1
293	SCO1	3.074772935	Tumor_G1
294	SURF1	6.254476827	Tumor_G1
295	ATP6V1E1	6.807385461	Tumor_G1
296	DLAT	4.676608525	Tumor_G1
297	PDC	0.081269309	Tumor_G1
298	SUCLG2	5.88953688	Tumor_G1
299	SLC25A5	7.891489604	Tumor_G1
300	UCP2	8.262363124	Tumor_G1
301	SOD2	4.996697414	Tumor_G1
302	NOX4	2.892953196	Tumor_G1
303	DUOX1	5.960436456	Tumor_G1
304	DUOXA1	6.377450783	Tumor_G1
305	DUOX2	6.378663554	Tumor_G1
306	DUOXA2	4.515654785	Tumor_G1
307	NDUFA1	7.691975432	Tumor_G1
308	NDUFA2	6.639135107	Tumor_G1
309	NDUFA8	6.945826638	Tumor_G1
310	NDUFB1	5.493533668	Tumor_G1
311	NDUFB10	7.557383977	Tumor_G1
312	NDUFB2	6.187796869	Tumor_G1
313	NDUFB3	6.071292217	Tumor_G1
314	NDUFB7	8.283140896	Tumor_G1
315	CYC1	6.670704783	Tumor_G1
316	ETFB	4.546556396	Tumor_G1
317	UQCR10	7.430563753	Tumor_G1
318	UQCRQ	6.882192074	Tumor_G1

319	UQCC3	3.964955315	Tumor_G1
320	COX15	4.731118465	Tumor_G1
321	COX5A	7.062441698	Tumor_G1
322	COX6B1	8.453083691	Tumor_G1
323	COX6C	6.18852254	Tumor_G1
324	COX7A2	6.58881744	Tumor_G1
325	COX7B	5.868960707	Tumor_G1
326	COX8A	9.331680603	Tumor_G1
327	SCO1	3.195184945	Tumor_G1
328	SURF1	5.931726343	Tumor_G1
329	ATP6V1E1	7.073270788	Tumor_G1
330	DLAT	5.181793403	Tumor_G1
331	PDC	0	Tumor_G1
332	SUCLG2	5.86000395	Tumor_G1
333	SLC25A5	8.084317592	Tumor_G1
334	UCP2	8.123542233	Tumor_G1
335	SOD2	5.619869079	Tumor_G1
336	NOX4	1.925875638	Tumor_G1
337	DUOX1	6.029668512	Tumor_G1
338	DUOXA1	6.254825815	Tumor_G1
339	DUOX2	5.942691315	Tumor_G1
340	DUOXA2	3.891223492	Tumor_G1
341	NDUFA1	8.060904678	Tumor_G1
342	NDUFA2	6.135548931	Tumor_G1
343	NDUFA8	7.012900958	Tumor_G1
344	NDUFB1	5.505747661	Tumor_G1
345	NDUFB10	7.14893891	Tumor_G1
346	NDUFB2	5.822030238	Tumor_G1
347	NDUFB3	6.418789642	Tumor_G1
348	NDUFB7	8.22999874	Tumor_G1
349	CYC1	6.548021645	Tumor_G1
350	ETFB	4.385047502	Tumor_G1
351	UQCR10	7.519346233	Tumor_G1
352	UQCRQ	6.655119956	Tumor_G1
353	UQCC3	3.570424769	Tumor_G1
354	COX15	5.091649546	Tumor_G1
355	COX5A	6.595833941	Tumor_G1
356	COX6B1	8.580362431	Tumor_G1
357	COX6C	5.897893979	Tumor_G1
358	COX7A2	6.541222121	Tumor_G1

359	COX7B	6.328756945	Tumor_G1
360	COX8A	9.173874245	Tumor_G1
361	SCO1	3.712471028	Tumor_G1
362	SURF1	6.150786736	Tumor_G1
363	ATP6V1E1	6.462788627	Tumor_G1
364	DLAT	4.841030494	Tumor_G1
365	PDC	0.106629552	Tumor_G1
366	SUCLG2	5.868892896	Tumor_G1
367	SLC25A5	8.225366268	Tumor_G1
368	UCP2	8.067048772	Tumor_G1
369	SOD2	5.833351318	Tumor_G1
370	NOX4	1.627297357	Tumor_G1
371	DUOX1	5.600909789	Tumor_G1
372	DUOXA1	5.690790141	Tumor_G1
373	DUOX2	5.657858085	Tumor_G1
374	DUOXA2	4.84346096	Tumor_G1
375	NDUFA1	7.56131842	Tumor_G1
376	NDUFA2	6.340863896	Tumor_G1
377	NDUFA8	6.787381796	Tumor_G1
378	NDUFB1	5.243556905	Tumor_G1
379	NDUFB10	6.98123149	Tumor_G1
380	NDUFB2	6.115547796	Tumor_G1
381	NDUFB3	5.994463607	Tumor_G1
382	NDUFB7	7.818199182	Tumor_G1
383	CYC1	6.43526567	Tumor_G1
384	ETFB	4.393070274	Tumor_G1
385	UQCR10	7.144283434	Tumor_G1
386	UQCRQ	6.715292106	Tumor_G1
387	UQCC3	3.702912842	Tumor_G1
388	COX15	4.73193958	Tumor_G1
389	COX5A	6.659922551	Tumor_G1
390	COX6B1	8.244618263	Tumor_G1
391	COX6C	5.943720932	Tumor_G1
392	COX7A2	6.551891722	Tumor_G1
393	COX7B	5.722816443	Tumor_G1
394	COX8A	8.298706337	Tumor_G1
395	SCO1	3.063627804	Tumor_G1
396	SURF1	5.949344267	Tumor_G1
397	ATP6V1E1	6.858590198	Tumor_G1
398	DLAT	4.86001937	Tumor_G1

399	PDC	0.049171915	Tumor_G1
400	SUCLG2	5.538469181	Tumor_G1
401	SLC25A5	8.119895502	Tumor_G1
402	UCP2	8.041958534	Tumor_G1
403	SOD2	5.72790981	Tumor_G1
404	NOX4	2.925059847	Tumor_G1
405	DUOX1	5.517164886	Tumor_G1
406	DUOXA1	5.81012927	Tumor_G1
407	DUOX2	5.20929963	Tumor_G1
408	DUOXA2	2.836703204	Tumor_G1
409	NDUFA1	7.553500728	Tumor_G1
410	NDUFA2	6.123070202	Tumor_G1
411	NDUFA8	6.669046652	Tumor_G1
412	NDUFB1	5.159912639	Tumor_G1
413	NDUFB10	7.031839849	Tumor_G1
414	NDUFB2	5.719130787	Tumor_G1
415	NDUFB3	5.851102939	Tumor_G1
416	NDUFB7	8.233225989	Tumor_G1
417	CYC1	6.436823756	Tumor_G1
418	ETFB	4.760591391	Tumor_G1
419	UQCR10	7.208500075	Tumor_G1
420	UQCRQ	6.919567328	Tumor_G1
421	UQCC3	3.834547245	Tumor_G1
422	COX15	4.351551776	Tumor_G1
423	COX5A	6.530556042	Tumor_G1
424	COX6B1	8.371168761	Tumor_G1
425	COX6C	5.815917716	Tumor_G1
426	COX7A2	6.313385197	Tumor_G1
427	COX7B	5.736873431	Tumor_G1
428	COX8A	8.593618239	Tumor_G1
429	SCO1	2.89542353	Tumor_G1
430	SURF1	5.952940534	Tumor_G1
431	ATP6V1E1	6.297310951	Tumor_G1
432	DLAT	4.333968749	Tumor_G1
433	PDC	0	Tumor_G1
434	SUCLG2	5.628962802	Tumor_G1
435	SLC25A5	7.770692156	Tumor_G1
436	UCP2	7.570451299	Tumor_G1
437	SOD2	5.26417118	Tumor_G1
438	NOX4	2.153850368	Tumor_G1

439	DUOX1	6.045315807	Tumor_G1
440	DUOXA1	6.049354131	Tumor_G1
441	DUOX2	5.957600571	Tumor_G1
442	DUOXA2	3.731884966	Tumor_G1
443	NDUFA1	7.694114085	Tumor_G1
444	NDUFA2	6.633895387	Tumor_G1
445	NDUFA8	7.049552291	Tumor_G1
446	NDUFB1	5.302264897	Tumor_G1
447	NDUFB10	7.475403229	Tumor_G1
448	NDUFB2	6.681317233	Tumor_G1
449	NDUFB3	6.083018618	Tumor_G1
450	NDUFB7	8.156261555	Tumor_G1
451	CYC1	6.762504777	Tumor_G1
452	ETFB	4.970871841	Tumor_G1
453	UQCR10	7.503059802	Tumor_G1
454	UQCRQ	7.131994153	Tumor_G1
455	UQCC3	4.188305104	Tumor_G1
456	COX15	4.719691144	Tumor_G1
457	COX5A	7.022056182	Tumor_G1
458	COX6B1	8.574928563	Tumor_G1
459	COX6C	6.357976187	Tumor_G1
460	COX7A2	6.621230893	Tumor_G1
461	COX7B	6.008341989	Tumor_G1
462	COX8A	9.423804609	Tumor_G1
463	SCO1	3.09143725	Tumor_G1
464	SURF1	6.354671717	Tumor_G1
465	ATP6V1E1	7.034613608	Tumor_G1
466	DLAT	5.012375155	Tumor_G1
467	PDC	0.140804418	Tumor_G1
468	SUCLG2	6.137949704	Tumor_G1
469	SLC25A5	8.005627349	Tumor_G1
470	UCP2	8.521496687	Tumor_G1
471	SOD2	5.111427443	Tumor_G1
472	NOX4	2.406282913	Tumor_G1
473	DUOX1	5.984190487	Tumor_G1
474	DUOXA1	6.416093506	Tumor_G1
475	DUOX2	6.638716221	Tumor_G1
476	DUOXA2	4.690077648	Tumor_G1
477	NDUFA1	7.895763147	Tumor_G1
478	NDUFA2	6.633712628	Tumor_G1

479	NDUFA8	6.859613696	Tumor_G1
480	NDUFB1	5.592163034	Tumor_G1
481	NDUFB10	7.298669621	Tumor_G1
482	NDUFB2	6.408526225	Tumor_G1
483	NDUFB3	6.232650902	Tumor_G1
484	NDUFB7	8.289143614	Tumor_G1
485	CYC1	6.514990824	Tumor_G1
486	ETFB	4.350487861	Tumor_G1
487	UQCR10	7.527485598	Tumor_G1
488	UQCRQ	7.188745246	Tumor_G1
489	UQCC3	3.986923182	Tumor_G1
490	COX15	4.463350284	Tumor_G1
491	COX5A	7.198217612	Tumor_G1
492	COX6B1	8.487673014	Tumor_G1
493	COX6C	6.442010415	Tumor_G1
494	COX7A2	6.744800887	Tumor_G1
495	COX7B	5.923648541	Tumor_G1
496	COX8A	8.808779556	Tumor_G1
497	SCO1	3.016280766	Tumor_G1
498	SURF1	5.746283274	Tumor_G1
499	ATP6V1E1	6.893942831	Tumor_G1
500	DLAT	5.04428074	Tumor_G1
501	PDC	0	Tumor_G1
502	SUCLG2	6.327284392	Tumor_G1
503	SLC25A5	8.085923652	Tumor_G1
504	UCP2	7.640337134	Tumor_G1
505	SOD2	5.669875215	Tumor_G1
506	NOX4	1.772651172	Tumor_G1
507	DUOX1	5.222270716	Tumor_G1
508	DUOXA1	5.491223644	Tumor_G1
509	DUOX2	4.472254923	Tumor_G1
510	DUOXA2	2.690962833	Tumor_G1
511	NDUFA1	8.097363822	Tumor_G1
512	NDUFA2	7.046133122	Tumor_G1
513	NDUFA8	6.967672096	Tumor_G1
514	NDUFB1	5.784584434	Tumor_G1
515	NDUFB10	7.419902792	Tumor_G1
516	NDUFB2	6.702822213	Tumor_G1
517	NDUFB3	6.343706889	Tumor_G1
518	NDUFB7	8.686509163	Tumor_G1

519	CYC1	7.152009264	Tumor_G1
520	ETFB	4.657587942	Tumor_G1
521	UQCR10	7.790160133	Tumor_G1
522	UQCRQ	7.304081059	Tumor_G1
523	UQCC3	4.289612925	Tumor_G1
524	COX15	4.424197558	Tumor_G1
525	COX5A	7.3030281	Tumor_G1
526	COX6B1	8.801745603	Tumor_G1
527	COX6C	6.312744819	Tumor_G1
528	COX7A2	7.008387866	Tumor_G1
529	COX7B	6.018699605	Tumor_G1
530	COX8A	9.295873531	Tumor_G1
531	SCO1	3.131928995	Tumor_G1
532	SURF1	6.182817238	Tumor_G1
533	ATP6V1E1	6.877985248	Tumor_G1
534	DLAT	4.760581561	Tumor_G1
535	PDC	0.050072893	Tumor_G1
536	SUCLG2	6.039629737	Tumor_G1
537	SLC25A5	8.664402544	Tumor_G1
538	UCP2	8.642819063	Tumor_G1
539	SOD2	5.604026857	Tumor_G1
540	NOX4	1.461107855	Tumor_G1
541	DUOX1	4.694239749	Tumor_G1
542	DUOXA1	4.953277731	Tumor_G1
543	DUOX2	3.748501476	Tumor_G1
544	DUOXA2	2.938426458	Tumor_G1
545	NDUFA1	7.706640214	Tumor_G1
546	NDUFA2	6.0211191	Tumor_G1
547	NDUFA8	6.252770286	Tumor_G1
548	NDUFB1	4.879502021	Tumor_G1
549	NDUFB10	6.880793445	Tumor_G1
550	NDUFB2	6.176888894	Tumor_G1
551	NDUFB3	5.660024744	Tumor_G1
552	NDUFB7	7.708107641	Tumor_G1
553	CYC1	6.526648119	Tumor_G1
554	ETFB	3.705003994	Tumor_G1
555	UQCR10	7.353308268	Tumor_G1
556	UQCRQ	6.559201348	Tumor_G1
557	UQCC3	3.548766073	Tumor_G1
558	COX15	3.961311137	Tumor_G1

559	COX5A	6.859345278	Tumor_G1
560	COX6B1	8.009169346	Tumor_G1
561	COX6C	5.907319801	Tumor_G1
562	COX7A2	6.106337159	Tumor_G1
563	COX7B	5.666893479	Tumor_G1
564	COX8A	8.775836719	Tumor_G1
565	SCO1	2.335343821	Tumor_G1
566	SURF1	5.551593593	Tumor_G1
567	ATP6V1E1	5.950855457	Tumor_G1
568	DLAT	3.851257942	Tumor_G1
569	PDC	0.087235612	Tumor_G1
570	SUCLG2	4.942628051	Tumor_G1
571	SLC25A5	7.725237263	Tumor_G1
572	UCP2	6.476174958	Tumor_G1
573	SOD2	6.015300469	Tumor_G1
574	NOX4	0.446600309	Tumor_G1
575	DUOX1	3.418431884	Tumor_G1
576	DUOXA1	3.183669369	Tumor_G1
577	DUOX2	3.411629015	Tumor_G1
578	DUOXA2	2.153055298	Tumor_G1
579	NDUFA1	7.727797501	Tumor_G1
580	NDUFA2	6.867667601	Tumor_G1
581	NDUFA8	6.805497587	Tumor_G1
582	NDUFB1	5.471855461	Tumor_G1
583	NDUFB10	7.243562203	Tumor_G1
584	NDUFB2	6.250953027	Tumor_G1
585	NDUFB3	6.008230899	Tumor_G1
586	NDUFB7	8.352352108	Tumor_G1
587	CYC1	6.665242199	Tumor_G1
588	ETFB	4.663385159	Tumor_G1
589	UQCR10	7.48776948	Tumor_G1
590	UQCRQ	7.16931387	Tumor_G1
591	UQCC3	4.038505405	Tumor_G1
592	COX15	4.450660038	Tumor_G1
593	COX5A	7.011364815	Tumor_G1
594	COX6B1	8.660061606	Tumor_G1
595	COX6C	6.364711148	Tumor_G1
596	COX7A2	6.814964917	Tumor_G1
597	COX7B	5.822128751	Tumor_G1
598	COX8A	9.062136675	Tumor_G1

599	SCO1	2.952718627	Tumor_G1
600	SURF1	6.243201304	Tumor_G1
601	ATP6V1E1	7.113331084	Tumor_G1
602	DLAT	4.777428983	Tumor_G1
603	PDC	0.148830023	Tumor_G1
604	SUCLG2	5.992705936	Tumor_G1
605	SLC25A5	7.852767545	Tumor_G1
606	UCP2	8.129906536	Tumor_G1
607	SOD2	4.965971178	Tumor_G1
608	NOX4	2.719290562	Tumor_G1
609	DUOX1	5.283178839	Tumor_G1
610	DUOXA1	5.517721617	Tumor_G1
611	DUOX2	4.591690866	Tumor_G1
612	DUOXA2	2.952670888	Tumor_G1
613	NDUFA1	7.548856419	Tumor_G1
614	NDUFA2	6.420657818	Tumor_G1
615	NDUFA8	6.916579044	Tumor_G1
616	NDUFB1	5.424540471	Tumor_G1
617	NDUFB10	7.341839127	Tumor_G1
618	NDUFB2	5.794364499	Tumor_G1
619	NDUFB3	5.933461604	Tumor_G1
620	NDUFB7	8.388608729	Tumor_G1
621	CYC1	6.477835723	Tumor_G1
622	ETFB	4.618462069	Tumor_G1
623	UQCR10	7.250927155	Tumor_G1
624	UQCRQ	7.102770757	Tumor_G1
625	UQCC3	3.997982572	Tumor_G1
626	COX15	4.542472624	Tumor_G1
627	COX5A	6.92292721	Tumor_G1
628	COX6B1	8.522927846	Tumor_G1
629	COX6C	6.119362284	Tumor_G1
630	COX7A2	6.522522569	Tumor_G1
631	COX7B	5.718534193	Tumor_G1
632	COX8A	8.998257924	Tumor_G1
633	SCO1	3.049812254	Tumor_G1
634	SURF1	6.039212833	Tumor_G1
635	ATP6V1E1	6.824017585	Tumor_G1
636	DLAT	4.651553607	Tumor_G1
637	PDC	0.366102496	Tumor_G1
638	SUCLG2	5.685980247	Tumor_G1

639	SLC25A5	7.947934547	Tumor_G1
640	UCP2	8.569366906	Tumor_G1
641	SOD2	4.630019792	Tumor_G1
642	NOX4	3.495556625	Tumor_G1
643	DUOX1	5.867145103	Tumor_G1
644	DUOXA1	6.391727142	Tumor_G1
645	DUOX2	5.8797055	Tumor_G1
646	DUOXA2	4.03789235	Tumor_G1
647	NDUFA1	7.391070428	Tumor_G1
648	NDUFA2	6.181976648	Tumor_G1
649	NDUFA8	6.587202708	Tumor_G1
650	NDUFB1	5.015161372	Tumor_G1
651	NDUFB10	6.986317694	Tumor_G1
652	NDUFB2	5.923798773	Tumor_G1
653	NDUFB3	5.865981761	Tumor_G1
654	NDUFB7	8.08094457	Tumor_G1
655	CYC1	6.495465065	Tumor_G1
656	ETFB	4.471710897	Tumor_G1
657	UQCR10	6.89737633	Tumor_G1
658	UQCRQ	6.619361879	Tumor_G1
659	UQCC3	3.465531937	Tumor_G1
660	COX15	5.03935514	Tumor_G1
661	COX5A	6.71821402	Tumor_G1
662	COX6B1	8.189747789	Tumor_G1
663	COX6C	5.800791566	Tumor_G1
664	COX7A2	6.364869553	Tumor_G1
665	COX7B	5.710754975	Tumor_G1
666	COX8A	8.964328942	Tumor_G1
667	SCO1	3.26911881	Tumor_G1
668	SURF1	5.911376154	Tumor_G1
669	ATP6V1E1	7.179804338	Tumor_G1
670	DLAT	5.572982008	Tumor_G1
671	PDC	0.225203806	Tumor_G1
672	SUCLG2	6.318806739	Tumor_G1
673	SLC25A5	8.556996726	Tumor_G1
674	UCP2	8.239689346	Tumor_G1
675	SOD2	5.819467302	Tumor_G1
676	NOX4	2.624654976	Tumor_G1
677	DUOX1	6.031965443	Tumor_G1
678	DUOXA1	5.946904846	Tumor_G1

679	DUOX2	5.896283744	Tumor_G1
680	DUOXA2	3.023268809	Tumor_G1
681	NDUFA1	7.369229069	Tumor_G1
682	NDUFA2	6.275948755	Tumor_G1
683	NDUFA8	6.480718976	Tumor_G1
684	NDUFB1	4.779630323	Tumor_G1
685	NDUFB10	7.140351836	Tumor_G1
686	NDUFB2	6.121706319	Tumor_G1
687	NDUFB3	5.6390733	Tumor_G1
688	NDUFB7	8.088739843	Tumor_G1
689	CYC1	6.728345901	Tumor_G1
690	ETFB	4.395230243	Tumor_G1
691	UQCR10	6.943461701	Tumor_G1
692	UQCRQ	6.371846732	Tumor_G1
693	UQCC3	3.93616436	Tumor_G1
694	COX15	4.96246818	Tumor_G1
695	COX5A	6.734415466	Tumor_G1
696	COX6B1	7.979897221	Tumor_G1
697	COX6C	5.839856562	Tumor_G1
698	COX7A2	6.267986928	Tumor_G1
699	COX7B	5.380541238	Tumor_G1
700	COX8A	9.05365279	Tumor_G1
701	SCO1	3.297323092	Tumor_G1
702	SURF1	6.035609075	Tumor_G1
703	ATP6V1E1	7.12125291	Tumor_G1
704	DLAT	5.522388485	Tumor_G1
705	PDC	0.061742218	Tumor_G1
706	SUCLG2	6.447393898	Tumor_G1
707	SLC25A5	8.475092722	Tumor_G1
708	UCP2	7.467218199	Tumor_G1
709	SOD2	5.804777648	Tumor_G1
710	NOX4	2.753480987	Tumor_G1
711	DUOX1	6.271117051	Tumor_G1
712	DUOXA1	6.022550452	Tumor_G1
713	DUOX2	5.846243603	Tumor_G1
714	DUOXA2	3.303291873	Tumor_G1
715	NDUFA1	7.471605335	Tumor_G1
716	NDUFA2	6.244583405	Tumor_G1
717	NDUFA8	6.475544696	Tumor_G1
718	NDUFB1	5.11552649	Tumor_G1

719	NDUFB10	7.134191436	Tumor_G1
720	NDUFB2	5.936792016	Tumor_G1
721	NDUFB3	5.850222128	Tumor_G1
722	NDUFB7	7.972339119	Tumor_G1
723	CYC1	6.457623574	Tumor_G1
724	ETFB	4.459627513	Tumor_G1
725	UQCR10	6.668817352	Tumor_G1
726	UQCRQ	6.772281338	Tumor_G1
727	UQCC3	3.858822711	Tumor_G1
728	COX15	4.418882167	Tumor_G1
729	COX5A	6.718165295	Tumor_G1
730	COX6B1	7.96015005	Tumor_G1
731	COX6C	5.90577943	Tumor_G1
732	COX7A2	6.355066194	Tumor_G1
733	COX7B	5.500459787	Tumor_G1
734	COX8A	9.058284427	Tumor_G1
735	SCO1	2.791548221	Tumor_G1
736	SURF1	6.04045129	Tumor_G1
737	ATP6V1E1	5.90815555	Tumor_G1
738	DLAT	4.519105297	Tumor_G1
739	PDC	0	Tumor_G1
740	SUCLG2	5.765925525	Tumor_G1
741	SLC25A5	7.662193137	Tumor_G1
742	UCP2	7.748632227	Tumor_G1
743	SOD2	6.068117252	Tumor_G1
744	NOX4	1.579266586	Tumor_G1
745	DUOX1	6.695823589	Tumor_G1
746	DUOXA1	6.654543079	Tumor_G1
747	DUOX2	7.807722785	Tumor_G1
748	DUOXA2	5.113873449	Tumor_G1
749	NDUFA1	7.198784251	Tumor_G1
750	NDUFA2	6.471471065	Tumor_G1
751	NDUFA8	5.972160281	Tumor_G1
752	NDUFB1	5.469654754	Tumor_G1
753	NDUFB10	7.485809861	Tumor_G1
754	NDUFB2	6.345405535	Tumor_G1
755	NDUFB3	5.969230159	Tumor_G1
756	NDUFB7	8.269225699	Tumor_G1
757	CYC1	6.667273228	Tumor_G1
758	ETFB	4.380437971	Tumor_G1

759	UQCR10	7.027163117	Tumor_G1
760	UQCRQ	7.025082694	Tumor_G1
761	UQCC3	3.892577441	Tumor_G1
762	COX15	4.377336311	Tumor_G1
763	COX5A	6.897695398	Tumor_G1
764	COX6B1	8.276096925	Tumor_G1
765	COX6C	5.737997899	Tumor_G1
766	COX7A2	6.262806481	Tumor_G1
767	COX7B	5.721053982	Tumor_G1
768	COX8A	9.209760268	Tumor_G1
769	SCO1	2.824052556	Tumor_G1
770	SURF1	5.618315701	Tumor_G1
771	ATP6V1E1	7.015220173	Tumor_G1
772	DLAT	4.934912978	Tumor_G1
773	PDC	0.267042024	Tumor_G1
774	SUCLG2	6.271686107	Tumor_G1
775	SLC25A5	8.038996848	Tumor_G1
776	UCP2	7.982506771	Tumor_G1
777	SOD2	6.095633517	Tumor_G1
778	NOX4	2.098547293	Tumor_G1
779	DUOX1	6.818948273	Tumor_G1
780	DUOXA1	6.263476888	Tumor_G1
781	DUOX2	6.724920441	Tumor_G1
782	DUOXA2	3.958992043	Tumor_G1
783	NDUFA1	7.225130381	Tumor_G1
784	NDUFA2	6.376647039	Tumor_G1
785	NDUFA8	6.426476801	Tumor_G1
786	NDUFB1	5.358111084	Tumor_G1
787	NDUFB10	7.030540212	Tumor_G1
788	NDUFB2	6.174999365	Tumor_G1
789	NDUFB3	5.908099903	Tumor_G1
790	NDUFB7	7.973037101	Tumor_G1
791	CYC1	6.393684152	Tumor_G1
792	ETFB	4.568338031	Tumor_G1
793	UQCR10	7.075691444	Tumor_G1
794	UQCRQ	6.972700017	Tumor_G1
795	UQCC3	3.780222228	Tumor_G1
796	COX15	4.46849469	Tumor_G1
797	COX5A	6.676683048	Tumor_G1
798	COX6B1	8.165432262	Tumor_G1

799	COX6C	5.924630589	Tumor_G1
800	COX7A2	6.516923335	Tumor_G1
801	COX7B	5.602119802	Tumor_G1
802	COX8A	8.863429582	Tumor_G1
803	SCO1	2.65845532	Tumor_G1
804	SURF1	5.728433321	Tumor_G1
805	ATP6V1E1	7.020184141	Tumor_G1
806	DLAT	4.950926097	Tumor_G1
807	PDC	0.239798426	Tumor_G1
808	SUCLG2	6.298156179	Tumor_G1
809	SLC25A5	7.817198508	Tumor_G1
810	UCP2	7.937214789	Tumor_G1
811	SOD2	5.55257484	Tumor_G1
812	NOX4	1.987575637	Tumor_G1
813	DUOX1	6.384834449	Tumor_G1
814	DUOXA1	6.404889565	Tumor_G1
815	DUOX2	6.388493057	Tumor_G1
816	DUOXA2	4.259639932	Tumor_G1
817	NDUFA1	7.335582876	Tumor_G1
818	NDUFA2	6.429450358	Tumor_G1
819	NDUFA8	6.403000935	Tumor_G1
820	NDUFB1	4.996011629	Tumor_G1
821	NDUFB10	7.4739702	Tumor_G1
822	NDUFB2	5.837906729	Tumor_G1
823	NDUFB3	5.579978152	Tumor_G1
824	NDUFB7	8.032083031	Tumor_G1
825	CYC1	7.046791584	Tumor_G1
826	ETFB	4.653933011	Tumor_G1
827	UQCR10	6.786889492	Tumor_G1
828	UQCRQ	6.628235748	Tumor_G1
829	UQCC3	3.369558542	Tumor_G1
830	COX15	5.253976591	Tumor_G1
831	COX5A	6.805074243	Tumor_G1
832	COX6B1	8.287214844	Tumor_G1
833	COX6C	5.666102025	Tumor_G1
834	COX7A2	6.388645208	Tumor_G1
835	COX7B	5.372893785	Tumor_G1
836	COX8A	7.917189923	Tumor_G1
837	SCO1	3.054613753	Tumor_G1
838	SURF1	5.83264132	Tumor_G1

839	ATP6V1E1	7.185535419	Tumor_G1
840	DLAT	4.418841129	Tumor_G1
841	PDC	0.147782531	Tumor_G1
842	SUCLG2	6.676509278	Tumor_G1
843	SLC25A5	8.106498981	Tumor_G1
844	UCP2	5.914409469	Tumor_G1
845	SOD2	4.439486474	Tumor_G1
846	NOX4	0.620153027	Tumor_G1
847	DUOX1	5.431582642	Tumor_G1
848	DUOXA1	5.400359439	Tumor_G1
849	DUOX2	4.494159388	Tumor_G1
850	DUOXA2	2.504594127	Tumor_G1
851	NDUFA1	7.679005786	Tumor_G1
852	NDUFA2	6.437277898	Tumor_G1
853	NDUFA8	6.70305265	Tumor_G1
854	NDUFB1	5.319329287	Tumor_G1
855	NDUFB10	7.191915464	Tumor_G1
856	NDUFB2	6.072308645	Tumor_G1
857	NDUFB3	6.05463813	Tumor_G1
858	NDUFB7	8.009680176	Tumor_G1
859	CYC1	6.39429489	Tumor_G1
860	ETFB	4.358845814	Tumor_G1
861	UQCR10	7.332100153	Tumor_G1
862	UQCRQ	6.933408871	Tumor_G1
863	UQCC3	3.644079517	Tumor_G1
864	COX15	4.578404684	Tumor_G1
865	COX5A	7.011306154	Tumor_G1
866	COX6B1	8.225195249	Tumor_G1
867	COX6C	5.939103669	Tumor_G1
868	COX7A2	6.544809418	Tumor_G1
869	COX7B	5.801695566	Tumor_G1
870	COX8A	9.040886508	Tumor_G1
871	SCO1	3.177536235	Tumor_G1
872	SURF1	5.754023896	Tumor_G1
873	ATP6V1E1	7.062111595	Tumor_G1
874	DLAT	5.173377688	Tumor_G1
875	PDC	0.169720471	Tumor_G1
876	SUCLG2	5.990747872	Tumor_G1
877	SLC25A5	8.260078099	Tumor_G1
878	UCP2	7.721788446	Tumor_G1

879	SOD2	6.577810186	Tumor_G1
880	NOX4	3.037708015	Tumor_G1
881	DUOX1	5.350893983	Tumor_G1
882	DUOXA1	5.433156745	Tumor_G1
883	DUOX2	5.012810172	Tumor_G1
884	DUOXA2	2.988737311	Tumor_G1
885	NDUFA1	8.074238462	Tumor_G1
886	NDUFA2	6.986078545	Tumor_G1
887	NDUFA8	6.210472593	Tumor_G1
888	NDUFB1	5.730450707	Tumor_G1
889	NDUFB10	7.330904704	Tumor_G1
890	NDUFB2	6.460882528	Tumor_G1
891	NDUFB3	6.356516252	Tumor_G1
892	NDUFB7	8.782327153	Tumor_G1
893	CYC1	6.729868529	Tumor_G1
894	ETFB	4.45938183	Tumor_G1
895	UQCR10	7.734510776	Tumor_G1
896	UQCRQ	7.355271206	Tumor_G1
897	UQCC3	4.190692002	Tumor_G1
898	COX15	4.070730011	Tumor_G1
899	COX5A	6.911795831	Tumor_G1
900	COX6B1	8.765335906	Tumor_G1
901	COX6C	6.405140172	Tumor_G1
902	COX7A2	6.714547931	Tumor_G1
903	COX7B	6.042906094	Tumor_G1
904	COX8A	9.185605346	Tumor_G1
905	SCO1	2.871112013	Tumor_G1
906	SURF1	5.146837285	Tumor_G1
907	ATP6V1E1	6.961977424	Tumor_G1
908	DLAT	4.749967858	Tumor_G1
909	PDC	0.085961942	Tumor_G1
910	SUCLG2	6.084998423	Tumor_G1
911	SLC25A5	7.903301868	Tumor_G1
912	UCP2	8.312701108	Tumor_G1
913	SOD2	5.161949635	Tumor_G1
914	NOX4	2.356101316	Tumor_G1
915	DUOX1	4.889380934	Tumor_G1
916	DUOXA1	5.179447028	Tumor_G1
917	DUOX2	4.521090757	Tumor_G1
918	DUOXA2	2.976756025	Tumor_G1

919	NDUFA1	8.147964061	Tumor_G1
920	NDUFA2	6.473712109	Tumor_G1
921	NDUFA8	6.383997209	Tumor_G1
922	NDUFB1	5.694673122	Tumor_G1
923	NDUFB10	6.653912467	Tumor_G1
924	NDUFB2	5.540993437	Tumor_G1
925	NDUFB3	4.696533109	Tumor_G1
926	NDUFB7	8.355660247	Tumor_G1
927	CYC1	5.577668599	Tumor_G1
928	ETFB	3.434462317	Tumor_G1
929	UQCR10	7.01757318	Tumor_G1
930	UQCRQ	6.56485993	Tumor_G1
931	UQCC3	3.275449986	Tumor_G1
932	COX15	3.078196919	Tumor_G1
933	COX5A	6.179400887	Tumor_G1
934	COX6B1	8.356194661	Tumor_G1
935	COX6C	6.305580391	Tumor_G1
936	COX7A2	5.731374857	Tumor_G1
937	COX7B	5.510347391	Tumor_G1
938	COX8A	8.232212494	Tumor_G1
939	SCO1	1.516955885	Tumor_G1
940	SURF1	4.892494301	Tumor_G1
941	ATP6V1E1	5.50632703	Tumor_G1
942	DLAT	2.714960844	Tumor_G1
943	PDC	0	Tumor_G1
944	SUCLG2	4.62570321	Tumor_G1
945	SLC25A5	6.001481647	Tumor_G1
946	UCP2	7.107369699	Tumor_G1
947	SOD2	4.734368343	Tumor_G1
948	NOX4	0.220494564	Tumor_G1
949	DUOX1	3.533868156	Tumor_G1
950	DUOXA1	3.252307244	Tumor_G1
951	DUOX2	3.514461628	Tumor_G1
952	DUOXA2	2.355522216	Tumor_G1
953	NDUFA1	7.776318288	Tumor_G1
954	NDUFA2	6.345310898	Tumor_G1
955	NDUFA8	6.7982902	Tumor_G1
956	NDUFB1	5.448917762	Tumor_G1
957	NDUFB10	7.560093017	Tumor_G1
958	NDUFB2	6.602791678	Tumor_G1

959	NDUFB3	5.884133249	Tumor_G1
960	NDUFB7	8.376187211	Tumor_G1
961	CYC1	7.299382345	Tumor_G1
962	ETFB	4.416374936	Tumor_G1
963	UQCR10	7.244765649	Tumor_G1
964	UQCRQ	6.732321737	Tumor_G1
965	UQCC3	4.256208016	Tumor_G1
966	COX15	5.26227034	Tumor_G1
967	COX5A	7.217917063	Tumor_G1
968	COX6B1	8.462951221	Tumor_G1
969	COX6C	6.41170288	Tumor_G1
970	COX7A2	6.68812277	Tumor_G1
971	COX7B	6.124442033	Tumor_G1
972	COX8A	9.594256157	Tumor_G1
973	SCO1	3.376814575	Tumor_G1
974	SURF1	6.256518774	Tumor_G1
975	ATP6V1E1	7.034384375	Tumor_G1
976	DLAT	5.378356433	Tumor_G1
977	PDC	0.200519417	Tumor_G1
978	SUCLG2	6.585212699	Tumor_G1
979	SLC25A5	9.048493889	Tumor_G1
980	UCP2	6.806094565	Tumor_G1
981	SOD2	5.22298234	Tumor_G1
982	NOX4	1.171715364	Tumor_G1
983	DUOX1	6.346286335	Tumor_G1
984	DUOXA1	6.180158551	Tumor_G1
985	DUOX2	6.362443569	Tumor_G1
986	DUOXA2	3.829008081	Tumor_G1
987	NDUFA1	6.420491762	Tumor_G1
988	NDUFA2	5.17233424	Tumor_G1
989	NDUFA8	5.090362096	Tumor_G1
990	NDUFB1	4.529589421	Tumor_G1
991	NDUFB10	5.858833238	Tumor_G1
992	NDUFB2	4.210197964	Tumor_G1
993	NDUFB3	3.209554968	Tumor_G1
994	NDUFB7	7.814892599	Tumor_G1
995	CYC1	3.773753609	Tumor_G1
996	ETFB	3.467246384	Tumor_G1
997	UQCR10	6.399689286	Tumor_G1
998	UQCRQ	4.98476984	Tumor_G1

999	UQCC3	3.583679786	Tumor_G1
1000	COX15	2.518597015	Tumor_G1
1001	COX5A	3.762525407	Tumor_G1
1002	COX6B1	8.369103054	Tumor_G1
1003	COX6C	4.894209989	Tumor_G1
1004	COX7A2	3.966719364	Tumor_G1
1005	COX7B	3.926206508	Tumor_G1
1006	COX8A	7.218360478	Tumor_G1
1007	SCO1	1.4076836	Tumor_G1
1008	SURF1	4.281438883	Tumor_G1
1009	ATP6V1E1	3.546164213	Tumor_G1
1010	DLAT	1.703317139	Tumor_G1
1011	PDC	0	Tumor_G1
1012	SUCLG2	2.483092526	Tumor_G1
1013	SLC25A5	4.426562827	Tumor_G1
1014	UCP2	6.232826917	Tumor_G1
1015	SOD2	2.011204622	Tumor_G1
1016	NOX4	0.425757633	Tumor_G1
1017	DUOX1	6.276327915	Tumor_G1
1018	DUOXA1	5.121629463	Tumor_G1
1019	DUOX2	5.891422317	Tumor_G1
1020	DUOXA2	5.665384897	Tumor_G1
1021	NDUFA1	7.585782513	Tumor_G1
1022	NDUFA2	6.2451617	Tumor_G1
1023	NDUFA8	6.724500147	Tumor_G1
1024	NDUFB1	5.131011544	Tumor_G1
1025	NDUFB10	7.149214404	Tumor_G1
1026	NDUFB2	6.196683968	Tumor_G1
1027	NDUFB3	5.987643061	Tumor_G1
1028	NDUFB7	8.240378829	Tumor_G1
1029	CYC1	6.606213229	Tumor_G1
1030	ETFB	4.517057103	Tumor_G1
1031	UQCR10	7.175281304	Tumor_G1
1032	UQCRQ	6.883071459	Tumor_G1
1033	UQCC3	3.728071575	Tumor_G1
1034	COX15	4.833466444	Tumor_G1
1035	COX5A	6.940397298	Tumor_G1
1036	COX6B1	8.298261692	Tumor_G1
1037	COX6C	6.18026569	Tumor_G1
1038	COX7A2	6.567631918	Tumor_G1

1039	COX7B	5.759780503	Tumor_G1
1040	COX8A	9.082451941	Tumor_G1
1041	SCO1	3.402626446	Tumor_G1
1042	SURF1	6.126598537	Tumor_G1
1043	ATP6V1E1	7.133624912	Tumor_G1
1044	DLAT	5.25712485	Tumor_G1
1045	PDC	0.041220521	Tumor_G1
1046	SUCLG2	6.362144029	Tumor_G1
1047	SLC25A5	8.390333686	Tumor_G1
1048	UCP2	8.040515296	Tumor_G1
1049	SOD2	5.133721286	Tumor_G1
1050	NOX4	1.948044484	Tumor_G1
1051	DUOX1	5.822923338	Tumor_G1
1052	DUOXA1	5.905678921	Tumor_G1
1053	DUOX2	5.61345673	Tumor_G1
1054	DUOXA2	2.869115675	Tumor_G1
1055	NDUFA1	7.417116943	Tumor_G1
1056	NDUFA2	6.376867607	Tumor_G1
1057	NDUFA8	6.575646914	Tumor_G1
1058	NDUFB1	4.964181899	Tumor_G1
1059	NDUFB10	7.30795584	Tumor_G1
1060	NDUFB2	5.830325595	Tumor_G1
1061	NDUFB3	5.928202343	Tumor_G1
1062	NDUFB7	8.200945284	Tumor_G1
1063	CYC1	6.509306857	Tumor_G1
1064	ETFB	4.274899842	Tumor_G1
1065	UQCR10	7.070275994	Tumor_G1
1066	UQCRQ	6.763713416	Tumor_G1
1067	UQCC3	3.899992076	Tumor_G1
1068	COX15	4.840207212	Tumor_G1
1069	COX5A	6.903356195	Tumor_G1
1070	COX6B1	8.233619339	Tumor_G1
1071	COX6C	5.843714548	Tumor_G1
1072	COX7A2	6.415790367	Tumor_G1
1073	COX7B	5.484562647	Tumor_G1
1074	COX8A	8.949560585	Tumor_G1
1075	SCO1	3.312208874	Tumor_G1
1076	SURF1	5.782586554	Tumor_G1
1077	ATP6V1E1	7.12660619	Tumor_G1
1078	DLAT	5.443697227	Tumor_G1

1079	PDC	0.052125097	Tumor_G1
1080	SUCLG2	6.052011449	Tumor_G1
1081	SLC25A5	8.109485595	Tumor_G1
1082	UCP2	7.929735583	Tumor_G1
1083	SOD2	5.591589746	Tumor_G1
1084	NOX4	3.066633132	Tumor_G1
1085	DUOX1	5.94006302	Tumor_G1
1086	DUOXA1	6.072861788	Tumor_G1
1087	DUOX2	6.001673012	Tumor_G1
1088	DUOXA2	3.927490325	Tumor_G1
1089	NDUFA1	7.662197128	Tumor_G1
1090	NDUFA2	5.763791216	Tumor_G1
1091	NDUFA8	6.651492475	Tumor_G1
1092	NDUFB1	5.026354622	Tumor_G1
1093	NDUFB10	6.860251366	Tumor_G1
1094	NDUFB2	5.76283656	Tumor_G1
1095	NDUFB3	5.928864281	Tumor_G1
1096	NDUFB7	8.162110842	Tumor_G1
1097	CYC1	6.110378903	Tumor_G1
1098	ETFB	4.612991717	Tumor_G1
1099	UQCR10	7.229966462	Tumor_G1
1100	UQCRQ	6.424562609	Tumor_G1
1101	UQCC3	3.409742649	Tumor_G1
1102	COX15	5.033804495	Tumor_G1
1103	COX5A	6.251449372	Tumor_G1
1104	COX6B1	8.278569985	Tumor_G1
1105	COX6C	5.629503229	Tumor_G1
1106	COX7A2	6.179918281	Tumor_G1
1107	COX7B	5.717440152	Tumor_G1
1108	COX8A	8.511925909	Tumor_G1
1109	SCO1	3.362001632	Tumor_G1
1110	SURF1	5.705803336	Tumor_G1
1111	ATP6V1E1	6.792681906	Tumor_G1
1112	DLAT	5.049972565	Tumor_G1
1113	PDC	0.189741484	Tumor_G1
1114	SUCLG2	6.029227448	Tumor_G1
1115	SLC25A5	7.628688788	Tumor_G1
1116	UCP2	8.332035803	Tumor_G1
1117	SOD2	4.811923183	Tumor_G1
1118	NOX4	1.952817241	Tumor_G1

1119	DUOX1	6.606282653	Tumor_G1
1120	DUOXA1	6.432896633	Tumor_G1
1121	DUOX2	7.077723544	Tumor_G1
1122	DUOXA2	4.628118512	Tumor_G1
1123	NDUFA1	7.842974007	Tumor_G1
1124	NDUFA2	6.00851895	Tumor_G1
1125	NDUFA8	6.833178854	Tumor_G1
1126	NDUFB1	5.771169534	Tumor_G1
1127	NDUFB10	6.959459454	Tumor_G1
1128	NDUFB2	6.096919465	Tumor_G1
1129	NDUFB3	5.952999844	Tumor_G1
1130	NDUFB7	8.296677507	Tumor_G1
1131	CYC1	6.695327671	Tumor_G1
1132	ETFB	4.378646017	Tumor_G1
1133	UQCR10	6.629841249	Tumor_G1
1134	UQCRQ	7.059847265	Tumor_G1
1135	UQCC3	3.575485228	Tumor_G1
1136	COX15	4.592583748	Tumor_G1
1137	COX5A	6.840197997	Tumor_G1
1138	COX6B1	8.637243856	Tumor_G1
1139	COX6C	6.163998019	Tumor_G1
1140	COX7A2	6.394887112	Tumor_G1
1141	COX7B	6.243829024	Tumor_G1
1142	COX8A	8.675848346	Tumor_G1
1143	SCO1	2.931219126	Tumor_G1
1144	SURF1	4.930297303	Tumor_G1
1145	ATP6V1E1	5.623721126	Tumor_G1
1146	DLAT	4.806687479	Tumor_G1
1147	PDC	0	Tumor_G1
1148	SUCLG2	6.187607151	Tumor_G1
1149	SLC25A5	8.226891461	Tumor_G1
1150	UCP2	7.216256087	Tumor_G1
1151	SOD2	3.903299032	Tumor_G1
1152	NOX4	0.956630604	Tumor_G1
1153	DUOX1	6.75867079	Tumor_G1
1154	DUOXA1	6.517126567	Tumor_G1
1155	DUOX2	8.763815033	Tumor_G1
1156	DUOXA2	6.031664542	Tumor_G1
1157	NDUFA1	7.644800707	Tumor_G1
1158	NDUFA2	6.433371663	Tumor_G1

1159	NDUFA8	6.945333509	Tumor_G1
1160	NDUFB1	5.172466926	Tumor_G1
1161	NDUFB10	7.444453095	Tumor_G1
1162	NDUFB2	6.699442759	Tumor_G1
1163	NDUFB3	6.121180188	Tumor_G1
1164	NDUFB7	8.516312614	Tumor_G1
1165	CYC1	7.058441361	Tumor_G1
1166	ETFB	4.766358648	Tumor_G1
1167	UQCR10	7.431625042	Tumor_G1
1168	UQCRQ	7.055344204	Tumor_G1
1169	UQCC3	3.896098162	Tumor_G1
1170	COX15	4.730695557	Tumor_G1
1171	COX5A	7.287489253	Tumor_G1
1172	COX6B1	8.591501866	Tumor_G1
1173	COX6C	6.252915742	Tumor_G1
1174	COX7A2	6.715901852	Tumor_G1
1175	COX7B	5.878938713	Tumor_G1
1176	COX8A	9.490809546	Tumor_G1
1177	SCO1	3.284104301	Tumor_G1
1178	SURF1	6.453709673	Tumor_G1
1179	ATP6V1E1	7.076009473	Tumor_G1
1180	DLAT	5.312437492	Tumor_G1
1181	PDC	0.062476323	Tumor_G1
1182	SUCLG2	6.420077799	Tumor_G1
1183	SLC25A5	8.978737531	Tumor_G1
1184	UCP2	8.926080965	Tumor_G1
1185	SOD2	5.721258302	Tumor_G1
1186	NOX4	1.99786676	Tumor_G1
1187	DUOX1	5.4441213	Tumor_G1
1188	DUOXA1	5.468264394	Tumor_G1
1189	DUOX2	5.09377165	Tumor_G1
1190	DUOXA2	3.267745113	Tumor_G1
1191	NDUFA1	7.61349758	Tumor_G1
1192	NDUFA2	6.014105166	Tumor_G1
1193	NDUFA8	6.893437143	Tumor_G1
1194	NDUFB1	5.125886464	Tumor_G1
1195	NDUFB10	7.238606002	Tumor_G1
1196	NDUFB2	5.515619088	Tumor_G1
1197	NDUFB3	5.961097261	Tumor_G1
1198	NDUFB7	8.356484909	Tumor_G1

1199	CYC1	6.256896001	Tumor_G1
1200	ETFB	4.525924062	Tumor_G1
1201	UQCR10	7.240260283	Tumor_G1
1202	UQCRQ	6.703935078	Tumor_G1
1203	UQCC3	3.753065049	Tumor_G1
1204	COX15	5.049204988	Tumor_G1
1205	COX5A	6.724305688	Tumor_G1
1206	COX6B1	8.68397903	Tumor_G1
1207	COX6C	5.772101443	Tumor_G1
1208	COX7A2	6.541426622	Tumor_G1
1209	COX7B	5.853311086	Tumor_G1
1210	COX8A	8.739438933	Tumor_G1
1211	SCO1	3.507099832	Tumor_G1
1212	SURF1	6.085034678	Tumor_G1
1213	ATP6V1E1	6.593178549	Tumor_G1
1214	DLAT	5.500879648	Tumor_G1
1215	PDC	0.295903586	Tumor_G1
1216	SUCLG2	6.21829146	Tumor_G1
1217	SLC25A5	8.086882885	Tumor_G1
1218	UCP2	8.734864096	Tumor_G1
1219	SOD2	4.811166672	Tumor_G1
1220	NOX4	2.994696538	Tumor_G1
1221	DUOX1	7.105683372	Tumor_G1
1222	DUOXA1	7.016458302	Tumor_G1
1223	DUOX2	7.825614565	Tumor_G1
1224	DUOXA2	5.13287664	Tumor_G1
1225	NDUFA1	7.751446838	Tumor_G1
1226	NDUFA2	6.568003069	Tumor_G1
1227	NDUFA8	6.91586711	Tumor_G1
1228	NDUFB1	5.25113913	Tumor_G1
1229	NDUFB10	7.185388072	Tumor_G1
1230	NDUFB2	6.28444038	Tumor_G1
1231	NDUFB3	5.985074005	Tumor_G1
1232	NDUFB7	8.357416274	Tumor_G1
1233	CYC1	6.916129261	Tumor_G1
1234	ETFB	5.100399908	Tumor_G1
1235	UQCR10	7.54904388	Tumor_G1
1236	UQCRQ	6.883250109	Tumor_G1
1237	UQCC3	4.379832466	Tumor_G1
1238	COX15	5.144820289	Tumor_G1

1239	COX5A	6.662815802	Tumor_G1
1240	COX6B1	8.363288906	Tumor_G1
1241	COX6C	6.177581328	Tumor_G1
1242	COX7A2	6.501153983	Tumor_G1
1243	COX7B	5.783247734	Tumor_G1
1244	COX8A	9.303291926	Tumor_G1
1245	SCO1	3.386282905	Tumor_G1
1246	SURF1	6.106018608	Tumor_G1
1247	ATP6V1E1	6.868194024	Tumor_G1
1248	DLAT	5.092210177	Tumor_G1
1249	PDC	0.124139527	Tumor_G1
1250	SUCLG2	6.256310003	Tumor_G1
1251	SLC25A5	8.026185642	Tumor_G1
1252	UCP2	8.155180142	Tumor_G1
1253	SOD2	5.632355532	Tumor_G1
1254	NOX4	2.086377095	Tumor_G1
1255	DUOX1	6.583510325	Tumor_G1
1256	DUOXA1	6.520632103	Tumor_G1
1257	DUOX2	6.985023848	Tumor_G1
1258	DUOXA2	4.750100367	Tumor_G1
1259	NDUFA1	7.484590016	Tumor_G1
1260	NDUFA2	6.258493719	Tumor_G1
1261	NDUFA8	6.852770864	Tumor_G1
1262	NDUFB1	5.092613623	Tumor_G1
1263	NDUFB10	7.37571625	Tumor_G1
1264	NDUFB2	5.62272391	Tumor_G1
1265	NDUFB3	5.851542463	Tumor_G1
1266	NDUFB7	8.193742264	Tumor_G1
1267	CYC1	6.761521358	Tumor_G1
1268	ETFB	4.917356915	Tumor_G1
1269	UQCR10	7.087582201	Tumor_G1
1270	UQCRQ	6.640967017	Tumor_G1
1271	UQCC3	4.21910324	Tumor_G1
1272	COX15	5.217287897	Tumor_G1
1273	COX5A	6.632005773	Tumor_G1
1274	COX6B1	8.210836879	Tumor_G1
1275	COX6C	5.861058341	Tumor_G1
1276	COX7A2	6.515352788	Tumor_G1
1277	COX7B	5.767054108	Tumor_G1
1278	COX8A	8.992205653	Tumor_G1

1279	SCO1	3.587452338	Tumor_G1
1280	SURF1	6.40394658	Tumor_G1
1281	ATP6V1E1	6.761896891	Tumor_G1
1282	DLAT	5.468100189	Tumor_G1
1283	PDC	0	Tumor_G1
1284	SUCLG2	6.210087588	Tumor_G1
1285	SLC25A5	8.13342033	Tumor_G1
1286	UCP2	7.805689769	Tumor_G1
1287	SOD2	6.68771664	Tumor_G1
1288	NOX4	3.026262797	Tumor_G1
1289	DUOX1	7.170731135	Tumor_G1
1290	DUOXA1	7.17402817	Tumor_G1
1291	DUOX2	7.586840625	Tumor_G1
1292	DUOXA2	4.422315745	Tumor_G1
1293	NDUFA1	7.413838497	Tumor_G1
1294	NDUFA2	6.223504917	Tumor_G1
1295	NDUFA8	6.827369522	Tumor_G1
1296	NDUFB1	4.957045895	Tumor_G1
1297	NDUFB10	7.198458651	Tumor_G1
1298	NDUFB2	6.082546039	Tumor_G1
1299	NDUFB3	5.869362636	Tumor_G1
1300	NDUFB7	8.216275581	Tumor_G1
1301	CYC1	6.707678452	Tumor_G1
1302	ETFB	4.73024626	Tumor_G1
1303	UQCR10	7.191238518	Tumor_G1
1304	UQCRQ	6.638410077	Tumor_G1
1305	UQCC3	4.220085285	Tumor_G1
1306	COX15	5.181495643	Tumor_G1
1307	COX5A	6.514895959	Tumor_G1
1308	COX6B1	8.31088478	Tumor_G1
1309	COX6C	5.937678151	Tumor_G1
1310	COX7A2	6.383733675	Tumor_G1
1311	COX7B	5.560162778	Tumor_G1
1312	COX8A	9.285845776	Tumor_G1
1313	SCO1	3.614817755	Tumor_G1
1314	SURF1	6.392947341	Tumor_G1
1315	ATP6V1E1	6.84508814	Tumor_G1
1316	DLAT	5.405920665	Tumor_G1
1317	PDC	0.187691317	Tumor_G1
1318	SUCLG2	5.972984638	Tumor_G1

1319	SLC25A5	8.084601755	Tumor_G1
1320	UCP2	8.556983355	Tumor_G1
1321	SOD2	5.190199661	Tumor_G1
1322	NOX4	2.367560079	Tumor_G1
1323	DUOX1	6.755810688	Tumor_G1
1324	DUOXA1	6.846668732	Tumor_G1
1325	DUOX2	8.0005845	Tumor_G1
1326	DUOXA2	5.770510134	Tumor_G1
1327	NDUFA1	7.924529571	Tumor_G1
1328	NDUFA2	6.354330657	Tumor_G1
1329	NDUFA8	6.938961497	Tumor_G1
1330	NDUFB1	5.543430828	Tumor_G1
1331	NDUFB10	7.247903409	Tumor_G1
1332	NDUFB2	6.339884864	Tumor_G1
1333	NDUFB3	6.132950415	Tumor_G1
1334	NDUFB7	8.065921251	Tumor_G1
1335	CYC1	6.71900684	Tumor_G1
1336	ETFB	4.511539155	Tumor_G1
1337	UQCR10	7.325729502	Tumor_G1
1338	UQCRQ	7.071120282	Tumor_G1
1339	UQCC3	3.591238211	Tumor_G1
1340	COX15	5.065795969	Tumor_G1
1341	COX5A	7.2048086	Tumor_G1
1342	COX6B1	8.578576404	Tumor_G1
1343	COX6C	6.506514032	Tumor_G1
1344	COX7A2	6.768961878	Tumor_G1
1345	COX7B	5.990913368	Tumor_G1
1346	COX8A	8.830054281	Tumor_G1
1347	SCO1	3.180673815	Tumor_G1
1348	SURF1	6.25369982	Tumor_G1
1349	ATP6V1E1	7.178183083	Tumor_G1
1350	DLAT	5.471881686	Tumor_G1
1351	PDC	0.190145468	Tumor_G1
1352	SUCLG2	6.402141761	Tumor_G1
1353	SLC25A5	8.056492038	Tumor_G1
1354	UCP2	8.392044268	Tumor_G1
1355	SOD2	6.022220831	Tumor_G1
1356	NOX4	3.11930076	Tumor_G1
1357	DUOX1	5.489171392	Tumor_G1
1358	DUOXA1	6.109741543	Tumor_G1

1359	DUOX2	6.188306639	Tumor_G1
1360	DUOXA2	3.522058255	Tumor_G1
1361	NDUFA1	7.767300394	Tumor_G1
1362	NDUFA2	6.564710014	Tumor_G1
1363	NDUFA8	6.855949907	Tumor_G1
1364	NDUFB1	5.572143442	Tumor_G1
1365	NDUFB10	7.361443426	Tumor_G1
1366	NDUFB2	6.304406309	Tumor_G1
1367	NDUFB3	6.03116474	Tumor_G1
1368	NDUFB7	8.442662717	Tumor_G1
1369	CYC1	6.721112551	Tumor_G1
1370	ETFB	5.051804008	Tumor_G1
1371	UQCR10	7.369698112	Tumor_G1
1372	UQCRQ	7.023261596	Tumor_G1
1373	UQCC3	4.326420161	Tumor_G1
1374	COX15	4.801821776	Tumor_G1
1375	COX5A	6.879178569	Tumor_G1
1376	COX6B1	8.591333782	Tumor_G1
1377	COX6C	6.462310302	Tumor_G1
1378	COX7A2	6.734373666	Tumor_G1
1379	COX7B	5.857944217	Tumor_G1
1380	COX8A	9.525471611	Tumor_G1
1381	SCO1	3.007938824	Tumor_G1
1382	SURF1	6.095217493	Tumor_G1
1383	ATP6V1E1	7.145343527	Tumor_G1
1384	DLAT	5.011489715	Tumor_G1
1385	PDC	0	Tumor_G1
1386	SUCLG2	6.286905707	Tumor_G1
1387	SLC25A5	8.123194944	Tumor_G1
1388	UCP2	7.67936974	Tumor_G1
1389	SOD2	5.416929472	Tumor_G1
1390	NOX4	2.039253612	Tumor_G1
1391	DUOX1	5.616714537	Tumor_G1
1392	DUOXA1	5.760304487	Tumor_G1
1393	DUOX2	5.163227551	Tumor_G1
1394	DUOXA2	3.418076325	Tumor_G1
1395	NDUFA1	7.868841979	Tumor_G1
1396	NDUFA2	7.130263425	Tumor_G1
1397	NDUFA8	7.127323551	Tumor_G1
1398	NDUFB1	5.847612329	Tumor_G1

1399	NDUFB10	7.469359789	Tumor_G1
1400	NDUFB2	5.904258232	Tumor_G1
1401	NDUFB3	5.850941332	Tumor_G1
1402	NDUFB7	8.681426046	Tumor_G1
1403	CYC1	6.340239669	Tumor_G1
1404	ETFB	4.988957559	Tumor_G1
1405	UQCR10	7.535481328	Tumor_G1
1406	UQCRQ	7.430279124	Tumor_G1
1407	UQCC3	4.179255677	Tumor_G1
1408	COX15	4.14904883	Tumor_G1
1409	COX5A	6.794602031	Tumor_G1
1410	COX6B1	9.127412895	Tumor_G1
1411	COX6C	6.441621957	Tumor_G1
1412	COX7A2	6.595284974	Tumor_G1
1413	COX7B	6.154587232	Tumor_G1
1414	COX8A	8.521302652	Tumor_G1
1415	SCO1	2.603447304	Tumor_G1
1416	SURF1	6.306282151	Tumor_G1
1417	ATP6V1E1	6.326240996	Tumor_G1
1418	DLAT	4.192827391	Tumor_G1
1419	PDC	0	Tumor_G1
1420	SUCLG2	5.341374762	Tumor_G1
1421	SLC25A5	7.5747568	Tumor_G1
1422	UCP2	6.945869579	Tumor_G1
1423	SOD2	5.444523817	Tumor_G1
1424	NOX4	2.069584502	Tumor_G1
1425	DUOX1	3.273378219	Tumor_G1
1426	DUOXA1	3.971134677	Tumor_G1
1427	DUOX2	1.748851439	Tumor_G1
1428	DUOXA2	1.279281105	Tumor_G1
1429	NDUFA1	7.773588667	Tumor_G1
1430	NDUFA2	6.258945213	Tumor_G1
1431	NDUFA8	6.837278552	Tumor_G1
1432	NDUFB1	5.339854836	Tumor_G1
1433	NDUFB10	7.024087995	Tumor_G1
1434	NDUFB2	6.185251596	Tumor_G1
1435	NDUFB3	6.296051967	Tumor_G1
1436	NDUFB7	8.226083691	Tumor_G1
1437	CYC1	6.612011265	Tumor_G1
1438	ETFB	4.88467557	Tumor_G1

1439	UQCR10	7.448948615	Tumor_G1
1440	UQCRQ	6.826559228	Tumor_G1
1441	UQCC3	3.774012511	Tumor_G1
1442	COX15	5.045721016	Tumor_G1
1443	COX5A	6.965729041	Tumor_G1
1444	COX6B1	8.479334076	Tumor_G1
1445	COX6C	6.160447771	Tumor_G1
1446	COX7A2	6.708234851	Tumor_G1
1447	COX7B	5.977445628	Tumor_G1
1448	COX8A	8.965613069	Tumor_G1
1449	SCO1	3.370695535	Tumor_G1
1450	SURF1	5.969281338	Tumor_G1
1451	ATP6V1E1	7.021310498	Tumor_G1
1452	DLAT	5.190676669	Tumor_G1
1453	PDC	0.232271048	Tumor_G1
1454	SUCLG2	6.223084424	Tumor_G1
1455	SLC25A5	8.416427871	Tumor_G1
1456	UCP2	7.860062029	Tumor_G1
1457	SOD2	5.674796609	Tumor_G1
1458	NOX4	2.971322608	Tumor_G1
1459	DUOX1	6.07004175	Tumor_G1
1460	DUOXA1	5.999429693	Tumor_G1
1461	DUOX2	6.576307362	Tumor_G1
1462	DUOXA2	4.193275566	Tumor_G1
1463	NDUFA1	7.963494569	Tumor_G1
1464	NDUFA2	6.561714906	Tumor_G1
1465	NDUFA8	7.084040091	Tumor_G1
1466	NDUFB1	5.539284905	Tumor_G1
1467	NDUFB10	7.566889388	Tumor_G1
1468	NDUFB2	6.047881144	Tumor_G1
1469	NDUFB3	6.230951468	Tumor_G1
1470	NDUFB7	8.507334821	Tumor_G1
1471	CYC1	6.376439144	Tumor_G1
1472	ETFB	4.719808051	Tumor_G1
1473	UQCR10	7.418182945	Tumor_G1
1474	UQCRQ	7.240777671	Tumor_G1
1475	UQCC3	3.965607418	Tumor_G1
1476	COX15	4.658173267	Tumor_G1
1477	COX5A	7.230938644	Tumor_G1
1478	COX6B1	8.715023477	Tumor_G1

1479	COX6C	6.46837085	Tumor_G1
1480	COX7A2	6.89169371	Tumor_G1
1481	COX7B	6.018131511	Tumor_G1
1482	COX8A	9.100319952	Tumor_G1
1483	SCO1	3.296612611	Tumor_G1
1484	SURF1	6.246975523	Tumor_G1
1485	ATP6V1E1	7.039897423	Tumor_G1
1486	DLAT	5.279167831	Tumor_G1
1487	PDC	0.11884254	Tumor_G1
1488	SUCLG2	5.970162142	Tumor_G1
1489	SLC25A5	7.688509924	Tumor_G1
1490	UCP2	8.837434932	Tumor_G1
1491	SOD2	5.169803501	Tumor_G1
1492	NOX4	3.883773944	Tumor_G1
1493	DUOX1	5.854969601	Tumor_G1
1494	DUOXA1	6.350173084	Tumor_G1
1495	DUOX2	6.914187835	Tumor_G1
1496	DUOXA2	4.773437165	Tumor_G1
1497	NDUFA1	7.912465048	Tumor_G1
1498	NDUFA2	6.348037184	Tumor_G1
1499	NDUFA8	7.045285074	Tumor_G1
1500	NDUFB1	5.317160225	Tumor_G1
1501	NDUFB10	7.254185502	Tumor_G1
1502	NDUFB2	5.771087239	Tumor_G1
1503	NDUFB3	6.360565924	Tumor_G1
1504	NDUFB7	8.272075464	Tumor_G1
1505	CYC1	6.730050166	Tumor_G1
1506	ETFB	4.61993984	Tumor_G1
1507	UQCR10	7.366729749	Tumor_G1
1508	UQCRQ	7.065789236	Tumor_G1
1509	UQCC3	3.685044647	Tumor_G1
1510	COX15	5.056472096	Tumor_G1
1511	COX5A	7.235229724	Tumor_G1
1512	COX6B1	8.484151709	Tumor_G1
1513	COX6C	6.291302175	Tumor_G1
1514	COX7A2	6.719134097	Tumor_G1
1515	COX7B	5.988923138	Tumor_G1
1516	COX8A	9.077973016	Tumor_G1
1517	SCO1	3.732919574	Tumor_G1
1518	SURF1	5.804256372	Tumor_G1

1519	ATP6V1E1	7.011351634	Tumor_G1
1520	DLAT	5.186847181	Tumor_G1
1521	PDC	0	Tumor_G1
1522	SUCLG2	6.149053116	Tumor_G1
1523	SLC25A5	9.034731577	Tumor_G1
1524	UCP2	7.750346227	Tumor_G1
1525	SOD2	6.39782187	Tumor_G1
1526	NOX4	3.560146448	Tumor_G1
1527	DUOX1	4.808100642	Tumor_G1
1528	DUOXA1	4.770074829	Tumor_G1
1529	DUOX2	4.137213782	Tumor_G1
1530	DUOXA2	2.606314437	Tumor_G1
1531	NDUFA1	7.725642496	Tumor_G1
1532	NDUFA2	6.643867724	Tumor_G1
1533	NDUFA8	6.858840449	Tumor_G1
1534	NDUFB1	5.27503258	Tumor_G1
1535	NDUFB10	7.264644314	Tumor_G1
1536	NDUFB2	6.554764529	Tumor_G1
1537	NDUFB3	6.220026308	Tumor_G1
1538	NDUFB7	8.518826741	Tumor_G1
1539	CYC1	6.826606683	Tumor_G1
1540	ETFB	4.667101906	Tumor_G1
1541	UQCR10	7.439933535	Tumor_G1
1542	UQCRQ	7.082033827	Tumor_G1
1543	UQCC3	3.911404934	Tumor_G1
1544	COX15	4.724715999	Tumor_G1
1545	COX5A	6.770923198	Tumor_G1
1546	COX6B1	8.716758096	Tumor_G1
1547	COX6C	6.329539436	Tumor_G1
1548	COX7A2	6.731476847	Tumor_G1
1549	COX7B	5.862947886	Tumor_G1
1550	COX8A	9.049283288	Tumor_G1
1551	SCO1	3.203884272	Tumor_G1
1552	SURF1	6.181466338	Tumor_G1
1553	ATP6V1E1	7.291714688	Tumor_G1
1554	DLAT	5.070340071	Tumor_G1
1555	PDC	0.10614446	Tumor_G1
1556	SUCLG2	6.161916792	Tumor_G1
1557	SLC25A5	8.477430652	Tumor_G1
1558	UCP2	8.342525761	Tumor_G1

1559	SOD2	5.555396234	Tumor_G1
1560	NOX4	3.985631342	Tumor_G1
1561	DUOX1	6.074338828	Tumor_G1
1562	DUOXA1	6.315735644	Tumor_G1
1563	DUOX2	5.395529232	Tumor_G1
1564	DUOXA2	3.281840763	Tumor_G1
1565	NDUFA1	7.63139356	Tumor_G1
1566	NDUFA2	6.350319277	Tumor_G1
1567	NDUFA8	6.50994713	Tumor_G1
1568	NDUFB1	5.3763353	Tumor_G1
1569	NDUFB10	7.070287285	Tumor_G1
1570	NDUFB2	5.91482246	Tumor_G1
1571	NDUFB3	5.946993142	Tumor_G1
1572	NDUFB7	8.234348699	Tumor_G1
1573	CYC1	6.261915042	Tumor_G1
1574	ETFB	4.595823716	Tumor_G1
1575	UQCR10	7.112726476	Tumor_G1
1576	UQCRQ	6.934949269	Tumor_G1
1577	UQCC3	3.993036845	Tumor_G1
1578	COX15	4.01398574	Tumor_G1
1579	COX5A	6.718187161	Tumor_G1
1580	COX6B1	8.314803967	Tumor_G1
1581	COX6C	5.94727658	Tumor_G1
1582	COX7A2	6.409016348	Tumor_G1
1583	COX7B	5.563660808	Tumor_G1
1584	COX8A	8.804096538	Tumor_G1
1585	SCO1	2.613024051	Tumor_G1
1586	SURF1	5.996393213	Tumor_G1
1587	ATP6V1E1	6.557961493	Tumor_G1
1588	DLAT	4.322985792	Tumor_G1
1589	PDC	0.086405242	Tumor_G1
1590	SUCLG2	5.507536794	Tumor_G1
1591	SLC25A5	7.466308215	Tumor_G1
1592	UCP2	6.986185072	Tumor_G1
1593	SOD2	5.122736934	Tumor_G1
1594	NOX4	2.559287332	Tumor_G1
1595	DUOX1	4.423824024	Tumor_G1
1596	DUOXA1	5.546658282	Tumor_G1
1597	DUOX2	5.209887907	Tumor_G1
1598	DUOXA2	3.826426733	Tumor_G1

1599	NDUFA1	7.781132238	Tumor_G1
1600	NDUFA2	6.583100956	Tumor_G1
1601	NDUFA8	6.834509311	Tumor_G1
1602	NDUFB1	5.376955222	Tumor_G1
1603	NDUFB10	7.21992826	Tumor_G1
1604	NDUFB2	6.317691864	Tumor_G1
1605	NDUFB3	6.206930814	Tumor_G1
1606	NDUFB7	8.18334618	Tumor_G1
1607	CYC1	6.950413159	Tumor_G1
1608	ETFB	4.698460242	Tumor_G1
1609	UQCR10	7.483000678	Tumor_G1
1610	UQCRQ	6.891611376	Tumor_G1
1611	UQCC3	3.677454191	Tumor_G1
1612	COX15	5.073384962	Tumor_G1
1613	COX5A	6.862452184	Tumor_G1
1614	COX6B1	8.630598985	Tumor_G1
1615	COX6C	6.085911564	Tumor_G1
1616	COX7A2	6.616218254	Tumor_G1
1617	COX7B	5.940081849	Tumor_G1
1618	COX8A	8.94714708	Tumor_G1
1619	SCO1	3.509857174	Tumor_G1
1620	SURF1	6.050536204	Tumor_G1
1621	ATP6V1E1	7.178894024	Tumor_G1
1622	DLAT	5.206088007	Tumor_G1
1623	PDC	0.323612646	Tumor_G1
1624	SUCLG2	6.40696611	Tumor_G1
1625	SLC25A5	8.681005967	Tumor_G1
1626	UCP2	7.563525195	Tumor_G1
1627	SOD2	5.857322224	Tumor_G1
1628	NOX4	2.275810762	Tumor_G1
1629	DUOX1	6.474822578	Tumor_G1
1630	DUOXA1	6.245500896	Tumor_G1
1631	DUOX2	6.36123871	Tumor_G1
1632	DUOXA2	4.119783877	Tumor_G1
1633	NDUFA1	7.760964781	Tumor_G1
1634	NDUFA2	6.580738187	Tumor_G1
1635	NDUFA8	6.860394417	Tumor_G1
1636	NDUFB1	5.375864425	Tumor_G1
1637	NDUFB10	7.269023145	Tumor_G1
1638	NDUFB2	6.474381085	Tumor_G1

1639	NDUFB3	6.18210568	Tumor_G1
1640	NDUFB7	8.369531869	Tumor_G1
1641	CYC1	6.764059615	Tumor_G1
1642	ETFB	4.939393745	Tumor_G1
1643	UQCR10	7.426264792	Tumor_G1
1644	UQCRQ	6.901306935	Tumor_G1
1645	UQCC3	3.99466632	Tumor_G1
1646	COX15	4.93756396	Tumor_G1
1647	COX5A	6.722382993	Tumor_G1
1648	COX6B1	8.443098706	Tumor_G1
1649	COX6C	6.202525795	Tumor_G1
1650	COX7A2	6.754013884	Tumor_G1
1651	COX7B	5.765767228	Tumor_G1
1652	COX8A	9.32744005	Tumor_G1
1653	SCO1	3.185255308	Tumor_G1
1654	SURF1	5.801386218	Tumor_G1
1655	ATP6V1E1	7.123138615	Tumor_G1
1656	DLAT	5.04221201	Tumor_G1
1657	PDC	0.045721512	Tumor_G1
1658	SUCLG2	6.471229906	Tumor_G1
1659	SLC25A5	7.99111877	Tumor_G1
1660	UCP2	8.349462222	Tumor_G1
1661	SOD2	5.202254889	Tumor_G1
1662	NOX4	2.192725574	Tumor_G1
1663	DUOX1	6.012085967	Tumor_G1
1664	DUOXA1	6.254328742	Tumor_G1
1665	DUOX2	5.388904755	Tumor_G1
1666	DUOXA2	3.579657861	Tumor_G1
1667	NDUFA1	7.752348499	Tumor_G1
1668	NDUFA2	6.800233564	Tumor_G1
1669	NDUFA8	6.893309001	Tumor_G1
1670	NDUFB1	5.461035477	Tumor_G1
1671	NDUFB10	7.388363768	Tumor_G1
1672	NDUFB2	6.507950598	Tumor_G1
1673	NDUFB3	6.154541275	Tumor_G1
1674	NDUFB7	8.352514326	Tumor_G1
1675	CYC1	6.894708449	Tumor_G1
1676	ETFB	4.772925627	Tumor_G1
1677	UQCR10	7.568009224	Tumor_G1
1678	UQCRQ	7.308999775	Tumor_G1

1679	UQCC3	4.178893538	Tumor_G1
1680	COX15	4.610833322	Tumor_G1
1681	COX5A	7.091442527	Tumor_G1
1682	COX6B1	8.637219048	Tumor_G1
1683	COX6C	6.284774941	Tumor_G1
1684	COX7A2	7.012361428	Tumor_G1
1685	COX7B	5.950516431	Tumor_G1
1686	COX8A	9.451160361	Tumor_G1
1687	SCO1	2.671346669	Tumor_G1
1688	SURF1	6.261483049	Tumor_G1
1689	ATP6V1E1	7.167920868	Tumor_G1
1690	DLAT	5.051280556	Tumor_G1
1691	PDC	0.10727847	Tumor_G1
1692	SUCLG2	6.315474872	Tumor_G1
1693	SLC25A5	8.042268279	Tumor_G1
1694	UCP2	7.252063782	Tumor_G1
1695	SOD2	4.883927007	Tumor_G1
1696	NOX4	2.069078713	Tumor_G1
1697	DUOX1	5.872405795	Tumor_G1
1698	DUOXA1	5.889785248	Tumor_G1
1699	DUOX2	5.14896078	Tumor_G1
1700	DUOXA2	3.269119269	Tumor_G1
1701	NDUFA1	8.2049675	Tumor_G1
1702	NDUFA2	7.074353225	Tumor_G1
1703	NDUFA8	7.016580989	Tumor_G1
1704	NDUFB1	5.753402963	Tumor_G1
1705	NDUFB10	7.732165075	Tumor_G1
1706	NDUFB2	6.678766096	Tumor_G1
1707	NDUFB3	6.206081849	Tumor_G1
1708	NDUFB7	8.66431158	Tumor_G1
1709	CYC1	6.713251997	Tumor_G1
1710	ETFB	4.805153276	Tumor_G1
1711	UQCR10	7.84492985	Tumor_G1
1712	UQCRQ	7.504477097	Tumor_G1
1713	UQCC3	4.189308513	Tumor_G1
1714	COX15	3.948088528	Tumor_G1
1715	COX5A	7.609819711	Tumor_G1
1716	COX6B1	8.996541329	Tumor_G1
1717	COX6C	6.742848692	Tumor_G1
1718	COX7A2	7.227119507	Tumor_G1

1719	COX7B	6.293920222	Tumor_G1
1720	COX8A	9.292995895	Tumor_G1
1721	SCO1	2.374416355	Tumor_G1
1722	SURF1	5.813850818	Tumor_G1
1723	ATP6V1E1	7.212771527	Tumor_G1
1724	DLAT	4.509614628	Tumor_G1
1725	PDC	0.056299519	Tumor_G1
1726	SUCLG2	5.637640407	Tumor_G1
1727	SLC25A5	7.360531148	Tumor_G1
1728	UCP2	8.310562211	Tumor_G1
1729	SOD2	5.406686664	Tumor_G1
1730	NOX4	2.867497066	Tumor_G1
1731	DUOX1	5.373923569	Tumor_G1
1732	DUOXA1	5.661224195	Tumor_G1
1733	DUOX2	5.083386255	Tumor_G1
1734	DUOXA2	3.412708488	Tumor_G1
1735	NDUFA1	7.68785047	Tumor_G1
1736	NDUFA2	6.296106152	Tumor_G1
1737	NDUFA8	6.64246727	Tumor_G1
1738	NDUFB1	5.33887929	Tumor_G1
1739	NDUFB10	7.227927088	Tumor_G1
1740	NDUFB2	5.93601908	Tumor_G1
1741	NDUFB3	6.140027665	Tumor_G1
1742	NDUFB7	8.165018004	Tumor_G1
1743	CYC1	6.585172816	Tumor_G1
1744	ETFB	4.337747066	Tumor_G1
1745	UQCR10	7.041596969	Tumor_G1
1746	UQCRQ	6.773845871	Tumor_G1
1747	UQCC3	3.689599234	Tumor_G1
1748	COX15	4.767449115	Tumor_G1
1749	COX5A	7.006669799	Tumor_G1
1750	COX6B1	8.198362412	Tumor_G1
1751	COX6C	5.994827629	Tumor_G1
1752	COX7A2	6.640722467	Tumor_G1
1753	COX7B	5.653856773	Tumor_G1
1754	COX8A	8.999776746	Tumor_G1
1755	SCO1	3.315148435	Tumor_G1
1756	SURF1	5.990400211	Tumor_G1
1757	ATP6V1E1	6.959961719	Tumor_G1
1758	DLAT	5.167321566	Tumor_G1

1759	PDC	0	Tumor_G1
1760	SUCLG2	6.096159931	Tumor_G1
1761	SLC25A5	8.570640003	Tumor_G1
1762	UCP2	8.049383529	Tumor_G1
1763	SOD2	6.082830122	Tumor_G1
1764	NOX4	3.049873861	Tumor_G1
1765	DUOX1	5.534791924	Tumor_G1
1766	DUOXA1	5.612886151	Tumor_G1
1767	DUOX2	5.05295303	Tumor_G1
1768	DUOXA2	3.656266041	Tumor_G1
1769	NDUFA1	7.72433789	Tumor_G1
1770	NDUFA2	6.459036439	Tumor_G1
1771	NDUFA8	6.817260832	Tumor_G1
1772	NDUFB1	5.527121285	Tumor_G1
1773	NDUFB10	7.37704117	Tumor_G1
1774	NDUFB2	6.271254997	Tumor_G1
1775	NDUFB3	6.128719433	Tumor_G1
1776	NDUFB7	8.306212433	Tumor_G1
1777	CYC1	6.481706299	Tumor_G1
1778	ETFB	4.650216129	Tumor_G1
1779	UQCR10	7.288929602	Tumor_G1
1780	UQCRQ	6.908008414	Tumor_G1
1781	UQCC3	4.446370546	Tumor_G1
1782	COX15	4.776026749	Tumor_G1
1783	COX5A	6.981180186	Tumor_G1
1784	COX6B1	8.34951773	Tumor_G1
1785	COX6C	6.098673202	Tumor_G1
1786	COX7A2	6.629479691	Tumor_G1
1787	COX7B	5.736287091	Tumor_G1
1788	COX8A	9.084378567	Tumor_G1
1789	SCO1	3.484845752	Tumor_G1
1790	SURF1	6.500150511	Tumor_G1
1791	ATP6V1E1	6.989166113	Tumor_G1
1792	DLAT	5.025991554	Tumor_G1
1793	PDC	0.341837982	Tumor_G1
1794	SUCLG2	5.709460572	Tumor_G1
1795	SLC25A5	7.770242782	Tumor_G1
1796	UCP2	8.061141903	Tumor_G1
1797	SOD2	5.053072007	Tumor_G1
1798	NOX4	3.003251538	Tumor_G1

1799	DUOX1	6.12060679	Tumor_G1
1800	DUOXA1	6.384858223	Tumor_G1
1801	DUOX2	7.022387844	Tumor_G1
1802	DUOXA2	5.290121501	Tumor_G1
1803	NDUFA1	7.322037046	Tumor_G1
1804	NDUFA2	6.230930096	Tumor_G1
1805	NDUFA8	6.517031095	Tumor_G1
1806	NDUFB1	4.99840779	Tumor_G1
1807	NDUFB10	6.805902608	Tumor_G1
1808	NDUFB2	6.036582927	Tumor_G1
1809	NDUFB3	5.862612039	Tumor_G1
1810	NDUFB7	7.869961841	Tumor_G1
1811	CYC1	6.37265524	Tumor_G1
1812	ETFB	4.277398437	Tumor_G1
1813	UQCR10	6.965748556	Tumor_G1
1814	UQCRQ	6.73540567	Tumor_G1
1815	UQCC3	3.682207252	Tumor_G1
1816	COX15	4.262326022	Tumor_G1
1817	COX5A	6.502866422	Tumor_G1
1818	COX6B1	8.147766555	Tumor_G1
1819	COX6C	5.853299636	Tumor_G1
1820	COX7A2	6.450537043	Tumor_G1
1821	COX7B	5.567319848	Tumor_G1
1822	COX8A	8.755390283	Tumor_G1
1823	SCO1	2.937712961	Tumor_G1
1824	SURF1	5.564294378	Tumor_G1
1825	ATP6V1E1	7.072829137	Tumor_G1
1826	DLAT	4.716326093	Tumor_G1
1827	PDC	0.1483389	Tumor_G1
1828	SUCLG2	5.916139619	Tumor_G1
1829	SLC25A5	8.044992568	Tumor_G1
1830	UCP2	7.713438966	Tumor_G1
1831	SOD2	5.85491531	Tumor_G1
1832	NOX4	3.537895646	Tumor_G1
1833	DUOX1	4.711669594	Tumor_G1
1834	DUOXA1	5.016589657	Tumor_G1
1835	DUOX2	3.644422646	Tumor_G1
1836	DUOXA2	2.013452799	Tumor_G1
1837	NDUFA1	8.046050717	Tumor_G1
1838	NDUFA2	7.014180714	Tumor_G1

1839	NDUFA8	6.853381034	Tumor_G1
1840	NDUFB1	5.608305014	Tumor_G1
1841	NDUFB10	7.431573827	Tumor_G1
1842	NDUFB2	6.771109882	Tumor_G1
1843	NDUFB3	6.419651578	Tumor_G1
1844	NDUFB7	8.397820506	Tumor_G1
1845	CYC1	6.719396336	Tumor_G1
1846	ETFB	4.752530554	Tumor_G1
1847	UQCR10	7.716209339	Tumor_G1
1848	UQCRQ	7.293067807	Tumor_G1
1849	UQCC3	4.121915895	Tumor_G1
1850	COX15	4.557025403	Tumor_G1
1851	COX5A	7.197051938	Tumor_G1
1852	COX6B1	8.611846877	Tumor_G1
1853	COX6C	6.350215047	Tumor_G1
1854	COX7A2	6.853910554	Tumor_G1
1855	COX7B	5.91620385	Tumor_G1
1856	COX8A	9.442952514	Tumor_G1
1857	SCO1	3.132693531	Tumor_G1
1858	SURF1	6.256908882	Tumor_G1
1859	ATP6V1E1	7.322530403	Tumor_G1
1860	DLAT	4.856016697	Tumor_G1
1861	PDC	0	Tumor_G1
1862	SUCLG2	6.197424662	Tumor_G1
1863	SLC25A5	8.047324579	Tumor_G1
1864	UCP2	8.489070597	Tumor_G1
1865	SOD2	4.797005864	Tumor_G1
1866	NOX4	1.802353912	Tumor_G1
1867	DUOX1	5.768096498	Tumor_G1
1868	DUOXA1	5.877550687	Tumor_G1
1869	DUOX2	5.553670841	Tumor_G1
1870	DUOXA2	3.135710565	Tumor_G1
1871	NDUFA1	7.484615897	Tumor_G1
1872	NDUFA2	6.718327022	Tumor_G1
1873	NDUFA8	6.953050434	Tumor_G1
1874	NDUFB1	5.562043873	Tumor_G1
1875	NDUFB10	7.483399997	Tumor_G1
1876	NDUFB2	6.402439414	Tumor_G1
1877	NDUFB3	5.959550594	Tumor_G1
1878	NDUFB7	8.605471317	Tumor_G1

1879	CYC1	6.733131726	Tumor_G1
1880	ETFB	4.69903648	Tumor_G1
1881	UQCR10	7.308686796	Tumor_G1
1882	UQCRQ	7.173873862	Tumor_G1
1883	UQCC3	4.16052107	Tumor_G1
1884	COX15	4.257163913	Tumor_G1
1885	COX5A	7.007677851	Tumor_G1
1886	COX6B1	8.64166788	Tumor_G1
1887	COX6C	6.196246074	Tumor_G1
1888	COX7A2	6.607046415	Tumor_G1
1889	COX7B	5.872558803	Tumor_G1
1890	COX8A	9.164011983	Tumor_G1
1891	SCO1	3.003894034	Tumor_G1
1892	SURF1	6.027528622	Tumor_G1
1893	ATP6V1E1	6.649939228	Tumor_G1
1894	DLAT	4.611900239	Tumor_G1
1895	PDC	0.242750617	Tumor_G1
1896	SUCLG2	5.499194998	Tumor_G1
1897	SLC25A5	7.969122604	Tumor_G1
1898	UCP2	7.827029722	Tumor_G1
1899	SOD2	5.013287552	Tumor_G1
1900	NOX4	2.045958744	Tumor_G1
1901	DUOX1	5.86105741	Tumor_G1
1902	DUOXA1	6.067660941	Tumor_G1
1903	DUOX2	6.311668643	Tumor_G1
1904	DUOXA2	4.775876978	Tumor_G1
1905	NDUFA1	7.477571442	Tumor_G1
1906	NDUFA2	6.44743937	Tumor_G1
1907	NDUFA8	6.768772525	Tumor_G1
1908	NDUFB1	5.23496634	Tumor_G1
1909	NDUFB10	7.358027554	Tumor_G1
1910	NDUFB2	6.443081836	Tumor_G1
1911	NDUFB3	5.950051089	Tumor_G1
1912	NDUFB7	8.323851279	Tumor_G1
1913	CYC1	6.538692595	Tumor_G1
1914	ETFB	4.779233087	Tumor_G1
1915	UQCR10	7.237383628	Tumor_G1
1916	UQCRQ	7.030150598	Tumor_G1
1917	UQCC3	3.864947695	Tumor_G1
1918	COX15	4.755461103	Tumor_G1

1919	COX5A	7.055106664	Tumor_G1
1920	COX6B1	8.24703056	Tumor_G1
1921	COX6C	6.08237859	Tumor_G1
1922	COX7A2	6.561263087	Tumor_G1
1923	COX7B	5.555400647	Tumor_G1
1924	COX8A	9.177081882	Tumor_G1
1925	SCO1	3.235573767	Tumor_G1
1926	SURF1	6.011742353	Tumor_G1
1927	ATP6V1E1	7.082637232	Tumor_G1
1928	DLAT	5.37893239	Tumor_G1
1929	PDC	0.161133701	Tumor_G1
1930	SUCLG2	6.148303967	Tumor_G1
1931	SLC25A5	8.224790454	Tumor_G1
1932	UCP2	8.466779353	Tumor_G1
1933	SOD2	6.306198834	Tumor_G1
1934	NOX4	2.702918438	Tumor_G1
1935	DUOX1	5.664512967	Tumor_G1
1936	DUOXA1	5.987878001	Tumor_G1
1937	DUOX2	6.342272838	Tumor_G1
1938	DUOXA2	4.402914235	Tumor_G1
1939	NDUFA1	7.829907652	Tumor_G1
1940	NDUFA2	6.739483833	Tumor_G1
1941	NDUFA8	6.873112736	Tumor_G1
1942	NDUFB1	5.536499604	Tumor_G1
1943	NDUFB10	7.609272121	Tumor_G1
1944	NDUFB2	6.490023984	Tumor_G1
1945	NDUFB3	5.875532596	Tumor_G1
1946	NDUFB7	8.580806363	Tumor_G1
1947	CYC1	6.578098871	Tumor_G1
1948	ETFB	4.975889737	Tumor_G1
1949	UQCR10	7.362993751	Tumor_G1
1950	UQCRQ	7.193379624	Tumor_G1
1951	UQCC3	4.474006758	Tumor_G1
1952	COX15	4.48137708	Tumor_G1
1953	COX5A	6.912301922	Tumor_G1
1954	COX6B1	8.503753323	Tumor_G1
1955	COX6C	6.393642973	Tumor_G1
1956	COX7A2	6.599501091	Tumor_G1
1957	COX7B	5.74349271	Tumor_G1
1958	COX8A	9.209501489	Tumor_G1

1959	SCO1	2.745663022	Tumor_G1
1960	SURF1	6.338617805	Tumor_G1
1961	ATP6V1E1	6.714404781	Tumor_G1
1962	DLAT	4.517646309	Tumor_G1
1963	PDC	0	Tumor_G1
1964	SUCLG2	5.971594521	Tumor_G1
1965	SLC25A5	7.717586377	Tumor_G1
1966	UCP2	8.005732693	Tumor_G1
1967	SOD2	4.080070111	Tumor_G1
1968	NOX4	2.098045551	Tumor_G1
1969	DUOX1	5.769734346	Tumor_G1
1970	DUOXA1	6.044340272	Tumor_G1
1971	DUOX2	6.084122611	Tumor_G1
1972	DUOXA2	5.117683767	Tumor_G1
1973	NDUFA1	7.282427783	Tumor_G1
1974	NDUFA2	5.948111866	Tumor_G1
1975	NDUFA8	6.473988754	Tumor_G1
1976	NDUFB1	4.937517291	Tumor_G1
1977	NDUFB10	6.879919255	Tumor_G1
1978	NDUFB2	6.041013386	Tumor_G1
1979	NDUFB3	5.807539614	Tumor_G1
1980	NDUFB7	7.940075661	Tumor_G1
1981	CYC1	6.492355869	Tumor_G1
1982	ETFB	4.428448544	Tumor_G1
1983	UQCR10	7.078977851	Tumor_G1
1984	UQCRQ	6.533746176	Tumor_G1
1985	UQCC3	3.641677289	Tumor_G1
1986	COX15	5.017053658	Tumor_G1
1987	COX5A	6.45766522	Tumor_G1
1988	COX6B1	7.940221786	Tumor_G1
1989	COX6C	5.745902375	Tumor_G1
1990	COX7A2	6.286193413	Tumor_G1
1991	COX7B	5.481753218	Tumor_G1
1992	COX8A	8.815918915	Tumor_G1
1993	SCO1	3.175471041	Tumor_G1
1994	SURF1	5.733369423	Tumor_G1
1995	ATP6V1E1	7.013249468	Tumor_G1
1996	DLAT	5.182670294	Tumor_G1
1997	PDC	0.060389533	Tumor_G1
1998	SUCLG2	6.335963332	Tumor_G1

1999	SLC25A5	7.687502468	Tumor_G1
2000	UCP2	8.711898841	Tumor_G1
2001	SOD2	5.733044392	Tumor_G1
2002	NOX4	2.099745116	Tumor_G1
2003	DUOX1	6.647721707	Tumor_G1
2004	DUOXA1	6.325342135	Tumor_G1
2005	DUOX2	6.95735664	Tumor_G1
2006	DUOXA2	4.419156601	Tumor_G1
2007	NDUFA1	7.87377226	Tumor_G1
2008	NDUFA2	6.646209482	Tumor_G1
2009	NDUFA8	6.314837109	Tumor_G1
2010	NDUFB1	5.575783192	Tumor_G1
2011	NDUFB10	7.186150979	Tumor_G1
2012	NDUFB2	6.26990898	Tumor_G1
2013	NDUFB3	6.357609663	Tumor_G1
2014	NDUFB7	8.138671033	Tumor_G1
2015	CYC1	6.727596245	Tumor_G1
2016	ETFB	4.279050718	Tumor_G1
2017	UQCR10	7.336699081	Tumor_G1
2018	UQCRQ	7.018969477	Tumor_G1
2019	UQCC3	3.841281546	Tumor_G1
2020	COX15	4.865823088	Tumor_G1
2021	COX5A	7.06808841	Tumor_G1
2022	COX6B1	8.652623107	Tumor_G1
2023	COX6C	6.25510402	Tumor_G1
2024	COX7A2	6.846560992	Tumor_G1
2025	COX7B	6.101843018	Tumor_G1
2026	COX8A	9.023245665	Tumor_G1
2027	SCO1	3.428100547	Tumor_G1
2028	SURF1	5.664746481	Tumor_G1
2029	ATP6V1E1	7.272999921	Tumor_G1
2030	DLAT	5.354548286	Tumor_G1
2031	PDC	0.108231192	Tumor_G1
2032	SUCLG2	6.506220646	Tumor_G1
2033	SLC25A5	8.80879014	Tumor_G1
2034	UCP2	8.249485	Tumor_G1
2035	SOD2	6.253015049	Tumor_G1
2036	NOX4	1.646378609	Tumor_G1
2037	DUOX1	5.127816438	Tumor_G1
2038	DUOXA1	4.824438316	Tumor_G1

2039	DUOX2	4.050220858	Tumor_G1
2040	DUOXA2	1.955875353	Tumor_G1
2041	NDUFA1	7.718047912	Tumor_G1
2042	NDUFA2	6.395194137	Tumor_G1
2043	NDUFA8	6.685151818	Tumor_G1
2044	NDUFB1	5.13690861	Tumor_G1
2045	NDUFB10	7.271828315	Tumor_G1
2046	NDUFB2	6.022271347	Tumor_G1
2047	NDUFB3	6.032389262	Tumor_G1
2048	NDUFB7	8.451660407	Tumor_G1
2049	CYC1	6.376653737	Tumor_G1
2050	ETFB	4.758206833	Tumor_G1
2051	UQCR10	7.34703434	Tumor_G1
2052	UQCRQ	6.926512574	Tumor_G1
2053	UQCC3	3.752659135	Tumor_G1
2054	COX15	4.384953898	Tumor_G1
2055	COX5A	6.774927266	Tumor_G1
2056	COX6B1	8.512279316	Tumor_G1
2057	COX6C	5.919639892	Tumor_G1
2058	COX7A2	6.440279611	Tumor_G1
2059	COX7B	5.743874371	Tumor_G1
2060	COX8A	9.168353233	Tumor_G1
2061	SCO1	2.824280584	Tumor_G1
2062	SURF1	6.248683585	Tumor_G1
2063	ATP6V1E1	7.121645966	Tumor_G1
2064	DLAT	4.615826366	Tumor_G1
2065	PDC	0.161239648	Tumor_G1
2066	SUCLG2	5.708797267	Tumor_G1
2067	SLC25A5	7.954689181	Tumor_G1
2068	UCP2	8.25325933	Tumor_G1
2069	SOD2	5.502130401	Tumor_G1
2070	NOX4	3.093807801	Tumor_G1
2071	DUOX1	5.179913784	Tumor_G1
2072	DUOXA1	5.694581456	Tumor_G1
2073	DUOX2	4.88805187	Tumor_G1
2074	DUOXA2	3.133249365	Tumor_G1
2075	NDUFA1	7.970926389	Tumor_G1
2076	NDUFA2	6.956234833	Tumor_G1
2077	NDUFA8	7.128139369	Tumor_G1
2078	NDUFB1	6.012677446	Tumor_G1

2079	NDUFB10	7.502206295	Tumor_G1
2080	NDUFB2	6.574758539	Tumor_G1
2081	NDUFB3	6.464791141	Tumor_G1
2082	NDUFB7	8.702185675	Tumor_G1
2083	CYC1	6.374867242	Tumor_G1
2084	ETFB	4.92109116	Tumor_G1
2085	UQCR10	7.834586936	Tumor_G1
2086	UQCRQ	7.258120344	Tumor_G1
2087	UQCC3	4.054185969	Tumor_G1
2088	COX15	4.405210298	Tumor_G1
2089	COX5A	6.914772327	Tumor_G1
2090	COX6B1	8.975997456	Tumor_G1
2091	COX6C	6.637510417	Tumor_G1
2092	COX7A2	6.562151329	Tumor_G1
2093	COX7B	6.314601984	Tumor_G1
2094	COX8A	9.195001044	Tumor_G1
2095	SCO1	3.04331729	Tumor_G1
2096	SURF1	6.473741027	Tumor_G1
2097	ATP6V1E1	7.061612489	Tumor_G1
2098	DLAT	4.435877585	Tumor_G1
2099	PDC	0.048141387	Tumor_G1
2100	SUCLG2	5.567429472	Tumor_G1
2101	SLC25A5	7.456329392	Tumor_G1
2102	UCP2	8.175665229	Tumor_G1
2103	SOD2	5.910676871	Tumor_G1
2104	NOX4	2.511721616	Tumor_G1
2105	DUOX1	5.110774569	Tumor_G1
2106	DUOXA1	5.674890035	Tumor_G1
2107	DUOX2	4.388879366	Tumor_G1
2108	DUOXA2	3.6954052	Tumor_G1
2109	NDUFA1	7.984762014	Tumor_G1
2110	NDUFA2	6.607668619	Tumor_G1
2111	NDUFA8	6.654044271	Tumor_G1
2112	NDUFB1	5.620367801	Tumor_G1
2113	NDUFB10	7.291192724	Tumor_G1
2114	NDUFB2	6.279372075	Tumor_G1
2115	NDUFB3	6.295748042	Tumor_G1
2116	NDUFB7	8.203732639	Tumor_G1
2117	CYC1	6.549292844	Tumor_G1
2118	ETFB	4.52943662	Tumor_G1

2119	UQCR10	7.334203699	Tumor_G1
2120	UQCRQ	7.27526183	Tumor_G1
2121	UQCC3	3.999712256	Tumor_G1
2122	COX15	4.425903653	Tumor_G1
2123	COX5A	7.303773189	Tumor_G1
2124	COX6B1	8.6462533	Tumor_G1
2125	COX6C	6.166183897	Tumor_G1
2126	COX7A2	6.963473075	Tumor_G1
2127	COX7B	6.133261551	Tumor_G1
2128	COX8A	9.331434594	Tumor_G1
2129	SCO1	2.87850711	Tumor_G1
2130	SURF1	5.914811056	Tumor_G1
2131	ATP6V1E1	6.753362777	Tumor_G1
2132	DLAT	4.83284798	Tumor_G1
2133	PDC	0.170070058	Tumor_G1
2134	SUCLG2	5.781802798	Tumor_G1
2135	SLC25A5	8.359566857	Tumor_G1
2136	UCP2	8.104739917	Tumor_G1
2137	SOD2	6.593392489	Tumor_G1
2138	NOX4	2.658340834	Tumor_G1
2139	DUOX1	5.231880243	Tumor_G1
2140	DUOXA1	5.543873225	Tumor_G1
2141	DUOX2	4.27827833	Tumor_G1
2142	DUOXA2	2.488882038	Tumor_G1
2143	NDUFA1	8.005221234	Tumor_G1
2144	NDUFA2	6.56793902	Tumor_G1
2145	NDUFA8	7.062795018	Tumor_G1
2146	NDUFB1	5.621776971	Tumor_G1
2147	NDUFB10	7.529008723	Tumor_G1
2148	NDUFB2	6.137166922	Tumor_G1
2149	NDUFB3	6.220914439	Tumor_G1
2150	NDUFB7	8.438677162	Tumor_G1
2151	CYC1	6.800878879	Tumor_G1
2152	ETFB	4.706643599	Tumor_G1
2153	UQCR10	7.339113871	Tumor_G1
2154	UQCRQ	7.002780565	Tumor_G1
2155	UQCC3	4.337484897	Tumor_G1
2156	COX15	4.880693446	Tumor_G1
2157	COX5A	7.251620355	Tumor_G1
2158	COX6B1	8.555993512	Tumor_G1

2159	COX6C	6.364570594	Tumor_G1
2160	COX7A2	6.773293414	Tumor_G1
2161	COX7B	5.862117123	Tumor_G1
2162	COX8A	9.258364953	Tumor_G1
2163	SCO1	3.362523432	Tumor_G1
2164	SURF1	5.973760046	Tumor_G1
2165	ATP6V1E1	6.983798377	Tumor_G1
2166	DLAT	5.014717243	Tumor_G1
2167	PDC	0.054316447	Tumor_G1
2168	SUCLG2	5.981778533	Tumor_G1
2169	SLC25A5	8.179020173	Tumor_G1
2170	UCP2	7.38417953	Tumor_G1
2171	SOD2	5.696455008	Tumor_G1
2172	NOX4	3.281306164	Tumor_G1
2173	DUOX1	5.453804955	Tumor_G1
2174	DUOXA1	5.950897188	Tumor_G1
2175	DUOX2	5.579955826	Tumor_G1
2176	DUOXA2	3.960064784	Tumor_G1
2177	NDUFA1	7.72277844	Tumor_G1
2178	NDUFA2	6.312085224	Tumor_G1
2179	NDUFA8	6.886009491	Tumor_G1
2180	NDUFB1	5.336201621	Tumor_G1
2181	NDUFB10	7.152640032	Tumor_G1
2182	NDUFB2	6.114825851	Tumor_G1
2183	NDUFB3	6.184779444	Tumor_G1
2184	NDUFB7	8.261665021	Tumor_G1
2185	CYC1	6.606818299	Tumor_G1
2186	ETFB	4.643931867	Tumor_G1
2187	UQCR10	7.24483967	Tumor_G1
2188	UQCRQ	6.782728589	Tumor_G1
2189	UQCC3	3.952606265	Tumor_G1
2190	COX15	4.877174813	Tumor_G1
2191	COX5A	6.757229618	Tumor_G1
2192	COX6B1	8.344431676	Tumor_G1
2193	COX6C	6.050824634	Tumor_G1
2194	COX7A2	6.595328014	Tumor_G1
2195	COX7B	5.850962932	Tumor_G1
2196	COX8A	8.960639478	Tumor_G1
2197	SCO1	3.497780317	Tumor_G1
2198	SURF1	5.802151298	Tumor_G1

2199	ATP6V1E1	7.056858404	Tumor_G1
2200	DLAT	5.221672886	Tumor_G1
2201	PDC	0.103137179	Tumor_G1
2202	SUCLG2	6.057592617	Tumor_G1
2203	SLC25A5	8.087253844	Tumor_G1
2204	UCP2	7.070151961	Tumor_G1
2205	SOD2	5.372277156	Tumor_G1
2206	NOX4	3.43175991	Tumor_G1
2207	DUOX1	6.686845426	Tumor_G1
2208	DUOXA1	6.754204679	Tumor_G1
2209	DUOX2	6.552697449	Tumor_G1
2210	DUOXA2	4.603559338	Tumor_G1
2211	NDUFA1	7.899765757	Tumor_G1
2212	NDUFA2	6.713562689	Tumor_G1
2213	NDUFA8	6.690264112	Tumor_G1
2214	NDUFB1	5.332555896	Tumor_G1
2215	NDUFB10	7.168903984	Tumor_G1
2216	NDUFB2	6.409254201	Tumor_G1
2217	NDUFB3	6.105692589	Tumor_G1
2218	NDUFB7	8.165778129	Tumor_G1
2219	CYC1	6.739859	Tumor_G1
2220	ETFB	4.717422758	Tumor_G1
2221	UQCR10	7.353914555	Tumor_G1
2222	UQCRQ	6.768018473	Tumor_G1
2223	UQCC3	4.024587765	Tumor_G1
2224	COX15	4.734080977	Tumor_G1
2225	COX5A	6.787830705	Tumor_G1
2226	COX6B1	8.351498564	Tumor_G1
2227	COX6C	6.165095341	Tumor_G1
2228	COX7A2	6.614260062	Tumor_G1
2229	COX7B	5.741288225	Tumor_G1
2230	COX8A	9.278711668	Tumor_G1
2231	SCO1	3.078064228	Tumor_G1
2232	SURF1	5.660821768	Tumor_G1
2233	ATP6V1E1	7.170727603	Tumor_G1
2234	DLAT	4.879187122	Tumor_G1
2235	PDC	0.345793962	Tumor_G1
2236	SUCLG2	6.475208525	Tumor_G1
2237	SLC25A5	8.058893792	Tumor_G1
2238	UCP2	8.212003908	Tumor_G1

2239	SOD2	5.147486752	Tumor_G1
2240	NOX4	1.335149851	Tumor_G1
2241	DUOX1	6.093780676	Tumor_G1
2242	DUOXA1	6.195352884	Tumor_G1
2243	DUOX2	5.292891724	Tumor_G1
2244	DUOXA2	3.31205661	Tumor_G1
2245	NDUFA1	7.800414967	Tumor_G1
2246	NDUFA2	6.501832545	Tumor_G1
2247	NDUFA8	6.6837757	Tumor_G1
2248	NDUFB1	5.364553217	Tumor_G1
2249	NDUFB10	7.222037779	Tumor_G1
2250	NDUFB2	6.253962711	Tumor_G1
2251	NDUFB3	6.110710187	Tumor_G1
2252	NDUFB7	8.06192073	Tumor_G1
2253	CYC1	6.375736093	Tumor_G1
2254	ETFB	4.233195918	Tumor_G1
2255	UQCR10	7.174873489	Tumor_G1
2256	UQCRQ	7.045813957	Tumor_G1
2257	UQCC3	3.912614818	Tumor_G1
2258	COX15	4.070673555	Tumor_G1
2259	COX5A	7.155323063	Tumor_G1
2260	COX6B1	8.363308475	Tumor_G1
2261	COX6C	5.798582824	Tumor_G1
2262	COX7A2	6.658469083	Tumor_G1
2263	COX7B	5.762726144	Tumor_G1
2264	COX8A	8.961331985	Tumor_G1
2265	SCO1	2.818770071	Tumor_G1
2266	SURF1	5.994196907	Tumor_G1
2267	ATP6V1E1	6.836082334	Tumor_G1
2268	DLAT	4.751253459	Tumor_G1
2269	PDC	0.122140213	Tumor_G1
2270	SUCLG2	5.505334012	Tumor_G1
2271	SLC25A5	7.72643073	Tumor_G1
2272	UCP2	7.886365883	Tumor_G1
2273	SOD2	5.958076578	Tumor_G1
2274	NOX4	2.10833615	Tumor_G1
2275	DUOX1	4.842883134	Tumor_G1
2276	DUOXA1	5.717821861	Tumor_G1
2277	DUOX2	5.011347193	Tumor_G1
2278	DUOXA2	3.563544171	Tumor_G1

2279	NDUFA1	7.710314542	Tumor_G1
2280	NDUFA2	6.39737676	Tumor_G1
2281	NDUFA8	6.857403902	Tumor_G1
2282	NDUFB1	5.366057851	Tumor_G1
2283	NDUFB10	7.314286543	Tumor_G1
2284	NDUFB2	6.169944236	Tumor_G1
2285	NDUFB3	6.103187765	Tumor_G1
2286	NDUFB7	8.446408243	Tumor_G1
2287	CYC1	6.685915131	Tumor_G1
2288	ETFB	4.612433071	Tumor_G1
2289	UQCR10	7.278144017	Tumor_G1
2290	UQCRQ	6.927023072	Tumor_G1
2291	UQCC3	4.061329872	Tumor_G1
2292	COX15	4.691463706	Tumor_G1
2293	COX5A	6.788545798	Tumor_G1
2294	COX6B1	8.287497301	Tumor_G1
2295	COX6C	6.296082795	Tumor_G1
2296	COX7A2	6.584644761	Tumor_G1
2297	COX7B	5.655923299	Tumor_G1
2298	COX8A	9.138113343	Tumor_G1
2299	SCO1	3.084630212	Tumor_G1
2300	SURF1	5.891034817	Tumor_G1
2301	ATP6V1E1	7.009235586	Tumor_G1
2302	DLAT	4.783309946	Tumor_G1
2303	PDC	0.387085181	Tumor_G1
2304	SUCLG2	5.730596804	Tumor_G1
2305	SLC25A5	7.944223912	Tumor_G1
2306	UCP2	7.793007779	Tumor_G1
2307	SOD2	5.600655815	Tumor_G1
2308	NOX4	2.89322402	Tumor_G1
2309	DUOX1	6.480570113	Tumor_G1
2310	DUOXA1	6.681415771	Tumor_G1
2311	DUOX2	6.154594301	Tumor_G1
2312	DUOXA2	4.454246846	Tumor_G1
2313	NDUFA1	7.705238818	Tumor_G1
2314	NDUFA2	6.596188947	Tumor_G1
2315	NDUFA8	6.673020461	Tumor_G1
2316	NDUFB1	5.227931841	Tumor_G1
2317	NDUFB10	7.12901027	Tumor_G1
2318	NDUFB2	6.365294393	Tumor_G1

2319	NDUFB3	5.783684407	Tumor_G1
2320	NDUFB7	8.074512292	Tumor_G1
2321	CYC1	6.541359582	Tumor_G1
2322	ETFB	4.155020396	Tumor_G1
2323	UQCR10	7.339148267	Tumor_G1
2324	UQCRQ	6.873996481	Tumor_G1
2325	UQCC3	3.998168021	Tumor_G1
2326	COX15	4.254713212	Tumor_G1
2327	COX5A	6.865924983	Tumor_G1
2328	COX6B1	8.39360667	Tumor_G1
2329	COX6C	6.240142664	Tumor_G1
2330	COX7A2	6.673716714	Tumor_G1
2331	COX7B	5.639891919	Tumor_G1
2332	COX8A	9.179901504	Tumor_G1
2333	SCO1	2.708961196	Tumor_G1
2334	SURF1	5.608540234	Tumor_G1
2335	ATP6V1E1	6.955867069	Tumor_G1
2336	DLAT	4.416198589	Tumor_G1
2337	PDC	0.093425188	Tumor_G1
2338	SUCLG2	5.800466601	Tumor_G1
2339	SLC25A5	8.0604853	Tumor_G1
2340	UCP2	7.587062457	Tumor_G1
2341	SOD2	4.324388131	Tumor_G1
2342	NOX4	1.663334958	Tumor_G1
2343	DUOX1	5.375088094	Tumor_G1
2344	DUOXA1	5.515811856	Tumor_G1
2345	DUOX2	4.8446262	Tumor_G1
2346	DUOXA2	3.049261661	Tumor_G1
2347	NDUFA1	7.017966116	Tumor_G1
2348	NDUFA2	6.163412347	Tumor_G1
2349	NDUFA8	6.384255547	Tumor_G1
2350	NDUFB1	4.510136725	Tumor_G1
2351	NDUFB10	6.848247031	Tumor_G1
2352	NDUFB2	5.841785807	Tumor_G1
2353	NDUFB3	5.434081688	Tumor_G1
2354	NDUFB7	7.897718932	Tumor_G1
2355	CYC1	6.603850512	Tumor_G1
2356	ETFB	4.586747236	Tumor_G1
2357	UQCR10	6.8609067	Tumor_G1
2358	UQCRQ	6.509199253	Tumor_G1

2359	UQCC3	3.735088622	Tumor_G1
2360	COX15	4.708318672	Tumor_G1
2361	COX5A	6.386416677	Tumor_G1
2362	COX6B1	8.231199984	Tumor_G1
2363	COX6C	5.789894864	Tumor_G1
2364	COX7A2	6.135958358	Tumor_G1
2365	COX7B	5.415084946	Tumor_G1
2366	COX8A	8.900391113	Tumor_G1
2367	SCO1	2.767059341	Tumor_G1
2368	SURF1	5.778706969	Tumor_G1
2369	ATP6V1E1	6.769302398	Tumor_G1
2370	DLAT	4.828310829	Tumor_G1
2371	PDC	0.05141007	Tumor_G1
2372	SUCLG2	6.15265629	Tumor_G1
2373	SLC25A5	8.19301228	Tumor_G1
2374	UCP2	8.276096123	Tumor_G1
2375	SOD2	4.87538643	Tumor_G1
2376	NOX4	2.543208936	Tumor_G1
2377	DUOX1	5.708171319	Tumor_G1
2378	DUOXA1	5.593440002	Tumor_G1
2379	DUOX2	5.731903927	Tumor_G1
2380	DUOXA2	3.383160694	Tumor_G1
2381	NDUFA1	7.401557894	Tumor_G1
2382	NDUFA2	6.201967531	Tumor_G1
2383	NDUFA8	6.478372477	Tumor_G1
2384	NDUFB1	4.927029398	Tumor_G1
2385	NDUFB10	6.895653816	Tumor_G1
2386	NDUFB2	5.758062563	Tumor_G1
2387	NDUFB3	5.905190029	Tumor_G1
2388	NDUFB7	7.651911662	Tumor_G1
2389	CYC1	5.986554922	Tumor_G1
2390	ETFB	3.922045802	Tumor_G1
2391	UQCR10	7.017344823	Tumor_G1
2392	UQCRQ	6.638636974	Tumor_G1
2393	UQCC3	3.382495985	Tumor_G1
2394	COX15	4.315469729	Tumor_G1
2395	COX5A	6.596803672	Tumor_G1
2396	COX6B1	8.113711966	Tumor_G1
2397	COX6C	5.86302626	Tumor_G1
2398	COX7A2	6.27729092	Tumor_G1

2399	COX7B	5.502655354	Tumor_G1
2400	COX8A	8.675725249	Tumor_G1
2401	SCO1	2.691153785	Tumor_G1
2402	SURF1	5.448892697	Tumor_G1
2403	ATP6V1E1	6.883243414	Tumor_G1
2404	DLAT	5.012912662	Tumor_G1
2405	PDC	0	Tumor_G1
2406	SUCLG2	6.05128174	Tumor_G1
2407	SLC25A5	7.633466599	Tumor_G1
2408	UCP2	7.291392917	Tumor_G1
2409	SOD2	6.565109782	Tumor_G1
2410	NOX4	3.092093202	Tumor_G1
2411	DUOX1	5.513125917	Tumor_G1
2412	DUOXA1	5.388520295	Tumor_G1
2413	DUOX2	5.045788199	Tumor_G1
2414	DUOXA2	2.717172176	Tumor_G1
2415	NDUFA1	7.779441606	Tumor_G1
2416	NDUFA2	6.449603501	Tumor_G1
2417	NDUFA8	6.850677228	Tumor_G1
2418	NDUFB1	5.301191044	Tumor_G1
2419	NDUFB10	7.273183328	Tumor_G1
2420	NDUFB2	6.203722339	Tumor_G1
2421	NDUFB3	6.001469116	Tumor_G1
2422	NDUFB7	8.002502397	Tumor_G1
2423	CYC1	6.815470048	Tumor_G1
2424	ETFB	4.791101575	Tumor_G1
2425	UQCR10	7.368287045	Tumor_G1
2426	UQCRQ	6.733629567	Tumor_G1
2427	UQCC3	3.585419314	Tumor_G1
2428	COX15	5.026189301	Tumor_G1
2429	COX5A	6.907967031	Tumor_G1
2430	COX6B1	8.411747964	Tumor_G1
2431	COX6C	6.185484831	Tumor_G1
2432	COX7A2	6.64209383	Tumor_G1
2433	COX7B	6.173919094	Tumor_G1
2434	COX8A	9.538794887	Tumor_G1
2435	SCO1	3.550675344	Tumor_G1
2436	SURF1	6.412751891	Tumor_G1
2437	ATP6V1E1	6.784101767	Tumor_G1
2438	DLAT	5.223547573	Tumor_G1

2439	PDC	0.223688933	Tumor_G1
2440	SUCLG2	5.985010503	Tumor_G1
2441	SLC25A5	8.563166312	Tumor_G1
2442	UCP2	7.389687027	Tumor_G1
2443	SOD2	6.423075057	Tumor_G1
2444	NOX4	1.990866883	Tumor_G1
2445	DUOX1	5.86272406	Tumor_G1
2446	DUOXA1	6.098436734	Tumor_G1
2447	DUOX2	6.051436174	Tumor_G1
2448	DUOXA2	2.953929348	Tumor_G1
2449	NDUFA1	7.385345298	Tumor_G1
2450	NDUFA2	6.271713796	Tumor_G1
2451	NDUFA8	6.928346126	Tumor_G1
2452	NDUFB1	5.208273888	Tumor_G1
2453	NDUFB10	7.428466194	Tumor_G1
2454	NDUFB2	6.472584737	Tumor_G1
2455	NDUFB3	5.384854376	Tumor_G1
2456	NDUFB7	8.361560908	Tumor_G1
2457	CYC1	7.293692311	Tumor_G1
2458	ETFB	4.762868827	Tumor_G1
2459	UQCR10	7.117789887	Tumor_G1
2460	UQCRQ	6.838864208	Tumor_G1
2461	UQCC3	4.494745163	Tumor_G1
2462	COX15	5.13353249	Tumor_G1
2463	COX5A	7.187504417	Tumor_G1
2464	COX6B1	8.45840404	Tumor_G1
2465	COX6C	6.206403069	Tumor_G1
2466	COX7A2	6.442557388	Tumor_G1
2467	COX7B	5.901934799	Tumor_G1
2468	COX8A	9.40880123	Tumor_G1
2469	SCO1	3.422479856	Tumor_G1
2470	SURF1	6.598758811	Tumor_G1
2471	ATP6V1E1	6.838210959	Tumor_G1
2472	DLAT	4.946491543	Tumor_G1
2473	PDC	0	Tumor_G1
2474	SUCLG2	6.639092506	Tumor_G1
2475	SLC25A5	9.005569873	Tumor_G1
2476	UCP2	6.506269559	Tumor_G1
2477	SOD2	5.018604692	Tumor_G1
2478	NOX4	1.377403222	Tumor_G1

2479	DUOX1	5.741160701	Tumor_G1
2480	DUOXA1	6.002060131	Tumor_G1
2481	DUOX2	6.575231554	Tumor_G1
2482	DUOXA2	4.150109186	Tumor_G1
2483	NDUFA1	7.526724628	Tumor_G1
2484	NDUFA2	5.810163624	Tumor_G1
2485	NDUFA8	6.753129758	Tumor_G1
2486	NDUFB1	5.020115393	Tumor_G1
2487	NDUFB10	7.176682768	Tumor_G1
2488	NDUFB2	5.678300543	Tumor_G1
2489	NDUFB3	6.047329985	Tumor_G1
2490	NDUFB7	7.943849937	Tumor_G1
2491	CYC1	6.33882935	Tumor_G1
2492	ETFB	4.545534901	Tumor_G1
2493	UQCR10	7.575463469	Tumor_G1
2494	UQCRQ	7.000988955	Tumor_G1
2495	UQCC3	3.008129031	Tumor_G1
2496	COX15	4.545947107	Tumor_G1
2497	COX5A	6.963369319	Tumor_G1
2498	COX6B1	8.504345434	Tumor_G1
2499	COX6C	5.946011819	Tumor_G1
2500	COX7A2	6.8032038	Tumor_G1
2501	COX7B	6.259929173	Tumor_G1
2502	COX8A	8.796351249	Tumor_G1
2503	SCO1	2.764221725	Tumor_G1
2504	SURF1	5.299191857	Tumor_G1
2505	ATP6V1E1	7.333531981	Tumor_G1
2506	DLAT	5.399902842	Tumor_G1
2507	PDC	0.170889799	Tumor_G1
2508	SUCLG2	6.52047178	Tumor_G1
2509	SLC25A5	8.447200509	Tumor_G1
2510	UCP2	8.245566134	Tumor_G1
2511	SOD2	5.064045897	Tumor_G1
2512	NOX4	3.465339994	Tumor_G1
2513	DUOX1	5.116313499	Tumor_G1
2514	DUOXA1	5.719536035	Tumor_G1
2515	DUOX2	5.088464139	Tumor_G1
2516	DUOXA2	2.168076565	Tumor_G1
2517	NDUFA1	7.344320086	Tumor_G1
2518	NDUFA2	5.946222618	Tumor_G1

2519	NDUFA8	6.739860395	Tumor_G1
2520	NDUFB1	5.024303928	Tumor_G1
2521	NDUFB10	7.164050359	Tumor_G1
2522	NDUFB2	5.955390717	Tumor_G1
2523	NDUFB3	5.973773524	Tumor_G1
2524	NDUFB7	7.72896463	Tumor_G1
2525	CYC1	6.268617645	Tumor_G1
2526	ETFB	4.436296675	Tumor_G1
2527	UQCR10	7.051610951	Tumor_G1
2528	UQCRQ	6.658084573	Tumor_G1
2529	UQCC3	3.794793932	Tumor_G1
2530	COX15	4.886220536	Tumor_G1
2531	COX5A	6.630676794	Tumor_G1
2532	COX6B1	8.000125645	Tumor_G1
2533	COX6C	5.845020464	Tumor_G1
2534	COX7A2	6.273563023	Tumor_G1
2535	COX7B	5.563241609	Tumor_G1
2536	COX8A	8.591274241	Tumor_G1
2537	SCO1	3.412286739	Tumor_G1
2538	SURF1	5.773389988	Tumor_G1
2539	ATP6V1E1	6.755016069	Tumor_G1
2540	DLAT	5.263645272	Tumor_G1
2541	PDC	0.236886214	Tumor_G1
2542	SUCLG2	6.076424744	Tumor_G1
2543	SLC25A5	7.850128231	Tumor_G1
2544	UCP2	7.738685311	Tumor_G1
2545	SOD2	5.618836576	Tumor_G1
2546	NOX4	2.729976526	Tumor_G1
2547	DUOX1	6.01901711	Tumor_G1
2548	DUOXA1	6.177073901	Tumor_G1
2549	DUOX2	6.297340902	Tumor_G1
2550	DUOXA2	4.346426766	Tumor_G1
2551	NDUFA1	7.330875288	Tumor_G1
2552	NDUFA2	5.946186517	Tumor_G1
2553	NDUFA8	6.582778946	Tumor_G1
2554	NDUFB1	5.148168371	Tumor_G1
2555	NDUFB10	7.006704745	Tumor_G1
2556	NDUFB2	6.001683434	Tumor_G1
2557	NDUFB3	5.971426232	Tumor_G1
2558	NDUFB7	7.715573867	Tumor_G1

2559	CYC1	6.498398556	Tumor_G1
2560	ETFB	4.126631616	Tumor_G1
2561	UQCR10	6.996384945	Tumor_G1
2562	UQCRQ	6.593428034	Tumor_G1
2563	UQCC3	3.523648167	Tumor_G1
2564	COX15	4.878974164	Tumor_G1
2565	COX5A	6.995664829	Tumor_G1
2566	COX6B1	8.168012235	Tumor_G1
2567	COX6C	5.983105619	Tumor_G1
2568	COX7A2	6.55386283	Tumor_G1
2569	COX7B	5.767059111	Tumor_G1
2570	COX8A	8.854471311	Tumor_G1
2571	SCO1	3.225003433	Tumor_G1
2572	SURF1	5.881430124	Tumor_G1
2573	ATP6V1E1	7.115530967	Tumor_G1
2574	DLAT	5.475793662	Tumor_G1
2575	PDC	0.110160576	Tumor_G1
2576	SUCLG2	6.53400743	Tumor_G1
2577	SLC25A5	8.707432575	Tumor_G1
2578	UCP2	8.339014461	Tumor_G1
2579	SOD2	7.003326552	Tumor_G1
2580	NOX4	3.295590339	Tumor_G1
2581	DUOX1	5.319823027	Tumor_G1
2582	DUOXA1	5.187812565	Tumor_G1
2583	DUOX2	5.524548407	Tumor_G1
2584	DUOXA2	2.956069188	Tumor_G1
2585	NDUFA1	7.423078547	Tumor_G1
2586	NDUFA2	6.213442344	Tumor_G1
2587	NDUFA8	6.489652225	Tumor_G1
2588	NDUFB1	5.095274356	Tumor_G1
2589	NDUFB10	6.763162858	Tumor_G1
2590	NDUFB2	5.745723865	Tumor_G1
2591	NDUFB3	5.836397134	Tumor_G1
2592	NDUFB7	7.416019271	Tumor_G1
2593	CYC1	6.367973463	Tumor_G1
2594	ETFB	4.384488854	Tumor_G1
2595	UQCR10	6.939237879	Tumor_G1
2596	UQCRQ	6.453788085	Tumor_G1
2597	UQCC3	3.688694873	Tumor_G1
2598	COX15	4.7510378	Tumor_G1

2599	COX5A	6.655480734	Tumor_G1
2600	COX6B1	8.236542913	Tumor_G1
2601	COX6C	5.819066145	Tumor_G1
2602	COX7A2	6.116371396	Tumor_G1
2603	COX7B	5.672942072	Tumor_G1
2604	COX8A	8.94559279	Tumor_G1
2605	SCO1	3.144195987	Tumor_G1
2606	SURF1	6.000165223	Tumor_G1
2607	ATP6V1E1	6.786038901	Tumor_G1
2608	DLAT	4.622744515	Tumor_G1
2609	PDC	0	Tumor_G1
2610	SUCLG2	5.792245395	Tumor_G1
2611	SLC25A5	8.594167676	Tumor_G1
2612	UCP2	8.40197727	Tumor_G1
2613	SOD2	6.699984217	Tumor_G1
2614	NOX4	2.835066639	Tumor_G1
2615	DUOX1	5.649205575	Tumor_G1
2616	DUOXA1	5.507835244	Tumor_G1
2617	DUOX2	5.672703591	Tumor_G1
2618	DUOXA2	2.670550235	Tumor_G1
2619	NDUFA1	7.746343133	Tumor_G1
2620	NDUFA2	5.924923536	Tumor_G1
2621	NDUFA8	6.865313493	Tumor_G1
2622	NDUFB1	5.528102714	Tumor_G1
2623	NDUFB10	7.095395817	Tumor_G1
2624	NDUFB2	5.191580769	Tumor_G1
2625	NDUFB3	5.884751023	Tumor_G1
2626	NDUFB7	8.300125668	Tumor_G1
2627	CYC1	5.831646448	Tumor_G1
2628	ETFB	4.365628042	Tumor_G1
2629	UQCR10	6.83320082	Tumor_G1
2630	UQCRQ	6.665846545	Tumor_G1
2631	UQCC3	3.45689329	Tumor_G1
2632	COX15	4.180083365	Tumor_G1
2633	COX5A	6.592813311	Tumor_G1
2634	COX6B1	8.836173814	Tumor_G1
2635	COX6C	5.754876644	Tumor_G1
2636	COX7A2	6.765545333	Tumor_G1
2637	COX7B	6.032031927	Tumor_G1
2638	COX8A	8.281022132	Tumor_G1

2639	SCO1	2.765546232	Tumor_G1
2640	SURF1	5.886605759	Tumor_G1
2641	ATP6V1E1	5.736645423	Tumor_G1
2642	DLAT	4.440675445	Tumor_G1
2643	PDC	0.216856301	Tumor_G1
2644	SUCLG2	5.700815353	Tumor_G1
2645	SLC25A5	6.899761574	Tumor_G1
2646	UCP2	7.843503452	Tumor_G1
2647	SOD2	3.918502881	Tumor_G1
2648	NOX4	2.824794419	Tumor_G1
2649	DUOX1	5.84129958	Tumor_G1
2650	DUOXA1	6.456780059	Tumor_G1
2651	DUOX2	7.731101689	Tumor_G1
2652	DUOXA2	4.802482398	Tumor_G1
2653	NDUFA1	7.55663962	Tumor_G1
2654	NDUFA2	6.430065159	Tumor_G1
2655	NDUFA8	6.316079014	Tumor_G1
2656	NDUFB1	5.257923561	Tumor_G1
2657	NDUFB10	6.736781634	Tumor_G1
2658	NDUFB2	5.711028141	Tumor_G1
2659	NDUFB3	6.127548806	Tumor_G1
2660	NDUFB7	7.753800034	Tumor_G1
2661	CYC1	5.914697332	Tumor_G1
2662	ETFB	4.422091613	Tumor_G1
2663	UQCR10	6.994018075	Tumor_G1
2664	UQCRQ	6.948134563	Tumor_G1
2665	UQCC3	3.237218573	Tumor_G1
2666	COX15	4.298152714	Tumor_G1
2667	COX5A	6.965365893	Tumor_G1
2668	COX6B1	8.271166814	Tumor_G1
2669	COX6C	6.130132504	Tumor_G1
2670	COX7A2	6.464256204	Tumor_G1
2671	COX7B	5.737894092	Tumor_G1
2672	COX8A	8.394858233	Tumor_G1
2673	SCO1	2.300390487	Tumor_G1
2674	SURF1	5.780650682	Tumor_G1
2675	ATP6V1E1	6.916913392	Tumor_G1
2676	DLAT	4.828796124	Tumor_G1
2677	PDC	0.040519914	Tumor_G1
2678	SUCLG2	6.039789203	Tumor_G1

2679	SLC25A5	7.526737883	Tumor_G1
2680	UCP2	7.755588994	Tumor_G1
2681	SOD2	6.07646076	Tumor_G1
2682	NOX4	2.454115426	Tumor_G1
2683	DUOX1	4.440682908	Tumor_G1
2684	DUOXA1	5.099813524	Tumor_G1
2685	DUOX2	4.002609723	Tumor_G1
2686	DUOXA2	1.911779956	Tumor_G1
2687	NDUFA1	8.114768575	Tumor_G1
2688	NDUFA2	6.107793006	Tumor_G1
2689	NDUFA8	7.056211929	Tumor_G1
2690	NDUFB1	5.338773911	Tumor_G1
2691	NDUFB10	7.256070411	Tumor_G1
2692	NDUFB2	5.643100067	Tumor_G1
2693	NDUFB3	6.258848984	Tumor_G1
2694	NDUFB7	8.348750124	Tumor_G1
2695	CYC1	6.545081901	Tumor_G1
2696	ETFB	4.510936525	Tumor_G1
2697	UQCR10	7.804561472	Tumor_G1
2698	UQCRQ	6.743655307	Tumor_G1
2699	UQCC3	3.78365686	Tumor_G1
2700	COX15	5.100481076	Tumor_G1
2701	COX5A	6.261369242	Tumor_G1
2702	COX6B1	8.617097532	Tumor_G1
2703	COX6C	6.052161367	Tumor_G1
2704	COX7A2	6.79399622	Tumor_G1
2705	COX7B	6.338076899	Tumor_G1
2706	COX8A	8.923951819	Tumor_G1
2707	SCO1	4.456268107	Tumor_G1
2708	SURF1	5.583520624	Tumor_G1
2709	ATP6V1E1	6.487770007	Tumor_G1
2710	DLAT	4.886681737	Tumor_G1
2711	PDC	0.303570671	Tumor_G1
2712	SUCLG2	6.147835546	Tumor_G1
2713	SLC25A5	8.165215822	Tumor_G1
2714	UCP2	6.942314786	Tumor_G1
2715	SOD2	3.699224729	Tumor_G1
2716	NOX4	2.228554537	Tumor_G1
2717	DUOX1	5.905744386	Tumor_G1
2718	DUOXA1	5.522330123	Tumor_G1

2719	DUOX2	6.055587839	Tumor_G1
2720	DUOXA2	6.005246851	Tumor_G1
2721	NDUFA1	7.963132887	Tumor_G1
2722	NDUFA2	6.356127077	Tumor_G1
2723	NDUFA8	6.282337787	Tumor_G1
2724	NDUFB1	5.29710309	Tumor_G1
2725	NDUFB10	6.541922105	Tumor_G1
2726	NDUFB2	5.192955359	Tumor_G1
2727	NDUFB3	6.110368825	Tumor_G1
2728	NDUFB7	7.935192585	Tumor_G1
2729	CYC1	6.082754866	Tumor_G1
2730	ETFB	3.510986122	Tumor_G1
2731	UQCR10	6.991366	Tumor_G1
2732	UQCRQ	6.949635396	Tumor_G1
2733	UQCC3	2.675874913	Tumor_G1
2734	COX15	4.473859894	Tumor_G1
2735	COX5A	6.867876004	Tumor_G1
2736	COX6B1	8.615315552	Tumor_G1
2737	COX6C	5.810092828	Tumor_G1
2738	COX7A2	6.417630251	Tumor_G1
2739	COX7B	5.799931162	Tumor_G1
2740	COX8A	7.959697163	Tumor_G1
2741	SCO1	2.915448995	Tumor_G1
2742	SURF1	4.9193742	Tumor_G1
2743	ATP6V1E1	6.292060667	Tumor_G1
2744	DLAT	4.270749468	Tumor_G1
2745	PDC	0.177462423	Tumor_G1
2746	SUCLG2	5.660310827	Tumor_G1
2747	SLC25A5	7.996439447	Tumor_G1
2748	UCP2	7.115995707	Tumor_G1
2749	SOD2	5.255134521	Tumor_G1
2750	NOX4	2.981893742	Tumor_G1
2751	DUOX1	5.734344516	Tumor_G1
2752	DUOXA1	4.762427839	Tumor_G1
2753	DUOX2	6.992375752	Tumor_G1
2754	DUOXA2	4.722905255	Tumor_G1
2755	NDUFA1	7.915150846	Tumor_G1
2756	NDUFA2	5.803708794	Tumor_G1
2757	NDUFA8	6.741787607	Tumor_G1
2758	NDUFB1	5.629843492	Tumor_G1

2759	NDUFB10	6.980869361	Tumor_G1
2760	NDUFB2	5.668402839	Tumor_G1
2761	NDUFB3	6.443123101	Tumor_G1
2762	NDUFB7	7.796672163	Tumor_G1
2763	CYC1	5.973633831	Tumor_G1
2764	ETFB	4.493547725	Tumor_G1
2765	UQCR10	7.241799028	Tumor_G1
2766	UQCRQ	6.601158399	Tumor_G1
2767	UQCC3	3.369449819	Tumor_G1
2768	COX15	5.255686438	Tumor_G1
2769	COX5A	6.316994922	Tumor_G1
2770	COX6B1	8.335087284	Tumor_G1
2771	COX6C	5.919015063	Tumor_G1
2772	COX7A2	6.658690935	Tumor_G1
2773	COX7B	6.200101363	Tumor_G1
2774	COX8A	8.191595583	Tumor_G1
2775	SCO1	3.689367161	Tumor_G1
2776	SURF1	6.051154134	Tumor_G1
2777	ATP6V1E1	6.589695881	Tumor_G1
2778	DLAT	5.111108424	Tumor_G1
2779	PDC	0.1160961	Tumor_G1
2780	SUCLG2	5.896565369	Tumor_G1
2781	SLC25A5	7.483479586	Tumor_G1
2782	UCP2	8.644147645	Tumor_G1
2783	SOD2	5.064958859	Tumor_G1
2784	NOX4	2.663998331	Tumor_G1
2785	DUOX1	6.749935589	Tumor_G1
2786	DUOXA1	6.88890108	Tumor_G1
2787	DUOX2	7.647524529	Tumor_G1
2788	DUOXA2	5.252614972	Tumor_G1
2789	NDUFA1	7.911001379	Tumor_G1
2790	NDUFA2	6.522408484	Tumor_G1
2791	NDUFA8	6.707540218	Tumor_G1
2792	NDUFB1	5.443764051	Tumor_G1
2793	NDUFB10	7.226018373	Tumor_G1
2794	NDUFB2	6.360846065	Tumor_G1
2795	NDUFB3	6.343470061	Tumor_G1
2796	NDUFB7	8.337324443	Tumor_G1
2797	CYC1	6.506498168	Tumor_G1
2798	ETFB	4.477842636	Tumor_G1

2799	UQCR10	7.188408151	Tumor_G1
2800	UQCRQ	7.062252513	Tumor_G1
2801	UQCC3	3.777649971	Tumor_G1
2802	COX15	5.032301643	Tumor_G1
2803	COX5A	6.90255445	Tumor_G1
2804	COX6B1	8.442804397	Tumor_G1
2805	COX6C	6.138897162	Tumor_G1
2806	COX7A2	6.843820977	Tumor_G1
2807	COX7B	5.970566885	Tumor_G1
2808	COX8A	9.144524698	Tumor_G1
2809	SCO1	3.505874218	Tumor_G1
2810	SURF1	6.067186137	Tumor_G1
2811	ATP6V1E1	6.879730054	Tumor_G1
2812	DLAT	5.743320154	Tumor_G1
2813	PDC	0.40971353	Tumor_G1
2814	SUCLG2	6.265838437	Tumor_G1
2815	SLC25A5	8.020019621	Tumor_G1
2816	UCP2	7.904506048	Tumor_G1
2817	SOD2	5.602907517	Tumor_G1
2818	NOX4	3.549987749	Tumor_G1
2819	DUOX1	5.834686669	Tumor_G1
2820	DUOXA1	6.101387011	Tumor_G1
2821	DUOX2	7.019266444	Tumor_G1
2822	DUOXA2	4.741006184	Tumor_G1
2823	NDUFA1	7.846095269	Tumor_G1
2824	NDUFA2	6.404683647	Tumor_G1
2825	NDUFA8	6.871479641	Tumor_G1
2826	NDUFB1	5.431357571	Tumor_G1
2827	NDUFB10	7.042878819	Tumor_G1
2828	NDUFB2	6.736594397	Tumor_G1
2829	NDUFB3	6.453897251	Tumor_G1
2830	NDUFB7	8.070003657	Tumor_G1
2831	CYC1	6.556770678	Tumor_G1
2832	ETFB	4.392711465	Tumor_G1
2833	UQCR10	7.449609315	Tumor_G1
2834	UQCRQ	7.184160583	Tumor_G1
2835	UQCC3	3.739798651	Tumor_G1
2836	COX15	4.96764442	Tumor_G1
2837	COX5A	7.17814779	Tumor_G1
2838	COX6B1	8.433348168	Tumor_G1

2839	COX6C	6.29902431	Tumor_G1
2840	COX7A2	6.684803333	Tumor_G1
2841	COX7B	5.986339616	Tumor_G1
2842	COX8A	9.130637782	Tumor_G1
2843	SCO1	3.49368645	Tumor_G1
2844	SURF1	6.11207123	Tumor_G1
2845	ATP6V1E1	7.295012785	Tumor_G1
2846	DLAT	5.65356934	Tumor_G1
2847	PDC	0	Tumor_G1
2848	SUCLG2	6.605611938	Tumor_G1
2849	SLC25A5	8.561777192	Tumor_G1
2850	UCP2	8.034110121	Tumor_G1
2851	SOD2	5.578304893	Tumor_G1
2852	NOX4	1.026978104	Tumor_G1
2853	DUOX1	6.140035801	Tumor_G1
2854	DUOXA1	6.031003268	Tumor_G1
2855	DUOX2	6.001074304	Tumor_G1
2856	DUOXA2	3.927715299	Tumor_G1
2857	NDUFA1	7.705086621	Tumor_G1
2858	NDUFA2	6.47676721	Tumor_G1
2859	NDUFA8	6.577193284	Tumor_G1
2860	NDUFB1	5.603109623	Tumor_G1
2861	NDUFB10	7.250265363	Tumor_G1
2862	NDUFB2	6.271577112	Tumor_G1
2863	NDUFB3	6.129410227	Tumor_G1
2864	NDUFB7	8.194138811	Tumor_G1
2865	CYC1	6.387714078	Tumor_G1
2866	ETFB	4.300573119	Tumor_G1
2867	UQCR10	7.268735193	Tumor_G1
2868	UQCRQ	7.161535918	Tumor_G1
2869	UQCC3	3.760687129	Tumor_G1
2870	COX15	4.413649974	Tumor_G1
2871	COX5A	7.013968215	Tumor_G1
2872	COX6B1	8.341441358	Tumor_G1
2873	COX6C	6.183923411	Tumor_G1
2874	COX7A2	6.49770985	Tumor_G1
2875	COX7B	5.647426266	Tumor_G1
2876	COX8A	8.810346247	Tumor_G1
2877	SCO1	2.741579379	Tumor_G1
2878	SURF1	6.057794485	Tumor_G1

2879	ATP6V1E1	6.750580913	Tumor_G1
2880	DLAT	4.84513932	Tumor_G1
2881	PDC	0.228516721	Tumor_G1
2882	SUCLG2	6.053510892	Tumor_G1
2883	SLC25A5	7.855737771	Tumor_G1
2884	UCP2	7.539158071	Tumor_G1
2885	SOD2	5.804295278	Tumor_G1
2886	NOX4	2.899481897	Tumor_G1
2887	DUOX1	5.60475375	Tumor_G1
2888	DUOXA1	5.544681493	Tumor_G1
2889	DUOX2	5.946848022	Tumor_G1
2890	DUOXA2	3.850520313	Tumor_G1
2891	NDUFA1	7.877654332	Tumor_G1
2892	NDUFA2	6.395757566	Tumor_G1
2893	NDUFA8	6.663910673	Tumor_G1
2894	NDUFB1	5.240483208	Tumor_G1
2895	NDUFB10	7.242975281	Tumor_G1
2896	NDUFB2	6.027442367	Tumor_G1
2897	NDUFB3	6.370930726	Tumor_G1
2898	NDUFB7	8.257769527	Tumor_G1
2899	CYC1	6.607650168	Tumor_G1
2900	ETFB	4.734108993	Tumor_G1
2901	UQCR10	7.343373194	Tumor_G1
2902	UQCRQ	6.937232208	Tumor_G1
2903	UQCC3	3.924846239	Tumor_G1
2904	COX15	5.056388696	Tumor_G1
2905	COX5A	7.117009254	Tumor_G1
2906	COX6B1	8.259292386	Tumor_G1
2907	COX6C	6.192549079	Tumor_G1
2908	COX7A2	6.361775305	Tumor_G1
2909	COX7B	5.769042586	Tumor_G1
2910	COX8A	9.097478063	Tumor_G1
2911	SCO1	3.430147021	Tumor_G1
2912	SURF1	6.155877306	Tumor_G1
2913	ATP6V1E1	6.914313825	Tumor_G1
2914	DLAT	5.408146715	Tumor_G1
2915	PDC	0.11978372	Tumor_G1
2916	SUCLG2	6.384579853	Tumor_G1
2917	SLC25A5	8.060890769	Tumor_G1
2918	UCP2	8.429176648	Tumor_G1

2919	SOD2	5.74026618	Tumor_G1
2920	NOX4	3.377646795	Tumor_G1
2921	DUOX1	6.991928392	Tumor_G1
2922	DUOXA1	6.807124606	Tumor_G1
2923	DUOX2	7.163790982	Tumor_G1
2924	DUOXA2	4.524375354	Tumor_G1
2925	NDUFA1	7.660703569	Tumor_G1
2926	NDUFA2	6.585631172	Tumor_G1
2927	NDUFA8	6.735720019	Tumor_G1
2928	NDUFB1	5.373285065	Tumor_G1
2929	NDUFB10	7.101707038	Tumor_G1
2930	NDUFB2	6.496504951	Tumor_G1
2931	NDUFB3	6.342592605	Tumor_G1
2932	NDUFB7	8.424291703	Tumor_G1
2933	CYC1	6.687146152	Tumor_G1
2934	ETFB	4.698910706	Tumor_G1
2935	UQCR10	7.461848983	Tumor_G1
2936	UQCRQ	7.083461198	Tumor_G1
2937	UQCC3	3.914237535	Tumor_G1
2938	COX15	4.658544182	Tumor_G1
2939	COX5A	6.968376145	Tumor_G1
2940	COX6B1	8.447583318	Tumor_G1
2941	COX6C	6.17373633	Tumor_G1
2942	COX7A2	6.46809042	Tumor_G1
2943	COX7B	5.689555584	Tumor_G1
2944	COX8A	9.095263257	Tumor_G1
2945	SCO1	3.223635197	Tumor_G1
2946	SURF1	6.100665971	Tumor_G1
2947	ATP6V1E1	7.296326113	Tumor_G1
2948	DLAT	5.220827406	Tumor_G1
2949	PDC	0.289760805	Tumor_G1
2950	SUCLG2	6.378585848	Tumor_G1
2951	SLC25A5	8.001048143	Tumor_G1
2952	UCP2	8.514904296	Tumor_G1
2953	SOD2	6.07458776	Tumor_G1
2954	NOX4	2.824347198	Tumor_G1
2955	DUOX1	5.610763804	Tumor_G1
2956	DUOXA1	5.94720032	Tumor_G1
2957	DUOX2	5.621089437	Tumor_G1
2958	DUOXA2	3.851057016	Tumor_G1

2959	NDUFA1	7.90324109	Tumor_G1
2960	NDUFA2	6.430125943	Tumor_G1
2961	NDUFA8	6.86867008	Tumor_G1
2962	NDUFB1	5.539035264	Tumor_G1
2963	NDUFB10	7.103523275	Tumor_G1
2964	NDUFB2	6.43378968	Tumor_G1
2965	NDUFB3	6.276313048	Tumor_G1
2966	NDUFB7	8.363706267	Tumor_G1
2967	CYC1	6.823845093	Tumor_G1
2968	ETFB	4.776324951	Tumor_G1
2969	UQCR10	7.489964417	Tumor_G1
2970	UQCRQ	7.179468874	Tumor_G1
2971	UQCC3	4.084938753	Tumor_G1
2972	COX15	5.080106583	Tumor_G1
2973	COX5A	7.070254941	Tumor_G1
2974	COX6B1	8.330788066	Tumor_G1
2975	COX6C	6.231582641	Tumor_G1
2976	COX7A2	6.716985909	Tumor_G1
2977	COX7B	5.736321912	Tumor_G1
2978	COX8A	9.355898572	Tumor_G1
2979	SCO1	3.387680965	Tumor_G1
2980	SURF1	6.119852085	Tumor_G1
2981	ATP6V1E1	7.136579982	Tumor_G1
2982	DLAT	5.357624216	Tumor_G1
2983	PDC	0.089964793	Tumor_G1
2984	SUCLG2	6.312180986	Tumor_G1
2985	SLC25A5	8.300582966	Tumor_G1
2986	UCP2	8.427062625	Tumor_G1
2987	SOD2	6.737545355	Tumor_G1
2988	NOX4	2.892120346	Tumor_G1
2989	DUOX1	6.059859387	Tumor_G1
2990	DUOXA1	6.397217173	Tumor_G1
2991	DUOX2	6.478801571	Tumor_G1
2992	DUOXA2	4.642012917	Tumor_G1
2993	NDUFA1	7.53734307	Tumor_G1
2994	NDUFA2	5.617825597	Tumor_G1
2995	NDUFA8	6.510109146	Tumor_G1
2996	NDUFB1	5.054046169	Tumor_G1
2997	NDUFB10	6.897446519	Tumor_G1
2998	NDUFB2	5.13823356	Tumor_G1

2999	NDUFB3	5.834455161	Tumor_G1
3000	NDUFB7	7.780293171	Tumor_G1
3001	CYC1	5.832418392	Tumor_G1
3002	ETFB	3.842105569	Tumor_G1
3003	UQCR10	7.275541312	Tumor_G1
3004	UQCRQ	6.384939991	Tumor_G1
3005	UQCC3	2.77359217	Tumor_G1
3006	COX15	4.822656119	Tumor_G1
3007	COX5A	6.536377882	Tumor_G1
3008	COX6B1	8.424072462	Tumor_G1
3009	COX6C	5.418075501	Tumor_G1
3010	COX7A2	6.588054048	Tumor_G1
3011	COX7B	5.815295808	Tumor_G1
3012	COX8A	8.03720681	Tumor_G1
3013	SCO1	2.926132622	Tumor_G1
3014	SURF1	5.465207764	Tumor_G1
3015	ATP6V1E1	7.055332281	Tumor_G1
3016	DLAT	5.289196234	Tumor_G1
3017	PDC	0.102845502	Tumor_G1
3018	SUCLG2	6.321464365	Tumor_G1
3019	SLC25A5	8.037585085	Tumor_G1
3020	UCP2	8.114534772	Tumor_G1
3021	SOD2	5.761339329	Tumor_G1
3022	NOX4	2.949969813	Tumor_G1
3023	DUOX1	6.12869817	Tumor_G1
3024	DUOXA1	6.119081835	Tumor_G1
3025	DUOX2	7.233991828	Tumor_G1
3026	DUOXA2	3.79095912	Tumor_G1
3027	NDUFA1	6.985585654	Tumor_G1
3028	NDUFA2	4.786166225	Tumor_G1
3029	NDUFA8	5.657832682	Tumor_G1
3030	NDUFB1	4.760719127	Tumor_G1
3031	NDUFB10	6.040585212	Tumor_G1
3032	NDUFB2	4.144595612	Tumor_G1
3033	NDUFB3	5.095095984	Tumor_G1
3034	NDUFB7	7.235052027	Tumor_G1
3035	CYC1	4.615251546	Tumor_G1
3036	ETFB	2.82218384	Tumor_G1
3037	UQCR10	6.49667292	Tumor_G1
3038	UQCRQ	5.839557671	Tumor_G1

3039	UQCC3	2.20703681	Tumor_G1
3040	COX15	2.893186769	Tumor_G1
3041	COX5A	5.565573971	Tumor_G1
3042	COX6B1	7.830384687	Tumor_G1
3043	COX6C	4.746847361	Tumor_G1
3044	COX7A2	5.540658429	Tumor_G1
3045	COX7B	5.173894967	Tumor_G1
3046	COX8A	6.833933491	Tumor_G1
3047	SCO1	1.790579931	Tumor_G1
3048	SURF1	4.020823273	Tumor_G1
3049	ATP6V1E1	5.421202454	Tumor_G1
3050	DLAT	3.23448881	Tumor_G1
3051	PDC	0.092959604	Tumor_G1
3052	SUCLG2	4.43245174	Tumor_G1
3053	SLC25A5	6.544829106	Tumor_G1
3054	UCP2	6.33126046	Tumor_G1
3055	SOD2	3.883325484	Tumor_G1
3056	NOX4	2.51651628	Tumor_G1
3057	DUOX1	3.78697937	Tumor_G1
3058	DUOXA1	3.941302104	Tumor_G1
3059	DUOX2	3.827890951	Tumor_G1
3060	DUOXA2	1.528199666	Tumor_G1
3061	NDUFA1	7.719198605	Tumor_G1
3062	NDUFA2	6.425258348	Tumor_G1
3063	NDUFA8	6.659931081	Tumor_G1
3064	NDUFB1	5.156683186	Tumor_G1
3065	NDUFB10	7.070059055	Tumor_G1
3066	NDUFB2	6.103658178	Tumor_G1
3067	NDUFB3	6.052281816	Tumor_G1
3068	NDUFB7	8.478736062	Tumor_G1
3069	CYC1	6.403094447	Tumor_G1
3070	ETFB	4.391879278	Tumor_G1
3071	UQCR10	7.180175834	Tumor_G1
3072	UQCRQ	7.093835533	Tumor_G1
3073	UQCC3	3.974794528	Tumor_G1
3074	COX15	4.210305148	Tumor_G1
3075	COX5A	6.778099569	Tumor_G1
3076	COX6B1	8.393613492	Tumor_G1
3077	COX6C	5.963711577	Tumor_G1
3078	COX7A2	6.608918357	Tumor_G1

3079	COX7B	5.651690494	Tumor_G1
3080	COX8A	9.070994756	Tumor_G1
3081	SCO1	2.716506832	Tumor_G1
3082	SURF1	5.872791543	Tumor_G1
3083	ATP6V1E1	6.947939325	Tumor_G1
3084	DLAT	4.854472901	Tumor_G1
3085	PDC	0	Tumor_G1
3086	SUCLG2	5.560549315	Tumor_G1
3087	SLC25A5	8.052708811	Tumor_G1
3088	UCP2	7.517524903	Tumor_G1
3089	SOD2	5.550714561	Tumor_G1
3090	NOX4	3.907298129	Tumor_G1
3091	DUOX1	4.801108864	Tumor_G1
3092	DUOXA1	5.26334573	Tumor_G1
3093	DUOX2	4.005353557	Tumor_G1
3094	DUOXA2	2.47525535	Tumor_G1
3095	NDUFA1	7.734675904	Tumor_G1
3096	NDUFA2	6.523643582	Tumor_G1
3097	NDUFA8	6.722172928	Tumor_G1
3098	NDUFB1	5.458164161	Tumor_G1
3099	NDUFB10	7.289400826	Tumor_G1
3100	NDUFB2	6.115780623	Tumor_G1
3101	NDUFB3	6.281379677	Tumor_G1
3102	NDUFB7	8.214584844	Tumor_G1
3103	CYC1	6.533310224	Tumor_G1
3104	ETFB	4.170756373	Tumor_G1
3105	UQCR10	7.224879607	Tumor_G1
3106	UQCRQ	7.168895437	Tumor_G1
3107	UQCC3	3.7193176	Tumor_G1
3108	COX15	4.543725511	Tumor_G1
3109	COX5A	7.209516408	Tumor_G1
3110	COX6B1	8.572604285	Tumor_G1
3111	COX6C	6.166340592	Tumor_G1
3112	COX7A2	6.792929102	Tumor_G1
3113	COX7B	5.741960436	Tumor_G1
3114	COX8A	8.911950854	Tumor_G1
3115	SCO1	3.09687517	Tumor_G1
3116	SURF1	5.838873087	Tumor_G1
3117	ATP6V1E1	6.988953121	Tumor_G1
3118	DLAT	5.139091598	Tumor_G1

3119	PDC	0.104555577	Tumor_G1
3120	SUCLG2	6.10732094	Tumor_G1
3121	SLC25A5	8.487255271	Tumor_G1
3122	UCP2	7.350814162	Tumor_G1
3123	SOD2	5.562982557	Tumor_G1
3124	NOX4	4.027931946	Tumor_G1
3125	DUOX1	4.936841344	Tumor_G1
3126	DUOXA1	4.973373417	Tumor_G1
3127	DUOX2	3.99707236	Tumor_G1
3128	DUOXA2	1.96212542	Tumor_G1
3129	NDUFA1	7.738590945	Tumor_G1
3130	NDUFA2	6.482824855	Tumor_G1
3131	NDUFA8	6.586776611	Tumor_G1
3132	NDUFB1	5.515283492	Tumor_G1
3133	NDUFB10	7.216271947	Tumor_G1
3134	NDUFB2	6.30427451	Tumor_G1
3135	NDUFB3	6.173795608	Tumor_G1
3136	NDUFB7	8.379072659	Tumor_G1
3137	CYC1	6.529680422	Tumor_G1
3138	ETFB	4.723054442	Tumor_G1
3139	UQCR10	7.399764975	Tumor_G1
3140	UQCRQ	7.250243908	Tumor_G1
3141	UQCC3	3.911435737	Tumor_G1
3142	COX15	4.466144101	Tumor_G1
3143	COX5A	7.001620476	Tumor_G1
3144	COX6B1	8.46270324	Tumor_G1
3145	COX6C	6.126298233	Tumor_G1
3146	COX7A2	6.610412181	Tumor_G1
3147	COX7B	5.746780195	Tumor_G1
3148	COX8A	9.048871104	Tumor_G1
3149	SCO1	2.623557119	Tumor_G1
3150	SURF1	6.166387367	Tumor_G1
3151	ATP6V1E1	7.031846238	Tumor_G1
3152	DLAT	4.699546219	Tumor_G1
3153	PDC	0.113627563	Tumor_G1
3154	SUCLG2	5.908721369	Tumor_G1
3155	SLC25A5	7.741550098	Tumor_G1
3156	UCP2	7.77956426	Tumor_G1
3157	SOD2	5.199913735	Tumor_G1
3158	NOX4	2.760823291	Tumor_G1

3159	DUOX1	4.740290263	Tumor_G1
3160	DUOXA1	5.522902937	Tumor_G1
3161	DUOX2	4.386824877	Tumor_G1
3162	DUOXA2	2.618499538	Tumor_G1
3163	NDUFA1	7.599061496	Tumor_G1
3164	NDUFA2	6.368292929	Tumor_G1
3165	NDUFA8	6.638779736	Tumor_G1
3166	NDUFB1	5.223273872	Tumor_G1
3167	NDUFB10	7.194295887	Tumor_G1
3168	NDUFB2	6.023051726	Tumor_G1
3169	NDUFB3	5.941337797	Tumor_G1
3170	NDUFB7	7.990005282	Tumor_G1
3171	CYC1	6.363919312	Tumor_G1
3172	ETFB	4.563173966	Tumor_G1
3173	UQCR10	7.125790781	Tumor_G1
3174	UQCRQ	6.858910945	Tumor_G1
3175	UQCC3	3.72494791	Tumor_G1
3176	COX15	4.760135642	Tumor_G1
3177	COX5A	6.842898581	Tumor_G1
3178	COX6B1	8.226374529	Tumor_G1
3179	COX6C	6.095191856	Tumor_G1
3180	COX7A2	6.541756499	Tumor_G1
3181	COX7B	5.675427117	Tumor_G1
3182	COX8A	8.904760487	Tumor_G1
3183	SCO1	2.956957865	Tumor_G1
3184	SURF1	5.74831892	Tumor_G1
3185	ATP6V1E1	6.951904086	Tumor_G1
3186	DLAT	4.921556176	Tumor_G1
3187	PDC	0.132256719	Tumor_G1
3188	SUCLG2	6.235694811	Tumor_G1
3189	SLC25A5	7.665130965	Tumor_G1
3190	UCP2	7.276459422	Tumor_G1
3191	SOD2	5.768483512	Tumor_G1
3192	NOX4	2.691940253	Tumor_G1
3193	DUOX1	6.482277643	Tumor_G1
3194	DUOXA1	6.420256316	Tumor_G1
3195	DUOX2	6.198027077	Tumor_G1
3196	DUOXA2	3.740429997	Tumor_G1
3197	NDUFA1	7.50578643	Tumor_G1
3198	NDUFA2	6.235412819	Tumor_G1

3199	NDUFA8	6.490294956	Tumor_G1
3200	NDUFB1	5.186130041	Tumor_G1
3201	NDUFB10	7.050060532	Tumor_G1
3202	NDUFB2	5.877454317	Tumor_G1
3203	NDUFB3	5.90304778	Tumor_G1
3204	NDUFB7	7.870927842	Tumor_G1
3205	CYC1	6.308089386	Tumor_G1
3206	ETFB	4.266513252	Tumor_G1
3207	UQCR10	7.014734162	Tumor_G1
3208	UQCRQ	6.838778614	Tumor_G1
3209	UQCC3	3.437269024	Tumor_G1
3210	COX15	4.379182496	Tumor_G1
3211	COX5A	6.975990932	Tumor_G1
3212	COX6B1	8.12896759	Tumor_G1
3213	COX6C	6.033498396	Tumor_G1
3214	COX7A2	6.502268189	Tumor_G1
3215	COX7B	5.776802009	Tumor_G1
3216	COX8A	8.976083376	Tumor_G1
3217	SCO1	2.927883083	Tumor_G1
3218	SURF1	5.72211932	Tumor_G1
3219	ATP6V1E1	6.848791134	Tumor_G1
3220	DLAT	4.967914215	Tumor_G1
3221	PDC	0.102766281	Tumor_G1
3222	SUCLG2	6.003480931	Tumor_G1
3223	SLC25A5	8.153594475	Tumor_G1
3224	UCP2	7.949718015	Tumor_G1
3225	SOD2	5.848384509	Tumor_G1
3226	NOX4	3.082011673	Tumor_G1
3227	DUOX1	5.246392615	Tumor_G1
3228	DUOXA1	5.425054592	Tumor_G1
3229	DUOX2	4.306234409	Tumor_G1
3230	DUOXA2	2.334836235	Tumor_G1
3231	NDUFA1	7.647994966	Tumor_G1
3232	NDUFA2	6.64246459	Tumor_G1
3233	NDUFA8	6.64681922	Tumor_G1
3234	NDUFB1	5.306522091	Tumor_G1
3235	NDUFB10	6.966667716	Tumor_G1
3236	NDUFB2	6.027844466	Tumor_G1
3237	NDUFB3	6.071334974	Tumor_G1
3238	NDUFB7	8.288505457	Tumor_G1

3239	CYC1	6.640715278	Tumor_G1
3240	ETFB	4.251608324	Tumor_G1
3241	UQCR10	7.380317168	Tumor_G1
3242	UQCRQ	7.018607647	Tumor_G1
3243	UQCC3	3.93318258	Tumor_G1
3244	COX15	4.642760871	Tumor_G1
3245	COX5A	6.685452922	Tumor_G1
3246	COX6B1	8.214246522	Tumor_G1
3247	COX6C	5.939313129	Tumor_G1
3248	COX7A2	6.681659043	Tumor_G1
3249	COX7B	5.692943187	Tumor_G1
3250	COX8A	8.973354244	Tumor_G1
3251	SCO1	3.087122906	Tumor_G1
3252	SURF1	5.713002645	Tumor_G1
3253	ATP6V1E1	7.347859303	Tumor_G1
3254	DLAT	5.045628345	Tumor_G1
3255	PDC	0.110891433	Tumor_G1
3256	SUCLG2	6.138534233	Tumor_G1
3257	SLC25A5	8.169999667	Tumor_G1
3258	UCP2	7.837988302	Tumor_G1
3259	SOD2	5.587199682	Tumor_G1
3260	NOX4	2.780966255	Tumor_G1
3261	DUOX1	5.885721112	Tumor_G1
3262	DUOXA1	6.009292	Tumor_G1
3263	DUOX2	5.334935403	Tumor_G1
3264	DUOXA2	2.749443725	Tumor_G1
3265	NDUFA1	7.677497227	Tumor_G1
3266	NDUFA2	6.759072427	Tumor_G1
3267	NDUFA8	6.64772696	Tumor_G1
3268	NDUFB1	5.330336498	Tumor_G1
3269	NDUFB10	7.075583639	Tumor_G1
3270	NDUFB2	6.387994159	Tumor_G1
3271	NDUFB3	6.096831341	Tumor_G1
3272	NDUFB7	8.408400553	Tumor_G1
3273	CYC1	6.307849702	Tumor_G1
3274	ETFB	5.045891671	Tumor_G1
3275	UQCR10	7.365644507	Tumor_G1
3276	UQCRQ	7.053376778	Tumor_G1
3277	UQCC3	4.32580636	Tumor_G1
3278	COX15	4.283630492	Tumor_G1

3279	COX5A	6.664263208	Tumor_G1
3280	COX6B1	8.654346662	Tumor_G1
3281	COX6C	6.104987803	Tumor_G1
3282	COX7A2	6.516834058	Tumor_G1
3283	COX7B	5.799446353	Tumor_G1
3284	COX8A	9.184962714	Tumor_G1
3285	SCO1	2.893742912	Tumor_G1
3286	SURF1	6.222204326	Tumor_G1
3287	ATP6V1E1	6.956079321	Tumor_G1
3288	DLAT	4.693533354	Tumor_G1
3289	PDC	0.098288418	Tumor_G1
3290	SUCLG2	5.495074923	Tumor_G1
3291	SLC25A5	7.83254227	Tumor_G1
3292	UCP2	8.452844373	Tumor_G1
3293	SOD2	4.524084273	Tumor_G1
3294	NOX4	3.116589164	Tumor_G1
3295	DUOX1	6.032337811	Tumor_G1
3296	DUOXA1	6.566147752	Tumor_G1
3297	DUOX2	5.756418744	Tumor_G1
3298	DUOXA2	3.905970418	Tumor_G1
3299	NDUFA1	7.851069874	Tumor_G1
3300	NDUFA2	6.777065398	Tumor_G1
3301	NDUFA8	6.81235384	Tumor_G1
3302	NDUFB1	5.423689421	Tumor_G1
3303	NDUFB10	7.282025264	Tumor_G1
3304	NDUFB2	6.609995508	Tumor_G1
3305	NDUFB3	6.370218033	Tumor_G1
3306	NDUFB7	8.299685474	Tumor_G1
3307	CYC1	6.610097503	Tumor_G1
3308	ETFB	4.530197975	Tumor_G1
3309	UQCR10	7.429170536	Tumor_G1
3310	UQCRQ	7.147762291	Tumor_G1
3311	UQCC3	3.920199694	Tumor_G1
3312	COX15	4.385085333	Tumor_G1
3313	COX5A	7.440643568	Tumor_G1
3314	COX6B1	8.599027411	Tumor_G1
3315	COX6C	6.261641019	Tumor_G1
3316	COX7A2	6.751127885	Tumor_G1
3317	COX7B	6.056422804	Tumor_G1
3318	COX8A	9.182497844	Tumor_G1

3319	SCO1	2.962177366	Tumor_G1
3320	SURF1	5.6858426	Tumor_G1
3321	ATP6V1E1	7.437250087	Tumor_G1
3322	DLAT	5.315244373	Tumor_G1
3323	PDC	0.12117104	Tumor_G1
3324	SUCLG2	6.095762572	Tumor_G1
3325	SLC25A5	8.288298245	Tumor_G1
3326	UCP2	8.368190133	Tumor_G1
3327	SOD2	6.542378605	Tumor_G1
3328	NOX4	2.207306959	Tumor_G1
3329	DUOX1	5.253185318	Tumor_G1
3330	DUOXA1	5.540677327	Tumor_G1
3331	DUOX2	5.267083056	Tumor_G1
3332	DUOXA2	3.661332127	Tumor_G1
3333	NDUFA1	7.472441927	Tumor_G1
3334	NDUFA2	7.352159525	Tumor_G1
3335	NDUFA8	5.991223336	Tumor_G1
3336	NDUFB1	5.333824837	Tumor_G1
3337	NDUFB10	7.927383578	Tumor_G1
3338	NDUFB2	6.515306864	Tumor_G1
3339	NDUFB3	6.367028817	Tumor_G1
3340	NDUFB7	8.514542428	Tumor_G1
3341	CYC1	7.601779994	Tumor_G1
3342	ETFB	4.413275899	Tumor_G1
3343	UQCR10	6.768538899	Tumor_G1
3344	UQCRQ	8.334318223	Tumor_G1
3345	UQCC3	4.594566923	Tumor_G1
3346	COX15	4.844666194	Tumor_G1
3347	COX5A	6.718051462	Tumor_G1
3348	COX6B1	8.432321447	Tumor_G1
3349	COX6C	6.364206996	Tumor_G1
3350	COX7A2	6.769388843	Tumor_G1
3351	COX7B	5.508106755	Tumor_G1
3352	COX8A	9.2305699	Tumor_G1
3353	SCO1	3.714696196	Tumor_G1
3354	SURF1	5.825063999	Tumor_G1
3355	ATP6V1E1	6.787530288	Tumor_G1
3356	DLAT	4.904730951	Tumor_G1
3357	PDC	0.301178339	Tumor_G1
3358	SUCLG2	6.23024195	Tumor_G1

3359	SLC25A5	8.260048784	Tumor_G1
3360	UCP2	7.183220531	Tumor_G1
3361	SOD2	5.163165366	Tumor_G1
3362	NOX4	3.364236902	Tumor_G1
3363	DUOX1	5.146527198	Tumor_G1
3364	DUOXA1	5.029512775	Tumor_G1
3365	DUOX2	4.049847428	Tumor_G1
3366	DUOXA2	2.557953261	Tumor_G1
3367	NDUFA1	7.582845335	Tumor_G1
3368	NDUFA2	6.571062256	Tumor_G1
3369	NDUFA8	6.153883639	Tumor_G1
3370	NDUFB1	5.142460511	Tumor_G1
3371	NDUFB10	6.704699952	Tumor_G1
3372	NDUFB2	6.304982656	Tumor_G1
3373	NDUFB3	6.06408641	Tumor_G1
3374	NDUFB7	7.936356299	Tumor_G1
3375	CYC1	6.395685571	Tumor_G1
3376	ETFB	3.954494129	Tumor_G1
3377	UQCR10	7.155298137	Tumor_G1
3378	UQCRQ	6.838200455	Tumor_G1
3379	UQCC3	3.786601185	Tumor_G1
3380	COX15	4.225792774	Tumor_G1
3381	COX5A	6.917262228	Tumor_G1
3382	COX6B1	8.116134994	Tumor_G1
3383	COX6C	5.780294404	Tumor_G1
3384	COX7A2	6.478307448	Tumor_G1
3385	COX7B	5.581862214	Tumor_G1
3386	COX8A	9.06873352	Tumor_G1
3387	SCO1	2.975086066	Tumor_G1
3388	SURF1	5.713534405	Tumor_G1
3389	ATP6V1E1	6.942287459	Tumor_G1
3390	DLAT	4.859796208	Tumor_G1
3391	PDC	0.081386087	Tumor_G1
3392	SUCLG2	6.20242321	Tumor_G1
3393	SLC25A5	8.196920178	Tumor_G1
3394	UCP2	7.571128919	Tumor_G1
3395	SOD2	5.767993859	Tumor_G1
3396	NOX4	1.209748593	Tumor_G1
3397	DUOX1	5.206031721	Tumor_G1
3398	DUOXA1	5.369345789	Tumor_G1

3399	DUOX2	5.628956364	Tumor_G1
3400	DUOXA2	3.222910101	Tumor_G1
3401	NDUFA1	8.210089681	Tumor_G1
3402	NDUFA2	7.195604253	Tumor_G1
3403	NDUFA8	6.272747286	Tumor_G1
3404	NDUFB1	5.712654832	Tumor_G1
3405	NDUFB10	7.826356595	Tumor_G1
3406	NDUFB2	6.566862831	Tumor_G1
3407	NDUFB3	6.454474539	Tumor_G1
3408	NDUFB7	8.953512012	Tumor_G1
3409	CYC1	6.972902427	Tumor_G1
3410	ETFB	4.796274705	Tumor_G1
3411	UQCR10	7.726126462	Tumor_G1
3412	UQCRQ	7.682287832	Tumor_G1
3413	UQCC3	4.522050823	Tumor_G1
3414	COX15	3.845228326	Tumor_G1
3415	COX5A	7.499845356	Tumor_G1
3416	COX6B1	9.136476791	Tumor_G1
3417	COX6C	6.419509561	Tumor_G1
3418	COX7A2	7.140101947	Tumor_G1
3419	COX7B	6.190822033	Tumor_G1
3420	COX8A	9.608566629	Tumor_G1
3421	SCO1	2.831996314	Tumor_G1
3422	SURF1	5.700671998	Tumor_G1
3423	ATP6V1E1	7.251491208	Tumor_G1
3424	DLAT	4.535853317	Tumor_G1
3425	PDC	0.077040826	Tumor_G1
3426	SUCLG2	6.022340429	Tumor_G1
3427	SLC25A5	7.806786768	Tumor_G1
3428	UCP2	8.335353258	Tumor_G1
3429	SOD2	5.695681097	Tumor_G1
3430	NOX4	2.557196011	Tumor_G1
3431	DUOX1	4.801699346	Tumor_G1
3432	DUOXA1	5.072428295	Tumor_G1
3433	DUOX2	4.516455361	Tumor_G1
3434	DUOXA2	3.225598684	Tumor_G1
3435	NDUFA1	7.955995763	Tumor_G1
3436	NDUFA2	6.710528592	Tumor_G1
3437	NDUFA8	6.9342347	Tumor_G1
3438	NDUFB1	5.821109271	Tumor_G1

3439	NDUFB10	7.452406825	Tumor_G1
3440	NDUFB2	6.438081322	Tumor_G1
3441	NDUFB3	6.016145135	Tumor_G1
3442	NDUFB7	8.335764766	Tumor_G1
3443	CYC1	6.377599014	Tumor_G1
3444	ETFB	4.878119609	Tumor_G1
3445	UQCR10	7.390880596	Tumor_G1
3446	UQCRQ	7.163370079	Tumor_G1
3447	UQCC3	4.309090002	Tumor_G1
3448	COX15	4.629548143	Tumor_G1
3449	COX5A	7.07399986	Tumor_G1
3450	COX6B1	8.604591327	Tumor_G1
3451	COX6C	6.399868808	Tumor_G1
3452	COX7A2	6.727489941	Tumor_G1
3453	COX7B	5.865964304	Tumor_G1
3454	COX8A	9.182726046	Tumor_G1
3455	SCO1	3.033715099	Tumor_G1
3456	SURF1	6.293925279	Tumor_G1
3457	ATP6V1E1	6.863378789	Tumor_G1
3458	DLAT	4.787329559	Tumor_G1
3459	PDC	0.478076579	Tumor_G1
3460	SUCLG2	5.39160477	Tumor_G1
3461	SLC25A5	7.487109121	Tumor_G1
3462	UCP2	8.982515504	Tumor_G1
3463	SOD2	4.233365859	Tumor_G1
3464	NOX4	1.437602493	Tumor_G1
3465	DUOX1	6.022200045	Tumor_G1
3466	DUOXA1	6.523435305	Tumor_G1
3467	DUOX2	6.49853057	Tumor_G1
3468	DUOXA2	5.000929619	Tumor_G1
3469	NDUFA1	7.480426309	Tumor_G1
3470	NDUFA2	6.551231532	Tumor_G1
3471	NDUFA8	6.960261519	Tumor_G1
3472	NDUFB1	5.168284096	Tumor_G1
3473	NDUFB10	7.501979281	Tumor_G1
3474	NDUFB2	6.671784119	Tumor_G1
3475	NDUFB3	5.940761276	Tumor_G1
3476	NDUFB7	8.326139179	Tumor_G1
3477	CYC1	7.132566075	Tumor_G1
3478	ETFB	4.68944769	Tumor_G1

3479	UQCR10	7.178944209	Tumor_G1
3480	UQCRQ	7.013086378	Tumor_G1
3481	UQCC3	4.183949827	Tumor_G1
3482	COX15	4.573338187	Tumor_G1
3483	COX5A	7.390944473	Tumor_G1
3484	COX6B1	8.603118125	Tumor_G1
3485	COX6C	5.970570344	Tumor_G1
3486	COX7A2	6.848208343	Tumor_G1
3487	COX7B	6.019955032	Tumor_G1
3488	COX8A	9.634659707	Tumor_G1
3489	SCO1	3.330021091	Tumor_G1
3490	SURF1	6.081807915	Tumor_G1
3491	ATP6V1E1	7.161075691	Tumor_G1
3492	DLAT	5.406350987	Tumor_G1
3493	PDC	0	Tumor_G1
3494	SUCLG2	6.214165939	Tumor_G1
3495	SLC25A5	9.033013886	Tumor_G1
3496	UCP2	7.921203332	Tumor_G1
3497	SOD2	4.84646303	Tumor_G1
3498	NOX4	1.75988594	Tumor_G1
3499	DUOX1	5.374244105	Tumor_G1
3500	DUOXA1	5.515255398	Tumor_G1
3501	DUOX2	5.486748909	Tumor_G1
3502	DUOXA2	3.346727697	Tumor_G1
3503	NDUFA1	6.819415076	Tumor_G1
3504	NDUFA2	6.098989961	Tumor_G1
3505	NDUFA8	5.897283523	Tumor_G1
3506	NDUFB1	4.688882141	Tumor_G1
3507	NDUFB10	6.604306415	Tumor_G1
3508	NDUFB2	5.339941561	Tumor_G1
3509	NDUFB3	5.488049378	Tumor_G1
3510	NDUFB7	7.623936992	Tumor_G1
3511	CYC1	6.030103482	Tumor_G1
3512	ETFB	3.739323122	Tumor_G1
3513	UQCR10	6.510591648	Tumor_G1
3514	UQCRQ	6.481816341	Tumor_G1
3515	UQCC3	3.397830954	Tumor_G1
3516	COX15	3.792308555	Tumor_G1
3517	COX5A	6.275812506	Tumor_G1
3518	COX6B1	7.678706386	Tumor_G1

3519	COX6C	4.919871128	Tumor_G1
3520	COX7A2	5.887497206	Tumor_G1
3521	COX7B	5.142648769	Tumor_G1
3522	COX8A	8.436466244	Tumor_G1
3523	SCO1	2.50285839	Tumor_G1
3524	SURF1	5.561339321	Tumor_G1
3525	ATP6V1E1	6.491029312	Tumor_G1
3526	DLAT	4.312456943	Tumor_G1
3527	PDC	0.12147023	Tumor_G1
3528	SUCLG2	5.397410888	Tumor_G1
3529	SLC25A5	7.501722329	Tumor_G1
3530	UCP2	7.357285894	Tumor_G1
3531	SOD2	6.261907591	Tumor_G1
3532	NOX4	1.798835775	Tumor_G1
3533	DUOX1	5.084935431	Tumor_G1
3534	DUOXA1	5.034227874	Tumor_G1
3535	DUOX2	4.299727752	Tumor_G1
3536	DUOXA2	2.680624262	Tumor_G1
3537	NDUFA1	7.249436346	Tumor_G1
3538	NDUFA2	6.209156863	Tumor_G1
3539	NDUFA8	6.230571155	Tumor_G1
3540	NDUFB1	5.066212242	Tumor_G1
3541	NDUFB10	6.806317671	Tumor_G1
3542	NDUFB2	5.870253745	Tumor_G1
3543	NDUFB3	5.237796433	Tumor_G1
3544	NDUFB7	8.012589216	Tumor_G1
3545	CYC1	5.866672755	Tumor_G1
3546	ETFB	4.022590228	Tumor_G1
3547	UQCR10	6.884617386	Tumor_G1
3548	UQCRQ	6.705959238	Tumor_G1
3549	UQCC3	3.704003652	Tumor_G1
3550	COX15	3.368467671	Tumor_G1
3551	COX5A	6.323318666	Tumor_G1
3552	COX6B1	8.161589124	Tumor_G1
3553	COX6C	5.846999906	Tumor_G1
3554	COX7A2	5.941404142	Tumor_G1
3555	COX7B	5.256799385	Tumor_G1
3556	COX8A	8.268144399	Tumor_G1
3557	SCO1	1.905753704	Tumor_G1
3558	SURF1	5.232316765	Tumor_G1

3559	ATP6V1E1	5.941167953	Tumor_G1
3560	DLAT	3.564293213	Tumor_G1
3561	PDC	0	Tumor_G1
3562	SUCLG2	4.893150795	Tumor_G1
3563	SLC25A5	6.329919403	Tumor_G1
3564	UCP2	7.690229487	Tumor_G1
3565	SOD2	3.485872323	Tumor_G1
3566	NOX4	1.376717403	Tumor_G1
3567	DUOX1	4.570346314	Tumor_G1
3568	DUOXA1	4.850643456	Tumor_G1
3569	DUOX2	4.038334991	Tumor_G1
3570	DUOXA2	2.869884336	Tumor_G1
3571	NDUFA1	7.524976507	Tumor_G1
3572	NDUFA2	6.512694997	Tumor_G1
3573	NDUFA8	6.532831856	Tumor_G1
3574	NDUFB1	5.008120316	Tumor_G1
3575	NDUFB10	7.299805401	Tumor_G1
3576	NDUFB2	6.110235968	Tumor_G1
3577	NDUFB3	5.836364484	Tumor_G1
3578	NDUFB7	8.17287174	Tumor_G1
3579	CYC1	6.490022914	Tumor_G1
3580	ETFB	4.294234416	Tumor_G1
3581	UQCR10	7.229086186	Tumor_G1
3582	UQCRQ	6.918108315	Tumor_G1
3583	UQCC3	3.946319136	Tumor_G1
3584	COX15	4.756641116	Tumor_G1
3585	COX5A	6.828282735	Tumor_G1
3586	COX6B1	8.293272919	Tumor_G1
3587	COX6C	5.90370324	Tumor_G1
3588	COX7A2	6.46652055	Tumor_G1
3589	COX7B	5.456123513	Tumor_G1
3590	COX8A	9.27433892	Tumor_G1
3591	SCO1	2.962625773	Tumor_G1
3592	SURF1	5.726843401	Tumor_G1
3593	ATP6V1E1	7.149339982	Tumor_G1
3594	DLAT	5.237314944	Tumor_G1
3595	PDC	0.050275374	Tumor_G1
3596	SUCLG2	6.590486003	Tumor_G1
3597	SLC25A5	8.303831861	Tumor_G1
3598	UCP2	7.901095399	Tumor_G1

3599	SOD2	6.200474612	Tumor_G1
3600	NOX4	2.854303379	Tumor_G1
3601	DUOX1	5.63205113	Tumor_G1
3602	DUOXA1	5.584945303	Tumor_G1
3603	DUOX2	4.849115302	Tumor_G1
3604	DUOXA2	2.439605014	Tumor_G1
3605	NDUFA1	7.139189535	Tumor_G1
3606	NDUFA2	6.038845901	Tumor_G1
3607	NDUFA8	6.375028359	Tumor_G1
3608	NDUFB1	4.691861166	Tumor_G1
3609	NDUFB10	7.044040003	Tumor_G1
3610	NDUFB2	5.946045049	Tumor_G1
3611	NDUFB3	5.339500877	Tumor_G1
3612	NDUFB7	7.821303755	Tumor_G1
3613	CYC1	6.617742483	Tumor_G1
3614	ETFB	4.437662762	Tumor_G1
3615	UQCR10	6.844135075	Tumor_G1
3616	UQCRQ	6.303561972	Tumor_G1
3617	UQCC3	3.71850422	Tumor_G1
3618	COX15	4.850893564	Tumor_G1
3619	COX5A	6.4812789	Tumor_G1
3620	COX6B1	7.952056376	Tumor_G1
3621	COX6C	5.737774171	Tumor_G1
3622	COX7A2	5.884119943	Tumor_G1
3623	COX7B	5.3152692	Tumor_G1
3624	COX8A	8.978111357	Tumor_G1
3625	SCO1	3.004510708	Tumor_G1
3626	SURF1	5.599048716	Tumor_G1
3627	ATP6V1E1	6.697528511	Tumor_G1
3628	DLAT	4.842795623	Tumor_G1
3629	PDC	0.159376486	Tumor_G1
3630	SUCLG2	6.200292811	Tumor_G1
3631	SLC25A5	7.619542917	Tumor_G1
3632	UCP2	7.866232231	Tumor_G1
3633	SOD2	5.176994815	Tumor_G1
3634	NOX4	1.036967394	Tumor_G1
3635	DUOX1	6.211908438	Tumor_G1
3636	DUOXA1	6.030600861	Tumor_G1
3637	DUOX2	6.131411893	Tumor_G1
3638	DUOXA2	3.473035873	Tumor_G1

3639	NDUFA1	7.282077341	Tumor_G1
3640	NDUFA2	6.103866158	Tumor_G1
3641	NDUFA8	6.384106333	Tumor_G1
3642	NDUFB1	5.080787872	Tumor_G1
3643	NDUFB10	6.822743205	Tumor_G1
3644	NDUFB2	5.949142916	Tumor_G1
3645	NDUFB3	5.858906896	Tumor_G1
3646	NDUFB7	7.868357099	Tumor_G1
3647	CYC1	6.245664015	Tumor_G1
3648	ETFB	4.270502012	Tumor_G1
3649	UQCR10	6.945168162	Tumor_G1
3650	UQCRQ	6.670976908	Tumor_G1
3651	UQCC3	3.60089871	Tumor_G1
3652	COX15	4.259763623	Tumor_G1
3653	COX5A	6.576765731	Tumor_G1
3654	COX6B1	8.020882463	Tumor_G1
3655	COX6C	5.749406106	Tumor_G1
3656	COX7A2	6.174036239	Tumor_G1
3657	COX7B	5.301861634	Tumor_G1
3658	COX8A	8.710892837	Tumor_G1
3659	SCO1	2.952034265	Tumor_G1
3660	SURF1	5.801145834	Tumor_G1
3661	ATP6V1E1	6.755830834	Tumor_G1
3662	DLAT	4.584098631	Tumor_G1
3663	PDC	0.088002364	Tumor_G1
3664	SUCLG2	5.612212447	Tumor_G1
3665	SLC25A5	7.872141726	Tumor_G1
3666	UCP2	7.85934995	Tumor_G1
3667	SOD2	4.834322402	Tumor_G1
3668	NOX4	2.445888116	Tumor_G1
3669	DUOX1	5.592710963	Tumor_G1
3670	DUOXA1	5.739903712	Tumor_G1
3671	DUOX2	5.468265915	Tumor_G1
3672	DUOXA2	2.811068981	Tumor_G1
3673	NDUFA1	8.016191681	Tumor_G1
3674	NDUFA2	6.680450055	Tumor_G1
3675	NDUFA8	6.838338822	Tumor_G1
3676	NDUFB1	5.457785742	Tumor_G1
3677	NDUFB10	7.513556528	Tumor_G1
3678	NDUFB2	5.973638338	Tumor_G1

3679	NDUFB3	5.844746622	Tumor_G1
3680	NDUFB7	8.64686387	Tumor_G1
3681	CYC1	6.705859297	Tumor_G1
3682	ETFB	4.713371333	Tumor_G1
3683	UQCR10	6.884725166	Tumor_G1
3684	UQCRQ	7.303451028	Tumor_G1
3685	UQCC3	4.36603136	Tumor_G1
3686	COX15	4.102962704	Tumor_G1
3687	COX5A	6.912672088	Tumor_G1
3688	COX6B1	8.426607445	Tumor_G1
3689	COX6C	6.118249401	Tumor_G1
3690	COX7A2	6.764615886	Tumor_G1
3691	COX7B	5.869584314	Tumor_G1
3692	COX8A	9.179711877	Tumor_G1
3693	SCO1	2.827830501	Tumor_G1
3694	SURF1	6.28611655	Tumor_G1
3695	ATP6V1E1	5.833219171	Tumor_G1
3696	DLAT	4.136742788	Tumor_G1
3697	PDC	0.061563953	Tumor_G1
3698	SUCLG2	5.075558089	Tumor_G1
3699	SLC25A5	7.583245638	Tumor_G1
3700	UCP2	8.076587047	Tumor_G1
3701	SOD2	5.056164978	Tumor_G1
3702	NOX4	1.7006057	Tumor_G1
3703	DUOX1	6.364507556	Tumor_G1
3704	DUOXA1	6.716571293	Tumor_G1
3705	DUOX2	7.08499323	Tumor_G1
3706	DUOXA2	5.503494568	Tumor_G1
3707	NDUFA1	8.225560785	Tumor_G1
3708	NDUFA2	6.553463373	Tumor_G1
3709	NDUFA8	7.347047472	Tumor_G1
3710	NDUFB1	5.73070217	Tumor_G1
3711	NDUFB10	7.249631741	Tumor_G1
3712	NDUFB2	6.068657505	Tumor_G1
3713	NDUFB3	6.687815422	Tumor_G1
3714	NDUFB7	8.118539108	Tumor_G1
3715	CYC1	7.19631629	Tumor_G1
3716	ETFB	4.839844394	Tumor_G1
3717	UQCR10	7.713951649	Tumor_G1
3718	UQCRQ	6.901237249	Tumor_G1

3719	UQCC3	3.862981338	Tumor_G1
3720	COX15	5.378065498	Tumor_G1
3721	COX5A	7.28256841	Tumor_G1
3722	COX6B1	8.443326031	Tumor_G1
3723	COX6C	6.527853473	Tumor_G1
3724	COX7A2	6.790197777	Tumor_G1
3725	COX7B	6.296307206	Tumor_G1
3726	COX8A	9.558534918	Tumor_G1
3727	SCO1	3.518677575	Tumor_G1
3728	SURF1	6.627047236	Tumor_G1
3729	ATP6V1E1	6.694782854	Tumor_G1
3730	DLAT	5.416469835	Tumor_G1
3731	PDC	0.17872959	Tumor_G1
3732	SUCLG2	6.169171129	Tumor_G1
3733	SLC25A5	8.325690301	Tumor_G1
3734	UCP2	7.680641309	Tumor_G1
3735	SOD2	6.701726693	Tumor_G1
3736	NOX4	1.286173992	Tumor_G1
3737	DUOX1	5.83773057	Tumor_G1
3738	DUOXA1	5.005289005	Tumor_G1
3739	DUOX2	4.95593108	Tumor_G1
3740	DUOXA2	2.879968397	Tumor_G1
3741	NDUFA1	7.590760933	Tumor_G1
3742	NDUFA2	5.423405712	Tumor_G1
3743	NDUFA8	6.26046956	Tumor_G1
3744	NDUFB1	5.220499492	Tumor_G1
3745	NDUFB10	6.38761807	Tumor_G1
3746	NDUFB2	4.798411119	Tumor_G1
3747	NDUFB3	5.896131525	Tumor_G1
3748	NDUFB7	7.44103612	Tumor_G1
3749	CYC1	5.599053189	Tumor_G1
3750	ETFB	3.567998766	Tumor_G1
3751	UQCR10	7.076056265	Tumor_G1
3752	UQCRQ	6.451992839	Tumor_G1
3753	UQCC3	2.618542402	Tumor_G1
3754	COX15	4.169588057	Tumor_G1
3755	COX5A	6.590537729	Tumor_G1
3756	COX6B1	8.408029308	Tumor_G1
3757	COX6C	5.438510373	Tumor_G1
3758	COX7A2	6.367471897	Tumor_G1

3759	COX7B	5.927797076	Tumor_G1
3760	COX8A	7.726753117	Tumor_G1
3761	SCO1	2.784903722	Tumor_G1
3762	SURF1	5.318122713	Tumor_G1
3763	ATP6V1E1	6.597996745	Tumor_G1
3764	DLAT	4.513636616	Tumor_G1
3765	PDC	0.163961428	Tumor_G1
3766	SUCLG2	5.60458294	Tumor_G1
3767	SLC25A5	7.584982816	Tumor_G1
3768	UCP2	7.420880074	Tumor_G1
3769	SOD2	5.677203089	Tumor_G1
3770	NOX4	2.96975116	Tumor_G1
3771	DUOX1	4.464722758	Tumor_G1
3772	DUOXA1	4.825880406	Tumor_G1
3773	DUOX2	3.651916489	Tumor_G1
3774	DUOXA2	1.958616417	Tumor_G1
3775	NDUFA1	8.047721961	Tumor_G1
3776	NDUFA2	6.483037521	Tumor_G1
3777	NDUFA8	6.825500267	Tumor_G1
3778	NDUFB1	5.638280503	Tumor_G1
3779	NDUFB10	7.353744118	Tumor_G1
3780	NDUFB2	6.172315978	Tumor_G1
3781	NDUFB3	6.55155758	Tumor_G1
3782	NDUFB7	8.491727351	Tumor_G1
3783	CYC1	6.458874488	Tumor_G1
3784	ETFB	4.905695	Tumor_G1
3785	UQCR10	7.626604719	Tumor_G1
3786	UQCRQ	7.194989674	Tumor_G1
3787	UQCC3	4.030750409	Tumor_G1
3788	COX15	4.884564691	Tumor_G1
3789	COX5A	7.208487466	Tumor_G1
3790	COX6B1	8.437776055	Tumor_G1
3791	COX6C	6.32285023	Tumor_G1
3792	COX7A2	6.835723577	Tumor_G1
3793	COX7B	5.835274861	Tumor_G1
3794	COX8A	8.990613448	Tumor_G1
3795	SCO1	3.75012178	Tumor_G1
3796	SURF1	5.913286553	Tumor_G1
3797	ATP6V1E1	7.014655015	Tumor_G1
3798	DLAT	5.856879907	Tumor_G1

3799	PDC	0.184302677	Tumor_G1
3800	SUCLG2	6.391703705	Tumor_G1
3801	SLC25A5	8.262711656	Tumor_G1
3802	UCP2	8.871832578	Tumor_G1
3803	SOD2	5.327496296	Tumor_G1
3804	NOX4	3.10309825	Tumor_G1
3805	DUOX1	6.588048626	Tumor_G1
3806	DUOXA1	6.712291823	Tumor_G1
3807	DUOX2	6.966403011	Tumor_G1
3808	DUOXA2	4.550452819	Tumor_G1
3809	NDUFA1	7.989789428	Tumor_G1
3810	NDUFA2	7.037450071	Tumor_G1
3811	NDUFA8	6.734812949	Tumor_G1
3812	NDUFB1	5.507210348	Tumor_G1
3813	NDUFB10	7.698697776	Tumor_G1
3814	NDUFB2	6.444512124	Tumor_G1
3815	NDUFB3	6.06799409	Tumor_G1
3816	NDUFB7	8.810438542	Tumor_G1
3817	CYC1	6.816154771	Tumor_G1
3818	ETFB	5.026677304	Tumor_G1
3819	UQCR10	7.706279857	Tumor_G1
3820	UQCRQ	7.394661429	Tumor_G1
3821	UQCC3	4.313444068	Tumor_G1
3822	COX15	4.067568532	Tumor_G1
3823	COX5A	7.288998329	Tumor_G1
3824	COX6B1	8.981457897	Tumor_G1
3825	COX6C	6.414160009	Tumor_G1
3826	COX7A2	6.791625226	Tumor_G1
3827	COX7B	5.836173649	Tumor_G1
3828	COX8A	9.305989453	Tumor_G1
3829	SCO1	2.48830035	Tumor_G1
3830	SURF1	6.228464427	Tumor_G1
3831	ATP6V1E1	7.156524807	Tumor_G1
3832	DLAT	4.13303671	Tumor_G1
3833	PDC	0.040192972	Tumor_G1
3834	SUCLG2	5.701492358	Tumor_G1
3835	SLC25A5	7.721264532	Tumor_G1
3836	UCP2	7.705129816	Tumor_G1
3837	SOD2	5.609612542	Tumor_G1
3838	NOX4	2.924140241	Tumor_G1

3839	DUOX1	5.899194575	Tumor_G1
3840	DUOXA1	5.799985626	Tumor_G1
3841	DUOX2	4.654671739	Tumor_G1
3842	DUOXA2	3.0301179	Tumor_G1
3843	NDUFA1	8.155860699	Tumor_G1
3844	NDUFA2	7.401112635	Tumor_G1
3845	NDUFA8	6.253013702	Tumor_G1
3846	NDUFB1	6.383187129	Tumor_G1
3847	NDUFB10	7.729360137	Tumor_G1
3848	NDUFB2	6.066904101	Tumor_G1
3849	NDUFB3	6.33088228	Tumor_G1
3850	NDUFB7	9.453956907	Tumor_G1
3851	CYC1	6.67942097	Tumor_G1
3852	ETFB	5.204806281	Tumor_G1
3853	UQCR10	7.785095382	Tumor_G1
3854	UQCRQ	7.78343114	Tumor_G1
3855	UQCC3	5.121895264	Tumor_G1
3856	COX15	4.112375655	Tumor_G1
3857	COX5A	7.220431857	Tumor_G1
3858	COX6B1	9.178613665	Tumor_G1
3859	COX6C	6.599877192	Tumor_G1
3860	COX7A2	6.828912181	Tumor_G1
3861	COX7B	6.255262158	Tumor_G1
3862	COX8A	9.315970442	Tumor_G1
3863	SCO1	2.43852774	Tumor_G1
3864	SURF1	5.998921069	Tumor_G1
3865	ATP6V1E1	6.659326661	Tumor_G1
3866	DLAT	4.694656742	Tumor_G1
3867	PDC	0	Tumor_G1
3868	SUCLG2	5.326831778	Tumor_G1
3869	SLC25A5	7.592501452	Tumor_G1
3870	UCP2	8.501630502	Tumor_G1
3871	SOD2	4.919118432	Tumor_G1
3872	NOX4	1.594266706	Tumor_G1
3873	DUOX1	4.942991643	Tumor_G1
3874	DUOXA1	4.947829568	Tumor_G1
3875	DUOX2	2.53765001	Tumor_G1
3876	DUOXA2	0.549875022	Tumor_G1
3877	NDUFA1	8.015370096	Tumor_G1
3878	NDUFA2	6.82295843	Tumor_G1

3879	NDUFA8	6.95815582	Tumor_G1
3880	NDUFB1	5.608170284	Tumor_G1
3881	NDUFB10	7.187048982	Tumor_G1
3882	NDUFB2	6.526045906	Tumor_G1
3883	NDUFB3	6.357978054	Tumor_G1
3884	NDUFB7	8.508521727	Tumor_G1
3885	CYC1	6.57950372	Tumor_G1
3886	ETFB	4.762692089	Tumor_G1
3887	UQCR10	7.465200786	Tumor_G1
3888	UQCRQ	7.278574562	Tumor_G1
3889	UQCC3	4.115799265	Tumor_G1
3890	COX15	4.349122173	Tumor_G1
3891	COX5A	7.030140086	Tumor_G1
3892	COX6B1	8.687943754	Tumor_G1
3893	COX6C	6.324620602	Tumor_G1
3894	COX7A2	6.971789436	Tumor_G1
3895	COX7B	5.978518617	Tumor_G1
3896	COX8A	9.151046039	Tumor_G1
3897	SCO1	2.998858711	Tumor_G1
3898	SURF1	6.110110706	Tumor_G1
3899	ATP6V1E1	7.151381352	Tumor_G1
3900	DLAT	5.065060302	Tumor_G1
3901	PDC	0	Tumor_G1
3902	SUCLG2	6.071607381	Tumor_G1
3903	SLC25A5	8.101604259	Tumor_G1
3904	UCP2	8.335536241	Tumor_G1
3905	SOD2	5.841846544	Tumor_G1
3906	NOX4	3.028144503	Tumor_G1
3907	DUOX1	5.346568353	Tumor_G1
3908	DUOXA1	5.693851785	Tumor_G1
3909	DUOX2	5.241204509	Tumor_G1
3910	DUOXA2	3.840765299	Tumor_G1
3911	NDUFA1	7.530272618	Tumor_G1
3912	NDUFA2	6.367451785	Tumor_G1
3913	NDUFA8	6.723390591	Tumor_G1
3914	NDUFB1	5.450826466	Tumor_G1
3915	NDUFB10	7.227398645	Tumor_G1
3916	NDUFB2	6.057482916	Tumor_G1
3917	NDUFB3	5.872466513	Tumor_G1
3918	NDUFB7	8.291089785	Tumor_G1

3919	CYC1	6.508568285	Tumor_G1
3920	ETFB	4.686963431	Tumor_G1
3921	UQCR10	7.277988981	Tumor_G1
3922	UQCRQ	6.81753483	Tumor_G1
3923	UQCC3	4.214710027	Tumor_G1
3924	COX15	4.545802886	Tumor_G1
3925	COX5A	6.718217414	Tumor_G1
3926	COX6B1	8.255490534	Tumor_G1
3927	COX6C	6.007058393	Tumor_G1
3928	COX7A2	6.485459342	Tumor_G1
3929	COX7B	5.61312635	Tumor_G1
3930	COX8A	8.810654002	Tumor_G1
3931	SCO1	2.864471168	Tumor_G1
3932	SURF1	6.04697571	Tumor_G1
3933	ATP6V1E1	6.738628612	Tumor_G1
3934	DLAT	4.91419975	Tumor_G1
3935	PDC	0.072149682	Tumor_G1
3936	SUCLG2	5.946936655	Tumor_G1
3937	SLC25A5	7.646464582	Tumor_G1
3938	UCP2	8.13921729	Tumor_G1
3939	SOD2	5.225327942	Tumor_G1
3940	NOX4	1.460261924	Tumor_G1
3941	DUOX1	6.198155146	Tumor_G1
3942	DUOXA1	6.373103669	Tumor_G1
3943	DUOX2	6.613039621	Tumor_G1
3944	DUOXA2	4.743276325	Tumor_G1
3945	NDUFA1	7.578997886	Tumor_G1
3946	NDUFA2	6.781299928	Tumor_G1
3947	NDUFA8	6.78793221	Tumor_G1
3948	NDUFB1	5.57814518	Tumor_G1
3949	NDUFB10	7.621764521	Tumor_G1
3950	NDUFB2	6.101717073	Tumor_G1
3951	NDUFB3	5.77734239	Tumor_G1
3952	NDUFB7	8.476924227	Tumor_G1
3953	CYC1	6.379983715	Tumor_G1
3954	ETFB	4.602167281	Tumor_G1
3955	UQCR10	7.38956464	Tumor_G1
3956	UQCRQ	7.172529387	Tumor_G1
3957	UQCC3	4.070270046	Tumor_G1
3958	COX15	3.943830209	Tumor_G1

3959	COX5A	6.970648147	Tumor_G1
3960	COX6B1	8.721988302	Tumor_G1
3961	COX6C	6.26494455	Tumor_G1
3962	COX7A2	6.588038276	Tumor_G1
3963	COX7B	5.74717314	Tumor_G1
3964	COX8A	8.909891368	Tumor_G1
3965	SCO1	2.466002352	Tumor_G1
3966	SURF1	5.642343409	Tumor_G1
3967	ATP6V1E1	6.780361836	Tumor_G1
3968	DLAT	4.330508043	Tumor_G1
3969	PDC	0	Tumor_G1
3970	SUCLG2	5.777352501	Tumor_G1
3971	SLC25A5	7.618140093	Tumor_G1
3972	UCP2	8.525275451	Tumor_G1
3973	SOD2	4.704510874	Tumor_G1
3974	NOX4	3.137465862	Tumor_G1
3975	DUOX1	4.956996852	Tumor_G1
3976	DUOXA1	5.280396337	Tumor_G1
3977	DUOX2	4.198357745	Tumor_G1
3978	DUOXA2	3.179197886	Tumor_G1
3979	NDUFA1	7.759345828	Tumor_G1
3980	NDUFA2	6.919851733	Tumor_G1
3981	NDUFA8	6.825999451	Tumor_G1
3982	NDUFB1	5.503386023	Tumor_G1
3983	NDUFB10	7.596128777	Tumor_G1
3984	NDUFB2	6.29374177	Tumor_G1
3985	NDUFB3	5.746656293	Tumor_G1
3986	NDUFB7	8.765928488	Tumor_G1
3987	CYC1	6.542122847	Tumor_G1
3988	ETFB	4.675747859	Tumor_G1
3989	UQCR10	7.560695034	Tumor_G1
3990	UQCRQ	7.280752281	Tumor_G1
3991	UQCC3	3.987561151	Tumor_G1
3992	COX15	3.827957258	Tumor_G1
3993	COX5A	7.08684999	Tumor_G1
3994	COX6B1	8.820545902	Tumor_G1
3995	COX6C	6.386395586	Tumor_G1
3996	COX7A2	6.797376891	Tumor_G1
3997	COX7B	5.839216115	Tumor_G1
3998	COX8A	9.211731226	Tumor_G1

3999	SCO1	2.32114496	Tumor_G1
4000	SURF1	5.923150586	Tumor_G1
4001	ATP6V1E1	6.896379724	Tumor_G1
4002	DLAT	4.560091876	Tumor_G1
4003	PDC	0.034095883	Tumor_G1
4004	SUCLG2	5.76968526	Tumor_G1
4005	SLC25A5	7.527707036	Tumor_G1
4006	UCP2	8.444829747	Tumor_G1
4007	SOD2	4.307430455	Tumor_G1
4008	NOX4	2.268706997	Tumor_G1
4009	DUOX1	5.583033914	Tumor_G1
4010	DUOXA1	5.743658478	Tumor_G1
4011	DUOX2	5.284340636	Tumor_G1
4012	DUOXA2	3.538501806	Tumor_G1
4013	NDUFA1	7.626215773	Tumor_G1
4014	NDUFA2	6.441782343	Tumor_G1
4015	NDUFA8	6.789623717	Tumor_G1
4016	NDUFB1	5.183888773	Tumor_G1
4017	NDUFB10	7.202746152	Tumor_G1
4018	NDUFB2	6.253687357	Tumor_G1
4019	NDUFB3	5.978706429	Tumor_G1
4020	NDUFB7	8.291856104	Tumor_G1
4021	CYC1	6.632747295	Tumor_G1
4022	ETFB	4.445207736	Tumor_G1
4023	UQCR10	7.094706435	Tumor_G1
4024	UQCRQ	6.995658207	Tumor_G1
4025	UQCC3	3.841981454	Tumor_G1
4026	COX15	4.357658928	Tumor_G1
4027	COX5A	7.107100674	Tumor_G1
4028	COX6B1	8.372836899	Tumor_G1
4029	COX6C	5.980500921	Tumor_G1
4030	COX7A2	6.595020418	Tumor_G1
4031	COX7B	5.793943762	Tumor_G1
4032	COX8A	9.082412637	Tumor_G1
4033	SCO1	2.920641876	Tumor_G1
4034	SURF1	6.161391982	Tumor_G1
4035	ATP6V1E1	7.130854598	Tumor_G1
4036	DLAT	5.202023405	Tumor_G1
4037	PDC	0.049888595	Tumor_G1
4038	SUCLG2	6.203829838	Tumor_G1

4039	SLC25A5	8.45345492	Tumor_G1
4040	UCP2	8.481431776	Tumor_G1
4041	SOD2	5.422117622	Tumor_G1
4042	NOX4	2.908306331	Tumor_G1
4043	DUOX1	5.636271476	Tumor_G1
4044	DUOXA1	5.620632297	Tumor_G1
4045	DUOX2	5.14754389	Tumor_G1
4046	DUOXA2	2.998425304	Tumor_G1
4047	NDUFA1	7.679893246	Tumor_G1
4048	NDUFA2	6.391895628	Tumor_G1
4049	NDUFA8	6.69965896	Tumor_G1
4050	NDUFB1	5.117243434	Tumor_G1
4051	NDUFB10	7.128206288	Tumor_G1
4052	NDUFB2	6.282916831	Tumor_G1
4053	NDUFB3	5.80561412	Tumor_G1
4054	NDUFB7	8.279069932	Tumor_G1
4055	CYC1	6.557561908	Tumor_G1
4056	ETFB	4.787184891	Tumor_G1
4057	UQCR10	6.627212846	Tumor_G1
4058	UQCRQ	7.060948697	Tumor_G1
4059	UQCC3	4.17944902	Tumor_G1
4060	COX15	4.384056145	Tumor_G1
4061	COX5A	6.683595566	Tumor_G1
4062	COX6B1	8.502399322	Tumor_G1
4063	COX6C	5.89662002	Tumor_G1
4064	COX7A2	6.514288311	Tumor_G1
4065	COX7B	5.604914024	Tumor_G1
4066	COX8A	9.288619241	Tumor_G1
4067	SCO1	2.770032786	Tumor_G1
4068	SURF1	5.868476573	Tumor_G1
4069	ATP6V1E1	6.093842524	Tumor_G1
4070	DLAT	4.841248307	Tumor_G1
4071	PDC	0	Tumor_G1
4072	SUCLG2	5.790574721	Tumor_G1
4073	SLC25A5	7.649762856	Tumor_G1
4074	UCP2	8.175224144	Tumor_G1
4075	SOD2	4.294908103	Tumor_G1
4076	NOX4	2.587920862	Tumor_G1
4077	DUOX1	5.794629411	Tumor_G1
4078	DUOXA1	5.999615468	Tumor_G1

4079	DUOX2	6.531224387	Tumor_G1
4080	DUOXA2	3.788493779	Tumor_G1
4081	NDUFA1	7.75461919	Tumor_G1
4082	NDUFA2	6.262906837	Tumor_G1
4083	NDUFA8	6.68963248	Tumor_G1
4084	NDUFB1	5.327308039	Tumor_G1
4085	NDUFB10	6.994818771	Tumor_G1
4086	NDUFB2	6.243336714	Tumor_G1
4087	NDUFB3	6.03783669	Tumor_G1
4088	NDUFB7	8.058578066	Tumor_G1
4089	CYC1	6.427156211	Tumor_G1
4090	ETFB	4.743521365	Tumor_G1
4091	UQCR10	7.125960924	Tumor_G1
4092	UQCRQ	6.736115487	Tumor_G1
4093	UQCC3	3.490865674	Tumor_G1
4094	COX15	4.853762196	Tumor_G1
4095	COX5A	6.776302594	Tumor_G1
4096	COX6B1	8.270167239	Tumor_G1
4097	COX6C	6.098870948	Tumor_G1
4098	COX7A2	6.542112874	Tumor_G1
4099	COX7B	5.725258746	Tumor_G1
4100	COX8A	8.972604605	Tumor_G1
4101	SCO1	3.294760233	Tumor_G1
4102	SURF1	5.927908515	Tumor_G1
4103	ATP6V1E1	6.886710768	Tumor_G1
4104	DLAT	5.407711855	Tumor_G1
4105	PDC	0.129066468	Tumor_G1
4106	SUCLG2	6.181854852	Tumor_G1
4107	SLC25A5	8.198129466	Tumor_G1
4108	UCP2	8.469722988	Tumor_G1
4109	SOD2	5.961984645	Tumor_G1
4110	NOX4	1.892754978	Tumor_G1
4111	DUOX1	6.494785404	Tumor_G1
4112	DUOXA1	6.597844918	Tumor_G1
4113	DUOX2	7.199914278	Tumor_G1
4114	DUOXA2	4.195337057	Tumor_G1
4115	NDUFA1	7.392030164	Tumor_G1
4116	NDUFA2	5.990398152	Tumor_G1
4117	NDUFA8	6.674991619	Tumor_G1
4118	NDUFB1	4.8916281	Tumor_G1

4119	NDUFB10	7.162683944	Tumor_G1
4120	NDUFB2	5.600798169	Tumor_G1
4121	NDUFB3	5.661787112	Tumor_G1
4122	NDUFB7	8.266164553	Tumor_G1
4123	CYC1	6.334666718	Tumor_G1
4124	ETFB	4.690190642	Tumor_G1
4125	UQCR10	7.193420021	Tumor_G1
4126	UQCRQ	6.652234587	Tumor_G1
4127	UQCC3	3.686010115	Tumor_G1
4128	COX15	4.624160046	Tumor_G1
4129	COX5A	6.385122531	Tumor_G1
4130	COX6B1	8.617669166	Tumor_G1
4131	COX6C	5.548876318	Tumor_G1
4132	COX7A2	6.22849605	Tumor_G1
4133	COX7B	5.618945887	Tumor_G1
4134	COX8A	8.770774055	Tumor_G1
4135	SCO1	2.993301621	Tumor_G1
4136	SURF1	6.030248296	Tumor_G1
4137	ATP6V1E1	6.804504606	Tumor_G1
4138	DLAT	4.660217533	Tumor_G1
4139	PDC	0.136638916	Tumor_G1
4140	SUCLG2	5.763269247	Tumor_G1
4141	SLC25A5	7.712824259	Tumor_G1
4142	UCP2	7.887343863	Tumor_G1
4143	SOD2	4.831310195	Tumor_G1
4144	NOX4	2.658390478	Tumor_G1
4145	DUOX1	6.566881065	Tumor_G1
4146	DUOXA1	6.440129357	Tumor_G1
4147	DUOX2	6.133687551	Tumor_G1
4148	DUOXA2	3.961898685	Tumor_G1
4149	NDUFA1	7.625511447	Tumor_G1
4150	NDUFA2	6.847207007	Tumor_G1
4151	NDUFA8	6.842083763	Tumor_G1
4152	NDUFB1	5.386980188	Tumor_G1
4153	NDUFB10	7.204002053	Tumor_G1
4154	NDUFB2	6.481777973	Tumor_G1
4155	NDUFB3	6.27202329	Tumor_G1
4156	NDUFB7	8.486139878	Tumor_G1
4157	CYC1	6.80668351	Tumor_G1
4158	ETFB	4.622878305	Tumor_G1

4159	UQCR10	7.43262301	Tumor_G1
4160	UQCRQ	7.204748758	Tumor_G1
4161	UQCC3	3.888925529	Tumor_G1
4162	COX15	4.677613222	Tumor_G1
4163	COX5A	6.949297754	Tumor_G1
4164	COX6B1	8.665005062	Tumor_G1
4165	COX6C	6.30737568	Tumor_G1
4166	COX7A2	6.930567638	Tumor_G1
4167	COX7B	6.10831709	Tumor_G1
4168	COX8A	9.280491665	Tumor_G1
4169	SCO1	2.876719402	Tumor_G1
4170	SURF1	5.839990844	Tumor_G1
4171	ATP6V1E1	7.403887276	Tumor_G1
4172	DLAT	5.180138134	Tumor_G1
4173	PDC	0.111639377	Tumor_G1
4174	SUCLG2	6.560190427	Tumor_G1
4175	SLC25A5	8.544383116	Tumor_G1
4176	UCP2	8.001200277	Tumor_G1
4177	SOD2	6.101326401	Tumor_G1
4178	NOX4	3.069669378	Tumor_G1
4179	DUOX1	5.280893209	Tumor_G1
4180	DUOXA1	5.772470448	Tumor_G1
4181	DUOX2	5.124462069	Tumor_G1
4182	DUOXA2	3.310518802	Tumor_G1
4183	NDUFA1	7.457460201	Tumor_G1
4184	NDUFA2	6.356837184	Tumor_G1
4185	NDUFA8	6.725977294	Tumor_G1
4186	NDUFB1	4.885786765	Tumor_G1
4187	NDUFB10	7.056184553	Tumor_G1
4188	NDUFB2	6.206034393	Tumor_G1
4189	NDUFB3	5.846167931	Tumor_G1
4190	NDUFB7	8.139478891	Tumor_G1
4191	CYC1	6.461405324	Tumor_G1
4192	ETFB	4.444523931	Tumor_G1
4193	UQCR10	7.080498423	Tumor_G1
4194	UQCRQ	6.637010578	Tumor_G1
4195	UQCC3	3.934216582	Tumor_G1
4196	COX15	4.965021579	Tumor_G1
4197	COX5A	6.678823767	Tumor_G1
4198	COX6B1	8.068196638	Tumor_G1

4199	COX6C	6.024854479	Tumor_G1
4200	COX7A2	6.449126505	Tumor_G1
4201	COX7B	5.71682563	Tumor_G1
4202	COX8A	9.038121522	Tumor_G1
4203	SCO1	3.327917418	Tumor_G1
4204	SURF1	5.849557719	Tumor_G1
4205	ATP6V1E1	7.012799145	Tumor_G1
4206	DLAT	5.639548774	Tumor_G1
4207	PDC	0.121378788	Tumor_G1
4208	SUCLG2	6.080978222	Tumor_G1
4209	SLC25A5	8.33971903	Tumor_G1
4210	UCP2	7.707422055	Tumor_G1
4211	SOD2	5.344122049	Tumor_G1
4212	NOX4	2.413676061	Tumor_G1
4213	DUOX1	6.300937235	Tumor_G1
4214	DUOXA1	6.628344577	Tumor_G1
4215	DUOX2	6.937519177	Tumor_G1
4216	DUOXA2	4.823317957	Tumor_G1
4217	NDUFA1	7.458600612	Tumor_G1
4218	NDUFA2	6.515622539	Tumor_G1
4219	NDUFA8	6.85425595	Tumor_G1
4220	NDUFB1	5.106450314	Tumor_G1
4221	NDUFB10	7.275558399	Tumor_G1
4222	NDUFB2	5.911676758	Tumor_G1
4223	NDUFB3	5.890465054	Tumor_G1
4224	NDUFB7	8.332264445	Tumor_G1
4225	CYC1	6.808556852	Tumor_G1
4226	ETFB	4.835256893	Tumor_G1
4227	UQCR10	7.372195584	Tumor_G1
4228	UQCRQ	6.849630835	Tumor_G1
4229	UQCC3	4.135364059	Tumor_G1
4230	COX15	4.871243145	Tumor_G1
4231	COX5A	6.815862922	Tumor_G1
4232	COX6B1	8.476384678	Tumor_G1
4233	COX6C	6.04596825	Tumor_G1
4234	COX7A2	6.509527545	Tumor_G1
4235	COX7B	5.769878	Tumor_G1
4236	COX8A	8.955571206	Tumor_G1
4237	SCO1	3.237074048	Tumor_G1
4238	SURF1	6.205922456	Tumor_G1

4239	ATP6V1E1	6.828553442	Tumor_G1
4240	DLAT	5.137683028	Tumor_G1
4241	PDC	0.109197301	Tumor_G1
4242	SUCLG2	6.064570813	Tumor_G1
4243	SLC25A5	8.112181791	Tumor_G1
4244	UCP2	8.486142112	Tumor_G1
4245	SOD2	6.258281707	Tumor_G1
4246	NOX4	3.257963646	Tumor_G1
4247	DUOX1	6.330298515	Tumor_G1
4248	DUOXA1	6.707167496	Tumor_G1
4249	DUOX2	6.696085623	Tumor_G1
4250	DUOXA2	4.702812484	Tumor_G1
4251	NDUFA1	8.169744991	Tumor_G1
4252	NDUFA2	5.968918498	Tumor_G1
4253	NDUFA8	6.877839246	Tumor_G1
4254	NDUFB1	5.663607767	Tumor_G1
4255	NDUFB10	7.131873727	Tumor_G1
4256	NDUFB2	5.454765279	Tumor_G1
4257	NDUFB3	6.467448664	Tumor_G1
4258	NDUFB7	8.179860537	Tumor_G1
4259	CYC1	6.276061446	Tumor_G1
4260	ETFB	4.498277854	Tumor_G1
4261	UQCR10	7.72920794	Tumor_G1
4262	UQCRQ	7.007489896	Tumor_G1
4263	UQCC3	3.688431334	Tumor_G1
4264	COX15	4.701923741	Tumor_G1
4265	COX5A	6.874165449	Tumor_G1
4266	COX6B1	8.678144385	Tumor_G1
4267	COX6C	5.963514665	Tumor_G1
4268	COX7A2	6.813888702	Tumor_G1
4269	COX7B	6.313626146	Tumor_G1
4270	COX8A	8.33377463	Tumor_G1
4271	SCO1	3.141053641	Tumor_G1
4272	SURF1	5.94742519	Tumor_G1
4273	ATP6V1E1	6.627844762	Tumor_G1
4274	DLAT	4.631367477	Tumor_G1
4275	PDC	0.044172376	Tumor_G1
4276	SUCLG2	5.698063348	Tumor_G1
4277	SLC25A5	7.474973352	Tumor_G1
4278	UCP2	8.818212975	Tumor_G1

4279	SOD2	5.351057166	Tumor_G1
4280	NOX4	1.826736004	Tumor_G1
4281	DUOX1	5.857196344	Tumor_G1
4282	DUOXA1	6.375407214	Tumor_G1
4283	DUOX2	6.748935868	Tumor_G1
4284	DUOXA2	4.923968607	Tumor_G1
4285	NDUFA1	7.690273109	Tumor_G1
4286	NDUFA2	6.624842936	Tumor_G1
4287	NDUFA8	7.006615755	Tumor_G1
4288	NDUFB1	5.297733463	Tumor_G1
4289	NDUFB10	7.696348051	Tumor_G1
4290	NDUFB2	6.188546392	Tumor_G1
4291	NDUFB3	6.05169281	Tumor_G1
4292	NDUFB7	8.490024148	Tumor_G1
4293	CYC1	6.882285873	Tumor_G1
4294	ETFB	4.659511269	Tumor_G1
4295	UQCR10	7.600554943	Tumor_G1
4296	UQCRQ	7.340243062	Tumor_G1
4297	UQCC3	4.41805393	Tumor_G1
4298	COX15	4.882226379	Tumor_G1
4299	COX5A	7.150842277	Tumor_G1
4300	COX6B1	8.642931255	Tumor_G1
4301	COX6C	6.120298814	Tumor_G1
4302	COX7A2	6.671201082	Tumor_G1
4303	COX7B	5.763220817	Tumor_G1
4304	COX8A	9.280322702	Tumor_G1
4305	SCO1	3.552663007	Tumor_G1
4306	SURF1	6.177135432	Tumor_G1
4307	ATP6V1E1	6.902725355	Tumor_G1
4308	DLAT	5.281827672	Tumor_G1
4309	PDC	0.140795941	Tumor_G1
4310	SUCLG2	6.003297031	Tumor_G1
4311	SLC25A5	8.493662421	Tumor_G1
4312	UCP2	7.788716237	Tumor_G1
4313	SOD2	5.194968922	Tumor_G1
4314	NOX4	4.157278759	Tumor_G1
4315	DUOX1	5.395154348	Tumor_G1
4316	DUOXA1	5.665201104	Tumor_G1
4317	DUOX2	4.052358295	Tumor_G1
4318	DUOXA2	2.484193	Tumor_G1

4319	NDUFA1	7.270321375	Tumor_G1
4320	NDUFA2	6.618900888	Tumor_G1
4321	NDUFA8	6.970180016	Tumor_G1
4322	NDUFB1	4.910890235	Tumor_G1
4323	NDUFB10	7.281856555	Tumor_G1
4324	NDUFB2	6.285222148	Tumor_G1
4325	NDUFB3	5.92168454	Tumor_G1
4326	NDUFB7	8.395116247	Tumor_G1
4327	CYC1	6.780088258	Tumor_G1
4328	ETFB	4.573640952	Tumor_G1
4329	UQCR10	7.277925386	Tumor_G1
4330	UQCRQ	6.968763964	Tumor_G1
4331	UQCC3	4.136665766	Tumor_G1
4332	COX15	4.740169247	Tumor_G1
4333	COX5A	7.176399329	Tumor_G1
4334	COX6B1	8.446943855	Tumor_G1
4335	COX6C	5.752536206	Tumor_G1
4336	COX7A2	6.531396149	Tumor_G1
4337	COX7B	5.726593357	Tumor_G1
4338	COX8A	9.188298929	Tumor_G1
4339	SCO1	3.201037632	Tumor_G1
4340	SURF1	6.476172718	Tumor_G1
4341	ATP6V1E1	7.349918668	Tumor_G1
4342	DLAT	5.819218561	Tumor_G1
4343	PDC	0.11658513	Tumor_G1
4344	SUCLG2	6.233394636	Tumor_G1
4345	SLC25A5	8.733252374	Tumor_G1
4346	UCP2	9.574959075	Tumor_G1
4347	SOD2	5.019209292	Tumor_G1
4348	NOX4	3.539793754	Tumor_G1
4349	DUOX1	5.987390505	Tumor_G1
4350	DUOXA1	5.913767848	Tumor_G1
4351	DUOX2	5.023034655	Tumor_G1
4352	DUOXA2	3.959915775	Tumor_G1
4353	NDUFA1	7.531863697	Tumor_G1
4354	NDUFA2	6.662280254	Tumor_G1
4355	NDUFA8	6.799766461	Tumor_G1
4356	NDUFB1	5.216322489	Tumor_G1
4357	NDUFB10	7.060057184	Tumor_G1
4358	NDUFB2	6.106174014	Tumor_G1

4359	NDUFB3	5.988983055	Tumor_G1
4360	NDUFB7	8.069076421	Tumor_G1
4361	CYC1	6.465307767	Tumor_G1
4362	ETFB	4.232971671	Tumor_G1
4363	UQCR10	7.302898179	Tumor_G1
4364	UQCRQ	6.922697376	Tumor_G1
4365	UQCC3	3.675876747	Tumor_G1
4366	COX15	4.361090747	Tumor_G1
4367	COX5A	6.892742295	Tumor_G1
4368	COX6B1	8.373388665	Tumor_G1
4369	COX6C	6.091327188	Tumor_G1
4370	COX7A2	6.744247603	Tumor_G1
4371	COX7B	5.903489355	Tumor_G1
4372	COX8A	8.89138445	Tumor_G1
4373	SCO1	2.849398178	Tumor_G1
4374	SURF1	5.923548374	Tumor_G1
4375	ATP6V1E1	6.896811807	Tumor_G1
4376	DLAT	5.114685072	Tumor_G1
4377	PDC	0.106828573	Tumor_G1
4378	SUCLG2	5.72582242	Tumor_G1
4379	SLC25A5	8.092757939	Tumor_G1
4380	UCP2	7.800652039	Tumor_G1
4381	SOD2	6.760100805	Tumor_G1
4382	NOX4	2.689953318	Tumor_G1
4383	DUOX1	5.098526784	Tumor_G1
4384	DUOXA1	5.409860984	Tumor_G1
4385	DUOX2	4.393667588	Tumor_G1
4386	DUOXA2	2.519708387	Tumor_G1
4387	NDUFA1	7.646819489	Tumor_G1
4388	NDUFA2	6.45644746	Tumor_G1
4389	NDUFA8	6.599485778	Tumor_G1
4390	NDUFB1	5.165842236	Tumor_G1
4391	NDUFB10	7.181226298	Tumor_G1
4392	NDUFB2	5.895909616	Tumor_G1
4393	NDUFB3	5.611768989	Tumor_G1
4394	NDUFB7	8.338416402	Tumor_G1
4395	CYC1	6.270082891	Tumor_G1
4396	ETFB	4.136417191	Tumor_G1
4397	UQCR10	7.166852492	Tumor_G1
4398	UQCRQ	6.968289375	Tumor_G1

4399	UQCC3	3.808614353	Tumor_G1
4400	COX15	3.880543535	Tumor_G1
4401	COX5A	6.833529523	Tumor_G1
4402	COX6B1	8.290833424	Tumor_G1
4403	COX6C	5.772096711	Tumor_G1
4404	COX7A2	6.365647607	Tumor_G1
4405	COX7B	5.526514271	Tumor_G1
4406	COX8A	8.877447941	Tumor_G1
4407	SCO1	2.597444971	Tumor_G1
4408	SURF1	5.735216346	Tumor_G1
4409	ATP6V1E1	6.48599107	Tumor_G1
4410	DLAT	4.392707752	Tumor_G1
4411	PDC	0.141580078	Tumor_G1
4412	SUCLG2	5.435271777	Tumor_G1
4413	SLC25A5	7.554392902	Tumor_G1
4414	UCP2	7.377227647	Tumor_G1
4415	SOD2	6.334478305	Tumor_G1
4416	NOX4	1.279103768	Tumor_G1
4417	DUOX1	4.800510223	Tumor_G1
4418	DUOXA1	5.025754128	Tumor_G1
4419	DUOX2	4.162207424	Tumor_G1
4420	DUOXA2	2.507198603	Tumor_G1
4421	NDUFA1	7.570752024	Tumor_G1
4422	NDUFA2	6.587163937	Tumor_G1
4423	NDUFA8	6.815102262	Tumor_G1
4424	NDUFB1	4.967179521	Tumor_G1
4425	NDUFB10	7.521226336	Tumor_G1
4426	NDUFB2	6.459789811	Tumor_G1
4427	NDUFB3	5.729542149	Tumor_G1
4428	NDUFB7	7.849619213	Tumor_G1
4429	CYC1	6.692732412	Tumor_G1
4430	ETFB	4.530844033	Tumor_G1
4431	UQCR10	7.377207516	Tumor_G1
4432	UQCRQ	6.877813517	Tumor_G1
4433	UQCC3	3.996726536	Tumor_G1
4434	COX15	4.630700592	Tumor_G1
4435	COX5A	6.974559494	Tumor_G1
4436	COX6B1	8.549906651	Tumor_G1
4437	COX6C	5.872242297	Tumor_G1
4438	COX7A2	6.517617076	Tumor_G1

4439	COX7B	5.575937997	Tumor_G1
4440	COX8A	9.093231356	Tumor_G1
4441	SCO1	2.882206952	Tumor_G1
4442	SURF1	6.00727511	Tumor_G1
4443	ATP6V1E1	7.046692875	Tumor_G1
4444	DLAT	5.251081333	Tumor_G1
4445	PDC	0.09265756	Tumor_G1
4446	SUCLG2	6.323541522	Tumor_G1
4447	SLC25A5	7.697414819	Tumor_G1
4448	UCP2	7.279698182	Tumor_G1
4449	SOD2	6.499325612	Tumor_G1
4450	NOX4	1.927800454	Tumor_G1
4451	DUOX1	5.754737038	Tumor_G1
4452	DUOXA1	6.063000089	Tumor_G1
4453	DUOX2	5.512655646	Tumor_G1
4454	DUOXA2	3.581593983	Tumor_G1
4455	NDUFA1	7.788466273	Tumor_G1
4456	NDUFA2	6.682233823	Tumor_G1
4457	NDUFA8	6.688330435	Tumor_G1
4458	NDUFB1	5.61776818	Tumor_G1
4459	NDUFB10	7.316204831	Tumor_G1
4460	NDUFB2	6.312078907	Tumor_G1
4461	NDUFB3	6.117679488	Tumor_G1
4462	NDUFB7	8.443427021	Tumor_G1
4463	CYC1	6.637264201	Tumor_G1
4464	ETFB	4.899435511	Tumor_G1
4465	UQCR10	7.547316355	Tumor_G1
4466	UQCRQ	7.194123356	Tumor_G1
4467	UQCC3	4.154231826	Tumor_G1
4468	COX15	4.2082815	Tumor_G1
4469	COX5A	6.984567023	Tumor_G1
4470	COX6B1	8.665585142	Tumor_G1
4471	COX6C	6.19852908	Tumor_G1
4472	COX7A2	6.839991003	Tumor_G1
4473	COX7B	5.801564451	Tumor_G1
4474	COX8A	9.324138912	Tumor_G1
4475	SCO1	2.837132357	Tumor_G1
4476	SURF1	5.841922857	Tumor_G1
4477	ATP6V1E1	7.073856202	Tumor_G1
4478	DLAT	4.515775123	Tumor_G1

4479	PDC	0	Tumor_G1
4480	SUCLG2	6.003035377	Tumor_G1
4481	SLC25A5	7.574417132	Tumor_G1
4482	UCP2	7.735784018	Tumor_G1
4483	SOD2	5.263805992	Tumor_G1
4484	NOX4	1.987106608	Tumor_G1
4485	DUOX1	5.496416035	Tumor_G1
4486	DUOXA1	6.011555817	Tumor_G1
4487	DUOX2	4.955998499	Tumor_G1
4488	DUOXA2	3.332878754	Tumor_G1
4489	NDUFA1	7.551669392	Tumor_G1
4490	NDUFA2	6.232266104	Tumor_G1
4491	NDUFA8	6.950922904	Tumor_G1
4492	NDUFB1	5.27615842	Tumor_G1
4493	NDUFB10	8.260156145	Tumor_G1
4494	NDUFB2	6.050250645	Tumor_G1
4495	NDUFB3	5.826488978	Tumor_G1
4496	NDUFB7	8.499547136	Tumor_G1
4497	CYC1	6.281962207	Tumor_G1
4498	ETFB	4.723496473	Tumor_G1
4499	UQCR10	7.340080697	Tumor_G1
4500	UQCRQ	7.087825949	Tumor_G1
4501	UQCC3	3.467593563	Tumor_G1
4502	COX15	4.175840802	Tumor_G1
4503	COX5A	7.300022472	Tumor_G1
4504	COX6B1	8.993004623	Tumor_G1
4505	COX6C	5.862102561	Tumor_G1
4506	COX7A2	6.532709856	Tumor_G1
4507	COX7B	5.988723208	Tumor_G1
4508	COX8A	9.126560273	Tumor_G1
4509	SCO1	2.004148503	Tumor_G1
4510	SURF1	6.197217614	Tumor_G1
4511	ATP6V1E1	6.842526131	Tumor_G1
4512	DLAT	4.262461666	Tumor_G1
4513	PDC	0.190493424	Tumor_G1
4514	SUCLG2	5.58791595	Tumor_G1
4515	SLC25A5	8.121976516	Tumor_G1
4516	UCP2	8.797691561	Tumor_G1
4517	SOD2	4.488693627	Tumor_G1
4518	NOX4	2.762892743	Tumor_G1

4519	DUOX1	5.252625998	Tumor_G1
4520	DUOXA1	5.719035766	Tumor_G1
4521	DUOX2	4.026123795	Tumor_G1
4522	DUOXA2	2.021058363	Tumor_G1
4523	NDUFA1	7.481232489	Tumor_G1
4524	NDUFA2	5.814216918	Tumor_G1
4525	NDUFA8	6.423777437	Tumor_G1
4526	NDUFB1	4.901603151	Tumor_G1
4527	NDUFB10	6.878123437	Tumor_G1
4528	NDUFB2	5.381957226	Tumor_G1
4529	NDUFB3	5.648063706	Tumor_G1
4530	NDUFB7	7.898451354	Tumor_G1
4531	CYC1	6.074242403	Tumor_G1
4532	ETFB	4.105502852	Tumor_G1
4533	UQCR10	6.965651772	Tumor_G1
4534	UQCRQ	6.462710003	Tumor_G1
4535	UQCC3	3.276175332	Tumor_G1
4536	COX15	4.73038658	Tumor_G1
4537	COX5A	6.286867095	Tumor_G1
4538	COX6B1	8.214397551	Tumor_G1
4539	COX6C	5.490771618	Tumor_G1
4540	COX7A2	5.885561704	Tumor_G1
4541	COX7B	5.747800463	Tumor_G1
4542	COX8A	8.69140485	Tumor_G1
4543	SCO1	3.013314837	Tumor_G1
4544	SURF1	5.455670065	Tumor_G1
4545	ATP6V1E1	6.597865148	Tumor_G1
4546	DLAT	5.089695513	Tumor_G1
4547	PDC	0.140597496	Tumor_G1
4548	SUCLG2	5.543589384	Tumor_G1
4549	SLC25A5	7.823740398	Tumor_G1
4550	UCP2	7.347244022	Tumor_G1
4551	SOD2	6.443459597	Tumor_G1
4552	NOX4	1.86467694	Tumor_G1
4553	DUOX1	6.047221686	Tumor_G1
4554	DUOXA1	6.213165792	Tumor_G1
4555	DUOX2	6.019146164	Tumor_G1
4556	DUOXA2	3.515759643	Tumor_G1
4557	NDUFA1	7.480505861	Tumor_G1
4558	NDUFA2	6.150239883	Tumor_G1

4559	NDUFA8	6.705819528	Tumor_G1
4560	NDUFB1	5.024880813	Tumor_G1
4561	NDUFB10	7.389665434	Tumor_G1
4562	NDUFB2	6.252860747	Tumor_G1
4563	NDUFB3	5.837197173	Tumor_G1
4564	NDUFB7	8.18203768	Tumor_G1
4565	CYC1	6.656156244	Tumor_G1
4566	ETFB	4.439038234	Tumor_G1
4567	UQCR10	7.059395054	Tumor_G1
4568	UQCRQ	6.754830527	Tumor_G1
4569	UQCC3	4.016925579	Tumor_G1
4570	COX15	4.967295741	Tumor_G1
4571	COX5A	7.029183614	Tumor_G1
4572	COX6B1	8.386929543	Tumor_G1
4573	COX6C	5.676776761	Tumor_G1
4574	COX7A2	6.515746759	Tumor_G1
4575	COX7B	5.806900769	Tumor_G1
4576	COX8A	9.196439243	Tumor_G1
4577	SCO1	3.42942346	Tumor_G1
4578	SURF1	6.107607059	Tumor_G1
4579	ATP6V1E1	7.006640526	Tumor_G1
4580	DLAT	5.352480743	Tumor_G1
4581	PDC	0.089775963	Tumor_G1
4582	SUCLG2	6.202124319	Tumor_G1
4583	SLC25A5	8.757176282	Tumor_G1
4584	UCP2	8.13723681	Tumor_G1
4585	SOD2	4.65937357	Tumor_G1
4586	NOX4	1.463697257	Tumor_G1
4587	DUOX1	5.951234434	Tumor_G1
4588	DUOXA1	6.328314041	Tumor_G1
4589	DUOX2	6.742155902	Tumor_G1
4590	DUOXA2	4.175218175	Tumor_G1
4591	NDUFA1	7.630635721	Tumor_G1
4592	NDUFA2	6.654563111	Tumor_G1
4593	NDUFA8	6.740879441	Tumor_G1
4594	NDUFB1	5.339773299	Tumor_G1
4595	NDUFB10	7.344564831	Tumor_G1
4596	NDUFB2	6.374309069	Tumor_G1
4597	NDUFB3	6.270793417	Tumor_G1
4598	NDUFB7	8.219968825	Tumor_G1

4599	CYC1	6.759978375	Tumor_G1
4600	ETFB	4.767861814	Tumor_G1
4601	UQCR10	7.537120092	Tumor_G1
4602	UQCRQ	7.180659215	Tumor_G1
4603	UQCC3	4.113513215	Tumor_G1
4604	COX15	4.730832236	Tumor_G1
4605	COX5A	7.152284396	Tumor_G1
4606	COX6B1	8.432466436	Tumor_G1
4607	COX6C	6.174875955	Tumor_G1
4608	COX7A2	6.789132565	Tumor_G1
4609	COX7B	5.789968869	Tumor_G1
4610	COX8A	9.254203745	Tumor_G1
4611	SCO1	3.184886161	Tumor_G1
4612	SURF1	6.209287472	Tumor_G1
4613	ATP6V1E1	7.252214558	Tumor_G1
4614	DLAT	5.450131702	Tumor_G1
4615	PDC	0.257466423	Tumor_G1
4616	SUCLG2	6.558527681	Tumor_G1
4617	SLC25A5	8.175236934	Tumor_G1
4618	UCP2	8.590923699	Tumor_G1
4619	SOD2	5.999153765	Tumor_G1
4620	NOX4	3.503672567	Tumor_G1
4621	DUOX1	6.060591197	Tumor_G1
4622	DUOXA1	6.052787073	Tumor_G1
4623	DUOX2	5.272954111	Tumor_G1
4624	DUOXA2	3.343488428	Tumor_G1
4625	NDUFA1	7.472015106	Tumor_G1
4626	NDUFA2	6.376771762	Tumor_G1
4627	NDUFA8	6.433993897	Tumor_G1
4628	NDUFB1	5.089042658	Tumor_G1
4629	NDUFB10	7.046716089	Tumor_G1
4630	NDUFB2	5.742845148	Tumor_G1
4631	NDUFB3	5.531334248	Tumor_G1
4632	NDUFB7	8.130968138	Tumor_G1
4633	CYC1	6.219907953	Tumor_G1
4634	ETFB	4.078850768	Tumor_G1
4635	UQCR10	7.036298365	Tumor_G1
4636	UQCRQ	6.686830492	Tumor_G1
4637	UQCC3	3.86959657	Tumor_G1
4638	COX15	3.979711665	Tumor_G1

4639	COX5A	6.69728249	Tumor_G1
4640	COX6B1	8.262582944	Tumor_G1
4641	COX6C	5.760949758	Tumor_G1
4642	COX7A2	6.119348888	Tumor_G1
4643	COX7B	5.308584148	Tumor_G1
4644	COX8A	8.738803559	Tumor_G1
4645	SCO1	2.364045433	Tumor_G1
4646	SURF1	5.715772143	Tumor_G1
4647	ATP6V1E1	6.250402747	Tumor_G1
4648	DLAT	4.206242935	Tumor_G1
4649	PDC	0.05305669	Tumor_G1
4650	SUCLG2	5.074385692	Tumor_G1
4651	SLC25A5	7.201955003	Tumor_G1
4652	UCP2	7.095651601	Tumor_G1
4653	SOD2	6.061027071	Tumor_G1
4654	NOX4	2.163686214	Tumor_G1
4655	DUOX1	4.986622696	Tumor_G1
4656	DUOXA1	5.121921337	Tumor_G1
4657	DUOX2	3.81537966	Tumor_G1
4658	DUOXA2	2.221199751	Tumor_G1
4659	NDUFA1	8.04298901	Tumor_G1
4660	NDUFA2	7.027049904	Tumor_G1
4661	NDUFA8	6.821455421	Tumor_G1
4662	NDUFB1	5.767457512	Tumor_G1
4663	NDUFB10	7.259619347	Tumor_G1
4664	NDUFB2	6.228401009	Tumor_G1
4665	NDUFB3	5.997732539	Tumor_G1
4666	NDUFB7	8.527806374	Tumor_G1
4667	CYC1	6.701106572	Tumor_G1
4668	ETFB	4.547786377	Tumor_G1
4669	UQCR10	7.456281541	Tumor_G1
4670	UQCRQ	7.266919766	Tumor_G1
4671	UQCC3	4.202639234	Tumor_G1
4672	COX15	4.192935433	Tumor_G1
4673	COX5A	6.840950052	Tumor_G1
4674	COX6B1	8.812807271	Tumor_G1
4675	COX6C	6.51772723	Tumor_G1
4676	COX7A2	6.83257024	Tumor_G1
4677	COX7B	5.864703641	Tumor_G1
4678	COX8A	8.768900432	Tumor_G1

4679	SCO1	2.500537117	Tumor_G1
4680	SURF1	6.151947235	Tumor_G1
4681	ATP6V1E1	6.699537525	Tumor_G1
4682	DLAT	4.299345662	Tumor_G1
4683	PDC	0.132770309	Tumor_G1
4684	SUCLG2	5.564536503	Tumor_G1
4685	SLC25A5	7.773710233	Tumor_G1
4686	UCP2	6.66186215	Tumor_G1
4687	SOD2	4.934595191	Tumor_G1
4688	NOX4	2.628141904	Tumor_G1
4689	DUOX1	5.005192974	Tumor_G1
4690	DUOXA1	5.289929332	Tumor_G1
4691	DUOX2	4.403985116	Tumor_G1
4692	DUOXA2	2.765387365	Tumor_G1
4693	NDUFA1	7.720429657	Tumor_G1
4694	NDUFA2	6.83139836	Tumor_G1
4695	NDUFA8	6.805119805	Tumor_G1
4696	NDUFB1	5.335265899	Tumor_G1
4697	NDUFB10	7.471419698	Tumor_G1
4698	NDUFB2	6.476735583	Tumor_G1
4699	NDUFB3	5.652451709	Tumor_G1
4700	NDUFB7	8.365125883	Tumor_G1
4701	CYC1	6.709961528	Tumor_G1
4702	ETFB	4.657208764	Tumor_G1
4703	UQCR10	7.420213915	Tumor_G1
4704	UQCRQ	6.812471732	Tumor_G1
4705	UQCC3	4.531999386	Tumor_G1
4706	COX15	4.875443985	Tumor_G1
4707	COX5A	6.772073149	Tumor_G1
4708	COX6B1	8.52568417	Tumor_G1
4709	COX6C	6.213089924	Tumor_G1
4710	COX7A2	6.601603856	Tumor_G1
4711	COX7B	5.847608956	Tumor_G1
4712	COX8A	9.450409891	Tumor_G1
4713	SCO1	2.987235109	Tumor_G1
4714	SURF1	5.830882945	Tumor_G1
4715	ATP6V1E1	6.92877351	Tumor_G1
4716	DLAT	4.969407738	Tumor_G1
4717	PDC	0	Tumor_G1
4718	SUCLG2	6.158962055	Tumor_G1

4719	SLC25A5	8.21057303	Tumor_G1
4720	UCP2	7.800610146	Tumor_G1
4721	SOD2	4.856053599	Tumor_G1
4722	NOX4	1.502386152	Tumor_G1
4723	DUOX1	6.222816097	Tumor_G1
4724	DUOXA1	6.388194049	Tumor_G1
4725	DUOX2	6.182980358	Tumor_G1
4726	DUOXA2	4.352979477	Tumor_G1
4727	NDUFA1	7.955253229	Tumor_G1
4728	NDUFA2	6.578181484	Tumor_G1
4729	NDUFA8	6.608504478	Tumor_G1
4730	NDUFB1	5.767443986	Tumor_G1
4731	NDUFB10	7.060110945	Tumor_G1
4732	NDUFB2	6.867428585	Tumor_G1
4733	NDUFB3	6.255034677	Tumor_G1
4734	NDUFB7	8.572212193	Tumor_G1
4735	CYC1	6.661403293	Tumor_G1
4736	ETFB	4.59952464	Tumor_G1
4737	UQCR10	7.511508735	Tumor_G1
4738	UQCRQ	7.2425072	Tumor_G1
4739	UQCC3	4.014986196	Tumor_G1
4740	COX15	4.762484832	Tumor_G1
4741	COX5A	6.846159752	Tumor_G1
4742	COX6B1	8.295500512	Tumor_G1
4743	COX6C	6.194207217	Tumor_G1
4744	COX7A2	7.178976698	Tumor_G1
4745	COX7B	5.982555417	Tumor_G1
4746	COX8A	9.110571231	Tumor_G1
4747	SCO1	3.132815113	Tumor_G1
4748	SURF1	5.989117299	Tumor_G1
4749	ATP6V1E1	6.731010935	Tumor_G1
4750	DLAT	5.074705058	Tumor_G1
4751	PDC	0.132073716	Tumor_G1
4752	SUCLG2	6.211965991	Tumor_G1
4753	SLC25A5	7.916746555	Tumor_G1
4754	UCP2	7.500366436	Tumor_G1
4755	SOD2	4.740495096	Tumor_G1
4756	NOX4	0.514168258	Tumor_G1
4757	DUOX1	6.161963441	Tumor_G1
4758	DUOXA1	6.062998435	Tumor_G1

4759	DUOX2	6.441933202	Tumor_G1
4760	DUOXA2	4.203503365	Tumor_G1
4761	NDUFA1	7.747432562	Tumor_G1
4762	NDUFA2	6.953305075	Tumor_G1
4763	NDUFA8	7.148547913	Tumor_G1
4764	NDUFB1	5.532087259	Tumor_G1
4765	NDUFB10	7.37261488	Tumor_G1
4766	NDUFB2	6.503187644	Tumor_G1
4767	NDUFB3	6.305342561	Tumor_G1
4768	NDUFB7	8.840708	Tumor_G1
4769	CYC1	6.94935479	Tumor_G1
4770	ETFB	4.916455868	Tumor_G1
4771	UQCR10	7.673815567	Tumor_G1
4772	UQCRQ	7.233363893	Tumor_G1
4773	UQCC3	4.181365761	Tumor_G1
4774	COX15	4.515658122	Tumor_G1
4775	COX5A	6.945607721	Tumor_G1
4776	COX6B1	8.693647762	Tumor_G1
4777	COX6C	6.392547278	Tumor_G1
4778	COX7A2	6.968854387	Tumor_G1
4779	COX7B	5.955460204	Tumor_G1
4780	COX8A	9.251712042	Tumor_G1
4781	SCO1	3.02252295	Tumor_G1
4782	SURF1	6.065748517	Tumor_G1
4783	ATP6V1E1	7.269135308	Tumor_G1
4784	DLAT	5.02609408	Tumor_G1
4785	PDC	0.253974174	Tumor_G1
4786	SUCLG2	6.354478815	Tumor_G1
4787	SLC25A5	8.111713804	Tumor_G1
4788	UCP2	7.596407965	Tumor_G1
4789	SOD2	4.820953951	Tumor_G1
4790	NOX4	3.014703898	Tumor_G1
4791	DUOX1	5.65653636	Tumor_G1
4792	DUOXA1	6.216996052	Tumor_G1
4793	DUOX2	5.835546606	Tumor_G1
4794	DUOXA2	3.914359274	Tumor_G1
4795	NDUFA1	8.211222161	Tumor_G1
4796	NDUFA2	6.662494022	Tumor_G1
4797	NDUFA8	7.116294405	Tumor_G1
4798	NDUFB1	5.781577721	Tumor_G1

4799	NDUFB10	7.224814864	Tumor_G1
4800	NDUFB2	6.438990154	Tumor_G1
4801	NDUFB3	6.558978826	Tumor_G1
4802	NDUFB7	8.451895864	Tumor_G1
4803	CYC1	6.742415847	Tumor_G1
4804	ETFB	4.812078021	Tumor_G1
4805	UQCR10	7.669236712	Tumor_G1
4806	UQCRQ	7.318075198	Tumor_G1
4807	UQCC3	4.03511629	Tumor_G1
4808	COX15	4.835089295	Tumor_G1
4809	COX5A	6.842294851	Tumor_G1
4810	COX6B1	8.69104545	Tumor_G1
4811	COX6C	6.447920859	Tumor_G1
4812	COX7A2	6.919248782	Tumor_G1
4813	COX7B	6.089130108	Tumor_G1
4814	COX8A	8.866980387	Tumor_G1
4815	SCO1	3.412092092	Tumor_G1
4816	SURF1	6.009047215	Tumor_G1
4817	ATP6V1E1	7.047534002	Tumor_G1
4818	DLAT	4.977412582	Tumor_G1
4819	PDC	0.154139995	Tumor_G1
4820	SUCLG2	6.145659919	Tumor_G1
4821	SLC25A5	8.370023523	Tumor_G1
4822	UCP2	7.474678587	Tumor_G1
4823	SOD2	5.732434958	Tumor_G1
4824	NOX4	3.500252858	Tumor_G1
4825	DUOX1	5.649915708	Tumor_G1
4826	DUOXA1	5.943911869	Tumor_G1
4827	DUOX2	5.30763069	Tumor_G1
4828	DUOXA2	3.814007621	Tumor_G1
4829	NDUFA1	7.670023474	Tumor_G1
4830	NDUFA2	6.366428376	Tumor_G1
4831	NDUFA8	6.743797104	Tumor_G1
4832	NDUFB1	5.256580761	Tumor_G1
4833	NDUFB10	6.988797156	Tumor_G1
4834	NDUFB2	6.225478397	Tumor_G1
4835	NDUFB3	6.07134808	Tumor_G1
4836	NDUFB7	8.014998882	Tumor_G1
4837	CYC1	6.369730023	Tumor_G1
4838	ETFB	4.445877103	Tumor_G1

4839	UQCR10	7.303797428	Tumor_G1
4840	UQCRQ	7.043312534	Tumor_G1
4841	UQCC3	3.620670569	Tumor_G1
4842	COX15	4.016532055	Tumor_G1
4843	COX5A	6.6570157	Tumor_G1
4844	COX6B1	8.437698957	Tumor_G1
4845	COX6C	6.248041788	Tumor_G1
4846	COX7A2	6.612935959	Tumor_G1
4847	COX7B	5.832407644	Tumor_G1
4848	COX8A	8.522666011	Tumor_G1
4849	SCO1	2.596967638	Tumor_G1
4850	SURF1	5.61628741	Tumor_G1
4851	ATP6V1E1	6.836380949	Tumor_G1
4852	DLAT	4.466807558	Tumor_G1
4853	PDC	0.044100836	Tumor_G1
4854	SUCLG2	5.885599784	Tumor_G1
4855	SLC25A5	7.619495309	Tumor_G1
4856	UCP2	7.513526035	Tumor_G1
4857	SOD2	3.990565856	Tumor_G1
4858	NOX4	1.520123642	Tumor_G1
4859	DUOX1	5.379419819	Tumor_G1
4860	DUOXA1	5.583703	Tumor_G1
4861	DUOX2	5.419388011	Tumor_G1
4862	DUOXA2	3.938957861	Tumor_G1
4863	NDUFA1	7.556247439	Tumor_G1
4864	NDUFA2	6.500614724	Tumor_G1
4865	NDUFA8	6.627328521	Tumor_G1
4866	NDUFB1	5.342782167	Tumor_G1
4867	NDUFB10	6.927247388	Tumor_G1
4868	NDUFB2	6.356223432	Tumor_G1
4869	NDUFB3	6.051792706	Tumor_G1
4870	NDUFB7	8.03523945	Tumor_G1
4871	CYC1	6.578859621	Tumor_G1
4872	ETFB	4.512809275	Tumor_G1
4873	UQCR10	7.257695004	Tumor_G1
4874	UQCRQ	7.025169666	Tumor_G1
4875	UQCC3	3.841498382	Tumor_G1
4876	COX15	4.293517542	Tumor_G1
4877	COX5A	6.623062357	Tumor_G1
4878	COX6B1	8.305815737	Tumor_G1

4879	COX6C	5.890830863	Tumor_G1
4880	COX7A2	6.590344874	Tumor_G1
4881	COX7B	5.724723486	Tumor_G1
4882	COX8A	8.768261841	Tumor_G1
4883	SCO1	2.864723356	Tumor_G1
4884	SURF1	5.659882225	Tumor_G1
4885	ATP6V1E1	6.928648657	Tumor_G1
4886	DLAT	4.780170394	Tumor_G1
4887	PDC	0.184557832	Tumor_G1
4888	SUCLG2	5.921190183	Tumor_G1
4889	SLC25A5	7.871383794	Tumor_G1
4890	UCP2	7.691994701	Tumor_G1
4891	SOD2	4.903102537	Tumor_G1
4892	NOX4	1.981707419	Tumor_G1
4893	DUOX1	5.809561451	Tumor_G1
4894	DUOXA1	5.987455865	Tumor_G1
4895	DUOX2	5.56698834	Tumor_G1
4896	DUOXA2	3.67382258	Tumor_G1
4897	NDUFA1	7.784487063	Tumor_G1
4898	NDUFA2	6.513796272	Tumor_G1
4899	NDUFA8	6.466966231	Tumor_G1
4900	NDUFB1	5.417730926	Tumor_G1
4901	NDUFB10	7.473209301	Tumor_G1
4902	NDUFB2	6.091358228	Tumor_G1
4903	NDUFB3	5.864274899	Tumor_G1
4904	NDUFB7	8.15687495	Tumor_G1
4905	CYC1	6.618232081	Tumor_G1
4906	ETFB	4.681146547	Tumor_G1
4907	UQCR10	7.287080933	Tumor_G1
4908	UQCRQ	7.178393622	Tumor_G1
4909	UQCC3	4.142592641	Tumor_G1
4910	COX15	4.501944803	Tumor_G1
4911	COX5A	7.074120247	Tumor_G1
4912	COX6B1	8.442904098	Tumor_G1
4913	COX6C	6.224110712	Tumor_G1
4914	COX7A2	6.854857117	Tumor_G1
4915	COX7B	5.85738769	Tumor_G1
4916	COX8A	9.269121985	Tumor_G1
4917	SCO1	2.932908669	Tumor_G1
4918	SURF1	6.091080382	Tumor_G1

4919	ATP6V1E1	6.911762767	Tumor_G1
4920	DLAT	4.998053867	Tumor_G1
4921	PDC	0.372985054	Tumor_G1
4922	SUCLG2	5.92892261	Tumor_G1
4923	SLC25A5	8.226790178	Tumor_G1
4924	UCP2	8.583314582	Tumor_G1
4925	SOD2	5.560722853	Tumor_G1
4926	NOX4	3.843869542	Tumor_G1
4927	DUOX1	6.83514438	Tumor_G1
4928	DUOXA1	7.197823717	Tumor_G1
4929	DUOX2	5.851189868	Tumor_G1
4930	DUOXA2	3.99434094	Tumor_G1
4931	NDUFA1	7.411059124	Tumor_G1
4932	NDUFA2	6.276278414	Tumor_G1
4933	NDUFA8	6.615745595	Tumor_G1
4934	NDUFB1	5.01783222	Tumor_G1
4935	NDUFB10	7.171713789	Tumor_G1
4936	NDUFB2	6.029177017	Tumor_G1
4937	NDUFB3	5.689546856	Tumor_G1
4938	NDUFB7	8.313790287	Tumor_G1
4939	CYC1	6.556906362	Tumor_G1
4940	ETFB	4.778047113	Tumor_G1
4941	UQCR10	7.152141306	Tumor_G1
4942	UQCRQ	6.782295451	Tumor_G1
4943	UQCC3	4.102129489	Tumor_G1
4944	COX15	4.552753779	Tumor_G1
4945	COX5A	6.67807587	Tumor_G1
4946	COX6B1	8.271127237	Tumor_G1
4947	COX6C	5.803476872	Tumor_G1
4948	COX7A2	6.16957337	Tumor_G1
4949	COX7B	5.424301739	Tumor_G1
4950	COX8A	8.986234452	Tumor_G1
4951	SCO1	3.07069341	Tumor_G1
4952	SURF1	6.184821115	Tumor_G1
4953	ATP6V1E1	6.538214039	Tumor_G1
4954	DLAT	4.642964602	Tumor_G1
4955	PDC	0.091876531	Tumor_G1
4956	SUCLG2	5.496248167	Tumor_G1
4957	SLC25A5	7.752403194	Tumor_G1
4958	UCP2	7.841801033	Tumor_G1

4959	SOD2	6.800450371	Tumor_G1
4960	NOX4	3.32998159	Tumor_G1
4961	DUOX1	6.122074715	Tumor_G1
4962	DUOXA1	6.450377457	Tumor_G1
4963	DUOX2	6.265359834	Tumor_G1
4964	DUOXA2	4.150102523	Tumor_G1
4965	NDUFA1	7.785043304	Tumor_G1
4966	NDUFA2	6.681414941	Tumor_G1
4967	NDUFA8	6.617429359	Tumor_G1
4968	NDUFB1	5.447699089	Tumor_G1
4969	NDUFB10	7.219519058	Tumor_G1
4970	NDUFB2	6.222823615	Tumor_G1
4971	NDUFB3	5.971356396	Tumor_G1
4972	NDUFB7	8.246879715	Tumor_G1
4973	CYC1	6.438025885	Tumor_G1
4974	ETFB	4.354476793	Tumor_G1
4975	UQCR10	7.269093598	Tumor_G1
4976	UQCRQ	7.056174688	Tumor_G1
4977	UQCC3	4.076658962	Tumor_G1
4978	COX15	4.010240054	Tumor_G1
4979	COX5A	6.666735824	Tumor_G1
4980	COX6B1	8.47926784	Tumor_G1
4981	COX6C	6.029663594	Tumor_G1
4982	COX7A2	6.548138597	Tumor_G1
4983	COX7B	5.752518088	Tumor_G1
4984	COX8A	9.143708413	Tumor_G1
4985	SCO1	2.595878694	Tumor_G1
4986	SURF1	5.937092734	Tumor_G1
4987	ATP6V1E1	6.661829873	Tumor_G1
4988	DLAT	4.299015166	Tumor_G1
4989	PDC	0.127264605	Tumor_G1
4990	SUCLG2	5.286666232	Tumor_G1
4991	SLC25A5	7.409983114	Tumor_G1
4992	UCP2	7.852590807	Tumor_G1
4993	SOD2	5.000558851	Tumor_G1
4994	NOX4	1.243251574	Tumor_G1
4995	DUOX1	5.560959167	Tumor_G1
4996	DUOXA1	5.92404136	Tumor_G1
4997	DUOX2	5.711963399	Tumor_G1
4998	DUOXA2	4.611009966	Tumor_G1

4999	NDUFA1	7.551120466	Tumor_G1
5000	NDUFA2	6.212403469	Tumor_G1
5001	NDUFA8	6.489365509	Tumor_G1
5002	NDUFB1	5.143447806	Tumor_G1
5003	NDUFB10	6.993018542	Tumor_G1
5004	NDUFB2	6.458736868	Tumor_G1
5005	NDUFB3	5.890981377	Tumor_G1
5006	NDUFB7	8.106926398	Tumor_G1
5007	CYC1	6.523215476	Tumor_G1
5008	ETFB	4.594683359	Tumor_G1
5009	UQCR10	7.009397046	Tumor_G1
5010	UQCRQ	6.530341437	Tumor_G1
5011	UQCC3	3.970283816	Tumor_G1
5012	COX15	4.801656705	Tumor_G1
5013	COX5A	6.755294311	Tumor_G1
5014	COX6B1	8.069543534	Tumor_G1
5015	COX6C	5.9399496	Tumor_G1
5016	COX7A2	6.354561949	Tumor_G1
5017	COX7B	5.412081213	Tumor_G1
5018	COX8A	8.986586127	Tumor_G1
5019	SCO1	3.174069069	Tumor_G1
5020	SURF1	5.960393028	Tumor_G1
5021	ATP6V1E1	6.991160522	Tumor_G1
5022	DLAT	5.108111658	Tumor_G1
5023	PDC	0.184392074	Tumor_G1
5024	SUCLG2	6.273803683	Tumor_G1
5025	SLC25A5	8.201330423	Tumor_G1
5026	UCP2	7.375923956	Tumor_G1
5027	SOD2	5.312512592	Tumor_G1
5028	NOX4	0.964077567	Tumor_G1
5029	DUOX1	6.205391813	Tumor_G1
5030	DUOXA1	6.290186364	Tumor_G1
5031	DUOX2	6.131230351	Tumor_G1
5032	DUOXA2	3.717142434	Tumor_G1
5033	NDUFA1	7.92859567	Tumor_G1
5034	NDUFA2	6.964519455	Tumor_G1
5035	NDUFA8	6.924290538	Tumor_G1
5036	NDUFB1	5.923080387	Tumor_G1
5037	NDUFB10	7.290303281	Tumor_G1
5038	NDUFB2	6.561155946	Tumor_G1

5039	NDUFB3	6.509623019	Tumor_G1
5040	NDUFB7	8.360016299	Tumor_G1
5041	CYC1	6.749083493	Tumor_G1
5042	ETFB	4.726560416	Tumor_G1
5043	UQCR10	7.819328449	Tumor_G1
5044	UQCRQ	7.593251313	Tumor_G1
5045	UQCC3	3.963910283	Tumor_G1
5046	COX15	4.626193812	Tumor_G1
5047	COX5A	7.444188247	Tumor_G1
5048	COX6B1	8.670232716	Tumor_G1
5049	COX6C	6.509291099	Tumor_G1
5050	COX7A2	7.152737716	Tumor_G1
5051	COX7B	6.355845507	Tumor_G1
5052	COX8A	9.326227195	Tumor_G1
5053	SCO1	3.062094594	Tumor_G1
5054	SURF1	5.862523036	Tumor_G1
5055	ATP6V1E1	7.478532196	Tumor_G1
5056	DLAT	5.465161635	Tumor_G1
5057	PDC	0.044549983	Tumor_G1
5058	SUCLG2	6.167076647	Tumor_G1
5059	SLC25A5	8.401985755	Tumor_G1
5060	UCP2	7.923392962	Tumor_G1
5061	SOD2	5.606494419	Tumor_G1
5062	NOX4	2.16087411	Tumor_G1
5063	DUOX1	5.687773538	Tumor_G1
5064	DUOXA1	5.725852463	Tumor_G1
5065	DUOX2	5.296899507	Tumor_G1
5066	DUOXA2	3.474026752	Tumor_G1
5067	NDUFA1	7.643345256	Tumor_G1
5068	NDUFA2	6.090831245	Tumor_G1
5069	NDUFA8	6.752680933	Tumor_G1
5070	NDUFB1	5.072699297	Tumor_G1
5071	NDUFB10	6.986483096	Tumor_G1
5072	NDUFB2	5.898626462	Tumor_G1
5073	NDUFB3	6.152575286	Tumor_G1
5074	NDUFB7	8.151015545	Tumor_G1
5075	CYC1	6.1364175	Tumor_G1
5076	ETFB	4.727466302	Tumor_G1
5077	UQCR10	7.205024469	Tumor_G1
5078	UQCRQ	6.820184593	Tumor_G1

5079	UQCC3	3.679702401	Tumor_G1
5080	COX15	4.897428708	Tumor_G1
5081	COX5A	6.89728505	Tumor_G1
5082	COX6B1	8.30687896	Tumor_G1
5083	COX6C	5.942665578	Tumor_G1
5084	COX7A2	6.52828758	Tumor_G1
5085	COX7B	5.93545912	Tumor_G1
5086	COX8A	8.433148658	Tumor_G1
5087	SCO1	3.59115044	Tumor_G1
5088	SURF1	5.851689038	Tumor_G1
5089	ATP6V1E1	6.851529417	Tumor_G1
5090	DLAT	5.43699985	Tumor_G1
5091	PDC	0.213360196	Tumor_G1
5092	SUCLG2	5.99741664	Tumor_G1
5093	SLC25A5	8.055050459	Tumor_G1
5094	UCP2	8.678086912	Tumor_G1
5095	SOD2	5.484492795	Tumor_G1
5096	NOX4	3.560762109	Tumor_G1
5097	DUOX1	6.954962436	Tumor_G1
5098	DUOXA1	7.163876097	Tumor_G1
5099	DUOX2	7.699150441	Tumor_G1
5100	DUOXA2	5.028046108	Tumor_G1
5101	NDUFA1	7.812675554	Tumor_G1
5102	NDUFA2	6.962723363	Tumor_G1
5103	NDUFA8	6.85255881	Tumor_G1
5104	NDUFB1	5.411675028	Tumor_G1
5105	NDUFB10	7.433127006	Tumor_G1
5106	NDUFB2	6.635690608	Tumor_G1
5107	NDUFB3	6.176902083	Tumor_G1
5108	NDUFB7	8.811165381	Tumor_G1
5109	CYC1	6.706937624	Tumor_G1
5110	ETFB	4.884520012	Tumor_G1
5111	UQCR10	7.675906034	Tumor_G1
5112	UQCRQ	7.35859139	Tumor_G1
5113	UQCC3	4.238364632	Tumor_G1
5114	COX15	4.141579047	Tumor_G1
5115	COX5A	7.020042896	Tumor_G1
5116	COX6B1	8.939196702	Tumor_G1
5117	COX6C	6.412491645	Tumor_G1
5118	COX7A2	7.078836085	Tumor_G1

5119	COX7B	6.035656218	Tumor_G1
5120	COX8A	9.163717993	Tumor_G1
5121	SCO1	2.601563116	Tumor_G1
5122	SURF1	6.491948987	Tumor_G1
5123	ATP6V1E1	7.194092559	Tumor_G1
5124	DLAT	4.532893436	Tumor_G1
5125	PDC	0.157503827	Tumor_G1
5126	SUCLG2	5.729605324	Tumor_G1
5127	SLC25A5	7.8825637	Tumor_G1
5128	UCP2	8.461179577	Tumor_G1
5129	SOD2	4.909657288	Tumor_G1
5130	NOX4	2.889579943	Tumor_G1
5131	DUOX1	4.89611899	Tumor_G1
5132	DUOXA1	5.54641263	Tumor_G1
5133	DUOX2	4.694403389	Tumor_G1
5134	DUOXA2	3.958378572	Tumor_G1
5135	NDUFA1	7.703070906	Tumor_G1
5136	NDUFA2	6.541493993	Tumor_G1
5137	NDUFA8	6.792087537	Tumor_G1
5138	NDUFB1	5.423812987	Tumor_G1
5139	NDUFB10	7.091396791	Tumor_G1
5140	NDUFB2	6.069056888	Tumor_G1
5141	NDUFB3	5.930392504	Tumor_G1
5142	NDUFB7	8.285640155	Tumor_G1
5143	CYC1	6.53008475	Tumor_G1
5144	ETFB	4.467619744	Tumor_G1
5145	UQCR10	7.219807901	Tumor_G1
5146	UQCRQ	6.926128635	Tumor_G1
5147	UQCC3	3.96000207	Tumor_G1
5148	COX15	4.413708834	Tumor_G1
5149	COX5A	6.957654066	Tumor_G1
5150	COX6B1	8.528896309	Tumor_G1
5151	COX6C	6.229689632	Tumor_G1
5152	COX7A2	6.643604623	Tumor_G1
5153	COX7B	5.798465589	Tumor_G1
5154	COX8A	8.80348037	Tumor_G1
5155	SCO1	2.876598786	Tumor_G1
5156	SURF1	6.102612415	Tumor_G1
5157	ATP6V1E1	6.892242374	Tumor_G1
5158	DLAT	4.839057317	Tumor_G1

5159	PDC	0.039481636	Tumor_G1
5160	SUCLG2	5.844138628	Tumor_G1
5161	SLC25A5	7.981438987	Tumor_G1
5162	UCP2	7.845664096	Tumor_G1
5163	SOD2	6.423924311	Tumor_G1
5164	NOX4	1.924939697	Tumor_G1
5165	DUOX1	5.07669612	Tumor_G1
5166	DUOXA1	5.236515502	Tumor_G1
5167	DUOX2	4.312490617	Tumor_G1
5168	DUOXA2	2.623807123	Tumor_G1
5169	NDUFA1	7.742419673	Tumor_G1
5170	NDUFA2	6.79195542	Tumor_G1
5171	NDUFA8	6.877090019	Tumor_G1
5172	NDUFB1	5.393396487	Tumor_G1
5173	NDUFB10	7.317211345	Tumor_G1
5174	NDUFB2	6.086894033	Tumor_G1
5175	NDUFB3	5.893376735	Tumor_G1
5176	NDUFB7	8.406662348	Tumor_G1
5177	CYC1	6.381950954	Tumor_G1
5178	ETFB	4.358368221	Tumor_G1
5179	UQCR10	7.329215862	Tumor_G1
5180	UQCRQ	7.164451583	Tumor_G1
5181	UQCC3	4.087127948	Tumor_G1
5182	COX15	4.101015667	Tumor_G1
5183	COX5A	6.932101936	Tumor_G1
5184	COX6B1	8.532929993	Tumor_G1
5185	COX6C	6.260041615	Tumor_G1
5186	COX7A2	6.642980325	Tumor_G1
5187	COX7B	5.712157526	Tumor_G1
5188	COX8A	8.89953092	Tumor_G1
5189	SCO1	2.422668561	Tumor_G1
5190	SURF1	5.992491482	Tumor_G1
5191	ATP6V1E1	6.692304044	Tumor_G1
5192	DLAT	4.546532039	Tumor_G1
5193	PDC	0.046739363	Tumor_G1
5194	SUCLG2	5.753199785	Tumor_G1
5195	SLC25A5	7.469110494	Tumor_G1
5196	UCP2	7.531505759	Tumor_G1
5197	SOD2	5.299289839	Tumor_G1
5198	NOX4	3.18824907	Tumor_G1

5199	DUOX1	4.472609955	Tumor_G1
5200	DUOXA1	5.04509794	Tumor_G1
5201	DUOX2	4.308996738	Tumor_G1
5202	DUOXA2	3.010897709	Tumor_G1
5203	NDUFA1	7.854807599	Tumor_G1
5204	NDUFA2	6.621182237	Tumor_G1
5205	NDUFA8	6.975209474	Tumor_G1
5206	NDUFB1	5.537874805	Tumor_G1
5207	NDUFB10	7.534525898	Tumor_G1
5208	NDUFB2	6.770885846	Tumor_G1
5209	NDUFB3	6.346966236	Tumor_G1
5210	NDUFB7	8.063349724	Tumor_G1
5211	CYC1	6.977454004	Tumor_G1
5212	ETFB	4.370114	Tumor_G1
5213	UQCR10	7.30233331	Tumor_G1
5214	UQCRQ	7.053629159	Tumor_G1
5215	UQCC3	4.397138178	Tumor_G1
5216	COX15	5.437662009	Tumor_G1
5217	COX5A	7.815289068	Tumor_G1
5218	COX6B1	8.743655144	Tumor_G1
5219	COX6C	6.449733096	Tumor_G1
5220	COX7A2	7.006400989	Tumor_G1
5221	COX7B	6.134565064	Tumor_G1
5222	COX8A	9.021999584	Tumor_G1
5223	SCO1	3.954103731	Tumor_G1
5224	SURF1	6.470824198	Tumor_G1
5225	ATP6V1E1	6.879304719	Tumor_G1
5226	DLAT	6.182478159	Tumor_G1
5227	PDC	0.202718966	Tumor_G1
5228	SUCLG2	6.951185385	Tumor_G1
5229	SLC25A5	9.143502453	Tumor_G1
5230	UCP2	5.731734854	Tumor_G1
5231	SOD2	5.823244983	Tumor_G1
5232	NOX4	0.621515827	Tumor_G1
5233	DUOX1	5.358294869	Tumor_G1
5234	DUOXA1	5.4875297	Tumor_G1
5235	DUOX2	4.747781185	Tumor_G1
5236	DUOXA2	2.21361782	Tumor_G1
5237	NDUFA1	7.975382344	Tumor_G1
5238	NDUFA2	6.72151817	Tumor_G1

5239	NDUFA8	6.795728542	Tumor_G1
5240	NDUFB1	5.455630006	Tumor_G1
5241	NDUFB10	7.51123957	Tumor_G1
5242	NDUFB2	6.697082098	Tumor_G1
5243	NDUFB3	6.310330596	Tumor_G1
5244	NDUFB7	8.517460305	Tumor_G1
5245	CYC1	6.720152527	Tumor_G1
5246	ETFB	4.408443703	Tumor_G1
5247	UQCR10	7.46072866	Tumor_G1
5248	UQCRQ	7.340005779	Tumor_G1
5249	UQCC3	4.164109631	Tumor_G1
5250	COX15	4.270518859	Tumor_G1
5251	COX5A	7.181863833	Tumor_G1
5252	COX6B1	8.745234215	Tumor_G1
5253	COX6C	6.241148403	Tumor_G1
5254	COX7A2	6.888374469	Tumor_G1
5255	COX7B	5.864759919	Tumor_G1
5256	COX8A	9.356206202	Tumor_G1
5257	SCO1	2.871094218	Tumor_G1
5258	SURF1	5.738503148	Tumor_G1
5259	ATP6V1E1	6.860678934	Tumor_G1
5260	DLAT	4.780461281	Tumor_G1
5261	PDC	0.128660621	Tumor_G1
5262	SUCLG2	5.83653455	Tumor_G1
5263	SLC25A5	7.882676542	Tumor_G1
5264	UCP2	8.006987057	Tumor_G1
5265	SOD2	5.195773467	Tumor_G1
5266	NOX4	2.284626176	Tumor_G1
5267	DUOX1	5.436028641	Tumor_G1
5268	DUOXA1	5.480857925	Tumor_G1
5269	DUOX2	4.855079663	Tumor_G1
5270	DUOXA2	3.748224329	Tumor_G1
5271	NDUFA1	7.616367681	Tumor_G1
5272	NDUFA2	6.387172941	Tumor_G1
5273	NDUFA8	6.500708627	Tumor_G1
5274	NDUFB1	5.451608435	Tumor_G1
5275	NDUFB10	7.034211963	Tumor_G1
5276	NDUFB2	6.200573501	Tumor_G1
5277	NDUFB3	6.122524912	Tumor_G1
5278	NDUFB7	8.264302551	Tumor_G1

5279	CYC1	6.452749808	Tumor_G1
5280	ETFB	4.649328057	Tumor_G1
5281	UQCR10	7.143423985	Tumor_G1
5282	UQCRQ	6.93927729	Tumor_G1
5283	UQCC3	3.991501576	Tumor_G1
5284	COX15	4.680200709	Tumor_G1
5285	COX5A	6.8906847	Tumor_G1
5286	COX6B1	8.232405654	Tumor_G1
5287	COX6C	6.05342021	Tumor_G1
5288	COX7A2	6.505579493	Tumor_G1
5289	COX7B	5.707558236	Tumor_G1
5290	COX8A	9.052700889	Tumor_G1
5291	SCO1	3.194896644	Tumor_G1
5292	SURF1	5.901391359	Tumor_G1
5293	ATP6V1E1	6.942495761	Tumor_G1
5294	DLAT	4.97253218	Tumor_G1
5295	PDC	0.226357463	Tumor_G1
5296	SUCLG2	5.992455519	Tumor_G1
5297	SLC25A5	8.141304559	Tumor_G1
5298	UCP2	8.118298682	Tumor_G1
5299	SOD2	5.214404574	Tumor_G1
5300	NOX4	2.453564058	Tumor_G1
5301	DUOX1	6.01872489	Tumor_G1
5302	DUOXA1	6.011354338	Tumor_G1
5303	DUOX2	5.266585822	Tumor_G1
5304	DUOXA2	3.446080911	Tumor_G1
5305	NDUFA1	8.099626962	Tumor_G1
5306	NDUFA2	6.728776613	Tumor_G1
5307	NDUFA8	6.834312925	Tumor_G1
5308	NDUFB1	5.895490397	Tumor_G1
5309	NDUFB10	7.106666972	Tumor_G1
5310	NDUFB2	5.360160209	Tumor_G1
5311	NDUFB3	4.581148646	Tumor_G1
5312	NDUFB7	8.499748151	Tumor_G1
5313	CYC1	5.745041037	Tumor_G1
5314	ETFB	4.032465287	Tumor_G1
5315	UQCR10	5.995687044	Tumor_G1
5316	UQCRQ	7.055122506	Tumor_G1
5317	UQCC3	4.1375752	Tumor_G1
5318	COX15	3.390387671	Tumor_G1

5319	COX5A	6.553347523	Tumor_G1
5320	COX6B1	8.657110624	Tumor_G1
5321	COX6C	6.275603621	Tumor_G1
5322	COX7A2	6.22177781	Tumor_G1
5323	COX7B	5.469459956	Tumor_G1
5324	COX8A	9.110886701	Tumor_G1
5325	SCO1	1.476141809	Tumor_G1
5326	SURF1	5.383839253	Tumor_G1
5327	ATP6V1E1	5.019534934	Tumor_G1
5328	DLAT	2.826123815	Tumor_G1
5329	PDC	0	Tumor_G1
5330	SUCLG2	4.615577391	Tumor_G1
5331	SLC25A5	5.680903441	Tumor_G1
5332	UCP2	7.26852656	Tumor_G1
5333	SOD2	2.673847819	Tumor_G1
5334	NOX4	1.466653033	Tumor_G1
5335	DUOX1	5.267619636	Tumor_G1
5336	DUOXA1	5.060708057	Tumor_G1
5337	DUOX2	5.658324437	Tumor_G1
5338	DUOXA2	4.774344035	Tumor_G1
5339	NDUFA1	7.598393714	Tumor_G1
5340	NDUFA2	6.469394577	Tumor_G1
5341	NDUFA8	6.777709173	Tumor_G1
5342	NDUFB1	5.403435487	Tumor_G1
5343	NDUFB10	7.311228677	Tumor_G1
5344	NDUFB2	6.319567078	Tumor_G1
5345	NDUFB3	5.857451134	Tumor_G1
5346	NDUFB7	8.347092639	Tumor_G1
5347	CYC1	6.414157303	Tumor_G1
5348	ETFB	4.597788379	Tumor_G1
5349	UQCR10	7.175835314	Tumor_G1
5350	UQCRQ	7.04422578	Tumor_G1
5351	UQCC3	3.763498935	Tumor_G1
5352	COX15	4.415884913	Tumor_G1
5353	COX5A	6.835902672	Tumor_G1
5354	COX6B1	8.300207188	Tumor_G1
5355	COX6C	6.229823139	Tumor_G1
5356	COX7A2	6.540959617	Tumor_G1
5357	COX7B	5.694286345	Tumor_G1
5358	COX8A	8.941314226	Tumor_G1

5359	SCO1	2.830663003	Tumor_G1
5360	SURF1	6.023504361	Tumor_G1
5361	ATP6V1E1	7.05540364	Tumor_G1
5362	DLAT	4.472975809	Tumor_G1
5363	PDC	0.053095957	Tumor_G1
5364	SUCLG2	6.059168634	Tumor_G1
5365	SLC25A5	7.554099168	Tumor_G1
5366	UCP2	8.639288863	Tumor_G1
5367	SOD2	5.108208662	Tumor_G1
5368	NOX4	2.022632318	Tumor_G1
5369	DUOX1	6.169792482	Tumor_G1
5370	DUOXA1	6.343892221	Tumor_G1
5371	DUOX2	5.758566613	Tumor_G1
5372	DUOXA2	3.820611093	Tumor_G1
5373	NDUFA1	7.616305945	Tumor_G1
5374	NDUFA2	6.586621549	Tumor_G1
5375	NDUFA8	7.061550146	Tumor_G1
5376	NDUFB1	5.352152411	Tumor_G1
5377	NDUFB10	7.196560806	Tumor_G1
5378	NDUFB2	6.153619666	Tumor_G1
5379	NDUFB3	5.783015293	Tumor_G1
5380	NDUFB7	8.167790951	Tumor_G1
5381	CYC1	6.796075273	Tumor_G1
5382	ETFB	4.929436678	Tumor_G1
5383	UQCR10	6.641368382	Tumor_G1
5384	UQCRQ	7.125816383	Tumor_G1
5385	UQCC3	4.099686586	Tumor_G1
5386	COX15	4.717450575	Tumor_G1
5387	COX5A	6.906575181	Tumor_G1
5388	COX6B1	8.462266089	Tumor_G1
5389	COX6C	6.26233841	Tumor_G1
5390	COX7A2	6.80784898	Tumor_G1
5391	COX7B	5.798100791	Tumor_G1
5392	COX8A	9.278710719	Tumor_G1
5393	SCO1	2.769472191	Tumor_G1
5394	SURF1	5.682315673	Tumor_G1
5395	ATP6V1E1	6.084171164	Tumor_G1
5396	DLAT	5.291527075	Tumor_G1
5397	PDC	0.357384513	Tumor_G1
5398	SUCLG2	6.539186194	Tumor_G1

5399	SLC25A5	7.421065747	Tumor_G1
5400	UCP2	7.920127345	Tumor_G1
5401	SOD2	3.639486717	Tumor_G1
5402	NOX4	0.932459212	Tumor_G1
5403	DUOX1	6.68265687	Tumor_G1
5404	DUOXA1	6.42478242	Tumor_G1
5405	DUOX2	7.777825645	Tumor_G1
5406	DUOXA2	5.026858167	Tumor_G1
5407	NDUFA1	7.767690778	Tumor_G1
5408	NDUFA2	6.586101182	Tumor_G1
5409	NDUFA8	6.910691714	Tumor_G1
5410	NDUFB1	5.686643646	Tumor_G1
5411	NDUFB10	7.45741785	Tumor_G1
5412	NDUFB2	6.773587177	Tumor_G1
5413	NDUFB3	5.762688094	Tumor_G1
5414	NDUFB7	8.246872568	Tumor_G1
5415	CYC1	6.473940792	Tumor_G1
5416	ETFB	4.518874876	Tumor_G1
5417	UQCR10	7.496210592	Tumor_G1
5418	UQCRQ	7.220962083	Tumor_G1
5419	UQCC3	3.852749239	Tumor_G1
5420	COX15	4.190621703	Tumor_G1
5421	COX5A	7.050657545	Tumor_G1
5422	COX6B1	8.395155264	Tumor_G1
5423	COX6C	6.286314681	Tumor_G1
5424	COX7A2	6.525192383	Tumor_G1
5425	COX7B	5.764185419	Tumor_G1
5426	COX8A	8.874663848	Tumor_G1
5427	SCO1	2.65849255	Tumor_G1
5428	SURF1	6.286634487	Tumor_G1
5429	ATP6V1E1	6.610008566	Tumor_G1
5430	DLAT	4.430865316	Tumor_G1
5431	PDC	0	Tumor_G1
5432	SUCLG2	5.830533254	Tumor_G1
5433	SLC25A5	7.47590702	Tumor_G1
5434	UCP2	7.802077115	Tumor_G1
5435	SOD2	4.988628534	Tumor_G1
5436	NOX4	0.854870675	Tumor_G1
5437	DUOX1	6.140079346	Tumor_G1
5438	DUOXA1	5.829975503	Tumor_G1

5439	DUOX2	7.121521651	Tumor_G1
5440	DUOXA2	6.017476407	Tumor_G1
5441	NDUFA1	7.888825622	Tumor_G1
5442	NDUFA2	7.140251938	Tumor_G1
5443	NDUFA8	7.042514407	Tumor_G1
5444	NDUFB1	5.903704741	Tumor_G1
5445	NDUFB10	7.668296882	Tumor_G1
5446	NDUFB2	6.450005531	Tumor_G1
5447	NDUFB3	6.258964478	Tumor_G1
5448	NDUFB7	8.754031572	Tumor_G1
5449	CYC1	6.925618789	Tumor_G1
5450	ETFB	5.096223618	Tumor_G1
5451	UQCR10	7.638214611	Tumor_G1
5452	UQCRQ	7.648090087	Tumor_G1
5453	UQCC3	4.376613785	Tumor_G1
5454	COX15	4.141371953	Tumor_G1
5455	COX5A	7.344012664	Tumor_G1
5456	COX6B1	8.972978948	Tumor_G1
5457	COX6C	6.494749772	Tumor_G1
5458	COX7A2	6.955442847	Tumor_G1
5459	COX7B	6.028273447	Tumor_G1
5460	COX8A	8.993964818	Tumor_G1
5461	SCO1	2.503608262	Tumor_G1
5462	SURF1	6.402441588	Tumor_G1
5463	ATP6V1E1	7.122392284	Tumor_G1
5464	DLAT	4.497843635	Tumor_G1
5465	PDC	0.127019646	Tumor_G1
5466	SUCLG2	6.241252151	Tumor_G1
5467	SLC25A5	7.864255636	Tumor_G1
5468	UCP2	7.690509346	Tumor_G1
5469	SOD2	5.447387393	Tumor_G1
5470	NOX4	3.203618163	Tumor_G1
5471	DUOX1	5.542459466	Tumor_G1
5472	DUOXA1	5.760808304	Tumor_G1
5473	DUOX2	3.90546913	Tumor_G1
5474	DUOXA2	2.629259206	Tumor_G1
5475	NDUFA1	7.278917966	Tumor_G1
5476	NDUFA2	6.058548793	Tumor_G1
5477	NDUFA8	6.448854592	Tumor_G1
5478	NDUFB1	4.884111243	Tumor_G1

5479	NDUFB10	6.789176597	Tumor_G1
5480	NDUFB2	5.857611732	Tumor_G1
5481	NDUFB3	5.900424362	Tumor_G1
5482	NDUFB7	7.991650507	Tumor_G1
5483	CYC1	6.433001963	Tumor_G1
5484	ETFB	4.259493956	Tumor_G1
5485	UQCR10	6.988939236	Tumor_G1
5486	UQCRQ	6.715501962	Tumor_G1
5487	UQCC3	3.450860868	Tumor_G1
5488	COX15	4.565841496	Tumor_G1
5489	COX5A	6.817016004	Tumor_G1
5490	COX6B1	8.100513094	Tumor_G1
5491	COX6C	5.830358737	Tumor_G1
5492	COX7A2	6.420290176	Tumor_G1
5493	COX7B	5.500341992	Tumor_G1
5494	COX8A	8.746105647	Tumor_G1
5495	SCO1	2.809028551	Tumor_G1
5496	SURF1	5.45113574	Tumor_G1
5497	ATP6V1E1	7.314830212	Tumor_G1
5498	DLAT	5.029961519	Tumor_G1
5499	PDC	0.217213171	Tumor_G1
5500	SUCLG2	6.260660426	Tumor_G1
5501	SLC25A5	8.140426499	Tumor_G1
5502	UCP2	8.81183997	Tumor_G1
5503	SOD2	5.90459313	Tumor_G1
5504	NOX4	2.500188871	Tumor_G1
5505	DUOX1	5.680339333	Tumor_G1
5506	DUOXA1	5.945993448	Tumor_G1
5507	DUOX2	6.132804101	Tumor_G1
5508	DUOXA2	3.097674842	Tumor_G1
5509	NDUFA1	8.132588998	Tumor_G2
5510	NDUFA2	7.113687329	Tumor_G2
5511	NDUFA8	7.373156954	Tumor_G2
5512	NDUFB1	5.98396003	Tumor_G2
5513	NDUFB10	8.168727046	Tumor_G2
5514	NDUFB2	7.251992448	Tumor_G2
5515	NDUFB3	6.229551563	Tumor_G2
5516	NDUFB7	9.381385843	Tumor_G2
5517	CYC1	7.116602709	Tumor_G2
5518	ETFB	6.47896645	Tumor_G2

5519	UQCR10	8.090370099	Tumor_G2
5520	UQCRQ	7.64530386	Tumor_G2
5521	UQCC3	5.097788786	Tumor_G2
5522	COX15	4.845430038	Tumor_G2
5523	COX5A	7.233394841	Tumor_G2
5524	COX6B1	9.270308446	Tumor_G2
5525	COX6C	6.653507586	Tumor_G2
5526	COX7A2	6.945660832	Tumor_G2
5527	COX7B	6.3131626	Tumor_G2
5528	COX8A	10.32600094	Tumor_G2
5529	SCO1	3.249560822	Tumor_G2
5530	SURF1	7.157434525	Tumor_G2
5531	ATP6V1E1	7.141138126	Tumor_G2
5532	DLAT	4.468673756	Tumor_G2
5533	PDC	0.057050275	Tumor_G2
5534	SUCLG2	5.640150257	Tumor_G2
5535	SLC25A5	7.336207836	Tumor_G2
5536	UCP2	9.37567875	Tumor_G2
5537	SOD2	4.129791131	Tumor_G2
5538	NOX4	2.297521126	Tumor_G2
5539	DUOX1	7.369753747	Tumor_G2
5540	DUOXA1	7.833724558	Tumor_G2
5541	DUOX2	7.499120588	Tumor_G2
5542	DUOXA2	6.486574208	Tumor_G2
5543	NDUFA1	7.861650538	Tumor_G2
5544	NDUFA2	6.578312336	Tumor_G2
5545	NDUFA8	7.15017338	Tumor_G2
5546	NDUFB1	5.837134066	Tumor_G2
5547	NDUFB10	7.828616494	Tumor_G2
5548	NDUFB2	6.617200417	Tumor_G2
5549	NDUFB3	6.22211683	Tumor_G2
5550	NDUFB7	8.734161756	Tumor_G2
5551	CYC1	6.774602896	Tumor_G2
5552	ETFB	5.193138799	Tumor_G2
5553	UQCR10	7.580819917	Tumor_G2
5554	UQCRQ	7.159016241	Tumor_G2
5555	UQCC3	4.752203607	Tumor_G2
5556	COX15	4.888395445	Tumor_G2
5557	COX5A	7.228350602	Tumor_G2
5558	COX6B1	8.88962446	Tumor_G2

5559	COX6C	6.445280124	Tumor_G2
5560	COX7A2	6.690768941	Tumor_G2
5561	COX7B	5.884120946	Tumor_G2
5562	COX8A	9.440231863	Tumor_G2
5563	SCO1	3.280826688	Tumor_G2
5564	SURF1	6.906536772	Tumor_G2
5565	ATP6V1E1	6.927356158	Tumor_G2
5566	DLAT	4.727227062	Tumor_G2
5567	PDC	0.058348169	Tumor_G2
5568	SUCLG2	5.864175752	Tumor_G2
5569	SLC25A5	7.435405245	Tumor_G2
5570	UCP2	9.613934549	Tumor_G2
5571	SOD2	4.807755733	Tumor_G2
5572	NOX4	2.116420829	Tumor_G2
5573	DUOX1	6.29389832	Tumor_G2
5574	DUOXA1	6.967148934	Tumor_G2
5575	DUOX2	6.84219408	Tumor_G2
5576	DUOXA2	5.228968963	Tumor_G2
5577	NDUFA1	8.168082771	Tumor_G2
5578	NDUFA2	7.035434935	Tumor_G2
5579	NDUFA8	7.227155545	Tumor_G2
5580	NDUFB1	5.877247806	Tumor_G2
5581	NDUFB10	7.760062455	Tumor_G2
5582	NDUFB2	6.736883728	Tumor_G2
5583	NDUFB3	6.514844656	Tumor_G2
5584	NDUFB7	9.072242372	Tumor_G2
5585	CYC1	7.106367249	Tumor_G2
5586	ETFB	5.589179309	Tumor_G2
5587	UQCR10	7.922693367	Tumor_G2
5588	UQCRQ	7.535468563	Tumor_G2
5589	UQCC3	5.250132709	Tumor_G2
5590	COX15	4.758726139	Tumor_G2
5591	COX5A	7.296520955	Tumor_G2
5592	COX6B1	8.926897685	Tumor_G2
5593	COX6C	6.706239155	Tumor_G2
5594	COX7A2	6.982399602	Tumor_G2
5595	COX7B	6.092568923	Tumor_G2
5596	COX8A	9.832905945	Tumor_G2
5597	SCO1	3.333036164	Tumor_G2
5598	SURF1	6.792642469	Tumor_G2

5599	ATP6V1E1	6.968074013	Tumor_G2
5600	DLAT	4.663733227	Tumor_G2
5601	PDC	0.124881583	Tumor_G2
5602	SUCLG2	5.883313101	Tumor_G2
5603	SLC25A5	7.738760767	Tumor_G2
5604	UCP2	8.742282911	Tumor_G2
5605	SOD2	4.562698701	Tumor_G2
5606	NOX4	2.438020212	Tumor_G2
5607	DUOX1	6.420586454	Tumor_G2
5608	DUOXA1	6.900076122	Tumor_G2
5609	DUOX2	7.051029998	Tumor_G2
5610	DUOXA2	5.190984641	Tumor_G2
5611	NDUFA1	7.603315402	Tumor_G2
5612	NDUFA2	6.636753158	Tumor_G2
5613	NDUFA8	6.92190321	Tumor_G2
5614	NDUFB1	5.491374095	Tumor_G2
5615	NDUFB10	7.465944233	Tumor_G2
5616	NDUFB2	6.466408723	Tumor_G2
5617	NDUFB3	6.177080509	Tumor_G2
5618	NDUFB7	8.481183284	Tumor_G2
5619	CYC1	6.82761484	Tumor_G2
5620	ETFB	5.079749739	Tumor_G2
5621	UQCR10	7.402510399	Tumor_G2
5622	UQCRQ	7.12136546	Tumor_G2
5623	UQCC3	4.496373264	Tumor_G2
5624	COX15	5.006436775	Tumor_G2
5625	COX5A	7.090564904	Tumor_G2
5626	COX6B1	8.549585987	Tumor_G2
5627	COX6C	6.112977919	Tumor_G2
5628	COX7A2	6.710938195	Tumor_G2
5629	COX7B	5.865488999	Tumor_G2
5630	COX8A	9.40061668	Tumor_G2
5631	SCO1	3.623975122	Tumor_G2
5632	SURF1	6.440574311	Tumor_G2
5633	ATP6V1E1	6.870813957	Tumor_G2
5634	DLAT	5.101688001	Tumor_G2
5635	PDC	0.073413686	Tumor_G2
5636	SUCLG2	5.954959223	Tumor_G2
5637	SLC25A5	8.420886066	Tumor_G2
5638	UCP2	8.997944289	Tumor_G2

5639	SOD2	5.252362549	Tumor_G2
5640	NOX4	2.240817021	Tumor_G2
5641	DUOX1	6.677132165	Tumor_G2
5642	DUOXA1	6.84300236	Tumor_G2
5643	DUOX2	6.413293229	Tumor_G2
5644	DUOXA2	4.698247789	Tumor_G2
5645	NDUFA1	8.239460963	Tumor_G2
5646	NDUFA2	7.344963566	Tumor_G2
5647	NDUFA8	7.464609578	Tumor_G2
5648	NDUFB1	5.649661902	Tumor_G2
5649	NDUFB10	7.731530524	Tumor_G2
5650	NDUFB2	7.054846163	Tumor_G2
5651	NDUFB3	6.50564909	Tumor_G2
5652	NDUFB7	8.713594641	Tumor_G2
5653	CYC1	7.396659459	Tumor_G2
5654	ETFB	5.538011514	Tumor_G2
5655	UQCR10	8.070185038	Tumor_G2
5656	UQCRQ	7.821461424	Tumor_G2
5657	UQCC3	4.345609933	Tumor_G2
5658	COX15	4.665192079	Tumor_G2
5659	COX5A	6.999726608	Tumor_G2
5660	COX6B1	8.365980339	Tumor_G2
5661	COX6C	6.440120952	Tumor_G2
5662	COX7A2	6.710997084	Tumor_G2
5663	COX7B	6.145577121	Tumor_G2
5664	COX8A	9.946117968	Tumor_G2
5665	SCO1	4.233593496	Tumor_G2
5666	SURF1	6.847235581	Tumor_G2
5667	ATP6V1E1	6.851233386	Tumor_G2
5668	DLAT	4.822222143	Tumor_G2
5669	PDC	0.078143425	Tumor_G2
5670	SUCLG2	7.09529511	Tumor_G2
5671	SLC25A5	8.368543434	Tumor_G2
5672	UCP2	8.372271555	Tumor_G2
5673	SOD2	4.894669795	Tumor_G2
5674	NOX4	1.83485894	Tumor_G2
5675	DUOX1	8.405977732	Tumor_G2
5676	DUOXA1	7.568114862	Tumor_G2
5677	DUOX2	9.749781095	Tumor_G2
5678	DUOXA2	7.086416072	Tumor_G2

5679	NDUFA1	8.394500839	Tumor_G2
5680	NDUFA2	7.331778953	Tumor_G2
5681	NDUFA8	7.602619119	Tumor_G2
5682	NDUFB1	5.913769738	Tumor_G2
5683	NDUFB10	8.069891897	Tumor_G2
5684	NDUFB2	6.595436981	Tumor_G2
5685	NDUFB3	6.2291513	Tumor_G2
5686	NDUFB7	9.001589043	Tumor_G2
5687	CYC1	7.217236267	Tumor_G2
5688	ETFB	5.290993626	Tumor_G2
5689	UQCR10	7.35282444	Tumor_G2
5690	UQCRQ	7.520641411	Tumor_G2
5691	UQCC3	4.946601224	Tumor_G2
5692	COX15	5.341496066	Tumor_G2
5693	COX5A	7.305304799	Tumor_G2
5694	COX6B1	8.957198432	Tumor_G2
5695	COX6C	6.77879591	Tumor_G2
5696	COX7A2	6.967141136	Tumor_G2
5697	COX7B	6.351644044	Tumor_G2
5698	COX8A	10.22545596	Tumor_G2
5699	SCO1	3.618180777	Tumor_G2
5700	SURF1	7.106225882	Tumor_G2
5701	ATP6V1E1	5.89878249	Tumor_G2
5702	DLAT	5.016474301	Tumor_G2
5703	PDC	0.318575987	Tumor_G2
5704	SUCLG2	6.710900999	Tumor_G2
5705	SLC25A5	7.894544216	Tumor_G2
5706	UCP2	7.704566995	Tumor_G2
5707	SOD2	4.883005758	Tumor_G2
5708	NOX4	1.664953249	Tumor_G2
5709	DUOX1	7.874582402	Tumor_G2
5710	DUOXA1	7.388589812	Tumor_G2
5711	DUOX2	10.5417359	Tumor_G2
5712	DUOXA2	8.492152114	Tumor_G2
5713	NDUFA1	8.059500046	Tumor_G2
5714	NDUFA2	6.661131313	Tumor_G2
5715	NDUFA8	7.181339286	Tumor_G2
5716	NDUFB1	5.82130282	Tumor_G2
5717	NDUFB10	7.584090935	Tumor_G2
5718	NDUFB2	6.460219976	Tumor_G2

5719	NDUFB3	6.490649287	Tumor_G2
5720	NDUFB7	8.598654901	Tumor_G2
5721	CYC1	6.732195028	Tumor_G2
5722	ETFB	5.243595125	Tumor_G2
5723	UQCR10	7.773255541	Tumor_G2
5724	UQCRQ	7.162710256	Tumor_G2
5725	UQCC3	4.728133499	Tumor_G2
5726	COX15	5.011525914	Tumor_G2
5727	COX5A	7.146919553	Tumor_G2
5728	COX6B1	8.711021659	Tumor_G2
5729	COX6C	6.561003805	Tumor_G2
5730	COX7A2	6.874878752	Tumor_G2
5731	COX7B	6.041859137	Tumor_G2
5732	COX8A	9.403125307	Tumor_G2
5733	SCO1	3.528400201	Tumor_G2
5734	SURF1	6.635624209	Tumor_G2
5735	ATP6V1E1	6.859423442	Tumor_G2
5736	DLAT	4.937140923	Tumor_G2
5737	PDC	0.127414371	Tumor_G2
5738	SUCLG2	6.025732663	Tumor_G2
5739	SLC25A5	7.886104614	Tumor_G2
5740	UCP2	9.629834559	Tumor_G2
5741	SOD2	4.71234983	Tumor_G2
5742	NOX4	0.878371266	Tumor_G2
5743	DUOX1	6.121250624	Tumor_G2
5744	DUOXA1	6.276505411	Tumor_G2
5745	DUOX2	7.395924111	Tumor_G2
5746	DUOXA2	5.293762336	Tumor_G2
5747	NDUFA1	7.981207828	Tumor_G2
5748	NDUFA2	6.790845378	Tumor_G2
5749	NDUFA8	7.284555412	Tumor_G2
5750	NDUFB1	5.809608832	Tumor_G2
5751	NDUFB10	7.465386136	Tumor_G2
5752	NDUFB2	7.166232381	Tumor_G2
5753	NDUFB3	6.501608181	Tumor_G2
5754	NDUFB7	8.655149123	Tumor_G2
5755	CYC1	7.281810376	Tumor_G2
5756	ETFB	5.269092828	Tumor_G2
5757	UQCR10	7.847363061	Tumor_G2
5758	UQCRQ	7.224973258	Tumor_G2

5759	UQCC3	4.468893026	Tumor_G2
5760	COX15	5.488319254	Tumor_G2
5761	COX5A	7.245654297	Tumor_G2
5762	COX6B1	8.490440405	Tumor_G2
5763	COX6C	6.413163185	Tumor_G2
5764	COX7A2	6.889062144	Tumor_G2
5765	COX7B	6.262570396	Tumor_G2
5766	COX8A	9.71470728	Tumor_G2
5767	SCO1	3.95323543	Tumor_G2
5768	SURF1	6.722018628	Tumor_G2
5769	ATP6V1E1	6.763599312	Tumor_G2
5770	DLAT	5.768285802	Tumor_G2
5771	PDC	0.065997244	Tumor_G2
5772	SUCLG2	6.718615045	Tumor_G2
5773	SLC25A5	8.79576	Tumor_G2
5774	UCP2	7.491900886	Tumor_G2
5775	SOD2	4.997605576	Tumor_G2
5776	NOX4	0.890149463	Tumor_G2
5777	DUOX1	7.646037415	Tumor_G2
5778	DUOXA1	6.683290366	Tumor_G2
5779	DUOX2	7.483484112	Tumor_G2
5780	DUOXA2	5.180490659	Tumor_G2
5781	NDUFA1	8.47310475	Tumor_G2
5782	NDUFA2	6.946968215	Tumor_G2
5783	NDUFA8	7.654805411	Tumor_G2
5784	NDUFB1	6.154993013	Tumor_G2
5785	NDUFB10	7.293299852	Tumor_G2
5786	NDUFB2	7.556202614	Tumor_G2
5787	NDUFB3	7.126433907	Tumor_G2
5788	NDUFB7	9.131522511	Tumor_G2
5789	CYC1	7.221046869	Tumor_G2
5790	ETFB	5.345314386	Tumor_G2
5791	UQCR10	8.322019965	Tumor_G2
5792	UQCRQ	7.527921727	Tumor_G2
5793	UQCC3	4.306263801	Tumor_G2
5794	COX15	5.628471462	Tumor_G2
5795	COX5A	7.211772355	Tumor_G2
5796	COX6B1	8.767542372	Tumor_G2
5797	COX6C	6.830346339	Tumor_G2
5798	COX7A2	7.017856557	Tumor_G2

5799	COX7B	6.68451212	Tumor_G2
5800	COX8A	9.99521251	Tumor_G2
5801	SCO1	4.460793267	Tumor_G2
5802	SURF1	6.823161491	Tumor_G2
5803	ATP6V1E1	6.996194378	Tumor_G2
5804	DLAT	5.382400346	Tumor_G2
5805	PDC	0.206739181	Tumor_G2
5806	SUCLG2	6.688951669	Tumor_G2
5807	SLC25A5	8.384420082	Tumor_G2
5808	UCP2	8.748682793	Tumor_G2
5809	SOD2	4.870563646	Tumor_G2
5810	NOX4	1.476337091	Tumor_G2
5811	DUOX1	7.463091211	Tumor_G2
5812	DUOXA1	6.466861527	Tumor_G2
5813	DUOX2	8.201223291	Tumor_G2
5814	DUOXA2	6.69134715	Tumor_G2
5815	NDUFA1	8.404610263	Tumor_G2
5816	NDUFA2	7.048148179	Tumor_G2
5817	NDUFA8	7.370252259	Tumor_G2
5818	NDUFB1	6.128919844	Tumor_G2
5819	NDUFB10	7.586684353	Tumor_G2
5820	NDUFB2	7.64564225	Tumor_G2
5821	NDUFB3	6.21775009	Tumor_G2
5822	NDUFB7	8.872541559	Tumor_G2
5823	CYC1	7.315041116	Tumor_G2
5824	ETFB	5.369895204	Tumor_G2
5825	UQCR10	7.950927409	Tumor_G2
5826	UQCRQ	8.094868926	Tumor_G2
5827	UQCC3	4.277387152	Tumor_G2
5828	COX15	4.619284469	Tumor_G2
5829	COX5A	7.532304884	Tumor_G2
5830	COX6B1	8.683771639	Tumor_G2
5831	COX6C	7.025737479	Tumor_G2
5832	COX7A2	7.426295835	Tumor_G2
5833	COX7B	6.416039838	Tumor_G2
5834	COX8A	10.09728584	Tumor_G2
5835	SCO1	3.135097623	Tumor_G2
5836	SURF1	6.808468334	Tumor_G2
5837	ATP6V1E1	6.873639206	Tumor_G2
5838	DLAT	4.505602989	Tumor_G2

5839	PDC	0	Tumor_G2
5840	SUCLG2	6.652415993	Tumor_G2
5841	SLC25A5	8.208682258	Tumor_G2
5842	UCP2	7.676924059	Tumor_G2
5843	SOD2	3.425386921	Tumor_G2
5844	NOX4	0.987964094	Tumor_G2
5845	DUOX1	6.766095045	Tumor_G2
5846	DUOXA1	5.81889249	Tumor_G2
5847	DUOX2	7.498979175	Tumor_G2
5848	DUOXA2	6.066273911	Tumor_G2
5849	NDUFA1	8.013425864	Tumor_G2
5850	NDUFA2	6.957288825	Tumor_G2
5851	NDUFA8	7.324516271	Tumor_G2
5852	NDUFB1	5.664945114	Tumor_G2
5853	NDUFB10	7.657378493	Tumor_G2
5854	NDUFB2	7.12564996	Tumor_G2
5855	NDUFB3	6.592276317	Tumor_G2
5856	NDUFB7	9.02269165	Tumor_G2
5857	CYC1	7.04830347	Tumor_G2
5858	ETFB	5.128653824	Tumor_G2
5859	UQCR10	8.06511644	Tumor_G2
5860	UQCRQ	7.300106848	Tumor_G2
5861	UQCC3	4.299214155	Tumor_G2
5862	COX15	5.16397924	Tumor_G2
5863	COX5A	7.132077893	Tumor_G2
5864	COX6B1	8.536096835	Tumor_G2
5865	COX6C	6.39707604	Tumor_G2
5866	COX7A2	6.612076206	Tumor_G2
5867	COX7B	6.087193441	Tumor_G2
5868	COX8A	9.870781845	Tumor_G2
5869	SCO1	4.276621223	Tumor_G2
5870	SURF1	6.916946854	Tumor_G2
5871	ATP6V1E1	6.655804557	Tumor_G2
5872	DLAT	5.187155448	Tumor_G2
5873	PDC	0.133943421	Tumor_G2
5874	SUCLG2	6.225343912	Tumor_G2
5875	SLC25A5	8.511649645	Tumor_G2
5876	UCP2	6.805967649	Tumor_G2
5877	SOD2	5.162548063	Tumor_G2
5878	NOX4	0.995442697	Tumor_G2

5879	DUOX1	8.142157143	Tumor_G2
5880	DUOXA1	6.998775882	Tumor_G2
5881	DUOX2	7.953433471	Tumor_G2
5882	DUOXA2	6.494397094	Tumor_G2
5883	NDUFA1	8.15016676	Tumor_G2
5884	NDUFA2	6.961543093	Tumor_G2
5885	NDUFA8	7.314117276	Tumor_G2
5886	NDUFB1	5.739256612	Tumor_G2
5887	NDUFB10	7.761277471	Tumor_G2
5888	NDUFB2	6.732774272	Tumor_G2
5889	NDUFB3	6.249770432	Tumor_G2
5890	NDUFB7	8.933372652	Tumor_G2
5891	CYC1	7.057129473	Tumor_G2
5892	ETFB	5.376414635	Tumor_G2
5893	UQCR10	7.80259569	Tumor_G2
5894	UQCRQ	7.251804432	Tumor_G2
5895	UQCC3	4.607433678	Tumor_G2
5896	COX15	5.102171266	Tumor_G2
5897	COX5A	7.040235741	Tumor_G2
5898	COX6B1	8.876025483	Tumor_G2
5899	COX6C	6.535611368	Tumor_G2
5900	COX7A2	6.841750957	Tumor_G2
5901	COX7B	6.08935425	Tumor_G2
5902	COX8A	9.776178431	Tumor_G2
5903	SCO1	3.377912573	Tumor_G2
5904	SURF1	7.00025906	Tumor_G2
5905	ATP6V1E1	6.88853097	Tumor_G2
5906	DLAT	4.80351728	Tumor_G2
5907	PDC	0.071244958	Tumor_G2
5908	SUCLG2	6.341851505	Tumor_G2
5909	SLC25A5	8.008653641	Tumor_G2
5910	UCP2	8.257176088	Tumor_G2
5911	SOD2	4.923902495	Tumor_G2
5912	NOX4	1.040083357	Tumor_G2
5913	DUOX1	6.906244272	Tumor_G2
5914	DUOXA1	6.900197049	Tumor_G2
5915	DUOX2	7.997902127	Tumor_G2
5916	DUOXA2	6.586774613	Tumor_G2
5917	NDUFA1	8.533411407	Tumor_G2
5918	NDUFA2	7.101303649	Tumor_G2

5919	NDUFA8	7.515512291	Tumor_G2
5920	NDUFB1	6.212524061	Tumor_G2
5921	NDUFB10	7.64216563	Tumor_G2
5922	NDUFB2	7.162881417	Tumor_G2
5923	NDUFB3	6.739402838	Tumor_G2
5924	NDUFB7	8.952897082	Tumor_G2
5925	CYC1	7.093328076	Tumor_G2
5926	ETFB	5.594770472	Tumor_G2
5927	UQCR10	7.489344217	Tumor_G2
5928	UQCRQ	7.692090565	Tumor_G2
5929	UQCC3	4.762859407	Tumor_G2
5930	COX15	5.064888462	Tumor_G2
5931	COX5A	7.270755924	Tumor_G2
5932	COX6B1	8.859948455	Tumor_G2
5933	COX6C	6.799786299	Tumor_G2
5934	COX7A2	7.125268151	Tumor_G2
5935	COX7B	6.419445539	Tumor_G2
5936	COX8A	10.06786482	Tumor_G2
5937	SCO1	3.755870777	Tumor_G2
5938	SURF1	6.8562483	Tumor_G2
5939	ATP6V1E1	6.193596675	Tumor_G2
5940	DLAT	5.114789398	Tumor_G2
5941	PDC	0.301890352	Tumor_G2
5942	SUCLG2	6.196097615	Tumor_G2
5943	SLC25A5	7.768191726	Tumor_G2
5944	UCP2	8.338245744	Tumor_G2
5945	SOD2	4.617508141	Tumor_G2
5946	NOX4	1.668103492	Tumor_G2
5947	DUOX1	8.343431595	Tumor_G2
5948	DUOXA1	7.797161212	Tumor_G2
5949	DUOX2	9.600139436	Tumor_G2
5950	DUOXA2	7.680060144	Tumor_G2
5951	NDUFA1	8.549269711	Tumor_G2
5952	NDUFA2	7.125378636	Tumor_G2
5953	NDUFA8	7.346401693	Tumor_G2
5954	NDUFB1	6.133577025	Tumor_G2
5955	NDUFB10	7.467593057	Tumor_G2
5956	NDUFB2	7.336123263	Tumor_G2
5957	NDUFB3	6.162059904	Tumor_G2
5958	NDUFB7	8.881845207	Tumor_G2

5959	CYC1	6.954316312	Tumor_G2
5960	ETFB	5.376665955	Tumor_G2
5961	UQCR10	7.068990836	Tumor_G2
5962	UQCRQ	7.650805085	Tumor_G2
5963	UQCC3	3.636062083	Tumor_G2
5964	COX15	4.837532129	Tumor_G2
5965	COX5A	7.313253959	Tumor_G2
5966	COX6B1	8.523552156	Tumor_G2
5967	COX6C	6.196906523	Tumor_G2
5968	COX7A2	6.802597363	Tumor_G2
5969	COX7B	6.456170079	Tumor_G2
5970	COX8A	9.384557086	Tumor_G2
5971	SCO1	4.00024529	Tumor_G2
5972	SURF1	6.887643905	Tumor_G2
5973	ATP6V1E1	6.338147301	Tumor_G2
5974	DLAT	5.024577041	Tumor_G2
5975	PDC	0.043299934	Tumor_G2
5976	SUCLG2	5.762178302	Tumor_G2
5977	SLC25A5	8.986123031	Tumor_G2
5978	UCP2	5.310780758	Tumor_G2
5979	SOD2	4.680768023	Tumor_G2
5980	NOX4	0.686171876	Tumor_G2
5981	DUOX1	6.361781781	Tumor_G2
5982	DUOXA1	5.940176642	Tumor_G2
5983	DUOX2	6.566986084	Tumor_G2
5984	DUOXA2	7.294907992	Tumor_G2
5985	NDUFA1	8.067614109	Tumor_G2
5986	NDUFA2	6.874426932	Tumor_G2
5987	NDUFA8	7.156155661	Tumor_G2
5988	NDUFB1	5.570508816	Tumor_G2
5989	NDUFB10	7.640763887	Tumor_G2
5990	NDUFB2	6.466324134	Tumor_G2
5991	NDUFB3	6.189256253	Tumor_G2
5992	NDUFB7	8.753538552	Tumor_G2
5993	CYC1	6.965739701	Tumor_G2
5994	ETFB	4.496172354	Tumor_G2
5995	UQCR10	7.380913413	Tumor_G2
5996	UQCRQ	7.037975945	Tumor_G2
5997	UQCC3	4.67216855	Tumor_G2
5998	COX15	5.275061635	Tumor_G2

5999	COX5A	6.757457524	Tumor_G2
6000	COX6B1	8.760559492	Tumor_G2
6001	COX6C	6.215783799	Tumor_G2
6002	COX7A2	6.789526825	Tumor_G2
6003	COX7B	5.836998403	Tumor_G2
6004	COX8A	9.47820377	Tumor_G2
6005	SCO1	3.686968025	Tumor_G2
6006	SURF1	6.973640844	Tumor_G2
6007	ATP6V1E1	6.773773997	Tumor_G2
6008	DLAT	5.478467929	Tumor_G2
6009	PDC	0.07448003	Tumor_G2
6010	SUCLG2	5.997375753	Tumor_G2
6011	SLC25A5	7.814614267	Tumor_G2
6012	UCP2	8.297157279	Tumor_G2
6013	SOD2	4.979472933	Tumor_G2
6014	NOX4	2.762101092	Tumor_G2
6015	DUOX1	5.750259232	Tumor_G2
6016	DUOXA1	6.555770797	Tumor_G2
6017	DUOX2	6.984900754	Tumor_G2
6018	DUOXA2	7.003081564	Tumor_G2
6019	NDUFA1	8.244612851	Tumor_G2
6020	NDUFA2	7.0376258	Tumor_G2
6021	NDUFA8	7.377292262	Tumor_G2
6022	NDUFB1	6.039744205	Tumor_G2
6023	NDUFB10	7.951042836	Tumor_G2
6024	NDUFB2	6.618423923	Tumor_G2
6025	NDUFB3	6.194752869	Tumor_G2
6026	NDUFB7	8.535402125	Tumor_G2
6027	CYC1	7.124579953	Tumor_G2
6028	ETFB	5.47962914	Tumor_G2
6029	UQCR10	7.954955799	Tumor_G2
6030	UQCRQ	7.278455822	Tumor_G2
6031	UQCC3	5.073336243	Tumor_G2
6032	COX15	5.223388814	Tumor_G2
6033	COX5A	7.115930299	Tumor_G2
6034	COX6B1	8.914065182	Tumor_G2
6035	COX6C	6.554107186	Tumor_G2
6036	COX7A2	6.797048842	Tumor_G2
6037	COX7B	6.174132254	Tumor_G2
6038	COX8A	9.955625274	Tumor_G2

6039	SCO1	3.272574869	Tumor_G2
6040	SURF1	6.962698053	Tumor_G2
6041	ATP6V1E1	6.578769807	Tumor_G2
6042	DLAT	4.879525543	Tumor_G2
6043	PDC	0	Tumor_G2
6044	SUCLG2	5.959545507	Tumor_G2
6045	SLC25A5	8.035801335	Tumor_G2
6046	UCP2	7.378063801	Tumor_G2
6047	SOD2	4.387072063	Tumor_G2
6048	NOX4	1.553076875	Tumor_G2
6049	DUOX1	7.618480953	Tumor_G2
6050	DUOXA1	7.527834544	Tumor_G2
6051	DUOX2	8.558507496	Tumor_G2
6052	DUOXA2	6.912740391	Tumor_G2
6053	NDUFA1	8.043684046	Tumor_G2
6054	NDUFA2	6.616221265	Tumor_G2
6055	NDUFA8	7.26198181	Tumor_G2
6056	NDUFB1	5.924976398	Tumor_G2
6057	NDUFB10	7.925459161	Tumor_G2
6058	NDUFB2	6.569691477	Tumor_G2
6059	NDUFB3	6.649080029	Tumor_G2
6060	NDUFB7	8.826230526	Tumor_G2
6061	CYC1	7.06389526	Tumor_G2
6062	ETFB	4.747745389	Tumor_G2
6063	UQCR10	7.764657671	Tumor_G2
6064	UQCRQ	7.402386134	Tumor_G2
6065	UQCC3	4.89605651	Tumor_G2
6066	COX15	4.511273101	Tumor_G2
6067	COX5A	7.287329781	Tumor_G2
6068	COX6B1	8.924110958	Tumor_G2
6069	COX6C	6.602521341	Tumor_G2
6070	COX7A2	6.968229486	Tumor_G2
6071	COX7B	6.115685178	Tumor_G2
6072	COX8A	9.536521713	Tumor_G2
6073	SCO1	3.187828998	Tumor_G2
6074	SURF1	6.845283326	Tumor_G2
6075	ATP6V1E1	6.925276899	Tumor_G2
6076	DLAT	4.678797558	Tumor_G2
6077	PDC	0.196072866	Tumor_G2
6078	SUCLG2	5.883527988	Tumor_G2

6079	SLC25A5	7.687162707	Tumor_G2
6080	UCP2	9.347167531	Tumor_G2
6081	SOD2	3.942449938	Tumor_G2
6082	NOX4	2.552955415	Tumor_G2
6083	DUOX1	6.443333081	Tumor_G2
6084	DUOXA1	6.702837219	Tumor_G2
6085	DUOX2	7.100182409	Tumor_G2
6086	DUOXA2	4.644041619	Tumor_G2
6087	NDUFA1	8.385254209	Tumor_G2
6088	NDUFA2	7.041763964	Tumor_G2
6089	NDUFA8	7.446527893	Tumor_G2
6090	NDUFB1	6.139296889	Tumor_G2
6091	NDUFB10	7.704786734	Tumor_G2
6092	NDUFB2	6.993811435	Tumor_G2
6093	NDUFB3	6.46209847	Tumor_G2
6094	NDUFB7	9.140016426	Tumor_G2
6095	CYC1	7.234739654	Tumor_G2
6096	ETFB	5.421753367	Tumor_G2
6097	UQCR10	7.982551905	Tumor_G2
6098	UQCRQ	7.453964865	Tumor_G2
6099	UQCC3	4.619090094	Tumor_G2
6100	COX15	5.076138059	Tumor_G2
6101	COX5A	7.404045606	Tumor_G2
6102	COX6B1	9.079464826	Tumor_G2
6103	COX6C	6.987639112	Tumor_G2
6104	COX7A2	7.225951981	Tumor_G2
6105	COX7B	6.526706523	Tumor_G2
6106	COX8A	9.767609945	Tumor_G2
6107	SCO1	3.387064506	Tumor_G2
6108	SURF1	7.126094416	Tumor_G2
6109	ATP6V1E1	6.998202613	Tumor_G2
6110	DLAT	5.008830984	Tumor_G2
6111	PDC	0.085498992	Tumor_G2
6112	SUCLG2	6.627188061	Tumor_G2
6113	SLC25A5	8.698837197	Tumor_G2
6114	UCP2	7.229985575	Tumor_G2
6115	SOD2	4.658830301	Tumor_G2
6116	NOX4	1.683822681	Tumor_G2
6117	DUOX1	7.373971619	Tumor_G2
6118	DUOXA1	6.803321875	Tumor_G2

6119	DUOX2	7.847887422	Tumor_G2
6120	DUOXA2	5.257728417	Tumor_G2
6121	NDUFA1	8.479349289	Tumor_G2
6122	NDUFA2	7.242084364	Tumor_G2
6123	NDUFA8	7.457141811	Tumor_G2
6124	NDUFB1	6.130352872	Tumor_G2
6125	NDUFB10	7.934387183	Tumor_G2
6126	NDUFB2	6.629726579	Tumor_G2
6127	NDUFB3	6.260780674	Tumor_G2
6128	NDUFB7	9.279951511	Tumor_G2
6129	CYC1	7.159660298	Tumor_G2
6130	ETFB	5.738181388	Tumor_G2
6131	UQCR10	8.033982744	Tumor_G2
6132	UQCRQ	7.297679147	Tumor_G2
6133	UQCC3	5.450437112	Tumor_G2
6134	COX15	5.463168177	Tumor_G2
6135	COX5A	6.845482363	Tumor_G2
6136	COX6B1	8.780029189	Tumor_G2
6137	COX6C	6.553884411	Tumor_G2
6138	COX7A2	6.752818088	Tumor_G2
6139	COX7B	6.004752542	Tumor_G2
6140	COX8A	10.01436714	Tumor_G2
6141	SCO1	3.528289531	Tumor_G2
6142	SURF1	7.203073617	Tumor_G2
6143	ATP6V1E1	6.441789901	Tumor_G2
6144	DLAT	5.020654796	Tumor_G2
6145	PDC	0.129464626	Tumor_G2
6146	SUCLG2	6.022300792	Tumor_G2
6147	SLC25A5	7.666704205	Tumor_G2
6148	UCP2	6.349235753	Tumor_G2
6149	SOD2	4.764268165	Tumor_G2
6150	NOX4	0.947119153	Tumor_G2
6151	DUOX1	7.211658482	Tumor_G2
6152	DUOXA1	6.752834901	Tumor_G2
6153	DUOX2	7.647851895	Tumor_G2
6154	DUOXA2	5.70098537	Tumor_G2
6155	NDUFA1	8.004971408	Tumor_G2
6156	NDUFA2	6.58672736	Tumor_G2
6157	NDUFA8	6.850291434	Tumor_G2
6158	NDUFB1	5.686238187	Tumor_G2

6159	NDUFB10	6.847570812	Tumor_G2
6160	NDUFB2	6.50899193	Tumor_G2
6161	NDUFB3	5.979531788	Tumor_G2
6162	NDUFB7	8.241196711	Tumor_G2
6163	CYC1	6.801639686	Tumor_G2
6164	ETFB	4.853185449	Tumor_G2
6165	UQCR10	7.288343034	Tumor_G2
6166	UQCRQ	7.202047812	Tumor_G2
6167	UQCC3	4.073810259	Tumor_G2
6168	COX15	4.408350641	Tumor_G2
6169	COX5A	6.76321464	Tumor_G2
6170	COX6B1	8.289516876	Tumor_G2
6171	COX6C	6.315465747	Tumor_G2
6172	COX7A2	6.618097892	Tumor_G2
6173	COX7B	5.918978772	Tumor_G2
6174	COX8A	9.318708055	Tumor_G2
6175	SCO1	2.68907642	Tumor_G2
6176	SURF1	6.040892713	Tumor_G2
6177	ATP6V1E1	6.648620766	Tumor_G2
6178	DLAT	4.274160103	Tumor_G2
6179	PDC	0	Tumor_G2
6180	SUCLG2	6.171676365	Tumor_G2
6181	SLC25A5	7.732145044	Tumor_G2
6182	UCP2	7.153756306	Tumor_G2
6183	SOD2	3.493574169	Tumor_G2
6184	NOX4	1.367294193	Tumor_G2
6185	DUOX1	6.798802886	Tumor_G2
6186	DUOXA1	6.113673812	Tumor_G2
6187	DUOX2	8.543398587	Tumor_G2
6188	DUOXA2	7.331513142	Tumor_G2
6189	NDUFA1	8.383368944	Tumor_G2
6190	NDUFA2	7.123141829	Tumor_G2
6191	NDUFA8	7.311097791	Tumor_G2
6192	NDUFB1	6.237172281	Tumor_G2
6193	NDUFB10	7.827132063	Tumor_G2
6194	NDUFB2	7.280072586	Tumor_G2
6195	NDUFB3	6.742157117	Tumor_G2
6196	NDUFB7	9.032639407	Tumor_G2
6197	CYC1	7.13571579	Tumor_G2
6198	ETFB	5.295515618	Tumor_G2

6199	UQCR10	8.072939482	Tumor_G2
6200	UQCRQ	7.659152652	Tumor_G2
6201	UQCC3	4.738705664	Tumor_G2
6202	COX15	4.971300028	Tumor_G2
6203	COX5A	7.240813253	Tumor_G2
6204	COX6B1	8.795656569	Tumor_G2
6205	COX6C	6.956702291	Tumor_G2
6206	COX7A2	7.186265461	Tumor_G2
6207	COX7B	6.458296463	Tumor_G2
6208	COX8A	10.04891436	Tumor_G2
6209	SCO1	3.529661621	Tumor_G2
6210	SURF1	6.729394813	Tumor_G2
6211	ATP6V1E1	7.007971962	Tumor_G2
6212	DLAT	5.124537685	Tumor_G2
6213	PDC	0.450892666	Tumor_G2
6214	SUCLG2	6.434888954	Tumor_G2
6215	SLC25A5	8.26772899	Tumor_G2
6216	UCP2	7.796728859	Tumor_G2
6217	SOD2	4.479093348	Tumor_G2
6218	NOX4	1.287836792	Tumor_G2
6219	DUOX1	7.615607562	Tumor_G2
6220	DUOXA1	7.35630683	Tumor_G2
6221	DUOX2	8.564977399	Tumor_G2
6222	DUOXA2	6.420466384	Tumor_G2
6223	NDUFA1	8.401057897	Tumor_G2
6224	NDUFA2	6.873415992	Tumor_G2
6225	NDUFA8	7.163150505	Tumor_G2
6226	NDUFB1	6.167740507	Tumor_G2
6227	NDUFB10	7.920955192	Tumor_G2
6228	NDUFB2	7.051037225	Tumor_G2
6229	NDUFB3	6.761218574	Tumor_G2
6230	NDUFB7	9.016045563	Tumor_G2
6231	CYC1	6.998966419	Tumor_G2
6232	ETFB	5.430696492	Tumor_G2
6233	UQCR10	7.952447889	Tumor_G2
6234	UQCRQ	7.668037779	Tumor_G2
6235	UQCC3	4.4785316	Tumor_G2
6236	COX15	4.684045787	Tumor_G2
6237	COX5A	7.385868766	Tumor_G2
6238	COX6B1	8.940633757	Tumor_G2

6239	COX6C	7.000375684	Tumor_G2
6240	COX7A2	7.266968082	Tumor_G2
6241	COX7B	6.459229112	Tumor_G2
6242	COX8A	9.875995551	Tumor_G2
6243	SCO1	3.366901527	Tumor_G2
6244	SURF1	6.877157233	Tumor_G2
6245	ATP6V1E1	6.972530092	Tumor_G2
6246	DLAT	4.938975629	Tumor_G2
6247	PDC	0.280047103	Tumor_G2
6248	SUCLG2	6.113886026	Tumor_G2
6249	SLC25A5	8.004408853	Tumor_G2
6250	UCP2	9.008526944	Tumor_G2
6251	SOD2	5.155701851	Tumor_G2
6252	NOX4	2.280564873	Tumor_G2
6253	DUOX1	7.149136503	Tumor_G2
6254	DUOXA1	7.415149412	Tumor_G2
6255	DUOX2	7.916583687	Tumor_G2
6256	DUOXA2	5.826237806	Tumor_G2
6257	NDUFA1	8.299299024	Tumor_G2
6258	NDUFA2	7.056280854	Tumor_G2
6259	NDUFA8	7.360247526	Tumor_G2
6260	NDUFB1	5.956001249	Tumor_G2
6261	NDUFB10	7.833075372	Tumor_G2
6262	NDUFB2	6.671437935	Tumor_G2
6263	NDUFB3	6.523684747	Tumor_G2
6264	NDUFB7	9.015204297	Tumor_G2
6265	CYC1	6.936112746	Tumor_G2
6266	ETFB	5.006549444	Tumor_G2
6267	UQCR10	7.795684406	Tumor_G2
6268	UQCRQ	7.634958131	Tumor_G2
6269	UQCC3	4.625235652	Tumor_G2
6270	COX15	4.308867338	Tumor_G2
6271	COX5A	7.352143905	Tumor_G2
6272	COX6B1	9.084250848	Tumor_G2
6273	COX6C	6.643301968	Tumor_G2
6274	COX7A2	7.349884393	Tumor_G2
6275	COX7B	6.319501799	Tumor_G2
6276	COX8A	9.800130165	Tumor_G2
6277	SCO1	3.221674755	Tumor_G2
6278	SURF1	6.54670491	Tumor_G2

6279	ATP6V1E1	7.260639198	Tumor_G2
6280	DLAT	4.690144146	Tumor_G2
6281	PDC	0.11469446	Tumor_G2
6282	SUCLG2	5.690119343	Tumor_G2
6283	SLC25A5	7.95700026	Tumor_G2
6284	UCP2	9.760781249	Tumor_G2
6285	SOD2	4.546548659	Tumor_G2
6286	NOX4	3.771788702	Tumor_G2
6287	DUOX1	6.385307539	Tumor_G2
6288	DUOXA1	6.877596314	Tumor_G2
6289	DUOX2	6.709242867	Tumor_G2
6290	DUOXA2	4.635746211	Tumor_G2
6291	NDUFA1	8.092506357	Tumor_G2
6292	NDUFA2	6.621354875	Tumor_G2
6293	NDUFA8	7.168367015	Tumor_G2
6294	NDUFB1	5.780152021	Tumor_G2
6295	NDUFB10	7.820676748	Tumor_G2
6296	NDUFB2	7.008843593	Tumor_G2
6297	NDUFB3	6.282007576	Tumor_G2
6298	NDUFB7	8.779228071	Tumor_G2
6299	CYC1	7.410219524	Tumor_G2
6300	ETFB	5.282962503	Tumor_G2
6301	UQCR10	7.714087627	Tumor_G2
6302	UQCRQ	7.32259548	Tumor_G2
6303	UQCC3	4.788688023	Tumor_G2
6304	COX15	5.188838559	Tumor_G2
6305	COX5A	7.377681641	Tumor_G2
6306	COX6B1	8.712301238	Tumor_G2
6307	COX6C	6.443315618	Tumor_G2
6308	COX7A2	6.825382975	Tumor_G2
6309	COX7B	6.085044501	Tumor_G2
6310	COX8A	9.943767273	Tumor_G2
6311	SCO1	3.720215625	Tumor_G2
6312	SURF1	6.910857639	Tumor_G2
6313	ATP6V1E1	7.219019757	Tumor_G2
6314	DLAT	4.944283092	Tumor_G2
6315	PDC	0	Tumor_G2
6316	SUCLG2	6.495950178	Tumor_G2
6317	SLC25A5	8.48513515	Tumor_G2
6318	UCP2	8.50529878	Tumor_G2

6319	SOD2	4.272573974	Tumor_G2
6320	NOX4	1.924917282	Tumor_G2
6321	DUOX1	6.691814828	Tumor_G2
6322	DUOXA1	6.831422032	Tumor_G2
6323	DUOX2	7.391218569	Tumor_G2
6324	DUOXA2	5.838747656	Tumor_G2
6325	NDUFA1	7.969278743	Tumor_G2
6326	NDUFA2	6.731320072	Tumor_G2
6327	NDUFA8	7.272133968	Tumor_G2
6328	NDUFB1	6.082618703	Tumor_G2
6329	NDUFB10	7.751314952	Tumor_G2
6330	NDUFB2	6.65265418	Tumor_G2
6331	NDUFB3	6.367859628	Tumor_G2
6332	NDUFB7	8.928273221	Tumor_G2
6333	CYC1	6.844829081	Tumor_G2
6334	ETFB	5.289350717	Tumor_G2
6335	UQCR10	7.691702424	Tumor_G2
6336	UQCRQ	7.335422689	Tumor_G2
6337	UQCC3	4.570990896	Tumor_G2
6338	COX15	4.723522299	Tumor_G2
6339	COX5A	6.832798024	Tumor_G2
6340	COX6B1	8.646961409	Tumor_G2
6341	COX6C	6.528198242	Tumor_G2
6342	COX7A2	6.678573564	Tumor_G2
6343	COX7B	6.178353007	Tumor_G2
6344	COX8A	9.714261924	Tumor_G2
6345	SCO1	3.364896594	Tumor_G2
6346	SURF1	6.467973688	Tumor_G2
6347	ATP6V1E1	6.799018051	Tumor_G2
6348	DLAT	4.912818236	Tumor_G2
6349	PDC	0.137910648	Tumor_G2
6350	SUCLG2	5.9863682	Tumor_G2
6351	SLC25A5	7.556056303	Tumor_G2
6352	UCP2	7.634882601	Tumor_G2
6353	SOD2	4.645159558	Tumor_G2
6354	NOX4	2.593266671	Tumor_G2
6355	DUOX1	8.109962939	Tumor_G2
6356	DUOXA1	7.877493316	Tumor_G2
6357	DUOX2	9.368483473	Tumor_G2
6358	DUOXA2	6.887621713	Tumor_G2

6359	NDUFA1	8.444199544	Tumor_G2
6360	NDUFA2	6.779725124	Tumor_G2
6361	NDUFA8	7.449950097	Tumor_G2
6362	NDUFB1	6.088675658	Tumor_G2
6363	NDUFB10	7.731508589	Tumor_G2
6364	NDUFB2	6.32636914	Tumor_G2
6365	NDUFB3	6.624015357	Tumor_G2
6366	NDUFB7	8.951215943	Tumor_G2
6367	CYC1	6.78807081	Tumor_G2
6368	ETFB	5.059442267	Tumor_G2
6369	UQCR10	7.788662059	Tumor_G2
6370	UQCRQ	7.458803997	Tumor_G2
6371	UQCC3	4.336485986	Tumor_G2
6372	COX15	4.928284137	Tumor_G2
6373	COX5A	7.226533175	Tumor_G2
6374	COX6B1	9.152950901	Tumor_G2
6375	COX6C	6.628351097	Tumor_G2
6376	COX7A2	6.914873962	Tumor_G2
6377	COX7B	6.621589986	Tumor_G2
6378	COX8A	9.619304455	Tumor_G2
6379	SCO1	3.128905327	Tumor_G2
6380	SURF1	6.708268904	Tumor_G2
6381	ATP6V1E1	6.574645009	Tumor_G2
6382	DLAT	4.775742496	Tumor_G2
6383	PDC	0.073280446	Tumor_G2
6384	SUCLG2	5.945194858	Tumor_G2
6385	SLC25A5	8.302990171	Tumor_G2
6386	UCP2	7.610015083	Tumor_G2
6387	SOD2	5.447196543	Tumor_G2
6388	NOX4	1.959743545	Tumor_G2
6389	DUOX1	7.01641008	Tumor_G2
6390	DUOXA1	6.725398157	Tumor_G2
6391	DUOX2	7.71669628	Tumor_G2
6392	DUOXA2	5.65078726	Tumor_G2
6393	NDUFA1	8.002999899	Tumor_G2
6394	NDUFA2	6.698082926	Tumor_G2
6395	NDUFA8	7.229630571	Tumor_G2
6396	NDUFB1	5.690675161	Tumor_G2
6397	NDUFB10	7.778235255	Tumor_G2
6398	NDUFB2	6.933922821	Tumor_G2

6399	NDUFB3	6.323763302	Tumor_G2
6400	NDUFB7	8.728990206	Tumor_G2
6401	CYC1	7.182772901	Tumor_G2
6402	ETFB	4.898522264	Tumor_G2
6403	UQCR10	7.675872836	Tumor_G2
6404	UQCRQ	7.240121365	Tumor_G2
6405	UQCC3	4.414303026	Tumor_G2
6406	COX15	5.138137015	Tumor_G2
6407	COX5A	7.540694416	Tumor_G2
6408	COX6B1	8.721402475	Tumor_G2
6409	COX6C	6.473058599	Tumor_G2
6410	COX7A2	6.920539492	Tumor_G2
6411	COX7B	6.198653571	Tumor_G2
6412	COX8A	9.571618305	Tumor_G2
6413	SCO1	3.638352048	Tumor_G2
6414	SURF1	6.56116318	Tumor_G2
6415	ATP6V1E1	7.340668989	Tumor_G2
6416	DLAT	5.397295999	Tumor_G2
6417	PDC	0	Tumor_G2
6418	SUCLG2	6.425065039	Tumor_G2
6419	SLC25A5	8.463400013	Tumor_G2
6420	UCP2	7.397912163	Tumor_G2
6421	SOD2	5.341203114	Tumor_G2
6422	NOX4	1.30620555	Tumor_G2
6423	DUOX1	5.911733684	Tumor_G2
6424	DUOXA1	6.167437635	Tumor_G2
6425	DUOX2	6.114422172	Tumor_G2
6426	DUOXA2	4.509215047	Tumor_G2
6427	NDUFA1	8.107075907	Tumor_G2
6428	NDUFA2	6.838634379	Tumor_G2
6429	NDUFA8	7.12210327	Tumor_G2
6430	NDUFB1	6.030190133	Tumor_G2
6431	NDUFB10	7.458563823	Tumor_G2
6432	NDUFB2	6.607802708	Tumor_G2
6433	NDUFB3	6.55663804	Tumor_G2
6434	NDUFB7	8.849996961	Tumor_G2
6435	CYC1	7.042817679	Tumor_G2
6436	ETFB	4.995418801	Tumor_G2
6437	UQCR10	7.633468121	Tumor_G2
6438	UQCRQ	7.320033482	Tumor_G2

6439	UQCC3	4.703931615	Tumor_G2
6440	COX15	4.793140322	Tumor_G2
6441	COX5A	6.896005503	Tumor_G2
6442	COX6B1	8.660903676	Tumor_G2
6443	COX6C	6.548226602	Tumor_G2
6444	COX7A2	6.797618679	Tumor_G2
6445	COX7B	6.087379522	Tumor_G2
6446	COX8A	9.618080146	Tumor_G2
6447	SCO1	3.328430974	Tumor_G2
6448	SURF1	6.440935771	Tumor_G2
6449	ATP6V1E1	6.591566669	Tumor_G2
6450	DLAT	4.77000134	Tumor_G2
6451	PDC	0.170558492	Tumor_G2
6452	SUCLG2	5.869435997	Tumor_G2
6453	SLC25A5	8.076857927	Tumor_G2
6454	UCP2	7.394233921	Tumor_G2
6455	SOD2	4.593677352	Tumor_G2
6456	NOX4	1.961375571	Tumor_G2
6457	DUOX1	7.908587972	Tumor_G2
6458	DUOXA1	7.763221298	Tumor_G2
6459	DUOX2	9.021905404	Tumor_G2
6460	DUOXA2	6.496012438	Tumor_G2
6461	NDUFA1	8.291593069	Tumor_G2
6462	NDUFA2	6.882442383	Tumor_G2
6463	NDUFA8	7.249136126	Tumor_G2
6464	NDUFB1	5.953109989	Tumor_G2
6465	NDUFB10	7.571944318	Tumor_G2
6466	NDUFB2	6.598976871	Tumor_G2
6467	NDUFB3	6.463448278	Tumor_G2
6468	NDUFB7	8.895498481	Tumor_G2
6469	CYC1	6.955830448	Tumor_G2
6470	ETFB	4.897022395	Tumor_G2
6471	UQCR10	7.799666479	Tumor_G2
6472	UQCRQ	7.415649958	Tumor_G2
6473	UQCC3	4.607014259	Tumor_G2
6474	COX15	4.61498426	Tumor_G2
6475	COX5A	7.001834441	Tumor_G2
6476	COX6B1	8.874503408	Tumor_G2
6477	COX6C	6.709941982	Tumor_G2
6478	COX7A2	7.164279896	Tumor_G2

6479	COX7B	6.359692545	Tumor_G2
6480	COX8A	9.818547591	Tumor_G2
6481	SCO1	3.274575292	Tumor_G2
6482	SURF1	6.714364261	Tumor_G2
6483	ATP6V1E1	7.000731105	Tumor_G2
6484	DLAT	4.898270505	Tumor_G2
6485	PDC	0.3131167	Tumor_G2
6486	SUCLG2	6.107274144	Tumor_G2
6487	SLC25A5	8.308557114	Tumor_G2
6488	UCP2	7.513311652	Tumor_G2
6489	SOD2	4.503528491	Tumor_G2
6490	NOX4	2.61980098	Tumor_G2
6491	DUOX1	7.42284498	Tumor_G2
6492	DUOXA1	7.413172181	Tumor_G2
6493	DUOX2	8.033689565	Tumor_G2
6494	DUOXA2	5.411245892	Tumor_G2
6495	NDUFA1	7.830680211	Tumor_G2
6496	NDUFA2	6.754689348	Tumor_G2
6497	NDUFA8	6.881705066	Tumor_G2
6498	NDUFB1	5.503914705	Tumor_G2
6499	NDUFB10	7.327850346	Tumor_G2
6500	NDUFB2	6.584093524	Tumor_G2
6501	NDUFB3	6.228021216	Tumor_G2
6502	NDUFB7	8.42188338	Tumor_G2
6503	CYC1	7.02099726	Tumor_G2
6504	ETFB	4.608646574	Tumor_G2
6505	UQCR10	7.620625396	Tumor_G2
6506	UQCRQ	7.039564331	Tumor_G2
6507	UQCC3	4.158808801	Tumor_G2
6508	COX15	5.214370362	Tumor_G2
6509	COX5A	6.966311727	Tumor_G2
6510	COX6B1	8.557674576	Tumor_G2
6511	COX6C	6.458900882	Tumor_G2
6512	COX7A2	6.90415852	Tumor_G2
6513	COX7B	5.994879227	Tumor_G2
6514	COX8A	9.483040292	Tumor_G2
6515	SCO1	3.262452365	Tumor_G2
6516	SURF1	6.056116743	Tumor_G2
6517	ATP6V1E1	7.283597585	Tumor_G2
6518	DLAT	5.153458102	Tumor_G2

6519	PDC	0.144811632	Tumor_G2
6520	SUCLG2	6.754934167	Tumor_G2
6521	SLC25A5	8.445455245	Tumor_G2
6522	UCP2	8.184565708	Tumor_G2
6523	SOD2	4.972504382	Tumor_G2
6524	NOX4	1.780388953	Tumor_G2
6525	DUOX1	6.768805341	Tumor_G2
6526	DUOXA1	6.745477122	Tumor_G2
6527	DUOX2	6.938747221	Tumor_G2
6528	DUOXA2	4.770525275	Tumor_G2
6529	NDUFA1	7.980287906	Tumor_G2
6530	NDUFA2	6.625958482	Tumor_G2
6531	NDUFA8	7.061432213	Tumor_G2
6532	NDUFB1	5.80487315	Tumor_G2
6533	NDUFB10	7.283613747	Tumor_G2
6534	NDUFB2	6.281646264	Tumor_G2
6535	NDUFB3	6.234772701	Tumor_G2
6536	NDUFB7	8.529079368	Tumor_G2
6537	CYC1	6.938848208	Tumor_G2
6538	ETFB	4.514564758	Tumor_G2
6539	UQCR10	7.346090307	Tumor_G2
6540	UQCRQ	7.141285086	Tumor_G2
6541	UQCC3	4.469831905	Tumor_G2
6542	COX15	4.736277387	Tumor_G2
6543	COX5A	6.673706303	Tumor_G2
6544	COX6B1	8.597259268	Tumor_G2
6545	COX6C	6.501941857	Tumor_G2
6546	COX7A2	6.783949094	Tumor_G2
6547	COX7B	6.080780424	Tumor_G2
6548	COX8A	9.26635688	Tumor_G2
6549	SCO1	3.328676801	Tumor_G2
6550	SURF1	5.956950607	Tumor_G2
6551	ATP6V1E1	6.677428284	Tumor_G2
6552	DLAT	4.848596547	Tumor_G2
6553	PDC	0.436504484	Tumor_G2
6554	SUCLG2	5.874862353	Tumor_G2
6555	SLC25A5	8.144283922	Tumor_G2
6556	UCP2	6.772623721	Tumor_G2
6557	SOD2	4.749740961	Tumor_G2
6558	NOX4	3.132656446	Tumor_G2

6559	DUOX1	6.959596201	Tumor_G2
6560	DUOXA1	6.890623153	Tumor_G2
6561	DUOX2	7.253055549	Tumor_G2
6562	DUOXA2	5.223149015	Tumor_G2
6563	NDUFA1	7.921765562	Tumor_G2
6564	NDUFA2	6.353541626	Tumor_G2
6565	NDUFA8	7.016572504	Tumor_G2
6566	NDUFB1	5.535599618	Tumor_G2
6567	NDUFB10	7.3686555	Tumor_G2
6568	NDUFB2	5.786948473	Tumor_G2
6569	NDUFB3	6.022085823	Tumor_G2
6570	NDUFB7	8.231908554	Tumor_G2
6571	CYC1	6.582805509	Tumor_G2
6572	ETFB	4.85803416	Tumor_G2
6573	UQCR10	6.720999029	Tumor_G2
6574	UQCRQ	6.873770102	Tumor_G2
6575	UQCC3	4.31402057	Tumor_G2
6576	COX15	4.794525503	Tumor_G2
6577	COX5A	6.646774964	Tumor_G2
6578	COX6B1	8.348001876	Tumor_G2
6579	COX6C	6.154489113	Tumor_G2
6580	COX7A2	6.487269793	Tumor_G2
6581	COX7B	5.770629786	Tumor_G2
6582	COX8A	9.12397858	Tumor_G2
6583	SCO1	3.270295016	Tumor_G2
6584	SURF1	6.45610489	Tumor_G2
6585	ATP6V1E1	5.951006425	Tumor_G2
6586	DLAT	4.892055646	Tumor_G2
6587	PDC	0.062271429	Tumor_G2
6588	SUCLG2	5.80857086	Tumor_G2
6589	SLC25A5	7.466337665	Tumor_G2
6590	UCP2	8.54342323	Tumor_G2
6591	SOD2	4.545862203	Tumor_G2
6592	NOX4	1.770801884	Tumor_G2
6593	DUOX1	7.099169808	Tumor_G2
6594	DUOXA1	7.289301634	Tumor_G2
6595	DUOX2	8.521192469	Tumor_G2
6596	DUOXA2	5.891035246	Tumor_G2
6597	NDUFA1	8.281263649	Tumor_G2
6598	NDUFA2	6.804311921	Tumor_G2

6599	NDUFA8	7.183964015	Tumor_G2
6600	NDUFB1	6.166517507	Tumor_G2
6601	NDUFB10	7.716341553	Tumor_G2
6602	NDUFB2	6.816849453	Tumor_G2
6603	NDUFB3	6.590415625	Tumor_G2
6604	NDUFB7	8.896329741	Tumor_G2
6605	CYC1	6.999227371	Tumor_G2
6606	ETFB	5.008584748	Tumor_G2
6607	UQCR10	7.705732195	Tumor_G2
6608	UQCRQ	7.520795598	Tumor_G2
6609	UQCC3	4.454898296	Tumor_G2
6610	COX15	4.843130526	Tumor_G2
6611	COX5A	6.815413543	Tumor_G2
6612	COX6B1	8.776259623	Tumor_G2
6613	COX6C	6.792845639	Tumor_G2
6614	COX7A2	6.973489485	Tumor_G2
6615	COX7B	6.330741251	Tumor_G2
6616	COX8A	9.417367732	Tumor_G2
6617	SCO1	3.415327461	Tumor_G2
6618	SURF1	6.494455894	Tumor_G2
6619	ATP6V1E1	6.711696743	Tumor_G2
6620	DLAT	4.706856162	Tumor_G2
6621	PDC	0.315528835	Tumor_G2
6622	SUCLG2	6.124172568	Tumor_G2
6623	SLC25A5	7.737721621	Tumor_G2
6624	UCP2	7.696248121	Tumor_G2
6625	SOD2	4.674924257	Tumor_G2
6626	NOX4	2.143643321	Tumor_G2
6627	DUOX1	7.397855714	Tumor_G2
6628	DUOXA1	7.37623261	Tumor_G2
6629	DUOX2	8.550680034	Tumor_G2
6630	DUOXA2	5.93889724	Tumor_G2
6631	NDUFA1	8.390826973	Tumor_G2
6632	NDUFA2	7.230361152	Tumor_G2
6633	NDUFA8	7.272438744	Tumor_G2
6634	NDUFB1	5.925178272	Tumor_G2
6635	NDUFB10	8.032970366	Tumor_G2
6636	NDUFB2	6.665730132	Tumor_G2
6637	NDUFB3	6.415666105	Tumor_G2
6638	NDUFB7	8.881582548	Tumor_G2

6639	CYC1	7.778036671	Tumor_G2
6640	ETFB	5.923405529	Tumor_G2
6641	UQCR10	8.120101406	Tumor_G2
6642	UQCRQ	7.513112149	Tumor_G2
6643	UQCC3	4.705555884	Tumor_G2
6644	COX15	5.442811755	Tumor_G2
6645	COX5A	7.777791351	Tumor_G2
6646	COX6B1	8.722093847	Tumor_G2
6647	COX6C	7.111538785	Tumor_G2
6648	COX7A2	7.176118553	Tumor_G2
6649	COX7B	6.467701623	Tumor_G2
6650	COX8A	10.10207167	Tumor_G2
6651	SCO1	3.833996711	Tumor_G2
6652	SURF1	6.726222702	Tumor_G2
6653	ATP6V1E1	6.811945498	Tumor_G2
6654	DLAT	5.743271929	Tumor_G2
6655	PDC	0.385588935	Tumor_G2
6656	SUCLG2	6.560774519	Tumor_G2
6657	SLC25A5	8.33990131	Tumor_G2
6658	UCP2	8.481121106	Tumor_G2
6659	SOD2	4.823844435	Tumor_G2
6660	NOX4	1.707420227	Tumor_G2
6661	DUOX1	7.896649234	Tumor_G2
6662	DUOXA1	6.543269429	Tumor_G2
6663	DUOX2	9.384251444	Tumor_G2
6664	DUOXA2	8.466598915	Tumor_G2
6665	NDUFA1	8.30057349	Tumor_G2
6666	NDUFA2	7.033833992	Tumor_G2
6667	NDUFA8	7.347996397	Tumor_G2
6668	NDUFB1	5.817141953	Tumor_G2
6669	NDUFB10	7.798599582	Tumor_G2
6670	NDUFB2	6.842288365	Tumor_G2
6671	NDUFB3	6.569824063	Tumor_G2
6672	NDUFB7	8.817298208	Tumor_G2
6673	CYC1	6.941888423	Tumor_G2
6674	ETFB	5.035737508	Tumor_G2
6675	UQCR10	8.08931231	Tumor_G2
6676	UQCRQ	7.6126464	Tumor_G2
6677	UQCC3	4.531503173	Tumor_G2
6678	COX15	4.873670636	Tumor_G2

6679	COX5A	7.414587952	Tumor_G2
6680	COX6B1	9.031363694	Tumor_G2
6681	COX6C	6.731299787	Tumor_G2
6682	COX7A2	7.357597944	Tumor_G2
6683	COX7B	6.378831782	Tumor_G2
6684	COX8A	9.524001566	Tumor_G2
6685	SCO1	3.543699771	Tumor_G2
6686	SURF1	6.757695088	Tumor_G2
6687	ATP6V1E1	7.591672281	Tumor_G2
6688	DLAT	5.341544137	Tumor_G2
6689	PDC	0	Tumor_G2
6690	SUCLG2	6.826108971	Tumor_G2
6691	SLC25A5	8.445407805	Tumor_G2
6692	UCP2	8.671042178	Tumor_G2
6693	SOD2	5.473919038	Tumor_G2
6694	NOX4	3.409517343	Tumor_G2
6695	DUOX1	5.860263684	Tumor_G2
6696	DUOXA1	6.326124924	Tumor_G2
6697	DUOX2	6.511752773	Tumor_G2
6698	DUOXA2	4.527061072	Tumor_G2
6699	NDUFA1	8.391224615	Tumor_G2
6700	NDUFA2	6.950560073	Tumor_G2
6701	NDUFA8	7.262089165	Tumor_G2
6702	NDUFB1	6.074275822	Tumor_G2
6703	NDUFB10	7.803600488	Tumor_G2
6704	NDUFB2	6.608813122	Tumor_G2
6705	NDUFB3	6.350685088	Tumor_G2
6706	NDUFB7	9.019967487	Tumor_G2
6707	CYC1	6.880598267	Tumor_G2
6708	ETFB	5.049477178	Tumor_G2
6709	UQCR10	7.821383354	Tumor_G2
6710	UQCRQ	7.634261912	Tumor_G2
6711	UQCC3	4.520290309	Tumor_G2
6712	COX15	4.865575041	Tumor_G2
6713	COX5A	6.933116507	Tumor_G2
6714	COX6B1	8.878601138	Tumor_G2
6715	COX6C	6.74101338	Tumor_G2
6716	COX7A2	6.94420012	Tumor_G2
6717	COX7B	6.317784581	Tumor_G2
6718	COX8A	9.507964987	Tumor_G2

6719	SCO1	3.484321769	Tumor_G2
6720	SURF1	6.61276817	Tumor_G2
6721	ATP6V1E1	6.872230242	Tumor_G2
6722	DLAT	4.830047902	Tumor_G2
6723	PDC	0.14678749	Tumor_G2
6724	SUCLG2	6.427839505	Tumor_G2
6725	SLC25A5	7.758513592	Tumor_G2
6726	UCP2	7.69540161	Tumor_G2
6727	SOD2	4.768355512	Tumor_G2
6728	NOX4	2.65916389	Tumor_G2
6729	DUOX1	7.456417374	Tumor_G2
6730	DUOXA1	7.32953976	Tumor_G2
6731	DUOX2	8.405251662	Tumor_G2
6732	DUOXA2	5.968029083	Tumor_G2
6733	NDUFA1	8.208396709	Tumor_G2
6734	NDUFA2	7.170505558	Tumor_G2
6735	NDUFA8	7.150424543	Tumor_G2
6736	NDUFB1	5.863254916	Tumor_G2
6737	NDUFB10	7.831819879	Tumor_G2
6738	NDUFB2	6.849819585	Tumor_G2
6739	NDUFB3	6.212917122	Tumor_G2
6740	NDUFB7	9.144579772	Tumor_G2
6741	CYC1	6.870225382	Tumor_G2
6742	ETFB	5.291363368	Tumor_G2
6743	UQCR10	7.815178748	Tumor_G2
6744	UQCRQ	7.453751692	Tumor_G2
6745	UQCC3	4.86118152	Tumor_G2
6746	COX15	4.582542285	Tumor_G2
6747	COX5A	7.160665927	Tumor_G2
6748	COX6B1	8.986317548	Tumor_G2
6749	COX6C	6.689374581	Tumor_G2
6750	COX7A2	6.866448942	Tumor_G2
6751	COX7B	6.12471314	Tumor_G2
6752	COX8A	9.69251156	Tumor_G2
6753	SCO1	3.121212959	Tumor_G2
6754	SURF1	6.35331576	Tumor_G2
6755	ATP6V1E1	6.998206457	Tumor_G2
6756	DLAT	4.56213517	Tumor_G2
6757	PDC	0.056694399	Tumor_G2
6758	SUCLG2	5.898279589	Tumor_G2

6759	SLC25A5	7.939304667	Tumor_G2
6760	UCP2	7.979422569	Tumor_G2
6761	SOD2	4.882224173	Tumor_G2
6762	NOX4	2.346216974	Tumor_G2
6763	DUOX1	5.862604406	Tumor_G2
6764	DUOXA1	6.043362386	Tumor_G2
6765	DUOX2	5.971464111	Tumor_G2
6766	DUOXA2	5.572773619	Tumor_G2
6767	NDUFA1	8.37175731	Tumor_G2
6768	NDUFA2	6.826975413	Tumor_G2
6769	NDUFA8	7.243018193	Tumor_G2
6770	NDUFB1	5.912173991	Tumor_G2
6771	NDUFB10	7.499089715	Tumor_G2
6772	NDUFB2	6.568819172	Tumor_G2
6773	NDUFB3	6.508388979	Tumor_G2
6774	NDUFB7	8.966529373	Tumor_G2
6775	CYC1	6.931933481	Tumor_G2
6776	ETFB	5.701516016	Tumor_G2
6777	UQCR10	7.887185441	Tumor_G2
6778	UQCRQ	7.348026049	Tumor_G2
6779	UQCC3	4.854574565	Tumor_G2
6780	COX15	5.034174329	Tumor_G2
6781	COX5A	6.988534384	Tumor_G2
6782	COX6B1	8.64768517	Tumor_G2
6783	COX6C	6.61222913	Tumor_G2
6784	COX7A2	6.862569273	Tumor_G2
6785	COX7B	6.283603466	Tumor_G2
6786	COX8A	9.591656412	Tumor_G2
6787	SCO1	3.389008529	Tumor_G2
6788	SURF1	6.550083273	Tumor_G2
6789	ATP6V1E1	6.992517753	Tumor_G2
6790	DLAT	5.418364653	Tumor_G2
6791	PDC	0.058253609	Tumor_G2
6792	SUCLG2	6.007884368	Tumor_G2
6793	SLC25A5	7.578103162	Tumor_G2
6794	UCP2	9.377251304	Tumor_G2
6795	SOD2	4.627938752	Tumor_G2
6796	NOX4	2.736762651	Tumor_G2
6797	DUOX1	8.150641202	Tumor_G2
6798	DUOXA1	7.990625758	Tumor_G2

6799	DUOX2	8.871059575	Tumor_G2
6800	DUOXA2	6.866702691	Tumor_G2
6801	NDUFA1	7.548637158	Tumor_G2
6802	NDUFA2	6.745544106	Tumor_G2
6803	NDUFA8	7.364592358	Tumor_G2
6804	NDUFB1	5.678336387	Tumor_G2
6805	NDUFB10	7.538721063	Tumor_G2
6806	NDUFB2	6.927807555	Tumor_G2
6807	NDUFB3	6.217491116	Tumor_G2
6808	NDUFB7	8.574907838	Tumor_G2
6809	CYC1	7.1466125	Tumor_G2
6810	ETFB	5.230086204	Tumor_G2
6811	UQCR10	7.635494049	Tumor_G2
6812	UQCRQ	7.565416008	Tumor_G2
6813	UQCC3	4.443211908	Tumor_G2
6814	COX15	4.834844554	Tumor_G2
6815	COX5A	7.374453983	Tumor_G2
6816	COX6B1	8.728589247	Tumor_G2
6817	COX6C	6.534522197	Tumor_G2
6818	COX7A2	7.023738345	Tumor_G2
6819	COX7B	6.361074948	Tumor_G2
6820	COX8A	10.27177545	Tumor_G2
6821	SCO1	3.307217455	Tumor_G2
6822	SURF1	5.795143329	Tumor_G2
6823	ATP6V1E1	7.186166804	Tumor_G2
6824	DLAT	5.145186311	Tumor_G2
6825	PDC	0.186588095	Tumor_G2
6826	SUCLG2	6.672494289	Tumor_G2
6827	SLC25A5	8.620764913	Tumor_G2
6828	UCP2	6.966088665	Tumor_G2
6829	SOD2	4.344692938	Tumor_G2
6830	NOX4	2.179537544	Tumor_G2
6831	DUOX1	7.620847331	Tumor_G2
6832	DUOXA1	7.19675979	Tumor_G2
6833	DUOX2	8.165402407	Tumor_G2
6834	DUOXA2	6.004022914	Tumor_G2
6835	NDUFA1	7.870854258	Tumor_G2
6836	NDUFA2	6.589495423	Tumor_G2
6837	NDUFA8	7.098686381	Tumor_G2
6838	NDUFB1	5.447250206	Tumor_G2

6839	NDUFB10	7.475698989	Tumor_G2
6840	NDUFB2	6.457853277	Tumor_G2
6841	NDUFB3	6.272930131	Tumor_G2
6842	NDUFB7	8.565038676	Tumor_G2
6843	CYC1	6.964331782	Tumor_G2
6844	ETFB	5.300567323	Tumor_G2
6845	UQCR10	6.814315907	Tumor_G2
6846	UQCRQ	7.080974726	Tumor_G2
6847	UQCC3	4.423717475	Tumor_G2
6848	COX15	5.273272406	Tumor_G2
6849	COX5A	6.892749636	Tumor_G2
6850	COX6B1	8.393971287	Tumor_G2
6851	COX6C	6.431883247	Tumor_G2
6852	COX7A2	6.767011542	Tumor_G2
6853	COX7B	6.088509333	Tumor_G2
6854	COX8A	9.573014642	Tumor_G2
6855	SCO1	3.712179511	Tumor_G2
6856	SURF1	6.437033655	Tumor_G2
6857	ATP6V1E1	6.375533626	Tumor_G2
6858	DLAT	5.622524191	Tumor_G2
6859	PDC	0	Tumor_G2
6860	SUCLG2	6.579357008	Tumor_G2
6861	SLC25A5	8.149976552	Tumor_G2
6862	UCP2	8.728047224	Tumor_G2
6863	SOD2	4.90551264	Tumor_G2
6864	NOX4	1.952840255	Tumor_G2
6865	DUOX1	6.979790571	Tumor_G2
6866	DUOXA1	7.105336081	Tumor_G2
6867	DUOX2	8.562911676	Tumor_G2
6868	DUOXA2	5.31493098	Tumor_G2
6869	NDUFA1	7.658265316	Tumor_G2
6870	NDUFA2	6.602191911	Tumor_G2
6871	NDUFA8	7.171857886	Tumor_G2
6872	NDUFB1	5.216195689	Tumor_G2
6873	NDUFB10	7.591949619	Tumor_G2
6874	NDUFB2	6.172273317	Tumor_G2
6875	NDUFB3	5.846476251	Tumor_G2
6876	NDUFB7	8.662864261	Tumor_G2
6877	CYC1	6.743149424	Tumor_G2
6878	ETFB	5.226272086	Tumor_G2

6879	UQCR10	7.484332283	Tumor_G2
6880	UQCRQ	6.951450692	Tumor_G2
6881	UQCC3	4.758408991	Tumor_G2
6882	COX15	4.833348992	Tumor_G2
6883	COX5A	6.915760394	Tumor_G2
6884	COX6B1	8.580603861	Tumor_G2
6885	COX6C	6.235920367	Tumor_G2
6886	COX7A2	6.571876939	Tumor_G2
6887	COX7B	5.898308897	Tumor_G2
6888	COX8A	9.334740281	Tumor_G2
6889	SCO1	3.201347289	Tumor_G2
6890	SURF1	6.795805309	Tumor_G2
6891	ATP6V1E1	6.906806523	Tumor_G2
6892	DLAT	4.955672082	Tumor_G2
6893	PDC	0.075236294	Tumor_G2
6894	SUCLG2	6.090808383	Tumor_G2
6895	SLC25A5	7.196769435	Tumor_G2
6896	UCP2	9.441846296	Tumor_G2
6897	SOD2	4.731055046	Tumor_G2
6898	NOX4	2.927598494	Tumor_G2
6899	DUOX1	6.100162543	Tumor_G2
6900	DUOXA1	6.60609387	Tumor_G2
6901	DUOX2	7.482236262	Tumor_G2
6902	DUOXA2	5.880217306	Tumor_G2
6903	NDUFA1	7.807936967	Tumor_G2
6904	NDUFA2	6.488876842	Tumor_G2
6905	NDUFA8	6.924460788	Tumor_G2
6906	NDUFB1	5.387421803	Tumor_G2
6907	NDUFB10	7.389637587	Tumor_G2
6908	NDUFB2	6.802666525	Tumor_G2
6909	NDUFB3	6.267115817	Tumor_G2
6910	NDUFB7	8.650647248	Tumor_G2
6911	CYC1	7.057584928	Tumor_G2
6912	ETFB	5.258522728	Tumor_G2
6913	UQCR10	7.645582636	Tumor_G2
6914	UQCRQ	7.002503266	Tumor_G2
6915	UQCC3	4.378075695	Tumor_G2
6916	COX15	5.24915378	Tumor_G2
6917	COX5A	6.871794767	Tumor_G2
6918	COX6B1	8.467789544	Tumor_G2

6919	COX6C	6.349253742	Tumor_G2
6920	COX7A2	6.623402964	Tumor_G2
6921	COX7B	5.800061628	Tumor_G2
6922	COX8A	9.734131614	Tumor_G2
6923	SCO1	3.478348563	Tumor_G2
6924	SURF1	6.76799265	Tumor_G2
6925	ATP6V1E1	7.058775257	Tumor_G2
6926	DLAT	5.381874965	Tumor_G2
6927	PDC	0.063482519	Tumor_G2
6928	SUCLG2	6.348879046	Tumor_G2
6929	SLC25A5	8.021316146	Tumor_G2
6930	UCP2	9.119069557	Tumor_G2
6931	SOD2	4.961496253	Tumor_G2
6932	NOX4	1.282593787	Tumor_G2
6933	DUOX1	7.705023527	Tumor_G2
6934	DUOXA1	7.226106529	Tumor_G2
6935	DUOX2	7.89464583	Tumor_G2
6936	DUOXA2	5.522806668	Tumor_G2
6937	NDUFA1	8.109186322	Tumor_G2
6938	NDUFA2	6.859409197	Tumor_G2
6939	NDUFA8	7.131207136	Tumor_G2
6940	NDUFB1	5.537753157	Tumor_G2
6941	NDUFB10	7.771404405	Tumor_G2
6942	NDUFB2	6.339612905	Tumor_G2
6943	NDUFB3	6.371822013	Tumor_G2
6944	NDUFB7	8.515232747	Tumor_G2
6945	CYC1	6.892125556	Tumor_G2
6946	ETFB	4.844219634	Tumor_G2
6947	UQCR10	7.618078622	Tumor_G2
6948	UQCRQ	7.403039457	Tumor_G2
6949	UQCC3	4.000284291	Tumor_G2
6950	COX15	5.196390061	Tumor_G2
6951	COX5A	7.254741909	Tumor_G2
6952	COX6B1	8.763513176	Tumor_G2
6953	COX6C	6.405058252	Tumor_G2
6954	COX7A2	6.901396383	Tumor_G2
6955	COX7B	6.084247134	Tumor_G2
6956	COX8A	9.426418927	Tumor_G2
6957	SCO1	3.662545516	Tumor_G2
6958	SURF1	6.333795859	Tumor_G2

6959	ATP6V1E1	7.636382218	Tumor_G2
6960	DLAT	5.616697051	Tumor_G2
6961	PDC	0.152294473	Tumor_G2
6962	SUCLG2	6.918700066	Tumor_G2
6963	SLC25A5	8.830855863	Tumor_G2
6964	UCP2	8.149380865	Tumor_G2
6965	SOD2	5.672249208	Tumor_G2
6966	NOX4	2.40932288	Tumor_G2
6967	DUOX1	6.32391326	Tumor_G2
6968	DUOXA1	6.539993306	Tumor_G2
6969	DUOX2	6.263364905	Tumor_G2
6970	DUOXA2	3.227500866	Tumor_G2
6971	NDUFA1	7.812987259	Tumor_G2
6972	NDUFA2	6.724345674	Tumor_G2
6973	NDUFA8	7.236703218	Tumor_G2
6974	NDUFB1	5.754462475	Tumor_G2
6975	NDUFB10	7.532766306	Tumor_G2
6976	NDUFB2	6.582653061	Tumor_G2
6977	NDUFB3	6.501136435	Tumor_G2
6978	NDUFB7	8.624318128	Tumor_G2
6979	CYC1	6.640970239	Tumor_G2
6980	ETFB	5.150813044	Tumor_G2
6981	UQCR10	7.682457916	Tumor_G2
6982	UQCRQ	7.354235482	Tumor_G2
6983	UQCC3	4.404112648	Tumor_G2
6984	COX15	4.903193391	Tumor_G2
6985	COX5A	7.289407991	Tumor_G2
6986	COX6B1	8.582805744	Tumor_G2
6987	COX6C	6.472225271	Tumor_G2
6988	COX7A2	6.742785038	Tumor_G2
6989	COX7B	6.064590228	Tumor_G2
6990	COX8A	9.433532772	Tumor_G2
6991	SCO1	3.826828951	Tumor_G2
6992	SURF1	6.395150996	Tumor_G2
6993	ATP6V1E1	6.916504117	Tumor_G2
6994	DLAT	5.369504805	Tumor_G2
6995	PDC	0.294378572	Tumor_G2
6996	SUCLG2	6.313411813	Tumor_G2
6997	SLC25A5	7.814589343	Tumor_G2
6998	UCP2	9.340449655	Tumor_G2

6999	SOD2	4.878750698	Tumor_G2
7000	NOX4	3.06496302	Tumor_G2
7001	DUOX1	6.860358717	Tumor_G2
7002	DUOXA1	7.212394128	Tumor_G2
7003	DUOX2	8.1289439	Tumor_G2
7004	DUOXA2	5.87234288	Tumor_G2
7005	NDUFA1	8.094180831	Tumor_G2
7006	NDUFA2	6.643280227	Tumor_G2
7007	NDUFA8	7.198952542	Tumor_G2
7008	NDUFB1	5.496752039	Tumor_G2
7009	NDUFB10	7.566689542	Tumor_G2
7010	NDUFB2	6.832655515	Tumor_G2
7011	NDUFB3	6.422121448	Tumor_G2
7012	NDUFB7	8.715606304	Tumor_G2
7013	CYC1	7.200593912	Tumor_G2
7014	ETFB	5.028003987	Tumor_G2
7015	UQCR10	7.73732362	Tumor_G2
7016	UQCRQ	7.187748772	Tumor_G2
7017	UQCC3	4.109433553	Tumor_G2
7018	COX15	5.166256371	Tumor_G2
7019	COX5A	7.141161729	Tumor_G2
7020	COX6B1	8.664436295	Tumor_G2
7021	COX6C	6.537586358	Tumor_G2
7022	COX7A2	6.705072879	Tumor_G2
7023	COX7B	6.194085532	Tumor_G2
7024	COX8A	9.892132479	Tumor_G2
7025	SCO1	3.805593508	Tumor_G2
7026	SURF1	6.36595473	Tumor_G2
7027	ATP6V1E1	6.894804403	Tumor_G2
7028	DLAT	5.425853518	Tumor_G2
7029	PDC	0.130743805	Tumor_G2
7030	SUCLG2	6.182000247	Tumor_G2
7031	SLC25A5	8.233257837	Tumor_G2
7032	UCP2	8.123062054	Tumor_G2
7033	SOD2	5.332181726	Tumor_G2
7034	NOX4	2.245950332	Tumor_G2
7035	DUOX1	6.646933356	Tumor_G2
7036	DUOXA1	6.540282013	Tumor_G2
7037	DUOX2	7.073893684	Tumor_G2
7038	DUOXA2	5.460563835	Tumor_G2

7039	NDUFA1	8.218567695	Tumor_G2
7040	NDUFA2	6.671859638	Tumor_G2
7041	NDUFA8	7.400763459	Tumor_G2
7042	NDUFB1	5.819478651	Tumor_G2
7043	NDUFB10	7.842344547	Tumor_G2
7044	NDUFB2	6.520132575	Tumor_G2
7045	NDUFB3	6.462068838	Tumor_G2
7046	NDUFB7	8.806126961	Tumor_G2
7047	CYC1	7.015919858	Tumor_G2
7048	ETFB	5.840964575	Tumor_G2
7049	UQCR10	7.277602858	Tumor_G2
7050	UQCRQ	7.352572059	Tumor_G2
7051	UQCC3	4.913598647	Tumor_G2
7052	COX15	5.28699384	Tumor_G2
7053	COX5A	6.993628465	Tumor_G2
7054	COX6B1	8.762718998	Tumor_G2
7055	COX6C	6.34061336	Tumor_G2
7056	COX7A2	6.673476882	Tumor_G2
7057	COX7B	6.0026128	Tumor_G2
7058	COX8A	9.61053008	Tumor_G2
7059	SCO1	3.54635427	Tumor_G2
7060	SURF1	6.892703844	Tumor_G2
7061	ATP6V1E1	6.864915583	Tumor_G2
7062	DLAT	5.047107132	Tumor_G2
7063	PDC	0.118552018	Tumor_G2
7064	SUCLG2	6.046753979	Tumor_G2
7065	SLC25A5	7.702431029	Tumor_G2
7066	UCP2	9.142838156	Tumor_G2
7067	SOD2	4.854148278	Tumor_G2
7068	NOX4	2.513309476	Tumor_G2
7069	DUOX1	7.173057347	Tumor_G2
7070	DUOXA1	7.300899899	Tumor_G2
7071	DUOX2	8.088716554	Tumor_G2
7072	DUOXA2	5.6546819	Tumor_G2
7073	NDUFA1	7.803357863	Tumor_G2
7074	NDUFA2	6.535140909	Tumor_G2
7075	NDUFA8	6.970381904	Tumor_G2
7076	NDUFB1	5.404338324	Tumor_G2
7077	NDUFB10	7.499895603	Tumor_G2
7078	NDUFB2	6.717591758	Tumor_G2

7079	NDUFB3	6.240906174	Tumor_G2
7080	NDUFB7	8.073152798	Tumor_G2
7081	CYC1	6.875569458	Tumor_G2
7082	ETFB	4.920919053	Tumor_G2
7083	UQCR10	7.398293049	Tumor_G2
7084	UQCRQ	6.898114098	Tumor_G2
7085	UQCC3	4.256862325	Tumor_G2
7086	COX15	4.963817211	Tumor_G2
7087	COX5A	7.083694556	Tumor_G2
7088	COX6B1	8.622200257	Tumor_G2
7089	COX6C	6.176373408	Tumor_G2
7090	COX7A2	6.752112842	Tumor_G2
7091	COX7B	6.09734356	Tumor_G2
7092	COX8A	9.376670438	Tumor_G2
7093	SCO1	3.505727827	Tumor_G2
7094	SURF1	6.382395159	Tumor_G2
7095	ATP6V1E1	6.985730601	Tumor_G2
7096	DLAT	5.285244074	Tumor_G2
7097	PDC	0.157299638	Tumor_G2
7098	SUCLG2	6.381106781	Tumor_G2
7099	SLC25A5	8.682620093	Tumor_G2
7100	UCP2	8.488710922	Tumor_G2
7101	SOD2	5.197727703	Tumor_G2
7102	NOX4	1.91967578	Tumor_G2
7103	DUOX1	5.937314576	Tumor_G2
7104	DUOXA1	6.243798392	Tumor_G2
7105	DUOX2	6.712437339	Tumor_G2
7106	DUOXA2	4.761874569	Tumor_G2
7107	NDUFA1	8.077416402	Tumor_G2
7108	NDUFA2	6.689128173	Tumor_G2
7109	NDUFA8	7.628056192	Tumor_G2
7110	NDUFB1	6.379947271	Tumor_G2
7111	NDUFB10	7.53841308	Tumor_G2
7112	NDUFB2	7.334914347	Tumor_G2
7113	NDUFB3	6.576308995	Tumor_G2
7114	NDUFB7	8.555776394	Tumor_G2
7115	CYC1	7.557973874	Tumor_G2
7116	ETFB	5.221088249	Tumor_G2
7117	UQCR10	7.763452443	Tumor_G2
7118	UQCRQ	7.307084722	Tumor_G2

7119	UQCC3	4.415600197	Tumor_G2
7120	COX15	5.831277923	Tumor_G2
7121	COX5A	7.655597356	Tumor_G2
7122	COX6B1	8.894241545	Tumor_G2
7123	COX6C	6.851452623	Tumor_G2
7124	COX7A2	7.266788939	Tumor_G2
7125	COX7B	6.939442554	Tumor_G2
7126	COX8A	9.97920003	Tumor_G2
7127	SCO1	4.094844934	Tumor_G2
7128	SURF1	6.535843902	Tumor_G2
7129	ATP6V1E1	7.497878038	Tumor_G2
7130	DLAT	6.018934424	Tumor_G2
7131	PDC	0.106933777	Tumor_G2
7132	SUCLG2	7.163320773	Tumor_G2
7133	SLC25A5	9.202617692	Tumor_G2
7134	UCP2	6.41213897	Tumor_G2
7135	SOD2	5.091491955	Tumor_G2
7136	NOX4	1.557497162	Tumor_G2
7137	DUOX1	7.086350798	Tumor_G2
7138	DUOXA1	6.245835471	Tumor_G2
7139	DUOX2	8.195896932	Tumor_G2
7140	DUOXA2	5.81508561	Tumor_G2
7141	NDUFA1	8.279365878	Tumor_G2
7142	NDUFA2	6.845752697	Tumor_G2
7143	NDUFA8	7.228418633	Tumor_G2
7144	NDUFB1	5.880177333	Tumor_G2
7145	NDUFB10	7.731399211	Tumor_G2
7146	NDUFB2	6.874920605	Tumor_G2
7147	NDUFB3	6.567341142	Tumor_G2
7148	NDUFB7	8.932695862	Tumor_G2
7149	CYC1	7.013736617	Tumor_G2
7150	ETFB	5.466714766	Tumor_G2
7151	UQCR10	8.100089931	Tumor_G2
7152	UQCRQ	7.599797537	Tumor_G2
7153	UQCC3	4.700616908	Tumor_G2
7154	COX15	5.300627696	Tumor_G2
7155	COX5A	7.239444973	Tumor_G2
7156	COX6B1	8.814604292	Tumor_G2
7157	COX6C	6.900978683	Tumor_G2
7158	COX7A2	7.01541809	Tumor_G2

7159	COX7B	6.343457355	Tumor_G2
7160	COX8A	9.56931978	Tumor_G2
7161	SCO1	3.267535779	Tumor_G2
7162	SURF1	6.773402744	Tumor_G2
7163	ATP6V1E1	7.323004236	Tumor_G2
7164	DLAT	5.248909613	Tumor_G2
7165	PDC	0.104007132	Tumor_G2
7166	SUCLG2	6.5899791	Tumor_G2
7167	SLC25A5	7.536915147	Tumor_G2
7168	UCP2	9.441178729	Tumor_G2
7169	SOD2	4.784347488	Tumor_G2
7170	NOX4	2.476318723	Tumor_G2
7171	DUOX1	6.773063228	Tumor_G2
7172	DUOXA1	7.078440318	Tumor_G2
7173	DUOX2	7.820229502	Tumor_G2
7174	DUOXA2	5.152479785	Tumor_G2
7175	NDUFA1	8.079054241	Tumor_G2
7176	NDUFA2	6.694497666	Tumor_G2
7177	NDUFA8	7.2707736	Tumor_G2
7178	NDUFB1	5.918534071	Tumor_G2
7179	NDUFB10	7.56034117	Tumor_G2
7180	NDUFB2	6.598738089	Tumor_G2
7181	NDUFB3	6.486697201	Tumor_G2
7182	NDUFB7	8.620372666	Tumor_G2
7183	CYC1	6.842894717	Tumor_G2
7184	ETFB	5.213615438	Tumor_G2
7185	UQCR10	7.788316543	Tumor_G2
7186	UQCRQ	7.275971806	Tumor_G2
7187	UQCC3	4.668575803	Tumor_G2
7188	COX15	5.340047952	Tumor_G2
7189	COX5A	7.082752317	Tumor_G2
7190	COX6B1	8.722035931	Tumor_G2
7191	COX6C	6.652601737	Tumor_G2
7192	COX7A2	6.913618953	Tumor_G2
7193	COX7B	6.087422068	Tumor_G2
7194	COX8A	9.538721355	Tumor_G2
7195	SCO1	3.744970384	Tumor_G2
7196	SURF1	6.597645023	Tumor_G2
7197	ATP6V1E1	6.927986738	Tumor_G2
7198	DLAT	5.413247231	Tumor_G2

7199	PDC	0.074731958	Tumor_G2
7200	SUCLG2	6.584766861	Tumor_G2
7201	SLC25A5	7.809859439	Tumor_G2
7202	UCP2	8.336507092	Tumor_G2
7203	SOD2	5.111499919	Tumor_G2
7204	NOX4	2.943251738	Tumor_G2
7205	DUOX1	6.521454468	Tumor_G2
7206	DUOXA1	6.729137538	Tumor_G2
7207	DUOX2	6.943945444	Tumor_G2
7208	DUOXA2	4.505465324	Tumor_G2
7209	NDUFA1	7.729183574	Tumor_G2
7210	NDUFA2	6.481480733	Tumor_G2
7211	NDUFA8	6.981796546	Tumor_G2
7212	NDUFB1	5.206323206	Tumor_G2
7213	NDUFB10	7.447375319	Tumor_G2
7214	NDUFB2	6.33696067	Tumor_G2
7215	NDUFB3	6.037367265	Tumor_G2
7216	NDUFB7	8.781362985	Tumor_G2
7217	CYC1	6.898354786	Tumor_G2
7218	ETFB	5.469957984	Tumor_G2
7219	UQCR10	7.645932526	Tumor_G2
7220	UQCRQ	6.980082186	Tumor_G2
7221	UQCC3	4.699314437	Tumor_G2
7222	COX15	5.241423571	Tumor_G2
7223	COX5A	6.791278826	Tumor_G2
7224	COX6B1	8.750671557	Tumor_G2
7225	COX6C	6.095646825	Tumor_G2
7226	COX7A2	6.478076197	Tumor_G2
7227	COX7B	5.673413016	Tumor_G2
7228	COX8A	9.207445217	Tumor_G2
7229	SCO1	3.319021805	Tumor_G2
7230	SURF1	6.678903717	Tumor_G2
7231	ATP6V1E1	6.909909749	Tumor_G2
7232	DLAT	5.216251709	Tumor_G2
7233	PDC	0	Tumor_G2
7234	SUCLG2	6.093630811	Tumor_G2
7235	SLC25A5	7.891325673	Tumor_G2
7236	UCP2	9.182487057	Tumor_G2
7237	SOD2	5.215114007	Tumor_G2
7238	NOX4	2.942244751	Tumor_G2

7239	DUOX1	7.369398158	Tumor_G2
7240	DUOXA1	7.320435097	Tumor_G2
7241	DUOX2	8.203166902	Tumor_G2
7242	DUOXA2	6.159808819	Tumor_G2
7243	NDUFA1	7.585117288	Tumor_G2
7244	NDUFA2	6.64747746	Tumor_G2
7245	NDUFA8	7.552873814	Tumor_G2
7246	NDUFB1	5.314838731	Tumor_G2
7247	NDUFB10	7.420970917	Tumor_G2
7248	NDUFB2	6.124348896	Tumor_G2
7249	NDUFB3	5.87796115	Tumor_G2
7250	NDUFB7	8.532855933	Tumor_G2
7251	CYC1	6.704272087	Tumor_G2
7252	ETFB	5.090296957	Tumor_G2
7253	UQCR10	7.669001773	Tumor_G2
7254	UQCRQ	7.171940189	Tumor_G2
7255	UQCC3	4.353849908	Tumor_G2
7256	COX15	4.717269242	Tumor_G2
7257	COX5A	7.007782039	Tumor_G2
7258	COX6B1	8.982950421	Tumor_G2
7259	COX6C	6.151835744	Tumor_G2
7260	COX7A2	6.724616944	Tumor_G2
7261	COX7B	6.015876976	Tumor_G2
7262	COX8A	9.073985834	Tumor_G2
7263	SCO1	3.222188106	Tumor_G2
7264	SURF1	6.501945334	Tumor_G2
7265	ATP6V1E1	6.969969455	Tumor_G2
7266	DLAT	5.044081271	Tumor_G2
7267	PDC	0	Tumor_G2
7268	SUCLG2	5.878590526	Tumor_G2
7269	SLC25A5	7.820007268	Tumor_G2
7270	UCP2	8.030118274	Tumor_G2
7271	SOD2	4.815521156	Tumor_G2
7272	NOX4	3.328051622	Tumor_G2
7273	DUOX1	6.576811935	Tumor_G2
7274	DUOXA1	6.891483725	Tumor_G2
7275	DUOX2	7.308950078	Tumor_G2
7276	DUOXA2	5.61683991	Tumor_G2
7277	NDUFA1	8.05324961	Tumor_G2
7278	NDUFA2	6.697501288	Tumor_G2

7279	NDUFA8	7.02499543	Tumor_G2
7280	NDUFB1	5.817037655	Tumor_G2
7281	NDUFB10	7.558261854	Tumor_G2
7282	NDUFB2	6.394092671	Tumor_G2
7283	NDUFB3	6.434940978	Tumor_G2
7284	NDUFB7	8.686529429	Tumor_G2
7285	CYC1	6.647191447	Tumor_G2
7286	ETFB	5.119236974	Tumor_G2
7287	UQCR10	7.668483697	Tumor_G2
7288	UQCRQ	7.200735942	Tumor_G2
7289	UQCC3	4.712855562	Tumor_G2
7290	COX15	5.004871285	Tumor_G2
7291	COX5A	6.929960993	Tumor_G2
7292	COX6B1	8.817400072	Tumor_G2
7293	COX6C	6.52741877	Tumor_G2
7294	COX7A2	6.775108962	Tumor_G2
7295	COX7B	6.101163083	Tumor_G2
7296	COX8A	9.474730876	Tumor_G2
7297	SCO1	3.341775194	Tumor_G2
7298	SURF1	6.840747607	Tumor_G2
7299	ATP6V1E1	6.725755466	Tumor_G2
7300	DLAT	4.913065679	Tumor_G2
7301	PDC	0	Tumor_G2
7302	SUCLG2	5.99867576	Tumor_G2
7303	SLC25A5	7.746822246	Tumor_G2
7304	UCP2	8.207803779	Tumor_G2
7305	SOD2	5.239016067	Tumor_G2
7306	NOX4	1.481093105	Tumor_G2
7307	DUOX1	6.907407258	Tumor_G2
7308	DUOXA1	6.7196991	Tumor_G2
7309	DUOX2	8.062193634	Tumor_G2
7310	DUOXA2	6.394887468	Tumor_G2
7311	NDUFA1	9.275993491	Tumor_G2
7312	NDUFA2	7.137957145	Tumor_G2
7313	NDUFA8	7.52633973	Tumor_G2
7314	NDUFB1	6.495295522	Tumor_G2
7315	NDUFB10	8.319806618	Tumor_G2
7316	NDUFB2	7.406052695	Tumor_G2
7317	NDUFB3	6.471524157	Tumor_G2
7318	NDUFB7	8.912091561	Tumor_G2

7319	CYC1	7.926505284	Tumor_G2
7320	ETFB	5.43084105	Tumor_G2
7321	UQCR10	7.749956779	Tumor_G2
7322	UQCRQ	8.016998923	Tumor_G2
7323	UQCC3	4.441702621	Tumor_G2
7324	COX15	5.453586428	Tumor_G2
7325	COX5A	8.442257111	Tumor_G2
7326	COX6B1	9.204539146	Tumor_G2
7327	COX6C	6.890032685	Tumor_G2
7328	COX7A2	8.287627774	Tumor_G2
7329	COX7B	7.864334597	Tumor_G2
7330	COX8A	10.60906879	Tumor_G2
7331	SCO1	3.99669968	Tumor_G2
7332	SURF1	5.268457044	Tumor_G2
7333	ATP6V1E1	6.4054411	Tumor_G2
7334	DLAT	5.836387495	Tumor_G2
7335	PDC	0	Tumor_G2
7336	SUCLG2	6.767752368	Tumor_G2
7337	SLC25A5	10.15379703	Tumor_G2
7338	UCP2	3.809718725	Tumor_G2
7339	SOD2	5.412421971	Tumor_G2
7340	NOX4	1.208010902	Tumor_G2
7341	DUOX1	3.813300537	Tumor_G2
7342	DUOXA1	3.966955542	Tumor_G2
7343	DUOX2	3.869553506	Tumor_G2
7344	DUOXA2	2.471423095	Tumor_G2
7345	NDUFA1	8.706020242	Tumor_G2
7346	NDUFA2	7.167483393	Tumor_G2
7347	NDUFA8	7.348326254	Tumor_G2
7348	NDUFB1	6.027296545	Tumor_G2
7349	NDUFB10	7.900587656	Tumor_G2
7350	NDUFB2	7.75045982	Tumor_G2
7351	NDUFB3	6.942985878	Tumor_G2
7352	NDUFB7	8.933825404	Tumor_G2
7353	CYC1	7.746161866	Tumor_G2
7354	ETFB	5.461276329	Tumor_G2
7355	UQCR10	8.166323664	Tumor_G2
7356	UQCRQ	7.623807068	Tumor_G2
7357	UQCC3	5.130228037	Tumor_G2
7358	COX15	5.804855126	Tumor_G2

7359	COX5A	7.675834501	Tumor_G2
7360	COX6B1	9.0267908	Tumor_G2
7361	COX6C	6.975258965	Tumor_G2
7362	COX7A2	7.426673376	Tumor_G2
7363	COX7B	6.926476006	Tumor_G2
7364	COX8A	10.05774892	Tumor_G2
7365	SCO1	4.401453477	Tumor_G2
7366	SURF1	7.174258767	Tumor_G2
7367	ATP6V1E1	7.252193141	Tumor_G2
7368	DLAT	5.615461155	Tumor_G2
7369	PDC	0.218426846	Tumor_G2
7370	SUCLG2	7.18191081	Tumor_G2
7371	SLC25A5	9.458761843	Tumor_G2
7372	UCP2	6.951401165	Tumor_G2
7373	SOD2	5.76567633	Tumor_G2
7374	NOX4	1.54198866	Tumor_G2
7375	DUOX1	5.975182011	Tumor_G2
7376	DUOXA1	6.734884266	Tumor_G2
7377	DUOX2	5.733327369	Tumor_G2
7378	DUOXA2	5.144283988	Tumor_G2
7379	NDUFA1	7.971578327	Tumor_G2
7380	NDUFA2	6.290718398	Tumor_G2
7381	NDUFA8	6.925322848	Tumor_G2
7382	NDUFB1	5.438398653	Tumor_G2
7383	NDUFB10	7.436964189	Tumor_G2
7384	NDUFB2	5.934575057	Tumor_G2
7385	NDUFB3	6.183947988	Tumor_G2
7386	NDUFB7	8.53885222	Tumor_G2
7387	CYC1	6.590204812	Tumor_G2
7388	ETFB	4.949080248	Tumor_G2
7389	UQCR10	7.469002052	Tumor_G2
7390	UQCRQ	7.057473962	Tumor_G2
7391	UQCC3	4.435929448	Tumor_G2
7392	COX15	4.918242733	Tumor_G2
7393	COX5A	6.926631167	Tumor_G2
7394	COX6B1	8.525976424	Tumor_G2
7395	COX6C	6.245141698	Tumor_G2
7396	COX7A2	6.593200556	Tumor_G2
7397	COX7B	5.97479097	Tumor_G2
7398	COX8A	9.179975229	Tumor_G2

7399	SCO1	3.33565817	Tumor_G2
7400	SURF1	6.178883927	Tumor_G2
7401	ATP6V1E1	6.706955625	Tumor_G2
7402	DLAT	4.83814858	Tumor_G2
7403	PDC	0.096318622	Tumor_G2
7404	SUCLG2	5.758882304	Tumor_G2
7405	SLC25A5	7.765357496	Tumor_G2
7406	UCP2	8.769759542	Tumor_G2
7407	SOD2	5.048003108	Tumor_G2
7408	NOX4	3.006598391	Tumor_G2
7409	DUOX1	6.547461915	Tumor_G2
7410	DUOXA1	6.973507611	Tumor_G2
7411	DUOX2	7.439805332	Tumor_G2
7412	DUOXA2	5.266792834	Tumor_G2
7413	NDUFA1	7.905304241	Tumor_G2
7414	NDUFA2	6.283569549	Tumor_G2
7415	NDUFA8	7.159007912	Tumor_G2
7416	NDUFB1	5.508190662	Tumor_G2
7417	NDUFB10	7.236166027	Tumor_G2
7418	NDUFB2	6.116454985	Tumor_G2
7419	NDUFB3	6.300599333	Tumor_G2
7420	NDUFB7	8.226206342	Tumor_G2
7421	CYC1	6.506977451	Tumor_G2
7422	ETFB	5.005535489	Tumor_G2
7423	UQCR10	7.395712885	Tumor_G2
7424	UQCRQ	6.925681011	Tumor_G2
7425	UQCC3	3.914778526	Tumor_G2
7426	COX15	5.068450525	Tumor_G2
7427	COX5A	6.765144219	Tumor_G2
7428	COX6B1	8.677584296	Tumor_G2
7429	COX6C	6.21663224	Tumor_G2
7430	COX7A2	6.716685353	Tumor_G2
7431	COX7B	6.020777455	Tumor_G2
7432	COX8A	8.66616496	Tumor_G2
7433	SCO1	3.529861637	Tumor_G2
7434	SURF1	6.308269421	Tumor_G2
7435	ATP6V1E1	6.922088472	Tumor_G2
7436	DLAT	5.107943118	Tumor_G2
7437	PDC	0.086018185	Tumor_G2
7438	SUCLG2	6.120868783	Tumor_G2

7439	SLC25A5	7.724412285	Tumor_G2
7440	UCP2	8.750171965	Tumor_G2
7441	SOD2	4.886705523	Tumor_G2
7442	NOX4	2.854902061	Tumor_G2
7443	DUOX1	6.757534534	Tumor_G2
7444	DUOXA1	7.113974983	Tumor_G2
7445	DUOX2	8.030143889	Tumor_G2
7446	DUOXA2	5.126515601	Tumor_G2
7447	NDUFA1	8.366357266	Tumor_G2
7448	NDUFA2	6.898141821	Tumor_G2
7449	NDUFA8	7.508564165	Tumor_G2
7450	NDUFB1	5.877225536	Tumor_G2
7451	NDUFB10	7.748627549	Tumor_G2
7452	NDUFB2	6.643472876	Tumor_G2
7453	NDUFB3	6.59881069	Tumor_G2
7454	NDUFB7	8.928108172	Tumor_G2
7455	CYC1	7.008798844	Tumor_G2
7456	ETFB	5.602763822	Tumor_G2
7457	UQCR10	7.555563534	Tumor_G2
7458	UQCRQ	7.266025269	Tumor_G2
7459	UQCC3	4.85274497	Tumor_G2
7460	COX15	5.148028304	Tumor_G2
7461	COX5A	7.059130076	Tumor_G2
7462	COX6B1	8.861197139	Tumor_G2
7463	COX6C	6.638292539	Tumor_G2
7464	COX7A2	6.828285769	Tumor_G2
7465	COX7B	6.153421258	Tumor_G2
7466	COX8A	9.637410622	Tumor_G2
7467	SCO1	3.525377535	Tumor_G2
7468	SURF1	6.639764919	Tumor_G2
7469	ATP6V1E1	6.658584793	Tumor_G2
7470	DLAT	5.093335362	Tumor_G2
7471	PDC	0.091670136	Tumor_G2
7472	SUCLG2	6.214492854	Tumor_G2
7473	SLC25A5	7.757879189	Tumor_G2
7474	UCP2	8.988378565	Tumor_G2
7475	SOD2	4.723581607	Tumor_G2
7476	NOX4	1.983320601	Tumor_G2
7477	DUOX1	7.318086486	Tumor_G2
7478	DUOXA1	7.256896832	Tumor_G2

7479	DUOX2	8.782773395	Tumor_G2
7480	DUOXA2	6.366566853	Tumor_G2
7481	NDUFA1	8.045569816	Tumor_G2
7482	NDUFA2	6.829798072	Tumor_G2
7483	NDUFA8	7.807660419	Tumor_G2
7484	NDUFB1	5.663109724	Tumor_G2
7485	NDUFB10	7.553016107	Tumor_G2
7486	NDUFB2	7.085494145	Tumor_G2
7487	NDUFB3	6.483018182	Tumor_G2
7488	NDUFB7	8.757272547	Tumor_G2
7489	CYC1	7.805040377	Tumor_G2
7490	ETFB	5.326721823	Tumor_G2
7491	UQCR10	7.203644071	Tumor_G2
7492	UQCRQ	7.534449905	Tumor_G2
7493	UQCC3	4.228549723	Tumor_G2
7494	COX15	5.327409881	Tumor_G2
7495	COX5A	7.520128836	Tumor_G2
7496	COX6B1	8.897810596	Tumor_G2
7497	COX6C	6.943957143	Tumor_G2
7498	COX7A2	7.139286649	Tumor_G2
7499	COX7B	6.733989095	Tumor_G2
7500	COX8A	10.11078669	Tumor_G2
7501	SCO1	3.565507367	Tumor_G2
7502	SURF1	6.340527191	Tumor_G2
7503	ATP6V1E1	6.597808581	Tumor_G2
7504	DLAT	5.418641953	Tumor_G2
7505	PDC	0.09608204	Tumor_G2
7506	SUCLG2	6.936670832	Tumor_G2
7507	SLC25A5	9.036717432	Tumor_G2
7508	UCP2	7.214238906	Tumor_G2
7509	SOD2	4.662323836	Tumor_G2
7510	NOX4	1.015108549	Tumor_G2
7511	DUOX1	7.23988653	Tumor_G2
7512	DUOXA1	6.415434468	Tumor_G2
7513	DUOX2	10.02132027	Tumor_G2
7514	DUOXA2	6.976821824	Tumor_G2
7515	NDUFA1	8.355419757	Tumor_G2
7516	NDUFA2	6.772265507	Tumor_G2
7517	NDUFA8	7.241111583	Tumor_G2
7518	NDUFB1	5.802865596	Tumor_G2

7519	NDUFB10	7.531544632	Tumor_G2
7520	NDUFB2	6.433444051	Tumor_G2
7521	NDUFB3	6.438232123	Tumor_G2
7522	NDUFB7	8.669404153	Tumor_G2
7523	CYC1	6.786383704	Tumor_G2
7524	ETFB	5.432306615	Tumor_G2
7525	UQCR10	7.54388908	Tumor_G2
7526	UQCRQ	7.213233379	Tumor_G2
7527	UQCC3	4.331717211	Tumor_G2
7528	COX15	5.28628741	Tumor_G2
7529	COX5A	6.810073631	Tumor_G2
7530	COX6B1	8.875413291	Tumor_G2
7531	COX6C	6.477939697	Tumor_G2
7532	COX7A2	6.907190855	Tumor_G2
7533	COX7B	6.041084901	Tumor_G2
7534	COX8A	9.190364844	Tumor_G2
7535	SCO1	3.417772734	Tumor_G2
7536	SURF1	6.440029739	Tumor_G2
7537	ATP6V1E1	6.916345765	Tumor_G2
7538	DLAT	5.064752068	Tumor_G2
7539	PDC	0.186269829	Tumor_G2
7540	SUCLG2	6.044317769	Tumor_G2
7541	SLC25A5	7.702505514	Tumor_G2
7542	UCP2	9.168765598	Tumor_G2
7543	SOD2	4.99228301	Tumor_G2
7544	NOX4	2.202377631	Tumor_G2
7545	DUOX1	7.135106022	Tumor_G2
7546	DUOXA1	7.283997688	Tumor_G2
7547	DUOX2	8.03204909	Tumor_G2
7548	DUOXA2	5.584649692	Tumor_G2
7549	NDUFA1	7.863352284	Tumor_G2
7550	NDUFA2	6.469528087	Tumor_G2
7551	NDUFA8	6.807090618	Tumor_G2
7552	NDUFB1	5.254281552	Tumor_G2
7553	NDUFB10	7.336981776	Tumor_G2
7554	NDUFB2	6.552386153	Tumor_G2
7555	NDUFB3	6.194731887	Tumor_G2
7556	NDUFB7	8.610934752	Tumor_G2
7557	CYC1	6.6353476	Tumor_G2
7558	ETFB	4.996311531	Tumor_G2

7559	UQCR10	7.463283553	Tumor_G2
7560	UQCRQ	6.989729033	Tumor_G2
7561	UQCC3	4.378669293	Tumor_G2
7562	COX15	4.753928549	Tumor_G2
7563	COX5A	6.831197576	Tumor_G2
7564	COX6B1	8.499033392	Tumor_G2
7565	COX6C	6.151927567	Tumor_G2
7566	COX7A2	6.659586131	Tumor_G2
7567	COX7B	5.766676703	Tumor_G2
7568	COX8A	9.306346665	Tumor_G2
7569	SCO1	3.264351979	Tumor_G2
7570	SURF1	6.130081572	Tumor_G2
7571	ATP6V1E1	7.229374256	Tumor_G2
7572	DLAT	5.047724805	Tumor_G2
7573	PDC	0.044659092	Tumor_G2
7574	SUCLG2	6.084024181	Tumor_G2
7575	SLC25A5	7.861307936	Tumor_G2
7576	UCP2	9.290102479	Tumor_G2
7577	SOD2	4.958591523	Tumor_G2
7578	NOX4	2.337331146	Tumor_G2
7579	DUOX1	7.013992578	Tumor_G2
7580	DUOXA1	7.153302472	Tumor_G2
7581	DUOX2	7.370971066	Tumor_G2
7582	DUOXA2	5.059873453	Tumor_G2
7583	NDUFA1	8.230057663	Tumor_G2
7584	NDUFA2	7.143147441	Tumor_G2
7585	NDUFA8	7.373918566	Tumor_G2
7586	NDUFB1	5.773304789	Tumor_G2
7587	NDUFB10	7.794802448	Tumor_G2
7588	NDUFB2	6.765504453	Tumor_G2
7589	NDUFB3	6.544838535	Tumor_G2
7590	NDUFB7	9.033147073	Tumor_G2
7591	CYC1	7.182443865	Tumor_G2
7592	ETFB	5.943226574	Tumor_G2
7593	UQCR10	7.245532491	Tumor_G2
7594	UQCRQ	7.346251882	Tumor_G2
7595	UQCC3	4.589159078	Tumor_G2
7596	COX15	5.048015393	Tumor_G2
7597	COX5A	6.881645008	Tumor_G2
7598	COX6B1	9.068599223	Tumor_G2

7599	COX6C	6.632287781	Tumor_G2
7600	COX7A2	7.209620132	Tumor_G2
7601	COX7B	6.234154852	Tumor_G2
7602	COX8A	9.460083167	Tumor_G2
7603	SCO1	3.405112658	Tumor_G2
7604	SURF1	6.977995574	Tumor_G2
7605	ATP6V1E1	6.469965443	Tumor_G2
7606	DLAT	5.005104962	Tumor_G2
7607	PDC	0.061607458	Tumor_G2
7608	SUCLG2	6.160601201	Tumor_G2
7609	SLC25A5	7.94002484	Tumor_G2
7610	UCP2	9.424845246	Tumor_G2
7611	SOD2	4.58926744	Tumor_G2
7612	NOX4	1.653787241	Tumor_G2
7613	DUOX1	7.099945633	Tumor_G2
7614	DUOXA1	7.14876036	Tumor_G2
7615	DUOX2	8.756688554	Tumor_G2
7616	DUOXA2	6.167131923	Tumor_G2
7617	NDUFA1	8.006158466	Tumor_G2
7618	NDUFA2	6.778560386	Tumor_G2
7619	NDUFA8	7.094779471	Tumor_G2
7620	NDUFB1	5.424276159	Tumor_G2
7621	NDUFB10	7.683383785	Tumor_G2
7622	NDUFB2	6.627129109	Tumor_G2
7623	NDUFB3	6.209896496	Tumor_G2
7624	NDUFB7	8.824592698	Tumor_G2
7625	CYC1	7.146343459	Tumor_G2
7626	ETFB	5.917532496	Tumor_G2
7627	UQCR10	7.139498236	Tumor_G2
7628	UQCRQ	7.322773736	Tumor_G2
7629	UQCC3	4.781519599	Tumor_G2
7630	COX15	4.719527352	Tumor_G2
7631	COX5A	6.899690105	Tumor_G2
7632	COX6B1	8.900637362	Tumor_G2
7633	COX6C	6.279012138	Tumor_G2
7634	COX7A2	7.043033943	Tumor_G2
7635	COX7B	6.126015176	Tumor_G2
7636	COX8A	9.664367087	Tumor_G2
7637	SCO1	3.24616852	Tumor_G2
7638	SURF1	6.36632291	Tumor_G2

7639	ATP6V1E1	6.422267732	Tumor_G2
7640	DLAT	4.723019078	Tumor_G2
7641	PDC	0.055420197	Tumor_G2
7642	SUCLG2	5.835925486	Tumor_G2
7643	SLC25A5	7.66078166	Tumor_G2
7644	UCP2	8.119078165	Tumor_G2
7645	SOD2	4.011226738	Tumor_G2
7646	NOX4	1.406850279	Tumor_G2
7647	DUOX1	7.556601549	Tumor_G2
7648	DUOXA1	7.322596865	Tumor_G2
7649	DUOX2	7.98711028	Tumor_G2
7650	DUOXA2	6.274986863	Tumor_G2
7651	NDUFA1	8.094629185	Tumor_G2
7652	NDUFA2	7.064737788	Tumor_G2
7653	NDUFA8	7.402471088	Tumor_G2
7654	NDUFB1	5.946945481	Tumor_G2
7655	NDUFB10	7.390266809	Tumor_G2
7656	NDUFB2	6.948196117	Tumor_G2
7657	NDUFB3	6.503897087	Tumor_G2
7658	NDUFB7	8.929193701	Tumor_G2
7659	CYC1	6.953482334	Tumor_G2
7660	ETFB	5.449898655	Tumor_G2
7661	UQCR10	7.211437009	Tumor_G2
7662	UQCRQ	7.452466616	Tumor_G2
7663	UQCC3	4.616905312	Tumor_G2
7664	COX15	5.179322398	Tumor_G2
7665	COX5A	7.041798262	Tumor_G2
7666	COX6B1	8.933690755	Tumor_G2
7667	COX6C	6.843339832	Tumor_G2
7668	COX7A2	6.983438277	Tumor_G2
7669	COX7B	6.353926907	Tumor_G2
7670	COX8A	9.809213733	Tumor_G2
7671	SCO1	3.518935245	Tumor_G2
7672	SURF1	7.15176188	Tumor_G2
7673	ATP6V1E1	7.21849067	Tumor_G2
7674	DLAT	4.985260258	Tumor_G2
7675	PDC	0.274916236	Tumor_G2
7676	SUCLG2	6.473100655	Tumor_G2
7677	SLC25A5	8.156960804	Tumor_G2
7678	UCP2	8.292265969	Tumor_G2

7679	SOD2	4.37015471	Tumor_G2
7680	NOX4	1.92382634	Tumor_G2
7681	DUOX1	8.563261973	Tumor_G2
7682	DUOXA1	8.224556249	Tumor_G2
7683	DUOX2	10.38903981	Tumor_G2
7684	DUOXA2	7.957347914	Tumor_G2
7685	NDUFA1	8.388966315	Tumor_G2
7686	NDUFA2	6.974098303	Tumor_G2
7687	NDUFA8	7.22326763	Tumor_G2
7688	NDUFB1	6.024957646	Tumor_G2
7689	NDUFB10	7.730585176	Tumor_G2
7690	NDUFB2	6.797064236	Tumor_G2
7691	NDUFB3	6.562561048	Tumor_G2
7692	NDUFB7	9.163336946	Tumor_G2
7693	CYC1	7.075409375	Tumor_G2
7694	ETFB	5.732986074	Tumor_G2
7695	UQCR10	7.967775673	Tumor_G2
7696	UQCRQ	7.34442956	Tumor_G2
7697	UQCC3	5.163567959	Tumor_G2
7698	COX15	5.180775989	Tumor_G2
7699	COX5A	7.005527959	Tumor_G2
7700	COX6B1	8.839946096	Tumor_G2
7701	COX6C	6.615490755	Tumor_G2
7702	COX7A2	6.8233743	Tumor_G2
7703	COX7B	6.076541467	Tumor_G2
7704	COX8A	9.960700201	Tumor_G2
7705	SCO1	3.357291821	Tumor_G2
7706	SURF1	6.601795106	Tumor_G2
7707	ATP6V1E1	6.88032349	Tumor_G2
7708	DLAT	4.867141669	Tumor_G2
7709	PDC	0.170090374	Tumor_G2
7710	SUCLG2	6.221416014	Tumor_G2
7711	SLC25A5	7.593919978	Tumor_G2
7712	UCP2	9.248726051	Tumor_G2
7713	SOD2	4.371624116	Tumor_G2
7714	NOX4	1.906307859	Tumor_G2
7715	DUOX1	6.937342164	Tumor_G2
7716	DUOXA1	6.850313141	Tumor_G2
7717	DUOX2	7.687905845	Tumor_G2
7718	DUOXA2	6.811276323	Tumor_G2

7719	NDUFA1	8.212139327	Tumor_G2
7720	NDUFA2	6.882389513	Tumor_G2
7721	NDUFA8	7.265976718	Tumor_G2
7722	NDUFB1	5.870526993	Tumor_G2
7723	NDUFB10	7.510132822	Tumor_G2
7724	NDUFB2	7.111763814	Tumor_G2
7725	NDUFB3	6.668122863	Tumor_G2
7726	NDUFB7	8.941617161	Tumor_G2
7727	CYC1	7.081080775	Tumor_G2
7728	ETFB	5.463291873	Tumor_G2
7729	UQCR10	7.929848901	Tumor_G2
7730	UQCRQ	7.382223621	Tumor_G2
7731	UQCC3	4.239740564	Tumor_G2
7732	COX15	5.233864857	Tumor_G2
7733	COX5A	7.017384055	Tumor_G2
7734	COX6B1	8.963228921	Tumor_G2
7735	COX6C	6.711105449	Tumor_G2
7736	COX7A2	6.927694378	Tumor_G2
7737	COX7B	6.249643818	Tumor_G2
7738	COX8A	9.402562777	Tumor_G2
7739	SCO1	3.856109657	Tumor_G2
7740	SURF1	6.809774108	Tumor_G2
7741	ATP6V1E1	6.776520508	Tumor_G2
7742	DLAT	5.047530686	Tumor_G2
7743	PDC	0.225943747	Tumor_G2
7744	SUCLG2	6.595055994	Tumor_G2
7745	SLC25A5	8.222527934	Tumor_G2
7746	UCP2	8.760657038	Tumor_G2
7747	SOD2	5.268888239	Tumor_G2
7748	NOX4	1.168353067	Tumor_G2
7749	DUOX1	7.495302615	Tumor_G2
7750	DUOXA1	6.948452848	Tumor_G2
7751	DUOX2	8.680152861	Tumor_G2
7752	DUOXA2	7.084833344	Tumor_G2
7753	NDUFA1	7.774795171	Tumor_G2
7754	NDUFA2	6.447953092	Tumor_G2
7755	NDUFA8	7.050905569	Tumor_G2
7756	NDUFB1	5.473677359	Tumor_G2
7757	NDUFB10	7.204374952	Tumor_G2
7758	NDUFB2	6.301973832	Tumor_G2

7759	NDUFB3	6.137737346	Tumor_G2
7760	NDUFB7	8.495491606	Tumor_G2
7761	CYC1	6.752535427	Tumor_G2
7762	ETFB	4.997968306	Tumor_G2
7763	UQCR10	7.655657688	Tumor_G2
7764	UQCRQ	6.872339516	Tumor_G2
7765	UQCC3	4.233115165	Tumor_G2
7766	COX15	5.06428622	Tumor_G2
7767	COX5A	6.754429147	Tumor_G2
7768	COX6B1	8.597334868	Tumor_G2
7769	COX6C	6.441184879	Tumor_G2
7770	COX7A2	6.764064285	Tumor_G2
7771	COX7B	5.915498663	Tumor_G2
7772	COX8A	9.007602744	Tumor_G2
7773	SCO1	3.465157969	Tumor_G2
7774	SURF1	6.638586028	Tumor_G2
7775	ATP6V1E1	6.883999375	Tumor_G2
7776	DLAT	5.207683734	Tumor_G2
7777	PDC	0.311327722	Tumor_G2
7778	SUCLG2	6.187209065	Tumor_G2
7779	SLC25A5	7.719402817	Tumor_G2
7780	UCP2	9.371456413	Tumor_G2
7781	SOD2	5.177930953	Tumor_G2
7782	NOX4	3.055057058	Tumor_G2
7783	DUOX1	6.79149209	Tumor_G2
7784	DUOXA1	7.16927608	Tumor_G2
7785	DUOX2	7.314387048	Tumor_G2
7786	DUOXA2	5.03540936	Tumor_G2
7787	NDUFA1	8.349077647	Tumor_G2
7788	NDUFA2	7.041218592	Tumor_G2
7789	NDUFA8	7.423920712	Tumor_G2
7790	NDUFB1	6.242565021	Tumor_G2
7791	NDUFB10	7.746007438	Tumor_G2
7792	NDUFB2	6.929025978	Tumor_G2
7793	NDUFB3	6.628141685	Tumor_G2
7794	NDUFB7	9.133372205	Tumor_G2
7795	CYC1	7.087972838	Tumor_G2
7796	ETFB	5.386501653	Tumor_G2
7797	UQCR10	7.958008196	Tumor_G2
7798	UQCRQ	7.566993164	Tumor_G2

7799	UQCC3	4.626236682	Tumor_G2
7800	COX15	4.945489838	Tumor_G2
7801	COX5A	7.125600683	Tumor_G2
7802	COX6B1	8.90751072	Tumor_G2
7803	COX6C	6.847145404	Tumor_G2
7804	COX7A2	7.110854527	Tumor_G2
7805	COX7B	6.408090202	Tumor_G2
7806	COX8A	9.54746849	Tumor_G2
7807	SCO1	3.451567529	Tumor_G2
7808	SURF1	6.513992043	Tumor_G2
7809	ATP6V1E1	6.894304059	Tumor_G2
7810	DLAT	5.057055647	Tumor_G2
7811	PDC	0.205822444	Tumor_G2
7812	SUCLG2	5.91581023	Tumor_G2
7813	SLC25A5	8.211888245	Tumor_G2
7814	UCP2	7.479625114	Tumor_G2
7815	SOD2	4.87299902	Tumor_G2
7816	NOX4	2.491312408	Tumor_G2
7817	DUOX1	7.379089867	Tumor_G2
7818	DUOXA1	7.065634181	Tumor_G2
7819	DUOX2	8.239607727	Tumor_G2
7820	DUOXA2	5.989747532	Tumor_G2
7821	NDUFA1	8.071309588	Tumor_G2
7822	NDUFA2	6.716673053	Tumor_G2
7823	NDUFA8	7.039228257	Tumor_G2
7824	NDUFB1	5.627619017	Tumor_G2
7825	NDUFB10	7.39956658	Tumor_G2
7826	NDUFB2	6.647753994	Tumor_G2
7827	NDUFB3	6.372631573	Tumor_G2
7828	NDUFB7	8.615905643	Tumor_G2
7829	CYC1	7.149936491	Tumor_G2
7830	ETFB	5.061570215	Tumor_G2
7831	UQCR10	7.627941406	Tumor_G2
7832	UQCRQ	7.096751752	Tumor_G2
7833	UQCC3	4.064097129	Tumor_G2
7834	COX15	5.081181978	Tumor_G2
7835	COX5A	6.896643558	Tumor_G2
7836	COX6B1	8.764684927	Tumor_G2
7837	COX6C	6.351483786	Tumor_G2
7838	COX7A2	6.935670377	Tumor_G2

7839	COX7B	6.134878192	Tumor_G2
7840	COX8A	9.24072701	Tumor_G2
7841	SCO1	3.517171183	Tumor_G2
7842	SURF1	6.600194732	Tumor_G2
7843	ATP6V1E1	6.998021989	Tumor_G2
7844	DLAT	5.165432296	Tumor_G2
7845	PDC	0.151900311	Tumor_G2
7846	SUCLG2	6.370887261	Tumor_G2
7847	SLC25A5	8.415565985	Tumor_G2
7848	UCP2	8.07528966	Tumor_G2
7849	SOD2	5.270112661	Tumor_G2
7850	NOX4	1.01743273	Tumor_G2
7851	DUOX1	6.472349715	Tumor_G2
7852	DUOXA1	6.652782158	Tumor_G2
7853	DUOX2	6.900818276	Tumor_G2
7854	DUOXA2	4.837548706	Tumor_G2
7855	NDUFA1	8.263395878	Tumor_G2
7856	NDUFA2	6.986762769	Tumor_G2
7857	NDUFA8	7.160301654	Tumor_G2
7858	NDUFB1	5.937269897	Tumor_G2
7859	NDUFB10	7.515417796	Tumor_G2
7860	NDUFB2	6.85204661	Tumor_G2
7861	NDUFB3	6.506640601	Tumor_G2
7862	NDUFB7	8.90077829	Tumor_G2
7863	CYC1	6.99829738	Tumor_G2
7864	ETFB	5.351464382	Tumor_G2
7865	UQCR10	7.878469245	Tumor_G2
7866	UQCRQ	7.249249044	Tumor_G2
7867	UQCC3	4.556090144	Tumor_G2
7868	COX15	5.202046261	Tumor_G2
7869	COX5A	6.796661545	Tumor_G2
7870	COX6B1	8.729942075	Tumor_G2
7871	COX6C	6.657055323	Tumor_G2
7872	COX7A2	7.029690414	Tumor_G2
7873	COX7B	6.109994972	Tumor_G2
7874	COX8A	9.562773788	Tumor_G2
7875	SCO1	3.537066134	Tumor_G2
7876	SURF1	7.056053212	Tumor_G2
7877	ATP6V1E1	6.788931156	Tumor_G2
7878	DLAT	5.039270206	Tumor_G2

7879	PDC	0.269001598	Tumor_G2
7880	SUCLG2	6.274016473	Tumor_G2
7881	SLC25A5	8.120190741	Tumor_G2
7882	UCP2	6.799312774	Tumor_G2
7883	SOD2	4.845105279	Tumor_G2
7884	NOX4	0.849124947	Tumor_G2
7885	DUOX1	6.90382247	Tumor_G2
7886	DUOXA1	6.893215923	Tumor_G2
7887	DUOX2	7.443577805	Tumor_G2
7888	DUOXA2	5.637727969	Tumor_G2
7889	NDUFA1	8.287101433	Tumor_G2
7890	NDUFA2	6.972088966	Tumor_G2
7891	NDUFA8	7.110117822	Tumor_G2
7892	NDUFB1	5.795805177	Tumor_G2
7893	NDUFB10	7.69026126	Tumor_G2
7894	NDUFB2	6.774969348	Tumor_G2
7895	NDUFB3	6.24160073	Tumor_G2
7896	NDUFB7	8.733672477	Tumor_G2
7897	CYC1	6.762372079	Tumor_G2
7898	ETFB	4.819224598	Tumor_G2
7899	UQCR10	7.861920565	Tumor_G2
7900	UQCRQ	7.366548268	Tumor_G2
7901	UQCC3	4.39250079	Tumor_G2
7902	COX15	4.343473722	Tumor_G2
7903	COX5A	7.083836308	Tumor_G2
7904	COX6B1	8.934372808	Tumor_G2
7905	COX6C	6.670633162	Tumor_G2
7906	COX7A2	7.122996132	Tumor_G2
7907	COX7B	6.198747789	Tumor_G2
7908	COX8A	9.392125695	Tumor_G2
7909	SCO1	2.628156592	Tumor_G2
7910	SURF1	6.04809389	Tumor_G2
7911	ATP6V1E1	7.078076401	Tumor_G2
7912	DLAT	4.303973597	Tumor_G2
7913	PDC	0.282566845	Tumor_G2
7914	SUCLG2	5.66971595	Tumor_G2
7915	SLC25A5	7.771471955	Tumor_G2
7916	UCP2	8.423168615	Tumor_G2
7917	SOD2	4.592962046	Tumor_G2
7918	NOX4	2.39974501	Tumor_G2

7919	DUOX1	5.940871516	Tumor_G2
7920	DUOXA1	6.038029078	Tumor_G2
7921	DUOX2	4.895333396	Tumor_G2
7922	DUOXA2	3.638827645	Tumor_G2
7923	NDUFA1	8.515856384	Tumor_G2
7924	NDUFA2	6.973779623	Tumor_G2
7925	NDUFA8	7.220968902	Tumor_G2
7926	NDUFB1	6.053913292	Tumor_G2
7927	NDUFB10	7.656247986	Tumor_G2
7928	NDUFB2	6.720559787	Tumor_G2
7929	NDUFB3	6.650939586	Tumor_G2
7930	NDUFB7	8.853061929	Tumor_G2
7931	CYC1	6.884437864	Tumor_G2
7932	ETFB	5.35719319	Tumor_G2
7933	UQCR10	8.017858976	Tumor_G2
7934	UQCRQ	7.349477812	Tumor_G2
7935	UQCC3	4.596221528	Tumor_G2
7936	COX15	4.761905102	Tumor_G2
7937	COX5A	7.123444044	Tumor_G2
7938	COX6B1	9.045959397	Tumor_G2
7939	COX6C	6.775699743	Tumor_G2
7940	COX7A2	7.164371966	Tumor_G2
7941	COX7B	6.315321829	Tumor_G2
7942	COX8A	9.300658144	Tumor_G2
7943	SCO1	3.174078123	Tumor_G2
7944	SURF1	6.618812421	Tumor_G2
7945	ATP6V1E1	6.934885291	Tumor_G2
7946	DLAT	4.690392811	Tumor_G2
7947	PDC	0.170514497	Tumor_G2
7948	SUCLG2	5.979244811	Tumor_G2
7949	SLC25A5	7.821579187	Tumor_G2
7950	UCP2	9.023724231	Tumor_G2
7951	SOD2	5.052248086	Tumor_G2
7952	NOX4	1.623207975	Tumor_G2
7953	DUOX1	6.694197412	Tumor_G2
7954	DUOXA1	6.7492651	Tumor_G2
7955	DUOX2	7.723849009	Tumor_G2
7956	DUOXA2	6.279430357	Tumor_G2
7957	NDUFA1	8.308414676	Tumor_G2
7958	NDUFA2	7.047944625	Tumor_G2

7959	NDUFA8	7.337747739	Tumor_G2
7960	NDUFB1	6.125379078	Tumor_G2
7961	NDUFB10	8.081013815	Tumor_G2
7962	NDUFB2	6.806894584	Tumor_G2
7963	NDUFB3	6.450307162	Tumor_G2
7964	NDUFB7	9.139569463	Tumor_G2
7965	CYC1	6.995571519	Tumor_G2
7966	ETFB	5.573826805	Tumor_G2
7967	UQCR10	7.989935189	Tumor_G2
7968	UQCRQ	7.594062185	Tumor_G2
7969	UQCC3	4.980981544	Tumor_G2
7970	COX15	4.793153597	Tumor_G2
7971	COX5A	7.26138544	Tumor_G2
7972	COX6B1	8.910704415	Tumor_G2
7973	COX6C	6.713613226	Tumor_G2
7974	COX7A2	7.003511387	Tumor_G2
7975	COX7B	6.223525191	Tumor_G2
7976	COX8A	9.76768712	Tumor_G2
7977	SCO1	3.210633999	Tumor_G2
7978	SURF1	6.742808388	Tumor_G2
7979	ATP6V1E1	6.809323688	Tumor_G2
7980	DLAT	4.702494409	Tumor_G2
7981	PDC	0.263065294	Tumor_G2
7982	SUCLG2	5.924123745	Tumor_G2
7983	SLC25A5	7.583766817	Tumor_G2
7984	UCP2	8.857997081	Tumor_G2
7985	SOD2	4.246104951	Tumor_G2
7986	NOX4	1.517472339	Tumor_G2
7987	DUOX1	7.1413928	Tumor_G2
7988	DUOXA1	7.030186205	Tumor_G2
7989	DUOX2	7.954888652	Tumor_G2
7990	DUOXA2	6.448023061	Tumor_G2
7991	NDUFA1	8.149698354	Tumor_G2
7992	NDUFA2	6.797724783	Tumor_G2
7993	NDUFA8	6.989281456	Tumor_G2
7994	NDUFB1	5.782221563	Tumor_G2
7995	NDUFB10	7.393601342	Tumor_G2
7996	NDUFB2	6.741176902	Tumor_G2
7997	NDUFB3	6.591841654	Tumor_G2
7998	NDUFB7	8.327345352	Tumor_G2

7999	CYC1	6.970416148	Tumor_G2
8000	ETFB	4.859533578	Tumor_G2
8001	UQCR10	7.683015264	Tumor_G2
8002	UQCRQ	7.075480779	Tumor_G2
8003	UQCC3	3.821837855	Tumor_G2
8004	COX15	5.110624663	Tumor_G2
8005	COX5A	6.972782739	Tumor_G2
8006	COX6B1	8.610984019	Tumor_G2
8007	COX6C	6.598574323	Tumor_G2
8008	COX7A2	7.00474146	Tumor_G2
8009	COX7B	6.283706337	Tumor_G2
8010	COX8A	9.214073582	Tumor_G2
8011	SCO1	3.328865783	Tumor_G2
8012	SURF1	6.288854011	Tumor_G2
8013	ATP6V1E1	7.114631139	Tumor_G2
8014	DLAT	5.168646968	Tumor_G2
8015	PDC	0	Tumor_G2
8016	SUCLG2	6.491180894	Tumor_G2
8017	SLC25A5	8.301420682	Tumor_G2
8018	UCP2	7.23360497	Tumor_G2
8019	SOD2	5.381791076	Tumor_G2
8020	NOX4	1.81553736	Tumor_G2
8021	DUOX1	6.44220419	Tumor_G2
8022	DUOXA1	6.275961509	Tumor_G2
8023	DUOX2	6.523865125	Tumor_G2
8024	DUOXA2	4.314921905	Tumor_G2
8025	NDUFA1	8.304201076	Tumor_G2
8026	NDUFA2	7.244148425	Tumor_G2
8027	NDUFA8	7.066187781	Tumor_G2
8028	NDUFB1	5.974361436	Tumor_G2
8029	NDUFB10	7.904132964	Tumor_G2
8030	NDUFB2	6.989989919	Tumor_G2
8031	NDUFB3	6.559750168	Tumor_G2
8032	NDUFB7	9.003247872	Tumor_G2
8033	CYC1	7.151027582	Tumor_G2
8034	ETFB	5.314459947	Tumor_G2
8035	UQCR10	8.024810869	Tumor_G2
8036	UQCRQ	7.681781491	Tumor_G2
8037	UQCC3	4.679948539	Tumor_G2
8038	COX15	4.817615728	Tumor_G2

8039	COX5A	7.312712638	Tumor_G2
8040	COX6B1	9.024450188	Tumor_G2
8041	COX6C	6.791118221	Tumor_G2
8042	COX7A2	7.257356658	Tumor_G2
8043	COX7B	6.309572036	Tumor_G2
8044	COX8A	9.783413158	Tumor_G2
8045	SCO1	3.056797735	Tumor_G2
8046	SURF1	6.088303318	Tumor_G2
8047	ATP6V1E1	7.465150277	Tumor_G2
8048	DLAT	4.983615756	Tumor_G2
8049	PDC	0.04488562	Tumor_G2
8050	SUCLG2	6.320698412	Tumor_G2
8051	SLC25A5	8.377044318	Tumor_G2
8052	UCP2	8.3313742	Tumor_G2
8053	SOD2	5.081112243	Tumor_G2
8054	NOX4	1.989459857	Tumor_G2
8055	DUOX1	6.008429619	Tumor_G2
8056	DUOXA1	6.224358733	Tumor_G2
8057	DUOX2	5.851542326	Tumor_G2
8058	DUOXA2	4.411360756	Tumor_G2
8059	NDUFA1	8.387538885	Tumor_G2
8060	NDUFA2	7.166020607	Tumor_G2
8061	NDUFA8	7.069434727	Tumor_G2
8062	NDUFB1	5.844816959	Tumor_G2
8063	NDUFB10	7.65226505	Tumor_G2
8064	NDUFB2	6.909582433	Tumor_G2
8065	NDUFB3	6.627552425	Tumor_G2
8066	NDUFB7	8.619639218	Tumor_G2
8067	CYC1	6.840615515	Tumor_G2
8068	ETFB	4.921705215	Tumor_G2
8069	UQCR10	7.806437743	Tumor_G2
8070	UQCRQ	7.534335181	Tumor_G2
8071	UQCC3	4.478367292	Tumor_G2
8072	COX15	4.618661124	Tumor_G2
8073	COX5A	7.586087524	Tumor_G2
8074	COX6B1	8.904623702	Tumor_G2
8075	COX6C	6.66482937	Tumor_G2
8076	COX7A2	7.18408717	Tumor_G2
8077	COX7B	6.189309145	Tumor_G2
8078	COX8A	9.583512018	Tumor_G2

8079	SCO1	3.120186351	Tumor_G2
8080	SURF1	6.038191378	Tumor_G2
8081	ATP6V1E1	7.286060504	Tumor_G2
8082	DLAT	5.131355791	Tumor_G2
8083	PDC	0.042809399	Tumor_G2
8084	SUCLG2	6.395310668	Tumor_G2
8085	SLC25A5	8.30678696	Tumor_G2
8086	UCP2	8.419369641	Tumor_G2
8087	SOD2	5.328737205	Tumor_G2
8088	NOX4	2.281972558	Tumor_G2
8089	DUOX1	5.418661671	Tumor_G2
8090	DUOXA1	5.706227932	Tumor_G2
8091	DUOX2	5.594173987	Tumor_G2
8092	DUOXA2	4.227450948	Tumor_G2
8093	NDUFA1	7.96833253	Tumor_G2
8094	NDUFA2	6.528783429	Tumor_G2
8095	NDUFA8	7.056231858	Tumor_G2
8096	NDUFB1	5.630346025	Tumor_G2
8097	NDUFB10	7.594198089	Tumor_G2
8098	NDUFB2	6.696522974	Tumor_G2
8099	NDUFB3	6.450366145	Tumor_G2
8100	NDUFB7	8.573751323	Tumor_G2
8101	CYC1	6.761074732	Tumor_G2
8102	ETFB	5.025676198	Tumor_G2
8103	UQCR10	7.507265315	Tumor_G2
8104	UQCRQ	7.187623246	Tumor_G2
8105	UQCC3	4.250799544	Tumor_G2
8106	COX15	4.729371617	Tumor_G2
8107	COX5A	7.045457915	Tumor_G2
8108	COX6B1	8.702749982	Tumor_G2
8109	COX6C	6.430121162	Tumor_G2
8110	COX7A2	7.006662073	Tumor_G2
8111	COX7B	6.097154263	Tumor_G2
8112	COX8A	9.595830427	Tumor_G2
8113	SCO1	3.406414019	Tumor_G2
8114	SURF1	6.407924546	Tumor_G2
8115	ATP6V1E1	6.950672319	Tumor_G2
8116	DLAT	4.997684883	Tumor_G2
8117	PDC	0.309805891	Tumor_G2
8118	SUCLG2	5.931718515	Tumor_G2

8119	SLC25A5	7.966607637	Tumor_G2
8120	UCP2	9.351769658	Tumor_G2
8121	SOD2	4.49924137	Tumor_G2
8122	NOX4	2.286446041	Tumor_G2
8123	DUOX1	6.599638324	Tumor_G2
8124	DUOXA1	6.885905535	Tumor_G2
8125	DUOX2	6.890799783	Tumor_G2
8126	DUOXA2	4.180581198	Tumor_G2
8127	NDUFA1	8.124450575	Tumor_G2
8128	NDUFA2	6.617546701	Tumor_G2
8129	NDUFA8	6.988353568	Tumor_G2
8130	NDUFB1	5.555585945	Tumor_G2
8131	NDUFB10	7.201244924	Tumor_G2
8132	NDUFB2	6.667802803	Tumor_G2
8133	NDUFB3	6.540567304	Tumor_G2
8134	NDUFB7	8.555236411	Tumor_G2
8135	CYC1	6.698838374	Tumor_G2
8136	ETFB	5.030863519	Tumor_G2
8137	UQCR10	7.425521779	Tumor_G2
8138	UQCRQ	7.273868137	Tumor_G2
8139	UQCC3	4.320300557	Tumor_G2
8140	COX15	4.757740496	Tumor_G2
8141	COX5A	7.022819593	Tumor_G2
8142	COX6B1	8.635368185	Tumor_G2
8143	COX6C	6.217729501	Tumor_G2
8144	COX7A2	6.833591178	Tumor_G2
8145	COX7B	5.919573384	Tumor_G2
8146	COX8A	9.427018844	Tumor_G2
8147	SCO1	3.244436509	Tumor_G2
8148	SURF1	6.210012545	Tumor_G2
8149	ATP6V1E1	7.12200657	Tumor_G2
8150	DLAT	5.057380915	Tumor_G2
8151	PDC	0.137838147	Tumor_G2
8152	SUCLG2	6.065277828	Tumor_G2
8153	SLC25A5	7.650124975	Tumor_G2
8154	UCP2	8.487806305	Tumor_G2
8155	SOD2	4.739303398	Tumor_G2
8156	NOX4	2.392346366	Tumor_G2
8157	DUOX1	5.947844875	Tumor_G2
8158	DUOXA1	6.254146155	Tumor_G2

8159	DUOX2	6.235122762	Tumor_G2
8160	DUOXA2	4.916791232	Tumor_G2
8161	NDUFA1	8.459208043	Tumor_G2
8162	NDUFA2	7.100778129	Tumor_G2
8163	NDUFA8	7.337127443	Tumor_G2
8164	NDUFB1	6.22868287	Tumor_G2
8165	NDUFB10	7.657735283	Tumor_G2
8166	NDUFB2	7.128933196	Tumor_G2
8167	NDUFB3	6.806053318	Tumor_G2
8168	NDUFB7	8.858006182	Tumor_G2
8169	CYC1	7.146419444	Tumor_G2
8170	ETFB	5.123165128	Tumor_G2
8171	UQCR10	7.955546781	Tumor_G2
8172	UQCRQ	7.82477491	Tumor_G2
8173	UQCC3	4.718972707	Tumor_G2
8174	COX15	4.271420565	Tumor_G2
8175	COX5A	7.343798202	Tumor_G2
8176	COX6B1	9.063788096	Tumor_G2
8177	COX6C	6.607669764	Tumor_G2
8178	COX7A2	7.336349812	Tumor_G2
8179	COX7B	6.564243265	Tumor_G2
8180	COX8A	9.796578117	Tumor_G2
8181	SCO1	3.363417826	Tumor_G2
8182	SURF1	6.265918793	Tumor_G2
8183	ATP6V1E1	7.319731881	Tumor_G2
8184	DLAT	5.059011676	Tumor_G2
8185	PDC	0.105089014	Tumor_G2
8186	SUCLG2	6.015361944	Tumor_G2
8187	SLC25A5	8.311738522	Tumor_G2
8188	UCP2	9.421423406	Tumor_G2
8189	SOD2	4.626702637	Tumor_G2
8190	NOX4	2.95667247	Tumor_G2
8191	DUOX1	5.865071398	Tumor_G2
8192	DUOXA1	6.474223177	Tumor_G2
8193	DUOX2	6.223150139	Tumor_G2
8194	DUOXA2	4.907743418	Tumor_G2
8195	NDUFA1	7.820251494	Tumor_G2
8196	NDUFA2	6.558163036	Tumor_G2
8197	NDUFA8	7.035126788	Tumor_G2
8198	NDUFB1	5.506000715	Tumor_G2

8199	NDUFB10	7.374200821	Tumor_G2
8200	NDUFB2	6.576218351	Tumor_G2
8201	NDUFB3	6.172679113	Tumor_G2
8202	NDUFB7	8.295775238	Tumor_G2
8203	CYC1	7.291961926	Tumor_G2
8204	ETFB	5.248174107	Tumor_G2
8205	UQCR10	6.4707038	Tumor_G2
8206	UQCRQ	7.141039203	Tumor_G2
8207	UQCC3	4.183977769	Tumor_G2
8208	COX15	4.810811748	Tumor_G2
8209	COX5A	7.093940105	Tumor_G2
8210	COX6B1	8.192768307	Tumor_G2
8211	COX6C	6.102845268	Tumor_G2
8212	COX7A2	6.699315476	Tumor_G2
8213	COX7B	5.829789417	Tumor_G2
8214	COX8A	9.604631589	Tumor_G2
8215	SCO1	3.132394811	Tumor_G2
8216	SURF1	6.288305457	Tumor_G2
8217	ATP6V1E1	6.229823796	Tumor_G2
8218	DLAT	4.743945861	Tumor_G2
8219	PDC	0.135517244	Tumor_G2
8220	SUCLG2	6.583681533	Tumor_G2
8221	SLC25A5	8.575203307	Tumor_G2
8222	UCP2	7.955162316	Tumor_G2
8223	SOD2	3.874352663	Tumor_G2
8224	NOX4	1.837661871	Tumor_G2
8225	DUOX1	7.167648087	Tumor_G2
8226	DUOXA1	6.298670782	Tumor_G2
8227	DUOX2	9.525120385	Tumor_G2
8228	DUOXA2	7.339653707	Tumor_G2
8229	NDUFA1	8.049111015	Tumor_G2
8230	NDUFA2	6.868252912	Tumor_G2
8231	NDUFA8	7.117516215	Tumor_G2
8232	NDUFB1	5.932874748	Tumor_G2
8233	NDUFB10	7.545447139	Tumor_G2
8234	NDUFB2	6.801660595	Tumor_G2
8235	NDUFB3	6.474255591	Tumor_G2
8236	NDUFB7	8.844480579	Tumor_G2
8237	CYC1	6.847471942	Tumor_G2
8238	ETFB	5.129917709	Tumor_G2

8239	UQCR10	7.716290393	Tumor_G2
8240	UQCRQ	7.483124882	Tumor_G2
8241	UQCC3	4.685274303	Tumor_G2
8242	COX15	4.852229882	Tumor_G2
8243	COX5A	6.999480277	Tumor_G2
8244	COX6B1	8.586900838	Tumor_G2
8245	COX6C	6.422619896	Tumor_G2
8246	COX7A2	6.794905441	Tumor_G2
8247	COX7B	6.036417421	Tumor_G2
8248	COX8A	9.656285194	Tumor_G2
8249	SCO1	3.254732709	Tumor_G2
8250	SURF1	6.60435662	Tumor_G2
8251	ATP6V1E1	6.686708816	Tumor_G2
8252	DLAT	4.834826675	Tumor_G2
8253	PDC	0.062150993	Tumor_G2
8254	SUCLG2	6.005748369	Tumor_G2
8255	SLC25A5	8.084263545	Tumor_G2
8256	UCP2	7.455795071	Tumor_G2
8257	SOD2	4.75053995	Tumor_G2
8258	NOX4	1.315612763	Tumor_G2
8259	DUOX1	7.659486061	Tumor_G2
8260	DUOXA1	7.423635388	Tumor_G2
8261	DUOX2	8.400969385	Tumor_G2
8262	DUOXA2	6.314002644	Tumor_G2
8263	NDUFA1	7.969258087	Tumor_G2
8264	NDUFA2	6.703781214	Tumor_G2
8265	NDUFA8	7.250229493	Tumor_G2
8266	NDUFB1	5.754133799	Tumor_G2
8267	NDUFB10	7.757284172	Tumor_G2
8268	NDUFB2	6.412763043	Tumor_G2
8269	NDUFB3	6.108870181	Tumor_G2
8270	NDUFB7	8.698507511	Tumor_G2
8271	CYC1	6.957298128	Tumor_G2
8272	ETFB	4.955381365	Tumor_G2
8273	UQCR10	7.586182558	Tumor_G2
8274	UQCRQ	7.263083214	Tumor_G2
8275	UQCC3	4.667661807	Tumor_G2
8276	COX15	5.043427431	Tumor_G2
8277	COX5A	7.100630608	Tumor_G2
8278	COX6B1	8.565625802	Tumor_G2

8279	COX6C	6.499289787	Tumor_G2
8280	COX7A2	6.849745158	Tumor_G2
8281	COX7B	6.000497129	Tumor_G2
8282	COX8A	9.390986472	Tumor_G2
8283	SCO1	3.44741242	Tumor_G2
8284	SURF1	6.349516256	Tumor_G2
8285	ATP6V1E1	6.761387189	Tumor_G2
8286	DLAT	5.134372133	Tumor_G2
8287	PDC	0.127173967	Tumor_G2
8288	SUCLG2	5.913607437	Tumor_G2
8289	SLC25A5	7.961532967	Tumor_G2
8290	UCP2	8.237624457	Tumor_G2
8291	SOD2	4.695024964	Tumor_G2
8292	NOX4	2.708415694	Tumor_G2
8293	DUOX1	7.308200115	Tumor_G2
8294	DUOXA1	7.072770292	Tumor_G2
8295	DUOX2	6.917489329	Tumor_G2
8296	DUOXA2	5.855813265	Tumor_G2
8297	NDUFA1	8.49618894	Tumor_G2
8298	NDUFA2	7.364853093	Tumor_G2
8299	NDUFA8	7.370595743	Tumor_G2
8300	NDUFB1	6.482595387	Tumor_G2
8301	NDUFB10	7.859278002	Tumor_G2
8302	NDUFB2	6.658359724	Tumor_G2
8303	NDUFB3	5.749504019	Tumor_G2
8304	NDUFB7	9.41530947	Tumor_G2
8305	CYC1	6.28589262	Tumor_G2
8306	ETFB	5.474148886	Tumor_G2
8307	UQCR10	6.943761634	Tumor_G2
8308	UQCRQ	7.822889812	Tumor_G2
8309	UQCC3	4.742422477	Tumor_G2
8310	COX15	4.282227291	Tumor_G2
8311	COX5A	5.989136018	Tumor_G2
8312	COX6B1	9.357318099	Tumor_G2
8313	COX6C	6.925601982	Tumor_G2
8314	COX7A2	6.695177522	Tumor_G2
8315	COX7B	6.142042811	Tumor_G2
8316	COX8A	9.504796138	Tumor_G2
8317	SCO1	2.379163082	Tumor_G2
8318	SURF1	5.952637656	Tumor_G2

8319	ATP6V1E1	6.214785231	Tumor_G2
8320	DLAT	3.99548067	Tumor_G2
8321	PDC	0.060473683	Tumor_G2
8322	SUCLG2	5.697193477	Tumor_G2
8323	SLC25A5	6.91372231	Tumor_G2
8324	UCP2	8.376549553	Tumor_G2
8325	SOD2	3.164744698	Tumor_G2
8326	NOX4	1.577252567	Tumor_G2
8327	DUOX1	6.237688693	Tumor_G2
8328	DUOXA1	5.748469604	Tumor_G2
8329	DUOX2	7.873122347	Tumor_G2
8330	DUOXA2	5.923067759	Tumor_G2
8331	NDUFA1	8.239991952	Tumor_G2
8332	NDUFA2	6.887907008	Tumor_G2
8333	NDUFA8	7.110075123	Tumor_G2
8334	NDUFB1	5.997228489	Tumor_G2
8335	NDUFB10	7.58175355	Tumor_G2
8336	NDUFB2	6.421767975	Tumor_G2
8337	NDUFB3	6.31621998	Tumor_G2
8338	NDUFB7	8.825828881	Tumor_G2
8339	CYC1	6.784388481	Tumor_G2
8340	ETFB	4.974061317	Tumor_G2
8341	UQCR10	7.753348693	Tumor_G2
8342	UQCRQ	7.259458593	Tumor_G2
8343	UQCC3	4.682876963	Tumor_G2
8344	COX15	4.381494109	Tumor_G2
8345	COX5A	6.929386232	Tumor_G2
8346	COX6B1	8.913657823	Tumor_G2
8347	COX6C	6.770178856	Tumor_G2
8348	COX7A2	6.996721486	Tumor_G2
8349	COX7B	6.202922276	Tumor_G2
8350	COX8A	9.431081927	Tumor_G2
8351	SCO1	3.068737264	Tumor_G2
8352	SURF1	6.509675755	Tumor_G2
8353	ATP6V1E1	6.730050887	Tumor_G2
8354	DLAT	4.809987467	Tumor_G2
8355	PDC	0.259433974	Tumor_G2
8356	SUCLG2	5.882243997	Tumor_G2
8357	SLC25A5	7.880636874	Tumor_G2
8358	UCP2	8.598992937	Tumor_G2

8359	SOD2	4.747005185	Tumor_G2
8360	NOX4	2.978283584	Tumor_G2
8361	DUOX1	6.460132263	Tumor_G2
8362	DUOXA1	6.96488357	Tumor_G2
8363	DUOX2	6.891719922	Tumor_G2
8364	DUOXA2	4.583220054	Tumor_G2
8365	NDUFA1	7.894495791	Tumor_G2
8366	NDUFA2	6.612934572	Tumor_G2
8367	NDUFA8	7.040199546	Tumor_G2
8368	NDUFB1	5.688712067	Tumor_G2
8369	NDUFB10	7.304300483	Tumor_G2
8370	NDUFB2	6.575067889	Tumor_G2
8371	NDUFB3	6.313286723	Tumor_G2
8372	NDUFB7	8.469367257	Tumor_G2
8373	CYC1	6.903762764	Tumor_G2
8374	ETFB	4.800871774	Tumor_G2
8375	UQCR10	7.454188908	Tumor_G2
8376	UQCRQ	7.021572563	Tumor_G2
8377	UQCC3	4.232265881	Tumor_G2
8378	COX15	4.778355375	Tumor_G2
8379	COX5A	7.023274203	Tumor_G2
8380	COX6B1	8.351919818	Tumor_G2
8381	COX6C	6.37132531	Tumor_G2
8382	COX7A2	6.675422855	Tumor_G2
8383	COX7B	6.164155579	Tumor_G2
8384	COX8A	9.51542152	Tumor_G2
8385	SCO1	3.520044317	Tumor_G2
8386	SURF1	6.021900182	Tumor_G2
8387	ATP6V1E1	6.470200544	Tumor_G2
8388	DLAT	5.235947033	Tumor_G2
8389	PDC	0.259378496	Tumor_G2
8390	SUCLG2	6.334809067	Tumor_G2
8391	SLC25A5	8.140446144	Tumor_G2
8392	UCP2	7.931352194	Tumor_G2
8393	SOD2	5.256411523	Tumor_G2
8394	NOX4	1.075561238	Tumor_G2
8395	DUOX1	7.303479424	Tumor_G2
8396	DUOXA1	6.94559434	Tumor_G2
8397	DUOX2	8.08795281	Tumor_G2
8398	DUOXA2	6.037920693	Tumor_G2

8399	NDUFA1	7.865640196	Tumor_G2
8400	NDUFA2	6.815502763	Tumor_G2
8401	NDUFA8	7.179459867	Tumor_G2
8402	NDUFB1	5.753317387	Tumor_G2
8403	NDUFB10	7.68624058	Tumor_G2
8404	NDUFB2	6.822755468	Tumor_G2
8405	NDUFB3	6.358714953	Tumor_G2
8406	NDUFB7	8.919185287	Tumor_G2
8407	CYC1	6.971853889	Tumor_G2
8408	ETFB	5.180573559	Tumor_G2
8409	UQCR10	7.788644099	Tumor_G2
8410	UQCRQ	7.542460565	Tumor_G2
8411	UQCC3	4.546425449	Tumor_G2
8412	COX15	4.76988447	Tumor_G2
8413	COX5A	7.176972879	Tumor_G2
8414	COX6B1	8.810978679	Tumor_G2
8415	COX6C	6.451715387	Tumor_G2
8416	COX7A2	6.852112082	Tumor_G2
8417	COX7B	6.115566333	Tumor_G2
8418	COX8A	9.507707098	Tumor_G2
8419	SCO1	3.517209186	Tumor_G2
8420	SURF1	6.646780473	Tumor_G2
8421	ATP6V1E1	6.937745899	Tumor_G2
8422	DLAT	4.796177745	Tumor_G2
8423	PDC	0	Tumor_G2
8424	SUCLG2	6.096034405	Tumor_G2
8425	SLC25A5	8.265969108	Tumor_G2
8426	UCP2	8.834838935	Tumor_G2
8427	SOD2	5.247876563	Tumor_G2
8428	NOX4	3.105405673	Tumor_G2
8429	DUOX1	7.068882259	Tumor_G2
8430	DUOXA1	6.985357826	Tumor_G2
8431	DUOX2	7.772755685	Tumor_G2
8432	DUOXA2	5.446776482	Tumor_G2
8433	NDUFA1	7.730826297	Tumor_G2
8434	NDUFA2	6.232140089	Tumor_G2
8435	NDUFA8	7.068791177	Tumor_G2
8436	NDUFB1	5.44849026	Tumor_G2
8437	NDUFB10	7.481611691	Tumor_G2
8438	NDUFB2	6.061792287	Tumor_G2

8439	NDUFB3	5.909576508	Tumor_G2
8440	NDUFB7	8.599375646	Tumor_G2
8441	CYC1	6.710282549	Tumor_G2
8442	ETFB	4.5088866	Tumor_G2
8443	UQCR10	7.307226112	Tumor_G2
8444	UQCRQ	7.081958152	Tumor_G2
8445	UQCC3	4.300092832	Tumor_G2
8446	COX15	5.002793085	Tumor_G2
8447	COX5A	7.007087383	Tumor_G2
8448	COX6B1	8.522605148	Tumor_G2
8449	COX6C	6.140810661	Tumor_G2
8450	COX7A2	6.682790097	Tumor_G2
8451	COX7B	5.948709603	Tumor_G2
8452	COX8A	9.206853824	Tumor_G2
8453	SCO1	3.376761402	Tumor_G2
8454	SURF1	6.632440485	Tumor_G2
8455	ATP6V1E1	6.649553798	Tumor_G2
8456	DLAT	5.238358563	Tumor_G2
8457	PDC	0.134585944	Tumor_G2
8458	SUCLG2	5.807269886	Tumor_G2
8459	SLC25A5	7.885098271	Tumor_G2
8460	UCP2	6.647238933	Tumor_G2
8461	SOD2	4.442987541	Tumor_G2
8462	NOX4	2.484421083	Tumor_G2
8463	DUOX1	6.358216164	Tumor_G2
8464	DUOXA1	6.480830553	Tumor_G2
8465	DUOX2	6.848863707	Tumor_G2
8466	DUOXA2	4.535844507	Tumor_G2
8467	NDUFA1	7.907922476	Tumor_G2
8468	NDUFA2	6.542845828	Tumor_G2
8469	NDUFA8	6.945122159	Tumor_G2
8470	NDUFB1	5.650669771	Tumor_G2
8471	NDUFB10	7.584534701	Tumor_G2
8472	NDUFB2	6.38049206	Tumor_G2
8473	NDUFB3	6.404298166	Tumor_G2
8474	NDUFB7	8.390913192	Tumor_G2
8475	CYC1	6.814872615	Tumor_G2
8476	ETFB	5.002484823	Tumor_G2
8477	UQCR10	7.582267057	Tumor_G2
8478	UQCRQ	7.043067095	Tumor_G2

8479	UQCC3	4.213979957	Tumor_G2
8480	COX15	5.151978046	Tumor_G2
8481	COX5A	7.135013079	Tumor_G2
8482	COX6B1	8.539170009	Tumor_G2
8483	COX6C	6.477194012	Tumor_G2
8484	COX7A2	6.736140183	Tumor_G2
8485	COX7B	5.947658677	Tumor_G2
8486	COX8A	9.40735626	Tumor_G2
8487	SCO1	3.518547714	Tumor_G2
8488	SURF1	6.736980654	Tumor_G2
8489	ATP6V1E1	7.122820832	Tumor_G2
8490	DLAT	5.10858289	Tumor_G2
8491	PDC	0.05428887	Tumor_G2
8492	SUCLG2	6.602584309	Tumor_G2
8493	SLC25A5	7.787258851	Tumor_G2
8494	UCP2	8.947642892	Tumor_G2
8495	SOD2	5.34506778	Tumor_G2
8496	NOX4	1.482387388	Tumor_G2
8497	DUOX1	6.613334878	Tumor_G2
8498	DUOXA1	6.679966364	Tumor_G2
8499	DUOX2	7.673319858	Tumor_G2
8500	DUOXA2	5.607870179	Tumor_G2
8501	NDUFA1	8.381814253	Tumor_G2
8502	NDUFA2	6.9997462	Tumor_G2
8503	NDUFA8	7.595024567	Tumor_G2
8504	NDUFB1	6.289363208	Tumor_G2
8505	NDUFB10	7.551978048	Tumor_G2
8506	NDUFB2	7.099724478	Tumor_G2
8507	NDUFB3	6.705813315	Tumor_G2
8508	NDUFB7	8.532164004	Tumor_G2
8509	CYC1	6.942361772	Tumor_G2
8510	ETFB	5.335619603	Tumor_G2
8511	UQCR10	6.955596716	Tumor_G2
8512	UQCRQ	7.580348883	Tumor_G2
8513	UQCC3	4.343938404	Tumor_G2
8514	COX15	5.408623557	Tumor_G2
8515	COX5A	7.339028953	Tumor_G2
8516	COX6B1	8.864354711	Tumor_G2
8517	COX6C	6.878455658	Tumor_G2
8518	COX7A2	7.11037376	Tumor_G2

8519	COX7B	6.275124728	Tumor_G2
8520	COX8A	9.786889003	Tumor_G2
8521	SCO1	4.188921207	Tumor_G2
8522	SURF1	7.208786802	Tumor_G2
8523	ATP6V1E1	6.241569355	Tumor_G2
8524	DLAT	5.301175682	Tumor_G2
8525	PDC	0.254474458	Tumor_G2
8526	SUCLG2	6.608952644	Tumor_G2
8527	SLC25A5	8.321512028	Tumor_G2
8528	UCP2	8.115346413	Tumor_G2
8529	SOD2	4.350087228	Tumor_G2
8530	NOX4	2.316982465	Tumor_G2
8531	DUOX1	7.513693731	Tumor_G2
8532	DUOXA1	7.28432022	Tumor_G2
8533	DUOX2	9.469746214	Tumor_G2
8534	DUOXA2	7.242563155	Tumor_G2
8535	NDUFA1	8.529348207	Tumor_G2
8536	NDUFA2	7.09489065	Tumor_G2
8537	NDUFA8	7.469586124	Tumor_G2
8538	NDUFB1	6.28268531	Tumor_G2
8539	NDUFB10	7.939232634	Tumor_G2
8540	NDUFB2	7.079248408	Tumor_G2
8541	NDUFB3	6.885649266	Tumor_G2
8542	NDUFB7	9.053160643	Tumor_G2
8543	CYC1	7.156047493	Tumor_G2
8544	ETFB	5.596640649	Tumor_G2
8545	UQCR10	8.101013422	Tumor_G2
8546	UQCRQ	7.709222975	Tumor_G2
8547	UQCC3	4.785565805	Tumor_G2
8548	COX15	5.249343519	Tumor_G2
8549	COX5A	7.476344033	Tumor_G2
8550	COX6B1	9.012322877	Tumor_G2
8551	COX6C	6.734330167	Tumor_G2
8552	COX7A2	7.380834219	Tumor_G2
8553	COX7B	6.619128382	Tumor_G2
8554	COX8A	9.931130902	Tumor_G2
8555	SCO1	3.474021888	Tumor_G2
8556	SURF1	7.05939625	Tumor_G2
8557	ATP6V1E1	7.039637429	Tumor_G2
8558	DLAT	5.362444456	Tumor_G2

8559	PDC	0	Tumor_G2
8560	SUCLG2	6.440632026	Tumor_G2
8561	SLC25A5	8.293543788	Tumor_G2
8562	UCP2	9.356762867	Tumor_G2
8563	SOD2	4.555547465	Tumor_G2
8564	NOX4	1.65611724	Tumor_G2
8565	DUOX1	7.203135863	Tumor_G2
8566	DUOXA1	7.167837525	Tumor_G2
8567	DUOX2	8.413063815	Tumor_G2
8568	DUOXA2	6.769502645	Tumor_G2
8569	NDUFA1	8.25054312	Tumor_G2
8570	NDUFA2	6.748960505	Tumor_G2
8571	NDUFA8	7.117991924	Tumor_G2
8572	NDUFB1	5.845190518	Tumor_G2
8573	NDUFB10	7.683748613	Tumor_G2
8574	NDUFB2	6.769830974	Tumor_G2
8575	NDUFB3	6.483607144	Tumor_G2
8576	NDUFB7	8.621882871	Tumor_G2
8577	CYC1	6.834716064	Tumor_G2
8578	ETFB	5.149577336	Tumor_G2
8579	UQCR10	7.688698191	Tumor_G2
8580	UQCRQ	7.398167621	Tumor_G2
8581	UQCC3	4.205653885	Tumor_G2
8582	COX15	4.808247928	Tumor_G2
8583	COX5A	7.490839751	Tumor_G2
8584	COX6B1	8.787783798	Tumor_G2
8585	COX6C	6.691961651	Tumor_G2
8586	COX7A2	6.929377896	Tumor_G2
8587	COX7B	6.221369364	Tumor_G2
8588	COX8A	9.607265024	Tumor_G2
8589	SCO1	3.302388173	Tumor_G2
8590	SURF1	6.319799577	Tumor_G2
8591	ATP6V1E1	7.206891564	Tumor_G2
8592	DLAT	5.184099458	Tumor_G2
8593	PDC	0.34253438	Tumor_G2
8594	SUCLG2	6.301527565	Tumor_G2
8595	SLC25A5	8.468081018	Tumor_G2
8596	UCP2	8.686880842	Tumor_G2
8597	SOD2	5.244616392	Tumor_G2
8598	NOX4	1.58206439	Tumor_G2

8599	DUOX1	6.487874355	Tumor_G2
8600	DUOXA1	6.359528146	Tumor_G2
8601	DUOX2	6.253843515	Tumor_G2
8602	DUOXA2	4.557635949	Tumor_G2
8603	NDUFA1	8.609465962	Tumor_G2
8604	NDUFA2	7.014895992	Tumor_G2
8605	NDUFA8	7.459287577	Tumor_G2
8606	NDUFB1	6.186199105	Tumor_G2
8607	NDUFB10	7.863361567	Tumor_G2
8608	NDUFB2	6.521836641	Tumor_G2
8609	NDUFB3	6.532116443	Tumor_G2
8610	NDUFB7	8.920613084	Tumor_G2
8611	CYC1	6.923190352	Tumor_G2
8612	ETFB	5.230968894	Tumor_G2
8613	UQCR10	8.09117446	Tumor_G2
8614	UQCRQ	7.615745026	Tumor_G2
8615	UQCC3	4.669130203	Tumor_G2
8616	COX15	4.786170072	Tumor_G2
8617	COX5A	7.462307965	Tumor_G2
8618	COX6B1	9.237943537	Tumor_G2
8619	COX6C	7.082704271	Tumor_G2
8620	COX7A2	7.122041431	Tumor_G2
8621	COX7B	6.384507712	Tumor_G2
8622	COX8A	9.460061061	Tumor_G2
8623	SCO1	3.194665879	Tumor_G2
8624	SURF1	6.652385434	Tumor_G2
8625	ATP6V1E1	7.018639361	Tumor_G2
8626	DLAT	4.816206053	Tumor_G2
8627	PDC	0	Tumor_G2
8628	SUCLG2	5.997571851	Tumor_G2
8629	SLC25A5	7.900979692	Tumor_G2
8630	UCP2	10.01325251	Tumor_G2
8631	SOD2	4.875280499	Tumor_G2
8632	NOX4	2.133204379	Tumor_G2
8633	DUOX1	6.654509183	Tumor_G2
8634	DUOXA1	6.87994748	Tumor_G2
8635	DUOX2	7.861921623	Tumor_G2
8636	DUOXA2	6.063177027	Tumor_G2
8637	NDUFA1	7.74286597	Tumor_G2
8638	NDUFA2	6.493806306	Tumor_G2

8639	NDUFA8	6.940127881	Tumor_G2
8640	NDUFB1	5.763467877	Tumor_G2
8641	NDUFB10	7.471215513	Tumor_G2
8642	NDUFB2	6.616202856	Tumor_G2
8643	NDUFB3	6.116121148	Tumor_G2
8644	NDUFB7	8.560514279	Tumor_G2
8645	CYC1	6.985182727	Tumor_G2
8646	ETFB	4.737097804	Tumor_G2
8647	UQCR10	7.489474963	Tumor_G2
8648	UQCRQ	7.140381213	Tumor_G2
8649	UQCC3	4.205898522	Tumor_G2
8650	COX15	4.821141039	Tumor_G2
8651	COX5A	7.251987048	Tumor_G2
8652	COX6B1	8.482586833	Tumor_G2
8653	COX6C	6.460312	Tumor_G2
8654	COX7A2	6.645329089	Tumor_G2
8655	COX7B	5.874403497	Tumor_G2
8656	COX8A	9.261992817	Tumor_G2
8657	SCO1	3.285188637	Tumor_G2
8658	SURF1	6.35386536	Tumor_G2
8659	ATP6V1E1	7.107847647	Tumor_G2
8660	DLAT	5.102325738	Tumor_G2
8661	PDC	0	Tumor_G2
8662	SUCLG2	6.469001103	Tumor_G2
8663	SLC25A5	8.358678578	Tumor_G2
8664	UCP2	7.59855686	Tumor_G2
8665	SOD2	4.771157445	Tumor_G2
8666	NOX4	1.557900378	Tumor_G2
8667	DUOX1	6.037517684	Tumor_G2
8668	DUOXA1	6.14998738	Tumor_G2
8669	DUOX2	5.94913879	Tumor_G2
8670	DUOXA2	4.006557349	Tumor_G2
8671	NDUFA1	8.400800436	Tumor_G2
8672	NDUFA2	7.116498353	Tumor_G2
8673	NDUFA8	7.466020808	Tumor_G2
8674	NDUFB1	6.186305374	Tumor_G2
8675	NDUFB10	7.742541357	Tumor_G2
8676	NDUFB2	6.88284948	Tumor_G2
8677	NDUFB3	6.479703196	Tumor_G2
8678	NDUFB7	9.011103374	Tumor_G2

8679	CYC1	7.027184036	Tumor_G2
8680	ETFB	5.690104437	Tumor_G2
8681	UQCR10	7.149140651	Tumor_G2
8682	UQCRQ	7.54322854	Tumor_G2
8683	UQCC3	5.201647814	Tumor_G2
8684	COX15	4.837619836	Tumor_G2
8685	COX5A	7.182444961	Tumor_G2
8686	COX6B1	9.017255076	Tumor_G2
8687	COX6C	6.75851421	Tumor_G2
8688	COX7A2	7.143002601	Tumor_G2
8689	COX7B	6.250865456	Tumor_G2
8690	COX8A	9.944886184	Tumor_G2
8691	SCO1	2.814161522	Tumor_G2
8692	SURF1	6.987483676	Tumor_G2
8693	ATP6V1E1	6.846495408	Tumor_G2
8694	DLAT	4.92111732	Tumor_G2
8695	PDC	0.119875591	Tumor_G2
8696	SUCLG2	6.193733769	Tumor_G2
8697	SLC25A5	7.507325581	Tumor_G2
8698	UCP2	8.531605211	Tumor_G2
8699	SOD2	4.190357704	Tumor_G2
8700	NOX4	1.995612903	Tumor_G2
8701	DUOX1	7.105471383	Tumor_G2
8702	DUOXA1	6.79819808	Tumor_G2
8703	DUOX2	8.045125744	Tumor_G2
8704	DUOXA2	6.851350451	Tumor_G2
8705	NDUFA1	8.094014644	Tumor_G2
8706	NDUFA2	6.999856317	Tumor_G2
8707	NDUFA8	7.058680876	Tumor_G2
8708	NDUFB1	5.721037977	Tumor_G2
8709	NDUFB10	7.607196256	Tumor_G2
8710	NDUFB2	7.032369485	Tumor_G2
8711	NDUFB3	6.156763512	Tumor_G2
8712	NDUFB7	8.853419825	Tumor_G2
8713	CYC1	6.95840388	Tumor_G2
8714	ETFB	5.490880759	Tumor_G2
8715	UQCR10	7.929814343	Tumor_G2
8716	UQCRQ	7.463026756	Tumor_G2
8717	UQCC3	4.433119575	Tumor_G2
8718	COX15	4.323201638	Tumor_G2

8719	COX5A	7.019695911	Tumor_G2
8720	COX6B1	9.096564108	Tumor_G2
8721	COX6C	6.530343093	Tumor_G2
8722	COX7A2	7.038593417	Tumor_G2
8723	COX7B	6.00558936	Tumor_G2
8724	COX8A	9.269138949	Tumor_G2
8725	SCO1	2.744391975	Tumor_G2
8726	SURF1	6.200133043	Tumor_G2
8727	ATP6V1E1	7.126048504	Tumor_G2
8728	DLAT	4.15497442	Tumor_G2
8729	PDC	0	Tumor_G2
8730	SUCLG2	5.7691991	Tumor_G2
8731	SLC25A5	8.0913033	Tumor_G2
8732	UCP2	8.09977305	Tumor_G2
8733	SOD2	3.998871149	Tumor_G2
8734	NOX4	2.160959643	Tumor_G2
8735	DUOX1	5.905711113	Tumor_G2
8736	DUOXA1	6.132180565	Tumor_G2
8737	DUOX2	5.849344343	Tumor_G2
8738	DUOXA2	4.241785065	Tumor_G2
8739	NDUFA1	8.293370336	Tumor_G2
8740	NDUFA2	6.908637457	Tumor_G2
8741	NDUFA8	7.119684124	Tumor_G2
8742	NDUFB1	5.915877967	Tumor_G2
8743	NDUFB10	7.445144605	Tumor_G2
8744	NDUFB2	7.017073771	Tumor_G2
8745	NDUFB3	6.600831175	Tumor_G2
8746	NDUFB7	8.793194321	Tumor_G2
8747	CYC1	6.982913046	Tumor_G2
8748	ETFB	5.043307647	Tumor_G2
8749	UQCR10	7.789441872	Tumor_G2
8750	UQCRQ	7.283963526	Tumor_G2
8751	UQCC3	4.373957828	Tumor_G2
8752	COX15	4.760039963	Tumor_G2
8753	COX5A	7.144118114	Tumor_G2
8754	COX6B1	8.856410325	Tumor_G2
8755	COX6C	6.601344963	Tumor_G2
8756	COX7A2	7.019396672	Tumor_G2
8757	COX7B	6.350854946	Tumor_G2
8758	COX8A	9.590898433	Tumor_G2

8759	SCO1	3.320701226	Tumor_G2
8760	SURF1	6.021970364	Tumor_G2
8761	ATP6V1E1	7.148641723	Tumor_G2
8762	DLAT	5.026819666	Tumor_G2
8763	PDC	0.119663923	Tumor_G2
8764	SUCLG2	6.017547004	Tumor_G2
8765	SLC25A5	8.210728955	Tumor_G2
8766	UCP2	8.156057073	Tumor_G2
8767	SOD2	4.826009386	Tumor_G2
8768	NOX4	1.523191951	Tumor_G2
8769	DUOX1	6.528407463	Tumor_G2
8770	DUOXA1	6.597229337	Tumor_G2
8771	DUOX2	6.318402518	Tumor_G2
8772	DUOXA2	4.722775488	Tumor_G2
8773	NDUFA1	8.71185314	Tumor_G2
8774	NDUFA2	7.093727523	Tumor_G2
8775	NDUFA8	7.425472627	Tumor_G2
8776	NDUFB1	6.056001118	Tumor_G2
8777	NDUFB10	7.660173306	Tumor_G2
8778	NDUFB2	7.113240608	Tumor_G2
8779	NDUFB3	6.838761093	Tumor_G2
8780	NDUFB7	9.001582833	Tumor_G2
8781	CYC1	7.311868612	Tumor_G2
8782	ETFB	5.21200579	Tumor_G2
8783	UQCR10	7.955500064	Tumor_G2
8784	UQCRQ	7.408887179	Tumor_G2
8785	UQCC3	4.838335785	Tumor_G2
8786	COX15	5.399001882	Tumor_G2
8787	COX5A	7.435605473	Tumor_G2
8788	COX6B1	8.873859222	Tumor_G2
8789	COX6C	6.945688081	Tumor_G2
8790	COX7A2	7.188913074	Tumor_G2
8791	COX7B	6.564253921	Tumor_G2
8792	COX8A	10.18472219	Tumor_G2
8793	SCO1	3.742340898	Tumor_G2
8794	SURF1	6.457308984	Tumor_G2
8795	ATP6V1E1	6.650694318	Tumor_G2
8796	DLAT	5.227204346	Tumor_G2
8797	PDC	0.439857739	Tumor_G2
8798	SUCLG2	6.37786816	Tumor_G2

8799	SLC25A5	8.102004744	Tumor_G2
8800	UCP2	6.092853152	Tumor_G2
8801	SOD2	4.780398927	Tumor_G2
8802	NOX4	1.040572641	Tumor_G2
8803	DUOX1	6.968310064	Tumor_G2
8804	DUOXA1	6.664299195	Tumor_G2
8805	DUOX2	7.175652481	Tumor_G2
8806	DUOXA2	6.229690894	Tumor_G2
8807	NDUFA1	8.274742882	Tumor_G2
8808	NDUFA2	7.186443239	Tumor_G2
8809	NDUFA8	7.150781872	Tumor_G2
8810	NDUFB1	5.954968759	Tumor_G2
8811	NDUFB10	7.628460315	Tumor_G2
8812	NDUFB2	7.122571878	Tumor_G2
8813	NDUFB3	6.751052941	Tumor_G2
8814	NDUFB7	8.675154713	Tumor_G2
8815	CYC1	7.353588105	Tumor_G2
8816	ETFB	5.171048325	Tumor_G2
8817	UQCR10	8.202434013	Tumor_G2
8818	UQCRQ	7.863343858	Tumor_G2
8819	UQCC3	4.670085634	Tumor_G2
8820	COX15	4.588358296	Tumor_G2
8821	COX5A	7.691351578	Tumor_G2
8822	COX6B1	8.92567918	Tumor_G2
8823	COX6C	6.621408299	Tumor_G2
8824	COX7A2	7.255706355	Tumor_G2
8825	COX7B	6.47000459	Tumor_G2
8826	COX8A	10.01346547	Tumor_G2
8827	SCO1	3.274499869	Tumor_G2
8828	SURF1	6.277787093	Tumor_G2
8829	ATP6V1E1	7.738213513	Tumor_G2
8830	DLAT	5.243746752	Tumor_G2
8831	PDC	0.251646627	Tumor_G2
8832	SUCLG2	6.473515165	Tumor_G2
8833	SLC25A5	8.25892072	Tumor_G2
8834	UCP2	8.200246039	Tumor_G2
8835	SOD2	4.576190282	Tumor_G2
8836	NOX4	1.457373402	Tumor_G2
8837	DUOX1	6.209296351	Tumor_G2
8838	DUOXA1	6.275761082	Tumor_G2

8839	DUOX2	5.293537134	Tumor_G2
8840	DUOXA2	3.873709413	Tumor_G2
8841	NDUFA1	8.373515863	Tumor_G2
8842	NDUFA2	7.029838112	Tumor_G2
8843	NDUFA8	7.481405883	Tumor_G2
8844	NDUFB1	6.273757191	Tumor_G2
8845	NDUFB10	7.772861376	Tumor_G2
8846	NDUFB2	7.145191527	Tumor_G2
8847	NDUFB3	6.89266756	Tumor_G2
8848	NDUFB7	8.870659397	Tumor_G2
8849	CYC1	7.216092523	Tumor_G2
8850	ETFB	5.317363124	Tumor_G2
8851	UQCR10	7.804833251	Tumor_G2
8852	UQCRQ	7.509018918	Tumor_G2
8853	UQCC3	4.695924748	Tumor_G2
8854	COX15	5.216824288	Tumor_G2
8855	COX5A	7.384069246	Tumor_G2
8856	COX6B1	8.973740516	Tumor_G2
8857	COX6C	6.732740775	Tumor_G2
8858	COX7A2	7.276166109	Tumor_G2
8859	COX7B	6.538001858	Tumor_G2
8860	COX8A	9.668241997	Tumor_G2
8861	SCO1	3.735662948	Tumor_G2
8862	SURF1	6.809288689	Tumor_G2
8863	ATP6V1E1	7.12240565	Tumor_G2
8864	DLAT	5.180475063	Tumor_G2
8865	PDC	0.157450628	Tumor_G2
8866	SUCLG2	6.580333225	Tumor_G2
8867	SLC25A5	8.65931413	Tumor_G2
8868	UCP2	7.56529739	Tumor_G2
8869	SOD2	5.000389938	Tumor_G2
8870	NOX4	1.848178726	Tumor_G2
8871	DUOX1	6.963098457	Tumor_G2
8872	DUOXA1	6.859686709	Tumor_G2
8873	DUOX2	7.060105081	Tumor_G2
8874	DUOXA2	5.676342884	Tumor_G2
8875	NDUFA1	8.26951815	Tumor_G2
8876	NDUFA2	7.083108426	Tumor_G2
8877	NDUFA8	7.127459724	Tumor_G2
8878	NDUFB1	6.371491005	Tumor_G2

8879	NDUFB10	7.768000439	Tumor_G2
8880	NDUFB2	6.296017038	Tumor_G2
8881	NDUFB3	5.529467544	Tumor_G2
8882	NDUFB7	8.985811898	Tumor_G2
8883	CYC1	6.524636863	Tumor_G2
8884	ETFB	4.682126693	Tumor_G2
8885	UQCR10	7.814481822	Tumor_G2
8886	UQCRQ	7.363023487	Tumor_G2
8887	UQCC3	4.663987473	Tumor_G2
8888	COX15	4.385898736	Tumor_G2
8889	COX5A	7.11703487	Tumor_G2
8890	COX6B1	9.176136331	Tumor_G2
8891	COX6C	6.778793213	Tumor_G2
8892	COX7A2	6.738389538	Tumor_G2
8893	COX7B	6.012114884	Tumor_G2
8894	COX8A	9.588116647	Tumor_G2
8895	SCO1	2.653635732	Tumor_G2
8896	SURF1	6.765128141	Tumor_G2
8897	ATP6V1E1	6.409923252	Tumor_G2
8898	DLAT	3.778534721	Tumor_G2
8899	PDC	0	Tumor_G2
8900	SUCLG2	5.324261973	Tumor_G2
8901	SLC25A5	6.853977484	Tumor_G2
8902	UCP2	8.102192814	Tumor_G2
8903	SOD2	3.797583175	Tumor_G2
8904	NOX4	1.792661543	Tumor_G2
8905	DUOX1	5.824035713	Tumor_G2
8906	DUOXA1	6.329223502	Tumor_G2
8907	DUOX2	6.129640971	Tumor_G2
8908	DUOXA2	4.962975948	Tumor_G2
8909	NDUFA1	8.401227026	Tumor_G2
8910	NDUFA2	7.270197966	Tumor_G2
8911	NDUFA8	7.409204609	Tumor_G2
8912	NDUFB1	6.33337496	Tumor_G2
8913	NDUFB10	7.919950909	Tumor_G2
8914	NDUFB2	6.933501353	Tumor_G2
8915	NDUFB3	6.075902875	Tumor_G2
8916	NDUFB7	9.736002294	Tumor_G2
8917	CYC1	6.863612559	Tumor_G2
8918	ETFB	5.84056182	Tumor_G2

8919	UQCR10	7.990754303	Tumor_G2
8920	UQCRQ	8.035273442	Tumor_G2
8921	UQCC3	5.146319136	Tumor_G2
8922	COX15	4.101108855	Tumor_G2
8923	COX5A	7.001127651	Tumor_G2
8924	COX6B1	9.224105725	Tumor_G2
8925	COX6C	6.681820008	Tumor_G2
8926	COX7A2	6.826983581	Tumor_G2
8927	COX7B	6.152998034	Tumor_G2
8928	COX8A	9.867078119	Tumor_G2
8929	SCO1	2.547188398	Tumor_G2
8930	SURF1	6.475998652	Tumor_G2
8931	ATP6V1E1	6.328710498	Tumor_G2
8932	DLAT	3.559170249	Tumor_G2
8933	PDC	0.096602658	Tumor_G2
8934	SUCLG2	5.051812387	Tumor_G2
8935	SLC25A5	6.572615827	Tumor_G2
8936	UCP2	8.145156677	Tumor_G2
8937	SOD2	3.063202049	Tumor_G2
8938	NOX4	1.113485896	Tumor_G2
8939	DUOX1	7.589125169	Tumor_G2
8940	DUOXA1	7.213514565	Tumor_G2
8941	DUOX2	8.141539165	Tumor_G2
8942	DUOXA2	7.050303787	Tumor_G2
8943	NDUFA1	8.000643742	Tumor_G2
8944	NDUFA2	6.584417597	Tumor_G2
8945	NDUFA8	6.863150533	Tumor_G2
8946	NDUFB1	5.652595286	Tumor_G2
8947	NDUFB10	7.235957382	Tumor_G2
8948	NDUFB2	6.265397826	Tumor_G2
8949	NDUFB3	6.305989426	Tumor_G2
8950	NDUFB7	8.415443092	Tumor_G2
8951	CYC1	6.894101583	Tumor_G2
8952	ETFB	4.779109904	Tumor_G2
8953	UQCR10	7.541852388	Tumor_G2
8954	UQCRQ	7.192581581	Tumor_G2
8955	UQCC3	4.245384259	Tumor_G2
8956	COX15	4.724126616	Tumor_G2
8957	COX5A	6.796800533	Tumor_G2
8958	COX6B1	8.475780487	Tumor_G2

8959	COX6C	6.381511607	Tumor_G2
8960	COX7A2	6.737742645	Tumor_G2
8961	COX7B	6.114880819	Tumor_G2
8962	COX8A	9.319250779	Tumor_G2
8963	SCO1	3.253879376	Tumor_G2
8964	SURF1	6.108395652	Tumor_G2
8965	ATP6V1E1	6.501470994	Tumor_G2
8966	DLAT	4.735889058	Tumor_G2
8967	PDC	0.057098749	Tumor_G2
8968	SUCLG2	5.725825657	Tumor_G2
8969	SLC25A5	7.890147883	Tumor_G2
8970	UCP2	7.542271225	Tumor_G2
8971	SOD2	5.389945625	Tumor_G2
8972	NOX4	1.53047872	Tumor_G2
8973	DUOX1	7.536895589	Tumor_G2
8974	DUOXA1	7.504451598	Tumor_G2
8975	DUOX2	7.802224557	Tumor_G2
8976	DUOXA2	5.717050532	Tumor_G2
8977	NDUFA1	7.58694453	Tumor_G2
8978	NDUFA2	6.58669265	Tumor_G2
8979	NDUFA8	7.176928457	Tumor_G2
8980	NDUFB1	5.445196513	Tumor_G2
8981	NDUFB10	7.352910862	Tumor_G2
8982	NDUFB2	6.48436461	Tumor_G2
8983	NDUFB3	6.009342255	Tumor_G2
8984	NDUFB7	8.661931689	Tumor_G2
8985	CYC1	6.700026569	Tumor_G2
8986	ETFB	5.263001561	Tumor_G2
8987	UQCR10	6.791544757	Tumor_G2
8988	UQCRQ	7.131088326	Tumor_G2
8989	UQCC3	4.484095144	Tumor_G2
8990	COX15	4.879319319	Tumor_G2
8991	COX5A	6.937973717	Tumor_G2
8992	COX6B1	8.632776193	Tumor_G2
8993	COX6C	6.273475604	Tumor_G2
8994	COX7A2	6.778126487	Tumor_G2
8995	COX7B	6.028454848	Tumor_G2
8996	COX8A	9.453902457	Tumor_G2
8997	SCO1	3.503715362	Tumor_G2
8998	SURF1	6.645404877	Tumor_G2

8999	ATP6V1E1	6.378963738	Tumor_G2
9000	DLAT	5.261852955	Tumor_G2
9001	PDC	0.267427461	Tumor_G2
9002	SUCLG2	6.08992233	Tumor_G2
9003	SLC25A5	8.217484653	Tumor_G2
9004	UCP2	9.050347481	Tumor_G2
9005	SOD2	4.972590608	Tumor_G2
9006	NOX4	2.80619918	Tumor_G2
9007	DUOX1	7.861396687	Tumor_G2
9008	DUOXA1	7.879018679	Tumor_G2
9009	DUOX2	9.362387161	Tumor_G2
9010	DUOXA2	6.7002081	Tumor_G2
9011	NDUFA1	7.751515953	Tumor_G2
9012	NDUFA2	6.558936273	Tumor_G2
9013	NDUFA8	7.052954645	Tumor_G2
9014	NDUFB1	5.459408256	Tumor_G2
9015	NDUFB10	7.582230014	Tumor_G2
9016	NDUFB2	5.94687777	Tumor_G2
9017	NDUFB3	6.05436755	Tumor_G2
9018	NDUFB7	8.135180469	Tumor_G2
9019	CYC1	6.628093737	Tumor_G2
9020	ETFB	4.765186673	Tumor_G2
9021	UQCR10	7.441724468	Tumor_G2
9022	UQCRQ	6.851644767	Tumor_G2
9023	UQCC3	4.380463499	Tumor_G2
9024	COX15	5.278329827	Tumor_G2
9025	COX5A	6.759735197	Tumor_G2
9026	COX6B1	8.506808251	Tumor_G2
9027	COX6C	6.265840627	Tumor_G2
9028	COX7A2	6.738649836	Tumor_G2
9029	COX7B	5.851020092	Tumor_G2
9030	COX8A	9.234371646	Tumor_G2
9031	SCO1	3.430152869	Tumor_G2
9032	SURF1	6.566534869	Tumor_G2
9033	ATP6V1E1	6.86405464	Tumor_G2
9034	DLAT	5.324551595	Tumor_G2
9035	PDC	0.706203668	Tumor_G2
9036	SUCLG2	6.253942599	Tumor_G2
9037	SLC25A5	7.601923115	Tumor_G2
9038	UCP2	8.702550556	Tumor_G2

9039	SOD2	4.618527062	Tumor_G2
9040	NOX4	3.706777456	Tumor_G2
9041	DUOX1	7.041487449	Tumor_G2
9042	DUOXA1	7.235064537	Tumor_G2
9043	DUOX2	8.339538557	Tumor_G2
9044	DUOXA2	6.037009342	Tumor_G2
9045	NDUFA1	8.326816785	Tumor_G2
9046	NDUFA2	7.026571252	Tumor_G2
9047	NDUFA8	6.950689422	Tumor_G2
9048	NDUFB1	6.122685026	Tumor_G2
9049	NDUFB10	7.782177636	Tumor_G2
9050	NDUFB2	6.225803262	Tumor_G2
9051	NDUFB3	5.869685117	Tumor_G2
9052	NDUFB7	8.862249693	Tumor_G2
9053	CYC1	6.26195362	Tumor_G2
9054	ETFB	4.830091318	Tumor_G2
9055	UQCR10	7.676762392	Tumor_G2
9056	UQCRQ	7.449198187	Tumor_G2
9057	UQCC3	4.730474023	Tumor_G2
9058	COX15	4.078573506	Tumor_G2
9059	COX5A	6.9665124	Tumor_G2
9060	COX6B1	8.954254439	Tumor_G2
9061	COX6C	6.728031817	Tumor_G2
9062	COX7A2	6.673974483	Tumor_G2
9063	COX7B	5.956761113	Tumor_G2
9064	COX8A	9.32924304	Tumor_G2
9065	SCO1	2.341891882	Tumor_G2
9066	SURF1	6.081929859	Tumor_G2
9067	ATP6V1E1	6.590741968	Tumor_G2
9068	DLAT	4.005355906	Tumor_G2
9069	PDC	0	Tumor_G2
9070	SUCLG2	5.139493776	Tumor_G2
9071	SLC25A5	7.071606624	Tumor_G2
9072	UCP2	8.020689597	Tumor_G2
9073	SOD2	4.482069101	Tumor_G2
9074	NOX4	2.983628044	Tumor_G2
9075	DUOX1	6.016545312	Tumor_G2
9076	DUOXA1	6.369146323	Tumor_G2
9077	DUOX2	6.12608291	Tumor_G2
9078	DUOXA2	4.597354096	Tumor_G2

9079	NDUFA1	7.935062945	Tumor_G2
9080	NDUFA2	6.641598317	Tumor_G2
9081	NDUFA8	6.901670844	Tumor_G2
9082	NDUFB1	5.873395814	Tumor_G2
9083	NDUFB10	7.37859083	Tumor_G2
9084	NDUFB2	6.541204576	Tumor_G2
9085	NDUFB3	6.386923535	Tumor_G2
9086	NDUFB7	8.634907667	Tumor_G2
9087	CYC1	6.967003473	Tumor_G2
9088	ETFB	4.864634479	Tumor_G2
9089	UQCR10	7.659855561	Tumor_G2
9090	UQCRQ	6.892948051	Tumor_G2
9091	UQCC3	4.224652405	Tumor_G2
9092	COX15	5.240781421	Tumor_G2
9093	COX5A	7.208286117	Tumor_G2
9094	COX6B1	8.39209331	Tumor_G2
9095	COX6C	6.36238904	Tumor_G2
9096	COX7A2	6.75813754	Tumor_G2
9097	COX7B	6.13836427	Tumor_G2
9098	COX8A	9.614426725	Tumor_G2
9099	SCO1	3.766524451	Tumor_G2
9100	SURF1	6.552613957	Tumor_G2
9101	ATP6V1E1	6.757882272	Tumor_G2
9102	DLAT	5.214924085	Tumor_G2
9103	PDC	0.07389739	Tumor_G2
9104	SUCLG2	6.38587096	Tumor_G2
9105	SLC25A5	8.473671736	Tumor_G2
9106	UCP2	7.901852624	Tumor_G2
9107	SOD2	5.340219504	Tumor_G2
9108	NOX4	0.778826406	Tumor_G2
9109	DUOX1	6.423874537	Tumor_G2
9110	DUOXA1	6.2288965	Tumor_G2
9111	DUOX2	6.888428861	Tumor_G2
9112	DUOXA2	5.549971845	Tumor_G2
9113	NDUFA1	7.938816488	Tumor_G2
9114	NDUFA2	6.629593874	Tumor_G2
9115	NDUFA8	7.081946899	Tumor_G2
9116	NDUFB1	5.68493008	Tumor_G2
9117	NDUFB10	7.289289292	Tumor_G2
9118	NDUFB2	6.917697685	Tumor_G2

9119	NDUFB3	6.482805987	Tumor_G2
9120	NDUFB7	8.506312048	Tumor_G2
9121	CYC1	6.914992604	Tumor_G2
9122	ETFB	5.190097655	Tumor_G2
9123	UQCR10	7.586790558	Tumor_G2
9124	UQCRQ	7.186424208	Tumor_G2
9125	UQCC3	4.393797778	Tumor_G2
9126	COX15	5.57348476	Tumor_G2
9127	COX5A	7.08393321	Tumor_G2
9128	COX6B1	8.313531077	Tumor_G2
9129	COX6C	6.612734103	Tumor_G2
9130	COX7A2	6.821081543	Tumor_G2
9131	COX7B	6.289132695	Tumor_G2
9132	COX8A	9.688172	Tumor_G2
9133	SCO1	3.782517254	Tumor_G2
9134	SURF1	6.825691918	Tumor_G2
9135	ATP6V1E1	6.797147169	Tumor_G2
9136	DLAT	5.718989242	Tumor_G2
9137	PDC	0.282920001	Tumor_G2
9138	SUCLG2	6.771773054	Tumor_G2
9139	SLC25A5	8.316314058	Tumor_G2
9140	UCP2	8.161302273	Tumor_G2
9141	SOD2	5.051279849	Tumor_G2
9142	NOX4	1.408009841	Tumor_G2
9143	DUOX1	7.564515925	Tumor_G2
9144	DUOXA1	7.104246991	Tumor_G2
9145	DUOX2	9.120245927	Tumor_G2
9146	DUOXA2	6.186732375	Tumor_G2
9147	NDUFA1	8.5943389	Tumor_G2
9148	NDUFA2	7.236165371	Tumor_G2
9149	NDUFA8	7.65106167	Tumor_G2
9150	NDUFB1	6.063278508	Tumor_G2
9151	NDUFB10	8.043048405	Tumor_G2
9152	NDUFB2	6.992810742	Tumor_G2
9153	NDUFB3	7.040600385	Tumor_G2
9154	NDUFB7	8.265387402	Tumor_G2
9155	CYC1	8.029428124	Tumor_G2
9156	ETFB	5.501285124	Tumor_G2
9157	UQCR10	8.270537071	Tumor_G2
9158	UQCRQ	7.585296194	Tumor_G2

9159	UQCC3	5.020122098	Tumor_G2
9160	COX15	5.151163345	Tumor_G2
9161	COX5A	8.024144038	Tumor_G2
9162	COX6B1	8.910446	Tumor_G2
9163	COX6C	6.740281259	Tumor_G2
9164	COX7A2	7.351292556	Tumor_G2
9165	COX7B	6.356614557	Tumor_G2
9166	COX8A	10.397324	Tumor_G2
9167	SCO1	3.842686576	Tumor_G2
9168	SURF1	6.805295252	Tumor_G2
9169	ATP6V1E1	7.060290497	Tumor_G2
9170	DLAT	5.434097741	Tumor_G2
9171	PDC	0	Tumor_G2
9172	SUCLG2	6.575877531	Tumor_G2
9173	SLC25A5	8.961965086	Tumor_G2
9174	UCP2	6.139119229	Tumor_G2
9175	SOD2	4.004935265	Tumor_G2
9176	NOX4	2.359386768	Tumor_G2
9177	DUOX1	7.787820942	Tumor_G2
9178	DUOXA1	7.123810921	Tumor_G2
9179	DUOX2	6.255249317	Tumor_G2
9180	DUOXA2	6.995374513	Tumor_G2
9181	NDUFA1	8.352602763	Tumor_G2
9182	NDUFA2	7.096638037	Tumor_G2
9183	NDUFA8	7.638220529	Tumor_G2
9184	NDUFB1	6.473216513	Tumor_G2
9185	NDUFB10	7.666103041	Tumor_G2
9186	NDUFB2	7.103697053	Tumor_G2
9187	NDUFB3	6.802644094	Tumor_G2
9188	NDUFB7	8.928798047	Tumor_G2
9189	CYC1	7.294574532	Tumor_G2
9190	ETFB	5.329909453	Tumor_G2
9191	UQCR10	7.296786413	Tumor_G2
9192	UQCRQ	7.795019325	Tumor_G2
9193	UQCC3	4.699831623	Tumor_G2
9194	COX15	4.957605456	Tumor_G2
9195	COX5A	7.62602532	Tumor_G2
9196	COX6B1	8.811045566	Tumor_G2
9197	COX6C	6.932502523	Tumor_G2
9198	COX7A2	7.139993312	Tumor_G2

9199	COX7B	6.582224493	Tumor_G2
9200	COX8A	10.03035075	Tumor_G2
9201	SCO1	3.961680033	Tumor_G2
9202	SURF1	6.82508182	Tumor_G2
9203	ATP6V1E1	5.933356361	Tumor_G2
9204	DLAT	5.567450188	Tumor_G2
9205	PDC	0.107859367	Tumor_G2
9206	SUCLG2	6.340926108	Tumor_G2
9207	SLC25A5	8.358500731	Tumor_G2
9208	UCP2	8.087902552	Tumor_G2
9209	SOD2	4.760928781	Tumor_G2
9210	NOX4	2.015653252	Tumor_G2
9211	DUOX1	7.367289024	Tumor_G2
9212	DUOXA1	7.29179278	Tumor_G2
9213	DUOX2	9.671667825	Tumor_G2
9214	DUOXA2	7.855097451	Tumor_G2
9215	NDUFA1	8.160094554	Tumor_G2
9216	NDUFA2	6.877954102	Tumor_G2
9217	NDUFA8	7.295017484	Tumor_G2
9218	NDUFB1	5.857507824	Tumor_G2
9219	NDUFB10	7.614712341	Tumor_G2
9220	NDUFB2	6.363967407	Tumor_G2
9221	NDUFB3	6.443588479	Tumor_G2
9222	NDUFB7	8.884801023	Tumor_G2
9223	CYC1	6.79334495	Tumor_G2
9224	ETFB	5.302792823	Tumor_G2
9225	UQCR10	7.733709481	Tumor_G2
9226	UQCRQ	7.433185562	Tumor_G2
9227	UQCC3	4.576322475	Tumor_G2
9228	COX15	4.838184812	Tumor_G2
9229	COX5A	7.180902314	Tumor_G2
9230	COX6B1	8.825310163	Tumor_G2
9231	COX6C	6.766926604	Tumor_G2
9232	COX7A2	6.849869495	Tumor_G2
9233	COX7B	6.202010214	Tumor_G2
9234	COX8A	9.381015916	Tumor_G2
9235	SCO1	3.52199259	Tumor_G2
9236	SURF1	6.51064067	Tumor_G2
9237	ATP6V1E1	7.031506955	Tumor_G2
9238	DLAT	5.050571445	Tumor_G2

9239	PDC	0.487679813	Tumor_G2
9240	SUCLG2	5.990646648	Tumor_G2
9241	SLC25A5	7.65000609	Tumor_G2
9242	UCP2	8.824075009	Tumor_G2
9243	SOD2	4.88503102	Tumor_G2
9244	NOX4	3.393887646	Tumor_G2
9245	DUOX1	5.782934296	Tumor_G2
9246	DUOXA1	6.62531424	Tumor_G2
9247	DUOX2	6.414114507	Tumor_G2
9248	DUOXA2	4.502804971	Tumor_G2
9249	NDUFA1	8.342098038	Tumor_G2
9250	NDUFA2	6.496541012	Tumor_G2
9251	NDUFA8	7.311838294	Tumor_G2
9252	NDUFB1	6.500368844	Tumor_G2
9253	NDUFB10	7.412315352	Tumor_G2
9254	NDUFB2	5.367449399	Tumor_G2
9255	NDUFB3	6.32930897	Tumor_G2
9256	NDUFB7	8.589918956	Tumor_G2
9257	CYC1	6.020151935	Tumor_G2
9258	ETFB	4.474404205	Tumor_G2
9259	UQCR10	8.061769764	Tumor_G2
9260	UQCRQ	7.736208846	Tumor_G2
9261	UQCC3	3.298679108	Tumor_G2
9262	COX15	4.218281736	Tumor_G2
9263	COX5A	6.803657409	Tumor_G2
9264	COX6B1	9.280046218	Tumor_G2
9265	COX6C	6.588576852	Tumor_G2
9266	COX7A2	7.097093837	Tumor_G2
9267	COX7B	6.877902236	Tumor_G2
9268	COX8A	8.305665468	Tumor_G2
9269	SCO1	2.57119848	Tumor_G2
9270	SURF1	5.491083859	Tumor_G2
9271	ATP6V1E1	6.281233117	Tumor_G2
9272	DLAT	4.616155693	Tumor_G2
9273	PDC	0	Tumor_G2
9274	SUCLG2	5.146788786	Tumor_G2
9275	SLC25A5	7.758879728	Tumor_G2
9276	UCP2	6.541879335	Tumor_G2
9277	SOD2	4.491903603	Tumor_G2
9278	NOX4	1.136931417	Tumor_G2

9279	DUOX1	6.029189005	Tumor_G2
9280	DUOXA1	5.770418568	Tumor_G2
9281	DUOX2	6.13643924	Tumor_G2
9282	DUOXA2	2.323941706	Tumor_G2
9283	NDUFA1	7.788378833	Tumor_G2
9284	NDUFA2	6.447680904	Tumor_G2
9285	NDUFA8	7.00372283	Tumor_G2
9286	NDUFB1	5.571605155	Tumor_G2
9287	NDUFB10	7.455530039	Tumor_G2
9288	NDUFB2	6.354301654	Tumor_G2
9289	NDUFB3	6.267193747	Tumor_G2
9290	NDUFB7	8.439075719	Tumor_G2
9291	CYC1	6.695016865	Tumor_G2
9292	ETFB	4.785415651	Tumor_G2
9293	UQCR10	7.400706081	Tumor_G2
9294	UQCRQ	7.067503619	Tumor_G2
9295	UQCC3	4.179520462	Tumor_G2
9296	COX15	5.206417079	Tumor_G2
9297	COX5A	7.060153773	Tumor_G2
9298	COX6B1	8.39686958	Tumor_G2
9299	COX6C	6.317033431	Tumor_G2
9300	COX7A2	6.889654438	Tumor_G2
9301	COX7B	5.838007244	Tumor_G2
9302	COX8A	9.259663613	Tumor_G2
9303	SCO1	3.451127897	Tumor_G2
9304	SURF1	6.248975137	Tumor_G2
9305	ATP6V1E1	7.179352825	Tumor_G2
9306	DLAT	5.489739695	Tumor_G2
9307	PDC	0.076238763	Tumor_G2
9308	SUCLG2	6.49231173	Tumor_G2
9309	SLC25A5	7.843250055	Tumor_G2
9310	UCP2	9.044628746	Tumor_G2
9311	SOD2	5.143028242	Tumor_G2
9312	NOX4	2.634331979	Tumor_G2
9313	DUOX1	6.585906541	Tumor_G2
9314	DUOXA1	6.785232904	Tumor_G2
9315	DUOX2	7.723160289	Tumor_G2
9316	DUOXA2	5.33844378	Tumor_G2
9317	NDUFA1	8.42095712	Tumor_G2
9318	NDUFA2	6.941064965	Tumor_G2

9319	NDUFA8	7.224860487	Tumor_G2
9320	NDUFB1	5.878373707	Tumor_G2
9321	NDUFB10	7.605164491	Tumor_G2
9322	NDUFB2	6.344179984	Tumor_G2
9323	NDUFB3	6.436100629	Tumor_G2
9324	NDUFB7	8.88499404	Tumor_G2
9325	CYC1	6.825164901	Tumor_G2
9326	ETFB	5.163851019	Tumor_G2
9327	UQCR10	8.015270836	Tumor_G2
9328	UQCRQ	7.356519804	Tumor_G2
9329	UQCC3	4.5329237	Tumor_G2
9330	COX15	4.977454759	Tumor_G2
9331	COX5A	7.17169434	Tumor_G2
9332	COX6B1	8.883831488	Tumor_G2
9333	COX6C	6.742905725	Tumor_G2
9334	COX7A2	6.958949046	Tumor_G2
9335	COX7B	6.147386962	Tumor_G2
9336	COX8A	9.569667764	Tumor_G2
9337	SCO1	3.179038854	Tumor_G2
9338	SURF1	6.561244498	Tumor_G2
9339	ATP6V1E1	6.910599929	Tumor_G2
9340	DLAT	5.00306611	Tumor_G2
9341	PDC	0	Tumor_G2
9342	SUCLG2	6.107659232	Tumor_G2
9343	SLC25A5	7.535987218	Tumor_G2
9344	UCP2	9.127995509	Tumor_G2
9345	SOD2	5.394706423	Tumor_G2
9346	NOX4	1.534967787	Tumor_G2
9347	DUOX1	6.79412916	Tumor_G2
9348	DUOXA1	6.86940928	Tumor_G2
9349	DUOX2	7.102612332	Tumor_G2
9350	DUOXA2	5.493448317	Tumor_G2
9351	NDUFA1	8.608909881	Tumor_G2
9352	NDUFA2	7.406223209	Tumor_G2
9353	NDUFA8	7.244896534	Tumor_G2
9354	NDUFB1	6.050459039	Tumor_G2
9355	NDUFB10	8.123662955	Tumor_G2
9356	NDUFB2	5.59162204	Tumor_G2
9357	NDUFB3	5.506353174	Tumor_G2
9358	NDUFB7	9.30633543	Tumor_G2

9359	CYC1	6.288069635	Tumor_G2
9360	ETFB	5.393139679	Tumor_G2
9361	UQCR10	8.079061855	Tumor_G2
9362	UQCRQ	7.67444826	Tumor_G2
9363	UQCC3	5.191470781	Tumor_G2
9364	COX15	4.549749075	Tumor_G2
9365	COX5A	7.181113013	Tumor_G2
9366	COX6B1	9.43308085	Tumor_G2
9367	COX6C	7.168790584	Tumor_G2
9368	COX7A2	6.752847678	Tumor_G2
9369	COX7B	5.907411378	Tumor_G2
9370	COX8A	9.285822965	Tumor_G2
9371	SCO1	2.570765809	Tumor_G2
9372	SURF1	6.256106329	Tumor_G2
9373	ATP6V1E1	6.128443207	Tumor_G2
9374	DLAT	4.04128758	Tumor_G2
9375	PDC	0.180353449	Tumor_G2
9376	SUCLG2	4.930775812	Tumor_G2
9377	SLC25A5	5.909473408	Tumor_G2
9378	UCP2	7.165038814	Tumor_G2
9379	SOD2	3.55647155	Tumor_G2
9380	NOX4	2.912628192	Tumor_G2
9381	DUOX1	5.537406899	Tumor_G2
9382	DUOXA1	6.453647328	Tumor_G2
9383	DUOX2	5.780634181	Tumor_G2
9384	DUOXA2	5.048614217	Tumor_G2
9385	NDUFA1	8.228429861	Tumor_G2
9386	NDUFA2	6.769465975	Tumor_G2
9387	NDUFA8	7.18571849	Tumor_G2
9388	NDUFB1	5.962010877	Tumor_G2
9389	NDUFB10	7.456205443	Tumor_G2
9390	NDUFB2	6.658533031	Tumor_G2
9391	NDUFB3	6.576702801	Tumor_G2
9392	NDUFB7	8.827392462	Tumor_G2
9393	CYC1	6.760427604	Tumor_G2
9394	ETFB	5.108324674	Tumor_G2
9395	UQCR10	7.664632986	Tumor_G2
9396	UQCRQ	7.34021803	Tumor_G2
9397	UQCC3	4.427428662	Tumor_G2
9398	COX15	4.560138783	Tumor_G2

9399	COX5A	6.947681753	Tumor_G2
9400	COX6B1	8.597558522	Tumor_G2
9401	COX6C	6.361124941	Tumor_G2
9402	COX7A2	6.986539028	Tumor_G2
9403	COX7B	6.28108372	Tumor_G2
9404	COX8A	9.568112806	Tumor_G2
9405	SCO1	3.278578591	Tumor_G2
9406	SURF1	6.407763714	Tumor_G2
9407	ATP6V1E1	6.875823746	Tumor_G2
9408	DLAT	4.873256631	Tumor_G2
9409	PDC	0.156509488	Tumor_G2
9410	SUCLG2	5.508539337	Tumor_G2
9411	SLC25A5	8.026300281	Tumor_G2
9412	UCP2	7.424422331	Tumor_G2
9413	SOD2	4.844276811	Tumor_G2
9414	NOX4	2.013319181	Tumor_G2
9415	DUOX1	7.726641538	Tumor_G2
9416	DUOXA1	7.783845584	Tumor_G2
9417	DUOX2	7.99210974	Tumor_G2
9418	DUOXA2	5.43872851	Tumor_G2
9419	NDUFA1	7.858222399	Tumor_G2
9420	NDUFA2	6.657865215	Tumor_G2
9421	NDUFA8	7.022370878	Tumor_G2
9422	NDUFB1	5.589236788	Tumor_G2
9423	NDUFB10	7.076720709	Tumor_G2
9424	NDUFB2	6.503810809	Tumor_G2
9425	NDUFB3	6.259340418	Tumor_G2
9426	NDUFB7	8.146078748	Tumor_G2
9427	CYC1	6.775760237	Tumor_G2
9428	ETFB	5.174351971	Tumor_G2
9429	UQCR10	6.789222347	Tumor_G2
9430	UQCRQ	6.867387325	Tumor_G2
9431	UQCC3	3.701757628	Tumor_G2
9432	COX15	5.25780161	Tumor_G2
9433	COX5A	6.627471612	Tumor_G2
9434	COX6B1	8.372469925	Tumor_G2
9435	COX6C	6.381437552	Tumor_G2
9436	COX7A2	6.785508297	Tumor_G2
9437	COX7B	5.910984497	Tumor_G2
9438	COX8A	9.042120819	Tumor_G2

9439	SCO1	3.994732416	Tumor_G2
9440	SURF1	6.593704792	Tumor_G2
9441	ATP6V1E1	6.144268763	Tumor_G2
9442	DLAT	5.455020975	Tumor_G2
9443	PDC	0.389393666	Tumor_G2
9444	SUCLG2	6.732289089	Tumor_G2
9445	SLC25A5	8.243703811	Tumor_G2
9446	UCP2	6.128719025	Tumor_G2
9447	SOD2	4.801925237	Tumor_G2
9448	NOX4	1.63681598	Tumor_G2
9449	DUOX1	7.872642353	Tumor_G2
9450	DUOXA1	7.448966193	Tumor_G2
9451	DUOX2	10.42453257	Tumor_G2
9452	DUOXA2	6.930425633	Tumor_G2
9453	NDUFA1	7.735354015	Tumor_G2
9454	NDUFA2	6.5886067	Tumor_G2
9455	NDUFA8	7.047525861	Tumor_G2
9456	NDUFB1	5.44370558	Tumor_G2
9457	NDUFB10	7.349864469	Tumor_G2
9458	NDUFB2	6.105641498	Tumor_G2
9459	NDUFB3	6.116876213	Tumor_G2
9460	NDUFB7	8.504400783	Tumor_G2
9461	CYC1	6.447167955	Tumor_G2
9462	ETFB	4.985292067	Tumor_G2
9463	UQCR10	7.482884511	Tumor_G2
9464	UQCRQ	6.984499413	Tumor_G2
9465	UQCC3	4.133339597	Tumor_G2
9466	COX15	4.643110305	Tumor_G2
9467	COX5A	6.91031643	Tumor_G2
9468	COX6B1	8.72589038	Tumor_G2
9469	COX6C	6.098860705	Tumor_G2
9470	COX7A2	6.742448885	Tumor_G2
9471	COX7B	5.921361826	Tumor_G2
9472	COX8A	9.183348476	Tumor_G2
9473	SCO1	3.153090873	Tumor_G2
9474	SURF1	6.643030899	Tumor_G2
9475	ATP6V1E1	7.030382651	Tumor_G2
9476	DLAT	5.080397108	Tumor_G2
9477	PDC	0.201050865	Tumor_G2
9478	SUCLG2	5.957242731	Tumor_G2

9479	SLC25A5	7.817979209	Tumor_G2
9480	UCP2	9.589514024	Tumor_G2
9481	SOD2	5.100400532	Tumor_G2
9482	NOX4	3.66970067	Tumor_G2
9483	DUOX1	6.415690658	Tumor_G2
9484	DUOXA1	7.040223452	Tumor_G2
9485	DUOX2	7.77357117	Tumor_G2
9486	DUOXA2	5.389152012	Tumor_G2
9487	NDUFA1	7.905856234	Tumor_G2
9488	NDUFA2	6.700875492	Tumor_G2
9489	NDUFA8	6.908484541	Tumor_G2
9490	NDUFB1	5.705569771	Tumor_G2
9491	NDUFB10	7.356444339	Tumor_G2
9492	NDUFB2	6.146607337	Tumor_G2
9493	NDUFB3	6.368106515	Tumor_G2
9494	NDUFB7	8.603535414	Tumor_G2
9495	CYC1	6.645195092	Tumor_G2
9496	ETFB	4.947405467	Tumor_G2
9497	UQCR10	7.482549789	Tumor_G2
9498	UQCRQ	6.995358048	Tumor_G2
9499	UQCC3	4.567486133	Tumor_G2
9500	COX15	4.798033507	Tumor_G2
9501	COX5A	6.779541857	Tumor_G2
9502	COX6B1	8.533106368	Tumor_G2
9503	COX6C	6.322565192	Tumor_G2
9504	COX7A2	6.767516101	Tumor_G2
9505	COX7B	5.871200562	Tumor_G2
9506	COX8A	9.160868862	Tumor_G2
9507	SCO1	3.246291576	Tumor_G2
9508	SURF1	6.261517823	Tumor_G2
9509	ATP6V1E1	6.970964754	Tumor_G2
9510	DLAT	4.920588145	Tumor_G2
9511	PDC	0.285309417	Tumor_G2
9512	SUCLG2	5.821377598	Tumor_G2
9513	SLC25A5	7.9956518	Tumor_G2
9514	UCP2	9.113466641	Tumor_G2
9515	SOD2	4.898010113	Tumor_G2
9516	NOX4	3.044881517	Tumor_G2
9517	DUOX1	6.596643281	Tumor_G2
9518	DUOXA1	6.665132441	Tumor_G2

9519	DUOX2	7.580725527	Tumor_G2
9520	DUOXA2	5.621825611	Tumor_G2
9521	NDUFA1	8.342675758	Tumor_G2
9522	NDUFA2	7.189420522	Tumor_G2
9523	NDUFA8	7.390588491	Tumor_G2
9524	NDUFB1	6.269040262	Tumor_G2
9525	NDUFB10	7.734684746	Tumor_G2
9526	NDUFB2	6.834123145	Tumor_G2
9527	NDUFB3	6.570149897	Tumor_G2
9528	NDUFB7	9.260059302	Tumor_G2
9529	CYC1	6.892777725	Tumor_G2
9530	ETFB	5.313619808	Tumor_G2
9531	UQCR10	7.851328946	Tumor_G2
9532	UQCRQ	7.567727975	Tumor_G2
9533	UQCC3	4.890917426	Tumor_G2
9534	COX15	4.626211604	Tumor_G2
9535	COX5A	7.066648979	Tumor_G2
9536	COX6B1	9.084727647	Tumor_G2
9537	COX6C	6.764598491	Tumor_G2
9538	COX7A2	6.876710934	Tumor_G2
9539	COX7B	6.310049591	Tumor_G2
9540	COX8A	9.937018655	Tumor_G2
9541	SCO1	2.83892883	Tumor_G2
9542	SURF1	6.370194175	Tumor_G2
9543	ATP6V1E1	6.816772193	Tumor_G2
9544	DLAT	4.395004079	Tumor_G2
9545	PDC	0.147980358	Tumor_G2
9546	SUCLG2	5.43883749	Tumor_G2
9547	SLC25A5	7.697014816	Tumor_G2
9548	UCP2	8.543804101	Tumor_G2
9549	SOD2	4.729648519	Tumor_G2
9550	NOX4	2.057796021	Tumor_G2
9551	DUOX1	6.404211499	Tumor_G2
9552	DUOXA1	6.371929277	Tumor_G2
9553	DUOX2	6.530100448	Tumor_G2
9554	DUOXA2	5.207178107	Tumor_G2
9555	NDUFA1	8.344885767	Tumor_G2
9556	NDUFA2	7.081499721	Tumor_G2
9557	NDUFA8	7.418462857	Tumor_G2
9558	NDUFB1	6.013362501	Tumor_G2

9559	NDUFB10	7.851978982	Tumor_G2
9560	NDUFB2	7.287591726	Tumor_G2
9561	NDUFB3	6.684702357	Tumor_G2
9562	NDUFB7	9.309967403	Tumor_G2
9563	CYC1	7.333372087	Tumor_G2
9564	ETFB	5.741932938	Tumor_G2
9565	UQCR10	7.939728845	Tumor_G2
9566	UQCRQ	7.51005591	Tumor_G2
9567	UQCC3	4.916575813	Tumor_G2
9568	COX15	4.90602042	Tumor_G2
9569	COX5A	7.022339837	Tumor_G2
9570	COX6B1	9.173971826	Tumor_G2
9571	COX6C	6.717541085	Tumor_G2
9572	COX7A2	7.203387463	Tumor_G2
9573	COX7B	6.223264485	Tumor_G2
9574	COX8A	9.57140362	Tumor_G2
9575	SCO1	3.300738378	Tumor_G2
9576	SURF1	7.030302281	Tumor_G2
9577	ATP6V1E1	7.012750923	Tumor_G2
9578	DLAT	4.556181683	Tumor_G2
9579	PDC	0	Tumor_G2
9580	SUCLG2	6.057996596	Tumor_G2
9581	SLC25A5	8.132215458	Tumor_G2
9582	UCP2	7.967440023	Tumor_G2
9583	SOD2	4.40276385	Tumor_G2
9584	NOX4	2.113485724	Tumor_G2
9585	DUOX1	7.233112627	Tumor_G2
9586	DUOXA1	7.438336641	Tumor_G2
9587	DUOX2	8.076083888	Tumor_G2
9588	DUOXA2	6.361148427	Tumor_G2
9589	NDUFA1	8.768334393	Tumor_G2
9590	NDUFA2	7.349310968	Tumor_G2
9591	NDUFA8	7.486254317	Tumor_G2
9592	NDUFB1	6.340753376	Tumor_G2
9593	NDUFB10	8.052683361	Tumor_G2
9594	NDUFB2	7.828468265	Tumor_G2
9595	NDUFB3	6.97189462	Tumor_G2
9596	NDUFB7	9.231225807	Tumor_G2
9597	CYC1	7.617760182	Tumor_G2
9598	ETFB	5.345071771	Tumor_G2

9599	UQCR10	8.34017299	Tumor_G2
9600	UQCRQ	7.847130435	Tumor_G2
9601	UQCC3	4.913660179	Tumor_G2
9602	COX15	4.788119111	Tumor_G2
9603	COX5A	7.93737729	Tumor_G2
9604	COX6B1	9.424270909	Tumor_G2
9605	COX6C	7.141373851	Tumor_G2
9606	COX7A2	7.6310836	Tumor_G2
9607	COX7B	6.95836278	Tumor_G2
9608	COX8A	10.18841306	Tumor_G2
9609	SCO1	3.375811858	Tumor_G2
9610	SURF1	6.27533763	Tumor_G2
9611	ATP6V1E1	7.77325877	Tumor_G2
9612	DLAT	5.426699501	Tumor_G2
9613	PDC	0.047176884	Tumor_G2
9614	SUCLG2	6.550973363	Tumor_G2
9615	SLC25A5	8.714501192	Tumor_G2
9616	UCP2	8.555638665	Tumor_G2
9617	SOD2	4.012412189	Tumor_G2
9618	NOX4	1.856479409	Tumor_G2
9619	DUOX1	6.235442158	Tumor_G2
9620	DUOXA1	6.177915071	Tumor_G2
9621	DUOX2	6.082750528	Tumor_G2
9622	DUOXA2	4.246800161	Tumor_G2
9623	NDUFA1	8.456943863	Tumor_G2
9624	NDUFA2	7.177039993	Tumor_G2
9625	NDUFA8	7.222154227	Tumor_G2
9626	NDUFB1	6.396206701	Tumor_G2
9627	NDUFB10	7.682088224	Tumor_G2
9628	NDUFB2	7.350292532	Tumor_G2
9629	NDUFB3	6.896244575	Tumor_G2
9630	NDUFB7	8.858583733	Tumor_G2
9631	CYC1	7.186343279	Tumor_G2
9632	ETFB	5.256591873	Tumor_G2
9633	UQCR10	8.146728433	Tumor_G2
9634	UQCRQ	7.493195019	Tumor_G2
9635	UQCC3	4.716993331	Tumor_G2
9636	COX15	5.181399817	Tumor_G2
9637	COX5A	7.365262466	Tumor_G2
9638	COX6B1	8.793390471	Tumor_G2

9639	COX6C	6.81001779	Tumor_G2
9640	COX7A2	7.328038589	Tumor_G2
9641	COX7B	6.501474571	Tumor_G2
9642	COX8A	10.1586921	Tumor_G2
9643	SCO1	4.392829372	Tumor_G2
9644	SURF1	7.016807465	Tumor_G2
9645	ATP6V1E1	7.006363292	Tumor_G2
9646	DLAT	5.385145092	Tumor_G2
9647	PDC	0.367869687	Tumor_G2
9648	SUCLG2	6.394093839	Tumor_G2
9649	SLC25A5	8.399206771	Tumor_G2
9650	UCP2	8.129596654	Tumor_G2
9651	SOD2	4.704031991	Tumor_G2
9652	NOX4	1.001820818	Tumor_G2
9653	DUOX1	7.237758606	Tumor_G2
9654	DUOXA1	7.10828148	Tumor_G2
9655	DUOX2	8.128787014	Tumor_G2
9656	DUOXA2	6.292354869	Tumor_G2
9657	NDUFA1	8.265079404	Tumor_G2
9658	NDUFA2	6.871406409	Tumor_G2
9659	NDUFA8	7.074216937	Tumor_G2
9660	NDUFB1	5.898998015	Tumor_G2
9661	NDUFB10	7.568891061	Tumor_G2
9662	NDUFB2	5.939550704	Tumor_G2
9663	NDUFB3	5.472606794	Tumor_G2
9664	NDUFB7	8.687310672	Tumor_G2
9665	CYC1	6.388142871	Tumor_G2
9666	ETFB	4.755424603	Tumor_G2
9667	UQCR10	6.67537133	Tumor_G2
9668	UQCRQ	7.129140018	Tumor_G2
9669	UQCC3	4.426944618	Tumor_G2
9670	COX15	4.425608719	Tumor_G2
9671	COX5A	6.964367246	Tumor_G2
9672	COX6B1	8.892739916	Tumor_G2
9673	COX6C	6.479594815	Tumor_G2
9674	COX7A2	6.611469126	Tumor_G2
9675	COX7B	5.827337614	Tumor_G2
9676	COX8A	9.202910891	Tumor_G2
9677	SCO1	2.381447104	Tumor_G2
9678	SURF1	6.283927582	Tumor_G2

9679	ATP6V1E1	5.764740981	Tumor_G2
9680	DLAT	3.992434225	Tumor_G2
9681	PDC	0.128436824	Tumor_G2
9682	SUCLG2	5.284913215	Tumor_G2
9683	SLC25A5	6.813765464	Tumor_G2
9684	UCP2	8.638477725	Tumor_G2
9685	SOD2	3.495755797	Tumor_G2
9686	NOX4	2.517099902	Tumor_G2
9687	DUOX1	5.483140216	Tumor_G2
9688	DUOXA1	5.751438495	Tumor_G2
9689	DUOX2	6.720263306	Tumor_G2
9690	DUOXA2	4.399965196	Tumor_G2
9691	NDUFA1	8.181428084	Tumor_G2
9692	NDUFA2	7.085162729	Tumor_G2
9693	NDUFA8	7.2831837	Tumor_G2
9694	NDUFB1	6.074525145	Tumor_G2
9695	NDUFB10	7.80796548	Tumor_G2
9696	NDUFB2	6.565322076	Tumor_G2
9697	NDUFB3	6.257008291	Tumor_G2
9698	NDUFB7	8.977760224	Tumor_G2
9699	CYC1	6.788102764	Tumor_G2
9700	ETFB	5.022644719	Tumor_G2
9701	UQCR10	7.629430119	Tumor_G2
9702	UQCRQ	7.470548196	Tumor_G2
9703	UQCC3	4.601962297	Tumor_G2
9704	COX15	4.464544815	Tumor_G2
9705	COX5A	7.202038597	Tumor_G2
9706	COX6B1	8.925453721	Tumor_G2
9707	COX6C	6.539973906	Tumor_G2
9708	COX7A2	7.038887203	Tumor_G2
9709	COX7B	6.26935351	Tumor_G2
9710	COX8A	9.42744512	Tumor_G2
9711	SCO1	2.909323469	Tumor_G2
9712	SURF1	6.385427264	Tumor_G2
9713	ATP6V1E1	6.690278016	Tumor_G2
9714	DLAT	4.608750615	Tumor_G2
9715	PDC	0.305462448	Tumor_G2
9716	SUCLG2	5.533552652	Tumor_G2
9717	SLC25A5	7.737643153	Tumor_G2
9718	UCP2	7.937085144	Tumor_G2

9719	SOD2	4.7009266	Tumor_G2
9720	NOX4	3.24450342	Tumor_G2
9721	DUOX1	6.373796812	Tumor_G2
9722	DUOXA1	6.624972623	Tumor_G2
9723	DUOX2	6.850335119	Tumor_G2
9724	DUOXA2	5.482328134	Tumor_G2
9725	NDUFA1	8.071956218	Tumor_G2
9726	NDUFA2	6.828633527	Tumor_G2
9727	NDUFA8	7.057169095	Tumor_G2
9728	NDUFB1	5.640799006	Tumor_G2
9729	NDUFB10	7.481549782	Tumor_G2
9730	NDUFB2	6.610202602	Tumor_G2
9731	NDUFB3	6.513176269	Tumor_G2
9732	NDUFB7	8.518871734	Tumor_G2
9733	CYC1	6.877209553	Tumor_G2
9734	ETFB	4.760992503	Tumor_G2
9735	UQCR10	7.648815433	Tumor_G2
9736	UQCRQ	7.264930849	Tumor_G2
9737	UQCC3	4.110308734	Tumor_G2
9738	COX15	4.762894285	Tumor_G2
9739	COX5A	7.146059638	Tumor_G2
9740	COX6B1	8.699557433	Tumor_G2
9741	COX6C	6.382722258	Tumor_G2
9742	COX7A2	6.969411349	Tumor_G2
9743	COX7B	6.267352539	Tumor_G2
9744	COX8A	9.473887763	Tumor_G2
9745	SCO1	3.361038444	Tumor_G2
9746	SURF1	6.151338044	Tumor_G2
9747	ATP6V1E1	6.880830241	Tumor_G2
9748	DLAT	5.059782899	Tumor_G2
9749	PDC	0.172570345	Tumor_G2
9750	SUCLG2	5.894215773	Tumor_G2
9751	SLC25A5	8.07241607	Tumor_G2
9752	UCP2	8.684382298	Tumor_G2
9753	SOD2	4.913501079	Tumor_G2
9754	NOX4	2.402952357	Tumor_G2
9755	DUOX1	6.565913887	Tumor_G2
9756	DUOXA1	6.940632792	Tumor_G2
9757	DUOX2	6.562349614	Tumor_G2
9758	DUOXA2	4.740730132	Tumor_G2

9759	NDUFA1	8.465935967	Tumor_G2
9760	NDUFA2	7.022421235	Tumor_G2
9761	NDUFA8	7.500994314	Tumor_G2
9762	NDUFB1	6.285550929	Tumor_G2
9763	NDUFB10	7.831928114	Tumor_G2
9764	NDUFB2	7.027065938	Tumor_G2
9765	NDUFB3	6.740745636	Tumor_G2
9766	NDUFB7	9.100054249	Tumor_G2
9767	CYC1	7.286926692	Tumor_G2
9768	ETFB	5.548423923	Tumor_G2
9769	UQCR10	7.878035435	Tumor_G2
9770	UQCRQ	7.836528215	Tumor_G2
9771	UQCC3	4.918082407	Tumor_G2
9772	COX15	5.201691098	Tumor_G2
9773	COX5A	7.483365399	Tumor_G2
9774	COX6B1	8.852739675	Tumor_G2
9775	COX6C	6.927348182	Tumor_G2
9776	COX7A2	7.194934219	Tumor_G2
9777	COX7B	6.495133607	Tumor_G2
9778	COX8A	10.26624763	Tumor_G2
9779	SCO1	3.635707488	Tumor_G2
9780	SURF1	7.154763887	Tumor_G2
9781	ATP6V1E1	6.807999939	Tumor_G2
9782	DLAT	5.059913283	Tumor_G2
9783	PDC	0.528085843	Tumor_G2
9784	SUCLG2	6.577643275	Tumor_G2
9785	SLC25A5	8.146380382	Tumor_G2
9786	UCP2	8.067451544	Tumor_G2
9787	SOD2	4.107000732	Tumor_G2
9788	NOX4	1.423785119	Tumor_G2
9789	DUOX1	8.360964064	Tumor_G2
9790	DUOXA1	7.702687024	Tumor_G2
9791	DUOX2	10.38982204	Tumor_G2
9792	DUOXA2	8.359734733	Tumor_G2
9793	NDUFA1	8.087163601	Tumor_G2
9794	NDUFA2	7.007676809	Tumor_G2
9795	NDUFA8	7.358041554	Tumor_G2
9796	NDUFB1	5.876754784	Tumor_G2
9797	NDUFB10	7.995255723	Tumor_G2
9798	NDUFB2	6.272887035	Tumor_G2

9799	NDUFB3	6.234675367	Tumor_G2
9800	NDUFB7	8.831444503	Tumor_G2
9801	CYC1	6.917281092	Tumor_G2
9802	ETFB	5.179973899	Tumor_G2
9803	UQCR10	7.065830357	Tumor_G2
9804	UQCRQ	7.506609057	Tumor_G2
9805	UQCC3	4.345613054	Tumor_G2
9806	COX15	4.625058707	Tumor_G2
9807	COX5A	7.227149753	Tumor_G2
9808	COX6B1	8.847740314	Tumor_G2
9809	COX6C	6.683201267	Tumor_G2
9810	COX7A2	6.904778814	Tumor_G2
9811	COX7B	6.220517334	Tumor_G2
9812	COX8A	9.601085176	Tumor_G2
9813	SCO1	3.094776227	Tumor_G2
9814	SURF1	6.248830457	Tumor_G2
9815	ATP6V1E1	6.162222558	Tumor_G2
9816	DLAT	4.717816449	Tumor_G2
9817	PDC	0.222113558	Tumor_G2
9818	SUCLG2	5.936024618	Tumor_G2
9819	SLC25A5	7.96586624	Tumor_G2
9820	UCP2	8.122228788	Tumor_G2
9821	SOD2	4.767632505	Tumor_G2
9822	NOX4	3.530627905	Tumor_G2
9823	DUOX1	6.697569738	Tumor_G2
9824	DUOXA1	6.824205262	Tumor_G2
9825	DUOX2	7.40217112	Tumor_G2
9826	DUOXA2	4.735139546	Tumor_G2
9827	NDUFA1	7.312822368	Tumor_G2
9828	NDUFA2	6.307955525	Tumor_G2
9829	NDUFA8	6.794888125	Tumor_G2
9830	NDUFB1	5.537784269	Tumor_G2
9831	NDUFB10	7.214475138	Tumor_G2
9832	NDUFB2	5.810965021	Tumor_G2
9833	NDUFB3	5.93059832	Tumor_G2
9834	NDUFB7	8.205338461	Tumor_G2
9835	CYC1	6.315979906	Tumor_G2
9836	ETFB	4.72977788	Tumor_G2
9837	UQCR10	7.222344201	Tumor_G2
9838	UQCRQ	6.917255511	Tumor_G2

9839	UQCC3	4.393947219	Tumor_G2
9840	COX15	5.020481099	Tumor_G2
9841	COX5A	6.597815273	Tumor_G2
9842	COX6B1	8.077201437	Tumor_G2
9843	COX6C	5.991961331	Tumor_G2
9844	COX7A2	6.325430573	Tumor_G2
9845	COX7B	5.666490728	Tumor_G2
9846	COX8A	9.14816103	Tumor_G2
9847	SCO1	3.671002593	Tumor_G2
9848	SURF1	6.357099397	Tumor_G2
9849	ATP6V1E1	6.463252568	Tumor_G2
9850	DLAT	5.029070438	Tumor_G2
9851	PDC	0.249732949	Tumor_G2
9852	SUCLG2	5.994388756	Tumor_G2
9853	SLC25A5	7.591701717	Tumor_G2
9854	UCP2	8.263186476	Tumor_G2
9855	SOD2	4.831945184	Tumor_G2
9856	NOX4	3.540684565	Tumor_G2
9857	DUOX1	7.476602617	Tumor_G2
9858	DUOXA1	7.88294122	Tumor_G2
9859	DUOX2	8.323259907	Tumor_G2
9860	DUOXA2	5.7570979	Tumor_G2
9861	NDUFA1	8.322110013	Tumor_G2
9862	NDUFA2	7.141429201	Tumor_G2
9863	NDUFA8	7.228045176	Tumor_G2
9864	NDUFB1	6.063272565	Tumor_G2
9865	NDUFB10	7.681653881	Tumor_G2
9866	NDUFB2	7.045987059	Tumor_G2
9867	NDUFB3	6.567540959	Tumor_G2
9868	NDUFB7	8.971295188	Tumor_G2
9869	CYC1	6.837069438	Tumor_G2
9870	ETFB	5.260325467	Tumor_G2
9871	UQCR10	8.070255666	Tumor_G2
9872	UQCRQ	7.504541545	Tumor_G2
9873	UQCC3	4.627352267	Tumor_G2
9874	COX15	5.009088132	Tumor_G2
9875	COX5A	7.148547611	Tumor_G2
9876	COX6B1	8.901321085	Tumor_G2
9877	COX6C	6.639509074	Tumor_G2
9878	COX7A2	6.928039504	Tumor_G2

9879	COX7B	6.31481751	Tumor_G2
9880	COX8A	10.02430977	Tumor_G2
9881	SCO1	3.564706379	Tumor_G2
9882	SURF1	7.007485043	Tumor_G2
9883	ATP6V1E1	6.783841612	Tumor_G2
9884	DLAT	4.94421069	Tumor_G2
9885	PDC	0.30309827	Tumor_G2
9886	SUCLG2	6.100288543	Tumor_G2
9887	SLC25A5	7.870646285	Tumor_G2
9888	UCP2	8.527702281	Tumor_G2
9889	SOD2	5.485979194	Tumor_G2
9890	NOX4	1.466293748	Tumor_G2
9891	DUOX1	7.276666331	Tumor_G2
9892	DUOXA1	7.067242106	Tumor_G2
9893	DUOX2	8.279544596	Tumor_G2
9894	DUOXA2	6.156545199	Tumor_G2
9895	NDUFA1	8.206344925	Tumor_G2
9896	NDUFA2	7.447597754	Tumor_G2
9897	NDUFA8	6.389678456	Tumor_G2
9898	NDUFB1	5.955993045	Tumor_G2
9899	NDUFB10	7.864303978	Tumor_G2
9900	NDUFB2	6.15295195	Tumor_G2
9901	NDUFB3	4.915426797	Tumor_G2
9902	NDUFB7	9.401186058	Tumor_G2
9903	CYC1	6.603853134	Tumor_G2
9904	ETFB	4.860813647	Tumor_G2
9905	UQCR10	7.284930921	Tumor_G2
9906	UQCRQ	7.289016808	Tumor_G2
9907	UQCC3	4.869472943	Tumor_G2
9908	COX15	4.857879244	Tumor_G2
9909	COX5A	6.547781076	Tumor_G2
9910	COX6B1	9.179841912	Tumor_G2
9911	COX6C	6.708513855	Tumor_G2
9912	COX7A2	6.383442045	Tumor_G2
9913	COX7B	5.523920941	Tumor_G2
9914	COX8A	9.415199322	Tumor_G2
9915	SCO1	2.554852572	Tumor_G2
9916	SURF1	6.434194865	Tumor_G2
9917	ATP6V1E1	5.279112603	Tumor_G2
9918	DLAT	3.172681615	Tumor_G2

9919	PDC	0.236119435	Tumor_G2
9920	SUCLG2	4.962551577	Tumor_G2
9921	SLC25A5	6.355818937	Tumor_G2
9922	UCP2	6.711832902	Tumor_G2
9923	SOD2	3.200335471	Tumor_G2
9924	NOX4	0.385109849	Tumor_G2
9925	DUOX1	7.543673707	Tumor_G2
9926	DUOXA1	7.405077631	Tumor_G2
9927	DUOX2	7.484620385	Tumor_G2
9928	DUOXA2	5.565743979	Tumor_G2
9929	NDUFA1	7.789704909	Tumor_G2
9930	NDUFA2	7.320088084	Tumor_G2
9931	NDUFA8	7.622864483	Tumor_G2
9932	NDUFB1	5.91087548	Tumor_G2
9933	NDUFB10	7.751293791	Tumor_G2
9934	NDUFB2	6.636931739	Tumor_G2
9935	NDUFB3	6.166025527	Tumor_G2
9936	NDUFB7	8.746597452	Tumor_G2
9937	CYC1	6.768028622	Tumor_G2
9938	ETFB	5.282845314	Tumor_G2
9939	UQCR10	6.799108095	Tumor_G2
9940	UQCRQ	7.86070399	Tumor_G2
9941	UQCC3	4.644859983	Tumor_G2
9942	COX15	4.731219929	Tumor_G2
9943	COX5A	7.045579799	Tumor_G2
9944	COX6B1	8.708974396	Tumor_G2
9945	COX6C	6.387275572	Tumor_G2
9946	COX7A2	6.850633889	Tumor_G2
9947	COX7B	5.997677651	Tumor_G2
9948	COX8A	9.390539016	Tumor_G2
9949	SCO1	3.308855374	Tumor_G2
9950	SURF1	6.720642317	Tumor_G2
9951	ATP6V1E1	6.353475803	Tumor_G2
9952	DLAT	4.826260996	Tumor_G2
9953	PDC	0.194106018	Tumor_G2
9954	SUCLG2	6.102033822	Tumor_G2
9955	SLC25A5	7.935639969	Tumor_G2
9956	UCP2	8.576024994	Tumor_G2
9957	SOD2	4.70571676	Tumor_G2
9958	NOX4	2.383431493	Tumor_G2

9959	DUOX1	6.562551659	Tumor_G2
9960	DUOXA1	6.87608983	Tumor_G2
9961	DUOX2	8.063048363	Tumor_G2
9962	DUOXA2	5.771028264	Tumor_G2
9963	NDUFA1	8.135677463	Tumor_G2
9964	NDUFA2	6.871010156	Tumor_G2
9965	NDUFA8	7.34336206	Tumor_G2
9966	NDUFB1	6.224863441	Tumor_G2
9967	NDUFB10	7.708497942	Tumor_G2
9968	NDUFB2	6.571902432	Tumor_G2
9969	NDUFB3	6.038414446	Tumor_G2
9970	NDUFB7	8.801580969	Tumor_G2
9971	CYC1	6.738322999	Tumor_G2
9972	ETFB	4.886036265	Tumor_G2
9973	UQCR10	7.52775971	Tumor_G2
9974	UQCRQ	7.500681245	Tumor_G2
9975	UQCC3	4.405409961	Tumor_G2
9976	COX15	4.46622842	Tumor_G2
9977	COX5A	6.918240313	Tumor_G2
9978	COX6B1	8.880871312	Tumor_G2
9979	COX6C	6.824377218	Tumor_G2
9980	COX7A2	6.82089824	Tumor_G2
9981	COX7B	6.289631571	Tumor_G2
9982	COX8A	9.486524494	Tumor_G2
9983	SCO1	3.1616881	Tumor_G2
9984	SURF1	6.352842826	Tumor_G2
9985	ATP6V1E1	6.555855562	Tumor_G2
9986	DLAT	4.557983961	Tumor_G2
9987	PDC	0.178533264	Tumor_G2
9988	SUCLG2	5.831433683	Tumor_G2
9989	SLC25A5	7.757764045	Tumor_G2
9990	UCP2	7.304817722	Tumor_G2
9991	SOD2	4.613164625	Tumor_G2
9992	NOX4	2.612194185	Tumor_G2
9993	DUOX1	7.425029593	Tumor_G2
9994	DUOXA1	7.190963849	Tumor_G2
9995	DUOX2	7.971738672	Tumor_G2
9996	DUOXA2	6.602724628	Tumor_G2
9997	NDUFA1	7.893667046	Tumor_G2
9998	NDUFA2	7.002940296	Tumor_G2

9999	NDUFA8	7.114340842	Tumor_G2
10000	NDUFB1	5.67769211	Tumor_G2
10001	NDUFB10	7.799144614	Tumor_G2
10002	NDUFB2	6.506106302	Tumor_G2
10003	NDUFB3	6.214524928	Tumor_G2
10004	NDUFB7	8.793913991	Tumor_G2
10005	CYC1	6.777061619	Tumor_G2
10006	ETFB	5.147660265	Tumor_G2
10007	UQCR10	7.772285298	Tumor_G2
10008	UQCRQ	7.534997353	Tumor_G2
10009	UQCC3	4.650662174	Tumor_G2
10010	COX15	4.498086933	Tumor_G2
10011	COX5A	7.172614429	Tumor_G2
10012	COX6B1	8.823253555	Tumor_G2
10013	COX6C	6.368554153	Tumor_G2
10014	COX7A2	6.999752244	Tumor_G2
10015	COX7B	6.001007589	Tumor_G2
10016	COX8A	9.400254978	Tumor_G2
10017	SCO1	3.054858731	Tumor_G2
10018	SURF1	6.67179011	Tumor_G2
10019	ATP6V1E1	7.369623175	Tumor_G2
10020	DLAT	4.830424339	Tumor_G2
10021	PDC	0.120461763	Tumor_G2
10022	SUCLG2	5.99189648	Tumor_G2
10023	SLC25A5	7.694389982	Tumor_G2
10024	UCP2	8.259027732	Tumor_G2
10025	SOD2	5.089649108	Tumor_G2
10026	NOX4	2.80468119	Tumor_G2
10027	DUOX1	5.548352089	Tumor_G2
10028	DUOXA1	6.03230174	Tumor_G2
10029	DUOX2	5.336054939	Tumor_G2
10030	DUOXA2	4.391068688	Tumor_G2
10031	NDUFA1	7.669892067	Tumor_G2
10032	NDUFA2	6.29721308	Tumor_G2
10033	NDUFA8	6.775896207	Tumor_G2
10034	NDUFB1	5.264332416	Tumor_G2
10035	NDUFB10	7.352379527	Tumor_G2
10036	NDUFB2	6.099764814	Tumor_G2
10037	NDUFB3	5.862310418	Tumor_G2
10038	NDUFB7	8.38459296	Tumor_G2

10039	CYC1	6.808335561	Tumor_G2
10040	ETFB	5.228266618	Tumor_G2
10041	UQCR10	6.7228503	Tumor_G2
10042	UQCRQ	6.67867859	Tumor_G2
10043	UQCC3	4.399981279	Tumor_G2
10044	COX15	5.56818714	Tumor_G2
10045	COX5A	6.636350742	Tumor_G2
10046	COX6B1	8.106328985	Tumor_G2
10047	COX6C	5.994396887	Tumor_G2
10048	COX7A2	6.2327631	Tumor_G2
10049	COX7B	5.683767191	Tumor_G2
10050	COX8A	9.441580003	Tumor_G2
10051	SCO1	3.784776915	Tumor_G2
10052	SURF1	6.22624651	Tumor_G2
10053	ATP6V1E1	6.338204915	Tumor_G2
10054	DLAT	5.47422947	Tumor_G2
10055	PDC	0.469877082	Tumor_G2
10056	SUCLG2	6.383816973	Tumor_G2
10057	SLC25A5	7.609456288	Tumor_G2
10058	UCP2	8.601504751	Tumor_G2
10059	SOD2	4.784127865	Tumor_G2
10060	NOX4	1.943732276	Tumor_G2
10061	DUOX1	7.867823897	Tumor_G2
10062	DUOXA1	7.816063275	Tumor_G2
10063	DUOX2	9.56979456	Tumor_G2
10064	DUOXA2	6.597381126	Tumor_G2
10065	NDUFA1	7.804977325	Tumor_G2
10066	NDUFA2	6.709173614	Tumor_G2
10067	NDUFA8	6.817964355	Tumor_G2
10068	NDUFB1	5.338531795	Tumor_G2
10069	NDUFB10	7.409245203	Tumor_G2
10070	NDUFB2	6.201593094	Tumor_G2
10071	NDUFB3	6.093133313	Tumor_G2
10072	NDUFB7	8.493929243	Tumor_G2
10073	CYC1	6.406645827	Tumor_G2
10074	ETFB	4.955194628	Tumor_G2
10075	UQCR10	7.551339678	Tumor_G2
10076	UQCRQ	7.145025664	Tumor_G2
10077	UQCC3	4.393938586	Tumor_G2
10078	COX15	4.931784662	Tumor_G2

10079	COX5A	6.836914012	Tumor_G2
10080	COX6B1	8.559763547	Tumor_G2
10081	COX6C	6.209418373	Tumor_G2
10082	COX7A2	6.594936312	Tumor_G2
10083	COX7B	5.903074832	Tumor_G2
10084	COX8A	9.083374385	Tumor_G2
10085	SCO1	3.239198332	Tumor_G2
10086	SURF1	6.396162099	Tumor_G2
10087	ATP6V1E1	6.99936709	Tumor_G2
10088	DLAT	5.442256998	Tumor_G2
10089	PDC	0.296789837	Tumor_G2
10090	SUCLG2	6.201429825	Tumor_G2
10091	SLC25A5	7.636770552	Tumor_G2
10092	UCP2	9.175590275	Tumor_G2
10093	SOD2	5.494684569	Tumor_G2
10094	NOX4	3.274927655	Tumor_G2
10095	DUOX1	6.370355747	Tumor_G2
10096	DUOXA1	6.406890386	Tumor_G2
10097	DUOX2	7.345479911	Tumor_G2
10098	DUOXA2	5.38247979	Tumor_G2
10099	NDUFA1	7.76765064	Tumor_G2
10100	NDUFA2	6.204910531	Tumor_G2
10101	NDUFA8	6.917787419	Tumor_G2
10102	NDUFB1	5.321714231	Tumor_G2
10103	NDUFB10	7.479683338	Tumor_G2
10104	NDUFB2	6.496671465	Tumor_G2
10105	NDUFB3	6.104023777	Tumor_G2
10106	NDUFB7	8.561503275	Tumor_G2
10107	CYC1	7.064910259	Tumor_G2
10108	ETFB	5.201108912	Tumor_G2
10109	UQCR10	7.496091882	Tumor_G2
10110	UQCRQ	6.876007802	Tumor_G2
10111	UQCC3	4.327520831	Tumor_G2
10112	COX15	5.545764471	Tumor_G2
10113	COX5A	6.889688314	Tumor_G2
10114	COX6B1	8.327480799	Tumor_G2
10115	COX6C	6.110721687	Tumor_G2
10116	COX7A2	6.565373279	Tumor_G2
10117	COX7B	5.970989857	Tumor_G2
10118	COX8A	9.542608542	Tumor_G2

10119	SCO1	3.718544645	Tumor_G2
10120	SURF1	6.645802093	Tumor_G2
10121	ATP6V1E1	7.11301052	Tumor_G2
10122	DLAT	5.352484821	Tumor_G2
10123	PDC	0.107184568	Tumor_G2
10124	SUCLG2	6.441683233	Tumor_G2
10125	SLC25A5	7.879733262	Tumor_G2
10126	UCP2	10.17720308	Tumor_G2
10127	SOD2	4.91722427	Tumor_G2
10128	NOX4	1.378998069	Tumor_G2
10129	DUOX1	7.691133926	Tumor_G2
10130	DUOXA1	7.471076988	Tumor_G2
10131	DUOX2	8.504822979	Tumor_G2
10132	DUOXA2	6.013533484	Tumor_G2
10133	NDUFA1	8.606557408	Tumor_G2
10134	NDUFA2	7.056940371	Tumor_G2
10135	NDUFA8	7.510887498	Tumor_G2
10136	NDUFB1	6.005939806	Tumor_G2
10137	NDUFB10	7.733994714	Tumor_G2
10138	NDUFB2	7.653393849	Tumor_G2
10139	NDUFB3	6.757807741	Tumor_G2
10140	NDUFB7	9.073527098	Tumor_G2
10141	CYC1	7.581862228	Tumor_G2
10142	ETFB	5.046592285	Tumor_G2
10143	UQCR10	7.935630554	Tumor_G2
10144	UQCRQ	7.658000748	Tumor_G2
10145	UQCC3	4.523229466	Tumor_G2
10146	COX15	5.207864086	Tumor_G2
10147	COX5A	7.623959789	Tumor_G2
10148	COX6B1	8.797273908	Tumor_G2
10149	COX6C	7.102200103	Tumor_G2
10150	COX7A2	7.408122859	Tumor_G2
10151	COX7B	6.763311119	Tumor_G2
10152	COX8A	10.19053156	Tumor_G2
10153	SCO1	3.642542642	Tumor_G2
10154	SURF1	6.723232208	Tumor_G2
10155	ATP6V1E1	7.28790652	Tumor_G2
10156	DLAT	5.240664817	Tumor_G2
10157	PDC	0.090132829	Tumor_G2
10158	SUCLG2	6.904973535	Tumor_G2

10159	SLC25A5	9.162836757	Tumor_G2
10160	UCP2	7.149930664	Tumor_G2
10161	SOD2	4.941144455	Tumor_G2
10162	NOX4	1.281086098	Tumor_G2
10163	DUOX1	6.638032993	Tumor_G2
10164	DUOXA1	5.99835041	Tumor_G2
10165	DUOX2	8.01445226	Tumor_G2
10166	DUOXA2	6.253374628	Tumor_G2
10167	NDUFA1	8.082949665	Tumor_G2
10168	NDUFA2	6.526099461	Tumor_G2
10169	NDUFA8	7.79869511	Tumor_G2
10170	NDUFB1	5.656857996	Tumor_G2
10171	NDUFB10	7.855350483	Tumor_G2
10172	NDUFB2	7.049887123	Tumor_G2
10173	NDUFB3	6.679396738	Tumor_G2
10174	NDUFB7	8.860040287	Tumor_G2
10175	CYC1	8.362846204	Tumor_G2
10176	ETFB	5.055449003	Tumor_G2
10177	UQCR10	7.870940648	Tumor_G2
10178	UQCRQ	7.030179561	Tumor_G2
10179	UQCC3	4.362381361	Tumor_G2
10180	COX15	5.324775023	Tumor_G2
10181	COX5A	8.157952696	Tumor_G2
10182	COX6B1	9.051058558	Tumor_G2
10183	COX6C	7.048138108	Tumor_G2
10184	COX7A2	7.051615858	Tumor_G2
10185	COX7B	7.160708278	Tumor_G2
10186	COX8A	10.46155601	Tumor_G2
10187	SCO1	3.784138046	Tumor_G2
10188	SURF1	6.491035777	Tumor_G2
10189	ATP6V1E1	6.92498378	Tumor_G2
10190	DLAT	5.446751344	Tumor_G2
10191	PDC	0.113798646	Tumor_G2
10192	SUCLG2	6.783011112	Tumor_G2
10193	SLC25A5	9.791893552	Tumor_G2
10194	UCP2	5.545953114	Tumor_G2
10195	SOD2	5.377081691	Tumor_G2
10196	NOX4	0.512635916	Tumor_G2
10197	DUOX1	5.71147648	Tumor_G2
10198	DUOXA1	5.113111222	Tumor_G2

10199	DUOX2	5.465956833	Tumor_G2
10200	DUOXA2	3.680176441	Tumor_G2
10201	NDUFA1	8.627805414	Tumor_G2
10202	NDUFA2	6.545263873	Tumor_G2
10203	NDUFA8	7.133730073	Tumor_G2
10204	NDUFB1	5.939217117	Tumor_G2
10205	NDUFB10	7.959496723	Tumor_G2
10206	NDUFB2	6.637546828	Tumor_G2
10207	NDUFB3	6.85945316	Tumor_G2
10208	NDUFB7	8.605560338	Tumor_G2
10209	CYC1	8.647938635	Tumor_G2
10210	ETFB	4.447938205	Tumor_G2
10211	UQCR10	8.359684421	Tumor_G2
10212	UQCRQ	7.623016088	Tumor_G2
10213	UQCC3	4.089731384	Tumor_G2
10214	COX15	5.318777315	Tumor_G2
10215	COX5A	8.795497536	Tumor_G2
10216	COX6B1	9.081663221	Tumor_G2
10217	COX6C	7.416568653	Tumor_G2
10218	COX7A2	7.598344618	Tumor_G2
10219	COX7B	7.570808673	Tumor_G2
10220	COX8A	10.57891562	Tumor_G2
10221	SCO1	3.070227243	Tumor_G2
10222	SURF1	5.210591046	Tumor_G2
10223	ATP6V1E1	6.233996551	Tumor_G2
10224	DLAT	5.971782382	Tumor_G2
10225	PDC	0	Tumor_G2
10226	SUCLG2	6.491203752	Tumor_G2
10227	SLC25A5	10.61195812	Tumor_G2
10228	UCP2	3.215634247	Tumor_G2
10229	SOD2	5.121312494	Tumor_G2
10230	NOX4	0.293249235	Tumor_G2
10231	DUOX1	5.036630432	Tumor_G2
10232	DUOXA1	3.379245931	Tumor_G2
10233	DUOX2	3.529178226	Tumor_G2
10234	DUOXA2	1.558879376	Tumor_G2
10235	NDUFA1	8.566426931	Tumor_G2
10236	NDUFA2	7.051944311	Tumor_G2
10237	NDUFA8	6.706719314	Tumor_G2
10238	NDUFB1	5.006427438	Tumor_G2

10239	NDUFB10	6.997158182	Tumor_G2
10240	NDUFB2	7.278105142	Tumor_G2
10241	NDUFB3	5.832278286	Tumor_G2
10242	NDUFB7	8.931797299	Tumor_G2
10243	CYC1	7.099393771	Tumor_G2
10244	ETFB	4.740515857	Tumor_G2
10245	UQCR10	7.049524167	Tumor_G2
10246	UQCRQ	7.242241089	Tumor_G2
10247	UQCC3	4.904520965	Tumor_G2
10248	COX15	5.061828311	Tumor_G2
10249	COX5A	7.423559437	Tumor_G2
10250	COX6B1	8.661460321	Tumor_G2
10251	COX6C	6.268209622	Tumor_G2
10252	COX7A2	6.518757041	Tumor_G2
10253	COX7B	6.724247867	Tumor_G2
10254	COX8A	9.897606701	Tumor_G2
10255	SCO1	3.640055444	Tumor_G2
10256	SURF1	5.335682509	Tumor_G2
10257	ATP6V1E1	6.533957808	Tumor_G2
10258	DLAT	5.244238144	Tumor_G2
10259	PDC	0	Tumor_G2
10260	SUCLG2	5.719755295	Tumor_G2
10261	SLC25A5	10.09950482	Tumor_G2
10262	UCP2	6.326680185	Tumor_G2
10263	SOD2	4.626209629	Tumor_G2
10264	NOX4	0.546335453	Tumor_G2
10265	DUOX1	5.659164406	Tumor_G2
10266	DUOXA1	4.905504429	Tumor_G2
10267	DUOX2	7.329813323	Tumor_G2
10268	DUOXA2	6.460111431	Tumor_G2
10269	NDUFA1	7.960976841	Tumor_G2
10270	NDUFA2	7.037855259	Tumor_G2
10271	NDUFA8	7.483663031	Tumor_G2
10272	NDUFB1	6.026353821	Tumor_G2
10273	NDUFB10	7.818382248	Tumor_G2
10274	NDUFB2	7.157733414	Tumor_G2
10275	NDUFB3	6.550080069	Tumor_G2
10276	NDUFB7	8.614209886	Tumor_G2
10277	CYC1	6.971867483	Tumor_G2
10278	ETFB	5.25255589	Tumor_G2

10279	UQCR10	7.573912905	Tumor_G2
10280	UQCRQ	7.355208114	Tumor_G2
10281	UQCC3	4.155392525	Tumor_G2
10282	COX15	5.358692969	Tumor_G2
10283	COX5A	6.909790474	Tumor_G2
10284	COX6B1	8.33203922	Tumor_G2
10285	COX6C	6.518754721	Tumor_G2
10286	COX7A2	6.873966905	Tumor_G2
10287	COX7B	6.04673282	Tumor_G2
10288	COX8A	9.717533012	Tumor_G2
10289	SCO1	4.393888881	Tumor_G2
10290	SURF1	7.14658199	Tumor_G2
10291	ATP6V1E1	6.514995619	Tumor_G2
10292	DLAT	5.290764704	Tumor_G2
10293	PDC	0.068013753	Tumor_G2
10294	SUCLG2	6.132906975	Tumor_G2
10295	SLC25A5	8.148769925	Tumor_G2
10296	UCP2	6.100810645	Tumor_G2
10297	SOD2	4.673948127	Tumor_G2
10298	NOX4	2.226291163	Tumor_G2
10299	DUOX1	6.39767707	Tumor_G2
10300	DUOXA1	6.073746276	Tumor_G2
10301	DUOX2	7.569461079	Tumor_G2
10302	DUOXA2	6.740994049	Tumor_G2
10303	NDUFA1	8.182181758	Tumor_G2
10304	NDUFA2	6.379015869	Tumor_G2
10305	NDUFA8	7.595273457	Tumor_G2
10306	NDUFB1	5.858588544	Tumor_G2
10307	NDUFB10	7.435314298	Tumor_G2
10308	NDUFB2	6.837097891	Tumor_G2
10309	NDUFB3	6.633758293	Tumor_G2
10310	NDUFB7	8.440612678	Tumor_G2
10311	CYC1	7.165253015	Tumor_G2
10312	ETFB	5.281717082	Tumor_G2
10313	UQCR10	7.68469479	Tumor_G2
10314	UQCRQ	7.294150885	Tumor_G2
10315	UQCC3	4.186989019	Tumor_G2
10316	COX15	5.28614518	Tumor_G2
10317	COX5A	7.583053675	Tumor_G2
10318	COX6B1	8.764004194	Tumor_G2

10319	COX6C	6.775466619	Tumor_G2
10320	COX7A2	7.176595135	Tumor_G2
10321	COX7B	6.932649193	Tumor_G2
10322	COX8A	10.1342137	Tumor_G2
10323	SCO1	3.339351923	Tumor_G2
10324	SURF1	6.551180766	Tumor_G2
10325	ATP6V1E1	6.968317114	Tumor_G2
10326	DLAT	5.712213397	Tumor_G2
10327	PDC	0	Tumor_G2
10328	SUCLG2	7.164737962	Tumor_G2
10329	SLC25A5	8.719875945	Tumor_G2
10330	UCP2	5.01808403	Tumor_G2
10331	SOD2	4.764650782	Tumor_G2
10332	NOX4	0.922907611	Tumor_G2
10333	DUOX1	7.442510022	Tumor_G2
10334	DUOXA1	6.364756559	Tumor_G2
10335	DUOX2	8.671263118	Tumor_G2
10336	DUOXA2	6.108166007	Tumor_G2
10337	NDUFA1	8.505682498	Tumor_G2
10338	NDUFA2	6.909398043	Tumor_G2
10339	NDUFA8	7.637138477	Tumor_G2
10340	NDUFB1	6.272120303	Tumor_G2
10341	NDUFB10	7.741418001	Tumor_G2
10342	NDUFB2	6.713587986	Tumor_G2
10343	NDUFB3	6.643532173	Tumor_G2
10344	NDUFB7	8.68639983	Tumor_G2
10345	CYC1	7.037409993	Tumor_G2
10346	ETFB	5.743837088	Tumor_G2
10347	UQCR10	7.118633225	Tumor_G2
10348	UQCRQ	7.661391635	Tumor_G2
10349	UQCC3	4.725152996	Tumor_G2
10350	COX15	5.265427965	Tumor_G2
10351	COX5A	7.260253844	Tumor_G2
10352	COX6B1	8.878917264	Tumor_G2
10353	COX6C	6.819828219	Tumor_G2
10354	COX7A2	7.0436332	Tumor_G2
10355	COX7B	6.485678118	Tumor_G2
10356	COX8A	9.901031354	Tumor_G2
10357	SCO1	3.620410405	Tumor_G2
10358	SURF1	6.556271015	Tumor_G2

10359	ATP6V1E1	7.254054368	Tumor_G2
10360	DLAT	5.209642562	Tumor_G2
10361	PDC	0.062585018	Tumor_G2
10362	SUCLG2	6.568167632	Tumor_G2
10363	SLC25A5	7.976572597	Tumor_G2
10364	UCP2	7.597766411	Tumor_G2
10365	SOD2	4.467646071	Tumor_G2
10366	NOX4	1.530690251	Tumor_G2
10367	DUOX1	9.247167993	Tumor_G2
10368	DUOXA1	8.665028992	Tumor_G2
10369	DUOX2	10.13103236	Tumor_G2
10370	DUOXA2	7.701317977	Tumor_G2
10371	NDUFA1	8.087161084	Tumor_G2
10372	NDUFA2	7.025356059	Tumor_G2
10373	NDUFA8	7.53959981	Tumor_G2
10374	NDUFB1	6.148060584	Tumor_G2
10375	NDUFB10	7.416864965	Tumor_G2
10376	NDUFB2	6.964021359	Tumor_G2
10377	NDUFB3	6.954623554	Tumor_G2
10378	NDUFB7	8.714938462	Tumor_G2
10379	CYC1	7.051332502	Tumor_G2
10380	ETFB	5.587681269	Tumor_G2
10381	UQCR10	7.03709038	Tumor_G2
10382	UQCRQ	7.758209944	Tumor_G2
10383	UQCC3	4.576951776	Tumor_G2
10384	COX15	5.434687338	Tumor_G2
10385	COX5A	7.160787917	Tumor_G2
10386	COX6B1	8.382497729	Tumor_G2
10387	COX6C	6.660477841	Tumor_G2
10388	COX7A2	7.143094535	Tumor_G2
10389	COX7B	6.446702154	Tumor_G2
10390	COX8A	9.844258994	Tumor_G2
10391	SCO1	4.476082748	Tumor_G2
10392	SURF1	6.817907419	Tumor_G2
10393	ATP6V1E1	6.330505868	Tumor_G2
10394	DLAT	5.496919885	Tumor_G2
10395	PDC	0.139186385	Tumor_G2
10396	SUCLG2	6.963790887	Tumor_G2
10397	SLC25A5	8.367427991	Tumor_G2
10398	UCP2	8.318272771	Tumor_G2

10399	SOD2	4.360685928	Tumor_G2
10400	NOX4	1.204873566	Tumor_G2
10401	DUOX1	8.044359095	Tumor_G2
10402	DUOXA1	7.380899814	Tumor_G2
10403	DUOX2	10.16548747	Tumor_G2
10404	DUOXA2	7.654440618	Tumor_G2
10405	NDUFA1	8.285257579	Tumor_G2
10406	NDUFA2	6.855055571	Tumor_G2
10407	NDUFA8	7.100121577	Tumor_G2
10408	NDUFB1	5.728094707	Tumor_G2
10409	NDUFB10	7.593255781	Tumor_G2
10410	NDUFB2	6.659051266	Tumor_G2
10411	NDUFB3	5.622700889	Tumor_G2
10412	NDUFB7	8.571850349	Tumor_G2
10413	CYC1	7.21632286	Tumor_G2
10414	ETFB	5.171188804	Tumor_G2
10415	UQCR10	7.743657095	Tumor_G2
10416	UQCRQ	7.201012665	Tumor_G2
10417	UQCC3	3.44659033	Tumor_G2
10418	COX15	5.329063268	Tumor_G2
10419	COX5A	7.164676637	Tumor_G2
10420	COX6B1	8.430823883	Tumor_G2
10421	COX6C	6.57099164	Tumor_G2
10422	COX7A2	6.815895533	Tumor_G2
10423	COX7B	6.521109974	Tumor_G2
10424	COX8A	9.207997482	Tumor_G2
10425	SCO1	3.821193313	Tumor_G2
10426	SURF1	6.152363489	Tumor_G2
10427	ATP6V1E1	6.900053485	Tumor_G2
10428	DLAT	4.586035896	Tumor_G2
10429	PDC	0.085920101	Tumor_G2
10430	SUCLG2	5.756683738	Tumor_G2
10431	SLC25A5	8.756449174	Tumor_G2
10432	UCP2	5.682132113	Tumor_G2
10433	SOD2	4.331669723	Tumor_G2
10434	NOX4	0.773899487	Tumor_G2
10435	DUOX1	5.250248914	Tumor_G2
10436	DUOXA1	4.694940835	Tumor_G2
10437	DUOX2	6.336922684	Tumor_G2
10438	DUOXA2	5.167033993	Tumor_G2

10439	NDUFA1	8.357985989	Tumor_G2
10440	NDUFA2	7.044152914	Tumor_G2
10441	NDUFA8	7.816202059	Tumor_G2
10442	NDUFB1	5.864356493	Tumor_G2
10443	NDUFB10	8.250427529	Tumor_G2
10444	NDUFB2	7.137741233	Tumor_G2
10445	NDUFB3	6.148273135	Tumor_G2
10446	NDUFB7	9.048377752	Tumor_G2
10447	CYC1	7.927147958	Tumor_G2
10448	ETFB	5.663791484	Tumor_G2
10449	UQCR10	8.082339106	Tumor_G2
10450	UQCRQ	7.62470575	Tumor_G2
10451	UQCC3	4.845596102	Tumor_G2
10452	COX15	5.33987013	Tumor_G2
10453	COX5A	7.908050544	Tumor_G2
10454	COX6B1	9.284704027	Tumor_G2
10455	COX6C	6.786969515	Tumor_G2
10456	COX7A2	7.114192968	Tumor_G2
10457	COX7B	6.818404991	Tumor_G2
10458	COX8A	10.48855336	Tumor_G2
10459	SCO1	3.658972034	Tumor_G2
10460	SURF1	6.4024581	Tumor_G2
10461	ATP6V1E1	7.467350012	Tumor_G2
10462	DLAT	5.136884068	Tumor_G2
10463	PDC	0.15642032	Tumor_G2
10464	SUCLG2	6.517504401	Tumor_G2
10465	SLC25A5	9.20239344	Tumor_G2
10466	UCP2	7.963218541	Tumor_G2
10467	SOD2	5.025229945	Tumor_G2
10468	NOX4	1.022419166	Tumor_G2
10469	DUOX1	6.507904978	Tumor_G2
10470	DUOXA1	6.612381838	Tumor_G2
10471	DUOX2	6.719009608	Tumor_G2
10472	DUOXA2	5.283356956	Tumor_G2
10473	NDUFA1	7.930513687	Tumor_G2
10474	NDUFA2	6.570494054	Tumor_G2
10475	NDUFA8	6.775428357	Tumor_G2
10476	NDUFB1	5.430626722	Tumor_G2
10477	NDUFB10	7.715803161	Tumor_G2
10478	NDUFB2	6.208469279	Tumor_G2

10479	NDUFB3	6.132162103	Tumor_G2
10480	NDUFB7	8.200079943	Tumor_G2
10481	CYC1	6.79602144	Tumor_G2
10482	ETFB	5.206755936	Tumor_G2
10483	UQCR10	7.063439271	Tumor_G2
10484	UQCRQ	6.865180585	Tumor_G2
10485	UQCC3	4.537382705	Tumor_G2
10486	COX15	4.924381602	Tumor_G2
10487	COX5A	6.872046165	Tumor_G2
10488	COX6B1	8.647533483	Tumor_G2
10489	COX6C	6.155689142	Tumor_G2
10490	COX7A2	6.656572703	Tumor_G2
10491	COX7B	5.940291846	Tumor_G2
10492	COX8A	9.475732925	Tumor_G2
10493	SCO1	3.281517327	Tumor_G2
10494	SURF1	6.388271244	Tumor_G2
10495	ATP6V1E1	6.516080733	Tumor_G2
10496	DLAT	5.047118051	Tumor_G2
10497	PDC	0.059572395	Tumor_G2
10498	SUCLG2	6.062984921	Tumor_G2
10499	SLC25A5	8.123278273	Tumor_G2
10500	UCP2	9.114119656	Tumor_G2
10501	SOD2	5.31064931	Tumor_G2
10502	NOX4	3.485986396	Tumor_G2
10503	DUOX1	6.701503393	Tumor_G2
10504	DUOXA1	7.096434381	Tumor_G2
10505	DUOX2	7.526571391	Tumor_G2
10506	DUOXA2	5.247867126	Tumor_G2
10507	NDUFA1	8.317202263	Tumor_G2
10508	NDUFA2	7.500140471	Tumor_G2
10509	NDUFA8	7.67411294	Tumor_G2
10510	NDUFB1	5.996408088	Tumor_G2
10511	NDUFB10	7.946000734	Tumor_G2
10512	NDUFB2	8.074936976	Tumor_G2
10513	NDUFB3	6.645066292	Tumor_G2
10514	NDUFB7	9.403783598	Tumor_G2
10515	CYC1	7.433933347	Tumor_G2
10516	ETFB	5.570167601	Tumor_G2
10517	UQCR10	7.687673432	Tumor_G2
10518	UQCRQ	7.63021033	Tumor_G2

10519	UQCC3	5.043474513	Tumor_G2
10520	COX15	5.385760169	Tumor_G2
10521	COX5A	7.184402222	Tumor_G2
10522	COX6B1	8.965936952	Tumor_G2
10523	COX6C	6.710223029	Tumor_G2
10524	COX7A2	7.021805967	Tumor_G2
10525	COX7B	6.43502871	Tumor_G2
10526	COX8A	10.51732225	Tumor_G2
10527	SCO1	3.817598889	Tumor_G2
10528	SURF1	7.59474986	Tumor_G2
10529	ATP6V1E1	6.29704613	Tumor_G2
10530	DLAT	5.1651178	Tumor_G2
10531	PDC	0	Tumor_G2
10532	SUCLG2	6.684842621	Tumor_G2
10533	SLC25A5	8.226826619	Tumor_G2
10534	UCP2	7.342456182	Tumor_G2
10535	SOD2	4.400535066	Tumor_G2
10536	NOX4	0.571647884	Tumor_G2
10537	DUOX1	6.835387272	Tumor_G2
10538	DUOXA1	6.847736333	Tumor_G2
10539	DUOX2	7.555460545	Tumor_G2
10540	DUOXA2	6.360853145	Tumor_G2
10541	NDUFA1	8.36145244	Tumor_G2
10542	NDUFA2	7.058183227	Tumor_G2
10543	NDUFA8	7.285691699	Tumor_G2
10544	NDUFB1	6.626073931	Tumor_G2
10545	NDUFB10	7.686357418	Tumor_G2
10546	NDUFB2	7.018929011	Tumor_G2
10547	NDUFB3	6.646795926	Tumor_G2
10548	NDUFB7	9.122643112	Tumor_G2
10549	CYC1	6.875847449	Tumor_G2
10550	ETFB	5.586626922	Tumor_G2
10551	UQCR10	7.909009987	Tumor_G2
10552	UQCRQ	7.791422565	Tumor_G2
10553	UQCC3	4.883575573	Tumor_G2
10554	COX15	4.900253644	Tumor_G2
10555	COX5A	7.330042257	Tumor_G2
10556	COX6B1	8.904931841	Tumor_G2
10557	COX6C	6.971275383	Tumor_G2
10558	COX7A2	7.154277413	Tumor_G2

10559	COX7B	6.590731237	Tumor_G2
10560	COX8A	9.925465108	Tumor_G2
10561	SCO1	3.320826736	Tumor_G2
10562	SURF1	6.964169242	Tumor_G2
10563	ATP6V1E1	6.686660743	Tumor_G2
10564	DLAT	4.999701113	Tumor_G2
10565	PDC	0.124853928	Tumor_G2
10566	SUCLG2	6.198284921	Tumor_G2
10567	SLC25A5	7.933430092	Tumor_G2
10568	UCP2	7.654119945	Tumor_G2
10569	SOD2	4.314051024	Tumor_G2
10570	NOX4	1.910725139	Tumor_G2
10571	DUOX1	7.837820741	Tumor_G2
10572	DUOXA1	7.290832381	Tumor_G2
10573	DUOX2	9.916376415	Tumor_G2
10574	DUOXA2	7.922213975	Tumor_G2
10575	NDUFA1	7.904761238	Tumor_G2
10576	NDUFA2	6.718939129	Tumor_G2
10577	NDUFA8	7.129112919	Tumor_G2
10578	NDUFB1	5.560117092	Tumor_G2
10579	NDUFB10	7.193043728	Tumor_G2
10580	NDUFB2	6.423308945	Tumor_G2
10581	NDUFB3	6.348245326	Tumor_G2
10582	NDUFB7	8.317071395	Tumor_G2
10583	CYC1	6.54928026	Tumor_G2
10584	ETFB	4.908775174	Tumor_G2
10585	UQCR10	7.666580013	Tumor_G2
10586	UQCRQ	6.974625631	Tumor_G2
10587	UQCC3	4.247292454	Tumor_G2
10588	COX15	5.253093924	Tumor_G2
10589	COX5A	6.774595784	Tumor_G2
10590	COX6B1	8.295096339	Tumor_G2
10591	COX6C	6.261949238	Tumor_G2
10592	COX7A2	6.543640533	Tumor_G2
10593	COX7B	5.819265746	Tumor_G2
10594	COX8A	9.723966992	Tumor_G2
10595	SCO1	4.192096615	Tumor_G2
10596	SURF1	6.865887166	Tumor_G2
10597	ATP6V1E1	6.580091312	Tumor_G2
10598	DLAT	5.222381711	Tumor_G2

10599	PDC	0.235879171	Tumor_G2
10600	SUCLG2	6.48531425	Tumor_G2
10601	SLC25A5	7.991366964	Tumor_G2
10602	UCP2	5.594486863	Tumor_G2
10603	SOD2	4.699761957	Tumor_G2
10604	NOX4	1.227824022	Tumor_G2
10605	DUOX1	7.604016832	Tumor_G2
10606	DUOXA1	6.687936295	Tumor_G2
10607	DUOX2	7.973511246	Tumor_G2
10608	DUOXA2	6.183253167	Tumor_G2
10609	NDUFA1	8.048710133	Tumor_G2
10610	NDUFA2	6.800241858	Tumor_G2
10611	NDUFA8	6.960602696	Tumor_G2
10612	NDUFB1	5.781317562	Tumor_G2
10613	NDUFB10	7.60363958	Tumor_G2
10614	NDUFB2	6.269117035	Tumor_G2
10615	NDUFB3	5.923229541	Tumor_G2
10616	NDUFB7	8.365037002	Tumor_G2
10617	CYC1	7.129990333	Tumor_G2
10618	ETFB	4.943218749	Tumor_G2
10619	UQCR10	6.75674453	Tumor_G2
10620	UQCRQ	7.299454973	Tumor_G2
10621	UQCC3	4.294669724	Tumor_G2
10622	COX15	4.540546032	Tumor_G2
10623	COX5A	7.384117704	Tumor_G2
10624	COX6B1	8.711938722	Tumor_G2
10625	COX6C	6.49306689	Tumor_G2
10626	COX7A2	6.759794137	Tumor_G2
10627	COX7B	6.133887676	Tumor_G2
10628	COX8A	9.95282626	Tumor_G2
10629	SCO1	2.853896963	Tumor_G2
10630	SURF1	6.025063187	Tumor_G2
10631	ATP6V1E1	6.015194854	Tumor_G2
10632	DLAT	4.728073835	Tumor_G2
10633	PDC	0.048433958	Tumor_G2
10634	SUCLG2	6.280593088	Tumor_G2
10635	SLC25A5	8.645202055	Tumor_G2
10636	UCP2	6.723350704	Tumor_G2
10637	SOD2	3.806441661	Tumor_G2
10638	NOX4	0.88551373	Tumor_G2

10639	DUOX1	5.844560964	Tumor_G2
10640	DUOXA1	5.303028881	Tumor_G2
10641	DUOX2	9.550303474	Tumor_G2
10642	DUOXA2	6.887138653	Tumor_G2
10643	NDUFA1	7.937227925	Tumor_G2
10644	NDUFA2	7.347305928	Tumor_G2
10645	NDUFA8	7.33610292	Tumor_G2
10646	NDUFB1	5.842762642	Tumor_G2
10647	NDUFB10	7.943785309	Tumor_G2
10648	NDUFB2	6.099767886	Tumor_G2
10649	NDUFB3	5.313983329	Tumor_G2
10650	NDUFB7	9.249922762	Tumor_G2
10651	CYC1	7.102746926	Tumor_G2
10652	ETFB	5.001741693	Tumor_G2
10653	UQCR10	8.037094899	Tumor_G2
10654	UQCRQ	7.260463793	Tumor_G2
10655	UQCC3	5.158786494	Tumor_G2
10656	COX15	4.991806565	Tumor_G2
10657	COX5A	6.821335306	Tumor_G2
10658	COX6B1	9.466749336	Tumor_G2
10659	COX6C	6.757666757	Tumor_G2
10660	COX7A2	6.675506802	Tumor_G2
10661	COX7B	5.953258989	Tumor_G2
10662	COX8A	9.441827468	Tumor_G2
10663	SCO1	3.105791576	Tumor_G2
10664	SURF1	7.134001611	Tumor_G2
10665	ATP6V1E1	6.540440854	Tumor_G2
10666	DLAT	4.487136798	Tumor_G2
10667	PDC	0.203613303	Tumor_G2
10668	SUCLG2	6.263334813	Tumor_G2
10669	SLC25A5	7.532598876	Tumor_G2
10670	UCP2	7.004427453	Tumor_G2
10671	SOD2	4.893138777	Tumor_G2
10672	NOX4	0.778910824	Tumor_G2
10673	DUOX1	7.332143921	Tumor_G2
10674	DUOXA1	6.912838838	Tumor_G2
10675	DUOX2	7.172740379	Tumor_G2
10676	DUOXA2	5.775073613	Tumor_G2
10677	NDUFA1	8.300596821	Tumor_G2
10678	NDUFA2	7.11882831	Tumor_G2

10679	NDUFA8	7.275469751	Tumor_G2
10680	NDUFB1	5.890643404	Tumor_G2
10681	NDUFB10	7.927379204	Tumor_G2
10682	NDUFB2	6.868312842	Tumor_G2
10683	NDUFB3	5.329389903	Tumor_G2
10684	NDUFB7	8.627342232	Tumor_G2
10685	CYC1	7.949856175	Tumor_G2
10686	ETFB	4.966738715	Tumor_G2
10687	UQCR10	7.529387435	Tumor_G2
10688	UQCRQ	7.495768598	Tumor_G2
10689	UQCC3	4.268536514	Tumor_G2
10690	COX15	4.72166578	Tumor_G2
10691	COX5A	7.446242184	Tumor_G2
10692	COX6B1	8.649258197	Tumor_G2
10693	COX6C	7.1600758	Tumor_G2
10694	COX7A2	6.974526204	Tumor_G2
10695	COX7B	6.779642051	Tumor_G2
10696	COX8A	9.752552646	Tumor_G2
10697	SCO1	3.365479563	Tumor_G2
10698	SURF1	6.205091114	Tumor_G2
10699	ATP6V1E1	6.665157137	Tumor_G2
10700	DLAT	5.077397346	Tumor_G2
10701	PDC	0	Tumor_G2
10702	SUCLG2	5.920748009	Tumor_G2
10703	SLC25A5	8.782952987	Tumor_G2
10704	UCP2	4.777769331	Tumor_G2
10705	SOD2	4.018352583	Tumor_G2
10706	NOX4	0.197939568	Tumor_G2
10707	DUOX1	4.785326986	Tumor_G2
10708	DUOXA1	4.768231072	Tumor_G2
10709	DUOX2	5.970081237	Tumor_G2
10710	DUOXA2	4.730056174	Tumor_G2
10711	NDUFA1	8.346371852	Tumor_G2
10712	NDUFA2	6.585829796	Tumor_G2
10713	NDUFA8	7.387401881	Tumor_G2
10714	NDUFB1	5.62309086	Tumor_G2
10715	NDUFB10	7.522111333	Tumor_G2
10716	NDUFB2	7.101325897	Tumor_G2
10717	NDUFB3	6.701075976	Tumor_G2
10718	NDUFB7	8.694912238	Tumor_G2

10719	CYC1	7.476309057	Tumor_G2
10720	ETFB	4.684303764	Tumor_G2
10721	UQCR10	7.841892918	Tumor_G2
10722	UQCRQ	7.153223691	Tumor_G2
10723	UQCC3	4.18893463	Tumor_G2
10724	COX15	5.314739396	Tumor_G2
10725	COX5A	7.58705176	Tumor_G2
10726	COX6B1	8.685094368	Tumor_G2
10727	COX6C	6.841209512	Tumor_G2
10728	COX7A2	7.128640925	Tumor_G2
10729	COX7B	6.479551373	Tumor_G2
10730	COX8A	9.948244741	Tumor_G2
10731	SCO1	3.959749281	Tumor_G2
10732	SURF1	6.097928966	Tumor_G2
10733	ATP6V1E1	7.119503634	Tumor_G2
10734	DLAT	5.689561097	Tumor_G2
10735	PDC	0.131566441	Tumor_G2
10736	SUCLG2	6.697348003	Tumor_G2
10737	SLC25A5	9.085420924	Tumor_G2
10738	UCP2	6.738216668	Tumor_G2
10739	SOD2	5.21441174	Tumor_G2
10740	NOX4	1.291092618	Tumor_G2
10741	DUOX1	6.288106062	Tumor_G2
10742	DUOXA1	6.197386898	Tumor_G2
10743	DUOX2	8.189672916	Tumor_G2
10744	DUOXA2	5.288912701	Tumor_G2
10745	NDUFA1	7.881192686	Tumor_G2
10746	NDUFA2	6.352376484	Tumor_G2
10747	NDUFA8	7.084963009	Tumor_G2
10748	NDUFB1	5.613913581	Tumor_G2
10749	NDUFB10	7.427249934	Tumor_G2
10750	NDUFB2	6.622832924	Tumor_G2
10751	NDUFB3	6.185168068	Tumor_G2
10752	NDUFB7	8.518042084	Tumor_G2
10753	CYC1	6.939048434	Tumor_G2
10754	ETFB	5.722345955	Tumor_G2
10755	UQCR10	7.494903641	Tumor_G2
10756	UQCRQ	7.013894369	Tumor_G2
10757	UQCC3	4.576197814	Tumor_G2
10758	COX15	5.206464812	Tumor_G2

10759	COX5A	6.922222621	Tumor_G2
10760	COX6B1	8.503249239	Tumor_G2
10761	COX6C	6.32266797	Tumor_G2
10762	COX7A2	6.924124767	Tumor_G2
10763	COX7B	5.919049731	Tumor_G2
10764	COX8A	9.337149475	Tumor_G2
10765	SCO1	3.656099293	Tumor_G2
10766	SURF1	6.540786828	Tumor_G2
10767	ATP6V1E1	6.876782126	Tumor_G2
10768	DLAT	5.154639613	Tumor_G2
10769	PDC	0.298896517	Tumor_G2
10770	SUCLG2	6.131138555	Tumor_G2
10771	SLC25A5	7.933998872	Tumor_G2
10772	UCP2	9.150292364	Tumor_G2
10773	SOD2	4.774386031	Tumor_G2
10774	NOX4	2.48056371	Tumor_G2
10775	DUOX1	7.734998606	Tumor_G2
10776	DUOXA1	7.834318882	Tumor_G2
10777	DUOX2	8.610334333	Tumor_G2
10778	DUOXA2	5.910205963	Tumor_G2
10779	NDUFA1	7.981379527	Tumor_G2
10780	NDUFA2	7.212150157	Tumor_G2
10781	NDUFA8	7.515856609	Tumor_G2
10782	NDUFB1	5.90084782	Tumor_G2
10783	NDUFB10	7.996161197	Tumor_G2
10784	NDUFB2	6.982812138	Tumor_G2
10785	NDUFB3	6.334281199	Tumor_G2
10786	NDUFB7	9.221963	Tumor_G2
10787	CYC1	7.07605949	Tumor_G2
10788	ETFB	5.590225117	Tumor_G2
10789	UQCR10	7.940483497	Tumor_G2
10790	UQCRQ	7.566846367	Tumor_G2
10791	UQCC3	4.859566888	Tumor_G2
10792	COX15	4.702225167	Tumor_G2
10793	COX5A	6.873314371	Tumor_G2
10794	COX6B1	8.983535095	Tumor_G2
10795	COX6C	6.661712714	Tumor_G2
10796	COX7A2	6.889714512	Tumor_G2
10797	COX7B	6.185317059	Tumor_G2
10798	COX8A	9.935767304	Tumor_G2

10799	SCO1	3.342695222	Tumor_G2
10800	SURF1	6.85376288	Tumor_G2
10801	ATP6V1E1	6.696747274	Tumor_G2
10802	DLAT	4.582129125	Tumor_G2
10803	PDC	0.054376327	Tumor_G2
10804	SUCLG2	6.142415069	Tumor_G2
10805	SLC25A5	7.997073491	Tumor_G2
10806	UCP2	8.208534008	Tumor_G2
10807	SOD2	4.609490584	Tumor_G2
10808	NOX4	1.366080885	Tumor_G2
10809	DUOX1	7.576925197	Tumor_G2
10810	DUOXA1	7.241257426	Tumor_G2
10811	DUOX2	8.718984049	Tumor_G2
10812	DUOXA2	6.649306123	Tumor_G2
10813	NDUFA1	8.744470885	Tumor_G2
10814	NDUFA2	6.733138328	Tumor_G2
10815	NDUFA8	7.79639754	Tumor_G2
10816	NDUFB1	6.00318386	Tumor_G2
10817	NDUFB10	7.525863134	Tumor_G2
10818	NDUFB2	7.257686164	Tumor_G2
10819	NDUFB3	7.017726834	Tumor_G2
10820	NDUFB7	8.794095221	Tumor_G2
10821	CYC1	7.455859737	Tumor_G2
10822	ETFB	4.651004139	Tumor_G2
10823	UQCR10	8.349266855	Tumor_G2
10824	UQCRQ	7.613345031	Tumor_G2
10825	UQCC3	3.594192663	Tumor_G2
10826	COX15	5.150021256	Tumor_G2
10827	COX5A	7.973940079	Tumor_G2
10828	COX6B1	8.831659309	Tumor_G2
10829	COX6C	6.97187977	Tumor_G2
10830	COX7A2	7.573796851	Tumor_G2
10831	COX7B	7.412295745	Tumor_G2
10832	COX8A	9.863717037	Tumor_G2
10833	SCO1	3.783607417	Tumor_G2
10834	SURF1	5.516427694	Tumor_G2
10835	ATP6V1E1	6.642351601	Tumor_G2
10836	DLAT	5.833851922	Tumor_G2
10837	PDC	0.038945123	Tumor_G2
10838	SUCLG2	6.662300904	Tumor_G2

10839	SLC25A5	9.164469642	Tumor_G2
10840	UCP2	6.828357075	Tumor_G2
10841	SOD2	5.552926218	Tumor_G2
10842	NOX4	0.798469486	Tumor_G2
10843	DUOX1	5.337899925	Tumor_G2
10844	DUOXA1	5.195106377	Tumor_G2
10845	DUOX2	6.042964571	Tumor_G2
10846	DUOXA2	4.384239853	Tumor_G2
10847	NDUFA1	8.694671215	Tumor_G2
10848	NDUFA2	6.865563856	Tumor_G2
10849	NDUFA8	7.511399713	Tumor_G2
10850	NDUFB1	6.35111803	Tumor_G2
10851	NDUFB10	7.194580407	Tumor_G2
10852	NDUFB2	7.809126025	Tumor_G2
10853	NDUFB3	7.034081052	Tumor_G2
10854	NDUFB7	8.841067381	Tumor_G2
10855	CYC1	7.73169605	Tumor_G2
10856	ETFB	4.586005123	Tumor_G2
10857	UQCR10	8.22468496	Tumor_G2
10858	UQCRQ	7.65177454	Tumor_G2
10859	UQCC3	3.974088815	Tumor_G2
10860	COX15	5.059410213	Tumor_G2
10861	COX5A	7.776350369	Tumor_G2
10862	COX6B1	8.942892644	Tumor_G2
10863	COX6C	7.170700992	Tumor_G2
10864	COX7A2	7.592785395	Tumor_G2
10865	COX7B	7.104927069	Tumor_G2
10866	COX8A	10.36261045	Tumor_G2
10867	SCO1	3.41788842	Tumor_G2
10868	SURF1	6.118470308	Tumor_G2
10869	ATP6V1E1	7.165346775	Tumor_G2
10870	DLAT	5.505692194	Tumor_G2
10871	PDC	0	Tumor_G2
10872	SUCLG2	6.756892386	Tumor_G2
10873	SLC25A5	9.076137981	Tumor_G2
10874	UCP2	6.895063575	Tumor_G2
10875	SOD2	5.05612552	Tumor_G2
10876	NOX4	1.529035039	Tumor_G2
10877	DUOX1	5.708652793	Tumor_G2
10878	DUOXA1	4.928523202	Tumor_G2

10879	DUOX2	6.615001834	Tumor_G2
10880	DUOXA2	4.296191031	Tumor_G2
10881	NDUFA1	8.259103978	Tumor_G2
10882	NDUFA2	6.999729903	Tumor_G2
10883	NDUFA8	7.213273975	Tumor_G2
10884	NDUFB1	5.831688871	Tumor_G2
10885	NDUFB10	7.744837031	Tumor_G2
10886	NDUFB2	7.183129106	Tumor_G2
10887	NDUFB3	6.355538019	Tumor_G2
10888	NDUFB7	9.211375764	Tumor_G2
10889	CYC1	7.02958763	Tumor_G2
10890	ETFB	6.146787262	Tumor_G2
10891	UQCR10	7.324089518	Tumor_G2
10892	UQCRQ	7.716429255	Tumor_G2
10893	UQCC3	4.817109652	Tumor_G2
10894	COX15	4.675250928	Tumor_G2
10895	COX5A	7.03191359	Tumor_G2
10896	COX6B1	8.960139134	Tumor_G2
10897	COX6C	6.669427469	Tumor_G2
10898	COX7A2	7.180262773	Tumor_G2
10899	COX7B	6.130352668	Tumor_G2
10900	COX8A	9.546408007	Tumor_G2
10901	SCO1	3.320638339	Tumor_G2
10902	SURF1	6.923831555	Tumor_G2
10903	ATP6V1E1	6.932193801	Tumor_G2
10904	DLAT	4.783097742	Tumor_G2
10905	PDC	0.237994964	Tumor_G2
10906	SUCLG2	6.147888826	Tumor_G2
10907	SLC25A5	7.584974961	Tumor_G2
10908	UCP2	8.746274423	Tumor_G2
10909	SOD2	4.55791646	Tumor_G2
10910	NOX4	2.077432815	Tumor_G2
10911	DUOX1	6.834820838	Tumor_G2
10912	DUOXA1	7.101613701	Tumor_G2
10913	DUOX2	7.490706971	Tumor_G2
10914	DUOXA2	5.321043849	Tumor_G2
10915	NDUFA1	8.886160469	Tumor_G2
10916	NDUFA2	7.202658572	Tumor_G2
10917	NDUFA8	6.74770217	Tumor_G2
10918	NDUFB1	6.0557054	Tumor_G2

10919	NDUFB10	7.464888142	Tumor_G2
10920	NDUFB2	7.328972568	Tumor_G2
10921	NDUFB3	5.624429275	Tumor_G2
10922	NDUFB7	9.083543508	Tumor_G2
10923	CYC1	7.604933096	Tumor_G2
10924	ETFB	5.087246886	Tumor_G2
10925	UQCR10	7.003045602	Tumor_G2
10926	UQCRQ	7.74965076	Tumor_G2
10927	UQCC3	4.393764131	Tumor_G2
10928	COX15	4.873239845	Tumor_G2
10929	COX5A	7.14043616	Tumor_G2
10930	COX6B1	8.854600781	Tumor_G2
10931	COX6C	7.252958967	Tumor_G2
10932	COX7A2	7.092657784	Tumor_G2
10933	COX7B	6.915348712	Tumor_G2
10934	COX8A	10.07039723	Tumor_G2
10935	SCO1	3.480878306	Tumor_G2
10936	SURF1	5.604948906	Tumor_G2
10937	ATP6V1E1	5.949039072	Tumor_G2
10938	DLAT	5.277651018	Tumor_G2
10939	PDC	0	Tumor_G2
10940	SUCLG2	6.103533541	Tumor_G2
10941	SLC25A5	9.271205586	Tumor_G2
10942	UCP2	3.578067992	Tumor_G2
10943	SOD2	4.700031274	Tumor_G2
10944	NOX4	0.823044966	Tumor_G2
10945	DUOX1	4.278566286	Tumor_G2
10946	DUOXA1	3.848312943	Tumor_G2
10947	DUOX2	4.345248203	Tumor_G2
10948	DUOXA2	3.754735944	Tumor_G2
10949	NDUFA1	8.332183216	Tumor_G2
10950	NDUFA2	6.84829248	Tumor_G2
10951	NDUFA8	7.257118575	Tumor_G2
10952	NDUFB1	6.075557056	Tumor_G2
10953	NDUFB10	7.796914436	Tumor_G2
10954	NDUFB2	7.16051987	Tumor_G2
10955	NDUFB3	6.634280209	Tumor_G2
10956	NDUFB7	8.722358861	Tumor_G2
10957	CYC1	7.540179222	Tumor_G2
10958	ETFB	5.050755825	Tumor_G2

10959	UQCR10	7.93503336	Tumor_G2
10960	UQCRQ	7.564400102	Tumor_G2
10961	UQCC3	4.532650931	Tumor_G2
10962	COX15	5.265809498	Tumor_G2
10963	COX5A	7.820795141	Tumor_G2
10964	COX6B1	8.736936615	Tumor_G2
10965	COX6C	6.904933712	Tumor_G2
10966	COX7A2	7.266346541	Tumor_G2
10967	COX7B	6.468522291	Tumor_G2
10968	COX8A	9.696768421	Tumor_G2
10969	SCO1	3.824100302	Tumor_G2
10970	SURF1	6.840365891	Tumor_G2
10971	ATP6V1E1	7.457016819	Tumor_G2
10972	DLAT	5.621274496	Tumor_G2
10973	PDC	0.039991309	Tumor_G2
10974	SUCLG2	6.926505307	Tumor_G2
10975	SLC25A5	9.058668318	Tumor_G2
10976	UCP2	8.678531016	Tumor_G2
10977	SOD2	5.150067019	Tumor_G2
10978	NOX4	1.140066197	Tumor_G2
10979	DUOX1	6.243246411	Tumor_G2
10980	DUOXA1	6.410095635	Tumor_G2
10981	DUOX2	6.596722404	Tumor_G2
10982	DUOXA2	4.175082143	Tumor_G2
10983	NDUFA1	8.160954446	Tumor_G2
10984	NDUFA2	6.600533108	Tumor_G2
10985	NDUFA8	7.108376868	Tumor_G2
10986	NDUFB1	5.799462495	Tumor_G2
10987	NDUFB10	7.170115152	Tumor_G2
10988	NDUFB2	6.595525275	Tumor_G2
10989	NDUFB3	6.569494848	Tumor_G2
10990	NDUFB7	8.507502918	Tumor_G2
10991	CYC1	6.738434748	Tumor_G2
10992	ETFB	5.046944071	Tumor_G2
10993	UQCR10	7.747001468	Tumor_G2
10994	UQCRQ	7.249937655	Tumor_G2
10995	UQCC3	4.174740218	Tumor_G2
10996	COX15	5.164289901	Tumor_G2
10997	COX5A	7.057805184	Tumor_G2
10998	COX6B1	8.544321738	Tumor_G2

10999	COX6C	6.493479065	Tumor_G2
11000	COX7A2	6.927457476	Tumor_G2
11001	COX7B	6.162376345	Tumor_G2
11002	COX8A	8.938372733	Tumor_G2
11003	SCO1	3.629195391	Tumor_G2
11004	SURF1	6.221676351	Tumor_G2
11005	ATP6V1E1	7.026491489	Tumor_G2
11006	DLAT	5.329321497	Tumor_G2
11007	PDC	0.297522583	Tumor_G2
11008	SUCLG2	6.511210885	Tumor_G2
11009	SLC25A5	8.01187271	Tumor_G2
11010	UCP2	8.838511601	Tumor_G2
11011	SOD2	5.358776167	Tumor_G2
11012	NOX4	1.776026955	Tumor_G2
11013	DUOX1	7.091908591	Tumor_G2
11014	DUOXA1	7.064541643	Tumor_G2
11015	DUOX2	8.038772458	Tumor_G2
11016	DUOXA2	5.326276429	Tumor_G2
11017	NDUFA1	8.200044914	Tumor_G2
11018	NDUFA2	6.894737439	Tumor_G2
11019	NDUFA8	7.59902654	Tumor_G2
11020	NDUFB1	6.326358061	Tumor_G2
11021	NDUFB10	7.832501442	Tumor_G2
11022	NDUFB2	7.10145299	Tumor_G2
11023	NDUFB3	6.711357059	Tumor_G2
11024	NDUFB7	8.854827587	Tumor_G2
11025	CYC1	5.943325992	Tumor_G2
11026	ETFB	5.515196274	Tumor_G2
11027	UQCR10	8.008009478	Tumor_G2
11028	UQCRQ	7.706467286	Tumor_G2
11029	UQCC3	4.504278683	Tumor_G2
11030	COX15	4.633330185	Tumor_G2
11031	COX5A	7.391154826	Tumor_G2
11032	COX6B1	9.01819938	Tumor_G2
11033	COX6C	6.834596667	Tumor_G2
11034	COX7A2	7.291234556	Tumor_G2
11035	COX7B	6.57230441	Tumor_G2
11036	COX8A	9.746609558	Tumor_G2
11037	SCO1	3.291699918	Tumor_G2
11038	SURF1	6.518975368	Tumor_G2

11039	ATP6V1E1	6.633392899	Tumor_G2
11040	DLAT	4.832428803	Tumor_G2
11041	PDC	0.097873024	Tumor_G2
11042	SUCLG2	5.567803821	Tumor_G2
11043	SLC25A5	7.748722132	Tumor_G2
11044	UCP2	8.118369955	Tumor_G2
11045	SOD2	4.44859252	Tumor_G2
11046	NOX4	1.479505822	Tumor_G2
11047	DUOX1	7.339376667	Tumor_G2
11048	DUOXA1	6.775146138	Tumor_G2
11049	DUOX2	9.493024879	Tumor_G2
11050	DUOXA2	8.391772629	Tumor_G2
11051	NDUFA1	8.300259746	Tumor_G2
11052	NDUFA2	6.785756407	Tumor_G2
11053	NDUFA8	7.253320521	Tumor_G2
11054	NDUFB1	5.848780579	Tumor_G2
11055	NDUFB10	7.60717827	Tumor_G2
11056	NDUFB2	6.569657798	Tumor_G2
11057	NDUFB3	6.500841313	Tumor_G2
11058	NDUFB7	8.727980335	Tumor_G2
11059	CYC1	6.763927224	Tumor_G2
11060	ETFB	4.943778877	Tumor_G2
11061	UQCR10	7.795807466	Tumor_G2
11062	UQCRQ	7.353440022	Tumor_G2
11063	UQCC3	4.656983684	Tumor_G2
11064	COX15	4.984302633	Tumor_G2
11065	COX5A	7.071733487	Tumor_G2
11066	COX6B1	8.768609758	Tumor_G2
11067	COX6C	6.577756224	Tumor_G2
11068	COX7A2	6.964342835	Tumor_G2
11069	COX7B	6.060035624	Tumor_G2
11070	COX8A	9.395453486	Tumor_G2
11071	SCO1	3.388107558	Tumor_G2
11072	SURF1	6.742331867	Tumor_G2
11073	ATP6V1E1	7.07033877	Tumor_G2
11074	DLAT	5.095755943	Tumor_G2
11075	PDC	0.061219381	Tumor_G2
11076	SUCLG2	6.063795831	Tumor_G2
11077	SLC25A5	7.789784969	Tumor_G2
11078	UCP2	8.218761127	Tumor_G2

11079	SOD2	5.229192443	Tumor_G2
11080	NOX4	3.456588075	Tumor_G2
11081	DUOX1	6.145078235	Tumor_G2
11082	DUOXA1	6.320634794	Tumor_G2
11083	DUOX2	6.32618939	Tumor_G2
11084	DUOXA2	5.027318958	Tumor_G2
11085	NDUFA1	7.809444203	Tumor_G2
11086	NDUFA2	6.552465921	Tumor_G2
11087	NDUFA8	6.964747001	Tumor_G2
11088	NDUFB1	5.577000962	Tumor_G2
11089	NDUFB10	7.404759633	Tumor_G2
11090	NDUFB2	6.787317784	Tumor_G2
11091	NDUFB3	6.336255915	Tumor_G2
11092	NDUFB7	8.70452035	Tumor_G2
11093	CYC1	7.099523652	Tumor_G2
11094	ETFB	5.313161516	Tumor_G2
11095	UQCR10	7.580825475	Tumor_G2
11096	UQCRQ	7.171783755	Tumor_G2
11097	UQCC3	4.58078026	Tumor_G2
11098	COX15	4.92701858	Tumor_G2
11099	COX5A	7.012491229	Tumor_G2
11100	COX6B1	8.511793566	Tumor_G2
11101	COX6C	6.42697923	Tumor_G2
11102	COX7A2	6.673397318	Tumor_G2
11103	COX7B	5.883253824	Tumor_G2
11104	COX8A	9.620871721	Tumor_G2
11105	SCO1	3.317494333	Tumor_G2
11106	SURF1	6.472767815	Tumor_G2
11107	ATP6V1E1	6.921467413	Tumor_G2
11108	DLAT	5.069922789	Tumor_G2
11109	PDC	0.098529543	Tumor_G2
11110	SUCLG2	6.385263577	Tumor_G2
11111	SLC25A5	8.115106174	Tumor_G2
11112	UCP2	8.696025798	Tumor_G2
11113	SOD2	4.805142294	Tumor_G2
11114	NOX4	1.947355391	Tumor_G2
11115	DUOX1	7.406070871	Tumor_G2
11116	DUOXA1	7.124303615	Tumor_G2
11117	DUOX2	8.075175536	Tumor_G2
11118	DUOXA2	6.052817202	Tumor_G2

11119	NDUFA1	8.407947634	Tumor_G2
11120	NDUFA2	7.130407835	Tumor_G2
11121	NDUFA8	7.513347201	Tumor_G2
11122	NDUFB1	6.280410424	Tumor_G2
11123	NDUFB10	7.912234116	Tumor_G2
11124	NDUFB2	6.964825788	Tumor_G2
11125	NDUFB3	6.693758194	Tumor_G2
11126	NDUFB7	9.091336313	Tumor_G2
11127	CYC1	7.003927503	Tumor_G2
11128	ETFB	5.450866765	Tumor_G2
11129	UQCR10	8.142970043	Tumor_G2
11130	UQCRQ	7.849861321	Tumor_G2
11131	UQCC3	4.813838795	Tumor_G2
11132	COX15	5.116000814	Tumor_G2
11133	COX5A	7.231960304	Tumor_G2
11134	COX6B1	8.963911344	Tumor_G2
11135	COX6C	6.975893167	Tumor_G2
11136	COX7A2	7.094401266	Tumor_G2
11137	COX7B	6.513814978	Tumor_G2
11138	COX8A	9.341101932	Tumor_G2
11139	SCO1	3.394248248	Tumor_G2
11140	SURF1	7.077316804	Tumor_G2
11141	ATP6V1E1	7.140336445	Tumor_G2
11142	DLAT	5.251102169	Tumor_G2
11143	PDC	0	Tumor_G2
11144	SUCLG2	6.438293493	Tumor_G2
11145	SLC25A5	7.742947745	Tumor_G2
11146	UCP2	7.796196121	Tumor_G2
11147	SOD2	4.837840055	Tumor_G2
11148	NOX4	2.685116279	Tumor_G2
11149	DUOX1	7.343670688	Tumor_G2
11150	DUOXA1	7.376852899	Tumor_G2
11151	DUOX2	8.110848807	Tumor_G2
11152	DUOXA2	5.73227994	Tumor_G2
11153	NDUFA1	8.369904989	Tumor_G2
11154	NDUFA2	6.369632417	Tumor_G2
11155	NDUFA8	7.161543021	Tumor_G2
11156	NDUFB1	6.402119444	Tumor_G2
11157	NDUFB10	7.527897206	Tumor_G2
11158	NDUFB2	5.857166588	Tumor_G2

11159	NDUFB3	6.045730303	Tumor_G2
11160	NDUFB7	8.834708536	Tumor_G2
11161	CYC1	6.178146156	Tumor_G2
11162	ETFB	4.801730273	Tumor_G2
11163	UQCR10	7.848918718	Tumor_G2
11164	UQCRQ	7.072006958	Tumor_G2
11165	UQCC3	3.457873408	Tumor_G2
11166	COX15	4.032179619	Tumor_G2
11167	COX5A	6.533432401	Tumor_G2
11168	COX6B1	9.231501542	Tumor_G2
11169	COX6C	6.362375551	Tumor_G2
11170	COX7A2	6.777800507	Tumor_G2
11171	COX7B	6.703348812	Tumor_G2
11172	COX8A	8.377645072	Tumor_G2
11173	SCO1	2.706878851	Tumor_G2
11174	SURF1	6.367270548	Tumor_G2
11175	ATP6V1E1	6.165283653	Tumor_G2
11176	DLAT	3.704030544	Tumor_G2
11177	PDC	0	Tumor_G2
11178	SUCLG2	4.740299543	Tumor_G2
11179	SLC25A5	7.049380556	Tumor_G2
11180	UCP2	8.206836465	Tumor_G2
11181	SOD2	3.910517302	Tumor_G2
11182	NOX4	1.582436297	Tumor_G2
11183	DUOX1	7.187529828	Tumor_G2
11184	DUOXA1	7.006726719	Tumor_G2
11185	DUOX2	7.758488051	Tumor_G2
11186	DUOXA2	5.069904234	Tumor_G2
11187	NDUFA1	8.290898329	Tumor_G2
11188	NDUFA2	6.895876708	Tumor_G2
11189	NDUFA8	6.967324061	Tumor_G2
11190	NDUFB1	6.005064064	Tumor_G2
11191	NDUFB10	7.28626355	Tumor_G2
11192	NDUFB2	6.799590427	Tumor_G2
11193	NDUFB3	6.733257822	Tumor_G2
11194	NDUFB7	8.552595526	Tumor_G2
11195	CYC1	6.882458824	Tumor_G2
11196	ETFB	5.119327636	Tumor_G2
11197	UQCR10	7.029560864	Tumor_G2
11198	UQCRQ	7.155242854	Tumor_G2

11199	UQCC3	4.131417007	Tumor_G2
11200	COX15	5.310209353	Tumor_G2
11201	COX5A	7.123118069	Tumor_G2
11202	COX6B1	8.533288697	Tumor_G2
11203	COX6C	6.682634526	Tumor_G2
11204	COX7A2	7.066369211	Tumor_G2
11205	COX7B	6.186018978	Tumor_G2
11206	COX8A	10.04355926	Tumor_G2
11207	SCO1	4.21638457	Tumor_G2
11208	SURF1	6.494908584	Tumor_G2
11209	ATP6V1E1	6.705024941	Tumor_G2
11210	DLAT	5.300214194	Tumor_G2
11211	PDC	0.259529277	Tumor_G2
11212	SUCLG2	6.929273724	Tumor_G2
11213	SLC25A5	8.564691382	Tumor_G2
11214	UCP2	7.661433912	Tumor_G2
11215	SOD2	4.054203642	Tumor_G2
11216	NOX4	2.148740838	Tumor_G2
11217	DUOX1	8.397226162	Tumor_G2
11218	DUOXA1	7.346509559	Tumor_G2
11219	DUOX2	10.87726088	Tumor_G2
11220	DUOXA2	8.654387262	Tumor_G2
11221	NDUFA1	8.469552272	Tumor_G2
11222	NDUFA2	7.006181694	Tumor_G2
11223	NDUFA8	7.334878545	Tumor_G2
11224	NDUFB1	6.072217828	Tumor_G2
11225	NDUFB10	7.53588796	Tumor_G2
11226	NDUFB2	6.688341638	Tumor_G2
11227	NDUFB3	6.701418295	Tumor_G2
11228	NDUFB7	8.922146583	Tumor_G2
11229	CYC1	7.009590356	Tumor_G2
11230	ETFB	5.064799348	Tumor_G2
11231	UQCR10	8.015081368	Tumor_G2
11232	UQCRQ	7.264840675	Tumor_G2
11233	UQCC3	4.472641668	Tumor_G2
11234	COX15	5.412173483	Tumor_G2
11235	COX5A	7.15338765	Tumor_G2
11236	COX6B1	8.731196497	Tumor_G2
11237	COX6C	6.874400403	Tumor_G2
11238	COX7A2	7.006012652	Tumor_G2

11239	COX7B	6.131586837	Tumor_G2
11240	COX8A	9.314762379	Tumor_G2
11241	SCO1	3.876528089	Tumor_G2
11242	SURF1	6.651955138	Tumor_G2
11243	ATP6V1E1	7.022507787	Tumor_G2
11244	DLAT	5.459418803	Tumor_G2
11245	PDC	0.066494358	Tumor_G2
11246	SUCLG2	6.43053462	Tumor_G2
11247	SLC25A5	8.066745557	Tumor_G2
11248	UCP2	7.51758452	Tumor_G2
11249	SOD2	5.197705086	Tumor_G2
11250	NOX4	2.235470193	Tumor_G2
11251	DUOX1	6.515988971	Tumor_G2
11252	DUOXA1	6.423776737	Tumor_G2
11253	DUOX2	7.042013654	Tumor_G2
11254	DUOXA2	6.009137722	Tumor_G2
11255	NDUFA1	8.718731236	Tumor_G2
11256	NDUFA2	7.141471613	Tumor_G2
11257	NDUFA8	7.294114262	Tumor_G2
11258	NDUFB1	6.33126693	Tumor_G2
11259	NDUFB10	7.814239334	Tumor_G2
11260	NDUFB2	6.81849751	Tumor_G2
11261	NDUFB3	6.69415428	Tumor_G2
11262	NDUFB7	9.158007329	Tumor_G2
11263	CYC1	6.959298291	Tumor_G2
11264	ETFB	5.444476385	Tumor_G2
11265	UQCR10	8.164591757	Tumor_G2
11266	UQCRQ	7.76584529	Tumor_G2
11267	UQCC3	4.874098304	Tumor_G2
11268	COX15	4.74831982	Tumor_G2
11269	COX5A	7.388926233	Tumor_G2
11270	COX6B1	9.249071155	Tumor_G2
11271	COX6C	7.086580173	Tumor_G2
11272	COX7A2	7.17941393	Tumor_G2
11273	COX7B	6.371405184	Tumor_G2
11274	COX8A	9.590162163	Tumor_G2
11275	SCO1	3.116742867	Tumor_G2
11276	SURF1	7.017935441	Tumor_G2
11277	ATP6V1E1	7.030832434	Tumor_G2
11278	DLAT	4.956819386	Tumor_G2

11279	PDC	0	Tumor_G2
11280	SUCLG2	6.091474047	Tumor_G2
11281	SLC25A5	7.336801063	Tumor_G2
11282	UCP2	8.940627478	Tumor_G2
11283	SOD2	4.890776414	Tumor_G2
11284	NOX4	2.913060611	Tumor_G2
11285	DUOX1	6.375458729	Tumor_G2
11286	DUOXA1	6.937090104	Tumor_G2
11287	DUOX2	7.264854813	Tumor_G2
11288	DUOXA2	5.287003967	Tumor_G2
11289	NDUFA1	8.571141568	Tumor_G2
11290	NDUFA2	7.148395837	Tumor_G2
11291	NDUFA8	7.247871914	Tumor_G2
11292	NDUFB1	6.471234158	Tumor_G2
11293	NDUFB10	7.858064412	Tumor_G2
11294	NDUFB2	6.422145911	Tumor_G2
11295	NDUFB3	6.131428094	Tumor_G2
11296	NDUFB7	8.89974018	Tumor_G2
11297	CYC1	6.419418204	Tumor_G2
11298	ETFB	4.600307553	Tumor_G2
11299	UQCR10	7.548938326	Tumor_G2
11300	UQCRQ	7.759111428	Tumor_G2
11301	UQCC3	4.527766004	Tumor_G2
11302	COX15	4.437508279	Tumor_G2
11303	COX5A	7.525659801	Tumor_G2
11304	COX6B1	9.324072036	Tumor_G2
11305	COX6C	7.090820044	Tumor_G2
11306	COX7A2	7.213017777	Tumor_G2
11307	COX7B	6.379014317	Tumor_G2
11308	COX8A	9.188746384	Tumor_G2
11309	SCO1	2.698644941	Tumor_G2
11310	SURF1	6.510543203	Tumor_G2
11311	ATP6V1E1	6.344915471	Tumor_G2
11312	DLAT	4.943372838	Tumor_G2
11313	PDC	0.093307007	Tumor_G2
11314	SUCLG2	5.767131503	Tumor_G2
11315	SLC25A5	7.347551691	Tumor_G2
11316	UCP2	7.789485043	Tumor_G2
11317	SOD2	4.398376078	Tumor_G2
11318	NOX4	2.95570382	Tumor_G2

11319	DUOX1	5.5911418	Tumor_G2
11320	DUOXA1	6.099538657	Tumor_G2
11321	DUOX2	7.058300152	Tumor_G2
11322	DUOXA2	4.488954577	Tumor_G2
11323	NDUFA1	8.421266457	Tumor_G2
11324	NDUFA2	7.08316925	Tumor_G2
11325	NDUFA8	7.278413291	Tumor_G2
11326	NDUFB1	6.197785633	Tumor_G2
11327	NDUFB10	7.574123255	Tumor_G2
11328	NDUFB2	6.363981278	Tumor_G2
11329	NDUFB3	6.569512596	Tumor_G2
11330	NDUFB7	8.75375753	Tumor_G2
11331	CYC1	6.753843501	Tumor_G2
11332	ETFB	5.128436917	Tumor_G2
11333	UQCR10	8.05824547	Tumor_G2
11334	UQCRQ	7.811984593	Tumor_G2
11335	UQCC3	4.373881581	Tumor_G2
11336	COX15	4.657767417	Tumor_G2
11337	COX5A	7.439290402	Tumor_G2
11338	COX6B1	9.111275003	Tumor_G2
11339	COX6C	6.747844718	Tumor_G2
11340	COX7A2	7.175667639	Tumor_G2
11341	COX7B	6.409646258	Tumor_G2
11342	COX8A	9.339550559	Tumor_G2
11343	SCO1	3.333155754	Tumor_G2
11344	SURF1	6.72711197	Tumor_G2
11345	ATP6V1E1	6.794078667	Tumor_G2
11346	DLAT	5.121368641	Tumor_G2
11347	PDC	0	Tumor_G2
11348	SUCLG2	6.048089489	Tumor_G2
11349	SLC25A5	8.37307918	Tumor_G2
11350	UCP2	8.162302406	Tumor_G2
11351	SOD2	5.624064172	Tumor_G2
11352	NOX4	2.964410817	Tumor_G2
11353	DUOX1	6.227599179	Tumor_G2
11354	DUOXA1	6.112276986	Tumor_G2
11355	DUOX2	6.132978461	Tumor_G2
11356	DUOXA2	4.352325006	Tumor_G2
11357	NDUFA1	8.589501704	Tumor_G2
11358	NDUFA2	7.563046313	Tumor_G2

11359	NDUFA8	7.384426386	Tumor_G2
11360	NDUFB1	6.064567841	Tumor_G2
11361	NDUFB10	7.966493145	Tumor_G2
11362	NDUFB2	7.667895706	Tumor_G2
11363	NDUFB3	6.770593414	Tumor_G2
11364	NDUFB7	9.176619051	Tumor_G2
11365	CYC1	7.372997242	Tumor_G2
11366	ETFB	5.40584689	Tumor_G2
11367	UQCR10	8.025057166	Tumor_G2
11368	UQCRQ	7.689161032	Tumor_G2
11369	UQCC3	5.427848884	Tumor_G2
11370	COX15	5.20738891	Tumor_G2
11371	COX5A	7.314083217	Tumor_G2
11372	COX6B1	9.071626205	Tumor_G2
11373	COX6C	6.696763086	Tumor_G2
11374	COX7A2	7.09410647	Tumor_G2
11375	COX7B	6.278018761	Tumor_G2
11376	COX8A	10.07658805	Tumor_G2
11377	SCO1	3.598229914	Tumor_G2
11378	SURF1	7.059619846	Tumor_G2
11379	ATP6V1E1	7.148768795	Tumor_G2
11380	DLAT	5.360490112	Tumor_G2
11381	PDC	0	Tumor_G2
11382	SUCLG2	6.674137603	Tumor_G2
11383	SLC25A5	8.516654492	Tumor_G2
11384	UCP2	8.419433288	Tumor_G2
11385	SOD2	4.873106339	Tumor_G2
11386	NOX4	1.278038233	Tumor_G2
11387	DUOX1	7.005024854	Tumor_G2
11388	DUOXA1	6.528393494	Tumor_G2
11389	DUOX2	7.935835597	Tumor_G2
11390	DUOXA2	6.987527663	Tumor_G2
11391	NDUFA1	8.565882691	Tumor_G2
11392	NDUFA2	7.081529102	Tumor_G2
11393	NDUFA8	7.31180507	Tumor_G2
11394	NDUFB1	6.094463294	Tumor_G2
11395	NDUFB10	7.774359763	Tumor_G2
11396	NDUFB2	7.083848302	Tumor_G2
11397	NDUFB3	6.733301122	Tumor_G2
11398	NDUFB7	9.20612164	Tumor_G2

11399	CYC1	7.155580274	Tumor_G2
11400	ETFB	5.679680457	Tumor_G2
11401	UQCR10	8.075521271	Tumor_G2
11402	UQCRQ	7.501694081	Tumor_G2
11403	UQCC3	5.016333374	Tumor_G2
11404	COX15	5.038870899	Tumor_G2
11405	COX5A	7.10418968	Tumor_G2
11406	COX6B1	9.007363164	Tumor_G2
11407	COX6C	6.712507132	Tumor_G2
11408	COX7A2	6.969987388	Tumor_G2
11409	COX7B	6.254254821	Tumor_G2
11410	COX8A	9.908166751	Tumor_G2
11411	SCO1	3.637164111	Tumor_G2
11412	SURF1	6.665811305	Tumor_G2
11413	ATP6V1E1	7.134579942	Tumor_G2
11414	DLAT	4.977699302	Tumor_G2
11415	PDC	0.377658655	Tumor_G2
11416	SUCLG2	5.989094396	Tumor_G2
11417	SLC25A5	7.405558424	Tumor_G2
11418	UCP2	8.787111506	Tumor_G2
11419	SOD2	4.579758443	Tumor_G2
11420	NOX4	1.786878261	Tumor_G2
11421	DUOX1	6.925954669	Tumor_G2
11422	DUOXA1	7.3220197	Tumor_G2
11423	DUOX2	8.425099686	Tumor_G2
11424	DUOXA2	6.928075069	Tumor_G2
11425	NDUFA1	8.427590989	Tumor_G2
11426	NDUFA2	7.393635924	Tumor_G2
11427	NDUFA8	7.514585068	Tumor_G2
11428	NDUFB1	6.262567953	Tumor_G2
11429	NDUFB10	7.707203285	Tumor_G2
11430	NDUFB2	7.361775154	Tumor_G2
11431	NDUFB3	6.907446265	Tumor_G2
11432	NDUFB7	9.183881706	Tumor_G2
11433	CYC1	7.273348187	Tumor_G2
11434	ETFB	5.442107635	Tumor_G2
11435	UQCR10	8.410973536	Tumor_G2
11436	UQCRQ	7.891501166	Tumor_G2
11437	UQCC3	4.965189448	Tumor_G2
11438	COX15	5.252771174	Tumor_G2

11439	COX5A	7.451075734	Tumor_G2
11440	COX6B1	8.958484073	Tumor_G2
11441	COX6C	6.915306482	Tumor_G2
11442	COX7A2	7.316852194	Tumor_G2
11443	COX7B	6.660898532	Tumor_G2
11444	COX8A	10.10919769	Tumor_G2
11445	SCO1	3.658164948	Tumor_G2
11446	SURF1	6.845568717	Tumor_G2
11447	ATP6V1E1	7.05820217	Tumor_G2
11448	DLAT	5.404619581	Tumor_G2
11449	PDC	0.267940673	Tumor_G2
11450	SUCLG2	6.653908267	Tumor_G2
11451	SLC25A5	8.351472584	Tumor_G2
11452	UCP2	7.216768888	Tumor_G2
11453	SOD2	4.429462168	Tumor_G2
11454	NOX4	1.335805687	Tumor_G2
11455	DUOX1	8.699314088	Tumor_G2
11456	DUOXA1	7.997716812	Tumor_G2
11457	DUOX2	9.512765054	Tumor_G2
11458	DUOXA2	7.043046438	Tumor_G2
11459	NDUFA1	8.033043006	Tumor_G2
11460	NDUFA2	6.924442242	Tumor_G2
11461	NDUFA8	7.345071207	Tumor_G2
11462	NDUFB1	5.7092051	Tumor_G2
11463	NDUFB10	7.379684934	Tumor_G2
11464	NDUFB2	6.669328941	Tumor_G2
11465	NDUFB3	6.362588466	Tumor_G2
11466	NDUFB7	8.828445832	Tumor_G2
11467	CYC1	7.209756106	Tumor_G2
11468	ETFB	5.216045429	Tumor_G2
11469	UQCR10	7.045690985	Tumor_G2
11470	UQCRQ	7.258414192	Tumor_G2
11471	UQCC3	4.859432012	Tumor_G2
11472	COX15	4.971416778	Tumor_G2
11473	COX5A	6.893873544	Tumor_G2
11474	COX6B1	8.658333481	Tumor_G2
11475	COX6C	6.22657693	Tumor_G2
11476	COX7A2	7.09212974	Tumor_G2
11477	COX7B	6.052010291	Tumor_G2
11478	COX8A	9.538187297	Tumor_G2

11479	SCO1	3.558213033	Tumor_G2
11480	SURF1	6.561946368	Tumor_G2
11481	ATP6V1E1	6.51901551	Tumor_G2
11482	DLAT	5.097178143	Tumor_G2
11483	PDC	0.064263365	Tumor_G2
11484	SUCLG2	6.357689368	Tumor_G2
11485	SLC25A5	7.808857107	Tumor_G2
11486	UCP2	8.242840573	Tumor_G2
11487	SOD2	4.103387871	Tumor_G2
11488	NOX4	1.705408704	Tumor_G2
11489	DUOX1	7.185530406	Tumor_G2
11490	DUOXA1	7.542543506	Tumor_G2
11491	DUOX2	8.528943174	Tumor_G2
11492	DUOXA2	6.323204847	Tumor_G2
11493	NDUFA1	9.01074633	Tumor_G2
11494	NDUFA2	7.619894098	Tumor_G2
11495	NDUFA8	7.604472642	Tumor_G2
11496	NDUFB1	5.579386181	Tumor_G2
11497	NDUFB10	8.246979609	Tumor_G2
11498	NDUFB2	7.147005411	Tumor_G2
11499	NDUFB3	6.676006957	Tumor_G2
11500	NDUFB7	9.484470865	Tumor_G2
11501	CYC1	7.816277388	Tumor_G2
11502	ETFB	4.816130924	Tumor_G2
11503	UQCR10	8.284180924	Tumor_G2
11504	UQCRQ	8.507737062	Tumor_G2
11505	UQCC3	4.734252629	Tumor_G2
11506	COX15	4.915404538	Tumor_G2
11507	COX5A	8.560840369	Tumor_G2
11508	COX6B1	9.541774804	Tumor_G2
11509	COX6C	6.92282268	Tumor_G2
11510	COX7A2	7.78823861	Tumor_G2
11511	COX7B	8.136747427	Tumor_G2
11512	COX8A	10.73232606	Tumor_G2
11513	SCO1	3.858967989	Tumor_G2
11514	SURF1	6.061202198	Tumor_G2
11515	ATP6V1E1	6.924039776	Tumor_G2
11516	DLAT	6.181457355	Tumor_G2
11517	PDC	0.064680771	Tumor_G2
11518	SUCLG2	6.753016692	Tumor_G2

11519	SLC25A5	10.30099179	Tumor_G2
11520	UCP2	3.49088526	Tumor_G2
11521	SOD2	5.623470104	Tumor_G2
11522	NOX4	0.306481374	Tumor_G2
11523	DUOX1	3.964910127	Tumor_G2
11524	DUOXA1	3.738823766	Tumor_G2
11525	DUOX2	3.70802588	Tumor_G2
11526	DUOXA2	1.926244257	Tumor_G2
11527	NDUFA1	8.209692477	Tumor_G2
11528	NDUFA2	7.048215278	Tumor_G2
11529	NDUFA8	7.231035738	Tumor_G2
11530	NDUFB1	6.061907052	Tumor_G2
11531	NDUFB10	7.846605794	Tumor_G2
11532	NDUFB2	6.587030339	Tumor_G2
11533	NDUFB3	5.927288675	Tumor_G2
11534	NDUFB7	8.909369246	Tumor_G2
11535	CYC1	6.758467338	Tumor_G2
11536	ETFB	5.098827445	Tumor_G2
11537	UQCR10	7.814934643	Tumor_G2
11538	UQCRQ	7.659694718	Tumor_G2
11539	UQCC3	4.416823347	Tumor_G2
11540	COX15	3.935518021	Tumor_G2
11541	COX5A	7.253896253	Tumor_G2
11542	COX6B1	9.166101238	Tumor_G2
11543	COX6C	6.839748721	Tumor_G2
11544	COX7A2	6.870185351	Tumor_G2
11545	COX7B	6.105079803	Tumor_G2
11546	COX8A	9.509542128	Tumor_G2
11547	SCO1	2.223779494	Tumor_G2
11548	SURF1	6.481902735	Tumor_G2
11549	ATP6V1E1	6.868095208	Tumor_G2
11550	DLAT	4.045003568	Tumor_G2
11551	PDC	0.122436574	Tumor_G2
11552	SUCLG2	5.658543949	Tumor_G2
11553	SLC25A5	7.015125189	Tumor_G2
11554	UCP2	8.582263614	Tumor_G2
11555	SOD2	4.23804823	Tumor_G2
11556	NOX4	1.784438784	Tumor_G2
11557	DUOX1	5.364051428	Tumor_G2
11558	DUOXA1	5.645350285	Tumor_G2

11559	DUOX2	5.428044565	Tumor_G2
11560	DUOXA2	4.612687418	Tumor_G2
11561	NDUFA1	8.069725618	Tumor_G2
11562	NDUFA2	6.691594118	Tumor_G2
11563	NDUFA8	7.187461688	Tumor_G2
11564	NDUFB1	5.720915836	Tumor_G2
11565	NDUFB10	7.621425663	Tumor_G2
11566	NDUFB2	6.434597766	Tumor_G2
11567	NDUFB3	6.110804754	Tumor_G2
11568	NDUFB7	8.746507548	Tumor_G2
11569	CYC1	6.94830821	Tumor_G2
11570	ETFB	4.831678434	Tumor_G2
11571	UQCR10	7.207164188	Tumor_G2
11572	UQCRQ	7.316174079	Tumor_G2
11573	UQCC3	4.385676509	Tumor_G2
11574	COX15	4.485376357	Tumor_G2
11575	COX5A	7.266178459	Tumor_G2
11576	COX6B1	8.6445443	Tumor_G2
11577	COX6C	6.514735114	Tumor_G2
11578	COX7A2	6.83496162	Tumor_G2
11579	COX7B	6.143202089	Tumor_G2
11580	COX8A	9.526025506	Tumor_G2
11581	SCO1	3.060659186	Tumor_G2
11582	SURF1	6.497552469	Tumor_G2
11583	ATP6V1E1	6.298562793	Tumor_G2
11584	DLAT	4.641483571	Tumor_G2
11585	PDC	0.093818989	Tumor_G2
11586	SUCLG2	6.157033764	Tumor_G2
11587	SLC25A5	8.065599263	Tumor_G2
11588	UCP2	8.343348927	Tumor_G2
11589	SOD2	4.835298928	Tumor_G2
11590	NOX4	1.74589142	Tumor_G2
11591	DUOX1	6.436907855	Tumor_G2
11592	DUOXA1	6.751050564	Tumor_G2
11593	DUOX2	7.729996751	Tumor_G2
11594	DUOXA2	5.817474694	Tumor_G2
11595	NDUFA1	8.489849512	Tumor_G2
11596	NDUFA2	7.460662892	Tumor_G2
11597	NDUFA8	7.457369836	Tumor_G2
11598	NDUFB1	6.740878218	Tumor_G2

11599	NDUFB10	7.879423357	Tumor_G2
11600	NDUFB2	6.004532747	Tumor_G2
11601	NDUFB3	5.583911801	Tumor_G2
11602	NDUFB7	9.563621946	Tumor_G2
11603	CYC1	6.355035996	Tumor_G2
11604	ETFB	5.451450478	Tumor_G2
11605	UQCR10	7.150125842	Tumor_G2
11606	UQCRQ	8.009460063	Tumor_G2
11607	UQCC3	4.806217256	Tumor_G2
11608	COX15	3.80745743	Tumor_G2
11609	COX5A	6.612488561	Tumor_G2
11610	COX6B1	9.422936759	Tumor_G2
11611	COX6C	7.213339215	Tumor_G2
11612	COX7A2	6.744936383	Tumor_G2
11613	COX7B	6.28355002	Tumor_G2
11614	COX8A	9.375373012	Tumor_G2
11615	SCO1	1.917702179	Tumor_G2
11616	SURF1	6.288581408	Tumor_G2
11617	ATP6V1E1	5.166446285	Tumor_G2
11618	DLAT	3.53368724	Tumor_G2
11619	PDC	0.036473585	Tumor_G2
11620	SUCLG2	5.02673664	Tumor_G2
11621	SLC25A5	5.434968384	Tumor_G2
11622	UCP2	7.984717006	Tumor_G2
11623	SOD2	2.994339418	Tumor_G2
11624	NOX4	1.358090248	Tumor_G2
11625	DUOX1	7.456010053	Tumor_G2
11626	DUOXA1	7.792651179	Tumor_G2
11627	DUOX2	8.710453574	Tumor_G2
11628	DUOXA2	6.730636981	Tumor_G2
11629	NDUFA1	7.876242416	Tumor_G2
11630	NDUFA2	7.008759381	Tumor_G2
11631	NDUFA8	7.28414909	Tumor_G2
11632	NDUFB1	6.043900412	Tumor_G2
11633	NDUFB10	7.377012268	Tumor_G2
11634	NDUFB2	6.667493049	Tumor_G2
11635	NDUFB3	6.102355055	Tumor_G2
11636	NDUFB7	8.837322808	Tumor_G2
11637	CYC1	7.283864794	Tumor_G2
11638	ETFB	5.390736955	Tumor_G2

11639	UQCR10	6.947570883	Tumor_G2
11640	UQCRQ	7.926253485	Tumor_G2
11641	UQCC3	4.357761186	Tumor_G2
11642	COX15	4.583262614	Tumor_G2
11643	COX5A	7.316229854	Tumor_G2
11644	COX6B1	8.808650595	Tumor_G2
11645	COX6C	6.911283199	Tumor_G2
11646	COX7A2	6.925821178	Tumor_G2
11647	COX7B	6.273520444	Tumor_G2
11648	COX8A	9.833582704	Tumor_G2
11649	SCO1	3.049061318	Tumor_G2
11650	SURF1	6.197436706	Tumor_G2
11651	ATP6V1E1	6.038418385	Tumor_G2
11652	DLAT	4.777606281	Tumor_G2
11653	PDC	0.139707419	Tumor_G2
11654	SUCLG2	6.450382452	Tumor_G2
11655	SLC25A5	7.938012606	Tumor_G2
11656	UCP2	7.437942638	Tumor_G2
11657	SOD2	3.904709379	Tumor_G2
11658	NOX4	1.422598515	Tumor_G2
11659	DUOX1	7.241705361	Tumor_G2
11660	DUOXA1	6.545337454	Tumor_G2
11661	DUOX2	9.289384	Tumor_G2
11662	DUOXA2	7.641378547	Tumor_G2
11663	NDUFA1	7.611468004	Tumor_G2
11664	NDUFA2	6.563575927	Tumor_G2
11665	NDUFA8	7.169175859	Tumor_G2
11666	NDUFB1	5.502126933	Tumor_G2
11667	NDUFB10	7.530096296	Tumor_G2
11668	NDUFB2	6.564902054	Tumor_G2
11669	NDUFB3	6.301902804	Tumor_G2
11670	NDUFB7	8.655310319	Tumor_G2
11671	CYC1	6.999973138	Tumor_G2
11672	ETFB	5.21715618	Tumor_G2
11673	UQCR10	7.534374622	Tumor_G2
11674	UQCRQ	7.046842893	Tumor_G2
11675	UQCC3	4.611970358	Tumor_G2
11676	COX15	5.127209461	Tumor_G2
11677	COX5A	6.864332257	Tumor_G2
11678	COX6B1	8.671580178	Tumor_G2

11679	COX6C	6.294377892	Tumor_G2
11680	COX7A2	6.746317239	Tumor_G2
11681	COX7B	5.94478904	Tumor_G2
11682	COX8A	9.506514773	Tumor_G2
11683	SCO1	3.66300854	Tumor_G2
11684	SURF1	6.924440769	Tumor_G2
11685	ATP6V1E1	6.700856945	Tumor_G2
11686	DLAT	4.997169653	Tumor_G2
11687	PDC	0.145090634	Tumor_G2
11688	SUCLG2	6.262745583	Tumor_G2
11689	SLC25A5	8.305374688	Tumor_G2
11690	UCP2	8.512149805	Tumor_G2
11691	SOD2	5.077008645	Tumor_G2
11692	NOX4	2.161650791	Tumor_G2
11693	DUOX1	7.400629802	Tumor_G2
11694	DUOXA1	7.038206637	Tumor_G2
11695	DUOX2	8.335026657	Tumor_G2
11696	DUOXA2	7.41841089	Tumor_G2
11697	NDUFA1	8.422201859	Tumor_G2
11698	NDUFA2	7.131822757	Tumor_G2
11699	NDUFA8	6.962700011	Tumor_G2
11700	NDUFB1	6.03886392	Tumor_G2
11701	NDUFB10	7.578967994	Tumor_G2
11702	NDUFB2	6.716625983	Tumor_G2
11703	NDUFB3	6.465297603	Tumor_G2
11704	NDUFB7	8.740678552	Tumor_G2
11705	CYC1	7.385726076	Tumor_G2
11706	ETFB	4.846249363	Tumor_G2
11707	UQCR10	7.795049804	Tumor_G2
11708	UQCRQ	7.130796312	Tumor_G2
11709	UQCC3	4.524172802	Tumor_G2
11710	COX15	5.279324965	Tumor_G2
11711	COX5A	7.233584497	Tumor_G2
11712	COX6B1	8.790864691	Tumor_G2
11713	COX6C	6.539718041	Tumor_G2
11714	COX7A2	7.287711818	Tumor_G2
11715	COX7B	6.401515084	Tumor_G2
11716	COX8A	9.592423273	Tumor_G2
11717	SCO1	2.989308615	Tumor_G2
11718	SURF1	6.383648505	Tumor_G2

11719	ATP6V1E1	7.210355451	Tumor_G2
11720	DLAT	5.015688057	Tumor_G2
11721	PDC	0.122576191	Tumor_G2
11722	SUCLG2	6.31298977	Tumor_G2
11723	SLC25A5	9.263508848	Tumor_G2
11724	UCP2	6.562548225	Tumor_G2
11725	SOD2	5.042904743	Tumor_G2
11726	NOX4	1.327431838	Tumor_G2
11727	DUOX1	5.77592866	Tumor_G2
11728	DUOXA1	5.842620143	Tumor_G2
11729	DUOX2	5.542588836	Tumor_G2
11730	DUOXA2	3.954687419	Tumor_G2
11731	NDUFA1	8.225384415	Tumor_G2
11732	NDUFA2	6.722150488	Tumor_G2
11733	NDUFA8	7.296009412	Tumor_G2
11734	NDUFB1	6.062210669	Tumor_G2
11735	NDUFB10	7.28534929	Tumor_G2
11736	NDUFB2	6.880038763	Tumor_G2
11737	NDUFB3	6.535304	Tumor_G2
11738	NDUFB7	8.616248775	Tumor_G2
11739	CYC1	7.173097908	Tumor_G2
11740	ETFB	5.08507478	Tumor_G2
11741	UQCR10	7.898324876	Tumor_G2
11742	UQCRQ	7.503085033	Tumor_G2
11743	UQCC3	4.057764984	Tumor_G2
11744	COX15	4.630604671	Tumor_G2
11745	COX5A	7.623502409	Tumor_G2
11746	COX6B1	8.845648437	Tumor_G2
11747	COX6C	6.891217286	Tumor_G2
11748	COX7A2	7.161875578	Tumor_G2
11749	COX7B	6.637444552	Tumor_G2
11750	COX8A	10.02180426	Tumor_G2
11751	SCO1	2.751702869	Tumor_G2
11752	SURF1	5.958839929	Tumor_G2
11753	ATP6V1E1	6.907076959	Tumor_G2
11754	DLAT	4.814445546	Tumor_G2
11755	PDC	0.070025057	Tumor_G2
11756	SUCLG2	6.027021002	Tumor_G2
11757	SLC25A5	8.349638775	Tumor_G2
11758	UCP2	6.621287204	Tumor_G2

11759	SOD2	4.510642762	Tumor_G2
11760	NOX4	1.49606901	Tumor_G2
11761	DUOX1	6.625924022	Tumor_G2
11762	DUOXA1	5.766455459	Tumor_G2
11763	DUOX2	7.67314041	Tumor_G2
11764	DUOXA2	6.219431207	Tumor_G2
11765	NDUFA1	7.721545844	Tumor_G2
11766	NDUFA2	6.412187338	Tumor_G2
11767	NDUFA8	6.771327287	Tumor_G2
11768	NDUFB1	5.303300604	Tumor_G2
11769	NDUFB10	7.120287378	Tumor_G2
11770	NDUFB2	6.369333079	Tumor_G2
11771	NDUFB3	6.00139746	Tumor_G2
11772	NDUFB7	8.415277112	Tumor_G2
11773	CYC1	6.692423779	Tumor_G2
11774	ETFB	5.02609808	Tumor_G2
11775	UQCR10	7.289379668	Tumor_G2
11776	UQCRQ	6.976480043	Tumor_G2
11777	UQCC3	4.187390678	Tumor_G2
11778	COX15	4.619561005	Tumor_G2
11779	COX5A	6.637973471	Tumor_G2
11780	COX6B1	8.379379324	Tumor_G2
11781	COX6C	6.02373119	Tumor_G2
11782	COX7A2	6.478158732	Tumor_G2
11783	COX7B	5.798957761	Tumor_G2
11784	COX8A	9.34472892	Tumor_G2
11785	SCO1	3.174895821	Tumor_G2
11786	SURF1	6.148870997	Tumor_G2
11787	ATP6V1E1	6.897769344	Tumor_G2
11788	DLAT	4.919076962	Tumor_G2
11789	PDC	0.088932576	Tumor_G2
11790	SUCLG2	5.592538979	Tumor_G2
11791	SLC25A5	7.810300401	Tumor_G2
11792	UCP2	10.00984951	Tumor_G2
11793	SOD2	4.418015467	Tumor_G2
11794	NOX4	2.634446972	Tumor_G2
11795	DUOX1	7.054126525	Tumor_G2
11796	DUOXA1	7.135847389	Tumor_G2
11797	DUOX2	7.930264475	Tumor_G2
11798	DUOXA2	5.347027265	Tumor_G2

11799	NDUFA1	7.814527057	Tumor_G2
11800	NDUFA2	6.468842265	Tumor_G2
11801	NDUFA8	6.890513407	Tumor_G2
11802	NDUFB1	5.355205091	Tumor_G2
11803	NDUFB10	7.595035739	Tumor_G2
11804	NDUFB2	6.474801603	Tumor_G2
11805	NDUFB3	5.98957772	Tumor_G2
11806	NDUFB7	8.576117368	Tumor_G2
11807	CYC1	6.790897888	Tumor_G2
11808	ETFB	4.923371516	Tumor_G2
11809	UQCR10	7.502139764	Tumor_G2
11810	UQCRQ	7.051339502	Tumor_G2
11811	UQCC3	4.262498617	Tumor_G2
11812	COX15	4.914712762	Tumor_G2
11813	COX5A	6.914803484	Tumor_G2
11814	COX6B1	8.590127235	Tumor_G2
11815	COX6C	6.233445844	Tumor_G2
11816	COX7A2	6.617732814	Tumor_G2
11817	COX7B	5.731537436	Tumor_G2
11818	COX8A	9.454093406	Tumor_G2
11819	SCO1	3.1592664	Tumor_G2
11820	SURF1	6.109512465	Tumor_G2
11821	ATP6V1E1	7.131992464	Tumor_G2
11822	DLAT	4.960737318	Tumor_G2
11823	PDC	0	Tumor_G2
11824	SUCLG2	6.2999383	Tumor_G2
11825	SLC25A5	8.23227581	Tumor_G2
11826	UCP2	8.569808652	Tumor_G2
11827	SOD2	4.809320258	Tumor_G2
11828	NOX4	2.436900758	Tumor_G2
11829	DUOX1	6.856845962	Tumor_G2
11830	DUOXA1	6.766591661	Tumor_G2
11831	DUOX2	7.459693242	Tumor_G2
11832	DUOXA2	5.000093066	Tumor_G2
11833	NDUFA1	8.031953693	Tumor_G2
11834	NDUFA2	6.765375757	Tumor_G2
11835	NDUFA8	7.054476965	Tumor_G2
11836	NDUFB1	5.355641927	Tumor_G2
11837	NDUFB10	7.593140742	Tumor_G2
11838	NDUFB2	6.501638639	Tumor_G2

11839	NDUFB3	6.090001002	Tumor_G2
11840	NDUFB7	8.523667699	Tumor_G2
11841	CYC1	7.044647135	Tumor_G2
11842	ETFB	5.193927969	Tumor_G2
11843	UQCR10	6.931631424	Tumor_G2
11844	UQCRQ	6.986058198	Tumor_G2
11845	UQCC3	4.181296821	Tumor_G2
11846	COX15	5.284847907	Tumor_G2
11847	COX5A	7.088350625	Tumor_G2
11848	COX6B1	8.627059431	Tumor_G2
11849	COX6C	6.418608117	Tumor_G2
11850	COX7A2	6.791968063	Tumor_G2
11851	COX7B	5.877695088	Tumor_G2
11852	COX8A	9.483309347	Tumor_G2
11853	SCO1	3.166531378	Tumor_G2
11854	SURF1	6.075688324	Tumor_G2
11855	ATP6V1E1	6.487478785	Tumor_G2
11856	DLAT	5.287109916	Tumor_G2
11857	PDC	0.243734881	Tumor_G2
11858	SUCLG2	6.785062389	Tumor_G2
11859	SLC25A5	7.797498642	Tumor_G2
11860	UCP2	9.013244019	Tumor_G2
11861	SOD2	4.170980701	Tumor_G2
11862	NOX4	1.595214144	Tumor_G2
11863	DUOX1	6.861441218	Tumor_G2
11864	DUOXA1	6.830095683	Tumor_G2
11865	DUOX2	8.387200535	Tumor_G2
11866	DUOXA2	5.384699154	Tumor_G2
11867	NDUFA1	8.014932106	Tumor_G2
11868	NDUFA2	6.742387996	Tumor_G2
11869	NDUFA8	7.3499137	Tumor_G2
11870	NDUFB1	5.803238974	Tumor_G2
11871	NDUFB10	7.787658756	Tumor_G2
11872	NDUFB2	6.612464262	Tumor_G2
11873	NDUFB3	6.661266463	Tumor_G2
11874	NDUFB7	8.904100616	Tumor_G2
11875	CYC1	7.421211241	Tumor_G2
11876	ETFB	5.278785959	Tumor_G2
11877	UQCR10	7.739520861	Tumor_G2
11878	UQCRQ	7.382282739	Tumor_G2

11879	UQCC3	4.778332322	Tumor_G2
11880	COX15	5.446192789	Tumor_G2
11881	COX5A	7.182646632	Tumor_G2
11882	COX6B1	8.448839682	Tumor_G2
11883	COX6C	6.569793441	Tumor_G2
11884	COX7A2	7.003984772	Tumor_G2
11885	COX7B	5.86455967	Tumor_G2
11886	COX8A	9.764907564	Tumor_G2
11887	SCO1	4.333276065	Tumor_G2
11888	SURF1	6.6528492	Tumor_G2
11889	ATP6V1E1	6.868650608	Tumor_G2
11890	DLAT	5.457503538	Tumor_G2
11891	PDC	0.058466614	Tumor_G2
11892	SUCLG2	6.583948993	Tumor_G2
11893	SLC25A5	8.543452737	Tumor_G2
11894	UCP2	7.776417843	Tumor_G2
11895	SOD2	4.450176087	Tumor_G2
11896	NOX4	1.989363046	Tumor_G2
11897	DUOX1	7.439035264	Tumor_G2
11898	DUOXA1	7.31554154	Tumor_G2
11899	DUOX2	9.382672893	Tumor_G2
11900	DUOXA2	6.81065363	Tumor_G2
11901	NDUFA1	7.999567403	Tumor_G2
11902	NDUFA2	7.111834171	Tumor_G2
11903	NDUFA8	7.110015477	Tumor_G2
11904	NDUFB1	5.932293097	Tumor_G2
11905	NDUFB10	7.621373204	Tumor_G2
11906	NDUFB2	6.707102072	Tumor_G2
11907	NDUFB3	6.280692652	Tumor_G2
11908	NDUFB7	8.621104586	Tumor_G2
11909	CYC1	7.236033353	Tumor_G2
11910	ETFB	4.707366427	Tumor_G2
11911	UQCR10	7.755991952	Tumor_G2
11912	UQCRQ	7.320485825	Tumor_G2
11913	UQCC3	4.505347767	Tumor_G2
11914	COX15	4.56990773	Tumor_G2
11915	COX5A	7.610499576	Tumor_G2
11916	COX6B1	9.157185234	Tumor_G2
11917	COX6C	6.601003675	Tumor_G2
11918	COX7A2	7.153174318	Tumor_G2

11919	COX7B	6.182768522	Tumor_G2
11920	COX8A	9.88559185	Tumor_G2
11921	SCO1	3.097482908	Tumor_G2
11922	SURF1	6.662763977	Tumor_G2
11923	ATP6V1E1	7.596207105	Tumor_G2
11924	DLAT	4.867311994	Tumor_G2
11925	PDC	0.043881815	Tumor_G2
11926	SUCLG2	6.326023319	Tumor_G2
11927	SLC25A5	9.229925005	Tumor_G2
11928	UCP2	7.243467387	Tumor_G2
11929	SOD2	4.651731946	Tumor_G2
11930	NOX4	0.820942121	Tumor_G2
11931	DUOX1	4.200903648	Tumor_G2
11932	DUOXA1	4.519676209	Tumor_G2
11933	DUOX2	3.215424026	Tumor_G2
11934	DUOXA2	1.248711509	Tumor_G2
11935	NDUFA1	8.015386927	Tumor_G2
11936	NDUFA2	6.899696615	Tumor_G2
11937	NDUFA8	7.098909374	Tumor_G2
11938	NDUFB1	5.674845305	Tumor_G2
11939	NDUFB10	7.718997162	Tumor_G2
11940	NDUFB2	6.770093583	Tumor_G2
11941	NDUFB3	6.24105358	Tumor_G2
11942	NDUFB7	8.690727043	Tumor_G2
11943	CYC1	7.189726336	Tumor_G2
11944	ETFB	5.175723753	Tumor_G2
11945	UQCR10	7.674331178	Tumor_G2
11946	UQCRQ	7.223267452	Tumor_G2
11947	UQCC3	4.471625645	Tumor_G2
11948	COX15	4.852128685	Tumor_G2
11949	COX5A	7.259606759	Tumor_G2
11950	COX6B1	8.735932741	Tumor_G2
11951	COX6C	6.552110748	Tumor_G2
11952	COX7A2	6.996247491	Tumor_G2
11953	COX7B	6.053222731	Tumor_G2
11954	COX8A	9.469269104	Tumor_G2
11955	SCO1	3.065785717	Tumor_G2
11956	SURF1	6.151991178	Tumor_G2
11957	ATP6V1E1	7.511516286	Tumor_G2
11958	DLAT	5.097980799	Tumor_G2

11959	PDC	0.104617516	Tumor_G2
11960	SUCLG2	6.420179774	Tumor_G2
11961	SLC25A5	8.61707328	Tumor_G2
11962	UCP2	8.093253496	Tumor_G2
11963	SOD2	4.506172317	Tumor_G2
11964	NOX4	1.913447246	Tumor_G2
11965	DUOX1	6.018956456	Tumor_G2
11966	DUOXA1	6.052822131	Tumor_G2
11967	DUOX2	5.482578814	Tumor_G2
11968	DUOXA2	2.842145731	Tumor_G2
11969	NDUFA1	7.80362166	Tumor_G2
11970	NDUFA2	6.502217264	Tumor_G2
11971	NDUFA8	7.168097318	Tumor_G2
11972	NDUFB1	5.423643553	Tumor_G2
11973	NDUFB10	7.517927635	Tumor_G2
11974	NDUFB2	6.907279886	Tumor_G2
11975	NDUFB3	6.458079813	Tumor_G2
11976	NDUFB7	8.348737953	Tumor_G2
11977	CYC1	6.887244468	Tumor_G2
11978	ETFB	5.530971966	Tumor_G2
11979	UQCR10	7.627601304	Tumor_G2
11980	UQCRQ	6.946076894	Tumor_G2
11981	UQCC3	4.462952633	Tumor_G2
11982	COX15	4.999002388	Tumor_G2
11983	COX5A	7.017353875	Tumor_G2
11984	COX6B1	8.700973552	Tumor_G2
11985	COX6C	6.367199198	Tumor_G2
11986	COX7A2	6.681015564	Tumor_G2
11987	COX7B	6.030830196	Tumor_G2
11988	COX8A	9.530230153	Tumor_G2
11989	SCO1	3.469975046	Tumor_G2
11990	SURF1	6.654381143	Tumor_G2
11991	ATP6V1E1	7.020828166	Tumor_G2
11992	DLAT	5.157323546	Tumor_G2
11993	PDC	0.200974441	Tumor_G2
11994	SUCLG2	6.452601036	Tumor_G2
11995	SLC25A5	8.162771262	Tumor_G2
11996	UCP2	9.430250626	Tumor_G2
11997	SOD2	4.579929095	Tumor_G2
11998	NOX4	2.001501404	Tumor_G2

11999	DUOX1	6.939753151	Tumor_G2
12000	DUOXA1	6.973472087	Tumor_G2
12001	DUOX2	8.010578299	Tumor_G2
12002	DUOXA2	5.997486161	Tumor_G2
12003	NDUFA1	8.045864463	Tumor_G2
12004	NDUFA2	7.075413101	Tumor_G2
12005	NDUFA8	7.549883365	Tumor_G2
12006	NDUFB1	5.792756064	Tumor_G2
12007	NDUFB10	7.922561305	Tumor_G2
12008	NDUFB2	6.861027558	Tumor_G2
12009	NDUFB3	6.198816945	Tumor_G2
12010	NDUFB7	9.163522822	Tumor_G2
12011	CYC1	7.17815005	Tumor_G2
12012	ETFB	5.871422847	Tumor_G2
12013	UQCR10	7.353302887	Tumor_G2
12014	UQCRQ	7.570815235	Tumor_G2
12015	UQCC3	5.247607203	Tumor_G2
12016	COX15	4.981268751	Tumor_G2
12017	COX5A	7.11469541	Tumor_G2
12018	COX6B1	8.956837473	Tumor_G2
12019	COX6C	6.795905226	Tumor_G2
12020	COX7A2	7.079955512	Tumor_G2
12021	COX7B	6.240855341	Tumor_G2
12022	COX8A	10.23945446	Tumor_G2
12023	SCO1	3.189673901	Tumor_G2
12024	SURF1	7.003353983	Tumor_G2
12025	ATP6V1E1	6.582587064	Tumor_G2
12026	DLAT	4.981040169	Tumor_G2
12027	PDC	0.063425927	Tumor_G2
12028	SUCLG2	6.198692095	Tumor_G2
12029	SLC25A5	8.027567674	Tumor_G2
12030	UCP2	9.347937155	Tumor_G2
12031	SOD2	4.096447082	Tumor_G2
12032	NOX4	2.21499636	Tumor_G2
12033	DUOX1	7.916095872	Tumor_G2
12034	DUOXA1	7.907417892	Tumor_G2
12035	DUOX2	8.866700763	Tumor_G2
12036	DUOXA2	6.803786023	Tumor_G2
12037	NDUFA1	7.919698513	Tumor_G2
12038	NDUFA2	7.078507595	Tumor_G2

12039	NDUFA8	7.524281755	Tumor_G2
12040	NDUFB1	5.847213697	Tumor_G2
12041	NDUFB10	7.698752704	Tumor_G2
12042	NDUFB2	7.388067432	Tumor_G2
12043	NDUFB3	6.700745756	Tumor_G2
12044	NDUFB7	8.968022326	Tumor_G2
12045	CYC1	7.466088908	Tumor_G2
12046	ETFB	5.474680127	Tumor_G2
12047	UQCR10	8.056723622	Tumor_G2
12048	UQCRQ	7.583308768	Tumor_G2
12049	UQCC3	4.822562969	Tumor_G2
12050	COX15	5.542132092	Tumor_G2
12051	COX5A	7.319770051	Tumor_G2
12052	COX6B1	8.79234353	Tumor_G2
12053	COX6C	6.748145969	Tumor_G2
12054	COX7A2	7.060717738	Tumor_G2
12055	COX7B	6.535593809	Tumor_G2
12056	COX8A	10.1394848	Tumor_G2
12057	SCO1	4.227438233	Tumor_G2
12058	SURF1	7.063783062	Tumor_G2
12059	ATP6V1E1	7.090657331	Tumor_G2
12060	DLAT	5.695003387	Tumor_G2
12061	PDC	0.0623983	Tumor_G2
12062	SUCLG2	6.891380821	Tumor_G2
12063	SLC25A5	8.52456769	Tumor_G2
12064	UCP2	8.276942137	Tumor_G2
12065	SOD2	4.938240572	Tumor_G2
12066	NOX4	1.395641916	Tumor_G2
12067	DUOX1	8.323279963	Tumor_G2
12068	DUOXA1	7.501251875	Tumor_G2
12069	DUOX2	9.440209076	Tumor_G2
12070	DUOXA2	7.247887137	Tumor_G2
12071	NDUFA1	7.663267578	Tumor_G2
12072	NDUFA2	6.723865651	Tumor_G2
12073	NDUFA8	7.246029854	Tumor_G2
12074	NDUFB1	5.37389339	Tumor_G2
12075	NDUFB10	7.353791757	Tumor_G2
12076	NDUFB2	6.275794009	Tumor_G2
12077	NDUFB3	6.221631103	Tumor_G2
12078	NDUFB7	8.642126259	Tumor_G2

12079	CYC1	7.113953462	Tumor_G2
12080	ETFB	5.405112974	Tumor_G2
12081	UQCR10	7.56493742	Tumor_G2
12082	UQCRQ	7.172942104	Tumor_G2
12083	UQCC3	4.542540053	Tumor_G2
12084	COX15	4.8434723	Tumor_G2
12085	COX5A	7.136639171	Tumor_G2
12086	COX6B1	8.743465588	Tumor_G2
12087	COX6C	6.384457151	Tumor_G2
12088	COX7A2	6.796558886	Tumor_G2
12089	COX7B	6.108326606	Tumor_G2
12090	COX8A	9.208848267	Tumor_G2
12091	SCO1	3.300104945	Tumor_G2
12092	SURF1	6.409012829	Tumor_G2
12093	ATP6V1E1	6.837388575	Tumor_G2
12094	DLAT	5.416880218	Tumor_G2
12095	PDC	0.126466658	Tumor_G2
12096	SUCLG2	6.112405945	Tumor_G2
12097	SLC25A5	8.216773017	Tumor_G2
12098	UCP2	9.138798994	Tumor_G2
12099	SOD2	4.362187645	Tumor_G2
12100	NOX4	3.412699022	Tumor_G2
12101	DUOX1	6.163791974	Tumor_G2
12102	DUOXA1	6.455969182	Tumor_G2
12103	DUOX2	7.129905478	Tumor_G2
12104	DUOXA2	6.07735064	Tumor_G2
12105	NDUFA1	8.061333215	Tumor_G2
12106	NDUFA2	6.70634124	Tumor_G2
12107	NDUFA8	7.389328366	Tumor_G2
12108	NDUFB1	5.458537526	Tumor_G2
12109	NDUFB10	7.777736208	Tumor_G2
12110	NDUFB2	6.680847333	Tumor_G2
12111	NDUFB3	6.374885223	Tumor_G2
12112	NDUFB7	8.219435877	Tumor_G2
12113	CYC1	6.98009877	Tumor_G2
12114	ETFB	5.065520682	Tumor_G2
12115	UQCR10	7.635307408	Tumor_G2
12116	UQCRQ	7.229031198	Tumor_G2
12117	UQCC3	4.368547794	Tumor_G2
12118	COX15	5.138204895	Tumor_G2

12119	COX5A	7.091592014	Tumor_G2
12120	COX6B1	8.611672976	Tumor_G2
12121	COX6C	6.473949169	Tumor_G2
12122	COX7A2	6.853116302	Tumor_G2
12123	COX7B	6.357465679	Tumor_G2
12124	COX8A	9.661075646	Tumor_G2
12125	SCO1	3.553026903	Tumor_G2
12126	SURF1	6.567405745	Tumor_G2
12127	ATP6V1E1	6.610816623	Tumor_G2
12128	DLAT	5.484563361	Tumor_G2
12129	PDC	0.403775208	Tumor_G2
12130	SUCLG2	6.32952812	Tumor_G2
12131	SLC25A5	8.130039257	Tumor_G2
12132	UCP2	6.864729833	Tumor_G2
12133	SOD2	4.661982579	Tumor_G2
12134	NOX4	2.696020205	Tumor_G2
12135	DUOX1	7.719144952	Tumor_G2
12136	DUOXA1	7.362501201	Tumor_G2
12137	DUOX2	8.942024372	Tumor_G2
12138	DUOXA2	6.79299222	Tumor_G2
12139	NDUFA1	8.305766741	Tumor_G2
12140	NDUFA2	7.323306565	Tumor_G2
12141	NDUFA8	7.308820586	Tumor_G2
12142	NDUFB1	5.983751909	Tumor_G2
12143	NDUFB10	7.916895305	Tumor_G2
12144	NDUFB2	6.781075699	Tumor_G2
12145	NDUFB3	6.187090042	Tumor_G2
12146	NDUFB7	9.161344662	Tumor_G2
12147	CYC1	6.924857595	Tumor_G2
12148	ETFB	5.893040045	Tumor_G2
12149	UQCR10	7.241522758	Tumor_G2
12150	UQCRQ	7.81533904	Tumor_G2
12151	UQCC3	5.137701527	Tumor_G2
12152	COX15	4.331650623	Tumor_G2
12153	COX5A	7.034104188	Tumor_G2
12154	COX6B1	9.225944645	Tumor_G2
12155	COX6C	6.553699049	Tumor_G2
12156	COX7A2	6.9324063	Tumor_G2
12157	COX7B	6.103663887	Tumor_G2
12158	COX8A	9.946183945	Tumor_G2

12159	SCO1	2.583899445	Tumor_G2
12160	SURF1	6.930225275	Tumor_G2
12161	ATP6V1E1	5.952741901	Tumor_G2
12162	DLAT	3.929665687	Tumor_G2
12163	PDC	0	Tumor_G2
12164	SUCLG2	5.334343929	Tumor_G2
12165	SLC25A5	7.72207586	Tumor_G2
12166	UCP2	8.248739867	Tumor_G2
12167	SOD2	4.714221393	Tumor_G2
12168	NOX4	2.338433664	Tumor_G2
12169	DUOX1	6.457738047	Tumor_G2
12170	DUOXA1	7.093495085	Tumor_G2
12171	DUOX2	7.057168721	Tumor_G2
12172	DUOXA2	6.095348056	Tumor_G2
12173	NDUFA1	8.008168981	Tumor_G2
12174	NDUFA2	7.026932039	Tumor_G2
12175	NDUFA8	7.137384682	Tumor_G2
12176	NDUFB1	5.765153915	Tumor_G2
12177	NDUFB10	7.6262407	Tumor_G2
12178	NDUFB2	6.712149041	Tumor_G2
12179	NDUFB3	6.523528986	Tumor_G2
12180	NDUFB7	8.227775123	Tumor_G2
12181	CYC1	7.197454334	Tumor_G2
12182	ETFB	5.145615007	Tumor_G2
12183	UQCR10	7.678773178	Tumor_G2
12184	UQCRQ	7.343765266	Tumor_G2
12185	UQCC3	4.238707275	Tumor_G2
12186	COX15	5.055752771	Tumor_G2
12187	COX5A	7.081414964	Tumor_G2
12188	COX6B1	8.727726119	Tumor_G2
12189	COX6C	6.331677554	Tumor_G2
12190	COX7A2	6.846029736	Tumor_G2
12191	COX7B	6.116723905	Tumor_G2
12192	COX8A	9.706446067	Tumor_G2
12193	SCO1	3.153863546	Tumor_G2
12194	SURF1	6.012882954	Tumor_G2
12195	ATP6V1E1	7.420046927	Tumor_G2
12196	DLAT	5.334897173	Tumor_G2
12197	PDC	0	Tumor_G2
12198	SUCLG2	6.590122359	Tumor_G2

12199	SLC25A5	8.441383468	Tumor_G2
12200	UCP2	6.880884961	Tumor_G2
12201	SOD2	4.706477781	Tumor_G2
12202	NOX4	1.488289844	Tumor_G2
12203	DUOX1	6.756609479	Tumor_G2
12204	DUOXA1	6.611301595	Tumor_G2
12205	DUOX2	6.43667225	Tumor_G2
12206	DUOXA2	4.687615724	Tumor_G2
12207	NDUFA1	7.875603935	Tumor_G2
12208	NDUFA2	6.914049364	Tumor_G2
12209	NDUFA8	7.281635001	Tumor_G2
12210	NDUFB1	5.936433089	Tumor_G2
12211	NDUFB10	7.712192687	Tumor_G2
12212	NDUFB2	6.651323811	Tumor_G2
12213	NDUFB3	6.349546118	Tumor_G2
12214	NDUFB7	8.872186444	Tumor_G2
12215	CYC1	7.114407646	Tumor_G2
12216	ETFB	5.252817252	Tumor_G2
12217	UQCR10	7.757180841	Tumor_G2
12218	UQCRQ	7.531824926	Tumor_G2
12219	UQCC3	4.862695866	Tumor_G2
12220	COX15	4.996564931	Tumor_G2
12221	COX5A	7.055551964	Tumor_G2
12222	COX6B1	8.53941718	Tumor_G2
12223	COX6C	6.560283551	Tumor_G2
12224	COX7A2	6.93097583	Tumor_G2
12225	COX7B	6.293129389	Tumor_G2
12226	COX8A	9.65906388	Tumor_G2
12227	SCO1	3.468616969	Tumor_G2
12228	SURF1	6.35289374	Tumor_G2
12229	ATP6V1E1	6.96883321	Tumor_G2
12230	DLAT	5.266501869	Tumor_G2
12231	PDC	0.329951197	Tumor_G2
12232	SUCLG2	5.962973371	Tumor_G2
12233	SLC25A5	8.291146456	Tumor_G2
12234	UCP2	8.033200402	Tumor_G2
12235	SOD2	6.15925988	Tumor_G2
12236	NOX4	2.991667515	Tumor_G2
12237	DUOX1	7.056479304	Tumor_G2
12238	DUOXA1	7.159480084	Tumor_G2

12239	DUOX2	7.341961879	Tumor_G2
12240	DUOXA2	5.742604205	Tumor_G2
12241	NDUFA1	8.103789558	Tumor_G2
12242	NDUFA2	6.931647894	Tumor_G2
12243	NDUFA8	7.353483728	Tumor_G2
12244	NDUFB1	5.714670915	Tumor_G2
12245	NDUFB10	7.869350732	Tumor_G2
12246	NDUFB2	6.936268114	Tumor_G2
12247	NDUFB3	6.438791128	Tumor_G2
12248	NDUFB7	9.152661037	Tumor_G2
12249	CYC1	7.20733301	Tumor_G2
12250	ETFB	5.682484698	Tumor_G2
12251	UQCR10	7.812037116	Tumor_G2
12252	UQCRQ	7.415144768	Tumor_G2
12253	UQCC3	5.034557134	Tumor_G2
12254	COX15	5.050022015	Tumor_G2
12255	COX5A	7.073655839	Tumor_G2
12256	COX6B1	8.877594742	Tumor_G2
12257	COX6C	6.546011683	Tumor_G2
12258	COX7A2	7.096515477	Tumor_G2
12259	COX7B	6.232449236	Tumor_G2
12260	COX8A	9.888579371	Tumor_G2
12261	SCO1	3.275255399	Tumor_G2
12262	SURF1	7.054170624	Tumor_G2
12263	ATP6V1E1	7.11746483	Tumor_G2
12264	DLAT	5.040008707	Tumor_G2
12265	PDC	0.066734633	Tumor_G2
12266	SUCLG2	6.227227935	Tumor_G2
12267	SLC25A5	8.113808166	Tumor_G2
12268	UCP2	9.136231788	Tumor_G2
12269	SOD2	4.941013745	Tumor_G2
12270	NOX4	1.826916071	Tumor_G2
12271	DUOX1	7.02757945	Tumor_G2
12272	DUOXA1	6.971772713	Tumor_G2
12273	DUOX2	7.448166227	Tumor_G2
12274	DUOXA2	5.511853905	Tumor_G2
12275	NDUFA1	8.308722084	Tumor_G2
12276	NDUFA2	7.095387702	Tumor_G2
12277	NDUFA8	7.302000929	Tumor_G2
12278	NDUFB1	5.858375203	Tumor_G2

12279	NDUFB10	7.956707043	Tumor_G2
12280	NDUFB2	7.181550965	Tumor_G2
12281	NDUFB3	6.546129733	Tumor_G2
12282	NDUFB7	8.851342202	Tumor_G2
12283	CYC1	7.560646143	Tumor_G2
12284	ETFB	5.026801932	Tumor_G2
12285	UQCR10	7.843723458	Tumor_G2
12286	UQCRQ	7.493424223	Tumor_G2
12287	UQCC3	4.705202645	Tumor_G2
12288	COX15	5.532678765	Tumor_G2
12289	COX5A	7.615792955	Tumor_G2
12290	COX6B1	8.97838409	Tumor_G2
12291	COX6C	6.739364198	Tumor_G2
12292	COX7A2	7.090053007	Tumor_G2
12293	COX7B	6.556105102	Tumor_G2
12294	COX8A	9.733762325	Tumor_G2
12295	SCO1	3.51581965	Tumor_G2
12296	SURF1	6.786904034	Tumor_G2
12297	ATP6V1E1	7.554450893	Tumor_G2
12298	DLAT	5.495508034	Tumor_G2
12299	PDC	0	Tumor_G2
12300	SUCLG2	6.953902342	Tumor_G2
12301	SLC25A5	9.301305178	Tumor_G2
12302	UCP2	7.558319107	Tumor_G2
12303	SOD2	5.180948144	Tumor_G2
12304	NOX4	1.587668675	Tumor_G2
12305	DUOX1	6.165191077	Tumor_G2
12306	DUOXA1	6.130745626	Tumor_G2
12307	DUOX2	6.465856302	Tumor_G2
12308	DUOXA2	4.772372841	Tumor_G2
12309	NDUFA1	8.259081194	Tumor_G2
12310	NDUFA2	7.215084414	Tumor_G2
12311	NDUFA8	7.557116017	Tumor_G2
12312	NDUFB1	5.979687834	Tumor_G2
12313	NDUFB10	7.945857397	Tumor_G2
12314	NDUFB2	6.565360005	Tumor_G2
12315	NDUFB3	6.137810798	Tumor_G2
12316	NDUFB7	8.943471483	Tumor_G2
12317	CYC1	7.218630074	Tumor_G2
12318	ETFB	5.923336562	Tumor_G2

12319	UQCR10	7.416719409	Tumor_G2
12320	UQCRQ	7.627959246	Tumor_G2
12321	UQCC3	4.861816301	Tumor_G2
12322	COX15	4.819240203	Tumor_G2
12323	COX5A	7.102269568	Tumor_G2
12324	COX6B1	8.969555923	Tumor_G2
12325	COX6C	6.717584493	Tumor_G2
12326	COX7A2	6.914473717	Tumor_G2
12327	COX7B	6.327541056	Tumor_G2
12328	COX8A	9.726164758	Tumor_G2
12329	SCO1	2.916245901	Tumor_G2
12330	SURF1	6.627207755	Tumor_G2
12331	ATP6V1E1	6.408310397	Tumor_G2
12332	DLAT	4.769719523	Tumor_G2
12333	PDC	0.050658877	Tumor_G2
12334	SUCLG2	6.234352633	Tumor_G2
12335	SLC25A5	7.482119877	Tumor_G2
12336	UCP2	9.225003702	Tumor_G2
12337	SOD2	4.208296612	Tumor_G2
12338	NOX4	2.463018127	Tumor_G2
12339	DUOX1	6.58189581	Tumor_G2
12340	DUOXA1	6.763243848	Tumor_G2
12341	DUOX2	7.839723932	Tumor_G2
12342	DUOXA2	5.727239443	Tumor_G2
12343	NDUFA1	7.928894041	Tumor_G2
12344	NDUFA2	6.52420685	Tumor_G2
12345	NDUFA8	7.025316785	Tumor_G2
12346	NDUFB1	5.786676646	Tumor_G2
12347	NDUFB10	7.430397168	Tumor_G2
12348	NDUFB2	6.457264068	Tumor_G2
12349	NDUFB3	6.284845441	Tumor_G2
12350	NDUFB7	8.644008033	Tumor_G2
12351	CYC1	6.777333063	Tumor_G2
12352	ETFB	5.303018282	Tumor_G2
12353	UQCR10	7.554555324	Tumor_G2
12354	UQCRQ	7.260184828	Tumor_G2
12355	UQCC3	4.469098018	Tumor_G2
12356	COX15	4.758248837	Tumor_G2
12357	COX5A	6.967415385	Tumor_G2
12358	COX6B1	8.514209714	Tumor_G2

12359	COX6C	6.437645242	Tumor_G2
12360	COX7A2	6.769366262	Tumor_G2
12361	COX7B	5.985196048	Tumor_G2
12362	COX8A	9.250402983	Tumor_G2
12363	SCO1	3.372611826	Tumor_G2
12364	SURF1	6.621115091	Tumor_G2
12365	ATP6V1E1	7.030700644	Tumor_G2
12366	DLAT	5.167223941	Tumor_G2
12367	PDC	0.144695521	Tumor_G2
12368	SUCLG2	6.037210841	Tumor_G2
12369	SLC25A5	7.907348773	Tumor_G2
12370	UCP2	9.222532391	Tumor_G2
12371	SOD2	4.821580353	Tumor_G2
12372	NOX4	2.535245049	Tumor_G2
12373	DUOX1	6.997239193	Tumor_G2
12374	DUOXA1	7.199609967	Tumor_G2
12375	DUOX2	7.587410903	Tumor_G2
12376	DUOXA2	5.578696728	Tumor_G2
12377	NDUFA1	7.690319656	Tumor_G2
12378	NDUFA2	6.792140981	Tumor_G2
12379	NDUFA8	7.178544345	Tumor_G2
12380	NDUFB1	5.43509405	Tumor_G2
12381	NDUFB10	7.606152557	Tumor_G2
12382	NDUFB2	6.720394396	Tumor_G2
12383	NDUFB3	6.122378961	Tumor_G2
12384	NDUFB7	8.777840412	Tumor_G2
12385	CYC1	7.064030812	Tumor_G2
12386	ETFB	5.396536251	Tumor_G2
12387	UQCR10	7.573688652	Tumor_G2
12388	UQCRQ	7.237731502	Tumor_G2
12389	UQCC3	4.595716836	Tumor_G2
12390	COX15	5.01011337	Tumor_G2
12391	COX5A	6.985597907	Tumor_G2
12392	COX6B1	8.728758379	Tumor_G2
12393	COX6C	6.553844484	Tumor_G2
12394	COX7A2	6.825512429	Tumor_G2
12395	COX7B	6.052297074	Tumor_G2
12396	COX8A	9.392649273	Tumor_G2
12397	SCO1	3.442897431	Tumor_G2
12398	SURF1	6.768587908	Tumor_G2

12399	ATP6V1E1	7.427370541	Tumor_G2
12400	DLAT	5.378332637	Tumor_G2
12401	PDC	0.103434158	Tumor_G2
12402	SUCLG2	6.895946385	Tumor_G2
12403	SLC25A5	8.496177972	Tumor_G2
12404	UCP2	8.427177385	Tumor_G2
12405	SOD2	5.591321568	Tumor_G2
12406	NOX4	2.430756679	Tumor_G2
12407	DUOX1	6.936702599	Tumor_G2
12408	DUOXA1	7.329352144	Tumor_G2
12409	DUOX2	7.593143231	Tumor_G2
12410	DUOXA2	5.467124129	Tumor_G2
12411	NDUFA1	7.770087721	Tumor_G2
12412	NDUFA2	6.711240242	Tumor_G2
12413	NDUFA8	6.969717032	Tumor_G2
12414	NDUFB1	5.541112401	Tumor_G2
12415	NDUFB10	7.51740867	Tumor_G2
12416	NDUFB2	6.585276284	Tumor_G2
12417	NDUFB3	6.082287823	Tumor_G2
12418	NDUFB7	8.641655738	Tumor_G2
12419	CYC1	6.867560048	Tumor_G2
12420	ETFB	5.115508137	Tumor_G2
12421	UQCR10	7.532841632	Tumor_G2
12422	UQCRQ	7.335576852	Tumor_G2
12423	UQCC3	4.466238617	Tumor_G2
12424	COX15	4.890780612	Tumor_G2
12425	COX5A	7.067662172	Tumor_G2
12426	COX6B1	8.651913942	Tumor_G2
12427	COX6C	6.358998876	Tumor_G2
12428	COX7A2	7.021053138	Tumor_G2
12429	COX7B	6.101921767	Tumor_G2
12430	COX8A	9.537287501	Tumor_G2
12431	SCO1	3.477282187	Tumor_G2
12432	SURF1	6.51701291	Tumor_G2
12433	ATP6V1E1	7.066216148	Tumor_G2
12434	DLAT	5.535490247	Tumor_G2
12435	PDC	0.122221262	Tumor_G2
12436	SUCLG2	6.27731484	Tumor_G2
12437	SLC25A5	8.424553001	Tumor_G2
12438	UCP2	9.143546118	Tumor_G2

12439	SOD2	5.409181818	Tumor_G2
12440	NOX4	2.894818147	Tumor_G2
12441	DUOX1	7.113359432	Tumor_G2
12442	DUOXA1	7.153136242	Tumor_G2
12443	DUOX2	7.901775747	Tumor_G2
12444	DUOXA2	5.414347266	Tumor_G2
12445	NDUFA1	7.674260502	Tumor_G2
12446	NDUFA2	6.432335765	Tumor_G2
12447	NDUFA8	7.048305302	Tumor_G2
12448	NDUFB1	5.4651137	Tumor_G2
12449	NDUFB10	7.444959868	Tumor_G2
12450	NDUFB2	6.167025253	Tumor_G2
12451	NDUFB3	5.873109932	Tumor_G2
12452	NDUFB7	8.432151126	Tumor_G2
12453	CYC1	6.594308226	Tumor_G2
12454	ETFB	5.152412988	Tumor_G2
12455	UQCR10	7.424440988	Tumor_G2
12456	UQCRQ	6.92844992	Tumor_G2
12457	UQCC3	4.849733346	Tumor_G2
12458	COX15	5.279359974	Tumor_G2
12459	COX5A	6.60174507	Tumor_G2
12460	COX6B1	8.356142505	Tumor_G2
12461	COX6C	6.168934365	Tumor_G2
12462	COX7A2	6.468598611	Tumor_G2
12463	COX7B	5.907303429	Tumor_G2
12464	COX8A	9.48769547	Tumor_G2
12465	SCO1	3.483302956	Tumor_G2
12466	SURF1	6.521026005	Tumor_G2
12467	ATP6V1E1	6.688025785	Tumor_G2
12468	DLAT	5.463164726	Tumor_G2
12469	PDC	0.213417219	Tumor_G2
12470	SUCLG2	6.135332019	Tumor_G2
12471	SLC25A5	7.577755094	Tumor_G2
12472	UCP2	8.261190492	Tumor_G2
12473	SOD2	4.487256367	Tumor_G2
12474	NOX4	2.144121434	Tumor_G2
12475	DUOX1	7.741579048	Tumor_G2
12476	DUOXA1	7.738500631	Tumor_G2
12477	DUOX2	8.358878324	Tumor_G2
12478	DUOXA2	6.65417738	Tumor_G2

12479	NDUFA1	8.067409818	Tumor_G2
12480	NDUFA2	6.383160619	Tumor_G2
12481	NDUFA8	6.758070163	Tumor_G2
12482	NDUFB1	5.35977612	Tumor_G2
12483	NDUFB10	7.600589389	Tumor_G2
12484	NDUFB2	6.072157066	Tumor_G2
12485	NDUFB3	6.189206898	Tumor_G2
12486	NDUFB7	8.627178475	Tumor_G2
12487	CYC1	6.612086297	Tumor_G2
12488	ETFB	4.961543263	Tumor_G2
12489	UQCR10	7.398532051	Tumor_G2
12490	UQCRQ	6.998998604	Tumor_G2
12491	UQCC3	4.090716644	Tumor_G2
12492	COX15	4.991064269	Tumor_G2
12493	COX5A	6.737713378	Tumor_G2
12494	COX6B1	8.478902329	Tumor_G2
12495	COX6C	6.13222277	Tumor_G2
12496	COX7A2	6.570194816	Tumor_G2
12497	COX7B	5.851086384	Tumor_G2
12498	COX8A	9.510245052	Tumor_G2
12499	SCO1	3.301950153	Tumor_G2
12500	SURF1	6.683738644	Tumor_G2
12501	ATP6V1E1	6.957854453	Tumor_G2
12502	DLAT	4.914508759	Tumor_G2
12503	PDC	0.156903765	Tumor_G2
12504	SUCLG2	5.853156303	Tumor_G2
12505	SLC25A5	7.890143558	Tumor_G2
12506	UCP2	8.967070407	Tumor_G2
12507	SOD2	5.56818788	Tumor_G2
12508	NOX4	2.771795569	Tumor_G2
12509	DUOX1	6.789748653	Tumor_G2
12510	DUOXA1	6.785458208	Tumor_G2
12511	DUOX2	7.074571476	Tumor_G2
12512	DUOXA2	4.256305391	Tumor_G2
12513	NDUFA1	7.735549796	Tumor_G2
12514	NDUFA2	6.557632713	Tumor_G2
12515	NDUFA8	7.169133062	Tumor_G2
12516	NDUFB1	5.76529449	Tumor_G2
12517	NDUFB10	7.24197086	Tumor_G2
12518	NDUFB2	6.483313707	Tumor_G2

12519	NDUFB3	6.271742838	Tumor_G2
12520	NDUFB7	8.657841351	Tumor_G2
12521	CYC1	6.982237327	Tumor_G2
12522	ETFB	4.976922459	Tumor_G2
12523	UQCR10	7.48365985	Tumor_G2
12524	UQCRQ	7.042992882	Tumor_G2
12525	UQCC3	4.456764043	Tumor_G2
12526	COX15	4.74708924	Tumor_G2
12527	COX5A	6.749270414	Tumor_G2
12528	COX6B1	8.510116565	Tumor_G2
12529	COX6C	6.483466996	Tumor_G2
12530	COX7A2	6.638269418	Tumor_G2
12531	COX7B	6.061868121	Tumor_G2
12532	COX8A	9.483681048	Tumor_G2
12533	SCO1	3.675635377	Tumor_G2
12534	SURF1	6.766258538	Tumor_G2
12535	ATP6V1E1	6.575312243	Tumor_G2
12536	DLAT	5.135072881	Tumor_G2
12537	PDC	0.135900759	Tumor_G2
12538	SUCLG2	6.461873831	Tumor_G2
12539	SLC25A5	8.424810662	Tumor_G2
12540	UCP2	6.532119946	Tumor_G2
12541	SOD2	5.370724212	Tumor_G2
12542	NOX4	1.52445785	Tumor_G2
12543	DUOX1	7.486071835	Tumor_G2
12544	DUOXA1	7.299105472	Tumor_G2
12545	DUOX2	8.052580412	Tumor_G2
12546	DUOXA2	5.266560001	Tumor_G2
12547	NDUFA1	7.641228505	Tumor_G2
12548	NDUFA2	6.587807576	Tumor_G2
12549	NDUFA8	7.056925216	Tumor_G2
12550	NDUFB1	5.352921718	Tumor_G2
12551	NDUFB10	7.343456145	Tumor_G2
12552	NDUFB2	6.630972434	Tumor_G2
12553	NDUFB3	6.208658724	Tumor_G2
12554	NDUFB7	8.401399925	Tumor_G2
12555	CYC1	6.865539275	Tumor_G2
12556	ETFB	5.185189372	Tumor_G2
12557	UQCR10	7.543513814	Tumor_G2
12558	UQCRQ	7.15526684	Tumor_G2

12559	UQCC3	4.545922821	Tumor_G2
12560	COX15	5.169339656	Tumor_G2
12561	COX5A	7.001573275	Tumor_G2
12562	COX6B1	8.488649988	Tumor_G2
12563	COX6C	6.389314422	Tumor_G2
12564	COX7A2	6.676570206	Tumor_G2
12565	COX7B	6.104867087	Tumor_G2
12566	COX8A	9.382375578	Tumor_G2
12567	SCO1	3.656823839	Tumor_G2
12568	SURF1	6.600854666	Tumor_G2
12569	ATP6V1E1	6.938294791	Tumor_G2
12570	DLAT	5.708166824	Tumor_G2
12571	PDC	0.09946732	Tumor_G2
12572	SUCLG2	6.230323499	Tumor_G2
12573	SLC25A5	8.255036075	Tumor_G2
12574	UCP2	8.985877459	Tumor_G2
12575	SOD2	4.808059956	Tumor_G2
12576	NOX4	1.47888156	Tumor_G2
12577	DUOX1	6.361668391	Tumor_G2
12578	DUOXA1	6.376159	Tumor_G2
12579	DUOX2	6.781059878	Tumor_G2
12580	DUOXA2	5.434596192	Tumor_G2
12581	NDUFA1	8.12943319	Tumor_G2
12582	NDUFA2	7.322654259	Tumor_G2
12583	NDUFA8	7.515187695	Tumor_G2
12584	NDUFB1	5.891170475	Tumor_G2
12585	NDUFB10	8.067876366	Tumor_G2
12586	NDUFB2	7.576011126	Tumor_G2
12587	NDUFB3	6.409637188	Tumor_G2
12588	NDUFB7	9.377058825	Tumor_G2
12589	CYC1	7.731961752	Tumor_G2
12590	ETFB	5.688726237	Tumor_G2
12591	UQCR10	7.231152166	Tumor_G2
12592	UQCRQ	7.450414435	Tumor_G2
12593	UQCC3	4.462828523	Tumor_G2
12594	COX15	5.605440252	Tumor_G2
12595	COX5A	7.167862316	Tumor_G2
12596	COX6B1	8.98855326	Tumor_G2
12597	COX6C	6.868125708	Tumor_G2
12598	COX7A2	7.418514546	Tumor_G2

12599	COX7B	6.673428174	Tumor_G2
12600	COX8A	10.13971202	Tumor_G2
12601	SCO1	4.447874509	Tumor_G2
12602	SURF1	7.647258	Tumor_G2
12603	ATP6V1E1	6.998051366	Tumor_G2
12604	DLAT	5.806235195	Tumor_G2
12605	PDC	0.13136068	Tumor_G2
12606	SUCLG2	6.990676314	Tumor_G2
12607	SLC25A5	8.47854868	Tumor_G2
12608	UCP2	7.719182795	Tumor_G2
12609	SOD2	4.84078048	Tumor_G2
12610	NOX4	1.361991279	Tumor_G2
12611	DUOX1	7.236650071	Tumor_G2
12612	DUOXA1	6.965058636	Tumor_G2
12613	DUOX2	7.683091863	Tumor_G2
12614	DUOXA2	6.671421368	Tumor_G2
12615	NDUFA1	8.357083848	Tumor_G2
12616	NDUFA2	7.272816324	Tumor_G2
12617	NDUFA8	7.278739662	Tumor_G2
12618	NDUFB1	6.388338378	Tumor_G2
12619	NDUFB10	7.84851775	Tumor_G2
12620	NDUFB2	6.702345838	Tumor_G2
12621	NDUFB3	6.538256678	Tumor_G2
12622	NDUFB7	9.015536536	Tumor_G2
12623	CYC1	6.898220228	Tumor_G2
12624	ETFB	5.310798167	Tumor_G2
12625	UQCR10	8.051414942	Tumor_G2
12626	UQCRQ	7.971672992	Tumor_G2
12627	UQCC3	4.942370986	Tumor_G2
12628	COX15	4.579098419	Tumor_G2
12629	COX5A	7.289714041	Tumor_G2
12630	COX6B1	9.068225474	Tumor_G2
12631	COX6C	6.803288169	Tumor_G2
12632	COX7A2	7.061493666	Tumor_G2
12633	COX7B	6.580992117	Tumor_G2
12634	COX8A	9.637865822	Tumor_G2
12635	SCO1	3.160361262	Tumor_G2
12636	SURF1	6.22212324	Tumor_G2
12637	ATP6V1E1	6.670472369	Tumor_G2
12638	DLAT	5.08049559	Tumor_G2

12639	PDC	0	Tumor_G2
12640	SUCLG2	5.467136085	Tumor_G2
12641	SLC25A5	8.280297548	Tumor_G2
12642	UCP2	8.205319135	Tumor_G2
12643	SOD2	5.101611004	Tumor_G2
12644	NOX4	2.14588454	Tumor_G2
12645	DUOX1	6.875594368	Tumor_G2
12646	DUOXA1	6.947427443	Tumor_G2
12647	DUOX2	6.704223087	Tumor_G2
12648	DUOXA2	4.776351781	Tumor_G2
12649	NDUFA1	8.12382237	Tumor_G2
12650	NDUFA2	6.912053008	Tumor_G2
12651	NDUFA8	7.297626232	Tumor_G2
12652	NDUFB1	6.023002628	Tumor_G2
12653	NDUFB10	7.642915869	Tumor_G2
12654	NDUFB2	6.566504466	Tumor_G2
12655	NDUFB3	6.410616061	Tumor_G2
12656	NDUFB7	8.68935771	Tumor_G2
12657	CYC1	6.885032868	Tumor_G2
12658	ETFB	5.151016443	Tumor_G2
12659	UQCR10	7.413648641	Tumor_G2
12660	UQCRQ	7.461952675	Tumor_G2
12661	UQCC3	4.509516964	Tumor_G2
12662	COX15	4.717121622	Tumor_G2
12663	COX5A	6.999889129	Tumor_G2
12664	COX6B1	8.829581096	Tumor_G2
12665	COX6C	6.562858902	Tumor_G2
12666	COX7A2	6.916234575	Tumor_G2
12667	COX7B	6.222989598	Tumor_G2
12668	COX8A	9.392828853	Tumor_G2
12669	SCO1	3.231965798	Tumor_G2
12670	SURF1	6.085157312	Tumor_G2
12671	ATP6V1E1	6.549795092	Tumor_G2
12672	DLAT	4.775982299	Tumor_G2
12673	PDC	0.059706994	Tumor_G2
12674	SUCLG2	5.903687854	Tumor_G2
12675	SLC25A5	7.852041995	Tumor_G2
12676	UCP2	8.938022159	Tumor_G2
12677	SOD2	4.326753995	Tumor_G2
12678	NOX4	2.64244219	Tumor_G2

12679	DUOX1	6.748143587	Tumor_G2
12680	DUOXA1	6.961890703	Tumor_G2
12681	DUOX2	7.388225782	Tumor_G2
12682	DUOXA2	5.290016121	Tumor_G2
12683	NDUFA1	8.094862185	Tumor_G2
12684	NDUFA2	6.955840624	Tumor_G2
12685	NDUFA8	7.352849351	Tumor_G2
12686	NDUFB1	6.051665311	Tumor_G2
12687	NDUFB10	7.699946262	Tumor_G2
12688	NDUFB2	7.065198283	Tumor_G2
12689	NDUFB3	6.62575948	Tumor_G2
12690	NDUFB7	8.991455053	Tumor_G2
12691	CYC1	7.235082816	Tumor_G2
12692	ETFB	5.358015359	Tumor_G2
12693	UQCR10	7.924680964	Tumor_G2
12694	UQCRQ	7.545478035	Tumor_G2
12695	UQCC3	4.801171777	Tumor_G2
12696	COX15	4.968705416	Tumor_G2
12697	COX5A	7.105697193	Tumor_G2
12698	COX6B1	8.716810084	Tumor_G2
12699	COX6C	6.75823094	Tumor_G2
12700	COX7A2	7.130293069	Tumor_G2
12701	COX7B	6.3081978	Tumor_G2
12702	COX8A	10.05199455	Tumor_G2
12703	SCO1	3.828386236	Tumor_G2
12704	SURF1	6.83851569	Tumor_G2
12705	ATP6V1E1	6.841024374	Tumor_G2
12706	DLAT	5.313897532	Tumor_G2
12707	PDC	0.094003642	Tumor_G2
12708	SUCLG2	6.509513305	Tumor_G2
12709	SLC25A5	8.427521159	Tumor_G2
12710	UCP2	5.540264503	Tumor_G2
12711	SOD2	4.592072796	Tumor_G2
12712	NOX4	0.90499376	Tumor_G2
12713	DUOX1	7.701252151	Tumor_G2
12714	DUOXA1	7.251953303	Tumor_G2
12715	DUOX2	8.813622848	Tumor_G2
12716	DUOXA2	6.917565147	Tumor_G2
12717	NDUFA1	7.743076219	Tumor_G2
12718	NDUFA2	6.857531885	Tumor_G2

12719	NDUFA8	5.729679624	Tumor_G2
12720	NDUFB1	5.45753949	Tumor_G2
12721	NDUFB10	7.689873033	Tumor_G2
12722	NDUFB2	6.396275705	Tumor_G2
12723	NDUFB3	6.359917366	Tumor_G2
12724	NDUFB7	8.677008421	Tumor_G2
12725	CYC1	7.321570029	Tumor_G2
12726	ETFB	4.617107337	Tumor_G2
12727	UQCR10	7.567357984	Tumor_G2
12728	UQCRQ	7.224831007	Tumor_G2
12729	UQCC3	4.069434691	Tumor_G2
12730	COX15	5.13024408	Tumor_G2
12731	COX5A	7.352583573	Tumor_G2
12732	COX6B1	8.609821456	Tumor_G2
12733	COX6C	6.315083959	Tumor_G2
12734	COX7A2	6.870930039	Tumor_G2
12735	COX7B	5.839925765	Tumor_G2
12736	COX8A	9.778038201	Tumor_G2
12737	SCO1	3.12306887	Tumor_G2
12738	SURF1	5.213120897	Tumor_G2
12739	ATP6V1E1	7.370085617	Tumor_G2
12740	DLAT	5.516495359	Tumor_G2
12741	PDC	0.118605943	Tumor_G2
12742	SUCLG2	7.197812596	Tumor_G2
12743	SLC25A5	8.283522902	Tumor_G2
12744	UCP2	8.045465551	Tumor_G2
12745	SOD2	4.729390537	Tumor_G2
12746	NOX4	0.674458033	Tumor_G2
12747	DUOX1	7.127990829	Tumor_G2
12748	DUOXA1	6.505246631	Tumor_G2
12749	DUOX2	6.899939536	Tumor_G2
12750	DUOXA2	4.103258154	Tumor_G2
12751	NDUFA1	8.293779224	Tumor_G2
12752	NDUFA2	6.93531568	Tumor_G2
12753	NDUFA8	7.254926215	Tumor_G2
12754	NDUFB1	5.737417295	Tumor_G2
12755	NDUFB10	7.835103372	Tumor_G2
12756	NDUFB2	6.93495718	Tumor_G2
12757	NDUFB3	6.381373185	Tumor_G2
12758	NDUFB7	8.948573251	Tumor_G2

12759	CYC1	7.01371497	Tumor_G2
12760	ETFB	5.274087332	Tumor_G2
12761	UQCR10	7.897579586	Tumor_G2
12762	UQCRQ	7.556915048	Tumor_G2
12763	UQCC3	4.565442978	Tumor_G2
12764	COX15	4.887189915	Tumor_G2
12765	COX5A	7.282920815	Tumor_G2
12766	COX6B1	8.88170368	Tumor_G2
12767	COX6C	6.482257556	Tumor_G2
12768	COX7A2	6.993864994	Tumor_G2
12769	COX7B	6.084863242	Tumor_G2
12770	COX8A	9.7480625	Tumor_G2
12771	SCO1	3.208875484	Tumor_G2
12772	SURF1	6.330107881	Tumor_G2
12773	ATP6V1E1	7.045330251	Tumor_G2
12774	DLAT	4.81353287	Tumor_G2
12775	PDC	0.056655792	Tumor_G2
12776	SUCLG2	6.195492396	Tumor_G2
12777	SLC25A5	8.270023248	Tumor_G2
12778	UCP2	8.240157261	Tumor_G2
12779	SOD2	4.997573269	Tumor_G2
12780	NOX4	1.267598781	Tumor_G2
12781	DUOX1	6.255184639	Tumor_G2
12782	DUOXA1	6.397555841	Tumor_G2
12783	DUOX2	6.331035679	Tumor_G2
12784	DUOXA2	4.906828288	Tumor_G2
12785	NDUFA1	7.968238385	Tumor_G2
12786	NDUFA2	6.368749556	Tumor_G2
12787	NDUFA8	7.13218712	Tumor_G2
12788	NDUFB1	5.3700668	Tumor_G2
12789	NDUFB10	7.425980518	Tumor_G2
12790	NDUFB2	6.831254414	Tumor_G2
12791	NDUFB3	6.065487466	Tumor_G2
12792	NDUFB7	8.462044724	Tumor_G2
12793	CYC1	7.003054236	Tumor_G2
12794	ETFB	4.879451278	Tumor_G2
12795	UQCR10	7.301913321	Tumor_G2
12796	UQCRQ	7.047004919	Tumor_G2
12797	UQCC3	4.359237908	Tumor_G2
12798	COX15	5.203071521	Tumor_G2

12799	COX5A	7.208093496	Tumor_G2
12800	COX6B1	8.491923016	Tumor_G2
12801	COX6C	6.225373261	Tumor_G2
12802	COX7A2	6.58259403	Tumor_G2
12803	COX7B	6.125156163	Tumor_G2
12804	COX8A	9.557646821	Tumor_G2
12805	SCO1	3.645954535	Tumor_G2
12806	SURF1	6.360162831	Tumor_G2
12807	ATP6V1E1	7.157352567	Tumor_G2
12808	DLAT	5.117641247	Tumor_G2
12809	PDC	0.271300184	Tumor_G2
12810	SUCLG2	6.56512431	Tumor_G2
12811	SLC25A5	8.350452929	Tumor_G2
12812	UCP2	6.915819525	Tumor_G2
12813	SOD2	4.977728873	Tumor_G2
12814	NOX4	1.130366131	Tumor_G2
12815	DUOX1	6.084738417	Tumor_G2
12816	DUOXA1	6.176899981	Tumor_G2
12817	DUOX2	6.55122053	Tumor_G2
12818	DUOXA2	4.300934776	Tumor_G2
12819	NDUFA1	7.843009569	Tumor_G2
12820	NDUFA2	6.490226374	Tumor_G2
12821	NDUFA8	7.009675276	Tumor_G2
12822	NDUFB1	5.467156707	Tumor_G2
12823	NDUFB10	7.47339296	Tumor_G2
12824	NDUFB2	6.623387017	Tumor_G2
12825	NDUFB3	6.143816229	Tumor_G2
12826	NDUFB7	8.491205698	Tumor_G2
12827	CYC1	7.018331336	Tumor_G2
12828	ETFB	5.190285354	Tumor_G2
12829	UQCR10	7.362396771	Tumor_G2
12830	UQCRQ	7.128998108	Tumor_G2
12831	UQCC3	4.207656018	Tumor_G2
12832	COX15	4.940706537	Tumor_G2
12833	COX5A	6.97361186	Tumor_G2
12834	COX6B1	8.631655998	Tumor_G2
12835	COX6C	6.20098389	Tumor_G2
12836	COX7A2	6.657165831	Tumor_G2
12837	COX7B	5.92527289	Tumor_G2
12838	COX8A	9.5694861	Tumor_G2

12839	SCO1	3.279013566	Tumor_G2
12840	SURF1	6.087454151	Tumor_G2
12841	ATP6V1E1	7.069069515	Tumor_G2
12842	DLAT	5.113262814	Tumor_G2
12843	PDC	0.057191517	Tumor_G2
12844	SUCLG2	6.162973908	Tumor_G2
12845	SLC25A5	8.159474951	Tumor_G2
12846	UCP2	8.868090714	Tumor_G2
12847	SOD2	4.762773374	Tumor_G2
12848	NOX4	2.952655228	Tumor_G2
12849	DUOX1	6.683478841	Tumor_G2
12850	DUOXA1	6.608511013	Tumor_G2
12851	DUOX2	6.916102549	Tumor_G2
12852	DUOXA2	4.739666534	Tumor_G2
12853	NDUFA1	7.979619687	Tumor_G2
12854	NDUFA2	6.581529153	Tumor_G2
12855	NDUFA8	7.348928495	Tumor_G2
12856	NDUFB1	5.588767667	Tumor_G2
12857	NDUFB10	7.725390832	Tumor_G2
12858	NDUFB2	6.942624556	Tumor_G2
12859	NDUFB3	6.27644679	Tumor_G2
12860	NDUFB7	8.765626781	Tumor_G2
12861	CYC1	7.123407999	Tumor_G2
12862	ETFB	5.187110034	Tumor_G2
12863	UQCR10	7.613600752	Tumor_G2
12864	UQCRQ	7.349330776	Tumor_G2
12865	UQCC3	4.410143772	Tumor_G2
12866	COX15	4.879984345	Tumor_G2
12867	COX5A	7.470721651	Tumor_G2
12868	COX6B1	8.795726128	Tumor_G2
12869	COX6C	6.454297851	Tumor_G2
12870	COX7A2	6.956855024	Tumor_G2
12871	COX7B	6.188178046	Tumor_G2
12872	COX8A	9.747567371	Tumor_G2
12873	SCO1	2.963785752	Tumor_G2
12874	SURF1	6.624198306	Tumor_G2
12875	ATP6V1E1	7.291098303	Tumor_G2
12876	DLAT	5.133955935	Tumor_G2
12877	PDC	0	Tumor_G2
12878	SUCLG2	6.218728424	Tumor_G2

12879	SLC25A5	8.487097996	Tumor_G2
12880	UCP2	9.584309137	Tumor_G2
12881	SOD2	4.343864101	Tumor_G2
12882	NOX4	1.772067657	Tumor_G2
12883	DUOX1	6.346267279	Tumor_G2
12884	DUOXA1	6.79523499	Tumor_G2
12885	DUOX2	6.701713141	Tumor_G2
12886	DUOXA2	4.368773482	Tumor_G2
12887	NDUFA1	8.754563952	Tumor_G2
12888	NDUFA2	7.37600341	Tumor_G2
12889	NDUFA8	7.475046148	Tumor_G2
12890	NDUFB1	6.821403107	Tumor_G2
12891	NDUFB10	8.05836747	Tumor_G2
12892	NDUFB2	6.404416156	Tumor_G2
12893	NDUFB3	6.053348528	Tumor_G2
12894	NDUFB7	9.546604424	Tumor_G2
12895	CYC1	6.631739474	Tumor_G2
12896	ETFB	5.204267243	Tumor_G2
12897	UQCR10	7.899304273	Tumor_G2
12898	UQCRQ	7.872332574	Tumor_G2
12899	UQCC3	4.805791124	Tumor_G2
12900	COX15	3.922060828	Tumor_G2
12901	COX5A	7.139001093	Tumor_G2
12902	COX6B1	9.49518865	Tumor_G2
12903	COX6C	7.184645785	Tumor_G2
12904	COX7A2	6.897012524	Tumor_G2
12905	COX7B	6.417725156	Tumor_G2
12906	COX8A	9.342703256	Tumor_G2
12907	SCO1	2.393748004	Tumor_G2
12908	SURF1	6.333997198	Tumor_G2
12909	ATP6V1E1	6.206984307	Tumor_G2
12910	DLAT	3.671433759	Tumor_G2
12911	PDC	0	Tumor_G2
12912	SUCLG2	5.109004786	Tumor_G2
12913	SLC25A5	6.739399019	Tumor_G2
12914	UCP2	7.330554569	Tumor_G2
12915	SOD2	3.747131391	Tumor_G2
12916	NOX4	2.094201031	Tumor_G2
12917	DUOX1	7.693770917	Tumor_G2
12918	DUOXA1	7.684793616	Tumor_G2

12919	DUOX2	8.077667017	Tumor_G2
12920	DUOXA2	6.648107895	Tumor_G2
12921	NDUFA1	8.48464552	Tumor_G2
12922	NDUFA2	7.128305574	Tumor_G2
12923	NDUFA8	7.143838751	Tumor_G2
12924	NDUFB1	6.334444026	Tumor_G2
12925	NDUFB10	7.678880422	Tumor_G2
12926	NDUFB2	6.397402385	Tumor_G2
12927	NDUFB3	5.724504858	Tumor_G2
12928	NDUFB7	9.048773876	Tumor_G2
12929	CYC1	6.16877458	Tumor_G2
12930	ETFB	4.803023804	Tumor_G2
12931	UQCR10	7.805214397	Tumor_G2
12932	UQCRQ	7.522785925	Tumor_G2
12933	UQCC3	4.168015348	Tumor_G2
12934	COX15	3.645342915	Tumor_G2
12935	COX5A	6.810538489	Tumor_G2
12936	COX6B1	9.289818331	Tumor_G2
12937	COX6C	6.90449543	Tumor_G2
12938	COX7A2	6.684826939	Tumor_G2
12939	COX7B	6.170623111	Tumor_G2
12940	COX8A	9.031261403	Tumor_G2
12941	SCO1	1.997154321	Tumor_G2
12942	SURF1	6.056513571	Tumor_G2
12943	ATP6V1E1	6.299636651	Tumor_G2
12944	DLAT	3.747477912	Tumor_G2
12945	PDC	0	Tumor_G2
12946	SUCLG2	5.002834246	Tumor_G2
12947	SLC25A5	6.429160017	Tumor_G2
12948	UCP2	8.473833188	Tumor_G2
12949	SOD2	3.692026998	Tumor_G2
12950	NOX4	1.569968105	Tumor_G2
12951	DUOX1	5.585592699	Tumor_G2
12952	DUOXA1	6.043363884	Tumor_G2
12953	DUOX2	6.278765264	Tumor_G2
12954	DUOXA2	4.761788258	Tumor_G2
12955	NDUFA1	8.493188601	Tumor_G2
12956	NDUFA2	7.048358983	Tumor_G2
12957	NDUFA8	7.405636701	Tumor_G2
12958	NDUFB1	6.242771148	Tumor_G2

12959	NDUFB10	7.813394015	Tumor_G2
12960	NDUFB2	6.938328137	Tumor_G2
12961	NDUFB3	6.733073864	Tumor_G2
12962	NDUFB7	9.391592048	Tumor_G2
12963	CYC1	7.313540019	Tumor_G2
12964	ETFB	5.67632004	Tumor_G2
12965	UQCR10	8.06412718	Tumor_G2
12966	UQCRQ	7.687327924	Tumor_G2
12967	UQCC3	4.992533656	Tumor_G2
12968	COX15	4.789952327	Tumor_G2
12969	COX5A	7.066521362	Tumor_G2
12970	COX6B1	9.015543807	Tumor_G2
12971	COX6C	6.838978497	Tumor_G2
12972	COX7A2	7.126142071	Tumor_G2
12973	COX7B	6.277709648	Tumor_G2
12974	COX8A	9.758293519	Tumor_G2
12975	SCO1	3.239422823	Tumor_G2
12976	SURF1	6.89634229	Tumor_G2
12977	ATP6V1E1	7.11669024	Tumor_G2
12978	DLAT	4.638830182	Tumor_G2
12979	PDC	0	Tumor_G2
12980	SUCLG2	5.932201794	Tumor_G2
12981	SLC25A5	8.011966648	Tumor_G2
12982	UCP2	8.397104578	Tumor_G2
12983	SOD2	4.054284694	Tumor_G2
12984	NOX4	2.093349431	Tumor_G2
12985	DUOX1	7.70678185	Tumor_G2
12986	DUOXA1	7.469982337	Tumor_G2
12987	DUOX2	8.375002919	Tumor_G2
12988	DUOXA2	6.151405001	Tumor_G2
12989	NDUFA1	8.004651848	Tumor_G2
12990	NDUFA2	6.553030391	Tumor_G2
12991	NDUFA8	6.932956174	Tumor_G2
12992	NDUFB1	5.632526763	Tumor_G2
12993	NDUFB10	7.449268641	Tumor_G2
12994	NDUFB2	6.50689203	Tumor_G2
12995	NDUFB3	6.392028043	Tumor_G2
12996	NDUFB7	8.75327964	Tumor_G2
12997	CYC1	6.929726094	Tumor_G2
12998	ETFB	5.093993453	Tumor_G2

12999	UQCR10	7.705018724	Tumor_G2
13000	UQCRQ	7.246456436	Tumor_G2
13001	UQCC3	4.750277362	Tumor_G2
13002	COX15	4.562290492	Tumor_G2
13003	COX5A	6.965263075	Tumor_G2
13004	COX6B1	8.555548559	Tumor_G2
13005	COX6C	6.363546563	Tumor_G2
13006	COX7A2	6.835829363	Tumor_G2
13007	COX7B	6.00240879	Tumor_G2
13008	COX8A	9.569238847	Tumor_G2
13009	SCO1	3.524181507	Tumor_G2
13010	SURF1	6.429048951	Tumor_G2
13011	ATP6V1E1	7.066893501	Tumor_G2
13012	DLAT	5.010728626	Tumor_G2
13013	PDC	0.219978678	Tumor_G2
13014	SUCLG2	6.034364035	Tumor_G2
13015	SLC25A5	7.750245773	Tumor_G2
13016	UCP2	9.727650664	Tumor_G2
13017	SOD2	4.709945328	Tumor_G2
13018	NOX4	2.940217277	Tumor_G2
13019	DUOX1	7.992541494	Tumor_G2
13020	DUOXA1	7.936362805	Tumor_G2
13021	DUOX2	8.647903028	Tumor_G2
13022	DUOXA2	5.9749015	Tumor_G2
13023	NDUFA1	8.194301732	Tumor_G2
13024	NDUFA2	6.789029518	Tumor_G2
13025	NDUFA8	7.283471518	Tumor_G2
13026	NDUFB1	5.719187534	Tumor_G2
13027	NDUFB10	7.322585296	Tumor_G2
13028	NDUFB2	6.890392248	Tumor_G2
13029	NDUFB3	6.603994313	Tumor_G2
13030	NDUFB7	8.58649849	Tumor_G2
13031	CYC1	7.016496278	Tumor_G2
13032	ETFB	5.243647154	Tumor_G2
13033	UQCR10	7.731871943	Tumor_G2
13034	UQCRQ	7.43747166	Tumor_G2
13035	UQCC3	3.745482712	Tumor_G2
13036	COX15	4.861204157	Tumor_G2
13037	COX5A	7.195923093	Tumor_G2
13038	COX6B1	8.863161162	Tumor_G2

13039	COX6C	6.651469968	Tumor_G2
13040	COX7A2	7.343750864	Tumor_G2
13041	COX7B	6.467770679	Tumor_G2
13042	COX8A	9.129708986	Tumor_G2
13043	SCO1	3.226752964	Tumor_G2
13044	SURF1	6.58559315	Tumor_G2
13045	ATP6V1E1	7.372100498	Tumor_G2
13046	DLAT	5.494897991	Tumor_G2
13047	PDC	0.048513094	Tumor_G2
13048	SUCLG2	6.462172986	Tumor_G2
13049	SLC25A5	8.676511788	Tumor_G2
13050	UCP2	9.010905926	Tumor_G2
13051	SOD2	5.488047935	Tumor_G2
13052	NOX4	3.307639985	Tumor_G2
13053	DUOX1	6.006686704	Tumor_G2
13054	DUOXA1	6.299230898	Tumor_G2
13055	DUOX2	5.89321893	Tumor_G2
13056	DUOXA2	3.314230514	Tumor_G2
13057	NDUFA1	8.660301382	Tumor_G2
13058	NDUFA2	7.419216298	Tumor_G2
13059	NDUFA8	6.809120271	Tumor_G2
13060	NDUFB1	6.412012829	Tumor_G2
13061	NDUFB10	7.632425632	Tumor_G2
13062	NDUFB2	7.069355646	Tumor_G2
13063	NDUFB3	6.747851214	Tumor_G2
13064	NDUFB7	9.481590735	Tumor_G2
13065	CYC1	7.012728716	Tumor_G2
13066	ETFB	5.356638628	Tumor_G2
13067	UQCR10	8.126842408	Tumor_G2
13068	UQCRQ	8.090454056	Tumor_G2
13069	UQCC3	4.612468507	Tumor_G2
13070	COX15	4.187255892	Tumor_G2
13071	COX5A	7.215599301	Tumor_G2
13072	COX6B1	9.137719718	Tumor_G2
13073	COX6C	7.070662269	Tumor_G2
13074	COX7A2	7.307602094	Tumor_G2
13075	COX7B	6.551770992	Tumor_G2
13076	COX8A	9.955731121	Tumor_G2
13077	SCO1	2.803312461	Tumor_G2
13078	SURF1	6.198281675	Tumor_G2

13079	ATP6V1E1	7.207239429	Tumor_G2
13080	DLAT	4.584549661	Tumor_G2
13081	PDC	0.138926182	Tumor_G2
13082	SUCLG2	6.155336255	Tumor_G2
13083	SLC25A5	7.683230896	Tumor_G2
13084	UCP2	9.770431898	Tumor_G2
13085	SOD2	4.198329522	Tumor_G2
13086	NOX4	2.012387142	Tumor_G2
13087	DUOX1	6.518894633	Tumor_G2
13088	DUOXA1	6.292464659	Tumor_G2
13089	DUOX2	7.129052432	Tumor_G2
13090	DUOXA2	4.913994999	Tumor_G2
13091	NDUFA1	8.3496172	Tumor_G2
13092	NDUFA2	7.232231126	Tumor_G2
13093	NDUFA8	7.133437711	Tumor_G2
13094	NDUFB1	6.148137331	Tumor_G2
13095	NDUFB10	7.775651324	Tumor_G2
13096	NDUFB2	6.025786397	Tumor_G2
13097	NDUFB3	5.304498025	Tumor_G2
13098	NDUFB7	9.375857099	Tumor_G2
13099	CYC1	6.433029195	Tumor_G2
13100	ETFB	5.043437917	Tumor_G2
13101	UQCR10	7.65791983	Tumor_G2
13102	UQCRQ	7.266113312	Tumor_G2
13103	UQCC3	4.934751779	Tumor_G2
13104	COX15	4.106120132	Tumor_G2
13105	COX5A	6.363724581	Tumor_G2
13106	COX6B1	9.254289789	Tumor_G2
13107	COX6C	6.575565778	Tumor_G2
13108	COX7A2	6.251578589	Tumor_G2
13109	COX7B	5.872106901	Tumor_G2
13110	COX8A	8.974473615	Tumor_G2
13111	SCO1	2.073939562	Tumor_G2
13112	SURF1	6.3619841	Tumor_G2
13113	ATP6V1E1	6.074915768	Tumor_G2
13114	DLAT	3.760942324	Tumor_G2
13115	PDC	0.440624404	Tumor_G2
13116	SUCLG2	5.316527351	Tumor_G2
13117	SLC25A5	5.857194975	Tumor_G2
13118	UCP2	7.562371473	Tumor_G2

13119	SOD2	3.395441767	Tumor_G2
13120	NOX4	2.216701262	Tumor_G2
13121	DUOX1	7.21213474	Tumor_G2
13122	DUOXA1	7.235337067	Tumor_G2
13123	DUOX2	7.557219979	Tumor_G2
13124	DUOXA2	6.471423586	Tumor_G2
13125	NDUFA1	8.193895045	Tumor_G2
13126	NDUFA2	6.700112604	Tumor_G2
13127	NDUFA8	7.02069994	Tumor_G2
13128	NDUFB1	5.617325142	Tumor_G2
13129	NDUFB10	7.805571436	Tumor_G2
13130	NDUFB2	6.473023232	Tumor_G2
13131	NDUFB3	6.241460885	Tumor_G2
13132	NDUFB7	8.97980514	Tumor_G2
13133	CYC1	6.828149651	Tumor_G2
13134	ETFB	5.048753673	Tumor_G2
13135	UQCR10	7.511190451	Tumor_G2
13136	UQCRQ	7.207021391	Tumor_G2
13137	UQCC3	4.199790044	Tumor_G2
13138	COX15	4.68105597	Tumor_G2
13139	COX5A	7.286456367	Tumor_G2
13140	COX6B1	8.760894839	Tumor_G2
13141	COX6C	6.429175307	Tumor_G2
13142	COX7A2	6.80287401	Tumor_G2
13143	COX7B	6.1025777	Tumor_G2
13144	COX8A	9.608330599	Tumor_G2
13145	SCO1	3.241580415	Tumor_G2
13146	SURF1	6.096040964	Tumor_G2
13147	ATP6V1E1	6.69916042	Tumor_G2
13148	DLAT	4.670642212	Tumor_G2
13149	PDC	0.226762831	Tumor_G2
13150	SUCLG2	5.477846832	Tumor_G2
13151	SLC25A5	7.965931296	Tumor_G2
13152	UCP2	8.085318639	Tumor_G2
13153	SOD2	4.707165752	Tumor_G2
13154	NOX4	3.095211826	Tumor_G2
13155	DUOX1	6.457444077	Tumor_G2
13156	DUOXA1	6.378967467	Tumor_G2
13157	DUOX2	6.843785998	Tumor_G2
13158	DUOXA2	4.926844029	Tumor_G2

13159	NDUFA1	8.43517745	Tumor_G2
13160	NDUFA2	7.36184391	Tumor_G2
13161	NDUFA8	7.156352807	Tumor_G2
13162	NDUFB1	5.726541816	Tumor_G2
13163	NDUFB10	7.401147255	Tumor_G2
13164	NDUFB2	7.186169314	Tumor_G2
13165	NDUFB3	5.605216713	Tumor_G2
13166	NDUFB7	9.062286969	Tumor_G2
13167	CYC1	7.283155251	Tumor_G2
13168	ETFB	5.258002624	Tumor_G2
13169	UQCR10	8.006674806	Tumor_G2
13170	UQCRQ	7.419104484	Tumor_G2
13171	UQCC3	4.269065273	Tumor_G2
13172	COX15	4.481473832	Tumor_G2
13173	COX5A	6.466846478	Tumor_G2
13174	COX6B1	8.540420253	Tumor_G2
13175	COX6C	6.944755088	Tumor_G2
13176	COX7A2	6.404541451	Tumor_G2
13177	COX7B	6.264783869	Tumor_G2
13178	COX8A	9.535892339	Tumor_G2
13179	SCO1	3.58889032	Tumor_G2
13180	SURF1	5.901524508	Tumor_G2
13181	ATP6V1E1	6.636826496	Tumor_G2
13182	DLAT	4.315709425	Tumor_G2
13183	PDC	0.03783654	Tumor_G2
13184	SUCLG2	5.751293982	Tumor_G2
13185	SLC25A5	9.049856073	Tumor_G2
13186	UCP2	5.554315771	Tumor_G2
13187	SOD2	4.001444908	Tumor_G2
13188	NOX4	0.657960037	Tumor_G2
13189	DUOX1	4.87115093	Tumor_G2
13190	DUOXA1	5.672470383	Tumor_G2
13191	DUOX2	5.693966617	Tumor_G2
13192	DUOXA2	5.234208476	Tumor_G2
13193	NDUFA1	8.332894296	Tumor_G2
13194	NDUFA2	7.159430851	Tumor_G2
13195	NDUFA8	7.353185312	Tumor_G2
13196	NDUFB1	6.024050559	Tumor_G2
13197	NDUFB10	7.476188505	Tumor_G2
13198	NDUFB2	6.774618573	Tumor_G2

13199	NDUFB3	6.402569217	Tumor_G2
13200	NDUFB7	8.944273786	Tumor_G2
13201	CYC1	6.921484099	Tumor_G2
13202	ETFB	5.093326587	Tumor_G2
13203	UQCR10	7.697348382	Tumor_G2
13204	UQCRQ	7.544958433	Tumor_G2
13205	UQCC3	4.687013326	Tumor_G2
13206	COX15	4.811385169	Tumor_G2
13207	COX5A	7.090485027	Tumor_G2
13208	COX6B1	8.859581563	Tumor_G2
13209	COX6C	6.704802144	Tumor_G2
13210	COX7A2	7.246047244	Tumor_G2
13211	COX7B	6.296506245	Tumor_G2
13212	COX8A	9.009952429	Tumor_G2
13213	SCO1	3.457641022	Tumor_G2
13214	SURF1	6.529059068	Tumor_G2
13215	ATP6V1E1	7.068933936	Tumor_G2
13216	DLAT	5.197805246	Tumor_G2
13217	PDC	0.455830049	Tumor_G2
13218	SUCLG2	6.062961228	Tumor_G2
13219	SLC25A5	8.057388511	Tumor_G2
13220	UCP2	9.249923055	Tumor_G2
13221	SOD2	4.931151776	Tumor_G2
13222	NOX4	2.815498582	Tumor_G2
13223	DUOX1	6.189018788	Tumor_G2
13224	DUOXA1	6.639416806	Tumor_G2
13225	DUOX2	6.846521781	Tumor_G2
13226	DUOXA2	5.134809876	Tumor_G2
13227	NDUFA1	8.09939152	Tumor_G2
13228	NDUFA2	6.990489396	Tumor_G2
13229	NDUFA8	7.150841973	Tumor_G2
13230	NDUFB1	5.81156702	Tumor_G2
13231	NDUFB10	7.622804104	Tumor_G2
13232	NDUFB2	6.895869096	Tumor_G2
13233	NDUFB3	6.616161338	Tumor_G2
13234	NDUFB7	8.613391721	Tumor_G2
13235	CYC1	7.176307477	Tumor_G2
13236	ETFB	5.085500591	Tumor_G2
13237	UQCR10	7.756290767	Tumor_G2
13238	UQCRQ	7.297764025	Tumor_G2

13239	UQCC3	4.407557806	Tumor_G2
13240	COX15	4.487701517	Tumor_G2
13241	COX5A	7.363772269	Tumor_G2
13242	COX6B1	8.500683051	Tumor_G2
13243	COX6C	6.694694488	Tumor_G2
13244	COX7A2	6.956176928	Tumor_G2
13245	COX7B	6.113253428	Tumor_G2
13246	COX8A	9.511496054	Tumor_G2
13247	SCO1	3.395443269	Tumor_G2
13248	SURF1	6.282158108	Tumor_G2
13249	ATP6V1E1	6.945769656	Tumor_G2
13250	DLAT	4.924568667	Tumor_G2
13251	PDC	0.089481793	Tumor_G2
13252	SUCLG2	6.435315318	Tumor_G2
13253	SLC25A5	8.389406602	Tumor_G2
13254	UCP2	7.873334682	Tumor_G2
13255	SOD2	5.133973728	Tumor_G2
13256	NOX4	1.356208794	Tumor_G2
13257	DUOX1	6.251609456	Tumor_G2
13258	DUOXA1	6.066425479	Tumor_G2
13259	DUOX2	6.921444048	Tumor_G2
13260	DUOXA2	4.671833493	Tumor_G2
13261	NDUFA1	8.228230863	Tumor_G2
13262	NDUFA2	6.718893833	Tumor_G2
13263	NDUFA8	7.264200317	Tumor_G2
13264	NDUFB1	5.679890446	Tumor_G2
13265	NDUFB10	7.64251934	Tumor_G2
13266	NDUFB2	6.733026658	Tumor_G2
13267	NDUFB3	6.478487358	Tumor_G2
13268	NDUFB7	8.734062872	Tumor_G2
13269	CYC1	6.935729746	Tumor_G2
13270	ETFB	5.425332983	Tumor_G2
13271	UQCR10	7.411102435	Tumor_G2
13272	UQCRQ	7.15362436	Tumor_G2
13273	UQCC3	4.420754585	Tumor_G2
13274	COX15	5.161778539	Tumor_G2
13275	COX5A	7.261181566	Tumor_G2
13276	COX6B1	8.71282047	Tumor_G2
13277	COX6C	6.477081431	Tumor_G2
13278	COX7A2	6.866504676	Tumor_G2

13279	COX7B	6.096708828	Tumor_G2
13280	COX8A	9.612996992	Tumor_G2
13281	SCO1	3.661648069	Tumor_G2
13282	SURF1	6.61006601	Tumor_G2
13283	ATP6V1E1	6.496500579	Tumor_G2
13284	DLAT	5.139882253	Tumor_G2
13285	PDC	0.238296996	Tumor_G2
13286	SUCLG2	6.393284773	Tumor_G2
13287	SLC25A5	8.056395559	Tumor_G2
13288	UCP2	8.752597512	Tumor_G2
13289	SOD2	4.890344195	Tumor_G2
13290	NOX4	1.375042846	Tumor_G2
13291	DUOX1	6.922134239	Tumor_G2
13292	DUOXA1	6.933004164	Tumor_G2
13293	DUOX2	8.064544913	Tumor_G2
13294	DUOXA2	6.202314076	Tumor_G2
13295	NDUFA1	8.657077369	Tumor_G2
13296	NDUFA2	7.216611459	Tumor_G2
13297	NDUFA8	7.666646298	Tumor_G2
13298	NDUFB1	6.316301305	Tumor_G2
13299	NDUFB10	7.234527361	Tumor_G2
13300	NDUFB2	7.768752778	Tumor_G2
13301	NDUFB3	6.998358871	Tumor_G2
13302	NDUFB7	8.862412672	Tumor_G2
13303	CYC1	7.844658284	Tumor_G2
13304	ETFB	4.933945023	Tumor_G2
13305	UQCR10	8.249745307	Tumor_G2
13306	UQCRQ	7.6469845	Tumor_G2
13307	UQCC3	3.981920256	Tumor_G2
13308	COX15	4.633575186	Tumor_G2
13309	COX5A	7.739392468	Tumor_G2
13310	COX6B1	9.110702526	Tumor_G2
13311	COX6C	6.982333407	Tumor_G2
13312	COX7A2	7.44033675	Tumor_G2
13313	COX7B	7.279077382	Tumor_G2
13314	COX8A	9.976607447	Tumor_G2
13315	SCO1	3.191744493	Tumor_G2
13316	SURF1	5.888116769	Tumor_G2
13317	ATP6V1E1	6.997661756	Tumor_G2
13318	DLAT	5.625403701	Tumor_G2

13319	PDC	0	Tumor_G2
13320	SUCLG2	6.445609136	Tumor_G2
13321	SLC25A5	9.038401542	Tumor_G2
13322	UCP2	6.99267528	Tumor_G2
13323	SOD2	4.867184241	Tumor_G2
13324	NOX4	0.591197731	Tumor_G2
13325	DUOX1	5.979994292	Tumor_G2
13326	DUOXA1	5.420406898	Tumor_G2
13327	DUOX2	6.794768629	Tumor_G2
13328	DUOXA2	4.600324818	Tumor_G2
13329	NDUFA1	7.999826841	Tumor_G2
13330	NDUFA2	6.897713229	Tumor_G2
13331	NDUFA8	7.238887047	Tumor_G2
13332	NDUFB1	5.743676446	Tumor_G2
13333	NDUFB10	7.4353738	Tumor_G2
13334	NDUFB2	7.158168658	Tumor_G2
13335	NDUFB3	6.488352652	Tumor_G2
13336	NDUFB7	8.797513893	Tumor_G2
13337	CYC1	7.169932019	Tumor_G2
13338	ETFB	4.899127237	Tumor_G2
13339	UQCR10	7.804413695	Tumor_G2
13340	UQCRQ	7.422771169	Tumor_G2
13341	UQCC3	4.448642613	Tumor_G2
13342	COX15	4.598495258	Tumor_G2
13343	COX5A	7.270438905	Tumor_G2
13344	COX6B1	8.685469234	Tumor_G2
13345	COX6C	6.55681643	Tumor_G2
13346	COX7A2	6.957876599	Tumor_G2
13347	COX7B	6.238081564	Tumor_G2
13348	COX8A	9.638770558	Tumor_G2
13349	SCO1	3.549173906	Tumor_G2
13350	SURF1	6.422475246	Tumor_G2
13351	ATP6V1E1	7.617102222	Tumor_G2
13352	DLAT	5.289202688	Tumor_G2
13353	PDC	0.3471465	Tumor_G2
13354	SUCLG2	6.531049431	Tumor_G2
13355	SLC25A5	8.391804086	Tumor_G2
13356	UCP2	8.501194885	Tumor_G2
13357	SOD2	4.755140149	Tumor_G2
13358	NOX4	1.423895376	Tumor_G2

13359	DUOX1	5.943242592	Tumor_G2
13360	DUOXA1	6.540995601	Tumor_G2
13361	DUOX2	7.028276747	Tumor_G2
13362	DUOXA2	4.381882593	Tumor_G2
13363	NDUFA1	8.447273352	Tumor_G2
13364	NDUFA2	7.302429763	Tumor_G2
13365	NDUFA8	7.553534881	Tumor_G2
13366	NDUFB1	6.393232836	Tumor_G2
13367	NDUFB10	8.000751443	Tumor_G2
13368	NDUFB2	7.162329284	Tumor_G2
13369	NDUFB3	6.591235389	Tumor_G2
13370	NDUFB7	9.201923206	Tumor_G2
13371	CYC1	7.501543716	Tumor_G2
13372	ETFB	5.628119805	Tumor_G2
13373	UQCR10	7.421882957	Tumor_G2
13374	UQCRQ	7.667032913	Tumor_G2
13375	UQCC3	5.276250289	Tumor_G2
13376	COX15	5.421974902	Tumor_G2
13377	COX5A	7.454805143	Tumor_G2
13378	COX6B1	8.892804686	Tumor_G2
13379	COX6C	6.86962041	Tumor_G2
13380	COX7A2	7.002174228	Tumor_G2
13381	COX7B	6.326152647	Tumor_G2
13382	COX8A	10.29412919	Tumor_G2
13383	SCO1	3.632876249	Tumor_G2
13384	SURF1	7.183550105	Tumor_G2
13385	ATP6V1E1	7.13959367	Tumor_G2
13386	DLAT	5.197376305	Tumor_G2
13387	PDC	0.224067608	Tumor_G2
13388	SUCLG2	6.424274129	Tumor_G2
13389	SLC25A5	8.37605138	Tumor_G2
13390	UCP2	9.231370535	Tumor_G2
13391	SOD2	4.387021318	Tumor_G2
13392	NOX4	1.882364659	Tumor_G2
13393	DUOX1	7.828799558	Tumor_G2
13394	DUOXA1	7.831978388	Tumor_G2
13395	DUOX2	9.3468355	Tumor_G2
13396	DUOXA2	6.459392198	Tumor_G2
13397	NDUFA1	8.102036245	Tumor_G2
13398	NDUFA2	6.749385578	Tumor_G2

13399	NDUFA8	7.221163294	Tumor_G2
13400	NDUFB1	5.428326884	Tumor_G2
13401	NDUFB10	7.137801302	Tumor_G2
13402	NDUFB2	6.668783585	Tumor_G2
13403	NDUFB3	6.435948649	Tumor_G2
13404	NDUFB7	7.669369351	Tumor_G2
13405	CYC1	6.991733788	Tumor_G2
13406	ETFB	5.570711769	Tumor_G2
13407	UQCR10	6.792569732	Tumor_G2
13408	UQCRQ	7.045841046	Tumor_G2
13409	UQCC3	4.115591535	Tumor_G2
13410	COX15	5.55319949	Tumor_G2
13411	COX5A	6.278596226	Tumor_G2
13412	COX6B1	8.805385692	Tumor_G2
13413	COX6C	6.194732446	Tumor_G2
13414	COX7A2	6.847915213	Tumor_G2
13415	COX7B	5.81383878	Tumor_G2
13416	COX8A	9.373597744	Tumor_G2
13417	SCO1	3.791993567	Tumor_G2
13418	SURF1	6.561741826	Tumor_G2
13419	ATP6V1E1	6.677037227	Tumor_G2
13420	DLAT	5.521316321	Tumor_G2
13421	PDC	0.373499368	Tumor_G2
13422	SUCLG2	6.411224389	Tumor_G2
13423	SLC25A5	7.435730279	Tumor_G2
13424	UCP2	5.775164747	Tumor_G2
13425	SOD2	4.519008162	Tumor_G2
13426	NOX4	0.889895189	Tumor_G2
13427	DUOX1	7.857998635	Tumor_G2
13428	DUOXA1	7.283736514	Tumor_G2
13429	DUOX2	8.557606206	Tumor_G2
13430	DUOXA2	6.119782626	Tumor_G2
13431	NDUFA1	7.828062425	Tumor_G2
13432	NDUFA2	6.265848183	Tumor_G2
13433	NDUFA8	6.755451343	Tumor_G2
13434	NDUFB1	5.70053076	Tumor_G2
13435	NDUFB10	7.378178871	Tumor_G2
13436	NDUFB2	5.995990726	Tumor_G2
13437	NDUFB3	6.104321158	Tumor_G2
13438	NDUFB7	8.260691147	Tumor_G2

13439	CYC1	6.552248352	Tumor_G2
13440	ETFB	4.794093301	Tumor_G2
13441	UQCR10	7.274280722	Tumor_G2
13442	UQCRQ	6.90990583	Tumor_G2
13443	UQCC3	4.217241756	Tumor_G2
13444	COX15	4.919047622	Tumor_G2
13445	COX5A	7.001172664	Tumor_G2
13446	COX6B1	8.254244904	Tumor_G2
13447	COX6C	6.159650397	Tumor_G2
13448	COX7A2	6.405783816	Tumor_G2
13449	COX7B	5.750787544	Tumor_G2
13450	COX8A	9.145627798	Tumor_G2
13451	SCO1	3.421916967	Tumor_G2
13452	SURF1	6.368527734	Tumor_G2
13453	ATP6V1E1	6.482023762	Tumor_G2
13454	DLAT	4.8113654	Tumor_G2
13455	PDC	0.223069469	Tumor_G2
13456	SUCLG2	5.905151447	Tumor_G2
13457	SLC25A5	7.534063902	Tumor_G2
13458	UCP2	8.927927106	Tumor_G2
13459	SOD2	5.307757542	Tumor_G2
13460	NOX4	1.675616087	Tumor_G2
13461	DUOX1	7.712677783	Tumor_G2
13462	DUOXA1	7.89738328	Tumor_G2
13463	DUOX2	8.325882052	Tumor_G2
13464	DUOXA2	5.791735775	Tumor_G2
13465	NDUFA1	8.39384811	Tumor_G2
13466	NDUFA2	7.164338589	Tumor_G2
13467	NDUFA8	7.022363702	Tumor_G2
13468	NDUFB1	5.851382773	Tumor_G2
13469	NDUFB10	7.913908807	Tumor_G2
13470	NDUFB2	6.503212239	Tumor_G2
13471	NDUFB3	6.235026672	Tumor_G2
13472	NDUFB7	9.187585223	Tumor_G2
13473	CYC1	7.138774415	Tumor_G2
13474	ETFB	4.322907308	Tumor_G2
13475	UQCR10	7.437356303	Tumor_G2
13476	UQCRQ	7.513445958	Tumor_G2
13477	UQCC3	5.102659833	Tumor_G2
13478	COX15	4.941013137	Tumor_G2

13479	COX5A	7.418582593	Tumor_G2
13480	COX6B1	9.137720625	Tumor_G2
13481	COX6C	6.533253268	Tumor_G2
13482	COX7A2	7.106953998	Tumor_G2
13483	COX7B	6.054674776	Tumor_G2
13484	COX8A	9.688084915	Tumor_G2
13485	SCO1	3.357991322	Tumor_G2
13486	SURF1	6.732929932	Tumor_G2
13487	ATP6V1E1	6.837096569	Tumor_G2
13488	DLAT	4.834421506	Tumor_G2
13489	PDC	0.151832573	Tumor_G2
13490	SUCLG2	5.717922145	Tumor_G2
13491	SLC25A5	7.805878096	Tumor_G2
13492	UCP2	8.468464339	Tumor_G2
13493	SOD2	4.431493359	Tumor_G2
13494	NOX4	1.516405781	Tumor_G2
13495	DUOX1	5.759604541	Tumor_G2
13496	DUOXA1	6.574438532	Tumor_G2
13497	DUOX2	7.026586514	Tumor_G2
13498	DUOXA2	7.000176658	Tumor_G2
13499	NDUFA1	8.270736638	Tumor_G2
13500	NDUFA2	7.028857637	Tumor_G2
13501	NDUFA8	7.102869396	Tumor_G2
13502	NDUFB1	6.046856863	Tumor_G2
13503	NDUFB10	7.519918083	Tumor_G2
13504	NDUFB2	6.956528412	Tumor_G2
13505	NDUFB3	6.665467777	Tumor_G2
13506	NDUFB7	8.751614474	Tumor_G2
13507	CYC1	6.983857453	Tumor_G2
13508	ETFB	4.883347306	Tumor_G2
13509	UQCR10	7.809278654	Tumor_G2
13510	UQCRQ	7.42316854	Tumor_G2
13511	UQCC3	4.760380495	Tumor_G2
13512	COX15	4.962978844	Tumor_G2
13513	COX5A	7.178779683	Tumor_G2
13514	COX6B1	8.786881701	Tumor_G2
13515	COX6C	6.467363811	Tumor_G2
13516	COX7A2	7.135016748	Tumor_G2
13517	COX7B	6.267755364	Tumor_G2
13518	COX8A	9.83031883	Tumor_G2

13519	SCO1	3.666468977	Tumor_G2
13520	SURF1	6.745380531	Tumor_G2
13521	ATP6V1E1	6.803349738	Tumor_G2
13522	DLAT	5.258321889	Tumor_G2
13523	PDC	0.183995711	Tumor_G2
13524	SUCLG2	6.043451587	Tumor_G2
13525	SLC25A5	7.985063391	Tumor_G2
13526	UCP2	7.57357888	Tumor_G2
13527	SOD2	4.710248821	Tumor_G2
13528	NOX4	0.343584638	Tumor_G2
13529	DUOX1	7.332219618	Tumor_G2
13530	DUOXA1	7.392292972	Tumor_G2
13531	DUOX2	8.410812909	Tumor_G2
13532	DUOXA2	7.141408635	Tumor_G2
13533	NDUFA1	7.726912559	Tumor_G2
13534	NDUFA2	6.632860827	Tumor_G2
13535	NDUFA8	6.856024738	Tumor_G2
13536	NDUFB1	5.597543633	Tumor_G2
13537	NDUFB10	7.129035815	Tumor_G2
13538	NDUFB2	6.467356485	Tumor_G2
13539	NDUFB3	6.163989545	Tumor_G2
13540	NDUFB7	8.40264847	Tumor_G2
13541	CYC1	6.736280018	Tumor_G2
13542	ETFB	4.769761684	Tumor_G2
13543	UQCR10	7.393824252	Tumor_G2
13544	UQCRQ	7.277395357	Tumor_G2
13545	UQCC3	4.057664017	Tumor_G2
13546	COX15	4.518190733	Tumor_G2
13547	COX5A	6.83680995	Tumor_G2
13548	COX6B1	8.456085133	Tumor_G2
13549	COX6C	6.3992582	Tumor_G2
13550	COX7A2	6.736694939	Tumor_G2
13551	COX7B	5.928818135	Tumor_G2
13552	COX8A	9.148754145	Tumor_G2
13553	SCO1	3.297467168	Tumor_G2
13554	SURF1	5.934990917	Tumor_G2
13555	ATP6V1E1	6.934544437	Tumor_G2
13556	DLAT	4.854769466	Tumor_G2
13557	PDC	0.198783684	Tumor_G2
13558	SUCLG2	6.009891756	Tumor_G2

13559	SLC25A5	8.036568883	Tumor_G2
13560	UCP2	8.694896166	Tumor_G2
13561	SOD2	5.021452454	Tumor_G2
13562	NOX4	2.744799004	Tumor_G2
13563	DUOX1	6.716647952	Tumor_G2
13564	DUOXA1	6.893569671	Tumor_G2
13565	DUOX2	7.059853394	Tumor_G2
13566	DUOXA2	4.630963452	Tumor_G2
13567	NDUFA1	8.017081551	Tumor_G2
13568	NDUFA2	6.723869122	Tumor_G2
13569	NDUFA8	7.220054461	Tumor_G2
13570	NDUFB1	6.16092505	Tumor_G2
13571	NDUFB10	7.556085435	Tumor_G2
13572	NDUFB2	6.893040992	Tumor_G2
13573	NDUFB3	6.545177798	Tumor_G2
13574	NDUFB7	8.659970668	Tumor_G2
13575	CYC1	7.006674605	Tumor_G2
13576	ETFB	5.424131159	Tumor_G2
13577	UQCR10	7.766115698	Tumor_G2
13578	UQCRQ	7.534741475	Tumor_G2
13579	UQCC3	4.373569706	Tumor_G2
13580	COX15	4.90378913	Tumor_G2
13581	COX5A	7.013546714	Tumor_G2
13582	COX6B1	8.615552662	Tumor_G2
13583	COX6C	6.781184913	Tumor_G2
13584	COX7A2	7.025530528	Tumor_G2
13585	COX7B	6.456880555	Tumor_G2
13586	COX8A	9.727191017	Tumor_G2
13587	SCO1	3.790320867	Tumor_G2
13588	SURF1	6.552397435	Tumor_G2
13589	ATP6V1E1	6.91452	Tumor_G2
13590	DLAT	5.192047813	Tumor_G2
13591	PDC	0.177730101	Tumor_G2
13592	SUCLG2	6.033624537	Tumor_G2
13593	SLC25A5	8.064233132	Tumor_G2
13594	UCP2	7.715599748	Tumor_G2
13595	SOD2	4.407510054	Tumor_G2
13596	NOX4	2.029022491	Tumor_G2
13597	DUOX1	8.389936889	Tumor_G2
13598	DUOXA1	8.046142036	Tumor_G2

13599	DUOX2	8.701986447	Tumor_G2
13600	DUOXA2	6.854458093	Tumor_G2
13601	NDUFA1	7.884470685	Tumor_G2
13602	NDUFA2	6.104673095	Tumor_G2
13603	NDUFA8	7.092753323	Tumor_G2
13604	NDUFB1	5.468745719	Tumor_G2
13605	NDUFB10	7.397622651	Tumor_G2
13606	NDUFB2	5.771250205	Tumor_G2
13607	NDUFB3	6.024663494	Tumor_G2
13608	NDUFB7	8.510377177	Tumor_G2
13609	CYC1	6.622743939	Tumor_G2
13610	ETFB	5.002430999	Tumor_G2
13611	UQCR10	6.983351792	Tumor_G2
13612	UQCRQ	6.942238296	Tumor_G2
13613	UQCC3	3.945205695	Tumor_G2
13614	COX15	4.893540369	Tumor_G2
13615	COX5A	6.644851261	Tumor_G2
13616	COX6B1	8.943098945	Tumor_G2
13617	COX6C	5.962868046	Tumor_G2
13618	COX7A2	6.706553871	Tumor_G2
13619	COX7B	6.163553315	Tumor_G2
13620	COX8A	9.115208915	Tumor_G2
13621	SCO1	3.381426795	Tumor_G2
13622	SURF1	6.08236262	Tumor_G2
13623	ATP6V1E1	6.297409585	Tumor_G2
13624	DLAT	5.004848523	Tumor_G2
13625	PDC	0.10245135	Tumor_G2
13626	SUCLG2	6.000043963	Tumor_G2
13627	SLC25A5	8.009054193	Tumor_G2
13628	UCP2	8.848954275	Tumor_G2
13629	SOD2	5.399296659	Tumor_G2
13630	NOX4	2.757667304	Tumor_G2
13631	DUOX1	7.090320524	Tumor_G2
13632	DUOXA1	7.226233656	Tumor_G2
13633	DUOX2	8.076012817	Tumor_G2
13634	DUOXA2	4.658237278	Tumor_G2
13635	NDUFA1	8.396266458	Tumor_G2
13636	NDUFA2	6.921812661	Tumor_G2
13637	NDUFA8	7.268057541	Tumor_G2
13638	NDUFB1	6.141643615	Tumor_G2

13639	NDUFB10	7.72655784	Tumor_G2
13640	NDUFB2	6.875744657	Tumor_G2
13641	NDUFB3	6.706406016	Tumor_G2
13642	NDUFB7	8.549314506	Tumor_G2
13643	CYC1	7.483421845	Tumor_G2
13644	ETFB	5.481779207	Tumor_G2
13645	UQCR10	7.868968498	Tumor_G2
13646	UQCRQ	7.209020856	Tumor_G2
13647	UQCC3	4.224561828	Tumor_G2
13648	COX15	5.542899103	Tumor_G2
13649	COX5A	7.376794671	Tumor_G2
13650	COX6B1	8.716166747	Tumor_G2
13651	COX6C	6.709214858	Tumor_G2
13652	COX7A2	7.014016729	Tumor_G2
13653	COX7B	6.258559587	Tumor_G2
13654	COX8A	10.04217495	Tumor_G2
13655	SCO1	4.256747347	Tumor_G2
13656	SURF1	6.579649756	Tumor_G2
13657	ATP6V1E1	6.73300723	Tumor_G2
13658	DLAT	5.243272611	Tumor_G2
13659	PDC	0.22021293	Tumor_G2
13660	SUCLG2	6.534958073	Tumor_G2
13661	SLC25A5	8.989453141	Tumor_G2
13662	UCP2	5.129005658	Tumor_G2
13663	SOD2	4.497143923	Tumor_G2
13664	NOX4	1.746063778	Tumor_G2
13665	DUOX1	7.664571329	Tumor_G2
13666	DUOXA1	7.193242367	Tumor_G2
13667	DUOX2	10.75549818	Tumor_G2
13668	DUOXA2	8.086142516	Tumor_G2
13669	NDUFA1	8.008720085	Tumor_G2
13670	NDUFA2	6.937481582	Tumor_G2
13671	NDUFA8	6.918839204	Tumor_G2
13672	NDUFB1	5.689960457	Tumor_G2
13673	NDUFB10	7.611912573	Tumor_G2
13674	NDUFB2	6.834731142	Tumor_G2
13675	NDUFB3	6.282567932	Tumor_G2
13676	NDUFB7	8.7577553	Tumor_G2
13677	CYC1	7.232876297	Tumor_G2
13678	ETFB	5.039662893	Tumor_G2

13679	UQCR10	7.927355372	Tumor_G2
13680	UQCRQ	7.153441547	Tumor_G2
13681	UQCC3	4.495428609	Tumor_G2
13682	COX15	4.985561931	Tumor_G2
13683	COX5A	7.003630927	Tumor_G2
13684	COX6B1	8.642127948	Tumor_G2
13685	COX6C	6.492020716	Tumor_G2
13686	COX7A2	6.836332109	Tumor_G2
13687	COX7B	5.910211265	Tumor_G2
13688	COX8A	9.578455768	Tumor_G2
13689	SCO1	3.19354563	Tumor_G2
13690	SURF1	6.258967178	Tumor_G2
13691	ATP6V1E1	7.233449681	Tumor_G2
13692	DLAT	5.154747951	Tumor_G2
13693	PDC	0.267402509	Tumor_G2
13694	SUCLG2	6.474161966	Tumor_G2
13695	SLC25A5	8.122219127	Tumor_G2
13696	UCP2	8.708953538	Tumor_G2
13697	SOD2	4.491896992	Tumor_G2
13698	NOX4	1.394755368	Tumor_G2
13699	DUOX1	6.55079752	Tumor_G2
13700	DUOXA1	6.468726426	Tumor_G2
13701	DUOX2	6.859874571	Tumor_G2
13702	DUOXA2	4.814192429	Tumor_G2
13703	NDUFA1	7.992152681	Tumor_G2
13704	NDUFA2	6.890180555	Tumor_G2
13705	NDUFA8	6.981842054	Tumor_G2
13706	NDUFB1	5.473137918	Tumor_G2
13707	NDUFB10	7.665595392	Tumor_G2
13708	NDUFB2	6.494063076	Tumor_G2
13709	NDUFB3	6.233242999	Tumor_G2
13710	NDUFB7	8.667922161	Tumor_G2
13711	CYC1	7.100028066	Tumor_G2
13712	ETFB	5.172785073	Tumor_G2
13713	UQCR10	7.485435437	Tumor_G2
13714	UQCRQ	7.33086508	Tumor_G2
13715	UQCC3	4.430597167	Tumor_G2
13716	COX15	4.755143268	Tumor_G2
13717	COX5A	7.063342049	Tumor_G2
13718	COX6B1	8.846416663	Tumor_G2

13719	COX6C	6.417097048	Tumor_G2
13720	COX7A2	7.049684648	Tumor_G2
13721	COX7B	6.019307009	Tumor_G2
13722	COX8A	9.536942977	Tumor_G2
13723	SCO1	3.086622245	Tumor_G2
13724	SURF1	6.357727092	Tumor_G2
13725	ATP6V1E1	7.163500611	Tumor_G2
13726	DLAT	5.034592291	Tumor_G2
13727	PDC	0.043468941	Tumor_G2
13728	SUCLG2	6.209381253	Tumor_G2
13729	SLC25A5	8.270480478	Tumor_G2
13730	UCP2	8.290209736	Tumor_G2
13731	SOD2	4.986804207	Tumor_G2
13732	NOX4	2.182968559	Tumor_G2
13733	DUOX1	6.704672215	Tumor_G2
13734	DUOXA1	6.592130371	Tumor_G2
13735	DUOX2	6.698708479	Tumor_G2
13736	DUOXA2	4.529758365	Tumor_G2
13737	NDUFA1	8.120752713	Tumor_G2
13738	NDUFA2	6.955881689	Tumor_G2
13739	NDUFA8	6.90882775	Tumor_G2
13740	NDUFB1	6.050096051	Tumor_G2
13741	NDUFB10	7.598172438	Tumor_G2
13742	NDUFB2	6.85840571	Tumor_G2
13743	NDUFB3	6.178379486	Tumor_G2
13744	NDUFB7	8.885285969	Tumor_G2
13745	CYC1	6.99790549	Tumor_G2
13746	ETFB	4.983396721	Tumor_G2
13747	UQCR10	7.854800879	Tumor_G2
13748	UQCRQ	7.412643622	Tumor_G2
13749	UQCC3	4.48932734	Tumor_G2
13750	COX15	4.343497398	Tumor_G2
13751	COX5A	7.02793435	Tumor_G2
13752	COX6B1	8.886159001	Tumor_G2
13753	COX6C	6.665469735	Tumor_G2
13754	COX7A2	7.074939498	Tumor_G2
13755	COX7B	6.097257995	Tumor_G2
13756	COX8A	9.74200465	Tumor_G2
13757	SCO1	2.511094207	Tumor_G2
13758	SURF1	6.19695367	Tumor_G2

13759	ATP6V1E1	7.156010268	Tumor_G2
13760	DLAT	4.468305619	Tumor_G2
13761	PDC	0.106226084	Tumor_G2
13762	SUCLG2	6.157038971	Tumor_G2
13763	SLC25A5	7.881656749	Tumor_G2
13764	UCP2	8.484131683	Tumor_G2
13765	SOD2	4.74344336	Tumor_G2
13766	NOX4	1.380070613	Tumor_G2
13767	DUOX1	5.851806442	Tumor_G2
13768	DUOXA1	5.988006468	Tumor_G2
13769	DUOX2	6.395561831	Tumor_G2
13770	DUOXA2	4.065511419	Tumor_G2
13771	NDUFA1	7.988018411	Tumor_G2
13772	NDUFA2	6.765523165	Tumor_G2
13773	NDUFA8	6.99809665	Tumor_G2
13774	NDUFB1	5.781388987	Tumor_G2
13775	NDUFB10	7.586735396	Tumor_G2
13776	NDUFB2	6.62791168	Tumor_G2
13777	NDUFB3	6.40190017	Tumor_G2
13778	NDUFB7	8.529726943	Tumor_G2
13779	CYC1	6.820959596	Tumor_G2
13780	ETFB	4.83483009	Tumor_G2
13781	UQCR10	7.729015673	Tumor_G2
13782	UQCRQ	7.404420075	Tumor_G2
13783	UQCC3	4.229476705	Tumor_G2
13784	COX15	4.553261879	Tumor_G2
13785	COX5A	7.125223126	Tumor_G2
13786	COX6B1	8.679560257	Tumor_G2
13787	COX6C	6.391305147	Tumor_G2
13788	COX7A2	6.873959437	Tumor_G2
13789	COX7B	5.952109526	Tumor_G2
13790	COX8A	9.199767461	Tumor_G2
13791	SCO1	3.392571314	Tumor_G2
13792	SURF1	6.530317896	Tumor_G2
13793	ATP6V1E1	7.137186976	Tumor_G2
13794	DLAT	5.184518137	Tumor_G2
13795	PDC	0.097163536	Tumor_G2
13796	SUCLG2	6.248043076	Tumor_G2
13797	SLC25A5	8.057104652	Tumor_G2
13798	UCP2	8.109963978	Tumor_G2

13799	SOD2	5.606777547	Tumor_G2
13800	NOX4	3.40320714	Tumor_G2
13801	DUOX1	5.921988072	Tumor_G2
13802	DUOXA1	6.286613815	Tumor_G2
13803	DUOX2	6.246064405	Tumor_G2
13804	DUOXA2	4.354013478	Tumor_G2
13805	NDUFA1	8.168530261	Tumor_G2
13806	NDUFA2	7.052510234	Tumor_G2
13807	NDUFA8	7.18817972	Tumor_G2
13808	NDUFB1	6.192456422	Tumor_G2
13809	NDUFB10	7.643234076	Tumor_G2
13810	NDUFB2	7.37451723	Tumor_G2
13811	NDUFB3	6.654524134	Tumor_G2
13812	NDUFB7	8.924098996	Tumor_G2
13813	CYC1	6.911763362	Tumor_G2
13814	ETFB	5.218653816	Tumor_G2
13815	UQCR10	7.2838684	Tumor_G2
13816	UQCRQ	7.785085935	Tumor_G2
13817	UQCC3	4.63648215	Tumor_G2
13818	COX15	4.841126867	Tumor_G2
13819	COX5A	7.39866344	Tumor_G2
13820	COX6B1	8.898201857	Tumor_G2
13821	COX6C	6.695024875	Tumor_G2
13822	COX7A2	7.031413929	Tumor_G2
13823	COX7B	6.26189432	Tumor_G2
13824	COX8A	9.874100977	Tumor_G2
13825	SCO1	3.61191367	Tumor_G2
13826	SURF1	6.976670319	Tumor_G2
13827	ATP6V1E1	5.954950148	Tumor_G2
13828	DLAT	5.110618924	Tumor_G2
13829	PDC	0.154072202	Tumor_G2
13830	SUCLG2	6.30638424	Tumor_G2
13831	SLC25A5	8.051658319	Tumor_G2
13832	UCP2	8.271733146	Tumor_G2
13833	SOD2	4.831471041	Tumor_G2
13834	NOX4	1.224551807	Tumor_G2
13835	DUOX1	7.051397513	Tumor_G2
13836	DUOXA1	6.638971248	Tumor_G2
13837	DUOX2	9.860735892	Tumor_G2
13838	DUOXA2	8.320068089	Tumor_G2

13839	NDUFA1	8.312889014	Tumor_G2
13840	NDUFA2	6.87741714	Tumor_G2
13841	NDUFA8	7.286117902	Tumor_G2
13842	NDUFB1	5.922582697	Tumor_G2
13843	NDUFB10	7.581747491	Tumor_G2
13844	NDUFB2	6.846932762	Tumor_G2
13845	NDUFB3	6.453818339	Tumor_G2
13846	NDUFB7	8.722098111	Tumor_G2
13847	CYC1	6.87259919	Tumor_G2
13848	ETFB	4.817734076	Tumor_G2
13849	UQCR10	7.775866423	Tumor_G2
13850	UQCRQ	7.350523758	Tumor_G2
13851	UQCC3	4.730018431	Tumor_G2
13852	COX15	4.859330293	Tumor_G2
13853	COX5A	7.336485721	Tumor_G2
13854	COX6B1	8.956631761	Tumor_G2
13855	COX6C	6.428602292	Tumor_G2
13856	COX7A2	7.071437909	Tumor_G2
13857	COX7B	6.233747008	Tumor_G2
13858	COX8A	9.504878164	Tumor_G2
13859	SCO1	3.522317515	Tumor_G2
13860	SURF1	6.759418562	Tumor_G2
13861	ATP6V1E1	7.054988738	Tumor_G2
13862	DLAT	4.982675077	Tumor_G2
13863	PDC	0	Tumor_G2
13864	SUCLG2	5.996252075	Tumor_G2
13865	SLC25A5	7.922778193	Tumor_G2
13866	UCP2	8.451692829	Tumor_G2
13867	SOD2	5.34669344	Tumor_G2
13868	NOX4	2.810011877	Tumor_G2
13869	DUOX1	5.703440298	Tumor_G2
13870	DUOXA1	6.309908041	Tumor_G2
13871	DUOX2	5.945280283	Tumor_G2
13872	DUOXA2	4.347947368	Tumor_G2
13873	NDUFA1	8.432925673	Tumor_G2
13874	NDUFA2	7.035921161	Tumor_G2
13875	NDUFA8	7.449540428	Tumor_G2
13876	NDUFB1	6.069461565	Tumor_G2
13877	NDUFB10	7.532415112	Tumor_G2
13878	NDUFB2	7.334958318	Tumor_G2

13879	NDUFB3	6.807071834	Tumor_G2
13880	NDUFB7	8.915042236	Tumor_G2
13881	CYC1	7.401632595	Tumor_G2
13882	ETFB	5.373197311	Tumor_G2
13883	UQCR10	7.779455842	Tumor_G2
13884	UQCRQ	7.465915327	Tumor_G2
13885	UQCC3	4.390705955	Tumor_G2
13886	COX15	5.16528205	Tumor_G2
13887	COX5A	7.38300716	Tumor_G2
13888	COX6B1	8.93179593	Tumor_G2
13889	COX6C	6.92393614	Tumor_G2
13890	COX7A2	7.358454374	Tumor_G2
13891	COX7B	6.663561874	Tumor_G2
13892	COX8A	9.92430054	Tumor_G2
13893	SCO1	3.688958962	Tumor_G2
13894	SURF1	6.872948919	Tumor_G2
13895	ATP6V1E1	6.827977975	Tumor_G2
13896	DLAT	5.372419285	Tumor_G2
13897	PDC	0.164962563	Tumor_G2
13898	SUCLG2	6.639829193	Tumor_G2
13899	SLC25A5	8.540179676	Tumor_G2
13900	UCP2	7.40229053	Tumor_G2
13901	SOD2	4.732108194	Tumor_G2
13902	NOX4	1.371620866	Tumor_G2
13903	DUOX1	6.439584017	Tumor_G2
13904	DUOXA1	6.35715967	Tumor_G2
13905	DUOX2	6.786158573	Tumor_G2
13906	DUOXA2	5.436903979	Tumor_G2
13907	NDUFA1	8.586133116	Tumor_G2
13908	NDUFA2	7.00317878	Tumor_G2
13909	NDUFA8	7.692899358	Tumor_G2
13910	NDUFB1	6.288406134	Tumor_G2
13911	NDUFB10	7.700637393	Tumor_G2
13912	NDUFB2	7.134663706	Tumor_G2
13913	NDUFB3	6.868811218	Tumor_G2
13914	NDUFB7	8.938913628	Tumor_G2
13915	CYC1	7.7140069	Tumor_G2
13916	ETFB	4.692187222	Tumor_G2
13917	UQCR10	7.917690467	Tumor_G2
13918	UQCRQ	7.745025924	Tumor_G2

13919	UQCC3	4.866440436	Tumor_G2
13920	COX15	4.728541162	Tumor_G2
13921	COX5A	7.998851765	Tumor_G2
13922	COX6B1	9.193247039	Tumor_G2
13923	COX6C	6.991921068	Tumor_G2
13924	COX7A2	7.448592824	Tumor_G2
13925	COX7B	7.059393027	Tumor_G2
13926	COX8A	9.894495393	Tumor_G2
13927	SCO1	3.739253277	Tumor_G2
13928	SURF1	6.539445211	Tumor_G2
13929	ATP6V1E1	7.316832275	Tumor_G2
13930	DLAT	5.238900186	Tumor_G2
13931	PDC	0	Tumor_G2
13932	SUCLG2	6.468681391	Tumor_G2
13933	SLC25A5	9.044123326	Tumor_G2
13934	UCP2	5.583617152	Tumor_G2
13935	SOD2	4.686933441	Tumor_G2
13936	NOX4	1.164885669	Tumor_G2
13937	DUOX1	5.369407971	Tumor_G2
13938	DUOXA1	6.037553736	Tumor_G2
13939	DUOX2	5.682647006	Tumor_G2
13940	DUOXA2	4.376118618	Tumor_G2
13941	NDUFA1	8.039819307	Tumor_G2
13942	NDUFA2	6.994822989	Tumor_G2
13943	NDUFA8	7.351629971	Tumor_G2
13944	NDUFB1	5.97129525	Tumor_G2
13945	NDUFB10	7.675879854	Tumor_G2
13946	NDUFB2	6.729840391	Tumor_G2
13947	NDUFB3	6.60934193	Tumor_G2
13948	NDUFB7	8.962468246	Tumor_G2
13949	CYC1	7.137885501	Tumor_G2
13950	ETFB	5.700016231	Tumor_G2
13951	UQCR10	7.862406754	Tumor_G2
13952	UQCRQ	7.522831653	Tumor_G2
13953	UQCC3	5.036103591	Tumor_G2
13954	COX15	5.003895664	Tumor_G2
13955	COX5A	7.098662417	Tumor_G2
13956	COX6B1	8.871373423	Tumor_G2
13957	COX6C	6.845528936	Tumor_G2
13958	COX7A2	7.292473808	Tumor_G2

13959	COX7B	6.234856817	Tumor_G2
13960	COX8A	9.776638854	Tumor_G2
13961	SCO1	3.406420982	Tumor_G2
13962	SURF1	6.771058966	Tumor_G2
13963	ATP6V1E1	6.909113742	Tumor_G2
13964	DLAT	4.842848945	Tumor_G2
13965	PDC	0.16539203	Tumor_G2
13966	SUCLG2	6.105007158	Tumor_G2
13967	SLC25A5	7.856845875	Tumor_G2
13968	UCP2	9.098971011	Tumor_G2
13969	SOD2	3.951143995	Tumor_G2
13970	NOX4	2.438347847	Tumor_G2
13971	DUOX1	6.604069644	Tumor_G2
13972	DUOXA1	7.057735951	Tumor_G2
13973	DUOX2	7.492217924	Tumor_G2
13974	DUOXA2	5.584929991	Tumor_G2
13975	NDUFA1	8.261751051	Tumor_G2
13976	NDUFA2	6.971778054	Tumor_G2
13977	NDUFA8	7.311511068	Tumor_G2
13978	NDUFB1	5.909650462	Tumor_G2
13979	NDUFB10	7.927639602	Tumor_G2
13980	NDUFB2	6.987948409	Tumor_G2
13981	NDUFB3	6.550450252	Tumor_G2
13982	NDUFB7	8.891549209	Tumor_G2
13983	CYC1	7.220049996	Tumor_G2
13984	ETFB	5.453933485	Tumor_G2
13985	UQCR10	7.74711328	Tumor_G2
13986	UQCRQ	7.569052339	Tumor_G2
13987	UQCC3	4.672491984	Tumor_G2
13988	COX15	4.935548069	Tumor_G2
13989	COX5A	7.449414329	Tumor_G2
13990	COX6B1	9.021713241	Tumor_G2
13991	COX6C	6.81036125	Tumor_G2
13992	COX7A2	7.21411678	Tumor_G2
13993	COX7B	6.318185118	Tumor_G2
13994	COX8A	9.824592558	Tumor_G2
13995	SCO1	3.091815964	Tumor_G2
13996	SURF1	6.870661724	Tumor_G2
13997	ATP6V1E1	7.072803368	Tumor_G2
13998	DLAT	4.717808921	Tumor_G2

13999	PDC	0.064676574	Tumor_G2
14000	SUCLG2	5.984761979	Tumor_G2
14001	SLC25A5	7.770662745	Tumor_G2
14002	UCP2	8.974030151	Tumor_G2
14003	SOD2	4.648030038	Tumor_G2
14004	NOX4	2.622094229	Tumor_G2
14005	DUOX1	6.758507473	Tumor_G2
14006	DUOXA1	6.906604226	Tumor_G2
14007	DUOX2	7.496695164	Tumor_G2
14008	DUOXA2	6.035422598	Tumor_G2
14009	NDUFA1	8.052153231	Tumor_G2
14010	NDUFA2	6.688218262	Tumor_G2
14011	NDUFA8	7.512043427	Tumor_G2
14012	NDUFB1	5.657216148	Tumor_G2
14013	NDUFB10	7.381251708	Tumor_G2
14014	NDUFB2	6.754489312	Tumor_G2
14015	NDUFB3	6.419550053	Tumor_G2
14016	NDUFB7	8.642947515	Tumor_G2
14017	CYC1	7.013498422	Tumor_G2
14018	ETFB	5.072421216	Tumor_G2
14019	UQCR10	7.693933934	Tumor_G2
14020	UQCRQ	7.380613385	Tumor_G2
14021	UQCC3	4.259865696	Tumor_G2
14022	COX15	5.111778948	Tumor_G2
14023	COX5A	7.564022197	Tumor_G2
14024	COX6B1	8.861468363	Tumor_G2
14025	COX6C	6.691353439	Tumor_G2
14026	COX7A2	7.040652901	Tumor_G2
14027	COX7B	6.400553756	Tumor_G2
14028	COX8A	9.262607394	Tumor_G2
14029	SCO1	3.663127273	Tumor_G2
14030	SURF1	6.467119883	Tumor_G2
14031	ATP6V1E1	6.907466636	Tumor_G2
14032	DLAT	5.716035711	Tumor_G2
14033	PDC	0	Tumor_G2
14034	SUCLG2	6.24402424	Tumor_G2
14035	SLC25A5	8.604493004	Tumor_G2
14036	UCP2	9.592845317	Tumor_G2
14037	SOD2	4.789067843	Tumor_G2
14038	NOX4	2.61589316	Tumor_G2

14039	DUOX1	6.083327088	Tumor_G2
14040	DUOXA1	6.549481801	Tumor_G2
14041	DUOX2	6.013485771	Tumor_G2
14042	DUOXA2	5.016267124	Tumor_G2
14043	NDUFA1	8.368650862	Tumor_G2
14044	NDUFA2	7.107829671	Tumor_G2
14045	NDUFA8	7.502595508	Tumor_G2
14046	NDUFB1	6.08399232	Tumor_G2
14047	NDUFB10	7.971174125	Tumor_G2
14048	NDUFB2	7.310432103	Tumor_G2
14049	NDUFB3	6.777699435	Tumor_G2
14050	NDUFB7	9.032516571	Tumor_G2
14051	CYC1	7.613399283	Tumor_G2
14052	ETFB	5.260480932	Tumor_G2
14053	UQCR10	8.067935111	Tumor_G2
14054	UQCRQ	7.693861373	Tumor_G2
14055	UQCC3	4.340666579	Tumor_G2
14056	COX15	4.774760964	Tumor_G2
14057	COX5A	7.737060009	Tumor_G2
14058	COX6B1	9.09384245	Tumor_G2
14059	COX6C	7.011805069	Tumor_G2
14060	COX7A2	7.338838088	Tumor_G2
14061	COX7B	6.726288811	Tumor_G2
14062	COX8A	10.20402334	Tumor_G2
14063	SCO1	3.280058597	Tumor_G2
14064	SURF1	6.519465963	Tumor_G2
14065	ATP6V1E1	7.375397043	Tumor_G2
14066	DLAT	4.962490636	Tumor_G2
14067	PDC	0.053446217	Tumor_G2
14068	SUCLG2	6.35847112	Tumor_G2
14069	SLC25A5	8.699365049	Tumor_G2
14070	UCP2	8.621167646	Tumor_G2
14071	SOD2	4.771477487	Tumor_G2
14072	NOX4	1.510273	Tumor_G2
14073	DUOX1	5.807042712	Tumor_G2
14074	DUOXA1	5.976909109	Tumor_G2
14075	DUOX2	6.797167869	Tumor_G2
14076	DUOXA2	5.332572191	Tumor_G2
14077	NDUFA1	7.87203082	Tumor_G2
14078	NDUFA2	6.708729782	Tumor_G2

14079	NDUFA8	7.118847714	Tumor_G2
14080	NDUFB1	5.548291005	Tumor_G2
14081	NDUFB10	7.758744155	Tumor_G2
14082	NDUFB2	6.888848403	Tumor_G2
14083	NDUFB3	6.069826336	Tumor_G2
14084	NDUFB7	8.688669753	Tumor_G2
14085	CYC1	7.200427555	Tumor_G2
14086	ETFB	4.810162259	Tumor_G2
14087	UQCR10	7.495990188	Tumor_G2
14088	UQCRQ	7.248490139	Tumor_G2
14089	UQCC3	4.668020394	Tumor_G2
14090	COX15	4.892870245	Tumor_G2
14091	COX5A	7.382273476	Tumor_G2
14092	COX6B1	8.670478866	Tumor_G2
14093	COX6C	6.413693128	Tumor_G2
14094	COX7A2	7.002352499	Tumor_G2
14095	COX7B	6.068271313	Tumor_G2
14096	COX8A	9.601165677	Tumor_G2
14097	SCO1	3.338677333	Tumor_G2
14098	SURF1	6.340999804	Tumor_G2
14099	ATP6V1E1	7.306979099	Tumor_G2
14100	DLAT	5.243254358	Tumor_G2
14101	PDC	0	Tumor_G2
14102	SUCLG2	6.447720961	Tumor_G2
14103	SLC25A5	8.749929421	Tumor_G2
14104	UCP2	7.361292019	Tumor_G2
14105	SOD2	4.594884393	Tumor_G2
14106	NOX4	1.312107917	Tumor_G2
14107	DUOX1	6.317960302	Tumor_G2
14108	DUOXA1	6.313497921	Tumor_G2
14109	DUOX2	6.028678957	Tumor_G2
14110	DUOXA2	3.966028902	Tumor_G2
14111	NDUFA1	7.983820074	Tumor_G2
14112	NDUFA2	7.042004212	Tumor_G2
14113	NDUFA8	7.271361786	Tumor_G2
14114	NDUFB1	5.850094135	Tumor_G2
14115	NDUFB10	7.893612193	Tumor_G2
14116	NDUFB2	6.416565743	Tumor_G2
14117	NDUFB3	6.019570121	Tumor_G2
14118	NDUFB7	8.898130473	Tumor_G2

14119	CYC1	6.752971784	Tumor_G2
14120	ETFB	5.262888225	Tumor_G2
14121	UQCR10	7.754158723	Tumor_G2
14122	UQCRQ	7.462775096	Tumor_G2
14123	UQCC3	4.588204388	Tumor_G2
14124	COX15	4.297189519	Tumor_G2
14125	COX5A	7.173541105	Tumor_G2
14126	COX6B1	9.039088925	Tumor_G2
14127	COX6C	6.469387679	Tumor_G2
14128	COX7A2	6.885093309	Tumor_G2
14129	COX7B	6.232598061	Tumor_G2
14130	COX8A	9.377225864	Tumor_G2
14131	SCO1	2.755208412	Tumor_G2
14132	SURF1	6.516888252	Tumor_G2
14133	ATP6V1E1	6.864199782	Tumor_G2
14134	DLAT	4.41891081	Tumor_G2
14135	PDC	0	Tumor_G2
14136	SUCLG2	5.43402809	Tumor_G2
14137	SLC25A5	6.984002243	Tumor_G2
14138	UCP2	9.592582298	Tumor_G2
14139	SOD2	4.223862589	Tumor_G2
14140	NOX4	2.300213412	Tumor_G2
14141	DUOX1	6.542608589	Tumor_G2
14142	DUOXA1	6.962927921	Tumor_G2
14143	DUOX2	7.161631746	Tumor_G2
14144	DUOXA2	5.729670487	Tumor_G2
14145	NDUFA1	8.316238175	Tumor_G2
14146	NDUFA2	7.149496775	Tumor_G2
14147	NDUFA8	7.342370053	Tumor_G2
14148	NDUFB1	6.103309582	Tumor_G2
14149	NDUFB10	8.061012689	Tumor_G2
14150	NDUFB2	6.589526804	Tumor_G2
14151	NDUFB3	6.513783595	Tumor_G2
14152	NDUFB7	9.109341887	Tumor_G2
14153	CYC1	6.990804638	Tumor_G2
14154	ETFB	5.609000717	Tumor_G2
14155	UQCR10	7.586715603	Tumor_G2
14156	UQCRQ	7.765752392	Tumor_G2
14157	UQCC3	4.960935015	Tumor_G2
14158	COX15	4.774945862	Tumor_G2

14159	COX5A	7.076830533	Tumor_G2
14160	COX6B1	8.979467928	Tumor_G2
14161	COX6C	6.790648985	Tumor_G2
14162	COX7A2	6.938151804	Tumor_G2
14163	COX7B	6.170074521	Tumor_G2
14164	COX8A	9.683976257	Tumor_G2
14165	SCO1	3.212956144	Tumor_G2
14166	SURF1	6.771506743	Tumor_G2
14167	ATP6V1E1	6.401200806	Tumor_G2
14168	DLAT	4.830506656	Tumor_G2
14169	PDC	0	Tumor_G2
14170	SUCLG2	5.938031876	Tumor_G2
14171	SLC25A5	7.503000626	Tumor_G2
14172	UCP2	8.448959291	Tumor_G2
14173	SOD2	4.670913779	Tumor_G2
14174	NOX4	2.265830269	Tumor_G2
14175	DUOX1	6.986773864	Tumor_G2
14176	DUOXA1	7.369576889	Tumor_G2
14177	DUOX2	7.884750808	Tumor_G2
14178	DUOXA2	5.655855911	Tumor_G2
14179	NDUFA1	7.879259492	Tumor_G2
14180	NDUFA2	6.753749882	Tumor_G2
14181	NDUFA8	6.962692457	Tumor_G2
14182	NDUFB1	5.730952049	Tumor_G2
14183	NDUFB10	7.538922081	Tumor_G2
14184	NDUFB2	6.650214509	Tumor_G2
14185	NDUFB3	6.079534352	Tumor_G2
14186	NDUFB7	8.702936052	Tumor_G2
14187	CYC1	6.739259771	Tumor_G2
14188	ETFB	5.136122886	Tumor_G2
14189	UQCR10	7.560566375	Tumor_G2
14190	UQCRQ	7.238424867	Tumor_G2
14191	UQCC3	4.415369296	Tumor_G2
14192	COX15	4.725380269	Tumor_G2
14193	COX5A	6.957224496	Tumor_G2
14194	COX6B1	8.820843985	Tumor_G2
14195	COX6C	6.364281117	Tumor_G2
14196	COX7A2	6.644492761	Tumor_G2
14197	COX7B	5.87069604	Tumor_G2
14198	COX8A	9.30894998	Tumor_G2

14199	SCO1	3.003758716	Tumor_G2
14200	SURF1	6.759031835	Tumor_G2
14201	ATP6V1E1	6.929163036	Tumor_G2
14202	DLAT	4.633683045	Tumor_G2
14203	PDC	0	Tumor_G2
14204	SUCLG2	5.924492274	Tumor_G2
14205	SLC25A5	7.765289023	Tumor_G2
14206	UCP2	8.165393564	Tumor_G2
14207	SOD2	5.187616761	Tumor_G2
14208	NOX4	2.576799549	Tumor_G2
14209	DUOX1	6.329485338	Tumor_G2
14210	DUOXA1	6.554174811	Tumor_G2
14211	DUOX2	6.460620852	Tumor_G2
14212	DUOXA2	4.977939222	Tumor_G2
14213	NDUFA1	8.001355738	Tumor_G2
14214	NDUFA2	6.879416196	Tumor_G2
14215	NDUFA8	7.15310959	Tumor_G2
14216	NDUFB1	5.792779219	Tumor_G2
14217	NDUFB10	7.897449676	Tumor_G2
14218	NDUFB2	6.473057073	Tumor_G2
14219	NDUFB3	6.274084779	Tumor_G2
14220	NDUFB7	8.978995104	Tumor_G2
14221	CYC1	7.139577522	Tumor_G2
14222	ETFB	5.708644825	Tumor_G2
14223	UQCR10	7.862867401	Tumor_G2
14224	UQCRQ	7.335189107	Tumor_G2
14225	UQCC3	4.904249871	Tumor_G2
14226	COX15	5.026125739	Tumor_G2
14227	COX5A	7.124787138	Tumor_G2
14228	COX6B1	8.866931867	Tumor_G2
14229	COX6C	6.399085365	Tumor_G2
14230	COX7A2	6.909836521	Tumor_G2
14231	COX7B	5.772393605	Tumor_G2
14232	COX8A	9.870686172	Tumor_G2
14233	SCO1	3.27720916	Tumor_G2
14234	SURF1	6.801945616	Tumor_G2
14235	ATP6V1E1	7.073907533	Tumor_G2
14236	DLAT	4.762730758	Tumor_G2
14237	PDC	0.074584696	Tumor_G2
14238	SUCLG2	5.958935461	Tumor_G2

14239	SLC25A5	7.705849199	Tumor_G2
14240	UCP2	8.991832106	Tumor_G2
14241	SOD2	4.876228996	Tumor_G2
14242	NOX4	2.945084838	Tumor_G2
14243	DUOX1	7.123681111	Tumor_G2
14244	DUOXA1	7.349597268	Tumor_G2
14245	DUOX2	7.520958193	Tumor_G2
14246	DUOXA2	6.077630098	Tumor_G2
14247	NDUFA1	8.240627187	Tumor_G2
14248	NDUFA2	6.974517346	Tumor_G2
14249	NDUFA8	7.24555558	Tumor_G2
14250	NDUFB1	5.978435409	Tumor_G2
14251	NDUFB10	8.043945535	Tumor_G2
14252	NDUFB2	6.946792891	Tumor_G2
14253	NDUFB3	6.354912688	Tumor_G2
14254	NDUFB7	9.276748411	Tumor_G2
14255	CYC1	7.12452563	Tumor_G2
14256	ETFB	5.966141926	Tumor_G2
14257	UQCR10	7.98249623	Tumor_G2
14258	UQCRQ	7.410652916	Tumor_G2
14259	UQCC3	5.017382106	Tumor_G2
14260	COX15	5.066410989	Tumor_G2
14261	COX5A	7.096290446	Tumor_G2
14262	COX6B1	9.087697637	Tumor_G2
14263	COX6C	6.591380444	Tumor_G2
14264	COX7A2	7.091082663	Tumor_G2
14265	COX7B	6.047728893	Tumor_G2
14266	COX8A	10.00675168	Tumor_G2
14267	SCO1	3.226234305	Tumor_G2
14268	SURF1	6.908448683	Tumor_G2
14269	ATP6V1E1	7.20132295	Tumor_G2
14270	DLAT	4.612211402	Tumor_G2
14271	PDC	0.238638961	Tumor_G2
14272	SUCLG2	5.873868249	Tumor_G2
14273	SLC25A5	7.391928863	Tumor_G2
14274	UCP2	9.387257076	Tumor_G2
14275	SOD2	4.027381369	Tumor_G2
14276	NOX4	1.509509074	Tumor_G2
14277	DUOX1	7.005277842	Tumor_G2
14278	DUOXA1	7.08687482	Tumor_G2

14279	DUOX2	7.673500016	Tumor_G2
14280	DUOXA2	6.186147335	Tumor_G2
14281	NDUFA1	7.521066273	Tumor_G2
14282	NDUFA2	6.42067019	Tumor_G2
14283	NDUFA8	7.070989439	Tumor_G2
14284	NDUFB1	5.367127685	Tumor_G2
14285	NDUFB10	7.381541731	Tumor_G2
14286	NDUFB2	6.263757948	Tumor_G2
14287	NDUFB3	6.092808934	Tumor_G2
14288	NDUFB7	8.588574092	Tumor_G2
14289	CYC1	6.682808213	Tumor_G2
14290	ETFB	4.948388597	Tumor_G2
14291	UQCR10	7.575151645	Tumor_G2
14292	UQCRQ	7.102132068	Tumor_G2
14293	UQCC3	4.35145316	Tumor_G2
14294	COX15	4.779006811	Tumor_G2
14295	COX5A	7.124786529	Tumor_G2
14296	COX6B1	8.538515437	Tumor_G2
14297	COX6C	6.322355773	Tumor_G2
14298	COX7A2	6.852154223	Tumor_G2
14299	COX7B	5.894026176	Tumor_G2
14300	COX8A	9.340001726	Tumor_G2
14301	SCO1	3.49130813	Tumor_G2
14302	SURF1	6.583957646	Tumor_G2
14303	ATP6V1E1	7.127802263	Tumor_G2
14304	DLAT	5.228671051	Tumor_G2
14305	PDC	0.268163827	Tumor_G2
14306	SUCLG2	6.474672322	Tumor_G2
14307	SLC25A5	7.489460484	Tumor_G2
14308	UCP2	10.14170622	Tumor_G2
14309	SOD2	4.673628008	Tumor_G2
14310	NOX4	2.835543308	Tumor_G2
14311	DUOX1	6.777164225	Tumor_G2
14312	DUOXA1	7.260846764	Tumor_G2
14313	DUOX2	8.326855705	Tumor_G2
14314	DUOXA2	5.65697994	Tumor_G2
14315	NDUFA1	7.744418292	Tumor_G2
14316	NDUFA2	6.621815904	Tumor_G2
14317	NDUFA8	7.18450371	Tumor_G2
14318	NDUFB1	5.740832938	Tumor_G2

14319	NDUFB10	7.60727646	Tumor_G2
14320	NDUFB2	6.161935099	Tumor_G2
14321	NDUFB3	6.06791302	Tumor_G2
14322	NDUFB7	8.648329265	Tumor_G2
14323	CYC1	6.703572213	Tumor_G2
14324	ETFB	5.258338146	Tumor_G2
14325	UQCR10	7.602189441	Tumor_G2
14326	UQCRQ	7.198320685	Tumor_G2
14327	UQCC3	4.406007244	Tumor_G2
14328	COX15	4.788002773	Tumor_G2
14329	COX5A	6.882007164	Tumor_G2
14330	COX6B1	8.632533942	Tumor_G2
14331	COX6C	6.504528582	Tumor_G2
14332	COX7A2	6.735479025	Tumor_G2
14333	COX7B	5.927340121	Tumor_G2
14334	COX8A	9.339919022	Tumor_G2
14335	SCO1	3.21537555	Tumor_G2
14336	SURF1	6.559633061	Tumor_G2
14337	ATP6V1E1	6.955076236	Tumor_G2
14338	DLAT	4.799734324	Tumor_G2
14339	PDC	0.530254869	Tumor_G2
14340	SUCLG2	6.133547188	Tumor_G2
14341	SLC25A5	7.184054263	Tumor_G2
14342	UCP2	9.62133139	Tumor_G2
14343	SOD2	4.113945944	Tumor_G2
14344	NOX4	1.961423367	Tumor_G2
14345	DUOX1	6.862108408	Tumor_G2
14346	DUOXA1	7.012372907	Tumor_G2
14347	DUOX2	7.942783168	Tumor_G2
14348	DUOXA2	5.321698227	Tumor_G2
14349	NDUFA1	8.524672152	Tumor_G2
14350	NDUFA2	6.98872705	Tumor_G2
14351	NDUFA8	7.402001838	Tumor_G2
14352	NDUFB1	6.172361523	Tumor_G2
14353	NDUFB10	7.755347238	Tumor_G2
14354	NDUFB2	7.256801526	Tumor_G2
14355	NDUFB3	6.661090207	Tumor_G2
14356	NDUFB7	8.957118968	Tumor_G2
14357	CYC1	7.047755839	Tumor_G2
14358	ETFB	5.644951763	Tumor_G2

14359	UQCR10	7.106823555	Tumor_G2
14360	UQCRQ	7.686528756	Tumor_G2
14361	UQCC3	4.602387711	Tumor_G2
14362	COX15	5.241499784	Tumor_G2
14363	COX5A	7.296171588	Tumor_G2
14364	COX6B1	8.714872641	Tumor_G2
14365	COX6C	6.750807605	Tumor_G2
14366	COX7A2	7.114769286	Tumor_G2
14367	COX7B	6.31786413	Tumor_G2
14368	COX8A	10.08287101	Tumor_G2
14369	SCO1	3.765318472	Tumor_G2
14370	SURF1	7.123199666	Tumor_G2
14371	ATP6V1E1	7.436294005	Tumor_G2
14372	DLAT	5.243760703	Tumor_G2
14373	PDC	0.372717672	Tumor_G2
14374	SUCLG2	6.633222716	Tumor_G2
14375	SLC25A5	8.304632072	Tumor_G2
14376	UCP2	8.116668125	Tumor_G2
14377	SOD2	4.378049555	Tumor_G2
14378	NOX4	1.08199268	Tumor_G2
14379	DUOX1	8.011436715	Tumor_G2
14380	DUOXA1	7.782936685	Tumor_G2
14381	DUOX2	10.23348118	Tumor_G2
14382	DUOXA2	7.691985358	Tumor_G2
14383	NDUFA1	9.898541883	Tumor_G3
14384	NDUFA2	8.292576226	Tumor_G3
14385	NDUFA8	7.736217241	Tumor_G3
14386	NDUFB1	7.43999063	Tumor_G3
14387	NDUFB10	8.131394793	Tumor_G3
14388	NDUFB2	6.552561223	Tumor_G3
14389	NDUFB3	5.860393187	Tumor_G3
14390	NDUFB7	9.930699625	Tumor_G3
14391	CYC1	6.51165398	Tumor_G3
14392	ETFB	5.371225214	Tumor_G3
14393	UQCR10	8.575467693	Tumor_G3
14394	UQCRQ	8.178275654	Tumor_G3
14395	UQCC3	5.257566388	Tumor_G3
14396	COX15	3.82922538	Tumor_G3
14397	COX5A	6.992266403	Tumor_G3
14398	COX6B1	10.08362297	Tumor_G3

14399	COX6C	7.571331862	Tumor_G3
14400	COX7A2	7.179447486	Tumor_G3
14401	COX7B	6.785771427	Tumor_G3
14402	COX8A	9.865157919	Tumor_G3
14403	SCO1	1.752365128	Tumor_G3
14404	SURF1	6.951967559	Tumor_G3
14405	ATP6V1E1	6.007590059	Tumor_G3
14406	DLAT	3.788322852	Tumor_G3
14407	PDC	0.179023048	Tumor_G3
14408	SUCLG2	4.931339433	Tumor_G3
14409	SLC25A5	5.949447018	Tumor_G3
14410	UCP2	7.394772359	Tumor_G3
14411	SOD2	3.558399108	Tumor_G3
14412	NOX4	1.000852954	Tumor_G3
14413	DUOX1	7.797291746	Tumor_G3
14414	DUOXA1	7.701750318	Tumor_G3
14415	DUOX2	7.960352253	Tumor_G3
14416	DUOXA2	6.568705799	Tumor_G3
14417	NDUFA1	9.087534411	Tumor_G3
14418	NDUFA2	7.369122621	Tumor_G3
14419	NDUFA8	7.885635559	Tumor_G3
14420	NDUFB1	6.33571518	Tumor_G3
14421	NDUFB10	8.064150881	Tumor_G3
14422	NDUFB2	7.099928017	Tumor_G3
14423	NDUFB3	6.98698306	Tumor_G3
14424	NDUFB7	8.811912393	Tumor_G3
14425	CYC1	8.005481775	Tumor_G3
14426	ETFB	6.485638038	Tumor_G3
14427	UQCR10	8.665016775	Tumor_G3
14428	UQCRQ	8.075433235	Tumor_G3
14429	UQCC3	4.770087013	Tumor_G3
14430	COX15	5.264275104	Tumor_G3
14431	COX5A	8.36665742	Tumor_G3
14432	COX6B1	9.332859294	Tumor_G3
14433	COX6C	7.586764509	Tumor_G3
14434	COX7A2	7.528878264	Tumor_G3
14435	COX7B	7.351953533	Tumor_G3
14436	COX8A	10.33588497	Tumor_G3
14437	SCO1	4.025404685	Tumor_G3
14438	SURF1	6.690208413	Tumor_G3

14439	ATP6V1E1	6.966236063	Tumor_G3
14440	DLAT	5.562310153	Tumor_G3
14441	PDC	0	Tumor_G3
14442	SUCLG2	6.404921776	Tumor_G3
14443	SLC25A5	8.543095142	Tumor_G3
14444	UCP2	8.777074979	Tumor_G3
14445	SOD2	4.999350492	Tumor_G3
14446	NOX4	0.953108748	Tumor_G3
14447	DUOX1	7.786966941	Tumor_G3
14448	DUOXA1	5.800818831	Tumor_G3
14449	DUOX2	7.215169263	Tumor_G3
14450	DUOXA2	7.980031862	Tumor_G3
14451	NDUFA1	8.959137812	Tumor_G3
14452	NDUFA2	7.515523888	Tumor_G3
14453	NDUFA8	7.962384726	Tumor_G3
14454	NDUFB1	6.550045709	Tumor_G3
14455	NDUFB10	8.150033846	Tumor_G3
14456	NDUFB2	7.221325683	Tumor_G3
14457	NDUFB3	6.992376798	Tumor_G3
14458	NDUFB7	9.52194418	Tumor_G3
14459	CYC1	7.882544595	Tumor_G3
14460	ETFB	6.654789373	Tumor_G3
14461	UQCR10	8.615822647	Tumor_G3
14462	UQCRQ	8.049916146	Tumor_G3
14463	UQCC3	5.256786215	Tumor_G3
14464	COX15	5.448516426	Tumor_G3
14465	COX5A	8.288210301	Tumor_G3
14466	COX6B1	9.265741992	Tumor_G3
14467	COX6C	7.641313459	Tumor_G3
14468	COX7A2	7.546217619	Tumor_G3
14469	COX7B	7.185043454	Tumor_G3
14470	COX8A	10.47221843	Tumor_G3
14471	SCO1	3.971000045	Tumor_G3
14472	SURF1	7.346629344	Tumor_G3
14473	ATP6V1E1	7.122310735	Tumor_G3
14474	DLAT	5.502913769	Tumor_G3
14475	PDC	0.091014874	Tumor_G3
14476	SUCLG2	6.824656031	Tumor_G3
14477	SLC25A5	8.641031811	Tumor_G3
14478	UCP2	8.444675513	Tumor_G3

14479	SOD2	4.935384053	Tumor_G3
14480	NOX4	1.636811212	Tumor_G3
14481	DUOX1	7.52465459	Tumor_G3
14482	DUOXA1	5.940414559	Tumor_G3
14483	DUOX2	8.440388978	Tumor_G3
14484	DUOXA2	8.974269068	Tumor_G3
14485	NDUFA1	8.553039923	Tumor_G3
14486	NDUFA2	7.352508441	Tumor_G3
14487	NDUFA8	7.527209338	Tumor_G3
14488	NDUFB1	6.200342822	Tumor_G3
14489	NDUFB10	8.071366865	Tumor_G3
14490	NDUFB2	7.223458328	Tumor_G3
14491	NDUFB3	7.072384443	Tumor_G3
14492	NDUFB7	7.895947752	Tumor_G3
14493	CYC1	7.284068691	Tumor_G3
14494	ETFB	5.113463284	Tumor_G3
14495	UQCR10	8.350837128	Tumor_G3
14496	UQCRQ	7.774088183	Tumor_G3
14497	UQCC3	4.132090672	Tumor_G3
14498	COX15	5.687873619	Tumor_G3
14499	COX5A	7.845804746	Tumor_G3
14500	COX6B1	8.999665516	Tumor_G3
14501	COX6C	7.03398831	Tumor_G3
14502	COX7A2	6.851905345	Tumor_G3
14503	COX7B	6.775813335	Tumor_G3
14504	COX8A	9.53163204	Tumor_G3
14505	SCO1	3.559669012	Tumor_G3
14506	SURF1	6.61075551	Tumor_G3
14507	ATP6V1E1	6.922747485	Tumor_G3
14508	DLAT	5.547052692	Tumor_G3
14509	PDC	0.246955999	Tumor_G3
14510	SUCLG2	6.053516425	Tumor_G3
14511	SLC25A5	8.152070417	Tumor_G3
14512	UCP2	6.453199544	Tumor_G3
14513	SOD2	5.156041142	Tumor_G3
14514	NOX4	0.345578897	Tumor_G3
14515	DUOX1	6.3622981	Tumor_G3
14516	DUOXA1	6.230044906	Tumor_G3
14517	DUOX2	6.491358095	Tumor_G3
14518	DUOXA2	4.608450683	Tumor_G3

14519	NDUFA1	8.576297457	Tumor_G3
14520	NDUFA2	7.002217096	Tumor_G3
14521	NDUFA8	7.890050405	Tumor_G3
14522	NDUFB1	6.123330008	Tumor_G3
14523	NDUFB10	8.132760262	Tumor_G3
14524	NDUFB2	7.572615065	Tumor_G3
14525	NDUFB3	6.679841601	Tumor_G3
14526	NDUFB7	8.755365314	Tumor_G3
14527	CYC1	7.644666093	Tumor_G3
14528	ETFB	5.537517414	Tumor_G3
14529	UQCR10	8.76426676	Tumor_G3
14530	UQCRQ	7.603982948	Tumor_G3
14531	UQCC3	4.84182728	Tumor_G3
14532	COX15	5.317695573	Tumor_G3
14533	COX5A	7.51807461	Tumor_G3
14534	COX6B1	9.179177847	Tumor_G3
14535	COX6C	7.002524269	Tumor_G3
14536	COX7A2	7.111097546	Tumor_G3
14537	COX7B	6.888335642	Tumor_G3
14538	COX8A	10.32596001	Tumor_G3
14539	SCO1	4.381906959	Tumor_G3
14540	SURF1	6.780865936	Tumor_G3
14541	ATP6V1E1	6.434500387	Tumor_G3
14542	DLAT	5.514954232	Tumor_G3
14543	PDC	0.252629849	Tumor_G3
14544	SUCLG2	6.200597373	Tumor_G3
14545	SLC25A5	8.5519048	Tumor_G3
14546	UCP2	7.818053036	Tumor_G3
14547	SOD2	5.137079085	Tumor_G3
14548	NOX4	2.122507455	Tumor_G3
14549	DUOX1	8.291241359	Tumor_G3
14550	DUOXA1	7.332691038	Tumor_G3
14551	DUOX2	8.919295908	Tumor_G3
14552	DUOXA2	8.167490532	Tumor_G3
14553	NDUFA1	8.795414463	Tumor_G3
14554	NDUFA2	7.562071447	Tumor_G3
14555	NDUFA8	7.318430946	Tumor_G3
14556	NDUFB1	6.580115034	Tumor_G3
14557	NDUFB10	8.271812986	Tumor_G3
14558	NDUFB2	7.026057878	Tumor_G3

14559	NDUFB3	6.913833966	Tumor_G3
14560	NDUFB7	9.356971394	Tumor_G3
14561	CYC1	7.353233111	Tumor_G3
14562	ETFB	5.686831927	Tumor_G3
14563	UQCR10	8.412979402	Tumor_G3
14564	UQCRQ	7.856320965	Tumor_G3
14565	UQCC3	5.266798996	Tumor_G3
14566	COX15	4.551038425	Tumor_G3
14567	COX5A	7.742823824	Tumor_G3
14568	COX6B1	9.558005943	Tumor_G3
14569	COX6C	7.161975212	Tumor_G3
14570	COX7A2	7.370025368	Tumor_G3
14571	COX7B	6.587228039	Tumor_G3
14572	COX8A	10.04456913	Tumor_G3
14573	SCO1	2.859335558	Tumor_G3
14574	SURF1	6.50999996	Tumor_G3
14575	ATP6V1E1	6.658592683	Tumor_G3
14576	DLAT	3.807782963	Tumor_G3
14577	PDC	0	Tumor_G3
14578	SUCLG2	5.546245851	Tumor_G3
14579	SLC25A5	8.584597168	Tumor_G3
14580	UCP2	8.379842718	Tumor_G3
14581	SOD2	4.624252455	Tumor_G3
14582	NOX4	0.495763327	Tumor_G3
14583	DUOX1	4.621775589	Tumor_G3
14584	DUOXA1	4.812591054	Tumor_G3
14585	DUOX2	4.416553995	Tumor_G3
14586	DUOXA2	4.734882572	Tumor_G3
14587	NDUFA1	9.221374131	Tumor_G3
14588	NDUFA2	7.701979417	Tumor_G3
14589	NDUFA8	8.102896943	Tumor_G3
14590	NDUFB1	7.357512872	Tumor_G3
14591	NDUFB10	8.279059971	Tumor_G3
14592	NDUFB2	7.750725533	Tumor_G3
14593	NDUFB3	7.40615409	Tumor_G3
14594	NDUFB7	9.729037512	Tumor_G3
14595	CYC1	7.718263824	Tumor_G3
14596	ETFB	6.326111067	Tumor_G3
14597	UQCR10	8.789455872	Tumor_G3
14598	UQCRQ	8.389364036	Tumor_G3

14599	UQCC3	5.046323555	Tumor_G3
14600	COX15	4.514653432	Tumor_G3
14601	COX5A	7.808626362	Tumor_G3
14602	COX6B1	9.638270478	Tumor_G3
14603	COX6C	7.44306451	Tumor_G3
14604	COX7A2	7.727062941	Tumor_G3
14605	COX7B	7.327727005	Tumor_G3
14606	COX8A	10.71374276	Tumor_G3
14607	SCO1	3.14720576	Tumor_G3
14608	SURF1	6.624324979	Tumor_G3
14609	ATP6V1E1	7.078251165	Tumor_G3
14610	DLAT	4.601349354	Tumor_G3
14611	PDC	0	Tumor_G3
14612	SUCLG2	5.466864856	Tumor_G3
14613	SLC25A5	8.293410073	Tumor_G3
14614	UCP2	7.010906826	Tumor_G3
14615	SOD2	4.645967229	Tumor_G3
14616	NOX4	2.302798046	Tumor_G3
14617	DUOX1	7.119221178	Tumor_G3
14618	DUOXA1	6.853323326	Tumor_G3
14619	DUOX2	7.17705412	Tumor_G3
14620	DUOXA2	5.620739379	Tumor_G3
14621	NDUFA1	8.736609035	Tumor_G3
14622	NDUFA2	7.888830678	Tumor_G3
14623	NDUFA8	7.753570345	Tumor_G3
14624	NDUFB1	7.127971509	Tumor_G3
14625	NDUFB10	8.352779814	Tumor_G3
14626	NDUFB2	7.785015549	Tumor_G3
14627	NDUFB3	7.631318043	Tumor_G3
14628	NDUFB7	9.704356185	Tumor_G3
14629	CYC1	7.643371333	Tumor_G3
14630	ETFB	5.516424399	Tumor_G3
14631	UQCR10	8.793184708	Tumor_G3
14632	UQCRQ	8.445265707	Tumor_G3
14633	UQCC3	5.437928755	Tumor_G3
14634	COX15	4.999057135	Tumor_G3
14635	COX5A	8.056754081	Tumor_G3
14636	COX6B1	9.628042915	Tumor_G3
14637	COX6C	7.493795423	Tumor_G3
14638	COX7A2	8.040830232	Tumor_G3

14639	COX7B	7.697001449	Tumor_G3
14640	COX8A	10.76236445	Tumor_G3
14641	SCO1	3.645359152	Tumor_G3
14642	SURF1	7.229231276	Tumor_G3
14643	ATP6V1E1	6.892698499	Tumor_G3
14644	DLAT	5.164403967	Tumor_G3
14645	PDC	0	Tumor_G3
14646	SUCLG2	6.202789558	Tumor_G3
14647	SLC25A5	8.670692196	Tumor_G3
14648	UCP2	8.004034505	Tumor_G3
14649	SOD2	4.515366003	Tumor_G3
14650	NOX4	2.370742693	Tumor_G3
14651	DUOX1	6.816647709	Tumor_G3
14652	DUOXA1	7.057916328	Tumor_G3
14653	DUOX2	8.789442886	Tumor_G3
14654	DUOXA2	7.978480798	Tumor_G3
14655	NDUFA1	9.106548319	Tumor_G3
14656	NDUFA2	8.097954838	Tumor_G3
14657	NDUFA8	7.461763313	Tumor_G3
14658	NDUFB1	6.693798255	Tumor_G3
14659	NDUFB10	8.55104297	Tumor_G3
14660	NDUFB2	8.377708041	Tumor_G3
14661	NDUFB3	7.259116706	Tumor_G3
14662	NDUFB7	10.03825797	Tumor_G3
14663	CYC1	7.600363364	Tumor_G3
14664	ETFB	5.895548939	Tumor_G3
14665	UQCR10	8.550955369	Tumor_G3
14666	UQCRQ	8.587256907	Tumor_G3
14667	UQCC3	5.589811544	Tumor_G3
14668	COX15	4.929496414	Tumor_G3
14669	COX5A	7.815093921	Tumor_G3
14670	COX6B1	9.312342421	Tumor_G3
14671	COX6C	7.223638432	Tumor_G3
14672	COX7A2	7.400134739	Tumor_G3
14673	COX7B	6.929136415	Tumor_G3
14674	COX8A	10.59553441	Tumor_G3
14675	SCO1	3.131097333	Tumor_G3
14676	SURF1	7.509877734	Tumor_G3
14677	ATP6V1E1	7.144844068	Tumor_G3
14678	DLAT	4.868956526	Tumor_G3

14679	PDC	0	Tumor_G3
14680	SUCLG2	5.878588568	Tumor_G3
14681	SLC25A5	8.38995202	Tumor_G3
14682	UCP2	6.628099975	Tumor_G3
14683	SOD2	4.79192776	Tumor_G3
14684	NOX4	0.295551438	Tumor_G3
14685	DUOX1	5.677605168	Tumor_G3
14686	DUOXA1	5.827436201	Tumor_G3
14687	DUOX2	6.591030348	Tumor_G3
14688	DUOXA2	7.275318932	Tumor_G3
14689	NDUFA1	9.330296163	Tumor_G3
14690	NDUFA2	8.174437865	Tumor_G3
14691	NDUFA8	8.289096435	Tumor_G3
14692	NDUFB1	7.451134258	Tumor_G3
14693	NDUFB10	8.89229361	Tumor_G3
14694	NDUFB2	7.360097565	Tumor_G3
14695	NDUFB3	6.655529888	Tumor_G3
14696	NDUFB7	10.11643221	Tumor_G3
14697	CYC1	7.316861794	Tumor_G3
14698	ETFB	6.534481777	Tumor_G3
14699	UQCR10	8.044107993	Tumor_G3
14700	UQCRQ	8.623478643	Tumor_G3
14701	UQCC3	5.962590819	Tumor_G3
14702	COX15	4.650274277	Tumor_G3
14703	COX5A	8.125410863	Tumor_G3
14704	COX6B1	10.02361065	Tumor_G3
14705	COX6C	7.65601572	Tumor_G3
14706	COX7A2	7.487455181	Tumor_G3
14707	COX7B	7.269211613	Tumor_G3
14708	COX8A	11.01540807	Tumor_G3
14709	SCO1	2.883433832	Tumor_G3
14710	SURF1	7.049374887	Tumor_G3
14711	ATP6V1E1	6.375982248	Tumor_G3
14712	DLAT	4.192273123	Tumor_G3
14713	PDC	0.136663902	Tumor_G3
14714	SUCLG2	5.61809424	Tumor_G3
14715	SLC25A5	7.502022885	Tumor_G3
14716	UCP2	8.704617821	Tumor_G3
14717	SOD2	3.834484907	Tumor_G3
14718	NOX4	1.988467294	Tumor_G3

14719	DUOX1	7.57301627	Tumor_G3
14720	DUOXA1	7.239682261	Tumor_G3
14721	DUOX2	8.821405572	Tumor_G3
14722	DUOXA2	7.694198537	Tumor_G3
14723	NDUFA1	8.486372969	Tumor_G3
14724	NDUFA2	7.470471367	Tumor_G3
14725	NDUFA8	8.385416243	Tumor_G3
14726	NDUFB1	6.066696663	Tumor_G3
14727	NDUFB10	8.252339689	Tumor_G3
14728	NDUFB2	8.29215566	Tumor_G3
14729	NDUFB3	6.713593166	Tumor_G3
14730	NDUFB7	9.884548796	Tumor_G3
14731	CYC1	7.858410942	Tumor_G3
14732	ETFB	6.196397949	Tumor_G3
14733	UQCR10	8.574210754	Tumor_G3
14734	UQCRQ	8.033096558	Tumor_G3
14735	UQCC3	5.453312702	Tumor_G3
14736	COX15	5.255575401	Tumor_G3
14737	COX5A	6.937029881	Tumor_G3
14738	COX6B1	9.314569262	Tumor_G3
14739	COX6C	6.919425211	Tumor_G3
14740	COX7A2	7.189901186	Tumor_G3
14741	COX7B	6.757874658	Tumor_G3
14742	COX8A	10.91109021	Tumor_G3
14743	SCO1	3.833489136	Tumor_G3
14744	SURF1	7.270614375	Tumor_G3
14745	ATP6V1E1	6.993284054	Tumor_G3
14746	DLAT	4.895383822	Tumor_G3
14747	PDC	0.154930819	Tumor_G3
14748	SUCLG2	6.31057126	Tumor_G3
14749	SLC25A5	8.646523251	Tumor_G3
14750	UCP2	9.260734305	Tumor_G3
14751	SOD2	4.437745763	Tumor_G3
14752	NOX4	2.461315527	Tumor_G3
14753	DUOX1	6.117100932	Tumor_G3
14754	DUOXA1	5.474903398	Tumor_G3
14755	DUOX2	5.928805365	Tumor_G3
14756	DUOXA2	5.375584318	Tumor_G3
14757	NDUFA1	8.778747635	Tumor_G3
14758	NDUFA2	7.6071948	Tumor_G3

14759	NDUFA8	7.806334334	Tumor_G3
14760	NDUFB1	6.551666054	Tumor_G3
14761	NDUFB10	8.06729541	Tumor_G3
14762	NDUFB2	7.50051725	Tumor_G3
14763	NDUFB3	6.741412483	Tumor_G3
14764	NDUFB7	9.587258735	Tumor_G3
14765	CYC1	7.525860844	Tumor_G3
14766	ETFB	5.806722233	Tumor_G3
14767	UQCR10	8.226576791	Tumor_G3
14768	UQCRQ	8.041372744	Tumor_G3
14769	UQCC3	5.30704105	Tumor_G3
14770	COX15	4.633052539	Tumor_G3
14771	COX5A	7.598256067	Tumor_G3
14772	COX6B1	9.484332787	Tumor_G3
14773	COX6C	7.229057503	Tumor_G3
14774	COX7A2	7.485101178	Tumor_G3
14775	COX7B	6.654350908	Tumor_G3
14776	COX8A	10.34890084	Tumor_G3
14777	SCO1	2.96942767	Tumor_G3
14778	SURF1	6.424427098	Tumor_G3
14779	ATP6V1E1	6.816991816	Tumor_G3
14780	DLAT	4.758407781	Tumor_G3
14781	PDC	0	Tumor_G3
14782	SUCLG2	5.724006006	Tumor_G3
14783	SLC25A5	8.375310948	Tumor_G3
14784	UCP2	8.31431667	Tumor_G3
14785	SOD2	5.054686199	Tumor_G3
14786	NOX4	1.374015869	Tumor_G3
14787	DUOX1	6.558788371	Tumor_G3
14788	DUOXA1	6.632760234	Tumor_G3
14789	DUOX2	7.390802612	Tumor_G3
14790	DUOXA2	6.060150358	Tumor_G3
14791	NDUFA1	8.489761328	Tumor_G3
14792	NDUFA2	7.717738456	Tumor_G3
14793	NDUFA8	7.341644889	Tumor_G3
14794	NDUFB1	6.253635135	Tumor_G3
14795	NDUFB10	7.688038943	Tumor_G3
14796	NDUFB2	7.442410721	Tumor_G3
14797	NDUFB3	6.634470623	Tumor_G3
14798	NDUFB7	9.449179841	Tumor_G3

14799	CYC1	7.317102932	Tumor_G3
14800	ETFB	5.542977248	Tumor_G3
14801	UQCR10	8.302999442	Tumor_G3
14802	UQCRQ	8.042409871	Tumor_G3
14803	UQCC3	5.021409102	Tumor_G3
14804	COX15	4.273677782	Tumor_G3
14805	COX5A	7.575952483	Tumor_G3
14806	COX6B1	9.467281475	Tumor_G3
14807	COX6C	7.046514794	Tumor_G3
14808	COX7A2	7.584504104	Tumor_G3
14809	COX7B	6.243489332	Tumor_G3
14810	COX8A	9.789315262	Tumor_G3
14811	SCO1	2.777969561	Tumor_G3
14812	SURF1	6.591444994	Tumor_G3
14813	ATP6V1E1	7.658490952	Tumor_G3
14814	DLAT	4.442487294	Tumor_G3
14815	PDC	0	Tumor_G3
14816	SUCLG2	6.090490153	Tumor_G3
14817	SLC25A5	8.379049339	Tumor_G3
14818	UCP2	8.385908836	Tumor_G3
14819	SOD2	4.991551939	Tumor_G3
14820	NOX4	1.927062624	Tumor_G3
14821	DUOX1	5.005943773	Tumor_G3
14822	DUOXA1	5.677541996	Tumor_G3
14823	DUOX2	4.053917948	Tumor_G3
14824	DUOXA2	3.72180284	Tumor_G3
14825	NDUFA1	8.698112594	Tumor_G3
14826	NDUFA2	7.929559564	Tumor_G3
14827	NDUFA8	8.070027212	Tumor_G3
14828	NDUFB1	6.755411065	Tumor_G3
14829	NDUFB10	8.6916988	Tumor_G3
14830	NDUFB2	8.192920442	Tumor_G3
14831	NDUFB3	7.011924992	Tumor_G3
14832	NDUFB7	10.39274298	Tumor_G3
14833	CYC1	8.15456681	Tumor_G3
14834	ETFB	6.832916603	Tumor_G3
14835	UQCR10	8.008628395	Tumor_G3
14836	UQCRQ	8.997327745	Tumor_G3
14837	UQCC3	5.825096549	Tumor_G3
14838	COX15	4.79149468	Tumor_G3

14839	COX5A	8.169765333	Tumor_G3
14840	COX6B1	9.967461494	Tumor_G3
14841	COX6C	7.571707959	Tumor_G3
14842	COX7A2	7.927207436	Tumor_G3
14843	COX7B	6.995925539	Tumor_G3
14844	COX8A	11.29907536	Tumor_G3
14845	SCO1	3.443608565	Tumor_G3
14846	SURF1	7.489042419	Tumor_G3
14847	ATP6V1E1	6.43853008	Tumor_G3
14848	DLAT	4.126479896	Tumor_G3
14849	PDC	0	Tumor_G3
14850	SUCLG2	6.028085371	Tumor_G3
14851	SLC25A5	8.83830858	Tumor_G3
14852	UCP2	8.956359341	Tumor_G3
14853	SOD2	3.987578271	Tumor_G3
14854	NOX4	0.974637894	Tumor_G3
14855	DUOX1	7.44974138	Tumor_G3
14856	DUOXA1	7.188476593	Tumor_G3
14857	DUOX2	8.479109552	Tumor_G3
14858	DUOXA2	8.119236689	Tumor_G3
14859	NDUFA1	8.668291736	Tumor_G3
14860	NDUFA2	7.10100372	Tumor_G3
14861	NDUFA8	7.866778257	Tumor_G3
14862	NDUFB1	6.320734779	Tumor_G3
14863	NDUFB10	8.373752777	Tumor_G3
14864	NDUFB2	7.44361745	Tumor_G3
14865	NDUFB3	6.627614365	Tumor_G3
14866	NDUFB7	9.627482745	Tumor_G3
14867	CYC1	7.75035522	Tumor_G3
14868	ETFB	5.594034707	Tumor_G3
14869	UQCR10	8.86807829	Tumor_G3
14870	UQCRQ	7.936021466	Tumor_G3
14871	UQCC3	5.247018177	Tumor_G3
14872	COX15	4.727694016	Tumor_G3
14873	COX5A	7.853053834	Tumor_G3
14874	COX6B1	9.334579657	Tumor_G3
14875	COX6C	6.939048005	Tumor_G3
14876	COX7A2	7.164796768	Tumor_G3
14877	COX7B	7.047537805	Tumor_G3
14878	COX8A	10.57779195	Tumor_G3

14879	SCO1	4.051448077	Tumor_G3
14880	SURF1	6.747257478	Tumor_G3
14881	ATP6V1E1	6.2624486	Tumor_G3
14882	DLAT	5.279814093	Tumor_G3
14883	PDC	0.285676216	Tumor_G3
14884	SUCLG2	5.584106518	Tumor_G3
14885	SLC25A5	8.438934139	Tumor_G3
14886	UCP2	7.872242768	Tumor_G3
14887	SOD2	4.664616633	Tumor_G3
14888	NOX4	2.051624456	Tumor_G3
14889	DUOX1	7.846359097	Tumor_G3
14890	DUOXA1	7.366454319	Tumor_G3
14891	DUOX2	8.85830774	Tumor_G3
14892	DUOXA2	7.829371737	Tumor_G3
14893	NDUFA1	8.351299568	Tumor_G3
14894	NDUFA2	7.127425304	Tumor_G3
14895	NDUFA8	7.416051745	Tumor_G3
14896	NDUFB1	6.633077728	Tumor_G3
14897	NDUFB10	7.82123759	Tumor_G3
14898	NDUFB2	6.662433575	Tumor_G3
14899	NDUFB3	6.477591099	Tumor_G3
14900	NDUFB7	9.247738425	Tumor_G3
14901	CYC1	7.003684789	Tumor_G3
14902	ETFB	5.280986558	Tumor_G3
14903	UQCR10	7.87475561	Tumor_G3
14904	UQCRQ	7.880870012	Tumor_G3
14905	UQCC3	4.801323167	Tumor_G3
14906	COX15	4.715297021	Tumor_G3
14907	COX5A	7.186008738	Tumor_G3
14908	COX6B1	9.052032298	Tumor_G3
14909	COX6C	7.181963395	Tumor_G3
14910	COX7A2	7.29561213	Tumor_G3
14911	COX7B	6.42325297	Tumor_G3
14912	COX8A	9.482428551	Tumor_G3
14913	SCO1	2.932203328	Tumor_G3
14914	SURF1	6.454162573	Tumor_G3
14915	ATP6V1E1	6.702865924	Tumor_G3
14916	DLAT	4.70564037	Tumor_G3
14917	PDC	0.166589472	Tumor_G3
14918	SUCLG2	5.98062425	Tumor_G3

14919	SLC25A5	7.677914729	Tumor_G3
14920	UCP2	6.690845799	Tumor_G3
14921	SOD2	4.357881117	Tumor_G3
14922	NOX4	2.199670664	Tumor_G3
14923	DUOX1	8.142460436	Tumor_G3
14924	DUOXA1	7.807679744	Tumor_G3
14925	DUOX2	8.258428572	Tumor_G3
14926	DUOXA2	6.063581588	Tumor_G3
14927	NDUFA1	9.109750437	Tumor_G3
14928	NDUFA2	7.939575616	Tumor_G3
14929	NDUFA8	7.785890655	Tumor_G3
14930	NDUFB1	6.883895995	Tumor_G3
14931	NDUFB10	8.358513386	Tumor_G3
14932	NDUFB2	6.577183373	Tumor_G3
14933	NDUFB3	6.491540105	Tumor_G3
14934	NDUFB7	9.87403885	Tumor_G3
14935	CYC1	7.314179341	Tumor_G3
14936	ETFB	5.500446233	Tumor_G3
14937	UQCR10	7.59727992	Tumor_G3
14938	UQCRQ	8.222400235	Tumor_G3
14939	UQCC3	5.612366086	Tumor_G3
14940	COX15	5.025665701	Tumor_G3
14941	COX5A	7.245841154	Tumor_G3
14942	COX6B1	9.691223591	Tumor_G3
14943	COX6C	7.570908263	Tumor_G3
14944	COX7A2	7.348228302	Tumor_G3
14945	COX7B	6.758933104	Tumor_G3
14946	COX8A	10.19237944	Tumor_G3
14947	SCO1	2.718943078	Tumor_G3
14948	SURF1	7.109463814	Tumor_G3
14949	ATP6V1E1	6.099995676	Tumor_G3
14950	DLAT	4.571572648	Tumor_G3
14951	PDC	0.2527517	Tumor_G3
14952	SUCLG2	6.107243608	Tumor_G3
14953	SLC25A5	7.352684979	Tumor_G3
14954	UCP2	7.391611148	Tumor_G3
14955	SOD2	3.628578689	Tumor_G3
14956	NOX4	1.913587056	Tumor_G3
14957	DUOX1	7.979151612	Tumor_G3
14958	DUOXA1	7.847688768	Tumor_G3

14959	DUOX2	9.287660269	Tumor_G3
14960	DUOXA2	7.290383828	Tumor_G3
14961	NDUFA1	8.673842654	Tumor_G3
14962	NDUFA2	6.976432508	Tumor_G3
14963	NDUFA8	7.616375541	Tumor_G3
14964	NDUFB1	6.141769836	Tumor_G3
14965	NDUFB10	8.046567913	Tumor_G3
14966	NDUFB2	7.534195144	Tumor_G3
14967	NDUFB3	6.525097009	Tumor_G3
14968	NDUFB7	9.078077194	Tumor_G3
14969	CYC1	7.586235301	Tumor_G3
14970	ETFB	5.490238592	Tumor_G3
14971	UQCR10	8.726960657	Tumor_G3
14972	UQCRQ	7.599725813	Tumor_G3
14973	UQCC3	5.082050498	Tumor_G3
14974	COX15	5.363930871	Tumor_G3
14975	COX5A	7.584118255	Tumor_G3
14976	COX6B1	9.210503117	Tumor_G3
14977	COX6C	7.021752153	Tumor_G3
14978	COX7A2	7.014497586	Tumor_G3
14979	COX7B	6.701139455	Tumor_G3
14980	COX8A	10.21902144	Tumor_G3
14981	SCO1	4.448010557	Tumor_G3
14982	SURF1	7.073714359	Tumor_G3
14983	ATP6V1E1	6.413269345	Tumor_G3
14984	DLAT	5.483879823	Tumor_G3
14985	PDC	0.301506282	Tumor_G3
14986	SUCLG2	6.331116704	Tumor_G3
14987	SLC25A5	8.781373768	Tumor_G3
14988	UCP2	8.189787869	Tumor_G3
14989	SOD2	4.936849579	Tumor_G3
14990	NOX4	2.487855636	Tumor_G3
14991	DUOX1	7.683077589	Tumor_G3
14992	DUOXA1	6.956541289	Tumor_G3
14993	DUOX2	8.936449041	Tumor_G3
14994	DUOXA2	8.219683294	Tumor_G3
14995	NDUFA1	8.171736944	Tumor_G3
14996	NDUFA2	7.733685131	Tumor_G3
14997	NDUFA8	7.350335746	Tumor_G3
14998	NDUFB1	6.427442408	Tumor_G3

14999	NDUFB10	8.672381966	Tumor_G3
15000	NDUFB2	7.306069359	Tumor_G3
15001	NDUFB3	6.237003658	Tumor_G3
15002	NDUFB7	9.860500896	Tumor_G3
15003	CYC1	7.178851266	Tumor_G3
15004	ETFB	6.23498888	Tumor_G3
15005	UQCR10	8.223549061	Tumor_G3
15006	UQCRQ	8.143615521	Tumor_G3
15007	UQCC3	5.434690103	Tumor_G3
15008	COX15	3.928641624	Tumor_G3
15009	COX5A	7.420486428	Tumor_G3
15010	COX6B1	9.688920553	Tumor_G3
15011	COX6C	7.016470616	Tumor_G3
15012	COX7A2	7.372992209	Tumor_G3
15013	COX7B	6.207315315	Tumor_G3
15014	COX8A	10.14017172	Tumor_G3
15015	SCO1	2.469032548	Tumor_G3
15016	SURF1	6.98895207	Tumor_G3
15017	ATP6V1E1	7.118149422	Tumor_G3
15018	DLAT	3.695149375	Tumor_G3
15019	PDC	0	Tumor_G3
15020	SUCLG2	5.247298002	Tumor_G3
15021	SLC25A5	7.736086028	Tumor_G3
15022	UCP2	7.910633539	Tumor_G3
15023	SOD2	4.524643687	Tumor_G3
15024	NOX4	3.669759423	Tumor_G3
15025	DUOX1	5.246047981	Tumor_G3
15026	DUOXA1	6.167587885	Tumor_G3
15027	DUOX2	4.401501387	Tumor_G3
15028	DUOXA2	3.817401168	Tumor_G3
15029	NDUFA1	9.084860244	Tumor_G3
15030	NDUFA2	8.090018777	Tumor_G3
15031	NDUFA8	8.299402167	Tumor_G3
15032	NDUFB1	7.114323481	Tumor_G3
15033	NDUFB10	8.559470805	Tumor_G3
15034	NDUFB2	8.203673323	Tumor_G3
15035	NDUFB3	7.312274583	Tumor_G3
15036	NDUFB7	9.796148672	Tumor_G3
15037	CYC1	7.844704934	Tumor_G3
15038	ETFB	6.49082716	Tumor_G3

15039	UQCR10	8.056324358	Tumor_G3
15040	UQCRQ	8.687167745	Tumor_G3
15041	UQCC3	5.487441087	Tumor_G3
15042	COX15	4.982718347	Tumor_G3
15043	COX5A	7.94980603	Tumor_G3
15044	COX6B1	10.14277365	Tumor_G3
15045	COX6C	7.330715578	Tumor_G3
15046	COX7A2	7.941682221	Tumor_G3
15047	COX7B	7.190800138	Tumor_G3
15048	COX8A	10.77183204	Tumor_G3
15049	SCO1	3.72297359	Tumor_G3
15050	SURF1	7.579832719	Tumor_G3
15051	ATP6V1E1	6.598173107	Tumor_G3
15052	DLAT	4.645310106	Tumor_G3
15053	PDC	0	Tumor_G3
15054	SUCLG2	6.245285779	Tumor_G3
15055	SLC25A5	8.576510926	Tumor_G3
15056	UCP2	8.721954373	Tumor_G3
15057	SOD2	3.852836747	Tumor_G3
15058	NOX4	0.573289314	Tumor_G3
15059	DUOX1	7.633718167	Tumor_G3
15060	DUOXA1	7.35779195	Tumor_G3
15061	DUOX2	9.539053441	Tumor_G3
15062	DUOXA2	8.32889005	Tumor_G3
15063	NDUFA1	8.751391559	Tumor_G3
15064	NDUFA2	7.201351601	Tumor_G3
15065	NDUFA8	7.756907765	Tumor_G3
15066	NDUFB1	6.351369495	Tumor_G3
15067	NDUFB10	8.240498378	Tumor_G3
15068	NDUFB2	7.34665966	Tumor_G3
15069	NDUFB3	6.639132541	Tumor_G3
15070	NDUFB7	9.341850002	Tumor_G3
15071	CYC1	7.651747253	Tumor_G3
15072	ETFB	5.716653634	Tumor_G3
15073	UQCR10	8.654667872	Tumor_G3
15074	UQCRQ	7.675497822	Tumor_G3
15075	UQCC3	5.091183681	Tumor_G3
15076	COX15	5.008478005	Tumor_G3
15077	COX5A	7.69628242	Tumor_G3
15078	COX6B1	9.445084235	Tumor_G3

15079	COX6C	6.979089907	Tumor_G3
15080	COX7A2	7.023797143	Tumor_G3
15081	COX7B	6.784363336	Tumor_G3
15082	COX8A	10.42101477	Tumor_G3
15083	SCO1	4.147365676	Tumor_G3
15084	SURF1	6.912711552	Tumor_G3
15085	ATP6V1E1	6.393648056	Tumor_G3
15086	DLAT	4.778110256	Tumor_G3
15087	PDC	0	Tumor_G3
15088	SUCLG2	6.006434369	Tumor_G3
15089	SLC25A5	8.516966327	Tumor_G3
15090	UCP2	8.002265512	Tumor_G3
15091	SOD2	4.641643246	Tumor_G3
15092	NOX4	2.105277069	Tumor_G3
15093	DUOX1	7.996714848	Tumor_G3
15094	DUOXA1	7.326078711	Tumor_G3
15095	DUOX2	8.821722391	Tumor_G3
15096	DUOXA2	7.771860945	Tumor_G3
15097	NDUFA1	8.275294163	Tumor_G3
15098	NDUFA2	7.156214176	Tumor_G3
15099	NDUFA8	7.327948949	Tumor_G3
15100	NDUFB1	5.989688366	Tumor_G3
15101	NDUFB10	7.743187546	Tumor_G3
15102	NDUFB2	6.967685054	Tumor_G3
15103	NDUFB3	6.6875802	Tumor_G3
15104	NDUFB7	8.992895914	Tumor_G3
15105	CYC1	6.8472303	Tumor_G3
15106	ETFB	5.127794837	Tumor_G3
15107	UQCR10	8.005779157	Tumor_G3
15108	UQCRQ	7.760125547	Tumor_G3
15109	UQCC3	4.489122692	Tumor_G3
15110	COX15	4.314765477	Tumor_G3
15111	COX5A	7.48667973	Tumor_G3
15112	COX6B1	9.153303534	Tumor_G3
15113	COX6C	6.809643277	Tumor_G3
15114	COX7A2	7.235769398	Tumor_G3
15115	COX7B	6.348155234	Tumor_G3
15116	COX8A	9.763456844	Tumor_G3
15117	SCO1	3.00478831	Tumor_G3
15118	SURF1	6.455334866	Tumor_G3

15119	ATP6V1E1	7.466169229	Tumor_G3
15120	DLAT	4.765622861	Tumor_G3
15121	PDC	0	Tumor_G3
15122	SUCLG2	5.891625795	Tumor_G3
15123	SLC25A5	8.835984835	Tumor_G3
15124	UCP2	7.67958319	Tumor_G3
15125	SOD2	5.021512877	Tumor_G3
15126	NOX4	3.414625217	Tumor_G3
15127	DUOX1	4.685477182	Tumor_G3
15128	DUOXA1	5.225907695	Tumor_G3
15129	DUOX2	3.691174063	Tumor_G3
15130	DUOXA2	1.942202699	Tumor_G3
15131	NDUFA1	9.290752509	Tumor_G3
15132	NDUFA2	7.998839716	Tumor_G3
15133	NDUFA8	7.82178731	Tumor_G3
15134	NDUFB1	7.276680019	Tumor_G3
15135	NDUFB10	9.001687609	Tumor_G3
15136	NDUFB2	7.890914037	Tumor_G3
15137	NDUFB3	6.856543553	Tumor_G3
15138	NDUFB7	10.70501325	Tumor_G3
15139	CYC1	6.836035054	Tumor_G3
15140	ETFB	6.731479954	Tumor_G3
15141	UQCR10	8.723495147	Tumor_G3
15142	UQCRQ	8.238059563	Tumor_G3
15143	UQCC3	6.11903901	Tumor_G3
15144	COX15	4.790653548	Tumor_G3
15145	COX5A	7.670467093	Tumor_G3
15146	COX6B1	10.1623335	Tumor_G3
15147	COX6C	7.591588901	Tumor_G3
15148	COX7A2	6.893440324	Tumor_G3
15149	COX7B	7.120055066	Tumor_G3
15150	COX8A	10.66820717	Tumor_G3
15151	SCO1	2.790669985	Tumor_G3
15152	SURF1	7.543630444	Tumor_G3
15153	ATP6V1E1	7.16249558	Tumor_G3
15154	DLAT	3.947977582	Tumor_G3
15155	PDC	0	Tumor_G3
15156	SUCLG2	5.257016512	Tumor_G3
15157	SLC25A5	7.095065521	Tumor_G3
15158	UCP2	9.07540934	Tumor_G3

15159	SOD2	3.583012061	Tumor_G3
15160	NOX4	1.687411383	Tumor_G3
15161	DUOX1	7.29531345	Tumor_G3
15162	DUOXA1	7.54039191	Tumor_G3
15163	DUOX2	6.969638718	Tumor_G3
15164	DUOXA2	5.998928672	Tumor_G3
15165	NDUFA1	9.116131283	Tumor_G3
15166	NDUFA2	7.586161207	Tumor_G3
15167	NDUFA8	7.794957183	Tumor_G3
15168	NDUFB1	6.820236886	Tumor_G3
15169	NDUFB10	8.456964026	Tumor_G3
15170	NDUFB2	7.315100034	Tumor_G3
15171	NDUFB3	6.994627826	Tumor_G3
15172	NDUFB7	9.965818323	Tumor_G3
15173	CYC1	7.254260087	Tumor_G3
15174	ETFB	6.006335732	Tumor_G3
15175	UQCR10	8.586821258	Tumor_G3
15176	UQCRQ	8.228989953	Tumor_G3
15177	UQCC3	5.42474636	Tumor_G3
15178	COX15	4.534691924	Tumor_G3
15179	COX5A	7.728628312	Tumor_G3
15180	COX6B1	9.712635688	Tumor_G3
15181	COX6C	7.447644858	Tumor_G3
15182	COX7A2	7.503669681	Tumor_G3
15183	COX7B	6.706738476	Tumor_G3
15184	COX8A	10.18805212	Tumor_G3
15185	SCO1	2.95486093	Tumor_G3
15186	SURF1	7.097008479	Tumor_G3
15187	ATP6V1E1	7.170607753	Tumor_G3
15188	DLAT	4.229722588	Tumor_G3
15189	PDC	0.063158639	Tumor_G3
15190	SUCLG2	5.699385771	Tumor_G3
15191	SLC25A5	7.819565206	Tumor_G3
15192	UCP2	9.373552694	Tumor_G3
15193	SOD2	3.884044636	Tumor_G3
15194	NOX4	1.822130902	Tumor_G3
15195	DUOX1	6.462719161	Tumor_G3
15196	DUOXA1	6.435861417	Tumor_G3
15197	DUOX2	7.073848606	Tumor_G3
15198	DUOXA2	6.668472978	Tumor_G3

15199	NDUFA1	8.844367767	Tumor_G3
15200	NDUFA2	7.472257506	Tumor_G3
15201	NDUFA8	7.696910605	Tumor_G3
15202	NDUFB1	6.73001465	Tumor_G3
15203	NDUFB10	7.922224786	Tumor_G3
15204	NDUFB2	7.476621934	Tumor_G3
15205	NDUFB3	6.894560033	Tumor_G3
15206	NDUFB7	9.331333559	Tumor_G3
15207	CYC1	7.213413877	Tumor_G3
15208	ETFB	5.468338754	Tumor_G3
15209	UQCR10	8.237255373	Tumor_G3
15210	UQCRQ	8.010087456	Tumor_G3
15211	UQCC3	4.818535048	Tumor_G3
15212	COX15	4.708460531	Tumor_G3
15213	COX5A	7.72354247	Tumor_G3
15214	COX6B1	9.221669844	Tumor_G3
15215	COX6C	7.158068653	Tumor_G3
15216	COX7A2	7.507903272	Tumor_G3
15217	COX7B	6.986951215	Tumor_G3
15218	COX8A	10.32833905	Tumor_G3
15219	SCO1	3.198272308	Tumor_G3
15220	SURF1	6.877296644	Tumor_G3
15221	ATP6V1E1	6.80371374	Tumor_G3
15222	DLAT	4.958734209	Tumor_G3
15223	PDC	0.241222903	Tumor_G3
15224	SUCLG2	6.087079589	Tumor_G3
15225	SLC25A5	8.158687984	Tumor_G3
15226	UCP2	7.824705183	Tumor_G3
15227	SOD2	4.062557102	Tumor_G3
15228	NOX4	0.863539007	Tumor_G3
15229	DUOX1	6.845936241	Tumor_G3
15230	DUOXA1	6.732412552	Tumor_G3
15231	DUOX2	7.732387349	Tumor_G3
15232	DUOXA2	6.791075243	Tumor_G3
15233	NDUFA1	8.718440487	Tumor_G3
15234	NDUFA2	7.565930895	Tumor_G3
15235	NDUFA8	7.452988876	Tumor_G3
15236	NDUFB1	6.635213791	Tumor_G3
15237	NDUFB10	7.737219813	Tumor_G3
15238	NDUFB2	7.38120868	Tumor_G3

15239	NDUFB3	6.694716938	Tumor_G3
15240	NDUFB7	9.417400258	Tumor_G3
15241	CYC1	7.262347515	Tumor_G3
15242	ETFB	5.138573966	Tumor_G3
15243	UQCR10	8.390398091	Tumor_G3
15244	UQCRQ	8.016404679	Tumor_G3
15245	UQCC3	4.893193349	Tumor_G3
15246	COX15	4.490588759	Tumor_G3
15247	COX5A	7.471094533	Tumor_G3
15248	COX6B1	9.439309281	Tumor_G3
15249	COX6C	7.415555664	Tumor_G3
15250	COX7A2	7.461749288	Tumor_G3
15251	COX7B	6.854538535	Tumor_G3
15252	COX8A	10.16888819	Tumor_G3
15253	SCO1	3.242087955	Tumor_G3
15254	SURF1	6.782718209	Tumor_G3
15255	ATP6V1E1	6.795818966	Tumor_G3
15256	DLAT	4.985529175	Tumor_G3
15257	PDC	0.24955754	Tumor_G3
15258	SUCLG2	6.145727691	Tumor_G3
15259	SLC25A5	7.891152096	Tumor_G3
15260	UCP2	7.151715377	Tumor_G3
15261	SOD2	4.188088071	Tumor_G3
15262	NOX4	1.925952895	Tumor_G3
15263	DUOX1	7.800192212	Tumor_G3
15264	DUOXA1	7.427000115	Tumor_G3
15265	DUOX2	8.396670541	Tumor_G3
15266	DUOXA2	6.79520995	Tumor_G3
15267	NDUFA1	8.755806838	Tumor_G3
15268	NDUFA2	7.555403569	Tumor_G3
15269	NDUFA8	7.694797709	Tumor_G3
15270	NDUFB1	6.416465842	Tumor_G3
15271	NDUFB10	8.072411913	Tumor_G3
15272	NDUFB2	7.344683254	Tumor_G3
15273	NDUFB3	6.734533105	Tumor_G3
15274	NDUFB7	9.31057958	Tumor_G3
15275	CYC1	7.559393295	Tumor_G3
15276	ETFB	5.827206607	Tumor_G3
15277	UQCR10	7.775510503	Tumor_G3
15278	UQCRQ	7.956035328	Tumor_G3

15279	UQCC3	5.31053111	Tumor_G3
15280	COX15	5.054181873	Tumor_G3
15281	COX5A	7.293220078	Tumor_G3
15282	COX6B1	9.329879493	Tumor_G3
15283	COX6C	7.056847949	Tumor_G3
15284	COX7A2	7.406322428	Tumor_G3
15285	COX7B	6.601917442	Tumor_G3
15286	COX8A	10.29207784	Tumor_G3
15287	SCO1	3.149179822	Tumor_G3
15288	SURF1	6.865342966	Tumor_G3
15289	ATP6V1E1	6.77475769	Tumor_G3
15290	DLAT	5.027538627	Tumor_G3
15291	PDC	0.274635247	Tumor_G3
15292	SUCLG2	6.344974338	Tumor_G3
15293	SLC25A5	8.051076498	Tumor_G3
15294	UCP2	8.729180892	Tumor_G3
15295	SOD2	4.074765649	Tumor_G3
15296	NOX4	2.38376152	Tumor_G3
15297	DUOX1	7.750375864	Tumor_G3
15298	DUOXA1	7.456319355	Tumor_G3
15299	DUOX2	9.101188314	Tumor_G3
15300	DUOXA2	6.869314416	Tumor_G3
15301	NDUFA1	8.782779066	Tumor_G3
15302	NDUFA2	7.761011854	Tumor_G3
15303	NDUFA8	7.662093464	Tumor_G3
15304	NDUFB1	6.776880821	Tumor_G3
15305	NDUFB10	8.131248154	Tumor_G3
15306	NDUFB2	7.032548514	Tumor_G3
15307	NDUFB3	6.709313848	Tumor_G3
15308	NDUFB7	9.600166852	Tumor_G3
15309	CYC1	7.417845569	Tumor_G3
15310	ETFB	5.717861039	Tumor_G3
15311	UQCR10	8.334700981	Tumor_G3
15312	UQCRQ	8.34877539	Tumor_G3
15313	UQCC3	5.205423903	Tumor_G3
15314	COX15	4.530661836	Tumor_G3
15315	COX5A	7.849284821	Tumor_G3
15316	COX6B1	9.461805995	Tumor_G3
15317	COX6C	7.465367977	Tumor_G3
15318	COX7A2	7.439172392	Tumor_G3

15319	COX7B	6.844721396	Tumor_G3
15320	COX8A	10.42148469	Tumor_G3
15321	SCO1	3.300990319	Tumor_G3
15322	SURF1	7.20090384	Tumor_G3
15323	ATP6V1E1	6.825211799	Tumor_G3
15324	DLAT	4.446251394	Tumor_G3
15325	PDC	0.118193238	Tumor_G3
15326	SUCLG2	6.077997088	Tumor_G3
15327	SLC25A5	8.001088102	Tumor_G3
15328	UCP2	7.669009942	Tumor_G3
15329	SOD2	4.133142565	Tumor_G3
15330	NOX4	0.669672379	Tumor_G3
15331	DUOX1	8.58247254	Tumor_G3
15332	DUOXA1	8.51700536	Tumor_G3
15333	DUOX2	9.095376516	Tumor_G3
15334	DUOXA2	7.921852981	Tumor_G3
15335	NDUFA1	8.385949944	Tumor_G3
15336	NDUFA2	7.566751828	Tumor_G3
15337	NDUFA8	7.584965024	Tumor_G3
15338	NDUFB1	6.335469821	Tumor_G3
15339	NDUFB10	8.395434536	Tumor_G3
15340	NDUFB2	7.28906916	Tumor_G3
15341	NDUFB3	6.616218909	Tumor_G3
15342	NDUFB7	9.742728524	Tumor_G3
15343	CYC1	7.419531091	Tumor_G3
15344	ETFB	6.019899114	Tumor_G3
15345	UQCR10	8.389364105	Tumor_G3
15346	UQCRQ	8.042043661	Tumor_G3
15347	UQCC3	5.558967914	Tumor_G3
15348	COX15	4.931501931	Tumor_G3
15349	COX5A	7.582042475	Tumor_G3
15350	COX6B1	9.394967743	Tumor_G3
15351	COX6C	7.169626591	Tumor_G3
15352	COX7A2	7.358872184	Tumor_G3
15353	COX7B	6.442539117	Tumor_G3
15354	COX8A	10.20859033	Tumor_G3
15355	SCO1	3.158566925	Tumor_G3
15356	SURF1	7.194895695	Tumor_G3
15357	ATP6V1E1	6.965608426	Tumor_G3
15358	DLAT	4.606329911	Tumor_G3

15359	PDC	0.067854306	Tumor_G3
15360	SUCLG2	5.919224717	Tumor_G3
15361	SLC25A5	7.616237411	Tumor_G3
15362	UCP2	9.621737426	Tumor_G3
15363	SOD2	5.212855005	Tumor_G3
15364	NOX4	1.011109189	Tumor_G3
15365	DUOX1	6.258361785	Tumor_G3
15366	DUOXA1	6.908962563	Tumor_G3
15367	DUOX2	7.159064958	Tumor_G3
15368	DUOXA2	6.872995161	Tumor_G3
15369	NDUFA1	8.464158725	Tumor_G3
15370	NDUFA2	7.192336757	Tumor_G3
15371	NDUFA8	7.164471454	Tumor_G3
15372	NDUFB1	6.483287585	Tumor_G3
15373	NDUFB10	8.110439846	Tumor_G3
15374	NDUFB2	6.758038379	Tumor_G3
15375	NDUFB3	7.140045055	Tumor_G3
15376	NDUFB7	9.540644787	Tumor_G3
15377	CYC1	7.185819166	Tumor_G3
15378	ETFB	5.786402969	Tumor_G3
15379	UQCR10	7.160178082	Tumor_G3
15380	UQCRQ	7.885786714	Tumor_G3
15381	UQCC3	5.012619285	Tumor_G3
15382	COX15	4.693474908	Tumor_G3
15383	COX5A	7.318760371	Tumor_G3
15384	COX6B1	9.058611344	Tumor_G3
15385	COX6C	6.915750005	Tumor_G3
15386	COX7A2	7.180316255	Tumor_G3
15387	COX7B	6.693227988	Tumor_G3
15388	COX8A	9.934051896	Tumor_G3
15389	SCO1	3.654593896	Tumor_G3
15390	SURF1	6.015752569	Tumor_G3
15391	ATP6V1E1	6.382049426	Tumor_G3
15392	DLAT	4.988749209	Tumor_G3
15393	PDC	0.059435341	Tumor_G3
15394	SUCLG2	6.455647876	Tumor_G3
15395	SLC25A5	8.252799036	Tumor_G3
15396	UCP2	7.98458886	Tumor_G3
15397	SOD2	4.624754187	Tumor_G3
15398	NOX4	2.381995322	Tumor_G3

15399	DUOX1	7.936344627	Tumor_G3
15400	DUOXA1	7.71502319	Tumor_G3
15401	DUOX2	9.507564109	Tumor_G3
15402	DUOXA2	7.095282598	Tumor_G3
15403	NDUFA1	8.647676438	Tumor_G3
15404	NDUFA2	7.370006605	Tumor_G3
15405	NDUFA8	7.544386349	Tumor_G3
15406	NDUFB1	6.322262538	Tumor_G3
15407	NDUFB10	8.320488633	Tumor_G3
15408	NDUFB2	7.285380811	Tumor_G3
15409	NDUFB3	6.68837607	Tumor_G3
15410	NDUFB7	9.40249499	Tumor_G3
15411	CYC1	7.020811073	Tumor_G3
15412	ETFB	5.836527641	Tumor_G3
15413	UQCR10	8.152454131	Tumor_G3
15414	UQCRQ	7.856311866	Tumor_G3
15415	UQCC3	5.157555314	Tumor_G3
15416	COX15	4.40962182	Tumor_G3
15417	COX5A	7.784018912	Tumor_G3
15418	COX6B1	9.454025576	Tumor_G3
15419	COX6C	7.132031291	Tumor_G3
15420	COX7A2	7.347988369	Tumor_G3
15421	COX7B	6.550865301	Tumor_G3
15422	COX8A	9.949745366	Tumor_G3
15423	SCO1	2.890430852	Tumor_G3
15424	SURF1	6.667955861	Tumor_G3
15425	ATP6V1E1	7.263921979	Tumor_G3
15426	DLAT	4.152529414	Tumor_G3
15427	PDC	0	Tumor_G3
15428	SUCLG2	5.537046578	Tumor_G3
15429	SLC25A5	8.185923248	Tumor_G3
15430	UCP2	8.050322468	Tumor_G3
15431	SOD2	4.851054592	Tumor_G3
15432	NOX4	2.570273369	Tumor_G3
15433	DUOX1	5.299251683	Tumor_G3
15434	DUOXA1	5.884612969	Tumor_G3
15435	DUOX2	4.864861376	Tumor_G3
15436	DUOXA2	4.452218803	Tumor_G3
15437	NDUFA1	8.759498758	Tumor_G3
15438	NDUFA2	7.364861449	Tumor_G3

15439	NDUFA8	7.626093693	Tumor_G3
15440	NDUFB1	6.519951089	Tumor_G3
15441	NDUFB10	8.013908472	Tumor_G3
15442	NDUFB2	6.048192145	Tumor_G3
15443	NDUFB3	7.077339837	Tumor_G3
15444	NDUFB7	9.492343588	Tumor_G3
15445	CYC1	7.200664145	Tumor_G3
15446	ETFB	5.523234321	Tumor_G3
15447	UQCR10	8.99876925	Tumor_G3
15448	UQCRQ	7.98759772	Tumor_G3
15449	UQCC3	5.178026195	Tumor_G3
15450	COX15	5.147650085	Tumor_G3
15451	COX5A	7.539595306	Tumor_G3
15452	COX6B1	9.158458586	Tumor_G3
15453	COX6C	8.022355361	Tumor_G3
15454	COX7A2	6.845843527	Tumor_G3
15455	COX7B	6.593155761	Tumor_G3
15456	COX8A	9.700645413	Tumor_G3
15457	SCO1	3.643427493	Tumor_G3
15458	SURF1	6.456648104	Tumor_G3
15459	ATP6V1E1	7.083774794	Tumor_G3
15460	DLAT	4.870333906	Tumor_G3
15461	PDC	0.067309287	Tumor_G3
15462	SUCLG2	5.812954168	Tumor_G3
15463	SLC25A5	7.941881136	Tumor_G3
15464	UCP2	6.021732103	Tumor_G3
15465	SOD2	4.683399969	Tumor_G3
15466	NOX4	2.262328297	Tumor_G3
15467	DUOX1	1.407442846	Tumor_G3
15468	DUOXA1	1.859795018	Tumor_G3
15469	DUOX2	0.810945686	Tumor_G3
15470	DUOXA2	0.170180073	Tumor_G3
15471	NDUFA1	8.345817795	Tumor_G3
15472	NDUFA2	7.833346479	Tumor_G3
15473	NDUFA8	7.454856341	Tumor_G3
15474	NDUFB1	5.963886866	Tumor_G3
15475	NDUFB10	8.594475728	Tumor_G3
15476	NDUFB2	7.210558125	Tumor_G3
15477	NDUFB3	6.305887086	Tumor_G3
15478	NDUFB7	9.933333219	Tumor_G3

15479	CYC1	7.328576485	Tumor_G3
15480	ETFB	6.453378933	Tumor_G3
15481	UQCR10	7.521642359	Tumor_G3
15482	UQCRQ	8.053907192	Tumor_G3
15483	UQCC3	5.878972234	Tumor_G3
15484	COX15	4.167720991	Tumor_G3
15485	COX5A	7.387642679	Tumor_G3
15486	COX6B1	9.669302353	Tumor_G3
15487	COX6C	6.819237931	Tumor_G3
15488	COX7A2	7.289382409	Tumor_G3
15489	COX7B	6.208516763	Tumor_G3
15490	COX8A	10.66000951	Tumor_G3
15491	SCO1	2.795707908	Tumor_G3
15492	SURF1	7.166039418	Tumor_G3
15493	ATP6V1E1	6.464877168	Tumor_G3
15494	DLAT	3.779093055	Tumor_G3
15495	PDC	0	Tumor_G3
15496	SUCLG2	5.280514952	Tumor_G3
15497	SLC25A5	7.815958976	Tumor_G3
15498	UCP2	8.828691785	Tumor_G3
15499	SOD2	4.271645682	Tumor_G3
15500	NOX4	2.867917252	Tumor_G3
15501	DUOX1	6.788263162	Tumor_G3
15502	DUOXA1	6.977501867	Tumor_G3
15503	DUOX2	7.287594618	Tumor_G3
15504	DUOXA2	5.773364105	Tumor_G3
15505	NDUFA1	8.618696035	Tumor_G3
15506	NDUFA2	7.373822269	Tumor_G3
15507	NDUFA8	7.817384819	Tumor_G3
15508	NDUFB1	6.332044577	Tumor_G3
15509	NDUFB10	8.289514005	Tumor_G3
15510	NDUFB2	7.324175167	Tumor_G3
15511	NDUFB3	6.837321619	Tumor_G3
15512	NDUFB7	9.537599327	Tumor_G3
15513	CYC1	7.342091193	Tumor_G3
15514	ETFB	5.742759865	Tumor_G3
15515	UQCR10	7.641098857	Tumor_G3
15516	UQCRQ	7.962131943	Tumor_G3
15517	UQCC3	4.835844195	Tumor_G3
15518	COX15	4.673623782	Tumor_G3

15519	COX5A	7.469578503	Tumor_G3
15520	COX6B1	9.201997667	Tumor_G3
15521	COX6C	6.948789278	Tumor_G3
15522	COX7A2	7.39372387	Tumor_G3
15523	COX7B	6.473230964	Tumor_G3
15524	COX8A	10.37918689	Tumor_G3
15525	SCO1	3.26636589	Tumor_G3
15526	SURF1	7.211230197	Tumor_G3
15527	ATP6V1E1	6.685235974	Tumor_G3
15528	DLAT	4.526105266	Tumor_G3
15529	PDC	0	Tumor_G3
15530	SUCLG2	5.608088325	Tumor_G3
15531	SLC25A5	8.332313355	Tumor_G3
15532	UCP2	7.297762356	Tumor_G3
15533	SOD2	4.318550595	Tumor_G3
15534	NOX4	1.18484604	Tumor_G3
15535	DUOX1	7.436687633	Tumor_G3
15536	DUOXA1	7.17954445	Tumor_G3
15537	DUOX2	8.856741463	Tumor_G3
15538	DUOXA2	7.079421421	Tumor_G3
15539	NDUFA1	8.628869386	Tumor_G3
15540	NDUFA2	7.036265981	Tumor_G3
15541	NDUFA8	7.545514993	Tumor_G3
15542	NDUFB1	6.218984749	Tumor_G3
15543	NDUFB10	7.877363974	Tumor_G3
15544	NDUFB2	7.262502541	Tumor_G3
15545	NDUFB3	6.805498336	Tumor_G3
15546	NDUFB7	8.979489263	Tumor_G3
15547	CYC1	7.166715238	Tumor_G3
15548	ETFB	5.399437184	Tumor_G3
15549	UQCR10	8.399056349	Tumor_G3
15550	UQCRQ	7.769180909	Tumor_G3
15551	UQCC3	4.763446672	Tumor_G3
15552	COX15	5.081167921	Tumor_G3
15553	COX5A	7.534595762	Tumor_G3
15554	COX6B1	8.889081347	Tumor_G3
15555	COX6C	6.916958223	Tumor_G3
15556	COX7A2	7.22208763	Tumor_G3
15557	COX7B	6.694920838	Tumor_G3
15558	COX8A	9.957497045	Tumor_G3

15559	SCO1	4.218716947	Tumor_G3
15560	SURF1	6.722435152	Tumor_G3
15561	ATP6V1E1	6.524998175	Tumor_G3
15562	DLAT	5.411002951	Tumor_G3
15563	PDC	0.105259603	Tumor_G3
15564	SUCLG2	6.386031925	Tumor_G3
15565	SLC25A5	8.416267199	Tumor_G3
15566	UCP2	7.372423073	Tumor_G3
15567	SOD2	5.104948883	Tumor_G3
15568	NOX4	2.038745016	Tumor_G3
15569	DUOX1	7.909737787	Tumor_G3
15570	DUOXA1	6.803332773	Tumor_G3
15571	DUOX2	9.212502444	Tumor_G3
15572	DUOXA2	7.865886237	Tumor_G3
15573	NDUFA1	8.850670178	Tumor_G3
15574	NDUFA2	7.437137097	Tumor_G3
15575	NDUFA8	7.789010962	Tumor_G3
15576	NDUFB1	6.826218286	Tumor_G3
15577	NDUFB10	8.226729231	Tumor_G3
15578	NDUFB2	7.517098705	Tumor_G3
15579	NDUFB3	7.112203586	Tumor_G3
15580	NDUFB7	8.338789136	Tumor_G3
15581	CYC1	7.660128416	Tumor_G3
15582	ETFB	5.843146688	Tumor_G3
15583	UQCR10	8.606544941	Tumor_G3
15584	UQCRQ	8.023766183	Tumor_G3
15585	UQCC3	4.795206591	Tumor_G3
15586	COX15	5.602781365	Tumor_G3
15587	COX5A	8.030755785	Tumor_G3
15588	COX6B1	8.949478767	Tumor_G3
15589	COX6C	7.512663306	Tumor_G3
15590	COX7A2	7.640629193	Tumor_G3
15591	COX7B	7.154592337	Tumor_G3
15592	COX8A	10.38198349	Tumor_G3
15593	SCO1	4.115526643	Tumor_G3
15594	SURF1	7.506357115	Tumor_G3
15595	ATP6V1E1	6.987803605	Tumor_G3
15596	DLAT	5.698283218	Tumor_G3
15597	PDC	0.230450839	Tumor_G3
15598	SUCLG2	7.114317044	Tumor_G3

15599	SLC25A5	8.272838631	Tumor_G3
15600	UCP2	8.094378807	Tumor_G3
15601	SOD2	4.73788707	Tumor_G3
15602	NOX4	0.455154874	Tumor_G3
15603	DUOX1	7.377508184	Tumor_G3
15604	DUOXA1	6.972942977	Tumor_G3
15605	DUOX2	7.432790431	Tumor_G3
15606	DUOXA2	5.911813299	Tumor_G3
15607	NDUFA1	8.129831425	Tumor_G3
15608	NDUFA2	8.221235504	Tumor_G3
15609	NDUFA8	7.161544144	Tumor_G3
15610	NDUFB1	6.153200732	Tumor_G3
15611	NDUFB10	7.94998811	Tumor_G3
15612	NDUFB2	7.209572874	Tumor_G3
15613	NDUFB3	6.68231243	Tumor_G3
15614	NDUFB7	9.467435767	Tumor_G3
15615	CYC1	7.168276738	Tumor_G3
15616	ETFB	5.317666207	Tumor_G3
15617	UQCR10	7.898925686	Tumor_G3
15618	UQCRQ	8.44440865	Tumor_G3
15619	UQCC3	4.972137931	Tumor_G3
15620	COX15	4.194478606	Tumor_G3
15621	COX5A	7.529832633	Tumor_G3
15622	COX6B1	9.392214293	Tumor_G3
15623	COX6C	6.658896308	Tumor_G3
15624	COX7A2	7.579637067	Tumor_G3
15625	COX7B	6.327083508	Tumor_G3
15626	COX8A	9.716320664	Tumor_G3
15627	SCO1	2.160016666	Tumor_G3
15628	SURF1	6.927388133	Tumor_G3
15629	ATP6V1E1	7.485223333	Tumor_G3
15630	DLAT	4.046742338	Tumor_G3
15631	PDC	0	Tumor_G3
15632	SUCLG2	5.746078488	Tumor_G3
15633	SLC25A5	8.154289348	Tumor_G3
15634	UCP2	7.5701644	Tumor_G3
15635	SOD2	5.29633422	Tumor_G3
15636	NOX4	2.237393516	Tumor_G3
15637	DUOX1	3.337413206	Tumor_G3
15638	DUOXA1	4.670308791	Tumor_G3

15639	DUOX2	1.980080948	Tumor_G3
15640	DUOXA2	1.434993123	Tumor_G3
15641	NDUFA1	8.621761378	Tumor_G3
15642	NDUFA2	7.31947368	Tumor_G3
15643	NDUFA8	7.404244933	Tumor_G3
15644	NDUFB1	6.588710378	Tumor_G3
15645	NDUFB10	7.871418024	Tumor_G3
15646	NDUFB2	6.79469212	Tumor_G3
15647	NDUFB3	6.380664923	Tumor_G3
15648	NDUFB7	9.229625957	Tumor_G3
15649	CYC1	6.933229004	Tumor_G3
15650	ETFB	5.251989639	Tumor_G3
15651	UQCR10	7.999679894	Tumor_G3
15652	UQCRQ	7.972367484	Tumor_G3
15653	UQCC3	4.754844335	Tumor_G3
15654	COX15	4.479612811	Tumor_G3
15655	COX5A	7.266018433	Tumor_G3
15656	COX6B1	9.247571881	Tumor_G3
15657	COX6C	7.086246506	Tumor_G3
15658	COX7A2	7.092934025	Tumor_G3
15659	COX7B	6.489059097	Tumor_G3
15660	COX8A	9.801940332	Tumor_G3
15661	SCO1	2.811494031	Tumor_G3
15662	SURF1	6.872110681	Tumor_G3
15663	ATP6V1E1	6.692018413	Tumor_G3
15664	DLAT	4.282350194	Tumor_G3
15665	PDC	0.314236674	Tumor_G3
15666	SUCLG2	5.600634271	Tumor_G3
15667	SLC25A5	7.23563328	Tumor_G3
15668	UCP2	7.770395198	Tumor_G3
15669	SOD2	3.931960241	Tumor_G3
15670	NOX4	1.390344509	Tumor_G3
15671	DUOX1	7.277884011	Tumor_G3
15672	DUOXA1	7.262958426	Tumor_G3
15673	DUOX2	8.044900267	Tumor_G3
15674	DUOXA2	5.764248854	Tumor_G3
15675	NDUFA1	9.092396285	Tumor_G3
15676	NDUFA2	8.101563077	Tumor_G3
15677	NDUFA8	7.849291578	Tumor_G3
15678	NDUFB1	7.569829	Tumor_G3

15679	NDUFB10	9.320614026	Tumor_G3
15680	NDUFB2	8.078853345	Tumor_G3
15681	NDUFB3	6.360984746	Tumor_G3
15682	NDUFB7	10.89941546	Tumor_G3
15683	CYC1	7.085111868	Tumor_G3
15684	ETFB	7.374976537	Tumor_G3
15685	UQCR10	8.84819408	Tumor_G3
15686	UQCRQ	9.029963232	Tumor_G3
15687	UQCC3	6.65466467	Tumor_G3
15688	COX15	4.247133936	Tumor_G3
15689	COX5A	7.733035085	Tumor_G3
15690	COX6B1	10.45539415	Tumor_G3
15691	COX6C	7.639946506	Tumor_G3
15692	COX7A2	7.651955577	Tumor_G3
15693	COX7B	6.764843109	Tumor_G3
15694	COX8A	10.24448195	Tumor_G3
15695	SCO1	2.580647128	Tumor_G3
15696	SURF1	7.608732401	Tumor_G3
15697	ATP6V1E1	6.797003488	Tumor_G3
15698	DLAT	3.476044288	Tumor_G3
15699	PDC	0	Tumor_G3
15700	SUCLG2	5.427961038	Tumor_G3
15701	SLC25A5	6.862065086	Tumor_G3
15702	UCP2	7.967512501	Tumor_G3
15703	SOD2	3.985570208	Tumor_G3
15704	NOX4	3.280429079	Tumor_G3
15705	DUOX1	5.443544466	Tumor_G3
15706	DUOXA1	6.687449642	Tumor_G3
15707	DUOX2	4.895039921	Tumor_G3
15708	DUOXA2	4.880067634	Tumor_G3
15709	NDUFA1	8.619878831	Tumor_G3
15710	NDUFA2	7.49350504	Tumor_G3
15711	NDUFA8	7.650292105	Tumor_G3
15712	NDUFB1	6.746193627	Tumor_G3
15713	NDUFB10	8.103465459	Tumor_G3
15714	NDUFB2	7.13508756	Tumor_G3
15715	NDUFB3	6.496136476	Tumor_G3
15716	NDUFB7	9.331099993	Tumor_G3
15717	CYC1	7.696578669	Tumor_G3
15718	ETFB	5.784493458	Tumor_G3

15719	UQCR10	7.90015084	Tumor_G3
15720	UQCRQ	8.116059545	Tumor_G3
15721	UQCC3	5.69092777	Tumor_G3
15722	COX15	5.214826558	Tumor_G3
15723	COX5A	7.893536443	Tumor_G3
15724	COX6B1	9.283264617	Tumor_G3
15725	COX6C	7.195319089	Tumor_G3
15726	COX7A2	7.407542918	Tumor_G3
15727	COX7B	6.480799024	Tumor_G3
15728	COX8A	10.7881778	Tumor_G3
15729	SCO1	3.887605482	Tumor_G3
15730	SURF1	7.036493033	Tumor_G3
15731	ATP6V1E1	6.894539527	Tumor_G3
15732	DLAT	4.982192786	Tumor_G3
15733	PDC	0.173715378	Tumor_G3
15734	SUCLG2	6.567119011	Tumor_G3
15735	SLC25A5	8.468512332	Tumor_G3
15736	UCP2	7.303883091	Tumor_G3
15737	SOD2	3.817741809	Tumor_G3
15738	NOX4	1.555500098	Tumor_G3
15739	DUOX1	8.278584759	Tumor_G3
15740	DUOXA1	7.843582516	Tumor_G3
15741	DUOX2	8.80658454	Tumor_G3
15742	DUOXA2	7.554693751	Tumor_G3
15743	NDUFA1	8.804528787	Tumor_G3
15744	NDUFA2	7.863727166	Tumor_G3
15745	NDUFA8	7.654577167	Tumor_G3
15746	NDUFB1	6.578366752	Tumor_G3
15747	NDUFB10	8.643377417	Tumor_G3
15748	NDUFB2	7.075805943	Tumor_G3
15749	NDUFB3	6.447828423	Tumor_G3
15750	NDUFB7	9.973245943	Tumor_G3
15751	CYC1	7.097226167	Tumor_G3
15752	ETFB	5.650383245	Tumor_G3
15753	UQCR10	8.50975083	Tumor_G3
15754	UQCRQ	8.202644454	Tumor_G3
15755	UQCC3	5.531708713	Tumor_G3
15756	COX15	4.397529376	Tumor_G3
15757	COX5A	7.583353146	Tumor_G3
15758	COX6B1	9.772670194	Tumor_G3

15759	COX6C	6.933327184	Tumor_G3
15760	COX7A2	7.2919631	Tumor_G3
15761	COX7B	6.580834298	Tumor_G3
15762	COX8A	10.07476065	Tumor_G3
15763	SCO1	2.604331702	Tumor_G3
15764	SURF1	7.350829675	Tumor_G3
15765	ATP6V1E1	7.034972643	Tumor_G3
15766	DLAT	4.081492778	Tumor_G3
15767	PDC	0.078632971	Tumor_G3
15768	SUCLG2	5.699083419	Tumor_G3
15769	SLC25A5	7.605236831	Tumor_G3
15770	UCP2	8.661358061	Tumor_G3
15771	SOD2	4.084518432	Tumor_G3
15772	NOX4	2.401050756	Tumor_G3
15773	DUOX1	5.946888829	Tumor_G3
15774	DUOXA1	6.61023239	Tumor_G3
15775	DUOX2	6.854042903	Tumor_G3
15776	DUOXA2	6.556535582	Tumor_G3
15777	NDUFA1	8.709566885	Tumor_G3
15778	NDUFA2	7.497251315	Tumor_G3
15779	NDUFA8	7.586810836	Tumor_G3
15780	NDUFB1	6.565302662	Tumor_G3
15781	NDUFB10	8.660641959	Tumor_G3
15782	NDUFB2	7.34828761	Tumor_G3
15783	NDUFB3	6.688332073	Tumor_G3
15784	NDUFB7	10.12396066	Tumor_G3
15785	CYC1	7.56795339	Tumor_G3
15786	ETFB	6.564580535	Tumor_G3
15787	UQCR10	8.435365252	Tumor_G3
15788	UQCRQ	8.29803121	Tumor_G3
15789	UQCC3	5.880790829	Tumor_G3
15790	COX15	4.552374417	Tumor_G3
15791	COX5A	7.456112637	Tumor_G3
15792	COX6B1	9.668060356	Tumor_G3
15793	COX6C	7.199845691	Tumor_G3
15794	COX7A2	7.589271821	Tumor_G3
15795	COX7B	6.447859373	Tumor_G3
15796	COX8A	10.516254	Tumor_G3
15797	SCO1	2.656532711	Tumor_G3
15798	SURF1	7.430785858	Tumor_G3

15799	ATP6V1E1	7.067349539	Tumor_G3
15800	DLAT	4.000024921	Tumor_G3
15801	PDC	0.05495034	Tumor_G3
15802	SUCLG2	5.249643888	Tumor_G3
15803	SLC25A5	7.918862939	Tumor_G3
15804	UCP2	9.223406427	Tumor_G3
15805	SOD2	4.128631055	Tumor_G3
15806	NOX4	1.928120775	Tumor_G3
15807	DUOX1	6.720229834	Tumor_G3
15808	DUOXA1	7.392112585	Tumor_G3
15809	DUOX2	6.531362418	Tumor_G3
15810	DUOXA2	5.694422375	Tumor_G3
15811	NDUFA1	8.437211146	Tumor_G3
15812	NDUFA2	8.528677483	Tumor_G3
15813	NDUFA8	7.24061662	Tumor_G3
15814	NDUFB1	7.361432083	Tumor_G3
15815	NDUFB10	8.971410197	Tumor_G3
15816	NDUFB2	7.657688403	Tumor_G3
15817	NDUFB3	6.596614701	Tumor_G3
15818	NDUFB7	10.6153305	Tumor_G3
15819	CYC1	8.166398811	Tumor_G3
15820	ETFB	6.721779586	Tumor_G3
15821	UQCR10	8.333160147	Tumor_G3
15822	UQCRQ	9.041059843	Tumor_G3
15823	UQCC3	5.206846138	Tumor_G3
15824	COX15	4.285284237	Tumor_G3
15825	COX5A	7.666248698	Tumor_G3
15826	COX6B1	10.55241784	Tumor_G3
15827	COX6C	7.709323429	Tumor_G3
15828	COX7A2	7.719244076	Tumor_G3
15829	COX7B	6.105267142	Tumor_G3
15830	COX8A	10.25695879	Tumor_G3
15831	SCO1	3.600394223	Tumor_G3
15832	SURF1	7.584708026	Tumor_G3
15833	ATP6V1E1	6.43553524	Tumor_G3
15834	DLAT	3.612706823	Tumor_G3
15835	PDC	0.060961191	Tumor_G3
15836	SUCLG2	6.020735575	Tumor_G3
15837	SLC25A5	8.622844127	Tumor_G3
15838	UCP2	8.066569051	Tumor_G3

15839	SOD2	3.039238318	Tumor_G3
15840	NOX4	1.374450597	Tumor_G3
15841	DUOX1	6.634394652	Tumor_G3
15842	DUOXA1	6.593414474	Tumor_G3
15843	DUOX2	8.463907166	Tumor_G3
15844	DUOXA2	7.245532164	Tumor_G3
15845	NDUFA1	9.051652598	Tumor_G3
15846	NDUFA2	7.874925271	Tumor_G3
15847	NDUFA8	7.711790189	Tumor_G3
15848	NDUFB1	6.45852852	Tumor_G3
15849	NDUFB10	8.067142222	Tumor_G3
15850	NDUFB2	7.700585741	Tumor_G3
15851	NDUFB3	6.422774704	Tumor_G3
15852	NDUFB7	9.96196867	Tumor_G3
15853	CYC1	7.718019822	Tumor_G3
15854	ETFB	6.270333396	Tumor_G3
15855	UQCR10	8.848476172	Tumor_G3
15856	UQCRQ	7.95957007	Tumor_G3
15857	UQCC3	5.37740759	Tumor_G3
15858	COX15	5.08728386	Tumor_G3
15859	COX5A	6.916586899	Tumor_G3
15860	COX6B1	9.290659427	Tumor_G3
15861	COX6C	7.143334884	Tumor_G3
15862	COX7A2	6.390008242	Tumor_G3
15863	COX7B	6.573387776	Tumor_G3
15864	COX8A	10.50479889	Tumor_G3
15865	SCO1	3.361770574	Tumor_G3
15866	SURF1	7.186042874	Tumor_G3
15867	ATP6V1E1	7.082683648	Tumor_G3
15868	DLAT	4.808504254	Tumor_G3
15869	PDC	0.081952836	Tumor_G3
15870	SUCLG2	6.460303071	Tumor_G3
15871	SLC25A5	8.137521464	Tumor_G3
15872	UCP2	8.363211574	Tumor_G3
15873	SOD2	3.460104242	Tumor_G3
15874	NOX4	1.081994398	Tumor_G3
15875	DUOX1	6.994379365	Tumor_G3
15876	DUOXA1	6.198869867	Tumor_G3
15877	DUOX2	8.732018283	Tumor_G3
15878	DUOXA2	8.025263409	Tumor_G3

15879	NDUFA1	8.963962498	Tumor_G3
15880	NDUFA2	8.773242545	Tumor_G3
15881	NDUFA8	7.9151609	Tumor_G3
15882	NDUFB1	7.92605125	Tumor_G3
15883	NDUFB10	8.886187353	Tumor_G3
15884	NDUFB2	7.596333185	Tumor_G3
15885	NDUFB3	6.257018319	Tumor_G3
15886	NDUFB7	11.16564884	Tumor_G3
15887	CYC1	6.306011938	Tumor_G3
15888	ETFB	7.694333582	Tumor_G3
15889	UQCR10	9.681908835	Tumor_G3
15890	UQCRQ	8.916186953	Tumor_G3
15891	UQCC3	6.411597083	Tumor_G3
15892	COX15	4.86438067	Tumor_G3
15893	COX5A	7.398735737	Tumor_G3
15894	COX6B1	10.96996966	Tumor_G3
15895	COX6C	8.012219463	Tumor_G3
15896	COX7A2	5.698510327	Tumor_G3
15897	COX7B	7.41768736	Tumor_G3
15898	COX8A	11.51883855	Tumor_G3
15899	SCO1	2.594860653	Tumor_G3
15900	SURF1	7.844244868	Tumor_G3
15901	ATP6V1E1	6.643729534	Tumor_G3
15902	DLAT	3.183864241	Tumor_G3
15903	PDC	0	Tumor_G3
15904	SUCLG2	4.217984383	Tumor_G3
15905	SLC25A5	4.950938925	Tumor_G3
15906	UCP2	8.101023883	Tumor_G3
15907	SOD2	2.908927514	Tumor_G3
15908	NOX4	1.587035317	Tumor_G3
15909	DUOX1	7.421717439	Tumor_G3
15910	DUOXA1	6.683580771	Tumor_G3
15911	DUOX2	8.293745274	Tumor_G3
15912	DUOXA2	8.730544708	Tumor_G3
15913	NDUFA1	8.262577638	Tumor_G3
15914	NDUFA2	8.47281946	Tumor_G3
15915	NDUFA8	7.320567684	Tumor_G3
15916	NDUFB1	6.980104863	Tumor_G3
15917	NDUFB10	8.780589458	Tumor_G3
15918	NDUFB2	7.346725779	Tumor_G3

15919	NDUFB3	6.022670767	Tumor_G3
15920	NDUFB7	10.49499168	Tumor_G3
15921	CYC1	6.056396001	Tumor_G3
15922	ETFB	7.170447908	Tumor_G3
15923	UQCR10	8.24933041	Tumor_G3
15924	UQCRQ	7.874140237	Tumor_G3
15925	UQCC3	6.428107359	Tumor_G3
15926	COX15	4.461703986	Tumor_G3
15927	COX5A	6.167104176	Tumor_G3
15928	COX6B1	10.34010546	Tumor_G3
15929	COX6C	6.94593747	Tumor_G3
15930	COX7A2	5.4689216	Tumor_G3
15931	COX7B	6.828438505	Tumor_G3
15932	COX8A	11.55495157	Tumor_G3
15933	SCO1	3.08254724	Tumor_G3
15934	SURF1	7.519405936	Tumor_G3
15935	ATP6V1E1	6.593333095	Tumor_G3
15936	DLAT	3.751465907	Tumor_G3
15937	PDC	0	Tumor_G3
15938	SUCLG2	4.057967519	Tumor_G3
15939	SLC25A5	4.960425813	Tumor_G3
15940	UCP2	8.615153841	Tumor_G3
15941	SOD2	2.607281997	Tumor_G3
15942	NOX4	1.357754166	Tumor_G3
15943	DUOX1	7.481256565	Tumor_G3
15944	DUOXA1	7.51693783	Tumor_G3
15945	DUOX2	8.153339182	Tumor_G3
15946	DUOXA2	7.974949925	Tumor_G3
15947	NDUFA1	8.807141649	Tumor_G3
15948	NDUFA2	7.202194899	Tumor_G3
15949	NDUFA8	7.839599985	Tumor_G3
15950	NDUFB1	6.224437415	Tumor_G3
15951	NDUFB10	8.19606895	Tumor_G3
15952	NDUFB2	7.720313988	Tumor_G3
15953	NDUFB3	6.931874185	Tumor_G3
15954	NDUFB7	9.09124687	Tumor_G3
15955	CYC1	8.124150965	Tumor_G3
15956	ETFB	5.583980422	Tumor_G3
15957	UQCR10	8.115825686	Tumor_G3
15958	UQCRQ	7.806051752	Tumor_G3

15959	UQCC3	4.950326699	Tumor_G3
15960	COX15	5.774821838	Tumor_G3
15961	COX5A	8.399757619	Tumor_G3
15962	COX6B1	9.389421316	Tumor_G3
15963	COX6C	7.368813222	Tumor_G3
15964	COX7A2	7.771411887	Tumor_G3
15965	COX7B	7.347841864	Tumor_G3
15966	COX8A	10.61927796	Tumor_G3
15967	SCO1	4.236664048	Tumor_G3
15968	SURF1	7.46366135	Tumor_G3
15969	ATP6V1E1	7.565909816	Tumor_G3
15970	DLAT	5.924219367	Tumor_G3
15971	PDC	0.11589913	Tumor_G3
15972	SUCLG2	7.460679994	Tumor_G3
15973	SLC25A5	9.883771735	Tumor_G3
15974	UCP2	5.0619941	Tumor_G3
15975	SOD2	5.34579638	Tumor_G3
15976	NOX4	1.564049319	Tumor_G3
15977	DUOX1	6.427552906	Tumor_G3
15978	DUOXA1	6.241300126	Tumor_G3
15979	DUOX2	8.000103427	Tumor_G3
15980	DUOXA2	5.958859257	Tumor_G3
15981	NDUFA1	8.504674977	Tumor_G3
15982	NDUFA2	7.948774791	Tumor_G3
15983	NDUFA8	7.256421915	Tumor_G3
15984	NDUFB1	7.245665926	Tumor_G3
15985	NDUFB10	8.177695421	Tumor_G3
15986	NDUFB2	7.993979729	Tumor_G3
15987	NDUFB3	6.731685812	Tumor_G3
15988	NDUFB7	9.650572121	Tumor_G3
15989	CYC1	7.271630816	Tumor_G3
15990	ETFB	6.204761697	Tumor_G3
15991	UQCR10	8.278974263	Tumor_G3
15992	UQCRQ	8.055956235	Tumor_G3
15993	UQCC3	5.115674777	Tumor_G3
15994	COX15	5.314975933	Tumor_G3
15995	COX5A	7.039201825	Tumor_G3
15996	COX6B1	8.771517993	Tumor_G3
15997	COX6C	6.758930399	Tumor_G3
15998	COX7A2	7.150179872	Tumor_G3

15999	COX7B	5.876546144	Tumor_G3
16000	COX8A	9.684979477	Tumor_G3
16001	SCO1	4.044979597	Tumor_G3
16002	SURF1	7.401420191	Tumor_G3
16003	ATP6V1E1	6.869906721	Tumor_G3
16004	DLAT	4.76719277	Tumor_G3
16005	PDC	0	Tumor_G3
16006	SUCLG2	5.724257383	Tumor_G3
16007	SLC25A5	7.919928023	Tumor_G3
16008	UCP2	7.641935599	Tumor_G3
16009	SOD2	3.819832582	Tumor_G3
16010	NOX4	1.4449707	Tumor_G3
16011	DUOX1	6.438165986	Tumor_G3
16012	DUOXA1	6.167788205	Tumor_G3
16013	DUOX2	6.929867233	Tumor_G3
16014	DUOXA2	7.953674618	Tumor_G3
16015	NDUFA1	9.086363538	Tumor_G3
16016	NDUFA2	7.320352562	Tumor_G3
16017	NDUFA8	7.783075216	Tumor_G3
16018	NDUFB1	6.462413653	Tumor_G3
16019	NDUFB10	7.737991069	Tumor_G3
16020	NDUFB2	7.20482986	Tumor_G3
16021	NDUFB3	7.272076848	Tumor_G3
16022	NDUFB7	8.596318485	Tumor_G3
16023	CYC1	8.125043425	Tumor_G3
16024	ETFB	6.042427345	Tumor_G3
16025	UQCR10	8.452959728	Tumor_G3
16026	UQCRQ	7.885355928	Tumor_G3
16027	UQCC3	4.711347544	Tumor_G3
16028	COX15	5.189888533	Tumor_G3
16029	COX5A	8.272133067	Tumor_G3
16030	COX6B1	9.151922479	Tumor_G3
16031	COX6C	7.497492596	Tumor_G3
16032	COX7A2	7.834461607	Tumor_G3
16033	COX7B	7.411206541	Tumor_G3
16034	COX8A	10.23118854	Tumor_G3
16035	SCO1	3.920530701	Tumor_G3
16036	SURF1	6.455965295	Tumor_G3
16037	ATP6V1E1	7.065178708	Tumor_G3
16038	DLAT	5.720226535	Tumor_G3

16039	PDC	0.06145207	Tumor_G3
16040	SUCLG2	7.016484013	Tumor_G3
16041	SLC25A5	9.122906501	Tumor_G3
16042	UCP2	8.450212171	Tumor_G3
16043	SOD2	5.105290259	Tumor_G3
16044	NOX4	2.238228036	Tumor_G3
16045	DUOX1	7.538932269	Tumor_G3
16046	DUOXA1	6.147116227	Tumor_G3
16047	DUOX2	7.387054513	Tumor_G3
16048	DUOXA2	7.947995369	Tumor_G3
16049	NDUFA1	9.312658127	Tumor_G3
16050	NDUFA2	7.424904809	Tumor_G3
16051	NDUFA8	8.162854806	Tumor_G3
16052	NDUFB1	6.998176912	Tumor_G3
16053	NDUFB10	8.31524959	Tumor_G3
16054	NDUFB2	7.995968883	Tumor_G3
16055	NDUFB3	7.267510251	Tumor_G3
16056	NDUFB7	9.510042768	Tumor_G3
16057	CYC1	7.91267196	Tumor_G3
16058	ETFB	5.45791978	Tumor_G3
16059	UQCR10	9.223366626	Tumor_G3
16060	UQCRQ	8.239271852	Tumor_G3
16061	UQCC3	4.894485396	Tumor_G3
16062	COX15	5.08210645	Tumor_G3
16063	COX5A	8.148461541	Tumor_G3
16064	COX6B1	9.714945374	Tumor_G3
16065	COX6C	7.710243628	Tumor_G3
16066	COX7A2	7.601024324	Tumor_G3
16067	COX7B	7.553409769	Tumor_G3
16068	COX8A	10.38943733	Tumor_G3
16069	SCO1	4.052987917	Tumor_G3
16070	SURF1	6.857091875	Tumor_G3
16071	ATP6V1E1	6.750621425	Tumor_G3
16072	DLAT	5.733177447	Tumor_G3
16073	PDC	0.137952849	Tumor_G3
16074	SUCLG2	6.296999349	Tumor_G3
16075	SLC25A5	8.90815862	Tumor_G3
16076	UCP2	5.113568467	Tumor_G3
16077	SOD2	5.095471079	Tumor_G3
16078	NOX4	1.897501236	Tumor_G3

16079	DUOX1	7.225893022	Tumor_G3
16080	DUOXA1	6.59129154	Tumor_G3
16081	DUOX2	7.782309269	Tumor_G3
16082	DUOXA2	6.83311544	Tumor_G3
16083	NDUFA1	8.617255062	Tumor_G3
16084	NDUFA2	7.393850226	Tumor_G3
16085	NDUFA8	7.384821408	Tumor_G3
16086	NDUFB1	6.485322042	Tumor_G3
16087	NDUFB10	7.840654273	Tumor_G3
16088	NDUFB2	6.742574323	Tumor_G3
16089	NDUFB3	6.444150585	Tumor_G3
16090	NDUFB7	9.493603448	Tumor_G3
16091	CYC1	7.192855843	Tumor_G3
16092	ETFB	5.716117523	Tumor_G3
16093	UQCR10	8.196178399	Tumor_G3
16094	UQCRQ	7.824752789	Tumor_G3
16095	UQCC3	5.079079402	Tumor_G3
16096	COX15	4.677551022	Tumor_G3
16097	COX5A	7.093734313	Tumor_G3
16098	COX6B1	9.183061798	Tumor_G3
16099	COX6C	7.02443519	Tumor_G3
16100	COX7A2	7.304856537	Tumor_G3
16101	COX7B	6.402019064	Tumor_G3
16102	COX8A	9.891761673	Tumor_G3
16103	SCO1	3.020358825	Tumor_G3
16104	SURF1	6.818000492	Tumor_G3
16105	ATP6V1E1	7.08035189	Tumor_G3
16106	DLAT	4.586231773	Tumor_G3
16107	PDC	0	Tumor_G3
16108	SUCLG2	5.755343693	Tumor_G3
16109	SLC25A5	7.771519147	Tumor_G3
16110	UCP2	8.000503991	Tumor_G3
16111	SOD2	4.45582107	Tumor_G3
16112	NOX4	1.574096605	Tumor_G3
16113	DUOX1	7.43077218	Tumor_G3
16114	DUOXA1	7.488398624	Tumor_G3
16115	DUOX2	7.247094318	Tumor_G3
16116	DUOXA2	5.101082059	Tumor_G3
16117	NDUFA1	9.083521052	Tumor_G3
16118	NDUFA2	7.074309892	Tumor_G3

16119	NDUFA8	7.851534836	Tumor_G3
16120	NDUFB1	6.696397724	Tumor_G3
16121	NDUFB10	7.726710153	Tumor_G3
16122	NDUFB2	7.85020638	Tumor_G3
16123	NDUFB3	7.328477897	Tumor_G3
16124	NDUFB7	9.457961822	Tumor_G3
16125	CYC1	7.998906232	Tumor_G3
16126	ETFB	5.256821694	Tumor_G3
16127	UQCR10	8.457623118	Tumor_G3
16128	UQCRQ	7.796127338	Tumor_G3
16129	UQCC3	4.542517171	Tumor_G3
16130	COX15	5.12429635	Tumor_G3
16131	COX5A	8.036554394	Tumor_G3
16132	COX6B1	9.132212272	Tumor_G3
16133	COX6C	7.287983131	Tumor_G3
16134	COX7A2	7.648065463	Tumor_G3
16135	COX7B	7.247745084	Tumor_G3
16136	COX8A	10.56776716	Tumor_G3
16137	SCO1	3.710440287	Tumor_G3
16138	SURF1	6.381951978	Tumor_G3
16139	ATP6V1E1	7.32668045	Tumor_G3
16140	DLAT	5.635968386	Tumor_G3
16141	PDC	0	Tumor_G3
16142	SUCLG2	6.884732401	Tumor_G3
16143	SLC25A5	9.375719238	Tumor_G3
16144	UCP2	7.238975778	Tumor_G3
16145	SOD2	5.123195005	Tumor_G3
16146	NOX4	0.552993448	Tumor_G3
16147	DUOX1	5.798439541	Tumor_G3
16148	DUOXA1	5.726868124	Tumor_G3
16149	DUOX2	6.007919277	Tumor_G3
16150	DUOXA2	5.186024085	Tumor_G3
16151	NDUFA1	9.117844345	Tumor_G3
16152	NDUFA2	7.869552076	Tumor_G3
16153	NDUFA8	7.881842527	Tumor_G3
16154	NDUFB1	7.115284462	Tumor_G3
16155	NDUFB10	7.763179294	Tumor_G3
16156	NDUFB2	7.459942255	Tumor_G3
16157	NDUFB3	6.953029793	Tumor_G3
16158	NDUFB7	9.442452673	Tumor_G3

16159	CYC1	6.777299893	Tumor_G3
16160	ETFB	5.00280057	Tumor_G3
16161	UQCR10	8.881838853	Tumor_G3
16162	UQCRQ	8.200384161	Tumor_G3
16163	UQCC3	4.475817542	Tumor_G3
16164	COX15	4.425925192	Tumor_G3
16165	COX5A	7.243223242	Tumor_G3
16166	COX6B1	9.678959448	Tumor_G3
16167	COX6C	7.475189043	Tumor_G3
16168	COX7A2	7.179084225	Tumor_G3
16169	COX7B	6.847144271	Tumor_G3
16170	COX8A	10.38365772	Tumor_G3
16171	SCO1	3.023745557	Tumor_G3
16172	SURF1	6.616008732	Tumor_G3
16173	ATP6V1E1	6.342665243	Tumor_G3
16174	DLAT	4.625280398	Tumor_G3
16175	PDC	0	Tumor_G3
16176	SUCLG2	5.999454455	Tumor_G3
16177	SLC25A5	7.695334745	Tumor_G3
16178	UCP2	7.834489362	Tumor_G3
16179	SOD2	3.951985707	Tumor_G3
16180	NOX4	1.556246267	Tumor_G3
16181	DUOX1	6.076749277	Tumor_G3
16182	DUOXA1	5.320847608	Tumor_G3
16183	DUOX2	6.182807427	Tumor_G3
16184	DUOXA2	4.694993849	Tumor_G3
16185	NDUFA1	8.937290451	Tumor_G3
16186	NDUFA2	7.561419023	Tumor_G3
16187	NDUFA8	8.042812635	Tumor_G3
16188	NDUFB1	6.876972586	Tumor_G3
16189	NDUFB10	8.111079285	Tumor_G3
16190	NDUFB2	7.283592363	Tumor_G3
16191	NDUFB3	7.12227918	Tumor_G3
16192	NDUFB7	9.36382563	Tumor_G3
16193	CYC1	6.800936852	Tumor_G3
16194	ETFB	5.639386096	Tumor_G3
16195	UQCR10	8.781268262	Tumor_G3
16196	UQCRQ	8.304655531	Tumor_G3
16197	UQCC3	5.048782187	Tumor_G3
16198	COX15	4.706731624	Tumor_G3

16199	COX5A	8.24467878	Tumor_G3
16200	COX6B1	9.519081858	Tumor_G3
16201	COX6C	7.560805889	Tumor_G3
16202	COX7A2	7.595412717	Tumor_G3
16203	COX7B	7.304607326	Tumor_G3
16204	COX8A	10.73804776	Tumor_G3
16205	SCO1	3.316986528	Tumor_G3
16206	SURF1	6.93180068	Tumor_G3
16207	ATP6V1E1	6.55047412	Tumor_G3
16208	DLAT	5.167076326	Tumor_G3
16209	PDC	0.04735039	Tumor_G3
16210	SUCLG2	6.417025354	Tumor_G3
16211	SLC25A5	7.870922727	Tumor_G3
16212	UCP2	7.858235049	Tumor_G3
16213	SOD2	4.523291853	Tumor_G3
16214	NOX4	1.5817973	Tumor_G3
16215	DUOX1	8.193265182	Tumor_G3
16216	DUOXA1	7.352051225	Tumor_G3
16217	DUOX2	9.340134287	Tumor_G3
16218	DUOXA2	7.653762924	Tumor_G3
16219	NDUFA1	9.198860126	Tumor_G3
16220	NDUFA2	7.932082611	Tumor_G3
16221	NDUFA8	8.399105709	Tumor_G3
16222	NDUFB1	7.043849771	Tumor_G3
16223	NDUFB10	8.202334046	Tumor_G3
16224	NDUFB2	8.734702427	Tumor_G3
16225	NDUFB3	7.667024591	Tumor_G3
16226	NDUFB7	10.02532828	Tumor_G3
16227	CYC1	8.157868312	Tumor_G3
16228	ETFB	5.785845098	Tumor_G3
16229	UQCR10	9.200516063	Tumor_G3
16230	UQCRQ	8.572307085	Tumor_G3
16231	UQCC3	4.482795361	Tumor_G3
16232	COX15	5.226116871	Tumor_G3
16233	COX5A	7.959178956	Tumor_G3
16234	COX6B1	9.799900525	Tumor_G3
16235	COX6C	7.634242329	Tumor_G3
16236	COX7A2	8.112126176	Tumor_G3
16237	COX7B	7.880575235	Tumor_G3
16238	COX8A	10.94850241	Tumor_G3

16239	SCO1	3.688673087	Tumor_G3
16240	SURF1	6.737726819	Tumor_G3
16241	ATP6V1E1	7.123752904	Tumor_G3
16242	DLAT	5.242424455	Tumor_G3
16243	PDC	0.105539507	Tumor_G3
16244	SUCLG2	6.794431968	Tumor_G3
16245	SLC25A5	8.695475779	Tumor_G3
16246	UCP2	9.38311066	Tumor_G3
16247	SOD2	4.069785152	Tumor_G3
16248	NOX4	2.05338746	Tumor_G3
16249	DUOX1	6.676196449	Tumor_G3
16250	DUOXA1	5.934895287	Tumor_G3
16251	DUOX2	7.234808904	Tumor_G3
16252	DUOXA2	6.495229884	Tumor_G3
16253	NDUFA1	8.956856547	Tumor_G3
16254	NDUFA2	7.536722283	Tumor_G3
16255	NDUFA8	8.123953072	Tumor_G3
16256	NDUFB1	6.526510579	Tumor_G3
16257	NDUFB10	8.288123071	Tumor_G3
16258	NDUFB2	8.08011647	Tumor_G3
16259	NDUFB3	7.260030436	Tumor_G3
16260	NDUFB7	9.510728727	Tumor_G3
16261	CYC1	8.136418603	Tumor_G3
16262	ETFB	5.138399249	Tumor_G3
16263	UQCR10	8.211595758	Tumor_G3
16264	UQCRQ	7.991902143	Tumor_G3
16265	UQCC3	5.437619359	Tumor_G3
16266	COX15	5.220926518	Tumor_G3
16267	COX5A	8.50149248	Tumor_G3
16268	COX6B1	9.34243436	Tumor_G3
16269	COX6C	7.407069693	Tumor_G3
16270	COX7A2	7.82188083	Tumor_G3
16271	COX7B	7.506807448	Tumor_G3
16272	COX8A	10.52324379	Tumor_G3
16273	SCO1	3.757657279	Tumor_G3
16274	SURF1	6.894061355	Tumor_G3
16275	ATP6V1E1	7.488236564	Tumor_G3
16276	DLAT	5.808351604	Tumor_G3
16277	PDC	0.054056827	Tumor_G3
16278	SUCLG2	6.391549726	Tumor_G3

16279	SLC25A5	8.996472956	Tumor_G3
16280	UCP2	3.536708017	Tumor_G3
16281	SOD2	4.764310548	Tumor_G3
16282	NOX4	1.690004927	Tumor_G3
16283	DUOX1	6.421060401	Tumor_G3
16284	DUOXA1	5.895844485	Tumor_G3
16285	DUOX2	7.40620559	Tumor_G3
16286	DUOXA2	6.777576803	Tumor_G3
16287	NDUFA1	8.761663948	Tumor_G3
16288	NDUFA2	7.674142822	Tumor_G3
16289	NDUFA8	7.539572951	Tumor_G3
16290	NDUFB1	6.337714107	Tumor_G3
16291	NDUFB10	7.930805841	Tumor_G3
16292	NDUFB2	7.282394586	Tumor_G3
16293	NDUFB3	6.75673495	Tumor_G3
16294	NDUFB7	9.444574308	Tumor_G3
16295	CYC1	7.65125648	Tumor_G3
16296	ETFB	5.478830975	Tumor_G3
16297	UQCR10	8.377483377	Tumor_G3
16298	UQCRQ	8.065213992	Tumor_G3
16299	UQCC3	5.351495621	Tumor_G3
16300	COX15	5.112067864	Tumor_G3
16301	COX5A	7.630534509	Tumor_G3
16302	COX6B1	8.967112713	Tumor_G3
16303	COX6C	7.177758225	Tumor_G3
16304	COX7A2	7.550768266	Tumor_G3
16305	COX7B	6.541421949	Tumor_G3
16306	COX8A	10.39332873	Tumor_G3
16307	SCO1	4.04906953	Tumor_G3
16308	SURF1	7.516808834	Tumor_G3
16309	ATP6V1E1	6.682273023	Tumor_G3
16310	DLAT	5.035025711	Tumor_G3
16311	PDC	0.294192643	Tumor_G3
16312	SUCLG2	6.516029385	Tumor_G3
16313	SLC25A5	8.666952645	Tumor_G3
16314	UCP2	6.97718113	Tumor_G3
16315	SOD2	4.271633462	Tumor_G3
16316	NOX4	1.811098467	Tumor_G3
16317	DUOX1	7.872304036	Tumor_G3
16318	DUOXA1	7.618209072	Tumor_G3

16319	DUOX2	8.760088448	Tumor_G3
16320	DUOXA2	6.905767859	Tumor_G3
16321	NDUFA1	9.102341021	Tumor_G3
16322	NDUFA2	7.963284657	Tumor_G3
16323	NDUFA8	8.250230864	Tumor_G3
16324	NDUFB1	7.315217807	Tumor_G3
16325	NDUFB10	8.569919414	Tumor_G3
16326	NDUFB2	7.372828118	Tumor_G3
16327	NDUFB3	6.866979003	Tumor_G3
16328	NDUFB7	10.05306496	Tumor_G3
16329	CYC1	7.37042212	Tumor_G3
16330	ETFB	5.662583392	Tumor_G3
16331	UQCR10	9.100301181	Tumor_G3
16332	UQCRQ	8.421502264	Tumor_G3
16333	UQCC3	5.426298004	Tumor_G3
16334	COX15	4.369763843	Tumor_G3
16335	COX5A	8.034673501	Tumor_G3
16336	COX6B1	10.43776068	Tumor_G3
16337	COX6C	8.145624524	Tumor_G3
16338	COX7A2	7.870647399	Tumor_G3
16339	COX7B	7.637271983	Tumor_G3
16340	COX8A	10.26568308	Tumor_G3
16341	SCO1	3.142602621	Tumor_G3
16342	SURF1	7.514654733	Tumor_G3
16343	ATP6V1E1	6.16731798	Tumor_G3
16344	DLAT	4.531015204	Tumor_G3
16345	PDC	0.06404782	Tumor_G3
16346	SUCLG2	5.589509648	Tumor_G3
16347	SLC25A5	7.698952609	Tumor_G3
16348	UCP2	8.197129216	Tumor_G3
16349	SOD2	3.828190612	Tumor_G3
16350	NOX4	1.52334061	Tumor_G3
16351	DUOX1	7.056131809	Tumor_G3
16352	DUOXA1	7.440561063	Tumor_G3
16353	DUOX2	7.760316999	Tumor_G3
16354	DUOXA2	6.51246046	Tumor_G3
16355	NDUFA1	8.52620053	Tumor_G3
16356	NDUFA2	7.533751314	Tumor_G3
16357	NDUFA8	7.375050795	Tumor_G3
16358	NDUFB1	6.279538858	Tumor_G3

16359	NDUFB10	7.869193388	Tumor_G3
16360	NDUFB2	7.087094328	Tumor_G3
16361	NDUFB3	6.696941461	Tumor_G3
16362	NDUFB7	9.234583685	Tumor_G3
16363	CYC1	7.115631224	Tumor_G3
16364	ETFB	5.419976268	Tumor_G3
16365	UQCR10	8.1810959	Tumor_G3
16366	UQCRQ	8.00611584	Tumor_G3
16367	UQCC3	4.492889923	Tumor_G3
16368	COX15	4.138576676	Tumor_G3
16369	COX5A	7.470225383	Tumor_G3
16370	COX6B1	9.430018515	Tumor_G3
16371	COX6C	7.09099297	Tumor_G3
16372	COX7A2	7.631620722	Tumor_G3
16373	COX7B	6.600147642	Tumor_G3
16374	COX8A	9.385176818	Tumor_G3
16375	SCO1	2.73282641	Tumor_G3
16376	SURF1	6.824874235	Tumor_G3
16377	ATP6V1E1	7.414293671	Tumor_G3
16378	DLAT	4.26311475	Tumor_G3
16379	PDC	0	Tumor_G3
16380	SUCLG2	5.784671954	Tumor_G3
16381	SLC25A5	8.369282864	Tumor_G3
16382	UCP2	8.25693498	Tumor_G3
16383	SOD2	5.523631559	Tumor_G3
16384	NOX4	2.550904346	Tumor_G3
16385	DUOX1	5.040291973	Tumor_G3
16386	DUOXA1	5.639542207	Tumor_G3
16387	DUOX2	4.150588683	Tumor_G3
16388	DUOXA2	2.682014702	Tumor_G3
16389	NDUFA1	8.784479021	Tumor_G3
16390	NDUFA2	7.548014104	Tumor_G3
16391	NDUFA8	7.888985648	Tumor_G3
16392	NDUFB1	6.92213915	Tumor_G3
16393	NDUFB10	7.863808105	Tumor_G3
16394	NDUFB2	7.69397209	Tumor_G3
16395	NDUFB3	7.165791556	Tumor_G3
16396	NDUFB7	9.706919578	Tumor_G3
16397	CYC1	7.429195322	Tumor_G3
16398	ETFB	5.584371223	Tumor_G3

16399	UQCR10	8.809176795	Tumor_G3
16400	UQCRQ	8.059005153	Tumor_G3
16401	UQCC3	4.887610036	Tumor_G3
16402	COX15	5.350676178	Tumor_G3
16403	COX5A	7.669656212	Tumor_G3
16404	COX6B1	9.344807111	Tumor_G3
16405	COX6C	7.262018232	Tumor_G3
16406	COX7A2	7.521303515	Tumor_G3
16407	COX7B	6.983523942	Tumor_G3
16408	COX8A	10.23544157	Tumor_G3
16409	SCO1	4.12216043	Tumor_G3
16410	SURF1	7.080575896	Tumor_G3
16411	ATP6V1E1	6.960722486	Tumor_G3
16412	DLAT	5.37526647	Tumor_G3
16413	PDC	0.144657909	Tumor_G3
16414	SUCLG2	6.840494192	Tumor_G3
16415	SLC25A5	8.396668821	Tumor_G3
16416	UCP2	7.039446731	Tumor_G3
16417	SOD2	4.769806938	Tumor_G3
16418	NOX4	2.034417927	Tumor_G3
16419	DUOX1	7.135437544	Tumor_G3
16420	DUOXA1	6.542061687	Tumor_G3
16421	DUOX2	7.359510457	Tumor_G3
16422	DUOXA2	7.084642787	Tumor_G3
16423	NDUFA1	8.447072671	Tumor_G3
16424	NDUFA2	7.322702079	Tumor_G3
16425	NDUFA8	8.254598017	Tumor_G3
16426	NDUFB1	6.155288134	Tumor_G3
16427	NDUFB10	8.338645763	Tumor_G3
16428	NDUFB2	7.780384455	Tumor_G3
16429	NDUFB3	7.073000421	Tumor_G3
16430	NDUFB7	9.362979715	Tumor_G3
16431	CYC1	8.295362618	Tumor_G3
16432	ETFB	5.675862996	Tumor_G3
16433	UQCR10	8.297701595	Tumor_G3
16434	UQCRQ	7.963625337	Tumor_G3
16435	UQCC3	5.007344142	Tumor_G3
16436	COX15	5.889725703	Tumor_G3
16437	COX5A	8.37976844	Tumor_G3
16438	COX6B1	9.385174004	Tumor_G3

16439	COX6C	7.234917194	Tumor_G3
16440	COX7A2	7.948197096	Tumor_G3
16441	COX7B	7.506971246	Tumor_G3
16442	COX8A	10.83594617	Tumor_G3
16443	SCO1	4.377933043	Tumor_G3
16444	SURF1	6.968121223	Tumor_G3
16445	ATP6V1E1	7.706031113	Tumor_G3
16446	DLAT	6.403130773	Tumor_G3
16447	PDC	0.155017357	Tumor_G3
16448	SUCLG2	7.919349289	Tumor_G3
16449	SLC25A5	9.971874073	Tumor_G3
16450	UCP2	4.938063858	Tumor_G3
16451	SOD2	5.466454139	Tumor_G3
16452	NOX4	2.018720016	Tumor_G3
16453	DUOX1	7.551401391	Tumor_G3
16454	DUOXA1	6.794528192	Tumor_G3
16455	DUOX2	8.487466916	Tumor_G3
16456	DUOXA2	7.152828426	Tumor_G3
16457	NDUFA1	9.020877254	Tumor_G3
16458	NDUFA2	8.052318675	Tumor_G3
16459	NDUFA8	7.484829728	Tumor_G3
16460	NDUFB1	7.084923774	Tumor_G3
16461	NDUFB10	8.53708064	Tumor_G3
16462	NDUFB2	7.792990357	Tumor_G3
16463	NDUFB3	6.87008107	Tumor_G3
16464	NDUFB7	10.27739025	Tumor_G3
16465	CYC1	7.315010125	Tumor_G3
16466	ETFB	6.275218613	Tumor_G3
16467	UQCR10	8.64543984	Tumor_G3
16468	UQCRQ	8.506924414	Tumor_G3
16469	UQCC3	5.570593958	Tumor_G3
16470	COX15	4.342707445	Tumor_G3
16471	COX5A	7.874494577	Tumor_G3
16472	COX6B1	9.882786804	Tumor_G3
16473	COX6C	7.095534716	Tumor_G3
16474	COX7A2	7.530496485	Tumor_G3
16475	COX7B	6.827897916	Tumor_G3
16476	COX8A	10.51011947	Tumor_G3
16477	SCO1	2.557771937	Tumor_G3
16478	SURF1	7.240879049	Tumor_G3

16479	ATP6V1E1	7.573965907	Tumor_G3
16480	DLAT	3.945061307	Tumor_G3
16481	PDC	0	Tumor_G3
16482	SUCLG2	5.4359193	Tumor_G3
16483	SLC25A5	8.147055123	Tumor_G3
16484	UCP2	8.676877494	Tumor_G3
16485	SOD2	4.526256854	Tumor_G3
16486	NOX4	1.316560072	Tumor_G3
16487	DUOX1	5.230477346	Tumor_G3
16488	DUOXA1	5.883229774	Tumor_G3
16489	DUOX2	4.549936151	Tumor_G3
16490	DUOXA2	4.515875377	Tumor_G3
16491	NDUFA1	9.35665309	Tumor_G3
16492	NDUFA2	7.980973445	Tumor_G3
16493	NDUFA8	7.731632375	Tumor_G3
16494	NDUFB1	6.891409989	Tumor_G3
16495	NDUFB10	8.645123386	Tumor_G3
16496	NDUFB2	7.921455216	Tumor_G3
16497	NDUFB3	6.863120388	Tumor_G3
16498	NDUFB7	10.06096132	Tumor_G3
16499	CYC1	7.62999897	Tumor_G3
16500	ETFB	5.781632584	Tumor_G3
16501	UQCR10	9.140884291	Tumor_G3
16502	UQCRQ	8.302788073	Tumor_G3
16503	UQCC3	6.016504659	Tumor_G3
16504	COX15	4.758505227	Tumor_G3
16505	COX5A	7.804487686	Tumor_G3
16506	COX6B1	10.05143884	Tumor_G3
16507	COX6C	7.328660262	Tumor_G3
16508	COX7A2	7.556415764	Tumor_G3
16509	COX7B	7.034522646	Tumor_G3
16510	COX8A	10.81245189	Tumor_G3
16511	SCO1	3.566199715	Tumor_G3
16512	SURF1	7.308349335	Tumor_G3
16513	ATP6V1E1	6.340973036	Tumor_G3
16514	DLAT	4.494951873	Tumor_G3
16515	PDC	0.058735579	Tumor_G3
16516	SUCLG2	5.634839063	Tumor_G3
16517	SLC25A5	7.800412584	Tumor_G3
16518	UCP2	7.61422042	Tumor_G3

16519	SOD2	3.949368229	Tumor_G3
16520	NOX4	1.610510147	Tumor_G3
16521	DUOX1	7.345483298	Tumor_G3
16522	DUOXA1	7.210759855	Tumor_G3
16523	DUOX2	7.668993657	Tumor_G3
16524	DUOXA2	7.775375491	Tumor_G3
16525	NDUFA1	8.897205978	Tumor_G3
16526	NDUFA2	7.28231853	Tumor_G3
16527	NDUFA8	7.928196657	Tumor_G3
16528	NDUFB1	6.64416115	Tumor_G3
16529	NDUFB10	7.823938399	Tumor_G3
16530	NDUFB2	7.509070273	Tumor_G3
16531	NDUFB3	7.144544245	Tumor_G3
16532	NDUFB7	9.432332122	Tumor_G3
16533	CYC1	7.711918881	Tumor_G3
16534	ETFB	5.685762806	Tumor_G3
16535	UQCR10	8.491191813	Tumor_G3
16536	UQCRQ	8.116690361	Tumor_G3
16537	UQCC3	5.051497679	Tumor_G3
16538	COX15	4.889157036	Tumor_G3
16539	COX5A	7.883333477	Tumor_G3
16540	COX6B1	9.342245276	Tumor_G3
16541	COX6C	7.437993693	Tumor_G3
16542	COX7A2	7.570861856	Tumor_G3
16543	COX7B	7.277226231	Tumor_G3
16544	COX8A	10.53871267	Tumor_G3
16545	SCO1	3.498203125	Tumor_G3
16546	SURF1	6.918854115	Tumor_G3
16547	ATP6V1E1	7.202319805	Tumor_G3
16548	DLAT	5.303103466	Tumor_G3
16549	PDC	0.062033326	Tumor_G3
16550	SUCLG2	6.640042468	Tumor_G3
16551	SLC25A5	8.619991472	Tumor_G3
16552	UCP2	5.156908381	Tumor_G3
16553	SOD2	4.622784113	Tumor_G3
16554	NOX4	1.135325082	Tumor_G3
16555	DUOX1	8.011120882	Tumor_G3
16556	DUOXA1	7.734610349	Tumor_G3
16557	DUOX2	8.465068182	Tumor_G3
16558	DUOXA2	6.551259991	Tumor_G3

16559	NDUFA1	8.076510558	Tumor_G3
16560	NDUFA2	7.404188739	Tumor_G3
16561	NDUFA8	7.472919923	Tumor_G3
16562	NDUFB1	5.919374419	Tumor_G3
16563	NDUFB10	8.413078558	Tumor_G3
16564	NDUFB2	7.190818294	Tumor_G3
16565	NDUFB3	6.247252987	Tumor_G3
16566	NDUFB7	9.613094426	Tumor_G3
16567	CYC1	7.419417438	Tumor_G3
16568	ETFB	6.122733819	Tumor_G3
16569	UQCR10	8.412834093	Tumor_G3
16570	UQCRQ	7.937290517	Tumor_G3
16571	UQCC3	5.434547366	Tumor_G3
16572	COX15	4.184696392	Tumor_G3
16573	COX5A	7.274300375	Tumor_G3
16574	COX6B1	9.536345489	Tumor_G3
16575	COX6C	6.781996242	Tumor_G3
16576	COX7A2	7.390513731	Tumor_G3
16577	COX7B	6.21395872	Tumor_G3
16578	COX8A	10.51230982	Tumor_G3
16579	SCO1	2.80357694	Tumor_G3
16580	SURF1	7.002309543	Tumor_G3
16581	ATP6V1E1	7.045679428	Tumor_G3
16582	DLAT	4.036459802	Tumor_G3
16583	PDC	0.159474961	Tumor_G3
16584	SUCLG2	5.778908477	Tumor_G3
16585	SLC25A5	7.776562565	Tumor_G3
16586	UCP2	9.265590428	Tumor_G3
16587	SOD2	4.229321049	Tumor_G3
16588	NOX4	1.988964591	Tumor_G3
16589	DUOX1	6.376260916	Tumor_G3
16590	DUOXA1	6.974233715	Tumor_G3
16591	DUOX2	6.521382839	Tumor_G3
16592	DUOXA2	5.519657215	Tumor_G3
16593	NDUFA1	9.315922724	Tumor_G3
16594	NDUFA2	7.968889245	Tumor_G3
16595	NDUFA8	7.535823091	Tumor_G3
16596	NDUFB1	6.240085493	Tumor_G3
16597	NDUFB10	9.155892329	Tumor_G3
16598	NDUFB2	7.239856724	Tumor_G3

16599	NDUFB3	6.14170339	Tumor_G3
16600	NDUFB7	9.254360649	Tumor_G3
16601	CYC1	7.936841076	Tumor_G3
16602	ETFB	6.497214234	Tumor_G3
16603	UQCR10	8.129165812	Tumor_G3
16604	UQCRQ	8.26656897	Tumor_G3
16605	UQCC3	5.709253831	Tumor_G3
16606	COX15	5.232776552	Tumor_G3
16607	COX5A	7.677508121	Tumor_G3
16608	COX6B1	9.246412189	Tumor_G3
16609	COX6C	7.614878594	Tumor_G3
16610	COX7A2	7.149415762	Tumor_G3
16611	COX7B	7.270707147	Tumor_G3
16612	COX8A	10.00118985	Tumor_G3
16613	SCO1	3.478120696	Tumor_G3
16614	SURF1	7.47227826	Tumor_G3
16615	ATP6V1E1	7.157597475	Tumor_G3
16616	DLAT	4.714426498	Tumor_G3
16617	PDC	0	Tumor_G3
16618	SUCLG2	5.412090292	Tumor_G3
16619	SLC25A5	8.894397181	Tumor_G3
16620	UCP2	8.679890827	Tumor_G3
16621	SOD2	4.129168433	Tumor_G3
16622	NOX4	1.156732074	Tumor_G3
16623	DUOX1	7.268684543	Tumor_G3
16624	DUOXA1	6.365551465	Tumor_G3
16625	DUOX2	7.621903491	Tumor_G3
16626	DUOXA2	9.189500478	Tumor_G3
16627	NDUFA1	8.955796341	Tumor_G3
16628	NDUFA2	7.34889247	Tumor_G3
16629	NDUFA8	7.769505652	Tumor_G3
16630	NDUFB1	6.756490404	Tumor_G3
16631	NDUFB10	8.450627469	Tumor_G3
16632	NDUFB2	7.898002013	Tumor_G3
16633	NDUFB3	7.204458689	Tumor_G3
16634	NDUFB7	9.519125179	Tumor_G3
16635	CYC1	7.673030518	Tumor_G3
16636	ETFB	5.928646994	Tumor_G3
16637	UQCR10	9.196277511	Tumor_G3
16638	UQCRQ	8.037869753	Tumor_G3

16639	UQCC3	5.142549453	Tumor_G3
16640	COX15	5.106171352	Tumor_G3
16641	COX5A	7.689596584	Tumor_G3
16642	COX6B1	9.375178154	Tumor_G3
16643	COX6C	7.380977521	Tumor_G3
16644	COX7A2	7.295148463	Tumor_G3
16645	COX7B	7.110334618	Tumor_G3
16646	COX8A	10.35528515	Tumor_G3
16647	SCO1	4.613150759	Tumor_G3
16648	SURF1	7.271262459	Tumor_G3
16649	ATP6V1E1	6.666033916	Tumor_G3
16650	DLAT	5.176533686	Tumor_G3
16651	PDC	0	Tumor_G3
16652	SUCLG2	6.415050962	Tumor_G3
16653	SLC25A5	8.887557601	Tumor_G3
16654	UCP2	6.698995601	Tumor_G3
16655	SOD2	4.875167597	Tumor_G3
16656	NOX4	0.798856243	Tumor_G3
16657	DUOX1	7.252462892	Tumor_G3
16658	DUOXA1	6.807440299	Tumor_G3
16659	DUOX2	7.421349538	Tumor_G3
16660	DUOXA2	7.662449778	Tumor_G3
16661	NDUFA1	9.15408625	Tumor_G3
16662	NDUFA2	8.011242731	Tumor_G3
16663	NDUFA8	8.269637255	Tumor_G3
16664	NDUFB1	7.308415564	Tumor_G3
16665	NDUFB10	8.392142034	Tumor_G3
16666	NDUFB2	7.67453067	Tumor_G3
16667	NDUFB3	7.183231354	Tumor_G3
16668	NDUFB7	9.764215992	Tumor_G3
16669	CYC1	7.353276671	Tumor_G3
16670	ETFB	6.220993611	Tumor_G3
16671	UQCR10	7.841967963	Tumor_G3
16672	UQCRQ	8.745875204	Tumor_G3
16673	UQCC3	5.299106413	Tumor_G3
16674	COX15	4.628069985	Tumor_G3
16675	COX5A	7.977453882	Tumor_G3
16676	COX6B1	10.03653578	Tumor_G3
16677	COX6C	7.799697415	Tumor_G3
16678	COX7A2	7.975276932	Tumor_G3

16679	COX7B	7.363806741	Tumor_G3
16680	COX8A	10.44807726	Tumor_G3
16681	SCO1	2.92891565	Tumor_G3
16682	SURF1	6.871165349	Tumor_G3
16683	ATP6V1E1	6.623259508	Tumor_G3
16684	DLAT	4.543001433	Tumor_G3
16685	PDC	0	Tumor_G3
16686	SUCLG2	6.039703111	Tumor_G3
16687	SLC25A5	7.934249667	Tumor_G3
16688	UCP2	8.597761192	Tumor_G3
16689	SOD2	3.882629718	Tumor_G3
16690	NOX4	2.256444028	Tumor_G3
16691	DUOX1	7.395877183	Tumor_G3
16692	DUOXA1	7.353004655	Tumor_G3
16693	DUOX2	8.606664124	Tumor_G3
16694	DUOXA2	6.69359741	Tumor_G3
16695	NDUFA1	8.996891979	Tumor_G3
16696	NDUFA2	7.693419504	Tumor_G3
16697	NDUFA8	7.778386093	Tumor_G3
16698	NDUFB1	6.959256966	Tumor_G3
16699	NDUFB10	8.242663475	Tumor_G3
16700	NDUFB2	7.847967476	Tumor_G3
16701	NDUFB3	7.147489956	Tumor_G3
16702	NDUFB7	9.691747383	Tumor_G3
16703	CYC1	7.686423136	Tumor_G3
16704	ETFB	5.942151781	Tumor_G3
16705	UQCR10	8.469188121	Tumor_G3
16706	UQCRQ	8.259484145	Tumor_G3
16707	UQCC3	5.228030746	Tumor_G3
16708	COX15	4.51058188	Tumor_G3
16709	COX5A	7.834639502	Tumor_G3
16710	COX6B1	9.687090157	Tumor_G3
16711	COX6C	7.253929937	Tumor_G3
16712	COX7A2	7.670370202	Tumor_G3
16713	COX7B	7.078974338	Tumor_G3
16714	COX8A	10.45453132	Tumor_G3
16715	SCO1	3.370369854	Tumor_G3
16716	SURF1	6.94611117	Tumor_G3
16717	ATP6V1E1	7.181098108	Tumor_G3
16718	DLAT	4.667266992	Tumor_G3

16719	PDC	0.051547103	Tumor_G3
16720	SUCLG2	6.258351772	Tumor_G3
16721	SLC25A5	8.331116431	Tumor_G3
16722	UCP2	7.845773012	Tumor_G3
16723	SOD2	4.569781536	Tumor_G3
16724	NOX4	1.218062492	Tumor_G3
16725	DUOX1	7.697202021	Tumor_G3
16726	DUOXA1	7.794892095	Tumor_G3
16727	DUOX2	8.474190922	Tumor_G3
16728	DUOXA2	7.207764393	Tumor_G3
16729	NDUFA1	8.08561258	Tumor_G3
16730	NDUFA2	7.775866769	Tumor_G3
16731	NDUFA8	6.864897778	Tumor_G3
16732	NDUFB1	6.593018598	Tumor_G3
16733	NDUFB10	9.140695963	Tumor_G3
16734	NDUFB2	7.432782152	Tumor_G3
16735	NDUFB3	5.182939736	Tumor_G3
16736	NDUFB7	10.46540893	Tumor_G3
16737	CYC1	6.711938914	Tumor_G3
16738	ETFB	6.817520347	Tumor_G3
16739	UQCR10	8.458893486	Tumor_G3
16740	UQCRQ	8.921945563	Tumor_G3
16741	UQCC3	6.314735307	Tumor_G3
16742	COX15	3.797118155	Tumor_G3
16743	COX5A	7.237145775	Tumor_G3
16744	COX6B1	10.21546936	Tumor_G3
16745	COX6C	6.9719734	Tumor_G3
16746	COX7A2	6.965552992	Tumor_G3
16747	COX7B	5.649916643	Tumor_G3
16748	COX8A	9.659606292	Tumor_G3
16749	SCO1	2.01812718	Tumor_G3
16750	SURF1	6.621332752	Tumor_G3
16751	ATP6V1E1	6.272879092	Tumor_G3
16752	DLAT	2.534253496	Tumor_G3
16753	PDC	0	Tumor_G3
16754	SUCLG2	5.141749085	Tumor_G3
16755	SLC25A5	6.615254981	Tumor_G3
16756	UCP2	7.217011703	Tumor_G3
16757	SOD2	4.929895551	Tumor_G3
16758	NOX4	2.299974556	Tumor_G3

16759	DUOX1	3.508664842	Tumor_G3
16760	DUOXA1	5.079602703	Tumor_G3
16761	DUOX2	1.870090443	Tumor_G3
16762	DUOXA2	2.086118431	Tumor_G3
16763	NDUFA1	8.076872773	Tumor_G3
16764	NDUFA2	7.236037446	Tumor_G3
16765	NDUFA8	7.386366001	Tumor_G3
16766	NDUFB1	5.767916547	Tumor_G3
16767	NDUFB10	8.184570677	Tumor_G3
16768	NDUFB2	6.907461437	Tumor_G3
16769	NDUFB3	6.420034311	Tumor_G3
16770	NDUFB7	9.43291971	Tumor_G3
16771	CYC1	7.18039454	Tumor_G3
16772	ETFB	6.215597688	Tumor_G3
16773	UQCR10	7.96080999	Tumor_G3
16774	UQCRQ	7.848194967	Tumor_G3
16775	UQCC3	5.301581804	Tumor_G3
16776	COX15	4.43597754	Tumor_G3
16777	COX5A	7.130901927	Tumor_G3
16778	COX6B1	9.222526585	Tumor_G3
16779	COX6C	6.526127254	Tumor_G3
16780	COX7A2	7.418776684	Tumor_G3
16781	COX7B	6.272115339	Tumor_G3
16782	COX8A	10.20496102	Tumor_G3
16783	SCO1	2.993994462	Tumor_G3
16784	SURF1	6.959226131	Tumor_G3
16785	ATP6V1E1	7.100190337	Tumor_G3
16786	DLAT	4.194726466	Tumor_G3
16787	PDC	0	Tumor_G3
16788	SUCLG2	5.462368606	Tumor_G3
16789	SLC25A5	8.005618819	Tumor_G3
16790	UCP2	9.731851926	Tumor_G3
16791	SOD2	4.690517753	Tumor_G3
16792	NOX4	2.968088965	Tumor_G3
16793	DUOX1	6.544096615	Tumor_G3
16794	DUOXA1	6.959579233	Tumor_G3
16795	DUOX2	6.795108704	Tumor_G3
16796	DUOXA2	5.062387278	Tumor_G3
16797	NDUFA1	8.743358795	Tumor_G3
16798	NDUFA2	8.005225487	Tumor_G3

16799	NDUFA8	7.58399764	Tumor_G3
16800	NDUFB1	6.069761844	Tumor_G3
16801	NDUFB10	7.819311567	Tumor_G3
16802	NDUFB2	7.135493369	Tumor_G3
16803	NDUFB3	6.634645711	Tumor_G3
16804	NDUFB7	9.378502392	Tumor_G3
16805	CYC1	7.298640492	Tumor_G3
16806	ETFB	6.838938658	Tumor_G3
16807	UQCR10	8.726038134	Tumor_G3
16808	UQCRQ	8.317844283	Tumor_G3
16809	UQCC3	4.155523292	Tumor_G3
16810	COX15	5.103790932	Tumor_G3
16811	COX5A	6.973773486	Tumor_G3
16812	COX6B1	9.930985518	Tumor_G3
16813	COX6C	7.094469306	Tumor_G3
16814	COX7A2	7.612580842	Tumor_G3
16815	COX7B	6.114167437	Tumor_G3
16816	COX8A	9.263261474	Tumor_G3
16817	SCO1	3.759413788	Tumor_G3
16818	SURF1	6.98127358	Tumor_G3
16819	ATP6V1E1	6.46448173	Tumor_G3
16820	DLAT	4.074650041	Tumor_G3
16821	PDC	0.203365903	Tumor_G3
16822	SUCLG2	6.093209508	Tumor_G3
16823	SLC25A5	7.867884185	Tumor_G3
16824	UCP2	6.76801991	Tumor_G3
16825	SOD2	3.498652958	Tumor_G3
16826	NOX4	1.35772657	Tumor_G3
16827	DUOX1	5.432390612	Tumor_G3
16828	DUOXA1	5.251096003	Tumor_G3
16829	DUOX2	5.527481047	Tumor_G3
16830	DUOXA2	4.378631063	Tumor_G3
16831	NDUFA1	8.672752447	Tumor_G3
16832	NDUFA2	7.541178904	Tumor_G3
16833	NDUFA8	7.708515508	Tumor_G3
16834	NDUFB1	6.571532444	Tumor_G3
16835	NDUFB10	7.92924286	Tumor_G3
16836	NDUFB2	7.39179056	Tumor_G3
16837	NDUFB3	6.863418261	Tumor_G3
16838	NDUFB7	9.387513337	Tumor_G3

16839	CYC1	7.212377913	Tumor_G3
16840	ETFB	5.436569422	Tumor_G3
16841	UQCR10	8.282597852	Tumor_G3
16842	UQCRQ	8.087001319	Tumor_G3
16843	UQCC3	4.823545909	Tumor_G3
16844	COX15	4.697910465	Tumor_G3
16845	COX5A	7.129953806	Tumor_G3
16846	COX6B1	9.248498434	Tumor_G3
16847	COX6C	7.106196355	Tumor_G3
16848	COX7A2	7.401199314	Tumor_G3
16849	COX7B	6.641382726	Tumor_G3
16850	COX8A	9.526349625	Tumor_G3
16851	SCO1	3.161991283	Tumor_G3
16852	SURF1	6.551725355	Tumor_G3
16853	ATP6V1E1	7.044366123	Tumor_G3
16854	DLAT	4.850483059	Tumor_G3
16855	PDC	0.100726444	Tumor_G3
16856	SUCLG2	5.934256385	Tumor_G3
16857	SLC25A5	7.711485593	Tumor_G3
16858	UCP2	7.203815443	Tumor_G3
16859	SOD2	4.565635558	Tumor_G3
16860	NOX4	1.971815351	Tumor_G3
16861	DUOX1	7.858401047	Tumor_G3
16862	DUOXA1	7.638420021	Tumor_G3
16863	DUOX2	8.860771961	Tumor_G3
16864	DUOXA2	7.23730975	Tumor_G3
16865	NDUFA1	9.421088101	Tumor_G3
16866	NDUFA2	7.885814489	Tumor_G3
16867	NDUFA8	8.113130853	Tumor_G3
16868	NDUFB1	6.851981663	Tumor_G3
16869	NDUFB10	8.195342439	Tumor_G3
16870	NDUFB2	8.357260993	Tumor_G3
16871	NDUFB3	7.525144003	Tumor_G3
16872	NDUFB7	9.764065153	Tumor_G3
16873	CYC1	7.633834838	Tumor_G3
16874	ETFB	6.393266675	Tumor_G3
16875	UQCR10	9.016709172	Tumor_G3
16876	UQCRQ	8.3084212	Tumor_G3
16877	UQCC3	4.394016255	Tumor_G3
16878	COX15	5.214293232	Tumor_G3

16879	COX5A	7.813311721	Tumor_G3
16880	COX6B1	9.724394906	Tumor_G3
16881	COX6C	7.502485738	Tumor_G3
16882	COX7A2	7.76815772	Tumor_G3
16883	COX7B	7.095572595	Tumor_G3
16884	COX8A	10.42773764	Tumor_G3
16885	SCO1	3.970176175	Tumor_G3
16886	SURF1	7.514931092	Tumor_G3
16887	ATP6V1E1	7.612264934	Tumor_G3
16888	DLAT	4.95071776	Tumor_G3
16889	PDC	0.049235462	Tumor_G3
16890	SUCLG2	6.361178645	Tumor_G3
16891	SLC25A5	8.59161387	Tumor_G3
16892	UCP2	8.049331608	Tumor_G3
16893	SOD2	4.144994286	Tumor_G3
16894	NOX4	0.580782619	Tumor_G3
16895	DUOX1	6.962825161	Tumor_G3
16896	DUOXA1	6.608713419	Tumor_G3
16897	DUOX2	7.104940139	Tumor_G3
16898	DUOXA2	5.628459407	Tumor_G3
16899	NDUFA1	8.70681749	Tumor_G3
16900	NDUFA2	7.786667073	Tumor_G3
16901	NDUFA8	7.66652915	Tumor_G3
16902	NDUFB1	6.487050552	Tumor_G3
16903	NDUFB10	8.338116216	Tumor_G3
16904	NDUFB2	6.692158628	Tumor_G3
16905	NDUFB3	6.101045771	Tumor_G3
16906	NDUFB7	9.89481994	Tumor_G3
16907	CYC1	7.06306192	Tumor_G3
16908	ETFB	5.641159179	Tumor_G3
16909	UQCR10	8.272860663	Tumor_G3
16910	UQCRQ	7.915534226	Tumor_G3
16911	UQCC3	5.676715849	Tumor_G3
16912	COX15	4.364820372	Tumor_G3
16913	COX5A	7.529754526	Tumor_G3
16914	COX6B1	9.794968895	Tumor_G3
16915	COX6C	6.970958874	Tumor_G3
16916	COX7A2	7.436090133	Tumor_G3
16917	COX7B	6.542749178	Tumor_G3
16918	COX8A	10.31373778	Tumor_G3

16919	SCO1	2.741568774	Tumor_G3
16920	SURF1	7.095998332	Tumor_G3
16921	ATP6V1E1	7.024937317	Tumor_G3
16922	DLAT	3.968844096	Tumor_G3
16923	PDC	0.227517681	Tumor_G3
16924	SUCLG2	5.428738104	Tumor_G3
16925	SLC25A5	6.900995765	Tumor_G3
16926	UCP2	9.270313966	Tumor_G3
16927	SOD2	3.895905362	Tumor_G3
16928	NOX4	1.733911265	Tumor_G3
16929	DUOX1	5.617167512	Tumor_G3
16930	DUOXA1	6.466521657	Tumor_G3
16931	DUOX2	6.54222021	Tumor_G3
16932	DUOXA2	5.997029673	Tumor_G3
16933	NDUFA1	8.749520228	Tumor_G3
16934	NDUFA2	7.248098295	Tumor_G3
16935	NDUFA8	7.699552184	Tumor_G3
16936	NDUFB1	6.351653879	Tumor_G3
16937	NDUFB10	7.918987295	Tumor_G3
16938	NDUFB2	7.549905968	Tumor_G3
16939	NDUFB3	7.19506048	Tumor_G3
16940	NDUFB7	8.213671129	Tumor_G3
16941	CYC1	7.473621167	Tumor_G3
16942	ETFB	5.245381342	Tumor_G3
16943	UQCR10	8.649165266	Tumor_G3
16944	UQCRQ	7.890454531	Tumor_G3
16945	UQCC3	4.240676451	Tumor_G3
16946	COX15	5.681653902	Tumor_G3
16947	COX5A	7.89728222	Tumor_G3
16948	COX6B1	8.878084863	Tumor_G3
16949	COX6C	7.197379665	Tumor_G3
16950	COX7A2	7.397900226	Tumor_G3
16951	COX7B	7.079113188	Tumor_G3
16952	COX8A	10.24762237	Tumor_G3
16953	SCO1	4.146749516	Tumor_G3
16954	SURF1	7.171144345	Tumor_G3
16955	ATP6V1E1	7.13162678	Tumor_G3
16956	DLAT	6.113304167	Tumor_G3
16957	PDC	0.333734161	Tumor_G3
16958	SUCLG2	7.179039152	Tumor_G3

16959	SLC25A5	8.424983708	Tumor_G3
16960	UCP2	8.407044222	Tumor_G3
16961	SOD2	5.214647214	Tumor_G3
16962	NOX4	0.919788973	Tumor_G3
16963	DUOX1	7.56758407	Tumor_G3
16964	DUOXA1	7.111300677	Tumor_G3
16965	DUOX2	8.48244911	Tumor_G3
16966	DUOXA2	6.021691857	Tumor_G3
16967	NDUFA1	8.99346138	Tumor_G3
16968	NDUFA2	7.303850895	Tumor_G3
16969	NDUFA8	7.736752423	Tumor_G3
16970	NDUFB1	7.005005508	Tumor_G3
16971	NDUFB10	8.120558234	Tumor_G3
16972	NDUFB2	7.804429215	Tumor_G3
16973	NDUFB3	7.246862207	Tumor_G3
16974	NDUFB7	9.116594237	Tumor_G3
16975	CYC1	8.038810973	Tumor_G3
16976	ETFB	5.482459208	Tumor_G3
16977	UQCR10	8.325973116	Tumor_G3
16978	UQCRQ	8.381661186	Tumor_G3
16979	UQCC3	4.36633255	Tumor_G3
16980	COX15	5.723152859	Tumor_G3
16981	COX5A	7.957283005	Tumor_G3
16982	COX6B1	8.880439328	Tumor_G3
16983	COX6C	7.262858634	Tumor_G3
16984	COX7A2	7.3050388	Tumor_G3
16985	COX7B	7.233468079	Tumor_G3
16986	COX8A	10.7401997	Tumor_G3
16987	SCO1	4.129451135	Tumor_G3
16988	SURF1	6.902919654	Tumor_G3
16989	ATP6V1E1	6.223697886	Tumor_G3
16990	DLAT	5.907002927	Tumor_G3
16991	PDC	0.039629468	Tumor_G3
16992	SUCLG2	7.240673411	Tumor_G3
16993	SLC25A5	8.633191863	Tumor_G3
16994	UCP2	4.882690852	Tumor_G3
16995	SOD2	5.630866048	Tumor_G3
16996	NOX4	1.690053663	Tumor_G3
16997	DUOX1	7.362874361	Tumor_G3
16998	DUOXA1	6.275115567	Tumor_G3

16999	DUOX2	7.712355636	Tumor_G3
17000	DUOXA2	6.899121308	Tumor_G3
17001	NDUFA1	8.506370007	Tumor_G3
17002	NDUFA2	7.287339925	Tumor_G3
17003	NDUFA8	7.604887532	Tumor_G3
17004	NDUFB1	6.476357268	Tumor_G3
17005	NDUFB10	8.346569666	Tumor_G3
17006	NDUFB2	7.696026668	Tumor_G3
17007	NDUFB3	7.074829311	Tumor_G3
17008	NDUFB7	9.494916894	Tumor_G3
17009	CYC1	7.669515913	Tumor_G3
17010	ETFB	6.102175548	Tumor_G3
17011	UQCR10	8.260870911	Tumor_G3
17012	UQCRQ	8.177444834	Tumor_G3
17013	UQCC3	5.000205428	Tumor_G3
17014	COX15	5.592217988	Tumor_G3
17015	COX5A	7.568489858	Tumor_G3
17016	COX6B1	8.96252118	Tumor_G3
17017	COX6C	7.233728051	Tumor_G3
17018	COX7A2	7.287019331	Tumor_G3
17019	COX7B	6.524325029	Tumor_G3
17020	COX8A	10.54924092	Tumor_G3
17021	SCO1	4.258518341	Tumor_G3
17022	SURF1	7.194158628	Tumor_G3
17023	ATP6V1E1	7.065032682	Tumor_G3
17024	DLAT	5.536161959	Tumor_G3
17025	PDC	0.10397665	Tumor_G3
17026	SUCLG2	7.203185992	Tumor_G3
17027	SLC25A5	8.421749329	Tumor_G3
17028	UCP2	8.290546835	Tumor_G3
17029	SOD2	4.57680417	Tumor_G3
17030	NOX4	0.791161914	Tumor_G3
17031	DUOX1	7.853995198	Tumor_G3
17032	DUOXA1	7.328823184	Tumor_G3
17033	DUOX2	9.742489288	Tumor_G3
17034	DUOXA2	7.553886018	Tumor_G3
17035	NDUFA1	9.040227798	Tumor_G3
17036	NDUFA2	7.287857814	Tumor_G3
17037	NDUFA8	7.787621051	Tumor_G3
17038	NDUFB1	6.698013826	Tumor_G3

17039	NDUFB10	7.850117311	Tumor_G3
17040	NDUFB2	7.596142751	Tumor_G3
17041	NDUFB3	7.519353567	Tumor_G3
17042	NDUFB7	8.198456386	Tumor_G3
17043	CYC1	8.38986095	Tumor_G3
17044	ETFB	5.800481289	Tumor_G3
17045	UQCR10	8.881817851	Tumor_G3
17046	UQCRQ	8.209184152	Tumor_G3
17047	UQCC3	4.671838069	Tumor_G3
17048	COX15	6.016003546	Tumor_G3
17049	COX5A	8.267980786	Tumor_G3
17050	COX6B1	9.665187362	Tumor_G3
17051	COX6C	7.433147237	Tumor_G3
17052	COX7A2	7.009284937	Tumor_G3
17053	COX7B	7.596600669	Tumor_G3
17054	COX8A	10.79988638	Tumor_G3
17055	SCO1	4.068366349	Tumor_G3
17056	SURF1	6.191232242	Tumor_G3
17057	ATP6V1E1	6.993858171	Tumor_G3
17058	DLAT	6.017878868	Tumor_G3
17059	PDC	0.270698135	Tumor_G3
17060	SUCLG2	6.828958298	Tumor_G3
17061	SLC25A5	8.921141715	Tumor_G3
17062	UCP2	7.667586314	Tumor_G3
17063	SOD2	4.860138673	Tumor_G3
17064	NOX4	1.832440681	Tumor_G3
17065	DUOX1	8.233664703	Tumor_G3
17066	DUOXA1	7.771975214	Tumor_G3
17067	DUOX2	8.096588435	Tumor_G3
17068	DUOXA2	5.978565266	Tumor_G3
17069	NDUFA1	8.36159864	Tumor_G3
17070	NDUFA2	7.529435788	Tumor_G3
17071	NDUFA8	7.554845961	Tumor_G3
17072	NDUFB1	6.402141617	Tumor_G3
17073	NDUFB10	7.979183471	Tumor_G3
17074	NDUFB2	7.46341333	Tumor_G3
17075	NDUFB3	6.897552178	Tumor_G3
17076	NDUFB7	9.451515472	Tumor_G3
17077	CYC1	7.31903783	Tumor_G3
17078	ETFB	5.616669102	Tumor_G3

17079	UQCR10	8.374374653	Tumor_G3
17080	UQCRQ	7.863653926	Tumor_G3
17081	UQCC3	5.310291595	Tumor_G3
17082	COX15	4.798765273	Tumor_G3
17083	COX5A	7.391946671	Tumor_G3
17084	COX6B1	9.233903736	Tumor_G3
17085	COX6C	6.96653095	Tumor_G3
17086	COX7A2	7.226137148	Tumor_G3
17087	COX7B	6.669679919	Tumor_G3
17088	COX8A	10.18705973	Tumor_G3
17089	SCO1	3.441626691	Tumor_G3
17090	SURF1	6.921430287	Tumor_G3
17091	ATP6V1E1	6.433390106	Tumor_G3
17092	DLAT	5.140634027	Tumor_G3
17093	PDC	0.325012748	Tumor_G3
17094	SUCLG2	6.25626501	Tumor_G3
17095	SLC25A5	8.061706686	Tumor_G3
17096	UCP2	7.98562303	Tumor_G3
17097	SOD2	4.679687365	Tumor_G3
17098	NOX4	1.19684266	Tumor_G3
17099	DUOX1	7.266581804	Tumor_G3
17100	DUOXA1	7.17783449	Tumor_G3
17101	DUOX2	8.93491205	Tumor_G3
17102	DUOXA2	7.946707011	Tumor_G3
17103	NDUFA1	8.900522468	Tumor_G3
17104	NDUFA2	7.374684645	Tumor_G3
17105	NDUFA8	7.788260836	Tumor_G3
17106	NDUFB1	6.568043913	Tumor_G3
17107	NDUFB10	8.19370893	Tumor_G3
17108	NDUFB2	8.031285612	Tumor_G3
17109	NDUFB3	7.085056066	Tumor_G3
17110	NDUFB7	9.314584152	Tumor_G3
17111	CYC1	8.092170034	Tumor_G3
17112	ETFB	5.777227695	Tumor_G3
17113	UQCR10	8.02140719	Tumor_G3
17114	UQCRQ	8.001072026	Tumor_G3
17115	UQCC3	4.956310268	Tumor_G3
17116	COX15	5.33390958	Tumor_G3
17117	COX5A	8.33577321	Tumor_G3
17118	COX6B1	9.259714067	Tumor_G3

17119	COX6C	7.328362913	Tumor_G3
17120	COX7A2	7.883691464	Tumor_G3
17121	COX7B	7.035248518	Tumor_G3
17122	COX8A	10.6743287	Tumor_G3
17123	SCO1	3.633795242	Tumor_G3
17124	SURF1	6.99455387	Tumor_G3
17125	ATP6V1E1	7.582877306	Tumor_G3
17126	DLAT	5.797214954	Tumor_G3
17127	PDC	0	Tumor_G3
17128	SUCLG2	7.305962668	Tumor_G3
17129	SLC25A5	9.837524588	Tumor_G3
17130	UCP2	7.275578458	Tumor_G3
17131	SOD2	5.049562844	Tumor_G3
17132	NOX4	0.737008606	Tumor_G3
17133	DUOX1	6.092055168	Tumor_G3
17134	DUOXA1	6.505625564	Tumor_G3
17135	DUOX2	7.857853687	Tumor_G3
17136	DUOXA2	5.776538246	Tumor_G3
17137	NDUFA1	9.134321493	Tumor_G3
17138	NDUFA2	8.36931632	Tumor_G3
17139	NDUFA8	7.637147222	Tumor_G3
17140	NDUFB1	7.680132432	Tumor_G3
17141	NDUFB10	9.470096346	Tumor_G3
17142	NDUFB2	8.526034759	Tumor_G3
17143	NDUFB3	5.84763259	Tumor_G3
17144	NDUFB7	11.01848272	Tumor_G3
17145	CYC1	7.143737131	Tumor_G3
17146	ETFB	7.517035377	Tumor_G3
17147	UQCR10	8.97404349	Tumor_G3
17148	UQCRQ	9.02214234	Tumor_G3
17149	UQCC3	6.970520983	Tumor_G3
17150	COX15	4.668817945	Tumor_G3
17151	COX5A	7.799922974	Tumor_G3
17152	COX6B1	10.66061094	Tumor_G3
17153	COX6C	7.543734149	Tumor_G3
17154	COX7A2	7.44590605	Tumor_G3
17155	COX7B	6.554659892	Tumor_G3
17156	COX8A	10.21029739	Tumor_G3
17157	SCO1	2.590155219	Tumor_G3
17158	SURF1	7.738759895	Tumor_G3

17159	ATP6V1E1	7.171874367	Tumor_G3
17160	DLAT	3.626348123	Tumor_G3
17161	PDC	0.193358213	Tumor_G3
17162	SUCLG2	5.8754342	Tumor_G3
17163	SLC25A5	7.017970324	Tumor_G3
17164	UCP2	7.040113881	Tumor_G3
17165	SOD2	3.779294183	Tumor_G3
17166	NOX4	1.346898679	Tumor_G3
17167	DUOX1	5.054701526	Tumor_G3
17168	DUOXA1	6.139416619	Tumor_G3
17169	DUOX2	4.619445227	Tumor_G3
17170	DUOXA2	4.237126406	Tumor_G3
17171	NDUFA1	8.399137839	Tumor_G3
17172	NDUFA2	7.288803707	Tumor_G3
17173	NDUFA8	7.379577071	Tumor_G3
17174	NDUFB1	6.190312629	Tumor_G3
17175	NDUFB10	8.145806626	Tumor_G3
17176	NDUFB2	7.309774006	Tumor_G3
17177	NDUFB3	6.504293195	Tumor_G3
17178	NDUFB7	9.46887702	Tumor_G3
17179	CYC1	7.246047592	Tumor_G3
17180	ETFB	5.638334257	Tumor_G3
17181	UQCR10	8.19317812	Tumor_G3
17182	UQCRQ	7.837958423	Tumor_G3
17183	UQCC3	4.960380198	Tumor_G3
17184	COX15	4.471865832	Tumor_G3
17185	COX5A	7.572950715	Tumor_G3
17186	COX6B1	9.428825739	Tumor_G3
17187	COX6C	6.931495118	Tumor_G3
17188	COX7A2	7.281807482	Tumor_G3
17189	COX7B	6.289590484	Tumor_G3
17190	COX8A	9.853640288	Tumor_G3
17191	SCO1	2.790987274	Tumor_G3
17192	SURF1	6.984641741	Tumor_G3
17193	ATP6V1E1	7.291409361	Tumor_G3
17194	DLAT	4.240738345	Tumor_G3
17195	PDC	0	Tumor_G3
17196	SUCLG2	5.87119353	Tumor_G3
17197	SLC25A5	7.959433066	Tumor_G3
17198	UCP2	8.610729977	Tumor_G3

17199	SOD2	4.971395974	Tumor_G3
17200	NOX4	2.725549758	Tumor_G3
17201	DUOX1	6.406850521	Tumor_G3
17202	DUOXA1	6.729597625	Tumor_G3
17203	DUOX2	5.967574296	Tumor_G3
17204	DUOXA2	5.151436101	Tumor_G3
17205	NDUFA1	9.457321502	Tumor_G3
17206	NDUFA2	7.983042056	Tumor_G3
17207	NDUFA8	7.806097521	Tumor_G3
17208	NDUFB1	7.341702205	Tumor_G3
17209	NDUFB10	8.052173247	Tumor_G3
17210	NDUFB2	6.85834686	Tumor_G3
17211	NDUFB3	6.292277666	Tumor_G3
17212	NDUFB7	9.464969083	Tumor_G3
17213	CYC1	7.446447928	Tumor_G3
17214	ETFB	5.899333536	Tumor_G3
17215	UQCR10	8.63128929	Tumor_G3
17216	UQCRQ	8.314732101	Tumor_G3
17217	UQCC3	4.721430345	Tumor_G3
17218	COX15	4.574293023	Tumor_G3
17219	COX5A	8.020303964	Tumor_G3
17220	COX6B1	9.604203756	Tumor_G3
17221	COX6C	7.878766807	Tumor_G3
17222	COX7A2	7.427497201	Tumor_G3
17223	COX7B	7.322292002	Tumor_G3
17224	COX8A	10.0799743	Tumor_G3
17225	SCO1	2.552981636	Tumor_G3
17226	SURF1	6.78934419	Tumor_G3
17227	ATP6V1E1	6.250606879	Tumor_G3
17228	DLAT	4.585389781	Tumor_G3
17229	PDC	0	Tumor_G3
17230	SUCLG2	5.66090801	Tumor_G3
17231	SLC25A5	6.664543517	Tumor_G3
17232	UCP2	9.210808485	Tumor_G3
17233	SOD2	3.735379728	Tumor_G3
17234	NOX4	1.04567906	Tumor_G3
17235	DUOX1	6.881754668	Tumor_G3
17236	DUOXA1	5.920346833	Tumor_G3
17237	DUOX2	7.556806298	Tumor_G3
17238	DUOXA2	8.675074883	Tumor_G3

17239	NDUFA1	8.621441533	Tumor_G3
17240	NDUFA2	7.793367145	Tumor_G3
17241	NDUFA8	7.721176944	Tumor_G3
17242	NDUFB1	6.634887404	Tumor_G3
17243	NDUFB10	8.434435903	Tumor_G3
17244	NDUFB2	6.212367088	Tumor_G3
17245	NDUFB3	5.755422162	Tumor_G3
17246	NDUFB7	9.784518881	Tumor_G3
17247	CYC1	6.913462099	Tumor_G3
17248	ETFB	5.49292669	Tumor_G3
17249	UQCR10	8.286204018	Tumor_G3
17250	UQCRQ	7.854548098	Tumor_G3
17251	UQCC3	5.535590085	Tumor_G3
17252	COX15	4.34456804	Tumor_G3
17253	COX5A	7.414249317	Tumor_G3
17254	COX6B1	9.741765208	Tumor_G3
17255	COX6C	7.211561454	Tumor_G3
17256	COX7A2	7.091441568	Tumor_G3
17257	COX7B	6.302017158	Tumor_G3
17258	COX8A	9.587010953	Tumor_G3
17259	SCO1	2.582134998	Tumor_G3
17260	SURF1	6.851222393	Tumor_G3
17261	ATP6V1E1	6.507119205	Tumor_G3
17262	DLAT	3.922377627	Tumor_G3
17263	PDC	0	Tumor_G3
17264	SUCLG2	4.66711963	Tumor_G3
17265	SLC25A5	6.307895763	Tumor_G3
17266	UCP2	10.02988868	Tumor_G3
17267	SOD2	3.853137725	Tumor_G3
17268	NOX4	2.483611102	Tumor_G3
17269	DUOX1	5.965487922	Tumor_G3
17270	DUOXA1	6.831212128	Tumor_G3
17271	DUOX2	6.73735988	Tumor_G3
17272	DUOXA2	5.756880612	Tumor_G3
17273	NDUFA1	8.795603144	Tumor_G3
17274	NDUFA2	7.61236437	Tumor_G3
17275	NDUFA8	7.722874594	Tumor_G3
17276	NDUFB1	6.552217326	Tumor_G3
17277	NDUFB10	8.384704778	Tumor_G3
17278	NDUFB2	6.819071369	Tumor_G3

17279	NDUFB3	6.465644904	Tumor_G3
17280	NDUFB7	9.777728909	Tumor_G3
17281	CYC1	7.108809367	Tumor_G3
17282	ETFB	5.735279267	Tumor_G3
17283	UQCR10	8.301763305	Tumor_G3
17284	UQCRQ	8.124621987	Tumor_G3
17285	UQCC3	5.320735876	Tumor_G3
17286	COX15	4.248273466	Tumor_G3
17287	COX5A	7.377390894	Tumor_G3
17288	COX6B1	9.776811779	Tumor_G3
17289	COX6C	7.21438183	Tumor_G3
17290	COX7A2	7.305184597	Tumor_G3
17291	COX7B	6.569340472	Tumor_G3
17292	COX8A	9.681848075	Tumor_G3
17293	SCO1	2.585227618	Tumor_G3
17294	SURF1	6.798991214	Tumor_G3
17295	ATP6V1E1	6.777848619	Tumor_G3
17296	DLAT	4.255355754	Tumor_G3
17297	PDC	0	Tumor_G3
17298	SUCLG2	5.300834893	Tumor_G3
17299	SLC25A5	7.15004538	Tumor_G3
17300	UCP2	9.143736679	Tumor_G3
17301	SOD2	4.080410873	Tumor_G3
17302	NOX4	3.034507301	Tumor_G3
17303	DUOX1	6.080842391	Tumor_G3
17304	DUOXA1	6.679932523	Tumor_G3
17305	DUOX2	6.358586383	Tumor_G3
17306	DUOXA2	5.636789801	Tumor_G3
17307	NDUFA1	8.802653949	Tumor_G3
17308	NDUFA2	7.32418053	Tumor_G3
17309	NDUFA8	7.582816476	Tumor_G3
17310	NDUFB1	6.718212627	Tumor_G3
17311	NDUFB10	8.041693382	Tumor_G3
17312	NDUFB2	7.261859879	Tumor_G3
17313	NDUFB3	6.670215366	Tumor_G3
17314	NDUFB7	9.263823553	Tumor_G3
17315	CYC1	7.324581156	Tumor_G3
17316	ETFB	5.426705503	Tumor_G3
17317	UQCR10	7.421414162	Tumor_G3
17318	UQCRQ	8.138700474	Tumor_G3

17319	UQCC3	4.459158364	Tumor_G3
17320	COX15	4.690855959	Tumor_G3
17321	COX5A	7.675114157	Tumor_G3
17322	COX6B1	9.141727243	Tumor_G3
17323	COX6C	7.271712248	Tumor_G3
17324	COX7A2	7.396565855	Tumor_G3
17325	COX7B	6.723113067	Tumor_G3
17326	COX8A	9.99735174	Tumor_G3
17327	SCO1	3.181287535	Tumor_G3
17328	SURF1	6.858746166	Tumor_G3
17329	ATP6V1E1	6.031769784	Tumor_G3
17330	DLAT	4.71488752	Tumor_G3
17331	PDC	0.205725016	Tumor_G3
17332	SUCLG2	6.363071196	Tumor_G3
17333	SLC25A5	7.743147055	Tumor_G3
17334	UCP2	8.187848421	Tumor_G3
17335	SOD2	4.042604393	Tumor_G3
17336	NOX4	2.195262383	Tumor_G3
17337	DUOX1	8.598742182	Tumor_G3
17338	DUOXA1	8.225281266	Tumor_G3
17339	DUOX2	9.266641748	Tumor_G3
17340	DUOXA2	7.001645035	Tumor_G3
