# Supplementary material for: Clinical Relevance and Tumor Growth Suppression of Mitochondrial ROS Regulators along NADH:Ubiquinone Oxidoreductase Subunit B3 in Thyroid Cancer
Source: Oxid Med Cell Longev. 2022 Jan 17;2022:8038857. doi: 10.1155/2022/8038857 (PMC8787455; doi:10.1155/2022/8038857)
Supplement: Supplementary 2 — Supplementary Table 2: a table showing primer sequences and production lengths of human complex I subunit genes and MnSOD. Supplementary Table 3: a table showing the names and corresponding reference PubMed IDs of 35 mitoROS regulators. Supplementary Table 1: a table showing the cluster to which each TCGA sample ID belongs to the postconsensus clustering of 510 diagnosed thyroid cancer patients. Supplementary Table 4: a table showing the detailed clinical information of each TCGA sample within each cluster. Supplementary Table 5: a table showing the PFS duration of each TCGA sample within each cluster. Supplementary Table 6: a table showing the effect of high (50%) or low (50%) expression of each of the 35 mitoSOX regulators on PFS of 510 thyroid cancer patients in TCGA cohort. Supplementary Table 7: a table showing the exact expression value of each of the 35 mitoROS regulators for 510 TCGA samples. Supplementary Table 8: a table showing the detailed clinical information of each of 510 thyroid cancer samples divided into NDUFB3high and NDUFB3low. Supplementary Table 9: a table showing the log2FC, average expression values, and statistical parameters of 16961 genes based on the comparison of NDUFB3low (25%, n = 127) with NDFFB3high (25%, n = 127). Supplementary Table 10: a table showing statistical parameters of each of the 18 enriched KEGG pathways based on the comparison of NDUFB3low with NDFFB3high. Supplementary Table 11: a table showing the score of each of the 80 genes in the OXPHOS pathway based on the comparison of NDUFB3low with NDFFB3high. [file 8038857.f2.zip › Supplementary table 9.pdf]

Name	logFC	AveExpr	t	P.Value	adj.P.Val	B
NDUFB3	-1.09136663	6.31234456	-29.20897714	1.64E-83	2.79E-79	179.3917534
ATP5PF	-0.963773616	6.77208906	-22.124294	2.24E-61	1.90E-57	129.0455613
NDUFB6	-0.914182236	6.20621312	-21.81579598	2.36E-60	1.34E-56	126.7139035
SUCLG1	-0.974284443	6.07562036	-21.10485038	5.58E-58	2.37E-54	121.3012767
BOLA3	-0.947246301	3.47380511	-20.97908898	1.48E-57	4.38E-54	120.3383077
NDUFA1:	-0.818722892	6.05785056	-20.97283888	1.55E-57	4.38E-54	120.2904078
COX7A2	-0.836530884	6.91643563	-20.07278229	1.70E-54	4.13E-51	113.3524598
MORN2	-1.107969433	5.42428643	-19.93260777	5.10E-54	1.08E-50	112.265089
TMEM14	-1.069690474	5.49308101	-19.66561744	4.15E-53	7.81E-50	110.1891682
GCSH	-1.493511156	3.96125735	-19.34202293	5.29E-52	8.98E-49	107.6650168
NDUFB2	-1.188724003	6.71950771	-19.27872634	8.72E-52	1.34E-48	107.1702806
ATP5MC	-0.941702637	5.92033624	-18.58391115	2.13E-49	3.01E-46	101.7195076
NDUFA8	-0.784984764	7.1817636	-18.2782545	2.42E-48	3.15E-45	99.31099672
GLRX5	-0.796341062	5.38719004	-18.22521693	3.69E-48	4.47E-45	98.89247066
UQCRH	-0.825315609	7.22489535	-18.19284094	4.77E-48	5.39E-45	98.63690393
ANAPC1	-0.909031716	3.95405601	-18.16854505	5.79E-48	6.09E-45	98.44507789
ATP5F1C	-0.768771878	7.60941026	-18.16179173	6.11E-48	6.09E-45	98.39175143
MRPS33	-0.960598112	4.53199966	-18.14327166	7.08E-48	6.67E-45	98.245497
ATP5PD	-0.87574813	6.91622254	-18.10025304	9.97E-48	8.72E-45	97.9056979
HSBP1	-0.856228772	5.19954172	-18.09641097	1.03E-47	8.72E-45	97.87534457
LAMTOR	-0.848215696	6.92078986	-17.86861051	6.32E-47	5.11E-44	96.07417912
PARK7	-0.853357009	8.18760805	-17.74088547	1.75E-46	1.35E-43	95.06306647
C15orf61	-0.820938598	2.55703268	-17.69821813	2.47E-46	1.77E-43	94.72511314
COX7B	-0.914552186	6.26610868	-17.69595231	2.51E-46	1.77E-43	94.70716386
VDAC3	-0.940531066	6.08461562	-17.65369573	3.52E-46	2.39E-43	94.3723709
RTN4IP1	-0.832798621	3.74934224	-17.63557545	4.07E-46	2.65E-43	94.22878002
BEX3	-1.216666792	8.54301211	-17.61983173	4.61E-46	2.90E-43	94.10400891
ATP5MC	-0.920433142	6.28588401	-17.51504819	1.07E-45	6.31E-43	93.2732898
ATP5MG	-0.7312178	7.06111501	-17.51356767	1.08E-45	6.31E-43	93.26154868
NDUFA1	-0.867912529	8.17262181	-17.49659237	1.24E-45	6.98E-43	93.12692086
MRPS11	-0.856075232	3.89986646	-17.45368106	1.74E-45	9.53E-43	92.78654245
COX8A	-1.056235889	9.58584415	-17.35708781	3.77E-45	2.00E-42	92.02006019
COX6C	-0.895119486	6.60591119	-17.35180601	3.93E-45	2.02E-42	91.97813697
SNAPC5	-0.705466857	4.78932851	-17.28358555	6.79E-45	3.39E-42	91.43654884
RIDA	-0.940415316	4.42619839	-17.26903243	7.63E-45	3.70E-42	91.32099055
POLR3K	-0.972479105	4.60514021	-17.15693578	1.87E-44	8.82E-42	90.43062027
ELAC1	-0.902643923	3.29914876	-16.99746632	6.72E-44	3.08E-41	89.16319344
ALG14	-0.739688935	2.52859803	-16.99199428	7.02E-44	3.13E-41	89.11968769
APOO	-0.9309396	5.12193303	-16.96034036	9.05E-44	3.94E-41	88.86800287

HDDC3	-0.719882219	3.77673858	-16.95263224	9.63E-44	4.08E-41	88.80670968
METTL23	-0.803587224	5.44293971	-16.84342814	2.31E-43	9.56E-41	87.93815084
MRPS28	-0.869017026	4.46517745	-16.83583031	2.46E-43	9.92E-41	87.87770823
NDUFS3	-0.661847354	5.71422434	-16.81845206	2.82E-43	1.11E-40	87.73945393
GON7	-0.754202266	4.66029876	-16.79756731	3.34E-43	1.29E-40	87.57329236
ATP5MP	-0.801296689	5.72681232	-16.77674096	3.95E-43	1.49E-40	87.40758361
ANAPC1	-0.875492991	6.42510629	-16.75794345	4.59E-43	1.69E-40	87.25800781
SDSL	-1.162990632	5.58024218	-16.69007113	7.91E-43	2.86E-40	86.71785815
FAM229E	-0.945498592	4.37937345	-16.64982948	1.09E-42	3.86E-40	86.39754907
POP5	-0.856100246	5.05191122	-16.59334807	1.72E-42	5.92E-40	85.94791484
MALSU1	-0.761890939	4.11241898	-16.59158937	1.74E-42	5.92E-40	85.93391311
NDUFA6	-0.901321463	6.71728473	-16.54951005	2.44E-42	8.13E-40	85.59888395
PRADC1	-1.042662904	5.04531792	-16.54478763	2.54E-42	8.28E-40	85.56128253
ELP6	-0.797154396	4.66008014	-16.53845738	2.67E-42	8.55E-40	85.51087827
MSRB2	-1.023102264	5.55736722	-16.53494858	2.75E-42	8.63E-40	85.48293925
LSM10	-0.78855413	5.82948698	-16.52090304	3.08E-42	9.49E-40	85.37109871
FXN	-0.724162196	3.34679723	-16.45565556	5.19E-42	1.56E-39	84.85150271
TMEM12	-0.664547996	5.44102572	-16.45440203	5.25E-42	1.56E-39	84.84151956
SMIM20	-0.844960504	5.57483966	-16.42976521	6.39E-42	1.87E-39	84.64530468
FBXO16	-0.946940962	2.28067155	-16.38793883	8.95E-42	2.57E-39	84.31216403
UQCR10	-0.910662315	7.70280164	-16.33001725	1.42E-41	4.03E-39	83.850783
HINT2	-0.864973789	5.05667864	-16.25997459	2.50E-41	6.95E-39	83.29278839
C11orf74	-1.578786658	4.7385646	-16.21828255	3.49E-41	9.48E-39	82.96062062
DECR1	-0.622934161	5.5715957	-16.21741125	3.52E-41	9.48E-39	82.95367868
NDUFB5	-0.767788467	5.13632797	-16.19102398	4.35E-41	1.15E-38	82.74343683
BNIP1	-0.756983648	3.71304687	-16.15904364	5.62E-41	1.47E-38	82.48862288
HSPB11	-1.051982219	4.60275305	-16.15579631	5.77E-41	1.48E-38	82.46274823
COPZ1	-0.74496838	7.23354378	-16.13674409	6.73E-41	1.70E-38	82.31093849
SNRNP2	-1.091616558	4.6088203	-16.13247431	6.96E-41	1.74E-38	82.27691616
CISD3	-0.908326049	5.40943298	-16.08783434	9.97E-41	2.45E-38	81.92120852
ECI2	-0.774952025	4.89656526	-16.04097995	1.45E-40	3.52E-38	81.54784156
ZBTB8OS	-0.637069317	4.1327266	-16.01159397	1.84E-40	4.36E-38	81.31366855
TMEM24	-0.752241479	3.74885418	-16.01071165	1.85E-40	4.36E-38	81.30663738
CYC1	-0.882927	6.96017208	-16.00917368	1.88E-40	4.36E-38	81.29438141
RPL26L1	-0.776385476	5.27017725	-15.98805274	2.22E-40	5.09E-38	81.12606889
TXNL4A	-0.697775931	4.77153828	-15.95336	2.94E-40	6.64E-38	80.84959973
MKKS	-0.795406204	4.0340548	-15.93175445	3.49E-40	7.79E-38	80.67742178
SLC25A2	-1.059840668	4.52566057	-15.92952948	3.56E-40	7.83E-38	80.65969067
COX5A	-0.801715098	7.19357577	-15.91210559	4.09E-40	8.89E-38	80.52083637
SLC35B1	-0.954341125	4.88748544	-15.87539704	5.49E-40	1.18E-37	80.22829823

MANF	-1.526630217	6.86362285	-15.86787221	5.84E-40	1.24E-37	80.16833119
COPS9	-1.026480561	5.74162387	-15.8460933	6.95E-40	1.46E-37	79.99477052
LSM3	-0.780147869	4.9919175	-15.76917984	1.29E-39	2.67E-37	79.38183732
ATP5F1E	-0.886160739	6.90228386	-15.72099211	1.90E-39	3.88E-37	78.99783291
QDPR	-0.951356105	4.78463938	-15.70436145	2.17E-39	4.37E-37	78.86530695
NDUFC1	-0.77670461	6.54297928	-15.70180992	2.22E-39	4.37E-37	78.84497454
CENPX	-1.028909084	5.21724697	-15.70176625	2.22E-39	4.37E-37	78.8446266
EIPR1	-0.914073387	4.23519255	-15.6808046	2.62E-39	5.11E-37	78.67759061
KRT10	-1.182198387	4.88124116	-15.67155887	2.83E-39	5.44E-37	78.60391551
DPCD	-0.806372542	4.99791853	-15.58461752	5.68E-39	1.08E-36	77.91115087
SSU72	-0.71149955	6.03404695	-15.58171175	5.81E-39	1.10E-36	77.88799835
FDX1	-0.927267486	5.04547093	-15.53903724	8.19E-39	1.53E-36	77.54798672
HGD	-2.906128409	3.69324535	-15.53027196	8.79E-39	1.62E-36	77.47815137
POP4	-0.569683708	3.80299394	-15.52385905	9.25E-39	1.69E-36	77.42705846
SDF2L1	-1.753185971	6.54614846	-15.5095129	1.04E-38	1.87E-36	77.31276176
MGST2	-0.792527254	5.05977625	-15.50840859	1.05E-38	1.87E-36	77.30396375
EIF2B2	-1.058277022	5.02626143	-15.49601841	1.16E-38	2.04E-36	77.20525256
EBAG9	-0.91402719	5.0040177	-15.48805691	1.23E-38	2.16E-36	77.14182519
NDUFB1	-0.926413408	5.89891093	-15.47837355	1.33E-38	2.31E-36	77.06468127
COX7C	-0.738516087	8.69184024	-15.4443966	1.75E-38	3.00E-36	76.79400905
TSFM	-0.638068361	4.45810069	-15.44030105	1.81E-38	3.07E-36	76.7613836
ATP5PO	-0.790607605	6.00696717	-15.43204441	1.93E-38	3.25E-36	76.69561134
NDUFA5	-0.876170678	5.13045755	-15.42859919	1.99E-38	3.31E-36	76.66816702
UQCRQ	-0.852952864	7.42522976	-15.40077721	2.49E-38	4.09E-36	76.4465466
ATP5PB	-0.832873492	6.72856796	-15.39170185	2.67E-38	4.36E-36	76.374258
KCNQ1	-1.175338912	6.65952727	-15.36832544	3.23E-38	5.21E-36	76.18806254
CMC2	-0.577818865	2.34171428	-15.36034162	3.44E-38	5.51E-36	76.12447274
NDUFC2	-1.023568138	6.53525763	-15.34487092	3.90E-38	6.18E-36	76.00125433
MFSD3	-1.04637598	5.52686493	-15.31271618	5.04E-38	7.92E-36	75.74516779
MPPED2	-2.163220144	2.15139702	-15.30520165	5.36E-38	8.34E-36	75.68532342
MGST3	-0.762891308	5.22660806	-15.29858876	5.65E-38	8.71E-36	75.63266043
PLEKHJ1	-1.155219582	4.78942408	-15.29054623	6.03E-38	9.21E-36	75.5686134
CRELD2	-1.459188733	4.81845095	-15.28190917	6.46E-38	9.78E-36	75.49983332
SPR	-0.974503266	5.63711721	-15.27803075	6.66E-38	1.00E-35	75.46894846
COX16	-0.77918213	5.26276567	-15.27287111	6.94E-38	1.03E-35	75.42786149
PEBP1	-0.858145444	10.815693	-15.25663547	7.91E-38	1.17E-35	75.29857819
MTCH1	-1.226655113	8.8479263	-15.19365324	1.31E-37	1.92E-35	74.79710904
SPX	-2.284886353	2.64901461	-15.18945551	1.36E-37	1.97E-35	74.76368966
MPC1	-1.003704059	6.0812808	-15.18788081	1.37E-37	1.97E-35	74.75115308
SWI5	-1.039459935	6.03657771	-15.18623228	1.39E-37	1.98E-35	74.7380288

CHCHD1	-0.700311354	6.06985084	-15.17784388	1.49E-37	2.10E-35	74.67124805
HSD17B:	-0.855742707	7.40989445	-15.17155694	1.57E-37	2.20E-35	74.62119827
C1QBP	-0.765657996	6.1558855	-15.11172104	2.53E-37	3.52E-35	74.14489943
SEC61B	-0.782714631	7.67337925	-15.09588631	2.87E-37	3.96E-35	74.01886913
SETMAR	-0.856226003	4.02390554	-15.03231454	4.79E-37	6.55E-35	73.51296437
TIMMDC	-0.705917126	5.88812926	-15.02796595	4.96E-37	6.73E-35	73.47836251
NDUFA4	-0.853734679	7.46949523	-15.01901935	5.33E-37	7.17E-35	73.4071759
PTRHD1	-0.947920401	5.17289835	-15.01588814	5.46E-37	7.29E-35	73.38226189
C2orf76	-0.730739828	3.97093864	-15.01472826	5.51E-37	7.31E-35	73.37303319
ENSA	-0.565035623	6.66427892	-15.00383987	6.02E-37	7.91E-35	73.28640068
COQ5	-0.680609115	5.28085434	-14.99043272	6.70E-37	8.74E-35	73.1797328
CRADD	-0.820950761	2.85413127	-14.97824357	7.39E-37	9.56E-35	73.08276024
TRAPPC	-1.202065058	6.18675201	-14.96912431	7.95E-37	1.02E-34	73.01021364
CDKN2A	-0.777923598	5.3967706	-14.96881291	7.97E-37	1.02E-34	73.0077364
COX6A1	-0.810525548	7.7366856	-14.96510377	8.21E-37	1.04E-34	72.9782299
SIRT4	-0.946459375	2.1388413	-14.95914306	8.61E-37	1.08E-34	72.93081272
MRPL48	-0.655257371	4.08182202	-14.95670268	8.78E-37	1.10E-34	72.91139998
PRDX5	-0.949580002	10.4800696	-14.9207977	1.17E-36	1.45E-34	72.62580514
IDH3B	-0.725567665	6.27200217	-14.8856301	1.55E-36	1.91E-34	72.34611756
COQ3	-0.63207233	3.78149554	-14.87628896	1.67E-36	2.04E-34	72.27183481
NDUFS4	-0.801004058	6.59420167	-14.85633951	1.96E-36	2.38E-34	72.11320277
NDUFB4	-0.611573768	6.98649697	-14.85298591	2.02E-36	2.43E-34	72.08653729
HSD17B8	-0.931712316	6.46007274	-14.82449403	2.53E-36	3.02E-34	71.86000694
SPA17	-0.844351466	3.39797188	-14.82389041	2.55E-36	3.02E-34	71.85520801
HEBP1	-0.948638013	6.21731135	-14.81986266	2.63E-36	3.10E-34	71.82318707
STOML1	-0.755565612	3.1750513	-14.80733427	2.91E-36	3.40E-34	71.72358932
NDRG2	-1.133917703	4.52692116	-14.78362377	3.52E-36	4.09E-34	71.5351128
COX20	-0.795450969	2.60240508	-14.76014649	4.24E-36	4.90E-34	71.34851169
TIMM21	-0.711013791	3.38998839	-14.74680287	4.72E-36	5.41E-34	71.24246426
PDIA3	-1.153303864	9.17333682	-14.71562499	6.06E-36	6.90E-34	70.99470848
TPMT	-1.168802338	6.97872637	-14.711505	6.27E-36	7.09E-34	70.96197193
NUCB2	-1.119479833	4.56244755	-14.69132448	7.37E-36	8.28E-34	70.80163193
TCTA	-0.894328555	6.39528996	-14.67578883	8.34E-36	9.31E-34	70.67820846
ATP5MF	-0.758557711	6.45517344	-14.67225328	8.58E-36	9.52E-34	70.65012167
HSP90B:	-1.321934796	10.0129794	-14.6684558	8.85E-36	9.75E-34	70.61995463
COX17	-0.777933962	5.37671457	-14.65160288	1.01E-35	1.11E-33	70.48608336
NUDT9	-0.800502425	5.26768357	-14.6297446	1.21E-35	1.31E-33	70.31247063
LDHB	-1.005574139	8.31274704	-14.62538005	1.25E-35	1.35E-33	70.27780705
TUSC2	-0.695461454	5.7268146	-14.61890222	1.32E-35	1.41E-33	70.22636127
DCTN3	-0.813466142	5.39820456	-14.57518112	1.87E-35	1.99E-33	69.87918648

RWDD1	-0.625065222	4.74175575	-14.54576327	2.36E-35	2.51E-33	69.64563969
TMEM37	-1.186332322	6.28698105	-14.54403972	2.40E-35	2.52E-33	69.63195783
CALR	-1.232375233	10.6242153	-14.53763101	2.52E-35	2.64E-33	69.58108544
UBB	-0.840457118	10.0562212	-14.52845458	2.71E-35	2.82E-33	69.50824639
TIMM8B	-0.704984491	5.97056002	-14.52129869	2.87E-35	2.97E-33	69.45144845
SPATA24	-0.836760115	3.21600854	-14.51358787	3.06E-35	3.14E-33	69.39024876
DNAJB11	-0.96951918	5.32312784	-14.50393868	3.30E-35	3.37E-33	69.31366876
C11orf1	-0.732286825	3.93142105	-14.49483264	3.55E-35	3.61E-33	69.24140356
UQCC2	-0.858174905	4.32770535	-14.48188652	3.94E-35	3.98E-33	69.13867101
POLR2G	-0.6763022	6.08223852	-14.47633936	4.12E-35	4.13E-33	69.09465468
ACADS	-0.915494164	5.20360723	-14.47254251	4.25E-35	4.24E-33	69.06452788
SLC25A4	-0.913437497	5.29163106	-14.46430029	4.54E-35	4.50E-33	68.99913093
COX14	-0.787543553	7.20419688	-14.45280025	4.97E-35	4.90E-33	68.90789113
EMC3	-0.763120351	5.8951892	-14.44529998	5.28E-35	5.18E-33	68.84838872
SMIM19	-0.807608985	4.95866084	-14.42775822	6.08E-35	5.92E-33	68.70923497
MRPS7	-0.707691961	5.44267212	-14.41618649	6.66E-35	6.46E-33	68.61744869
FUNDC1	-0.692855124	5.13533339	-14.39720964	7.76E-35	7.47E-33	68.4669411
CMC1	-0.462343063	2.06667567	-14.39590535	7.84E-35	7.51E-33	68.45659735
MICOS10	-0.688477401	4.07754416	-14.3896984	8.24E-35	7.85E-33	68.40737396
MRPS9	-0.605525273	5.37579486	-14.38083933	8.84E-35	8.38E-33	68.33712206
OSCP1	-0.869691035	3.91118601	-14.37771056	9.07E-35	8.54E-33	68.31231204
HMBS	-0.983990665	3.86219489	-14.37098592	9.57E-35	8.96E-33	68.2589901
WASHC3	-0.59885085	4.89464182	-14.36201869	1.03E-34	9.58E-33	68.18788978
SRPRB	-0.899674205	5.26095582	-14.35422144	1.09E-34	1.01E-32	68.1260697
ALDH9A1	-1.058412243	7.46017679	-14.35213807	1.11E-34	1.03E-32	68.10955247
SYTL3	-1.239576236	4.7196463	-14.35081101	1.12E-34	1.03E-32	68.09903145
MRPL47	-0.605135907	5.8010235	-14.3222426	1.41E-34	1.29E-32	67.87256348
WWOX	-1.010629116	3.67553789	-14.31748115	1.47E-34	1.33E-32	67.83482302
ETFA	-0.753840474	4.98433675	-14.30925192	1.57E-34	1.41E-32	67.76959902
CYB561	-1.137765172	5.6841637	-14.30741445	1.59E-34	1.43E-32	67.75503605
COMMD7	-0.639432772	6.4371579	-14.29752006	1.72E-34	1.54E-32	67.67662056
SMIM7	-0.712592811	4.84115223	-14.29457808	1.76E-34	1.56E-32	67.65330575
CLN5	-1.033028914	4.79353398	-14.28169652	1.95E-34	1.73E-32	67.55122696
GTF2A2	-0.643502872	4.89966702	-14.27814653	2.01E-34	1.77E-32	67.52309719
COQ9	-0.807654234	5.31667578	-14.27256376	2.10E-34	1.84E-32	67.47886126
SNX11	-0.786659202	4.71792447	-14.27134534	2.12E-34	1.84E-32	67.46920717
SDHAF3	-0.946747437	3.77821988	-14.27097054	2.13E-34	1.84E-32	67.46623751
TPI1	-0.698206442	8.33198786	-14.25659938	2.39E-34	2.05E-32	67.35237549
BCDIN3D	-0.789642914	2.61856095	-14.25404907	2.44E-34	2.09E-32	67.33217076
TMEM21	-0.761408949	2.80047409	-14.25049332	2.51E-34	2.14E-32	67.30400124

DAD1	-0.778899153	8.96553363	-14.24748116	2.57E-34	2.18E-32	67.28013869
FAHD2A	-0.944186286	3.74985354	-14.23932589	2.74E-34	2.31E-32	67.21553491
TIMM17A	-0.82105316	5.18906605	-14.23114982	2.92E-34	2.46E-32	67.15077048
PPIL1	-1.197115249	5.49809655	-14.22941836	2.97E-34	2.48E-32	67.13705573
CA4	-2.856553469	2.9751396	-14.22790092	3.00E-34	2.50E-32	67.12503637
NDUFAF	-0.655191534	4.887673	-14.22158268	3.16E-34	2.61E-32	67.07499228
ECHS1	-0.845863176	8.06010338	-14.20454761	3.62E-34	2.98E-32	66.94007717
AIFM1	-0.774181179	5.00682013	-14.18455569	4.24E-34	3.48E-32	66.78176737
MRPL22	-0.50832551	3.6933174	-14.18317458	4.29E-34	3.50E-32	66.77083177
ZSCAN5A	-0.506009299	1.38415973	-14.1826128	4.31E-34	3.50E-32	66.76638366
UQCRB	-0.723143974	5.96193176	-14.16760074	4.86E-34	3.92E-32	66.64752639
EID2	-0.634359845	4.62281994	-14.16577779	4.93E-34	3.96E-32	66.63309426
MRPL20	-0.701302162	6.34565744	-14.15921206	5.19E-34	4.16E-32	66.58111572
CINP	-0.52801639	3.97094176	-14.15462965	5.39E-34	4.29E-32	66.5448401
PTPMT1	-0.588625493	4.36797702	-14.15091021	5.55E-34	4.40E-32	66.51539697
CLYBL	-0.757648561	2.30671855	-14.1499511	5.59E-34	4.41E-32	66.50780479
BCAT2	-0.772302764	4.51732275	-14.14603133	5.77E-34	4.51E-32	66.47677699
NDUFS2	-0.727939759	6.03062133	-14.14593745	5.77E-34	4.51E-32	66.47603388
FH	-0.769292529	5.97041425	-14.13852778	6.13E-34	4.77E-32	66.41738398
CETN2	-0.853372562	6.92373862	-14.12501277	6.82E-34	5.29E-32	66.31041753
PPIB	-1.037624889	8.88859191	-14.11687484	7.28E-34	5.61E-32	66.24601458
NEDD8	-0.679164691	5.67030367	-14.11296284	7.51E-34	5.77E-32	66.21505693
C16orf89	-2.171029713	8.35525466	-14.10323013	8.12E-34	6.20E-32	66.13804134
LRTOMT	-0.749552968	2.74510147	-14.09465547	8.69E-34	6.60E-32	66.07019474
MRPS14	-0.773564514	4.96536293	-14.09430674	8.72E-34	6.60E-32	66.06743552
COA6	-0.68815233	5.08302113	-14.08684948	9.25E-34	6.98E-32	66.0084345
SEC11C	-1.052434608	5.47066009	-14.08569912	9.34E-34	7.01E-32	65.99933332
MRPS26	-0.893197506	7.16594764	-14.08386067	9.48E-34	7.08E-32	65.9847884
SNF8	-0.570522722	5.08339492	-14.08274169	9.56E-34	7.11E-32	65.97593572
JTB	-0.704111688	7.98604095	-14.06799534	1.08E-33	7.97E-32	65.85927947
PEF1	-0.714138384	7.72683703	-14.06695235	1.08E-33	8.00E-32	65.85102911
ATP5ME	-0.892856927	7.94404285	-14.04962935	1.25E-33	9.14E-32	65.7140097
C1orf122	-0.948854497	5.624044	-14.04823893	1.26E-33	9.20E-32	65.70301278
SPCS2	-0.836767708	5.62812856	-14.04469743	1.30E-33	9.43E-32	65.67500357
EMC8	-0.609988378	4.13169601	-14.04145436	1.33E-33	9.63E-32	65.64935538
TMX2	-0.890200763	6.60329178	-14.0283989	1.47E-33	1.06E-31	65.54611206
HMOX2	-0.622318547	5.30871317	-14.02590272	1.50E-33	1.08E-31	65.52637346
BTBD11	-1.854637724	3.31813961	-14.02062822	1.57E-33	1.12E-31	65.48466681
TMA7	-0.775143297	7.80620719	-14.01523252	1.64E-33	1.17E-31	65.44200376
FKBP1A	-0.800709969	8.08844688	-14.01028216	1.70E-33	1.21E-31	65.40286372

MRPS22	-0.554638081	4.01080252	-14.00869141	1.73E-33	1.22E-31	65.39028687
BCCIP	-0.639539506	5.01831277	-13.99044374	2.00E-33	1.40E-31	65.24602907
SLC25A3	-0.961501906	2.44898241	-13.97475761	2.26E-33	1.59E-31	65.12204072
SMIM10L	-0.660428686	4.35957301	-13.95624253	2.62E-33	1.83E-31	64.97571458
TMEM12	-0.73205567	6.33949265	-13.9505183	2.74E-33	1.91E-31	64.9304806
PXMP2	-0.888922946	3.86608844	-13.9437355	2.90E-33	2.01E-31	64.87688475
MRO	-1.774424494	1.38739837	-13.94056	2.97E-33	2.05E-31	64.85179398
COA4	-0.641530065	5.92611364	-13.93637557	3.07E-33	2.11E-31	64.81873249
NT5C3B	-0.910253809	5.84410705	-13.92984443	3.24E-33	2.21E-31	64.76713194
FKBP4	-0.923646441	6.16339964	-13.91728878	3.58E-33	2.43E-31	64.66794265
PEX11A	-1.103581354	3.6123645	-13.91716832	3.58E-33	2.43E-31	64.6669911
LDHD	-1.722975365	3.9216153	-13.8983051	4.16E-33	2.81E-31	64.51799475
SLC25A3	-0.645911793	7.20736545	-13.89154131	4.39E-33	2.95E-31	64.46457569
RPP25L	-0.75606044	5.76682524	-13.89118604	4.40E-33	2.95E-31	64.46176988
GCDH	-0.674450731	4.03312053	-13.86715728	5.33E-33	3.56E-31	64.27202457
SULT1A1	-1.344963513	3.73833359	-13.86040119	5.62E-33	3.73E-31	64.21868249
MRPL24	-0.655490506	6.59765987	-13.86023554	5.63E-33	3.73E-31	64.21737465
DSTN	-0.828985116	8.12823009	-13.85758439	5.75E-33	3.80E-31	64.19644378
WSCD2	-2.624189402	2.1006274	-13.84518376	6.35E-33	4.17E-31	64.09854784
NCBP2A	-0.750789157	5.54409176	-13.84082168	6.57E-33	4.30E-31	64.06411458
RAB5IF	-0.778809687	4.74779148	-13.83199237	7.05E-33	4.59E-31	63.99442259
2-Mar	-1.370274026	4.86477063	-13.83180385	7.06E-33	4.59E-31	63.99293459
MOCS2	-0.684872385	4.7880034	-13.82933126	7.20E-33	4.66E-31	63.97341898
FAM104E	-0.686366926	3.85372317	-13.82139799	7.67E-33	4.95E-31	63.91080657
CYB5D2	-0.95898822	5.43566305	-13.82011843	7.75E-33	4.98E-31	63.90070829
GTF2H5	-0.569026771	3.86269757	-13.8162955	7.99E-33	5.11E-31	63.87053848
NFU1	-0.658289521	5.45030823	-13.80616241	8.66E-33	5.52E-31	63.79057573
ATP5F1A	-0.828852245	6.98274551	-13.79389666	9.55E-33	6.04E-31	63.69379469
APOM	-0.822129556	2.71194961	-13.79388321	9.55E-33	6.04E-31	63.69368856
COMMD1	-0.659586964	5.18038366	-13.7819526	1.05E-32	6.62E-31	63.59956354
UQCR11	-0.779848912	6.48644433	-13.77976764	1.07E-32	6.71E-31	63.58232683
VPS25	-0.713416927	5.65009941	-13.7667375	1.19E-32	7.42E-31	63.4795429
MTX2	-0.731141999	5.69431519	-13.76277342	1.22E-32	7.63E-31	63.44827629
C9orf116	-1.059541595	3.12444307	-13.73476722	1.53E-32	9.50E-31	63.22741522
DHRS7	-0.962312447	6.54150841	-13.72902924	1.60E-32	9.90E-31	63.18217278
LYRM4	-0.657558006	3.23603974	-13.7239501	1.67E-32	1.03E-30	63.14212739
KATNAL1	-1.436743456	2.4228608	-13.70582657	1.92E-32	1.18E-30	62.99925401
PDHB	-0.580677887	5.22911349	-13.70325617	1.96E-32	1.20E-30	62.97899297
ATP5MD	-0.723481631	7.4044699	-13.69963679	2.02E-32	1.23E-30	62.95046442
ESAM	-1.281323834	7.14347879	-13.69951762	2.02E-32	1.23E-30	62.94952513

SURF1	-0.782515483	6.41713862	-13.6813681	2.34E-32	1.42E-30	62.80648473
ACSF2	-1.125901602	5.2458958	-13.67801907	2.40E-32	1.45E-30	62.78009339
HSPA5	-1.475377759	9.66959932	-13.6655054	2.65E-32	1.59E-30	62.68149046
RILP	-0.878608702	5.44060578	-13.65888735	2.79E-32	1.67E-30	62.62934831
YIPF1	-1.051782744	5.95372122	-13.65509471	2.88E-32	1.72E-30	62.59946866
PIH1D2	-0.770692314	2.27851454	-13.63574424	3.36E-32	2.00E-30	62.44703892
MRPL13	-0.654593084	4.00968179	-13.63467364	3.39E-32	2.01E-30	62.43860648
COMMD8	-0.842872057	4.61481014	-13.62661519	3.61E-32	2.13E-30	62.37513806
ACYP2	-0.685321477	3.14218631	-13.60335593	4.34E-32	2.56E-30	62.19198033
MRPL51	-0.66521736	7.33841674	-13.59659967	4.58E-32	2.69E-30	62.13878651
MTX1	-0.593349085	3.80004529	-13.59351027	4.70E-32	2.75E-30	62.11446418
CFAP53	-0.942073921	2.95188416	-13.59170955	4.76E-32	2.78E-30	62.10028781
XPA	-0.906070855	5.19976792	-13.59110926	4.79E-32	2.78E-30	62.09556208
STMP1	-0.701431462	5.17774476	-13.58350918	5.08E-32	2.94E-30	62.03573327
SMDT1	-0.863429564	6.76937984	-13.56828837	5.74E-32	3.31E-30	61.91592896
TXNL1	-0.873968156	5.47311322	-13.56784964	5.76E-32	3.31E-30	61.91247603
MTIF3	-0.611224463	5.993219	-13.56575139	5.85E-32	3.35E-30	61.89596223
SULT1A2	-1.543366081	2.72125052	-13.56412573	5.93E-32	3.39E-30	61.88316821
MRPL34	-0.802881428	6.23873835	-13.56123518	6.07E-32	3.45E-30	61.86041993
PRDX2	-0.725893852	8.13997691	-13.55653878	6.30E-32	3.57E-30	61.82346145
COPZ2	-1.240011048	4.59477975	-13.54529382	6.89E-32	3.89E-30	61.73497725
ARHGAP	-1.670953116	2.95003329	-13.53204063	7.65E-32	4.31E-30	61.63070567
MDP1	-0.719885731	2.86151659	-13.51476407	8.77E-32	4.93E-30	61.49480429
PPCS	-0.665408337	6.13867072	-13.51190397	8.97E-32	5.02E-30	61.47230882
CNPPD1	-0.712333069	6.87539597	-13.50983307	9.12E-32	5.09E-30	61.45602111
SLC25A4	-1.179143039	4.89782424	-13.5040618	9.55E-32	5.31E-30	61.41063186
DNAJC19	-0.59101638	4.4908446	-13.49686406	1.01E-31	5.60E-30	61.35402833
EEF1AKI	-0.659681328	4.1352457	-13.48958878	1.07E-31	5.92E-30	61.29682012
TMEM25	-0.768980461	4.83486525	-13.48017282	1.15E-31	6.35E-30	61.22278629
IFT22	-0.701534942	5.06414901	-13.47994236	1.16E-31	6.35E-30	61.22097439
PDRG1	-0.694398419	4.84936505	-13.47767785	1.18E-31	6.44E-30	61.20317086
GNA14	-1.889067638	3.63111054	-13.47358417	1.22E-31	6.63E-30	61.17098756
TXN2	-0.673618332	7.02093004	-13.46716933	1.28E-31	6.96E-30	61.12055931
C3orf33	-0.997332317	3.92070933	-13.46530222	1.30E-31	7.04E-30	61.10588234
FAM167A	-2.849215159	4.61370037	-13.45555874	1.40E-31	7.58E-30	61.02929629
MTFR1L	-0.693680357	5.12697298	-13.45336406	1.43E-31	7.69E-30	61.01204693
SF3B5	-0.70487499	8.21622744	-13.44604569	1.51E-31	8.12E-30	60.95453046
LAMTOR	-0.536706589	6.35793172	-13.43527108	1.65E-31	8.82E-30	60.86986022
ATOX1	-0.835941248	4.81231179	-13.42566485	1.78E-31	9.48E-30	60.79438111
KIF9	-0.800259545	3.09159727	-13.4118367	1.98E-31	1.05E-29	60.68574502

C3orf18	-0.868139091	3.71108268	-13.40741293	2.05E-31	1.09E-29	60.65099524
MRPS36	-0.638406257	5.61701212	-13.3916021	2.33E-31	1.23E-29	60.52681313
CDADC1	-0.882098732	3.24791618	-13.38893829	2.38E-31	1.25E-29	60.50589343
NDUFAB	-0.728763614	6.41040981	-13.38589583	2.44E-31	1.28E-29	60.48200086
ECSIT	-0.811028506	5.34844161	-13.38117917	2.53E-31	1.32E-29	60.44496264
TOMM5	-0.599062967	3.69188581	-13.37937941	2.57E-31	1.34E-29	60.43083032
MIEN1	-0.621870681	6.08285489	-13.35680499	3.07E-31	1.60E-29	60.25359668
CENPS	-0.714365074	3.10555047	-13.3452493	3.36E-31	1.74E-29	60.16289204
CRELD1	-1.067366355	5.87690738	-13.33608784	3.61E-31	1.87E-29	60.09099026
CAPSL	-2.090945411	2.01489069	-13.33281363	3.71E-31	1.91E-29	60.06529539
DHRS7B	-0.673987954	4.45125444	-13.33082052	3.77E-31	1.94E-29	60.04965474
TRIP4	-0.770837694	4.98422481	-13.3252984	3.94E-31	2.02E-29	60.00632269
ERI3	-0.616764453	5.69077116	-13.31091197	4.41E-31	2.25E-29	59.89344742
SRP19	-0.632848321	4.47948309	-13.30722589	4.54E-31	2.31E-29	59.86453005
SDHD	-0.880237014	6.36841937	-13.30059802	4.79E-31	2.43E-29	59.81253783
TMEM14	-0.611364602	7.96161975	-13.28291853	5.50E-31	2.79E-29	59.67387399
SFXN4	-0.874475819	5.28811971	-13.28020943	5.62E-31	2.84E-29	59.65262884
AK6	-0.639405438	4.84143169	-13.26186983	6.50E-31	3.27E-29	59.50882774
SDHB	-0.608637712	6.11006958	-13.25480328	6.88E-31	3.45E-29	59.45342828
NDUFV3	-0.553717616	4.49888601	-13.24875502	7.21E-31	3.61E-29	59.40601624
MTHFS	-0.831895972	3.48486917	-13.23462171	8.06E-31	4.02E-29	59.29524101
PIR	-1.218107452	3.37381929	-13.23230188	8.21E-31	4.09E-29	59.27706052
TMCO1	-0.787874699	5.61940899	-13.23129143	8.28E-31	4.11E-29	59.26914185
COA3	-0.662004127	6.27305979	-13.22652006	8.60E-31	4.25E-29	59.23175095
TMEM11	-0.560913766	4.58294801	-13.22317485	8.83E-31	4.35E-29	59.20553774
OXSM	-0.617670588	3.72059366	-13.21237939	9.61E-31	4.73E-29	59.12095211
ATP5IF1	-0.728029114	7.53540524	-13.18906347	1.16E-30	5.67E-29	58.9383085
FAAH	-1.038951478	5.47178656	-13.18312777	1.21E-30	5.92E-29	58.89182117
OAZ2	-0.775486244	5.54651173	-13.17465799	1.30E-30	6.31E-29	58.82549411
ECI1	-0.991335135	6.64313829	-13.16803437	1.36E-30	6.62E-29	58.7736299
DERL2	-0.721298983	4.62186259	-13.1678617	1.37E-30	6.62E-29	58.77227795
TFF3	-3.879491737	5.47456694	-13.16515094	1.40E-30	6.73E-29	58.75105365
CHCHD2	-0.726394135	8.79956232	-13.16510277	1.40E-30	6.73E-29	58.75067649
B9D1	-0.845472084	4.75697373	-13.16443918	1.40E-30	6.75E-29	58.74548095
MRPL2	-0.54872934	5.19848948	-13.15692403	1.49E-30	7.14E-29	58.68664489
C16orf91	-0.750710766	4.64239816	-13.14037499	1.70E-30	8.11E-29	58.5571048
PREB	-0.581695295	5.43679822	-13.13349624	1.79E-30	8.54E-29	58.50326939
ABCB8	-0.671295803	3.78552378	-13.11437483	2.08E-30	9.90E-29	58.35364709
TSR2	-0.671148512	7.2512592	-13.11244532	2.12E-30	1.00E-28	58.3385512
PNPLA4	-0.934886699	4.70422287	-13.09835239	2.37E-30	1.12E-28	58.22830584

POLR2K	-0.604161913	6.08430969	-13.0950836	2.43E-30	1.14E-28	58.20273823
SRA1	-0.612527142	5.33353326	-13.09307961	2.47E-30	1.16E-28	58.18706406
AAMDC	-0.823252586	4.71088037	-13.08483485	2.63E-30	1.23E-28	58.12258305
NDUFA1(	-0.582214839	4.72494421	-13.07823119	2.77E-30	1.30E-28	58.07094241
HINT1	-0.779347873	8.26838313	-13.07669691	2.81E-30	1.31E-28	58.0589451
HACL1	-0.744318086	4.50981773	-13.07524744	2.84E-30	1.32E-28	58.04761117
MRPL21	-0.725152091	5.19212907	-13.05598108	3.30E-30	1.53E-28	57.89698397
UCHL3	-0.672419584	4.03496044	-13.05546576	3.32E-30	1.53E-28	57.8929557
PGP	-0.752374811	4.71464482	-13.04135259	3.71E-30	1.71E-28	57.78264527
PSMC4	-0.571387505	6.84912287	-13.02593481	4.19E-30	1.92E-28	57.66216472
CENPS-(	-0.50834287	1.02594468	-13.01676706	4.50E-30	2.06E-28	57.59053765
CCDC51	-0.700634566	4.0346608	-13.01602852	4.53E-30	2.07E-28	57.58476787
ANAPC1(	-0.582725779	6.59422746	-13.00478674	4.95E-30	2.26E-28	57.49695129
MDH2	-0.818014311	6.98189711	-12.99733508	5.25E-30	2.39E-28	57.43874997
SPCS1	-0.694599147	6.02124179	-12.99622278	5.29E-30	2.40E-28	57.43006287
SPATA4	-0.706066415	0.85494791	-12.99229117	5.46E-30	2.47E-28	57.39935823
FARS2	-0.769405957	4.5605683	-12.99106307	5.51E-30	2.49E-28	57.38976755
CISD2	-0.691556416	5.30706083	-12.97981153	6.02E-30	2.71E-28	57.30190833
PDCD6	-0.609171463	4.80388654	-12.97512838	6.25E-30	2.80E-28	57.26534392
SLC39A7	-0.984148312	7.59812346	-12.97373301	6.32E-30	2.83E-28	57.2544498
MRPL36	-0.562669663	4.80667798	-12.97197038	6.41E-30	2.86E-28	57.2406888
CAMKMT	-0.608517191	2.77871877	-12.97068095	6.47E-30	2.88E-28	57.23062226
MRPL58	-0.546158686	5.53780681	-12.96980952	6.52E-30	2.89E-28	57.22381919
ETFRF1	-0.750236718	4.87775796	-12.96012328	7.03E-30	3.11E-28	57.14820698
TMEM50	-1.09130813	6.18739685	-12.95674064	7.22E-30	3.19E-28	57.12180423
PTGES2	-0.616695173	5.65808245	-12.95300468	7.44E-30	3.28E-28	57.09264537
FKBP2	-0.983647336	7.94539534	-12.95060688	7.58E-30	3.33E-28	57.0739317
GSTK1	-0.634236752	6.52309943	-12.94050078	8.21E-30	3.60E-28	56.99506595
PDHA1	-0.675977464	5.78268975	-12.93559358	8.53E-30	3.73E-28	56.95677574
MRPS10	-0.688288222	5.5546206	-12.92539012	9.24E-30	4.03E-28	56.87716923
MCRIP2	-1.038963686	4.08289526	-12.92474096	9.29E-30	4.04E-28	56.87210496
PKIG	-0.689977973	6.35766374	-12.90853539	1.06E-29	4.58E-28	56.74569825
LMAN2L	-0.899514119	5.23739009	-12.90307154	1.10E-29	4.77E-28	56.70308646
COX4I1	-0.687117891	7.65402572	-12.88969596	1.22E-29	5.28E-28	56.59878808
BORCS7	-0.877177436	6.00060025	-12.887081	1.25E-29	5.38E-28	56.57840012
OCIAD1	-0.769114595	6.63748362	-12.88486816	1.27E-29	5.46E-28	56.56114801
BDH1	-0.911109672	3.42138668	-12.87748098	1.35E-29	5.77E-28	56.50355935
UQCRFS	-0.832358326	5.27810178	-12.87657432	1.36E-29	5.80E-28	56.49649172
IDNK	-0.766017757	3.64646235	-12.87102115	1.42E-29	6.04E-28	56.4532057
MPDU1	-0.633501621	5.83338513	-12.85708302	1.58E-29	6.73E-28	56.34457769

TLE6	-1.213974819	2.80124482	-12.85581861	1.60E-29	6.78E-28	56.33472466
MON1A	-0.684249794	3.45370845	-12.84522495	1.74E-29	7.35E-28	56.25218024
MLEC	-1.059045079	6.97316051	-12.84436721	1.75E-29	7.38E-28	56.24549752
HSPE1	-0.678503212	6.24434184	-12.83790781	1.84E-29	7.74E-28	56.19517454
MRPL33	-0.784143611	5.73902927	-12.83737288	1.85E-29	7.75E-28	56.19100727
MRPL32	-0.616794551	4.8713023	-12.81826966	2.15E-29	8.99E-28	56.04221336
MAP1LC	-1.188690977	4.97011908	-12.80815726	2.32E-29	9.71E-28	55.96346751
NAA20	-0.628278902	6.14930666	-12.80738548	2.34E-29	9.74E-28	55.95745819
NDFIP1	-0.871084289	7.21286579	-12.80433144	2.39E-29	9.95E-28	55.9336791
HYKK	-0.556186796	1.39331049	-12.80147024	2.45E-29	1.02E-27	55.91140267
PNKD	-0.638128399	5.72088398	-12.80000021	2.48E-29	1.02E-27	55.89995786
SH3BGR	-1.10064149	4.02455213	-12.79779573	2.52E-29	1.04E-27	55.88279554
ASRGL1	-0.965730034	2.52373	-12.79583789	2.56E-29	1.05E-27	55.86755386
ADAL	-0.805923308	2.79843461	-12.78668216	2.75E-29	1.13E-27	55.79628399
ATP6V0E	-0.689170421	6.67890395	-12.78325057	2.83E-29	1.16E-27	55.76957465
CHCHD5	-0.674030234	4.36591192	-12.78275932	2.84E-29	1.16E-27	55.76575118
NDUFA9	-0.501240974	3.48428123	-12.78047707	2.89E-29	1.18E-27	55.74798859
IMMP1L	-0.535357744	3.2558588	-12.7789348	2.92E-29	1.19E-27	55.73598558
RTN3	-0.842439384	7.10707411	-12.77286587	3.07E-29	1.24E-27	55.68875614
POLD2	-0.732810734	5.62961163	-12.76825864	3.18E-29	1.29E-27	55.65290512
ENY2	-0.58275877	4.22816356	-12.76681224	3.22E-29	1.30E-27	55.64165064
SNX17	-0.708397559	6.7178386	-12.76236221	3.33E-29	1.34E-27	55.60702639
DNAL4	-0.760964096	5.39584837	-12.7603305	3.38E-29	1.36E-27	55.59121918
PINX1	-0.504186668	2.14856635	-12.75500795	3.53E-29	1.41E-27	55.54981101
TMED9	-0.687624673	7.76046783	-12.75491274	3.53E-29	1.41E-27	55.54907035
MMP15	-1.251413733	6.86906579	-12.74832543	3.72E-29	1.48E-27	55.49782793
C20orf96	-0.890262259	4.67405322	-12.74650748	3.77E-29	1.50E-27	55.48368718
RDH11	-0.836730341	5.82473417	-12.73768132	4.04E-29	1.61E-27	55.41504011
MED19	-0.743010924	4.34935297	-12.736707	4.07E-29	1.61E-27	55.40746284
TIMM17E	-0.828286512	5.1201231	-12.72980766	4.30E-29	1.70E-27	55.35381008
MRPS34	-0.731416561	7.16117208	-12.72926009	4.32E-29	1.70E-27	55.34955222
EMC7	-0.624510987	7.03872348	-12.72803877	4.36E-29	1.72E-27	55.34005532
TCF7L1	-1.625245324	3.59398016	-12.72761444	4.37E-29	1.72E-27	55.33675586
NDUFS6	-0.74828129	6.65310049	-12.72629311	4.42E-29	1.73E-27	55.32648167
NFS1	-0.591965865	3.3701327	-12.71586601	4.80E-29	1.87E-27	55.24541244
UBE2D2	-0.593811593	6.3227041	-12.70725557	5.13E-29	2.00E-27	55.17847848
AACS	-0.693899992	2.75623125	-12.70214819	5.34E-29	2.08E-27	55.13878062
ERP29	-0.688457725	7.69306889	-12.70151531	5.37E-29	2.08E-27	55.13386172
SPATA1	-0.605313039	1.13103053	-12.69825463	5.51E-29	2.13E-27	55.10851979
MCRS1	-0.596644449	6.03771508	-12.69812913	5.51E-29	2.13E-27	55.1075444

PMVK	-0.726755248	7.00529718	-12.69560195	5.62E-29	2.16E-27	55.08790425
TACO1	-0.556132983	4.48968408	-12.69548275	5.63E-29	2.16E-27	55.08697786
NELFCD	-0.738307002	5.12231649	-12.69235648	5.77E-29	2.21E-27	55.06268308
NMNAT1	-0.773756906	3.47280288	-12.68694891	6.02E-29	2.30E-27	55.02066303
PRRG2	-0.869265165	5.0490179	-12.68083387	6.31E-29	2.41E-27	54.97315033
POLR3G	-0.74577455	5.76853955	-12.673473	6.69E-29	2.55E-27	54.91596456
LGI3	-2.088777166	3.30157921	-12.67042237	6.85E-29	2.60E-27	54.89226671
SHLD1	-0.645424383	3.63362912	-12.67009551	6.87E-29	2.60E-27	54.88972769
SLC4A4	-2.226408684	2.91563942	-12.66377309	7.21E-29	2.73E-27	54.84061845
ETFB	-0.845626335	5.11235777	-12.66337008	7.24E-29	2.73E-27	54.83748828
MANBAL	-0.735049931	6.69877261	-12.66317411	7.25E-29	2.73E-27	54.83596614
OARD1	-0.688497536	4.15202083	-12.6622175	7.30E-29	2.75E-27	54.82853626
RUVBL1	-0.608119784	3.92478799	-12.65894664	7.49E-29	2.81E-27	54.80313279
SMIM8	-0.443281811	1.72586857	-12.6564956	7.64E-29	2.86E-27	54.78409748
IAH1	-0.571643661	4.27505498	-12.64604415	8.29E-29	3.10E-27	54.70293873
MT1F	-2.837154931	6.36093613	-12.63647986	8.93E-29	3.33E-27	54.62868229
SELENBI	-1.226172609	7.14988289	-12.6326984	9.20E-29	3.42E-27	54.59932685
GRPEL1	-0.527674161	4.23409739	-12.62838075	9.52E-29	3.53E-27	54.56581136
ST7	-0.697459326	4.10877511	-12.61903252	1.02E-28	3.79E-27	54.49325537
SLC5A8	-2.796601884	2.98240026	-12.61048195	1.10E-28	4.05E-27	54.42690107
PRPSAP	-0.809393961	4.50898287	-12.60569784	1.14E-28	4.19E-27	54.3897798
ATPAF1	-0.56529389	5.10492375	-12.60368375	1.15E-28	4.25E-27	54.37415288
SOD1	-0.674676555	8.13408657	-12.59844112	1.20E-28	4.42E-27	54.33347908
C11orf65	-0.409885553	0.85944277	-12.59065053	1.28E-28	4.69E-27	54.27304471
NDUFB1	-0.76323189	7.79318347	-12.58403268	1.35E-28	4.92E-27	54.22171437
MED11	-0.555014668	5.25417118	-12.56930635	1.51E-28	5.51E-27	54.10751442
INAFM2	-1.186148827	6.60482434	-12.56838024	1.52E-28	5.54E-27	54.1003336
COPS3	-0.618979385	5.48185268	-12.56121521	1.61E-28	5.84E-27	54.0447823
ISOC2	-0.884719061	4.88636037	-12.56101926	1.61E-28	5.84E-27	54.04326316
MESD	-0.810157331	5.77022057	-12.55988978	1.63E-28	5.88E-27	54.03450682
EPHX1	-1.088817202	7.16852777	-12.55651143	1.67E-28	6.03E-27	54.00831733
ACAD10	-0.792333824	3.68707629	-12.55064628	1.75E-28	6.30E-27	53.96285356
DHRS4	-0.666588094	3.89404402	-12.53477202	1.98E-28	7.12E-27	53.83982903
NDUFA2	-0.750070062	6.91343933	-12.53363539	2.00E-28	7.16E-27	53.83102163
PDIA5	-1.211886952	4.72450028	-12.53010388	2.05E-28	7.35E-27	53.80365817
TMEM16	-0.910132395	5.01469664	-12.52870017	2.08E-28	7.41E-27	53.79278225
FHL1	-2.188834007	6.1969674	-12.52764898	2.09E-28	7.46E-27	53.78463783
C16orf46	-1.019799137	2.74166341	-12.52626608	2.12E-28	7.51E-27	53.7739236
DCXR	-1.075031056	6.02898771	-12.52621138	2.12E-28	7.51E-27	53.77349979
MRPS18	-0.495518714	3.05468391	-12.5245669	2.14E-28	7.59E-27	53.76075933

CCDC13	-0.430985048	0.83986751	-12.52082889	2.21E-28	7.80E-27	53.73180079
NME5	-0.719018573	3.8912597	-12.50251811	2.55E-28	8.98E-27	53.5899761
TM2D2	-0.768902028	5.06745169	-12.49832076	2.63E-28	9.26E-27	53.55747284
CXorf40A	-0.650362088	3.22243113	-12.4941173	2.72E-28	9.55E-27	53.52492479
TEX264	-0.6245419	5.47149395	-12.49215253	2.76E-28	9.68E-27	53.50971221
P3H4	-0.766704625	4.89798762	-12.48947973	2.82E-28	9.87E-27	53.48901841
ATG10	-0.673342156	2.70088098	-12.48108315	3.01E-28	1.05E-26	53.42401598
TMEM22	-1.097750965	3.16226318	-12.47820518	3.08E-28	1.07E-26	53.40173843
METTL5	-0.545621831	4.79302045	-12.46556098	3.40E-28	1.18E-26	53.30387799
ANXA6	-0.950510258	6.42508621	-12.46436781	3.43E-28	1.19E-26	53.29464461
OSTC	-0.751614238	6.85174626	-12.4618741	3.50E-28	1.21E-26	53.27534773
GIN1	-0.575912334	2.62789891	-12.4618168	3.50E-28	1.21E-26	53.27490434
GSTZ1	-0.670288347	3.39944353	-12.46081574	3.53E-28	1.22E-26	53.2671581
SUB1	-0.572991943	5.10815959	-12.45864735	3.59E-28	1.23E-26	53.25037967
NAA38	-0.798940529	6.02283186	-12.45198123	3.78E-28	1.30E-26	53.1988034
TIMM23	-0.547292075	6.90543051	-12.45143769	3.80E-28	1.30E-26	53.19459822
RIBC1	-0.793012677	2.35641664	-12.45120545	3.80E-28	1.30E-26	53.19280156
SRP14	-0.618071678	8.78826346	-12.44318317	4.05E-28	1.38E-26	53.1307423
SDHAF1	-0.753379264	5.27773504	-12.43955732	4.17E-28	1.42E-26	53.10269637
IFT20	-0.651882856	4.48294335	-12.43818878	4.21E-28	1.43E-26	53.0921112
SORBS2	-1.796464954	4.51143968	-12.43663787	4.26E-28	1.45E-26	53.08011589
COASY	-0.622749306	6.01079997	-12.4288346	4.53E-28	1.53E-26	53.0197678
NAXD	-0.742405895	5.93861376	-12.42514498	4.66E-28	1.58E-26	52.9912366
RGCC	-1.54997371	7.17016973	-12.42222536	4.77E-28	1.61E-26	52.96866121
ALG5	-0.751064377	6.01654345	-12.41717982	4.96E-28	1.67E-26	52.92965046
ILVBL	-0.833438851	5.83425614	-12.41274347	5.14E-28	1.72E-26	52.89535305
AAGAB	-0.750540922	5.32002829	-12.41152556	5.18E-28	1.74E-26	52.88593796
MYT1	-0.740894797	0.99037148	-12.40378833	5.51E-28	1.84E-26	52.82613004
RWDD4	-0.703955136	3.83433671	-12.39435529	5.93E-28	1.98E-26	52.75322599
SEPHS2	-0.664184493	6.28943608	-12.38791542	6.23E-28	2.08E-26	52.70346278
UGP2	-0.725988855	5.42390572	-12.38472574	6.39E-28	2.13E-26	52.67881735
NBDY	-0.640952118	6.53036275	-12.36424879	7.50E-28	2.49E-26	52.52063683
RMDN1	-0.715768026	4.64685745	-12.36303425	7.57E-28	2.51E-26	52.51125679
GSTM4	-0.842414604	4.300637	-12.3515886	8.27E-28	2.74E-26	52.42287182
NAXE	-0.661469133	7.0716958	-12.34845915	8.48E-28	2.79E-26	52.39870924
TUFM	-0.572512041	7.89543224	-12.34842128	8.48E-28	2.79E-26	52.39841684
UQCC1	-0.600273413	4.16507334	-12.34772525	8.53E-28	2.80E-26	52.39304304
HMGCL	-0.578323177	5.3224973	-12.3403373	9.03E-28	2.96E-26	52.33600745
ATP6V0E	-0.65944321	8.55358188	-12.33752893	9.23E-28	3.02E-26	52.31432881
UROS	-0.577109493	4.4120964	-12.32581333	1.01E-27	3.30E-26	52.223906

HAX1	-0.541752538	6.70499015	-12.32506234	1.02E-27	3.32E-26	52.21811045
RGN	-1.189273313	4.59933961	-12.32314997	1.03E-27	3.36E-26	52.20335278
SMIM27	-0.609120746	3.2766331	-12.3217675	1.04E-27	3.39E-26	52.19268463
DMAC1	-0.643535253	5.53983577	-12.31422571	1.11E-27	3.59E-26	52.13449216
ARHGAF	-1.061085648	2.7354855	-12.30773259	1.16E-27	3.77E-26	52.0843984
GMPPB	-0.781269325	4.01731332	-12.29993927	1.24E-27	4.00E-26	52.02428249
TMEM88	-1.519336662	4.63997122	-12.29700753	1.27E-27	4.08E-26	52.00167017
GALT	-0.703542198	4.29497306	-12.28586817	1.38E-27	4.44E-26	51.91576562
PDAP1	-0.538957129	6.83065213	-12.28433207	1.40E-27	4.49E-26	51.90392109
DYNLT1	-0.703967485	5.97578226	-12.28213008	1.42E-27	4.56E-26	51.88694265
PRPS1	-0.995401229	5.6539878	-12.27905754	1.46E-27	4.66E-26	51.86325307
C8orf48	-0.870297062	2.48460574	-12.27701231	1.48E-27	4.72E-26	51.84748503
SPATA3	-0.593714246	2.34642561	-12.27428584	1.51E-27	4.81E-26	51.82646592
PIGP	-0.599180827	3.22414643	-12.27234858	1.53E-27	4.87E-26	51.81153174
MRPL35	-0.696002303	4.58368358	-12.27225827	1.53E-27	4.87E-26	51.81083553
ITPR1	-1.7710268	3.54684354	-12.27077779	1.55E-27	4.92E-26	51.79942309
HMGN5	-0.978381175	4.29086739	-12.26433316	1.63E-27	5.16E-26	51.74974793
FLRT1	-1.835114455	1.58610673	-12.24565124	1.89E-27	5.96E-26	51.60578553
ZMYND1	-0.875662679	3.13536963	-12.24182215	1.94E-27	6.13E-26	51.57628562
MIOX	-2.052758618	2.85762787	-12.23487782	2.05E-27	6.46E-26	51.52279141
MRPL15	-0.690902747	5.99569243	-12.23151327	2.11E-27	6.62E-26	51.49687613
TMEM22	-0.601800001	4.70337184	-12.21183713	2.45E-27	7.69E-26	51.34535849
LIPT2	-0.760731187	2.44280516	-12.20923877	2.50E-27	7.84E-26	51.32535434
NDUFAF	-0.79152991	5.41086357	-12.20822227	2.52E-27	7.88E-26	51.31752885
TMCO4	-0.682206899	5.15312678	-12.2079856	2.53E-27	7.88E-26	51.31570691
COQ4	-0.840249478	5.72232993	-12.20130678	2.66E-27	8.29E-26	51.26429469
BFSP1	-1.15922784	2.20938971	-12.19825446	2.73E-27	8.47E-26	51.24080106
SPAG7	-0.568493309	6.35494552	-12.19047963	2.90E-27	8.98E-26	51.1809651
WDR61	-0.693893772	4.33437721	-12.18950513	2.92E-27	9.03E-26	51.17346595
FHIT	-0.651769175	2.8147588	-12.18757615	2.96E-27	9.15E-26	51.15862218
ARL3	-0.676986218	5.22797047	-12.18693718	2.98E-27	9.18E-26	51.15370536
PLEKHD	-1.134669066	0.88335605	-12.18355316	3.06E-27	9.41E-26	51.12766664
SNRPG	-0.686834485	5.76217043	-12.18256347	3.08E-27	9.47E-26	51.12005173
COX5B	-0.816788148	7.82738517	-12.17624931	3.24E-27	9.92E-26	51.07147291
ELP5	-0.592189673	5.14800672	-12.17560145	3.25E-27	9.96E-26	51.06648887
GOT2	-0.694615915	6.38783317	-12.17360509	3.30E-27	1.01E-25	51.05113118
CYCS	-0.917097628	5.67081891	-12.1731711	3.31E-27	1.01E-25	51.0477927
FUNDC2	-0.567232436	4.3520005	-12.16804248	3.45E-27	1.05E-25	51.00834244
LSM4	-0.625367668	6.29158436	-12.16217784	3.61E-27	1.10E-25	50.96323596
ATP5F1E	-0.923779413	9.17933753	-12.16076196	3.65E-27	1.11E-25	50.95234689

DNAJC1	-0.751008122	6.00284714	-12.15856156	3.71E-27	1.12E-25	50.93542497
TPST1	-0.938440633	3.85488355	-12.15652488	3.77E-27	1.14E-25	50.9197629
CLCNKB	-1.707428755	2.67376947	-12.15511238	3.81E-27	1.15E-25	50.90890117
MRPL16	-0.482649373	4.65368335	-12.14853595	4.01E-27	1.21E-25	50.85833467
ARMC6	-0.596555117	4.85110111	-12.13721072	4.38E-27	1.32E-25	50.77127138
RSPH1	-1.058603471	2.9932251	-12.1261865	4.77E-27	1.43E-25	50.68654282
OST4	-0.639921522	9.02879537	-12.11590264	5.17E-27	1.55E-25	50.6075229
TCEAL3	-0.717257346	5.96009914	-12.11539923	5.19E-27	1.55E-25	50.60365522
DUSP19	-0.823905888	1.75786618	-12.11426708	5.23E-27	1.56E-25	50.5949571
C12orf45	-0.65150217	4.42047199	-12.10298541	5.71E-27	1.70E-25	50.50829381
CFAP99	-0.483655256	0.6170716	-12.10001798	5.84E-27	1.74E-25	50.48550228
FMC1	-0.892034408	5.00463969	-12.09720026	5.97E-27	1.77E-25	50.46386192
ADIPOR2	-0.920901014	5.35698055	-12.09517944	6.07E-27	1.80E-25	50.4483427
CFAP46	-0.824175106	1.3112042	-12.08069155	6.79E-27	2.01E-25	50.33710105
NIT2	-0.478303118	3.78078142	-12.08059161	6.79E-27	2.01E-25	50.33633379
C16orf71	-0.622248941	0.89078847	-12.07890855	6.88E-27	2.03E-25	50.32341314
ATPSCK	-0.719741351	3.80304308	-12.07775566	6.95E-27	2.05E-25	50.31456291
MRPS17	-0.529900386	3.79174667	-12.07613731	7.03E-27	2.07E-25	50.30213981
MYCBP	-0.75708409	4.51874904	-12.07192732	7.27E-27	2.13E-25	50.26982445
SYP	-0.512265951	1.00933431	-12.06939694	7.41E-27	2.17E-25	50.25040309
ARL4D	-1.109462956	6.48869449	-12.06754595	7.52E-27	2.20E-25	50.23619686
LGMN	-1.102198281	6.64042384	-12.05074367	8.56E-27	2.50E-25	50.10726778
CDON	-1.553832331	2.99034141	-12.04210314	9.15E-27	2.67E-25	50.04098526
OTOS	-2.489681862	5.39179479	-12.03696286	9.53E-27	2.77E-25	50.00155975
ZC2HC1(	-0.77496254	3.01398242	-12.03230041	9.88E-27	2.87E-25	49.96580316
CCDC16	-0.915332262	5.04003271	-12.02574231	1.04E-26	3.01E-25	49.9155151
MRS2	-0.665564834	4.09187869	-12.02242617	1.07E-26	3.09E-25	49.89008946
SMIM4	-0.845963265	5.00823143	-12.01878352	1.10E-26	3.17E-25	49.8621627
PCBD1	-0.587561082	7.17051857	-12.01503228	1.13E-26	3.26E-25	49.83340582
GNG11	-1.198322636	4.95315794	-11.99827075	1.29E-26	3.70E-25	49.70494261
CPT2	-0.741728229	4.50553911	-11.9953811	1.31E-26	3.78E-25	49.68280078
BCAP29	-0.967130012	5.12654787	-11.99255318	1.34E-26	3.85E-25	49.66113347
COQ6	-0.456618273	2.63973999	-11.9878656	1.39E-26	3.99E-25	49.62522063
MRPS5	-0.500262981	4.49480852	-11.9786981	1.50E-26	4.26E-25	49.55499711
DIRAS2	-1.161405128	1.13897996	-11.97869729	1.50E-26	4.26E-25	49.55499093
CIB4	-1.873017173	1.36027309	-11.978668	1.50E-26	4.26E-25	49.55476662
MPC2	-0.634621924	5.82741381	-11.97836042	1.50E-26	4.27E-25	49.55241083
THAP11	-0.679633162	5.16704427	-11.96697244	1.64E-26	4.65E-25	49.46519974
TTC30B	-0.84307496	3.07656476	-11.96622154	1.65E-26	4.67E-25	49.45945011
RNF167	-0.590266385	7.09565381	-11.96123282	1.71E-26	4.85E-25	49.42125355

SPRYD4	-0.522815919	3.06028183	-11.95892643	1.74E-26	4.92E-25	49.40359607
C6orf136	-0.57106903	4.63573062	-11.95177103	1.84E-26	5.20E-25	49.34882089
CCDC58	-0.55586364	4.15732858	-11.95098431	1.85E-26	5.22E-25	49.34279905
CYB561I	-0.675730267	4.79685608	-11.94864095	1.89E-26	5.30E-25	49.32486275
NDUFAF	-0.677429226	4.06225958	-11.93311084	2.13E-26	5.97E-25	49.20601894
RAB13	-0.560761409	7.01443679	-11.92696438	2.23E-26	6.25E-25	49.1589952
COMMD(	-0.726320338	6.38420405	-11.92395444	2.28E-26	6.39E-25	49.13597009
SLC25A1	-0.578032005	6.46570104	-11.9223025	2.31E-26	6.46E-25	49.12333394
NRL	-0.656841036	2.20916075	-11.92141025	2.33E-26	6.49E-25	49.11650904
IQCG	-0.693927612	2.09803641	-11.91820546	2.39E-26	6.64E-25	49.09199665
POLDIP2	-0.530962531	7.1338945	-11.91740968	2.40E-26	6.67E-25	49.08591026
TPO	-3.999579536	6.98325197	-11.90794842	2.58E-26	7.17E-25	49.01355627
SNRPC	-0.582362629	7.17593981	-11.90642903	2.61E-26	7.24E-25	49.00193845
IMPA2	-1.235406174	3.84595041	-11.90271663	2.69E-26	7.44E-25	48.97355374
TMED3	-0.673463838	4.74042582	-11.90064953	2.73E-26	7.55E-25	48.95774993
SHISA4	-0.930160813	5.75931786	-11.89816863	2.78E-26	7.68E-25	48.93878347
RBP7	-1.217279328	5.39465831	-11.89413667	2.87E-26	7.91E-25	48.90796165
HPF1	-0.586127886	5.12066954	-11.88672835	3.04E-26	8.36E-25	48.85133731
PPP1R42	-0.603514637	0.67504926	-11.88390346	3.11E-26	8.53E-25	48.82974841
TMEM23	-0.680280319	6.99574092	-11.88282083	3.13E-26	8.59E-25	48.8214749
RIC8B	-0.853607416	3.22706284	-11.88110801	3.18E-26	8.69E-25	48.80838588
NTPCR	-0.633162784	3.32786572	-11.88009689	3.20E-26	8.74E-25	48.80065938
C15orf65	-0.749310262	2.76025466	-11.87562088	3.31E-26	9.04E-25	48.76645797
ARMC7	-0.683586228	4.15490694	-11.8733495	3.37E-26	9.18E-25	48.74910371
PIGBOS1	-0.689879881	5.11661231	-11.86931115	3.48E-26	9.46E-25	48.71825134
P4HB	-0.721424857	8.53312769	-11.86663169	3.55E-26	9.64E-25	48.69778238
HIST1H4	-1.765634535	3.14171055	-11.85558188	3.87E-26	1.05E-24	48.61338444
DEPTOR	-1.618453274	5.71105171	-11.85536161	3.87E-26	1.05E-24	48.61170225
DYNLL2	-0.698826141	6.70029163	-11.85183375	3.98E-26	1.08E-24	48.58476149
MRPL1	-0.529257173	4.78116224	-11.84373606	4.24E-26	1.14E-24	48.52293168
CDK2AP	-0.912846147	7.12244398	-11.83913447	4.39E-26	1.18E-24	48.48780167
C12orf73	-0.6507086	3.29038983	-11.83420241	4.56E-26	1.23E-24	48.45015317
PEX11G	-0.800221912	3.84766056	-11.8210458	5.05E-26	1.35E-24	48.34974528
PPA2	-0.550974601	5.33267697	-11.81728178	5.20E-26	1.39E-24	48.32102522
PSMD9	-0.541916805	3.21108445	-11.81580865	5.25E-26	1.41E-24	48.30978572
TMEM20	-0.77683703	7.66260199	-11.81022072	5.49E-26	1.47E-24	48.26715536
BAD	-0.755656159	6.44602879	-11.8082229	5.57E-26	1.49E-24	48.25191545
UXT	-0.56900395	6.990733	-11.79594646	6.12E-26	1.63E-24	48.15828354
SEC61G	-0.734033094	6.18262034	-11.79463363	6.19E-26	1.64E-24	48.14827235
SAP18	-0.531109663	6.81896534	-11.79259785	6.28E-26	1.67E-24	48.1327488

MCTS1	-0.540386934	4.8626457	-11.78926973	6.45E-26	1.71E-24	48.10737236
PRPSAP	-0.659409844	4.35282253	-11.78514884	6.65E-26	1.76E-24	48.07595404
ZNHIT3	-0.564075503	3.72610016	-11.78203494	6.82E-26	1.80E-24	48.05221538
GSTM3	-1.50222971	4.6410833	-11.77689312	7.09E-26	1.87E-24	48.01302089
CBR1	-1.001719282	5.91368443	-11.77325842	7.29E-26	1.92E-24	47.98531776
STXBP5l	-1.377064584	1.11510041	-11.77307562	7.30E-26	1.92E-24	47.98392453
GRHPR	-0.596451155	5.01699418	-11.77157469	7.39E-26	1.94E-24	47.97248551
NOP10	-0.580871977	8.00571723	-11.74486697	9.07E-26	2.38E-24	47.769009
PPIA	-0.507245498	7.38612701	-11.74321866	9.19E-26	2.40E-24	47.75645554
GRIN2C	-2.102744326	2.26406488	-11.7417753	9.29E-26	2.43E-24	47.74546349
PRELID1	-0.514730965	5.76748526	-11.74130345	9.32E-26	2.43E-24	47.7418701
KCTD8	-1.422805147	1.74649579	-11.73794253	9.57E-26	2.49E-24	47.71627642
ALDOA	-0.621514873	8.76739153	-11.73791934	9.57E-26	2.49E-24	47.71609982
ESD	-0.627236047	6.42502148	-11.73259556	9.97E-26	2.59E-24	47.67556332
IMMT	-0.727032061	5.76112854	-11.72962686	1.02E-25	2.64E-24	47.65296126
LCA5L	-0.635029316	1.66915712	-11.71891881	1.11E-25	2.87E-24	47.57145028
AK2	-0.630352735	5.97707948	-11.71524598	1.14E-25	2.94E-24	47.54349729
DYNLRB	-0.856089883	1.51472787	-11.71286369	1.16E-25	2.99E-24	47.52536774
RRP36	-0.575924186	5.32728997	-11.70741655	1.21E-25	3.12E-24	47.48391834
TIMM13	-0.717836836	6.28254777	-11.70705151	1.21E-25	3.12E-24	47.48114077
NDUFAF	-0.584849573	5.21894232	-11.69933046	1.29E-25	3.31E-24	47.42239873
SMUG1	-0.543097721	3.99609086	-11.69880312	1.29E-25	3.32E-24	47.4183871
UNG	-0.714957312	5.57597489	-11.69239863	1.36E-25	3.48E-24	47.36967091
LLPH	-0.529484114	3.06276956	-11.69174688	1.36E-25	3.49E-24	47.36471384
ZNF346	-0.633985279	3.53198336	-11.68867728	1.40E-25	3.57E-24	47.34136788
PDIA4	-1.19918058	8.4470224	-11.68555889	1.43E-25	3.65E-24	47.31765286
C18orf32	-0.580166764	2.56435777	-11.68481151	1.44E-25	3.66E-24	47.31196935
HDDC2	-0.629016198	5.07246816	-11.68292446	1.46E-25	3.71E-24	47.29761973
IL11RA	-1.064906722	4.3689369	-11.66711364	1.65E-25	4.18E-24	47.17741731
ERH	-0.550179083	7.57203294	-11.66685147	1.65E-25	4.19E-24	47.17542456
MMAB	-0.646563249	4.30761048	-11.66302613	1.70E-25	4.31E-24	47.14634977
NECAB3	-0.886668467	4.45338482	-11.65895241	1.75E-25	4.44E-24	47.1153904
GATB	-0.448068886	2.38895806	-11.65703107	1.78E-25	4.49E-24	47.10078979
MYL6	-0.648344501	9.00249658	-11.65652951	1.79E-25	4.51E-24	47.09697839
NIPSNAI	-0.812907518	5.473136	-11.65537377	1.80E-25	4.54E-24	47.08819621
NFYC	-0.605217428	4.90044267	-11.64445964	1.96E-25	4.93E-24	47.005275
HSD11B:	-1.671638846	2.95251542	-11.64133752	2.01E-25	5.04E-24	46.98155873
CUEDC2	-0.602544348	6.91788671	-11.63898355	2.05E-25	5.12E-24	46.96367878
NAGLU	-0.769721824	6.01438059	-11.63568207	2.10E-25	5.25E-24	46.93860365
COPS6	-0.520606335	6.81879826	-11.63139767	2.17E-25	5.41E-24	46.90606639

RPS19BI	-0.805625767	5.72517493	-11.62983631	2.19E-25	5.46E-24	46.89420976
ATPAF2	-0.483867792	3.56920627	-11.62980439	2.19E-25	5.46E-24	46.89396741
PPIC	-0.762117937	6.56738767	-11.62972896	2.20E-25	5.46E-24	46.89339462
MDH1	-0.629517988	6.3626329	-11.61691381	2.42E-25	6.02E-24	46.79609785
CDK5	-0.540770597	4.02736494	-11.61232335	2.51E-25	6.22E-24	46.76125362
LRRFIP2	-0.57613849	3.65462978	-11.61143372	2.53E-25	6.25E-24	46.75450128
FZD8	-1.112445452	3.33797025	-11.59820908	2.80E-25	6.91E-24	46.65414446
SCO1	-0.69137141	3.23303969	-11.59367152	2.89E-25	7.15E-24	46.61971873
BTNL9	-1.543522547	2.84111202	-11.58961515	2.99E-25	7.36E-24	46.58894721
SEPHS1	-0.681209418	5.79167579	-11.58735379	3.04E-25	7.48E-24	46.57179402
C15orf40	-0.471456002	2.34034771	-11.58295368	3.14E-25	7.72E-24	46.53842066
DIO1	-3.753301549	4.97111922	-11.58071255	3.20E-25	7.85E-24	46.52142391
BEX5	-1.138829862	5.35271582	-11.57683214	3.29E-25	8.07E-24	46.4919973
RNF7	-0.545946048	5.84078339	-11.57619315	3.31E-25	8.10E-24	46.4871519
SIRT5	-0.564899094	3.55253846	-11.57266762	3.40E-25	8.31E-24	46.46041956
ALG1	-0.712642971	3.87024908	-11.56990055	3.47E-25	8.47E-24	46.43944005
RAMP3	-1.17422508	6.45518819	-11.56894175	3.50E-25	8.52E-24	46.43217091
MCEE	-0.590213108	3.80952193	-11.56398569	3.63E-25	8.84E-24	46.39459963
MLF2	-0.500369255	7.48784304	-11.56301545	3.66E-25	8.89E-24	46.38724491
SUMO1	-0.633490318	7.18061061	-11.56214719	3.68E-25	8.94E-24	46.38066345
ANKRD4	-0.833242727	3.47076806	-11.56166611	3.70E-25	8.96E-24	46.37701685
NUDT22	-0.624631384	4.57677696	-11.55779654	3.81E-25	9.21E-24	46.34768736
PDE6D	-0.548311886	3.97265823	-11.55768783	3.81E-25	9.21E-24	46.34686345
KCNAB1	-2.353768012	3.63573583	-11.55687369	3.84E-25	9.26E-24	46.34069304
LSM1	-0.648144356	5.02864457	-11.55515812	3.89E-25	9.36E-24	46.3276912
ADIG	-1.481973972	1.23598405	-11.55407771	3.92E-25	9.43E-24	46.31950331
TMEM80	-0.863146848	4.58039485	-11.5527305	3.96E-25	9.51E-24	46.30929385
METTL1	-0.679853318	4.24473171	-11.55225404	3.97E-25	9.53E-24	46.30568324
NSMCE3	-0.718812994	5.55142844	-11.55120897	4.01E-25	9.60E-24	46.29776377
PSMB6	-0.592724872	7.63704705	-11.5495376	4.06E-25	9.71E-24	46.28509884
BLMH	-0.57175762	4.32494282	-11.54377563	4.24E-25	1.01E-23	46.2414412
MRPS31	-0.556272696	4.8571809	-11.5436961	4.24E-25	1.01E-23	46.2408387
PDE7B	-1.423537778	2.74280705	-11.54142409	4.32E-25	1.03E-23	46.22362589
ID4	-1.269158256	8.23627354	-11.53888079	4.40E-25	1.05E-23	46.20435906
C7orf50	-0.696664962	6.14835861	-11.53767781	4.44E-25	1.06E-23	46.19524632
PSPH	-0.928861761	3.86144944	-11.53667932	4.48E-25	1.06E-23	46.18768286
ZNF781	-0.803192228	1.35566312	-11.53345442	4.59E-25	1.09E-23	46.16325588
MATN2	-1.744107327	5.64803628	-11.52823555	4.78E-25	1.13E-23	46.12373017
THEM6	-0.669783657	5.11579251	-11.52761431	4.80E-25	1.13E-23	46.11902554
SLC27A4	-1.344082836	5.1076206	-11.52537257	4.88E-25	1.15E-23	46.10204943

RAD51C	-0.463368924	2.56390347	-11.52420542	4.93E-25	1.16E-23	46.09321137
MAPRE3	-0.801745666	4.273443	-11.52113148	5.04E-25	1.19E-23	46.0699357
COA5	-0.61216182	5.37350567	-11.51622826	5.24E-25	1.23E-23	46.03281288
FAM200B	-0.591804989	4.2428069	-11.51585392	5.25E-25	1.23E-23	46.02997888
TRUB2	-0.529291921	4.31626421	-11.51576688	5.25E-25	1.23E-23	46.02931998
DYNC2LI	-0.654018402	4.22183501	-11.51526448	5.27E-25	1.23E-23	46.02551659
ELOC	-0.491923941	4.44967273	-11.51341994	5.35E-25	1.25E-23	46.01155296
ZCRB1	-0.60414153	5.88106954	-11.51331529	5.35E-25	1.25E-23	46.01076077
ERO1B	-1.28138027	3.87790547	-11.51317994	5.36E-25	1.25E-23	46.0097362
SNX3	-0.66383095	7.68542511	-11.51274153	5.38E-25	1.25E-23	46.00641742
TCEAL9	-0.780321099	7.21443572	-11.51016232	5.48E-25	1.27E-23	45.98689379
SYNM	-1.090697259	2.5751686	-11.50612504	5.66E-25	1.31E-23	45.95633584
GLT8D1	-0.676378557	5.93294259	-11.50455439	5.72E-25	1.33E-23	45.94444862
ANKRD9	-0.934585166	2.68115229	-11.50415809	5.74E-25	1.33E-23	45.94144938
NAP1L5	-0.9147144	4.28273597	-11.50344404	5.77E-25	1.33E-23	45.93604545
ATRAID	-0.649970208	7.80257626	-11.50098814	5.88E-25	1.36E-23	45.91745989
NFKBIB	-0.557236667	5.05127022	-11.4974893	6.04E-25	1.39E-23	45.89098383
DEFB131	-1.374358191	2.07264287	-11.49176086	6.31E-25	1.45E-23	45.84764161
MPLKIP	-0.538606365	3.39198748	-11.48964968	6.41E-25	1.47E-23	45.83166985
MROH8	-0.647219503	1.90889401	-11.48884117	6.45E-25	1.48E-23	45.82555345
ALDH4A1	-1.252240052	3.20740975	-11.47556732	7.14E-25	1.64E-23	45.72515594
TAF11	-0.584472669	5.56790992	-11.46948865	7.48E-25	1.71E-23	45.67919187
SDHAF2	-0.604287309	4.39654268	-11.46633562	7.66E-25	1.75E-23	45.65535314
ECE2	-0.734709564	1.88344263	-11.46599718	7.68E-25	1.75E-23	45.65279451
C11orf71	-0.71144862	4.48027295	-11.45689128	8.24E-25	1.88E-23	45.583961
ZNF32	-0.606732804	6.2951514	-11.45233477	8.53E-25	1.94E-23	45.54952388
CEP70	-0.734866909	4.41888055	-11.44371237	9.11E-25	2.07E-23	45.48436964
NDUFB7	-0.774337558	8.80064577	-11.4374053	9.56E-25	2.17E-23	45.43672081
ETFBKM	-0.551525347	1.68451819	-11.4362385	9.64E-25	2.19E-23	45.42790678
CYP17A1	-1.12052636	1.45456069	-11.43605231	9.66E-25	2.19E-23	45.42650033
GPER1	-1.295040274	3.78474816	-11.43498687	9.74E-25	2.20E-23	45.41845227
SNUPN	-0.545282881	4.04841261	-11.43260549	9.91E-25	2.24E-23	45.40046473
GPR137	-0.603844195	4.74505865	-11.42425414	1.06E-24	2.38E-23	45.33739315
STK25	-0.745618139	5.2782865	-11.42419108	1.06E-24	2.38E-23	45.33691703
GPAM	-1.404154901	3.19744221	-11.41793489	1.11E-24	2.49E-23	45.28967839
GMDS	-0.692128595	3.70046212	-11.41701865	1.12E-24	2.51E-23	45.28276085
TMEM11	-0.753141653	2.36536287	-11.41283286	1.15E-24	2.59E-23	45.25116064
VPS16	-0.576496342	5.13397839	-11.41011953	1.18E-24	2.64E-23	45.23067869
ATP6V1D	-0.610951804	6.32446858	-11.3990867	1.28E-24	2.86E-23	45.14741179
TIMM22	-0.521273203	4.51303145	-11.39800633	1.29E-24	2.88E-23	45.1392594

SELENO	-0.669719354	6.43042235	-11.39188299	1.35E-24	3.02E-23	45.09305798
HTATIP2	-0.789617533	5.26147515	-11.38985192	1.37E-24	3.06E-23	45.07773507
SMIM26	-0.552503813	6.3923213	-11.38699826	1.40E-24	3.12E-23	45.05620788
ATG4A	-0.639213872	4.37902355	-11.38281427	1.45E-24	3.22E-23	45.02464813
ZC3HC1	-0.492825283	4.03609113	-11.38278854	1.45E-24	3.22E-23	45.02445401
MLYCD	-0.556833498	2.01208306	-11.37896368	1.49E-24	3.31E-23	44.99560649
VEGFB	-0.614023045	7.32926443	-11.37709146	1.51E-24	3.35E-23	44.98148718
TIMM10	-0.670546718	6.36181899	-11.37509156	1.54E-24	3.40E-23	44.96640575
CYP2S1	2.132951421	3.19083729	11.37362171	1.55E-24	3.43E-23	44.95532206
TFCP2L1	-1.966903018	2.86232125	-11.36605445	1.65E-24	3.63E-23	44.89826691
DUS2	-0.496136083	2.75877525	-11.36514551	1.66E-24	3.65E-23	44.89141457
MTRF1L	-0.524179857	2.73522539	-11.36445031	1.67E-24	3.67E-23	44.88617367
TCEANC	-0.48134536	1.71128115	-11.36272974	1.69E-24	3.71E-23	44.87320337
PPFIA3	-0.69696994	1.97919163	-11.36032578	1.72E-24	3.77E-23	44.85508244
NDUFB1(	-0.60305263	7.63568037	-11.35264237	1.82E-24	4.00E-23	44.79717377
SAT2	-0.788782098	6.92319618	-11.34978161	1.86E-24	4.08E-23	44.77561587
SGTA	-0.592156367	6.32281639	-11.34291794	1.96E-24	4.29E-23	44.7239005
SNRPE	-0.637532421	5.96609006	-11.34169963	1.98E-24	4.33E-23	44.714722
LYPLAL1	-0.576053556	4.58454545	-11.3387136	2.03E-24	4.42E-23	44.6922273
BLVRB	-0.666883423	6.53962187	-11.32235852	2.30E-24	5.00E-23	44.56905376
EIF3M	-0.600870766	5.65499102	-11.32098811	2.32E-24	5.04E-23	44.5587356
C10orf14	-0.568089318	2.04531503	-11.32045197	2.33E-24	5.06E-23	44.55469897
WFS1	-1.071873135	5.61568805	-11.31936558	2.35E-24	5.09E-23	44.54651961
TMED4	-0.73586265	7.70023797	-11.3165508	2.40E-24	5.20E-23	44.52532869
GBA2	-0.662216448	5.43928899	-11.31213868	2.48E-24	5.37E-23	44.49211571
SNTA1	-1.098042749	7.13235932	-11.31121697	2.50E-24	5.40E-23	44.48517789
BOD1	-0.705083109	6.25025498	-11.31013634	2.52E-24	5.44E-23	44.47704412
KPTN	-0.642370598	3.04656235	-11.30847497	2.55E-24	5.50E-23	44.46453976
METRN	-1.282000587	4.52754299	-11.30749851	2.57E-24	5.53E-23	44.45719067
HDHD5	-0.620196485	3.63736564	-11.29857726	2.75E-24	5.91E-23	44.39005656
KMT5A	-0.728220927	4.73114114	-11.29845965	2.75E-24	5.91E-23	44.38917169
IQCH	-0.666710437	1.41911574	-11.29522078	2.82E-24	6.05E-23	44.36480299
SOD3	-1.84150259	5.93250373	-11.28574614	3.03E-24	6.49E-23	44.29353079
RAB1A	-0.749642328	7.34176766	-11.28111394	3.14E-24	6.72E-23	44.25869265
G6PC3	-0.629672976	6.09673243	-11.27958131	3.18E-24	6.79E-23	44.247167
SLC18B1	-0.796754594	4.3250982	-11.27602581	3.26E-24	6.96E-23	44.22043103
EXOC3L:	-0.990126241	3.08415695	-11.27283824	3.34E-24	7.13E-23	44.19646413
ATP6V0I	-0.750009971	5.50906849	-11.27018101	3.41E-24	7.26E-23	44.17648646
MRPL54	-0.742855536	6.9543448	-11.26670384	3.50E-24	7.45E-23	44.1503467
ERCC8	-0.50845785	2.34726412	-11.26403421	3.58E-24	7.59E-23	44.13027953

IFT46	-0.602028199	4.71440878	-11.26077657	3.66E-24	7.77E-23	44.10579449
COQ7	-0.57145254	4.24296419	-11.25566367	3.81E-24	8.07E-23	44.0673697
HSD17B4	-0.86704574	6.23320757	-11.25425847	3.85E-24	8.14E-23	44.0568103
MDH1B	-0.873745183	2.08729427	-11.2499803	3.98E-24	8.40E-23	44.02466451
NUDCD2	-0.552007934	3.27769877	-11.2407229	4.27E-24	9.00E-23	43.95511944
BRK1	-0.63980424	8.71849612	-11.23984849	4.30E-24	9.05E-23	43.94855153
RABGEF	-0.611763705	2.34181904	-11.2397493	4.30E-24	9.05E-23	43.94780646
LSM5	-0.513474561	4.21580178	-11.23411934	4.49E-24	9.43E-23	43.90552265
UQCRC1	-0.535867567	7.37963033	-11.23118768	4.59E-24	9.63E-23	43.88350728
SH2D6	-0.976908535	0.88737904	-11.22620074	4.76E-24	9.99E-23	43.84606206
STK16	-0.635007441	4.68643063	-11.22425914	4.83E-24	1.01E-22	43.83148482
MRPS18	-0.564901195	6.91626383	-11.2237914	4.85E-24	1.01E-22	43.82797317
C5orf30	-0.951896098	3.13528563	-11.22378839	4.85E-24	1.01E-22	43.82795062
MOK	-0.696263722	2.05509578	-11.22189902	4.92E-24	1.03E-22	43.81376647
LRRC28	-0.481148883	2.40645928	-11.22115954	4.95E-24	1.03E-22	43.8082152
2-Mar	-0.640525551	6.04557063	-11.21611922	5.14E-24	1.07E-22	43.7703806
DMAC2	-0.492485442	4.86650742	-11.21223416	5.30E-24	1.10E-22	43.74122182
NOL7	-0.506461848	6.48451842	-11.2110449	5.34E-24	1.11E-22	43.73229661
GFOD2	-0.801150922	2.88690333	-11.20962095	5.40E-24	1.12E-22	43.72161061
ZSCAN1	-1.056121842	1.88188681	-11.20839027	5.45E-24	1.13E-22	43.71237532
NUDT16	-0.771502459	4.65484197	-11.20793113	5.47E-24	1.13E-22	43.7089299
NUDT5	-0.513773105	4.90351225	-11.20569501	5.57E-24	1.15E-22	43.69215071
C8orf76	-0.571387278	2.85304407	-11.19592456	5.99E-24	1.24E-22	43.61884943
RPN1	-0.664312001	8.22925932	-11.19449748	6.06E-24	1.25E-22	43.60814477
MBLAC1	-0.761439502	2.53069828	-11.19381652	6.09E-24	1.25E-22	43.60303702
CDH16	-2.533112908	2.52471972	-11.19060255	6.24E-24	1.28E-22	43.57893092
CFAP221	-0.603695736	0.86291526	-11.18604411	6.46E-24	1.33E-22	43.54474487
UST	-1.242272896	5.41093507	-11.18440904	6.54E-24	1.34E-22	43.53248376
ATAD3C	-0.844030536	4.07649593	-11.18359229	6.58E-24	1.35E-22	43.5263594
PGF	-1.844960356	4.97475875	-11.18339006	6.59E-24	1.35E-22	43.52484296
KLHDC9	-0.703952134	4.50477217	-11.18062686	6.73E-24	1.38E-22	43.50412432
PTGR2	-0.618699922	3.11974705	-11.17871586	6.83E-24	1.39E-22	43.48979648
RAC3	-1.010694984	3.13082923	-11.17553817	6.99E-24	1.43E-22	43.46597349
ZMAT4	-2.158210012	1.79834815	-11.17534206	7.00E-24	1.43E-22	43.46450334
ZNF438	-0.586935565	3.80023593	-11.17463479	7.04E-24	1.43E-22	43.45920128
PPCDC	-0.572572975	3.37283554	-11.17021159	7.28E-24	1.48E-22	43.42604554
CETP	-1.414024391	2.69602336	-11.16805221	7.40E-24	1.50E-22	43.40986069
SRI	-0.656515908	5.64315348	-11.16653937	7.49E-24	1.52E-22	43.39852239
PYM1	-0.572748088	5.55220985	-11.16545519	7.55E-24	1.53E-22	43.39039709
SAMD13	-0.886223678	2.86351083	-11.16091731	7.81E-24	1.58E-22	43.35639124

TRAPPC	-0.564399356	4.27141444	-11.15248483	8.33E-24	1.68E-22	43.29321277
TRPT1	-0.57214595	5.43471537	-11.15081221	8.43E-24	1.70E-22	43.28068295
B3GNT3	3.10937549	3.90181137	11.14779063	8.63E-24	1.74E-22	43.25804959
PSMG1	-0.600084237	5.08223824	-11.1452321	8.80E-24	1.77E-22	43.23888637
IDI1	-0.749676453	4.65763819	-11.1398082	9.17E-24	1.84E-22	43.19826667
FKBP3	-0.552974912	5.82035428	-11.1294931	9.91E-24	1.99E-22	43.12103547
GALNT1	-1.68744259	3.17125608	-11.12890801	9.95E-24	2.00E-22	43.11665552
ATP6V1(	-1.064495046	2.2485615	-11.12112732	1.06E-23	2.11E-22	43.05841734
FIS1	-0.68827335	7.8874013	-11.11909506	1.07E-23	2.14E-22	43.04320823
MT1G	-4.136679141	7.30760668	-11.11665085	1.09E-23	2.18E-22	43.02491742
CYB5A	-0.633010475	2.79471574	-11.11604904	1.10E-23	2.19E-22	43.02041414
PCCB	-0.835393	4.31505058	-11.11417454	1.11E-23	2.22E-22	43.00638785
PAFAH2	-0.750619141	4.87841499	-11.11389612	1.11E-23	2.22E-22	43.00430459
MED8	-0.628021663	5.4523983	-11.11383645	1.12E-23	2.22E-22	43.00385809
PRUNE1	-0.912565034	5.4887262	-11.11306571	1.12E-23	2.23E-22	42.99809121
LIPG	-1.681818568	5.67894999	-11.11200094	1.13E-23	2.24E-22	42.99012458
RBPMS2	-1.041736683	4.29859579	-11.11154898	1.13E-23	2.25E-22	42.98674304
ALDH2	-0.980815602	4.86720407	-11.11100317	1.14E-23	2.26E-22	42.98265943
PSMB5	-0.584040611	7.12379627	-11.10743437	1.17E-23	2.31E-22	42.95596019
EEF2KM	-0.588065832	3.26656972	-11.10616606	1.18E-23	2.33E-22	42.9464723
SLC2A8	-0.704122769	3.71083239	-11.10323831	1.21E-23	2.38E-22	42.924572
ATP5MC	-0.498568775	7.37472486	-11.10252898	1.21E-23	2.39E-22	42.9192663
CCDC11(	-0.879298511	1.71108163	-11.10131403	1.23E-23	2.41E-22	42.91017901
FAM3A	-0.640158865	5.37754609	-11.0965552	1.27E-23	2.50E-22	42.87458818
PRDX1	-0.863429517	9.4824939	-11.09119285	1.32E-23	2.60E-22	42.83449008
MAPK10	-0.887926162	2.8122836	-11.0886706	1.35E-23	2.65E-22	42.81563176
ZCCHC1	-0.495784298	5.67249142	-11.08481591	1.39E-23	2.72E-22	42.78681391
ADH5	-0.697637059	5.90160042	-11.08460571	1.39E-23	2.72E-22	42.78524251
UQCRHL	-0.489291326	1.53941549	-11.08275946	1.41E-23	2.76E-22	42.77144119
TRMO	-0.637631828	3.56150186	-11.08234957	1.41E-23	2.76E-22	42.76837725
PIK3R3	-0.989439214	4.05480179	-11.0774423	1.47E-23	2.86E-22	42.73169814
IFT43	-0.669059779	4.36961774	-11.07732125	1.47E-23	2.86E-22	42.73079344
FOXRED	-0.488008082	3.37410975	-11.0725336	1.52E-23	2.96E-22	42.69501406
RBKS	-0.663316528	3.39714686	-11.07192585	1.53E-23	2.97E-22	42.69047256
RWDD2E	-0.608677827	4.21059275	-11.06934032	1.56E-23	3.03E-22	42.67115278
DDOST	-0.70401948	8.04318858	-11.0686156	1.57E-23	3.04E-22	42.66573781
GMPPA	-0.637386616	4.4862046	-11.05282768	1.77E-23	3.42E-22	42.5478033
COPS5	-0.479142666	4.23886729	-11.047752	1.84E-23	3.55E-22	42.50990089
SPEF1	-0.955917459	2.09256732	-11.04737788	1.84E-23	3.56E-22	42.50710741
NDUFB9	-0.558561445	7.2535846	-11.04246915	1.91E-23	3.69E-22	42.47045797

TMEM25	-0.673217689	5.03147973	-11.04214771	1.92E-23	3.69E-22	42.46805822
JAZF1	-0.91245238	4.43747585	-11.04099881	1.93E-23	3.72E-22	42.45948126
GPS1	-0.525741104	5.64710151	-11.03896011	1.96E-23	3.78E-22	42.44426231
RABL3	-0.756095565	4.22889117	-11.03433124	2.03E-23	3.90E-22	42.40971145
RTF2	-0.535816373	6.12871866	-11.03252898	2.06E-23	3.95E-22	42.39626036
ELMO1	-1.636922562	3.72919091	-11.0279389	2.13E-23	4.09E-22	42.36200605
AHNAK2	1.917563419	2.85456287	11.0235309	2.21E-23	4.22E-22	42.3291152
CCDC15	-0.660736971	1.84702658	-11.02153591	2.24E-23	4.28E-22	42.31423089
MAIP1	-0.611930346	4.54304612	-11.01550853	2.34E-23	4.47E-22	42.26926731
CLCNKA	-1.900454779	1.87761291	-11.01528338	2.35E-23	4.48E-22	42.2675879
BCL2	-1.271559747	4.5986378	-11.01280083	2.39E-23	4.56E-22	42.249071
HDAC11	-0.604414898	4.52662356	-11.01135309	2.42E-23	4.60E-22	42.23827325
GEMIN6	-0.483203144	3.09522614	-11.01096155	2.42E-23	4.61E-22	42.23535303
ZNF580	-0.797622323	5.20575667	-11.00777194	2.48E-23	4.72E-22	42.21156593
MINDY1	-0.91548801	5.38660101	-11.00767061	2.49E-23	4.72E-22	42.21081026
MPV17L2	-0.588152028	4.42181941	-11.0068221	2.50E-23	4.74E-22	42.20448276
CASC1	-0.830851898	2.33258017	-11.00423791	2.55E-23	4.83E-22	42.18521298
MRPL11	-0.555918529	5.0500055	-11.00269566	2.58E-23	4.88E-22	42.17371355
DYRK4	-0.528230961	3.58673614	-11.00220237	2.59E-23	4.89E-22	42.17003558
ZMAT5	-0.632175076	4.00129128	-10.99974045	2.64E-23	4.98E-22	42.15168028
ATG4D	-0.587986924	4.42807713	-10.99723864	2.69E-23	5.07E-22	42.13302905
AKR7A2	-0.643982084	6.09080143	-10.9954815	2.72E-23	5.13E-22	42.11993034
MRPL27	-0.6364807	5.37404921	-10.99194641	2.80E-23	5.26E-22	42.09357993
TSPAN7	-1.479414845	4.52817106	-10.99003305	2.84E-23	5.33E-22	42.07931911
YKT6	-0.565986847	6.06784573	-10.98335528	2.98E-23	5.60E-22	42.02955475
CCDC87	-0.622957584	1.17510093	-10.98018435	3.06E-23	5.73E-22	42.00592796
FAM50B	-0.811366417	3.70142145	-10.97957012	3.07E-23	5.75E-22	42.00135155
ANKRD5	-0.651930707	4.38374207	-10.97644621	3.14E-23	5.88E-22	41.97807798
SUOX	-0.631106056	4.41538687	-10.97290475	3.23E-23	6.03E-22	41.95169638
RCAN2	-1.357814805	4.05269591	-10.97123617	3.27E-23	6.10E-22	41.93926762
LYRM7	-0.758515369	3.41779056	-10.96659442	3.39E-23	6.31E-22	41.90469613
DGAT2	-1.690539251	3.48324945	-10.96654912	3.39E-23	6.31E-22	41.90435876
CBX8	-0.674618929	2.79515692	-10.96516465	3.42E-23	6.37E-22	41.89404828
PCCA	-0.719002344	3.82201464	-10.96421424	3.45E-23	6.40E-22	41.88697063
SRP9	-0.713071893	7.59610177	-10.95817068	3.61E-23	6.69E-22	41.84196983
AUH	-0.73622713	5.04526698	-10.95759432	3.62E-23	6.72E-22	41.83767874
SWSAP1	-0.577048276	3.17876447	-10.95564653	3.68E-23	6.81E-22	41.82317746
SLC2A4F	-0.683342205	6.26108613	-10.95532198	3.69E-23	6.82E-22	41.82076133
NSMCE4	-0.52218989	4.4814137	-10.9517257	3.79E-23	7.00E-22	41.79398984
CHCHD6	-0.661581957	3.9531176	-10.95145689	3.79E-23	7.00E-22	41.79198886

TTC30A	-0.889011896	2.64863707	-10.94894576	3.87E-23	7.13E-22	41.77329754
C17orf97	-0.806045929	3.68036475	-10.94597674	3.95E-23	7.28E-22	41.75119995
NDUFV1	-0.588996703	6.504199	-10.94536323	3.97E-23	7.31E-22	41.74663397
GRIK4	-1.28867269	1.21050387	-10.94342031	4.03E-23	7.41E-22	41.73217487
RILPL1	-0.873228936	3.57525527	-10.94244776	4.06E-23	7.45E-22	41.72493751
EBNA1BI	-0.614750997	5.51809106	-10.93954618	4.15E-23	7.61E-22	41.7033464
IVD	-0.821473314	6.44285622	-10.93933004	4.16E-23	7.61E-22	41.70173815
TMEM14	-0.754955944	5.63876112	-10.93072186	4.43E-23	8.11E-22	41.63769593
ASL	-0.73241525	4.84227516	-10.9273173	4.55E-23	8.31E-22	41.61237206
C22orf39	-0.656045317	3.89226505	-10.92394754	4.67E-23	8.52E-22	41.58730984
TMEM38	-1.067385032	2.40392429	-10.91940911	4.83E-23	8.80E-22	41.55356014
PSMC5	-0.545315297	6.68302782	-10.91612802	4.95E-23	9.01E-22	41.52916375
FGFBP3	-0.705979735	2.39839054	-10.91611897	4.95E-23	9.01E-22	41.52909647
UBXN8	-0.748817576	3.96420671	-10.9152114	4.98E-23	9.06E-22	41.52234869
MVD	-0.697143539	4.25160009	-10.91182473	5.11E-23	9.28E-22	41.49717079
IMP4	-0.430913849	5.16137629	-10.90908233	5.22E-23	9.46E-22	41.47678476
HIGD1A	-0.65515597	6.63256976	-10.90628822	5.33E-23	9.65E-22	41.45601621
TCEAL4	-0.584954948	7.29650307	-10.90432687	5.41E-23	9.79E-22	41.44143871
DTD2	-0.69621916	3.49074076	-10.90329263	5.45E-23	9.85E-22	41.43375223
CCDC10	-0.632192173	3.35944663	-10.90191998	5.51E-23	9.94E-22	41.42355106
PSMA4	-0.509383259	5.20550708	-10.89619113	5.75E-23	1.04E-21	41.38098083
BCAP31	-0.622970447	8.19954309	-10.89455972	5.82E-23	1.05E-21	41.36885955
NPRL2	-0.543123085	4.22481532	-10.89330882	5.87E-23	1.06E-21	41.3595659
NQO1	-1.708603624	6.3416244	-10.88997942	6.02E-23	1.08E-21	41.33483179
UBL7	-0.558960234	6.2761565	-10.88455275	6.27E-23	1.13E-21	41.2945229
SLC15A2	-1.242883306	2.85850324	-10.88019309	6.48E-23	1.16E-21	41.262145
STAC	1.411965617	1.73036716	10.87603576	6.69E-23	1.20E-21	41.23127407
C8orf33	-0.723650755	5.50301502	-10.87109803	6.94E-23	1.24E-21	41.19461379
AMZ2	-0.49092062	5.42449247	-10.86888592	7.05E-23	1.26E-21	41.17819193
AFG1L	-0.412798787	1.50791797	-10.86211058	7.42E-23	1.33E-21	41.12790195
RBFA	-0.472115845	3.61591947	-10.85618793	7.76E-23	1.38E-21	41.0839504
TRAPPC	-0.518264433	3.56334719	-10.85617432	7.76E-23	1.38E-21	41.08384941
CARS	-0.583064016	4.35073396	-10.85559688	7.79E-23	1.39E-21	41.07956478
CISD1	-0.647368431	4.26334048	-10.85258352	7.97E-23	1.42E-21	41.05720668
ERAL1	-0.50350561	5.74233734	-10.85050348	8.10E-23	1.44E-21	41.04177485
ACP1	-0.614726292	5.76365433	-10.85013656	8.12E-23	1.44E-21	41.03905273
BBS4	-0.773846023	4.05943289	-10.8496993	8.15E-23	1.45E-21	41.03580886
AIFM2	-0.697810911	4.16798433	-10.84568935	8.39E-23	1.49E-21	41.00606287
NDUFAF	-0.39016592	2.09968497	-10.84476078	8.45E-23	1.50E-21	40.99917526
WDR25	-0.711438034	3.87669147	-10.84345932	8.54E-23	1.51E-21	40.98952211

KCNIP3	-1.351657477	3.62929319	-10.83845274	8.86E-23	1.57E-21	40.95239154
PHKB	-0.839193727	4.45694308	-10.83476968	9.11E-23	1.61E-21	40.92508067
CHCHD1	-1.179100569	6.37315786	-10.83168516	9.32E-23	1.64E-21	40.90221072
POMP	-0.555559412	7.43940822	-10.82899782	9.51E-23	1.68E-21	40.88228765
PPARGC	-1.627442764	2.60074729	-10.82734209	9.63E-23	1.69E-21	40.87001348
GABARA	-0.591672514	6.87155142	-10.82671458	9.68E-23	1.70E-21	40.86536182
BUD31	-0.514074434	5.90903392	-10.82520332	9.79E-23	1.72E-21	40.85415952
C1orf56	-0.610277867	4.55518911	-10.82326358	9.93E-23	1.74E-21	40.83978183
KLC4	-0.564528461	4.27293186	-10.82160481	1.01E-22	1.76E-21	40.82748751
NUDT8	-0.773835372	4.35762522	-10.81769135	1.04E-22	1.81E-21	40.79848489
MAGEH1	-0.841985273	5.76090567	-10.81736734	1.04E-22	1.81E-21	40.79608385
FLYWCH	-0.58495479	5.84618903	-10.81628865	1.05E-22	1.83E-21	40.78809046
MPI	-0.543909092	3.9657793	-10.8153739	1.05E-22	1.84E-21	40.78131212
THNSL1	-0.727125469	3.56298614	-10.81433633	1.06E-22	1.85E-21	40.77362398
FECH	-0.751245166	4.06713394	-10.81316191	1.07E-22	1.87E-21	40.7649221
TMEM18	-0.57664722	4.42619551	-10.81058669	1.09E-22	1.90E-21	40.74584223
ZSWIM7	-0.674668754	4.82999017	-10.80873548	1.11E-22	1.92E-21	40.73212764
GLRX2	-0.510331661	3.10641911	-10.8069156	1.12E-22	1.95E-21	40.71864596
FCGBP	-1.638205879	6.62797565	-10.80521948	1.14E-22	1.97E-21	40.70608187
HAGH	-0.512898517	4.72053019	-10.8042209	1.15E-22	1.98E-21	40.69868519
PLVAP	-1.312266015	8.8335876	-10.80388847	1.15E-22	1.99E-21	40.69622286
ATP6V1H	-0.595029339	5.38844086	-10.8028428	1.16E-22	2.00E-21	40.68847769
UBE2M	-0.506181927	6.86069328	-10.79784642	1.20E-22	2.07E-21	40.65147403
1-Mar	-1.336472943	3.93862265	-10.79466637	1.23E-22	2.12E-21	40.62792559
POR	-0.873392099	6.84390831	-10.78903862	1.28E-22	2.21E-21	40.58625809
COX6B1	-0.610802691	8.86511895	-10.78641859	1.31E-22	2.25E-21	40.56686228
SERPINE	-0.567468841	6.28757199	-10.78546321	1.32E-22	2.27E-21	40.55979013
LMAN2	-0.700523669	8.19127532	-10.78088892	1.36E-22	2.34E-21	40.5259325
COPS4	-0.744791779	5.16974092	-10.775993	1.41E-22	2.43E-21	40.48970018
MRM3	-0.548009443	4.24645591	-10.77418484	1.43E-22	2.46E-21	40.47632041
EMD	-0.531235645	6.91483606	-10.77179723	1.46E-22	2.50E-21	40.45865422
RAB17	-0.821773136	4.64139824	-10.77092429	1.47E-22	2.51E-21	40.45219559
C2orf74	-0.742479857	3.15671469	-10.7641419	1.55E-22	2.64E-21	40.40202137
ZNF763	-0.613188155	2.17147652	-10.76203891	1.57E-22	2.68E-21	40.3864664
PEX2	-0.644280328	4.83072089	-10.76185953	1.57E-22	2.68E-21	40.38513971
ABHD6	-0.80266277	3.36675694	-10.76006673	1.59E-22	2.72E-21	40.37188005
COPE	-0.56860181	7.31702093	-10.75697332	1.63E-22	2.78E-21	40.34900303
TMEM23	-0.462216723	0.8709223	-10.75264149	1.68E-22	2.87E-21	40.31697149
DUSP15	-1.081275454	2.20057175	-10.74743837	1.75E-22	2.98E-21	40.27850372
SLIRP	-0.599009383	5.14785768	-10.74498649	1.78E-22	3.03E-21	40.2603788

SMIM30	-0.616327456	6.00409638	-10.74115671	1.84E-22	3.11E-21	40.23207127
MRPL40	-0.879210895	6.77095785	-10.73828732	1.87E-22	3.18E-21	40.21086483
ERP44	-0.674157658	5.90970689	-10.73744626	1.89E-22	3.19E-21	40.20464938
METTL2:	-0.566136681	3.03616114	-10.73698928	1.89E-22	3.20E-21	40.20127229
FN1	3.365305375	9.07048436	10.7293232	2.00E-22	3.39E-21	40.14462863
ATG4C	-0.730058282	3.80409154	-10.72596362	2.06E-22	3.47E-21	40.11980994
ATP6V0E	-1.157787186	6.03613254	-10.72556474	2.06E-22	3.48E-21	40.11686346
ST6GALI	2.146533241	2.14818163	10.72367523	2.09E-22	3.52E-21	40.10290634
ARIH2OS	-0.658765335	2.47633041	-10.72319828	2.10E-22	3.53E-21	40.09938339
ISCA1	-0.703803994	4.90779546	-10.72192543	2.12E-22	3.56E-21	40.08998201
MZT2B	-0.926191874	7.07373513	-10.72078252	2.14E-22	3.59E-21	40.08154068
EMCN	-1.210471142	4.08102145	-10.71764894	2.19E-22	3.67E-21	40.05839842
FAM192/	-0.480512612	5.60856018	-10.71696796	2.20E-22	3.68E-21	40.05336949
ACAT1	-0.960389485	4.97923892	-10.71503246	2.23E-22	3.73E-21	40.03907698
RILPL2	-0.799374588	4.90620021	-10.71438007	2.24E-22	3.75E-21	40.03425966
PGD	-0.746898587	6.95042323	-10.71282151	2.27E-22	3.79E-21	40.02275159
ADGRV1	-0.872234036	0.86406779	-10.71106273	2.30E-22	3.84E-21	40.00976593
BCAS2	-0.617919602	6.27874866	-10.7053723	2.40E-22	4.00E-21	39.96775692
MMACH(	-0.52215993	2.54651838	-10.70170515	2.46E-22	4.10E-21	39.94068911
SELENO	-0.59379478	5.5029569	-10.69998848	2.50E-22	4.15E-21	39.92801926
CXXC4	-1.336300255	2.24618791	-10.69856172	2.52E-22	4.19E-21	39.91748972
DCTPP1	-0.785692184	5.17335958	-10.69769758	2.54E-22	4.22E-21	39.91111252
RABEPK	-0.530934384	3.85243761	-10.69609282	2.57E-22	4.26E-21	39.89927034
CPQ	-1.146412305	7.26724785	-10.69175937	2.65E-22	4.40E-21	39.8672953
UBL5	-0.634867398	7.52901596	-10.69030052	2.68E-22	4.44E-21	39.85653204
POLB	-0.464942228	3.38131305	-10.68957269	2.70E-22	4.46E-21	39.8511624
ACACB	-0.927252285	2.78367751	-10.67448434	3.02E-22	4.99E-21	39.73987771
MAGOH	-0.570704471	5.85188304	-10.6738359	3.03E-22	5.01E-21	39.73509644
KCNE3	-1.459385734	3.30136992	-10.67265083	3.06E-22	5.05E-21	39.72635862
LRP1B	-1.141938903	0.7650378	-10.67223189	3.07E-22	5.06E-21	39.72326977
PYGM	-1.547759841	1.81497545	-10.67107907	3.10E-22	5.10E-21	39.71477027
ARFIP2	-0.604141691	5.18697983	-10.67087007	3.10E-22	5.10E-21	39.71322939
C6orf52	-0.76619614	1.23630523	-10.67010396	3.12E-22	5.12E-21	39.70758119
SLC39A1	-0.915222531	4.69990478	-10.66913792	3.14E-22	5.16E-21	39.70045933
HYOU1	-1.3542628	6.04102774	-10.66768094	3.17E-22	5.21E-21	39.68971849
TOMM22	-0.552472806	6.25786467	-10.66726139	3.18E-22	5.22E-21	39.68662574
ELP4	-0.543716488	3.62804395	-10.66660545	3.20E-22	5.24E-21	39.6817904
DPY30	-0.633763241	5.78051879	-10.66523258	3.23E-22	5.29E-21	39.67167054
SNRPN	-0.813762221	7.54481398	-10.66166167	3.32E-22	5.43E-21	39.64535064
APRT	-0.579330244	7.15214779	-10.65740979	3.43E-22	5.59E-21	39.61401593

TPST2	-0.98375178	5.10767668	-10.65706883	3.44E-22	5.60E-21	39.61150338
TMEM17	-0.566664509	3.46927078	-10.656108	3.46E-22	5.64E-21	39.60442315
COLEC1	-1.347877952	2.13735488	-10.6549499	3.49E-22	5.68E-21	39.59588961
C4orf33	-0.729409963	2.40808831	-10.65184125	3.57E-22	5.81E-21	39.57298508
NUDT7	-0.670302954	2.81447138	-10.65173168	3.58E-22	5.81E-21	39.57217782
CGNL1	-1.526281144	4.85119076	-10.65014113	3.62E-22	5.87E-21	39.56045973
ARL16	-0.669504196	5.09207904	-10.64734684	3.69E-22	5.99E-21	39.53987484
ZNF599	-0.545995294	2.35677777	-10.64611136	3.73E-22	6.04E-21	39.53077398
LAMTOR	-0.743128425	6.70978917	-10.64522814	3.75E-22	6.07E-21	39.52426822
MRPS21	-0.550919031	7.13420984	-10.64428812	3.78E-22	6.11E-21	39.51734432
UBE3D	-0.399887746	1.5344292	-10.64399408	3.79E-22	6.12E-21	39.51517857
INSL3	0.919021917	0.9956942	10.63737057	3.98E-22	6.42E-21	39.46639888
DUSP22	-0.523406514	4.52927068	-10.63516369	4.04E-22	6.52E-21	39.45014862
FBXO8	-0.645895943	4.77069985	-10.63403604	4.08E-22	6.57E-21	39.44184573
SPAAR	-0.809545951	1.73198794	-10.63384581	4.08E-22	6.57E-21	39.44044506
NME4	-0.72304945	5.73196624	-10.63345351	4.10E-22	6.59E-21	39.4375567
HLF	-1.635816319	3.08830164	-10.62974905	4.21E-22	6.76E-21	39.41028362
STRAP	-0.693405708	7.19270519	-10.62766907	4.28E-22	6.86E-21	39.39497196
DNAJC3(	-0.507740713	5.11277153	-10.62661286	4.31E-22	6.91E-21	39.38719716
TRIM16L	-0.932989696	2.19078675	-10.62636751	4.32E-22	6.92E-21	39.38539123
ALAS1	-0.738138973	5.91532261	-10.62480796	4.37E-22	6.99E-21	39.37391196
FAM32A	-0.464968736	6.7279549	-10.62289066	4.43E-22	7.08E-21	39.35980034
PARL	-0.528781496	6.00361667	-10.62249236	4.44E-22	7.10E-21	39.35686892
OXNAD1	-0.483258257	2.93557987	-10.61969061	4.54E-22	7.24E-21	39.33624968
MRPL57	-0.553426661	6.21626445	-10.61880937	4.57E-22	7.28E-21	39.32976468
C12orf57	-0.720340253	6.76622925	-10.61805509	4.59E-22	7.32E-21	39.32421417
CDC26	-0.630678567	4.1749627	-10.61253942	4.79E-22	7.61E-21	39.28363058
KLHDC8I	-0.692774934	6.25372292	-10.60629991	5.01E-22	7.97E-21	39.23773096
COA1	-0.487846909	3.19726322	-10.60597418	5.02E-22	7.97E-21	39.23533505
IGSF22	-0.54348837	1.54723495	-10.60595425	5.03E-22	7.97E-21	39.23518849
BCL7B	-0.465458942	5.60887308	-10.60579243	5.03E-22	7.98E-21	39.23399822
SNN	-0.77347076	5.63428699	-10.60498406	5.06E-22	8.02E-21	39.22805247
MED7	-0.498402539	3.9047119	-10.60068822	5.23E-22	8.27E-21	39.19645856
TMEM18	-0.55950929	4.05674917	-10.59601314	5.41E-22	8.55E-21	39.16208114
PPIF	-0.961957897	5.73093341	-10.59485489	5.46E-22	8.62E-21	39.15356505
JMJD8	-0.682478624	6.14393472	-10.59402671	5.49E-22	8.66E-21	39.14747609
STKLD1	-0.420634245	0.94302843	-10.59398854	5.49E-22	8.66E-21	39.14719541
ZNF684	-0.548415849	1.96530481	-10.5926363	5.55E-22	8.74E-21	39.13725382
FBXO9	-0.792797838	4.23093031	-10.59158852	5.59E-22	8.80E-21	39.1295509
DNAJC2!	-0.641740708	3.57619179	-10.59145405	5.60E-22	8.80E-21	39.12856232

STX18	-0.462820061	3.91959115	-10.58997114	5.66E-22	8.89E-21	39.11766103
PSMD6	-0.45523457	4.26033172	-10.58764471	5.76E-22	9.03E-21	39.10056
PITHD1	-0.555607412	6.05103463	-10.58516437	5.87E-22	9.19E-21	39.08232926
FAXDC2	-1.111501613	4.06588533	-10.58360984	5.93E-22	9.29E-21	39.07090408
RDH14	-0.574761144	4.97727191	-10.58348834	5.94E-22	9.29E-21	39.07001115
FAM53A	-0.56682671	1.66315795	-10.57966373	6.11E-22	9.55E-21	39.04190476
DCUN1D	-0.579887521	4.35928409	-10.5795614	6.11E-22	9.55E-21	39.04115285
PPM1L	-1.229977362	2.86694629	-10.57625944	6.27E-22	9.78E-21	39.01689062
DGLUCY	-0.599447822	4.45081607	-10.57448044	6.35E-22	9.90E-21	39.00382
ANAPC1	-0.621177757	5.46264263	-10.57230367	6.45E-22	1.01E-20	38.98782815
MRPS15	-0.475688355	5.66640274	-10.57158084	6.49E-22	1.01E-20	38.9825181
ACSS1	-0.775409514	4.3026737	-10.57089421	6.52E-22	1.01E-20	38.97747411
CSRP2	-0.915460416	3.30786268	-10.56793924	6.67E-22	1.04E-20	38.95576825
TMPRSS	2.99301171	3.37259789	10.56422994	6.85E-22	1.06E-20	38.92852481
LHPP	-0.788745178	5.6564938	-10.56414931	6.86E-22	1.06E-20	38.92793266
MTERF4	-0.689674803	3.44970788	-10.56039899	7.05E-22	1.09E-20	38.90039182
FTCDNL	-0.871595236	2.15650103	-10.55813669	7.17E-22	1.11E-20	38.88378025
CIAPIN1	-0.589531056	4.73582283	-10.55615207	7.28E-22	1.12E-20	38.86920873
PDIA6	-1.261193631	7.29064303	-10.55484574	7.35E-22	1.13E-20	38.85961792
CYB5R1	-0.602553114	5.24906941	-10.55074555	7.57E-22	1.17E-20	38.82951833
LYRM2	-0.600902886	4.11706735	-10.54978484	7.63E-22	1.18E-20	38.8224664
CCDC14	-0.642923691	2.79668774	-10.54949796	7.64E-22	1.18E-20	38.82036069
MTCH2	-0.569184054	6.28213717	-10.54775081	7.74E-22	1.19E-20	38.80753673
POLR1C	-0.562797182	4.90237971	-10.54244818	8.05E-22	1.24E-20	38.76862113
UQCRC2	-0.681686893	6.28921021	-10.53942815	8.24E-22	1.27E-20	38.7464607
EBF4	-1.974133568	4.22116623	-10.53871212	8.28E-22	1.27E-20	38.74120702
DHRS12	-0.52603206	4.12538946	-10.53855225	8.29E-22	1.27E-20	38.74003397
CCNC	-0.679587356	4.90518851	-10.53843375	8.30E-22	1.27E-20	38.73916452
ZC3H8	-0.625124407	2.49537018	-10.53575821	8.46E-22	1.30E-20	38.71953474
MYDGF	-0.666231465	7.2307041	-10.53476312	8.53E-22	1.30E-20	38.71223451
SAMM50	-0.610754353	4.24388786	-10.53166903	8.72E-22	1.33E-20	38.6895372
TMEM19	-0.467544835	3.58252675	-10.53140091	8.74E-22	1.33E-20	38.6875705
PKNOX2	-1.946882814	2.15974243	-10.52776666	8.98E-22	1.37E-20	38.66091436
HDHD3	-0.623447407	5.78067695	-10.52566064	9.12E-22	1.39E-20	38.64546896
MED9	-0.50821328	3.83585055	-10.52436124	9.21E-22	1.40E-20	38.63593987
NTHL1	-0.75321182	4.00061411	-10.52417532	9.22E-22	1.40E-20	38.63457649
PTS	-0.555211766	4.3549506	-10.52384245	9.25E-22	1.41E-20	38.63213549
UFC1	-0.489856869	7.08355887	-10.51866239	9.61E-22	1.46E-20	38.59415314
ASB8	-0.559920192	4.58068933	-10.51389204	9.95E-22	1.51E-20	38.55918137
FAM136	-0.530852067	5.30305975	-10.5112628	1.01E-21	1.54E-20	38.53990896

VPS45	-0.530861314	4.42481627	-10.51034678	1.02E-21	1.55E-20	38.53319497
POLR3B	-0.673655563	3.03832595	-10.51028157	1.02E-21	1.55E-20	38.53271701
SUPT4H1	-0.485680495	6.40107894	-10.51000125	1.02E-21	1.55E-20	38.53066239
AP4S1	-0.570325212	2.78216882	-10.50822507	1.04E-21	1.57E-20	38.5176446
SYS1	-0.546200304	4.88150695	-10.50405537	1.07E-21	1.62E-20	38.48708789
PYY	-0.724555971	0.96987902	-10.50390664	1.07E-21	1.62E-20	38.48599805
C1orf53	-0.83258305	3.17996475	-10.50359449	1.07E-21	1.62E-20	38.48371077
TKFC	-0.585225678	4.37354649	-10.50194368	1.09E-21	1.64E-20	38.47161472
RGS16	-1.386335455	6.18824385	-10.50002601	1.10E-21	1.66E-20	38.45756418
HABP4	-0.622894134	4.244067	-10.49674988	1.13E-21	1.70E-20	38.43356284
LRRC45	-0.697231083	3.62198436	-10.49037142	1.18E-21	1.78E-20	38.38684182
RCCD1	-0.538970945	2.54339286	-10.48977412	1.19E-21	1.78E-20	38.38246728
COX10	-0.595992248	3.48682647	-10.48617497	1.22E-21	1.83E-20	38.35610972
UROD	-0.545272491	6.58308602	-10.48528448	1.23E-21	1.84E-20	38.349589
SPIN2B	-0.50467502	2.47738957	-10.47023406	1.38E-21	2.06E-20	38.23941328
DUSP28	-0.492132414	2.3205286	-10.46689883	1.41E-21	2.11E-20	38.21500641
MRPL52	-0.593326055	5.28413673	-10.46631651	1.42E-21	2.11E-20	38.21074541
ARV1	-0.585857026	5.14263114	-10.46627635	1.42E-21	2.11E-20	38.21045154
SCFD1	-0.574177454	4.7973141	-10.46514098	1.43E-21	2.13E-20	38.20214396
WDR83O	-0.597877458	7.04447414	-10.46064026	1.48E-21	2.20E-20	38.16921525
UQCC3	-0.732483792	4.4503948	-10.45679919	1.52E-21	2.26E-20	38.14111727
SLC25A3	-0.829989548	6.69459457	-10.4560574	1.53E-21	2.27E-20	38.13569143
POLR2L	-0.696230336	8.01606901	-10.45564324	1.53E-21	2.27E-20	38.13266211
FARSB	-0.484814841	4.94738363	-10.45559164	1.53E-21	2.27E-20	38.13228472
KCNQ3	1.534645809	2.39707868	10.45301885	1.56E-21	2.32E-20	38.11346746
TOMM7	-0.631723722	7.85282922	-10.45042797	1.59E-21	2.36E-20	38.09451989
CYSTM1	-0.610613085	7.32701324	-10.44877403	1.61E-21	2.39E-20	38.08242524
COPRS	-0.714879359	6.79880831	-10.44707912	1.63E-21	2.41E-20	38.07003181
CZIB	-0.533070418	4.85975846	-10.44665424	1.64E-21	2.42E-20	38.06692514
PRDX3	-0.698775855	7.21702033	-10.44375983	1.67E-21	2.47E-20	38.045763
RPL6	-0.582669167	8.77048626	-10.44351646	1.68E-21	2.47E-20	38.04398374
STX5	-0.608315988	6.29751379	-10.44197135	1.69E-21	2.50E-20	38.03268791
CCDC14	-1.078346626	2.88002395	-10.44175379	1.70E-21	2.50E-20	38.03109748
ATG3	-0.5466585	4.80159024	-10.44168977	1.70E-21	2.50E-20	38.03062946
PSMG4	-0.547517485	2.49084757	-10.44160915	1.70E-21	2.50E-20	38.03004012
DCLK2	-0.951170964	2.56482398	-10.4384133	1.74E-21	2.55E-20	38.00667868
RPP14	-0.532001354	3.13951769	-10.43621416	1.77E-21	2.59E-20	37.99060478
KCNN4	2.512420846	3.91220208	10.43491653	1.79E-21	2.62E-20	37.98112076
MRPL39	-0.454394577	5.26145611	-10.43411574	1.80E-21	2.63E-20	37.97526831
ECH1	-0.621706367	7.45714518	-10.43365806	1.80E-21	2.64E-20	37.97192349

NUP37	-0.54879011	3.72049664	-10.4283949	1.87E-21	2.74E-20	37.93346338
PSMC3	-0.523661199	7.27278004	-10.42481887	1.92E-21	2.81E-20	37.90733628
C12orf43	-0.412401659	3.06574352	-10.42122907	1.98E-21	2.88E-20	37.88111223
ADI1	-0.625464619	5.43558599	-10.41701132	2.04E-21	2.97E-20	37.85030553
ABCB6	-0.69876567	1.98356568	-10.4146957	2.07E-21	3.02E-20	37.83339417
HSD17B1	-0.530971659	1.57032303	-10.4093977	2.16E-21	3.14E-20	37.79470778
PPP2R3(	-0.566619866	3.45697917	-10.40883266	2.16E-21	3.15E-20	37.79058228
NDUFS7	-0.539698417	4.357993	-10.40718522	2.19E-21	3.18E-20	37.77855443
ACVRL1	-0.933608704	4.29709952	-10.3994973	2.32E-21	3.37E-20	37.72243553
FAM218A	-0.892581215	2.1807783	-10.39577195	2.38E-21	3.46E-20	37.69524789
TOMM40	-0.612887905	3.80457917	-10.39533567	2.39E-21	3.47E-20	37.69206415
PSMA2	-0.504430765	3.17104105	-10.39039449	2.48E-21	3.59E-20	37.65601012
METTL7A	-0.939964965	8.27603864	-10.38920985	2.50E-21	3.62E-20	37.6473672
GALNT1(	-1.128910028	4.26654238	-10.38827211	2.52E-21	3.64E-20	37.64052594
OR2W3	-1.406561819	2.88324232	-10.38179252	2.64E-21	3.82E-20	37.59326099
FDFT1	-0.633481078	5.28423024	-10.37864662	2.70E-21	3.90E-20	37.57031773
WNT4	-1.645824508	1.83786007	-10.37756631	2.73E-21	3.93E-20	37.56243963
WDR93	-0.64541068	1.13755149	-10.37699916	2.74E-21	3.95E-20	37.5583038
COPS8	-0.513017034	4.85412944	-10.37428756	2.79E-21	4.02E-20	37.53853145
TXNRD2	-0.54467536	3.41204771	-10.3730391	2.82E-21	4.06E-20	37.52942866
HIBCH	-0.622393265	4.14700425	-10.36981731	2.89E-21	4.15E-20	37.50593999
SLC39A1	-1.169240084	5.75211334	-10.36757053	2.93E-21	4.21E-20	37.48956144
C12orf60	-0.376262112	0.84982645	-10.36628199	2.96E-21	4.25E-20	37.48016894
CKB	-1.284388454	7.34646431	-10.36396447	3.01E-21	4.32E-20	37.46327702
PPIE	-0.525728271	4.43330256	-10.36092579	3.08E-21	4.41E-20	37.44113111
LRRIQ3	-0.412150845	0.49936197	-10.3596365	3.11E-21	4.45E-20	37.43173558
SLC35G1	-0.77653628	1.88969418	-10.35958265	3.11E-21	4.45E-20	37.43134315
MRPL53	-0.58216279	2.57659833	-10.35685217	3.18E-21	4.54E-20	37.4114467
NSDHL	-0.55542352	5.21480741	-10.35462214	3.23E-21	4.61E-20	37.39519852
ADAMTS	1.199154806	1.2778486	10.34754396	3.40E-21	4.85E-20	37.34363567
SOCS2	-0.902036829	2.83534142	-10.34613964	3.44E-21	4.90E-20	37.33340726
PRKG1	-1.183725172	2.46546599	-10.3452769	3.46E-21	4.93E-20	37.32712373
ADM2	-1.645937314	3.77482413	-10.34493949	3.47E-21	4.93E-20	37.32466633
UMPS	-0.557305198	3.42845063	-10.34433816	3.48E-21	4.95E-20	37.32028694
SPHK2	-0.714348332	3.42952089	-10.34135606	3.56E-21	5.06E-20	37.2985701
SLC27A5	-0.53311769	1.98168974	-10.34057045	3.58E-21	5.08E-20	37.29284942
PYCR3	-0.625538827	4.12738285	-10.33875715	3.63E-21	5.15E-20	37.27964591
IMP3	-0.534630305	5.67858761	-10.33795141	3.65E-21	5.17E-20	37.27377916
TOR2A	-0.607936511	3.29722734	-10.33748028	3.66E-21	5.19E-20	37.2703489
UFD1	-0.532696688	4.13769417	-10.33670718	3.68E-21	5.21E-20	37.2647202

SPG7	-0.646143622	4.66332979	-10.33275196	3.79E-21	5.36E-20	37.23592591
AP5S1	-0.52419331	3.54806796	-10.32746689	3.94E-21	5.57E-20	37.1974573
DDRGK1	-0.67751851	6.96155498	-10.32643477	3.97E-21	5.61E-20	37.1899457
UBAC1	-0.461692209	5.05567969	-10.32579026	3.99E-21	5.63E-20	37.18525526
FBLN7	-0.994384315	2.895563	-10.32304323	4.07E-21	5.74E-20	37.16526482
MIA2	-0.600908642	1.9211585	-10.3219713	4.11E-21	5.78E-20	37.15746485
PDGFD	-1.160293676	4.20242425	-10.32109047	4.13E-21	5.81E-20	37.15105575
COA8	-0.582083222	4.41786111	-10.32007677	4.16E-21	5.85E-20	37.14368008
C16orf86	-0.799797389	2.01757845	-10.31930714	4.19E-21	5.88E-20	37.13808041
HERPUD	-0.860991932	6.58915382	-10.31457184	4.34E-21	6.08E-20	37.10363139
SLC25A1	-1.484267068	3.82341731	-10.31171218	4.43E-21	6.21E-20	37.08283069
SNU13	-0.563616221	7.29018364	-10.31134848	4.44E-21	6.22E-20	37.08018537
CCDC96	-0.608887867	2.41614989	-10.31118524	4.44E-21	6.22E-20	37.07899808
ROPN1L	-0.516145525	0.60880077	-10.30880703	4.52E-21	6.32E-20	37.06170152
NHLRC1	-0.713691704	2.58426528	-10.3082253	4.54E-21	6.35E-20	37.05747089
AAMP	-0.617743067	6.94927573	-10.30547612	4.64E-21	6.47E-20	37.03747878
ANAPC7	-0.400213964	4.51815185	-10.30024126	4.82E-21	6.72E-20	36.99941684
SLC35D2	-0.753980503	5.21307365	-10.29468807	5.02E-21	6.99E-20	36.95904906
VTI1B	-0.534534416	5.46115854	-10.29465848	5.02E-21	6.99E-20	36.95883399
GNAI1	-1.208320685	3.82267217	-10.29059561	5.17E-21	7.19E-20	36.92930544
AP2S1	-0.545611423	6.42080188	-10.28619744	5.34E-21	7.43E-20	36.89734541
GNG7	-0.564793625	3.55413402	-10.28437544	5.41E-21	7.52E-20	36.88410723
NRSN2	-0.575792937	5.83742653	-10.28024046	5.58E-21	7.74E-20	36.85406702
ALKBH4	-0.610985626	4.72142556	-10.27901651	5.63E-21	7.81E-20	36.84517609
FBXO15	-0.471901185	0.6472006	-10.27875136	5.64E-21	7.82E-20	36.84325007
HIST1H3	-0.499261678	0.6359301	-10.27823765	5.66E-21	7.84E-20	36.83951866
ACOT13	-0.443136518	4.26839505	-10.27819207	5.66E-21	7.84E-20	36.83918756
CFAP36	-0.550683004	6.00423601	-10.27494538	5.80E-21	8.02E-20	36.81560611
OGDHL	-1.747857249	3.67858824	-10.26988097	6.02E-21	8.31E-20	36.77882832
BEX2	-0.828657095	7.37013856	-10.26985028	6.02E-21	8.31E-20	36.77860552
PRKCQ	-0.894076587	4.25967762	-10.26736309	6.13E-21	8.46E-20	36.76054628
CFAP20	-0.536610269	5.30023981	-10.26397137	6.29E-21	8.66E-20	36.73592232
HIBADH	-0.871162716	6.52392752	-10.25774431	6.58E-21	9.06E-20	36.69072236
ESRRA	-0.731475942	5.05401167	-10.25711612	6.61E-21	9.10E-20	36.68616326
DHODH	-0.474613013	2.26697074	-10.24490817	7.23E-21	9.94E-20	36.59758556
POMGNT	-0.671000729	4.89961966	-10.23700596	7.66E-21	1.05E-19	36.54027258
SENP8	-0.565233211	2.17860344	-10.2360828	7.72E-21	1.06E-19	36.53357828
TOMM40	-0.471745332	5.20797866	-10.23598133	7.72E-21	1.06E-19	36.5328425
GEMIN7	-0.496362189	3.94685849	-10.23573042	7.74E-21	1.06E-19	36.53102308
TXNDC1!	-0.604527217	5.81750869	-10.23384946	7.84E-21	1.07E-19	36.51738433

SLC25A2	-0.576129966	3.87852401	-10.23099319	8.01E-21	1.10E-19	36.4966756
CHCHD4	-0.539813905	4.58239311	-10.22255654	8.52E-21	1.16E-19	36.43552174
MPPE1	-0.825215182	3.50315108	-10.21635723	8.92E-21	1.22E-19	36.39059888
SLC26A7	-2.261443479	5.82777648	-10.2120543	9.20E-21	1.26E-19	36.35942465
PRKACB	-1.107177448	4.11322728	-10.21027485	9.32E-21	1.27E-19	36.34653439
LYRM9	-0.646805707	3.63661731	-10.2083817	9.45E-21	1.29E-19	36.3328215
LNP1	-0.854287057	3.20563339	-10.20779501	9.49E-21	1.29E-19	36.32857205
UNC119	-0.534061259	3.76474305	-10.20271064	9.85E-21	1.34E-19	36.29174998
MCAT	-0.5785458	4.49236209	-10.20269616	9.85E-21	1.34E-19	36.29164511
SRSF9	-0.729565582	6.02777044	-10.19774376	1.02E-20	1.39E-19	36.25578615
ORC5	-0.503034856	3.45273708	-10.19599787	1.03E-20	1.40E-19	36.24314638
OAZ1	-0.500468965	8.33665531	-10.19471705	1.04E-20	1.42E-19	36.23387413
EIF2B3	-0.476510167	4.28609905	-10.19449831	1.05E-20	1.42E-19	36.23229066
NUDC	-0.529045085	7.14987679	-10.19313328	1.06E-20	1.43E-19	36.22240949
SIRT2	-0.532160841	5.06008956	-10.19211813	1.06E-20	1.44E-19	36.21506133
TG	-1.830703586	12.6676832	-10.18934941	1.09E-20	1.47E-19	36.19502165
DDX41	-0.444840896	5.46573429	-10.18871363	1.09E-20	1.47E-19	36.19042029
NOL3	-0.616656923	5.01000528	-10.18826643	1.10E-20	1.48E-19	36.1871838
FAM204A	-0.469998772	3.05557774	-10.18789626	1.10E-20	1.48E-19	36.18450479
CDHR3	-0.471280652	0.86120174	-10.18775463	1.10E-20	1.48E-19	36.18347982
PPP5D1	-0.307807375	0.78221526	-10.18716599	1.10E-20	1.49E-19	36.17921989
GGTLC1	-1.096214222	1.78059301	-10.18241371	1.14E-20	1.54E-19	36.14483178
PSMD14	-0.530569647	4.31940511	-10.18130296	1.15E-20	1.55E-19	36.13679522
EIF3K	-0.532192925	7.48666811	-10.18113111	1.15E-20	1.55E-19	36.13555195
ACYP1	-0.655280944	3.85879841	-10.18060459	1.16E-20	1.55E-19	36.13174255
RAP1GA	-1.500567668	6.16600594	-10.18010288	1.16E-20	1.56E-19	36.12811282
TP53INP	-0.949629325	5.19572813	-10.17807237	1.18E-20	1.58E-19	36.11342323
LMOD1	-1.336379502	4.05387311	-10.1778062	1.18E-20	1.58E-19	36.11149775
C17orf75	-0.531006643	3.80445683	-10.17677028	1.19E-20	1.59E-19	36.10400405
DNAJC28	-0.59233048	2.55112237	-10.17575184	1.20E-20	1.60E-19	36.09663704
ECSCR	-0.914390079	3.83449006	-10.17534825	1.20E-20	1.61E-19	36.09371774
CYP46A1	-0.450698332	0.63151991	-10.17505024	1.21E-20	1.61E-19	36.09156213
C16orf74	-1.300410621	2.66058458	-10.17294143	1.23E-20	1.63E-19	36.07630935
CLEC3B	-1.326164438	5.97755056	-10.16826176	1.27E-20	1.69E-19	36.04246654
TMEM14	-0.622813646	7.4718799	-10.16735954	1.28E-20	1.70E-19	36.03594259
SKA2	-0.587595254	4.96695667	-10.16677384	1.28E-20	1.70E-19	36.03170749
PRXL2A	-0.592392736	5.47713046	-10.16480909	1.30E-20	1.73E-19	36.01750146
RUNX1	1.280645933	2.97999383	10.16187522	1.33E-20	1.76E-19	35.99629049
IQGAP2	-1.617473225	3.8262374	-10.15884326	1.36E-20	1.80E-19	35.97437303
PCYT2	-0.53153453	4.08957641	-10.15802934	1.37E-20	1.81E-19	35.96848985

TFB2M	-0.736134413	4.59570206	-10.15577561	1.39E-20	1.84E-19	35.95220035
DCTN2	-0.488913098	6.40214839	-10.15321245	1.42E-20	1.87E-19	35.93367635
MOAP1	-0.75760077	5.93875578	-10.15219133	1.43E-20	1.89E-19	35.92629718
KIF6	-0.445206738	0.52730299	-10.14941875	1.46E-20	1.92E-19	35.90626269
BOLA1	-0.643130749	4.98894266	-10.14401038	1.51E-20	2.00E-19	35.86718885
TMEM42	-0.592101679	4.53161332	-10.14300796	1.52E-20	2.01E-19	35.85994769
ETFDH	-0.662610278	3.65004734	-10.14245899	1.53E-20	2.02E-19	35.85598218
AKTIP	-0.579293732	4.34175841	-10.14216323	1.53E-20	2.02E-19	35.85384577
EFCC1	-1.021945961	2.11594372	-10.14173123	1.54E-20	2.03E-19	35.85072534
ZNF691	-0.564677039	4.52959575	-10.14033293	1.55E-20	2.05E-19	35.84062543
MT1X	-1.37931902	7.72572367	-10.13681509	1.60E-20	2.10E-19	35.81521872
FRAT2	-0.57882459	3.82838688	-10.13590579	1.61E-20	2.11E-19	35.8086522
TMEM10	-0.596488213	5.35666269	-10.13588324	1.61E-20	2.11E-19	35.8084893
DNPH1	-0.666348063	5.62325181	-10.13313522	1.64E-20	2.15E-19	35.78864581
TTC1	-0.449355786	6.28416553	-10.13199792	1.65E-20	2.17E-19	35.78043404
TRAPPC	-0.505629289	4.88993492	-10.13128558	1.66E-20	2.18E-19	35.77529088
PDCD2	-0.514230516	4.4151117	-10.13116513	1.66E-20	2.18E-19	35.7744212
FASTKD:	-0.594074839	3.44009356	-10.13092243	1.67E-20	2.18E-19	35.77266891
FABP4	-2.428911576	3.2819485	-10.13034498	1.67E-20	2.19E-19	35.76849985
SEMA3D	-2.209207445	1.96938382	-10.12971802	1.68E-20	2.19E-19	35.76397337
PFKM	-0.853804989	4.61804362	-10.12823672	1.70E-20	2.22E-19	35.75327934
EEF1AKI	-0.538708868	3.3900089	-10.12804276	1.70E-20	2.22E-19	35.75187915
FAM189/	-1.316750436	1.54947128	-10.1272314	1.71E-20	2.23E-19	35.74602198
LEKR1	-0.498157234	0.90643188	-10.12535625	1.73E-20	2.26E-19	35.73248619
B9D2	-0.67148342	3.91005111	-10.12384768	1.75E-20	2.28E-19	35.72159733
ENDOG	-0.687462303	3.83992449	-10.12245537	1.77E-20	2.30E-19	35.71154823
MTG2	-0.523588213	4.0304396	-10.11891657	1.82E-20	2.36E-19	35.68600929
PIGF	-0.596953826	2.96209105	-10.11795801	1.83E-20	2.38E-19	35.67909221
HSDL2	-0.73037558	5.64213512	-10.11034101	1.94E-20	2.51E-19	35.62413672
PSMB7	-0.450640322	7.18219941	-10.10595143	2.00E-20	2.59E-19	35.59247464
GPD1L	-0.922633688	5.51453876	-10.10276911	2.05E-20	2.65E-19	35.5695242
BIN1	-0.627237558	4.37940305	-10.10018277	2.08E-20	2.70E-19	35.55087414
DNAAF4	-0.570946755	2.08593471	-10.09714509	2.13E-20	2.75E-19	35.52897204
ALDH7A:	-0.737285081	4.89772635	-10.09604048	2.15E-20	2.78E-19	35.52100836
LIPT1	-0.612604766	3.49625988	-10.09480297	2.17E-20	2.80E-19	35.51208704
COX7A2I	-0.50362369	5.58001846	-10.09447676	2.17E-20	2.80E-19	35.50973542
NAPA	-0.598238133	5.36611341	-10.09307365	2.20E-20	2.83E-19	35.49962092
FAIM	-0.615836247	3.50854212	-10.09301908	2.20E-20	2.83E-19	35.49922751
QRSL1	-0.525918364	3.72221913	-10.09229608	2.21E-20	2.84E-19	35.49401595
FAM227E	-0.554574874	2.03215895	-10.09121308	2.23E-20	2.86E-19	35.48620963

KY	-0.883323039	1.28551066	-10.09071174	2.23E-20	2.87E-19	35.48259614
B4GALT	-0.54414655	4.53709066	-10.08953246	2.25E-20	2.89E-19	35.47409646
SPATA7	-0.569784557	2.92490345	-10.08846024	2.27E-20	2.91E-19	35.46636875
MAPK8IF	-0.751655214	3.45073369	-10.0852271	2.32E-20	2.98E-19	35.44306907
DUOXA2	-1.90904245	5.18522183	-10.08055025	2.41E-20	3.08E-19	35.409371
UBE2V2	-0.554112764	4.34718198	-10.0739002	2.52E-20	3.23E-19	35.36146692
TMEM10	-0.585347342	5.53608398	-10.07377882	2.53E-20	3.23E-19	35.36059267
FUCA2	-0.511834571	6.20294086	-10.06878804	2.62E-20	3.35E-19	35.32465031
RPF1	-0.54251296	5.75635195	-10.06817628	2.63E-20	3.36E-19	35.32024513
TMEM17	-1.450248539	3.7825873	-10.06639014	2.67E-20	3.40E-19	35.30738401
SLC34A2	3.742880382	8.40538712	10.05979411	2.80E-20	3.57E-19	35.25989767
TARBP2	-0.586405088	3.98908242	-10.05769383	2.84E-20	3.62E-19	35.24478013
ME1	-1.2033652	3.02592743	-10.05395047	2.92E-20	3.72E-19	35.21783916
SLC16A2	-1.490921956	4.26694955	-10.05118011	2.98E-20	3.79E-19	35.19790374
MYCT1	-1.035193705	3.68089027	-10.04962288	3.01E-20	3.83E-19	35.186699
BEAN1	1.008129045	1.70677828	10.04798858	3.05E-20	3.87E-19	35.17494047
UBE2D4	-0.39804573	3.96353429	-10.04567334	3.10E-20	3.94E-19	35.15828419
TMPRSS	2.370766719	2.81449182	10.03647634	3.32E-20	4.21E-19	35.09213537
CORO2B	-1.144981896	2.22610037	-10.03634478	3.32E-20	4.21E-19	35.09118933
TMEM14	-0.617753354	7.18211113	-10.03406382	3.37E-20	4.27E-19	35.07478786
PROX1	-1.042185716	1.76173352	-10.03354199	3.39E-20	4.29E-19	35.07103575
THAP4	-0.502253835	5.39220409	-10.0322514	3.42E-20	4.32E-19	35.06175658
GRB14	-0.830844129	2.03431739	-10.0314766	3.44E-20	4.34E-19	35.05618612
VIPR1	-1.041991943	1.44241465	-10.02997807	3.48E-20	4.39E-19	35.04541279
CKMT2	-1.470830483	2.81130792	-10.02834309	3.52E-20	4.44E-19	35.03365935
ZFAND2	-0.711177346	4.55435738	-10.02798493	3.53E-20	4.45E-19	35.03108479
DTWD1	-0.492865154	2.24340114	-10.02768839	3.53E-20	4.45E-19	35.02895311
TMEM59	-0.658249767	6.98490695	-10.02719858	3.55E-20	4.47E-19	35.02543226
TBCA	-0.572403033	6.45844637	-10.02607766	3.58E-20	4.50E-19	35.01737515
HADH	-0.642670328	5.71538674	-10.02531653	3.60E-20	4.52E-19	35.01190442
FBXO10	-0.660855949	1.88002856	-10.02502183	3.60E-20	4.53E-19	35.00978628
AIMP2	-0.743358892	4.84701024	-10.02493139	3.61E-20	4.53E-19	35.00913625
TSACC	-0.448106251	0.79413327	-10.02444507	3.62E-20	4.54E-19	35.00564086
MAGED2	-0.649329716	8.21295173	-10.02297435	3.66E-20	4.58E-19	34.99507074
CCDC28	-0.644343579	6.56086836	-10.02079009	3.72E-20	4.65E-19	34.97937362
TRIM41	-0.565095603	3.62348625	-10.02015319	3.73E-20	4.67E-19	34.97479679
IGFBPL1	-1.67732349	2.08828408	-10.01580629	3.85E-20	4.82E-19	34.94356323
C9orf78	-0.475846266	5.6324061	-10.01463756	3.89E-20	4.86E-19	34.93516658
PLPP1	-1.079203878	6.44048752	-10.01092405	3.99E-20	4.99E-19	34.90849002
GPAT3	-1.105200949	4.47751681	-10.00468878	4.18E-20	5.21E-19	34.86370775

SERF2	-0.588458581	7.45168129	-10.00326407	4.22E-20	5.26E-19	34.85347704
MAPKAP	-0.568459933	5.28912557	-9.995938941	4.45E-20	5.55E-19	34.80088606
CFAP20(	-0.191250347	0.23022781	-9.995829146	4.45E-20	5.55E-19	34.80009791
CCM2L	-0.872893669	2.97230531	-9.994746449	4.49E-20	5.59E-19	34.79232611
TRMT12	-0.531632086	4.10468628	-9.990125369	4.64E-20	5.77E-19	34.75915928
LARP7	-0.566650327	5.2248816	-9.989333843	4.67E-20	5.80E-19	34.75347894
KIAA161	-0.716128981	1.98919802	-9.98724533	4.74E-20	5.89E-19	34.73849179
UFSP1	-0.666490256	2.42846454	-9.986645236	4.76E-20	5.91E-19	34.73418578
SSBP1	-0.448541781	5.13450096	-9.986572001	4.76E-20	5.91E-19	34.73366028
STOML2	-0.508233479	7.00302816	-9.986183417	4.78E-20	5.92E-19	34.73087204
CCDC18	-0.568991373	1.62462553	-9.985613207	4.80E-20	5.94E-19	34.72678064
PDE6B	-0.624105656	3.27413679	-9.984860834	4.82E-20	5.97E-19	34.72138234
NFIA	-0.790992677	4.75908391	-9.981771742	4.93E-20	6.10E-19	34.69921987
LRRC6	-0.692853157	2.82362741	-9.980989081	4.96E-20	6.13E-19	34.6936052
KIT	-1.930618111	3.3018151	-9.980191432	4.99E-20	6.16E-19	34.6878832
AAK1	-0.85092033	3.45706985	-9.979919558	5.00E-20	6.17E-19	34.68593294
MRPL43	-0.511297464	5.99507887	-9.9792741	5.02E-20	6.19E-19	34.6813029
SLC9A3F	-0.865061138	6.48540419	-9.979022134	5.03E-20	6.20E-19	34.67949552
ABCF3	-0.487874614	5.12197141	-9.977576019	5.09E-20	6.26E-19	34.66912274
PRSS16	-0.804329091	3.76107858	-9.973806411	5.23E-20	6.43E-19	34.64208698
KLHDC3	-0.573577946	7.15639157	-9.972044681	5.29E-20	6.51E-19	34.62945333
ANGPTL	-1.437555173	3.69269763	-9.971648806	5.31E-20	6.52E-19	34.62661458
GAPDH	-0.519987566	10.6421027	-9.967909457	5.45E-20	6.69E-19	34.59980279
FAM162	-0.494384185	5.64862006	-9.963864731	5.62E-20	6.89E-19	34.57080635
ZNF428	-0.626169647	6.24023027	-9.962179349	5.69E-20	6.97E-19	34.55872546
ZNF396	-0.572309583	2.01280411	-9.961825675	5.70E-20	6.98E-19	34.55619042
PSMC2	-0.494936323	6.1061824	-9.961574295	5.71E-20	6.99E-19	34.55438862
PPP1R3	-0.746960326	4.5694624	-9.960466488	5.76E-20	7.04E-19	34.54644851
UBE2E3	-0.5978721	5.90537339	-9.960093793	5.77E-20	7.05E-19	34.54377733
NGDN	-0.493747255	4.15273083	-9.958994268	5.82E-20	7.11E-19	34.5358971
MGAT1	-0.592741017	6.4691547	-9.958240862	5.85E-20	7.14E-19	34.53049769
TMEM15	-0.907853302	3.79693392	-9.957803461	5.87E-20	7.16E-19	34.52736308
ZNRD2	-0.537011835	4.99064587	-9.95407487	6.03E-20	7.35E-19	34.5006447
CCDC65	-0.74550318	2.19422945	-9.95242217	6.10E-20	7.43E-19	34.48880317
LENG1	-0.553765455	4.72849158	-9.951290906	6.15E-20	7.49E-19	34.48069821
SNPH	-0.834706002	3.04120681	-9.947898939	6.31E-20	7.67E-19	34.45639882
TRMT10	-0.510870514	4.77001024	-9.947118499	6.34E-20	7.71E-19	34.45080841
TATDN3	-0.446326741	3.61479975	-9.943546622	6.51E-20	7.90E-19	34.42522501
CPT1A	-0.838843008	4.40885462	-9.94213125	6.58E-20	7.98E-19	34.41508859
TMEM25	-0.556215332	6.05278838	-9.936244981	6.86E-20	8.32E-19	34.37293992

ABHD10	-0.552704004	5.54786451	-9.935794899	6.88E-20	8.34E-19	34.36971756
GLA	-0.515349664	4.2820165	-9.934529752	6.95E-20	8.41E-19	34.36066008
MRPL28	-0.588366005	6.14689617	-9.933830784	6.98E-20	8.45E-19	34.35565623
MRFAP1	-0.564581644	8.50139437	-9.933438915	7.00E-20	8.47E-19	34.35285094
FRMD5	1.468682098	1.89399482	9.929818067	7.19E-20	8.68E-19	34.32693252
PFN2	-0.763258941	6.92526635	-9.92979726	7.19E-20	8.68E-19	34.32678359
RPS6KL1	-0.668889439	1.05930704	-9.928564033	7.25E-20	8.75E-19	34.31795698
TYSND1	-0.541284707	4.17807078	-9.926894115	7.34E-20	8.85E-19	34.3060056
POLR3F	-0.487210574	3.72789718	-9.92420921	7.49E-20	9.02E-19	34.28679195
ACOT2	-0.55598408	4.26358462	-9.922254313	7.59E-20	9.14E-19	34.27280381
VPS4A	-0.479464592	5.37114209	-9.920442395	7.69E-20	9.25E-19	34.25983984
MRPL49	-0.684405938	6.15877908	-9.917821758	7.84E-20	9.43E-19	34.24109145
ABHD14I	-0.504926702	6.98197713	-9.916970463	7.89E-20	9.48E-19	34.23500166
TCTN1	-0.672321366	4.0806215	-9.916614552	7.91E-20	9.49E-19	34.23245569
CETN3	-0.539634224	3.83026142	-9.913771519	8.07E-20	9.69E-19	34.21211983
SYNE1	-1.093838995	2.63317045	-9.91168818	8.20E-20	9.83E-19	34.19721961
CD36	-1.652003249	2.92363629	-9.909680905	8.32E-20	9.96E-19	34.18286471
EFCAB2	-0.648388154	2.60287167	-9.908682276	8.38E-20	1.00E-18	34.17572356
DLD	-0.801105306	5.19542937	-9.905022233	8.60E-20	1.03E-18	34.14955349
DIO2	-1.641227335	6.15616326	-9.903526832	8.70E-20	1.04E-18	34.13886229
PTDSS2	-0.602330262	4.20683748	-9.90075984	8.87E-20	1.06E-18	34.11908188
R3HCC1	-0.514098283	5.29416052	-9.900595434	8.88E-20	1.06E-18	34.11790667
TPRKB	-0.455957404	4.32494302	-9.89923048	8.97E-20	1.07E-18	34.10814999
RSAD1	-0.486448698	5.07164694	-9.896323265	9.16E-20	1.09E-18	34.08737124
AUP1	-0.527968377	6.94162363	-9.894458363	9.28E-20	1.11E-18	34.07404364
PRKRIP1	-0.582391002	4.8635694	-9.893924849	9.32E-20	1.11E-18	34.07023106
PSMA3	-0.466660028	6.06697076	-9.892945579	9.39E-20	1.12E-18	34.06323329
RIPOR3	-0.828562537	1.09742432	-9.89283113	9.39E-20	1.12E-18	34.06241546
UFSP2	-0.630481591	5.20136046	-9.892557526	9.41E-20	1.12E-18	34.06046038
SEC62	-0.641439997	6.52400346	-9.892116087	9.44E-20	1.12E-18	34.05730605
CXorf58	-0.228423475	0.3777094	-9.890251126	9.57E-20	1.14E-18	34.04398055
SFTPB	3.823812612	6.45643155	9.88955196	9.62E-20	1.14E-18	34.03898515
KIZ	-0.685494066	3.66446905	-9.888575863	9.69E-20	1.15E-18	34.03201142
MNAT1	-0.479375137	3.53332789	-9.880784947	1.02E-19	1.21E-18	33.97636011
RNF14	-0.555753615	5.18292701	-9.876067365	1.06E-19	1.25E-18	33.94267142
ACAA1	-0.578017143	4.96978182	-9.87414711	1.08E-19	1.27E-18	33.92896076
RPS6KA(	-0.882159516	2.40156026	-9.873502072	1.08E-19	1.28E-18	33.92435544
TMEM9B	-0.618767621	6.61926568	-9.866368622	1.14E-19	1.34E-18	33.87343434
GTF3C1	-0.874018839	5.45994939	-9.86394951	1.16E-19	1.37E-18	33.85616959
ALKBH3	-0.467450327	4.42718354	-9.861321825	1.18E-19	1.39E-18	33.83741843

EPHX2	-0.666230973	4.89210136	-9.860805487	1.18E-19	1.39E-18	33.8337341
SNRNP2	-0.544022478	4.98811058	-9.856484305	1.22E-19	1.44E-18	33.80290373
ETV2	-0.522073357	1.11581588	-9.852967758	1.25E-19	1.47E-18	33.77781864
HIGD2A	-0.543461944	7.83071631	-9.851802693	1.26E-19	1.49E-18	33.7695086
STX8	-0.526436888	4.85807979	-9.847007031	1.31E-19	1.54E-18	33.7353073
HIST1H1	-1.551817479	7.80970617	-9.845323478	1.32E-19	1.55E-18	33.72330244
TRMT10/	-0.58321145	2.38511211	-9.844468718	1.33E-19	1.56E-18	33.71720778
BTNL8	-0.268583216	0.24087351	-9.842956274	1.35E-19	1.58E-18	33.70642425
MAP2K6	-0.587809145	1.069481	-9.841916746	1.36E-19	1.59E-18	33.69901298
SYS1-DE	-0.414927045	0.82791922	-9.84105093	1.37E-19	1.60E-18	33.69284045
MED20	-0.583234043	4.10632147	-9.836670907	1.41E-19	1.65E-18	33.66161835
POLE4	-0.704953209	4.94387217	-9.83486232	1.43E-19	1.67E-18	33.64872802
TRIT1	-0.655632649	4.54313023	-9.83339955	1.44E-19	1.69E-18	33.63830321
GKAP1	-0.571283347	3.44815979	-9.833286696	1.44E-19	1.69E-18	33.63749896
ESRRG	-0.821366109	1.32878769	-9.832936479	1.45E-19	1.69E-18	33.63500317
ATP23	-0.565176679	3.7970931	-9.830735614	1.47E-19	1.71E-18	33.61931978
PSMF1	-0.434158646	6.24401992	-9.830512811	1.47E-19	1.72E-18	33.61773217
ZNF219	-0.828717867	4.73396724	-9.830097004	1.48E-19	1.72E-18	33.61476933
AGBL4	-0.36381492	0.5795364	-9.828875801	1.49E-19	1.73E-18	33.60606796
ZNF576	-0.480702747	3.68657717	-9.828172095	1.50E-19	1.74E-18	33.6010541
EDF1	-0.653079237	9.54410921	-9.823423832	1.55E-19	1.80E-18	33.56722726
TMEM20	-1.086715427	5.46194788	-9.821303691	1.57E-19	1.83E-18	33.55212566
SPACA9	-0.658920319	4.2324962	-9.820638617	1.58E-19	1.84E-18	33.54738869
SYBU	-1.030203703	2.62791296	-9.815274916	1.64E-19	1.91E-18	33.50919116
ASXL3	-0.642496316	0.48515756	-9.815167536	1.65E-19	1.91E-18	33.50842655
PIGH	-0.673462553	4.50011411	-9.81453688	1.65E-19	1.91E-18	33.50393598
CLEC14/	-0.967991883	5.70990456	-9.81412191	1.66E-19	1.92E-18	33.50098127
DCTN6	-0.543983324	5.66654268	-9.808853862	1.72E-19	1.99E-18	33.46347607
NEK11	-0.662690269	3.26242159	-9.80834445	1.73E-19	2.00E-18	33.45984986
SCGB2A	-1.528915238	2.81210616	-9.802295232	1.81E-19	2.08E-18	33.41679543
MICOS1:	-0.576944229	6.26338438	-9.80165885	1.81E-19	2.09E-18	33.41226677
NANS	-0.530709505	4.44444603	-9.800698957	1.83E-19	2.11E-18	33.40543618
NDUFV2	-0.544502117	3.4827368	-9.798561417	1.85E-19	2.14E-18	33.39022654
CDPF1	-0.570596402	3.92520026	-9.798418904	1.86E-19	2.14E-18	33.38921254
CDIPT	-0.473755698	6.60733978	-9.797320605	1.87E-19	2.15E-18	33.38139824
NUDT15	-0.564077407	4.81683302	-9.794169996	1.91E-19	2.20E-18	33.35898412
FAM234E	-1.009617709	2.69224952	-9.794165946	1.91E-19	2.20E-18	33.35895532
AKT1S1	-0.480467679	4.84451226	-9.793806507	1.92E-19	2.20E-18	33.35639839
CRY2	-0.8082925	5.07287862	-9.793708472	1.92E-19	2.20E-18	33.35570102
SAYSD1	-0.437649526	4.44157029	-9.793520433	1.92E-19	2.20E-18	33.3543634

RNF150	-1.067034117	1.7833513	-9.793484927	1.92E-19	2.20E-18	33.35411083
PAIP2B	-0.980113817	2.67929029	-9.791573924	1.95E-19	2.23E-18	33.34051756
ANO10	-0.493373017	4.7040443	-9.791457155	1.95E-19	2.23E-18	33.33968701
NUBPL	-0.53472849	2.54682572	-9.789173669	1.98E-19	2.27E-18	33.32344586
BCS1L	-0.521330173	4.00333628	-9.783853993	2.06E-19	2.36E-18	33.28561669
HMGCR	-0.861499099	4.43104856	-9.781974282	2.09E-19	2.39E-18	33.27225195
RASIP1	-0.913698658	3.60848405	-9.78105652	2.10E-19	2.40E-18	33.26572709
TSGA10	-0.571939736	2.32325375	-9.780680265	2.11E-19	2.41E-18	33.26305217
ALOX5	2.374334256	4.44263518	9.776978876	2.17E-19	2.47E-18	33.23674029
SPARCL	-1.270864024	7.47826891	-9.772333058	2.24E-19	2.55E-18	33.20372118
HK1	-0.755423896	6.22555302	-9.765844421	2.35E-19	2.67E-18	33.15761658
ENO4	-0.372195829	0.61788534	-9.764767459	2.37E-19	2.69E-18	33.14996565
DIS3L	-0.648283734	4.19024843	-9.75962469	2.45E-19	2.79E-18	33.11343574
NPR1	-0.897064896	2.88946735	-9.756599797	2.51E-19	2.85E-18	33.09195354
C19orf73	-0.675374982	2.18798294	-9.755998247	2.52E-19	2.86E-18	33.08768182
AKAP3	-0.705817482	1.26983441	-9.755598629	2.53E-19	2.87E-18	33.08484411
TFB1M	-0.502976147	3.79810666	-9.754164156	2.55E-19	2.89E-18	33.0746583
PRKAG1	-0.527009432	5.2413193	-9.746673744	2.69E-19	3.05E-18	33.02148195
CDC42EI	1.108141854	3.92178351	9.746524436	2.70E-19	3.05E-18	33.02042216
TEFM	-0.450139735	2.54752091	-9.746519653	2.70E-19	3.05E-18	33.02038821
HADHB	-0.686993342	6.64934536	-9.746136829	2.70E-19	3.06E-18	33.01767096
ZDHHC4	-0.610294311	5.34167964	-9.743265938	2.76E-19	3.12E-18	32.99729522
CCT6B	-0.530680203	1.68849425	-9.741022555	2.81E-19	3.17E-18	32.98137504
AHSA1	-0.445063221	6.13810177	-9.739287158	2.84E-19	3.21E-18	32.96906093
NDUFB8	-0.562750078	6.00571486	-9.737820048	2.87E-19	3.24E-18	32.95865132
KDR	-1.311612604	5.22009764	-9.736781951	2.89E-19	3.26E-18	32.95128613
AFG3L2	-0.737618574	4.74272619	-9.733758955	2.96E-19	3.33E-18	32.92984033
SLC35A4	-0.497387701	6.02836564	-9.730939628	3.02E-19	3.39E-18	32.90984214
KCTD15	-0.935603329	3.56712781	-9.730285838	3.03E-19	3.41E-18	32.90520502
GSS	-0.44475871	6.55187783	-9.72965713	3.04E-19	3.42E-18	32.90074594
SAR1B	-0.545677994	4.21361352	-9.729316722	3.05E-19	3.43E-18	32.89833167
DUS1L	-0.530895903	5.33975405	-9.727368672	3.09E-19	3.47E-18	32.88451627
SLC25A3	-0.513851499	6.9082409	-9.726257712	3.12E-19	3.50E-18	32.87663801
LDB2	-0.967717261	3.97298105	-9.725625554	3.13E-19	3.51E-18	32.8721553
C19orf25	-0.579492307	4.40225811	-9.725399874	3.14E-19	3.52E-18	32.87055501
HIST1H4	-0.910844627	4.28331148	-9.724396183	3.16E-19	3.54E-18	32.86343808
ACOX2	-0.749952212	4.78068086	-9.723194415	3.19E-19	3.57E-18	32.85491707
FKBPL	-0.632052687	4.60790383	-9.720634485	3.25E-19	3.63E-18	32.83676777
FLT1	-1.229102371	4.88866625	-9.717117144	3.33E-19	3.72E-18	32.8118342
HMGN3	-0.570181219	8.0510463	-9.71669396	3.34E-19	3.73E-18	32.80883464

DNAL1	-0.66390775	3.13589137	-9.715428363	3.37E-19	3.76E-18	32.79986433
ITFG2	-0.594353659	4.50808092	-9.71264319	3.44E-19	3.83E-18	32.78012544
PRMT3	-0.768105826	3.31953918	-9.712051268	3.45E-19	3.85E-18	32.77593075
PTCD2	-0.496200743	1.97383161	-9.711207254	3.47E-19	3.87E-18	32.76994978
CCDC12	-0.669325102	2.70700308	-9.708372119	3.55E-19	3.95E-18	32.74986083
TMEM25	-0.472479603	0.58490137	-9.708176149	3.55E-19	3.95E-18	32.74847235
ELOVL1	-0.753071246	6.78682438	-9.707854774	3.56E-19	3.96E-18	32.74619537
ITPA	-0.569171961	6.35833548	-9.707222666	3.58E-19	3.97E-18	32.74171691
SIL1	-0.599750762	6.33651784	-9.705626263	3.62E-19	4.01E-18	32.73040706
SHBG	-0.438277695	0.94412434	-9.700390044	3.75E-19	4.16E-18	32.69331663
TECR	-0.511524839	5.98207529	-9.700245945	3.76E-19	4.17E-18	32.69229604
CXCL17	2.369193743	3.53735119	9.698155864	3.82E-19	4.23E-18	32.67749375
PRCP	-0.707051819	4.42913802	-9.696710427	3.85E-19	4.27E-18	32.6672578
NUDT16I	-0.771647877	6.18388078	-9.695524312	3.89E-19	4.30E-18	32.65885877
RBM45	-0.433914857	2.80529712	-9.693515769	3.94E-19	4.36E-18	32.6446371
DDX1	-0.601694311	6.51287852	-9.689170474	4.07E-19	4.50E-18	32.61387451
FASTKD:	-0.549758894	2.9285276	-9.688112705	4.10E-19	4.53E-18	32.60638697
LMO2	-0.842094053	4.19567012	-9.685199402	4.19E-19	4.62E-18	32.58576678
FASTK	-0.518006144	5.69588862	-9.684069472	4.22E-19	4.65E-18	32.57776998
DHX29	-0.713261609	4.78033131	-9.679474377	4.36E-19	4.81E-18	32.54525373
TMSB15/	-1.097961614	1.83999866	-9.678831072	4.38E-19	4.83E-18	32.54070208
BUD23	-0.496275222	5.1421196	-9.677737965	4.42E-19	4.86E-18	32.53296821
H2AFV	-0.547176701	6.32202669	-9.67731132	4.43E-19	4.87E-18	32.52994976
TCEAL1	-0.6337701	5.46732509	-9.67466087	4.51E-19	4.96E-18	32.51119957
FZD4	-0.869563201	4.20644225	-9.67367057	4.55E-19	4.99E-18	32.50419445
RPS27A	-0.639766834	8.73693131	-9.671216992	4.63E-19	5.08E-18	32.48683994
IPO13	-0.591849699	5.4565059	-9.669500361	4.68E-19	5.14E-18	32.47469917
ERGIC3	-0.541116108	7.08156632	-9.668907701	4.70E-19	5.16E-18	32.47050784
TBC1D2	-0.589705656	3.03307772	-9.667930554	4.74E-19	5.19E-18	32.46359767
NDUFA3	-0.669811148	6.21514305	-9.66692295	4.77E-19	5.22E-18	32.45647244
TMEM17	-0.556443912	4.61489269	-9.666177856	4.80E-19	5.25E-18	32.45120377
ANKEF1	-0.814991538	2.91440399	-9.663923275	4.88E-19	5.33E-18	32.43526242
NEK5	-0.295472822	0.59089364	-9.663297487	4.90E-19	5.35E-18	32.430838
EID1	-0.629149458	8.3261074	-9.663036751	4.91E-19	5.36E-18	32.4289946
ZFAND6	-0.594408431	5.92149722	-9.662872401	4.91E-19	5.36E-18	32.42783265
TM4SF1	-1.054776393	3.40729496	-9.662288596	4.93E-19	5.38E-18	32.42370526
SDF4	-0.555705883	6.70856676	-9.66048282	5.00E-19	5.45E-18	32.4109395
CRABP1	-2.824350565	4.90812375	-9.660236119	5.01E-19	5.45E-18	32.40919555
PHB	-0.540335679	6.22622336	-9.65879555	5.06E-19	5.51E-18	32.3990125
CATIP	-0.682154109	1.91650809	-9.657351972	5.11E-19	5.56E-18	32.38880888

SKP1	-0.59357929	6.17153371	-9.653841898	5.24E-19	5.70E-18	32.36400163
RIMKLA	-0.60501724	0.69707311	-9.652412072	5.29E-19	5.75E-18	32.35389761
CBR4	-0.67887479	4.33292952	-9.651454794	5.33E-19	5.79E-18	32.3471333
HADHA	-0.589936223	7.29734432	-9.65016489	5.38E-19	5.84E-18	32.33801907
EXOSC7	-0.522090942	4.70237534	-9.649626365	5.40E-19	5.86E-18	32.33421412
C2orf15	-0.61544079	3.20381516	-9.640315776	5.77E-19	6.25E-18	32.26844565
NAIF1	-0.440422014	3.19520742	-9.640305434	5.77E-19	6.25E-18	32.26837262
RAD51D	-0.456858003	2.28979348	-9.6389624	5.83E-19	6.31E-18	32.25888809
TALDO1	-0.66507538	7.57655813	-9.638123649	5.86E-19	6.34E-18	32.25296512
GAR1	-0.500644627	4.54184288	-9.638007018	5.87E-19	6.34E-18	32.25214154
RARB	-0.917124132	2.95686745	-9.637860378	5.87E-19	6.35E-18	32.25110605
SLC29A4	-1.443265706	2.2029662	-9.634464846	6.02E-19	6.50E-18	32.22713075
BANP	-0.479004199	2.35245278	-9.634350271	6.02E-19	6.50E-18	32.22632182
ITM2A	-1.157060639	5.95460856	-9.633947015	6.04E-19	6.51E-18	32.22347478
UBE2O	-0.683776331	4.45044141	-9.630270603	6.20E-19	6.68E-18	32.19752131
EIF4E2	-0.409555647	4.494084	-9.629833848	6.22E-19	6.70E-18	32.19443837
TEX261	-0.657468758	6.27939005	-9.626067398	6.39E-19	6.88E-18	32.16785463
TSPAN15	-0.73182587	4.34705726	-9.625695205	6.41E-19	6.89E-18	32.16522794
MAGT1	-0.890993198	6.48152805	-9.623695347	6.50E-19	6.99E-18	32.1511151
FAM241B	-0.586782143	4.41475673	-9.619234212	6.71E-19	7.21E-18	32.11963813
COMMD9	-0.323405271	4.03600012	-9.618393071	6.75E-19	7.25E-18	32.11370396
TSPAN33	-1.377860661	4.20875899	-9.615296469	6.90E-19	7.40E-18	32.09185978
TCFL5	-0.865222189	4.12230395	-9.614549641	6.94E-19	7.44E-18	32.08659197
APIP	-0.581258131	3.77322116	-9.612700421	7.03E-19	7.53E-18	32.07354917
VEGFA	-1.275965206	6.0652999	-9.612689026	7.03E-19	7.53E-18	32.0734688
DCAF4	-0.575386952	3.52531083	-9.612587938	7.04E-19	7.53E-18	32.07275585
ATF7IP2	-0.824503346	2.31056689	-9.610175919	7.16E-19	7.66E-18	32.0557454
COL23A1	-1.789112294	4.92221047	-9.610027147	7.17E-19	7.66E-18	32.05469627
CANX	-0.833041551	8.67494285	-9.609469704	7.19E-19	7.68E-18	32.05076529
SCPEP1	-0.898216622	7.17199733	-9.606437995	7.35E-19	7.85E-18	32.02938813
NETO2	-0.87600598	1.80640379	-9.603619218	7.50E-19	8.00E-18	32.00951521
NOSTRIN	-0.847013015	2.59435987	-9.602544392	7.56E-19	8.06E-18	32.0019382
BRMS1	-0.498374037	5.83314095	-9.601917051	7.59E-19	8.09E-18	31.99751594
MTFP1	-0.604394512	2.68488616	-9.600494186	7.67E-19	8.17E-18	31.98748634
GMPR2	-0.543339095	6.34247017	-9.599358085	7.73E-19	8.23E-18	31.9794786
SNX4	-0.788253608	5.6897037	-9.595532986	7.95E-19	8.45E-18	31.95252089
COG2	-0.530969974	3.23439512	-9.593915317	8.04E-19	8.54E-18	31.94112175
OTUB1	-0.483894758	5.92894559	-9.593728446	8.05E-19	8.55E-18	31.93980499
ZNF467	-0.823619833	4.03703511	-9.591086902	8.20E-19	8.71E-18	31.92119306
JMJD4	-0.47258999	3.51724888	-9.587686682	8.40E-19	8.91E-18	31.89723915

EML1	-0.885284355	2.83099466	-9.585370182	8.54E-19	9.06E-18	31.88092213
TBX22	-0.790023194	0.57828658	-9.583438567	8.66E-19	9.18E-18	31.86731758
SPATA2	-0.628838631	4.04621226	-9.58144952	8.79E-19	9.30E-18	31.85330988
C6orf226	-0.636758255	5.04689874	-9.581015673	8.81E-19	9.33E-18	31.85025473
SPINT2	-0.796682272	8.68069479	-9.580811078	8.83E-19	9.33E-18	31.84881399
C5orf63	-0.524319907	1.39170968	-9.57848547	8.97E-19	9.48E-18	31.83243833
GHITM	-0.681846853	7.67372898	-9.576831029	9.08E-19	9.59E-18	31.8207898
PAX6	-0.178199544	0.39818855	-9.574389818	9.24E-19	9.75E-18	31.80360353
MT1M	-1.787533841	3.34672285	-9.573742091	9.28E-19	9.79E-18	31.79904383
RPUSD2	-0.458154092	3.70350134	-9.568131956	9.66E-19	1.02E-17	31.75955722
DGAT1	-0.553549235	4.87845232	-9.567988435	9.67E-19	1.02E-17	31.7585472
DPAGT1	-0.536298084	4.34179051	-9.566585145	9.77E-19	1.03E-17	31.74867199
SNRPA1	-0.515297265	4.05964808	-9.565253536	9.86E-19	1.04E-17	31.73930185
CDIP1	-0.766363847	5.25845686	-9.561453909	1.01E-18	1.07E-17	31.71256837
CWC15	-0.412679458	5.99273578	-9.560153119	1.02E-18	1.07E-17	31.7034174
RRP8	-0.459992039	3.47315904	-9.558253361	1.04E-18	1.09E-17	31.69005378
JAK3	1.322482235	2.69735263	9.558143881	1.04E-18	1.09E-17	31.68928369
KCNJ13	-1.278151305	1.12973294	-9.551317066	1.09E-18	1.14E-17	31.64127188
SPATA2(	-0.701255964	5.98119525	-9.550040016	1.10E-18	1.15E-17	31.63229241
SLC46A3	-0.866152528	4.11854755	-9.548371905	1.11E-18	1.17E-17	31.62056407
AGTR1	-1.192159227	1.20217027	-9.543627708	1.15E-18	1.20E-17	31.58721335
FBXO36	-0.699231957	2.9427851	-9.540415445	1.18E-18	1.23E-17	31.56463625
MUC1	2.171081042	5.02595284	9.538143767	1.20E-18	1.25E-17	31.54867214
SCARB1	-0.806939079	3.84541179	-9.536995378	1.21E-18	1.26E-17	31.54060257
TMEM12	-0.468465496	5.76146703	-9.536977224	1.21E-18	1.26E-17	31.54047501
MYOM2	-0.627884113	1.21159966	-9.531941266	1.25E-18	1.30E-17	31.50509351
NUCB1	-0.514380242	8.1244849	-9.531930116	1.25E-18	1.30E-17	31.50501519
FLT4	-1.073134418	3.47098276	-9.529071503	1.28E-18	1.33E-17	31.48493517
FBXL5	-0.771771573	6.36708699	-9.528136018	1.28E-18	1.34E-17	31.47836457
CNDP2	-0.685421888	6.16072484	-9.523768928	1.32E-18	1.38E-17	31.44769536
BCO2	-0.716515998	1.05751408	-9.523687677	1.33E-18	1.38E-17	31.44712481
PKIA	-1.165492916	4.47770055	-9.522525665	1.34E-18	1.39E-17	31.43896538
GCAT	-0.740550283	4.96013816	-9.520863112	1.35E-18	1.41E-17	31.42729208
ATP2C2	-1.04145477	1.38742577	-9.520191225	1.36E-18	1.41E-17	31.42257482
RAB1B	-0.527025758	8.13998037	-9.519742646	1.36E-18	1.42E-17	31.41942548
SAE1	-0.706344026	6.63861583	-9.518338555	1.38E-18	1.43E-17	31.40956822
TBC1D3(	-0.563243215	1.25840269	-9.515490491	1.41E-18	1.46E-17	31.38957583
CCDC17:	-0.669366173	1.2609168	-9.5139054	1.42E-18	1.47E-17	31.37845029
ERCC2	-0.54865359	3.73395916	-9.513368256	1.43E-18	1.48E-17	31.37468035
DUT	-0.595449356	5.26722723	-9.513126701	1.43E-18	1.48E-17	31.37298502

SUCLA2	-0.758426192	5.12681352	-9.51026775	1.46E-18	1.51E-17	31.35292144
PET100	-0.761594011	4.8947977	-9.508513652	1.48E-18	1.53E-17	31.34061292
PSMA7	-0.536829394	7.56319355	-9.508225725	1.48E-18	1.53E-17	31.33859264
ACADM	-0.84181326	4.69794118	-9.507705048	1.49E-18	1.53E-17	31.33493931
TMEM16	-0.619274621	5.50949192	-9.506986113	1.49E-18	1.54E-17	31.32989506
DNAJC4	-0.504459453	5.89993303	-9.501294533	1.55E-18	1.60E-17	31.2899677
NIT1	-0.511624962	4.93007075	-9.497486038	1.60E-18	1.65E-17	31.26325685
SLC33A1	-0.720139377	3.98666089	-9.495233784	1.62E-18	1.67E-17	31.24746308
PHPT1	-0.593414438	7.60465671	-9.494280681	1.63E-18	1.68E-17	31.24078005
MLLT3	-0.740828197	3.47993286	-9.491482944	1.67E-18	1.71E-17	31.22116455
USHBP1	-0.741818953	1.98221899	-9.488851531	1.70E-18	1.75E-17	31.2027177
TERF2IP	-0.503685228	6.70375954	-9.487293196	1.72E-18	1.76E-17	31.19179454
KCNE2	-0.5298055	1.24484074	-9.484202253	1.75E-18	1.80E-17	31.17013108
MACC1	1.370205815	2.37014437	9.480592934	1.80E-18	1.85E-17	31.14483875
CFAP57	-0.557400822	0.89761038	-9.479774036	1.81E-18	1.86E-17	31.13910096
KLLN	-0.479621668	1.04712646	-9.476118881	1.86E-18	1.90E-17	31.11349318
KCTD2	-0.691416638	5.13841133	-9.475055034	1.87E-18	1.92E-17	31.10604082
FAM114A	-0.529827415	3.24094148	-9.474588559	1.88E-18	1.92E-17	31.10277325
TTC25	-0.634497773	2.64526418	-9.470042714	1.94E-18	1.98E-17	31.0709344
RXYLT1	-0.532394556	3.08899001	-9.469480892	1.95E-18	1.99E-17	31.06699994
PLCD4	-0.82709019	1.72026503	-9.466843504	1.98E-18	2.03E-17	31.04853169
STRA6	1.797741933	2.49642202	9.465852996	2.00E-18	2.04E-17	31.04159632
CLNS1A	-0.538194893	5.6121614	-9.463972849	2.03E-18	2.07E-17	31.02843281
TUSC1	-0.709817229	4.47479012	-9.461776002	2.06E-18	2.10E-17	31.01305355
GOLGA8	-0.835111797	1.56386296	-9.461749753	2.06E-18	2.10E-17	31.01286981
CFDP1	-0.535317769	5.94026798	-9.461147021	2.07E-18	2.10E-17	31.00865062
MKS1	-0.540958236	3.47260216	-9.460530661	2.08E-18	2.11E-17	31.00433618
GGPS1	-0.451020863	4.99463031	-9.457379832	2.12E-18	2.16E-17	30.98228286
SEC11A	-0.549111783	6.38169834	-9.456761974	2.13E-18	2.17E-17	30.97795875
BSPRY	-0.687657392	6.48126911	-9.456385616	2.14E-18	2.17E-17	30.97532486
GEMIN8	-0.56857545	4.66077004	-9.456307992	2.14E-18	2.17E-17	30.97478163
NPR3	-1.621289202	2.32942752	-9.454961537	2.16E-18	2.19E-17	30.96535909
COX19	-0.558465165	3.06357789	-9.452587211	2.20E-18	2.23E-17	30.94874505
EDN3	-2.417428668	2.83666885	-9.450427103	2.23E-18	2.26E-17	30.93363171
RBM48	-0.496192175	3.22439529	-9.446901595	2.29E-18	2.32E-17	30.90896882
TMEM19	-0.630583255	2.34336565	-9.444036274	2.33E-18	2.36E-17	30.88892756
SPAG16	-0.486395885	3.18017866	-9.443655624	2.34E-18	2.37E-17	30.88626536
SLC10A4	-0.752852495	1.00061928	-9.44363456	2.34E-18	2.37E-17	30.88611804
SPTY2D1	-0.378953314	1.08226893	-9.442731951	2.35E-18	2.38E-17	30.87980555
C9orf43	-0.466409363	0.85579645	-9.441380785	2.38E-18	2.40E-17	30.87035657

DNAJA2	-0.480875455	5.55668694	-9.440316298	2.39E-18	2.42E-17	30.86291286
NUBP2	-0.531209189	4.82753728	-9.440201297	2.40E-18	2.42E-17	30.8621087
FAM185A	-0.455286275	2.46291229	-9.438230454	2.43E-18	2.45E-17	30.84832819
MCCC2	-0.767154349	5.61752848	-9.436632967	2.46E-18	2.48E-17	30.83715927
HES1	-0.970661429	7.31645555	-9.433050208	2.52E-18	2.54E-17	30.81211352
OCEL1	-0.642531122	4.98585974	-9.431696876	2.55E-18	2.56E-17	30.80265407
PIGQ	-0.58261159	5.54624736	-9.429066821	2.59E-18	2.61E-17	30.7842725
TMEM92	1.497430344	1.58723301	9.425743639	2.66E-18	2.67E-17	30.76105018
CAVIN2	-1.410888492	5.74535749	-9.425337424	2.66E-18	2.68E-17	30.75821183
DUSP14	-0.786048409	5.68611726	-9.424972156	2.67E-18	2.68E-17	30.75565964
ACTR10	-0.531829148	5.60978127	-9.424357052	2.68E-18	2.69E-17	30.75136191
POMGNT	-0.5009068	5.60127513	-9.420650907	2.75E-18	2.76E-17	30.72546995
CRYZL1	-0.489628627	3.5077093	-9.418269391	2.80E-18	2.81E-17	30.70883473
EEF1AKI	-0.651049061	3.68235573	-9.416198797	2.84E-18	2.85E-17	30.694373
TATDN2	-0.458467334	4.17382721	-9.413918339	2.89E-18	2.89E-17	30.67844729
CIAO2A	-0.549033572	6.00513096	-9.413574818	2.89E-18	2.90E-17	30.67604845
RIOX2	-0.962517732	4.43621771	-9.41213906	2.92E-18	2.92E-17	30.66602288
ABHD14A	-0.798691998	5.80529574	-9.40979675	2.97E-18	2.97E-17	30.64966866
DHRS4L2	-0.606041417	3.72316943	-9.409320095	2.98E-18	2.98E-17	30.64634085
HEY1	-0.815987995	3.16862719	-9.408121421	3.01E-18	3.00E-17	30.63797256
BCKDHB	-0.696375287	3.95113973	-9.407464529	3.02E-18	3.01E-17	30.63338683
UBE2G2	-0.611793359	4.98109398	-9.405318633	3.07E-18	3.06E-17	30.61840751
CDC123	-0.456270829	5.93827795	-9.404586911	3.08E-18	3.07E-17	30.61330014
GABRB3	-1.344609822	1.68537721	-9.396381348	3.27E-18	3.25E-17	30.55603902
RPA3	-0.514074031	4.21943523	-9.39509683	3.30E-18	3.28E-17	30.54707743
ADGRA3	-0.893643756	3.62237429	-9.394383575	3.31E-18	3.30E-17	30.54210158
PRR15L	-1.34321228	6.33923312	-9.393165855	3.34E-18	3.32E-17	30.53360686
ZNF22	-0.692425113	4.73803178	-9.389460462	3.43E-18	3.41E-17	30.50776165
PHOSPH	-0.65156281	2.87077992	-9.388215312	3.46E-18	3.44E-17	30.4990778
CWH43	-1.71810303	1.9471883	-9.387232232	3.49E-18	3.46E-17	30.49222206
RAB28	-0.569857193	4.47692426	-9.386683007	3.50E-18	3.47E-17	30.48839206
TTYH2	-1.088008681	3.15304556	-9.386650181	3.50E-18	3.47E-17	30.48816316
MOCS1	-0.639380651	3.86308296	-9.385014501	3.54E-18	3.51E-17	30.47675747
SEM1	-0.520486881	4.21490004	-9.38426897	3.56E-18	3.52E-17	30.47155917
ALOX15B	2.218055805	4.43233389	9.380317733	3.66E-18	3.62E-17	30.44401203
APTX	-0.390200936	4.01311354	-9.37821795	3.71E-18	3.67E-17	30.4293751
EMC4	-0.434685833	5.6560867	-9.37805399	3.72E-18	3.68E-17	30.42823226
C2orf42	-0.532707589	3.25704135	-9.375160344	3.80E-18	3.75E-17	30.40806435
RGS14	-0.683817361	4.05629804	-9.372295368	3.87E-18	3.82E-17	30.38809925
TMEM13	-0.677366563	3.63779243	-9.371228274	3.90E-18	3.85E-17	30.38066377

SLC41A3	-0.556795669	4.9230357	-9.369892964	3.94E-18	3.89E-17	30.37135995
RGS6	-0.724287475	2.14015979	-9.367070794	4.02E-18	3.96E-17	30.3516985
TRPC6	-1.008024049	2.7825666	-9.366187764	4.04E-18	3.98E-17	30.34554722
DBT	-0.626184559	3.26244907	-9.36351457	4.12E-18	4.06E-17	30.32692717
FAM81B	-0.75950052	1.30046815	-9.363382944	4.12E-18	4.06E-17	30.32601041
CIB1	-0.538164976	8.12106436	-9.362879397	4.14E-18	4.07E-17	30.32250328
SNAPIN	-0.480200857	6.12158222	-9.362857924	4.14E-18	4.07E-17	30.32235372
CCDC13	-0.547302648	4.35231569	-9.360212005	4.22E-18	4.14E-17	30.30392684
C12orf10	-0.521710755	6.04165267	-9.35990558	4.23E-18	4.15E-17	30.30179298
HARBI1	-0.464025556	2.16662069	-9.358280313	4.28E-18	4.19E-17	30.29047563
ADSSL1	-0.581422366	3.07977173	-9.356600791	4.33E-18	4.24E-17	30.27878149
SFXN2	-0.653385217	2.89588626	-9.355764872	4.35E-18	4.26E-17	30.27296155
PPID	-0.576808481	4.87342551	-9.354914969	4.38E-18	4.29E-17	30.26704451
TMEM13	-0.411784495	3.42052054	-9.351979175	4.47E-18	4.38E-17	30.24660748
COL8A2	1.888704653	5.22140584	9.349148019	4.56E-18	4.46E-17	30.22690184
SPOP	-0.475265833	5.07928274	-9.348911399	4.57E-18	4.47E-17	30.22525503
BMP8A	-2.141960091	2.34674658	-9.348830333	4.57E-18	4.47E-17	30.22469083
DPH5	-0.49001494	4.55658505	-9.347872328	4.60E-18	4.49E-17	30.21802362
COLEC1	-0.765259935	0.94916135	-9.345834735	4.67E-18	4.56E-17	30.20384415
DCSTAM	3.49909712	4.34613684	9.345368398	4.68E-18	4.57E-17	30.20059916
TSG101	-0.480105187	5.82537319	-9.344639456	4.71E-18	4.59E-17	30.19552699
PLEK2	-0.636773151	6.10246542	-9.344196104	4.72E-18	4.60E-17	30.19244213
FAM241	-0.640146091	2.28271412	-9.344170957	4.72E-18	4.60E-17	30.19226715
ENKUR	-0.45099585	0.55702623	-9.343483919	4.75E-18	4.62E-17	30.18748685
GGT7	-0.739859069	5.11982409	-9.343337731	4.75E-18	4.62E-17	30.18646973
NWD1	-0.772032581	0.69880575	-9.342859184	4.77E-18	4.63E-17	30.1831402
RTRAF	-0.423495105	5.50622158	-9.337615699	4.95E-18	4.81E-17	30.14666377
TCEAL8	-0.538545368	7.15310104	-9.337211837	4.96E-18	4.82E-17	30.14385471
TXNDC1	-0.535484182	4.53671036	-9.336423229	4.99E-18	4.84E-17	30.13836973
TMEM20	-0.481856108	6.36734802	-9.332130823	5.14E-18	4.99E-17	30.10851883
COL9A3	-2.735534047	2.96120788	-9.327799807	5.30E-18	5.14E-17	30.07840625
SNRPB2	-0.409735261	5.8433921	-9.325335352	5.39E-18	5.22E-17	30.0612745
MLH1	-0.582679052	5.0607139	-9.323058278	5.48E-18	5.31E-17	30.04544731
IL17RC	-0.568519771	5.07506897	-9.319495818	5.62E-18	5.44E-17	30.02068964
UPF3B	-0.548196295	4.08395759	-9.318171043	5.67E-18	5.49E-17	30.01148416
TUBB3	0.762233099	0.87101727	9.317790837	5.69E-18	5.50E-17	30.00884234
OS9	-0.707097096	7.43686254	-9.317085964	5.72E-18	5.52E-17	30.00394475
VRK3	-0.521330472	3.42828232	-9.314934612	5.80E-18	5.60E-17	29.98899786
SFTPD	-0.903444888	4.22767065	-9.313182316	5.87E-18	5.67E-17	29.97682473
SDHAF4	-0.491514973	4.66679745	-9.311748285	5.93E-18	5.72E-17	29.96686342

HIST1H2	-1.129371082	6.73314912	-9.310280802	6.00E-18	5.78E-17	29.95667051
HSPA1L	-0.663039717	2.75111874	-9.309217961	6.04E-18	5.82E-17	29.94928867
TRAPPC	-0.468658241	4.82128105	-9.302883307	6.32E-18	6.08E-17	29.90530071
TMEM12	-0.693259845	5.94605418	-9.300781295	6.41E-18	6.17E-17	29.89070756
B4GAT1	-0.630313516	6.11288822	-9.299775072	6.46E-18	6.21E-17	29.88372246
FOXJ1	-1.456953285	1.5783657	-9.299084493	6.49E-18	6.23E-17	29.87892874
TM2D3	-0.519726219	3.88738516	-9.298103966	6.53E-18	6.27E-17	29.87212262
FAM162E	-1.015588783	2.20739482	-9.297769461	6.55E-18	6.28E-17	29.86980082
NFE2	-0.846553127	1.68430113	-9.29739779	6.56E-18	6.30E-17	29.86722108
DNAAF1	-0.783767846	0.98335515	-9.292977362	6.77E-18	6.49E-17	29.83654319
DNASE2	-0.528236516	6.91575606	-9.289665439	6.93E-18	6.64E-17	29.81356306
GALM	-0.489188396	5.45447568	-9.289487282	6.94E-18	6.65E-17	29.81232702
RNF135	-0.45474418	4.75347606	-9.288867795	6.97E-18	6.67E-17	29.80802913
IRS1	-1.231298529	4.32987278	-9.280026528	7.42E-18	7.10E-17	29.74670548
C1orf50	-0.423905825	3.76641333	-9.278708207	7.49E-18	7.16E-17	29.73756399
PIH1D1	-0.530844724	5.72589647	-9.275605075	7.65E-18	7.31E-17	29.7160488
C1QTNF	-0.359218999	0.41206792	-9.275460904	7.66E-18	7.31E-17	29.71504929
TBPL1	-0.469842782	2.9294955	-9.272635201	7.81E-18	7.46E-17	29.69546087
MRPS35	-0.559384817	6.27248659	-9.272294254	7.83E-18	7.47E-17	29.69309754
COMTD1	-0.822870022	4.33349669	-9.270871042	7.91E-18	7.54E-17	29.68323281
ROGDI	-0.582501196	3.80761587	-9.270620062	7.92E-18	7.55E-17	29.68149326
DNAJC8	-0.462795392	6.81567554	-9.270068416	7.95E-18	7.58E-17	29.67766988
CA2	-1.303859239	3.92036097	-9.267631742	8.09E-18	7.70E-17	29.66078298
METTL2!	-0.473827077	2.66937452	-9.26560494	8.21E-18	7.81E-17	29.6467383
IQCC	-0.627270037	3.41205685	-9.265286413	8.23E-18	7.82E-17	29.64453121
CLPTM1I	-0.573408956	6.30130348	-9.2651239	8.24E-18	7.83E-17	29.64340516
MSRA	-0.44757634	3.94227417	-9.264302377	8.28E-18	7.87E-17	29.63771302
C2orf73	-0.477987806	0.69973492	-9.263828191	8.31E-18	7.89E-17	29.6344276
COPS7A	-0.483728641	6.35107153	-9.262701481	8.38E-18	7.95E-17	29.62662148
PIN1	-0.496337655	4.6748552	-9.25899242	8.60E-18	8.15E-17	29.60092755
PEX12	-0.669468167	3.83481028	-9.258188368	8.65E-18	8.19E-17	29.59535828
RGS8	-1.52424957	1.04604495	-9.258098551	8.65E-18	8.19E-17	29.59473618
PDE8B	-1.447266584	6.39004694	-9.255251865	8.83E-18	8.35E-17	29.57502061
DHRSX	-0.530671203	4.59454941	-9.254216511	8.89E-18	8.41E-17	29.56785071
MARVEL	-0.819057369	5.16896269	-9.251284044	9.07E-18	8.58E-17	29.54754532
ZC4H2	-0.688747571	2.67133446	-9.251189751	9.08E-18	8.58E-17	29.54689246
RBX1	-0.546120764	5.68736925	-9.250908547	9.10E-18	8.59E-17	29.54494548
SPTBN2	1.218205751	2.34102912	9.248499632	9.25E-18	8.73E-17	29.52826806
PRKN	-0.500618833	1.62533708	-9.248000733	9.29E-18	8.76E-17	29.52481436
FAM220A	-0.543813109	4.51935754	-9.247841642	9.30E-18	8.76E-17	29.52371304

HM13	-0.530606136	5.71369769	-9.243018439	9.62E-18	9.06E-17	29.49032868
SELENO	-1.630507	1.79033603	-9.239761627	9.84E-18	9.27E-17	29.46779117
PTPA	-0.5044351	6.62790114	-9.23907638	9.89E-18	9.30E-17	29.4630497
PPP2CA	-0.4746939	6.27456217	-9.235035942	1.02E-17	9.57E-17	29.43509593
CTH	-0.786297662	2.4909768	-9.234517644	1.02E-17	9.60E-17	29.43151053
DMAC2L	-0.459954806	3.23038685	-9.227564824	1.07E-17	1.01E-16	29.38342305
IDH3G	-0.378394681	6.06721304	-9.22575915	1.09E-17	1.02E-16	29.37093751
RABEP2	-0.458415609	4.58126477	-9.224978582	1.09E-17	1.02E-16	29.36554055
FGGY	-0.482218019	2.49246976	-9.222694535	1.11E-17	1.04E-16	29.34974965
RING1	-0.518292805	7.06169357	-9.222494458	1.11E-17	1.04E-16	29.3483665
TEKT4	-0.822274272	0.74476383	-9.220995238	1.12E-17	1.05E-16	29.33800271
KLHL8	-0.826172081	3.79089408	-9.217059452	1.15E-17	1.08E-16	29.31079949
PGK1	-0.766161053	6.98902752	-9.214408324	1.18E-17	1.10E-16	29.29247879
SHANK2	-0.895831422	1.7863554	-9.209981681	1.21E-17	1.13E-16	29.26189419
NME1	-0.511713763	4.79616894	-9.207593402	1.23E-17	1.15E-16	29.24539613
PTPRR	-0.59688859	1.06811246	-9.206890016	1.24E-17	1.16E-16	29.2405376
AKT2	-0.820343241	5.83581173	-9.206656142	1.24E-17	1.16E-16	29.23892219
NABP2	-0.407300927	5.61550714	-9.205740572	1.25E-17	1.17E-16	29.23259839
ROBO4	-0.993007548	4.06733674	-9.203266061	1.27E-17	1.19E-16	29.21550862
SLCO2A1	-1.148813178	5.98191145	-9.202707956	1.28E-17	1.19E-16	29.21165449
TMED1	-0.699029936	5.28079138	-9.201466107	1.29E-17	1.20E-16	29.203079
LINGO2	-1.103976877	0.89281026	-9.19407893	1.35E-17	1.26E-16	29.15207947
LSM2	-0.52139649	6.31502823	-9.192979931	1.37E-17	1.27E-16	29.14449396
HIRIP3	-0.495682269	4.52776669	-9.185957539	1.43E-17	1.33E-16	29.09603476
STARD3I	-0.590119308	4.8391109	-9.184842058	1.45E-17	1.34E-16	29.08833891
HCCS	-0.505872842	4.55773176	-9.184217743	1.45E-17	1.35E-16	29.08403187
PDCD5	-0.446712782	5.49621756	-9.180831012	1.49E-17	1.38E-16	29.06067003
RABAC1	-0.642721541	6.67615722	-9.179459182	1.50E-17	1.39E-16	29.05120831
CLUL1	-0.877287407	1.57448297	-9.178167528	1.51E-17	1.40E-16	29.04230022
LRRC27	-0.465075821	2.46452622	-9.175706991	1.54E-17	1.43E-16	29.0253325
AGBL5	-0.528562876	4.73646788	-9.17355239	1.56E-17	1.45E-16	29.01047638
THAP1	-0.543327838	3.90394782	-9.170131668	1.60E-17	1.48E-16	28.98689386
PAK1	-0.719166983	5.53141288	-9.168315858	1.62E-17	1.50E-16	28.97437743
RAB2A	-0.491787407	6.45366881	-9.166571225	1.64E-17	1.52E-16	28.9623528
WDR72	-1.402425438	3.7282771	-9.16546878	1.65E-17	1.53E-16	28.95475496
CEP19	-0.571323988	2.42395834	-9.165436009	1.65E-17	1.53E-16	28.95452911
INPP5J	-1.267424343	7.33929594	-9.164766568	1.66E-17	1.54E-16	28.94991569
NMRAL1	-0.468583696	4.97767291	-9.16421649	1.67E-17	1.54E-16	28.94612496
ZNHIT1	-0.63511284	5.62047056	-9.162192687	1.69E-17	1.56E-16	28.93217945
ANAPC1	-0.463000784	3.30799533	-9.161826546	1.70E-17	1.57E-16	28.92965663

ALKBH5	-0.505625699	6.74705387	-9.161366135	1.70E-17	1.57E-16	28.92648433
CCNJL	-0.66966722	3.06256232	-9.160662362	1.71E-17	1.58E-16	28.92163539
AARS	-0.731188824	6.86042483	-9.160555292	1.71E-17	1.58E-16	28.9208977
SPG21	-0.485277263	6.3542664	-9.156521218	1.76E-17	1.62E-16	28.89310705
ZNF480	-0.496100434	3.18936956	-9.156271909	1.76E-17	1.62E-16	28.89138977
YWHAE	-0.416108619	8.83458606	-9.153926067	1.79E-17	1.65E-16	28.87523236
KBTBD3	-0.440665459	2.27759117	-9.152439267	1.81E-17	1.66E-16	28.86499285
FAM86C:	-0.453558477	3.34958157	-9.150872882	1.83E-17	1.68E-16	28.85420614
UGT8	-1.183006916	2.2544117	-9.141951366	1.95E-17	1.79E-16	28.79278707
KBTBD7	-0.657311589	3.33373591	-9.141825573	1.95E-17	1.79E-16	28.79192128
EIF2B5	-0.435578095	4.63435584	-9.140807418	1.97E-17	1.80E-16	28.78491389
ALKBH7	-0.677396046	6.67114461	-9.140490896	1.97E-17	1.80E-16	28.78273553
TXNDC1:	-0.47651862	5.73987584	-9.137373463	2.01E-17	1.84E-16	28.76128282
HPCAL1	-0.728186827	4.75864917	-9.137199534	2.02E-17	1.84E-16	28.76008602
SUMF1	-0.540612117	4.78043241	-9.137177071	2.02E-17	1.84E-16	28.75993146
EID2B	-0.578103977	3.14362519	-9.13692181	2.02E-17	1.85E-16	28.75817506
COX11	-0.555522343	4.22905364	-9.136790023	2.02E-17	1.85E-16	28.75726827
ZNF30	-0.486832412	2.30031035	-9.135264512	2.04E-17	1.87E-16	28.7467721
PLEKHN	1.160616396	2.78168882	9.133852714	2.06E-17	1.88E-16	28.73705912
RHBDD3	-0.584226664	4.39257929	-9.133212198	2.07E-17	1.89E-16	28.73265271
SMTN	-0.835226892	3.14123347	-9.133190053	2.07E-17	1.89E-16	28.73250036
CGRRF1	-0.5277856	3.50152492	-9.131440295	2.10E-17	1.91E-16	28.72046376
MRM2	-0.460403715	5.04513739	-9.130899629	2.11E-17	1.92E-16	28.71674475
SFTPC	-0.969710702	0.93803859	-9.119130033	2.29E-17	2.08E-16	28.63581438
RAB4A	-0.598721753	5.48202978	-9.118872792	2.29E-17	2.08E-16	28.63404612
GALNT1{	-1.066812716	5.59254712	-9.116903837	2.32E-17	2.11E-16	28.62051251
KHDRBS	-1.428878971	2.05419109	-9.116091967	2.33E-17	2.12E-16	28.61493255
PBX4	-0.972909885	2.61676565	-9.116014998	2.34E-17	2.12E-16	28.61440356
LY6G5C	-0.515735658	1.70581033	-9.115919179	2.34E-17	2.12E-16	28.61374502
CHI3L1	3.094484769	5.85748835	9.112419931	2.39E-17	2.17E-16	28.58969791
STK40	-0.70249005	5.14807658	-9.108310485	2.46E-17	2.24E-16	28.56146347
RWDD2/	-0.521725311	3.04618968	-9.107966751	2.47E-17	2.24E-16	28.5591021
POLR3H	-0.558261916	4.420784	-9.107604043	2.48E-17	2.24E-16	28.55661043
ASB9	-0.660281121	2.33981028	-9.105467255	2.51E-17	2.28E-16	28.5419325
RPL17	-0.625734683	5.61253536	-9.105254651	2.52E-17	2.28E-16	28.54047219
MZT2A	-0.807881342	5.0198909	-9.102969809	2.56E-17	2.31E-16	28.52477939
SPATA4!	-0.353085671	0.46479222	-9.10079421	2.60E-17	2.35E-16	28.50983875
ANKMY2	-0.658454538	4.92999308	-9.098327877	2.64E-17	2.39E-16	28.49290373
ASPRV1	-0.497366744	1.27242399	-9.09420597	2.72E-17	2.46E-16	28.46460597
ARF1	-0.424728563	8.86919857	-9.08802289	2.84E-17	2.56E-16	28.42217007

MTIF2	-0.465618058	4.77871517	-9.085921757	2.88E-17	2.60E-16	28.40775285
GPIHBP1	-1.008442839	2.19718594	-9.083975447	2.92E-17	2.63E-16	28.3943995
ZNF330	-0.582223361	5.47075602	-9.083504632	2.93E-17	2.64E-16	28.39116953
GPKOW	-0.541197589	5.46738133	-9.080852406	2.98E-17	2.69E-16	28.37297581
CYP27B1	-0.964713078	2.44597295	-9.074388808	3.12E-17	2.81E-16	28.32864826
THRA	-0.561507426	4.96531656	-9.072877091	3.15E-17	2.84E-16	28.31828318
CBY1	-0.520117507	5.26521977	-9.072017253	3.17E-17	2.85E-16	28.3123881
TCTEX1D	-0.540377605	3.72354193	-9.066601786	3.29E-17	2.96E-16	28.27526604
ECHDC3	-0.784684582	4.72157451	-9.065499166	3.32E-17	2.98E-16	28.26770916
NEXMIF	-0.373689198	0.40516616	-9.064225221	3.35E-17	3.01E-16	28.25897869
WDCP	-0.505541787	2.51302032	-9.062673583	3.38E-17	3.04E-16	28.248346
TMEM60	-0.426794068	5.77685165	-9.061627572	3.41E-17	3.06E-16	28.24117868
C17orf80	-0.563022007	4.09818775	-9.059865497	3.45E-17	3.09E-16	28.2291058
CWC27	-0.489184032	4.16894178	-9.058113517	3.49E-17	3.13E-16	28.21710328
FOPNL	-0.557981819	5.57884148	-9.057973323	3.50E-17	3.13E-16	28.21614289
PARD6A	-0.648510916	2.73768988	-9.057233809	3.51E-17	3.15E-16	28.211077
PHF5A	-0.518551776	5.43845484	-9.056912542	3.52E-17	3.15E-16	28.2088763
NQO2	-0.44265351	2.97430617	-9.056582804	3.53E-17	3.16E-16	28.2066176
WBP1	-0.717638375	4.71254615	-9.054840612	3.57E-17	3.19E-16	28.19468436
SMIM12	-0.462853631	3.75437404	-9.054495564	3.58E-17	3.20E-16	28.19232107
PSMB4	-0.429935436	8.01854213	-9.053870672	3.60E-17	3.21E-16	28.1880412
DCTD	-0.623600147	6.70789076	-9.053527673	3.61E-17	3.22E-16	28.18569208
PET117	-0.41540086	3.63632891	-9.052910123	3.62E-17	3.23E-16	28.18146273
GAS8	-0.515759	4.44286366	-9.052757167	3.63E-17	3.23E-16	28.18041522
BEGAIN	-0.3348974	0.43238864	-9.05098639	3.67E-17	3.27E-16	28.16828882
SLC26A4	-2.301169321	5.79010767	-9.050935257	3.67E-17	3.27E-16	28.16793867
MTERF1	-0.425343309	2.58284155	-9.050581486	3.68E-17	3.28E-16	28.16551617
PSMD3	-0.460579555	5.90821256	-9.050483242	3.68E-17	3.28E-16	28.16484345
KYAT1	-0.537084603	3.14034055	-9.050365659	3.69E-17	3.28E-16	28.16403829
MT1H	-3.205596739	4.09908665	-9.049564841	3.71E-17	3.29E-16	28.15855482
DENND6	-0.679863378	4.01131812	-9.046342298	3.79E-17	3.37E-16	28.13649152
CDC25A	-0.599243436	1.75417147	-9.045695325	3.81E-17	3.38E-16	28.13206248
ZNF433	-0.50737086	3.01684209	-9.045552865	3.81E-17	3.38E-16	28.13108725
LRP2	-1.579383897	4.10232514	-9.044811392	3.83E-17	3.40E-16	28.12601151
APOA1	-1.610998113	1.88667323	-9.042239304	3.90E-17	3.46E-16	28.10840601
MAP6	-0.745704867	2.52974921	-9.0400957	3.96E-17	3.51E-16	28.09373537
ACOT8	-0.431868385	4.03121522	-9.039828992	3.97E-17	3.51E-16	28.09191017
PPP1CC	-0.462590512	5.32768874	-9.039645466	3.97E-17	3.51E-16	28.09065423
TRIM16	-0.768168874	2.74503109	-9.039387712	3.98E-17	3.52E-16	28.08889035
QRFPR	-0.922348703	1.17029594	-9.039247081	3.98E-17	3.52E-16	28.08792799

PNMA8A	-1.083600642	5.00142092	-9.039166081	3.98E-17	3.52E-16	28.0873737
THOC7	-0.397119013	5.71801304	-9.037624862	4.03E-17	3.56E-16	28.07682739
HSDL1	-0.595166673	4.69872144	-9.035470433	4.09E-17	3.61E-16	28.06208653
STUB1	-0.59920766	6.14278759	-9.035258756	4.09E-17	3.61E-16	28.06063831
MRPL42	-0.40476149	3.09922176	-9.033284163	4.15E-17	3.66E-16	28.04712967
CD24	-0.860424425	10.2376986	-9.027542488	4.32E-17	3.81E-16	28.00785818
TRMT61/	-0.541111313	4.78394128	-9.026000587	4.37E-17	3.84E-16	27.99731418
ABLIM2	-0.750657306	3.32537884	-9.024445797	4.41E-17	3.88E-16	27.98668299
TTLL1	-0.557579142	4.1032741	-9.019927673	4.55E-17	4.01E-16	27.95579476
SGK1	-1.108866687	4.38531775	-9.0189375	4.58E-17	4.03E-16	27.94902648
LRRC8D	-0.637809369	4.15411595	-9.018392824	4.60E-17	4.04E-16	27.94530354
CUTA	-0.511936859	8.18620714	-9.016736366	4.65E-17	4.09E-16	27.93398213
AHCY	-0.58307976	7.24593825	-9.011751538	4.82E-17	4.23E-16	27.89991874
TXNL4B	-0.512308684	3.21129398	-9.009035249	4.91E-17	4.31E-16	27.8813613
ZNF511	-0.545133538	3.81355443	-9.007877102	4.95E-17	4.34E-16	27.87344982
SVBP	-0.515957461	5.18666875	-9.005945721	5.02E-17	4.40E-16	27.86025744
FBXL2	-0.623754401	3.21766068	-9.003724092	5.09E-17	4.46E-16	27.8450843
WASF3	-1.052845191	3.67121893	-9.003295204	5.11E-17	4.47E-16	27.84215534
VLDLR	-1.058439767	3.15161053	-9.001336003	5.18E-17	4.53E-16	27.82877645
MAGOHE	-0.368891857	2.91269236	-8.996945609	5.34E-17	4.67E-16	27.79880104
LRGUK	-0.385302831	0.48337585	-8.996680922	5.35E-17	4.68E-16	27.79699414
CAAP1	-0.610707902	5.04646551	-8.995130828	5.41E-17	4.72E-16	27.78641284
PSMD8	-0.468498374	6.98637003	-8.993888804	5.45E-17	4.76E-16	27.77793518
SEC13	-0.466863546	5.92809354	-8.993736769	5.46E-17	4.76E-16	27.77689748
CCNQ	-0.44936883	5.17076286	-8.993578003	5.46E-17	4.77E-16	27.77581384
ZCCHC3	-0.556784637	4.96007911	-8.993417744	5.47E-17	4.77E-16	27.77472003
PHB2	-0.44779471	7.63274883	-8.99255364	5.50E-17	4.80E-16	27.76882245
MED31	-0.44872456	3.44339039	-8.991787268	5.53E-17	4.82E-16	27.76359214
KCNJ8	-0.94029964	3.3628738	-8.989425092	5.62E-17	4.90E-16	27.7474723
EIF4E	-0.472332335	3.02996277	-8.988753982	5.65E-17	4.92E-16	27.74289294
HDAC3	-0.553393208	4.95140715	-8.986618671	5.73E-17	4.99E-16	27.72832372
DNASE1	-1.257337021	2.61096384	-8.985944656	5.76E-17	5.01E-16	27.72372529
SCN4B	-1.177647377	3.11795484	-8.983887703	5.84E-17	5.08E-16	27.70969294
RAVER2	-0.582552006	2.78165491	-8.983552164	5.86E-17	5.09E-16	27.70740408
NLK	-0.554780898	4.37199773	-8.983197214	5.87E-17	5.10E-16	27.70498286
MRPS12	-0.480065907	4.81610122	-8.980393423	5.99E-17	5.19E-16	27.68585909
SPINK5	-1.031320454	1.19588687	-8.977531984	6.10E-17	5.29E-16	27.66634532
NAGS	-0.884712139	4.02599262	-8.975236821	6.20E-17	5.38E-16	27.65069563
ASTE1	-0.555841208	3.27953113	-8.973236115	6.29E-17	5.45E-16	27.63705541
ACTR5	-0.431768209	3.2911405	-8.971625948	6.36E-17	5.51E-16	27.62607892

GPR160	-0.793579237	2.50039947	-8.970952919	6.39E-17	5.53E-16	27.62149119
ELP3	-0.500612019	4.39726393	-8.970622208	6.40E-17	5.54E-16	27.61923695
EBPL	-0.626916356	5.20796139	-8.970190533	6.42E-17	5.55E-16	27.61629457
LXN	-0.567069653	4.64850379	-8.967429424	6.55E-17	5.66E-16	27.59747605
PRR13	-0.451620198	6.42006734	-8.967013424	6.56E-17	5.67E-16	27.59464104
HIST1H2	-0.975591702	6.89495443	-8.962526443	6.77E-17	5.84E-16	27.56406691
FBXL17	-0.641575967	4.19089411	-8.961689643	6.81E-17	5.88E-16	27.55836586
CFAP41(	-0.547962836	4.27388064	-8.958939973	6.94E-17	5.99E-16	27.53963454
DGKI	-1.382317795	1.99746597	-8.957872875	6.99E-17	6.03E-16	27.53236606
AKR1E2	-0.619281119	0.97907652	-8.956941563	7.04E-17	6.06E-16	27.52602284
NUDT18	-0.607626551	4.35555655	-8.955062516	7.13E-17	6.14E-16	27.51322559
MARK3	-0.47992583	4.53404869	-8.952790939	7.24E-17	6.23E-16	27.49775688
TIMM50	-0.378383547	4.9500624	-8.95107025	7.33E-17	6.30E-16	27.48604091
RAG1	-0.823161359	0.86758165	-8.949542109	7.41E-17	6.36E-16	27.47563696
PCBD2	-0.464790031	2.55273968	-8.949531081	7.41E-17	6.36E-16	27.47556188
CD2BP2	-0.557347339	6.40719572	-8.949083593	7.43E-17	6.38E-16	27.47251545
PPT2	-0.549586216	2.74584166	-8.942213251	7.79E-17	6.69E-16	27.42575318
TMEM26	-0.720978043	4.30713574	-8.940149239	7.90E-17	6.78E-16	27.41170836
PAIP2	-0.638650984	5.58118205	-8.938242556	8.01E-17	6.87E-16	27.39873561
PLEKHB:	-0.981040327	4.97275785	-8.936172045	8.12E-17	6.96E-16	27.38464983
TMEM19	-0.552673171	3.58849734	-8.933917034	8.25E-17	7.07E-16	27.36931084
PEG3	-0.932821362	2.13478548	-8.933474599	8.27E-17	7.08E-16	27.36630155
UBXN6	-0.502061648	7.1004416	-8.93294171	8.31E-17	7.11E-16	27.36267713
OAT	-0.714162085	5.85745342	-8.931661259	8.38E-17	7.17E-16	27.35396867
ID3	-1.128218985	7.69907463	-8.931308668	8.40E-17	7.18E-16	27.35157078
PLAAT1	-0.929239149	3.09025952	-8.930599152	8.44E-17	7.21E-16	27.34674567
PTPRE	1.416956001	3.59362581	8.930583572	8.44E-17	7.21E-16	27.34663972
BET1	-0.465613373	3.67599529	-8.929053806	8.53E-17	7.28E-16	27.33623715
TMEM16	-0.820458136	4.01847231	-8.928777285	8.55E-17	7.29E-16	27.33435688
TOMM20	-0.429118597	1.23741103	-8.928325683	8.57E-17	7.31E-16	27.33128617
DNAJB6	-0.520760443	3.57533884	-8.928079446	8.59E-17	7.32E-16	27.32961189
ARMC8	-0.501002291	3.50338474	-8.926165449	8.70E-17	7.41E-16	27.31659857
RPP38	-0.40868132	4.49223088	-8.926020294	8.71E-17	7.41E-16	27.31561171
RASGRF	-0.740091936	2.46378998	-8.926017526	8.71E-17	7.41E-16	27.31559289
IFT88	-0.581448865	3.67802397	-8.923654023	8.85E-17	7.53E-16	27.29952551
LIAS	-0.490502569	3.59722627	-8.922704013	8.91E-17	7.58E-16	27.29306786
GTF3C6	-0.44314728	6.71484245	-8.922334334	8.93E-17	7.59E-16	27.29055508
RMND5B	-0.434291592	4.50955341	-8.921017851	9.02E-17	7.66E-16	27.28160713
ZDHHC2	-0.431812985	4.09445921	-8.920848681	9.03E-17	7.66E-16	27.28045735
PIGV	-0.568935445	4.61189307	-8.920592406	9.04E-17	7.67E-16	27.27871559

ACSF3	-0.550428043	3.20076828	-8.91844036	9.18E-17	7.78E-16	27.26409028
RIOK2	-0.474959686	3.642586	-8.91810398	9.20E-17	7.80E-16	27.26180441
ZNF226	-0.48732851	3.34849024	-8.914659528	9.42E-17	7.98E-16	27.23840023
P2RY6	1.06165774	2.09132971	8.913493448	9.50E-17	8.04E-16	27.23047808
PEPD	-0.526102662	5.71214274	-8.911130258	9.65E-17	8.17E-16	27.21442464
C6orf89	-0.621028264	5.97027639	-8.910673746	9.68E-17	8.19E-16	27.21132376
SLC2A4	-1.024320384	1.81501353	-8.907628048	9.89E-17	8.36E-16	27.19063781
HHEX	-1.027049485	7.20059987	-8.904608585	1.01E-16	8.53E-16	27.1701337
PPP2R5I	-0.493172075	4.47852841	-8.904404398	1.01E-16	8.54E-16	27.16874727
RBBP8N	-0.989737852	2.91726586	-8.904071222	1.01E-16	8.55E-16	27.16648504
B3GNT9	-0.816351479	5.70155522	-8.901127921	1.03E-16	8.73E-16	27.14650227
DPM2	-0.56686152	5.89044465	-8.899635241	1.04E-16	8.81E-16	27.13636943
WBP1L	-0.644600314	6.43866651	-8.894607956	1.08E-16	9.12E-16	27.10224903
ALDH1A:	-1.388390161	7.18823976	-8.892452367	1.10E-16	9.25E-16	27.08762205
ANKRD1	-0.502603349	0.98331115	-8.892262395	1.10E-16	9.26E-16	27.08633307
RAB10	-0.595025352	6.806875	-8.89110272	1.11E-16	9.32E-16	27.07846483
GPT2	-0.808216352	2.94988895	-8.8896274	1.12E-16	9.42E-16	27.06845577
KRT80	1.575783064	3.51512315	8.886823753	1.14E-16	9.59E-16	27.0494373
PPP3CC	-0.453895241	4.43221854	-8.886243261	1.15E-16	9.63E-16	27.04549994
DDTL	-0.555285889	4.45828408	-8.88513803	1.15E-16	9.70E-16	27.03800376
C9orf85	-0.472958473	2.84354222	-8.884787323	1.16E-16	9.71E-16	27.03562521
TLE1	-0.750599088	3.58079091	-8.883016312	1.17E-16	9.83E-16	27.02361467
ARHGEF	-0.256468697	0.49165993	-8.882208303	1.18E-16	9.88E-16	27.01813539
TARS2	-0.446685353	4.59148339	-8.881556933	1.18E-16	9.92E-16	27.0137185
PXMP4	-0.875921349	4.00211731	-8.88037602	1.19E-16	9.99E-16	27.00571125
SDHC	-0.567244283	4.1277605	-8.875680784	1.23E-16	1.03E-15	26.97388048
FPGT	-0.575347534	3.47540075	-8.875385915	1.23E-16	1.03E-15	26.97188174
STYXL1	-0.441926651	5.43039594	-8.873758475	1.25E-16	1.04E-15	26.96085098
INPP5A	-0.635321625	4.46919151	-8.871928999	1.26E-16	1.06E-15	26.94845209
C18orf21	-0.413298696	4.23697289	-8.870674535	1.27E-16	1.07E-15	26.93995101
EMC10	-0.564711944	6.5925332	-8.87049327	1.28E-16	1.07E-15	26.93872269
CCDC15	-1.063753888	2.19917845	-8.869725879	1.28E-16	1.07E-15	26.93352271
RRAGA	-0.536376942	7.40210183	-8.869254974	1.29E-16	1.07E-15	26.93033188
EARS2	-0.5396216	4.0686028	-8.869168723	1.29E-16	1.07E-15	26.92974746
CD34	-0.915899026	4.78754829	-8.867915342	1.30E-16	1.08E-15	26.92125511
TMBIM4	-0.515893724	5.73942851	-8.86751501	1.30E-16	1.09E-15	26.91854277
CIAO2B	-0.481406322	7.67545693	-8.866312718	1.31E-16	1.09E-15	26.91039736
STK32C	-0.518571848	2.70012298	-8.865884768	1.32E-16	1.10E-15	26.90749819
RAB40B	-0.580165503	3.02786759	-8.864705514	1.33E-16	1.11E-15	26.89950963
GOT1	-0.871069501	5.86991908	-8.861604608	1.36E-16	1.13E-15	26.87850601

GAL3ST3	-1.085111649	4.90996628	-8.861058957	1.36E-16	1.13E-15	26.87481051
NAT16	-1.198830566	0.98139691	-8.858739554	1.38E-16	1.15E-15	26.85910336
KCNK3	-1.378585026	2.95784869	-8.858336719	1.39E-16	1.15E-15	26.85637555
MYO1G	1.599108521	2.76894449	8.856981784	1.40E-16	1.16E-15	26.84720107
PPIL6	-0.635490372	2.85224181	-8.855943433	1.41E-16	1.17E-15	26.84017073
ATAD3A	-0.442610082	4.31564449	-8.855685684	1.41E-16	1.17E-15	26.83842565
DGCR6L	-0.57640364	7.16359036	-8.853225816	1.44E-16	1.19E-15	26.82177268
MAPRE1	-0.558865606	7.25632573	-8.848641839	1.48E-16	1.23E-15	26.79074629
PDE10A	-1.047900851	1.96575894	-8.848071077	1.49E-16	1.23E-15	26.78688372
URI1	-0.559737541	5.46137268	-8.847855102	1.49E-16	1.23E-15	26.78542216
NUDT13	-0.456873482	1.76528217	-8.846961648	1.50E-16	1.24E-15	26.77937615
GLO1	-0.476542697	6.73354295	-8.845429708	1.52E-16	1.25E-15	26.76901024
PBDC1	-0.47133067	6.01772631	-8.845087212	1.52E-16	1.26E-15	26.76669286
ETNPPL	-0.990583183	0.70342483	-8.842865272	1.54E-16	1.27E-15	26.75166005
HIRA	-0.865473715	5.68825311	-8.841435611	1.56E-16	1.29E-15	26.74198855
RANBP3	-0.765178285	1.08959516	-8.841197671	1.56E-16	1.29E-15	26.740379
REPIN1	-0.519952315	5.93420391	-8.840486629	1.57E-16	1.29E-15	26.73556926
AKIP1	-0.462151126	4.97280868	-8.836603345	1.61E-16	1.33E-15	26.70930497
MT1E	-1.617089764	7.79092003	-8.830283032	1.68E-16	1.39E-15	26.66657111
ZADH2	-0.547489409	4.08450658	-8.828740216	1.70E-16	1.40E-15	26.65614205
CYTH3	-0.754766833	4.57312938	-8.827564057	1.71E-16	1.41E-15	26.64819216
CFL2	-0.590049526	3.93068204	-8.82714617	1.72E-16	1.41E-15	26.64536771
C8orf88	-0.950027036	1.81794347	-8.825113748	1.74E-16	1.43E-15	26.63163183
GPM6A	-0.963862076	1.31652991	-8.824825869	1.75E-16	1.44E-15	26.62968637
PARP8	-0.654241194	4.28868749	-8.822037242	1.78E-16	1.46E-15	26.61084283
CNPY2	-0.539846043	5.16711952	-8.821877189	1.78E-16	1.46E-15	26.60976141
ACTBL2	0.842861207	0.81846295	8.821133242	1.79E-16	1.47E-15	26.60473493
CHMP2A	-0.49921433	7.86847035	-8.820092979	1.80E-16	1.48E-15	26.59770678
DYNLRB	-0.476665943	7.33584185	-8.819973479	1.81E-16	1.48E-15	26.59689944
ANKRD3	-0.778661168	2.60791544	-8.817964893	1.83E-16	1.50E-15	26.58333051
ALG8	-0.470779442	5.0342632	-8.816830808	1.84E-16	1.51E-15	26.57566997
LCLAT1	-0.61354996	3.12448116	-8.814956639	1.87E-16	1.53E-15	26.56301143
NME7	-0.610890414	3.29231938	-8.814075422	1.88E-16	1.54E-15	26.55706001
PDGFB	-0.798821671	4.51205589	-8.813885715	1.88E-16	1.54E-15	26.55577883
ATXN10	-0.611245263	5.85702799	-8.809215045	1.94E-16	1.59E-15	26.52424041
ZC3H10	-0.423765952	2.49414993	-8.807530016	1.97E-16	1.61E-15	26.51286454
CFAP298	-0.483044401	4.55730237	-8.806626674	1.98E-16	1.62E-15	26.50676643
NSL1	-0.471198617	3.95696481	-8.80655595	1.98E-16	1.62E-15	26.50628901
STT3B	-0.670518206	6.63295118	-8.805994258	1.99E-16	1.62E-15	26.50249743
MANSC1	-0.632877347	3.85218058	-8.803363225	2.02E-16	1.65E-15	26.48473892

MRPS18.	-0.507562495	5.35668596	-8.802700225	2.03E-16	1.66E-15	26.48026436
TRIAP1	-0.436034613	5.2739989	-8.802228153	2.04E-16	1.66E-15	26.47707847
ADPRM	-0.567363263	4.16787546	-8.800987807	2.06E-16	1.67E-15	26.46870815
GORASF	-0.544133906	4.61332154	-8.797953394	2.10E-16	1.71E-15	26.44823343
ZNF420	-0.536770768	2.97280273	-8.797209968	2.11E-16	1.72E-15	26.44321773
CHST14	-0.727548368	4.365592	-8.797060865	2.11E-16	1.72E-15	26.4422118
WDR4	-0.45470433	3.23292848	-8.796671554	2.12E-16	1.72E-15	26.43958534
NMRK1	-0.586824371	5.07324156	-8.792501424	2.18E-16	1.77E-15	26.4114557
RAE1	-0.458494819	3.79909845	-8.791756704	2.19E-16	1.78E-15	26.40643293
NECTIN4	1.787303336	3.72350288	8.790899849	2.20E-16	1.79E-15	26.40065416
CFAP29	-0.143051176	0.1306983	-8.789866025	2.22E-16	1.80E-15	26.39368227
CCDC71	-0.525495537	5.75094619	-8.788212031	2.24E-16	1.82E-15	26.382529
NUP35	-0.489704381	2.906469	-8.783810786	2.31E-16	1.87E-15	26.35285584
RNASET	-0.593250388	4.26966308	-8.779926414	2.37E-16	1.92E-15	26.32667403
STT3A	-0.630091369	6.0169234	-8.779734077	2.38E-16	1.93E-15	26.32537778
TMEM20	-0.481024731	6.21061035	-8.777434504	2.41E-16	1.96E-15	26.30988109
TBXA2R	-0.831974204	2.44896786	-8.777276018	2.42E-16	1.96E-15	26.30881314
COMMD:	-0.522049377	4.98774714	-8.777050632	2.42E-16	1.96E-15	26.3072944
BTD	-0.641404341	4.6172861	-8.774872103	2.46E-16	1.99E-15	26.29261577
DZIP3	-0.614874602	3.68571919	-8.774672867	2.46E-16	1.99E-15	26.29127344
CRAT	-0.523215901	5.22551876	-8.774303695	2.47E-16	1.99E-15	26.28878623
COX15	-0.488079989	4.75443713	-8.770336073	2.53E-16	2.05E-15	26.26205886
CCNB1IF	-0.593327038	5.38064924	-8.770149953	2.54E-16	2.05E-15	26.26080525
ANP32A	-0.489096194	5.50449243	-8.769928585	2.54E-16	2.05E-15	26.25931424
TMEM11	-0.515861425	6.80059376	-8.769148899	2.56E-16	2.06E-15	26.25406288
ZNF688	-0.514483351	3.85624666	-8.769102666	2.56E-16	2.06E-15	26.2537515
LRRC73	-0.597574992	1.64531341	-8.767995491	2.58E-16	2.07E-15	26.24629487
SLC20A2	-0.784350732	6.23183776	-8.76655123	2.60E-16	2.09E-15	26.2365688
GPR155	-0.808185389	2.2629088	-8.766260515	2.61E-16	2.10E-15	26.23461114
RXRB	-0.52444782	5.67573077	-8.765933011	2.61E-16	2.10E-15	26.23240579
TTC12	-0.647706063	3.61753442	-8.765543329	2.62E-16	2.11E-15	26.22978181
RFX4	-0.137637078	0.10757869	-8.763475911	2.66E-16	2.13E-15	26.21586156
ERLEC1	-0.645101405	6.81189707	-8.760628762	2.71E-16	2.17E-15	26.19669415
SLC25A1	-0.522896915	4.12863684	-8.760582767	2.71E-16	2.17E-15	26.19638453
L3MBTL4	-0.662174092	1.77299927	-8.758339876	2.75E-16	2.21E-15	26.18128747
MUL1	-0.610826122	5.49588529	-8.756807934	2.78E-16	2.23E-15	26.17097704
KATNA1	-0.47486258	4.56188762	-8.752700291	2.86E-16	2.29E-15	26.14333617
RBIS	-0.480366164	4.82410945	-8.752477547	2.86E-16	2.29E-15	26.1418375
PIGX	-0.411068905	3.86893624	-8.750796523	2.90E-16	2.32E-15	26.13052782
CEBPZO	-0.498690604	5.0427293	-8.750724124	2.90E-16	2.32E-15	26.13004076

HDAC1	-0.61047176	7.11802403	-8.750638523	2.90E-16	2.32E-15	26.12946488
CDNF	-0.68204963	2.61934876	-8.750229354	2.91E-16	2.33E-15	26.12671226
SERP1	-0.554722522	6.68524248	-8.749397467	2.92E-16	2.34E-15	26.12111608
SYT1	1.301572038	1.38704014	8.747905111	2.95E-16	2.36E-15	26.11107759
MLST8	-0.457953273	5.29098055	-8.744798986	3.02E-16	2.41E-15	26.09018684
SPCS3	-0.659016977	5.86365484	-8.743913976	3.04E-16	2.42E-15	26.0842353
CUTC	-0.459841917	4.81557153	-8.74267879	3.06E-16	2.44E-15	26.07592941
FAM53B	-0.645735624	5.47002882	-8.74173022	3.08E-16	2.46E-15	26.06955128
HHLA3	-0.511356545	4.79034161	-8.740739516	3.10E-16	2.47E-15	26.06289024
SYNDIG1	-1.219417033	2.20555197	-8.740111374	3.12E-16	2.48E-15	26.05866711
BZW2	-0.561436888	5.51163601	-8.740041898	3.12E-16	2.48E-15	26.05820002
SGSM3	-0.543147656	5.1616262	-8.737563571	3.17E-16	2.52E-15	26.04153937
SLC49A4	-0.600964603	3.93110608	-8.733951037	3.25E-16	2.58E-15	26.01725853
VPS28	-0.534005034	6.79829102	-8.733287698	3.26E-16	2.60E-15	26.01280064
AMACR	-0.672426333	1.83580619	-8.733047563	3.27E-16	2.60E-15	26.01118689
HSPBP1	-0.487815061	5.93833732	-8.731539065	3.30E-16	2.62E-15	26.00105002
NADK2	-0.540767391	4.00059115	-8.73078027	3.32E-16	2.64E-15	25.9959514
GARNL3	-0.558781052	1.51025494	-8.730566112	3.33E-16	2.64E-15	25.99451243
UNC119B	-0.783063187	5.1801641	-8.730019036	3.34E-16	2.65E-15	25.99083663
CDH5	-0.824898923	5.09518172	-8.728712105	3.37E-16	2.67E-15	25.98205585
NKIRAS1	-0.465022926	3.61011244	-8.728702442	3.37E-16	2.67E-15	25.98199093
MACO1	-0.506910271	5.18787796	-8.727682873	3.39E-16	2.69E-15	25.97514132
NIPSNAP	-0.561829385	5.95693355	-8.727513565	3.39E-16	2.69E-15	25.97400393
KLHL22	-0.504403374	4.61801815	-8.724773686	3.46E-16	2.74E-15	25.95559937
FAM110D	-0.82901384	2.76870463	-8.724356192	3.47E-16	2.74E-15	25.95279521
NSUN4	-0.486358312	3.2401425	-8.722133943	3.52E-16	2.78E-15	25.93787041
ERBB3	1.396225742	4.24487712	8.719436283	3.59E-16	2.83E-15	25.91975545
ITGB8	1.239052518	2.2100364	8.717250847	3.64E-16	2.88E-15	25.90508233
TBL2	-0.570115595	4.87634157	-8.716610298	3.66E-16	2.89E-15	25.90078203
SIAE	-0.789954086	5.50595669	-8.715927811	3.67E-16	2.90E-15	25.89620037
AP3S2	-0.519586462	3.65110477	-8.713156827	3.74E-16	2.95E-15	25.87760024
CLMN	-0.706839408	1.55582263	-8.712201806	3.77E-16	2.97E-15	25.87119043
HIST1H2	-1.204636176	2.77287071	-8.710635089	3.81E-16	3.00E-15	25.86067593
COG8	-0.495713094	3.59253183	-8.710114011	3.82E-16	3.01E-15	25.85717912
LIFR	-1.229907281	3.07018069	-8.707434171	3.89E-16	3.06E-15	25.83919722
SNRNP3	-0.477107024	4.06095747	-8.70380998	3.99E-16	3.14E-15	25.81488344
TSR3	-0.508918562	6.80551858	-8.703151768	4.01E-16	3.15E-15	25.81046825
MIPEP	-0.542378829	4.08659307	-8.70233605	4.03E-16	3.17E-15	25.80499678
CLPB	-0.484825	3.27932757	-8.700942211	4.07E-16	3.20E-15	25.79564817
TRMT5	-0.4296282	3.14066584	-8.700123589	4.09E-16	3.21E-15	25.79015798

MTMR4	-0.696742737	3.79800647	-8.699600498	4.11E-16	3.22E-15	25.78664995
TMED10	-0.666379758	7.7223129	-8.699027302	4.12E-16	3.23E-15	25.78280604
POLR2J	-0.560648671	6.42575805	-8.69894098	4.12E-16	3.24E-15	25.78222716
TMEM10	-0.586937867	3.8788016	-8.698837263	4.13E-16	3.24E-15	25.78153164
GIMAP8	-0.875043853	3.84314554	-8.69707892	4.18E-16	3.27E-15	25.76974098
PSTPIP2	0.699752387	1.87736538	8.692990782	4.29E-16	3.36E-15	25.74233275
SPOCK2	1.87597356	5.93426835	8.692790719	4.30E-16	3.37E-15	25.74099164
PRCC	-0.468503041	5.43559515	-8.691821459	4.33E-16	3.39E-15	25.73449451
FAM98A	-0.527759471	4.62976878	-8.691351854	4.34E-16	3.40E-15	25.7313468
GOLGA8	-0.943367777	1.07504156	-8.690769457	4.36E-16	3.41E-15	25.72744319
PRKCE	-0.609166918	1.77315007	-8.690623732	4.36E-16	3.41E-15	25.72646646
VDAC2	-0.497736362	5.85196963	-8.68877495	4.42E-16	3.45E-15	25.71407574
CCDC25	-0.520184756	5.17921865	-8.688469787	4.43E-16	3.46E-15	25.71203064
WDR92	-0.319965919	1.22882151	-8.687413355	4.46E-16	3.48E-15	25.7049511
PCDH12	-0.892957057	3.80425931	-8.684406649	4.55E-16	3.55E-15	25.68480463
DPM1	-0.534781575	6.18067168	-8.680685144	4.67E-16	3.64E-15	25.65987387
TXNDC1	-0.490951619	6.18836189	-8.680323641	4.68E-16	3.65E-15	25.65745243
PTP4A2	-0.519402561	6.97182044	-8.6800768	4.69E-16	3.65E-15	25.65579906
SLC35B3	-0.624562712	5.14461951	-8.677858201	4.76E-16	3.71E-15	25.64093976
ARL4A	-0.584285884	3.26701578	-8.675518748	4.84E-16	3.76E-15	25.62527326
RFESD	-0.287663819	1.24833777	-8.672905311	4.92E-16	3.83E-15	25.6077747
ALG3	-0.447857565	4.78971219	-8.672728905	4.93E-16	3.83E-15	25.60659365
SERPINA	2.607669499	8.40740909	8.672487688	4.94E-16	3.84E-15	25.60497872
SPSB2	-0.600996111	4.5490479	-8.670297908	5.01E-16	3.89E-15	25.5903194
AIMP1	-0.468430209	5.60067761	-8.670023567	5.02E-16	3.90E-15	25.58848298
SOSTDC	-0.935463325	0.78091347	-8.668594182	5.07E-16	3.93E-15	25.57891532
CCS	-0.592412236	5.59309012	-8.668236836	5.08E-16	3.94E-15	25.57652354
C1orf210	-0.746963913	5.38281779	-8.667514173	5.11E-16	3.96E-15	25.57168679
NPPC	-1.352922198	1.38399257	-8.663868564	5.23E-16	4.06E-15	25.54729025
PPP1R3I	-0.58880714	3.18445964	-8.663848031	5.23E-16	4.06E-15	25.54715286
AKIRIN1	-0.750764817	5.94734745	-8.662806153	5.27E-16	4.08E-15	25.54018162
C4orf47	-0.752391389	2.38376216	-8.661693178	5.31E-16	4.11E-15	25.53273516
RUVBL2	-0.460261313	6.13351462	-8.661214273	5.33E-16	4.12E-15	25.52953116
PRMT7	-0.488279564	3.07624542	-8.656555067	5.50E-16	4.25E-15	25.49836494
ACKR3	-1.062832042	5.17815963	-8.655522294	5.54E-16	4.28E-15	25.49145778
UBA5	-0.498369083	4.16018672	-8.654759522	5.57E-16	4.30E-15	25.48635667
POLR2C	-0.501702429	5.85659787	-8.652940647	5.64E-16	4.35E-15	25.47419376
FGF12	-1.022099608	1.4666658	-8.651701341	5.68E-16	4.39E-15	25.46590726
HOGA1	-0.745843284	0.79462141	-8.650857855	5.72E-16	4.41E-15	25.46026774
PLCH1	-1.006477489	1.57725878	-8.650593683	5.73E-16	4.42E-15	25.45850156

ADAMTS	-1.0164997	2.26836073	-8.647976395	5.83E-16	4.49E-15	25.44100466
ZFP3	-0.620277098	2.94373268	-8.644504882	5.97E-16	4.60E-15	25.41780161
TAF13	-0.572231311	5.53110725	-8.643509562	6.01E-16	4.63E-15	25.41114999
SFXN5	-0.490341656	2.36660035	-8.639739493	6.16E-16	4.75E-15	25.38595879
PMPCA	-0.400984784	4.67586206	-8.638256493	6.23E-16	4.79E-15	25.37605119
ZFPL1	-0.462529716	3.37825482	-8.637449243	6.26E-16	4.82E-15	25.37065852
FCGRT	-0.713096844	6.38736188	-8.636258053	6.31E-16	4.85E-15	25.36270151
ZNF775	-0.560630855	2.9620614	-8.635861705	6.33E-16	4.86E-15	25.36005409
MOB2	-0.479151624	4.62834832	-8.634662647	6.38E-16	4.90E-15	25.35204534
FAM155E	-1.839621417	4.03254213	-8.634189305	6.40E-16	4.91E-15	25.34888396
CDH11	1.392933611	2.18886632	8.63329552	6.44E-16	4.94E-15	25.34291476
PGAP2	-0.546663554	4.61638501	-8.631953865	6.50E-16	4.98E-15	25.33395507
AVPR1A	-1.192056654	1.8837129	-8.63068484	6.55E-16	5.03E-15	25.32548111
C11orf52	-0.199048235	0.2708262	-8.630073829	6.58E-16	5.04E-15	25.32140131
HIST1H2	-0.512470814	1.06706019	-8.625125622	6.81E-16	5.21E-15	25.28836727
DPP6	-1.341303359	1.27854411	-8.623387059	6.89E-16	5.27E-15	25.27676315
KCNK16	-1.352688231	0.96327175	-8.62325617	6.89E-16	5.28E-15	25.27588958
CEACAM	1.901250829	2.08311041	8.619998431	7.05E-16	5.39E-15	25.25414929
MET	1.428032546	6.96089416	8.619206738	7.08E-16	5.42E-15	25.24886666
ZNRF1	-0.510000777	4.24047099	-8.619138677	7.09E-16	5.42E-15	25.24841253
SMARCA	-0.775684554	6.08418885	-8.617364204	7.17E-16	5.48E-15	25.23657325
DLG2	-0.926869599	1.24512432	-8.616895743	7.20E-16	5.49E-15	25.23344791
GINM1	-0.588993989	7.16756882	-8.615411825	7.27E-16	5.55E-15	25.22354853
NT5M	-0.628549002	2.17565708	-8.615010073	7.29E-16	5.56E-15	25.22086855
EMID1	-1.557529873	3.67906432	-8.613318077	7.37E-16	5.62E-15	25.2095825
MID1	-0.651074882	2.03376296	-8.611761206	7.45E-16	5.68E-15	25.19919883
NUS1	-0.597186386	4.62028595	-8.611302252	7.47E-16	5.69E-15	25.196138
HIST1H2	-0.784626056	1.31723021	-8.610315455	7.52E-16	5.73E-15	25.18955722
NUBP1	-0.43454891	5.17386087	-8.608534571	7.61E-16	5.80E-15	25.17768184
ROR1	0.837211483	1.17148061	8.608075014	7.64E-16	5.81E-15	25.17461761
NDUFS8	-0.473316052	6.05760392	-8.606209166	7.73E-16	5.88E-15	25.16217749
CHADL	-0.706839651	2.98533369	-8.606017488	7.74E-16	5.89E-15	25.1608996
DDT	-0.580083048	5.32158424	-8.604238063	7.84E-16	5.96E-15	25.1490372
FOXP2	-0.775275922	1.02432705	-8.603686422	7.87E-16	5.98E-15	25.14536001
FBXW9	-0.579920375	4.15501271	-8.60335653	7.89E-16	5.99E-15	25.14316104
TFG	-0.504316714	5.9265463	-8.602446891	7.93E-16	6.02E-15	25.13709786
MLX	-0.465834442	5.05605922	-8.599907979	8.07E-16	6.12E-15	25.12017667
AIG1	-0.47774412	4.91015151	-8.598942594	8.12E-16	6.16E-15	25.11374335
DUS4L	-0.424776332	2.36923848	-8.597593262	8.20E-16	6.21E-15	25.10475208
EBI3	1.244578936	2.94570875	8.596793418	8.24E-16	6.24E-15	25.09942269

SCML2	-0.52286327	1.13911372	-8.596065243	8.28E-16	6.27E-15	25.09457106
THAP3	-0.509280659	3.91435647	-8.595263131	8.33E-16	6.30E-15	25.08922707
ISCU	-0.504947372	7.24123538	-8.594902733	8.35E-16	6.32E-15	25.08682605
ERICH6	-0.194566258	0.33807937	-8.592732383	8.47E-16	6.41E-15	25.07236804
REEP5	-0.529336093	8.19687607	-8.592200374	8.50E-16	6.43E-15	25.06882431
CCDC40	-0.544761527	1.71085682	-8.590888249	8.58E-16	6.48E-15	25.06008473
CALM3	-0.547052548	7.84418088	-8.588826649	8.70E-16	6.57E-15	25.04635465
SLC25A4	-0.664801868	3.02030375	-8.588605552	8.71E-16	6.58E-15	25.04488228
YBX1	-0.486937353	8.70339034	-8.586861777	8.82E-16	6.65E-15	25.03327046
PEAR1	-0.842284702	2.42945278	-8.586126257	8.86E-16	6.68E-15	25.02837302
SF3B6	-0.487857574	7.21509123	-8.5859724	8.87E-16	6.69E-15	25.02734859
RNF5	-0.458773932	7.0159595	-8.585260382	8.91E-16	6.71E-15	25.02260791
PLAUR	1.372760939	3.49340909	8.583110207	9.04E-16	6.81E-15	25.00829313
USP16	-0.588543744	4.5818894	-8.582031223	9.11E-16	6.86E-15	25.00111055
DIPK2B	-0.749815428	3.53179169	-8.581037237	9.17E-16	6.90E-15	24.99449422
FBXO25	-0.463481797	3.74751923	-8.580891738	9.18E-16	6.90E-15	24.99352576
NICN1	-0.524924475	5.00446455	-8.580329709	9.21E-16	6.93E-15	24.98978492
ELF3	1.39051922	3.74059188	8.580071513	9.23E-16	6.94E-15	24.98806642
SYPL1	-0.655773854	7.11379538	-8.578028571	9.36E-16	7.03E-15	24.97447004
HIST1H3	-0.900388411	1.65445399	-8.577932965	9.36E-16	7.03E-15	24.97383379
NR1H3	-0.568520812	3.50587852	-8.5772186	9.41E-16	7.06E-15	24.96907993
CCL20	1.300744071	1.54281972	8.575207184	9.54E-16	7.15E-15	24.95569581
MED29	-0.48940919	6.54812882	-8.574121239	9.61E-16	7.20E-15	24.94847056
URM1	-0.420393591	5.34967806	-8.573486117	9.65E-16	7.23E-15	24.94424506
WDR78	-0.521229358	2.3467153	-8.573376287	9.66E-16	7.23E-15	24.94351437
EFCAB1:	-0.535549872	0.90744146	-8.570493851	9.85E-16	7.37E-15	24.92433968
RCC1L	-0.410697794	5.63869979	-8.569196076	9.93E-16	7.43E-15	24.91570772
PEX7	-0.516656034	4.45245354	-8.569083546	9.94E-16	7.43E-15	24.91495928
TAF8	-0.488304805	3.62469062	-8.569051083	9.94E-16	7.43E-15	24.91474336
MYL6B	-0.563973287	5.88901849	-8.568953942	9.95E-16	7.44E-15	24.91409728
VAPB	-0.653042273	4.44063444	-8.567927919	1.00E-15	7.48E-15	24.90727345
KRBOX4	-0.479185941	3.0902315	-8.565930677	1.02E-15	7.58E-15	24.89399158
GBP2	1.359847076	4.7164879	8.565215145	1.02E-15	7.62E-15	24.88923363
ISCA2	-0.497893449	5.4192246	-8.563878768	1.03E-15	7.68E-15	24.88034795
IPCEF1	-1.453819265	2.29313528	-8.562614149	1.04E-15	7.74E-15	24.8719401
AURKAIF	-0.553530916	7.06288194	-8.562014754	1.04E-15	7.77E-15	24.86795524
SYAP1	-0.467678752	5.04876119	-8.561800916	1.04E-15	7.78E-15	24.86653366
CALCOC	-0.551710558	5.07365725	-8.559544777	1.06E-15	7.90E-15	24.85153615
PTPRU	1.205458812	4.12335271	8.558063433	1.07E-15	7.97E-15	24.84169022
MEA1	-0.421384019	7.59967392	-8.556505573	1.08E-15	8.05E-15	24.83133674

GPAA1	-0.615086306	6.93078576	-8.555156398	1.09E-15	8.12E-15	24.82237101
UNC5CL	1.11106293	2.4529338	8.554501705	1.10E-15	8.15E-15	24.81802063
KIF17	-0.51343456	1.49179847	-8.554157525	1.10E-15	8.17E-15	24.81573366
TIMP4	-0.77936904	1.0804376	-8.553842604	1.10E-15	8.18E-15	24.81364115
SMAD6	-0.742803634	3.42835734	-8.553379161	1.11E-15	8.21E-15	24.81056185
TIMM10E	-0.503640702	5.39105832	-8.547350987	1.15E-15	8.54E-15	24.77051673
EIF2D	-0.4101501	4.68608091	-8.546007554	1.16E-15	8.62E-15	24.76159445
STMN3	-0.836429447	6.71603217	-8.545860152	1.16E-15	8.62E-15	24.76061554
DTNB	-0.562377482	4.0474043	-8.545433457	1.17E-15	8.64E-15	24.75778188
MSANTD	-0.511152739	4.11546698	-8.544491709	1.17E-15	8.69E-15	24.75152804
ANKRD1	-0.392903446	2.96397265	-8.543799051	1.18E-15	8.73E-15	24.74692857
NAP1L2	-0.790060373	3.83631498	-8.543712537	1.18E-15	8.73E-15	24.7463541
PLA2R1	-1.247818172	2.832586	-8.542953145	1.19E-15	8.77E-15	24.74131177
ARSG	-0.613574442	2.70398306	-8.542118669	1.19E-15	8.82E-15	24.73577116
MVB12A	-0.460397676	5.23813184	-8.540767785	1.20E-15	8.89E-15	24.72680244
CREM	-0.536789689	3.41817526	-8.54068679	1.20E-15	8.90E-15	24.72626473
CYB5B	-0.398002901	4.915392	-8.540190667	1.21E-15	8.92E-15	24.72297111
ENDOV	-0.478578136	3.31450177	-8.540115237	1.21E-15	8.92E-15	24.72247035
NHP2	-0.523039354	6.39418639	-8.539262175	1.22E-15	8.97E-15	24.71680739
LRRC23	-0.531023327	3.89704547	-8.539223118	1.22E-15	8.97E-15	24.71654813
PEX11B	-0.488887084	6.1438367	-8.539122231	1.22E-15	8.97E-15	24.71587842
ACAD9	-0.397883271	5.07262424	-8.538536135	1.22E-15	9.00E-15	24.7119879
SDCCAG	-0.559001108	4.73366526	-8.535331552	1.25E-15	9.19E-15	24.69071842
THTPA	-0.682259047	3.22037663	-8.533470586	1.26E-15	9.31E-15	24.67836884
RBM4B	-0.506260331	3.66338645	-8.533396523	1.26E-15	9.31E-15	24.67787738
CMAS	-0.482009432	5.57610449	-8.532027774	1.28E-15	9.39E-15	24.6687952
ZCWPW	-0.541770383	3.24424152	-8.52822059	1.31E-15	9.63E-15	24.64353732
GNRH2	-0.507536921	0.6611189	-8.52703765	1.32E-15	9.70E-15	24.63569065
ALYREF	-0.573378406	6.21479531	-8.526567702	1.32E-15	9.73E-15	24.63257356
FZD9	-0.305416392	0.35827046	-8.526045205	1.33E-15	9.76E-15	24.62910804
RER1	-0.394888393	5.60842464	-8.52598509	1.33E-15	9.76E-15	24.62870933
CCT3	-0.468723198	7.22640063	-8.525491967	1.33E-15	9.79E-15	24.62543875
NME9	-0.43949612	0.98062262	-8.518321134	1.40E-15	1.03E-14	24.577891
RALY	-0.43347723	6.51470744	-8.517375211	1.41E-15	1.03E-14	24.57162051
ZNF658	-0.42926583	1.25847953	-8.516915421	1.41E-15	1.04E-14	24.56857272
BACH2	0.589802334	1.13498736	8.516121597	1.42E-15	1.04E-14	24.56331095
BMP7	-0.788462106	6.30713187	-8.513241887	1.45E-15	1.06E-14	24.54422542
ADCY7	0.788801163	1.25898807	8.511885705	1.46E-15	1.07E-14	24.53523845
ALDH3B	1.23232798	3.79789018	8.511066854	1.47E-15	1.08E-14	24.52981258
COIL	-0.511231149	4.40821625	-8.508834918	1.49E-15	1.09E-14	24.5150248

ELAC2	-0.421006075	5.05872612	-8.508430502	1.50E-15	1.09E-14	24.51234556
C2CD4A	1.560759921	1.84669397	8.506279416	1.52E-15	1.11E-14	24.49809588
TECTB	-0.516470114	0.37304059	-8.504091938	1.54E-15	1.12E-14	24.48360719
MTLN	-0.739316213	4.55386705	-8.503046418	1.55E-15	1.13E-14	24.47668295
FIBP	-0.461503384	5.93115511	-8.502652303	1.55E-15	1.13E-14	24.47407294
LCN2	2.391320152	4.64147571	8.50221363	1.56E-15	1.14E-14	24.47116793
BLCAP	-0.406222016	5.13122867	-8.50041951	1.58E-15	1.15E-14	24.45928764
TMEM16	-0.555641282	5.43973363	-8.498955652	1.59E-15	1.16E-14	24.44959531
CACNG4	1.506054753	1.7295845	8.496316087	1.62E-15	1.18E-14	24.43212089
NUDT6	-0.397848922	1.32336806	-8.496069341	1.63E-15	1.18E-14	24.43048754
MAPK8	-0.539987515	3.58620441	-8.496042262	1.63E-15	1.18E-14	24.43030829
MRPL19	-0.545441966	4.24788826	-8.495749008	1.63E-15	1.18E-14	24.42836711
ST3GAL(	-0.903196378	2.24992008	-8.495562129	1.63E-15	1.19E-14	24.4271301
FAM216/	-0.456572812	2.22060719	-8.495504656	1.63E-15	1.19E-14	24.42674967
ACOT11	-0.768595921	2.16016184	-8.495125044	1.64E-15	1.19E-14	24.42423694
AR	-1.010909643	1.57798593	-8.49281011	1.66E-15	1.21E-14	24.40891534
DRC3	-0.698374945	2.78022924	-8.492136147	1.67E-15	1.21E-14	24.40445508
RRNAD1	-0.505134932	5.32406611	-8.491642598	1.67E-15	1.22E-14	24.40118893
NEXN	-0.890401778	2.32112432	-8.488552133	1.71E-15	1.24E-14	24.3807396
CA5A	-0.162923338	0.17272021	-8.486124288	1.74E-15	1.26E-14	24.36467768
GRB10	-0.83040754	4.21266706	-8.483560873	1.77E-15	1.28E-14	24.34772165
WNT10A	1.580579387	2.08071345	8.483038836	1.77E-15	1.29E-14	24.34426892
PPIH	-0.431229511	5.35322376	-8.48198083	1.79E-15	1.29E-14	24.33727169
BORCS6	-0.474192737	3.8346965	-8.481647723	1.79E-15	1.30E-14	24.33506875
FAM167E	-0.833210601	5.12483211	-8.481645039	1.79E-15	1.30E-14	24.335051
PSMB1	-0.439840101	7.62006867	-8.480926484	1.80E-15	1.30E-14	24.33029914
SLC35G2	-0.565764121	2.50651637	-8.477577559	1.84E-15	1.33E-14	24.30815543
ITM2B	-0.544488096	7.95075982	-8.475970246	1.86E-15	1.34E-14	24.29752932
MAPRE2	-0.689502939	4.14840732	-8.47437307	1.88E-15	1.36E-14	24.28697134
C7orf26	-0.467193378	4.89932174	-8.472900061	1.90E-15	1.37E-14	24.27723514
DNAAF3	-0.825887056	1.67524118	-8.470849161	1.92E-15	1.39E-14	24.26368081
EIF5A	-0.368930532	8.36259891	-8.470048934	1.94E-15	1.40E-14	24.25839264
CDC37	-0.450707179	7.26318424	-8.468831036	1.95E-15	1.41E-14	24.25034489
CAMTA1	-0.423720288	3.66590048	-8.467767128	1.97E-15	1.42E-14	24.24331522
KDM8	-0.387288864	2.56874902	-8.4659175	1.99E-15	1.43E-14	24.23109516
ARHGEF	1.187056114	1.71440453	8.465826213	1.99E-15	1.43E-14	24.23049209
ADAM22	-0.676219826	1.23400884	-8.465710599	1.99E-15	1.43E-14	24.22972831
PRMT9	-0.616843181	3.64261656	-8.465202884	2.00E-15	1.44E-14	24.22637427
MCUR1	-0.542928155	4.77913178	-8.464107297	2.01E-15	1.45E-14	24.21913703
FBXO44	-0.486167972	5.10980358	-8.463495693	2.02E-15	1.45E-14	24.21509712

LMNTD2	-0.69058873	1.84070626	-8.459776391	2.07E-15	1.49E-14	24.19053304
AGL	-0.656486886	3.53302581	-8.458064567	2.10E-15	1.51E-14	24.17922935
PRKAG2	-0.466790357	3.48093388	-8.45748089	2.11E-15	1.51E-14	24.17537545
HIST2H2	-1.006789044	5.03758258	-8.457171947	2.11E-15	1.51E-14	24.17333563
APEH	-0.541671478	6.48678412	-8.456104496	2.12E-15	1.52E-14	24.166288
TRAF2	-0.454457037	4.37318084	-8.450327558	2.21E-15	1.58E-14	24.1281556
OXLD1	-0.575938004	5.21317082	-8.449400865	2.22E-15	1.59E-14	24.12204005
SLC27A6	2.502953265	3.87994355	8.448545925	2.24E-15	1.60E-14	24.11639836
BEND5	-0.673168238	3.45217875	-8.444790796	2.29E-15	1.64E-14	24.0916223
STAMBP	-0.4550643	4.03631865	-8.442833903	2.32E-15	1.66E-14	24.07871332
CLBA1	-0.608271391	4.07050656	-8.441818216	2.34E-15	1.67E-14	24.07201383
FBXO4	-0.495472646	3.85128372	-8.440608683	2.36E-15	1.69E-14	24.06403633
DNAJC3	-0.808093673	5.96882568	-8.439300474	2.38E-15	1.70E-14	24.05540872
DDX49	-0.439159685	5.54839487	-8.437652457	2.40E-15	1.72E-14	24.04454116
UCK1	-0.455087858	5.74562248	-8.436210067	2.43E-15	1.73E-14	24.03503055
TMEM9	-0.59299329	6.61379585	-8.435729992	2.44E-15	1.74E-14	24.03186531
GNMT	-0.717569253	1.57291065	-8.435423958	2.44E-15	1.74E-14	24.02984761
MTFR1	-0.561479117	3.10288841	-8.434413129	2.46E-15	1.75E-14	24.02318346
ZMAT2	-0.529091229	7.18971328	-8.433886556	2.47E-15	1.76E-14	24.01971207
PSENEN	-0.529977279	6.40655664	-8.432519243	2.49E-15	1.77E-14	24.01069873
PELO	-0.568859298	4.16290317	-8.432130981	2.49E-15	1.78E-14	24.00813946
OSBPL1A	-0.686553298	3.57666298	-8.430531982	2.52E-15	1.80E-14	23.99760017
BBOF1	-0.636135881	3.33733564	-8.430107517	2.53E-15	1.80E-14	23.99480264
NGRN	-0.659853286	4.3124599	-8.429650338	2.54E-15	1.81E-14	23.99178957
IFI27L1	-0.496951756	3.22340397	-8.426769863	2.59E-15	1.84E-14	23.97280781
ELMOD2	-0.498299432	3.94337128	-8.426619182	2.59E-15	1.84E-14	23.97181495
ZBTB22	-0.483655881	5.69166003	-8.424155863	2.63E-15	1.87E-14	23.95558522
ICAM1	1.440901937	5.64806075	8.423662223	2.64E-15	1.88E-14	23.95233317
CAMK2G	-0.489605215	3.62795448	-8.423414786	2.64E-15	1.88E-14	23.95070311
UBTF	-0.484955147	6.2712044	-8.423389687	2.64E-15	1.88E-14	23.95053777
STIP1	-0.490035253	6.1281303	-8.422839051	2.65E-15	1.88E-14	23.94691042
SERPINI	-0.600096527	3.36308322	-8.42195827	2.67E-15	1.89E-14	23.9411085
MRPL4	-0.490535502	4.98292993	-8.420870896	2.69E-15	1.91E-14	23.93394618
ZNF536	-0.708273141	0.54018804	-8.419612408	2.71E-15	1.92E-14	23.92565741
ARPC1A	-0.404888066	7.21132277	-8.417087695	2.76E-15	1.95E-14	23.90903103
EXOSC1	-0.327881921	4.56282418	-8.416955172	2.76E-15	1.96E-14	23.90815838
SNRPD3	-0.45075722	6.60602304	-8.416479451	2.77E-15	1.96E-14	23.90502588
ATP6V1E	-0.470762336	2.02842311	-8.415988019	2.78E-15	1.97E-14	23.90179003
TUBA8	-0.190108474	0.23918498	-8.414607417	2.80E-15	1.98E-14	23.8927
FAM117A	-0.61966775	4.5324534	-8.412383576	2.85E-15	2.01E-14	23.87805977

GABRB2	1.951939606	3.55672845	8.411866661	2.86E-15	2.02E-14	23.87465707
XK	-0.724594492	1.3051963	-8.411171153	2.87E-15	2.03E-14	23.87007893
SOX12	-0.582462024	4.32336523	-8.407093332	2.95E-15	2.08E-14	23.84324123
ALDH1B1	-0.876190348	4.52366124	-8.40664852	2.96E-15	2.09E-14	23.8403142
AJM1	-0.61896795	1.95461981	-8.406128847	2.97E-15	2.09E-14	23.83689466
INTS13	-0.45245274	4.08317603	-8.406030362	2.97E-15	2.09E-14	23.83624663
AP3M2	-0.497693397	3.42556352	-8.404923674	2.99E-15	2.11E-14	23.82896488
MED10	-0.47577644	5.05853136	-8.402994529	3.03E-15	2.14E-14	23.81627285
DUSP12	-0.530359219	4.37850634	-8.40221549	3.05E-15	2.15E-14	23.81114795
SUCLG2	-0.553132626	6.05656708	-8.399417563	3.10E-15	2.19E-14	23.79274402
AQP11	-0.476358291	1.93828965	-8.392116284	3.26E-15	2.29E-14	23.74473473
RBM20	-0.57388982	0.79796561	-8.391236573	3.28E-15	2.31E-14	23.73895182
PTPN18	-0.419134059	4.91177827	-8.390604763	3.29E-15	2.32E-14	23.73479874
TMEM10	-0.598515954	6.99087876	-8.389766415	3.31E-15	2.33E-14	23.72928828
ZRANB3	-0.388950598	1.46890936	-8.389396318	3.32E-15	2.33E-14	23.72685574
B3GAT3	-0.518948812	5.83276794	-8.389110587	3.33E-15	2.34E-14	23.72497775
DTYMK	-0.496522161	4.39236406	-8.387171943	3.37E-15	2.36E-14	23.71223683
INHBC	-0.488235865	0.50379434	-8.384578198	3.43E-15	2.41E-14	23.69519315
FUT3	0.812918522	0.84356187	8.384131799	3.44E-15	2.41E-14	23.69226013
TIFA	-0.634318458	3.91225889	-8.380607407	3.52E-15	2.47E-14	23.66910658
HES2	0.5866207	0.71487028	8.380418539	3.52E-15	2.47E-14	23.66786596
PSMB3	-0.462381318	8.00350646	-8.375638174	3.64E-15	2.55E-14	23.63647053
LRRN2	-1.128347569	2.42605112	-8.372652753	3.71E-15	2.60E-14	23.61686868
LUC7L2	-0.501992847	4.7627526	-8.371978608	3.73E-15	2.61E-14	23.61244289
MTA3	-0.417507176	3.31673867	-8.371506881	3.74E-15	2.62E-14	23.60934611
RANBP1	-0.628400375	3.25851624	-8.3702271	3.77E-15	2.64E-14	23.60094512
MMRN2	-0.795786889	4.73336863	-8.369209206	3.80E-15	2.65E-14	23.59426378
KCTD6	-0.488199175	3.29380264	-8.369156825	3.80E-15	2.65E-14	23.59391998
AAAS	-0.419084901	4.8195966	-8.368794058	3.81E-15	2.66E-14	23.59153894
DRG1	-0.465097482	6.17837544	-8.368559662	3.81E-15	2.66E-14	23.59000049
TTI2	-0.425656203	2.86477537	-8.366992872	3.85E-15	2.69E-14	23.57971763
RCBTB2	-0.614054544	4.95975341	-8.365904694	3.88E-15	2.71E-14	23.57257655
PPP2R5I	-0.449011855	5.24636298	-8.365387954	3.90E-15	2.72E-14	23.56918566
MAGED1	-0.675182378	6.89172051	-8.364952344	3.91E-15	2.72E-14	23.56632725
SPECC1	-0.486963543	4.2273914	-8.363163455	3.95E-15	2.75E-14	23.55458971
SEC22A	-0.461913829	3.47141671	-8.361719257	3.99E-15	2.78E-14	23.54511485
TACSTD	2.838594727	6.89952483	8.361534725	4.00E-15	2.78E-14	23.54390427
DHRS13	-0.535157082	1.95225856	-8.360414073	4.03E-15	2.80E-14	23.53655281
SYT12	2.154496384	2.86921599	8.358768521	4.07E-15	2.83E-14	23.52575904
ALKBH2	-0.453701384	4.01683429	-8.357863539	4.10E-15	2.85E-14	23.51982345

MAP2K5	-0.536429977	3.63988788	-8.355839382	4.15E-15	2.89E-14	23.50654874
CCDC36	-0.256541233	0.44785704	-8.355685674	4.16E-15	2.89E-14	23.50554078
ZNF717	-0.399742466	2.09513286	-8.35337947	4.22E-15	2.93E-14	23.49041876
SMPD2	-0.441000512	4.4317484	-8.352564065	4.24E-15	2.95E-14	23.48507264
EPM2A	-0.448813227	2.07936591	-8.352384903	4.25E-15	2.95E-14	23.48389802
POPDC2	-0.644691601	2.62611681	-8.351703032	4.27E-15	2.96E-14	23.47942768
SETD3	-0.496722703	5.84144472	-8.350472829	4.30E-15	2.98E-14	23.471363
MAP4K2	-0.498063356	3.26380727	-8.34900412	4.34E-15	3.01E-14	23.46173568
ST7L	-0.39460718	2.38701845	-8.348623913	4.36E-15	3.02E-14	23.45924359
STARD1:	-0.852060505	3.57538268	-8.347422414	4.39E-15	3.04E-14	23.45136874
MRPS16	-0.398527392	6.0352581	-8.34701375	4.40E-15	3.05E-14	23.44869042
RND2	-1.173264251	3.84366929	-8.345043871	4.46E-15	3.09E-14	23.43578122
CLN3	-0.374782284	2.87233386	-8.344953267	4.46E-15	3.09E-14	23.43518751
CCL22	1.224572535	1.66171491	8.344588472	4.47E-15	3.09E-14	23.4327971
PYURF	-0.558457036	2.86291134	-8.34305896	4.52E-15	3.12E-14	23.42277526
HYLS1	-0.387410295	2.0055275	-8.34269501	4.53E-15	3.13E-14	23.4203907
ATG4B	-0.496242566	4.5734755	-8.339758164	4.62E-15	3.19E-14	23.40115099
NELFE	-0.395601153	6.29157391	-8.336318117	4.73E-15	3.26E-14	23.37861965
PDE2A	-0.866375108	2.84342763	-8.335413819	4.76E-15	3.28E-14	23.37269763
CYYR1	-0.808071953	4.32851347	-8.335080949	4.77E-15	3.29E-14	23.37051785
TARDBP	-0.424622502	5.6929776	-8.334106011	4.80E-15	3.31E-14	23.36413379
MTFMT	-0.414170232	3.26162063	-8.33247782	4.85E-15	3.34E-14	23.35347306
DMTN	-0.552693205	5.49279299	-8.331534705	4.88E-15	3.36E-14	23.34729848
MAGIX	-0.542033245	2.68259385	-8.328748393	4.97E-15	3.42E-14	23.32905879
CCDC24	-0.633592365	4.22048532	-8.327751347	5.00E-15	3.44E-14	23.3225328
NAT9	-0.488721616	4.73542216	-8.32753436	5.01E-15	3.45E-14	23.32111261
WDR83	-0.52563821	3.65297729	-8.326875986	5.03E-15	3.46E-14	23.31680365
CBWD2	-0.479771889	2.49870115	-8.325590078	5.08E-15	3.49E-14	23.30838813
SMIM10	-0.742540908	3.03396306	-8.325425054	5.08E-15	3.49E-14	23.3073082
SCP2	-0.576554649	5.50261094	-8.325423983	5.08E-15	3.49E-14	23.30730119
SH3BGR	-0.535083255	7.37836112	-8.323708774	5.14E-15	3.53E-14	23.29607743
FAM3D	-0.749222195	1.45397783	-8.323023544	5.16E-15	3.54E-14	23.29159388
POLI	-0.685345379	3.28157522	-8.322216769	5.19E-15	3.56E-14	23.28631532
EHHADH	-0.604760859	3.07434199	-8.321651519	5.21E-15	3.57E-14	23.28261718
PAX8	-0.781317928	8.4577054	-8.320098236	5.26E-15	3.61E-14	23.27245559
LPGAT1	-0.674877861	4.80380579	-8.319483965	5.29E-15	3.62E-14	23.26843732
FAM98C	-0.478690877	3.75237855	-8.315833779	5.42E-15	3.71E-14	23.24456306
KDELR2	-0.531531816	7.38003434	-8.31505394	5.44E-15	3.73E-14	23.23946326
HKDC1	-1.202372408	2.23896065	-8.314665462	5.46E-15	3.74E-14	23.23692288
MKRN2	-0.534800497	5.14571494	-8.314519554	5.46E-15	3.74E-14	23.23596876

ZCWPW:	-0.415310677	1.4833715	-8.313420752	5.50E-15	3.76E-14	23.2287838
NIPSNAF	-0.527468878	4.585998	-8.311324069	5.58E-15	3.81E-14	23.21507531
DNAH7	-0.382733871	0.7733846	-8.31127135	5.58E-15	3.81E-14	23.21473066
RPP40	-0.419143729	2.55211873	-8.310476598	5.61E-15	3.83E-14	23.20953496
HPSE	0.883425767	1.90898108	8.310452241	5.61E-15	3.83E-14	23.20937573
PLCD3	1.499157512	5.18664697	8.310101084	5.63E-15	3.84E-14	23.20708014
NOVA2	-0.591466449	1.7724753	-8.310086221	5.63E-15	3.84E-14	23.20698297
NABP1	0.676478475	1.28598346	8.309846958	5.64E-15	3.84E-14	23.20541889
CNTN5	-0.630143073	0.57290817	-8.303260303	5.89E-15	4.01E-14	23.16237145
MRPL3	-0.49335791	6.35328716	-8.301531578	5.96E-15	4.06E-14	23.15107651
ACSM5	-0.813377133	1.73808238	-8.296505602	6.16E-15	4.19E-14	23.11824607
SH3BP5I	-0.510723492	4.03954307	-8.29489267	6.22E-15	4.24E-14	23.10771257
ARHGEF	-0.780552065	3.62118446	-8.290597185	6.40E-15	4.36E-14	23.07966599
EVA1A	1.530026168	3.5835354	8.290295275	6.42E-15	4.36E-14	23.07769503
AVPR2	-0.708568008	1.50443672	-8.290252237	6.42E-15	4.36E-14	23.07741408
LARS2	-0.602091988	3.6469961	-8.287958921	6.52E-15	4.43E-14	23.06244407
GDPGP1	-0.36943275	2.12512564	-8.287167354	6.55E-15	4.45E-14	23.05727753
ASPSCR	-0.471762583	3.39778555	-8.287137873	6.55E-15	4.45E-14	23.05708512
EML2	-0.530685756	4.77591026	-8.286846237	6.56E-15	4.46E-14	23.05518169
FITM2	-0.759624898	3.71780179	-8.284997418	6.65E-15	4.51E-14	23.04311588
UBE2N	-0.426305414	4.80721141	-8.280814934	6.83E-15	4.63E-14	23.01582575
EIF3J	-0.499321708	5.54702147	-8.279358196	6.90E-15	4.68E-14	23.0063226
TAL1	-0.656395144	1.62106504	-8.278826371	6.92E-15	4.69E-14	23.00285343
ZDHHC1	-0.493619825	1.5400447	-8.27880334	6.92E-15	4.69E-14	23.00270321
ENPP3	-1.31884473	1.23856275	-8.27821887	6.95E-15	4.71E-14	22.99889078
USF2	-0.435097352	6.43016858	-8.277759623	6.97E-15	4.72E-14	22.99589528
DBI	-0.652922846	7.23730325	-8.277026638	7.01E-15	4.74E-14	22.99111449
GDPD1	-0.634865721	2.38662943	-8.275868974	7.06E-15	4.78E-14	22.98356429
TIMM9	-0.406825805	5.50473559	-8.272994205	7.19E-15	4.87E-14	22.96481787
GCNT2	-0.627729467	1.52736928	-8.272509201	7.22E-15	4.88E-14	22.96165552
CEBPA	-1.046633054	3.57420426	-8.272318166	7.23E-15	4.88E-14	22.96040995
TRAPPC	-0.418687005	2.89583783	-8.269854201	7.35E-15	4.96E-14	22.94434614
TSSC4	-0.485029891	5.4868692	-8.265131932	7.58E-15	5.12E-14	22.91356703
GRPEL2	-0.402837096	3.30524438	-8.264449479	7.61E-15	5.14E-14	22.90911973
DNAJB2	-0.508422034	6.44863587	-8.263300722	7.67E-15	5.18E-14	22.90163418
PPP1CA	-0.467807717	7.6218579	-8.260972713	7.79E-15	5.25E-14	22.8864662
TADA2A	-0.464550983	3.57248272	-8.260587945	7.81E-15	5.27E-14	22.88395951
RND1	-0.955801222	2.70211021	-8.260338003	7.82E-15	5.27E-14	22.88233122
RBBP7	-0.461848794	5.56011304	-8.260185509	7.83E-15	5.28E-14	22.88133779
CCDC30	-0.444875518	1.5535598	-8.259787237	7.85E-15	5.29E-14	22.87874327

PGBD4	-0.308859877	1.09521659	-8.259266598	7.88E-15	5.30E-14	22.8753517
GLMN	-0.479133357	3.10278315	-8.258768233	7.91E-15	5.32E-14	22.87210534
KIN	-0.417153009	2.85579964	-8.257273379	7.98E-15	5.37E-14	22.86236853
TXNRD3	-0.473063416	2.93944716	-8.25643147	8.03E-15	5.40E-14	22.85688516
PIAS2	-0.442346882	2.65994274	-8.254256922	8.14E-15	5.47E-14	22.84272378
SKIDA1	-0.732006993	1.85101717	-8.252573674	8.24E-15	5.53E-14	22.83176338
SURF6	-0.486128572	4.96932017	-8.248373431	8.47E-15	5.69E-14	22.80441931
TRIM58	-1.296379434	2.86143879	-8.247732596	8.50E-15	5.71E-14	22.80024811
PIN4	-0.483790733	4.38663115	-8.247197727	8.53E-15	5.72E-14	22.79676679
PKHD1L1	-1.955719311	2.19636957	-8.247186269	8.53E-15	5.72E-14	22.79669221
MPHOSPH	-0.439512698	3.79720246	-8.246513531	8.57E-15	5.75E-14	22.79231372
CCDC59	-0.410600804	4.21203839	-8.244895417	8.66E-15	5.81E-14	22.78178314
ABCC8	-1.408351022	1.58162321	-8.244601696	8.68E-15	5.82E-14	22.77987174
TMEM38	-0.875161471	4.31920609	-8.243105693	8.77E-15	5.87E-14	22.77013711
DAP	-0.572811709	7.79516874	-8.241793174	8.84E-15	5.92E-14	22.76159727
NES	-0.966222217	4.8922461	-8.241657147	8.85E-15	5.92E-14	22.76071226
PPP2R5A	-0.538850731	5.91921644	-8.241113107	8.88E-15	5.94E-14	22.75717275
TECTA	-0.339752052	0.78906534	-8.240723786	8.91E-15	5.95E-14	22.75463993
TSNAXIP	-0.480841521	0.6551535	-8.240122276	8.94E-15	5.98E-14	22.75072679
TMEM17	-0.507017289	6.74447616	-8.239596408	8.97E-15	5.99E-14	22.74730588
MAPK4	-1.143140208	1.19198743	-8.239395606	8.99E-15	6.00E-14	22.74599964
CCDC11	-0.467670784	5.60924147	-8.238540682	9.04E-15	6.03E-14	22.74043848
APMAP	-0.691158508	8.66216974	-8.237404525	9.10E-15	6.07E-14	22.73304845
CFAP43	-0.591694227	1.2949659	-8.236285686	9.17E-15	6.12E-14	22.72577164
SACM1L	-0.578756006	4.72571463	-8.235846147	9.20E-15	6.13E-14	22.72291309
GCK	-0.614636756	0.54888609	-8.234063693	9.31E-15	6.20E-14	22.71132174
TCEA3	-0.583175659	6.0839973	-8.232146091	9.43E-15	6.28E-14	22.69885315
CCDC12	-0.468934854	6.644089	-8.232085897	9.43E-15	6.28E-14	22.69846178
CDC14B	-0.688298364	3.21347314	-8.231324867	9.48E-15	6.31E-14	22.69351391
NDUFA7	-0.556965625	3.00426908	-8.230600791	9.52E-15	6.34E-14	22.68880655
EDNRB	-1.262463443	4.53507904	-8.230283197	9.54E-15	6.35E-14	22.68674189
NDUFA1	-0.658400592	5.66225245	-8.227466122	9.72E-15	6.46E-14	22.66843021
PGGT1B	-0.466176746	2.89349915	-8.223119306	1.00E-14	6.65E-14	22.64018199
GADD45	-1.072777871	4.11615296	-8.222951802	1.00E-14	6.65E-14	22.63909361
TRIM39	-0.428091383	3.88886485	-8.222619964	1.00E-14	6.67E-14	22.63693751
FBXL22	-0.538016808	1.60645417	-8.222368938	1.01E-14	6.67E-14	22.63530652
NSF	-0.509187962	4.58979545	-8.221378149	1.01E-14	6.72E-14	22.62886933
CCDC15	-0.448080482	4.22365468	-8.220453311	1.02E-14	6.75E-14	22.62286103
HSPA12	-0.701770519	3.33907097	-8.2194376	1.03E-14	6.80E-14	22.61626282
SMPD1	-0.553646692	6.35836731	-8.218982226	1.03E-14	6.81E-14	22.61330479

SUMF2	-0.665934124	6.85806135	-8.217766948	1.04E-14	6.87E-14	22.60541104
ITGA1	-0.767739217	3.17037909	-8.21766208	1.04E-14	6.87E-14	22.60472991
SAXO2	-0.479992032	1.09278305	-8.217344452	1.04E-14	6.88E-14	22.6026669
PIGU	-0.468333922	5.05215083	-8.213181408	1.07E-14	7.07E-14	22.57563208
RNLS	-0.498801358	3.47513597	-8.213172513	1.07E-14	7.07E-14	22.57557433
RFT1	-0.430838683	3.82183843	-8.21130852	1.08E-14	7.15E-14	22.56347215
VGLL1	1.20310091	1.22270836	8.208803645	1.10E-14	7.27E-14	22.54721148
FASTKD!	-0.531368927	3.96965958	-8.208053617	1.10E-14	7.30E-14	22.54234314
ANKRD3	-0.3743521	2.56594359	-8.207458155	1.11E-14	7.33E-14	22.53847827
BCAS3	-0.476826742	2.68230492	-8.204992655	1.13E-14	7.44E-14	22.52247754
TNFSF1ℓ	0.885756641	1.04529468	8.204118699	1.13E-14	7.48E-14	22.51680638
VPS37D	-0.611406151	3.07395784	-8.203737754	1.14E-14	7.50E-14	22.5143345
HNRNPA	-0.467564064	7.8889162	-8.202988871	1.14E-14	7.53E-14	22.50947535
PFDN5	-0.501493998	8.35043997	-8.201563465	1.15E-14	7.60E-14	22.50022727
KLK7	2.675342995	3.54448403	8.20006574	1.16E-14	7.67E-14	22.49051099
TMEM18	-0.5108152	3.9825265	-8.198651268	1.18E-14	7.74E-14	22.48133576
ATG13	-0.478536157	5.28519067	-8.197769988	1.18E-14	7.79E-14	22.47561963
ZNF582	-0.442182678	2.8122325	-8.197135571	1.19E-14	7.82E-14	22.47150492
SUSD5	-1.034266481	2.14352286	-8.196784167	1.19E-14	7.83E-14	22.46922586
NENF	-0.554713965	6.96140754	-8.196006991	1.20E-14	7.87E-14	22.46418562
PCMT1	-0.575463664	6.20501935	-8.194321191	1.21E-14	7.95E-14	22.45325363
PYROXD	-0.802256119	3.48368827	-8.193586475	1.22E-14	7.99E-14	22.44848958
LAMTOR	-0.448718574	5.29394691	-8.192835776	1.22E-14	8.02E-14	22.44362216
PSMD13	-0.406139056	5.93709604	-8.191046402	1.24E-14	8.12E-14	22.43202116
AGMAT	-0.611057671	1.62120736	-8.190522245	1.24E-14	8.14E-14	22.42862319
CCDC12(	-0.612021013	2.9966004	-8.187815343	1.26E-14	8.28E-14	22.41107708
PPP1R1:	-0.601071822	4.82535862	-8.185466472	1.28E-14	8.41E-14	22.39585446
TRA2B	-0.518515688	4.87229868	-8.183436787	1.30E-14	8.52E-14	22.38270248
TBC1D4	-0.934305139	3.9228405	-8.182867942	1.30E-14	8.55E-14	22.37901681
NOD1	1.369086993	4.26045506	8.17994154	1.33E-14	8.71E-14	22.36005839
RIOK1	-0.473457494	3.83326127	-8.177056542	1.36E-14	8.87E-14	22.34137208
TSPAN3:	-0.55482533	5.22621018	-8.176485946	1.36E-14	8.90E-14	22.33767675
9-Mar	-0.417631131	4.61487069	-8.176297758	1.36E-14	8.91E-14	22.33645803
DNAJC5	-0.50947042	6.13758999	-8.175201989	1.37E-14	8.97E-14	22.32936206
ADRM1	-0.406398722	6.79788659	-8.174925079	1.37E-14	8.99E-14	22.32756894
SQLE	-0.677202575	3.34476638	-8.174536285	1.38E-14	9.01E-14	22.32505137
SSNA1	-0.476728809	6.78681899	-8.172060434	1.40E-14	9.15E-14	22.30902109
NDUFAF	-0.436668664	6.86047098	-8.167845961	1.44E-14	9.40E-14	22.28174035
LDLR	1.27334161	2.92512225	8.167268793	1.45E-14	9.44E-14	22.27800492
LOX	1.346308673	3.01643838	8.166056671	1.46E-14	9.51E-14	22.27016057

MUC21	1.881527901	2.32646772	8.161664299	1.50E-14	9.78E-14	22.24174068
ACSL3	-0.642872831	5.03552829	-8.160685796	1.51E-14	9.84E-14	22.2354107
STC2	-0.929540812	2.7003788	-8.158863212	1.53E-14	9.96E-14	22.22362152
INAVA	0.992339788	1.0596895	8.15720175	1.54E-14	1.01E-13	22.21287587
HSPBAP	-0.372832721	2.83143045	-8.156888622	1.55E-14	1.01E-13	22.21085084
GZF1	-0.523701703	3.34316195	-8.155160563	1.57E-14	1.02E-13	22.19967609
SFN	2.21212315	4.39433673	8.153705204	1.58E-14	1.03E-13	22.19026587
USE1	-0.492828698	4.94963627	-8.152562173	1.59E-14	1.04E-13	22.18287582
EAPP	-0.510447678	6.11005402	-8.152294969	1.59E-14	1.04E-13	22.18114836
SLC39A9	-0.551572761	4.96299501	-8.149991565	1.62E-14	1.05E-13	22.16625828
SAR1A	-0.487261949	5.6322189	-8.149682245	1.62E-14	1.05E-13	22.1642589
NGEF	1.806112235	3.71432067	8.14949185	1.62E-14	1.06E-13	22.16302826
SPATA5L	-0.465279784	3.09173051	-8.14824899	1.64E-14	1.06E-13	22.15499525
BMP8B	-0.876261834	2.68321307	-8.146520148	1.66E-14	1.08E-13	22.14382237
SCD	1.188706402	3.43255489	8.146320144	1.66E-14	1.08E-13	22.14252991
LDAH	-0.531364468	3.04650944	-8.144649608	1.68E-14	1.09E-13	22.13173532
CAPZB	-0.401399976	7.66477498	-8.143497436	1.69E-14	1.10E-13	22.12429104
NOL4	-0.678947801	0.5240393	-8.139505278	1.73E-14	1.12E-13	22.09850211
TOMM70	-0.464468156	5.46456005	-8.139444875	1.74E-14	1.12E-13	22.09811197
CRCP	-0.505397281	5.19920783	-8.138746966	1.74E-14	1.13E-13	22.09360433
P2RX5-T	-0.1849368	0.45631398	-8.137109544	1.76E-14	1.14E-13	22.08302948
FCHO1	0.903771317	1.53940793	8.136962235	1.76E-14	1.14E-13	22.08207819
UBAC2	-0.456243107	5.79051233	-8.136617963	1.77E-14	1.14E-13	22.07985498
KLHL23	-0.750128745	3.03822839	-8.136266864	1.77E-14	1.15E-13	22.07758775
TACC2	-0.709779448	3.63125586	-8.135448293	1.78E-14	1.15E-13	22.07230202
OR2T8	-0.596520868	0.7948609	-8.134653818	1.79E-14	1.16E-13	22.06717218
MRPL44	-0.487248703	5.27055023	-8.133513188	1.80E-14	1.17E-13	22.05980777
PTGES3L	-0.297209134	0.52266097	-8.131313681	1.83E-14	1.18E-13	22.0456085
SLC45A4	-0.671127522	3.45568149	-8.131059234	1.83E-14	1.18E-13	22.04396602
DHX35	-0.482730375	2.98706444	-8.1305805	1.84E-14	1.19E-13	22.04087584
GATM	-1.158252278	3.7249661	-8.130471524	1.84E-14	1.19E-13	22.04017241
MRFAP1	-0.447061005	6.77717285	-8.130167541	1.84E-14	1.19E-13	22.0382103
LBHD1	-0.472827969	1.79761545	-8.127068898	1.88E-14	1.21E-13	22.01821201
DZANK1	-0.551909987	2.0657264	-8.126807064	1.89E-14	1.21E-13	22.01652236
RNF8	-0.373537002	2.95882264	-8.1259987	1.90E-14	1.22E-13	22.0113061
UNC50	-0.542147451	5.9780049	-8.124645145	1.91E-14	1.23E-13	22.00257249
PLGRKT	-0.530952712	5.64051114	-8.1226304	1.94E-14	1.25E-13	21.98957424
CNNM2	-0.469732966	2.28220823	-8.118915364	1.99E-14	1.28E-13	21.96561142
CCDC10	-0.516972628	4.98315253	-8.116463056	2.02E-14	1.30E-13	21.94979701
MTMR11	0.958063142	2.84656619	8.114147876	2.05E-14	1.32E-13	21.9348695

IER2	-1.020485762	7.24612102	-8.11327714	2.06E-14	1.32E-13	21.92925593
SMARCA	-0.448356739	3.97879784	-8.112206988	2.07E-14	1.33E-13	21.92235723
SDF2	-0.512395791	5.7678873	-8.111168905	2.09E-14	1.34E-13	21.91566578
PSMA1	-0.380203591	5.60626327	-8.110797422	2.09E-14	1.34E-13	21.91327133
LAYN	-0.879414321	3.00453532	-8.109758336	2.11E-14	1.35E-13	21.90657409
RUNDC1	-0.496019571	4.46455378	-8.108502211	2.13E-14	1.36E-13	21.89847865
STARD8	-0.816846604	2.94805326	-8.107530958	2.14E-14	1.37E-13	21.89221964
FER1L6	-0.715075635	0.46003953	-8.106734053	2.15E-14	1.38E-13	21.88708451
MTERF3	-0.387417951	4.37551657	-8.105202046	2.17E-14	1.39E-13	21.87721334
SRSF8	-0.496696059	5.78706223	-8.10171732	2.22E-14	1.42E-13	21.85476432
CLDN10	1.920099509	2.3187156	8.10115488	2.23E-14	1.43E-13	21.85114155
HNRNPM	-0.576271524	6.51409926	-8.100783791	2.24E-14	1.43E-13	21.84875138
UBE2F	-0.418297129	4.68713318	-8.100436794	2.24E-14	1.43E-13	21.84651645
CNPY4	-0.553533891	4.33483296	-8.100226926	2.24E-14	1.43E-13	21.84516476
MRPS27	-0.476413551	5.05284402	-8.099769813	2.25E-14	1.44E-13	21.84222073
SLC25A1	-0.407998008	4.30365018	-8.098216863	2.27E-14	1.45E-13	21.8322197
IL4I1	1.260169408	2.11956624	8.097636835	2.28E-14	1.46E-13	21.82848459
ANXA9	-0.725892432	5.48810203	-8.097544803	2.28E-14	1.46E-13	21.82789196
MRPS24	-0.509272527	3.18948161	-8.096537625	2.30E-14	1.47E-13	21.82140663
CCDC13	-0.506185405	3.02755241	-8.09606694	2.31E-14	1.47E-13	21.818376
ARSK	-0.565595415	2.68828101	-8.095772588	2.31E-14	1.47E-13	21.8164808
QPRT	-1.474719516	3.36972281	-8.093634179	2.34E-14	1.49E-13	21.8027137
MAOA	-0.977700826	7.32223659	-8.09355212	2.34E-14	1.49E-13	21.80218545
MECR	-0.392114516	3.97115498	-8.091744376	2.37E-14	1.51E-13	21.79054892
ZNF180	-0.4960465	2.83965262	-8.091660151	2.37E-14	1.51E-13	21.79000679
PAK1IP1	-0.496573436	4.59044966	-8.089002218	2.41E-14	1.54E-13	21.77290046
PAF1	-0.440645448	6.79239628	-8.088655777	2.42E-14	1.54E-13	21.77067102
LEO1	-0.54205052	4.77849224	-8.086966185	2.45E-14	1.56E-13	21.75979888
LRR1	-0.395954146	2.79057634	-8.08695829	2.45E-14	1.56E-13	21.75974808
SYTL5	1.751813005	2.84022451	8.086076847	2.46E-14	1.56E-13	21.75407674
ZNF544	-0.421003264	3.63514121	-8.084705621	2.48E-14	1.58E-13	21.74525477
TEAD4	-0.657291703	3.93769546	-8.083576231	2.50E-14	1.59E-13	21.73798936
GTF2H2(	-0.367349466	1.09485997	-8.083138669	2.51E-14	1.59E-13	21.73517467
ANKS3	-0.542110938	3.03216714	-8.08128859	2.54E-14	1.61E-13	21.72327471
CATSPE	-0.358821184	0.98170922	-8.079144286	2.58E-14	1.63E-13	21.70948427
IST1	-0.431840534	5.68836278	-8.071680103	2.70E-14	1.71E-13	21.66149755
CREB5	0.941736881	1.75098902	8.071371572	2.71E-14	1.72E-13	21.65951459
TP53I11	-0.853414245	4.52282332	-8.069876542	2.74E-14	1.73E-13	21.64990652
TSNAX	-0.469128998	4.28865284	-8.069842002	2.74E-14	1.73E-13	21.64968455
OGDH	-0.57732048	6.74671832	-8.06918211	2.75E-14	1.74E-13	21.64544399

THAP9	-0.561014461	2.67679173	-8.066120366	2.80E-14	1.77E-13	21.62577146
ARF4	-0.572311901	7.66514586	-8.063548369	2.85E-14	1.80E-13	21.6092491
ALG12	-0.668229014	3.39139439	-8.0623932	2.87E-14	1.82E-13	21.60182938
CACNA2	-0.809696184	1.28116094	-8.057773556	2.96E-14	1.87E-13	21.57216344
PPM1G	-0.381727239	6.30554536	-8.057300508	2.97E-14	1.88E-13	21.56912624
CAB39L	-0.605166456	3.20723534	-8.057238012	2.97E-14	1.88E-13	21.56872499
RITA1	-0.41285335	5.17587418	-8.057141637	2.97E-14	1.88E-13	21.56810623
CNBD2	-0.107082016	0.21790773	-8.056394116	2.99E-14	1.89E-13	21.56330705
DIS3L2	-0.406344489	3.10920368	-8.055773353	3.00E-14	1.89E-13	21.55932187
ITGAE	-0.475844681	2.5434445	-8.055191707	3.01E-14	1.90E-13	21.55558798
EXOSC4	-0.538544789	5.18700842	-8.052413365	3.07E-14	1.93E-13	21.53775457
FLAD1	-0.37313344	4.85928241	-8.049859003	3.12E-14	1.96E-13	21.52136205
C8orf82	-0.503362855	4.21678228	-8.049517974	3.12E-14	1.97E-13	21.51917375
DPF3	-0.279149422	0.59884703	-8.049124012	3.13E-14	1.97E-13	21.51664585
BSG	-0.442857413	9.30759476	-8.047025339	3.18E-14	2.00E-13	21.50318074
FAM151I	-0.252219811	0.94970075	-8.046256241	3.19E-14	2.01E-13	21.49824672
PDLIM4	2.075525228	5.60633819	8.045600823	3.21E-14	2.02E-13	21.49404221
ABHD8	-0.468278027	5.03412901	-8.045223822	3.21E-14	2.02E-13	21.49162385
CPLANE	-0.596332723	4.04416186	-8.044876929	3.22E-14	2.02E-13	21.48939868
ATP6AP2	-0.575360256	8.23957002	-8.043671929	3.25E-14	2.04E-13	21.48166955
ABHD17I	-0.584190049	3.5831866	-8.042988711	3.26E-14	2.05E-13	21.47728756
SSB	-0.544979662	5.9735791	-8.0415451	3.29E-14	2.07E-13	21.46802931
RNF121	-0.54089074	4.70227663	-8.038613684	3.35E-14	2.11E-13	21.44923242
ZKSCAN	-0.434554924	3.01034663	-8.03665094	3.40E-14	2.13E-13	21.43664915
DDX59	-0.503474917	3.40798634	-8.03563447	3.42E-14	2.15E-13	21.43013323
KLF15	-0.890703117	2.60330225	-8.035393927	3.43E-14	2.15E-13	21.42859133
NAA60	-0.431694674	4.05571698	-8.033895083	3.46E-14	2.17E-13	21.41898428
HNRNPA	-0.509687798	6.9586413	-8.032926311	3.48E-14	2.18E-13	21.41277537
TNFRSF	1.141393006	2.08965849	8.030301472	3.54E-14	2.22E-13	21.39595488
FBXL15	-0.632355501	4.63155025	-8.030044304	3.55E-14	2.22E-13	21.39430706
GPANK1	-0.472318638	4.35893737	-8.028717547	3.58E-14	2.24E-13	21.38580635
ING3	-0.518690973	2.97319417	-8.027867588	3.60E-14	2.25E-13	21.38036098
TNFRSF	-1.510013287	4.8699436	-8.027334916	3.61E-14	2.26E-13	21.37694853
TTC23	-0.448536168	3.65357259	-8.023723358	3.70E-14	2.31E-13	21.3538154
ITGB4	1.164580915	4.92899293	8.02238104	3.73E-14	2.33E-13	21.34521902
ING2	-0.576204023	4.76755226	-8.020896983	3.76E-14	2.35E-13	21.33571593
GRHL3	1.052027236	1.89034361	8.019783847	3.79E-14	2.37E-13	21.3285887
C16orf95	-0.238152501	0.68285515	-8.018049407	3.84E-14	2.39E-13	21.31748454
CHRM3	-0.66253496	1.19612849	-8.017630505	3.85E-14	2.40E-13	21.31480288
ADGRL4	-0.759757652	4.70345901	-8.014738226	3.92E-14	2.44E-13	21.29628982

MRPL45	-0.519015877	5.99318921	-8.012016395	3.99E-14	2.49E-13	21.27887141
CXorf40E	-0.521380088	3.92175455	-8.011778641	3.99E-14	2.49E-13	21.27735007
PDSS1	-0.355084306	2.14208569	-8.010651715	4.02E-14	2.51E-13	21.27013945
TSPAN3	-0.557993677	7.06906003	-8.010396769	4.03E-14	2.51E-13	21.26850826
MAD2L1I	-0.467706321	4.87215379	-8.010388814	4.03E-14	2.51E-13	21.26845737
GABARA	-0.530841351	6.00966133	-8.009723459	4.05E-14	2.52E-13	21.26420047
RNF10	-0.381805091	6.4999289	-8.00932612	4.06E-14	2.53E-13	21.26165842
CHTOP	-0.433227618	5.57875718	-8.008813244	4.07E-14	2.53E-13	21.25837732
CD1D	-0.659567902	2.47549492	-8.007011205	4.12E-14	2.56E-13	21.24684983
DNAJB9	-0.770619798	5.97124694	-8.006195614	4.14E-14	2.57E-13	21.24163308
ARHGEF	-0.649985057	4.43886585	-8.004685737	4.18E-14	2.60E-13	21.23197632
TFAP2C	0.971231642	2.86800982	8.004317576	4.19E-14	2.60E-13	21.22962183
WDR20	-0.505383348	3.59564516	-8.003734742	4.21E-14	2.61E-13	21.22589457
HEXIM2	-0.520345663	2.91243408	-8.002464517	4.24E-14	2.63E-13	21.21777197
RBM4	-0.477010364	3.01700056	-7.999812936	4.32E-14	2.68E-13	21.20081863
PSMD10	-0.487394743	5.6601976	-7.999339931	4.33E-14	2.69E-13	21.19779474
JAGN1	-0.448728003	5.95428037	-7.998720575	4.35E-14	2.70E-13	21.1938354
CCDC32	-0.437976441	2.68764454	-7.996585912	4.41E-14	2.73E-13	21.18019062
C1D	-0.537934794	3.78348072	-7.996204173	4.42E-14	2.74E-13	21.17775077
DLG3	-0.532057686	4.89373901	-7.995229003	4.45E-14	2.75E-13	21.17151838
HDHD2	-0.713302561	4.51551815	-7.99176674	4.55E-14	2.82E-13	21.14939445
MGAT3	1.65968211	3.49274347	7.991466291	4.56E-14	2.82E-13	21.14747484
SELENO	-0.819497221	6.20145302	-7.990901232	4.58E-14	2.83E-13	21.14386473
ACE	-0.862701573	3.30108014	-7.990867865	4.58E-14	2.83E-13	21.14365156
IFT52	-0.505785993	5.40369316	-7.984914747	4.76E-14	2.94E-13	21.10562699
YAE1	-0.406686297	3.57450356	-7.982259551	4.84E-14	2.99E-13	21.08867284
SLC48A1	-0.588986207	5.91040458	-7.982222534	4.84E-14	2.99E-13	21.08843651
LYSMD1	-0.499156434	3.66134543	-7.982039698	4.85E-14	2.99E-13	21.08726918
TMEM70	-0.415848731	3.76487661	-7.980970426	4.88E-14	3.01E-13	21.08044265
ABCG2	-1.035037329	2.88510888	-7.980532007	4.89E-14	3.02E-13	21.07764383
SFT2D1	-0.471739123	5.41653588	-7.979896725	4.91E-14	3.03E-13	21.0735884
SRBD1	-0.574423499	3.53614311	-7.97911593	4.94E-14	3.05E-13	21.06860434
BAP1	-0.410246195	5.63395732	-7.978395637	4.96E-14	3.06E-13	21.06400675
DARS	-0.496181736	5.96555761	-7.976117696	5.04E-14	3.10E-13	21.04946839
ARHGEF	-0.78931853	5.15240133	-7.976106503	5.04E-14	3.10E-13	21.04939696
CABIN1	-0.577012126	4.9706837	-7.9753279	5.06E-14	3.12E-13	21.04442831
PSMA5	-0.388323951	5.28992518	-7.975058418	5.07E-14	3.12E-13	21.04270868
ZC3H15	-0.522992363	5.80467366	-7.974358995	5.09E-14	3.13E-13	21.03824565
FA2H	-0.884785033	2.04357925	-7.972674037	5.15E-14	3.17E-13	21.02749488
FARSA	-0.424219542	6.16769472	-7.972606912	5.15E-14	3.17E-13	21.02706663

ARNTL2	0.700225157	1.40583395	7.971821242	5.18E-14	3.18E-13	21.02205419
CSTF1	-0.429754869	4.19054322	-7.971812429	5.18E-14	3.18E-13	21.02199797
VTCN1	1.499042022	1.35784941	7.971155714	5.20E-14	3.19E-13	21.01780848
SMARCA	-0.733069943	5.75473315	-7.971056128	5.20E-14	3.19E-13	21.0171732
RAB6B	-0.664706718	2.82882972	-7.968613666	5.29E-14	3.24E-13	21.00159354
FGF11	-0.446737764	0.48158669	-7.968490836	5.29E-14	3.25E-13	21.00081012
SOX18	-1.028039335	4.32909783	-7.968454964	5.29E-14	3.25E-13	21.00058133
NUP88	-0.481137893	4.26482153	-7.968114016	5.30E-14	3.25E-13	20.99840679
RNF32	-0.338278403	1.23061254	-7.967911337	5.31E-14	3.25E-13	20.99711414
PCBP2	-0.393012838	7.16236001	-7.967601102	5.32E-14	3.26E-13	20.99513557
SLC22A2	-0.592954911	1.78048308	-7.96756648	5.32E-14	3.26E-13	20.99491476
CMBL	-0.869381145	4.52774642	-7.967296464	5.33E-14	3.26E-13	20.99319273
TBC1D19	-0.498619133	2.95025695	-7.966625339	5.36E-14	3.28E-13	20.98891277
SYVN1	-0.517873676	5.79915455	-7.965572948	5.39E-14	3.30E-13	20.98220181
DYNLL1	-0.448050603	6.95639921	-7.964827954	5.42E-14	3.31E-13	20.9774514
CDKN2C	-0.798176824	3.69268202	-7.964417232	5.43E-14	3.32E-13	20.97483258
FOXO1	-0.802126352	4.90007388	-7.961702295	5.53E-14	3.38E-13	20.95752374
GMNN	-0.513494469	3.59189193	-7.96061737	5.57E-14	3.40E-13	20.95060788
PITX3	0.509229994	0.55265817	7.959723371	5.60E-14	3.42E-13	20.94490952
SALL2	-0.905300071	3.96463474	-7.959594367	5.61E-14	3.42E-13	20.94408728
SCGN	-0.679819314	0.65864657	-7.958710084	5.64E-14	3.44E-13	20.93845127
SHARPIN	-0.40830985	6.20362987	-7.958451737	5.65E-14	3.44E-13	20.93680476
NT5DC1	-0.550899704	4.57522683	-7.957968511	5.66E-14	3.45E-13	20.93372513
PERM1	0.81331905	1.77076698	7.956349527	5.72E-14	3.49E-13	20.92340804
HTRA2	-0.44123115	4.46526798	-7.956286365	5.73E-14	3.49E-13	20.92300556
KRCC1	-0.535084153	6.29447272	-7.955718417	5.75E-14	3.50E-13	20.91938659
MCF2L	-0.603071279	2.55120487	-7.955400882	5.76E-14	3.51E-13	20.91736332
DHDDS	-0.408339552	4.49531935	-7.95419128	5.81E-14	3.53E-13	20.90965643
RPS6KA1	-0.435110442	5.46621264	-7.953855487	5.82E-14	3.54E-13	20.90751707
SNAP47	-0.475055622	4.01677175	-7.953169774	5.84E-14	3.55E-13	20.90314852
PLEKHA(	1.130790292	2.18776901	7.952438063	5.87E-14	3.57E-13	20.89848718
CREG1	-0.575210618	7.32046182	-7.952197026	5.88E-14	3.57E-13	20.89695172
NAA40	-0.546642336	4.04157002	-7.949872469	5.97E-14	3.63E-13	20.88214519
NOTCH4	-0.825756584	3.80232078	-7.949439235	5.99E-14	3.64E-13	20.87938594
FAAP20	-0.535826472	3.83461341	-7.948213128	6.03E-14	3.66E-13	20.87157741
PCTP	-0.437124443	3.17959295	-7.947690364	6.05E-14	3.67E-13	20.86824839
CYREN	-0.422226979	3.39206416	-7.946487743	6.10E-14	3.70E-13	20.86059044
ACAD8	-0.715726651	4.62473807	-7.945631746	6.14E-14	3.72E-13	20.85514012
FAM120A	-0.529853929	5.7303397	-7.94393853	6.20E-14	3.76E-13	20.8443601
NRIP2	-0.665933062	3.32948646	-7.94305875	6.24E-14	3.78E-13	20.83875944

PUS3	-0.377196074	3.9916453	-7.942852256	6.25E-14	3.78E-13	20.83744496
ZNF76	-0.446277066	4.66954559	-7.942477153	6.26E-14	3.79E-13	20.83505721
IL17D	-0.546556914	1.5814048	-7.94233744	6.27E-14	3.79E-13	20.83416787
RPUSD3	-0.362007075	3.92678902	-7.941856708	6.29E-14	3.80E-13	20.83110787
STARD7	-0.524834046	6.63670958	-7.941285028	6.31E-14	3.82E-13	20.8274691
SHLD3	-0.363864266	2.15872363	-7.941260851	6.31E-14	3.82E-13	20.82731521
MARCO	1.730960819	2.97003634	7.940062633	6.36E-14	3.84E-13	20.81968903
ATP5F1D	-0.665640437	6.55385526	-7.939646538	6.38E-14	3.85E-13	20.81704091
C19orf54	-0.455347964	3.60314415	-7.939466178	6.39E-14	3.86E-13	20.81589308
EMC2	-0.466989128	4.77067291	-7.938156117	6.44E-14	3.89E-13	20.80755623
RNASEH	-0.448432746	3.74970395	-7.937370086	6.47E-14	3.91E-13	20.80255455
CDKL3	-0.301961789	1.18983131	-7.935260527	6.56E-14	3.96E-13	20.78913247
ZNF444	-0.580561838	4.41707783	-7.935061877	6.57E-14	3.96E-13	20.78786867
BORCS8	-0.372996842	3.26200582	-7.933369231	6.64E-14	4.01E-13	20.77710093
NT5C	-0.52333139	5.7105422	-7.932343548	6.69E-14	4.03E-13	20.77057673
SLC25A5	-0.708226296	8.0549071	-7.931394962	6.73E-14	4.05E-13	20.76454338
TMEM17	-1.35370133	3.26362922	-7.930319735	6.77E-14	4.08E-13	20.75770508
ZNF77	-0.429360644	2.44288215	-7.929069134	6.83E-14	4.11E-13	20.74975213
YIPF3	-0.482756659	7.18563937	-7.927935404	6.88E-14	4.14E-13	20.74254306
SDHA	-0.51609287	4.87089082	-7.927115211	6.92E-14	4.16E-13	20.73732807
AK8	-0.601477509	3.08725237	-7.92485335	7.02E-14	4.22E-13	20.72294828
ARL6	-0.431894338	2.55913018	-7.924475076	7.04E-14	4.23E-13	20.72054364
AQP4	-1.46595049	2.87583462	-7.924100944	7.05E-14	4.24E-13	20.7181654
GTF2IRD	-0.39221924	1.54737393	-7.923121427	7.10E-14	4.26E-13	20.71193925
DPM3	-0.638272296	7.2307309	-7.923076453	7.10E-14	4.26E-13	20.71165339
PBX3	-1.26372544	6.37492011	-7.922929017	7.11E-14	4.27E-13	20.71071627
DDX28	-0.416043348	4.16365309	-7.922212011	7.14E-14	4.28E-13	20.7061591
MLH3	-0.498561966	3.39496505	-7.921422588	7.18E-14	4.31E-13	20.70114193
QRICH2	-0.667655944	1.86125615	-7.920407522	7.22E-14	4.33E-13	20.69469114
SLC25A3	-0.496650223	3.18383598	-7.92020323	7.23E-14	4.33E-13	20.69339292
CRIPT	-0.359185797	4.22794924	-7.920199862	7.23E-14	4.33E-13	20.69337151
AP5M1	-0.479528016	3.43214337	-7.919515558	7.26E-14	4.35E-13	20.68902308
IGDCC3	-0.928908507	1.25034632	-7.918002082	7.34E-14	4.39E-13	20.67940645
BTF3L4	-0.42593371	4.68840503	-7.917396745	7.36E-14	4.41E-13	20.67556045
CLP1	-0.487705091	3.44167565	-7.917226796	7.37E-14	4.41E-13	20.67448072
DCAF13	-0.418656968	3.29818131	-7.913853744	7.54E-14	4.51E-13	20.65305361
SUPV3L1	-0.337869308	4.00464844	-7.911656286	7.64E-14	4.57E-13	20.63909737
SIVA1	-0.559678273	5.28367067	-7.910712446	7.69E-14	4.60E-13	20.63310368
ZNF804B	-0.997442482	0.73869615	-7.908903024	7.78E-14	4.65E-13	20.62161448
USP40	-0.518393747	4.08056551	-7.908063686	7.82E-14	4.67E-13	20.61628552

TYW3	-0.510789261	4.13785805	-7.907460483	7.85E-14	4.69E-13	20.61245599
ZNF835	-0.471395443	1.74112831	-7.906346149	7.91E-14	4.72E-13	20.60538192
THEM4	-0.447132004	4.10873197	-7.906096703	7.92E-14	4.73E-13	20.60379846
TUBGCP	-0.497240877	3.1100806	-7.906093244	7.92E-14	4.73E-13	20.60377651
LPCAT2	-0.856188931	5.96386677	-7.905683666	7.94E-14	4.74E-13	20.60117661
RPL41	-0.615631637	9.34731803	-7.905153959	7.97E-14	4.75E-13	20.59781428
TTC34	-0.490332613	0.88614851	-7.902687241	8.10E-14	4.83E-13	20.58215854
C14orf28	-0.427322913	2.4441925	-7.902130057	8.13E-14	4.84E-13	20.57862262
PJA1	-0.784430478	5.70576761	-7.899198566	8.28E-14	4.93E-13	20.56002171
ATG5	-0.470540637	4.94736934	-7.898025839	8.35E-14	4.97E-13	20.55258169
SCOC	-0.491739116	5.10532633	-7.894212348	8.55E-14	5.09E-13	20.52839276
EFHB	-0.432174496	0.77152118	-7.893826487	8.57E-14	5.10E-13	20.52594564
ZNF630	-0.482972598	2.34373299	-7.891613854	8.70E-14	5.17E-13	20.51191459
SYNJ2BP	-0.511038881	4.06974065	-7.889579692	8.81E-14	5.24E-13	20.49901739
CALM1	-0.4479812	6.88049108	-7.887746895	8.92E-14	5.30E-13	20.48739864
IP6K3	-1.294080306	2.53655715	-7.887604255	8.93E-14	5.30E-13	20.48649447
WRNIP1	-0.447993747	5.75885248	-7.887318887	8.94E-14	5.31E-13	20.48468558
ABHD15	-0.525903089	4.13243213	-7.887241768	8.95E-14	5.31E-13	20.48419675
TOR1A	-0.449934517	5.08675395	-7.886166431	9.01E-14	5.35E-13	20.47738083
PRXL2C	-0.51503382	4.07922883	-7.885773982	9.03E-14	5.36E-13	20.47489348
TPRG1L	-0.501059809	6.84229951	-7.885430447	9.05E-14	5.37E-13	20.4727162
F8	-0.705177909	2.54472447	-7.882133656	9.25E-14	5.48E-13	20.45182452
HARS2	-0.414607484	4.84308132	-7.881733604	9.27E-14	5.49E-13	20.44928977
ARHGEF	-0.5643499	3.74387437	-7.880265887	9.36E-14	5.54E-13	20.43999088
RFXANK	-0.52270417	6.2220154	-7.879820788	9.38E-14	5.56E-13	20.43717111
HIGD1B	-1.005584827	3.41536827	-7.873640603	9.77E-14	5.78E-13	20.39802874
EEF1E1	-0.401570235	3.63155283	-7.871848911	9.88E-14	5.85E-13	20.38668451
EXTL2	-0.562198062	3.30840001	-7.871455689	9.90E-14	5.86E-13	20.384195
ZNF709	-0.245082163	0.64907131	-7.870255961	9.98E-14	5.90E-13	20.37659994
YDJC	-0.603356579	5.06856471	-7.868591283	1.01E-13	5.96E-13	20.3660626
HRC	-0.762722707	1.9175746	-7.868383279	1.01E-13	5.97E-13	20.36474605
PTPN4	-0.759736833	3.24993985	-7.865448766	1.03E-13	6.08E-13	20.34617432
MCCC1	-0.493718593	4.51188672	-7.865205661	1.03E-13	6.09E-13	20.34463597
DCPS	-0.359574978	3.77514756	-7.863774349	1.04E-13	6.14E-13	20.33557929
CCNA1	0.962635047	1.43996266	7.863377582	1.04E-13	6.16E-13	20.33306891
MTURN	-0.660176264	4.20274696	-7.863109336	1.05E-13	6.17E-13	20.33137174
GUK1	-0.43680874	6.64754261	-7.861619104	1.06E-13	6.22E-13	20.3219438
PDLIM3	-0.935071984	2.28601492	-7.860248305	1.06E-13	6.28E-13	20.31327241
RRBP1	-0.661966237	8.26693723	-7.859790986	1.07E-13	6.29E-13	20.31037971
HAUS1	-0.410110608	4.21363938	-7.85940084	1.07E-13	6.31E-13	20.30791199

DSC3	1.133317055	1.07881327	7.858353079	1.08E-13	6.35E-13	20.30128513
NEB	-0.983891747	0.84295463	-7.857317165	1.08E-13	6.39E-13	20.29473372
C9orf153	-0.143124746	0.2021843	-7.857182143	1.09E-13	6.39E-13	20.29387985
GLDC	-1.505975173	1.32454864	-7.855038333	1.10E-13	6.48E-13	20.28032362
EGFL7	-0.837773312	5.90315227	-7.852177073	1.12E-13	6.59E-13	20.26223418
IFT140	-0.673904391	4.48663131	-7.85053334	1.13E-13	6.66E-13	20.25184399
STOX2	-0.60268528	2.08460882	-7.850413306	1.13E-13	6.66E-13	20.2510853
NOP16	-0.384179025	3.5857735	-7.849303958	1.14E-13	6.71E-13	20.24407383
STXBP4	-0.425413773	1.72188305	-7.848887846	1.15E-13	6.73E-13	20.241444
C3	1.956899541	6.11743726	7.847309276	1.16E-13	6.79E-13	20.23146825
CCL13	1.544857083	2.58884948	7.843151104	1.19E-13	6.97E-13	20.20519659
STK17B	1.000449206	3.1716255	7.841818145	1.20E-13	7.03E-13	20.19677664
RPL7L1	-0.475214291	5.26986614	-7.840321007	1.21E-13	7.10E-13	20.18732067
SEMA6A	-0.570521417	1.9713148	-7.840038134	1.21E-13	7.11E-13	20.18553416
LTF	-1.16070644	2.305019	-7.839632605	1.22E-13	7.12E-13	20.18297307
ZNF747	-0.374195068	3.88044753	-7.839081105	1.22E-13	7.15E-13	20.17949024
LRRC70	-0.599982314	1.32239093	-7.836967208	1.24E-13	7.24E-13	20.16614197
DCAF11	-0.514433466	5.83172632	-7.834969679	1.25E-13	7.33E-13	20.15353053
BEX1	-1.726718087	4.21125867	-7.834862601	1.25E-13	7.33E-13	20.15285454
RAB3A	-0.571905488	2.82109511	-7.834859714	1.25E-13	7.33E-13	20.15283632
CHMP4B	-0.404898117	8.30779505	-7.833648886	1.26E-13	7.39E-13	20.14519275
COQ8B	-0.457770647	4.44395169	-7.832938897	1.27E-13	7.42E-13	20.14071116
LARGE2	-0.735574127	4.2882959	-7.832379766	1.27E-13	7.44E-13	20.13718199
SAP30	-0.575451296	4.16853826	-7.832156677	1.28E-13	7.45E-13	20.13577391
BAK1	-0.418878228	4.02273652	-7.831949826	1.28E-13	7.46E-13	20.13446835
MORN4	-0.450384084	4.20022981	-7.830472009	1.29E-13	7.53E-13	20.12514159
SAAL1	-0.348907026	3.19674116	-7.830136031	1.29E-13	7.54E-13	20.12302132
FAM76A	-0.45590028	3.89337028	-7.82899053	1.30E-13	7.59E-13	20.1157928
KCNK2	-1.213802826	1.95112301	-7.828933456	1.30E-13	7.59E-13	20.11543266
CAMK1D	-0.756141501	3.57747053	-7.828446244	1.31E-13	7.61E-13	20.11235838
BIK	-0.617421983	2.80606292	-7.827205816	1.32E-13	7.67E-13	20.10453189
NECAB1	-0.62760812	1.12669077	-7.827110485	1.32E-13	7.67E-13	20.10393043
ARL1	-0.510363887	6.32797242	-7.827109338	1.32E-13	7.67E-13	20.10392319
GIMAP7	-0.825965886	5.19771267	-7.824971604	1.34E-13	7.77E-13	20.09043703
MRPL30	-0.395793458	4.64305292	-7.824764531	1.34E-13	7.78E-13	20.0891308
COQ10A	-0.430689467	3.23238873	-7.824550454	1.34E-13	7.79E-13	20.08778042
PMPCB	-0.417600774	5.43465267	-7.824012068	1.34E-13	7.81E-13	20.08438441
MYPOP	-0.34697476	3.38064076	-7.820937787	1.37E-13	7.97E-13	20.06499533
MPP1	-0.463970466	3.77239413	-7.817659964	1.40E-13	8.13E-13	20.04432768
CD164	-0.63061466	7.67509272	-7.815896519	1.42E-13	8.22E-13	20.03321084

RAMP2	-0.801747618	5.98709411	-7.8136182	1.44E-13	8.34E-13	20.01885047
ODAM	-0.975166492	0.7599046	-7.813322716	1.44E-13	8.35E-13	20.01698821
C9orf24	-0.550897836	0.67595706	-7.811603376	1.45E-13	8.44E-13	20.00615307
LRRC47	-0.476681915	5.56020081	-7.810119393	1.47E-13	8.52E-13	19.99680231
YBEY	-0.51024292	4.33718908	-7.810037222	1.47E-13	8.52E-13	19.99628457
GRINA	-0.486960719	8.44159718	-7.809826714	1.47E-13	8.53E-13	19.99495823
HEMK1	-0.42626973	2.56882044	-7.806443916	1.50E-13	8.72E-13	19.97364736
ELOB	-0.536782817	8.62334959	-7.805694047	1.51E-13	8.75E-13	19.96892412
PIK3C2B	-0.612588111	3.30824736	-7.804329763	1.52E-13	8.83E-13	19.96033154
EHF	1.280738752	2.33107147	7.803908569	1.53E-13	8.85E-13	19.95767895
RASGEF	0.797434491	2.21556124	7.802187279	1.55E-13	8.94E-13	19.94683955
SAMD15	-0.661392507	2.20161794	-7.801410002	1.55E-13	8.99E-13	19.94194532
MICAL2	0.931270249	3.22239874	7.799445765	1.57E-13	9.10E-13	19.92957857
SMOX	-0.777401816	3.56751472	-7.799264186	1.57E-13	9.10E-13	19.92843545
PDSS2	-0.526734664	4.57955762	-7.797471328	1.59E-13	9.21E-13	19.91714952
TTC19	-0.486579437	5.12246894	-7.797231918	1.59E-13	9.22E-13	19.91564258
CHST12	-0.37861419	2.44247185	-7.794937719	1.62E-13	9.35E-13	19.90120334
MFF	-0.367447104	4.77306804	-7.794863636	1.62E-13	9.35E-13	19.90073712
GFM2	-0.536552334	4.08738343	-7.793849191	1.63E-13	9.41E-13	19.89435327
DNALI1	-1.053736097	3.93508583	-7.793148225	1.64E-13	9.45E-13	19.88994243
MRPL18	-0.40254242	6.5749538	-7.792480015	1.64E-13	9.49E-13	19.88573793
NIM1K	-0.586493425	0.75534844	-7.789835789	1.67E-13	9.64E-13	19.86910216
TMEM23	0.247940586	0.32450969	7.789746994	1.67E-13	9.65E-13	19.86854358
HMG20B	-0.444199956	5.64737807	-7.788068037	1.69E-13	9.75E-13	19.85798254
TM2D1	-0.537206474	4.03498618	-7.787466751	1.70E-13	9.78E-13	19.85420064
PFKFB1	-0.398978699	0.52821541	-7.784010437	1.74E-13	9.99E-13	19.83246504
COX4I2	-1.147046393	4.65127957	-7.7839997	1.74E-13	9.99E-13	19.83239753
CASP9	-0.520505895	3.78476838	-7.782664293	1.75E-13	1.01E-12	19.8240012
PRCD	-0.218732119	0.46877723	-7.781220671	1.77E-13	1.02E-12	19.81492547
IQCE	-0.483330924	3.84882608	-7.779876207	1.78E-13	1.03E-12	19.80647406
XPNPEP	-0.40409632	4.59612181	-7.779632843	1.78E-13	1.03E-12	19.80494435
INPPL1	-0.483941051	5.64738102	-7.778236346	1.80E-13	1.04E-12	19.79616697
COL25A1	-0.758830531	0.92506874	-7.776488867	1.82E-13	1.05E-12	19.78518494
SCFD2	-0.442498866	3.81808288	-7.774676884	1.84E-13	1.06E-12	19.77379914
CUX1	-0.48285012	4.91492663	-7.773230174	1.86E-13	1.07E-12	19.76470975
MSRB1	-0.423501213	4.73094921	-7.77252402	1.87E-13	1.07E-12	19.7602735
DDX56	-0.437687908	5.60587016	-7.771682286	1.88E-13	1.08E-12	19.75498583
INTS14	-0.487974073	5.10350028	-7.768805687	1.91E-13	1.10E-12	19.73691805
KLK10	2.483238952	4.26796694	7.767917959	1.92E-13	1.10E-12	19.73134311
EEFSEC	-0.382535334	4.62533056	-7.767597863	1.93E-13	1.10E-12	19.72933299

SLC9B1	-0.252171325	0.76788352	-7.766979743	1.94E-13	1.11E-12	19.72545152
PRTG	-0.806161016	2.15073949	-7.765364088	1.96E-13	1.12E-12	19.71530696
PTDSS1	-0.544883403	5.11531104	-7.764552546	1.97E-13	1.13E-12	19.71021184
USP19	-0.5121148	5.37694537	-7.764373411	1.97E-13	1.13E-12	19.70908722
DSC2	0.951581581	2.63001904	7.76091894	2.01E-13	1.15E-12	19.68740294
ZCCHC1	-0.40571179	3.71923394	-7.760199414	2.02E-13	1.16E-12	19.68288711
RSPH6A	-1.05182724	2.24729244	-7.759131242	2.03E-13	1.16E-12	19.6761836
ZNF771	-0.432814044	1.65312051	-7.759064441	2.04E-13	1.16E-12	19.67576441
EIF1B	-0.492975869	6.26433831	-7.75815433	2.05E-13	1.17E-12	19.67005333
DNAJC7	-0.370344237	4.65336663	-7.757508996	2.06E-13	1.17E-12	19.66600401
FICD	-0.592841069	3.15492314	-7.756226249	2.07E-13	1.18E-12	19.6579557
DMKN	-0.806785136	5.48783676	-7.755435715	2.08E-13	1.19E-12	19.65299608
CDK10	-0.595747199	5.14088815	-7.755215412	2.09E-13	1.19E-12	19.65161401
PUS7	-0.57876248	2.98798357	-7.751716665	2.13E-13	1.22E-12	19.62966785
RADIL	-0.520642984	0.71621963	-7.750897517	2.14E-13	1.22E-12	19.62453056
FSTL3	1.091631175	5.01231835	7.750335271	2.15E-13	1.23E-12	19.62100464
VWA1	-0.76761921	5.96611842	-7.749129314	2.17E-13	1.24E-12	19.61344243
ABT1	-0.3991457	4.81095777	-7.749083977	2.17E-13	1.24E-12	19.61315815
CCDC43	-0.380143361	4.73231626	-7.748946233	2.17E-13	1.24E-12	19.61229445
NSFL1C	-0.394781064	5.26078412	-7.747496503	2.19E-13	1.25E-12	19.6032047
TSPAN1	-0.439252663	4.16548819	-7.74417057	2.24E-13	1.27E-12	19.58235524
ATP1A1	-0.681585708	9.28555063	-7.744100624	2.24E-13	1.27E-12	19.58191682
DYNC1I1	-0.616886672	1.28037299	-7.743891502	2.24E-13	1.28E-12	19.58060608
RBM47	-0.603553407	5.97496195	-7.742772341	2.26E-13	1.28E-12	19.57359173
ZNF18	-0.382182798	3.28492952	-7.742101316	2.27E-13	1.29E-12	19.56938638
MEIG1	-0.305340956	0.5882431	-7.740200822	2.30E-13	1.30E-12	19.5574771
TSEN15	-0.416948683	5.15864798	-7.740080322	2.30E-13	1.31E-12	19.55672206
UBE2D3	-0.443443622	6.18365197	-7.739834672	2.30E-13	1.31E-12	19.55518286
XKR8	-0.435451803	4.03727657	-7.739500276	2.31E-13	1.31E-12	19.55308764
SCARF1	-0.684535959	4.13061064	-7.739326483	2.31E-13	1.31E-12	19.55199874
SS18L2	-0.379864663	4.96701264	-7.738606255	2.32E-13	1.32E-12	19.54748628
KIF22	-0.618679323	5.98259956	-7.738553004	2.32E-13	1.32E-12	19.54715266
GOSR2	-0.443974041	3.56584701	-7.735206767	2.37E-13	1.34E-12	19.52619092
LIX1L	-0.657730942	4.2878713	-7.73489587	2.37E-13	1.35E-12	19.52424366
PHF20L1	-0.593704499	2.92369128	-7.734867691	2.38E-13	1.35E-12	19.52406717
SAMD5	-0.909030439	1.24614548	-7.732853533	2.41E-13	1.36E-12	19.51145299
MAP7D3	-0.546551465	3.10786021	-7.732342201	2.41E-13	1.37E-12	19.50825096
SLC39A3	-0.405103957	3.6248748	-7.732263912	2.42E-13	1.37E-12	19.50776072
DYRK3	-0.565963394	2.64129822	-7.732138821	2.42E-13	1.37E-12	19.50697741
CFB	1.325144458	2.4351411	7.730974383	2.43E-13	1.38E-12	19.49968616

PRMT1	-0.468643486	6.24810505	-7.728979797	2.47E-13	1.39E-12	19.48719844
EXOSC3	-0.357007588	3.75311088	-7.728937719	2.47E-13	1.39E-12	19.48693502
TAPT1	-0.454809313	3.60817138	-7.727646798	2.49E-13	1.41E-12	19.4788539
GPR89A	-0.226878529	1.27129249	-7.727409461	2.49E-13	1.41E-12	19.47736827
HIST1H2	-1.088382166	4.04190819	-7.727207247	2.49E-13	1.41E-12	19.47610252
TMPRSS	1.245061436	1.18813538	7.718802583	2.63E-13	1.49E-12	19.42351197
CLDN23	-0.59035448	3.93182603	-7.717756668	2.65E-13	1.49E-12	19.41696984
MRGBP	-0.414518557	4.37740845	-7.715061439	2.69E-13	1.52E-12	19.40011391
OSBPL2	-0.411972961	3.91409992	-7.713009332	2.73E-13	1.54E-12	19.38728251
RBM8A	-0.443382383	5.71039303	-7.712222672	2.74E-13	1.55E-12	19.38236425
OR13A1	0.359836519	0.35350368	7.711326723	2.76E-13	1.55E-12	19.37676309
RHCE	-0.414836824	0.77855166	-7.710845193	2.77E-13	1.56E-12	19.3737529
CEBPG	-0.710609086	4.62737975	-7.710558037	2.77E-13	1.56E-12	19.37195785
RPP25	-0.625166466	4.23801925	-7.709618423	2.79E-13	1.57E-12	19.36608451
SLC22A3	2.149633946	4.39351634	7.709425374	2.79E-13	1.57E-12	19.36487785
ADCY1	-0.899743067	2.05964705	-7.708433982	2.81E-13	1.58E-12	19.35868143
ERLIN2	-0.616537647	5.07530397	-7.708109211	2.82E-13	1.58E-12	19.35665165
RAMAC	-0.424599432	4.2775716	-7.705050658	2.87E-13	1.61E-12	19.33753864
HIST1H4	-0.621932473	0.90688739	-7.703682815	2.90E-13	1.63E-12	19.32899246
AK7	-0.55377426	1.15042246	-7.703490125	2.90E-13	1.63E-12	19.32778863
ZMPSTE	-0.523579285	5.48846186	-7.703375101	2.90E-13	1.63E-12	19.32707002
CIAO3	-0.375694885	3.52345893	-7.703300371	2.90E-13	1.63E-12	19.32660315
IRAK1BP	-0.450473808	2.26766553	-7.701104144	2.94E-13	1.65E-12	19.3128837
UXS1	-0.58502096	4.77384571	-7.700790759	2.95E-13	1.66E-12	19.31092623
NANP	-0.441184577	2.48647468	-7.700714631	2.95E-13	1.66E-12	19.31045073
CARD11	1.140180899	2.2188698	7.700707474	2.95E-13	1.66E-12	19.31040603
RASL11A	-0.816371023	2.79336317	-7.700217225	2.96E-13	1.66E-12	19.30734396
BRF1	-0.370457783	2.29398493	-7.699792424	2.97E-13	1.66E-12	19.30469077
RFXAP	-0.477009602	2.98485877	-7.698774775	2.99E-13	1.67E-12	19.29833519
ARMC5	-0.5612181	3.62732478	-7.697906037	3.01E-13	1.68E-12	19.29291002
VBP1	-0.502183676	5.70544774	-7.6967682	3.03E-13	1.69E-12	19.28580494
SLC14A1	-0.850832307	1.52412191	-7.695289602	3.06E-13	1.71E-12	19.276573
FOXF2	-0.844230014	1.39848753	-7.693302169	3.09E-13	1.73E-12	19.26416578
MRPL12	-0.564792527	5.36819899	-7.692727546	3.11E-13	1.74E-12	19.26057888
NCOA5	-0.546254557	5.29970845	-7.692491248	3.11E-13	1.74E-12	19.25910391
TMEM64	-0.748290806	4.62891428	-7.69172278	3.13E-13	1.75E-12	19.25430734
EIF2S2	-0.450324414	6.29110003	-7.691600505	3.13E-13	1.75E-12	19.25354416
ZFP36L2	-0.756199118	7.75996091	-7.690453555	3.15E-13	1.76E-12	19.24638583
BPHL	-0.401408426	2.24830316	-7.690111889	3.16E-13	1.76E-12	19.24425356
ZNF569	-0.471741027	2.51851879	-7.689678514	3.17E-13	1.77E-12	19.24154903

CATSPE	-0.334039439	1.05734053	-7.689620693	3.17E-13	1.77E-12	19.2411882
ICA1	-0.39958518	3.75534114	-7.689057333	3.18E-13	1.77E-12	19.23767265
PYGO2	-0.500054517	6.2985242	-7.688644046	3.19E-13	1.78E-12	19.2350937
RUSC2	-0.514234099	4.23647421	-7.688474942	3.19E-13	1.78E-12	19.2340385
DIPK1A	-0.587133548	3.28159486	-7.688122317	3.20E-13	1.78E-12	19.23183819
ZNF446	-0.427560077	3.16481752	-7.688101962	3.20E-13	1.78E-12	19.23171118
AEN	-0.57077377	4.28777207	-7.687513604	3.21E-13	1.79E-12	19.22804009
IRX6	-1.047117351	0.80550901	-7.687211104	3.22E-13	1.79E-12	19.22615269
GDF9	-0.351076657	0.79517818	-7.686518551	3.23E-13	1.80E-12	19.2218318
CACYBP	-0.385821233	4.75057576	-7.683139388	3.30E-13	1.84E-12	19.20075241
CABLES:	-0.689903383	3.3766661	-7.681475189	3.34E-13	1.85E-12	19.19037317
LDLRAP:	-0.452266381	4.92634824	-7.680963415	3.35E-13	1.86E-12	19.18718164
CCT5	-0.512123962	5.71646644	-7.680031062	3.37E-13	1.87E-12	19.18136762
C2CD2	-0.460266354	3.05235542	-7.680015606	3.37E-13	1.87E-12	19.18127124
RIMS4	-1.255319844	2.21039311	-7.677877637	3.41E-13	1.90E-12	19.16794085
SNRPD1	-0.389693016	4.4075621	-7.676895578	3.43E-13	1.91E-12	19.16181842
HLTF	-0.69690703	4.54111337	-7.676874337	3.43E-13	1.91E-12	19.161686
ARL14EI	-0.436073039	4.16537324	-7.676160817	3.45E-13	1.91E-12	19.15723803
EIF5	-0.619659266	5.8798786	-7.676152355	3.45E-13	1.91E-12	19.15718528
BRD2	-0.581211761	6.79779094	-7.675637291	3.46E-13	1.92E-12	19.15397462
TEX2	-0.745427143	4.89982884	-7.675463346	3.47E-13	1.92E-12	19.15289036
TUBG2	-0.582481659	4.71883593	-7.675277061	3.47E-13	1.92E-12	19.15172919
ADCK1	-0.4181087	2.92489258	-7.674200836	3.49E-13	1.93E-12	19.14502115
TMEM41	-0.512144047	5.20746936	-7.673908584	3.50E-13	1.94E-12	19.14319967
CTNNBII	-0.480975088	5.56671229	-7.672685858	3.53E-13	1.95E-12	19.13557938
SYN3	-0.163567605	0.24537819	-7.671782745	3.55E-13	1.96E-12	19.12995148
GIMAP6	-0.764471854	4.16268849	-7.669756572	3.59E-13	1.99E-12	19.11732655
FAHD1	-0.400283468	4.74170048	-7.668777284	3.62E-13	2.00E-12	19.11122542
CCDC12	-0.364498393	2.6982951	-7.667151605	3.65E-13	2.02E-12	19.10109825
FAM151/	-0.489840171	0.86618058	-7.665611771	3.69E-13	2.04E-12	19.09150709
ZNF487	-0.417859035	2.36691364	-7.665329177	3.70E-13	2.04E-12	19.08974703
PGBD2	-0.490499743	3.02892858	-7.664997291	3.70E-13	2.05E-12	19.08768001
STK33	-0.511954445	3.05420556	-7.662436091	3.76E-13	2.08E-12	19.07173054
CCDC90I	-0.407003523	4.35648717	-7.662416905	3.76E-13	2.08E-12	19.07161107
BCL6B	-0.783896971	3.64962632	-7.661208811	3.79E-13	2.09E-12	19.06408902
KIF19	-0.946774273	0.88839314	-7.660836885	3.80E-13	2.10E-12	19.06177342
SLC16A1	-0.638781949	1.54337777	-7.659874764	3.83E-13	2.11E-12	19.0557836
ZNF527	-0.359089822	1.9016891	-7.659549258	3.83E-13	2.11E-12	19.05375722
GFM1	-0.583306232	3.99808878	-7.659212312	3.84E-13	2.12E-12	19.05165969
SH2D3C	-0.733056256	4.08880057	-7.658403541	3.86E-13	2.13E-12	19.04662521

TIGD1	-0.500243431	2.72938303	-7.652904138	4.00E-13	2.20E-12	19.01240107
RHOBTB	-0.800031767	2.73659988	-7.651046524	4.04E-13	2.23E-12	19.00084416
MORN1	-0.451631311	2.2454081	-7.650793991	4.05E-13	2.23E-12	18.99927319
TIGD6	-0.309458774	2.64094141	-7.65017989	4.07E-13	2.24E-12	18.9954531
RPS27L	-0.399370611	5.20858306	-7.65013776	4.07E-13	2.24E-12	18.99519104
C9orf92	-0.495207093	0.5216492	-7.649152023	4.09E-13	2.25E-12	18.98905957
ACSL1	-0.672299581	5.16858839	-7.646779431	4.16E-13	2.28E-12	18.97430363
NEIL2	-0.542481288	4.74469544	-7.646405271	4.17E-13	2.29E-12	18.97197687
KCTD10	-0.575235374	5.12676196	-7.645683611	4.18E-13	2.30E-12	18.96748932
UBE2L3	-0.392978805	5.96950104	-7.64562986	4.19E-13	2.30E-12	18.96715509
AAR2	-0.427445213	5.43829303	-7.645124141	4.20E-13	2.31E-12	18.96401052
FAM13A	-0.734319198	3.93883027	-7.644922797	4.20E-13	2.31E-12	18.9627586
MAGEL2	-0.661216099	0.84922705	-7.643985266	4.23E-13	2.32E-12	18.95692946
ZNF830	-0.438576598	4.52841508	-7.643136564	4.25E-13	2.33E-12	18.95165301
OLFM2	-1.292157465	3.56617953	-7.64211017	4.28E-13	2.35E-12	18.94527231
RAB27B	1.092108013	1.15036712	7.64091509	4.31E-13	2.36E-12	18.93784364
ZNF787	-0.419277322	4.50400193	-7.640157316	4.33E-13	2.37E-12	18.93313367
EMG1	-0.37086979	3.45325843	-7.639632646	4.35E-13	2.38E-12	18.92987273
COMMD!	-0.378226612	4.98345648	-7.639598025	4.35E-13	2.38E-12	18.92965755
ITGAX	0.986646447	2.49472466	7.637741358	4.40E-13	2.41E-12	18.91811913
ROMO1	-0.62505715	7.87792422	-7.636574945	4.43E-13	2.42E-12	18.91087124
PEX3	-0.581170814	4.84416646	-7.636562789	4.43E-13	2.42E-12	18.91079571
PRDM11	-0.706456258	2.7315043	-7.635724826	4.46E-13	2.44E-12	18.90558919
SLC28A3	0.688076101	0.97601143	7.634253519	4.50E-13	2.46E-12	18.89644838
ACY1	-0.458741961	2.73281645	-7.633414245	4.52E-13	2.47E-12	18.89123471
CBX1	-0.523805185	5.98652049	-7.633341307	4.52E-13	2.47E-12	18.89078162
FMOD	-1.068237883	5.97128552	-7.631847689	4.57E-13	2.49E-12	18.88150403
SEC23B	-0.722517034	5.90194616	-7.631673664	4.57E-13	2.50E-12	18.88042314
RAD17	-0.440830426	4.50162052	-7.631437576	4.58E-13	2.50E-12	18.87895681
HSPB7	-1.410301265	2.94968209	-7.631034958	4.59E-13	2.50E-12	18.87645623
MRPL17	-0.391307896	6.36848163	-7.631027814	4.59E-13	2.50E-12	18.87641186
SLC24A3	-0.958712782	1.99914798	-7.630541288	4.61E-13	2.51E-12	18.87339026
ZNF415	-0.465920845	3.56376562	-7.630115219	4.62E-13	2.52E-12	18.87074423
PCED1A	-0.562441212	6.05722967	-7.629208668	4.64E-13	2.53E-12	18.86511455
ST3GAL2	-0.476353414	3.08926552	-7.629099541	4.65E-13	2.53E-12	18.8644369
CRLF2	1.15201025	1.24186374	7.627380496	4.70E-13	2.56E-12	18.85376289
NPC1	0.63709712	3.45701326	7.624639736	4.78E-13	2.60E-12	18.8367479
PEX16	-0.431190634	5.18284442	-7.623124727	4.83E-13	2.63E-12	18.82734419
YY1	-0.456581553	5.82241906	-7.622141128	4.86E-13	2.64E-12	18.82123958
MDM1	-0.494278688	2.69162994	-7.620226807	4.92E-13	2.67E-12	18.80935996

CSRP1	-0.483460657	6.71238898	-7.619716701	4.93E-13	2.68E-12	18.80619473
RTN4RL1	-0.523093631	1.12561926	-7.619581974	4.94E-13	2.68E-12	18.80535877
EXT1	-0.596308447	4.06341082	-7.619507891	4.94E-13	2.68E-12	18.80489909
CCDC17	-0.431182541	0.54143105	-7.617663215	5.00E-13	2.71E-12	18.79345412
PAGR1	-0.463993913	4.1450348	-7.61730542	5.01E-13	2.72E-12	18.79123444
EFHC2	-0.786162593	1.52527419	-7.617214782	5.01E-13	2.72E-12	18.79067216
CTSL	-0.47807294	7.08596255	-7.610871056	5.21E-13	2.83E-12	18.75132837
FAM200A	-0.416053228	3.7513601	-7.60951474	5.26E-13	2.85E-12	18.74291917
IER3IP1	-0.442889081	5.46288389	-7.609376061	5.26E-13	2.85E-12	18.74205941
FBXW5	-0.393024804	6.76187076	-7.608808918	5.28E-13	2.86E-12	18.73854343
DNAJA3	-0.48950911	4.58886514	-7.608599606	5.29E-13	2.87E-12	18.73724584
AQP9	0.735414316	1.03493604	7.608515598	5.29E-13	2.87E-12	18.73672506
GOLGA8	-0.543247551	0.54603069	-7.608460474	5.29E-13	2.87E-12	18.73638334
MMEL1	-0.779885966	0.61585796	-7.607491487	5.33E-13	2.88E-12	18.7303767
DPH3	-0.463637388	4.51550293	-7.605753705	5.39E-13	2.91E-12	18.71960558
FAM83F	-0.616574861	2.98144825	-7.605199164	5.40E-13	2.92E-12	18.71616876
POMT2	-0.495415924	3.19539274	-7.604535348	5.43E-13	2.94E-12	18.71205489
CCT7	-0.428019584	7.31694189	-7.601726729	5.52E-13	2.99E-12	18.69465156
ANAPC5	-0.363840528	4.74773529	-7.601491692	5.53E-13	2.99E-12	18.69319535
GTPBP6	-0.43743332	5.84455188	-7.60133807	5.54E-13	2.99E-12	18.69224358
TDGF1	-0.360411293	0.37555313	-7.600592387	5.56E-13	3.01E-12	18.68762386
ZNHIT2	-0.562779296	5.23062148	-7.599940402	5.59E-13	3.02E-12	18.68358486
SLC6A20	1.270134979	1.20883966	7.597988456	5.66E-13	3.05E-12	18.67149399
FAM118B	-0.441481385	3.65482714	-7.596799315	5.70E-13	3.07E-12	18.66412909
ZNF132	-0.547150529	3.05948074	-7.596762346	5.70E-13	3.07E-12	18.66390014
RPL21	-0.641847998	7.96710502	-7.596515409	5.71E-13	3.08E-12	18.66237084
OPLAH	-0.727528564	2.58926313	-7.59563583	5.74E-13	3.09E-12	18.65692381
XXYLT1	-0.443099316	2.91310721	-7.593785957	5.81E-13	3.13E-12	18.64546925
TSSK6	-0.52200916	1.96351169	-7.593163073	5.83E-13	3.14E-12	18.6416127
CHKA	-0.618976634	4.9884233	-7.593159741	5.83E-13	3.14E-12	18.64159207
ADAMTS	-0.871912005	3.78629169	-7.591983471	5.87E-13	3.16E-12	18.63430981
WDR77	-0.348415865	4.34371612	-7.591937656	5.88E-13	3.16E-12	18.63402618
RPN2	-0.602906124	8.73983363	-7.590994906	5.91E-13	3.18E-12	18.62819017
C1GALT1	-0.576136704	1.55927802	-7.590198597	5.94E-13	3.20E-12	18.62326105
SLC25A4	-0.631590994	0.61142365	-7.589641758	5.96E-13	3.21E-12	18.61981443
PA2G4	-0.311348398	6.48308401	-7.589449254	5.97E-13	3.21E-12	18.61862294
EWSR1	-0.442665474	5.8553439	-7.588748996	5.99E-13	3.22E-12	18.61428892
KIAA055	-0.654481284	3.89547008	-7.586945502	6.06E-13	3.26E-12	18.60312791
HIST1H2	-0.609807525	1.88267615	-7.585980381	6.10E-13	3.28E-12	18.5971559
APOA5	-0.198579936	0.13597598	-7.58357407	6.19E-13	3.33E-12	18.58226814

FOXO6	-0.710219271	3.44318672	-7.583292548	6.20E-13	3.33E-12	18.58052656
ANKRD3	-0.381222478	0.87167616	-7.583092734	6.21E-13	3.33E-12	18.57929049
PHF11	-0.473045858	4.42071389	-7.5807145	6.31E-13	3.38E-12	18.56457995
SCAMP3	-0.429751044	6.34709822	-7.579941941	6.34E-13	3.40E-12	18.55980193
PTGS2	1.175599734	2.26217358	7.578990181	6.37E-13	3.42E-12	18.55391603
TSPAN1(	-0.513757442	1.7065	-7.578710586	6.39E-13	3.42E-12	18.55218704
RETSAT	-0.661231847	6.32175281	-7.578695666	6.39E-13	3.42E-12	18.55209478
GLT8D2	-1.077284182	3.15101073	-7.578033034	6.41E-13	3.43E-12	18.54799728
LAMB3	2.062150824	5.30178186	7.575681284	6.51E-13	3.48E-12	18.53345669
PRPF19	-0.374273745	7.10985031	-7.575611255	6.51E-13	3.49E-12	18.53302375
SYPL2	-0.859640863	3.10483112	-7.575273348	6.53E-13	3.49E-12	18.53093476
ABCF1	-0.461630277	5.48048995	-7.572903243	6.62E-13	3.54E-12	18.51628406
SMYD3	-0.529168002	2.50718712	-7.572493505	6.64E-13	3.55E-12	18.51375157
ARL5A	-0.618191649	4.68281625	-7.57110966	6.70E-13	3.58E-12	18.50519903
TRAM1L:	-0.609849836	3.10550776	-7.570559022	6.72E-13	3.59E-12	18.50179621
P2RY8	-0.862183609	3.37848128	-7.570110844	6.74E-13	3.60E-12	18.49902669
TMEM21	-0.433772327	7.30230199	-7.569843628	6.75E-13	3.61E-12	18.49737547
HIST1H3	-0.874446536	2.83918563	-7.567196872	6.87E-13	3.67E-12	18.48102228
TFEB	-0.419534691	4.44710323	-7.567119975	6.87E-13	3.67E-12	18.48054722
NSMCE1	-0.424028279	5.77583408	-7.566274662	6.91E-13	3.68E-12	18.47532518
BRSK2	-0.402806171	0.49764071	-7.565242049	6.95E-13	3.71E-12	18.46894657
NDUFS5	-0.485583528	9.67259968	-7.561536898	7.11E-13	3.79E-12	18.44606382
PTPN9	-0.610400355	4.59889776	-7.56132244	7.12E-13	3.80E-12	18.44473957
HMGCS1	-0.634836649	4.2664763	-7.560926824	7.14E-13	3.81E-12	18.44229673
SNAPC1	-0.505385475	3.7578734	-7.560644005	7.15E-13	3.81E-12	18.44055044
PSKH1	-0.512985465	4.81216854	-7.559779917	7.19E-13	3.83E-12	18.43521532
PODNL1	0.798698346	1.01230753	7.557192721	7.31E-13	3.89E-12	18.41924354
EMC9	-0.56038465	4.89708908	-7.557090834	7.32E-13	3.89E-12	18.41861462
AHR	0.942129729	5.50937884	7.556973417	7.32E-13	3.90E-12	18.41788985
C7orf25	-0.190772815	0.72094659	-7.555111971	7.41E-13	3.94E-12	18.40640077
COL11A:	1.280269168	1.01805246	7.552137773	7.55E-13	4.01E-12	18.38804736
GVQW3	-0.276825061	0.63968423	-7.551573296	7.57E-13	4.03E-12	18.38456456
EIF2S1	-0.444232003	4.93356824	-7.551515429	7.58E-13	4.03E-12	18.38420753
GABRA6	0.153101097	0.15222032	7.55134318	7.58E-13	4.03E-12	18.3831448
CHURC1	-0.322408292	2.32322543	-7.55053237	7.62E-13	4.05E-12	18.37814252
PRR18	-0.327925019	0.31249356	-7.550388834	7.63E-13	4.05E-12	18.37725701
TMEM50	-0.412924535	6.7885315	-7.549730159	7.66E-13	4.07E-12	18.37319362
MSX2	-0.507681796	0.96599369	-7.549461041	7.68E-13	4.07E-12	18.37153349
TMUB2	-0.436477816	5.33621935	-7.549215236	7.69E-13	4.08E-12	18.37001721
LZTFL1	-0.552663951	4.13344066	-7.54838433	7.73E-13	4.10E-12	18.36489187

CLCC1	-0.537284766	3.87898749	-7.547341632	7.78E-13	4.12E-12	18.35846062
LSG1	-0.444501563	4.57373164	-7.546829666	7.80E-13	4.13E-12	18.35530307
TCEANC	-0.335819426	1.93572401	-7.545648406	7.86E-13	4.16E-12	18.34801818
C2CD4B	1.190207907	2.11464394	7.545502477	7.87E-13	4.17E-12	18.34711828
IMMP2L	-0.479072685	4.04799915	-7.544124157	7.94E-13	4.20E-12	18.33861914
MSI1	-1.1433321	1.42652017	-7.541414133	8.07E-13	4.27E-12	18.32191117
BIRC3	1.221441349	2.59264747	7.537284447	8.28E-13	4.38E-12	18.29645795
PAMR1	-0.721134504	2.17242933	-7.535704543	8.37E-13	4.43E-12	18.2867226
SLC30A9	-0.530220261	5.02293927	-7.535262617	8.39E-13	4.44E-12	18.28399968
TUBG1	-0.391405698	5.12828024	-7.534738997	8.42E-13	4.45E-12	18.28077355
SARAF	-0.599216412	8.77937207	-7.533010937	8.51E-13	4.50E-12	18.27012761
TSPAN18	-1.211587974	4.31121794	-7.531979516	8.57E-13	4.52E-12	18.26377414
COL1A1	1.809327854	6.52060389	7.52816659	8.77E-13	4.63E-12	18.24029165
VAV3	-1.104393397	3.33540504	-7.527606844	8.80E-13	4.65E-12	18.236845
AFAP1L1	-0.703693768	3.14861698	-7.526397207	8.87E-13	4.68E-12	18.2293972
ASF1A	-0.491565754	5.12752026	-7.526317714	8.87E-13	4.68E-12	18.22890778
POLR3E	-0.430831793	3.8574904	-7.525579588	8.92E-13	4.70E-12	18.2243635
TMEM25	-0.453658188	6.69534568	-7.524799889	8.96E-13	4.72E-12	18.21956359
FNTB	-0.360477689	3.15129355	-7.524775124	8.96E-13	4.72E-12	18.21941114
DDX31	-0.508727951	3.97473553	-7.52186274	9.13E-13	4.81E-12	18.201485
HGH1	-0.399710861	5.14458784	-7.521318537	9.16E-13	4.82E-12	18.19813585
YIF1A	-0.45958572	6.32205153	-7.521179025	9.16E-13	4.83E-12	18.19727728
EMC6	-0.670929846	3.25786061	-7.521004848	9.18E-13	4.83E-12	18.1962054
TRAPPC	-0.357625843	6.24751394	-7.519999704	9.23E-13	4.86E-12	18.19002008
CYP26B1	-0.99624621	1.9565035	-7.519864121	9.24E-13	4.86E-12	18.18918578
MSANTD	-0.519931313	3.48191739	-7.519525128	9.26E-13	4.87E-12	18.18709987
ACRBP	-0.834199192	3.17458298	-7.517142067	9.40E-13	4.94E-12	18.17243798
SLC7A6	-0.569311829	2.11479962	-7.515557835	9.49E-13	4.99E-12	18.16269257
ATG101	-0.507921352	4.79693179	-7.514643285	9.55E-13	5.02E-12	18.15706729
PARP3	-0.502540729	5.0889776	-7.513611884	9.61E-13	5.05E-12	18.15072382
PITPNC1	-0.681987523	3.11542942	-7.512832023	9.66E-13	5.07E-12	18.14592777
ATP6V1F	-0.329931725	8.29830854	-7.510827099	9.78E-13	5.13E-12	18.13359918
SLC26A3	-0.372988412	0.2948542	-7.509918531	9.83E-13	5.16E-12	18.12801295
CENPO	-0.425843759	2.24027869	-7.507524715	9.98E-13	5.24E-12	18.11329689
DNAH9	-0.340001593	0.38418482	-7.507387277	9.99E-13	5.24E-12	18.11245207
CFAP65	-0.371068776	0.46976273	-7.506557708	1.00E-12	5.27E-12	18.10735303
SPEF2	-0.476140237	1.86376892	-7.505600549	1.01E-12	5.30E-12	18.10147019
DRAXIN	0.6120603	1.00152259	7.504848642	1.02E-12	5.32E-12	18.09684919
ST3GAL3	-0.464731489	3.67249742	-7.503336865	1.02E-12	5.37E-12	18.08755915
SLC26A9	-0.607723853	1.05025612	-7.502013155	1.03E-12	5.41E-12	18.07942578

SYNGR1	-0.826694238	2.67003658	-7.500558936	1.04E-12	5.46E-12	18.07049156
KIAA132	-1.314490078	1.73863641	-7.500394505	1.04E-12	5.46E-12	18.06948143
PRELID3	-0.561046418	5.82819201	-7.499522895	1.05E-12	5.49E-12	18.06412717
PBXIP1	-0.766734961	8.45830548	-7.499040576	1.05E-12	5.51E-12	18.06116448
C1orf131	-0.368827653	2.35240459	-7.497484293	1.06E-12	5.56E-12	18.05160568
TMBIM6	-0.494867092	9.14604869	-7.491383254	1.10E-12	5.77E-12	18.0141449
DBNL	-0.375807725	4.85964476	-7.491381125	1.10E-12	5.77E-12	18.01413183
RAB9B	-0.579097602	1.75763477	-7.490120572	1.11E-12	5.81E-12	18.00639438
TNFAIP8	-0.659930449	3.18778468	-7.490038108	1.11E-12	5.82E-12	18.00588824
RALB	-0.486681026	5.93711931	-7.488807362	1.12E-12	5.86E-12	17.9983346
MYCL	-0.935444922	2.16708091	-7.486230853	1.14E-12	5.95E-12	17.982524
HCN4	0.749780958	1.37842553	7.485989241	1.14E-12	5.96E-12	17.98104154
PLA2G4I	-1.015084271	1.60752786	-7.48419045	1.16E-12	6.02E-12	17.97000565
PSMD7	-0.443023981	5.43898071	-7.484146251	1.16E-12	6.02E-12	17.9697345
KMT5B	-0.544047248	4.65754868	-7.483605505	1.16E-12	6.04E-12	17.96641727
GOLGA8	-0.667258541	0.66668332	-7.481967959	1.17E-12	6.10E-12	17.95637262
ROPN1B	-0.317078469	0.42074902	-7.480920302	1.18E-12	6.14E-12	17.94994707
SELENO	-0.518469516	6.65033744	-7.480428561	1.18E-12	6.16E-12	17.94693129
RPS26	-0.833875043	7.58024471	-7.479039713	1.19E-12	6.21E-12	17.93841437
TPPP	-0.864924296	2.60664007	-7.478402082	1.20E-12	6.23E-12	17.93450452
ZNF875	-0.438127614	4.42508072	-7.478083716	1.20E-12	6.24E-12	17.93255243
TMEM18	-0.410611723	4.55069432	-7.476890798	1.21E-12	6.29E-12	17.92523843
ZNF20	-0.193074899	0.65695397	-7.475619076	1.22E-12	6.34E-12	17.91744209
CBWD1	-0.376810254	1.80388186	-7.474973466	1.22E-12	6.36E-12	17.91348448
PDZK1IP	2.219263888	5.06054218	7.47495936	1.22E-12	6.36E-12	17.91339801
SETD4	-0.450009483	2.90277125	-7.474852951	1.22E-12	6.36E-12	17.91274574
IL1RN	1.237900801	3.31990519	7.474276256	1.23E-12	6.38E-12	17.9092108
MTRF1	-0.389503345	2.38532749	-7.474248727	1.23E-12	6.38E-12	17.90904206
IFT27	-0.521784019	3.59390361	-7.473458986	1.24E-12	6.41E-12	17.90420152
CCNB1	-0.45658709	3.99697946	-7.472274808	1.24E-12	6.46E-12	17.89694397
ZNF652	-0.479087958	3.57523486	-7.471437307	1.25E-12	6.49E-12	17.89181158
SLC25A1	-0.514052059	3.43253716	-7.471150247	1.25E-12	6.50E-12	17.89005249
EBF3	-0.729724237	2.15232805	-7.467477205	1.28E-12	6.65E-12	17.86754815
EDA	-1.161411636	2.78731221	-7.465054124	1.30E-12	6.75E-12	17.85270607
QPCTL	-0.456709995	4.41711072	-7.464342021	1.31E-12	6.77E-12	17.84834482
3-Mar	-0.393071953	1.04378539	-7.463661293	1.31E-12	6.80E-12	17.84417597
WDR5B	-0.460303186	3.62841142	-7.463290105	1.32E-12	6.81E-12	17.84190288
SNCA	-0.708171919	1.91778849	-7.461482214	1.33E-12	6.89E-12	17.83083271
NKIRAS2	-0.331414751	4.2215559	-7.461241456	1.33E-12	6.90E-12	17.82935862
SCEL	1.673640211	4.64785012	7.461134578	1.33E-12	6.90E-12	17.82870424

ZMYND1	-0.644144517	6.34850897	-7.460615372	1.34E-12	6.92E-12	17.82552543
TFF2	-0.725918368	0.50175996	-7.45932546	1.35E-12	6.97E-12	17.81762861
CHID1	-0.462607737	5.79187582	-7.458310462	1.36E-12	7.02E-12	17.81141543
NECTIN3	-1.006659418	4.01654755	-7.457421805	1.37E-12	7.05E-12	17.80597608
RBM23	-0.485405242	4.24680329	-7.457402656	1.37E-12	7.05E-12	17.80585887
TTC23L	-0.30505448	0.62130675	-7.45695475	1.37E-12	7.07E-12	17.80311746
KDM4B	-0.556845638	3.90198757	-7.456413269	1.37E-12	7.09E-12	17.79980346
N6AMT1	-0.434377853	2.9584058	-7.454694064	1.39E-12	7.16E-12	17.78928252
KNTC1	0.425049085	1.85021625	7.454253904	1.39E-12	7.18E-12	17.78658914
LRRC42	-0.591202298	5.30208733	-7.452116206	1.41E-12	7.28E-12	17.77350983
UCHL5	-0.477253368	2.86745487	-7.451465998	1.42E-12	7.30E-12	17.76953207
RSPO4	1.490912495	3.30569943	7.449570576	1.43E-12	7.39E-12	17.75793777
TAF12	-0.349484565	4.89603742	-7.448883025	1.44E-12	7.42E-12	17.75373249
TMEM47	-0.904863015	4.08046195	-7.448735085	1.44E-12	7.42E-12	17.75282768
KIFC3	-0.546031036	4.8674076	-7.44797534	1.45E-12	7.46E-12	17.74818118
SLC20A1	0.712199511	4.32511834	7.447805892	1.45E-12	7.46E-12	17.7471449
GATA2	-0.640034487	2.12964394	-7.445631997	1.47E-12	7.56E-12	17.73385154
SLC14A2	-1.246806698	1.0278163	-7.445588002	1.47E-12	7.56E-12	17.73358253
ARMT1	-0.605006216	5.3798077	-7.445051963	1.47E-12	7.58E-12	17.73030505
EPHA10	0.987282292	1.65832302	7.444728031	1.48E-12	7.60E-12	17.72832452
C4orf46	-0.457170685	2.81294057	-7.443377847	1.49E-12	7.66E-12	17.72007004
KLF2	-0.982012578	5.24986895	-7.441876128	1.50E-12	7.73E-12	17.71089028
MEPCE	-0.495210345	6.57318187	-7.441394397	1.51E-12	7.75E-12	17.70794579
PMF1	-0.444613218	5.38142011	-7.441046754	1.51E-12	7.76E-12	17.70582096
NACA	-0.366309344	7.78129533	-7.440834243	1.51E-12	7.77E-12	17.70452211
UPRT	-0.400794574	4.08088485	-7.440488227	1.52E-12	7.79E-12	17.70240732
ACADSB	-0.67810572	3.71455914	-7.439745938	1.52E-12	7.82E-12	17.69787081
ADO	-0.495560811	4.46065273	-7.439050322	1.53E-12	7.85E-12	17.69361981
DMGDH	-0.801756517	1.99466411	-7.438753513	1.53E-12	7.86E-12	17.69180605
VCAN	1.22185228	2.34834189	7.437398108	1.55E-12	7.93E-12	17.68352393
GABARA	-0.388063494	6.97643904	-7.436037834	1.56E-12	7.99E-12	17.67521304
BUD13	-0.427827845	4.82048484	-7.435691466	1.56E-12	8.01E-12	17.67309698
SLC6A4	-0.222558312	0.25413265	-7.435570402	1.56E-12	8.01E-12	17.67235739
STPG1	-0.383066001	2.70256684	-7.432910534	1.59E-12	8.14E-12	17.65610983
SPN	0.839973453	1.8700315	7.431200037	1.61E-12	8.23E-12	17.64566339
RXFP1	-0.35380158	0.44577727	-7.427615518	1.64E-12	8.41E-12	17.62377684
TBC1D2(	-0.407416279	5.0630399	-7.426956809	1.65E-12	8.44E-12	17.6197556
SPIRE2	-0.739681009	2.14820587	-7.42676596	1.65E-12	8.45E-12	17.61859057
PTRH2	-0.335134352	3.1658525	-7.425940039	1.66E-12	8.49E-12	17.61354894
TXNDC9	-0.486793079	4.57726067	-7.425884276	1.66E-12	8.49E-12	17.61320857

MISP	1.409114354	2.37636353	7.424605112	1.67E-12	8.55E-12	17.605401
ZFYVE21	-0.360488304	4.70926346	-7.42410696	1.68E-12	8.58E-12	17.60236069
CDH6	1.038760137	3.07654846	7.424039558	1.68E-12	8.58E-12	17.60194933
CSF2	1.077760611	1.66621922	7.423333591	1.69E-12	8.61E-12	17.59764094
INTS9	-0.382215483	3.77334693	-7.422306154	1.70E-12	8.67E-12	17.59137114
RGS17	-0.710705025	0.9695825	-7.421900644	1.70E-12	8.69E-12	17.58889673
ZFAND2I	-0.489616705	5.63252382	-7.421384728	1.71E-12	8.71E-12	17.58574874
DGUOK	-0.461515761	6.2705394	-7.421021034	1.71E-12	8.73E-12	17.58352966
FIZ1	-0.358098241	3.60163822	-7.420748651	1.72E-12	8.74E-12	17.58186775
ARMH3	-0.455913883	4.49506162	-7.420327267	1.72E-12	8.76E-12	17.57929683
RBM14-F	-0.290817065	1.05443103	-7.419388503	1.73E-12	8.81E-12	17.57356962
DUOX1	-0.962194113	6.40240071	-7.417935563	1.75E-12	8.89E-12	17.56470645
GRAMD1	-0.841098424	2.53240939	-7.416672226	1.76E-12	8.95E-12	17.5570008
MMD	-0.810229158	3.85101465	-7.415950096	1.77E-12	8.99E-12	17.55259659
CAP2	-0.638484198	3.02747629	-7.413683575	1.79E-12	9.12E-12	17.53877506
TMED7	-0.582242038	6.54627821	-7.413548337	1.79E-12	9.12E-12	17.53795045
PLOD1	-0.519310157	6.30532729	-7.412497292	1.81E-12	9.18E-12	17.53154204
MRPL37	-0.410909677	5.84478459	-7.41208	1.81E-12	9.20E-12	17.52899791
SRSF3	-0.464049765	6.37967459	-7.41045265	1.83E-12	9.29E-12	17.51907719
PDE8A	-0.509998989	3.41713562	-7.403481052	1.91E-12	9.70E-12	17.47659257
NCS1	-0.647318474	5.05500439	-7.403358158	1.91E-12	9.70E-12	17.47584389
IVL	1.489867447	1.66600443	7.400942393	1.94E-12	9.85E-12	17.4611285
PPP2R2I	-0.319781742	3.86309649	-7.400642451	1.94E-12	9.86E-12	17.45930165
AKR1A1	-0.411168605	7.02735077	-7.400489722	1.95E-12	9.87E-12	17.45837145
EML6	-0.554800377	1.29506656	-7.400068009	1.95E-12	9.89E-12	17.45580305
THSD7B	-0.497057227	0.80147589	-7.399931246	1.95E-12	9.90E-12	17.45497012
HDGFL3	-0.437728338	3.72870288	-7.398742317	1.97E-12	9.97E-12	17.44772966
CLPTM1	-0.469778759	6.49077137	-7.39765582	1.98E-12	1.00E-11	17.44111366
CD55	1.409947294	6.82095924	7.397321958	1.98E-12	1.00E-11	17.4390808
UPF3A	-0.490872283	4.80327266	-7.394918336	2.01E-12	1.02E-11	17.42444709
GHR	-0.575714517	1.2919702	-7.391400595	2.06E-12	1.04E-11	17.40303595
SDR16C5	0.753032607	0.7610333	7.389771932	2.08E-12	1.05E-11	17.39312513
UBALD1	-0.42908058	4.88524762	-7.389510853	2.08E-12	1.05E-11	17.39153653
NOP56	-0.428887411	5.28277872	-7.387756318	2.10E-12	1.06E-11	17.38086159
HLA-G	1.509434486	3.72214429	7.386853251	2.12E-12	1.07E-11	17.37536778
MACROI	-0.562186043	5.86040622	-7.386601923	2.12E-12	1.07E-11	17.3738389
GALK2	-0.478171171	2.78350775	-7.386445887	2.12E-12	1.07E-11	17.37288972
RPL19	-0.408828145	10.4111341	-7.383991062	2.15E-12	1.09E-11	17.35795853
ZFP41	-0.408418002	2.3172182	-7.383314539	2.16E-12	1.09E-11	17.35384422
RCHY1	-0.523130016	3.75499468	-7.382335658	2.18E-12	1.10E-11	17.34789154

ING4	-0.468954908	5.67061657	-7.382047229	2.18E-12	1.10E-11	17.34613767
TMEM18	-0.431235203	2.0586931	-7.381160398	2.19E-12	1.11E-11	17.34074533
SCRN2	-0.514162109	6.12222474	-7.380852199	2.20E-12	1.11E-11	17.33887143
LAMTOR	-0.40885923	6.30280635	-7.379424978	2.22E-12	1.12E-11	17.33019439
PDCL3	-0.40279676	4.87958974	-7.377380729	2.24E-12	1.13E-11	17.31776791
HSD17B:	-0.543823112	5.00473388	-7.374528044	2.28E-12	1.15E-11	17.30043088
FAM210/	-0.431692692	3.52428843	-7.374502284	2.28E-12	1.15E-11	17.30027434
TAF6L	-0.413201224	3.98559086	-7.372134146	2.32E-12	1.17E-11	17.28588545
GAMT	-0.557518639	5.71224635	-7.367149067	2.39E-12	1.20E-11	17.25560572
FAM81A	-0.662529644	1.87274877	-7.366442117	2.40E-12	1.21E-11	17.25131274
LETM1	-0.470370038	4.23988914	-7.364877775	2.43E-12	1.22E-11	17.24181414
BOP1	-0.55511547	5.22699467	-7.364387549	2.43E-12	1.22E-11	17.23883778
ARL14EF	0.339605306	0.34053593	7.362702668	2.46E-12	1.24E-11	17.22860919
FCSK	-0.377024876	3.95134642	-7.360924419	2.49E-12	1.25E-11	17.21781543
FAM219/	-0.456024706	3.88370604	-7.359956714	2.50E-12	1.26E-11	17.21194228
MIS12	-0.483264141	4.15970158	-7.359736925	2.50E-12	1.26E-11	17.21060841
TGM2	1.089224286	5.23105868	7.359528026	2.51E-12	1.26E-11	17.20934066
HSD17B:	-0.619356695	0.86506343	-7.359395967	2.51E-12	1.26E-11	17.20853925
PDZD9	-0.147354908	0.26664684	-7.359047584	2.51E-12	1.26E-11	17.20642509
COG7	-0.409130186	4.72669449	-7.358980142	2.52E-12	1.26E-11	17.20601582
PNO1	-0.481077235	4.49076981	-7.358450188	2.52E-12	1.27E-11	17.20279994
NMNAT3	-0.553014877	2.29633099	-7.356461189	2.55E-12	1.28E-11	17.19073159
BRD7	-0.382787241	4.66245295	-7.356092501	2.56E-12	1.28E-11	17.18849479
BAG1	-0.446832457	5.43369142	-7.355073898	2.58E-12	1.29E-11	17.18231538
TMEM18	0.857871789	1.95180017	7.349878011	2.66E-12	1.33E-11	17.15080293
AP4B1	-0.449674316	3.11831304	-7.346698743	2.71E-12	1.36E-11	17.13152818
AGPAT1	-0.474470152	6.07914058	-7.345008212	2.74E-12	1.37E-11	17.12128131
SRSF1	-0.432747986	6.8072736	-7.344443295	2.75E-12	1.38E-11	17.1178575
BTBD6	-0.399456394	5.88366371	-7.342516686	2.78E-12	1.39E-11	17.10618214
P4HTM	-0.486734186	5.1114451	-7.340938237	2.81E-12	1.41E-11	17.09661813
TMED2	-0.572077937	7.75841895	-7.337865643	2.87E-12	1.43E-11	17.07800476
FAH	-0.47230869	3.90617262	-7.337083556	2.88E-12	1.44E-11	17.07326779
WSB2	-0.507306897	5.71057272	-7.337053056	2.88E-12	1.44E-11	17.07308307
PIGK	-0.523902556	4.44605625	-7.335909741	2.90E-12	1.45E-11	17.06615881
CLDND2	-0.570889408	1.92383233	-7.334689243	2.92E-12	1.46E-11	17.05876788
ZNF329	-0.508392905	3.61718236	-7.333374379	2.95E-12	1.47E-11	17.05080641
CENPT	-0.52233373	3.36291421	-7.333236259	2.95E-12	1.47E-11	17.04997014
TRPM3	-0.350214031	0.25998309	-7.33276726	2.96E-12	1.48E-11	17.04713063
SYT9	-0.460655122	0.57761087	-7.332742394	2.96E-12	1.48E-11	17.04698008
AQP7	-0.689738413	0.80435795	-7.331189382	2.99E-12	1.49E-11	17.03757835

LIMS2	-0.66758547	3.22813953	-7.330525913	3.00E-12	1.50E-11	17.03356218
TIGD7	-0.365231158	2.02618618	-7.327842099	3.05E-12	1.52E-11	17.01731874
MBD3	-0.454452465	4.94621845	-7.327414719	3.06E-12	1.52E-11	17.01473243
CACNB4	-0.610131289	0.7944697	-7.327317785	3.06E-12	1.52E-11	17.01414585
IMPACT	-0.577919451	4.73312661	-7.327074981	3.06E-12	1.53E-11	17.01267656
VAPA	-0.407422799	5.46648398	-7.326978771	3.06E-12	1.53E-11	17.01209438
TBKBP1	-0.521905412	3.67853849	-7.3268645	3.07E-12	1.53E-11	17.01140291
DCTN5	-0.442123773	4.07872135	-7.325971361	3.08E-12	1.54E-11	17.00599862
RAG2	-1.369695515	0.89359175	-7.324721928	3.11E-12	1.55E-11	16.99843917
KAT8	-0.388552752	5.27886575	-7.324001005	3.12E-12	1.55E-11	16.99407774
ILF2	-0.502405856	7.63901437	-7.323897822	3.12E-12	1.55E-11	16.99345353
SSBP3	-0.528574469	4.82507498	-7.322366512	3.15E-12	1.57E-11	16.98419046
PNRC2	-0.460564136	6.7967686	-7.321100215	3.18E-12	1.58E-11	16.97653144
ZBED3	-0.574529268	4.47598276	-7.320375518	3.19E-12	1.59E-11	16.97214859
TERF1	-0.461966659	2.99326535	-7.32025912	3.19E-12	1.59E-11	16.97144466
MEN1	-0.414283872	5.0343624	-7.319544237	3.21E-12	1.59E-11	16.9671215
FRG1	-0.424188324	5.3795221	-7.3183826	3.23E-12	1.60E-11	16.96009723
BNC1	0.835406894	1.07118884	7.317094615	3.26E-12	1.62E-11	16.95230981
AP3B1	-0.541232033	4.93969967	-7.316876831	3.26E-12	1.62E-11	16.95099313
RPS13	-0.425912796	8.76127314	-7.316449242	3.27E-12	1.62E-11	16.94840809
SFMBT1	-0.478705453	2.45226429	-7.315702539	3.29E-12	1.63E-11	16.94389403
NOL10	-0.352833134	4.45854071	-7.31525715	3.29E-12	1.63E-11	16.94120165
DCP1B	-0.39388775	3.87396559	-7.308192786	3.44E-12	1.71E-11	16.89851195
CLK3	-0.385925413	3.23533946	-7.307295684	3.46E-12	1.71E-11	16.89309272
RARS2	-0.392899578	5.19767377	-7.306590474	3.47E-12	1.72E-11	16.88883298
MAFB	-0.813320219	5.36129911	-7.306419257	3.48E-12	1.72E-11	16.88779881
LETMD1	-0.442413513	5.24362578	-7.3061203	3.48E-12	1.73E-11	16.8859931
SCYL1	-0.337824876	6.41286222	-7.305469146	3.50E-12	1.73E-11	16.88206029
IFT74	-0.485283404	4.14070751	-7.305465017	3.50E-12	1.73E-11	16.88203535
HID1	-0.566887755	3.83868992	-7.304889846	3.51E-12	1.74E-11	16.87856165
ELMOD3	-0.435600236	3.16324305	-7.301955024	3.58E-12	1.77E-11	16.86083978
CFAP157	-0.277200662	0.82655018	-7.301386286	3.59E-12	1.77E-11	16.857406
RAD9A	-0.504344217	3.59143787	-7.299110035	3.64E-12	1.80E-11	16.84366481
RPA2	-0.485450031	6.71374023	-7.297717944	3.67E-12	1.81E-11	16.83526246
COL4A6	-0.413345694	0.45368968	-7.297690781	3.67E-12	1.81E-11	16.83509852
GIMAP1	-0.610528797	2.73342464	-7.297409487	3.68E-12	1.82E-11	16.83340083
BANF1	-0.417089267	7.91773327	-7.295609613	3.72E-12	1.84E-11	16.82253905
ZNF280B	-0.500909525	1.76293275	-7.294799207	3.74E-12	1.84E-11	16.81764903
ACOT1	-0.528569113	2.42289919	-7.294671939	3.74E-12	1.84E-11	16.81688113
TMEM26	-0.534804487	5.46916123	-7.292935303	3.78E-12	1.86E-11	16.80640352

KSR1	-0.655231781	3.01257844	-7.291655862	3.81E-12	1.88E-11	16.79868533
ZNF174	-0.411630732	3.66237695	-7.291523827	3.81E-12	1.88E-11	16.79788889
DUSP5	1.491350949	6.65385347	7.291521384	3.81E-12	1.88E-11	16.79787415
MMADH(	-0.535486074	6.20705986	-7.291088058	3.82E-12	1.88E-11	16.79526036
NUTF2	-0.364735529	5.78262151	-7.290834376	3.83E-12	1.89E-11	16.79373022
IBA57	-0.382993523	1.77789108	-7.290482732	3.84E-12	1.89E-11	16.79160925
RAB20	-0.682278036	6.05150659	-7.290155168	3.84E-12	1.89E-11	16.78963359
TMEM14	-0.3399298	2.70643728	-7.288558347	3.88E-12	1.91E-11	16.78000337
CD274	0.812576076	2.7011023	7.287583924	3.91E-12	1.92E-11	16.77412742
HDAC8	-0.339424338	3.84372812	-7.287441383	3.91E-12	1.92E-11	16.77326792
RPS3A	-0.509751045	8.98419591	-7.285796224	3.95E-12	1.94E-11	16.76334862
RUNX2	1.068458374	2.32241481	7.284036137	3.99E-12	1.96E-11	16.752738
TMUB1	-0.409570865	5.97887036	-7.283881135	4.00E-12	1.96E-11	16.75180366
DEPDC1	1.218715743	2.40789228	7.283509699	4.00E-12	1.97E-11	16.74956471
PAICS	-0.481011146	4.62952376	-7.283008561	4.02E-12	1.97E-11	16.74654407
GNAS	-0.751150577	9.66153818	-7.282714918	4.02E-12	1.98E-11	16.74477418
POMT1	-0.441519373	4.05937398	-7.282040236	4.04E-12	1.98E-11	16.74070781
MRPL50	-0.406980165	4.43162164	-7.281473665	4.06E-12	1.99E-11	16.73729323
CCDC12	-0.475292185	4.99270023	-7.280987886	4.07E-12	1.99E-11	16.7343657
ABTB2	1.048362863	3.47323415	7.280644042	4.08E-12	2.00E-11	16.73229362
ARL2	-0.457787752	5.70986448	-7.280481716	4.08E-12	2.00E-11	16.73131542
CRLS1	-0.349125206	4.68580004	-7.279507007	4.10E-12	2.01E-11	16.72544202
SIKE1	-0.46189946	3.69667967	-7.279238789	4.11E-12	2.01E-11	16.72382589
ERMARD	-0.401518175	3.96610358	-7.27862934	4.13E-12	2.02E-11	16.72015382
FAM171E	-0.941497709	2.05498771	-7.275081696	4.22E-12	2.07E-11	16.69878249
ZNF491	-0.371967079	1.19783847	-7.273625798	4.26E-12	2.08E-11	16.69001401
ITFG1	-0.603241029	5.01229362	-7.273288416	4.26E-12	2.09E-11	16.68798221
PSAT1	-1.308366469	3.69702572	-7.272912775	4.27E-12	2.09E-11	16.68572008
TMEM12	-0.376999696	5.08443918	-7.272332427	4.29E-12	2.10E-11	16.68222535
SLPI	2.106299394	7.03913718	7.272064658	4.30E-12	2.10E-11	16.68061296
LRMDA	-0.328109146	3.23586391	-7.267388419	4.42E-12	2.16E-11	16.65246098
POLR1D	-0.346503974	4.77953958	-7.267132113	4.43E-12	2.16E-11	16.65091831
CCT2	-0.450430456	5.95018147	-7.265317037	4.48E-12	2.19E-11	16.63999461
TMEM20	-0.405183347	2.78586605	-7.26404548	4.51E-12	2.20E-11	16.63234305
SPON1	1.18867817	1.70798096	7.263765078	4.52E-12	2.21E-11	16.63065585
SLCO4A	0.889935083	1.98895099	7.261420639	4.59E-12	2.24E-11	16.61655091
SUGP2	-0.560889011	4.74836503	-7.25852839	4.67E-12	2.28E-11	16.59915428
ZPR1	-0.394112916	4.0948375	-7.257246417	4.71E-12	2.30E-11	16.59144478
PDCD4	-0.643880331	7.77808543	-7.256584447	4.72E-12	2.30E-11	16.58746419
PAPOLA	-0.454474651	5.0634252	-7.255526222	4.76E-12	2.32E-11	16.58110132

KIF1A	-1.055474853	1.00114434	-7.253849452	4.80E-12	2.34E-11	16.57102052
PARP1	-0.546956942	5.45963227	-7.253445872	4.82E-12	2.35E-11	16.56859441
KRT222	-0.87130092	1.14973922	-7.2513538	4.88E-12	2.38E-11	16.55601941
ZFAND3	-0.516369515	6.2579057	-7.250867965	4.89E-12	2.38E-11	16.5530995
ZNF843	-0.353875415	1.18187669	-7.250802068	4.89E-12	2.38E-11	16.55270346
EEPD1	-0.391534124	1.59027587	-7.250742393	4.90E-12	2.38E-11	16.55234482
TLK2	-0.378279591	3.53618516	-7.25059007	4.90E-12	2.38E-11	16.55142939
TNC	1.53553893	4.13576414	7.248828656	4.95E-12	2.41E-11	16.54084448
PRICKLE	-0.580144704	2.98932581	-7.246989563	5.01E-12	2.44E-11	16.52979459
GJB6	-1.122697975	2.15599552	-7.246585965	5.02E-12	2.44E-11	16.52736989
CDC37L1	-0.403688662	4.54181226	-7.245413596	5.06E-12	2.46E-11	16.52032712
BDH2	-0.500987615	6.26513733	-7.244779549	5.08E-12	2.47E-11	16.51651852
COL9A1	-0.299135088	0.38872172	-7.244709048	5.08E-12	2.47E-11	16.51609504
ZNF680	-0.599917252	4.16079678	-7.243714248	5.11E-12	2.48E-11	16.51011996
ITGA2	1.260803473	4.05388802	7.243283771	5.13E-12	2.49E-11	16.50753454
WDR59	-0.440586975	3.93883901	-7.241362859	5.19E-12	2.52E-11	16.4959989
UTP11	-0.352025773	4.556406	-7.24037588	5.22E-12	2.53E-11	16.4900726
CPSF4	-0.36534628	3.87748625	-7.239954796	5.23E-12	2.54E-11	16.48754436
ZNF34	-0.433260384	3.66765866	-7.238961284	5.26E-12	2.55E-11	16.48157958
ARSJ	0.849003891	1.54042544	7.238676103	5.27E-12	2.56E-11	16.47986754
NDRG3	-0.424601425	5.47465806	-7.23723985	5.32E-12	2.58E-11	16.47124584
CITED2	-0.98013425	7.36000447	-7.237178875	5.32E-12	2.58E-11	16.47087984
TRIM61	-0.570767877	1.46811486	-7.237092725	5.32E-12	2.58E-11	16.47036273
GPATCH	-0.36990767	4.46743669	-7.23614821	5.35E-12	2.59E-11	16.46469359
AOX1	-0.99985691	1.36157477	-7.23457321	5.41E-12	2.62E-11	16.45524125
CBX4	-0.542566048	5.30271222	-7.233848836	5.43E-12	2.63E-11	16.45089438
MMUT	-0.524802917	5.00172787	-7.232520373	5.47E-12	2.65E-11	16.44292321
DEGS2	-0.713983328	3.17093012	-7.231311666	5.51E-12	2.67E-11	16.43567144
MFAP1	-0.560031905	5.80730413	-7.230768512	5.53E-12	2.68E-11	16.432413
GEMIN2	-0.349360568	2.9254044	-7.229892572	5.56E-12	2.69E-11	16.42715846
CRYL1	-0.536375055	5.9575764	-7.226172874	5.69E-12	2.75E-11	16.40484965
TINCR	1.034268717	1.13537961	7.225134118	5.73E-12	2.77E-11	16.39862109
LRRC2	-0.917031076	4.03747404	-7.224406049	5.75E-12	2.78E-11	16.39425581
ST8SIA4	0.746453676	3.08326008	7.224374	5.75E-12	2.78E-11	16.39406366
ZNF768	-0.353128468	5.98631569	-7.223826579	5.77E-12	2.79E-11	16.3907817
ACTR6	-0.436983699	4.17788984	-7.223034542	5.80E-12	2.80E-11	16.38603347
YIPF6	-0.486208807	4.61935278	-7.22275781	5.81E-12	2.80E-11	16.38437455
SRRD	-0.534112572	3.64785513	-7.221706069	5.85E-12	2.82E-11	16.37807009
VCP	-0.445957689	7.45317572	-7.21891458	5.95E-12	2.87E-11	16.36134001
LRRC4B	-0.569245652	1.35450732	-7.218759685	5.95E-12	2.87E-11	16.36041181

KRT19	1.947011738	7.38285732	7.218008968	5.98E-12	2.88E-11	16.35591336
KLHDC7/	-0.721803797	2.84262308	-7.216722652	6.03E-12	2.90E-11	16.34820623
VPS54	-0.467466953	4.41891664	-7.216651626	6.03E-12	2.90E-11	16.34778069
NOSIP	-0.492352075	5.11563496	-7.215623172	6.07E-12	2.92E-11	16.34161926
TGIF2	-0.525759163	4.38613123	-7.214311526	6.12E-12	2.94E-11	16.33376209
FGFBP2	-0.492544635	0.74450956	-7.211986735	6.21E-12	2.99E-11	16.31983816
RPAP2	-0.399592918	2.16951531	-7.211402396	6.23E-12	3.00E-11	16.31633883
ZNF222	-0.397731188	2.41562303	-7.210610841	6.26E-12	3.01E-11	16.31159888
NRN1	-0.584729531	2.69021514	-7.210471931	6.26E-12	3.01E-11	16.3107671
HSPA9	-0.585061082	7.25584604	-7.21044118	6.26E-12	3.01E-11	16.31058297
CC2D2A	-0.563149356	2.8079583	-7.210369317	6.27E-12	3.01E-11	16.31015267
ZKSCAN	-0.400435855	2.23488226	-7.210282631	6.27E-12	3.01E-11	16.30963362
KHDC1	-0.542874308	2.03809622	-7.208968632	6.32E-12	3.04E-11	16.30176619
LIN7B	-0.526576864	3.16912734	-7.205984843	6.44E-12	3.09E-11	16.28390461
C12orf65	-0.338933884	3.5360212	-7.20585985	6.44E-12	3.09E-11	16.28315648
ZNF2	-0.375304475	2.42312482	-7.205818261	6.44E-12	3.09E-11	16.28290756
GTF2IRD	-0.414333581	2.2560851	-7.205609512	6.45E-12	3.09E-11	16.28165814
GOLGA7	-0.395655833	5.55702095	-7.204291801	6.50E-12	3.12E-11	16.27377188
MRTFA	-0.442392047	4.4982667	-7.204177043	6.51E-12	3.12E-11	16.27308511
DHRS11	-0.441593256	3.71045355	-7.203634063	6.53E-12	3.13E-11	16.26983579
PCID2	-0.4282025	4.08380807	-7.203233447	6.55E-12	3.14E-11	16.26743851
C7orf31	-0.39944989	2.74907646	-7.202388177	6.58E-12	3.15E-11	16.26238071
VPS51	-0.502101318	5.89735399	-7.201324138	6.62E-12	3.17E-11	16.25601444
MFAP3L	-0.692325011	1.2047305	-7.200357125	6.66E-12	3.19E-11	16.25022922
PRTFDC	-0.705764022	2.58380666	-7.200001528	6.68E-12	3.20E-11	16.24810197
HPD	-0.27707568	0.62755066	-7.199789311	6.69E-12	3.20E-11	16.24683248
MCEMP1	1.058673214	1.14903311	7.19927876	6.71E-12	3.21E-11	16.24377844
CLIC3	-1.014852652	8.28771002	-7.199026773	6.72E-12	3.21E-11	16.24227115
GRSF1	-0.413219886	5.26960149	-7.197389804	6.78E-12	3.24E-11	16.23248024
HNRNPL	-0.377682163	6.37226326	-7.196839311	6.81E-12	3.25E-11	16.22918802
TFCP2	-0.549070984	4.82099023	-7.194364401	6.91E-12	3.30E-11	16.21438884
C21orf59	-0.239563011	0.51059576	-7.192187575	7.00E-12	3.34E-11	16.2013749
ADPRH	-0.437826252	3.37739935	-7.190423657	7.08E-12	3.38E-11	16.1908314
CAPS2	-0.446198461	1.67961784	-7.190097331	7.09E-12	3.39E-11	16.18888104
SNRPF	-0.420358253	4.87753054	-7.189092916	7.14E-12	3.41E-11	16.18287828
KRT27	-1.073017296	1.99577775	-7.187786789	7.19E-12	3.43E-11	16.1750732
USP44	-0.347838281	0.83073592	-7.186927263	7.23E-12	3.45E-11	16.16993741
UTP6	-0.351020804	4.00394341	-7.186722015	7.24E-12	3.45E-11	16.16871109
BHLHB9	-0.483060855	2.45857412	-7.183221378	7.40E-12	3.53E-11	16.14779889
PDE1B	-0.629136547	1.69248863	-7.182534765	7.43E-12	3.54E-11	16.14369797

GPN2	-0.37002967	4.33075513	-7.180569805	7.52E-12	3.58E-11	16.13196335
ARHGEF	-0.646214475	2.58038137	-7.180361937	7.53E-12	3.58E-11	16.1307221
OSMR	0.906484727	3.60676385	7.180166661	7.53E-12	3.59E-11	16.12955606
HSD17B1	-0.519760612	2.94967839	-7.179128375	7.58E-12	3.61E-11	16.12335656
TBCD	-0.425162209	4.67114306	-7.178997049	7.59E-12	3.61E-11	16.12257248
SEMA3C	0.893171073	1.52352797	7.176723786	7.69E-12	3.66E-11	16.10900131
PSMC1	-0.505654959	2.90685677	-7.17669106	7.70E-12	3.66E-11	16.10880596
ERG	-0.622439658	2.82174298	-7.176405953	7.71E-12	3.67E-11	16.1071041
DET1	-0.403250576	2.47174009	-7.17587036	7.73E-12	3.68E-11	16.10390717
MYO1H	-0.204299656	0.35762887	-7.175405363	7.76E-12	3.69E-11	16.10113176
ZSWIM9	-0.448264195	3.82864419	-7.173125123	7.86E-12	3.74E-11	16.08752346
OGFOD2	-0.282229993	1.11572719	-7.17254095	7.89E-12	3.75E-11	16.08403763
PWWP3I	-1.204629122	2.26656535	-7.172331307	7.90E-12	3.75E-11	16.0827867
POLR2E	-0.411029141	6.39126023	-7.168132847	8.11E-12	3.85E-11	16.05773998
CFAP58	-0.329402829	0.81060869	-7.16808566	8.11E-12	3.85E-11	16.05745854
GNPAT	-0.331346408	5.47800233	-7.166495965	8.19E-12	3.88E-11	16.04797749
C12orf4	-0.365190718	3.29879467	-7.166142358	8.21E-12	3.89E-11	16.04586874
TM7SF2	-0.679066058	5.94537404	-7.165825094	8.22E-12	3.90E-11	16.04397679
TMEM18	-0.589210675	5.00116286	-7.165770904	8.22E-12	3.90E-11	16.04365364
CDC42EI	-0.629333356	4.70296345	-7.165381104	8.24E-12	3.91E-11	16.04132922
CFAP61	-0.165000874	0.2348601	-7.165014238	8.26E-12	3.91E-11	16.03914163
GPR153	0.823119454	2.1613626	7.164113363	8.31E-12	3.93E-11	16.0337701
RAN	-0.426565569	6.61088306	-7.163565301	8.34E-12	3.95E-11	16.03050247
MAEA	-0.381175139	4.48067368	-7.16037396	8.50E-12	4.02E-11	16.01147849
MGME1	-0.433905409	4.76207712	-7.160039605	8.52E-12	4.03E-11	16.00948569
ULK4	-0.417323758	2.12063621	-7.158812302	8.58E-12	4.06E-11	16.00217131
GTF2E1	-0.425915925	3.01132256	-7.156972006	8.68E-12	4.10E-11	15.99120523
GAP43	1.607619817	2.06734902	7.15626079	8.71E-12	4.12E-11	15.98696769
B4GALT3	-0.418356795	5.28733902	-7.155587331	8.75E-12	4.14E-11	15.98295537
SLC31A1	-0.592329543	4.16785335	-7.155094774	8.78E-12	4.15E-11	15.98002098
TCHP	-0.37896207	2.8297733	-7.152472226	8.92E-12	4.21E-11	15.96439953
LGALSL	-0.518705684	2.94826083	-7.152211453	8.93E-12	4.22E-11	15.96284642
TIMM8A	-0.344143163	2.79730385	-7.150145836	9.04E-12	4.27E-11	15.95054537
SCUBE3	-1.275082375	4.33072112	-7.149420867	9.08E-12	4.29E-11	15.94622863
EPPK1	0.762594428	1.49309802	7.149158824	9.10E-12	4.29E-11	15.9446684
DPH2	-0.390153831	3.96988792	-7.147244067	9.20E-12	4.34E-11	15.9332689
PSMG3	-0.441838679	5.08926357	-7.146754558	9.23E-12	4.35E-11	15.93035494
FAM189A	-0.9472736	5.84594784	-7.14649451	9.25E-12	4.36E-11	15.92880696
RHOD	-0.490742963	6.2689129	-7.142905163	9.45E-12	4.45E-11	15.90744477
PSMC6	-0.381492211	4.57045081	-7.139531934	9.65E-12	4.55E-11	15.88737533

TNFSF9	1.06194603	1.47006994	7.139482413	9.65E-12	4.55E-11	15.88708074
ZSCAN1ᵋ	-0.471721776	5.04798249	-7.138890273	9.68E-12	4.56E-11	15.8835584
ACBD5	-0.603102193	5.03217978	-7.138400762	9.71E-12	4.57E-11	15.88064669
SULT1C4	-0.639728383	1.38512318	-7.137250843	9.78E-12	4.60E-11	15.87380728
MMP11	1.049546099	2.3025196	7.137065116	9.79E-12	4.61E-11	15.87270269
CLUAP1	-0.394594228	3.80820735	-7.136972816	9.80E-12	4.61E-11	15.87215375
L3MBTL2	-0.404780587	4.11992643	-7.134712225	9.93E-12	4.67E-11	15.85871084
TUBD1	-0.48193989	3.24165989	-7.134696009	9.93E-12	4.67E-11	15.85861442
C2orf88	-0.590169136	1.68730023	-7.134051893	9.97E-12	4.69E-11	15.85478462
GTF2H1	-0.434275421	3.90428751	-7.131991966	1.01E-11	4.75E-11	15.84253821
WDR45	-0.37960685	5.35749486	-7.131738656	1.01E-11	4.75E-11	15.84103243
ACBD6	-0.304748364	3.77593138	-7.129279172	1.03E-11	4.82E-11	15.82641406
ARHGEF	-0.504831158	3.51942231	-7.128501718	1.03E-11	4.84E-11	15.82179383
POFUT2	-0.505167832	4.37266962	-7.128133118	1.03E-11	4.85E-11	15.81960344
TOE1	-0.462284362	4.41247074	-7.127945214	1.03E-11	4.86E-11	15.81848686
CREB3	-0.402692377	6.65574275	-7.126579749	1.04E-11	4.90E-11	15.81037345
PSTK	-0.267816665	1.60879254	-7.125125433	1.05E-11	4.94E-11	15.80173324
DISP2	-0.556251911	1.37634063	-7.122433198	1.07E-11	5.02E-11	15.78574155
OGFOD3	-0.429451143	4.79643567	-7.121296342	1.08E-11	5.05E-11	15.77898993
PDE5A	1.341784083	5.44177433	7.120105031	1.09E-11	5.09E-11	15.77191567
TOM1L2	-0.561959523	5.78895968	-7.119259691	1.09E-11	5.11E-11	15.76689634
AGPAT4	-0.541742019	1.31358084	-7.118628461	1.09E-11	5.13E-11	15.76314858
MAGI1	-0.579996288	3.85356097	-7.118279681	1.10E-11	5.14E-11	15.76107788
MAN1C1	-0.717880269	4.45500847	-7.11628493	1.11E-11	5.20E-11	15.74923643
NIF3L1	-0.408502651	4.77409367	-7.116134084	1.11E-11	5.20E-11	15.74834105
DUOX2	-1.439892739	6.86014999	-7.115269936	1.12E-11	5.23E-11	15.74321196
LMF2	-0.442452714	6.39391388	-7.112826762	1.13E-11	5.30E-11	15.72871293
ENO3	-1.108495636	2.81279171	-7.110373651	1.15E-11	5.38E-11	15.71415828
LRRC7	-0.432086016	0.63260432	-7.109665399	1.16E-11	5.40E-11	15.70995675
MAPK3	-0.376946707	5.94707444	-7.108847642	1.16E-11	5.43E-11	15.70510595
KTI12	-0.403218822	3.82671522	-7.108417886	1.16E-11	5.44E-11	15.70255686
CYP1B1	1.698490806	4.12899042	7.10818386	1.17E-11	5.45E-11	15.70116878
L1CAM	0.539304173	0.73216002	7.107455488	1.17E-11	5.47E-11	15.69684879
SMIM15	-0.435448918	5.90186945	-7.106928425	1.18E-11	5.49E-11	15.69372294
SNRNP4	-0.441930468	4.55445868	-7.106479772	1.18E-11	5.50E-11	15.69106225
BHLHE4(	0.993246654	7.53870827	7.106335843	1.18E-11	5.50E-11	15.69020871
ARFGAP	-0.378675066	5.7278435	-7.105301196	1.19E-11	5.54E-11	15.68407335
PIWIL2	-0.401943719	0.55633032	-7.104935276	1.19E-11	5.55E-11	15.68190362
MTHFSD	-0.355173217	3.0594982	-7.103225888	1.20E-11	5.60E-11	15.67176875
TADA2B	-0.382340746	4.63206049	-7.103180617	1.20E-11	5.60E-11	15.67150037

MRPL10	-0.367233121	5.73609455	-7.102747147	1.21E-11	5.62E-11	15.66893062
ZNF35	-0.333145419	2.65818978	-7.099596232	1.23E-11	5.72E-11	15.65025415
CNTNAP	-0.212795719	0.2511979	-7.09950734	1.23E-11	5.72E-11	15.64972733
TTF1	-0.442334684	4.08213157	-7.098188981	1.24E-11	5.77E-11	15.64191471
RDH13	-0.384438939	2.50768205	-7.095114494	1.26E-11	5.87E-11	15.62369903
SDK1	1.207921382	2.16501271	7.094100604	1.27E-11	5.91E-11	15.6176931
CCDC86	-0.447864926	4.69003577	-7.094060266	1.27E-11	5.91E-11	15.61745416
PRIMA1	-0.735302444	0.80804785	-7.093571004	1.27E-11	5.93E-11	15.61455616
TMEM24	-0.510509965	2.08484385	-7.093339625	1.28E-11	5.93E-11	15.61318571
IL1RAP	1.048175277	2.6753292	7.091528991	1.29E-11	6.00E-11	15.60246235
FBXO3	-0.447223292	3.61444956	-7.089284599	1.31E-11	6.08E-11	15.58917264
ODR4	-0.456575276	4.83241604	-7.088682193	1.31E-11	6.10E-11	15.5856061
CHMP6	-0.446707253	5.45791614	-7.088168059	1.32E-11	6.11E-11	15.58256233
GPI	-0.386545203	6.24998306	-7.087562593	1.32E-11	6.13E-11	15.57897805
DNTT	-0.43729892	0.32045811	-7.087348623	1.32E-11	6.14E-11	15.57771142
SLC25A2	-0.853979625	6.97949887	-7.085681944	1.34E-11	6.20E-11	15.56784615
MMP7	1.783317881	2.520041	7.085216477	1.34E-11	6.22E-11	15.56509127
ZNF671	-0.493303092	3.73708156	-7.085158515	1.34E-11	6.22E-11	15.56474823
WDR63	-0.455242489	1.0854877	-7.0849291	1.34E-11	6.22E-11	15.56339049
CTIF	-0.476151305	3.84354792	-7.084579019	1.35E-11	6.24E-11	15.56131866
TKT	-0.516833235	7.02261784	-7.083622458	1.35E-11	6.27E-11	15.55565797
FOXA2	-1.160266323	1.69354726	-7.083336226	1.36E-11	6.28E-11	15.55396421
SLC43A1	-0.860831447	3.07449971	-7.083069902	1.36E-11	6.29E-11	15.5523883
TAMM41	-0.311313341	2.23447911	-7.081014851	1.37E-11	6.36E-11	15.54022936
MRRF	-0.346254436	2.98771094	-7.080958224	1.37E-11	6.36E-11	15.53989436
ZDHHC1	-0.539918977	4.5465243	-7.080933959	1.38E-11	6.36E-11	15.5397508
OCA2	-1.070870875	1.34934324	-7.078984337	1.39E-11	6.44E-11	15.52821793
CHMP7	-0.354707271	4.90825253	-7.077847759	1.40E-11	6.48E-11	15.52149555
IRAK1	-0.577844895	4.87154643	-7.077129488	1.41E-11	6.51E-11	15.51724766
METTL2A	-0.392312534	3.41839243	-7.076383007	1.41E-11	6.53E-11	15.51283323
MORF4L	-0.44872652	7.75070797	-7.076278905	1.41E-11	6.54E-11	15.51221763
MOB4	-0.447507165	4.2312008	-7.075610409	1.42E-11	6.56E-11	15.50826469
TNN	-0.79673816	1.03127208	-7.075215301	1.42E-11	6.57E-11	15.50592846
ISY1	-0.407243903	4.1261892	-7.074252796	1.43E-11	6.61E-11	15.50023764
TRMU	-0.435490568	2.96101258	-7.07334222	1.44E-11	6.65E-11	15.49485432
USP5	-0.456322277	6.29882618	-7.073272834	1.44E-11	6.65E-11	15.49444413
ADGRF4	0.659968858	0.58143165	7.07258831	1.45E-11	6.67E-11	15.49039757
GABRD	-1.000827982	3.31236874	-7.070547027	1.46E-11	6.75E-11	15.47833209
CST5	1.138984973	1.37293173	7.069922783	1.47E-11	6.78E-11	15.47464282
EFCAB6	-0.413503649	1.53840644	-7.068994179	1.48E-11	6.81E-11	15.46915519

KCNIP4	-0.623571301	1.41962055	-7.067213898	1.49E-11	6.88E-11	15.45863588
RNF225	0.653885251	0.88038544	7.064469431	1.52E-11	7.00E-11	15.44242289
PELI1	1.039674372	4.24627583	7.06400427	1.52E-11	7.01E-11	15.43967537
PPP1R1(	-0.519860615	4.2271631	-7.0636289	1.53E-11	7.03E-11	15.43745829
MICU2	-0.453679664	5.51718101	-7.059934935	1.56E-11	7.19E-11	15.41564456
ISG20L2	-0.434403181	4.09192235	-7.059786891	1.56E-11	7.19E-11	15.41477049
FAM20A	1.482559724	4.62367996	7.058685251	1.57E-11	7.24E-11	15.40826662
TMEM16	-0.500509543	3.79084356	-7.053654236	1.62E-11	7.46E-11	15.37857318
SUGT1	-0.353448636	3.48249723	-7.051423074	1.64E-11	7.55E-11	15.36540926
ZSCAN2:	-0.38250803	3.03819493	-7.049704241	1.66E-11	7.63E-11	15.35527
TCF25	-0.429430006	6.0100992	-7.048581751	1.67E-11	7.68E-11	15.34864942
EPHB3	1.213676273	3.25915176	7.0468396	1.69E-11	7.76E-11	15.33837541
CHAC2	-0.415410366	1.79865996	-7.045998166	1.70E-11	7.80E-11	15.33341383
GALNT1:	-0.485761756	4.78715573	-7.045820381	1.70E-11	7.80E-11	15.33236556
MRC1	1.0568612	2.86776717	7.045263779	1.70E-11	7.83E-11	15.32908378
SLC9A1	-0.488781586	5.00020284	-7.0443635	1.71E-11	7.87E-11	15.32377603
PHLPP1	-0.515610555	3.15973998	-7.043897636	1.72E-11	7.89E-11	15.32102962
GRASP	-0.702566582	3.52518398	-7.043629957	1.72E-11	7.90E-11	15.31945163
CPEB3	-0.545268963	2.68922346	-7.043590015	1.72E-11	7.90E-11	15.31921617
LSM14B	-0.430116451	5.25830561	-7.042576677	1.73E-11	7.94E-11	15.31324285
C4orf19	-0.516362068	0.97467367	-7.042046015	1.74E-11	7.97E-11	15.31011498
TSTD1	-0.534476533	8.0532765	-7.040579748	1.75E-11	8.04E-11	15.30147324
DMAP1	-0.438660962	4.68804498	-7.039979814	1.76E-11	8.06E-11	15.29793776
WIPI2	-0.339143056	4.89997177	-7.039791416	1.76E-11	8.07E-11	15.29682755
GIPC3	-0.70058393	2.67807475	-7.039332732	1.77E-11	8.09E-11	15.29412465
GPATCH	-0.552785746	3.93377233	-7.03899763	1.77E-11	8.10E-11	15.29215006
XPNPEP	-0.397472164	2.44292646	-7.036174246	1.80E-11	8.24E-11	15.27551582
PI4KB	-0.438685566	5.79892216	-7.035519073	1.81E-11	8.27E-11	15.27165645
GOLGA7	0.887585457	2.06229502	7.034231134	1.82E-11	8.33E-11	15.26407039
ASB3	-0.341355205	1.5933411	-7.033078331	1.83E-11	8.39E-11	15.2572811
PARM1	-0.75972741	7.31536651	-7.032265092	1.84E-11	8.43E-11	15.25249208
TLE5	-0.394252174	8.50425138	-7.030709412	1.86E-11	8.50E-11	15.24333199
TRIM46	0.54000768	0.74669716	7.030478575	1.86E-11	8.51E-11	15.24197291
IL1RL1	1.846498497	2.36545732	7.027806641	1.89E-11	8.65E-11	15.22624368
ZNF559	-0.490462225	3.29974668	-7.027166575	1.90E-11	8.68E-11	15.22247633
ADCY9	-0.632653246	4.76892543	-7.026782077	1.91E-11	8.70E-11	15.22021333
ZNF469	0.399704539	0.61386824	7.026617857	1.91E-11	8.70E-11	15.21924681
LEMD2	-0.373995354	4.62955052	-7.026579699	1.91E-11	8.70E-11	15.21902224
DNAAF2	-0.440125816	3.86079337	-7.025763052	1.92E-11	8.74E-11	15.21421615
TMEM10	-0.449669318	4.17617354	-7.024508422	1.93E-11	8.81E-11	15.2068332

OR2T33	-0.192387331	0.19291777	-7.024102695	1.94E-11	8.83E-11	15.20444587
ALG2	-0.469985028	5.5586953	-7.02365902	1.94E-11	8.85E-11	15.20183535
APBA2	-0.766455471	1.65136448	-7.023488591	1.94E-11	8.85E-11	15.2008326
QTRT1	-0.51946173	5.14225749	-7.023369219	1.94E-11	8.86E-11	15.20013026
GLI4	-0.510140439	3.30438941	-7.022258333	1.96E-11	8.92E-11	15.19359464
CD70	1.077768821	1.34357398	7.02190126	1.96E-11	8.93E-11	15.19149404
ACADVL	-0.487097003	7.87318658	-7.02133125	1.97E-11	8.96E-11	15.18814092
FGF2	0.678630134	2.35658131	7.021030851	1.97E-11	8.97E-11	15.18637387
GNG10	-0.562421627	3.9851303	-7.019520763	1.99E-11	9.05E-11	15.17749181
TMED5	-0.580813081	4.61546204	-7.018716117	2.00E-11	9.09E-11	15.17275957
VAMP2	-0.456412884	6.71109264	-7.018615031	2.00E-11	9.10E-11	15.17216509
CLDN5	-0.918177852	4.22834965	-7.018463677	2.00E-11	9.10E-11	15.171275
C16orf96	-0.281966092	0.54537431	-7.018341773	2.00E-11	9.10E-11	15.17055812
RNF215	-0.410541303	2.74887992	-7.018306683	2.00E-11	9.10E-11	15.17035176
ACAT2	-0.38785332	3.0641151	-7.018306146	2.00E-11	9.10E-11	15.1703486
THAP7	-0.417345158	5.16015978	-7.018025687	2.01E-11	9.12E-11	15.16869933
ZFP62	-0.472906482	4.30671528	-7.017125661	2.02E-11	9.16E-11	15.16340693
MARCKS	-0.675427418	9.07064201	-7.016897604	2.02E-11	9.17E-11	15.16206596
SEPTIN4	-0.486652838	1.68949561	-7.01664998	2.02E-11	9.19E-11	15.16060997
C4orf3	-0.499430677	7.17052761	-7.016276447	2.03E-11	9.20E-11	15.15841372
PHYH	-0.608377838	5.6555333	-7.015820728	2.03E-11	9.23E-11	15.15573436
CFAP52	-0.228718436	0.38770817	-7.014048838	2.06E-11	9.32E-11	15.1453178
RAB29	-0.653844047	5.16429598	-7.014019942	2.06E-11	9.32E-11	15.14514794
E2F4	-0.357575173	5.43736318	-7.013669989	2.06E-11	9.34E-11	15.14309086
FRMPD3	-0.86111575	1.9367535	-7.013535244	2.06E-11	9.34E-11	15.14229882
SLC6A14	0.929217349	1.00950413	7.012769765	2.07E-11	9.38E-11	15.13779951
NCAM1	-1.289640289	3.09440613	-7.012603793	2.07E-11	9.39E-11	15.136824
IK	-0.384044824	6.615169	-7.012432181	2.08E-11	9.40E-11	15.13581537
CNTN3	-0.636305601	1.05223515	-7.011370946	2.09E-11	9.46E-11	15.12957842
GABRG1	-0.294654385	0.28176064	-7.011068657	2.09E-11	9.47E-11	15.12780196
TRAPPC	-0.671845706	0.62562064	-7.01086471	2.10E-11	9.48E-11	15.12660346
SLA	-0.873177038	3.39891509	-7.010782199	2.10E-11	9.48E-11	15.12611858
ARMC2	-0.330255031	1.54667408	-7.006645817	2.15E-11	9.72E-11	15.10181627
FBXO42	-0.37621848	3.62779376	-7.006587	2.15E-11	9.72E-11	15.10147077
MAMLD1	1.025073492	2.8531734	7.00568886	2.16E-11	9.77E-11	15.09619528
JAM3	-0.599823573	3.46997837	-7.004214175	2.18E-11	9.85E-11	15.08753426
DDX25	-0.368345821	0.55502564	-7.004168109	2.18E-11	9.85E-11	15.08726374
UMAD1	-0.514390356	4.58209044	-7.004160872	2.18E-11	9.85E-11	15.08722124
CDC23	-0.438668939	4.31966896	-7.002643066	2.20E-11	9.94E-11	15.0783083
TUBGCP	-0.364096158	5.05530911	-7.001758839	2.21E-11	9.99E-11	15.07311651

RNF26	-0.455652438	5.3089941	-7.001164172	2.22E-11	1.00E-10	15.06962513
TIAM2	-0.568661131	1.92733375	-7.001110992	2.22E-11	1.00E-10	15.06931292
SCRN3	-0.549380676	3.655964	-7.000890469	2.23E-11	1.00E-10	15.06801825
PTRH1	-0.123450521	0.24810669	-6.999547067	2.24E-11	1.01E-10	15.06013189
KBTBD1:	-0.554854902	1.78988983	-6.999263323	2.25E-11	1.01E-10	15.05846632
YARS2	-0.332418635	4.07496938	-6.998413972	2.26E-11	1.02E-10	15.05348093
SRARP	-0.430315285	0.30542189	-6.997769195	2.27E-11	1.02E-10	15.04969658
TRAPPC	-0.40640406	7.76810347	-6.997697539	2.27E-11	1.02E-10	15.04927603
PAPPA	0.623583178	1.04353574	6.997308487	2.27E-11	1.02E-10	15.04699272
PROS1	1.441337331	6.67154085	6.995680481	2.30E-11	1.03E-10	15.03743906
VDAC1	-0.383627447	6.94821536	-6.994790282	2.31E-11	1.04E-10	15.03221572
WDR53	-0.306590449	3.61989767	-6.994621149	2.31E-11	1.04E-10	15.03122337
PAXX	-0.622032991	6.20181125	-6.994298139	2.31E-11	1.04E-10	15.02932822
GALNT5	0.733970019	0.97248541	6.993870475	2.32E-11	1.04E-10	15.02681913
RPS6KB:	-0.402811758	4.23155406	-6.990812378	2.36E-11	1.06E-10	15.00888045
CIITA	0.842430588	2.20459425	6.989646736	2.38E-11	1.07E-10	15.00204425
TMC8	1.026324162	3.43700121	6.988767028	2.39E-11	1.07E-10	14.99688548
CDK5RA	-0.301494182	4.27088938	-6.988112735	2.40E-11	1.08E-10	14.99304888
SDC2	-0.847646003	7.85340801	-6.98702588	2.42E-11	1.09E-10	14.98667638
CCL17	1.517983706	2.52720647	6.986801152	2.42E-11	1.09E-10	14.98535883
ELOA	-0.434874656	4.97571664	-6.986654717	2.42E-11	1.09E-10	14.98450031
SUMO2	-0.37282166	7.07192582	-6.986632526	2.42E-11	1.09E-10	14.98437021
TM6SF1	-0.765860084	2.76762005	-6.986561851	2.42E-11	1.09E-10	14.98395587
ITGA3	0.883113715	7.56856187	6.985560594	2.44E-11	1.09E-10	14.97808611
NPHP1	-0.384569072	2.04373969	-6.985296627	2.44E-11	1.09E-10	14.97653873
CLPP	-0.384067488	5.77363287	-6.981376242	2.50E-11	1.12E-10	14.953562
GNPDA2	-0.416863955	3.1947563	-6.981269796	2.50E-11	1.12E-10	14.95293826
KPNA5	-0.476129583	3.46978629	-6.980197228	2.52E-11	1.13E-10	14.9466537
NT5C3A	-0.403912689	4.71540133	-6.978710123	2.54E-11	1.14E-10	14.93794131
CCL14	-0.542080733	1.06633931	-6.978588878	2.54E-11	1.14E-10	14.93723104
CECR2	-0.400472682	0.4588423	-6.978086323	2.55E-11	1.14E-10	14.93428708
TNFAIP2	0.834260519	4.78391522	6.977710041	2.56E-11	1.14E-10	14.93208292
ENKD1	-0.583329807	4.60177998	-6.976458277	2.58E-11	1.15E-10	14.924751
CA12	-1.640180534	4.05756844	-6.97615148	2.58E-11	1.15E-10	14.92295414
MED18	-0.356259104	4.42821401	-6.975779894	2.59E-11	1.16E-10	14.9207779
WDFY4	0.645386056	1.32790305	6.975603829	2.59E-11	1.16E-10	14.91974677
TBX2	-0.958333501	3.23357279	-6.97434217	2.61E-11	1.17E-10	14.91235841
ICAM5	1.278615992	3.11608497	6.974027832	2.61E-11	1.17E-10	14.91051777
ZNF3	-0.36337745	4.14127682	-6.973397635	2.62E-11	1.17E-10	14.90682774
ATXN7L3	-0.516559193	5.70585004	-6.972831367	2.63E-11	1.18E-10	14.90351224

C6orf47	-0.348799808	5.46935719	-6.971505406	2.65E-11	1.18E-10	14.89574944
DCDC1	-0.1787354	0.30068188	-6.970904184	2.66E-11	1.19E-10	14.89222994
NOD2	0.482676881	0.81009643	6.97041393	2.67E-11	1.19E-10	14.88936018
KAT5	-0.542444129	5.13538257	-6.96872954	2.70E-11	1.20E-10	14.87950147
HSD11B1	-0.432160481	3.54632837	-6.968221722	2.71E-11	1.21E-10	14.87652953
DST	0.827108423	2.61169635	6.966710554	2.73E-11	1.22E-10	14.8676865
EIF3I	-0.308180885	8.57203889	-6.96647162	2.73E-11	1.22E-10	14.86628843
TRMT9B	-0.767249123	3.30404798	-6.966231443	2.74E-11	1.22E-10	14.86488312
DXO	-0.400102807	4.56994422	-6.965882269	2.74E-11	1.22E-10	14.86284011
ZBTB2	-0.60094206	5.31041061	-6.965272504	2.75E-11	1.23E-10	14.85927255
RPL22	-0.412645489	7.63282527	-6.964201206	2.77E-11	1.23E-10	14.85300521
FAM166B	-0.353907086	0.85854061	-6.963995092	2.77E-11	1.23E-10	14.85179948
TNFAIP8	-0.575112309	2.74480765	-6.962847683	2.79E-11	1.24E-10	14.84508774
AKR1C3	-0.944953123	2.12843995	-6.962783266	2.79E-11	1.24E-10	14.84471096
NUMB	-0.445448664	4.28934679	-6.961596704	2.81E-11	1.25E-10	14.83777103
RBM42	-0.432069234	6.76831959	-6.960708088	2.83E-11	1.26E-10	14.83257424
DCAF17	-0.560052999	3.09083458	-6.959711079	2.85E-11	1.27E-10	14.82674409
ALDH6A1	-0.648227923	4.21750231	-6.958241556	2.87E-11	1.28E-10	14.81815189
STX10	-0.411805094	5.57981747	-6.957832803	2.88E-11	1.28E-10	14.81576215
PINK1	-0.646758514	4.75924244	-6.95745863	2.88E-11	1.28E-10	14.81357467
ZDHHC1	-0.482062101	1.09205482	-6.956165315	2.91E-11	1.29E-10	14.80601435
PAPSS2	-0.938944946	2.84883936	-6.95550733	2.92E-11	1.30E-10	14.80216833
RMDN3	-0.41562982	5.09313825	-6.952327883	2.97E-11	1.32E-10	14.78358751
C5orf49	-0.544844698	0.97138924	-6.951831057	2.98E-11	1.32E-10	14.78068457
RABL6	-0.34598928	4.99955046	-6.951139761	3.00E-11	1.33E-10	14.77664557
ADCK2	-0.445628805	5.22838847	-6.950654506	3.00E-11	1.33E-10	14.77381056
RXRA	-0.438861627	5.00071616	-6.950127528	3.01E-11	1.34E-10	14.77073194
NIP7	-0.377921368	4.22053162	-6.949321566	3.03E-11	1.34E-10	14.7660238
SDR42E1	-0.407645527	1.80475263	-6.949172967	3.03E-11	1.34E-10	14.76515578
RNPEP	-0.405808971	6.46133613	-6.949006335	3.03E-11	1.34E-10	14.76418243
PTCD3	-0.383078537	3.83958094	-6.948593473	3.04E-11	1.35E-10	14.76177086
GOLGA8	-0.580995769	0.88136685	-6.948551481	3.04E-11	1.35E-10	14.76152559
TMEM17	-0.780385212	2.40653251	-6.947302288	3.07E-11	1.36E-10	14.75422954
JOSD2	-0.549318674	5.37292971	-6.946580315	3.08E-11	1.36E-10	14.75001318
RANBP1	-0.406317966	4.79015295	-6.946414957	3.08E-11	1.36E-10	14.74904752
TCEAL7	-0.627226183	1.89828235	-6.946234204	3.08E-11	1.36E-10	14.74799199
SCTR	-0.519815583	1.04480126	-6.944858799	3.11E-11	1.38E-10	14.73996065
GCFC2	-0.448264327	3.6225008	-6.94326009	3.14E-11	1.39E-10	14.73062676
TMEM12	-0.466322429	5.69540876	-6.943073343	3.14E-11	1.39E-10	14.72953655
TMEM16	1.390173125	2.57029824	6.94300109	3.14E-11	1.39E-10	14.72911475

RNF185	-0.548292256	5.73097657	-6.942960759	3.15E-11	1.39E-10	14.72887931
HMGN2	-0.524301873	8.0615212	-6.942188567	3.16E-11	1.40E-10	14.72437161
CCNO	-0.51306407	4.36017997	-6.939999056	3.20E-11	1.41E-10	14.7115921
MINPP1	-0.500097478	4.38857099	-6.937410986	3.25E-11	1.43E-10	14.69648987
PDZD11	-0.311777452	6.46194964	-6.936998298	3.26E-11	1.44E-10	14.69408206
MXD4	-0.508101511	5.80295854	-6.936068967	3.28E-11	1.45E-10	14.68866027
CATSPE	0.917696595	1.58405492	6.935708858	3.28E-11	1.45E-10	14.6865595
COP1	-0.364353049	4.89426729	-6.934783754	3.30E-11	1.46E-10	14.68116305
RALGPS	-0.513389465	2.77282656	-6.932020146	3.36E-11	1.48E-10	14.66504491
ENTR1	-0.380973833	4.59192446	-6.931997442	3.36E-11	1.48E-10	14.66491251
EIF1AX	-0.46473665	5.65795456	-6.931580233	3.37E-11	1.48E-10	14.66247962
PACSIN2	-0.526793864	6.04042191	-6.930659901	3.38E-11	1.49E-10	14.65711318
LTV1	-0.410400651	4.80922291	-6.927632227	3.45E-11	1.52E-10	14.63946234
GALNS	-0.383041013	3.26113628	-6.926399367	3.47E-11	1.53E-10	14.63227647
GVQW2	-0.241755932	0.62705436	-6.92496521	3.50E-11	1.54E-10	14.62391843
FAM49A	-0.593415272	5.74684995	-6.924046352	3.52E-11	1.55E-10	14.61856409
RAPGEF	-0.674915786	2.29699206	-6.923286815	3.54E-11	1.56E-10	14.6141385
ZNF350	-0.413695839	3.21917056	-6.922593983	3.55E-11	1.56E-10	14.61010187
ID1	-1.034099888	8.2831497	-6.922123871	3.56E-11	1.57E-10	14.60736303
TSEN2	-0.351450771	2.66978047	-6.921741524	3.57E-11	1.57E-10	14.6051356
NARS	-0.454765815	6.99718494	-6.920735397	3.59E-11	1.58E-10	14.59927461
PICK1	-0.391102961	4.40021138	-6.919788867	3.61E-11	1.59E-10	14.59376133
PSMD4	-0.362626155	6.92075402	-6.919315558	3.62E-11	1.59E-10	14.59100463
PAK4	-0.444782387	4.8878022	-6.919272722	3.62E-11	1.59E-10	14.59075515
BANK1	-0.718018436	3.48324684	-6.918440812	3.64E-11	1.60E-10	14.58591016
KCMF1	-0.3669383	4.55785209	-6.91809982	3.65E-11	1.60E-10	14.58392437
TRIP6	-0.485375966	6.71573972	-6.917353523	3.66E-11	1.61E-10	14.57957848
TRAPPC	-0.391416151	4.58165687	-6.917171853	3.67E-11	1.61E-10	14.57852062
RYR2	-0.701428489	0.8323766	-6.917107384	3.67E-11	1.61E-10	14.57814522
NUP62CI	-0.576891032	2.73805423	-6.914619626	3.72E-11	1.63E-10	14.563661
FAP	0.836416961	1.39798025	6.913673474	3.74E-11	1.64E-10	14.55815325
PRR36	0.940926684	3.58096367	6.913218394	3.75E-11	1.65E-10	14.55550431
PGM5	-0.606803698	1.78581752	-6.913145001	3.76E-11	1.65E-10	14.55507712
PARP16	-0.352495143	3.55911041	-6.911230087	3.80E-11	1.66E-10	14.54393216
CHMP1A	-0.368753468	6.68479431	-6.910259302	3.82E-11	1.67E-10	14.53828292
SRPRA	-0.418337604	7.11247198	-6.909220011	3.84E-11	1.68E-10	14.53223563
TRIR	-0.454199488	7.75512439	-6.908734833	3.86E-11	1.69E-10	14.52941275
ZNF181	-0.424733196	2.7253708	-6.907103968	3.89E-11	1.70E-10	14.51992499
RNF11	-0.526330105	7.14447008	-6.904857019	3.95E-11	1.73E-10	14.50685561
DRG2	-0.368973967	3.72823405	-6.904702336	3.95E-11	1.73E-10	14.505956

DAZAP2	-0.465433879	7.29398362	-6.902806463	3.99E-11	1.75E-10	14.49493108
BAIAP2L	-0.4007578	4.64896382	-6.902767703	3.99E-11	1.75E-10	14.4947057
C11orf54	-0.539845367	4.17426446	-6.902036647	4.01E-11	1.75E-10	14.49045502
RAB5C	-0.47889998	7.02834161	-6.901545301	4.02E-11	1.76E-10	14.48759829
CNOT11	-0.369200781	5.19658661	-6.901001955	4.04E-11	1.76E-10	14.4844394
CXCL5	0.698944227	0.61164717	6.900645479	4.05E-11	1.77E-10	14.48236701
METTL6	-0.312923265	2.55544441	-6.900194475	4.06E-11	1.77E-10	14.47974519
ALG6	-0.330935165	2.79686745	-6.898377058	4.10E-11	1.79E-10	14.46918119
ASAH1	-0.666819734	8.22136491	-6.898244771	4.10E-11	1.79E-10	14.46841233
SLC35E3	-0.272133402	1.44034002	-6.897187588	4.13E-11	1.80E-10	14.46226826
APOLD1	-1.097614417	5.07688797	-6.896429687	4.15E-11	1.81E-10	14.45786394
S100A10	1.22569728	7.23584182	6.894637692	4.19E-11	1.83E-10	14.4474516
FER1L5	-0.134032345	0.16125989	-6.892806498	4.24E-11	1.85E-10	14.43681341
ZNF19	-0.333454068	1.92440869	-6.892349674	4.25E-11	1.85E-10	14.43415982
KITLG	-0.670281923	2.41584745	-6.889924026	4.31E-11	1.88E-10	14.42007181
ARFRP1	-0.39258851	4.15087604	-6.889758529	4.32E-11	1.88E-10	14.41911074
PTMS	-0.541273961	9.39349365	-6.889600994	4.32E-11	1.88E-10	14.41819593
YIPF4	-0.415845142	4.10539124	-6.888863143	4.34E-11	1.89E-10	14.41391134
CKAP4	-0.480553407	5.92974689	-6.888651204	4.34E-11	1.89E-10	14.41268071
PODXL	-0.753335029	5.79801997	-6.886218746	4.41E-11	1.92E-10	14.39855836
ZNF442	-0.314236923	1.43133196	-6.886096269	4.41E-11	1.92E-10	14.39784737
ANKRD4	-0.436651785	3.51690151	-6.884101521	4.46E-11	1.94E-10	14.38626897
TIPRL	-0.379082581	5.1042689	-6.883657032	4.47E-11	1.94E-10	14.38368927
ZNF541	-0.3328227	0.54384287	-6.883280927	4.48E-11	1.95E-10	14.38150654
MRPS6	-0.509481659	6.06984334	-6.883050417	4.49E-11	1.95E-10	14.38016882
SELENO	-0.544663396	6.93415367	-6.882306176	4.51E-11	1.96E-10	14.37584996
SGF29	-0.433874441	5.00971462	-6.881906517	4.52E-11	1.96E-10	14.37353085
LRFN1	-0.524428251	1.02130436	-6.881422907	4.53E-11	1.97E-10	14.37072472
ARHGEF	-0.786541807	3.29398304	-6.879380341	4.59E-11	1.99E-10	14.35887432
CKMT1B	-0.558777985	1.34489957	-6.876417432	4.67E-11	2.03E-10	14.34168863
SLC25A1	-0.349677619	2.89347094	-6.874465724	4.72E-11	2.05E-10	14.33037096
PLPBP	-0.413903266	5.85046368	-6.874416759	4.73E-11	2.05E-10	14.33008705
IGDCC4	-0.989094876	3.12728449	-6.872893244	4.77E-11	2.07E-10	14.32125399
ATP6V1C	-0.385753077	8.30115218	-6.872599138	4.78E-11	2.07E-10	14.31954897
MGMT	-0.439771486	4.85037916	-6.872434154	4.78E-11	2.07E-10	14.31859253
AXIN2	-0.778317304	2.99031739	-6.871266614	4.82E-11	2.09E-10	14.31182457
FCF1	-0.537881461	4.86105088	-6.869157623	4.88E-11	2.11E-10	14.29960124
ELF4	0.637114712	3.28665539	6.867938119	4.91E-11	2.13E-10	14.29253438
FRY	-0.490671065	1.99665581	-6.867892394	4.91E-11	2.13E-10	14.29226943
TOM1L1	-0.64488548	3.90616545	-6.867734713	4.92E-11	2.13E-10	14.29135576

CCPG1	-0.512225151	3.70772928	-6.86514961	4.99E-11	2.16E-10	14.27637861
CEP85	-0.540137204	2.83898201	-6.863460308	5.04E-11	2.18E-10	14.26659351
FAXC	0.710998396	2.01225344	6.862223997	5.08E-11	2.20E-10	14.25943336
FAM181E	-0.614226744	0.93619271	-6.861446437	5.10E-11	2.21E-10	14.25493054
NMB	-1.38928291	6.58138937	-6.860139116	5.14E-11	2.22E-10	14.24736069
BBIP1	-0.418583605	2.97055357	-6.859755433	5.15E-11	2.23E-10	14.24513921
ORMDL2	-0.381291964	4.75875216	-6.859564794	5.16E-11	2.23E-10	14.24403547
CEP57L1	-0.270118319	1.89463509	-6.858361571	5.20E-11	2.24E-10	14.23706966
FAM49B	-0.431074457	3.75068038	-6.857909882	5.21E-11	2.25E-10	14.23445492
GNG5	-0.368356479	7.99360464	-6.856231875	5.26E-11	2.27E-10	14.22474229
HIST1H3	-0.671036731	1.76374455	-6.855841862	5.28E-11	2.28E-10	14.22248506
MESP1	-0.629679747	1.81101271	-6.853946901	5.33E-11	2.30E-10	14.21151905
VEGFD	-0.200164599	0.27930299	-6.85355577	5.35E-11	2.31E-10	14.20925586
GSK3A	-0.286039017	5.60950408	-6.85350315	5.35E-11	2.31E-10	14.2089514
SUMO3	-0.395901962	6.60527253	-6.85201855	5.40E-11	2.33E-10	14.20036197
HEMGN	-0.224679918	0.17421414	-6.851650324	5.41E-11	2.33E-10	14.19823172
SH3RF3	-0.622405939	2.12603594	-6.850266414	5.45E-11	2.35E-10	14.1902263
CCDC9B	-0.71398421	3.79503665	-6.850241455	5.45E-11	2.35E-10	14.19008193
MEST	-0.474044091	3.90988833	-6.850054697	5.46E-11	2.35E-10	14.1890017
ZBTB48	-0.441218466	4.20538799	-6.847876838	5.53E-11	2.38E-10	14.17640609
NFE2L3	1.318737192	3.90637507	6.847854006	5.53E-11	2.38E-10	14.17627406
FBXL3	-0.614961974	4.81935564	-6.847603412	5.54E-11	2.38E-10	14.17482493
ARMCX1	-0.60886829	4.98850433	-6.847540048	5.54E-11	2.38E-10	14.17445852
PDIK1L	-0.547381234	4.50345079	-6.845834521	5.60E-11	2.41E-10	14.1645969
KCNK17	-1.237760271	1.89167291	-6.84573144	5.60E-11	2.41E-10	14.16400092
MTERF2	-0.486130981	4.00079008	-6.845228568	5.62E-11	2.41E-10	14.16109359
CLUH	-0.410425212	4.60305369	-6.844312643	5.65E-11	2.43E-10	14.15579859
LIN9	-0.411555575	1.87046956	-6.843783128	5.66E-11	2.43E-10	14.15273767
CDK8	-0.351083415	3.26134019	-6.84086806	5.76E-11	2.48E-10	14.1358897
SPPL3	-0.38679895	5.25884355	-6.840386357	5.78E-11	2.48E-10	14.13310611
ZNF425	-0.463612084	2.3751748	-6.840309813	5.78E-11	2.48E-10	14.13266381
GLOD4	-0.380460659	5.27063665	-6.840244268	5.78E-11	2.48E-10	14.13228506
ZNF138	-0.436785597	3.24643477	-6.839453836	5.81E-11	2.49E-10	14.12771781
ZNF14	-0.452816476	3.63663715	-6.839367785	5.81E-11	2.49E-10	14.12722062
PPP1R7	-0.62996675	6.13725398	-6.838907937	5.83E-11	2.50E-10	14.12456373
PPIL2	-0.475509467	4.56658436	-6.838529442	5.84E-11	2.51E-10	14.12237697
CCDC61	-0.440947653	4.20587181	-6.837131227	5.89E-11	2.53E-10	14.11429948
ARSI	0.630798017	0.70597156	6.836200441	5.92E-11	2.54E-10	14.10892297
CLTB	-0.410430612	6.91814067	-6.835160629	5.96E-11	2.55E-10	14.10291728
JDP2	-0.562098145	3.16026885	-6.833671688	6.01E-11	2.58E-10	14.09431864

DDIT3	-0.823063178	7.09662936	-6.833221516	6.03E-11	2.58E-10	14.09171914
MAGI2	-0.485966554	2.41735511	-6.833122995	6.03E-11	2.58E-10	14.09115026
RAB41	-0.367641161	1.51535652	-6.832581183	6.05E-11	2.59E-10	14.08802179
RAB9A	-0.40730145	5.02268026	-6.832549552	6.05E-11	2.59E-10	14.08783915
NUP42	-0.38171005	3.88629177	-6.832133934	6.07E-11	2.60E-10	14.08543946
UNC45A	-0.375619371	5.18883507	-6.830738347	6.12E-11	2.62E-10	14.07738236
CLCN4	-0.592820373	3.48475462	-6.830282545	6.13E-11	2.62E-10	14.07475114
ANO2	-0.325855022	0.6174125	-6.829930976	6.15E-11	2.63E-10	14.07272171
RDH12	-0.696293337	1.27837469	-6.829448136	6.16E-11	2.64E-10	14.06993464
SDR39U1	-0.470360059	3.42407117	-6.826565895	6.27E-11	2.68E-10	14.05330046
TCAF1	-0.514372447	3.51714	-6.825639808	6.30E-11	2.69E-10	14.04795679
ASCC2	-0.338532403	5.49108471	-6.823671435	6.38E-11	2.72E-10	14.03660064
TMEM22	-0.431119871	5.40940113	-6.823232819	6.39E-11	2.73E-10	14.03407044
HIKESHI	-0.332220599	4.07184649	-6.822652003	6.42E-11	2.74E-10	14.03072011
HSCB	-0.42764698	4.71897024	-6.821011739	6.48E-11	2.77E-10	14.02125963
NOL11	-0.393353173	4.49088229	-6.8195983	6.53E-11	2.79E-10	14.01310865
ABRAXA	-0.407398419	4.48762243	-6.819594432	6.53E-11	2.79E-10	14.01308635
PLAC9	-0.920415029	3.61117962	-6.818519583	6.57E-11	2.80E-10	14.00688872
NACAD	0.566368618	1.27060216	6.817944194	6.60E-11	2.81E-10	14.00357129
TMEM16	-0.459693154	4.78191728	-6.81676615	6.64E-11	2.83E-10	13.99677982
CLEC2D	0.559929738	2.00616597	6.816334147	6.66E-11	2.84E-10	13.9942895
DHFR2	-0.487448759	3.11633915	-6.815935988	6.68E-11	2.84E-10	13.99199438
INKA2	-0.396233772	1.7448753	-6.813125914	6.79E-11	2.89E-10	13.97579882
COX7A1	-1.236642926	5.3836037	-6.812142301	6.83E-11	2.91E-10	13.97013097
ORC4	-0.38934215	3.49326371	-6.812058253	6.83E-11	2.91E-10	13.96964668
SSPO	-0.405836654	0.50954204	-6.810198088	6.90E-11	2.94E-10	13.95892953
AHCYL2	-0.682518887	4.94906901	-6.810090158	6.91E-11	2.94E-10	13.95830777
NINJ1	-0.440579719	7.48049739	-6.80917706	6.95E-11	2.96E-10	13.95304784
ZPBP	-0.140359104	0.17197225	-6.808442203	6.98E-11	2.97E-10	13.94881503
FAM186B	-0.236965292	0.62360617	-6.807233377	7.03E-11	2.99E-10	13.94185283
PSMB2	-0.326565173	5.85801315	-6.806263656	7.07E-11	3.00E-10	13.93626836
COL10A1	1.327313437	1.81197266	6.805553283	7.10E-11	3.02E-10	13.93217779
ZFYVE9	-0.600467302	4.2008473	-6.804328701	7.15E-11	3.04E-10	13.92512692
ZNF664	-0.600174783	6.52706742	-6.802992056	7.20E-11	3.06E-10	13.91743182
EBP	-0.361169234	5.23090945	-6.80275755	7.21E-11	3.06E-10	13.91608187
ORMDL3	-0.603026304	7.2443338	-6.802631586	7.22E-11	3.06E-10	13.91535676
ZFYVE28	-0.465185325	2.62818849	-6.802572795	7.22E-11	3.07E-10	13.91501834
MED30	-0.463738982	4.06322866	-6.801580912	7.26E-11	3.08E-10	13.90930896
NPRL3	-0.377132135	4.40125109	-6.801332584	7.27E-11	3.09E-10	13.90787965
MMP13	0.927042686	0.65434043	6.800258157	7.32E-11	3.10E-10	13.90169595

AMOT	0.8672119	3.72188531	6.799926506	7.33E-11	3.11E-10	13.89978732
CALCRL	-0.846712788	4.40599043	-6.798874353	7.38E-11	3.13E-10	13.89373267
ATL3	-0.433233191	5.28787173	-6.79736113	7.45E-11	3.16E-10	13.88502592
SHQ1	-0.373532091	3.28321063	-6.797034825	7.46E-11	3.16E-10	13.88314861
TRNT1	-0.380209859	3.41859509	-6.796610892	7.48E-11	3.17E-10	13.88070971
CROT	-0.662946634	5.27453811	-6.796532576	7.48E-11	3.17E-10	13.88025918
RAC2	1.226372973	4.4644898	6.795572267	7.53E-11	3.19E-10	13.87473494
SLC25A1	-0.474573396	4.50658849	-6.795158268	7.54E-11	3.19E-10	13.87235354
NDUFS1	-0.482835998	4.42714406	-6.795140544	7.54E-11	3.19E-10	13.8722516
IRS4	-0.412561271	0.35837138	-6.794982389	7.55E-11	3.19E-10	13.87134189
FANCC	-0.383472378	2.15605281	-6.794606898	7.57E-11	3.20E-10	13.86918213
FUT1	-0.430383147	2.62538643	-6.793231962	7.63E-11	3.23E-10	13.86127444
REEP1	-0.580895126	1.14076315	-6.790825906	7.74E-11	3.27E-10	13.84743912
RORC	-0.619728576	4.82676763	-6.790185225	7.77E-11	3.28E-10	13.84375565
METTL2	-0.300477751	2.1443041	-6.787259303	7.90E-11	3.34E-10	13.82693668
PLA2G3	-0.743065386	0.99251054	-6.786870471	7.92E-11	3.35E-10	13.82470195
RFLNB	-0.724730282	4.34630256	-6.785499861	7.98E-11	3.37E-10	13.81682536
DAP3	-0.366944106	5.70117	-6.783721753	8.07E-11	3.41E-10	13.80660862
CCDC17	-0.64278024	2.69257705	-6.783413461	8.08E-11	3.41E-10	13.80483741
SLC50A1	-0.383460832	6.14739666	-6.782876525	8.11E-11	3.42E-10	13.80175272
H2AFZ	-0.411686651	6.67789666	-6.782817242	8.11E-11	3.42E-10	13.80141215
CYHR1	-0.423738961	4.39732539	-6.781586198	8.17E-11	3.45E-10	13.7943405
DERPC	-0.580940298	6.13123517	-6.781383845	8.18E-11	3.45E-10	13.79317818
CHTF8	-0.580940298	6.13123517	-6.781383845	8.18E-11	3.45E-10	13.79317818
RPL27	-0.427945305	9.33799644	-6.780364974	8.23E-11	3.47E-10	13.78732614
FANCF	-0.3709894	3.61297554	-6.779112192	8.29E-11	3.49E-10	13.78013143
RAB24	-0.478108704	3.78494222	-6.778908237	8.30E-11	3.50E-10	13.77896021
ARFGAP	-0.567005902	5.87804759	-6.7780058	8.34E-11	3.51E-10	13.77377821
GLTPD2	-0.417867732	1.29323015	-6.777094269	8.39E-11	3.53E-10	13.76854448
TIGD5	-0.379914347	3.34808463	-6.776749784	8.40E-11	3.54E-10	13.76656667
PAK6	-0.213283291	0.50745346	-6.775976063	8.44E-11	3.55E-10	13.76212474
LIMK2	-0.583029117	5.12402196	-6.775313602	8.48E-11	3.57E-10	13.75832183
EID3	-0.473065419	1.8093714	-6.774281594	8.53E-11	3.59E-10	13.75239803
MNS1	-0.458597577	2.17998127	-6.774038589	8.54E-11	3.59E-10	13.75100325
YJU2	-0.387283357	5.66184804	-6.772610026	8.61E-11	3.62E-10	13.74280443
MFSD9	-0.444320814	2.70209553	-6.771703213	8.66E-11	3.64E-10	13.73760066
NDUFA4	-0.869981505	5.01797241	-6.771456989	8.67E-11	3.64E-10	13.73618779
USP7	-0.397389318	5.29993772	-6.771203674	8.68E-11	3.65E-10	13.73473425
CDK20	-0.423300391	2.90647607	-6.770180982	8.73E-11	3.67E-10	13.72886639
BCL2L2	-0.540440255	5.49975067	-6.768859195	8.80E-11	3.70E-10	13.72128334

NNT	-0.666810451	4.7102648	-6.767811525	8.86E-11	3.72E-10	13.71527362
FOXP3	0.733983163	1.72949258	6.765240895	8.99E-11	3.77E-10	13.70053053
MST1R	0.874684009	2.70193995	6.763473454	9.08E-11	3.81E-10	13.69039615
F12	-0.553317015	1.15289361	-6.762861945	9.12E-11	3.82E-10	13.68689023
ZNF589	-0.486222218	3.70785754	-6.762097753	9.16E-11	3.84E-10	13.68250926
TOR3A	-0.392257327	4.85002428	-6.762011701	9.16E-11	3.84E-10	13.68201596
TRMT61I	-0.487992664	3.59106182	-6.760949979	9.22E-11	3.86E-10	13.67592993
PSMD12	-0.347374421	4.68236652	-6.760920854	9.22E-11	3.86E-10	13.67576298
TRPV6	-0.629702879	0.80586087	-6.758967858	9.33E-11	3.91E-10	13.66456973
ILDR1	-0.563624393	4.53592171	-6.758403161	9.36E-11	3.92E-10	13.66133369
FMNL3	-0.481543067	4.11709837	-6.75761885	9.40E-11	3.94E-10	13.65683944
TIMP1	1.533704515	9.34593253	6.757099349	9.43E-11	3.95E-10	13.6538628
EPS15L1	-0.338288181	3.80750212	-6.756201865	9.48E-11	3.97E-10	13.64872077
PLXNC1	1.140974127	3.86454969	6.756149247	9.48E-11	3.97E-10	13.64841932
VMAC	-0.376242734	3.09363362	-6.754797847	9.56E-11	4.00E-10	13.64067758
SYNGR3	0.458039333	0.797242	6.754616521	9.57E-11	4.00E-10	13.6396389
ITGAM	0.785483514	2.50748809	6.753729138	9.62E-11	4.02E-10	13.63455604
CIRBP	-0.500995665	7.82742745	-6.752440771	9.69E-11	4.05E-10	13.62717721
LRPAP1	-0.481470822	6.30081109	-6.750791144	9.78E-11	4.09E-10	13.61773077
MMP16	0.991275182	1.91490411	6.749914447	9.84E-11	4.11E-10	13.6127111
WDR45B	-0.412327324	6.03132113	-6.749613338	9.85E-11	4.12E-10	13.61098716
IFIT5	0.656003302	4.44851779	6.749490052	9.86E-11	4.12E-10	13.61028132
EED	-0.321475531	2.78326607	-6.748565757	9.91E-11	4.14E-10	13.60498984
RFX8	-0.461906039	0.95981806	-6.748008555	9.95E-11	4.15E-10	13.60180018
STK19	-0.400181632	3.4105495	-6.747947776	9.95E-11	4.15E-10	13.60145226
ZNF287	-0.441921213	2.75087908	-6.747797696	9.96E-11	4.15E-10	13.60059317
SNRPD2	-0.421856714	7.85017386	-6.747234813	9.99E-11	4.17E-10	13.59737124
ABHD1	-0.290373498	0.65982944	-6.746391219	1.00E-10	4.19E-10	13.59254287
DPH6	-0.313969356	2.22227981	-6.745421629	1.01E-10	4.21E-10	13.58699387
ZDHHC7	-0.421851631	5.34414278	-6.743636812	1.02E-10	4.25E-10	13.57678076
BLOC1S:	-0.372563415	5.50684165	-6.74226175	1.03E-10	4.29E-10	13.56891363
C17orf58	-0.395248068	4.69780258	-6.74128275	1.03E-10	4.31E-10	13.56331318
DDX50	-0.461173545	5.64748544	-6.741047428	1.04E-10	4.31E-10	13.56196709
CAMK2N	1.515795627	6.41040615	6.740916918	1.04E-10	4.32E-10	13.56122055
LYPLA1	-0.460547118	4.24030589	-6.74091438	1.04E-10	4.32E-10	13.56120603
PGRMC2	-0.448447058	6.15594799	-6.740876137	1.04E-10	4.32E-10	13.56098728
AASDH	-0.478582557	3.08680691	-6.740823135	1.04E-10	4.32E-10	13.5606841
BTF3	-0.395966097	8.82833523	-6.739398176	1.05E-10	4.35E-10	13.5525339
INHBA	0.942484454	2.0541601	6.739312388	1.05E-10	4.35E-10	13.55204327
SLC25A2	-0.46409325	6.69773882	-6.738607049	1.05E-10	4.37E-10	13.54800949

RBM34	-0.257363879	1.48069139	-6.738297941	1.05E-10	4.38E-10	13.54624182
TMEM19	-0.230567914	0.44985281	-6.737792695	1.06E-10	4.39E-10	13.54335263
DELE1	-0.394726798	5.15908327	-6.737203544	1.06E-10	4.40E-10	13.53998382
DHPS	-0.3415258	5.55717526	-6.73714007	1.06E-10	4.40E-10	13.53962088
ASPA	-0.367851988	0.6403835	-6.736769253	1.06E-10	4.41E-10	13.53750065
ZNF613	-0.396961841	2.78166323	-6.736232609	1.07E-10	4.42E-10	13.53443241
FSIP1	-0.268595895	0.76211266	-6.736049996	1.07E-10	4.43E-10	13.53338836
SHISA2	-1.207998528	5.85745662	-6.735927397	1.07E-10	4.43E-10	13.53268744
MRM1	-0.386897605	3.7942486	-6.735689241	1.07E-10	4.43E-10	13.53132589
KCTD18	-0.520333425	3.87469295	-6.735606844	1.07E-10	4.44E-10	13.53085483
CDR2	-0.486721095	5.21132304	-6.733654927	1.08E-10	4.49E-10	13.51969697
ATRN	-0.601843588	5.34257521	-6.73346633	1.08E-10	4.49E-10	13.518619
BLOC1S!	-0.408467136	3.84090104	-6.733104808	1.09E-10	4.50E-10	13.51655269
DIABLO	-0.407390194	2.48892826	-6.732758473	1.09E-10	4.51E-10	13.51457327
ITGA10	-0.565739314	1.66603049	-6.729389821	1.11E-10	4.59E-10	13.49532393
ZFP82	-0.349480934	2.06482715	-6.729170359	1.11E-10	4.60E-10	13.49407011
LMNTD1	-0.270497783	0.20764168	-6.728933482	1.11E-10	4.60E-10	13.49271681
ARHGEF	-0.50257054	3.79344459	-6.728454067	1.11E-10	4.62E-10	13.48997799
PALMD	-0.544708505	2.4911114	-6.728050045	1.12E-10	4.63E-10	13.48766998
BNIP3	-0.506244947	4.49607939	-6.726904593	1.13E-10	4.66E-10	13.48112702
LARS	-0.405005	5.22760341	-6.726627228	1.13E-10	4.66E-10	13.47954279
PIGT	-0.527089806	7.56464425	-6.726513475	1.13E-10	4.66E-10	13.47889309
DRAM1	0.802475875	4.17590568	6.726310614	1.13E-10	4.67E-10	13.47773444
C9orf40	-0.333906029	2.63806473	-6.723026662	1.15E-10	4.76E-10	13.45898158
ZFPM1	-0.430898439	2.20272996	-6.722954296	1.15E-10	4.76E-10	13.45856841
CHRNA7	-0.384609382	0.52311459	-6.722031993	1.16E-10	4.78E-10	13.45330283
NXT1	-0.374606366	5.20873869	-6.721985299	1.16E-10	4.78E-10	13.45303626
CST2	1.664916015	2.04151652	6.721688677	1.16E-10	4.79E-10	13.45134291
PPP2R5(	-0.385531578	4.74761162	-6.721662478	1.16E-10	4.79E-10	13.45119335
OSCAR	0.858535142	2.30833697	6.71978686	1.17E-10	4.84E-10	13.4404871
GOLGA1	-0.432552927	3.53569192	-6.719617494	1.17E-10	4.84E-10	13.43952044
ERVK3-1	-0.403080388	3.25856993	-6.717455161	1.19E-10	4.90E-10	13.4271804
TSPAN1'	-0.401428154	4.39311508	-6.71628633	1.20E-10	4.94E-10	13.42051125
FAM174E	-0.576125988	5.77380516	-6.716278041	1.20E-10	4.94E-10	13.42046396
BRF2	-0.498799808	3.01795222	-6.71495484	1.21E-10	4.97E-10	13.41291499
SLC38A1	-0.447656274	6.49834094	-6.713134031	1.22E-10	5.03E-10	13.40252882
SAP30BI	-0.34563183	4.40331787	-6.71135936	1.23E-10	5.08E-10	13.39240773
CRY1	-0.525811739	4.14992853	-6.711171839	1.23E-10	5.08E-10	13.39133839
EGF	-0.633342443	0.95178299	-6.706715526	1.27E-10	5.21E-10	13.36593242
GJC3	-0.20422163	0.41911906	-6.705725205	1.27E-10	5.24E-10	13.36028809

OPA3	-0.367913114	3.52140096	-6.704369069	1.28E-10	5.28E-10	13.35255974
C11orf98	-0.423801867	3.56657764	-6.703606124	1.29E-10	5.30E-10	13.34821235
ZNF814	-0.376227248	2.30457809	-6.702435503	1.30E-10	5.34E-10	13.34154262
AMIGO1	-0.482692816	3.87097819	-6.69896779	1.32E-10	5.45E-10	13.32178979
NRXN2	-0.724194205	1.48211456	-6.69815833	1.33E-10	5.47E-10	13.31717996
PARD6G	-0.495106407	4.20832921	-6.697964433	1.33E-10	5.48E-10	13.31607579
DDIT4L	-0.578150294	0.87033133	-6.697124428	1.34E-10	5.50E-10	13.31129252
PHACTR	0.559309135	0.69103021	6.697014326	1.34E-10	5.50E-10	13.31066559
MRTO4	-0.339664185	5.06349555	-6.69652005	1.34E-10	5.52E-10	13.30785124
MED4	-0.348578936	5.75411216	-6.696516597	1.34E-10	5.52E-10	13.30783159
KAT2B	-0.638981838	4.35350217	-6.696463978	1.34E-10	5.52E-10	13.30753199
BTBD17	-0.872213436	0.59821429	-6.696131274	1.35E-10	5.53E-10	13.30563771
DCUN1D	-0.434799559	3.43554935	-6.695365799	1.35E-10	5.55E-10	13.30127966
DNAJB1:	-0.312830033	5.31106763	-6.695172515	1.35E-10	5.56E-10	13.3001793
FAF2	-0.457612089	4.95914369	-6.693913102	1.36E-10	5.59E-10	13.29301003
EXOC6	-0.537485648	4.73719901	-6.692484544	1.37E-10	5.64E-10	13.28487904
EIF4H	-0.492205738	7.69542202	-6.691693604	1.38E-10	5.66E-10	13.28037774
BYSL	-0.379623378	3.91550756	-6.690121045	1.39E-10	5.71E-10	13.27142928
LYNX1	0.751930796	3.98179082	6.690099415	1.39E-10	5.71E-10	13.27130621
SORD	-0.897090931	6.20583374	-6.688124873	1.41E-10	5.78E-10	13.26007243
BLOC1S:	-0.351027162	3.8938028	-6.685842861	1.43E-10	5.85E-10	13.24709226
SYN1	0.769045215	1.35397372	6.685026391	1.44E-10	5.88E-10	13.2424489
VPS26B	-0.368623799	4.75413015	-6.684289115	1.44E-10	5.91E-10	13.23825627
KHK	-0.483560457	1.79322945	-6.683693878	1.45E-10	5.92E-10	13.2348716
C19orf44	-0.364814554	2.97993767	-6.683546165	1.45E-10	5.93E-10	13.2340317
PRPF31	-0.381411826	5.99678747	-6.682806889	1.45E-10	5.95E-10	13.22982835
BEND6	0.574821365	0.89982564	6.681272044	1.47E-10	6.00E-10	13.22110262
DDX23	-0.419988802	5.69374689	-6.680975372	1.47E-10	6.01E-10	13.21941618
KBTBD6	-0.428227162	3.33041887	-6.680837491	1.47E-10	6.02E-10	13.2186324
HUS1	-0.338763177	3.36180997	-6.680482659	1.47E-10	6.03E-10	13.21661545
ATP1B2	-1.097937833	3.42039252	-6.679666412	1.48E-10	6.05E-10	13.21197596
SF3A3	-0.3841287	5.54134567	-6.677817393	1.50E-10	6.12E-10	13.20146777
PCIF1	-0.389217208	6.05422599	-6.677726766	1.50E-10	6.12E-10	13.20095278
INTS10	-0.434707158	5.06255891	-6.676727594	1.51E-10	6.15E-10	13.19527528
KDF1	-0.400838936	5.35246742	-6.676467803	1.51E-10	6.16E-10	13.19379918
RPL31	-0.454749768	8.50906527	-6.676020398	1.51E-10	6.18E-10	13.19125719
SELENO	-0.507246283	8.43128461	-6.674998305	1.52E-10	6.21E-10	13.1854505
OPN1SW	-0.490952845	2.33135235	-6.674923483	1.52E-10	6.21E-10	13.18502544
LSM8	-0.287710462	3.46121097	-6.674421543	1.53E-10	6.23E-10	13.18217408
AS3MT	-0.268700069	0.56560158	-6.673743213	1.53E-10	6.25E-10	13.17832094

LRRC46	-0.427809148	2.34661896	-6.673353963	1.54E-10	6.27E-10	13.17610999
DPY19L2	-0.798340433	2.27125016	-6.671691489	1.55E-10	6.32E-10	13.16666813
NKAPD1	-0.380146223	4.29024858	-6.67018992	1.57E-10	6.38E-10	13.15814154
RPL7	-0.474228041	9.13334203	-6.669154401	1.57E-10	6.42E-10	13.15226218
CRYBG2	0.523151826	0.76027296	6.667254752	1.59E-10	6.48E-10	13.14147822
PDCD2L	-0.347588758	3.42433231	-6.666485603	1.60E-10	6.51E-10	13.13711251
TANGO2	-0.466253927	4.30944268	-6.666391499	1.60E-10	6.51E-10	13.1365784
JHY	-0.399675879	2.43842158	-6.666332629	1.60E-10	6.51E-10	13.13624427
ZBTB17	-0.341481889	4.28338787	-6.666324067	1.60E-10	6.51E-10	13.13619567
TEKT3	-0.289327553	0.49722858	-6.665366708	1.61E-10	6.55E-10	13.13076227
IL2RA	0.693090847	1.37514255	6.663428378	1.63E-10	6.62E-10	13.11976314
H1F0	-0.570824143	7.71083804	-6.663259703	1.63E-10	6.63E-10	13.1188061
FAM122A	-0.448996556	4.21022136	-6.663202376	1.63E-10	6.63E-10	13.11848083
LINS1	-0.373825308	2.82314766	-6.662811328	1.63E-10	6.64E-10	13.11626214
EPHB1	-0.777986948	2.02778062	-6.66093563	1.65E-10	6.71E-10	13.10562125
SPECC1	0.566508776	2.71687228	6.659371939	1.67E-10	6.77E-10	13.096752
MRPS2	-0.364594637	5.294397	-6.659249133	1.67E-10	6.77E-10	13.0960555
PRDM16	-0.7419212	3.01543319	-6.659017668	1.67E-10	6.78E-10	13.09474277
TTLL7	-0.495393069	1.01189228	-6.658201017	1.68E-10	6.81E-10	13.09011149
DTX3	-0.471465141	4.94722381	-6.657976535	1.68E-10	6.82E-10	13.0888385
DEDD2	-0.398934846	5.10681237	-6.657027879	1.69E-10	6.85E-10	13.08345923
TIPIN	-0.367022244	2.69624568	-6.655403892	1.71E-10	6.92E-10	13.07425182
BLOC1S	-0.480377338	6.23902478	-6.653270553	1.73E-10	7.00E-10	13.06215896
SPATA1	0.176517475	0.25448619	6.653155775	1.73E-10	7.01E-10	13.06150841
DDX19A	-0.374663262	4.06106032	-6.652848035	1.73E-10	7.02E-10	13.05976423
TRIM2	-0.646652037	5.30188572	-6.652217001	1.74E-10	7.04E-10	13.05618789
SIGLEC6	1.292377796	1.2666038	6.652010466	1.74E-10	7.05E-10	13.05501742
DMBX1	1.048238067	1.13985598	6.6485608	1.77E-10	7.19E-10	13.03547133
RIN1	0.678487154	2.20250595	6.647852648	1.78E-10	7.22E-10	13.03145977
HSD17B	-1.552948408	4.23786704	-6.646348529	1.80E-10	7.28E-10	13.02294018
LIN37	-0.31935267	2.25949238	-6.645424155	1.81E-10	7.31E-10	13.01770504
MRPL55	-0.437858982	5.7978663	-6.644772902	1.81E-10	7.34E-10	13.01401701
RBM18	-0.430546319	4.22424373	-6.64430269	1.82E-10	7.36E-10	13.01135437
USP21	-0.404469939	4.53184965	-6.644112689	1.82E-10	7.36E-10	13.01027851
PHYKPL	-0.407233188	3.86700456	-6.641818306	1.84E-10	7.46E-10	12.99728843
WRAP53	-0.341759574	2.6084817	-6.640687423	1.86E-10	7.51E-10	12.99088688
PLCL2	-0.508864169	1.99863396	-6.640144126	1.86E-10	7.53E-10	12.98781173
ABI3	-0.576061283	4.05770248	-6.639032668	1.87E-10	7.58E-10	12.98152126
STARD1	-0.449614929	5.6336053	-6.638970183	1.88E-10	7.58E-10	12.98116764
CACNA2	-1.013877981	2.80555902	-6.63870801	1.88E-10	7.59E-10	12.97968395

SETD6	-0.385649265	3.23108104	-6.634910283	1.92E-10	7.75E-10	12.95819645
FXYD3	0.999078655	1.20425208	6.634684803	1.92E-10	7.76E-10	12.95692096
RPL18A	-0.492267342	7.86535355	-6.634620511	1.92E-10	7.76E-10	12.95655728
CDSN	0.503754652	0.60639906	6.632846542	1.94E-10	7.84E-10	12.94652347
SMO	-0.723432785	4.22663391	-6.628594962	1.99E-10	8.04E-10	12.92248364
SLC25A2	-0.324917368	5.48261198	-6.627409499	2.01E-10	8.09E-10	12.91578259
HARS	-0.312759405	4.58362242	-6.62729337	2.01E-10	8.09E-10	12.91512619
REELD1	0.620570662	0.84483696	6.625958205	2.02E-10	8.15E-10	12.90758002
E4F1	-0.441963712	4.28362039	-6.624678945	2.04E-10	8.21E-10	12.90035083
NUDT12	-0.519729809	4.30411314	-6.622929692	2.06E-10	8.29E-10	12.89046727
DOK6	-0.464781077	0.80139714	-6.622490255	2.06E-10	8.31E-10	12.88798466
CBWD5	-0.18205972	0.59516358	-6.622284577	2.07E-10	8.32E-10	12.88682273
CXCL8	1.223344019	2.53834558	6.622188224	2.07E-10	8.32E-10	12.88627841
USP20	-0.438528038	5.13026917	-6.620881536	2.08E-10	8.38E-10	12.87889719
PAAF1	-0.362554647	3.39003336	-6.620640053	2.09E-10	8.39E-10	12.87753322
ZBED8	-0.438234429	3.28298594	-6.620505831	2.09E-10	8.40E-10	12.87677511
RDX	-0.645332872	5.22501166	-6.619998468	2.09E-10	8.42E-10	12.87390951
PWP1	-0.359322545	5.44731377	-6.619383581	2.10E-10	8.45E-10	12.87043682
OLR1	1.051363168	2.84659846	6.6189123	2.11E-10	8.47E-10	12.86777533
ZNF212	-0.413702294	4.04050086	-6.618459994	2.11E-10	8.49E-10	12.86522112
FDXR	-0.528032386	4.30346632	-6.615637572	2.15E-10	8.63E-10	12.84928547
CNOT10	-0.316548281	4.07862142	-6.615516547	2.15E-10	8.63E-10	12.84860226
CYB561D	-0.452647634	3.76441998	-6.613425453	2.17E-10	8.73E-10	12.836799
EREG	0.71949983	0.58771595	6.610976499	2.20E-10	8.86E-10	12.82297915
SPRY4	-0.674517628	4.76129102	-6.610283321	2.21E-10	8.89E-10	12.81906808
PANK1	-0.625796947	2.47820746	-6.609215595	2.23E-10	8.94E-10	12.81304431
COX18	-0.318052211	3.1329105	-6.609178283	2.23E-10	8.94E-10	12.81283382
FAM8A1	-0.51565623	5.2576672	-6.608850294	2.23E-10	8.96E-10	12.81098355
RPL35A	-0.413707158	8.82337937	-6.604897413	2.28E-10	9.16E-10	12.7886895
FBXO5	-0.451864789	2.29247487	-6.604891597	2.28E-10	9.16E-10	12.78865671
SYDE1	-0.496367423	3.22736736	-6.604406434	2.29E-10	9.18E-10	12.78592106
VPS29	-0.312901276	5.7764143	-6.602073131	2.32E-10	9.31E-10	12.77276647
COMP	1.824566751	3.67670279	6.598610114	2.37E-10	9.49E-10	12.75324889
ZBTB5	-0.490367134	3.7494125	-6.598205497	2.37E-10	9.51E-10	12.75096894
THAP5	-0.458590487	4.05759116	-6.597969617	2.38E-10	9.52E-10	12.74963985
ZNF689	-0.389715361	3.33933039	-6.59687993	2.39E-10	9.58E-10	12.74350029
CLIP3	0.967800682	3.8686484	6.596426669	2.40E-10	9.60E-10	12.74094671
ASPH	-0.653396008	3.99078652	-6.596300381	2.40E-10	9.61E-10	12.74023526
RPL5	-0.421025973	10.1895448	-6.596194324	2.40E-10	9.61E-10	12.73963779
GSPT1	-0.454767021	5.52732109	-6.596099754	2.40E-10	9.61E-10	12.73910503

ARRDC4	-0.716486438	4.50911811	-6.595996425	2.40E-10	9.62E-10	12.73852293
C17orf50	-0.374580284	0.68729635	-6.595564912	2.41E-10	9.64E-10	12.73609211
CHRAC1	-0.367577675	5.19886719	-6.594136847	2.43E-10	9.72E-10	12.72804828
LRIG1	-0.596734794	3.99221466	-6.593636971	2.44E-10	9.74E-10	12.72523293
LAMC3	-0.607855645	0.86595746	-6.593505385	2.44E-10	9.75E-10	12.72449184
SH3RF1	-0.62781202	4.19273712	-6.593087067	2.44E-10	9.77E-10	12.72213599
CARTPT	-2.036359126	1.82840687	-6.592476972	2.45E-10	9.80E-10	12.71870028
SMYD4	-0.380720488	2.84835877	-6.590801784	2.48E-10	9.89E-10	12.70926773
ZDHHC1	-0.374142236	2.02723216	-6.589726207	2.49E-10	9.95E-10	12.70321233
CYP7B1	-0.701693718	2.35506789	-6.585931293	2.55E-10	1.02E-09	12.68185291
COL4A5	-0.792708588	3.11698258	-6.584974477	2.56E-10	1.02E-09	12.67646892
HIST2H4	-0.16295841	0.27917331	-6.584931573	2.56E-10	1.02E-09	12.67622751
OTUD7A	-0.241953902	0.69920702	-6.584342184	2.57E-10	1.03E-09	12.67291131
AK3	-0.45010843	5.88123055	-6.583687212	2.58E-10	1.03E-09	12.66922636
CACNB2	-0.51456239	1.14029021	-6.582781342	2.59E-10	1.03E-09	12.66413026
BRMS1L	-0.442738712	3.16743587	-6.581866806	2.61E-10	1.04E-09	12.6589859
HEBP2	-0.328530672	4.03921163	-6.580383677	2.63E-10	1.05E-09	12.65064423
LRRIQ1	-0.316477957	0.76324017	-6.57974924	2.64E-10	1.05E-09	12.64707634
ZFAND1	-0.447564502	4.75026481	-6.578388996	2.66E-10	1.06E-09	12.63942752
FUT2	0.493152113	1.42061335	6.578107895	2.66E-10	1.06E-09	12.63784699
DHTKD1	-0.515218679	4.20779936	-6.575214115	2.71E-10	1.08E-09	12.62157913
MLLT1	-0.507623052	5.77650829	-6.573779941	2.73E-10	1.09E-09	12.61351858
SLC9A3F	-0.660725592	5.573283	-6.5732847	2.74E-10	1.09E-09	12.61073544
FTSJ3	-0.418933869	4.93877372	-6.573129138	2.74E-10	1.09E-09	12.60986124
THOP1	-0.381693938	3.84020553	-6.572979821	2.74E-10	1.09E-09	12.60902217
NOLC1	-0.526113625	5.81100871	-6.571317237	2.77E-10	1.10E-09	12.59968023
PRELID2	-0.263903663	1.26998754	-6.570035433	2.79E-10	1.11E-09	12.59247902
PLCL1	-0.532056582	1.69342642	-6.569723779	2.80E-10	1.11E-09	12.59072829
PHTF1	-0.369976709	2.53005642	-6.569374901	2.80E-10	1.11E-09	12.58876852
TRADD	-0.435110356	4.90218135	-6.568808396	2.81E-10	1.12E-09	12.58558642
SELENO	-0.421510636	4.95838415	-6.568559456	2.82E-10	1.12E-09	12.58418817
ERG28	-0.357494193	6.10122664	-6.568471723	2.82E-10	1.12E-09	12.5836954
CCDC77	-0.349233677	2.73181733	-6.568107867	2.82E-10	1.12E-09	12.58165177
GTF3C5	-0.381132919	5.26328248	-6.565954561	2.86E-10	1.13E-09	12.56955921
AP1S1	-0.419476605	6.50401	-6.565562433	2.86E-10	1.14E-09	12.56735739
CXCL16	0.596041401	5.74502684	6.565270034	2.87E-10	1.14E-09	12.56571561
FDPS	-0.419018254	5.50570476	-6.565095383	2.87E-10	1.14E-09	12.564735
ACO2	-0.720709615	6.01256809	-6.563903669	2.89E-10	1.15E-09	12.55804439
PARN	-0.37477171	5.50849145	-6.56343593	2.90E-10	1.15E-09	12.55541861
WDR31	-0.513137365	2.6703757	-6.562458289	2.92E-10	1.16E-09	12.54993078

KDM1B	-0.598291269	4.67349414	-6.561823852	2.93E-10	1.16E-09	12.54636979
CCDC10:	-0.471947078	4.21628334	-6.561644658	2.93E-10	1.16E-09	12.54536405
LIPH	1.559327553	4.65813754	6.560376392	2.95E-10	1.17E-09	12.53824634
LRRC59	-0.453228723	5.85812477	-6.559468967	2.97E-10	1.17E-09	12.53315433
LGALS3	1.338577011	8.04049266	6.559359431	2.97E-10	1.17E-09	12.5325397
MAOB	-1.097028688	2.55544761	-6.559052797	2.97E-10	1.18E-09	12.53081916
LYRM1	-0.334361272	4.85678432	-6.558507322	2.98E-10	1.18E-09	12.52775861
ITGB2	1.039432066	4.32221146	6.55801668	2.99E-10	1.18E-09	12.52500587
H1FX	-0.493118404	7.39828991	-6.557903583	2.99E-10	1.18E-09	12.52437136
USP49	-0.495190266	1.69261305	-6.552976715	3.08E-10	1.22E-09	12.49673771
BOLA2-S	-0.420593198	1.59259937	-6.551965865	3.10E-10	1.22E-09	12.49106991
SMARCE	-0.357088439	4.32981174	-6.551548577	3.10E-10	1.23E-09	12.48873037
BUB1B-F	-0.132219535	0.29156055	-6.551187537	3.11E-10	1.23E-09	12.48670628
C11orf94	-0.393406794	0.82376066	-6.549594556	3.14E-10	1.24E-09	12.47777651
DYNC1LI	-0.375686976	3.98730842	-6.549577294	3.14E-10	1.24E-09	12.47767976
ARPC4	-0.366190268	6.76692695	-6.549017839	3.15E-10	1.24E-09	12.474544
ICMT	-0.427909112	6.10045821	-6.548925988	3.15E-10	1.24E-09	12.47402919
C1QTNF	-0.189271873	0.27410627	-6.548787996	3.15E-10	1.24E-09	12.47325578
UPP1	0.850757704	4.78068089	6.548665338	3.16E-10	1.25E-09	12.47256832
RNASE1	-0.635262478	6.74395337	-6.547948827	3.17E-10	1.25E-09	12.46855269
C4orf45	-0.132652007	0.17215473	-6.547834939	3.17E-10	1.25E-09	12.46791444
TNK1	-0.37091512	3.55612637	-6.547406727	3.18E-10	1.25E-09	12.46551474
HIST1H4	-0.586578928	1.09456393	-6.547183239	3.18E-10	1.25E-09	12.46426235
C19orf33	1.55257921	6.17030734	6.546555726	3.19E-10	1.26E-09	12.46074605
MAP1LC	-0.575584086	1.2990351	-6.543528173	3.25E-10	1.28E-09	12.44378439
ERCC1	-0.320456408	5.05323668	-6.542543826	3.27E-10	1.29E-09	12.43827085
DNAJC1!	-0.475414933	4.29247797	-6.542500068	3.27E-10	1.29E-09	12.43802577
C1orf158	-0.261632339	0.17581769	-6.542226799	3.27E-10	1.29E-09	12.43649525
ADRA2B	-0.437794743	1.00046193	-6.541495371	3.29E-10	1.29E-09	12.43239889
C9orf64	-0.568405614	4.79204637	-6.541375371	3.29E-10	1.30E-09	12.43172686
PIGS	-0.585368203	4.96271275	-6.540345932	3.31E-10	1.30E-09	12.42596212
CADPS2	-0.589035147	3.92219462	-6.540114619	3.31E-10	1.30E-09	12.42466688
TMEM79	0.799417716	3.86611747	6.539927157	3.32E-10	1.30E-09	12.42361722
RUNX1T	-0.359436883	0.94030162	-6.537774749	3.36E-10	1.32E-09	12.41156662
ANK3	-0.620523286	2.85866022	-6.537629977	3.36E-10	1.32E-09	12.41075619
SLC35A1	-0.405008681	4.32316491	-6.536858389	3.38E-10	1.33E-09	12.40643709
IGSF6	0.782033288	2.71342593	6.536168638	3.39E-10	1.33E-09	12.40257639
CSTF3	-0.381981673	4.3636347	-6.535829571	3.40E-10	1.33E-09	12.40067866
TMEM86.	-0.333275557	2.82620921	-6.535347127	3.41E-10	1.34E-09	12.39797858
CCDC17	-0.328022941	4.32169999	-6.5346867	3.42E-10	1.34E-09	12.39428263

GPX3	-0.894864636	11.1330279	-6.534483645	3.42E-10	1.34E-09	12.39314632
PRRT4	-1.042683076	0.94642928	-6.533928184	3.43E-10	1.35E-09	12.39003805
GNA15	0.767621016	2.23115908	6.531460328	3.48E-10	1.37E-09	12.37623065
COG5	-0.530954368	3.7260422	-6.531258425	3.49E-10	1.37E-09	12.37510118
IL22RA1	-0.902744423	2.39741531	-6.529983433	3.51E-10	1.38E-09	12.36796935
RPAIN	-0.339192507	3.43599534	-6.529923798	3.51E-10	1.38E-09	12.3676358
VEPH1	-0.372531807	0.74141793	-6.529374636	3.52E-10	1.38E-09	12.36456431
GATC	-0.323353797	4.09231997	-6.52844939	3.54E-10	1.39E-09	12.35938979
DLL4	-0.773021899	3.79030219	-6.527842488	3.56E-10	1.39E-09	12.35599592
CERS4	-0.544731424	6.4236575	-6.526804709	3.58E-10	1.40E-09	12.35019306
TCEA1	-0.532200223	5.44056644	-6.526190847	3.59E-10	1.41E-09	12.34676088
CHST10	-0.577446988	4.08895525	-6.52601532	3.59E-10	1.41E-09	12.34577953
HAP1	-0.651325082	0.9315304	-6.525660806	3.60E-10	1.41E-09	12.34379755
AOC2	-0.32187547	0.76334918	-6.524298736	3.63E-10	1.42E-09	12.33618332
HAPLN3	0.845635062	2.81885557	6.524293906	3.63E-10	1.42E-09	12.33615633
USP46	-0.508992978	3.02563088	-6.522528399	3.67E-10	1.43E-09	12.32628851
ADAM12	0.767201844	1.16442277	6.521555826	3.69E-10	1.44E-09	12.32085339
SH3TC1	-0.467190256	2.65730386	-6.519890581	3.72E-10	1.46E-09	12.3115487
HPS6	-0.343338269	4.63533847	-6.519823917	3.72E-10	1.46E-09	12.31117624
DAXX	-0.324856688	5.60294968	-6.519376084	3.73E-10	1.46E-09	12.30867424
MTPAP	-0.339583781	3.06954028	-6.51698633	3.78E-10	1.48E-09	12.29532502
FAM234/	-0.37727213	4.65100231	-6.516377385	3.80E-10	1.48E-09	12.29192399
MYRIP	-0.51105278	1.31447745	-6.516373156	3.80E-10	1.48E-09	12.29190037
MAP11	-0.447097783	4.29092075	-6.516359487	3.80E-10	1.48E-09	12.29182403
GDF10	-1.075913022	1.18754543	-6.515688936	3.81E-10	1.49E-09	12.2880792
ZNF17	-0.361901177	2.47554294	-6.51453636	3.84E-10	1.50E-09	12.28164305
MMP12	0.943422339	1.09089989	6.513940127	3.85E-10	1.50E-09	12.27831391
DTD1	-0.400452379	5.40197596	-6.513791643	3.85E-10	1.50E-09	12.27748487
GDF15	1.401943856	6.31521389	6.513762436	3.85E-10	1.50E-09	12.2773218
HRH1	0.828181822	2.58300931	6.510496992	3.93E-10	1.53E-09	12.25909304
INO80E	-0.398184938	4.45084762	-6.510212033	3.93E-10	1.53E-09	12.25750261
ECHDC2	-0.496112257	4.86987189	-6.509023789	3.96E-10	1.54E-09	12.25087128
IPO11	-0.427502536	3.27133283	-6.509005178	3.96E-10	1.54E-09	12.25076743
TRPC4	-0.387972368	0.89581874	-6.508558357	3.97E-10	1.55E-09	12.24827404
HTATSF:	-0.484164435	6.34218431	-6.50852922	3.97E-10	1.55E-09	12.24811146
MS4A14	0.339488462	0.77747016	6.507377116	4.00E-10	1.56E-09	12.24168299
NKAP	-0.295817271	3.26627327	-6.50726079	4.00E-10	1.56E-09	12.24103396
DTX4	1.43811283	6.5546372	6.507164885	4.00E-10	1.56E-09	12.24049888
RNF223	0.376970669	0.43332775	6.505799865	4.03E-10	1.57E-09	12.23288362
MYH3	-0.717139913	1.34951247	-6.504615607	4.06E-10	1.58E-09	12.22627774

TRIM4	-0.534819549	4.84834293	-6.504537365	4.06E-10	1.58E-09	12.22584133
PMEL	-0.338452305	1.47112513	-6.50420606	4.07E-10	1.58E-09	12.22399346
PDHX	-0.368153368	4.08644899	-6.503485085	4.09E-10	1.59E-09	12.2199724
YEATS4	-0.462914361	4.54238702	-6.502032982	4.12E-10	1.60E-09	12.21187464
NEU4	-0.3232161	0.31069464	-6.500624122	4.15E-10	1.61E-09	12.20401926
PSMD1	-0.421262439	5.58435525	-6.500367083	4.16E-10	1.62E-09	12.20258622
GTPBP4	-0.378051256	4.09535068	-6.500135821	4.17E-10	1.62E-09	12.20129692
SRD5A3	-0.463892264	3.32944224	-6.499440143	4.18E-10	1.62E-09	12.19741869
PIGO	-0.52258754	4.14354462	-6.499159813	4.19E-10	1.63E-09	12.195856
BIVM	-0.390954457	3.16937339	-6.499076815	4.19E-10	1.63E-09	12.19539334
NTMT1	-0.379116278	4.05068501	-6.498692103	4.20E-10	1.63E-09	12.19324888
TCP10L	-0.214914578	0.58807649	-6.497202689	4.24E-10	1.64E-09	12.18494744
TLN2	-0.695592196	2.89588291	-6.495275104	4.28E-10	1.66E-09	12.17420583
MRPL46	-0.353931276	2.8596226	-6.495185953	4.28E-10	1.66E-09	12.17370909
ST6GALI	-0.685989368	3.2978848	-6.49467303	4.30E-10	1.67E-09	12.1708512
RP9	-0.345700367	4.03217973	-6.493904505	4.32E-10	1.67E-09	12.16656945
CWC25	-0.365209999	4.20634496	-6.493704868	4.32E-10	1.67E-09	12.16545726
ADAMTS	-0.450817689	1.29383815	-6.493144551	4.33E-10	1.68E-09	12.16233582
SOX3	-0.830898478	0.57528144	-6.490896684	4.39E-10	1.70E-09	12.14981524
GJB3	1.41167036	3.44424566	6.488194213	4.46E-10	1.73E-09	12.13476663
HEATR3	-0.367746976	3.22507808	-6.485426752	4.53E-10	1.75E-09	12.11936077
MAP3K4	-0.40753346	3.50445492	-6.482751568	4.60E-10	1.78E-09	12.10447309
ADCY8	1.181463731	1.40603544	6.479682578	4.68E-10	1.81E-09	12.08739924
PRKD1	-0.488376493	2.59485405	-6.478517339	4.71E-10	1.82E-09	12.08091814
RSL24D1	-0.377424243	6.42695979	-6.478468342	4.71E-10	1.82E-09	12.08064564
HLA-DQE	1.389362495	4.64090987	6.477683037	4.73E-10	1.83E-09	12.07627824
KANK3	-0.57612402	3.48013115	-6.477518452	4.74E-10	1.83E-09	12.07536296
DOK7	1.082907525	2.56267524	6.477136202	4.75E-10	1.84E-09	12.07323729
RNF144A	0.676841833	3.34742435	6.476465774	4.77E-10	1.84E-09	12.06950929
CMPK1	-0.441599993	6.89715562	-6.475257712	4.80E-10	1.85E-09	12.06279241
KCNC4	-0.328849643	1.1718265	-6.472040465	4.89E-10	1.89E-09	12.04490876
DEXI	-0.452087562	3.9885552	-6.471304706	4.91E-10	1.90E-09	12.04081981
ST6GAL2	-0.990321034	5.22380103	-6.469437399	4.96E-10	1.92E-09	12.03044382
NAP1L4	-0.33824422	6.39202947	-6.466877171	5.03E-10	1.94E-09	12.01622099
MBOAT2	0.80298464	4.01506969	6.466581525	5.04E-10	1.95E-09	12.01457885
NCALD	-0.453101078	1.77658301	-6.466195815	5.05E-10	1.95E-09	12.01243654
C1GALT1	-0.458185988	3.34834012	-6.464170602	5.11E-10	1.97E-09	12.0011896
RAP1A	-0.374853677	5.55816357	-6.463899763	5.12E-10	1.97E-09	11.9996857
SLTM	-0.459618274	5.07849293	-6.463641884	5.13E-10	1.98E-09	11.9982538
GSTA4	-0.532008348	4.71260174	-6.463358219	5.14E-10	1.98E-09	11.99667877

PEX5	-0.428032396	4.55630312	-6.462048949	5.17E-10	1.99E-09	11.98940979
CCDC15:	-0.431744019	1.61047944	-6.46200348	5.18E-10	1.99E-09	11.98915737
PPM1F	-0.398273758	3.45562971	-6.461926047	5.18E-10	1.99E-09	11.9887275
CTU2	-0.360798567	3.94277463	-6.461404229	5.19E-10	2.00E-09	11.98583074
TMEM12	-0.378234734	2.43543904	-6.460787414	5.21E-10	2.01E-09	11.98240683
COG1	-0.350287175	4.57412869	-6.460398222	5.22E-10	2.01E-09	11.98024657
CBX7	-0.502415505	5.13880038	-6.460369909	5.22E-10	2.01E-09	11.98008942
TRAPPC	-0.419903674	4.52514505	-6.459212668	5.26E-10	2.02E-09	11.97366658
TMEM21	-0.40477018	4.38459817	-6.458805401	5.27E-10	2.03E-09	11.97140639
ARPC5	-0.338437829	5.20035941	-6.458563723	5.28E-10	2.03E-09	11.97006521
MYCN	-0.727274067	1.64521234	-6.458212152	5.29E-10	2.03E-09	11.96811425
SCUBE1	-0.880452763	1.20491022	-6.457461106	5.31E-10	2.04E-09	11.96394675
MMP20	0.103307334	0.07423797	6.457181496	5.32E-10	2.04E-09	11.9623953
LRRC57	-0.378892237	3.19946886	-6.45408729	5.41E-10	2.08E-09	11.94522999
NCBP2	-0.353108068	5.67945739	-6.453063448	5.44E-10	2.09E-09	11.93955146
CBLL1	-0.438436063	3.94384882	-6.452667038	5.46E-10	2.10E-09	11.93735302
TFDP1	-0.46794755	5.52464948	-6.452628977	5.46E-10	2.10E-09	11.93714195
ATP7B	-0.425754414	1.25077317	-6.450507617	5.52E-10	2.12E-09	11.92537889
TGM1	0.843434178	2.0432199	6.450472557	5.53E-10	2.12E-09	11.92518451
RPRM	-0.692680552	1.36009973	-6.450095597	5.54E-10	2.13E-09	11.92309454
HLX	-0.624006099	2.48054832	-6.449279317	5.56E-10	2.13E-09	11.91856917
RPUSD1	-0.3421664	4.35242118	-6.44878841	5.58E-10	2.14E-09	11.91584783
MIGA2	-0.37621855	4.15035246	-6.447867328	5.61E-10	2.15E-09	11.91074223
SLC2A11	-0.436178987	3.06752175	-6.444308464	5.72E-10	2.19E-09	11.8910202
QARS	-0.320538275	6.69455434	-6.441677897	5.81E-10	2.23E-09	11.87644751
CCDC81	-0.214711298	0.59701643	-6.440883971	5.83E-10	2.24E-09	11.8720502
KIAA031!	-0.446067509	5.32953615	-6.440238132	5.86E-10	2.24E-09	11.86847338
ZC3H7A	-0.470162266	4.44793212	-6.439640372	5.88E-10	2.25E-09	11.86516307
TXNRD1	-0.632994822	4.48941029	-6.438750213	5.91E-10	2.26E-09	11.86023391
KIF7	-0.522134226	2.78320972	-6.437993266	5.93E-10	2.27E-09	11.85604278
USP30	-0.404295316	3.69629899	-6.437938445	5.93E-10	2.27E-09	11.85573925
PLAU	1.430414877	6.22768368	6.437785587	5.94E-10	2.27E-09	11.85489294
SRP54	-0.442732062	5.35964956	-6.437629102	5.94E-10	2.27E-09	11.85402657
RHOJ	-0.538009229	3.24116972	-6.437471948	5.95E-10	2.28E-09	11.8531565
EIF6	-0.365433311	7.44290256	-6.436958498	5.97E-10	2.28E-09	11.85031395
MVK	-0.370767299	3.88055934	-6.436777189	5.97E-10	2.28E-09	11.84931023
WBP4	-0.348773535	4.41903757	-6.436419697	5.98E-10	2.29E-09	11.84733123
VPS33B	-0.315459232	3.74467348	-6.435532038	6.01E-10	2.30E-09	11.84241768
ACVR1B	-0.476751538	4.31610651	-6.434441236	6.05E-10	2.31E-09	11.83638032
ZNF674	-0.297539172	1.4429031	-6.433262454	6.09E-10	2.33E-09	11.82985683

SELENO	-0.400485595	6.39572866	-6.432800728	6.11E-10	2.33E-09	11.82730183
CHRM1	-0.08076905	0.08616273	-6.432006379	6.14E-10	2.34E-09	11.82290655
WDR41	-0.361226144	4.24297074	-6.430158192	6.20E-10	2.37E-09	11.81268169
S100B	1.394820942	2.5618958	6.430043942	6.20E-10	2.37E-09	11.81204968
ZBTB3	-0.393816015	2.81345316	-6.428813955	6.25E-10	2.38E-09	11.8052462
SMAD7	-0.604513576	5.12212817	-6.427138163	6.31E-10	2.41E-09	11.79597832
SLC25A3	-0.516782323	1.49671461	-6.426404213	6.33E-10	2.42E-09	11.7919198
RIBC2	-0.518958334	1.84927073	-6.425393113	6.37E-10	2.43E-09	11.78632927
PHLDA1	-0.789268726	5.92718711	-6.424783204	6.39E-10	2.44E-09	11.78295729
C1orf116	1.239770034	4.38316259	6.424661871	6.40E-10	2.44E-09	11.78228651
PMF1-BC	-0.482808795	2.90074017	-6.423797131	6.43E-10	2.45E-09	11.77750612
EXD2	-0.421035669	3.90465304	-6.423509496	6.44E-10	2.45E-09	11.77591614
TRIM24	-0.447653404	3.50636558	-6.422992655	6.46E-10	2.46E-09	11.7730593
MYO10	-0.608739801	4.29184439	-6.422733834	6.47E-10	2.46E-09	11.77162872
BCAS4	0.664432119	3.4357639	6.421271967	6.52E-10	2.48E-09	11.76354937
MCUB	0.843348285	3.27104631	6.421193663	6.52E-10	2.48E-09	11.76311664
P2RX3	-0.171246549	0.15810601	-6.420591646	6.55E-10	2.49E-09	11.75978986
FATE1	-0.52720663	1.01593588	-6.419759619	6.58E-10	2.50E-09	11.75519241
SAXO1	-0.095823398	0.14424548	-6.419672272	6.58E-10	2.50E-09	11.75470978
TCP1	-0.376944577	6.24336882	-6.419304217	6.59E-10	2.51E-09	11.75267622
UBE2E1	-0.354959911	5.95065727	-6.418864108	6.61E-10	2.51E-09	11.75024465
FADS2	-0.605274147	2.2804647	-6.418402729	6.63E-10	2.52E-09	11.74769569
DHX40	-0.446661171	4.82140997	-6.418213657	6.63E-10	2.52E-09	11.74665117
DOLPP1	-0.419090318	4.35507899	-6.418132189	6.64E-10	2.52E-09	11.74620111
C1GALT1	-0.443866579	5.61733404	-6.417793621	6.65E-10	2.53E-09	11.74433079
RNF13	-0.502759212	5.96309459	-6.416069363	6.71E-10	2.55E-09	11.73480669
MEF2C	-0.543301694	3.65025904	-6.415478123	6.74E-10	2.56E-09	11.73154135
ALDH18A	-0.430595319	6.35969257	-6.415464965	6.74E-10	2.56E-09	11.73146868
CCDC16	-0.215147223	0.28621647	-6.415168344	6.75E-10	2.56E-09	11.72983057
INTS12	-0.383567089	4.20093775	-6.414346279	6.78E-10	2.57E-09	11.72529093
MED25	-0.402105782	5.09545919	-6.414300522	6.78E-10	2.57E-09	11.72503826
EPAS1	-0.685643677	6.88862563	-6.412065488	6.87E-10	2.61E-09	11.71269805
IRF5	0.559604296	3.16791532	6.411104367	6.91E-10	2.62E-09	11.70739241
SHANK3	-0.639897075	4.10274152	-6.410438509	6.93E-10	2.63E-09	11.70371703
PCDHAC	0.678724231	1.24939877	6.409904209	6.95E-10	2.64E-09	11.70076802
FXR2	-0.361284832	5.1077159	-6.408676891	7.00E-10	2.65E-09	11.69399465
PSME3	-0.409781251	5.53201453	-6.408114836	7.02E-10	2.66E-09	11.69089307
ATP6AP1	-0.374999711	7.35593441	-6.406757682	7.08E-10	2.68E-09	11.68340472
SUGP1	-0.343104403	4.27623954	-6.40593545	7.11E-10	2.69E-09	11.67886846
NUTM2B	-0.107483381	0.20691832	-6.40391377	7.19E-10	2.72E-09	11.66771662

MTMR10	-0.429210148	3.91683225	-6.403079417	7.23E-10	2.74E-09	11.66311497
SLC5A5	-1.414234482	1.26707717	-6.402595815	7.25E-10	2.74E-09	11.66044799
URGCP	-0.327015534	4.27493277	-6.398654403	7.41E-10	2.80E-09	11.63871722
DYM	-0.399193834	4.68280233	-6.398208103	7.43E-10	2.81E-09	11.63625718
PLA2G12	-0.423333006	4.79122033	-6.396290593	7.51E-10	2.84E-09	11.62568912
G6PD	-0.521330006	6.1853256	-6.395734473	7.53E-10	2.85E-09	11.62262458
FOLR3	-0.535379478	0.67653162	-6.394799691	7.57E-10	2.86E-09	11.61747383
ACSS2	-0.299320858	4.1149706	-6.394024013	7.61E-10	2.87E-09	11.61320018
CHPF	-0.494547032	7.16848305	-6.393979177	7.61E-10	2.87E-09	11.61295316
TUT1	-0.386071601	3.81329701	-6.393740326	7.62E-10	2.88E-09	11.61163727
NLGN2	0.558657465	3.41100811	6.393318913	7.64E-10	2.88E-09	11.6093157
SUV39H2	-0.378322359	2.44113917	-6.392398009	7.68E-10	2.90E-09	11.6042428
SRR	-0.351226376	3.01335167	-6.3920416	7.69E-10	2.90E-09	11.60227962
MCFD2	-0.612178135	6.41061594	-6.388453569	7.85E-10	2.96E-09	11.58252041
OSBPL6	-0.463168695	0.7902671	-6.386081233	7.95E-10	3.00E-09	11.56946042
TNFSF14	0.415054437	0.69912067	6.385590199	7.98E-10	3.01E-09	11.56675766
DTWD2	-0.568243874	2.87273975	-6.385290096	7.99E-10	3.01E-09	11.56510589
DBH	-0.546197629	1.08402469	-6.384573417	8.02E-10	3.03E-09	11.56116153
AIP	-0.357211872	7.39671696	-6.384388399	8.03E-10	3.03E-09	11.56014331
WDR89	-0.368466631	4.11902498	-6.38416193	8.04E-10	3.03E-09	11.55889699
PREP	-0.371380474	4.79504261	-6.383521971	8.07E-10	3.04E-09	11.55537531
SPOUT1	-0.355902212	4.44495816	-6.383206186	8.08E-10	3.05E-09	11.55363765
MAZ	-0.535295147	4.9395788	-6.381146357	8.18E-10	3.08E-09	11.54230459
TRAF6	-0.432261928	2.93371311	-6.380392385	8.21E-10	3.09E-09	11.53815694
PIFO	-0.43958033	4.02140359	-6.379329351	8.26E-10	3.11E-09	11.53230972
WDR13	-0.339025014	5.39578046	-6.378158629	8.32E-10	3.13E-09	11.52587099
GLYCTK	-0.362855614	2.4546594	-6.377873464	8.33E-10	3.14E-09	11.52430276
GP5	-0.258197057	0.36043512	-6.377199854	8.36E-10	3.15E-09	11.52059856
RNF187	-0.53498932	7.57966321	-6.377131831	8.36E-10	3.15E-09	11.52022451
GAS2L1	-0.355137396	3.85827438	-6.3770923	8.37E-10	3.15E-09	11.52000714
FRA10AC	-0.367529819	3.54416607	-6.375977489	8.42E-10	3.17E-09	11.51387744
GOLGA8	-0.121877496	0.15361709	-6.37548597	8.44E-10	3.17E-09	11.51117511
HIST1H3	-0.350392638	0.66424405	-6.375190799	8.46E-10	3.18E-09	11.50955236
GTF2H2	-0.365840486	0.94147882	-6.375151876	8.46E-10	3.18E-09	11.50933838
TERF2	-0.368849646	4.32355566	-6.374931606	8.47E-10	3.18E-09	11.50812744
RNF170	-0.47040613	4.61571663	-6.373921217	8.52E-10	3.20E-09	11.50257324
NCF2	0.874740142	3.04567672	6.372058134	8.61E-10	3.23E-09	11.49233336
TBC1D13	-0.459068643	5.46232578	-6.368399386	8.79E-10	3.30E-09	11.47223046
OSTF1	-0.378576717	6.68307061	-6.367408908	8.84E-10	3.32E-09	11.46678974
SLC5A6	-0.352554221	3.25387513	-6.365540096	8.93E-10	3.35E-09	11.45652599

TMEM23	-0.776009219	3.74012737	-6.365280103	8.94E-10	3.36E-09	11.45509825
TFPT	-0.419526306	5.3823152	-6.365236205	8.94E-10	3.36E-09	11.45485719
ENPEP	-0.70233517	2.25306801	-6.362551734	9.08E-10	3.41E-09	11.44011807
PLLP	-0.392328189	2.65288659	-6.360269334	9.20E-10	3.45E-09	11.42759008
VPS9D1	-0.344919649	4.38446335	-6.359991727	9.21E-10	3.45E-09	11.42606653
SH3BP2	0.497147296	3.23087039	6.359489559	9.24E-10	3.46E-09	11.42331068
CHRNB1	-0.488538416	4.33029678	-6.35936999	9.24E-10	3.46E-09	11.42265452
TMEM24	-0.369583615	6.6307311	-6.358908014	9.27E-10	3.47E-09	11.42011941
SH2B1	-0.405854547	5.15103022	-6.358607032	9.28E-10	3.48E-09	11.41846784
ACTR1A	-0.277454172	6.33450943	-6.358565938	9.29E-10	3.48E-09	11.41824234
ERICH2	-0.777547431	2.24414119	-6.358367878	9.30E-10	3.48E-09	11.41715556
CUX2	-1.133373658	0.95726783	-6.357306791	9.35E-10	3.50E-09	11.41133369
CCDC91	-0.411436244	3.87115479	-6.356920456	9.37E-10	3.51E-09	11.40921415
WDR5	-0.365123133	4.86501206	-6.35677634	9.38E-10	3.51E-09	11.40842351
ANKRD2	-0.468371608	1.62280302	-6.353449835	9.56E-10	3.58E-09	11.39017761
PREX2	-0.638653713	1.92722852	-6.353257617	9.57E-10	3.58E-09	11.3891235
MBD2	-0.408826074	5.75290905	-6.353021089	9.58E-10	3.58E-09	11.38782644
WWTR1	-0.504316784	4.60800041	-6.352476927	9.61E-10	3.59E-09	11.38484251
RPL11	-0.381986124	10.2316019	-6.352140226	9.63E-10	3.60E-09	11.38299629
NMU	1.852716515	3.5462007	6.350603924	9.71E-10	3.63E-09	11.37457326
MPP6	-0.494214025	2.07360473	-6.349073658	9.79E-10	3.66E-09	11.3661848
ETV7	0.871510288	2.36229176	6.348861073	9.81E-10	3.66E-09	11.36501959
NRDE2	-0.314272934	2.17431595	-6.348689476	9.82E-10	3.67E-09	11.36407906
OLFM3	-0.281908229	0.19045935	-6.348026134	9.85E-10	3.68E-09	11.36044344
TANGO6	-0.389953604	2.62456067	-6.348004492	9.85E-10	3.68E-09	11.36032482
SOX17	-0.630346582	2.54679317	-6.347737093	9.87E-10	3.68E-09	11.35885936
FAM178E	0.699882619	1.27338663	6.347605305	9.88E-10	3.68E-09	11.35813711
ZNF837	-0.487556489	2.92283248	-6.345828867	9.97E-10	3.72E-09	11.34840269
RAB40C	-0.372646387	4.18372416	-6.345367071	1.00E-09	3.73E-09	11.34587249
CLEC5A	0.675007062	1.73432978	6.345167132	1.00E-09	3.73E-09	11.34477706
NOXRED	-0.323085526	1.02070273	-6.343740047	1.01E-09	3.76E-09	11.33695903
GDAP1	-0.4492603	3.41404244	-6.342256756	1.02E-09	3.79E-09	11.32883444
SCNN1A	1.338894876	5.8728292	6.341856803	1.02E-09	3.80E-09	11.32664397
KCNJ11	-0.730243829	3.48315866	-6.341204799	1.02E-09	3.81E-09	11.32307328
ABHD17A	-0.376281489	4.01901797	-6.340987727	1.02E-09	3.82E-09	11.32188455
ZNF696	-0.368236033	2.54267117	-6.34090333	1.03E-09	3.82E-09	11.32142238
CD109	0.964710506	2.51064876	6.340720688	1.03E-09	3.82E-09	11.32042223
MAPK14	-0.481606782	5.17350156	-6.340399769	1.03E-09	3.83E-09	11.31866492
GRB7	0.877908159	4.51716106	6.340307242	1.03E-09	3.83E-09	11.31815826
WNT11	-0.806444575	1.34745191	-6.33957324	1.03E-09	3.84E-09	11.31413924

IL17RD	0.740890036	1.99775651	6.338201047	1.04E-09	3.87E-09	11.30662673
GATD1	-0.351736094	5.30605546	-6.338046274	1.04E-09	3.87E-09	11.30577944
R3HDM1	-0.368999075	3.31651502	-6.337678208	1.04E-09	3.88E-09	11.30376459
GTF3A	-0.29955665	5.89138513	-6.337517123	1.05E-09	3.88E-09	11.3028828
TMEM63	-0.582467402	1.25610237	-6.336413619	1.05E-09	3.91E-09	11.29684265
MLF1	-0.494922476	3.27597387	-6.334326828	1.06E-09	3.95E-09	11.28542245
ZNF782	-0.3664972	1.86636943	-6.334279689	1.06E-09	3.95E-09	11.28516451
NDUFAF	-0.332837678	3.31596617	-6.334198555	1.06E-09	3.95E-09	11.28472055
TIMP3	-0.92305278	2.77914453	-6.333910443	1.07E-09	3.96E-09	11.28314406
CCN4	0.642837565	0.89916861	6.331984547	1.08E-09	4.00E-09	11.27260731
PSMD11	-0.326202006	4.80801903	-6.33163265	1.08E-09	4.01E-09	11.2706823
UBLCP1	-0.411064909	4.87287401	-6.330219253	1.09E-09	4.04E-09	11.26295128
FBF1	-0.682187573	3.04246135	-6.330105875	1.09E-09	4.04E-09	11.26233117
RNF25	-0.349433266	5.09966545	-6.329674971	1.09E-09	4.05E-09	11.25997448
CPPED1	-0.406087459	3.54988613	-6.328766858	1.10E-09	4.07E-09	11.25500825
C1orf194	-0.399241059	0.40307515	-6.328492132	1.10E-09	4.08E-09	11.25350595
POP7	-0.330944275	5.90486152	-6.326703999	1.11E-09	4.12E-09	11.24372892
POLR2H	-0.380066401	5.26209186	-6.326266864	1.11E-09	4.12E-09	11.2413391
LY6G6C	1.102887092	1.7697389	6.326176366	1.11E-09	4.13E-09	11.24084436
CHRNA4	-0.523859685	0.48799377	-6.324453315	1.12E-09	4.17E-09	11.23142566
CNRIP1	-0.441328626	2.43775621	-6.323932705	1.13E-09	4.18E-09	11.22858023
RWDD3	-0.40180527	3.33308083	-6.321679423	1.14E-09	4.23E-09	11.2162667
MPP2	-0.757564832	2.63103688	-6.321498188	1.14E-09	4.23E-09	11.21527645
DHDH	0.788297295	1.89288937	6.320991343	1.15E-09	4.24E-09	11.21250719
AARSD1	-0.316866507	2.23146122	-6.320652835	1.15E-09	4.25E-09	11.21065776
GLIS3	-0.639573736	3.67978839	-6.31974321	1.15E-09	4.27E-09	11.20568842
PGA5	-0.345982828	0.34348522	-6.319398548	1.16E-09	4.28E-09	11.20380565
CPOX	-0.434970429	3.50008309	-6.319219204	1.16E-09	4.28E-09	11.20282598
CNST	-0.50265234	3.97860421	-6.318925471	1.16E-09	4.29E-09	11.2012215
CTNNBL	-0.347729948	4.83228121	-6.318783965	1.16E-09	4.29E-09	11.20044857
TNPO3	-0.412676747	5.41916655	-6.318644902	1.16E-09	4.29E-09	11.19968899
SMARCD	-0.432667373	4.97928379	-6.317686376	1.17E-09	4.31E-09	11.19445373
CCDC8	-0.917831553	5.07691019	-6.31547558	1.18E-09	4.37E-09	11.18238105
ADD3	-0.637065446	5.65648817	-6.31424385	1.19E-09	4.40E-09	11.17565617
HMGN1	-0.414913225	6.61439931	-6.313908217	1.19E-09	4.40E-09	11.17382388
NFKBIL1	-0.38942079	5.713707	-6.31384584	1.19E-09	4.40E-09	11.17348336
SH2B2	-0.486689839	3.27875401	-6.31305138	1.20E-09	4.42E-09	11.16914655
PHGDH	-0.80104909	5.1352555	-6.312363527	1.20E-09	4.44E-09	11.16539202
GPNMB	1.347763423	4.83564379	6.311657208	1.21E-09	4.46E-09	11.161537
RHOT1	-0.423382963	4.57890273	-6.310451187	1.22E-09	4.49E-09	11.15495538

C11orf80	0.579740536	3.60811933	6.309805111	1.22E-09	4.50E-09	11.15142993
ASNSD1	-0.367066636	5.55516636	-6.308803997	1.23E-09	4.52E-09	11.14596766
PIGC	-0.293415744	4.11386555	-6.306111171	1.25E-09	4.59E-09	11.13127821
UBE2J2	-0.325134143	4.8435715	-6.306095392	1.25E-09	4.59E-09	11.13119215
TRMT2B	-0.399661531	3.8099651	-6.303757459	1.26E-09	4.65E-09	11.11844239
POU2F3	0.679599761	2.25466839	6.303699298	1.26E-09	4.65E-09	11.11812526
ACTR3B	-0.281477828	2.08651109	-6.301910277	1.28E-09	4.70E-09	11.10837136
LAMB4	-0.187946246	0.20177595	-6.301499594	1.28E-09	4.71E-09	11.10613256
H3F3A	-0.422689908	4.80543854	-6.300942853	1.28E-09	4.72E-09	11.10309772
DRAM2	-0.311332821	5.15410842	-6.300412627	1.29E-09	4.73E-09	11.10020759
PLEKHA	-0.415379539	2.53143931	-6.299789919	1.29E-09	4.75E-09	11.09681359
TDRD9	-1.107804386	2.16173266	-6.299299574	1.29E-09	4.76E-09	11.0941412
ARHGAP	-0.433900162	3.3833227	-6.298902806	1.30E-09	4.77E-09	11.09197891
ADRB1	-0.365575645	0.68112164	-6.296364032	1.32E-09	4.84E-09	11.07814558
ZNF391	-0.445778295	2.28635219	-6.295711827	1.32E-09	4.85E-09	11.07459249
C14orf93	-0.350863128	3.52185104	-6.29544312	1.32E-09	4.86E-09	11.0731287
TIE1	-0.586675976	4.13042362	-6.294988867	1.33E-09	4.87E-09	11.07065425
SYCE3	-0.655627763	2.5498014	-6.29424071	1.33E-09	4.89E-09	11.06657911
ZNF512E	-0.477770387	4.45275134	-6.293156541	1.34E-09	4.92E-09	11.06067436
ZNF555	-0.321341005	1.64759048	-6.292896431	1.34E-09	4.93E-09	11.05925783
RBM28	-0.300625445	2.49434312	-6.291484084	1.35E-09	4.96E-09	11.05156706
GRAP	-0.400099575	1.04501156	-6.291228766	1.35E-09	4.97E-09	11.05017689
HESX1	-0.353119536	1.45299568	-6.291226543	1.35E-09	4.97E-09	11.05016479
VILL	-0.573408869	2.67135297	-6.290997032	1.36E-09	4.97E-09	11.04891516
CDK5RA	-0.441112103	5.91395738	-6.288582381	1.37E-09	5.04E-09	11.03577014
CLDN16	1.788613581	4.94223662	6.288441913	1.38E-09	5.04E-09	11.03500557
CTDP1	-0.314741966	3.55377844	-6.288428238	1.38E-09	5.04E-09	11.03493113
DNAJB4	-0.594225075	4.72403607	-6.288340107	1.38E-09	5.04E-09	11.03445144
MAPT	-0.531770228	0.69889236	-6.287641203	1.38E-09	5.06E-09	11.0306475
PUF60	-0.422598383	6.22414537	-6.287609002	1.38E-09	5.06E-09	11.03047224
CPO	-0.367649381	1.18573058	-6.285868875	1.40E-09	5.11E-09	11.0210026
TADA3	-0.376119911	6.52247116	-6.285834562	1.40E-09	5.11E-09	11.02081589
MYBPH	0.874533314	1.34901194	6.28459517	1.41E-09	5.14E-09	11.0140724
SPTBN4	-0.401306866	0.98455249	-6.284555029	1.41E-09	5.14E-09	11.01385401
11-Mar	0.460534448	0.46920832	6.283729757	1.41E-09	5.17E-09	11.00936429
PANK4	-0.359773749	3.99944429	-6.283568331	1.41E-09	5.17E-09	11.00848614
FOXF1	-0.597534974	1.8060779	-6.283471638	1.41E-09	5.17E-09	11.00796014
PEX14	-0.312254766	4.85657696	-6.283123145	1.42E-09	5.18E-09	11.00606442
ZNF354A	-0.32462055	3.56733228	-6.282481035	1.42E-09	5.20E-09	11.00257171
THUMPD	-0.358112783	3.03525948	-6.282381633	1.42E-09	5.20E-09	11.00203104

DNAJC2:	-0.550904025	4.39574786	-6.281245557	1.43E-09	5.23E-09	10.99585214
KDM4D	-0.255964155	1.18881589	-6.281238315	1.43E-09	5.23E-09	10.99581275
GOLPH3	-0.427677454	5.28545683	-6.280953762	1.43E-09	5.24E-09	10.99426525
SSRP1	-0.439108944	6.13102961	-6.280810762	1.43E-09	5.24E-09	10.99348759
SYT3	-0.453482836	0.51046262	-6.280225875	1.44E-09	5.26E-09	10.99030698
PLEKHG	-0.776882474	1.2013912	-6.279812933	1.44E-09	5.27E-09	10.98806153
GFER	-0.351282549	4.60013253	-6.279304344	1.45E-09	5.28E-09	10.98529614
EHD4	-0.440390689	5.14263072	-6.279275487	1.45E-09	5.28E-09	10.98513924
PWWP2I	-0.441010252	5.33228462	-6.278803884	1.45E-09	5.30E-09	10.98257511
ANAPC2	-0.371359209	4.40597217	-6.277340102	1.46E-09	5.34E-09	10.97461735
GPX8	-0.48805543	4.87417633	-6.277328398	1.46E-09	5.34E-09	10.97455372
PGAP3	-0.458956459	6.41748481	-6.277020277	1.47E-09	5.34E-09	10.97287882
AKR1C1	-0.773640764	1.02849854	-6.276530807	1.47E-09	5.36E-09	10.97021824
TOR1B	-0.390991068	4.57674473	-6.276530419	1.47E-09	5.36E-09	10.97021614
C1orf109	-0.428521121	3.23857195	-6.275531795	1.48E-09	5.39E-09	10.96478847
C9orf152	-0.358099603	0.34916908	-6.275108016	1.48E-09	5.40E-09	10.96248536
JKAMP	-0.448869604	4.7117478	-6.274424703	1.49E-09	5.42E-09	10.958772
EIF2B4	-0.313967832	4.56678218	-6.273740903	1.49E-09	5.44E-09	10.9550563
VWF	-0.887048788	5.89286521	-6.273726621	1.49E-09	5.44E-09	10.95497869
SHMT1	-0.471473067	5.01737491	-6.270605379	1.52E-09	5.53E-09	10.93802194
NAT1	-0.502345453	1.7454542	-6.270451223	1.52E-09	5.53E-09	10.93718462
EFCAB1:	-0.307989065	2.15489167	-6.269971052	1.52E-09	5.55E-09	10.93457659
VEGFC	-0.518427946	6.04551779	-6.269715303	1.53E-09	5.55E-09	10.93318756
TMEM17	-0.395215255	1.88061668	-6.269020626	1.53E-09	5.57E-09	10.92941483
RIC8A	-0.34498366	5.43301807	-6.268461934	1.54E-09	5.59E-09	10.92638084
HIST1H2	-0.76886557	5.46524443	-6.268037547	1.54E-09	5.60E-09	10.92407633
SLC1A2	-0.270236565	0.29839639	-6.267847607	1.54E-09	5.61E-09	10.92304496
CREB3L4	-0.461642749	4.10117071	-6.267504413	1.55E-09	5.62E-09	10.92118147
DRAP1	-0.456851648	8.12139042	-6.267004388	1.55E-09	5.63E-09	10.91846654
NPTXR	0.72720995	4.54422935	6.265966299	1.56E-09	5.66E-09	10.91283067
TENM2	-0.46836373	0.43722967	-6.264972397	1.57E-09	5.69E-09	10.90743533
DAND5	-0.374293437	0.49157321	-6.263743394	1.58E-09	5.73E-09	10.90076462
PTGES3	-0.4304333	7.72949084	-6.259884666	1.61E-09	5.85E-09	10.8798267
MED6	-0.340606566	3.76432891	-6.259831136	1.61E-09	5.85E-09	10.8795363
DEAF1	-0.424590681	5.39737411	-6.259493877	1.62E-09	5.86E-09	10.87770676
CIR1	-0.34428783	5.69205522	-6.259445144	1.62E-09	5.86E-09	10.8774424
WDR34	-0.433816886	6.9698318	-6.259321017	1.62E-09	5.87E-09	10.87676906
RIMKLB	-0.461246257	3.68750335	-6.259022594	1.62E-09	5.87E-09	10.87515029
PLPP3	-0.879878037	8.68364966	-6.258914441	1.62E-09	5.88E-09	10.87456363
CENPBD	-0.49431358	2.77066725	-6.258092975	1.63E-09	5.90E-09	10.87010799

SSR1	-0.398496195	5.33058041	-6.256811109	1.64E-09	5.94E-09	10.86315598
ELOF1	-0.321268334	5.37088774	-6.25679367	1.64E-09	5.94E-09	10.86306141
PNPO	-0.568926714	5.02181647	-6.255248901	1.65E-09	5.99E-09	10.854685
TRIM5	-0.465581997	4.21305274	-6.254106664	1.66E-09	6.03E-09	10.84849226
CSGALN	-0.846170701	4.64910304	-6.253648504	1.67E-09	6.04E-09	10.84600855
RHOT2	-0.406825917	5.29074909	-6.252950449	1.68E-09	6.07E-09	10.84222459
ZNF773	-0.293982187	1.95851765	-6.252318738	1.68E-09	6.09E-09	10.83880054
CFAP47	-0.181529567	0.13586471	-6.251741125	1.69E-09	6.10E-09	10.83566994
SRRT	-0.372287813	5.88711556	-6.25138225	1.69E-09	6.12E-09	10.83372498
FOXC1	-0.639050451	2.83630744	-6.250693126	1.70E-09	6.14E-09	10.82999043
OXA1L	-0.274642026	6.44517584	-6.250066845	1.70E-09	6.16E-09	10.82659671
KCNJ2	1.152052413	3.33634533	6.248098603	1.72E-09	6.22E-09	10.81593273
METTL4	-0.346949542	2.68567178	-6.246527546	1.74E-09	6.28E-09	10.80742248
ZKSCAN	-0.446604298	1.88774584	-6.243917748	1.76E-09	6.37E-09	10.79328894
RERGL	-0.642638771	1.00796899	-6.242694882	1.77E-09	6.41E-09	10.78666793
PFKFB2	-0.79707406	4.48946906	-6.242375811	1.78E-09	6.42E-09	10.78494052
ZNF518E	-0.564122081	2.98185712	-6.24165556	1.78E-09	6.44E-09	10.78104143
MAP3K1(	-0.326781716	2.8694185	-6.240801371	1.79E-09	6.47E-09	10.77641769
BSCL2	-0.40789569	3.0175902	-6.240428183	1.80E-09	6.49E-09	10.77439776
FREM2	-0.797228851	2.57833008	-6.239639077	1.80E-09	6.51E-09	10.77012691
HS6ST3	-0.701006231	1.02236419	-6.239434702	1.81E-09	6.52E-09	10.76902084
ELFN2	0.580592225	0.51254681	6.238541077	1.82E-09	6.55E-09	10.76418491
METAP1	-0.34358368	1.85273427	-6.238128001	1.82E-09	6.56E-09	10.76194968
GALC	-0.493951286	4.09668507	-6.238024618	1.82E-09	6.57E-09	10.76139028
PPP1R1(	-0.460150576	5.82184519	-6.235690522	1.84E-09	6.65E-09	10.74876228
KAT7	-0.434972787	4.21897151	-6.235308902	1.85E-09	6.66E-09	10.74669795
HIF3A	-0.397524693	0.72162957	-6.235217077	1.85E-09	6.66E-09	10.74620125
ZNF785	-0.379778583	3.20507778	-6.234768509	1.85E-09	6.68E-09	10.74377493
SLC25A1	-0.411746937	6.69856976	-6.233402657	1.87E-09	6.73E-09	10.73638776
CLTCL1	-0.398357977	1.88276807	-6.233397407	1.87E-09	6.73E-09	10.73635937
RHOB	-0.772798304	8.00680792	-6.231409962	1.89E-09	6.80E-09	10.72561248
MXRA7	-0.675228866	6.48561108	-6.230998161	1.89E-09	6.81E-09	10.72338602
GPR4	-0.638424241	3.89648533	-6.230431615	1.90E-09	6.83E-09	10.72032309
PCDH9	0.342722555	0.45393196	6.228942192	1.91E-09	6.89E-09	10.71227178
VEZT	-0.471498827	5.08704126	-6.228722239	1.92E-09	6.90E-09	10.71108291
SZRD1	-0.33405695	6.50145075	-6.228703752	1.92E-09	6.90E-09	10.71098299
UBE2K	-0.405436722	5.50187203	-6.22812454	1.92E-09	6.92E-09	10.70785243
YWHAB	-0.406078312	7.90047617	-6.22795803	1.92E-09	6.92E-09	10.70695251
SDK2	-0.645516874	1.10142401	-6.226490655	1.94E-09	6.98E-09	10.69902269
ZFAND4	-0.370541789	2.54677415	-6.22645871	1.94E-09	6.98E-09	10.69885007

ZNF653	-0.365008418	3.0257536	-6.225533291	1.95E-09	7.01E-09	10.69384976
IGFBP7	-0.635320612	9.13588468	-6.225080203	1.96E-09	7.03E-09	10.69140178
CNOT3	-0.379465466	4.53390434	-6.224870459	1.96E-09	7.03E-09	10.69026861
CH25H	1.117803749	2.78993525	6.224867215	1.96E-09	7.03E-09	10.69025109
HPRT1	-0.314516525	5.79619462	-6.22478572	1.96E-09	7.03E-09	10.6898108
MUC16	0.481497748	0.40076884	6.224369733	1.96E-09	7.05E-09	10.68756348
GOLGA8	-0.183777501	0.25801821	-6.224131456	1.97E-09	7.06E-09	10.68627626
TNFRSF:	0.95155947	4.5595924	6.221799765	1.99E-09	7.15E-09	10.67368194
RIPK1	-0.39928079	4.85683518	-6.221027484	2.00E-09	7.18E-09	10.66951134
FBXO24	-0.235620216	0.63575464	-6.220515061	2.01E-09	7.19E-09	10.66674427
GUF1	-0.350251068	4.00843237	-6.22007588	2.01E-09	7.21E-09	10.66437284
IYD	-1.387232584	7.25137968	-6.218931805	2.02E-09	7.25E-09	10.65819581
TBP	-0.345232397	4.34953458	-6.218413602	2.03E-09	7.27E-09	10.65539822
CEP63	-0.338098827	3.33176323	-6.217401004	2.04E-09	7.31E-09	10.64993209
INKA1	-0.620990829	2.78336304	-6.217319325	2.04E-09	7.32E-09	10.6494912
TRAPPC	-0.31134706	3.23127244	-6.216784161	2.05E-09	7.34E-09	10.64660261
LTO1	-0.329917564	2.73446756	-6.215644086	2.06E-09	7.38E-09	10.64044957
THG1L	-0.383890292	4.16337326	-6.21547854	2.06E-09	7.39E-09	10.63955618
LRRCC1	-0.500599365	3.43405675	-6.215383539	2.06E-09	7.39E-09	10.6390435
PRKAB1	-0.392786949	4.58270276	-6.214175056	2.08E-09	7.44E-09	10.63252236
GP9	-0.186828565	0.19790528	-6.21406689	2.08E-09	7.44E-09	10.63193873
CLEC1A	-0.447859096	1.89116584	-6.213816501	2.08E-09	7.45E-09	10.63058773
TARSL2	-0.367290948	3.58641852	-6.213807624	2.08E-09	7.45E-09	10.63053984
MAN1B1	-0.399254606	5.08321289	-6.213063835	2.09E-09	7.48E-09	10.6265269
LYSMD2	-0.472446003	3.91500613	-6.212436401	2.10E-09	7.50E-09	10.62314199
BORCS8	-0.293836035	1.08638557	-6.211873687	2.10E-09	7.52E-09	10.62010646
TSPAN1!	-0.123390926	0.08409622	-6.209111957	2.14E-09	7.64E-09	10.60521137
ASGR1	-0.668730702	2.01146311	-6.206755077	2.16E-09	7.73E-09	10.59250366
E2F5	-0.372632812	2.66948475	-6.20561172	2.18E-09	7.78E-09	10.58634025
SHROOM	0.812675993	3.97164766	6.205307899	2.18E-09	7.79E-09	10.58470261
RPS17	-0.39896168	8.90239519	-6.204462499	2.19E-09	7.83E-09	10.58014607
BANF2	-0.366399004	0.28509814	-6.203528244	2.20E-09	7.87E-09	10.57511115
BEX4	-0.517646962	8.01474073	-6.203021629	2.21E-09	7.89E-09	10.57238112
PRKCZ	-0.535266479	3.90982513	-6.20265169	2.21E-09	7.90E-09	10.57038772
EXTL3	-0.372529862	3.43040373	-6.202351567	2.22E-09	7.91E-09	10.56877057
LIPJ	-0.142087948	0.14319002	-6.202319847	2.22E-09	7.91E-09	10.56859966
TONSL	-0.423391909	2.40540331	-6.202157675	2.22E-09	7.92E-09	10.56772586
PDF	-0.480961041	2.68641787	-6.201549321	2.23E-09	7.94E-09	10.56444813
ZNF641	-0.391409038	3.42133963	-6.200352227	2.24E-09	7.99E-09	10.55799906
CDH13	-0.639478773	2.58804529	-6.199977324	2.25E-09	8.01E-09	10.55597954

VTI1A	-0.312334575	2.98220101	-6.199470056	2.25E-09	8.03E-09	10.55324715
C3orf62	-0.328802058	3.0566958	-6.199001067	2.26E-09	8.05E-09	10.55072109
JAM2	-0.578046137	3.10704949	-6.198840624	2.26E-09	8.05E-09	10.54985695
RASSF9	-0.465721299	1.0429557	-6.197958271	2.27E-09	8.09E-09	10.54510491
PSMA6	-0.387525445	3.12876157	-6.195644044	2.30E-09	8.19E-09	10.53264369
HMBOX1	-0.343396768	2.90522227	-6.189312539	2.38E-09	8.48E-09	10.49856852
CTDSP1	-0.388375838	7.00841846	-6.189011099	2.39E-09	8.50E-09	10.49694686
TSGA10I	-0.917322761	1.60536345	-6.188886696	2.39E-09	8.50E-09	10.49627762
CXCL2	1.390349098	3.67403177	6.188882393	2.39E-09	8.50E-09	10.49625448
NUAK2	-0.644794753	4.33148421	-6.187984686	2.40E-09	8.54E-09	10.49142549
MRPS23	-0.368090661	4.81608728	-6.187946774	2.40E-09	8.54E-09	10.49122156
SLC17A9	0.711426553	1.88314064	6.187885504	2.40E-09	8.54E-09	10.49089199
SPRTN	-0.343538278	2.34011702	-6.187287949	2.41E-09	8.57E-09	10.48767793
SLC4A1A	-0.355353632	4.26795978	-6.1870992	2.41E-09	8.57E-09	10.48666275
SETDB2	-0.378956649	3.15789596	-6.18622445	2.42E-09	8.61E-09	10.48195824
TFF1	-0.661264866	0.57489014	-6.18459007	2.45E-09	8.69E-09	10.47316969
BABAM2	-0.350513394	4.99549957	-6.183568732	2.46E-09	8.74E-09	10.46767852
ADGRD1	-0.794322325	2.60245795	-6.182840702	2.47E-09	8.77E-09	10.46376471
KIF5C	-0.717886612	1.32429216	-6.182089954	2.48E-09	8.80E-09	10.45972913
SH3BP5	-0.724007675	3.24383218	-6.18180683	2.48E-09	8.82E-09	10.45820732
PLPP6	-0.401795497	3.90578728	-6.181587271	2.49E-09	8.83E-09	10.4570272
PRORP	-0.284777564	1.80282478	-6.180856259	2.50E-09	8.86E-09	10.45309829
SMYD2	-0.38795559	4.39815192	-6.179825933	2.51E-09	8.91E-09	10.44756128
GADD45	-0.511098503	6.55923317	-6.179530523	2.52E-09	8.92E-09	10.44597385
ABCA8	-0.755106008	1.88108244	-6.177482665	2.54E-09	9.02E-09	10.43497099
TTC9B	-0.240933461	0.41848228	-6.177199559	2.55E-09	9.03E-09	10.43345011
XPO6	-0.342173134	5.14460645	-6.175623689	2.57E-09	9.11E-09	10.42498529
EIF2B1	-0.292980448	5.06388681	-6.175330518	2.57E-09	9.12E-09	10.42341069
TBCC	-0.362355982	5.63661645	-6.174867287	2.58E-09	9.14E-09	10.42092283
RPP30	-0.247442715	3.6052046	-6.17441877	2.59E-09	9.16E-09	10.41851412
XKR4	0.177365114	0.15821909	6.173774031	2.60E-09	9.19E-09	10.41505185
ARPC3	-0.283888526	7.473598	-6.171748278	2.63E-09	9.30E-09	10.40417523
CAPN7	-0.479797272	4.65656213	-6.170154502	2.65E-09	9.38E-09	10.39561983
UBE2Q2	-0.449630405	5.20298734	-6.170039843	2.65E-09	9.38E-09	10.3950044
USP39	-0.338355476	5.29808455	-6.168634671	2.67E-09	9.45E-09	10.38746289
ECHDC1	-0.393703214	4.9638272	-6.167838861	2.68E-09	9.49E-09	10.38319237
RHEB	-0.410180369	5.92608503	-6.167330102	2.69E-09	9.51E-09	10.38046245
CHCHD3	-0.45762658	5.25920662	-6.167119813	2.69E-09	9.52E-09	10.37933412
VANGL1	-0.524195406	2.87304553	-6.165841051	2.71E-09	9.59E-09	10.3724734
KCNRG	-0.189650621	0.56529052	-6.165350784	2.72E-09	9.61E-09	10.36984334

TM9SF1	-0.413987088	3.07531327	-6.164811703	2.73E-09	9.64E-09	10.36695158
PI4K2B	-0.411566845	3.15610478	-6.164357811	2.73E-09	9.66E-09	10.36451694
C3orf70	-0.442727633	1.47632141	-6.164335812	2.74E-09	9.66E-09	10.36439895
UBIAD1	-0.363762552	3.7650407	-6.163633423	2.75E-09	9.70E-09	10.36063167
GCLC	-0.570301435	3.99259679	-6.163240137	2.75E-09	9.71E-09	10.35852241
OLA1	-0.359303089	5.29561879	-6.163214772	2.75E-09	9.71E-09	10.35838638
TIMM44	-0.332891272	4.67764587	-6.163211992	2.75E-09	9.71E-09	10.35837147
FMO4	-0.358716631	2.7821878	-6.16259279	2.76E-09	9.74E-09	10.3550508
KRT15	0.715473652	1.30484137	6.162136575	2.77E-09	9.77E-09	10.35260436
DNTTIP2	-0.328598064	4.51140526	-6.161562071	2.78E-09	9.79E-09	10.34952378
PPP6C	-0.379703378	5.74834619	-6.15969605	2.81E-09	9.89E-09	10.33951938
ZHX3	-0.51624825	3.72329904	-6.159649047	2.81E-09	9.89E-09	10.33926741
CCL18	1.647685074	3.99184524	6.159528374	2.81E-09	9.90E-09	10.33862052
IQGAP3	0.484563962	1.13204126	6.159321395	2.81E-09	9.91E-09	10.33751099
TMEM18	-0.358278458	3.62900412	-6.158792024	2.82E-09	9.93E-09	10.33467338
AKAP7	-0.432182851	2.59609085	-6.1585815	2.82E-09	9.94E-09	10.33354495
C1orf52	-0.350597959	3.58708096	-6.158419287	2.83E-09	9.95E-09	10.33267549
POFUT1	-0.423912895	4.99288011	-6.158234549	2.83E-09	9.96E-09	10.33168532
CD74	0.94163567	10.5698719	6.157079574	2.85E-09	1.00E-08	10.3254953
FBXW10	-0.209881379	0.20826024	-6.156865995	2.85E-09	1.00E-08	10.32435073
RPS21	-0.46762689	9.57250263	-6.154627093	2.89E-09	1.02E-08	10.31235422
DAPP1	0.62853616	1.13036877	6.153376099	2.90E-09	1.02E-08	10.30565254
SENP3	-0.350686371	4.21868639	-6.152181125	2.92E-09	1.03E-08	10.29925191
LRG1	1.166836618	2.92381838	6.151940693	2.93E-09	1.03E-08	10.2979642
SLC10A7	-0.367700319	2.09209543	-6.150994576	2.94E-09	1.03E-08	10.29289731
PRMT5	-0.608754596	5.14419971	-6.150011632	2.96E-09	1.04E-08	10.28763382
GYG2	-0.593672279	1.56302189	-6.149994674	2.96E-09	1.04E-08	10.28754302
PALM	0.960719747	4.79744792	6.149727417	2.96E-09	1.04E-08	10.28611202
LRWD1	-0.329798023	3.49316475	-6.14926448	2.97E-09	1.04E-08	10.28363337
HOMER1	-0.596513055	3.88395647	-6.14879759	2.98E-09	1.05E-08	10.2811337
HTR1E	-0.315259697	0.27399869	-6.148756093	2.98E-09	1.05E-08	10.28091154
C6orf118	-0.16460234	0.13677015	-6.14747176	3.00E-09	1.05E-08	10.27403614
AOC3	-0.745116064	3.71398962	-6.146505626	3.02E-09	1.06E-08	10.26886485
TARS	-0.447095609	5.05350602	-6.146499392	3.02E-09	1.06E-08	10.26883148
HAUS7	-0.383397213	1.37153117	-6.146339957	3.02E-09	1.06E-08	10.26797816
MCRIP1	-0.406954586	6.26992377	-6.145917884	3.03E-09	1.06E-08	10.26571922
DFFA	-0.316070212	4.39049036	-6.145652726	3.03E-09	1.06E-08	10.26430016
PXYLP1	-0.675066303	3.22587939	-6.14490595	3.04E-09	1.07E-08	10.26030383
ZNF414	-0.37917682	4.11824138	-6.144889913	3.04E-09	1.07E-08	10.26021801
JMJD6	-0.351145745	3.37996149	-6.144547986	3.05E-09	1.07E-08	10.25838833

C5orf47	-0.142241227	0.17668408	-6.144351761	3.05E-09	1.07E-08	10.25733834
CHCHD7	-0.340607017	4.13592783	-6.144326092	3.05E-09	1.07E-08	10.25720099
CNIH1	-0.393780388	5.19071215	-6.143406555	3.07E-09	1.07E-08	10.25228097
MPP5	-0.543065884	4.34775784	-6.143380437	3.07E-09	1.07E-08	10.25214123
AKAP10	-0.457201508	4.23958381	-6.143016203	3.08E-09	1.08E-08	10.25019254
PLA2G2E	0.718988413	0.81316294	6.143007474	3.08E-09	1.08E-08	10.25014584
SLC35F5	-0.562772361	4.83753627	-6.142670625	3.08E-09	1.08E-08	10.24834374
EVA1B	-0.70860667	5.00719375	-6.141972821	3.09E-09	1.08E-08	10.24461081
COG3	-0.436692106	4.18456641	-6.141144749	3.11E-09	1.09E-08	10.24018141
ONECUT	-0.083359844	0.08999092	-6.139386034	3.14E-09	1.10E-08	10.23077543
SEC61A1	-0.37697208	7.75002486	-6.138960303	3.14E-09	1.10E-08	10.22849884
ZNF75A	-0.407599504	3.04432602	-6.138614975	3.15E-09	1.10E-08	10.22665228
BVES	-0.48019208	0.9162086	-6.137792197	3.16E-09	1.11E-08	10.22225298
GYS1	-0.307442825	5.12073306	-6.137633501	3.17E-09	1.11E-08	10.22140451
CRYZ	-0.519414389	5.19342953	-6.135891832	3.20E-09	1.12E-08	10.21209364
HS1BP3	-0.326571325	3.90007391	-6.135345324	3.21E-09	1.12E-08	10.20917244
ANKS1B	-0.385924976	0.92033994	-6.133377315	3.24E-09	1.13E-08	10.19865463
LCP1	1.043370402	4.9128451	6.132775646	3.25E-09	1.14E-08	10.19543957
EGLN1	-0.434715441	4.5007841	-6.132669777	3.25E-09	1.14E-08	10.19487388
WDR70	-0.304182424	3.4991602	-6.129858616	3.31E-09	1.15E-08	10.17985556
NR2F6	-0.428444665	5.99565732	-6.128933928	3.32E-09	1.16E-08	10.17491664
VTA1	-0.376271645	3.79322242	-6.128810414	3.32E-09	1.16E-08	10.17425697
CNOT4	-0.360202311	3.60974604	-6.127927322	3.34E-09	1.17E-08	10.1695408
NME1-NM	-0.365716649	1.54562116	-6.127676306	3.34E-09	1.17E-08	10.16820034
CLN6	-0.320261134	4.81786163	-6.127457863	3.35E-09	1.17E-08	10.16703386
ZNF284	-0.252398254	1.51569722	-6.12601309	3.38E-09	1.18E-08	10.15931956
ARMCX5	-0.403141476	3.57210054	-6.124803097	3.40E-09	1.18E-08	10.1528599
ASPHD1	0.843541433	1.75137541	6.12457872	3.40E-09	1.19E-08	10.15166214
RSPH4A	-0.448260375	0.95469639	-6.124418313	3.41E-09	1.19E-08	10.15080589
STK11IP	-0.395433127	3.69702213	-6.124075999	3.41E-09	1.19E-08	10.14897867
THAP6	-0.34305608	2.94590962	-6.123474064	3.42E-09	1.19E-08	10.14576582
SHLD2	-0.423886861	4.05419343	-6.122464211	3.44E-09	1.20E-08	10.14037621
KLK6	1.267175556	1.42441507	6.122448832	3.44E-09	1.20E-08	10.14029414
RTCA	-0.298528675	4.83227834	-6.122336476	3.44E-09	1.20E-08	10.13969454
MPHOSP	-0.35857661	4.55541736	-6.121799193	3.45E-09	1.20E-08	10.13682737
PIWIL1	-1.331769123	2.19720192	-6.121501089	3.46E-09	1.20E-08	10.13523663
PCMTD1	-0.473206654	5.14621416	-6.12093679	3.47E-09	1.21E-08	10.1322256
FOXE1	-0.772398778	7.8440808	-6.120583946	3.48E-09	1.21E-08	10.13034297
POLD4	-0.35506903	4.24981951	-6.11977564	3.49E-09	1.21E-08	10.12603049
SMCO4	-0.478205765	5.87009067	-6.119327899	3.50E-09	1.22E-08	10.12364188

OTOR	-0.099548292	0.11338429	-6.119287696	3.50E-09	1.22E-08	10.12342742
DRC1	-0.287590478	0.28250678	-6.119162457	3.50E-09	1.22E-08	10.12275932
GPATCH	-0.323871785	3.26045283	-6.118809919	3.51E-09	1.22E-08	10.12087872
ZNF540	-0.476510491	3.02299651	-6.118269708	3.52E-09	1.22E-08	10.11799716
SLC2A9	-0.398436919	1.52184361	-6.11715764	3.54E-09	1.23E-08	10.11206583
PDCD10	-0.42726862	4.66681329	-6.11638969	3.56E-09	1.24E-08	10.10797035
USP2	-0.605732133	2.18866323	-6.113764538	3.61E-09	1.25E-08	10.09397331
PIGB	-0.413734169	3.43328922	-6.113041866	3.62E-09	1.26E-08	10.09012088
CFTR	0.291500705	0.30562101	6.112600719	3.63E-09	1.26E-08	10.08776937
FOCAD	-0.471531841	4.32900526	-6.112289234	3.64E-09	1.26E-08	10.0861091
TNFSF11	0.542210358	0.6563332	6.112186227	3.64E-09	1.26E-08	10.08556006
KIAA119	-0.429589289	6.74928863	-6.110901688	3.67E-09	1.27E-08	10.07871398
FKRP	-0.342712316	3.51909098	-6.110745465	3.67E-09	1.27E-08	10.07788145
CD300LF	0.642508119	1.67261	6.110646034	3.67E-09	1.27E-08	10.07735157
BRD3OS	-0.428791492	4.14402428	-6.109792524	3.69E-09	1.28E-08	10.07280343
HNRNPD	-0.339684474	6.25281058	-6.109786655	3.69E-09	1.28E-08	10.07277216
TGFBR2	-0.676928392	6.04883463	-6.108969527	3.71E-09	1.28E-08	10.06841834
TBC1D2	0.831081715	5.78144658	6.108692066	3.71E-09	1.29E-08	10.06694007
AP1B1	-0.357334064	5.770366	-6.108489957	3.72E-09	1.29E-08	10.06586329
RPL30	-0.37802799	8.74067032	-6.108477192	3.72E-09	1.29E-08	10.06579529
CCAR2	-0.326635323	5.21122144	-6.10794348	3.73E-09	1.29E-08	10.06295197
C11orf68	-0.379335788	6.5416409	-6.107728383	3.73E-09	1.29E-08	10.06180611
METTL2B	-0.304914008	3.38587284	-6.107644477	3.73E-09	1.29E-08	10.06135913
SSR2	-0.333579478	6.06831436	-6.106158011	3.76E-09	1.30E-08	10.05344133
KNSTRN	-0.669896972	4.29724731	-6.10477061	3.79E-09	1.31E-08	10.04605251
ZNF813	-0.349231515	2.08745468	-6.10417272	3.80E-09	1.32E-08	10.04286874
MON1B	-0.381151932	4.6604418	-6.103994266	3.81E-09	1.32E-08	10.04191851
ALG13	-0.383218799	3.56949434	-6.102166412	3.85E-09	1.33E-08	10.03218682
COG4	-0.35792639	5.52746453	-6.10175522	3.85E-09	1.33E-08	10.02999789
DLST	-0.430297034	6.20410182	-6.101603652	3.86E-09	1.33E-08	10.02919106
FAM124A	-0.526243743	1.11822114	-6.100428467	3.88E-09	1.34E-08	10.02293583
TMX1	-0.461460361	4.8954051	-6.100322091	3.88E-09	1.34E-08	10.02236966
PYROXD	-0.322461031	3.35730018	-6.097345491	3.95E-09	1.36E-08	10.00653013
PRXL2B	-0.409776844	4.47084141	-6.096779128	3.96E-09	1.37E-08	10.00351697
SERTM1	-1.138479878	1.38598705	-6.09664752	3.96E-09	1.37E-08	10.00281682
FGFR1O	-0.351930543	2.07243641	-6.095912497	3.98E-09	1.37E-08	9.998906729
KANSL2	-0.389392473	4.50876425	-6.09521048	3.99E-09	1.38E-08	9.995172545
U2AF2	-0.36063358	6.74600669	-6.093897847	4.02E-09	1.39E-08	9.988191221
PGAM1	-0.543540789	4.71200706	-6.09301312	4.04E-09	1.39E-08	9.98348638
RNPS1	-0.314169936	5.20711475	-6.092771754	4.05E-09	1.40E-08	9.982202921

GALK1	-0.379312729	3.85950911	-6.092180069	4.06E-09	1.40E-08	9.979056805
C1orf43	-0.386025635	7.83691226	-6.091754418	4.07E-09	1.40E-08	9.976793668
CDC42	-0.367977118	7.39169638	-6.091597919	4.07E-09	1.40E-08	9.975961611
WDR60	-0.429195731	3.51250511	-6.08991579	4.11E-09	1.42E-08	9.967019256
BCKDHA	-0.547847932	2.93178759	-6.089249725	4.13E-09	1.42E-08	9.963478906
PPP1R3I	-0.372713449	3.14432178	-6.088940991	4.13E-09	1.42E-08	9.961837981
TAF10	-0.340512065	5.08027379	-6.087758558	4.16E-09	1.43E-08	9.95555391
ZBED9	-0.371584964	1.5810381	-6.086370387	4.19E-09	1.44E-08	9.948177599
MAN2B2	-0.460609256	5.19500623	-6.083265471	4.26E-09	1.47E-08	9.931683587
POMC	-0.740086506	1.52441494	-6.082351835	4.29E-09	1.47E-08	9.926831347
ICAM2	-0.51551424	3.41495804	-6.081404994	4.31E-09	1.48E-08	9.921803326
CTHRC1	1.273615156	3.34315574	6.080358448	4.33E-09	1.49E-08	9.916246521
ZDHHC3	-0.421799763	4.79399658	-6.080216442	4.34E-09	1.49E-08	9.915492574
RAB4B	-0.363908001	3.60134322	-6.079872094	4.34E-09	1.49E-08	9.913664391
ZNF324	-0.314602148	2.87102075	-6.078807041	4.37E-09	1.50E-08	9.908010396
SND1	-0.3995708	6.63758673	-6.078376402	4.38E-09	1.50E-08	9.905724488
SLC19A1	-0.587722392	1.84240293	-6.07753131	4.40E-09	1.51E-08	9.901238954
PARS2	-0.388214834	3.0640905	-6.077133399	4.41E-09	1.51E-08	9.8991271
PPM1H	-0.554516182	2.7255731	-6.076009556	4.44E-09	1.52E-08	9.893163036
GANAB	-0.47946865	7.9920414	-6.075793051	4.44E-09	1.53E-08	9.892014169
KCP	0.772634288	1.818186	6.075696727	4.44E-09	1.53E-08	9.891503046
AFTPH	-0.342397411	4.52509344	-6.075199866	4.46E-09	1.53E-08	9.888866644
AANAT	-0.147663392	0.26737244	-6.074619593	4.47E-09	1.53E-08	9.885787851
KIF1BP	-0.453013383	5.31039901	-6.07461793	4.47E-09	1.53E-08	9.885779028
SLC35B2	-0.404918708	6.30529279	-6.074550748	4.47E-09	1.53E-08	9.885422593
ENPP5	-0.561941102	5.80529115	-6.073575437	4.49E-09	1.54E-08	9.88024836
NOMO1	-0.364116212	4.49981774	-6.073132359	4.51E-09	1.55E-08	9.877897938
CD81	-0.394029075	7.84773592	-6.071968758	4.53E-09	1.55E-08	9.871725938
AATF	-0.307929006	5.08586252	-6.071789281	4.54E-09	1.56E-08	9.87077403
CHMP5	-0.425031881	6.78529205	-6.071754993	4.54E-09	1.56E-08	9.870592176
FBXW4	-0.306132206	5.752061	-6.071025705	4.56E-09	1.56E-08	9.866724419
SNAP29	-0.323317391	4.47461337	-6.070779441	4.56E-09	1.56E-08	9.865418445
SESN1	-0.419320831	5.12563853	-6.070177861	4.58E-09	1.57E-08	9.862228343
HK3	0.740332904	1.84472317	6.069991199	4.58E-09	1.57E-08	9.861238548
SLC4A5	-0.891569585	3.05464967	-6.06889863	4.61E-09	1.58E-08	9.855445528
SLC25A4	-0.39779536	4.03812673	-6.068737744	4.61E-09	1.58E-08	9.854592548
SFRP1	-1.578204904	2.47876727	-6.068541171	4.62E-09	1.58E-08	9.853550381
ZNF563	-0.358299163	2.95280827	-6.0680481	4.63E-09	1.58E-08	9.850936391
KCNN2	-0.223779478	0.62445761	-6.06779471	4.64E-09	1.59E-08	9.84959312
C6orf223	-0.357046551	0.73677885	-6.067347409	4.65E-09	1.59E-08	9.847221987

ADGRE3	0.15687701	0.20053608	6.064868746	4.71E-09	1.61E-08	9.834085028
IDH2	-0.410524055	5.80611595	-6.062102873	4.78E-09	1.64E-08	9.819430588
GPAT4	-0.308147934	4.69491527	-6.060056784	4.84E-09	1.65E-08	9.808592997
CUL5	-0.44047402	4.24536493	-6.059753151	4.85E-09	1.66E-08	9.806984967
TMEM12	-0.601712492	7.67016	-6.059734136	4.85E-09	1.66E-08	9.806884268
USP48	-0.374247213	4.31373166	-6.059497553	4.85E-09	1.66E-08	9.80563138
DGKE	-0.331201985	1.94552642	-6.058747172	4.87E-09	1.66E-08	9.801657771
IRF2BP2	-0.464474033	6.87049313	-6.057144232	4.92E-09	1.68E-08	9.793170712
PSD4	-0.464990179	4.41432972	-6.056893414	4.92E-09	1.68E-08	9.791842861
FAM181/	-0.738968548	1.74019108	-6.056754778	4.93E-09	1.68E-08	9.791108926
ERGIC2	-0.324627937	4.48560925	-6.052861571	5.03E-09	1.72E-08	9.770503617
SDCBP2	-0.617203318	4.34991681	-6.052630576	5.04E-09	1.72E-08	9.76928136
NSUN6	-0.391049965	3.1886558	-6.052309648	5.05E-09	1.72E-08	9.767583297
BIRC7	1.396236593	2.26927051	6.051651676	5.06E-09	1.73E-08	9.76410211
PMAIP1	0.869209574	2.73209912	6.051534198	5.07E-09	1.73E-08	9.763480591
DCBLD1	-0.480857166	2.77816589	-6.051223705	5.08E-09	1.73E-08	9.761837962
LZIC	-0.434834504	4.49527923	-6.050467616	5.10E-09	1.74E-08	9.757838222
BBS12	-0.46066175	3.80580999	-6.050317861	5.10E-09	1.74E-08	9.757046057
TMEM16	-0.69193445	4.61653355	-6.048689011	5.15E-09	1.75E-08	9.748430805
PTGR1	-0.557346636	4.97422762	-6.04836473	5.16E-09	1.76E-08	9.74671584
RPS25	-0.370593839	10.0293445	-6.045723447	5.23E-09	1.78E-08	9.732749926
FNTA	-0.314212039	4.22348856	-6.045703471	5.23E-09	1.78E-08	9.732644323
RHOC	-0.363531387	7.18452578	-6.045411199	5.24E-09	1.78E-08	9.731099204
ITGA2B	-0.42012688	1.15214618	-6.044422509	5.27E-09	1.79E-08	9.725872834
SDC4	1.011350849	9.41170425	6.04389192	5.28E-09	1.80E-08	9.723068321
SORT1	-0.428972654	6.09534716	-6.042668049	5.32E-09	1.81E-08	9.716600062
MB	-0.736815666	1.14413553	-6.041896018	5.34E-09	1.82E-08	9.712520316
GUSB	-0.326799828	6.06121803	-6.041875408	5.34E-09	1.82E-08	9.712411412
TGFBR3I	0.632714269	1.05339886	6.041516252	5.35E-09	1.82E-08	9.710513616
COA7	-0.393765621	3.83420058	-6.041454965	5.35E-09	1.82E-08	9.71018978
PRICKLE	0.837456083	3.72779597	6.038481573	5.44E-09	1.85E-08	9.69448161
TAOK2	-0.360000399	4.73424361	-6.03821292	5.45E-09	1.85E-08	9.693062627
GRWD1	-0.339158388	4.35933598	-6.036698426	5.49E-09	1.87E-08	9.685064183
P2RX4	-0.345668451	3.60562002	-6.036247397	5.51E-09	1.87E-08	9.682682469
SPOCD1	0.505573194	0.72486888	6.036033371	5.51E-09	1.87E-08	9.681552325
FAM133E	-0.357380015	2.6362965	-6.035851505	5.52E-09	1.87E-08	9.680592027
PTPN22	0.561287211	1.14325131	6.035174554	5.54E-09	1.88E-08	9.677017727
TTPAL	-0.402176231	3.70661471	-6.034786288	5.55E-09	1.88E-08	9.674967824
D2HGDH	-0.465367316	3.97999526	-6.034031246	5.57E-09	1.89E-08	9.670981758
CABCOC	-0.773816578	0.97419669	-6.032296272	5.62E-09	1.91E-08	9.661823784

ZNF844	-0.451343799	3.86790498	-6.032198834	5.63E-09	1.91E-08	9.66130952
HIC2	-0.477359527	2.09510166	-6.031829642	5.64E-09	1.91E-08	9.659361037
MFSD11	-0.362030506	3.677113	-6.031001068	5.66E-09	1.92E-08	9.654988401
CTSC	0.917712717	5.53602812	6.029130908	5.72E-09	1.94E-08	9.645120659
FAM86B:	-0.407769879	1.50580742	-6.028026403	5.76E-09	1.95E-08	9.639293912
NCK1	-0.429564744	4.49482756	-6.028018425	5.76E-09	1.95E-08	9.639251825
DDX51	-0.343738009	3.89421674	-6.02436728	5.87E-09	1.99E-08	9.619996155
IQCD	-0.350302028	2.58891756	-6.022877459	5.92E-09	2.01E-08	9.612141547
TRMT10I	-0.406191329	3.50684293	-6.021769369	5.95E-09	2.02E-08	9.606300437
SOX4	0.778964724	5.86571642	6.020518427	6.00E-09	2.03E-08	9.599707278
METTL2	-0.456118584	0.5925598	-6.018769285	6.05E-09	2.05E-08	9.590490051
SP2	-0.486666924	4.84960831	-6.018557626	6.06E-09	2.05E-08	9.589374839
DCLK3	-0.333143599	0.59180118	-6.018129274	6.07E-09	2.06E-08	9.587117976
IRF2BP1	-0.368847373	5.57363926	-6.017538541	6.09E-09	2.06E-08	9.584005771
CDK11B	-0.322340532	4.70079574	-6.01752083	6.09E-09	2.06E-08	9.583912463
CAND2	-0.509962004	2.35298962	-6.017222431	6.10E-09	2.06E-08	9.582340474
GOLGA5	-0.400407539	5.81023428	-6.016790163	6.12E-09	2.07E-08	9.580063359
ZNF263	-0.33482685	3.86849585	-6.016033788	6.14E-09	2.08E-08	9.576079198
UNC13D	0.612918759	2.21436262	6.015903882	6.15E-09	2.08E-08	9.57539496
KLHL14	-0.558043111	4.36116546	-6.015825585	6.15E-09	2.08E-08	9.574982564
INIP	-0.417538434	4.11666829	-6.015533886	6.16E-09	2.08E-08	9.5734462
UCKL1	-0.346146074	4.8947247	-6.012634715	6.26E-09	2.11E-08	9.558179416
ALDH1A:	0.963809563	3.00267189	6.011482949	6.30E-09	2.13E-08	9.552115853
LRRC4	-0.485604235	1.40657266	-6.01120577	6.31E-09	2.13E-08	9.550656756
FASTKD:	-0.383328235	3.76349245	-6.010171335	6.34E-09	2.14E-08	9.545211821
TMEM81	-0.281536191	2.51390661	-6.010015863	6.35E-09	2.14E-08	9.544393526
ZBTB45	-0.332810199	4.04877562	-6.009636466	6.36E-09	2.15E-08	9.542396719
SYT14	0.633906395	2.5335475	6.009556378	6.36E-09	2.15E-08	9.54197522
CYB5D1	-0.48120123	1.56041944	-6.00864923	6.39E-09	2.16E-08	9.537201234
LYZ	1.47654719	5.89692964	6.008528511	6.40E-09	2.16E-08	9.536565976
SFRP2	1.993831297	4.21173224	6.008161728	6.41E-09	2.16E-08	9.534635911
PRR9	-0.335266242	0.28698891	-6.008132206	6.41E-09	2.16E-08	9.534480565
TASP1	-0.434464253	3.42136013	-6.006366214	6.47E-09	2.18E-08	9.525188952
SLC25A6	-0.375332914	9.68123332	-6.006294681	6.47E-09	2.18E-08	9.52481263
MAP10	-0.577362464	2.03870437	-6.003136748	6.59E-09	2.22E-08	9.508202716
CD1C	1.049635073	2.01652056	6.002801823	6.60E-09	2.22E-08	9.506441477
SRSF2	-0.389095196	6.59348155	-6.002127601	6.62E-09	2.23E-08	9.502896244
POLG2	-0.373208683	2.97154865	-6.001886421	6.63E-09	2.23E-08	9.501628131
LRRC34	-0.348632832	1.27713778	-5.999315021	6.72E-09	2.26E-08	9.488110198
PARP11	-0.383524823	3.02797731	-5.996723423	6.82E-09	2.29E-08	9.47449049

PTOV1	-0.420861053	6.27537913	-5.996542116	6.82E-09	2.30E-08	9.473537831
CCR8	0.430259746	0.5363364	5.994145244	6.91E-09	2.33E-08	9.460945704
MTO1	-0.31002065	2.84671115	-5.993601274	6.93E-09	2.33E-08	9.458088449
CYGB	-0.717459895	4.48593591	-5.992385691	6.98E-09	2.35E-08	9.451704187
PLA2G15	-0.388193871	4.30703507	-5.992259233	6.98E-09	2.35E-08	9.451040087
ZNF672	-0.311052559	5.24666889	-5.991860491	7.00E-09	2.35E-08	9.448946134
ILKAP	-0.434398966	4.59057256	-5.990946172	7.03E-09	2.36E-08	9.444145074
SLC6A17	-0.544114021	0.51504171	-5.989146982	7.10E-09	2.39E-08	9.434699203
PDE4C	0.876853287	2.49998382	5.989031942	7.11E-09	2.39E-08	9.434095307
SLC27A3	-0.430557378	3.44208575	-5.988936051	7.11E-09	2.39E-08	9.433591938
KARS	-0.274221166	6.44355245	-5.988052965	7.14E-09	2.40E-08	9.42895658
YIF1B	-0.320892984	4.58208134	-5.986641909	7.20E-09	2.42E-08	9.421550952
C4orf36	-0.343910049	2.31712264	-5.986254185	7.21E-09	2.42E-08	9.419516297
SLFN11	-0.529921167	3.93545449	-5.986086099	7.22E-09	2.42E-08	9.418634259
LTB	1.239296699	3.00779345	5.985789111	7.23E-09	2.43E-08	9.417075857
DCLK1	0.521776181	1.09762845	5.985618607	7.24E-09	2.43E-08	9.416181191
CYP20A1	-0.348709212	3.69357099	-5.985199046	7.26E-09	2.43E-08	9.413979749
NAGK	-0.374758562	4.38520945	-5.984906471	7.27E-09	2.44E-08	9.412444678
PHF14	-0.330840796	3.5349446	-5.984190058	7.29E-09	2.45E-08	9.408686056
C15orf62	0.45611327	1.83616774	5.98373977	7.31E-09	2.45E-08	9.406323824
RIMBP2	-0.647430522	1.26678749	-5.982625015	7.36E-09	2.47E-08	9.400476336
SCAMP5	-0.465405151	3.94615674	-5.9815826	7.40E-09	2.48E-08	9.395009053
MED21	-0.380539553	4.65319021	-5.979026429	7.50E-09	2.51E-08	9.381605424
STMN2	0.777640249	1.09912634	5.978881397	7.51E-09	2.51E-08	9.380845061
ZNF217	0.614266112	4.87045521	5.978744088	7.51E-09	2.52E-08	9.380125195
CRPPA	-0.249667658	1.01645793	-5.978231713	7.53E-09	2.52E-08	9.377439101
ANKRD2	-0.377560264	3.73279352	-5.977584944	7.56E-09	2.53E-08	9.374048709
HELQ	-0.336974697	3.6759859	-5.977514954	7.56E-09	2.53E-08	9.373681834
TM9SF2	-0.524941784	7.43276418	-5.977248439	7.57E-09	2.53E-08	9.372284842
NMT1	-0.331843039	5.8250895	-5.976590124	7.60E-09	2.54E-08	9.368834349
SLC4A3	-0.574787683	1.84094809	-5.974069261	7.70E-09	2.58E-08	9.35562416
SNX29	-0.485930803	3.27473792	-5.973451723	7.73E-09	2.59E-08	9.352388686
TM4SF1	1.073388342	6.50830333	5.973201278	7.74E-09	2.59E-08	9.3510766
CES2	-0.426303879	5.64284436	-5.971266564	7.82E-09	2.61E-08	9.340941992
EFR3B	-0.638143626	2.91934774	-5.970483081	7.85E-09	2.63E-08	9.336838582
TMC6	0.772400758	4.70283214	5.97016518	7.87E-09	2.63E-08	9.335173723
RASGRF	0.917655835	1.5207241	5.969015636	7.92E-09	2.65E-08	9.32915408
MAF1	-0.295036977	7.25265095	-5.968809648	7.92E-09	2.65E-08	9.32807551
MOSPD3	-0.373883596	4.75237885	-5.967788243	7.97E-09	2.66E-08	9.322727746
AKAP1	-0.546246134	5.7099274	-5.965206897	8.08E-09	2.70E-08	9.30921569

ZNF789	-0.366868471	2.40608585	-5.963282421	8.16E-09	2.73E-08	9.299144895
CDH22	0.810828759	1.49242024	5.962621792	8.19E-09	2.73E-08	9.295688384
B4GALT4	-0.375267628	3.66412413	-5.961234364	8.25E-09	2.75E-08	9.288430093
NSRP1	-0.405474098	4.63920471	-5.961062426	8.26E-09	2.76E-08	9.28753069
SPATA6	-0.407066648	3.48099462	-5.961029619	8.26E-09	2.76E-08	9.287359081
CERK	-0.531819121	5.2099331	-5.95782748	8.41E-09	2.80E-08	9.270612484
RNF130	-0.292990415	4.13465141	-5.957770471	8.41E-09	2.80E-08	9.270314401
P2RY1	-0.338160816	1.12149828	-5.957530791	8.42E-09	2.81E-08	9.269061199
DHRS9	0.742513316	1.1956339	5.957140336	8.44E-09	2.81E-08	9.267019732
PRKCH	-0.516923044	3.99222714	-5.955645713	8.51E-09	2.83E-08	9.259206137
EIF4G3	-0.464037751	5.31433336	-5.954000233	8.58E-09	2.86E-08	9.250605609
INO80B-\	-0.065685916	0.08791418	-5.953883713	8.59E-09	2.86E-08	9.249996653
TMEM21	-0.375761939	6.30646882	-5.951509497	8.70E-09	2.90E-08	9.23759054
CTNNA2	-0.19553291	0.16049958	-5.950127877	8.76E-09	2.92E-08	9.230372811
PAPOLG	-0.504421187	3.63731771	-5.94997271	8.77E-09	2.92E-08	9.229562283
ZDHHC6	-0.301447049	4.81447664	-5.94981568	8.78E-09	2.92E-08	9.22874204
LSM6	-0.324088664	3.54977307	-5.949718346	8.78E-09	2.92E-08	9.228233626
ARIH2	-0.391305266	4.81592069	-5.949617642	8.79E-09	2.92E-08	9.227707611
C16orf70	-0.352611977	2.91043389	-5.948497131	8.84E-09	2.94E-08	9.221855254
STXBP3	-0.383828269	4.74973558	-5.946749678	8.92E-09	2.97E-08	9.212730081
SNRNP4	-0.363144257	2.81384016	-5.945346602	8.99E-09	2.99E-08	9.205404711
PNPT1	-0.392486543	3.90331535	-5.944895915	9.01E-09	3.00E-08	9.20305198
HIST1H2	-0.546910132	1.10704882	-5.944190178	9.04E-09	3.01E-08	9.199368076
CRYBB3	-0.532346063	1.48381974	-5.944047867	9.05E-09	3.01E-08	9.198625263
PPP1R1I	1.119439175	2.16641789	5.943996772	9.05E-09	3.01E-08	9.198358565
CREBL2	-0.412248525	6.56768579	-5.943826435	9.06E-09	3.01E-08	9.197469492
BAIAP3	1.028744207	2.81677923	5.943751924	9.07E-09	3.01E-08	9.197080582
LAD1	0.844805652	5.03365822	5.941088891	9.20E-09	3.05E-08	9.183183471
TYW1	-0.369322973	4.18721035	-5.940855098	9.21E-09	3.06E-08	9.181963641
METAP2	-0.377221842	5.59363559	-5.93899384	9.30E-09	3.09E-08	9.1722537
GIMAP5	-0.337932043	0.94439092	-5.938138899	9.34E-09	3.10E-08	9.167794358
HAT1	-0.441847849	4.13523792	-5.937673719	9.37E-09	3.11E-08	9.165368198
SPTBN1	-0.568692047	5.829657	-5.936504623	9.43E-09	3.13E-08	9.15927138
PTEN	-0.354208116	4.7474603	-5.934969667	9.50E-09	3.15E-08	9.151267989
IGIP	-0.402232636	4.57923826	-5.933675786	9.57E-09	3.17E-08	9.144522804
THSD1	-0.478044228	2.52987646	-5.933412614	9.58E-09	3.18E-08	9.143150983
IFT57	-0.460760127	5.91671056	-5.933061515	9.60E-09	3.18E-08	9.141320911
CFL1	-0.331100023	8.1830214	-5.93205114	9.65E-09	3.20E-08	9.136054867
C20orf20	-0.254994973	0.66933959	-5.931255243	9.69E-09	3.21E-08	9.131907151
TMEM24	-0.589967724	6.05809198	-5.930672582	9.72E-09	3.22E-08	9.128870957

HNRNPH	-0.386362481	6.3043865	-5.930622539	9.73E-09	3.22E-08	9.128610199
BAMBI	-0.712533721	1.62028145	-5.93007775	9.76E-09	3.23E-08	9.125771575
RETREG	-0.447523383	3.90790708	-5.929133391	9.80E-09	3.25E-08	9.12085146
LIMCH1	-0.561952225	4.76252675	-5.929052655	9.81E-09	3.25E-08	9.120430853
GOLGA8	-0.157829083	0.1998086	-5.928824831	9.82E-09	3.25E-08	9.119243988
IGFBP6	1.581912977	7.31181775	5.928345324	9.85E-09	3.26E-08	9.11674608
FMNL1	0.609672403	3.16952833	5.928200609	9.85E-09	3.26E-08	9.115992242
CMSS1	-0.304728599	3.41750722	-5.925769576	9.98E-09	3.30E-08	9.10333079
LNX1	-0.578696594	4.10942596	-5.924850738	1.00E-08	3.32E-08	9.098546265
ALDH1L1	-0.651913684	0.75294309	-5.92429228	1.01E-08	3.33E-08	9.095638566
PIGW	-0.394576259	3.05257422	-5.924146617	1.01E-08	3.33E-08	9.094880185
NKX2-1	-0.557520601	8.16701774	-5.922397642	1.02E-08	3.36E-08	9.085775392
SLC25A3	0.513140612	4.30516047	5.922104097	1.02E-08	3.36E-08	9.084247457
MROH6	0.795970817	2.75691355	5.921228376	1.02E-08	3.38E-08	9.079689577
AUNIP	-0.206406135	0.89942391	-5.921177893	1.02E-08	3.38E-08	9.079426842
ZHX1	-0.50645415	4.76056667	-5.919445187	1.03E-08	3.41E-08	9.070410159
INPP1	-0.299876427	4.55050788	-5.918187883	1.04E-08	3.43E-08	9.063868637
FCGR2A	0.731709344	3.06176288	5.918123336	1.04E-08	3.43E-08	9.063532839
FAM20C	0.701769024	5.05919859	5.917000156	1.05E-08	3.45E-08	9.057690073
STARD4	-0.467704856	2.44300918	-5.916777561	1.05E-08	3.46E-08	9.056532241
PCLO	-0.40433715	1.39680485	-5.916067424	1.05E-08	3.47E-08	9.052838656
KLHDC2	-0.475954907	4.77238587	-5.914928026	1.06E-08	3.49E-08	9.046913098
EIF2AK3	-0.548058792	4.16546111	-5.913731477	1.06E-08	3.51E-08	9.040691247
TTC36	-0.375115602	0.90380129	-5.913145225	1.07E-08	3.52E-08	9.037643189
NPIPB9	-0.146723174	0.2270362	-5.911904871	1.08E-08	3.54E-08	9.031195054
RASL12	-0.595633297	2.93517338	-5.911706958	1.08E-08	3.55E-08	9.030166272
LYAR	-0.311038111	3.59864276	-5.908600782	1.09E-08	3.61E-08	9.01402335
GPR150	0.208288947	0.41558036	5.908058249	1.10E-08	3.62E-08	9.011204444
PRMT6	-0.432991564	4.23559698	-5.90688048	1.10E-08	3.64E-08	9.005085639
FHAD1	-0.458548272	1.01104435	-5.906868682	1.10E-08	3.64E-08	9.005024351
NUPR2	-1.047854523	4.10021518	-5.905516827	1.11E-08	3.66E-08	8.998002278
SEC22C	-0.363815604	4.03696341	-5.905144406	1.11E-08	3.67E-08	8.996067988
FRAT1	-0.363111456	2.91088737	-5.905056946	1.12E-08	3.67E-08	8.99561375
FAM107A	-0.89683254	5.0204083	-5.904609091	1.12E-08	3.68E-08	8.993287822
PIH1D3	-0.110971176	0.10818951	-5.902720499	1.13E-08	3.72E-08	8.983480911
KEAP1	-0.322401783	6.15451122	-5.902108566	1.13E-08	3.73E-08	8.980303834
TCEAL2	-1.456359727	3.7720442	-5.900683936	1.14E-08	3.76E-08	8.972908305
LRRC4C	-0.56467953	1.09709309	-5.900566865	1.14E-08	3.76E-08	8.972300629
SLC35F2	0.660028333	4.61977306	5.899471741	1.15E-08	3.78E-08	8.966616633
ABCD4	-0.394802953	4.08955637	-5.899043912	1.15E-08	3.79E-08	8.964396303

TPRA1	-0.314089003	5.19692136	-5.8982369	1.16E-08	3.80E-08	8.960208433
TMEM18	-0.302962965	2.50385074	-5.897866822	1.16E-08	3.81E-08	8.958288113
APPBP2	-0.429972277	3.79245561	-5.897572335	1.16E-08	3.81E-08	8.956760097
MINDY3	-0.357397157	4.1278473	-5.897299565	1.16E-08	3.82E-08	8.955344819
SIM2	-0.32559164	0.6118661	-5.89650658	1.17E-08	3.83E-08	8.951230664
MAGI3	-0.624943114	4.09528297	-5.895883971	1.17E-08	3.85E-08	8.948000743
DCUN1D	-0.384346144	3.71674935	-5.895618343	1.17E-08	3.85E-08	8.946622819
RPS7	-0.363141956	7.68656484	-5.895432745	1.17E-08	3.85E-08	8.945660072
TREX2	-0.307454735	0.66501908	-5.895213291	1.18E-08	3.86E-08	8.944521734
FUZ	-0.376026695	4.74575685	-5.895189529	1.18E-08	3.86E-08	8.944398479
CLDN1	1.519946033	7.49517375	5.895175478	1.18E-08	3.86E-08	8.944325595
PFDN1	-0.27587809	6.59846321	-5.893271523	1.19E-08	3.90E-08	8.934450927
BBS5	-0.408246166	3.23261578	-5.892273569	1.19E-08	3.92E-08	8.929276106
POLR2I	-0.38170692	6.23567633	-5.892016751	1.20E-08	3.92E-08	8.927944503
ANKRD7	-0.317412873	0.75715322	-5.89166667	1.20E-08	3.93E-08	8.926129402
PRKCSH	-0.337764434	7.73665008	-5.890842313	1.20E-08	3.94E-08	8.921855596
PIGN	-0.435339423	3.29693767	-5.889790129	1.21E-08	3.96E-08	8.9164013
DHX57	-0.347184928	3.23689824	-5.88960228	1.21E-08	3.97E-08	8.915427607
JPT2	-0.388591829	5.67032345	-5.887765865	1.22E-08	4.01E-08	8.905910035
FHOD3	-0.663515221	1.79591279	-5.887635701	1.22E-08	4.01E-08	8.90523552
LYST	0.541678291	3.08835664	5.887354225	1.23E-08	4.01E-08	8.903776944
PEX10	-0.371346366	4.10584492	-5.887338663	1.23E-08	4.01E-08	8.903696302
TTLL13P	-0.092904362	0.19040713	-5.886553882	1.23E-08	4.03E-08	8.899629935
ZNF596	-0.380537627	2.0088864	-5.886307632	1.23E-08	4.03E-08	8.898354069
PHAX	-0.359602165	4.56463346	-5.885958867	1.23E-08	4.04E-08	8.896547126
PTPRN2	-0.6468754	4.28739772	-5.885840895	1.24E-08	4.04E-08	8.895935933
TMEM25	-0.396382135	2.26421434	-5.885745273	1.24E-08	4.04E-08	8.895440535
DCLRE1/	-0.382434528	3.22642759	-5.881861717	1.26E-08	4.13E-08	8.875325954
DIRAS1	0.671939962	1.2772579	5.880272157	1.27E-08	4.16E-08	8.867095861
FDXACB	-0.250449027	1.61533115	-5.879945238	1.27E-08	4.17E-08	8.865403415
REXO4	-0.328778651	5.19596817	-5.879164525	1.28E-08	4.18E-08	8.861361991
HNRNPH	-0.379920872	7.12199902	-5.879004441	1.28E-08	4.19E-08	8.860533358
STK11	-0.338222808	4.6030651	-5.878967501	1.28E-08	4.19E-08	8.860342148
LIN7A	-0.627971723	1.72801101	-5.878345636	1.29E-08	4.20E-08	8.857123389
RND3	0.793769281	3.41496247	5.877514753	1.29E-08	4.22E-08	8.852823161
THYN1	-0.318690872	6.46098018	-5.877496121	1.29E-08	4.22E-08	8.85272674
TMEM8B	-0.424895192	4.61428641	-5.877435424	1.29E-08	4.22E-08	8.852412619
MEIS2	-0.441526603	1.21895044	-5.877181917	1.29E-08	4.22E-08	8.851100704
CHMP2B	-0.430917876	6.03435261	-5.876653265	1.30E-08	4.23E-08	8.848365032
TBCCD1	-0.405557781	3.53336596	-5.876622558	1.30E-08	4.23E-08	8.848206131

ZNF521	-0.636906133	2.33829672	-5.876580826	1.30E-08	4.23E-08	8.847990188
ARF3	-0.344354804	7.36577945	-5.876247326	1.30E-08	4.24E-08	8.8462645
FOLR1	-0.931993698	5.43659677	-5.875131916	1.31E-08	4.26E-08	8.840493379
TBC1D17	-0.343129759	5.47286685	-5.873910668	1.32E-08	4.29E-08	8.834175616
FSD2	-0.236165627	0.30698057	-5.873773058	1.32E-08	4.29E-08	8.833463796
SMARCD	-0.344670373	6.03977673	-5.871790812	1.33E-08	4.34E-08	8.823211541
SERF1A	-0.134298314	0.41411191	-5.871069196	1.34E-08	4.35E-08	8.819479971
PRSS22	0.785039049	3.79875695	5.870970805	1.34E-08	4.36E-08	8.818971205
CASTOR	-0.421187946	2.46376492	-5.870714253	1.34E-08	4.36E-08	8.817644643
CCL7	0.384564087	0.49771197	5.870001172	1.34E-08	4.38E-08	8.813957722
ALKBH1	-0.338609376	3.74654136	-5.86997222	1.34E-08	4.38E-08	8.813808034
PTTG1	-0.765073158	5.62716319	-5.868762159	1.35E-08	4.40E-08	8.807552326
MYLK4	-0.260170143	0.86695809	-5.86861224	1.35E-08	4.41E-08	8.80677735
NAA10	-0.348656152	4.25536555	-5.868239365	1.36E-08	4.41E-08	8.804849916
PRKCA	-0.579151655	1.87840782	-5.866691314	1.37E-08	4.45E-08	8.796848863
LRRC61	-0.449929955	4.64414568	-5.866302643	1.37E-08	4.46E-08	8.794840282
SEC63	-0.472670269	5.93779064	-5.865731817	1.37E-08	4.47E-08	8.791890537
RERG	-0.457325168	1.47637049	-5.865208735	1.38E-08	4.48E-08	8.789187705
DNAI2	-0.13807082	0.15851675	-5.864417442	1.38E-08	4.50E-08	8.785099339
MROH7	-0.385182377	0.81172244	-5.862243541	1.40E-08	4.55E-08	8.773869632
GPBP1	-0.34622808	5.63532332	-5.860625824	1.41E-08	4.59E-08	8.765515064
LILRB3	0.332807419	0.93749431	5.859236211	1.42E-08	4.62E-08	8.75833992
CPNE2	-0.415493177	4.10396531	-5.857835377	1.43E-08	4.66E-08	8.751108152
CSF2RA	0.637253692	1.98420502	5.857264111	1.44E-08	4.67E-08	8.748159384
SSC4D	-0.90015575	2.10832222	-5.856302191	1.45E-08	4.69E-08	8.743194634
UBR7	-0.336327805	5.04099012	-5.853720033	1.47E-08	4.76E-08	8.72987043
E2F6	-0.354756439	3.15335466	-5.85321208	1.47E-08	4.77E-08	8.727249865
ALAD	-0.42080914	5.44299588	-5.853019097	1.47E-08	4.77E-08	8.7262543
SIAH1	-0.289399785	2.94525328	-5.852909361	1.47E-08	4.78E-08	8.725688204
ORC2	-0.365129734	3.66039034	-5.852735868	1.47E-08	4.78E-08	8.724793213
PSPN	-0.316127893	2.38277052	-5.850808347	1.49E-08	4.83E-08	8.714851207
PPP6R2	-0.337343841	5.34060173	-5.850644448	1.49E-08	4.83E-08	8.71400594
CXCL14	2.297829758	5.59465121	5.850562682	1.49E-08	4.83E-08	8.713584264
SIK3	-0.418307627	4.17088267	-5.850227979	1.49E-08	4.84E-08	8.711858201
FERMT1	-0.695057102	1.51363575	-5.846169303	1.53E-08	4.94E-08	8.690933611
EPB41L4	-0.572793956	4.76796959	-5.845763843	1.53E-08	4.95E-08	8.68884386
MORF4L	-0.421804852	6.7553382	-5.845255893	1.53E-08	4.97E-08	8.686226034
COMMD	-0.312416259	4.61536204	-5.8426403	1.55E-08	5.03E-08	8.67274877
FLYWCH	-0.354225453	3.93484721	-5.842179092	1.56E-08	5.04E-08	8.670372803
SLC16A6	-0.516494816	1.89463263	-5.842015389	1.56E-08	5.05E-08	8.669529497

MEGF8	-0.431957297	4.5570402	-5.841456768	1.56E-08	5.06E-08	8.666651949
TMEM35	-0.544874397	4.87205946	-5.840101392	1.58E-08	5.10E-08	8.659671048
SCN4A	-0.787904445	2.560855	-5.838645113	1.59E-08	5.14E-08	8.652171825
ATF2	-0.428315454	4.91456256	-5.838081885	1.59E-08	5.15E-08	8.649271823
SLC16A1	-0.503782818	3.27878129	-5.837603212	1.60E-08	5.16E-08	8.646807351
ADAM8	0.608469001	2.18550951	5.837121977	1.60E-08	5.17E-08	8.644329844
GPM6B	-0.479822603	1.93397374	-5.837021441	1.60E-08	5.18E-08	8.643812286
PIANP	0.749549767	1.60774027	5.836989597	1.60E-08	5.18E-08	8.64364835
PPARD	-0.414311184	6.17546517	-5.83556772	1.61E-08	5.21E-08	8.63632925
DNAJC2	-0.38070056	2.23927106	-5.835031265	1.62E-08	5.23E-08	8.633568205
C3orf14	-0.287222514	2.20906614	-5.833832308	1.63E-08	5.26E-08	8.627398076
LIN7C	-0.383393142	4.8219879	-5.833819922	1.63E-08	5.26E-08	8.627334338
TSEN34	-0.364368518	5.70371071	-5.833484744	1.63E-08	5.27E-08	8.625609605
TATDN1	-0.358573335	3.92314829	-5.8332936	1.63E-08	5.27E-08	8.624626062
UBL4A	-0.355446468	5.95519722	-5.832988609	1.64E-08	5.28E-08	8.623056769
ACTR3C	-0.262864184	1.20149424	-5.832488044	1.64E-08	5.29E-08	8.620481313
HOOK2	-0.461206777	4.8895744	-5.831676469	1.65E-08	5.32E-08	8.616306032
RNF212	-0.792236865	2.08903791	-5.830936366	1.65E-08	5.33E-08	8.612498836
ANKK1	-0.37909034	0.57911944	-5.829935137	1.66E-08	5.36E-08	8.607348963
LCMT2	-0.347854691	2.9613744	-5.826718092	1.69E-08	5.45E-08	8.590806496
RAF1	-0.365318552	5.48767265	-5.825223666	1.70E-08	5.50E-08	8.583124337
DLAT	-0.443754255	4.8742496	-5.824479225	1.71E-08	5.52E-08	8.579298072
BCAT1	0.760022187	2.14046288	5.823662953	1.72E-08	5.54E-08	8.575103041
SKP2	-0.37181796	2.69015477	-5.823658079	1.72E-08	5.54E-08	8.575077992
PRIMPOL	-0.355736821	3.44646625	-5.822846443	1.73E-08	5.56E-08	8.570907235
POT1	-0.376803182	3.68056884	-5.822152342	1.73E-08	5.58E-08	8.567340805
PAQR6	0.679294491	2.3640246	5.821445933	1.74E-08	5.60E-08	8.563711472
PLEC	0.431688349	5.49340697	5.821227563	1.74E-08	5.61E-08	8.562589616
IL2RB	0.883817915	2.7595578	5.821095132	1.74E-08	5.61E-08	8.561909278
FBXO33	-0.403259414	4.3577273	-5.820722838	1.75E-08	5.62E-08	8.55999676
AXDND1	-0.10997873	0.20192285	-5.820687916	1.75E-08	5.62E-08	8.559817365
APOBR	0.666239721	2.55500332	5.818048225	1.77E-08	5.70E-08	8.546259695
STIM1	-0.503109028	5.90175558	-5.817987927	1.77E-08	5.70E-08	8.545950055
ZNRD1	-0.349861036	3.62238494	-5.81731803	1.78E-08	5.72E-08	8.542510184
RAB11B	-0.364223515	8.12884525	-5.814959639	1.80E-08	5.79E-08	8.53040244
SLU7	-0.358384091	5.40538199	-5.814633364	1.80E-08	5.80E-08	8.528727673
NDUFA1	-0.457867947	5.06899127	-5.814267326	1.81E-08	5.81E-08	8.526848887
GABPA	-0.444591035	4.35257572	-5.812853124	1.82E-08	5.85E-08	8.519590983
PANK2	-0.336144227	4.03611497	-5.812312013	1.82E-08	5.86E-08	8.516814273
CTSE	1.444451013	3.70292606	5.811987693	1.83E-08	5.87E-08	8.515150122

GHDC	-0.55797337	5.86426877	-5.811422514	1.83E-08	5.89E-08	8.512250242
GNPTG	-0.317441356	6.0634266	-5.810956977	1.84E-08	5.90E-08	8.509861778
MRPL23	-0.421176227	4.95554456	-5.810737591	1.84E-08	5.91E-08	8.508736261
TMEM45	-0.555612592	1.76657836	-5.809868427	1.85E-08	5.93E-08	8.504277486
MYLIP	-0.608168864	6.31061967	-5.808637123	1.86E-08	5.97E-08	8.497961828
GTPBP8	-0.326751534	3.32916828	-5.807976093	1.87E-08	5.99E-08	8.494571671
CAMK1	-0.540862482	3.24787546	-5.807655938	1.87E-08	6.00E-08	8.492929827
C16orf72	-0.3653811	3.45878011	-5.807505414	1.87E-08	6.00E-08	8.492157922
FSCN1	-0.564194972	5.13967899	-5.807198879	1.87E-08	6.01E-08	8.490586023
TTC39B	0.586686956	3.12873719	5.806389587	1.88E-08	6.04E-08	8.486436316
C8G	-0.408591426	1.4564918	-5.805373987	1.89E-08	6.07E-08	8.481229369
SSR4	-0.429366196	6.95059467	-5.80530812	1.89E-08	6.07E-08	8.480891698
RFNG	-0.324861527	5.13825869	-5.803898161	1.91E-08	6.11E-08	8.47366411
RCN2	-0.528030739	4.87916403	-5.803273367	1.91E-08	6.13E-08	8.470461786
PPP3R1	-0.334413288	5.20703026	-5.802379515	1.92E-08	6.16E-08	8.465880888
ZNF185	-0.57898189	5.05964413	-5.801926418	1.93E-08	6.17E-08	8.463559019
PHF23	-0.369352687	5.79865303	-5.801841573	1.93E-08	6.18E-08	8.463124252
PTGES3I	-0.073576319	0.08818087	-5.801497831	1.93E-08	6.19E-08	8.461362877
ZFP1	-0.384702757	3.6456483	-5.801394895	1.93E-08	6.19E-08	8.460835441
ANO3	-0.277370993	0.62797034	-5.801090574	1.94E-08	6.20E-08	8.459276153
EDARAD	-0.657776246	1.74735764	-5.800833387	1.94E-08	6.20E-08	8.457958421
ZNF780A	-0.364613307	2.81158889	-5.800182826	1.95E-08	6.22E-08	8.454625386
REM1	-0.613420284	2.01845898	-5.799970472	1.95E-08	6.23E-08	8.453537488
C2CD5	-0.428368118	4.41680871	-5.799144587	1.96E-08	6.26E-08	8.449306741
PTCD1	-0.235305225	2.13662775	-5.798766515	1.96E-08	6.27E-08	8.447370157
TM4SF4	1.19043986	1.99843678	5.798002706	1.97E-08	6.29E-08	8.443458012
AGPAT5	-0.50570414	3.88569524	-5.797527252	1.97E-08	6.31E-08	8.441022988
LRRTM1	-0.859110288	0.96827608	-5.797259418	1.98E-08	6.31E-08	8.439651353
PPME1	-0.34090868	4.58635009	-5.797025877	1.98E-08	6.32E-08	8.438455381
ODF3L2	-0.143403944	0.26099688	-5.796471313	1.98E-08	6.34E-08	8.435615581
PRKAR2	-0.464696361	4.75304857	-5.795803657	1.99E-08	6.36E-08	8.432196941
SNX12	-0.389424277	6.2636646	-5.794876959	2.00E-08	6.39E-08	8.427452408
MCIDAS	-0.405684463	1.69828805	-5.794801531	2.00E-08	6.39E-08	8.427066254
RIPOR1	-0.397461902	4.75861144	-5.794790753	2.00E-08	6.39E-08	8.427011078
DPP4	1.542442634	5.38791353	5.794416631	2.01E-08	6.40E-08	8.425095828
DECR2	-0.412963862	3.06038534	-5.793674111	2.01E-08	6.42E-08	8.421294909
TCTN3	-0.658995241	7.06337111	-5.792794405	2.02E-08	6.45E-08	8.416792221
C1orf35	-0.394414929	3.92654029	-5.791102236	2.04E-08	6.51E-08	8.408132508
SLC12A4	-0.431957572	4.97717631	-5.790278887	2.05E-08	6.54E-08	8.4039197
SLC51A	-0.282671373	1.37705618	-5.789765297	2.05E-08	6.55E-08	8.401292061

ZNF436	-0.522890743	4.47071258	-5.789548388	2.06E-08	6.56E-08	8.400182363
TMC3	-0.484230394	0.8015244	-5.789454784	2.06E-08	6.56E-08	8.399703493
RNF141	-0.424992328	4.2330585	-5.789010435	2.06E-08	6.57E-08	8.397430348
PTPRB	-0.571308016	3.42687309	-5.788697806	2.07E-08	6.58E-08	8.395831118
USO1	-0.51849236	6.11671246	-5.787550647	2.08E-08	6.62E-08	8.389963479
MRPL41	-0.466319739	6.21371368	-5.787458351	2.08E-08	6.62E-08	8.389491428
STRBP	-0.363168782	4.28159018	-5.787385119	2.08E-08	6.63E-08	8.389116886
TEX14	-0.084375373	0.20463747	-5.787371965	2.08E-08	6.63E-08	8.389049611
ATF4	-0.377811697	8.01632448	-5.786107085	2.09E-08	6.67E-08	8.382581025
MAJIN	-0.252601566	0.37558503	-5.784750652	2.11E-08	6.71E-08	8.375645446
SLC1A5	0.852553256	4.48172881	5.784670885	2.11E-08	6.72E-08	8.375237625
TCF4	-0.479063532	3.05672605	-5.784401953	2.11E-08	6.72E-08	8.373862711
GAS2	-0.235221203	0.75785496	-5.783480095	2.12E-08	6.76E-08	8.369150092
CTBP2	-0.506578157	4.99402418	-5.78311431	2.13E-08	6.77E-08	8.367280322
PLD1	-0.335913791	1.44000393	-5.783021306	2.13E-08	6.77E-08	8.366804935
ABITRAM	-0.357871183	4.69195858	-5.781973084	2.14E-08	6.81E-08	8.361447351
NRG2	-0.250685041	0.43644483	-5.780898689	2.15E-08	6.84E-08	8.355956771
SLIT3	-0.811395354	4.85090142	-5.780630209	2.16E-08	6.85E-08	8.354584858
ARHGAP	0.638815596	4.1598369	5.780536927	2.16E-08	6.85E-08	8.354108206
PHYHIP	1.012461139	3.77573591	5.78041914	2.16E-08	6.86E-08	8.353506343
MXI1	-0.442989415	5.45644241	-5.779935218	2.16E-08	6.87E-08	8.351033724
TAS2R14	-0.349529979	1.4843953	-5.778802832	2.18E-08	6.91E-08	8.345248381
PDZD4	-0.421886998	1.45154644	-5.777219168	2.19E-08	6.97E-08	8.337158921
CUL1	-0.325869045	5.89648796	-5.776897262	2.20E-08	6.98E-08	8.335514812
HECW2	-0.563989175	2.2832169	-5.776796496	2.20E-08	6.98E-08	8.335000171
RFTN2	-0.360169243	1.34317429	-5.773587422	2.24E-08	7.10E-08	8.318614156
USP33	-0.384441234	4.6940994	-5.772695972	2.25E-08	7.13E-08	8.314063521
WARS2	-0.337654658	3.67555967	-5.77147563	2.26E-08	7.17E-08	8.307834847
PGS1	-0.36918896	4.16814259	-5.771474075	2.26E-08	7.17E-08	8.307826907
GATAD1	-0.398331773	5.22645618	-5.769567293	2.28E-08	7.25E-08	8.298096646
STX6	-0.33584584	4.66467792	-5.769188385	2.29E-08	7.26E-08	8.296163385
UCK2	-0.398619833	2.58955409	-5.768110306	2.30E-08	7.30E-08	8.290663347
MARK2	-0.38753092	5.06832478	-5.76792158	2.30E-08	7.30E-08	8.289700602
SULT2B1	0.863508209	2.64436024	5.767245798	2.31E-08	7.33E-08	8.286253452
PRKRA	-0.30708522	4.8532937	-5.767154705	2.31E-08	7.33E-08	8.285788812
EPCAM	-0.511908626	9.05334811	-5.766846188	2.32E-08	7.34E-08	8.284215197
CCZ1B	-0.288547377	1.68687341	-5.76537093	2.34E-08	7.40E-08	8.27669142
CDKN2A	0.718156724	1.66841899	5.765213821	2.34E-08	7.40E-08	8.275890258
ST6GAL1	-1.077562631	4.81002376	-5.763340453	2.36E-08	7.47E-08	8.266338462
TCAF2	-0.466097006	1.05080586	-5.762226606	2.37E-08	7.52E-08	8.260660391

ZSCAN3:	-0.874072017	3.09581448	-5.762049446	2.38E-08	7.52E-08	8.259757359
YBX3	0.487784426	5.57375783	5.760908026	2.39E-08	7.57E-08	8.25393974
FAM168E	-0.478633231	6.10809176	-5.760038011	2.40E-08	7.60E-08	8.249506022
DBR1	-0.37980003	3.84889569	-5.758410131	2.42E-08	7.66E-08	8.241211507
FLNA	0.717520563	7.54275204	5.757959041	2.43E-08	7.68E-08	8.238913391
UBXN11	-0.487765072	4.76362803	-5.757623644	2.43E-08	7.69E-08	8.237204777
CNTFR	-1.400154211	1.66262993	-5.757600486	2.43E-08	7.69E-08	8.237086802
SMIM5	-0.648341894	3.66729311	-5.757262257	2.44E-08	7.70E-08	8.235363844
EPHX3	0.585204014	1.27823148	5.757016128	2.44E-08	7.71E-08	8.234110091
CORO1B	-0.308293099	6.37667271	-5.757006818	2.44E-08	7.71E-08	8.234062668
IGFL2	1.214638018	1.91122769	5.755479859	2.46E-08	7.77E-08	8.226285476
ARVCF	-0.474452149	4.96502268	-5.755199012	2.46E-08	7.78E-08	8.224855222
ZP1	-0.231196744	0.2682439	-5.754931515	2.47E-08	7.79E-08	8.223493005
MT1HL1	-0.494137226	0.48063617	-5.754025825	2.48E-08	7.83E-08	8.218881183
ANKS1A	-0.482318277	4.25029805	-5.751667225	2.51E-08	7.92E-08	8.206873679
ZFP64	-0.362260703	2.7800497	-5.751456246	2.51E-08	7.93E-08	8.205799784
KDM4C	-0.356217763	2.88500049	-5.750699212	2.52E-08	7.96E-08	8.201946681
ZNF440	-0.327151857	2.14048919	-5.75041961	2.53E-08	7.97E-08	8.200523674
GDF11	-0.43996409	2.68331472	-5.75027113	2.53E-08	7.97E-08	8.199768024
EPHB6	-0.893952926	4.72932714	-5.749522696	2.54E-08	8.00E-08	8.195959296
KLRG1	-0.32171633	1.04307574	-5.748088463	2.56E-08	8.06E-08	8.188661648
FOXQ1	1.331763529	4.96828452	5.747435729	2.57E-08	8.09E-08	8.185340878
GMEB2	-0.33319598	4.68100608	-5.746829592	2.57E-08	8.11E-08	8.182257431
VPS72	-0.310761767	4.8127381	-5.746586696	2.58E-08	8.12E-08	8.181021879
ATE1	-0.454024427	4.33033117	-5.745582804	2.59E-08	8.16E-08	8.175915748
CCN1	-0.982510783	7.88861069	-5.745483628	2.59E-08	8.17E-08	8.175411343
PSMG2	-0.272368917	5.52577944	-5.744272469	2.61E-08	8.22E-08	8.169251982
PITPNA	-0.384650955	6.33767413	-5.743982761	2.61E-08	8.23E-08	8.167778818
ZNF697	-0.436297908	2.72323868	-5.743165037	2.62E-08	8.26E-08	8.163621
SPI1	0.767701705	4.22172522	5.742893841	2.63E-08	8.27E-08	8.162242174
ADGRL2	-0.825353083	4.76512972	-5.742695852	2.63E-08	8.28E-08	8.161235578
OR4D10	0.618934718	0.8232639	5.74167904	2.64E-08	8.32E-08	8.156066442
MARS2	-0.44915464	2.71262879	-5.741532079	2.65E-08	8.33E-08	8.155319394
BDKRB2	-0.70503216	2.41366887	-5.740809763	2.66E-08	8.36E-08	8.151647879
RPL23	-0.343385826	8.48242277	-5.739857006	2.67E-08	8.40E-08	8.146805576
NALCN	0.604378505	0.99623081	5.739658843	2.67E-08	8.40E-08	8.145798513
PROB1	-0.392017262	1.73289936	-5.739383567	2.68E-08	8.41E-08	8.144399599
ZNF57	-0.300962537	2.50762465	-5.737772452	2.70E-08	8.48E-08	8.136213187
CTLA4	0.708261401	1.18061353	5.737637487	2.70E-08	8.49E-08	8.13552748
RGS5	-0.862589743	6.32159988	-5.735917168	2.73E-08	8.56E-08	8.126788294

CFH	1.106462247	4.48406572	5.734660717	2.74E-08	8.62E-08	8.120406823
GGNBP2	-0.339016317	4.48261567	-5.733877233	2.75E-08	8.65E-08	8.116428086
EIF4A3	-0.377160463	5.30496121	-5.733771582	2.76E-08	8.65E-08	8.115891592
NOL12	-0.346311417	2.53976542	-5.733159623	2.77E-08	8.68E-08	8.112784237
ARHGEF	-0.659658996	2.31199175	-5.733121144	2.77E-08	8.68E-08	8.11258886
NEURL1	0.276354633	0.42912344	5.732051057	2.78E-08	8.73E-08	8.107155901
LPAR2	-0.390940202	3.88789305	-5.731044402	2.80E-08	8.77E-08	8.102045712
C1orf74	-0.309849933	1.56133819	-5.729561487	2.82E-08	8.84E-08	8.094519092
MARS	-0.359146592	5.11609923	-5.729492306	2.82E-08	8.84E-08	8.094167998
APLN	-0.844566033	2.2213475	-5.72931511	2.82E-08	8.85E-08	8.093268741
SLC10A3	-0.40845542	5.53375167	-5.72810885	2.84E-08	8.90E-08	8.087147609
EPYC	0.618578673	0.45788626	5.727533186	2.85E-08	8.93E-08	8.084226771
STYK1	0.267873765	0.47769384	5.727422473	2.85E-08	8.93E-08	8.083665056
ZBTB25	-0.306739013	2.41310313	-5.727217891	2.85E-08	8.94E-08	8.082627106
DCAKD	-0.343750947	6.3035322	-5.726740535	2.86E-08	8.96E-08	8.080205346
PPP2CB	-0.411956951	6.45081992	-5.725787292	2.87E-08	9.00E-08	8.075369738
FAM184A	-0.61709062	2.58252819	-5.725508685	2.88E-08	9.01E-08	8.073956542
MED22	-0.392623783	4.23662179	-5.724843912	2.89E-08	9.04E-08	8.070584775
METTL8	-0.256389762	1.63619427	-5.724453013	2.89E-08	9.06E-08	8.068602258
ASCC1	-0.370933989	3.71614118	-5.722291175	2.93E-08	9.16E-08	8.057639973
FTSJ1	-0.318648264	5.4430465	-5.722273268	2.93E-08	9.16E-08	8.057549183
C11orf58	-0.416930424	5.63787439	-5.721057983	2.95E-08	9.22E-08	8.051388116
ADAMTS	-0.388095514	1.81105024	-5.720995886	2.95E-08	9.22E-08	8.051073334
ZRSR2	-0.33978299	4.93610642	-5.720598631	2.95E-08	9.23E-08	8.049059625
OTUB2	-0.232521414	0.70205246	-5.720222525	2.96E-08	9.25E-08	8.047153223
VPS39	-0.346471174	5.11784842	-5.719767845	2.97E-08	9.27E-08	8.044848678
PREPL	-0.471649534	4.83782826	-5.719503034	2.97E-08	9.28E-08	8.043506548
JAML	0.656464765	1.83421205	5.7180177	2.99E-08	9.35E-08	8.035979377
BRINP1	0.931246151	2.08980072	5.717576481	3.00E-08	9.37E-08	8.033743722
MAB21L4	0.415824742	0.6121076	5.717195931	3.01E-08	9.39E-08	8.031815583
DOHH	-0.332950328	3.98504149	-5.715988547	3.02E-08	9.45E-08	8.025698763
EIF1	-0.319024225	8.65582199	-5.71567446	3.03E-08	9.46E-08	8.024107706
NLRC5	0.580364313	2.66299628	5.715427771	3.03E-08	9.47E-08	8.022858111
CSNK1G	-0.39259188	3.06278874	-5.714212144	3.05E-08	9.53E-08	8.016701008
SNX2	-0.457162943	6.17407208	-5.713243997	3.07E-08	9.58E-08	8.011798102
ACTR1B	-0.37310069	6.68195369	-5.71304689	3.07E-08	9.58E-08	8.010799988
UBE2S	-0.415615124	3.09631036	-5.71281369	3.07E-08	9.59E-08	8.009619143
NIBAN1	0.828939234	3.61927364	5.712236702	3.08E-08	9.62E-08	8.006697626
TGIF2-RA	-0.167138044	0.28986021	-5.71109734	3.10E-08	9.68E-08	8.000929266
CD1E	0.875707167	1.44694211	5.710432405	3.11E-08	9.71E-08	7.997563241

ADAT2	-0.370721526	1.62556089	-5.710282074	3.12E-08	9.71E-08	7.996802282
CCT4	-0.439543795	7.15551631	-5.709962516	3.12E-08	9.73E-08	7.995184758
MIB2	-0.38867178	3.95025758	-5.709080906	3.14E-08	9.77E-08	7.990722638
SLAMF8	0.882195368	2.6033413	5.708627777	3.14E-08	9.79E-08	7.988429412
ZNF568	-0.306928775	2.08034913	-5.707200085	3.17E-08	9.86E-08	7.981204962
HYAL1	-0.41250584	2.23877745	-5.706701448	3.17E-08	9.89E-08	7.978682075
U2AF1L4	-0.476754306	3.60829982	-5.706066785	3.19E-08	9.92E-08	7.975471198
ADGRE2	0.408829646	0.84768146	5.704138886	3.22E-08	1.00E-07	7.965719302
C19orf47	-0.248369366	3.37837984	-5.70346851	3.23E-08	1.01E-07	7.962328936
LAMB2	-0.448177171	6.93406921	-5.702631502	3.24E-08	1.01E-07	7.958096276
RNF4	-0.384408938	4.76687738	-5.702381465	3.25E-08	1.01E-07	7.95683196
ADGRF3	-0.211717864	0.68447864	-5.700323809	3.28E-08	1.02E-07	7.946429008
PDCD1L	0.595135001	1.66172762	5.699538044	3.30E-08	1.03E-07	7.942457162
HSPA4	-0.361427231	6.24532627	-5.699375513	3.30E-08	1.03E-07	7.941635658
PACRG	-0.469863097	1.31766212	-5.69813785	3.32E-08	1.03E-07	7.935380574
TSPYL5	-0.563514147	4.12378095	-5.697364095	3.33E-08	1.04E-07	7.93147059
GOSR1	-0.345440689	3.99818267	-5.696573808	3.35E-08	1.04E-07	7.927477493
MZF1	-0.469902746	3.94256841	-5.695876018	3.36E-08	1.04E-07	7.923952112
LRRC43	-0.415982915	1.65768463	-5.695868616	3.36E-08	1.04E-07	7.923914714
PCDHB1	-0.643652771	1.72948066	-5.695061833	3.37E-08	1.05E-07	7.919839098
KDM1A	-0.346549692	5.41217218	-5.693526065	3.40E-08	1.06E-07	7.912082114
PFDN6	-0.336829896	5.18672928	-5.693277377	3.40E-08	1.06E-07	7.910826173
KCNMB3	-0.139130984	0.45807341	-5.693085596	3.41E-08	1.06E-07	7.909857659
BSDC1	-0.349973146	5.6907507	-5.692342214	3.42E-08	1.06E-07	7.906103729
WIPI1	-0.378860937	4.15723292	-5.691700232	3.43E-08	1.07E-07	7.902862155
SRPX2	0.708948321	1.69293872	5.691049344	3.44E-08	1.07E-07	7.899575898
SH3KBP	-0.414275129	4.58648888	-5.690655804	3.45E-08	1.07E-07	7.897589106
ZNF740	-0.378588643	3.96777888	-5.687234481	3.51E-08	1.09E-07	7.880320977
SAFB2	-0.321222463	5.26704415	-5.686880425	3.52E-08	1.09E-07	7.87853444
ZDHHC2	-0.430574434	2.32778492	-5.685985069	3.54E-08	1.10E-07	7.874016936
AK9	-0.314972351	1.97055476	-5.684588642	3.56E-08	1.10E-07	7.866972393
TRAPPC	-0.298823378	0.71046441	-5.684428841	3.56E-08	1.11E-07	7.866166327
CCDC47	-0.485130836	6.18312033	-5.683117833	3.59E-08	1.11E-07	7.85955406
MEOX2	-0.594788679	1.91728313	-5.683111911	3.59E-08	1.11E-07	7.859524197
RNF114	-0.331050179	6.04854681	-5.682793914	3.59E-08	1.11E-07	7.857920508
TCAIM	-0.297554742	3.00619802	-5.681996791	3.61E-08	1.12E-07	7.853900851
RASD2	0.908080059	3.75114117	5.681541862	3.62E-08	1.12E-07	7.851606973
RMND5A	-0.449341856	4.54599557	-5.681036865	3.63E-08	1.12E-07	7.849060809
MXRA8	1.182550039	7.00258246	5.680915377	3.63E-08	1.12E-07	7.848448298
ASAP3	-0.396326344	5.08539648	-5.680366887	3.64E-08	1.13E-07	7.845683085

SMARCD	-0.346571363	5.17453669	-5.678172876	3.68E-08	1.14E-07	7.834624054
ESPN	0.987804012	1.70434802	5.677630564	3.69E-08	1.14E-07	7.831891014
POLL	-0.308095498	4.2561497	-5.677461354	3.70E-08	1.14E-07	7.831038301
LILRA6	0.303088469	0.60407646	5.677126107	3.70E-08	1.15E-07	7.829348926
DPP3	-0.363513787	4.70887846	-5.676535705	3.71E-08	1.15E-07	7.826373965
KCNE4	-0.665760954	2.96398749	-5.675076085	3.74E-08	1.16E-07	7.819020149
RPL15	-0.345895349	8.90165354	-5.674944461	3.74E-08	1.16E-07	7.818357078
RPRD1B	-0.444266753	4.20343521	-5.674745218	3.75E-08	1.16E-07	7.817353387
SURF4	-0.459532768	7.60183916	-5.673766829	3.77E-08	1.16E-07	7.812425144
EIF2A	-0.381331819	6.11433035	-5.673325254	3.78E-08	1.17E-07	7.810201099
CD99L2	-0.391230292	4.66427348	-5.673102106	3.78E-08	1.17E-07	7.80907724
AIF1L	-0.775485354	5.94879575	-5.671663171	3.81E-08	1.18E-07	7.801831032
BRI3BP	-0.379395374	3.04077548	-5.670803626	3.83E-08	1.18E-07	7.797503203
ENOSF1	-0.466575198	3.94524785	-5.67075506	3.83E-08	1.18E-07	7.79725869
SMOC2	-1.033783811	4.0308119	-5.66882945	3.87E-08	1.19E-07	7.787565112
PNRC1	-0.357507278	6.76681405	-5.667241124	3.90E-08	1.20E-07	7.779571358
PECAM1	-0.499492919	6.14465687	-5.66600299	3.92E-08	1.21E-07	7.773341258
ZNF572	-0.347222043	1.73408104	-5.665099944	3.94E-08	1.22E-07	7.768797942
ARFIP1	-0.38964897	4.99524068	-5.663660648	3.97E-08	1.22E-07	7.761557851
ZMYND1	-0.533954233	2.26668085	-5.663472968	3.97E-08	1.23E-07	7.760613871
HHAT	-0.445014773	3.36346613	-5.663085211	3.98E-08	1.23E-07	7.758663633
IFT172	-0.365281603	3.72759549	-5.66283808	3.99E-08	1.23E-07	7.757420729
SYT13	-1.03290575	2.44162812	-5.66256509	3.99E-08	1.23E-07	7.756047827
ZNF280D	-0.331455968	2.7971287	-5.66251402	3.99E-08	1.23E-07	7.755790991
TTC5	-0.325608252	2.6405943	-5.66229232	4.00E-08	1.23E-07	7.754676078
SIRT7	-0.318480907	3.17492992	-5.662154628	4.00E-08	1.23E-07	7.753983649
KIAA132	-0.682575948	2.23611084	-5.660804104	4.03E-08	1.24E-07	7.747192811
GSTO1	-0.301386877	7.40035566	-5.659327358	4.06E-08	1.25E-07	7.739768736
CSF1	0.567745209	4.18967932	5.658909277	4.07E-08	1.25E-07	7.737667178
KCNJ10	-0.447288105	0.80257605	-5.658883322	4.07E-08	1.25E-07	7.737536718
DBN1	-0.414103233	5.95791286	-5.658803175	4.07E-08	1.25E-07	7.737133857
LRTM2	-0.195434623	0.14318698	-5.658537192	4.08E-08	1.25E-07	7.735796934
SEL1L3	0.901109155	5.08316286	5.656759291	4.11E-08	1.27E-07	7.726861816
CD1A	1.121643227	1.69943714	5.656269913	4.12E-08	1.27E-07	7.724402752
PRRT1	-0.469999612	1.61909773	-5.655808096	4.13E-08	1.27E-07	7.722082333
PKP2	0.652521204	1.25530405	5.655336891	4.14E-08	1.27E-07	7.719714897
CARD8	-0.354789555	3.07196039	-5.6552747	4.15E-08	1.27E-07	7.719402447
SARS	-0.420858616	7.21420117	-5.655130986	4.15E-08	1.28E-07	7.718680432
GIMAP4	-0.516835988	5.17634766	-5.654525278	4.16E-08	1.28E-07	7.715637528
KANSL3	-0.355268246	4.37032897	-5.653712151	4.18E-08	1.28E-07	7.711553017

LRRN4	0.284117332	0.34454056	5.651418857	4.23E-08	1.30E-07	7.700035757
PDZRN3	0.551330098	1.08999327	5.649739962	4.27E-08	1.31E-07	7.691606408
PHLDB2	0.624081717	2.57900524	5.649421567	4.27E-08	1.31E-07	7.690008038
NSMCE2	-0.257703621	4.19710484	-5.648457194	4.29E-08	1.32E-07	7.68516723
BPNT1	-0.380090996	4.98474791	-5.647594315	4.31E-08	1.32E-07	7.680836431
NOP58	-0.340208662	5.56848907	-5.64732243	4.32E-08	1.33E-07	7.679471943
HBS1L	-0.309588824	3.51746411	-5.646836551	4.33E-08	1.33E-07	7.677033628
ZNF273	-0.468838217	2.12122313	-5.646344745	4.34E-08	1.33E-07	7.674565738
C20orf14	-0.174615647	0.53821761	-5.644656861	4.38E-08	1.34E-07	7.666097174
ACP5	0.976900677	4.48843805	5.644338145	4.39E-08	1.35E-07	7.664498315
CCDC74	-0.558564438	1.93086884	-5.643962141	4.40E-08	1.35E-07	7.662612152
ERMN	0.154780487	0.19929756	5.643724158	4.40E-08	1.35E-07	7.661418402
MAP2K1	-0.418534441	5.13727118	-5.643686387	4.40E-08	1.35E-07	7.661228943
STC1	-0.819655534	4.92284956	-5.643590017	4.40E-08	1.35E-07	7.660745553
SLITRK5	-0.41163401	0.36235938	-5.641666635	4.45E-08	1.36E-07	7.651099254
NFE2L1	-0.434187335	7.42770395	-5.641181155	4.46E-08	1.37E-07	7.648664837
FGFR2	-0.661544369	4.48245912	-5.640684597	4.47E-08	1.37E-07	7.646175045
AP3D1	-0.356719966	5.9587848	-5.640004363	4.49E-08	1.37E-07	7.642764553
TRIM68	-0.433936889	4.03644723	-5.63985886	4.49E-08	1.38E-07	7.64203509
EBF2	-0.516398752	1.66792029	-5.639491522	4.50E-08	1.38E-07	7.640193533
TOLLIP	-0.307596247	5.53476754	-5.638761225	4.52E-08	1.38E-07	7.636532662
GPC3	-0.676045186	1.23443113	-5.638306686	4.53E-08	1.39E-07	7.63425431
SIGMAR	-0.330609446	6.34161884	-5.636950331	4.56E-08	1.39E-07	7.6274565
NAPB	-0.339811418	3.22901399	-5.636683293	4.56E-08	1.40E-07	7.626118301
GTF2E2	-0.291379135	5.26156609	-5.636231539	4.58E-08	1.40E-07	7.623854554
DHX30	-0.309220757	5.20949085	-5.636157709	4.58E-08	1.40E-07	7.623484606
CCDC13	-0.389311745	4.69106432	-5.63596773	4.58E-08	1.40E-07	7.622532671
NFE2L2	-0.510010693	6.32698087	-5.635953522	4.58E-08	1.40E-07	7.622461478
GID4	-0.337804704	3.52457728	-5.635165385	4.60E-08	1.41E-07	7.618512608
GGCX	-0.40659131	3.9436538	-5.635030767	4.60E-08	1.41E-07	7.617838161
ARMC10	-0.296830146	4.57679687	-5.633803078	4.63E-08	1.42E-07	7.611687934
METRNL	-0.392917446	6.08101271	-5.63363243	4.64E-08	1.42E-07	7.610833136
MTPN	-0.455887205	7.09078203	-5.633586053	4.64E-08	1.42E-07	7.610600834
TDP2	-0.302264	5.18370153	-5.633083772	4.65E-08	1.42E-07	7.608084981
SH3BP1	0.556526457	2.64353251	5.632865254	4.66E-08	1.42E-07	7.606990514
DOK2	0.73185765	2.60984491	5.632511801	4.66E-08	1.42E-07	7.60522028
CFHR3	0.145519007	0.1997845	5.630611189	4.71E-08	1.44E-07	7.595702732
ZNF764	-0.263152937	2.9527453	-5.630598933	4.71E-08	1.44E-07	7.595641364
PTPRA	-0.360949178	6.4334411	-5.630238661	4.72E-08	1.44E-07	7.593837546
HACD4	-0.3700784	2.15703766	-5.629438548	4.74E-08	1.45E-07	7.589831836

ADAMTS	0.485852654	1.10438792	5.628278023	4.77E-08	1.45E-07	7.584022535
CSF2RB	0.70359438	2.31921411	5.627047191	4.80E-08	1.46E-07	7.57786232
STIM2	-0.351326125	3.74019372	-5.626620646	4.81E-08	1.47E-07	7.57572774
SBNO1	-0.414284581	4.09485227	-5.62592018	4.82E-08	1.47E-07	7.57222264
RAPGEF	-0.530545507	3.84418117	-5.625565253	4.83E-08	1.47E-07	7.570446729
LRRC3B	-0.086631006	0.10095335	-5.624211013	4.87E-08	1.48E-07	7.563671465
SNTB2	-0.414192109	4.16266132	-5.623465385	4.89E-08	1.49E-07	7.55994163
PEX19	-0.437386937	6.2006978	-5.623071433	4.90E-08	1.49E-07	7.557971134
TBCEL	-0.387770956	3.26567782	-5.622699513	4.91E-08	1.49E-07	7.556110935
YWHAQ	-0.4417417	7.57901999	-5.622383102	4.91E-08	1.50E-07	7.554528443
MFSD5	-0.270291141	5.17296578	-5.622322774	4.92E-08	1.50E-07	7.554226726
NRARP	-0.610155368	2.88090073	-5.621626751	4.93E-08	1.50E-07	7.550745932
RARS	-0.317691283	5.49711567	-5.620572467	4.96E-08	1.51E-07	7.545474118
HILPDA	-0.359706001	3.28203887	-5.619007018	5.00E-08	1.52E-07	7.537647708
RAB26	-0.438612034	1.69299459	-5.618970958	5.00E-08	1.52E-07	7.537467452
LRRC15	0.949322273	1.15492593	5.618947319	5.00E-08	1.52E-07	7.537349279
SMIM32	-0.638719925	0.55160648	-5.615451418	5.09E-08	1.55E-07	7.519877928
ORMDL1	-0.362542028	5.11832794	-5.615321204	5.10E-08	1.55E-07	7.519227323
RNF145	-0.526264211	5.95580655	-5.61527068	5.10E-08	1.55E-07	7.518974887
MSTO1	-0.308273573	3.10214928	-5.61510148	5.10E-08	1.55E-07	7.518129522
CARMIL3	-0.178810876	0.5479286	-5.614956899	5.11E-08	1.55E-07	7.51740717
R3HCC1I	-0.378151841	3.59863785	-5.614267333	5.12E-08	1.56E-07	7.513962184
LRSAM1	-0.323943949	4.32698124	-5.613174861	5.15E-08	1.57E-07	7.508505007
ING5	-0.38641315	3.63267076	-5.613140646	5.15E-08	1.57E-07	7.508334111
IFFO2	-0.519529051	4.13946677	-5.613096097	5.15E-08	1.57E-07	7.508111597
RTCB	-0.440125127	6.13222663	-5.612256338	5.18E-08	1.57E-07	7.503917394
PCGF1	-0.279146526	4.14422335	-5.611689272	5.19E-08	1.58E-07	7.501085441
ZSWIM3	-0.320651099	2.61143737	-5.609958498	5.24E-08	1.59E-07	7.492443266
BTBD3	-0.441135241	3.95537913	-5.609683625	5.25E-08	1.59E-07	7.491070948
NKX3-2	0.317968784	0.38835978	5.608745398	5.27E-08	1.60E-07	7.486387201
TAPBPL	-0.507336145	5.33675656	-5.608654493	5.27E-08	1.60E-07	7.485933425
APP	-0.529624115	9.04198383	-5.606728855	5.33E-08	1.62E-07	7.476322432
NRIP1	0.806045741	4.10952845	5.606653384	5.33E-08	1.62E-07	7.475945806
CFAP69	-0.38603927	1.61379798	-5.60533362	5.36E-08	1.63E-07	7.469360328
AKAP8	-0.330523917	4.47298864	-5.604798359	5.38E-08	1.63E-07	7.466689778
C1QL2	0.898015498	0.99252397	5.604380229	5.39E-08	1.63E-07	7.464603764
FHL2	-0.645400866	2.97145557	-5.604136441	5.40E-08	1.64E-07	7.463387584
WNT7A	0.355540786	0.32951154	5.604099771	5.40E-08	1.64E-07	7.463204653
ABCG5	-0.061638335	0.09312504	-5.603992273	5.40E-08	1.64E-07	7.462668397
TRPM5	-0.374481555	0.57436473	-5.6033107	5.42E-08	1.64E-07	7.459268529

ZNF227	-0.315402135	2.83887935	-5.601292061	5.48E-08	1.66E-07	7.449200923
ZNF512	-0.417356026	4.89190818	-5.600445867	5.50E-08	1.67E-07	7.444981524
NSUN5	-0.313513139	4.39997881	-5.599780514	5.52E-08	1.67E-07	7.441664206
YTHDF2	-0.315445889	5.69976913	-5.599441891	5.53E-08	1.67E-07	7.439976015
POP1	-0.25983818	2.04710993	-5.599166951	5.54E-08	1.68E-07	7.438605372
IL2RG	1.059003416	3.39784499	5.598244066	5.56E-08	1.68E-07	7.434004952
APCDD1	0.310496983	0.31868109	5.597121534	5.60E-08	1.69E-07	7.428410129
SERF1B	-0.199904595	0.63927688	-5.596843327	5.60E-08	1.69E-07	7.42702365
MCAM	-0.60938512	5.24128704	-5.596831332	5.60E-08	1.69E-07	7.42696387
GPX7	-0.565114598	4.88291588	-5.596814563	5.60E-08	1.69E-07	7.426880301
GRAMD4	-0.484220458	4.89644586	-5.596691977	5.61E-08	1.70E-07	7.426269399
MAFF	0.752635005	3.86543024	5.596489546	5.61E-08	1.70E-07	7.425260614
GNB2	-0.347971937	7.84790762	-5.596186188	5.62E-08	1.70E-07	7.423748929
IKBKG	-0.360786109	2.21558429	-5.594235842	5.68E-08	1.72E-07	7.414031538
CAB39	-0.468684323	5.74994252	-5.594170101	5.68E-08	1.72E-07	7.413704033
VPS52	-0.379074739	5.31838698	-5.593649755	5.70E-08	1.72E-07	7.411111939
CKMT1A	-0.360530093	0.9285536	-5.593547958	5.70E-08	1.72E-07	7.410604858
PPP2R1I	-0.340134475	3.36798203	-5.593260636	5.71E-08	1.72E-07	7.409173668
EPN1	-0.293657064	4.47167324	-5.591701129	5.75E-08	1.74E-07	7.401406553
CST6	1.899263877	6.81082801	5.590857041	5.78E-08	1.74E-07	7.397203283
NDN	-0.657651791	5.37052767	-5.590512604	5.79E-08	1.75E-07	7.395488249
SNURF	-0.362334613	1.76848366	-5.590157644	5.80E-08	1.75E-07	7.393720906
GJB1	-0.329564432	0.28690754	-5.589245402	5.83E-08	1.76E-07	7.389179251
OSBPL9	-0.360984559	5.01103183	-5.589108742	5.83E-08	1.76E-07	7.388498933
HLA-DOI	0.908181639	2.39498261	5.589076716	5.83E-08	1.76E-07	7.388339505
GPR84	0.422217056	1.01768019	5.588621661	5.85E-08	1.76E-07	7.386074256
ZNF607	-0.323825204	2.83137408	-5.588603621	5.85E-08	1.76E-07	7.385984457
C10orf90	-0.321826041	0.26801128	-5.58857902	5.85E-08	1.76E-07	7.385861995
ZNF197	-0.354089181	3.43530486	-5.587860377	5.87E-08	1.77E-07	7.382284934
SAA1	0.915014559	1.2297075	5.587627849	5.88E-08	1.77E-07	7.381127593
TAF5	-0.26236772	2.0867355	-5.587155284	5.89E-08	1.77E-07	7.378775663
TNFSF4	0.478705408	1.28323467	5.585496237	5.94E-08	1.79E-07	7.370519896
CPNE4	0.867356344	1.35172507	5.585378003	5.94E-08	1.79E-07	7.369931612
CDC42EI	0.807067391	4.50759605	5.584817958	5.96E-08	1.79E-07	7.367145192
TAF9	-0.261660292	5.05964682	-5.58421483	5.98E-08	1.80E-07	7.364144661
FJX1	-0.516830411	2.94500493	-5.584170173	5.98E-08	1.80E-07	7.363922504
TAF7	-0.391783785	7.04563499	-5.582982933	6.02E-08	1.81E-07	7.358016826
THBS2	1.033770729	3.47646105	5.582944818	6.02E-08	1.81E-07	7.357827244
DNTTIP1	-0.309396971	5.63169497	-5.580811268	6.08E-08	1.83E-07	7.347216867
MT2A	-0.74318513	8.94116223	-5.580081365	6.11E-08	1.84E-07	7.343587708

ST3GAL1	-0.623976837	6.62468225	-5.579512298	6.12E-08	1.84E-07	7.340758498
SCARA3	1.202658309	6.13413868	5.578876851	6.14E-08	1.85E-07	7.33759954
ENG	-0.613386466	7.39891507	-5.578719622	6.15E-08	1.85E-07	7.336817959
RPAP3	-0.333228923	3.93651933	-5.577809407	6.18E-08	1.86E-07	7.332293653
VMA21	-0.320326366	4.89862537	-5.577659083	6.18E-08	1.86E-07	7.331546512
HNRNPA	-0.341980845	2.86047133	-5.574886015	6.27E-08	1.88E-07	7.317766572
FIP1L1	-0.374099562	4.00494391	-5.573666973	6.31E-08	1.90E-07	7.311710603
DNM1L	-0.300226112	4.58507545	-5.57365578	6.31E-08	1.90E-07	7.311655004
ARHGAP	-0.554205542	4.37630383	-5.572870158	6.34E-08	1.90E-07	7.307752738
DEK	-0.480620967	6.52784483	-5.572238456	6.36E-08	1.91E-07	7.304615324
SIRPA	0.573758065	3.70836022	5.570983657	6.40E-08	1.92E-07	7.29838405
CBWD6	-0.147359536	0.52592636	-5.570330778	6.42E-08	1.93E-07	7.29514232
KLHL13	0.394080555	0.65397349	5.569768991	6.44E-08	1.93E-07	7.292353129
PGRMC1	-0.457273681	7.77529955	-5.569761095	6.44E-08	1.93E-07	7.292313927
C20orf27	-0.398222657	5.08672556	-5.56972435	6.44E-08	1.93E-07	7.292131499
PPP1R1	-0.293847798	6.51711545	-5.569309989	6.45E-08	1.94E-07	7.290074409
KATNB1	-0.309022191	3.91066616	-5.569195777	6.46E-08	1.94E-07	7.289507428
MVP	0.590705312	6.76557537	5.568384537	6.48E-08	1.94E-07	7.285480444
ANO5	-0.526127996	1.60456712	-5.567735381	6.51E-08	1.95E-07	7.282258379
BATF3	0.49317721	1.42813085	5.567402244	6.52E-08	1.95E-07	7.280604975
GRB2	-0.326618785	6.4922789	-5.567291171	6.52E-08	1.95E-07	7.280053721
PLAC8L1	-0.269665153	0.60707488	-5.566421284	6.55E-08	1.96E-07	7.275736792
WDR3	-0.381294208	3.08005185	-5.565683799	6.57E-08	1.97E-07	7.272077342
RAB11FI	-0.499713087	3.79241409	-5.563957604	6.63E-08	1.99E-07	7.263513327
PPP2R2A	-0.374416773	4.43400498	-5.563246736	6.66E-08	1.99E-07	7.259987162
P3H2	-0.460814414	2.19245527	-5.563151518	6.66E-08	1.99E-07	7.259514875
IRS2	0.624623	3.84873269	5.562846737	6.67E-08	2.00E-07	7.258003181
SECISBF	-0.384351214	4.3199991	-5.561784535	6.71E-08	2.01E-07	7.252735232
HLA-DQA	1.137484414	4.75495178	5.56166045	6.71E-08	2.01E-07	7.252119888
ZHX1-C8	-0.205666013	0.93140811	-5.561315264	6.72E-08	2.01E-07	7.25040815
HLA-DOA	1.15772788	3.92011842	5.56102471	6.73E-08	2.01E-07	7.248967393
NATD1	-0.461900127	4.53736978	-5.56085496	6.74E-08	2.02E-07	7.248125686
C10orf88	-0.285395638	3.11003411	-5.560338272	6.76E-08	2.02E-07	7.245563815
ABAT	-0.746915677	4.97420627	-5.559432255	6.79E-08	2.03E-07	7.241071994
GK	-0.291388039	1.85809407	-5.558389951	6.82E-08	2.04E-07	7.235905208
PQBP1	-0.328570281	6.59795706	-5.557863791	6.84E-08	2.05E-07	7.23329728
SERHL2	-0.375082647	1.22920767	-5.556366437	6.90E-08	2.06E-07	7.225876652
EXOSC2	-0.291576734	3.62742438	-5.555866708	6.91E-08	2.06E-07	7.223400431
NEU1	-0.396936258	6.63415716	-5.555860055	6.91E-08	2.06E-07	7.223367467
NR1D1	0.871614803	5.70848627	5.555678374	6.92E-08	2.07E-07	7.222467261

SCGB3A	-0.396372607	0.32174235	-5.554242977	6.97E-08	2.08E-07	7.215355842
S100A4	1.136916416	7.8499207	5.55296192	7.02E-08	2.09E-07	7.209010294
CACNA1	-0.597843992	2.33135043	-5.552253	7.04E-08	2.10E-07	7.205499241
AGPAT3	-0.357080147	4.91692483	-5.551438908	7.07E-08	2.11E-07	7.201467741
HACD3	-0.564780519	6.17900343	-5.551424738	7.07E-08	2.11E-07	7.201397575
DCAF8	-0.346722602	5.32173938	-5.551173294	7.08E-08	2.11E-07	7.200152488
PPP1R8	-0.335770593	5.53316877	-5.550960634	7.09E-08	2.11E-07	7.199099477
CEP89	-0.27578809	3.25640185	-5.549938097	7.13E-08	2.13E-07	7.194036729
TPGS2	-0.32010521	4.17726908	-5.549777543	7.13E-08	2.13E-07	7.193241868
TMOD1	-0.832461765	2.8756866	-5.547933972	7.20E-08	2.15E-07	7.184116098
METTL17	-0.326579318	4.27339205	-5.547533481	7.21E-08	2.15E-07	7.182133965
INTS8	-0.323738744	3.35303464	-5.547413063	7.22E-08	2.15E-07	7.181538005
ARF5	-0.297045392	7.47545312	-5.546711916	7.24E-08	2.16E-07	7.178068171
UTP4	-0.334525587	4.50869589	-5.546615258	7.25E-08	2.16E-07	7.177589858
TREM1	0.861766577	2.17166357	5.546326623	7.26E-08	2.16E-07	7.176161586
PIGG	-0.366047675	3.75366065	-5.546225476	7.26E-08	2.16E-07	7.175661084
SERBP1	-0.362931429	6.54329315	-5.545472849	7.29E-08	2.17E-07	7.171937127
EEF1AKI	-0.413955092	4.55752391	-5.545228757	7.30E-08	2.17E-07	7.170729457
C16orf58	-0.575024992	5.36979951	-5.54465689	7.32E-08	2.18E-07	7.167900251
ALOX5AI	0.926271674	4.15132612	5.544235674	7.34E-08	2.18E-07	7.165816513
PRDX4	-0.429983107	6.31543055	-5.54371441	7.36E-08	2.19E-07	7.163238011
TMEM23	-0.160322351	0.12138048	-5.542707966	7.39E-08	2.20E-07	7.158260041
CD86	0.648185545	2.28987523	5.542470869	7.40E-08	2.20E-07	7.15708744
EGFL6	0.474637799	0.88323414	5.542297116	7.41E-08	2.20E-07	7.156228143
NR2F2	-0.43948331	4.03804157	-5.540827918	7.46E-08	2.22E-07	7.14896304
PAN2	-0.474718111	4.30175817	-5.540800103	7.47E-08	2.22E-07	7.148825511
SMARCE	-0.338172562	6.70208745	-5.540424312	7.48E-08	2.22E-07	7.146967494
IL36RN	0.490248859	0.90568988	5.540338426	7.48E-08	2.22E-07	7.146542868
ZFP2	-0.32422323	2.31141878	-5.539880174	7.50E-08	2.23E-07	7.144277304
SRF	-0.437711035	5.39255787	-5.539763093	7.50E-08	2.23E-07	7.143698485
JUN	-0.82418317	8.07335754	-5.539653852	7.51E-08	2.23E-07	7.143158435
VKORC1	-0.35602971	5.39421549	-5.539388037	7.52E-08	2.23E-07	7.141844377
CCT6A	-0.307239164	6.64358975	-5.538632699	7.55E-08	2.24E-07	7.138110624
SART1	-0.29291514	5.94562584	-5.537972416	7.57E-08	2.25E-07	7.134847068
GALNT9	-0.81174383	1.6977497	-5.535387261	7.67E-08	2.28E-07	7.122072469
RCAN1	-0.764796712	3.9468429	-5.534483663	7.71E-08	2.29E-07	7.117608424
CNKSR2	0.408504141	0.78297929	5.534351348	7.71E-08	2.29E-07	7.116954796
GNAL	-0.587616287	2.15329394	-5.534055222	7.73E-08	2.29E-07	7.115492
RPL37	-0.344751193	7.79473046	-5.533959855	7.73E-08	2.29E-07	7.115020916
GLYR1	-0.410379157	6.01790314	-5.533641709	7.74E-08	2.30E-07	7.113449434

HSPB1	-0.47468825	9.47114172	-5.533506	7.75E-08	2.30E-07	7.112779123
SHTN1	-0.415071651	4.26153228	-5.533207242	7.76E-08	2.30E-07	7.111303496
DIPK2A	-0.436002468	3.69161418	-5.531657307	7.82E-08	2.32E-07	7.103649076
NEMP2	-0.275504989	2.28975754	-5.531651059	7.82E-08	2.32E-07	7.103618224
PTPN21	-0.493610452	4.40892591	-5.531621473	7.82E-08	2.32E-07	7.103472126
FBXO30	-0.470567011	3.04249473	-5.531081554	7.84E-08	2.32E-07	7.100806121
TEDDM1	-0.078394368	0.08063348	-5.530864883	7.85E-08	2.33E-07	7.099736304
NIPA1	-0.468433431	3.28153719	-5.529603367	7.90E-08	2.34E-07	7.093508198
DNMT3B	-0.369784195	1.64727666	-5.529010297	7.93E-08	2.35E-07	7.090580594
IPO8	-0.374263458	4.59477749	-5.52900151	7.93E-08	2.35E-07	7.090537219
DOLK	-0.337995458	5.13578651	-5.527942371	7.97E-08	2.36E-07	7.085309561
ZNF85	-0.351484395	2.53119446	-5.527760694	7.98E-08	2.36E-07	7.08441292
FNDC5	-0.652121532	1.9680065	-5.527723757	7.98E-08	2.36E-07	7.084230629
CNOT7	-0.357784081	4.85239063	-5.527376662	7.99E-08	2.36E-07	7.082517672
C2orf68	-0.288615011	4.52668728	-5.526923552	8.01E-08	2.37E-07	7.080281646
BARX2	-0.419153031	0.38624849	-5.525900513	8.05E-08	2.38E-07	7.075233647
RHOA	-0.333861245	9.28706966	-5.52553629	8.07E-08	2.39E-07	7.073436628
PHC2	-0.3391291	6.44075953	-5.52357553	8.15E-08	2.41E-07	7.063764167
COQ8A	-0.376220037	3.82426701	-5.522491631	8.19E-08	2.42E-07	7.058418428
MAP2K2	-0.289820442	6.08202049	-5.522319418	8.20E-08	2.42E-07	7.057569158
ZBTB12	-0.464370612	3.24983805	-5.522246597	8.21E-08	2.42E-07	7.057210051
AGGF1	-0.373863042	4.54132202	-5.520611049	8.27E-08	2.44E-07	7.04914545
NPTN	-0.459500303	6.5058095	-5.519121024	8.34E-08	2.46E-07	7.04180004
GALNT1	-0.443591877	1.17119319	-5.518622602	8.36E-08	2.47E-07	7.039343304
NCAN	-0.098981196	0.10950563	-5.518164625	8.38E-08	2.47E-07	7.037086077
CCR9	0.302964808	0.24852242	5.518153198	8.38E-08	2.47E-07	7.03702976
WDYHV1	-0.331704502	3.76959397	-5.517328838	8.41E-08	2.48E-07	7.032967122
FUBP3	-0.459412439	5.50150235	-5.516972938	8.43E-08	2.49E-07	7.031213312
S100A6	0.822572002	12.1087699	5.516302998	8.46E-08	2.50E-07	7.027912207
RAB11A	-0.355101776	6.64701355	-5.515646917	8.48E-08	2.50E-07	7.024679702
CCR4	0.567010491	0.85079876	5.515622764	8.49E-08	2.50E-07	7.02456071
ZSCAN2	-0.349427363	2.86476351	-5.515527494	8.49E-08	2.50E-07	7.024091342
MEI4	-0.580008767	1.15290524	-5.515115308	8.51E-08	2.51E-07	7.022060694
KCNN3	-0.445516856	1.95392901	-5.513501164	8.58E-08	2.53E-07	7.014109709
FAM174	-0.316236368	5.67306816	-5.513477175	8.58E-08	2.53E-07	7.013991559
MFN2	-0.420182884	6.84072511	-5.512356085	8.63E-08	2.54E-07	7.008470368
ABCC6	-0.525165676	2.7980585	-5.510628436	8.70E-08	2.56E-07	6.999963701
PCK2	-0.438102649	5.265666	-5.510046614	8.73E-08	2.57E-07	6.997099374
CD207	1.1281425	1.95969422	5.509853598	8.74E-08	2.57E-07	6.996149201
DDX24	-0.366711906	6.53175941	-5.508844981	8.78E-08	2.59E-07	6.991184453

SNTG2	-0.268081923	0.53910801	-5.507660593	8.84E-08	2.60E-07	6.985355415
SLIT2	0.847364227	2.81013089	5.507097578	8.86E-08	2.61E-07	6.98258485
PSME4	-0.377214904	4.36906816	-5.506917492	8.87E-08	2.61E-07	6.981698703
CWC22	-0.390384228	4.8011783	-5.506809648	8.87E-08	2.61E-07	6.981168047
RPS10	-0.388860219	7.94497776	-5.506311352	8.90E-08	2.62E-07	6.978716245
L2HGDH	-0.415871511	2.25255523	-5.503280904	9.03E-08	2.66E-07	6.963809078
HPGDS	0.519706919	1.68969364	5.502630849	9.06E-08	2.67E-07	6.960612213
ZNF586	-0.289186822	2.8287882	-5.502220734	9.08E-08	2.67E-07	6.958595488
PIK3R4	-0.460505965	4.55979703	-5.502220381	9.08E-08	2.67E-07	6.958593752
RIMS2	0.577943361	1.27493081	5.502001871	9.09E-08	2.67E-07	6.957519285
GSTO2	-0.41359612	3.07036758	-5.501748691	9.11E-08	2.68E-07	6.95627438
MRPS30	-0.283460748	4.25199575	-5.501651873	9.11E-08	2.68E-07	6.955798333
FZD3	-0.423799855	2.46157788	-5.501204531	9.13E-08	2.68E-07	6.953598859
NME3	-0.58284389	6.60759861	-5.500419414	9.17E-08	2.69E-07	6.949738967
RAB3IP	-0.336058371	2.63283921	-5.499691956	9.20E-08	2.70E-07	6.946162934
KRTDAP	-0.401826345	1.08566704	-5.499003275	9.23E-08	2.71E-07	6.942777865
HDAC4	-0.371558581	2.11198735	-5.49850476	9.26E-08	2.72E-07	6.940327724
BMPR1A	-0.410681898	3.83096323	-5.498218849	9.27E-08	2.72E-07	6.93892259
FAM161E	-0.359623672	2.7351847	-5.498156857	9.27E-08	2.72E-07	6.938617929
DMPK	-0.527281363	3.65001608	-5.497442763	9.31E-08	2.73E-07	6.935108726
DLG1	-0.423193268	4.47816998	-5.496822078	9.34E-08	2.74E-07	6.932058843
WDR33	-0.307313643	3.92437367	-5.496817202	9.34E-08	2.74E-07	6.932034888
SDCBP	-0.413310648	6.37391053	-5.495812484	9.38E-08	2.75E-07	6.927098546
MFSD10	-0.475399247	6.21524247	-5.495398322	9.40E-08	2.76E-07	6.925063909
MFSD1	-0.410334703	5.26154777	-5.494650349	9.44E-08	2.77E-07	6.921389681
B3GALT(	-0.364733457	5.11667943	-5.494608116	9.44E-08	2.77E-07	6.921182232
OSGIN1	-0.591181336	2.94032095	-5.494202294	9.46E-08	2.77E-07	6.919188912
ZNF605	-0.346368764	2.74138968	-5.494201886	9.46E-08	2.77E-07	6.91918691
LANCL2	-0.309724605	4.49623294	-5.49363822	9.49E-08	2.78E-07	6.916418478
TSPYL1	-0.460573988	7.26264062	-5.493055802	9.52E-08	2.79E-07	6.913558185
CCDC16:	-0.565328199	1.92197974	-5.492001366	9.57E-08	2.80E-07	6.908380385
CDH3	1.162494428	4.99647139	5.491691871	9.58E-08	2.81E-07	6.906860764
HLCS	-0.393557256	3.65519477	-5.490010466	9.66E-08	2.83E-07	6.89860623
ZNF879	-0.337520804	2.38533128	-5.487239178	9.80E-08	2.87E-07	6.885005473
PNPLA8	-0.433337694	4.45531704	-5.487168232	9.80E-08	2.87E-07	6.88465736
CDC25B	0.558098007	5.19501591	5.48677629	9.82E-08	2.88E-07	6.882734265
RAB22A	-0.312836935	4.30788062	-5.48673757	9.83E-08	2.88E-07	6.882544291
MYNN	-0.302324813	3.14427676	-5.486522627	9.84E-08	2.88E-07	6.88148971
MIS18A	-0.307494175	3.76290996	-5.486046962	9.86E-08	2.88E-07	6.879156052
KIAA201:	-0.368469836	6.83602342	-5.485996393	9.86E-08	2.88E-07	6.878907966

DNAH6	-0.328087253	0.93744863	-5.485039683	9.91E-08	2.90E-07	6.874214779
MOV10L1	-0.130514217	0.25741545	-5.48440693	9.94E-08	2.91E-07	6.871111136
RMDN2	-0.34927571	2.17721359	-5.48438144	9.94E-08	2.91E-07	6.870986112
DALRD3	-0.355607049	4.79528628	-5.483791957	9.97E-08	2.91E-07	6.868094973
NR1I3	-0.107016115	0.28081968	-5.482659392	1.00E-07	2.93E-07	6.862540951
PRPF38A	-0.362737224	4.84714435	-5.482079705	1.01E-07	2.94E-07	6.859698561
VPS37B	-0.353738505	4.86639868	-5.481832019	1.01E-07	2.94E-07	6.858484146
CXXC1	-0.329217024	5.06435395	-5.481280134	1.01E-07	2.95E-07	6.855778393
HOXA4	-0.267221078	0.81998445	-5.481184499	1.01E-07	2.95E-07	6.855309541
SPINK13	-0.404775134	0.85793071	-5.48111564	1.01E-07	2.95E-07	6.854971959
SCAND1	-0.473255836	6.22078834	-5.480832531	1.01E-07	2.96E-07	6.853584066
TNFRSF9	0.452551874	0.68658588	5.480584472	1.01E-07	2.96E-07	6.852368042
EHD1	-0.354991886	5.06133868	-5.478696015	1.02E-07	2.99E-07	6.843111957
URGCP-I	-0.127874981	0.28198056	-5.478559979	1.02E-07	2.99E-07	6.842445288
PEX1	-0.406755704	3.33479036	-5.477852897	1.03E-07	3.00E-07	6.838980312
KCNT2	0.418997689	0.90513704	5.47643035	1.04E-07	3.02E-07	6.832010351
SYNCRIP	-0.381082675	6.00328123	-5.475005789	1.04E-07	3.04E-07	6.825031958
CAMKK2	-0.314806961	4.44563116	-5.474245143	1.05E-07	3.05E-07	6.821306421
ZNF862	-0.39881695	3.03853003	-5.473606806	1.05E-07	3.06E-07	6.818180253
ZNF571	-0.264228361	2.01394542	-5.470716263	1.07E-07	3.11E-07	6.804027813
UTP14C	-0.559697202	4.05011375	-5.470574805	1.07E-07	3.11E-07	6.80333537
MEX3A	0.568684661	1.80085559	5.470359191	1.07E-07	3.11E-07	6.802279961
ACACA	-0.402826363	3.34509268	-5.468639523	1.08E-07	3.14E-07	6.793863499
DDX19B	-0.253535085	3.94444504	-5.468215518	1.08E-07	3.14E-07	6.791788638
CD37	0.746661417	2.8868923	5.467980851	1.08E-07	3.15E-07	6.790640354
HRAS	-0.385486237	5.49485236	-5.467371692	1.08E-07	3.16E-07	6.787659774
SIGLEC1	0.804093043	1.5774539	5.464913795	1.10E-07	3.19E-07	6.775636081
PTPRQ	-0.337162558	0.39679242	-5.464852059	1.10E-07	3.19E-07	6.775334132
RPS14	-0.354323072	8.91701108	-5.464771668	1.10E-07	3.20E-07	6.774940946
PPP1R3C	-0.592170988	2.03482049	-5.464746136	1.10E-07	3.20E-07	6.774816069
GTPBP10	-0.277912687	2.75866033	-5.464307728	1.10E-07	3.20E-07	6.772671938
SENP2	-0.388602523	4.28673612	-5.464286996	1.10E-07	3.20E-07	6.772570551
C15orf48	0.996887612	3.9229348	5.463711975	1.10E-07	3.21E-07	6.769758498
TTC9	-0.447609351	4.30369008	-5.463316331	1.11E-07	3.22E-07	6.767823798
SSR3	-0.419645156	6.98849569	-5.463236365	1.11E-07	3.22E-07	6.767432776
TMEM20	-0.41239623	4.06224889	-5.462572683	1.11E-07	3.23E-07	6.764187656
TTC38	-0.35136845	4.65577105	-5.462401996	1.11E-07	3.23E-07	6.763353121
CDCA7L	-0.678704047	4.2216569	-5.461826724	1.11E-07	3.24E-07	6.760540609
PERP	0.717314255	7.02595858	5.460362543	1.12E-07	3.26E-07	6.753383264
HEPHL1	-0.258828312	0.50841766	-5.460274624	1.12E-07	3.26E-07	6.752953536

CRISPLD	-0.741437273	3.24506852	-5.458691268	1.13E-07	3.29E-07	6.745215439
BET1L	-0.310327583	6.04168194	-5.457165269	1.14E-07	3.31E-07	6.737759332
GJD4	-0.249590147	0.23071639	-5.456842096	1.14E-07	3.32E-07	6.736180502
COMMD2	-0.353677879	4.42146462	-5.456444023	1.14E-07	3.32E-07	6.73423586
THBS1	0.91717488	4.21851318	5.455395452	1.15E-07	3.34E-07	6.729113981
GGTLC2	-0.525496987	0.87863138	-5.454910741	1.15E-07	3.35E-07	6.726746611
PLOD3	-0.284421224	5.02162879	-5.454717467	1.16E-07	3.35E-07	6.72580269
PODXL2	-0.62624888	4.20191293	-5.454673162	1.16E-07	3.35E-07	6.725586315
EFCAB7	-0.312803861	2.29254025	-5.454601565	1.16E-07	3.35E-07	6.725236656
KLHDC10	-0.35425842	4.92098349	-5.453449078	1.16E-07	3.37E-07	6.719608729
GPN3	-0.363127914	4.64087209	-5.453096485	1.16E-07	3.38E-07	6.717887107
EDN2	-0.615349587	1.20320813	-5.452814631	1.17E-07	3.38E-07	6.716510944
PPP2R1A	-0.29214025	7.27771048	-5.452728333	1.17E-07	3.38E-07	6.716089605
TPM1	-0.396924082	4.77572297	-5.452555724	1.17E-07	3.38E-07	6.715246874
MLXIPL	0.815846031	2.66390787	5.450882187	1.18E-07	3.41E-07	6.707077246
PUDP	-0.366457695	4.4065316	-5.449885498	1.18E-07	3.43E-07	6.7022127
IKZF3	0.766562485	2.6120552	5.449025305	1.19E-07	3.44E-07	6.698014915
ZNF225	-0.273552628	1.67581493	-5.448564209	1.19E-07	3.45E-07	6.695764961
DAAM2	-0.76088467	2.86530064	-5.448328308	1.19E-07	3.45E-07	6.694613917
RTKN2	0.298534493	0.69633021	5.448119855	1.19E-07	3.46E-07	6.693596842
ZCCHC9	-0.340784443	3.5585632	-5.447482817	1.20E-07	3.47E-07	6.690488811
EFCAB5	-0.128105726	0.30571698	-5.446342586	1.20E-07	3.49E-07	6.684926479
NEK1	-0.432333901	2.95060746	-5.446261956	1.21E-07	3.49E-07	6.684533181
TFRC	-0.555451613	3.95214711	-5.444774224	1.21E-07	3.51E-07	6.677277127
ZNF235	-0.271948216	1.41902793	-5.441935678	1.23E-07	3.56E-07	6.663437159
PCARE	-0.258908973	0.6515984	-5.439422504	1.25E-07	3.61E-07	6.651188384
PLXNA3	0.500890265	4.06370065	5.438074999	1.26E-07	3.63E-07	6.644622726
CCNH	-0.30538449	4.51273549	-5.43805113	1.26E-07	3.63E-07	6.644506436
SEC14L3	-0.260617284	0.17602393	-5.437670997	1.26E-07	3.64E-07	6.642654492
TIAL1	-0.305707654	4.97131357	-5.437602085	1.26E-07	3.64E-07	6.642318776
METTL15	-0.3024918	3.03407838	-5.436405868	1.27E-07	3.66E-07	6.636491741
LRRIQ4	-0.072569259	0.09964173	-5.436265286	1.27E-07	3.66E-07	6.635807005
NCF4	0.69265707	3.12718701	5.435318772	1.27E-07	3.68E-07	6.631197138
DLX4	-0.35571031	0.67811795	-5.434572357	1.28E-07	3.69E-07	6.627562278
ORAI1	-0.365915221	5.23068967	-5.434382524	1.28E-07	3.69E-07	6.626637901
SF3B2	-0.338631244	6.48597411	-5.434305108	1.28E-07	3.70E-07	6.626260938
FAM111B	0.690118343	2.19208818	5.433965288	1.28E-07	3.70E-07	6.624606292
TMED8	-0.43945521	4.12813564	-5.432870061	1.29E-07	3.72E-07	6.619273989
NOC2L	-0.302431285	5.34138013	-5.432336998	1.29E-07	3.73E-07	6.616678988
AP2A2	-0.269543616	4.21872462	-5.431034313	1.30E-07	3.75E-07	6.610338243

FDX2	-0.380604074	3.36323	-5.430111563	1.31E-07	3.77E-07	6.605847538
ZNF248	-0.365908499	3.22605448	-5.429647016	1.31E-07	3.78E-07	6.60358698
MNDA	0.733590787	3.1817518	5.428994564	1.31E-07	3.79E-07	6.600412304
NEK9	-0.371135744	4.62030009	-5.427233441	1.33E-07	3.82E-07	6.591844605
TTC26	-0.352763534	2.82426771	-5.426778113	1.33E-07	3.83E-07	6.589629833
PSRC1	-0.316681849	2.0081828	-5.426611801	1.33E-07	3.83E-07	6.588820911
FOSL2	0.651392471	5.13555903	5.425698576	1.34E-07	3.85E-07	6.584379426
ANXA7	-0.351782573	7.51188968	-5.424836017	1.34E-07	3.87E-07	6.580184902
NUFIP1	-0.283987387	2.9896145	-5.423739968	1.35E-07	3.89E-07	6.574855707
MED27	-0.323258868	4.74845989	-5.421267163	1.37E-07	3.94E-07	6.562835605
ELFN1	-0.391308871	1.64300991	-5.420112302	1.37E-07	3.96E-07	6.557223411
UIMC1	-0.3132862	3.91062885	-5.419556397	1.38E-07	3.97E-07	6.554522256
RBM27	-0.286648707	2.84032015	-5.417211563	1.39E-07	4.01E-07	6.543131079
MSR1	0.655355642	2.37059752	5.416201461	1.40E-07	4.03E-07	6.538225223
ACOT4	-0.355630033	2.21786904	-5.415839791	1.40E-07	4.04E-07	6.536468843
CHMP1B	-0.504923669	6.6319013	-5.41577056	1.40E-07	4.04E-07	6.536132647
TCEAL5	-0.659109892	0.86437299	-5.415357687	1.41E-07	4.05E-07	6.534127744
CFAP161	-0.385460447	0.56730821	-5.41480248	1.41E-07	4.06E-07	6.53143186
CLEC7A	0.59670968	2.00593105	5.41412062	1.42E-07	4.07E-07	6.528121293
RNF113A	-0.266707085	4.99041187	-5.413947949	1.42E-07	4.08E-07	6.527282996
DEDD	-0.270595736	5.09495348	-5.412480263	1.43E-07	4.11E-07	6.520158394
SMCO2	0.127741858	0.16104134	5.411266356	1.44E-07	4.13E-07	6.514266872
CBY3	-0.277438221	0.78029933	-5.411080201	1.44E-07	4.13E-07	6.513363485
HTRA1	-0.548654705	6.97505166	-5.410464511	1.44E-07	4.15E-07	6.510375807
HAPLN2	-0.266357356	0.3738591	-5.408684728	1.46E-07	4.18E-07	6.501740805
ZNF749	-0.316574644	1.93599565	-5.407151022	1.47E-07	4.21E-07	6.494301505
ANTXRL	-0.105630136	0.12387685	-5.406311952	1.47E-07	4.23E-07	6.490232272
B3GNT6	0.155412895	0.13061745	5.405788316	1.48E-07	4.24E-07	6.487693052
IFI27	-0.610759068	5.02990777	-5.40558801	1.48E-07	4.24E-07	6.48672178
MAP3K6	0.407366622	4.82160845	5.403842742	1.49E-07	4.28E-07	6.478260271
TEKT2	-0.62402862	2.14605768	-5.403271381	1.50E-07	4.29E-07	6.47549064
MUTYH	-0.341968105	3.65176917	-5.40305889	1.50E-07	4.30E-07	6.47446066
MILR1	0.692932868	2.69590201	5.401998292	1.51E-07	4.32E-07	6.469320263
FAM98B	-0.349432942	4.04294904	-5.401307654	1.51E-07	4.33E-07	6.465973381
RNF180	-0.441929714	3.56328613	-5.401027756	1.51E-07	4.34E-07	6.464617073
RAB30	-0.458014078	2.71915245	-5.399746707	1.52E-07	4.36E-07	6.458410168
KIAA125	-0.153234048	0.35079551	-5.39703346	1.54E-07	4.42E-07	6.445267887
SYF2	-0.319335654	6.33721477	-5.396662031	1.55E-07	4.43E-07	6.443469189
RASGRF	0.561706576	1.16701714	5.395290421	1.56E-07	4.46E-07	6.436827818
PEBP4	-1.081090551	6.02702355	-5.394194609	1.57E-07	4.48E-07	6.431522831

SFXN3	0.422487924	4.81777558	5.394057936	1.57E-07	4.49E-07	6.430861236
CXorf56	-0.367480991	4.95420071	-5.393095321	1.57E-07	4.51E-07	6.426201879
LAMP5	1.297402122	2.97345419	5.391352504	1.59E-07	4.55E-07	6.41776778
HNRNPU	-0.289521677	5.57673894	-5.390188452	1.60E-07	4.57E-07	6.412135738
SMU1	-0.312086092	5.06289918	-5.389852608	1.60E-07	4.58E-07	6.410510997
SMPD4	-0.367404673	4.61419238	-5.388765515	1.61E-07	4.60E-07	6.405252439
TBX6	-0.358820159	1.55626548	-5.388190673	1.61E-07	4.62E-07	6.402472118
SPRR1B	0.676614756	0.64046033	5.387292289	1.62E-07	4.64E-07	6.398127398
RAB11FI	0.866934737	3.74404476	5.387064685	1.62E-07	4.64E-07	6.397026762
ABCB10	-0.399935664	3.29818896	-5.386709553	1.62E-07	4.65E-07	6.395309507
FRMPD2	-0.436878017	0.84012862	-5.386561626	1.63E-07	4.65E-07	6.394594226
UBTD2	-0.361383797	4.54521276	-5.38635097	1.63E-07	4.65E-07	6.393575656
PPP1R2	-0.368216338	5.67539309	-5.385310798	1.64E-07	4.68E-07	6.388546648
TADA1	-0.337805473	3.53276906	-5.385106015	1.64E-07	4.68E-07	6.387556661
APOL4	0.639860715	1.96354475	5.384853781	1.64E-07	4.69E-07	6.386337317
PFDN4	-0.271992259	4.23916152	-5.384773476	1.64E-07	4.69E-07	6.385949117
AK4	0.467220344	2.12140875	5.38471665	1.64E-07	4.69E-07	6.385674422
MPZ	-0.345782071	1.59950741	-5.384599078	1.64E-07	4.69E-07	6.385106082
FFAR4	0.3128287	0.64202084	5.384369332	1.64E-07	4.70E-07	6.383995535
KRI1	-0.327931106	4.7142359	-5.384112499	1.65E-07	4.70E-07	6.382754091
PGAM5	-0.304022323	4.58180107	-5.383666138	1.65E-07	4.71E-07	6.38059665
FGD5	-0.464737087	3.31267495	-5.382834816	1.66E-07	4.73E-07	6.376578925
CTR9	-0.420074557	5.20877453	-5.381183668	1.67E-07	4.77E-07	6.36860049
NOX4	0.506266753	1.84677863	5.380604597	1.68E-07	4.78E-07	6.365802846
ZCCHC7	-0.311882639	4.08143579	-5.380036908	1.68E-07	4.79E-07	6.363060427
GIPC2	-0.27914002	0.97768382	-5.379046713	1.69E-07	4.82E-07	6.358277502
CD68	0.42757885	0.90344266	5.378879597	1.69E-07	4.82E-07	6.357470351
FAM207A	-0.355670687	4.14396402	-5.378814155	1.69E-07	4.82E-07	6.357154278
GGACT	-0.308146407	2.3431782	-5.377475538	1.70E-07	4.85E-07	6.35068971
CFAP45	-0.346438427	0.85565213	-5.377062854	1.70E-07	4.86E-07	6.348696995
TRABD	-0.383723045	5.07063709	-5.37699342	1.71E-07	4.86E-07	6.348361732
TRIM39-I	-0.311055911	2.96920574	-5.376486295	1.71E-07	4.87E-07	6.34591319
RHBDD2	-0.371477585	8.36734438	-5.375160881	1.72E-07	4.90E-07	6.339514577
SLC6A3	-0.294305627	0.35518919	-5.373610303	1.73E-07	4.94E-07	6.332030555
SLC25A4	-0.365403737	3.88884783	-5.372513928	1.74E-07	4.97E-07	6.326739825
TOP1MT	-0.320509806	4.11084883	-5.371881279	1.75E-07	4.98E-07	6.32368727
GPRASP	-0.493140618	4.54353129	-5.371216978	1.76E-07	5.00E-07	6.320482302
ARMCX5	-0.493140618	4.54353129	-5.371216978	1.76E-07	5.00E-07	6.320482302
TOX4	-0.334735741	5.07063061	-5.37042807	1.76E-07	5.02E-07	6.316676569
P4HA1	-0.440988194	5.19789721	-5.369873761	1.77E-07	5.03E-07	6.314002817

SLC9A2	-0.462566422	1.29125361	-5.369681923	1.77E-07	5.03E-07	6.313077522
DTNBP1	-0.322259088	4.66328269	-5.369206813	1.77E-07	5.04E-07	6.310786037
TMEM18	-0.417513638	4.86753671	-5.369134605	1.77E-07	5.05E-07	6.310437784
AXL	0.578299793	4.56589693	5.367653665	1.79E-07	5.08E-07	6.303296223
FAM126E	-0.324463286	2.54300639	-5.367576521	1.79E-07	5.08E-07	6.302924254
PPOX	-0.313019423	3.51354089	-5.366579701	1.80E-07	5.11E-07	6.298118216
RASL10E	-0.476146229	1.17482707	-5.366287984	1.80E-07	5.11E-07	6.296711873
PPP1R14	-0.595266091	2.70829326	-5.366202537	1.80E-07	5.12E-07	6.296299949
MAPK12	-0.329625597	1.30149325	-5.365927235	1.80E-07	5.12E-07	6.294972819
SERGEF	-0.656863019	5.6078403	-5.365871527	1.80E-07	5.12E-07	6.294704274
LAS1L	-0.247355393	4.67878668	-5.365122775	1.81E-07	5.14E-07	6.29109511
PCSK4	-0.431003894	2.0426137	-5.364844217	1.81E-07	5.15E-07	6.289752495
ZNF530	-0.199566718	1.31983283	-5.364498897	1.82E-07	5.15E-07	6.288088172
TTC9C	-0.269843821	4.33445278	-5.364477094	1.82E-07	5.15E-07	6.287983094
ZNF584	-0.278276439	3.15487875	-5.36417466	1.82E-07	5.16E-07	6.286525543
TTLL9	-0.201037746	0.4712154	-5.363583611	1.82E-07	5.18E-07	6.283677227
CLIC2	-0.449957025	5.81808626	-5.363422515	1.82E-07	5.18E-07	6.282900938
ABR	0.425008886	4.79838208	5.362527972	1.83E-07	5.20E-07	6.278590638
CNTN2	-0.127162264	0.13690848	-5.362204291	1.84E-07	5.21E-07	6.277031146
ESM1	-0.995194525	3.84477688	-5.361944276	1.84E-07	5.21E-07	6.275778447
SMAD2	-0.312473223	3.43176264	-5.361175836	1.85E-07	5.23E-07	6.272076545
ALG9	-0.277270825	2.11282099	-5.360319115	1.85E-07	5.26E-07	6.267949857
JAKMIP3	-0.232974145	0.3225542	-5.356978218	1.88E-07	5.34E-07	6.251862316
TREH	-0.474182913	0.69234587	-5.356572345	1.89E-07	5.35E-07	6.249908447
TMEM87	-0.526197802	6.28229573	-5.356491131	1.89E-07	5.35E-07	6.249517499
ETF1	-0.384027791	6.07474257	-5.356086746	1.89E-07	5.36E-07	6.247570931
ABCA12	0.177570529	0.26540059	5.354762326	1.91E-07	5.40E-07	6.241196469
WFIKKN	-0.242380971	0.44737469	-5.354523205	1.91E-07	5.40E-07	6.240045704
TRAF4	-0.364535005	5.23087815	-5.353946529	1.91E-07	5.42E-07	6.237270643
RPP21	-0.332609584	3.86233057	-5.353862665	1.91E-07	5.42E-07	6.236867094
SLC9A5	0.426375791	1.41787206	5.351983437	1.93E-07	5.47E-07	6.227825695
RETN	0.844594969	1.24045169	5.351827208	1.93E-07	5.47E-07	6.227074155
HSPA8	-0.650714453	8.66945042	-5.351711057	1.93E-07	5.48E-07	6.226515421
ANGPT4	-0.49395216	0.99984138	-5.351459588	1.94E-07	5.48E-07	6.225305786
NFYB	-0.311673187	4.15374381	-5.350747106	1.94E-07	5.50E-07	6.221878804
WTIP	0.575743046	2.80061718	5.350738565	1.94E-07	5.50E-07	6.221837725
PRSS8	-0.488803798	7.13591915	-5.349564492	1.96E-07	5.53E-07	6.21619132
TMEM39	-0.309437973	4.57128872	-5.348555869	1.96E-07	5.56E-07	6.211341397
WDR12	-0.296609875	2.81760836	-5.347881104	1.97E-07	5.57E-07	6.208097226
GTF2B	-0.320461107	5.33884574	-5.347836132	1.97E-07	5.58E-07	6.207881017

DLGAP1	-0.39811955	0.64905145	-5.347179635	1.98E-07	5.59E-07	6.204725008
DIP2C	-0.464965813	2.94647967	-5.345325367	2.00E-07	5.64E-07	6.19581257
LAPTM4I	-0.48322021	6.0474733	-5.344541587	2.00E-07	5.66E-07	6.192046116
SLC7A6(	-0.27920902	1.87423046	-5.343115055	2.02E-07	5.70E-07	6.185192051
UBA52	-0.326368553	8.12916489	-5.342983968	2.02E-07	5.71E-07	6.184562294
RFC2	-0.273725518	4.80536812	-5.342723023	2.02E-07	5.71E-07	6.183308713
SCGB2B	-0.232879878	0.86673201	-5.342681449	2.02E-07	5.71E-07	6.183108992
PPL	0.867196227	6.50538612	5.342466166	2.03E-07	5.72E-07	6.182074817
PNMA6A	-0.663823219	2.34599193	-5.341589903	2.03E-07	5.74E-07	6.177865762
NCMAP	-0.691779161	3.94195809	-5.340641085	2.04E-07	5.77E-07	6.17330882
ZNF706	-0.247233727	4.66308786	-5.339917213	2.05E-07	5.79E-07	6.169832674
SERPINI	-0.522126911	0.44503888	-5.339340976	2.06E-07	5.80E-07	6.167065766
TPD52L2	-0.260448381	6.07468436	-5.339232376	2.06E-07	5.81E-07	6.166544327
STXBP1	-0.442676501	4.67969794	-5.338996825	2.06E-07	5.81E-07	6.165413371
CIPC	-0.459498727	4.54123894	-5.337527885	2.08E-07	5.85E-07	6.158361421
PLEKHA	0.957929179	5.80512496	5.336847396	2.08E-07	5.87E-07	6.15509512
NUAK1	-0.436235137	2.48025258	-5.336808091	2.08E-07	5.87E-07	6.154906465
NCKAP1	0.639452887	2.11789861	5.334865855	2.10E-07	5.93E-07	6.145585744
SAMD14	-0.211634736	0.71368414	-5.334479283	2.11E-07	5.94E-07	6.143730926
TRIM28	-0.324395431	7.62806942	-5.333639029	2.12E-07	5.96E-07	6.139699647
PPP1R2	-0.510878856	5.50689835	-5.333417037	2.12E-07	5.97E-07	6.138634686
SIX5	-0.486640848	3.88399033	-5.333147664	2.12E-07	5.97E-07	6.137342469
ZNF595	-0.295161865	2.73026307	-5.333125794	2.12E-07	5.97E-07	6.137237555
ZNF382	-0.2327366	1.36336696	-5.33264302	2.13E-07	5.99E-07	6.134921765
ZNF546	-0.295484219	1.56105499	-5.332349518	2.13E-07	6.00E-07	6.133513964
CD52	1.043105357	5.04322861	5.331726568	2.14E-07	6.01E-07	6.130526154
AKR1C2	-0.708338781	0.75872695	-5.331299291	2.14E-07	6.02E-07	6.128476995
MAT2A	-0.638874113	6.78032717	-5.331228313	2.14E-07	6.03E-07	6.128136607
YARS	-0.344107314	5.02474274	-5.331170607	2.14E-07	6.03E-07	6.127859871
NBL1	0.867864837	4.34545099	5.331150117	2.14E-07	6.03E-07	6.127761609
MICOS1(	0.867864837	4.34545099	5.331150117	2.14E-07	6.03E-07	6.127761609
DNAJC2	-0.277377179	3.75714296	-5.331066355	2.14E-07	6.03E-07	6.127359923
RECK	-0.34686461	2.52850241	-5.331030648	2.14E-07	6.03E-07	6.127188687
TRMT11	-0.333119354	8.14255682	-5.330960292	2.14E-07	6.03E-07	6.126851297
PDCD7	-0.32884406	4.67853311	-5.330611838	2.15E-07	6.04E-07	6.125180343
TVP23C-	-0.147072249	0.37432292	-5.329824688	2.16E-07	6.06E-07	6.121406018
LPAR5	1.128751993	5.04886562	5.329121669	2.16E-07	6.08E-07	6.118035473
DDX20	-0.312324623	3.12101821	-5.328993462	2.17E-07	6.08E-07	6.117420835
FAU	-0.358106735	9.42380721	-5.328824902	2.17E-07	6.09E-07	6.11661276
HINFP	-0.259237009	2.81111298	-5.328287134	2.17E-07	6.10E-07	6.114034846

ZNF790	-0.299154929	2.58275847	-5.32760473	2.18E-07	6.12E-07	6.110763885
HEXA	-0.291081512	4.88210552	-5.326833026	2.19E-07	6.14E-07	6.10706529
PRPF3	-0.350170046	4.40811719	-5.324806676	2.21E-07	6.21E-07	6.097355501
PSEN2	-0.28765807	4.07544778	-5.32415556	2.22E-07	6.22E-07	6.094236138
CHURC1	-0.428666952	4.6777735	-5.324046035	2.22E-07	6.23E-07	6.093711459
EIF5B	-0.326963638	5.78267772	-5.32302013	2.23E-07	6.26E-07	6.088797255
FBL	-0.335688482	7.17865063	-5.32262434	2.23E-07	6.27E-07	6.086901578
MAFA	-0.526584803	0.60363842	-5.322391695	2.24E-07	6.27E-07	6.085787353
HLA-DRA	1.021577015	9.77849659	5.321923243	2.24E-07	6.29E-07	6.083543877
NEK10	-0.087909375	0.17025419	-5.321503799	2.25E-07	6.30E-07	6.08153524
RPS16	-0.33018831	9.39267269	-5.319546258	2.27E-07	6.36E-07	6.072162632
ZBED1	-0.351523111	5.06251034	-5.316532666	2.30E-07	6.46E-07	6.057739101
BTRC	-0.354356049	3.80944276	-5.315966597	2.31E-07	6.47E-07	6.055030533
DUSP1	-0.8785133	9.12752736	-5.315395918	2.32E-07	6.49E-07	6.052300142
DNAJC1(	-0.372364856	3.96670409	-5.315179125	2.32E-07	6.50E-07	6.051262964
KIF16B	-0.445481418	3.40966223	-5.314738025	2.32E-07	6.51E-07	6.04915277
HOMER3	-0.380121975	5.23737751	-5.313682982	2.34E-07	6.54E-07	6.044106076
HYDIN	-0.219681349	0.52749402	-5.313424919	2.34E-07	6.55E-07	6.042871778
HHIPL2	-0.786876546	0.86531774	-5.312688135	2.35E-07	6.57E-07	6.039348055
RUSC1	-0.323510787	3.70332269	-5.312406482	2.35E-07	6.58E-07	6.038001133
GLB1	-0.444461715	6.28750954	-5.312310876	2.35E-07	6.58E-07	6.037543941
RRN3	-0.344000839	4.99848822	-5.3123081	2.35E-07	6.58E-07	6.037530665
CCDC92	-0.269032829	4.71210721	-5.312256406	2.35E-07	6.58E-07	6.037283466
TTC39C	0.395926573	1.4951687	5.311629031	2.36E-07	6.60E-07	6.034283505
GYS2	-0.120651898	0.14907866	-5.311195192	2.36E-07	6.61E-07	6.032209152
SEC22B	-0.34808401	3.89439416	-5.30929532	2.39E-07	6.67E-07	6.02312673
PGM2	-0.425032754	4.30144461	-5.308862379	2.39E-07	6.69E-07	6.021057401
CPXM1	-1.274028955	3.89618108	-5.308168953	2.40E-07	6.71E-07	6.017743313
C1orf189	-0.245709934	0.406007	-5.307088524	2.41E-07	6.74E-07	6.012580313
TMEM10	1.128103141	2.71939401	5.306370528	2.42E-07	6.77E-07	6.009149721
DISC1	-0.252895772	1.30450738	-5.306325985	2.42E-07	6.77E-07	6.008936909
CPSF3	-0.285561349	4.97633075	-5.305076239	2.44E-07	6.81E-07	6.00296654
HLA-DPA	1.025951123	6.46431722	5.304912908	2.44E-07	6.81E-07	6.002186345
ZNF570	-0.299601939	2.4029555	-5.304646734	2.44E-07	6.82E-07	6.000914939
MXRA5	0.760446658	5.15879735	5.304265757	2.45E-07	6.83E-07	5.999095251
DYDC2	-0.141776218	0.23250807	-5.30143477	2.48E-07	6.93E-07	5.985576696
KRIT1	-0.441327195	4.16262834	-5.301234801	2.48E-07	6.93E-07	5.984622022
TACR3	-0.067902725	0.05087253	-5.300666496	2.49E-07	6.95E-07	5.981909025
C16orf87	-0.247193429	1.86050621	-5.300531117	2.49E-07	6.96E-07	5.981262779
SEMA7A	0.528048064	1.82285436	5.300392502	2.49E-07	6.96E-07	5.980601103

HIST4H4	-0.361380295	1.30874618	-5.299779331	2.50E-07	6.98E-07	5.977674308
DENND2	-0.461549732	1.52884267	-5.298881638	2.51E-07	7.01E-07	5.973389914
WRAP73	-0.241093541	3.12568395	-5.298407678	2.52E-07	7.02E-07	5.971128095
RPL23A	-0.337995154	8.64980093	-5.297837388	2.53E-07	7.04E-07	5.968406785
GLTP	-0.275487504	6.60751105	-5.29766807	2.53E-07	7.05E-07	5.967598882
SMS	-0.337907727	6.43509211	-5.296964369	2.54E-07	7.07E-07	5.964241372
SLC25A2	-0.61469419	3.84673659	-5.296891618	2.54E-07	7.07E-07	5.963894283
FAM13C	-0.377174959	1.12981005	-5.294574263	2.57E-07	7.15E-07	5.95284033
ZNF268	-0.306356801	2.84116264	-5.294334494	2.57E-07	7.16E-07	5.951696836
ERICH4	-0.106049829	0.15902628	-5.293306064	2.58E-07	7.19E-07	5.946792572
OSBP	-0.403802263	5.64251459	-5.291545343	2.61E-07	7.26E-07	5.938398013
BRCC3	-0.367919183	4.49835062	-5.29097622	2.61E-07	7.28E-07	5.935685092
PITRM1	-0.305925217	4.69979025	-5.290705504	2.62E-07	7.28E-07	5.934394718
LIPC	-0.403476942	0.62321995	-5.289986182	2.63E-07	7.31E-07	5.930966298
ACBD7	0.845110433	2.42689966	5.289604078	2.63E-07	7.32E-07	5.929145275
JPH2	-0.396418638	0.99714098	-5.289510638	2.63E-07	7.32E-07	5.928699977
PSG8	0.804148191	0.76053877	5.289385148	2.63E-07	7.33E-07	5.928101947
NSUN2	-0.297624842	4.74516238	-5.289254224	2.63E-07	7.33E-07	5.927478038
GRK4	-0.331500484	2.57677889	-5.289076948	2.64E-07	7.34E-07	5.926633261
ARHGEF	-0.516285708	4.85211921	-5.288333707	2.65E-07	7.36E-07	5.923091723
IFIT2	0.605614983	3.78278741	5.288187775	2.65E-07	7.36E-07	5.922396405
TNS1	-0.516222303	5.35851577	-5.287924163	2.65E-07	7.37E-07	5.921140417
EPHA4	0.883537536	4.86587328	5.287470569	2.66E-07	7.39E-07	5.918979373
SLC35F6	-0.338474353	5.893059	-5.286603748	2.67E-07	7.42E-07	5.914850025
COL7A1	0.651935412	1.29959368	5.28616626	2.68E-07	7.43E-07	5.912766127
SLC35A2	-0.363338289	4.85653348	-5.285595386	2.68E-07	7.45E-07	5.910047085
EVI5L	-0.289700882	3.81705807	-5.284094554	2.70E-07	7.51E-07	5.902899815
SPRYD3	-0.412015294	6.76764948	-5.283932083	2.70E-07	7.51E-07	5.902126194
CELF1	-0.31247269	4.95325477	-5.281552314	2.74E-07	7.60E-07	5.89079687
KLHDC1	-0.433386897	2.76061913	-5.281544788	2.74E-07	7.60E-07	5.890761047
INO80C	-0.316244442	3.3149741	-5.279173452	2.77E-07	7.69E-07	5.879475954
DNAJC1(	-0.324780915	3.32025787	-5.278805927	2.77E-07	7.70E-07	5.877727283
PIP	-0.560268433	0.56938731	-5.278418629	2.78E-07	7.71E-07	5.875884641
BIN3	-0.318719742	3.33076036	-5.278344706	2.78E-07	7.71E-07	5.87553295
NAA30	-0.366598398	3.48735046	-5.278023451	2.78E-07	7.73E-07	5.874004616
CYP4V2	-0.380599505	3.93669012	-5.276986321	2.80E-07	7.76E-07	5.869071098
LRP6	-0.431403835	3.54824413	-5.276855065	2.80E-07	7.77E-07	5.868446783
SARNP	-0.228532957	1.84863925	-5.276110775	2.81E-07	7.79E-07	5.864906828
PLEKHH	-0.660722918	4.6513764	-5.276046315	2.81E-07	7.80E-07	5.864600262
CTSZ	-0.398287569	8.02850181	-5.275914841	2.81E-07	7.80E-07	5.863974998

CNPY3	-0.335805364	7.20490639	-5.27564019	2.82E-07	7.81E-07	5.86266886
SMAD9	-0.767673386	4.14919082	-5.275517971	2.82E-07	7.81E-07	5.862087648
DMRTC1	-0.082798707	0.12077001	-5.275335135	2.82E-07	7.82E-07	5.86121819
B4GALN'	0.70531674	3.68762967	5.273016877	2.85E-07	7.91E-07	5.85019607
TPD52	-0.358446293	5.3003201	-5.272967452	2.85E-07	7.91E-07	5.849961125
TBC1D8	-0.3639126	2.49258838	-5.271191649	2.88E-07	7.97E-07	5.841520792
THUMPD	-0.387767307	5.12454394	-5.271126035	2.88E-07	7.98E-07	5.841208974
PPARG	-0.634300613	2.57920757	-5.270792352	2.89E-07	7.99E-07	5.839623262
TSC2	-0.345161598	4.92645153	-5.26994568	2.90E-07	8.02E-07	5.835600109
RPL34	-0.388842182	8.85471841	-5.269918145	2.90E-07	8.02E-07	5.835469282
RAB6A	-0.386010859	6.38275292	-5.269908839	2.90E-07	8.02E-07	5.835425066
CXorf65	0.264772012	0.36880183	5.269296546	2.91E-07	8.04E-07	5.832515954
ADSL	-0.307442303	4.58736745	-5.26870983	2.92E-07	8.06E-07	5.829728623
ADCY4	-0.435565702	2.53352967	-5.268703074	2.92E-07	8.06E-07	5.829696527
MCMDC2	-0.144639364	0.61902382	-5.268084561	2.92E-07	8.09E-07	5.826758408
MBD1	-0.310035569	4.74493499	-5.2679981	2.93E-07	8.09E-07	5.826347714
OMA1	-0.319953281	4.23650824	-5.265939744	2.96E-07	8.17E-07	5.816572044
HNRNPK	-0.35280639	8.24566784	-5.265199773	2.97E-07	8.20E-07	5.813058479
CLDN9	1.040113018	3.64020602	5.264814282	2.97E-07	8.21E-07	5.811228226
CDC16	-0.262679928	5.79447033	-5.261759398	3.02E-07	8.33E-07	5.796727937
FRMPD1	-0.132370819	0.19923603	-5.26169629	3.02E-07	8.33E-07	5.796428461
KAT14	-0.322006687	4.12986157	-5.261516436	3.02E-07	8.34E-07	5.795574986
CENPJ	-0.601541486	2.5467732	-5.260783402	3.03E-07	8.37E-07	5.792096708
NLRX1	-0.329363967	4.26618468	-5.260112025	3.04E-07	8.40E-07	5.788911335
ZGPAT	-0.310421808	2.70219476	-5.260012126	3.04E-07	8.40E-07	5.788437392
MUC20	0.660398912	2.91676012	5.259582586	3.05E-07	8.42E-07	5.786399625
SIRT3	-0.341913994	4.24595975	-5.259250722	3.05E-07	8.43E-07	5.784825333
NXPH3	-0.351978572	1.27519474	-5.258771119	3.06E-07	8.45E-07	5.782550339
SNX20	0.57928306	1.37682784	5.258681839	3.06E-07	8.45E-07	5.782126859
IP6K2	-0.392469611	5.53328615	-5.258466123	3.07E-07	8.46E-07	5.781103683
BLZF1	-0.356871083	4.23039771	-5.258349756	3.07E-07	8.46E-07	5.780551745
TRIM26	-0.312325215	5.50999132	-5.258117588	3.07E-07	8.47E-07	5.779450589
AKAP17A	-0.396784234	5.23516364	-5.258006909	3.07E-07	8.47E-07	5.778925659
PAFAH1I	-0.391348885	5.73940218	-5.257811615	3.08E-07	8.48E-07	5.77799944
REEP4	-0.304478741	4.83385788	-5.257213745	3.08E-07	8.50E-07	5.775164101
MMP9	1.157344754	4.2928653	5.256884973	3.09E-07	8.51E-07	5.773605041
ERBB4	-0.563820164	1.21382015	-5.256181789	3.10E-07	8.54E-07	5.770270757
ERCC3	-0.309478746	4.20880823	-5.256090682	3.10E-07	8.54E-07	5.769838783
MIA3	-0.39134509	4.41780954	-5.255945417	3.10E-07	8.55E-07	5.769150034
SCCPDH	-0.353967542	5.98444301	-5.254023387	3.13E-07	8.63E-07	5.760038515

PTER	-0.421609968	3.96852532	-5.254011691	3.13E-07	8.63E-07	5.759983079
RNF157	-0.59046141	1.41184915	-5.252413666	3.16E-07	8.69E-07	5.752409583
C14orf11	-0.303203268	5.7686488	-5.251864978	3.17E-07	8.72E-07	5.749809619
EFHC1	-0.453520658	3.50419036	-5.251789527	3.17E-07	8.72E-07	5.749452112
DSN1	-0.284816841	4.00912758	-5.251699658	3.17E-07	8.72E-07	5.749026296
RASSF1	-0.306687617	4.00385332	-5.251590173	3.17E-07	8.72E-07	5.74850754
DUOXA1	-0.612432231	6.34353112	-5.25043724	3.19E-07	8.77E-07	5.7430453
SPAG5	-0.562284441	2.31363535	-5.25011901	3.19E-07	8.78E-07	5.741537798
TULP4	-0.324808771	3.55387552	-5.249581862	3.20E-07	8.80E-07	5.738993413
MYEOV	0.454908042	0.73511232	5.249267671	3.21E-07	8.82E-07	5.737505237
ZNF79	-0.327451009	3.05786718	-5.249023483	3.21E-07	8.83E-07	5.73634868
HCK	0.712372499	3.0839645	5.248713368	3.22E-07	8.84E-07	5.734879935
FN3K	-0.318695424	4.44061491	-5.247909864	3.23E-07	8.87E-07	5.731074761
NEPRO	-0.326839636	4.43307468	-5.2467255	3.25E-07	8.92E-07	5.72546679
ZNF408	-0.281807581	3.75820176	-5.246506312	3.25E-07	8.93E-07	5.72442905
SH3GL2	-0.818776611	1.06148909	-5.246501018	3.25E-07	8.93E-07	5.724403985
EPC1	-0.341228968	3.49840341	-5.246077132	3.26E-07	8.95E-07	5.722397201
GJC1	-0.528648267	2.31774187	-5.244813417	3.28E-07	9.00E-07	5.716415225
RP1	0.094171336	0.07777038	5.244630141	3.28E-07	9.01E-07	5.715547755
PLEKHB:	-0.464532208	6.0778414	-5.244337599	3.29E-07	9.02E-07	5.714163172
UTP15	-0.251930723	2.70613957	-5.244111733	3.29E-07	9.03E-07	5.7130942
SLC45A1	-0.394931992	1.79796778	-5.244006613	3.29E-07	9.03E-07	5.712596706
ZNF606	-0.324160935	3.04085087	-5.241067694	3.34E-07	9.16E-07	5.698691127
RPL36AL	-0.350445656	9.4684375	-5.237730758	3.39E-07	9.31E-07	5.682909925
PPP6R3	-0.338427582	5.29055444	-5.237293325	3.40E-07	9.33E-07	5.680841797
USP8	-0.359381242	3.19647297	-5.236377198	3.42E-07	9.37E-07	5.676510912
BRAP	-0.243551407	4.21059932	-5.235558267	3.43E-07	9.40E-07	5.672640028
TELO2	-0.335738497	4.70199496	-5.235075082	3.44E-07	9.42E-07	5.67035636
TMEM69	-0.276457993	5.20281512	-5.234638663	3.44E-07	9.44E-07	5.668293868
PPP1R3!	-0.427248267	5.43190767	-5.234220179	3.45E-07	9.46E-07	5.666316268
RNASEL	-0.374063323	3.64240462	-5.233249721	3.47E-07	9.50E-07	5.66173073
C2	0.670772761	1.95137819	5.232739796	3.48E-07	9.53E-07	5.659321544
SPNS2	-0.437689229	2.83798803	-5.232014285	3.49E-07	9.56E-07	5.655894125
PTPRO	0.319361458	0.74750902	5.231769091	3.49E-07	9.57E-07	5.654735882
SARS2	-0.305637331	2.93033306	-5.231111534	3.50E-07	9.60E-07	5.65162994
METTL9	-0.354955734	5.85798321	-5.230145436	3.52E-07	9.64E-07	5.647067186
RIOX1	-0.279315965	4.08022784	-5.230038504	3.52E-07	9.64E-07	5.646562205
RYR1	0.719470817	2.50598676	5.228694717	3.55E-07	9.71E-07	5.640216908
FAM214/	-0.443610614	4.05928638	-5.227685871	3.56E-07	9.75E-07	5.635454055
TM9SF3	-0.366847091	6.69462471	-5.22742706	3.57E-07	9.76E-07	5.634232302

SLC7A4	0.404226576	0.81826198	5.225873474	3.60E-07	9.84E-07	5.626899422
RASSF1(	-0.427637983	0.50018044	-5.225819269	3.60E-07	9.84E-07	5.62664361
TMEM19	-0.373322921	0.66060848	-5.225137143	3.61E-07	9.87E-07	5.623424574
ELOVL2	-0.457787737	1.25966544	-5.224867333	3.61E-07	9.88E-07	5.622151405
SCN1B	0.62671643	3.70524741	5.224656328	3.62E-07	9.89E-07	5.621155755
EEF1D	-0.355468063	5.51227557	-5.224475342	3.62E-07	9.90E-07	5.620301781
PDXDC1	-0.370601519	5.41529878	-5.224369925	3.62E-07	9.90E-07	5.61980439
SPRYD7	-0.279398387	3.96554299	-5.223402285	3.64E-07	9.94E-07	5.615239102
RPL10A	-0.424460151	10.284954	-5.223201218	3.64E-07	9.95E-07	5.614290563
CXCL1	0.585213945	1.02066912	5.221433874	3.67E-07	1.00E-06	5.605954328
USH1C	-0.394776167	0.39944862	-5.221087871	3.68E-07	1.01E-06	5.604322564
POLDIP3	-0.440307274	6.37360232	-5.220944704	3.68E-07	1.01E-06	5.603647407
RPS23	-0.329110803	8.62842377	-5.220038464	3.70E-07	1.01E-06	5.599374048
LAPTM4/	-0.437715623	9.12344802	-5.220002732	3.70E-07	1.01E-06	5.599205566
MEAK7	-0.246922065	2.66317717	-5.219064046	3.72E-07	1.01E-06	5.594779864
CRIP3	-0.295036342	0.68402699	-5.217479033	3.75E-07	1.02E-06	5.587308324
GATA5	-0.995832453	1.26118941	-5.216388995	3.77E-07	1.03E-06	5.582171091
PLPPR1	0.221183419	0.21753742	5.215793589	3.78E-07	1.03E-06	5.579365374
HAVCR2	0.649543241	2.48015597	5.214928842	3.79E-07	1.03E-06	5.575290908
LEAP2	-0.304475404	1.08491943	-5.21422359	3.81E-07	1.04E-06	5.571968347
SLC12A9	-0.306784065	3.74121948	-5.213579238	3.82E-07	1.04E-06	5.568933008
FHL5	-0.632858162	1.98620707	-5.213043993	3.83E-07	1.04E-06	5.566411873
ZNF44	-0.317669667	3.13028207	-5.212496005	3.84E-07	1.05E-06	5.563830929
TRAPPC	-0.377429808	4.27999303	-5.212146265	3.84E-07	1.05E-06	5.562183817
MAP3K1:	-0.27259246	3.62010121	-5.211818089	3.85E-07	1.05E-06	5.560638344
FPR3	0.768744828	2.81011752	5.211622718	3.85E-07	1.05E-06	5.559718328
SDC3	0.542437124	5.54992961	5.21158196	3.86E-07	1.05E-06	5.559526398
AMD1	-0.374655473	4.89631294	-5.211579313	3.86E-07	1.05E-06	5.55951393
CXCL3	0.416901977	0.80284163	5.211066345	3.87E-07	1.05E-06	5.557098467
CASTOR	0.474060828	1.83428022	5.210776518	3.87E-07	1.05E-06	5.555733818
TVP23C	-0.246077895	1.63233001	-5.208899969	3.91E-07	1.06E-06	5.54689956
AKNA	0.514214747	3.68138071	5.208595243	3.91E-07	1.06E-06	5.545465239
FSCN2	-0.437992141	3.51736563	-5.207763437	3.93E-07	1.07E-06	5.541550336
SEMA6D	-0.698927892	2.19598139	-5.207652266	3.93E-07	1.07E-06	5.541027149
NRBF2	-0.3183542	4.71660825	-5.207378608	3.94E-07	1.07E-06	5.53973931
PEX6	-0.38988407	5.54507846	-5.207131825	3.94E-07	1.07E-06	5.538577989
PTMA	-0.363453163	9.57994392	-5.206846558	3.95E-07	1.07E-06	5.537235631
CEP126	-0.403944367	2.022572	-5.206482708	3.95E-07	1.07E-06	5.535523571
EVI2B	0.819304219	3.27692805	5.206249557	3.96E-07	1.08E-06	5.534426549
KLHL12	-0.329691758	5.25835914	-5.206071293	3.96E-07	1.08E-06	5.533587813

TRIM52	-0.340426765	2.8467864	-5.20597768	3.96E-07	1.08E-06	5.533147373
ENDOU	-0.344221656	0.86476748	-5.205850755	3.96E-07	1.08E-06	5.53255021
LRP3	-0.393489769	4.30071417	-5.204566702	3.99E-07	1.08E-06	5.526509591
CFAP77	-0.187083205	0.22035849	-5.204386681	3.99E-07	1.08E-06	5.525662806
ZNF383	-0.285016551	2.29386386	-5.204010221	4.00E-07	1.09E-06	5.523892093
IL23A	0.424634784	2.0205268	5.203427221	4.01E-07	1.09E-06	5.521150097
KCTD3	-0.395466702	5.02335536	-5.203131981	4.02E-07	1.09E-06	5.519761606
DCHS2	0.297154516	0.53576287	5.20279137	4.02E-07	1.09E-06	5.518159816
MTX3	-0.357648126	3.49360323	-5.202579976	4.03E-07	1.09E-06	5.517165736
SLC38A6	-0.222166556	2.44620978	-5.201766039	4.04E-07	1.10E-06	5.513338507
CHRFAM	-0.243012846	0.47007196	-5.201757245	4.04E-07	1.10E-06	5.51329716
RRAS2	-0.361946264	4.43594567	-5.201229884	4.05E-07	1.10E-06	5.510817708
C1orf127	-0.187424156	0.45145039	-5.20111599	4.06E-07	1.10E-06	5.51028225
ZNF720	-0.312159485	2.71604454	-5.201044579	4.06E-07	1.10E-06	5.509946523
ZNF112	-0.361201235	2.57727506	-5.200923323	4.06E-07	1.10E-06	5.509376467
OR4D6	0.242742521	0.2758962	5.200123853	4.08E-07	1.11E-06	5.505618226
SYDE2	-0.402001048	1.67438572	-5.199894614	4.08E-07	1.11E-06	5.504540678
ZBTB46	-0.302225858	3.81402833	-5.198568035	4.11E-07	1.11E-06	5.498305797
RAB7A	-0.25555762	8.57216833	-5.197782291	4.12E-07	1.12E-06	5.49461343
TAX1BP1	-0.355470431	5.94703885	-5.197142714	4.14E-07	1.12E-06	5.491608265
UNC13B	-0.396869333	4.60752119	-5.195616799	4.17E-07	1.13E-06	5.484439689
CUL2	-0.310117436	4.17459772	-5.194923518	4.18E-07	1.13E-06	5.481183298
CFAP74	-0.1253131	0.2111769	-5.193562829	4.21E-07	1.14E-06	5.474793059
ATMIN	-0.328437698	5.08276347	-5.192640969	4.23E-07	1.14E-06	5.470464477
C1orf174	-0.288405556	3.8440319	-5.190616674	4.27E-07	1.16E-06	5.460961601
RAPGEF	-0.229944853	1.88678102	-5.188435583	4.31E-07	1.17E-06	5.45072601
SMKR1	-0.398544679	1.83407771	-5.187997357	4.32E-07	1.17E-06	5.448669892
SPDL1	-0.278408483	2.07799497	-5.187257284	4.34E-07	1.17E-06	5.44519785
CCDC7	-0.178204022	0.75330706	-5.18654196	4.35E-07	1.18E-06	5.441842297
ZNF639	-0.318255469	3.88418206	-5.186144447	4.36E-07	1.18E-06	5.439977745
ZNF497	-0.340860377	1.76138332	-5.186042931	4.37E-07	1.18E-06	5.439501597
RNASEK	-0.18532139	0.83816953	-5.185412695	4.38E-07	1.18E-06	5.436545733
AMN1	-0.327620406	2.39468895	-5.184980209	4.39E-07	1.19E-06	5.434517497
PADI1	0.141569118	0.11659193	5.181897173	4.45E-07	1.20E-06	5.42006293
CCDC154	-0.670544474	2.2791783	-5.181118697	4.47E-07	1.21E-06	5.416414206
SLC6A13	-0.600547094	2.3560934	-5.180817506	4.48E-07	1.21E-06	5.415002643
SMAD4	-0.332059754	4.27757446	-5.179765567	4.50E-07	1.22E-06	5.410073132
ZBED5	-0.326084956	4.05898537	-5.179559487	4.50E-07	1.22E-06	5.409107514
RAB3C	0.162387082	0.23772233	5.178898517	4.52E-07	1.22E-06	5.406010647
CTSO	-0.479338815	5.89946033	-5.178436121	4.53E-07	1.22E-06	5.403844355

EDDM13	-0.138968801	0.41453001	-5.177648891	4.55E-07	1.23E-06	5.400156601
ATP4A	-0.413627287	0.55848217	-5.177037629	4.56E-07	1.23E-06	5.397293476
ANP32B	-0.332627898	7.92465586	-5.176360498	4.58E-07	1.24E-06	5.39412214
ZNF616	-0.297244882	2.79081624	-5.176215685	4.58E-07	1.24E-06	5.393443957
NPNT	-0.585967886	8.14838464	-5.17565102	4.59E-07	1.24E-06	5.390799672
CPSF2	-0.312646735	3.75361055	-5.17507834	4.60E-07	1.24E-06	5.388118095
ADGRG3	-0.362245307	0.57866929	-5.174692326	4.61E-07	1.24E-06	5.386310716
PPP4R3A	-0.319637195	5.12966565	-5.172386261	4.66E-07	1.26E-06	5.375515635
TMEM19	-0.347319245	3.83262094	-5.170448499	4.71E-07	1.27E-06	5.366447658
IGSF3	0.647716788	4.4347634	5.170235538	4.71E-07	1.27E-06	5.365451249
IER5L	0.695652622	4.19798636	5.168551129	4.75E-07	1.28E-06	5.357571364
BRPF1	-0.327012848	4.13325796	-5.16816804	4.76E-07	1.28E-06	5.355779516
PTPRJ	-0.40253741	4.59587654	-5.168065767	4.76E-07	1.28E-06	5.355301166
BCL2A1	0.761607553	2.51702918	5.167382492	4.78E-07	1.29E-06	5.352105552
SLC30A5	-0.392130446	5.09407865	-5.167072946	4.79E-07	1.29E-06	5.350657949
VSIG1	0.334315383	0.67352716	5.166608779	4.80E-07	1.29E-06	5.34848738
SDR42E2	-0.111609597	0.26869729	-5.166376091	4.80E-07	1.29E-06	5.347399333
ZBTB40	-0.364631184	3.60588131	-5.165463863	4.82E-07	1.30E-06	5.343134134
DKC1	-0.325652692	5.40545371	-5.16490934	4.84E-07	1.30E-06	5.340541714
CFAP54	-0.174418149	0.39876872	-5.164737502	4.84E-07	1.30E-06	5.33973841
SPINDOC	-0.307682418	4.21022152	-5.164603593	4.84E-07	1.30E-06	5.339112428
COL8A1	0.728601353	6.21123109	5.164568573	4.84E-07	1.30E-06	5.338948725
C19orf81	-0.677611732	0.95834002	-5.163916209	4.86E-07	1.31E-06	5.335899349
TBRG4	-0.22717152	4.40451671	-5.163863387	4.86E-07	1.31E-06	5.335652454
UGGT1	-0.395201168	4.39512082	-5.16343719	4.87E-07	1.31E-06	5.33366044
PITPNB	-0.311562012	4.75703123	-5.163386904	4.87E-07	1.31E-06	5.33342542
ABCB1	-0.477278599	1.86021485	-5.163263754	4.88E-07	1.31E-06	5.332849856
RAB19	-0.549409163	2.05809385	-5.162914836	4.88E-07	1.31E-06	5.331219185
MYL5	-0.362544867	2.69267299	-5.161768416	4.91E-07	1.32E-06	5.325862013
BCL2L10	-0.561988132	1.69480581	-5.161615657	4.91E-07	1.32E-06	5.325148248
WAC	-0.348925154	5.12020276	-5.16134005	4.92E-07	1.32E-06	5.323860523
DNAAF5	-0.284627863	3.62480982	-5.160896681	4.93E-07	1.33E-06	5.321789076
PHF1	-0.300175957	6.70807484	-5.16074699	4.93E-07	1.33E-06	5.321089744
H2AFY2	-0.364591379	5.48520767	-5.160352661	4.94E-07	1.33E-06	5.31924758
CTXN1	0.967541898	4.76087724	5.159406124	4.97E-07	1.33E-06	5.314826164
CDK6	-0.445491859	2.16503628	-5.158411128	4.99E-07	1.34E-06	5.310179096
CS	-0.343010996	5.70235254	-5.157377787	5.02E-07	1.35E-06	5.305353712
ARNTL	0.85951909	4.31769515	5.156260759	5.04E-07	1.35E-06	5.300138418
PACRGL	-0.343191261	3.23657958	-5.156122297	5.05E-07	1.35E-06	5.299492017
TDRD6	-0.45680105	1.12144641	-5.154099164	5.10E-07	1.37E-06	5.290048742

SLC1A1	-0.853659149	4.3287749	-5.153847479	5.10E-07	1.37E-06	5.288874174
ACBD4	-0.351617846	4.55747609	-5.152966552	5.12E-07	1.37E-06	5.28476342
TPGS1	-0.539096029	1.90529877	-5.152916473	5.13E-07	1.38E-06	5.284529747
LAPTM5	0.724045023	6.20898146	5.152167231	5.14E-07	1.38E-06	5.281033963
ZMYND1	-0.258465421	4.1169378	-5.152108116	5.15E-07	1.38E-06	5.280758166
UBE2R2	-0.271692483	5.98479158	-5.151721525	5.15E-07	1.38E-06	5.278954599
PWWP3/	-0.357980404	3.52325043	-5.15140334	5.16E-07	1.38E-06	5.277470257
SNAP25	0.667581019	1.67582806	5.151348249	5.16E-07	1.38E-06	5.277213259
KLF9	-0.523992526	5.22471235	-5.150779322	5.18E-07	1.39E-06	5.274559401
NAPEPL	-0.404180724	3.70232812	-5.150701449	5.18E-07	1.39E-06	5.274196166
INSC	-0.334412196	0.46149522	-5.149961163	5.20E-07	1.39E-06	5.270743373
MCPH1	-0.278074592	2.78501084	-5.149942969	5.20E-07	1.39E-06	5.270658518
KCTD5	-0.33566523	4.48327514	-5.149294714	5.22E-07	1.40E-06	5.267635305
SERPINE	0.325598301	0.34185035	5.149180322	5.22E-07	1.40E-06	5.26710186
RNASEH	-0.391768343	4.4858884	-5.149166843	5.22E-07	1.40E-06	5.267039003
LENG9	0.504742461	2.45749386	5.148771338	5.23E-07	1.40E-06	5.26519471
HFE	-0.399296607	3.32961515	-5.147135059	5.27E-07	1.41E-06	5.257565742
MAPK11	-0.395948757	3.67693251	-5.145887847	5.30E-07	1.42E-06	5.251752076
GBE1	-0.374278604	4.01034081	-5.145878512	5.30E-07	1.42E-06	5.251708564
TENT5B	-0.594997545	2.75890263	-5.145610577	5.31E-07	1.42E-06	5.25045978
NT5E	0.911756946	5.57105735	5.143900533	5.35E-07	1.43E-06	5.24249091
TVP23B	-0.407742026	4.07400809	-5.142864589	5.38E-07	1.44E-06	5.23766442
GPC4	-0.497415063	4.46157502	-5.14285208	5.38E-07	1.44E-06	5.237606144
ESS2	-0.270812963	3.57475141	-5.142740701	5.38E-07	1.44E-06	5.237087274
SCAP	-0.315420421	5.62815593	-5.142052656	5.40E-07	1.44E-06	5.233882154
COQ2	-0.289415905	3.73565924	-5.141946782	5.40E-07	1.44E-06	5.23338899
DNAJA4	-0.453857757	3.37610734	-5.140801692	5.43E-07	1.45E-06	5.228055674
IGHMBP:	-0.27130109	2.99605321	-5.140478971	5.44E-07	1.45E-06	5.226552762
DGAT2L(	0.140895711	0.21691624	5.139700049	5.46E-07	1.46E-06	5.222925628
TAF15	-0.293108209	6.17588721	-5.139654769	5.46E-07	1.46E-06	5.222714788
ATP13A1	-0.289771097	5.06707407	-5.13943646	5.47E-07	1.46E-06	5.2216983
ATP6V1E	-0.302318756	6.76741931	-5.139420205	5.47E-07	1.46E-06	5.221622615
TESMIN	-0.283410703	1.13567023	-5.138394103	5.50E-07	1.47E-06	5.216845352
LDB3	-0.35202376	1.07357442	-5.138006541	5.51E-07	1.47E-06	5.215041171
ZNF547	-0.297201516	1.66727913	-5.136657529	5.54E-07	1.48E-06	5.208762092
SIGLEC9	0.410270536	1.35487708	5.135912861	5.56E-07	1.48E-06	5.205296549
RPS6KC	-0.309398584	3.13217321	-5.134993033	5.59E-07	1.49E-06	5.201016414
TCEA2	-0.340577616	5.10320497	-5.132919801	5.64E-07	1.51E-06	5.191371547
MAP7	-0.389790703	4.68947591	-5.132356916	5.66E-07	1.51E-06	5.1887535
CD164L2	0.787731985	2.22654541	5.130431224	5.71E-07	1.52E-06	5.179798641

CNBP	-0.274573557	9.08567915	-5.130304244	5.72E-07	1.52E-06	5.179208252
ANKRD4	-0.335344354	3.79594587	-5.129994936	5.72E-07	1.53E-06	5.177770192
EPHA5	-0.327137002	0.26342234	-5.129910979	5.73E-07	1.53E-06	5.177379865
LIF	0.702402776	3.14195771	5.127938995	5.78E-07	1.54E-06	5.168213322
COPG1	-0.283665176	7.2845813	-5.127788109	5.79E-07	1.54E-06	5.167512063
LYSMD3	-0.453578654	4.13224904	-5.127570973	5.79E-07	1.54E-06	5.166502932
MPV17	-0.290895519	5.42642585	-5.127062106	5.81E-07	1.55E-06	5.164138126
CHIC2	-0.362364181	4.61910099	-5.126691267	5.82E-07	1.55E-06	5.162414879
PEX13	-0.368163164	4.33692077	-5.125890571	5.84E-07	1.56E-06	5.158694492
ZNF626	-0.378525354	3.2710439	-5.124001786	5.89E-07	1.57E-06	5.149920229
FRMD4B	-0.645016744	4.2019587	-5.122877161	5.92E-07	1.58E-06	5.144697085
TUBA4B	-0.340132715	0.44090808	-5.122732799	5.93E-07	1.58E-06	5.144026687
EIF4G2	-0.427043959	8.08761799	-5.121234256	5.97E-07	1.59E-06	5.137068546
ARL4C	0.717261132	2.96943522	5.120637865	5.99E-07	1.59E-06	5.134299805
RCBTB1	-0.502524994	5.68093709	-5.119751412	6.01E-07	1.60E-06	5.130184933
CLCN2	-0.269274327	2.48742027	-5.119437718	6.02E-07	1.60E-06	5.128728922
FCER1A	0.957742525	2.58476978	5.119134925	6.03E-07	1.60E-06	5.127323576
SBSPON	-0.423968262	1.09421968	-5.118507576	6.05E-07	1.61E-06	5.124412088
B3GALN	-0.553805287	2.22237714	-5.117357236	6.08E-07	1.62E-06	5.119074186
MPHOSF	-0.325526991	1.68127381	-5.117086251	6.09E-07	1.62E-06	5.117816883
CMTM4	-0.565513675	6.0859004	-5.116476327	6.11E-07	1.62E-06	5.114987181
NADK	-0.354400688	4.70211725	-5.115359942	6.14E-07	1.63E-06	5.1098085
SNED1	-0.549901948	3.19317245	-5.115251137	6.15E-07	1.63E-06	5.109303825
FADD	-0.372687578	4.10040741	-5.114388308	6.17E-07	1.64E-06	5.105302035
BHMT2	-0.962168224	3.36394754	-5.113051402	6.21E-07	1.65E-06	5.099102573
VRK1	-0.265547475	3.37441094	-5.112103831	6.24E-07	1.66E-06	5.094709321
ASB1	-0.374608708	3.57418988	-5.111320341	6.26E-07	1.66E-06	5.091077307
NAMPT	0.555939432	4.20218704	5.111089116	6.27E-07	1.66E-06	5.090005502
IL7R	0.826500828	1.95177745	5.110900388	6.28E-07	1.67E-06	5.089130719
CLEC4D	0.154382636	0.20802638	5.110030089	6.30E-07	1.67E-06	5.085097085
TUB	-0.558884939	3.1193689	-5.110018204	6.30E-07	1.67E-06	5.085042006
A2M	-0.482037798	7.69087814	-5.10991703	6.30E-07	1.67E-06	5.084573121
PIM2	0.75938607	4.09909823	5.109233585	6.33E-07	1.68E-06	5.081405961
KCNAB2	0.414639743	2.50515843	5.109232793	6.33E-07	1.68E-06	5.081402291
PLEKHG	0.653394231	2.91079043	5.109075781	6.33E-07	1.68E-06	5.08067473
CCDC19	-0.193284414	0.60782025	-5.108984342	6.33E-07	1.68E-06	5.080251029
TMC4	-0.406424979	7.0462363	-5.108800244	6.34E-07	1.68E-06	5.079397987
NCBP1	-0.344280153	4.2109448	-5.10817086	6.36E-07	1.69E-06	5.07648186
CD101	0.331606907	0.96573269	5.108039542	6.36E-07	1.69E-06	5.07587346
KLHDC7I	0.852228817	2.34025452	5.107341967	6.38E-07	1.69E-06	5.07264179

MSX1	-0.413548191	2.1063535	-5.107209292	6.39E-07	1.69E-06	5.072027184
ADRA1D	-0.197445548	0.36790781	-5.107080338	6.39E-07	1.69E-06	5.071429826
SLMAP	-0.433057136	3.4066797	-5.10700474	6.39E-07	1.69E-06	5.071079639
KIAA158(	-0.303805202	2.78716297	-5.106829109	6.40E-07	1.70E-06	5.070266088
NGLY1	-0.289178085	3.46409609	-5.105924984	6.43E-07	1.70E-06	5.066078409
ZNF285	-0.259659655	1.60193613	-5.105711199	6.43E-07	1.70E-06	5.065088297
HCFC2	-0.381551528	3.02558509	-5.105552997	6.44E-07	1.70E-06	5.064355634
THBS4	-1.120273584	3.02098719	-5.105430643	6.44E-07	1.71E-06	5.063789
EPX	-0.115773801	0.25001526	-5.104511242	6.47E-07	1.71E-06	5.059531516
TMPRSS	-0.661827204	1.41399688	-5.10389347	6.49E-07	1.72E-06	5.056671144
LY6E	0.869191989	7.06307016	5.103181245	6.51E-07	1.72E-06	5.053373791
DDX10	-0.311446843	3.03315372	-5.103061702	6.52E-07	1.72E-06	5.052820385
GPR89B	-0.170719845	1.27720354	-5.10239488	6.54E-07	1.73E-06	5.049733627
BCL3	0.469686394	4.73368532	5.102242832	6.54E-07	1.73E-06	5.049029837
ATRNL1	-0.468401687	0.53390574	-5.101126704	6.58E-07	1.74E-06	5.043864079
TBC1D3:	-0.249053784	1.23385522	-5.100641049	6.59E-07	1.74E-06	5.04161662
NR2F1	0.693616352	3.45553133	5.100050831	6.61E-07	1.75E-06	5.038885508
DLK2	-0.22682903	0.76155335	-5.099935409	6.61E-07	1.75E-06	5.038351446
GRM4	0.586533186	0.83345119	5.099766294	6.62E-07	1.75E-06	5.037568964
SH2D4B	-0.143010706	0.16632667	-5.099519925	6.63E-07	1.75E-06	5.036429069
RSRC1	-0.306864513	3.66083931	-5.0990773	6.64E-07	1.75E-06	5.034381257
C22orf23	-0.429730546	2.26529783	-5.098464704	6.66E-07	1.76E-06	5.031547309
TEK	-0.547299269	3.59741452	-5.097845912	6.68E-07	1.76E-06	5.028684976
IGSF9	0.596003724	2.27842607	5.097556836	6.69E-07	1.77E-06	5.027347904
DNASE1	-0.312565189	2.06595821	-5.097534006	6.69E-07	1.77E-06	5.027242311
ZNF282	-0.289321979	4.64269251	-5.097362643	6.70E-07	1.77E-06	5.026449728
FN3KRP	-0.309055826	5.351336	-5.096213845	6.73E-07	1.78E-06	5.021136925
CENPQ	-0.287996823	3.02128984	-5.094494464	6.79E-07	1.79E-06	5.013187193
SYNE4	-0.358863044	4.44595296	-5.094339579	6.79E-07	1.79E-06	5.012471172
RFFL	-0.521452377	4.00869199	-5.09342655	6.82E-07	1.80E-06	5.008250684
SAMSN1	0.635703099	1.67636448	5.093290434	6.83E-07	1.80E-06	5.007621537
UFM1	-0.329237371	5.77221867	-5.093068765	6.84E-07	1.80E-06	5.006596983
WDR73	-0.276174111	1.58957999	-5.091885032	6.87E-07	1.81E-06	5.001126395
WDR37	-0.351292921	3.84018076	-5.090208798	6.93E-07	1.83E-06	4.993381496
C10orf55	0.604208478	1.46589814	5.090189613	6.93E-07	1.83E-06	4.993292866
LIPF	-0.149715113	0.09705551	-5.088621171	6.98E-07	1.84E-06	4.986047918
SPTBN5	-0.359714699	1.32885301	-5.088186404	7.00E-07	1.84E-06	4.984039963
FMO1	-0.327292078	0.6122127	-5.086690722	7.05E-07	1.86E-06	4.977133286
ADCYAP	-0.621983429	1.04335847	-5.086286844	7.06E-07	1.86E-06	4.975268564
TTC32	-0.393421969	3.73435221	-5.085636398	7.08E-07	1.87E-06	4.97226568

HNRNPA	-0.344275507	6.09708226	-5.085180925	7.10E-07	1.87E-06	4.970163107
TMEM39.	-0.373958679	4.32210848	-5.085029693	7.10E-07	1.87E-06	4.969465018
TNIP2	-0.249688563	5.44214682	-5.084717994	7.11E-07	1.87E-06	4.968026263
VASN	0.652692492	6.63313424	5.083242133	7.16E-07	1.89E-06	4.961214897
GLB1L	-0.395035398	4.15052057	-5.082224629	7.20E-07	1.90E-06	4.956519867
SELENO	-0.385893335	3.48688543	-5.081091053	7.24E-07	1.91E-06	4.951290155
EPN3	-0.539031393	2.23823778	-5.080840271	7.25E-07	1.91E-06	4.950133312
PPP5C	-0.30809039	4.98259427	-5.079338858	7.30E-07	1.92E-06	4.943208341
PPP1R1:	-0.389133036	2.40521411	-5.078498617	7.33E-07	1.93E-06	4.939333625
LHFPL1	-0.442945632	1.06707652	-5.07845634	7.33E-07	1.93E-06	4.93913868
NXN	0.686503408	4.48137278	5.078424299	7.33E-07	1.93E-06	4.938990939
PHKG2	-0.297508948	4.10786401	-5.078074002	7.34E-07	1.93E-06	4.937375742
GABPB1	-0.293726059	3.38624629	-5.076649726	7.39E-07	1.94E-06	4.930809428
IREB2	-0.419428406	4.00337994	-5.074974034	7.45E-07	1.96E-06	4.923085948
GLIPR1L	-0.376475535	1.22955151	-5.074662845	7.46E-07	1.96E-06	4.921651864
CNOT2	-0.240474456	3.77391743	-5.074479996	7.47E-07	1.96E-06	4.92080926
GREM1	0.462811186	0.61250642	5.072661905	7.54E-07	1.98E-06	4.912432458
SEZ6L2	1.108894232	3.85382746	5.072228083	7.55E-07	1.98E-06	4.910434
ICE2	-0.310564663	3.06555069	-5.070799358	7.60E-07	2.00E-06	4.903853373
SBDS	-0.358347613	7.67111654	-5.070574627	7.61E-07	2.00E-06	4.902818414
ITGA8	-0.440428641	1.98660267	-5.070213666	7.63E-07	2.00E-06	4.901156148
SRGAP2	0.378779721	2.32598389	5.069680511	7.64E-07	2.01E-06	4.898701084
PDE4B	-0.551618894	2.70493886	-5.068560114	7.69E-07	2.02E-06	4.893542588
DIXDC1	-0.454926465	3.34706247	-5.068510119	7.69E-07	2.02E-06	4.893312428
TRIM3	-0.22477474	2.17319125	-5.068301054	7.69E-07	2.02E-06	4.892349966
ADGRF1	0.740987989	3.179752	5.067805553	7.71E-07	2.02E-06	4.89006899
NBR1	-0.379424051	6.28153605	-5.067420804	7.73E-07	2.03E-06	4.888297971
PTAFR	0.636394571	2.81474021	5.066872047	7.75E-07	2.03E-06	4.885772207
KIAA195	-0.298426643	1.73172816	-5.066780471	7.75E-07	2.03E-06	4.885350727
HIGD2B	-0.122935641	0.12636642	-5.06657759	7.76E-07	2.03E-06	4.884417001
GRP	0.696597453	0.97585135	5.066221762	7.77E-07	2.04E-06	4.882779424
ABRACL	-0.307809625	6.08592003	-5.065698827	7.79E-07	2.04E-06	4.880372971
ZNF429	-0.38418246	2.96237969	-5.065062956	7.81E-07	2.05E-06	4.877447077
CD300LC	-0.356301794	0.4831478	-5.064860208	7.82E-07	2.05E-06	4.876514217
PRRG3	-0.335182657	0.53527071	-5.063909408	7.86E-07	2.06E-06	4.872139919
ZBTB26	-0.3728339	3.15743654	-5.063889295	7.86E-07	2.06E-06	4.872047391
NAT2	-0.062625755	0.07998041	-5.063638416	7.87E-07	2.06E-06	4.870893299
PTPN7	0.642002058	1.55805845	5.063303041	7.88E-07	2.06E-06	4.869350582
LTBP1	0.739160161	3.17261938	5.061864991	7.93E-07	2.08E-06	4.862736535
SELPLG	0.682954481	4.07567179	5.061204272	7.96E-07	2.08E-06	4.859698197

ZBTB14	-0.335970713	3.93366628	-5.060688501	7.98E-07	2.09E-06	4.857326628
NOP2	-0.260435877	4.29759146	-5.060152557	8.00E-07	2.09E-06	4.854862513
STAU1	-0.359377474	6.95721753	-5.05847163	8.06E-07	2.11E-06	4.847135485
TSPAN5	-0.556357114	3.33642943	-5.058172584	8.08E-07	2.11E-06	4.845761021
TP53RK	-0.270781888	4.28043545	-5.058097791	8.08E-07	2.11E-06	4.845417273
PATZ1	-0.362471287	5.47906682	-5.057770184	8.09E-07	2.12E-06	4.843911637
GRK5	-0.549308156	3.36907729	-5.056764482	8.13E-07	2.13E-06	4.839290067
ZNF416	-0.313669797	3.07668986	-5.056051573	8.16E-07	2.13E-06	4.836014445
KTN1	-0.417938289	5.38934747	-5.055552589	8.18E-07	2.14E-06	4.833721973
CD84	0.551511391	1.54666305	5.053799253	8.25E-07	2.16E-06	4.825668125
LUM	1.403111313	4.84317483	5.053186151	8.27E-07	2.16E-06	4.822852417
TMPRSS	-0.146712974	0.37146327	-5.053168707	8.27E-07	2.16E-06	4.822772306
TSPAN1:	-0.564729298	4.70310794	-5.05217667	8.31E-07	2.17E-06	4.818216919
TRAF3IP	-0.375002667	4.34821115	-5.052118098	8.31E-07	2.17E-06	4.817947984
ZC3H18	-0.292442522	4.08743726	-5.051904823	8.32E-07	2.17E-06	4.81696874
CES3	-0.422535523	1.03398214	-5.051032419	8.36E-07	2.18E-06	4.81296348
MEDAG	0.842497747	3.76322198	5.050825674	8.36E-07	2.18E-06	4.812014386
C8orf58	-0.287604193	2.96160554	-5.04944675	8.42E-07	2.20E-06	4.805685034
KCNA2	-0.374413139	0.45741081	-5.049318048	8.42E-07	2.20E-06	4.805094353
STX12	-0.421575676	6.82639606	-5.048316752	8.46E-07	2.21E-06	4.800499322
ZBTB49	-0.323614642	2.70052831	-5.046655878	8.53E-07	2.23E-06	4.792879069
TENT4B	-0.317403316	2.95423975	-5.046538531	8.54E-07	2.23E-06	4.792340749
ZNF737	-0.548738775	3.49500646	-5.046099025	8.55E-07	2.23E-06	4.790324636
RPAP1	-0.314051781	3.42413057	-5.04584072	8.56E-07	2.23E-06	4.789139798
ZNF517	-0.291634062	3.24662085	-5.045666439	8.57E-07	2.23E-06	4.788340407
FIGNL1	0.373650914	2.4401083	5.045663888	8.57E-07	2.23E-06	4.788328706
GTF2F1	-0.29709286	6.91435377	-5.045487653	8.58E-07	2.24E-06	4.78752037
GDI2	-0.34827193	7.25638479	-5.045439995	8.58E-07	2.24E-06	4.787301782
FAM120E	-0.337361339	4.41393077	-5.045195392	8.59E-07	2.24E-06	4.786179918
CUEDC1	-0.368540151	4.36607154	-5.044260309	8.63E-07	2.25E-06	4.781891587
TBCK	-0.318395506	3.0944669	-5.044131865	8.63E-07	2.25E-06	4.781302585
ITGBL1	0.629218642	1.43289941	5.044077416	8.64E-07	2.25E-06	4.781052904
GATD3B	-0.319174323	1.25535807	-5.044025836	8.64E-07	2.25E-06	4.780816383
TREML2	0.193650716	0.27880812	5.04362141	8.66E-07	2.25E-06	4.77896194
NUGGC	-0.58800964	1.13392527	-5.042753359	8.69E-07	2.26E-06	4.774982015
PTH1R	-0.411416018	1.08050687	-5.042550149	8.70E-07	2.26E-06	4.7740504
SNX19	-0.398123662	5.02584248	-5.042218759	8.71E-07	2.27E-06	4.772531206
APBA3	-0.245593365	3.19806297	-5.042209947	8.71E-07	2.27E-06	4.772490813
CHD3	-0.407896857	5.42797058	-5.042113928	8.72E-07	2.27E-06	4.772050649
RAB5B	-0.265045557	6.58556417	-5.039135152	8.84E-07	2.30E-06	4.758398948

FAM189E	-0.309784229	3.44189036	-5.038670149	8.86E-07	2.31E-06	4.756268441
KCTD21	-0.319734175	3.91294807	-5.037887457	8.89E-07	2.31E-06	4.752682734
TAFA2	-0.79242125	1.3725965	-5.037843581	8.90E-07	2.31E-06	4.752481745
PAG1	0.487653003	2.1026101	5.036924737	8.94E-07	2.32E-06	4.748272908
HFM1	-0.321047975	0.73120899	-5.036623549	8.95E-07	2.33E-06	4.74689343
CD1B	0.547342246	0.73157234	5.036590504	8.95E-07	2.33E-06	4.746742085
SSC5D	0.577734007	1.7773405	5.036503709	8.95E-07	2.33E-06	4.746344564
FAR1	-0.34361297	4.14325752	-5.036203089	8.97E-07	2.33E-06	4.744967782
ARMS2	-0.1319327	0.15211397	-5.03538906	9.00E-07	2.34E-06	4.741240022
HMGA2	0.899790021	2.66186482	5.034921125	9.02E-07	2.34E-06	4.739097387
ZMYM6	-0.2363796	2.54644571	-5.034879252	9.02E-07	2.34E-06	4.738905663
CD5	0.694881301	1.68480718	5.03380196	9.07E-07	2.36E-06	4.733973485
TFIP11	-0.408267426	3.98784264	-5.033474512	9.08E-07	2.36E-06	4.732474495
SRSF7	-0.336144398	6.06074763	-5.033434397	9.08E-07	2.36E-06	4.732290865
CRLF1	1.570925789	4.52941739	5.033406646	9.09E-07	2.36E-06	4.73216383
METTL14	-0.330642098	3.65009799	-5.032927233	9.11E-07	2.36E-06	4.729969347
MR1	-0.405819739	4.4517285	-5.032214912	9.14E-07	2.37E-06	4.726709061
NPIPB6	-0.217810651	0.47285154	-5.031071637	9.19E-07	2.38E-06	4.721477092
EIF2AK4	-0.340752273	4.01150864	-5.031003007	9.19E-07	2.38E-06	4.72116305
IL4R	0.474523882	4.62172257	5.03084372	9.20E-07	2.39E-06	4.720434191
VPS50	-0.299025965	2.91728209	-5.030219997	9.22E-07	2.39E-06	4.717580361
KXD1	-0.256958714	5.50120454	-5.030130385	9.23E-07	2.39E-06	4.717170369
TRIM22	0.651636512	4.83992584	5.02960511	9.25E-07	2.40E-06	4.714767246
HENMT1	-0.43239173	2.22380154	-5.028764581	9.29E-07	2.41E-06	4.710922274
TRIM27	-0.28505434	4.67812112	-5.028551937	9.30E-07	2.41E-06	4.709949625
HSD11B1	0.594786267	1.25734397	5.028473667	9.30E-07	2.41E-06	4.709591622
DDX27	-0.287224783	5.20822982	-5.028030808	9.32E-07	2.41E-06	4.707566082
IGLL5	1.707189291	2.82595097	5.027757383	9.33E-07	2.42E-06	4.706315572
EIF2AK1	-0.201107861	6.3461921	-5.027587613	9.34E-07	2.42E-06	4.705539154
YPEL5	-0.324404442	6.67385634	-5.025946854	9.41E-07	2.44E-06	4.698036512
IDS	0.483149197	5.88705346	5.025528328	9.43E-07	2.44E-06	4.696123053
EXOSC9	-0.243028572	3.70954341	-5.025138724	9.45E-07	2.45E-06	4.69434194
LGI2	-0.514653786	1.22235481	-5.024725933	9.47E-07	2.45E-06	4.69245495
MITD1	-0.273005036	3.42837953	-5.022360837	9.57E-07	2.48E-06	4.681645841
CLEC12A	0.401960227	0.92190025	5.021648932	9.61E-07	2.49E-06	4.678393068
UGT2B11	-0.140674918	0.17867505	-5.021517322	9.61E-07	2.49E-06	4.677791767
RAC1	-0.241212115	7.9743129	-5.01936895	9.71E-07	2.51E-06	4.667978114
FBXO22	-0.262845939	2.98858693	-5.018732042	9.74E-07	2.52E-06	4.665069411
CSNK2A	-0.306632282	5.32463526	-5.017945967	9.78E-07	2.53E-06	4.661479896
ZCCHC4	-0.253768216	2.70945952	-5.017768888	9.78E-07	2.53E-06	4.660671348

ACAA2	-0.583515486	5.63146714	-5.017682945	9.79E-07	2.53E-06	4.66027894
ZNF16	-0.293073881	3.28103896	-5.017108049	9.82E-07	2.54E-06	4.657654154
OCRL	-0.401292847	4.76380787	-5.016796518	9.83E-07	2.54E-06	4.656231905
RC3H1	-0.359303537	3.16061479	-5.01555453	9.89E-07	2.56E-06	4.650562523
IFITM10	0.414671839	1.52485692	5.015535939	9.89E-07	2.56E-06	4.65047767
TMLHE	-0.318882749	2.86890019	-5.01499412	9.91E-07	2.56E-06	4.648004761
CD177	1.165005407	1.31870624	5.014903511	9.92E-07	2.56E-06	4.647591235
TRMT1L	-0.317252595	3.99639203	-5.014498234	9.94E-07	2.57E-06	4.645741688
AGR3	-0.590565032	0.88029063	-5.014196505	9.95E-07	2.57E-06	4.644364778
CDH2	-1.220018855	4.4537179	-5.014104111	9.96E-07	2.57E-06	4.64394316
LEMD1	0.673229296	2.702702	5.013956885	9.96E-07	2.57E-06	4.643271344
REL	0.413467606	1.54963428	5.013502464	9.98E-07	2.58E-06	4.64119785
COL5A1	0.865989388	3.50343698	5.012687461	1.00E-06	2.59E-06	4.637479429
UGT3A2	-0.514602324	0.52198565	-5.011286414	1.01E-06	2.60E-06	4.631088359
GPA33	0.255166673	0.34090529	5.010329302	1.01E-06	2.62E-06	4.626723206
GSDMD	-0.33890228	5.57372126	-5.010225049	1.01E-06	2.62E-06	4.626247777
TOGARA	-0.385159523	3.01956293	-5.010084032	1.01E-06	2.62E-06	4.625604698
NBEA	-0.630595206	2.99461939	-5.009859144	1.02E-06	2.62E-06	4.62457918
IQCA1	-0.563440775	4.71352775	-5.00981126	1.02E-06	2.62E-06	4.62436083
GPR83	-0.663480699	1.70039025	-5.00889944	1.02E-06	2.63E-06	4.620203231
TCTEX1I	-0.402179997	1.26312266	-5.008872856	1.02E-06	2.63E-06	4.620082023
ASB6	-0.241041828	4.10229013	-5.008181359	1.02E-06	2.64E-06	4.616929453
KIAA175	0.289979649	0.57715367	5.007460422	1.03E-06	2.65E-06	4.613643045
ZNF583	-0.234325196	1.59334102	-5.007254377	1.03E-06	2.65E-06	4.612703854
ZNF362	-0.321611555	5.3122089	-5.007234455	1.03E-06	2.65E-06	4.612613047
IQCK	-0.420596972	3.9821869	-5.005852029	1.04E-06	2.67E-06	4.606312545
XRCC6	-0.341451912	7.90842501	-5.005582469	1.04E-06	2.67E-06	4.605084177
ZNF839	-0.30886966	2.61192717	-5.005503116	1.04E-06	2.67E-06	4.604722576
WDR18	-0.340710675	5.39399102	-5.005488985	1.04E-06	2.67E-06	4.604658185
H3F3B	-0.336043771	8.30048282	-5.00520867	1.04E-06	2.67E-06	4.60338088
TMEM23	-0.342126689	3.33702882	-5.004839449	1.04E-06	2.68E-06	4.601698547
SLC37A4	-0.2595298	3.71356019	-5.001529873	1.06E-06	2.72E-06	4.586623236
GRIK5	-0.551967701	4.17993091	-5.001463745	1.06E-06	2.72E-06	4.586322103
ATP2A3	-0.550512243	3.85237502	-5.001173552	1.06E-06	2.72E-06	4.585000663
KIFAP3	-0.314513402	4.44410649	-5.001122576	1.06E-06	2.72E-06	4.584768539
TRIM66	-0.394817771	2.08277847	-5.000633289	1.06E-06	2.73E-06	4.582540655
BMS1	-0.263528379	4.19258212	-4.99984498	1.07E-06	2.74E-06	4.578951595
CYP21A2	0.344083514	0.90981383	4.999665471	1.07E-06	2.74E-06	4.578134381
WDFY2	-0.264893853	2.81661966	-4.998493637	1.07E-06	2.76E-06	4.572800208
ZUP1	-0.264943808	3.16738707	-4.998162584	1.07E-06	2.76E-06	4.571293444

ISM1	-0.872821218	5.08487716	-4.997975372	1.07E-06	2.76E-06	4.570441397
ZNF513	-0.371401353	4.80328765	-4.997679869	1.08E-06	2.76E-06	4.56909655
THEMIS2	0.650308386	4.56877825	4.997436811	1.08E-06	2.77E-06	4.567990427
SSBP2	-0.597155223	3.67785647	-4.997264081	1.08E-06	2.77E-06	4.567204384
PNKP	-0.293521804	5.00188052	-4.997180739	1.08E-06	2.77E-06	4.566825131
UBE2Q1	-0.271996579	5.93502849	-4.99428332	1.09E-06	2.81E-06	4.553643367
NRF1	-0.28849973	4.05606141	-4.993982619	1.09E-06	2.81E-06	4.552275691
SH2D5	0.388819207	1.16498552	4.992709798	1.10E-06	2.83E-06	4.546487287
KLHL10	-0.152583619	0.36229372	-4.991819592	1.11E-06	2.84E-06	4.542439615
SLC30A4	-0.345661462	2.48511604	-4.991765373	1.11E-06	2.84E-06	4.542193108
P2RY10	0.563631191	0.9305251	4.991506768	1.11E-06	2.84E-06	4.541017381
BPIFA2	-0.364081387	0.95594657	-4.991092556	1.11E-06	2.85E-06	4.539134304
HSPA14	-0.289853675	3.52060906	-4.990849722	1.11E-06	2.85E-06	4.538030398
CORT	-0.214573145	0.65339658	-4.990737133	1.11E-06	2.85E-06	4.537518595
LRRC32	-0.525845095	4.54435177	-4.990631764	1.11E-06	2.85E-06	4.537039616
SYT8	0.254630861	0.29134417	4.990154618	1.11E-06	2.86E-06	4.534870754
PDE4A	-0.526444105	4.05717983	-4.988775669	1.12E-06	2.88E-06	4.528603718
PABPC5	-0.322869833	1.27854589	-4.988749242	1.12E-06	2.88E-06	4.528483626
MEGF6	0.474119119	2.44655266	4.988668827	1.12E-06	2.88E-06	4.528118204
RAB5A	-0.325815461	5.86093325	-4.985843329	1.14E-06	2.92E-06	4.515281572
XYLT2	-0.274734136	4.57070686	-4.98572212	1.14E-06	2.92E-06	4.514731036
MAP1A	-0.55050512	2.22917112	-4.985719476	1.14E-06	2.92E-06	4.514719026
GPR146	-0.246037986	0.9101897	-4.985655512	1.14E-06	2.92E-06	4.514428504
MOB3B	-0.512778747	2.65260398	-4.985461826	1.14E-06	2.92E-06	4.513548807
ARAF	-0.335271538	5.87042209	-4.984751862	1.14E-06	2.93E-06	4.510324485
LSM7	-0.356401134	5.61009811	-4.983377269	1.15E-06	2.95E-06	4.504082805
ELP1	-0.370901677	4.44097259	-4.983137083	1.15E-06	2.95E-06	4.502992329
SRP68	-0.332733739	5.70261574	-4.982872201	1.15E-06	2.95E-06	4.501789774
KCNK6	0.445516932	2.13116038	4.982478445	1.16E-06	2.96E-06	4.500002239
AURKA	-0.311979415	1.76620631	-4.982252664	1.16E-06	2.96E-06	4.498977313
RSBN1L	-0.33994495	4.05890997	-4.981978308	1.16E-06	2.96E-06	4.497731929
EN1	0.18197934	0.25998275	4.981862841	1.16E-06	2.97E-06	4.49720781
YRDC	-0.280236405	4.37500753	-4.981731462	1.16E-06	2.97E-06	4.496611473
BTK	0.532116382	1.72550831	4.981708217	1.16E-06	2.97E-06	4.496505964
MROH2A	-0.79193369	0.74107807	-4.981580404	1.16E-06	2.97E-06	4.495925829
HLA-DQB	0.990940597	6.03369057	4.980677503	1.17E-06	2.98E-06	4.491827969
CCDC18	-0.303284105	1.52145262	-4.980590619	1.17E-06	2.98E-06	4.491433674
APEX1	-0.320030026	7.74988479	-4.980218145	1.17E-06	2.99E-06	4.489743387
RRP15	-0.300337122	2.96060985	-4.977455073	1.18E-06	3.02E-06	4.477207801
HYI	-0.402567721	4.11103751	-4.976159055	1.19E-06	3.04E-06	4.471329959

NKPD1	-0.211851958	0.37194996	-4.975411172	1.20E-06	3.05E-06	4.46793865
BDKRB1	-0.238432755	0.47786587	-4.975020008	1.20E-06	3.06E-06	4.466165072
ZNF230	-0.250110652	1.95217222	-4.974807441	1.20E-06	3.06E-06	4.465201315
TRO	-0.590623059	2.47769745	-4.974590908	1.20E-06	3.06E-06	4.464219612
IBTK	-0.35579399	4.35351181	-4.97446754	1.20E-06	3.06E-06	4.46366031
GMPS	-0.327175028	4.19929403	-4.974311624	1.20E-06	3.07E-06	4.462953467
GSC	-0.334504991	0.76767601	-4.974274075	1.20E-06	3.07E-06	4.46278324
USP11	-0.375363091	6.52394524	-4.973872801	1.20E-06	3.07E-06	4.460964163
MRC2	1.087463094	5.73684739	4.973612636	1.21E-06	3.08E-06	4.459784835
SLC2A3	0.719243372	4.37511847	4.972790134	1.21E-06	3.09E-06	4.456056757
MAPK9	-0.291948133	4.2355982	-4.972182294	1.21E-06	3.09E-06	4.453301986
NPIPB2	-0.665154349	1.57750127	-4.971284192	1.22E-06	3.11E-06	4.449232237
CIB2	-0.606560094	2.994656	-4.970904059	1.22E-06	3.11E-06	4.447509844
CAT	-0.565647471	7.49729788	-4.970149901	1.23E-06	3.12E-06	4.44409306
NAA35	-0.354022253	4.11840899	-4.969770727	1.23E-06	3.13E-06	4.442375334
OPHN1	-0.397370349	1.96649168	-4.969642454	1.23E-06	3.13E-06	4.441794262
CSPG4	-0.583715351	3.12289139	-4.96961946	1.23E-06	3.13E-06	4.4416901
TRERF1	-0.362750008	1.85378097	-4.969562758	1.23E-06	3.13E-06	4.441433245
OLAH	0.597650421	1.44246664	4.968356779	1.24E-06	3.15E-06	4.435970859
CTAGE4	-0.147393137	0.31955667	-4.968135037	1.24E-06	3.15E-06	4.434966616
CERS2	-0.439135771	6.87051047	-4.967625028	1.24E-06	3.16E-06	4.432656982
TMC7	-0.258731095	1.12570375	-4.966787078	1.24E-06	3.17E-06	4.428862659
DDX5	-0.411163714	8.06168027	-4.966680146	1.25E-06	3.17E-06	4.4283785
COPS7B	-0.271898877	4.22939769	-4.965982973	1.25E-06	3.18E-06	4.425222083
TAFA1	-0.10330814	0.23033896	-4.965702255	1.25E-06	3.18E-06	4.42395125
UNC79	-0.143506563	0.35514468	-4.965079681	1.25E-06	3.19E-06	4.421133018
CASKIN2	-0.352084031	5.04911678	-4.96430458	1.26E-06	3.20E-06	4.417624743
TNFRSF	0.528321433	3.27205215	4.962812399	1.27E-06	3.23E-06	4.410872081
GOLM1	-0.474741142	5.76043864	-4.962804392	1.27E-06	3.23E-06	4.410835848
EPHB4	-0.374066765	4.15803412	-4.961964724	1.27E-06	3.24E-06	4.407036784
LAMA4	-0.495997956	3.66962172	-4.961685935	1.27E-06	3.24E-06	4.405775529
SPDYE3	-0.235727346	2.52972661	-4.960967323	1.28E-06	3.25E-06	4.40252475
TRDMT1	-0.28248537	1.85252262	-4.960792344	1.28E-06	3.25E-06	4.40173326
LARP4B	-0.289910618	4.27308299	-4.960323905	1.28E-06	3.26E-06	4.399614457
WDR24	-0.264530432	4.05311592	-4.960319496	1.28E-06	3.26E-06	4.399594515
THNSL2	-0.589473905	4.67994724	-4.95998974	1.29E-06	3.27E-06	4.398103093
SLC15A1	-0.323496917	0.47323377	-4.959143354	1.29E-06	3.28E-06	4.394275425
SALL4	-0.50395433	1.20111912	-4.95837344	1.29E-06	3.29E-06	4.390794058
PDCL	-0.32644955	4.25359162	-4.958289673	1.30E-06	3.29E-06	4.390415309
ZKSCAN	-0.313041928	2.72947314	-4.957184185	1.30E-06	3.31E-06	4.385417409

ADCY5	-0.316565221	0.7242487	-4.957082615	1.30E-06	3.31E-06	4.38495826
BICC1	0.508069388	1.62299667	4.95642425	1.31E-06	3.32E-06	4.381982283
BUB3	-0.249231241	4.46666983	-4.954909697	1.32E-06	3.34E-06	4.375137356
NINL	-0.406003776	3.49920037	-4.954105757	1.32E-06	3.35E-06	4.371504703
AP1M2	-0.3522134	7.63023739	-4.954028349	1.32E-06	3.35E-06	4.371154954
AGMO	-0.270458778	0.35268448	-4.95393329	1.32E-06	3.35E-06	4.370725464
PPP1R1!	-0.415835641	5.24872011	-4.953758418	1.32E-06	3.36E-06	4.36993538
HIST1H2	-0.261340993	0.5065871	-4.953042701	1.33E-06	3.37E-06	4.366701961
FOS	-1.063331272	8.5879241	-4.952682725	1.33E-06	3.37E-06	4.365075833
TNPO1	-0.395864364	4.77389721	-4.952412725	1.33E-06	3.38E-06	4.363856216
G3BP2	-0.458581032	5.96202351	-4.951948883	1.33E-06	3.38E-06	4.36176113
PSPC1	-0.31002692	5.21145644	-4.951465132	1.34E-06	3.39E-06	4.35957629
RMND1	-0.313348713	5.30021443	-4.951241138	1.34E-06	3.39E-06	4.358564687
SVIP	-0.330526204	5.38145291	-4.950507975	1.34E-06	3.40E-06	4.355253849
SEPTIN2	-0.392007959	7.47862689	-4.950135004	1.35E-06	3.41E-06	4.353569729
KDELR1	-0.281071877	8.44187009	-4.949509085	1.35E-06	3.42E-06	4.350743681
ITM2C	-0.657722234	7.24995333	-4.949487198	1.35E-06	3.42E-06	4.350644866
ST6GALI	-0.388199108	4.57085525	-4.949256931	1.35E-06	3.42E-06	4.349605276
ZFYVE27	-0.301203863	4.9745782	-4.948367262	1.36E-06	3.44E-06	4.34558906
AMFR	-0.330547494	6.23313439	-4.947972569	1.36E-06	3.44E-06	4.343807498
THBD	-0.588419744	4.56163481	-4.946903985	1.37E-06	3.46E-06	4.338984711
USP47	-0.335979386	4.46813605	-4.946751427	1.37E-06	3.46E-06	4.338296252
TLL2	-0.38870614	0.59361126	-4.946647672	1.37E-06	3.46E-06	4.337828037
NOB1	-0.326885268	6.13653295	-4.945939539	1.37E-06	3.47E-06	4.334632669
ZNF148	-0.425866652	4.44092962	-4.945924229	1.37E-06	3.47E-06	4.334563586
NARS2	-0.341416386	4.32503737	-4.944873163	1.38E-06	3.49E-06	4.329821482
SYT15	-0.109249106	0.20811378	-4.944424329	1.38E-06	3.50E-06	4.327796722
NPY5R	-0.121149568	0.17086826	-4.942866449	1.39E-06	3.52E-06	4.320770072
TRMT13	-0.348567053	3.30815853	-4.940077338	1.41E-06	3.57E-06	4.30819465
DROSHA	-0.307144219	4.16350099	-4.939381017	1.42E-06	3.58E-06	4.305056024
UBR3	-0.430474359	4.30522325	-4.938947318	1.42E-06	3.58E-06	4.303101336
SEMA3G	-0.526392871	3.56557786	-4.93815857	1.42E-06	3.60E-06	4.299546798
LMBRD2	-0.403941082	3.32343821	-4.936709009	1.43E-06	3.62E-06	4.293015489
EXOC3	-0.281731234	4.57052313	-4.936221563	1.44E-06	3.63E-06	4.290819548
PYCR2	-0.239090206	5.57791823	-4.936014688	1.44E-06	3.63E-06	4.289887636
SFXN1	-0.2748088	3.57511381	-4.935650696	1.44E-06	3.64E-06	4.28824803
YTHDF1	-0.281270101	5.98537725	-4.935528922	1.44E-06	3.64E-06	4.28769952
CEACAN	0.287946598	0.49841691	4.935454963	1.44E-06	3.64E-06	4.287366392
SUPT7L	-0.293195472	4.66313471	-4.935324323	1.44E-06	3.64E-06	4.286777965
SNAP91	-0.304700729	0.37498068	-4.934958595	1.44E-06	3.65E-06	4.285130728

B3GALN	-0.447643981	4.43882555	-4.9331647	1.46E-06	3.68E-06	4.277052507
TCIM	0.972953128	6.55527449	4.933087022	1.46E-06	3.68E-06	4.276702765
CSMD2	-0.163472272	0.28084097	-4.931080065	1.47E-06	3.71E-06	4.267668079
ZNF134	-0.334824323	3.88781785	-4.930531506	1.48E-06	3.72E-06	4.26519917
ZNF704	-0.417367291	2.97245407	-4.929388924	1.48E-06	3.74E-06	4.260057457
WDR38	-0.229352701	0.39677947	-4.929024773	1.49E-06	3.75E-06	4.258418954
ARPC5L	-0.28163828	5.30271719	-4.928722678	1.49E-06	3.75E-06	4.257059746
IGFL1	0.291518403	0.29448325	4.927316643	1.50E-06	3.78E-06	4.250734531
GAA	-0.343969479	6.35621344	-4.92681152	1.50E-06	3.78E-06	4.248462536
KCTD17	0.626999918	3.66062175	4.926691704	1.50E-06	3.79E-06	4.247923645
S1PR5	-0.268545882	0.81754467	-4.92617721	1.51E-06	3.79E-06	4.24560975
STEAP1	0.543353727	1.03272351	4.925880202	1.51E-06	3.80E-06	4.244274069
LMBR1	-0.399961652	3.95323018	-4.925821597	1.51E-06	3.80E-06	4.244010522
C11orf49	-0.361915554	4.72919499	-4.925790918	1.51E-06	3.80E-06	4.243872558
ANGPT2	-0.56414862	2.79777433	-4.925171904	1.51E-06	3.81E-06	4.241089039
ZMYND1	0.329046056	2.02025818	4.924441725	1.52E-06	3.82E-06	4.237806009
APH1A	-0.326833091	7.62307685	-4.924261432	1.52E-06	3.82E-06	4.236995439
IL17C	0.12868043	0.24678242	4.923478173	1.52E-06	3.84E-06	4.233474304
SIRPB1	0.304796059	0.53197209	4.921463509	1.54E-06	3.87E-06	4.224419531
TBX3	-0.497385666	6.6603842	-4.920941019	1.54E-06	3.88E-06	4.222071732
HNRNPF	-0.365735237	5.45220088	-4.920733359	1.54E-06	3.89E-06	4.221138674
FAM47E	-0.322726814	1.97382963	-4.9204937	1.55E-06	3.89E-06	4.22006188
TUBA3D	-0.269055254	0.74731119	-4.920007965	1.55E-06	3.90E-06	4.21787959
DUSP2	0.959463841	2.99144637	4.919082533	1.56E-06	3.91E-06	4.213722338
RGP1	-0.367488581	4.50420575	-4.91812623	1.56E-06	3.93E-06	4.209427087
PRDM5	-0.315657618	1.96863705	-4.913927155	1.59E-06	4.01E-06	4.190575048
SNW1	-0.320162249	6.34995186	-4.91378528	1.60E-06	4.01E-06	4.189938325
NXPH4	-0.600415577	0.94623565	-4.912223079	1.61E-06	4.04E-06	4.182928278
ITGB1BF	-0.30605899	4.36760072	-4.912162684	1.61E-06	4.04E-06	4.182657308
NNMT	0.877976505	3.49648356	4.911651523	1.61E-06	4.05E-06	4.180364003
PCED1B	0.585954547	3.27730566	4.91156446	1.61E-06	4.05E-06	4.17997342
LRRTM2	-0.077996898	0.16259934	-4.910607817	1.62E-06	4.07E-06	4.175682072
RPE	-0.336482069	4.82411516	-4.908327545	1.64E-06	4.11E-06	4.165455929
CTSH	0.811836487	6.94135728	4.907099799	1.65E-06	4.13E-06	4.159951592
RBM14	-0.286066866	5.1841616	-4.905715832	1.66E-06	4.16E-06	4.153748232
RPL39	-0.338347562	6.93533182	-4.905169518	1.66E-06	4.17E-06	4.151299887
RPS29	-0.376727127	6.29661679	-4.905102329	1.66E-06	4.17E-06	4.150998787
CSTA	0.618411976	1.8811766	4.904916743	1.66E-06	4.17E-06	4.150167133
MTREX	-0.343509986	4.71224856	-4.904706656	1.66E-06	4.18E-06	4.14922571
PCF11	-0.369392908	4.00782817	-4.904661185	1.66E-06	4.18E-06	4.149021958

WDR48	-0.339342471	4.7612471	-4.904095975	1.67E-06	4.19E-06	4.146489381
HMGB1	-0.313124078	6.38425829	-4.903334838	1.67E-06	4.20E-06	4.143079283
AADAT	-0.462996346	2.85237628	-4.901989304	1.69E-06	4.23E-06	4.137052003
HIVEP3	-0.375879005	2.34828844	-4.901908622	1.69E-06	4.23E-06	4.136690635
SYNDIG1	0.609111794	1.74192751	4.901674838	1.69E-06	4.23E-06	4.135643562
LMLN	-0.297176943	2.32687214	-4.900645131	1.70E-06	4.25E-06	4.13103219
LTA	0.408651825	0.69251632	4.900387122	1.70E-06	4.26E-06	4.129876864
PCDHB4	-0.327391004	1.11018849	-4.899966523	1.70E-06	4.26E-06	4.127993595
CCDC9	-0.313994871	4.74938614	-4.899822861	1.70E-06	4.27E-06	4.127350363
DYRK1A	-0.312420696	4.53452718	-4.899756792	1.70E-06	4.27E-06	4.127054556
PDE12	-0.309045076	3.48936657	-4.899242185	1.71E-06	4.28E-06	4.124750617
FBXO46	-0.281749429	4.4292263	-4.899012444	1.71E-06	4.28E-06	4.123722116
KDM2B	-0.262330546	2.6218617	-4.898242839	1.71E-06	4.30E-06	4.120277041
MKNK2	-0.31316794	7.06802127	-4.898170196	1.72E-06	4.30E-06	4.119951882
KIAA154	-0.370293986	1.79599698	-4.897421418	1.72E-06	4.31E-06	4.116600512
TP53I13	-0.380823188	4.74730971	-4.897366574	1.72E-06	4.31E-06	4.116355056
CTU1	-0.379490911	2.46130386	-4.896530193	1.73E-06	4.33E-06	4.112612127
MADD	-0.280472523	4.04650293	-4.895929852	1.73E-06	4.34E-06	4.109925841
5-Mar	-0.322829283	5.04646581	-4.895034997	1.74E-06	4.36E-06	4.105922222
IFRD2	-0.305077889	5.24357735	-4.894972489	1.74E-06	4.36E-06	4.105642583
C17orf53	0.24668837	1.17486398	4.894704898	1.74E-06	4.36E-06	4.104445499
ZEB1	-0.457251773	3.04672995	-4.894517531	1.74E-06	4.36E-06	4.103607335
CDK4	-0.430698351	5.89221357	-4.894355379	1.75E-06	4.37E-06	4.102881989
MOSMO	-0.296634387	2.80873797	-4.89429175	1.75E-06	4.37E-06	4.10259737
COQ10B	-0.304070541	5.31158895	-4.894158549	1.75E-06	4.37E-06	4.102001549
CALHM5	-0.281899862	1.19744867	-4.893955699	1.75E-06	4.37E-06	4.101094211
DHX34	-0.306419829	3.77261805	-4.893875881	1.75E-06	4.37E-06	4.100737193
ALDH5A1	-0.363191508	4.95298565	-4.893851035	1.75E-06	4.37E-06	4.100626061
PECR	-0.534915201	4.56319822	-4.892765971	1.76E-06	4.40E-06	4.095773231
RNF40	-0.444622788	4.8759199	-4.892509283	1.76E-06	4.40E-06	4.094625353
GALNTL6	0.299783299	0.6729882	4.891668055	1.77E-06	4.42E-06	4.090863825
UBOX5	-0.261152906	3.50714227	-4.890771715	1.78E-06	4.43E-06	4.086856458
ANK2	-0.571512045	1.3603358	-4.889792799	1.78E-06	4.45E-06	4.082480606
CACTIN	-0.270922327	3.44683658	-4.889775562	1.78E-06	4.45E-06	4.08240356
CDS2	-0.297349734	4.37730742	-4.889270519	1.79E-06	4.46E-06	4.080146257
KCTD1	-0.374872259	3.27789642	-4.889112732	1.79E-06	4.47E-06	4.079441062
COL6A6	-0.202976022	0.23408512	-4.888996143	1.79E-06	4.47E-06	4.078920009
GASK1B	-0.528742998	3.71263643	-4.888770184	1.79E-06	4.47E-06	4.077910189
CAPZA2	-0.339320868	5.40572216	-4.888046373	1.80E-06	4.49E-06	4.074675705
CFAP44	-0.462788725	1.63812153	-4.88788198	1.80E-06	4.49E-06	4.073941141

HRK	0.784501989	2.21781777	4.887705029	1.80E-06	4.49E-06	4.073150486
DAW1	0.235094149	0.353348	4.885847047	1.82E-06	4.53E-06	4.064850052
AGK	-0.27264563	3.734304	-4.885179952	1.82E-06	4.54E-06	4.061870477
UBE3A	-0.344038167	4.55356735	-4.884038205	1.83E-06	4.57E-06	4.056771661
CCNDBP	-0.383044456	5.12036425	-4.882013421	1.85E-06	4.61E-06	4.047731803
DNASE1	-0.349884524	4.38959005	-4.881925109	1.85E-06	4.61E-06	4.047337595
APOL1	0.696516868	5.71356697	4.881784651	1.85E-06	4.61E-06	4.046710632
SELENO	-0.357083984	6.76049188	-4.881731697	1.85E-06	4.61E-06	4.046474263
GPR176	-0.43837223	3.00374936	-4.881459775	1.85E-06	4.62E-06	4.045260533
BATF	0.761726511	2.23901575	4.881248773	1.86E-06	4.62E-06	4.044318762
CRNKL1	-0.274943737	4.45687287	-4.880366928	1.86E-06	4.64E-06	4.040383153
RPS15A	-0.324349588	7.52089779	-4.879629764	1.87E-06	4.66E-06	4.037093699
SNX5	-0.37627006	6.71943158	-4.877808653	1.89E-06	4.69E-06	4.028969108
HSPD1	-0.362124363	6.97851603	-4.877396272	1.89E-06	4.70E-06	4.027129683
ARTN	-0.253180544	1.06945737	-4.877353176	1.89E-06	4.70E-06	4.026937463
ZC3H3	-0.335169642	4.68141138	-4.877211402	1.89E-06	4.71E-06	4.026305116
COPB2	-0.371457831	5.86854178	-4.876888387	1.89E-06	4.71E-06	4.024864444
ZSCAN1	-0.371232162	2.47988396	-4.875956083	1.90E-06	4.73E-06	4.020706744
CHMP3	-0.366642444	5.4476478	-4.874941468	1.91E-06	4.75E-06	4.016182719
COLEC1	0.642960778	1.66709152	4.87416578	1.92E-06	4.77E-06	4.012724562
CEP41	-0.233188819	2.01777322	-4.873760135	1.92E-06	4.78E-06	4.010916305
EVPLL	-0.26920012	0.69651749	-4.873220547	1.93E-06	4.79E-06	4.008511162
ATP5MF	-0.065685813	0.15262074	-4.871164854	1.94E-06	4.83E-06	3.999350197
MDFIC	0.490282847	5.17723568	4.869645713	1.96E-06	4.87E-06	3.992582375
TUNAR	-0.681935164	0.61202879	-4.868148071	1.97E-06	4.90E-06	3.985912048
SULT1C2	-0.52492526	3.38847401	-4.868073067	1.97E-06	4.90E-06	3.985578031
ATP6V1F	0.515747771	1.57613067	4.867833115	1.97E-06	4.91E-06	3.984509483
C8orf37	-0.325790649	2.10012388	-4.865852925	1.99E-06	4.95E-06	3.97569303
VAC14	-0.211052228	4.60706273	-4.865305179	2.00E-06	4.96E-06	3.973254811
ZNF593	-0.346968189	2.8891563	-4.864477367	2.01E-06	4.98E-06	3.969570347
ZNF343	-0.25910434	2.89652137	-4.86257222	2.02E-06	5.02E-06	3.961092813
NELFB	-0.298393158	6.35443342	-4.861026738	2.04E-06	5.06E-06	3.954217741
DDX55	-0.271951514	3.1299847	-4.8603904	2.04E-06	5.07E-06	3.951387519
CCDC13	-0.175340906	0.86445804	-4.860130263	2.05E-06	5.08E-06	3.950230605
SELL	0.726992451	3.03067574	4.859643525	2.05E-06	5.09E-06	3.948066059
HLA-DRE	0.876780688	9.04401683	4.858863673	2.06E-06	5.11E-06	3.944598399
ACE2	-0.329744103	1.44471334	-4.858169739	2.07E-06	5.12E-06	3.941513166
CHST7	-0.549679053	3.31163218	-4.858018401	2.07E-06	5.13E-06	3.940840365
TBXT	-0.396601787	0.30875755	-4.8576196	2.07E-06	5.14E-06	3.93906751
RNF181	-0.332988803	7.53830933	-4.856644756	2.08E-06	5.16E-06	3.934734377

GSTM2	-0.514746457	3.06602465	-4.856107201	2.08E-06	5.17E-06	3.932345281
CD80	0.301738485	0.63732476	4.855874238	2.09E-06	5.17E-06	3.931309971
TINF2	-0.31278036	5.79758976	-4.855660473	2.09E-06	5.18E-06	3.930360019
UVRAG	-0.313246706	3.51887373	-4.855044472	2.10E-06	5.19E-06	3.927622759
ARHGAF	-0.417832742	1.19872739	-4.854502961	2.10E-06	5.21E-06	3.925216742
P2RX7	0.419374119	1.52716773	4.852872764	2.12E-06	5.24E-06	3.917974867
STAT1	0.726955857	6.52950143	4.852392535	2.12E-06	5.25E-06	3.915841916
CARHSP	-0.29034136	4.49930664	-4.852348475	2.12E-06	5.25E-06	3.915646228
CLINT1	-0.432423392	5.37745268	-4.851483419	2.13E-06	5.28E-06	3.911804532
DNAJA1	-0.415805819	7.32583754	-4.851226845	2.13E-06	5.28E-06	3.910665199
UNC80	-0.115451535	0.18109095	-4.851226774	2.13E-06	5.28E-06	3.910664886
FES	-0.35531558	4.12314573	-4.850816714	2.14E-06	5.29E-06	3.908844091
WFDC1	-0.2378439	0.77563229	-4.850382272	2.14E-06	5.30E-06	3.906915178
FBXO48	-0.193344495	1.57227713	-4.84953617	2.15E-06	5.32E-06	3.903158911
GSR	-0.317845428	5.8644893	-4.84941457	2.15E-06	5.32E-06	3.902619116
LAGE3	-0.398443614	5.65762147	-4.848424427	2.16E-06	5.34E-06	3.89822416
ZNF629	-0.286722832	4.44491657	-4.848061912	2.16E-06	5.35E-06	3.896615251
TMEM52	-0.27110908	0.90702965	-4.847621075	2.17E-06	5.36E-06	3.89465887
SPIDR	-0.369257557	4.51693342	-4.846647627	2.18E-06	5.39E-06	3.890339341
CDS1	-0.393792141	5.70295034	-4.845531642	2.19E-06	5.41E-06	3.885388218
HIST1H4	-0.579319588	1.60191915	-4.845139635	2.19E-06	5.42E-06	3.883649285
FLOT2	-0.263740148	6.82807117	-4.843985883	2.20E-06	5.45E-06	3.878531944
FBXL12	-0.283110297	4.19308681	-4.842896341	2.22E-06	5.48E-06	3.873700333
ATP2A2	-0.456085501	5.66457747	-4.842879893	2.22E-06	5.48E-06	3.873627402
EIF3B	-0.257782525	5.53288401	-4.842733701	2.22E-06	5.48E-06	3.872979179
NVL	-0.280636143	3.7382057	-4.841946276	2.23E-06	5.50E-06	3.869487976
CD53	0.791147373	4.42714332	4.841808156	2.23E-06	5.50E-06	3.868875641
COL18A1	-0.472701641	6.0074901	-4.841403079	2.23E-06	5.51E-06	3.86707988
SUV39H1	-0.220975489	2.67900268	-4.84130419	2.23E-06	5.51E-06	3.866641508
RASGEF	-0.486337956	2.18517126	-4.840727308	2.24E-06	5.53E-06	3.86408437
PAX1	-0.523857787	0.40915171	-4.840504911	2.24E-06	5.53E-06	3.863098625
ACP6	-0.384179312	3.35868446	-4.840381164	2.24E-06	5.53E-06	3.862550145
RNF144E	-0.553784801	5.90215161	-4.838689769	2.26E-06	5.58E-06	3.855054623
HBB	-0.947724828	5.49133106	-4.838429283	2.26E-06	5.58E-06	3.85390046
CDH4	0.888056402	2.27811302	4.836661515	2.28E-06	5.63E-06	3.846069165
ERICH1	-0.224780329	2.56998966	-4.836391109	2.28E-06	5.63E-06	3.844871467
LRRC14	-0.32590819	3.71472037	-4.836359991	2.28E-06	5.63E-06	3.84473364
GPATCH	-0.295949764	3.3509177	-4.835873178	2.29E-06	5.64E-06	3.842577566
ACOT6	-0.079973586	0.11220752	-4.835851256	2.29E-06	5.64E-06	3.842480479
ABCF2	-0.283138147	5.18906689	-4.835523898	2.29E-06	5.65E-06	3.84103073

ARMC12	-0.24730364	0.6324134	-4.835507118	2.29E-06	5.65E-06	3.840956422
NKD2	0.474746327	1.16339855	4.83531883	2.29E-06	5.66E-06	3.840122602
EXOSC5	-0.320580014	5.19999097	-4.834507888	2.30E-06	5.68E-06	3.836531721
RPS6	-0.320320893	10.8821883	-4.834150361	2.31E-06	5.69E-06	3.834948733
NEK8	-0.310711369	3.68145374	-4.833940226	2.31E-06	5.69E-06	3.834018385
CCR1	0.588899899	2.47260024	4.833422076	2.31E-06	5.70E-06	3.831724484
XDH	0.547649048	0.69323511	4.833206857	2.32E-06	5.71E-06	3.830771749
RRAGB	-0.364462904	4.6407823	-4.833182634	2.32E-06	5.71E-06	3.830664519
ICOS	0.485495462	0.83609914	4.832470961	2.32E-06	5.73E-06	3.827514337
CRHBP	-0.596489837	1.75146996	-4.832141593	2.33E-06	5.73E-06	3.826056536
COPS2	-0.301647773	4.94055438	-4.832101453	2.33E-06	5.73E-06	3.82587888
PNPLA5	0.725704574	1.16370868	4.831794865	2.33E-06	5.74E-06	3.82452199
IWS1	-0.318016451	5.27937706	-4.830162574	2.35E-06	5.78E-06	3.817299036
GNAI3	-0.254645914	3.02402252	-4.829763471	2.35E-06	5.79E-06	3.815533295
RNFT1	-0.390644796	3.4546408	-4.829572371	2.36E-06	5.80E-06	3.81468786
INSYN2E	-0.344187142	0.29954259	-4.829332896	2.36E-06	5.80E-06	3.813628456
GDF7	-0.184606178	0.3735218	-4.829091519	2.36E-06	5.81E-06	3.812560674
STK3	-0.264720869	2.57418963	-4.828655411	2.37E-06	5.82E-06	3.810631578
HIST1H4	-0.331264895	0.62373084	-4.828163859	2.37E-06	5.83E-06	3.808457405
CACNA2	-0.443702877	3.46607369	-4.828012261	2.37E-06	5.83E-06	3.807786911
RNF20	-0.373125896	5.15552144	-4.827436048	2.38E-06	5.85E-06	3.805238575
SBF1	-0.440603556	5.60296914	-4.826015223	2.40E-06	5.89E-06	3.798955977
CNOT9	-0.272264698	4.48357087	-4.824850855	2.41E-06	5.92E-06	3.793808522
PHACTR	-0.348884451	4.35020961	-4.824658583	2.41E-06	5.92E-06	3.792958627
NR3C1	-0.365521037	4.38397068	-4.824087325	2.42E-06	5.94E-06	3.790433665
LRRC36	-0.134653913	0.27163338	-4.822943038	2.43E-06	5.97E-06	3.785376658
CCNB3	-0.129645245	0.3916455	-4.820503442	2.46E-06	6.03E-06	3.774598569
ATP10D	-0.459532533	3.17778375	-4.820016982	2.46E-06	6.05E-06	3.772449941
C15orf41	-0.176653389	1.19474077	-4.819464623	2.47E-06	6.06E-06	3.770010467
FAM50A	-0.260195441	6.69195806	-4.819249215	2.47E-06	6.07E-06	3.769059186
GOPC	-0.368824696	4.97839879	-4.818119834	2.48E-06	6.10E-06	3.764072224
HDAC2	-0.283693841	3.9097253	-4.818028055	2.48E-06	6.10E-06	3.763667002
PDPK1	-0.348834204	3.424999	-4.817482264	2.49E-06	6.11E-06	3.761257357
RPL26	-0.34493668	8.71089488	-4.817029744	2.50E-06	6.12E-06	3.759259678
FBH1	-0.307177852	4.65709202	-4.815956584	2.51E-06	6.15E-06	3.754522763
RNASE2	0.467316986	1.1646974	4.81560097	2.51E-06	6.16E-06	3.752953278
ZBTB16	-0.645353903	2.35984903	-4.815522102	2.51E-06	6.16E-06	3.752605214
PEMT	-0.368961447	6.25886223	-4.813123217	2.54E-06	6.23E-06	3.742020536
RPA1	-0.353560378	5.64003518	-4.812997874	2.54E-06	6.23E-06	3.741467604
SPATS2	-0.295013197	4.41164368	-4.811566885	2.56E-06	6.27E-06	3.735155834

CEBPZ	-0.348418495	5.13445586	-4.811202881	2.56E-06	6.28E-06	3.733550545
PUS7L	-0.295193886	2.36590428	-4.81080271	2.57E-06	6.29E-06	3.731785876
HSPB8	-0.825073174	4.86275258	-4.809326267	2.59E-06	6.34E-06	3.725276123
NCL	-0.32950306	7.8685058	-4.808369819	2.60E-06	6.36E-06	3.721059955
ZNF500	-0.298513007	3.04774012	-4.808284749	2.60E-06	6.36E-06	3.72068499
MYO1F	0.482189122	2.30272075	4.807879654	2.60E-06	6.38E-06	3.718899506
DGKH	0.356504486	1.83371073	4.807738624	2.60E-06	6.38E-06	3.71827794
NMUR2	-0.33984979	0.56410538	-4.807297066	2.61E-06	6.39E-06	3.716331933
RPF2	-0.230910766	4.58330469	-4.806875008	2.62E-06	6.40E-06	3.714472006
TRAF3	-0.336801728	3.44600708	-4.806711552	2.62E-06	6.41E-06	3.713751728
CYTL1	-0.502978966	1.64254532	-4.806611322	2.62E-06	6.41E-06	3.713310065
SAC3D1	-0.326803494	3.3244854	-4.805979435	2.63E-06	6.43E-06	3.710525844
EDEM2	-0.265525268	5.62366327	-4.805925961	2.63E-06	6.43E-06	3.710290244
SCNM1	-0.26482825	4.43905627	-4.804952296	2.64E-06	6.45E-06	3.706000718
EXT2	-0.320988695	5.40192013	-4.804433326	2.64E-06	6.47E-06	3.703714672
UEVLD	-0.330005716	3.48285793	-4.804176139	2.65E-06	6.48E-06	3.702581844
GPBP1L1	-0.322166457	5.90046192	-4.804004173	2.65E-06	6.48E-06	3.701824421
FKBP9	-0.365014248	5.66494571	-4.80340175	2.66E-06	6.50E-06	3.69917122
CYBB	0.726058538	3.76183283	4.803279112	2.66E-06	6.50E-06	3.698631129
MBTPS1	-0.361464499	5.78057834	-4.802681523	2.67E-06	6.52E-06	3.695999546
RNF183	0.563669102	1.09117395	4.802535502	2.67E-06	6.52E-06	3.695356561
SP3	-0.397948259	4.61668639	-4.802464121	2.67E-06	6.52E-06	3.695042252
EPHA6	-0.171714998	0.31314878	-4.802261203	2.67E-06	6.53E-06	3.694148764
TRIM13	-0.286171735	3.69780774	-4.801503352	2.68E-06	6.55E-06	3.690812079
ODF3L1	-0.606225441	1.28897548	-4.801279611	2.68E-06	6.55E-06	3.689827071
POLR3G	-0.252755007	1.32563802	-4.800655545	2.69E-06	6.57E-06	3.687079858
SGMS2	0.749906592	4.59328462	4.800004246	2.70E-06	6.59E-06	3.684213077
ZNF587E	-0.277237731	2.5511602	-4.799894311	2.70E-06	6.59E-06	3.683729215
CATSPE	-0.074531427	0.08804932	-4.798734328	2.71E-06	6.63E-06	3.67862429
TMEM15	0.379109249	0.93725321	4.798400019	2.72E-06	6.64E-06	3.677153237
COL24A1	0.129370874	0.21667185	4.797146245	2.73E-06	6.67E-06	3.671637033
GLE1	-0.286027261	4.98181554	-4.797077813	2.74E-06	6.67E-06	3.671335988
HLA-B	0.65620348	10.4226704	4.79586296	2.75E-06	6.71E-06	3.665992234
SMR3A	-0.444019048	0.52680415	-4.795544401	2.75E-06	6.72E-06	3.664591183
GORASF	-0.327433048	5.94681671	-4.795178421	2.76E-06	6.73E-06	3.662981661
UCP3	-0.260295124	1.19305767	-4.795164335	2.76E-06	6.73E-06	3.662919716
NT5DC4	0.066395441	0.08074931	4.794498821	2.77E-06	6.75E-06	3.659993167
HIST1H3	-0.1251315	0.15325334	-4.794082055	2.77E-06	6.76E-06	3.658160639
NEBL	-0.50334513	4.88872757	-4.792691427	2.79E-06	6.80E-06	3.652046988
YBX2	-0.456945849	1.18349072	-4.791974277	2.80E-06	6.82E-06	3.648894745

RPS11	-0.297805221	11.1672023	-4.791694582	2.80E-06	6.83E-06	3.647665449
DPYSL3	0.86605468	5.9408009	4.790059864	2.82E-06	6.88E-06	3.640481844
TMCC3	0.608460717	4.81065881	4.789957836	2.83E-06	6.88E-06	3.640033557
SPTLC3	-0.41821916	4.08582194	-4.789097863	2.84E-06	6.91E-06	3.636255382
TWNK	-0.288313451	3.23698558	-4.786723075	2.87E-06	6.98E-06	3.625825006
SLC5A4	-0.342630734	0.90146485	-4.786307038	2.87E-06	7.00E-06	3.623998161
PRDX6	-0.279496351	8.21862192	-4.786304708	2.87E-06	7.00E-06	3.623987931
NCAM2	0.251842988	0.49193042	4.785053316	2.89E-06	7.04E-06	3.618493793
SH3BGR	0.403663529	8.08784354	4.784917589	2.89E-06	7.04E-06	3.617897968
UPK3A	-0.628607424	0.88444425	-4.784850992	2.89E-06	7.04E-06	3.61760562
WAS	0.611338053	2.93226808	4.784338494	2.90E-06	7.06E-06	3.615355966
WTAP	-0.309547683	5.43821308	-4.783586444	2.91E-06	7.08E-06	3.61205514
SIRPB2	0.324449373	1.0399822	4.783304534	2.91E-06	7.09E-06	3.610817917
CDKN2B	0.55015196	2.78421047	4.783288276	2.91E-06	7.09E-06	3.610746569
SERINC3	-0.338559254	6.97066398	-4.782587079	2.92E-06	7.11E-06	3.607669491
MYOM1	-0.358302211	1.42595525	-4.782153003	2.93E-06	7.12E-06	3.605764818
LRRC75B	-0.544681992	4.66421354	-4.782087652	2.93E-06	7.12E-06	3.605478076
BLOC1S4	-0.300290831	4.86959924	-4.781124391	2.94E-06	7.15E-06	3.601251965
CST3	-0.360062971	8.3158557	-4.779503885	2.96E-06	7.20E-06	3.594143925
PAPLN	0.687041991	4.66182347	4.778926992	2.97E-06	7.22E-06	3.591613982
C19orf53	-0.308947291	8.03658544	-4.778350407	2.98E-06	7.24E-06	3.589085641
ELAVL1	-0.244118706	5.13992724	-4.77733496	2.99E-06	7.27E-06	3.584633503
TBC1D1(	-0.518379201	3.40234693	-4.777302556	2.99E-06	7.27E-06	3.584491443
OTUD5	-0.273237294	6.33524743	-4.775207081	3.02E-06	7.34E-06	3.575306568
ZBED6CL	-0.364928457	3.4166662	-4.775034069	3.03E-06	7.35E-06	3.574548375
HAS3	-0.709800634	3.2711542	-4.774236667	3.04E-06	7.37E-06	3.571054195
TMEM25	-0.299184848	6.50189952	-4.7737699	3.04E-06	7.39E-06	3.569009071
OXCT2	-0.389026315	0.88550147	-4.773240817	3.05E-06	7.40E-06	3.56669111
CALM2	-0.341731543	7.93798139	-4.772459432	3.06E-06	7.43E-06	3.563268188
WDR66	-0.393773899	2.18375323	-4.772286331	3.06E-06	7.43E-06	3.562509968
HDGF	-0.323142141	7.52430796	-4.772190017	3.06E-06	7.44E-06	3.562088104
CYB561A	-0.305385403	3.14234924	-4.771863091	3.07E-06	7.45E-06	3.560656181
TOM1	-0.282264025	5.21510565	-4.771766953	3.07E-06	7.45E-06	3.560235118
EHD2	0.436953242	6.55086017	4.771169939	3.08E-06	7.47E-06	3.557620484
NTN3	-0.149512744	0.19207363	-4.769955051	3.10E-06	7.51E-06	3.552300697
PLEKHF1	0.445903566	2.42701631	4.769821506	3.10E-06	7.51E-06	3.551715997
PTBP2	-0.352412315	2.44708152	-4.769697273	3.10E-06	7.51E-06	3.551172075
RASGRF	-0.374696658	1.96173835	-4.768845266	3.11E-06	7.54E-06	3.547442128
WWP1	-0.33284581	4.30975051	-4.768350259	3.12E-06	7.56E-06	3.545275326
THAP12	-0.302986578	3.84420836	-4.768130104	3.12E-06	7.57E-06	3.544311697

FAM184E	-0.069902386	0.25780312	-4.768048989	3.12E-06	7.57E-06	3.543956661
YIPF5	-0.373133184	4.99328483	-4.767694948	3.13E-06	7.58E-06	3.542407107
MAP2	0.658860159	4.21220168	4.765984527	3.15E-06	7.64E-06	3.534922359
VAMP8	-0.352806838	8.77820168	-4.765435392	3.16E-06	7.65E-06	3.532519835
DNAH10	-0.088149811	0.19496253	-4.763949588	3.18E-06	7.71E-06	3.526020451
KCNC1	-0.058792133	0.0788536	-4.763624472	3.19E-06	7.72E-06	3.524598514
PTPRC	0.742367896	2.71843475	4.76283094	3.20E-06	7.74E-06	3.521128242
POLR1B	-0.375417279	3.90849156	-4.761952524	3.21E-06	7.77E-06	3.517287322
STAM2	-0.348344284	4.06480972	-4.761881238	3.21E-06	7.77E-06	3.516975645
TIGIT	0.578688044	1.10888219	4.76152873	3.22E-06	7.79E-06	3.515434473
ARHGAP	0.597650139	3.01316395	4.760012823	3.24E-06	7.84E-06	3.508807972
ZNF878	-0.11856285	0.36861811	-4.759850194	3.24E-06	7.84E-06	3.508097177
NSD2	-0.358628663	3.38763272	-4.759543828	3.25E-06	7.85E-06	3.506758208
IBSP	0.423643993	0.50626824	4.758613013	3.26E-06	7.88E-06	3.502690537
ILK	-0.446328933	3.39694296	-4.75834431	3.26E-06	7.89E-06	3.501516427
PLPPR3	0.208885321	0.32080466	4.757087579	3.28E-06	7.94E-06	3.496025819
WNT9A	-0.379636427	2.12575452	-4.757034899	3.28E-06	7.94E-06	3.495795689
COL16A1	0.456265071	4.76134123	4.756515383	3.29E-06	7.96E-06	3.493526319
SLC35D1	-0.388090669	3.47535711	-4.75601615	3.30E-06	7.97E-06	3.491345744
KIF13A	-0.425931183	4.48279778	-4.75598711	3.30E-06	7.97E-06	3.49121891
PRKAA2	-0.367580668	2.31230177	-4.754602944	3.32E-06	8.02E-06	3.485174114
CA14	-0.349433023	1.3014966	-4.753862684	3.33E-06	8.05E-06	3.481941929
SPPL2B	-0.34189213	4.05701295	-4.75371834	3.33E-06	8.05E-06	3.481311732
RAB25	-0.370680307	7.4098104	-4.753429123	3.34E-06	8.06E-06	3.480049076
PAIP1	-0.32798144	5.46066717	-4.753273997	3.34E-06	8.07E-06	3.479371854
RNF39	0.442446599	1.62054844	4.750714914	3.38E-06	8.16E-06	3.468202577
CPA2	-0.085220984	0.12308424	-4.750691665	3.38E-06	8.16E-06	3.468101125
ITGB6	0.641026345	3.81014117	4.750156267	3.39E-06	8.18E-06	3.465764996
YAF2	-0.334892376	2.95902302	-4.749859044	3.39E-06	8.19E-06	3.4644682
PIK3R5	0.436054894	1.46812285	4.74913873	3.40E-06	8.21E-06	3.461325726
RARRES	0.648393807	1.87858487	4.749038771	3.41E-06	8.22E-06	3.460889676
CT62	-0.190362203	0.45018157	-4.747814671	3.42E-06	8.26E-06	3.45555037
PCYOX1	-0.468719228	6.56114222	-4.747682742	3.43E-06	8.26E-06	3.454974991
SLX4IP	-0.264839122	2.59941257	-4.747429894	3.43E-06	8.27E-06	3.453872281
ASAH2B	-0.19940154	1.83688698	-4.747119985	3.43E-06	8.28E-06	3.45252079
KRR1	-0.271883564	3.34845369	-4.746838649	3.44E-06	8.29E-06	3.451293966
DDX54	-0.273406708	5.70996639	-4.746768117	3.44E-06	8.29E-06	3.450986404
METTL3	-0.352719629	4.20021609	-4.744632946	3.47E-06	8.37E-06	3.441677656
NOG	-0.486009615	1.00359638	-4.744527165	3.48E-06	8.38E-06	3.441216572
POPDC3	-0.594019368	0.59726699	-4.74334409	3.49E-06	8.42E-06	3.436060303

MPG	-0.337818889	6.48756295	-4.742635152	3.51E-06	8.45E-06	3.432971008
SS18	-0.272578645	5.63600676	-4.741480848	3.52E-06	8.49E-06	3.427941802
PES1	-0.307347441	5.4623455	-4.74085054	3.53E-06	8.51E-06	3.425196033
FEZ1	-0.293273303	1.30452995	-4.73999163	3.55E-06	8.54E-06	3.421454916
TEX19	-0.059123178	0.08431274	-4.739955736	3.55E-06	8.54E-06	3.421298588
IL10RB	-0.272682311	4.9383516	-4.739797505	3.55E-06	8.55E-06	3.420609452
BABAM1	-0.25071133	5.13518467	-4.739657029	3.55E-06	8.55E-06	3.419997661
TCHH	0.097500507	0.18425866	4.738784741	3.57E-06	8.59E-06	3.416199079
QSOX1	0.563530268	5.33370836	4.738150167	3.58E-06	8.61E-06	3.413436047
AKAP8L	-0.29222713	4.92619025	-4.738128875	3.58E-06	8.61E-06	3.413343343
SLAMF1	0.488983636	0.94317061	4.735998098	3.61E-06	8.69E-06	3.404067919
PPY	-0.586946764	0.39283696	-4.734731722	3.63E-06	8.74E-06	3.398556954
S100A11	0.503203019	10.2476855	4.734173734	3.64E-06	8.76E-06	3.39612911
SAFB	-0.257048875	5.33401465	-4.733218384	3.66E-06	8.80E-06	3.391972885
VPS26C	-0.279369709	3.69218556	-4.73156976	3.69E-06	8.86E-06	3.384802238
SLC37A3	-0.30191652	3.55731036	-4.731565497	3.69E-06	8.86E-06	3.384783697
GPN1	-0.296408705	4.82279932	-4.730519002	3.70E-06	8.90E-06	3.380233087
TGFBRA	-0.354718219	4.28230203	-4.728721969	3.73E-06	8.97E-06	3.372420779
FKBP15	-0.261056237	3.86919736	-4.728042501	3.75E-06	9.00E-06	3.369467553
GCNT4	0.505726639	1.52101519	4.72797686	3.75E-06	9.00E-06	3.369182269
RPL24	-0.35525635	9.75882616	-4.726208548	3.78E-06	9.07E-06	3.361498271
ENPP4	-0.395943361	4.21447388	-4.725344631	3.79E-06	9.11E-06	3.357745094
B3GALT2	-0.520856026	0.57550442	-4.72321283	3.83E-06	9.19E-06	3.348486217
AATK	-0.539270525	1.99875355	-4.722102918	3.85E-06	9.24E-06	3.343667018
BAG2	-0.333571984	2.6199744	-4.721566494	3.86E-06	9.26E-06	3.341338226
MICB	0.41401853	1.82189507	4.720781804	3.87E-06	9.29E-06	3.337932025
ZNF501	-0.314741441	2.03522319	-4.720606588	3.87E-06	9.30E-06	3.337171507
DCTN1	-0.320427806	5.28846801	-4.719949355	3.88E-06	9.32E-06	3.334319033
HCAR2	0.423051978	0.77145387	4.71990111	3.89E-06	9.32E-06	3.334109652
RAD23A	-0.278473383	6.76407826	-4.719523501	3.89E-06	9.34E-06	3.332470947
SNX24	-0.269714882	4.39712388	-4.71935794	3.90E-06	9.35E-06	3.331752496
ABHD5	-0.401076217	4.01824939	-4.71884652	3.90E-06	9.37E-06	3.329533334
DDB1	-0.320115004	6.08917934	-4.718312273	3.91E-06	9.39E-06	3.32721533
MFNG	-0.375069522	3.30847029	-4.718084995	3.92E-06	9.40E-06	3.326229279
CDCP1	0.562774309	4.67260442	4.717858337	3.92E-06	9.40E-06	3.32524596
LST1	0.624941668	3.1399753	4.71783588	3.92E-06	9.40E-06	3.325148536
CCDC28B	-0.425132791	3.29679965	-4.717242476	3.93E-06	9.43E-06	3.32257434
EI24	-0.26570641	5.96284903	-4.717148823	3.93E-06	9.43E-06	3.322168093
ANXA2	0.67391903	7.28595531	4.716654529	3.94E-06	9.45E-06	3.32002408
RECQL5	-0.307928543	4.36305353	-4.715853508	3.96E-06	9.48E-06	3.316550036

DEUP1	-0.149920746	0.37806704	-4.715802369	3.96E-06	9.48E-06	3.31632826
THADA	-0.379589559	3.47968557	-4.715697327	3.96E-06	9.49E-06	3.315872732
GPD1	-0.473058465	1.8587522	-4.715068532	3.97E-06	9.51E-06	3.313146056
SMN1	-0.268502	2.12402783	-4.714740436	3.98E-06	9.52E-06	3.311723436
HOOK3	0.413344785	4.34141808	4.714123541	3.99E-06	9.55E-06	3.309048815
EHBP1L1	0.478874124	4.0892462	4.713979026	3.99E-06	9.55E-06	3.308422296
JRK	-0.326992961	2.33023116	-4.713916432	3.99E-06	9.56E-06	3.308150935
REX1BD	-0.435442137	4.28485539	-4.713579728	4.00E-06	9.57E-06	3.306691293
NUPR1	-0.878774833	6.05224556	-4.71310343	4.01E-06	9.59E-06	3.304626647
ARL8A	-0.241646401	6.53143022	-4.712419666	4.02E-06	9.62E-06	3.301662992
RRP7A	-0.374145869	5.08054308	-4.711882103	4.03E-06	9.64E-06	3.29933327
ACKR2	-0.194938533	0.56617209	-4.711360644	4.04E-06	9.66E-06	3.297073555
TPP2	-0.347116291	4.49899894	-4.710996817	4.05E-06	9.67E-06	3.295497055
NRG1	-0.601513292	1.79337191	-4.710918516	4.05E-06	9.68E-06	3.295157785
PFKL	-0.307141528	5.69285282	-4.710841643	4.05E-06	9.68E-06	3.294824701
KLHL4	-0.182848557	0.34148937	-4.710396203	4.06E-06	9.70E-06	3.292894759
GSKIP	-0.292732912	3.71680369	-4.709940964	4.06E-06	9.72E-06	3.290922515
S100A2	0.895420158	4.59143258	4.708832449	4.09E-06	9.76E-06	3.286120735
GABPB2	-0.266219726	2.70138129	-4.708798646	4.09E-06	9.76E-06	3.285974327
TNFSF13	0.54533899	2.09339378	4.708326844	4.09E-06	9.78E-06	3.283930913
FPR2	0.251858273	0.29907714	4.707522004	4.11E-06	9.82E-06	3.280445478
LYN	0.434318247	4.83124843	4.70727784	4.11E-06	9.83E-06	3.279388202
RPL10	-0.309354619	9.57051093	-4.705089717	4.15E-06	9.92E-06	3.269915281
AASDHP	-0.332991422	4.24767534	-4.703609325	4.18E-06	9.99E-06	3.263508399
MOSPD2	-0.409272453	3.73441711	-4.702740637	4.20E-06	1.00E-05	3.259749657
VIP	-0.308344205	0.76984148	-4.702638237	4.20E-06	1.00E-05	3.25930662
TRIM63	0.590344415	1.30405463	4.701839417	4.22E-06	1.01E-05	3.255850769
GPR135	-0.295653751	1.72790766	-4.701247788	4.23E-06	1.01E-05	3.253291583
RIPOR2	0.610515416	2.14243296	4.701074198	4.23E-06	1.01E-05	3.252540743
HIC1	-0.370355604	1.65846659	-4.6997217	4.26E-06	1.02E-05	3.246691495
GNL1	-0.315395905	5.3335747	-4.699057351	4.27E-06	1.02E-05	3.243818855
CDC73	-0.39420195	4.45667803	-4.696858353	4.31E-06	1.03E-05	3.234312839
SLC39A1	-0.302446035	6.7681545	-4.696417909	4.32E-06	1.03E-05	3.232409303
APOL5	-0.135199383	0.26750984	-4.696241435	4.32E-06	1.03E-05	3.231646648
EMP2	0.537781486	4.7432546	4.695120953	4.35E-06	1.04E-05	3.226804907
OCM	-0.176828831	0.40305667	-4.694995904	4.35E-06	1.04E-05	3.226264612
TAOK3	-0.34129155	3.90573148	-4.694914668	4.35E-06	1.04E-05	3.225913628
TRPM6	-0.164745848	0.27092312	-4.694133514	4.36E-06	1.04E-05	3.222538866
SNRNP7	-0.400747114	6.84314421	-4.693672264	4.37E-06	1.04E-05	3.22054638
GTF3C4	-0.387003539	3.79896062	-4.693492886	4.38E-06	1.04E-05	3.219771557

GLIPR1L	-0.179710471	0.66952289	-4.693066222	4.39E-06	1.04E-05	3.217928681
SLC29A2	-0.337946607	3.94060083	-4.692525646	4.40E-06	1.05E-05	3.215593994
CITED4	0.541314141	4.7866551	4.691506009	4.42E-06	1.05E-05	3.21119091
PRPH2	-0.665428627	1.52169139	-4.690561819	4.44E-06	1.06E-05	3.207114342
PLCB2	0.474147212	2.08858261	4.689806054	4.45E-06	1.06E-05	3.203851806
PIBF1	-0.387440451	4.35988165	-4.688742302	4.47E-06	1.06E-05	3.199260474
VAMP1	-0.571826296	4.34875875	-4.688588465	4.48E-06	1.07E-05	3.198596563
REP15	-0.179091487	0.82092901	-4.688502929	4.48E-06	1.07E-05	3.198227422
INHA	-0.337782822	0.94211489	-4.687759962	4.49E-06	1.07E-05	3.195021313
TTI1	-0.290974667	4.18450477	-4.687420761	4.50E-06	1.07E-05	3.193557706
MYCBPA	-0.230558642	0.62594194	-4.687320348	4.50E-06	1.07E-05	3.193124458
TSHR	-0.541603674	8.08516589	-4.686296554	4.52E-06	1.08E-05	3.188707549
PRICKLE	-0.260820286	0.90563928	-4.686064345	4.53E-06	1.08E-05	3.187705853
KLHL3	-0.520683173	2.40180434	-4.686026363	4.53E-06	1.08E-05	3.187542012
HIST2H2	-0.405101188	1.70650824	-4.685659135	4.53E-06	1.08E-05	3.185957968
RPS27	-0.377157734	10.7685414	-4.684368493	4.56E-06	1.08E-05	3.180391604
RTL8A	-0.245104371	6.79451807	-4.684035486	4.57E-06	1.09E-05	3.178955601
TMEM13	-0.287152778	4.4427686	-4.683775169	4.57E-06	1.09E-05	3.177833113
KIF5A	-0.693231023	1.37047333	-4.683708921	4.57E-06	1.09E-05	3.177547459
AVEN	-0.320508003	4.00879951	-4.682127612	4.61E-06	1.09E-05	3.170730053
PPP6R1	-0.348010101	5.51060358	-4.682064651	4.61E-06	1.09E-05	3.170458651
DHRS1	-0.289369289	4.81143288	-4.680235112	4.65E-06	1.10E-05	3.162573576
SCYL3	-0.30570415	2.9808442	-4.679600567	4.66E-06	1.11E-05	3.159839374
PLD4	0.396524191	1.63712466	4.679251136	4.67E-06	1.11E-05	3.158333838
RPIA	-0.258998527	5.01461996	-4.678738424	4.68E-06	1.11E-05	3.156124972
ZNF565	-0.21326069	1.61751854	-4.678293429	4.69E-06	1.11E-05	3.154208006
VAMP5	-0.543053035	6.43743096	-4.677989647	4.69E-06	1.11E-05	3.152899454
STK38	0.3444984	4.65855911	4.677948546	4.69E-06	1.11E-05	3.152722419
FRK	-0.345269358	0.78589963	-4.677824878	4.70E-06	1.11E-05	3.152189735
CTCF	-0.31883535	5.33660351	-4.677531626	4.70E-06	1.12E-05	3.150926647
RNF34	-0.26631059	4.08502396	-4.677130328	4.71E-06	1.12E-05	3.149198293
PVALB	-0.884942673	1.0736226	-4.676958165	4.72E-06	1.12E-05	3.148456839
MOGS	-0.278662864	5.75752892	-4.676934553	4.72E-06	1.12E-05	3.148355152
RTP3	-0.15287394	0.23489541	-4.675381046	4.75E-06	1.13E-05	3.141665748
AGFG2	-0.296062019	4.05168728	-4.674663736	4.76E-06	1.13E-05	3.138577642
GID8	-0.307904405	5.7107179	-4.674439667	4.77E-06	1.13E-05	3.137613078
ITGA11	0.454347223	1.34417198	4.674316467	4.77E-06	1.13E-05	3.13708275
GUCA1B	-0.285167812	1.03439142	-4.673920404	4.78E-06	1.13E-05	3.135377929
TMEM14	-0.36282079	3.12785363	-4.673134116	4.80E-06	1.14E-05	3.131993774
DAPK2	0.858621098	5.90220984	4.672536129	4.81E-06	1.14E-05	3.129420381

GTF3C2	-0.30881053	5.06429283	-4.671654614	4.83E-06	1.14E-05	3.125627354
GBA	-0.330053816	5.50650358	-4.671315205	4.84E-06	1.15E-05	3.124167088
SLC52A2	-0.287722181	5.18372804	-4.670834087	4.85E-06	1.15E-05	3.122097293
NUP50	-0.310775727	3.75432187	-4.670199232	4.86E-06	1.15E-05	3.119366381
HMGCS2	-0.452343018	0.36848663	-4.6697977	4.87E-06	1.15E-05	3.117639302
GMPR	-0.37913959	3.72369998	-4.669763267	4.87E-06	1.15E-05	3.117491207
PSTPIP1	0.498250297	1.44687329	4.669710107	4.87E-06	1.15E-05	3.117262562
GSG1	-0.053697456	0.09950852	-4.668032843	4.91E-06	1.16E-05	3.110049742
SQOR	-0.284964534	5.41949058	-4.667981155	4.91E-06	1.16E-05	3.109827499
FAAP24	-0.258077663	2.15692286	-4.667916212	4.91E-06	1.16E-05	3.10954827
GFPT1	-0.464148391	5.61138408	-4.667564668	4.92E-06	1.16E-05	3.108036813
CACNA1	-0.129287702	0.35478944	-4.666685433	4.94E-06	1.17E-05	3.104256989
PPIL4	-0.377334728	5.4480915	-4.666107487	4.95E-06	1.17E-05	3.101772727
TMED6	-0.160420381	0.91005241	-4.666007851	4.95E-06	1.17E-05	3.101344475
KRT85	-0.478595309	0.29304366	-4.665737556	4.96E-06	1.17E-05	3.100182741
RAB11FI	-0.30618297	4.87921349	-4.664554901	4.99E-06	1.18E-05	3.095100333
TPK1	-0.226077505	1.71978926	-4.663251986	5.01E-06	1.19E-05	3.089502366
EXOSC6	-0.308009677	4.57783256	-4.663030758	5.02E-06	1.19E-05	3.088551993
TP53TG5	-0.180060609	0.62919545	-4.662229729	5.04E-06	1.19E-05	3.08511117
GABRR3	-0.038233154	0.0295947	-4.662006718	5.04E-06	1.19E-05	3.084153311
PIK3CD	0.527809415	2.92038817	4.661821908	5.05E-06	1.19E-05	3.083359563
TECPR1	-0.272628085	3.83125119	-4.661797804	5.05E-06	1.19E-05	3.083256037
KLC3	0.457034171	1.53552006	4.660555484	5.08E-06	1.20E-05	3.077921047
BOLA2B	-0.178583629	0.66985898	-4.660174866	5.08E-06	1.20E-05	3.076286774
IVNS1AB	-0.353499864	5.41366405	-4.658388718	5.12E-06	1.21E-05	3.068619011
RPS12	-0.383682016	11.3718309	-4.658008699	5.13E-06	1.21E-05	3.066987942
CD302	-0.477655162	2.97008045	-4.657957451	5.13E-06	1.21E-05	3.066767995
GPR87	0.436657338	0.58378889	4.65718522	5.15E-06	1.22E-05	3.063453904
GYPC	-0.461552571	3.43563833	-4.656560906	5.17E-06	1.22E-05	3.060774952
FCN3	-0.523344687	2.69084992	-4.655904196	5.18E-06	1.22E-05	3.057957317
MPV17L	-0.361695144	2.28523657	-4.654776102	5.21E-06	1.23E-05	3.05311797
CCL23	0.411081014	0.90713497	4.651943504	5.27E-06	1.24E-05	3.040970931
KCTD7	-0.350734109	2.83350575	-4.651887006	5.28E-06	1.24E-05	3.040728715
DHX9	-0.390468169	6.33158393	-4.651719207	5.28E-06	1.25E-05	3.040009347
MINDY4	-0.267875321	1.15143733	-4.651334091	5.29E-06	1.25E-05	3.038358403
HS6ST1	-0.356982892	3.96178005	-4.650954534	5.30E-06	1.25E-05	3.036731398
ZSCAN2(	-0.312996266	4.08889136	-4.64989872	5.32E-06	1.25E-05	3.032206155
BRCA2	0.176866758	0.69883248	4.649734525	5.33E-06	1.26E-05	3.031502492
ERMP1	-0.490392902	5.64292794	-4.649660072	5.33E-06	1.26E-05	3.031183425
TMEM22	-0.385949874	3.47093029	-4.64922778	5.34E-06	1.26E-05	3.029330945

YTHDC1	-0.316191299	5.19522664	-4.64868537	5.35E-06	1.26E-05	3.02700678
FNDC11	0.277817334	0.71868549	4.648593452	5.35E-06	1.26E-05	3.026612947
C10orf14	0.371317609	0.64547232	4.64792226	5.37E-06	1.26E-05	3.023737337
CUL3	-0.354354442	4.31538403	-4.64740853	5.38E-06	1.27E-05	3.02153658
NUDT4	-0.361082139	3.99799965	-4.646595169	5.40E-06	1.27E-05	3.018052663
EPB41L5	-0.431267008	3.69675801	-4.646308708	5.41E-06	1.27E-05	3.016825771
ARHGAP	-0.441186732	3.72183471	-4.645743559	5.42E-06	1.28E-05	3.014405463
KLK11	1.4040031	2.31747735	4.645608282	5.43E-06	1.28E-05	3.013826161
ZNF92	-0.344675867	2.91796121	-4.644679478	5.45E-06	1.28E-05	3.009849105
TMEM94	-0.286642643	5.0316846	-4.644455058	5.45E-06	1.28E-05	3.008888255
TAB1	-0.268726064	4.54863913	-4.643662842	5.47E-06	1.29E-05	3.005496727
GDE1	-0.30396517	5.8309459	-4.643428748	5.48E-06	1.29E-05	3.004494646
HDLBP	-0.316874087	6.9505089	-4.64329996	5.48E-06	1.29E-05	3.003943363
ATP8B1	-0.471005843	4.97278564	-4.642952691	5.49E-06	1.29E-05	3.002456934
TGFB2	0.693460496	2.8072376	4.642798023	5.49E-06	1.29E-05	3.001794931
ZNF799	-0.186171159	1.44960944	-4.642780278	5.50E-06	1.29E-05	3.001718983
TSPAN9	-0.393865492	5.24472829	-4.642502909	5.50E-06	1.29E-05	3.000531851
ATAD1	-0.294983852	4.94443015	-4.642161778	5.51E-06	1.30E-05	2.999071905
ZNF45	-0.333448982	3.22543174	-4.642124314	5.51E-06	1.30E-05	2.998911575
KDM4A	-0.362670402	5.06189469	-4.641562246	5.53E-06	1.30E-05	2.996506289
ZNF100	0.447888102	2.60497874	4.641558075	5.53E-06	1.30E-05	2.996488445
DLL3	-0.420647973	0.65527581	-4.641120711	5.54E-06	1.30E-05	2.994616984
PPP2R5I	-0.288838881	4.25164576	-4.639333327	5.58E-06	1.31E-05	2.986970398
CD2	0.877341032	3.24176033	4.638909246	5.59E-06	1.31E-05	2.985156506
ALDH3A2	-0.377673737	6.68387737	-4.638315495	5.61E-06	1.32E-05	2.982617132
NIPAL4	0.339257451	0.66810481	4.637532451	5.63E-06	1.32E-05	2.979268606
HEATR4	-0.138889228	0.62548191	-4.637421058	5.63E-06	1.32E-05	2.978792295
EVI2A	0.610298671	2.52588182	4.637268992	5.63E-06	1.32E-05	2.978142086
IFITM2	-0.449943941	6.33287457	-4.634825849	5.69E-06	1.34E-05	2.967698057
LOXL1	0.577871473	3.31934143	4.634607891	5.70E-06	1.34E-05	2.966766546
PLXNA4	-0.651436883	2.02651045	-4.63458516	5.70E-06	1.34E-05	2.966669403
GRM3	-0.108009878	0.1217374	-4.633846676	5.72E-06	1.34E-05	2.963513561
ODF2	-0.273960924	3.96865516	-4.633686956	5.72E-06	1.34E-05	2.962831067
TRAK1	-0.331842067	4.8714522	-4.633072461	5.74E-06	1.35E-05	2.960205481
SERP2	-0.497308847	2.38816609	-4.632577027	5.75E-06	1.35E-05	2.958088826
TJAP1	-0.273255137	3.94791108	-4.632465973	5.75E-06	1.35E-05	2.957614393
RCAN3	-0.352450953	4.53168844	-4.632023805	5.77E-06	1.35E-05	2.955725505
UPK2	0.476219799	0.86756725	4.630213405	5.81E-06	1.36E-05	2.9479933
GALE	0.737598904	5.32950676	4.62876723	5.85E-06	1.37E-05	2.941818529
LSP1	0.726673571	3.4246455	4.628685454	5.85E-06	1.37E-05	2.941469417

PMM1	-0.277211169	3.62845368	-4.628458122	5.86E-06	1.37E-05	2.940498933
DENR	-0.293683078	5.69543392	-4.628406604	5.86E-06	1.37E-05	2.940279006
PRKAR1.	-0.377473403	7.51090245	-4.628400543	5.86E-06	1.37E-05	2.940253133
KCTD12	0.624654006	5.17461564	4.628387661	5.86E-06	1.37E-05	2.940198141
GNG3	-0.154573664	0.54945784	-4.628168509	5.87E-06	1.37E-05	2.93926263
TRAFD1	-0.282199041	4.85192563	-4.628131331	5.87E-06	1.37E-05	2.93910393
WDR11	-0.349226788	4.1422289	-4.628023033	5.87E-06	1.37E-05	2.938641645
CALHM4	-0.219575143	0.14490532	-4.628004359	5.87E-06	1.37E-05	2.938561934
ACOX3	-0.251222237	3.98603609	-4.626005677	5.92E-06	1.39E-05	2.930031981
COMT	-0.305461626	5.71557904	-4.625982442	5.92E-06	1.39E-05	2.929932839
PLGLB1	-0.090138941	0.23273978	-4.625426347	5.94E-06	1.39E-05	2.927560106
NUP54	-0.308399196	4.51769818	-4.624656689	5.96E-06	1.39E-05	2.924276547
TSPEAR	-0.501580233	1.05396654	-4.624550924	5.96E-06	1.39E-05	2.923825365
NPEPL1	-0.345781074	2.51901066	-4.623649739	5.98E-06	1.40E-05	2.919981335
FUBP1	-0.351136174	5.68105017	-4.623605313	5.99E-06	1.40E-05	2.919791852
TRPM4	-0.284980702	4.08645254	-4.62345059	5.99E-06	1.40E-05	2.919131949
CRBN	-0.26468101	4.251458	-4.622151494	6.02E-06	1.41E-05	2.913591937
RNH1	-0.317579264	6.28700575	-4.621979735	6.03E-06	1.41E-05	2.912859569
ABCA5	-0.337840928	2.71961136	-4.621914763	6.03E-06	1.41E-05	2.912582536
COL3A1	0.963289228	6.71510541	4.621653658	6.04E-06	1.41E-05	2.911469259
DFFB	-0.257973115	2.3463784	-4.621346599	6.05E-06	1.41E-05	2.910160113
PSG1	0.574041674	0.78448747	4.619937222	6.08E-06	1.42E-05	2.904152182
ART5	-0.386615809	0.92677175	-4.619577613	6.09E-06	1.42E-05	2.902619479
CHRDL2	0.189091625	0.33376854	4.618710014	6.12E-06	1.43E-05	2.898922069
GPR3	-0.447474416	1.72201438	-4.618259267	6.13E-06	1.43E-05	2.897001374
FGFR4	-0.214531385	0.60679248	-4.618253491	6.13E-06	1.43E-05	2.896976761
MAGEF1	-0.325711909	7.26794986	-4.615873549	6.19E-06	1.45E-05	2.886838125
CHUK	-0.296481064	3.32315223	-4.615444098	6.21E-06	1.45E-05	2.885009118
TTC8	-0.359866237	4.12693463	-4.614569215	6.23E-06	1.45E-05	2.881283491
TGFBR1	0.584391539	5.19030233	4.61320116	6.27E-06	1.46E-05	2.875458918
RAB38	-0.428395838	4.52041002	-4.612628177	6.28E-06	1.47E-05	2.87301985
TMEM18	-0.367117026	4.90153845	-4.612554491	6.29E-06	1.47E-05	2.8727062
CCSER1	-0.20070155	0.58994397	-4.612135018	6.30E-06	1.47E-05	2.870920777
NAE1	-0.291161824	4.84420213	-4.611473504	6.32E-06	1.47E-05	2.868105417
ELAVL2	-0.108094319	0.25140164	-4.610736748	6.34E-06	1.48E-05	2.864970235
CXorf38	-0.265847722	4.03588585	-4.610702734	6.34E-06	1.48E-05	2.864825504
CSTF2T	-0.335860111	4.67064166	-4.610582855	6.34E-06	1.48E-05	2.864315417
MTSS1	-0.525927158	3.31872488	-4.608614928	6.40E-06	1.49E-05	2.855943447
KIF25	-0.058153179	0.10535521	-4.608554094	6.40E-06	1.49E-05	2.855684693
NKX2-3	-0.471112641	0.57451356	-4.607615467	6.43E-06	1.50E-05	2.851692688

SOS2	-0.357953089	4.59417426	-4.607070356	6.44E-06	1.50E-05	2.849374633
SRSF6	-0.340728039	6.68118819	-4.605818722	6.48E-06	1.51E-05	2.844053005
HSPA12/	-0.536908569	2.77839477	-4.605184498	6.50E-06	1.51E-05	2.841356914
CLEC2B	0.460207111	3.36195289	4.60364161	6.54E-06	1.52E-05	2.834799398
PUS1	-0.229229159	3.22000607	-4.603338316	6.55E-06	1.53E-05	2.83351057
CD27	-0.992679366	3.82409786	-4.602928638	6.56E-06	1.53E-05	2.831769782
IKZF1	0.510759357	1.43369686	4.601935834	6.59E-06	1.53E-05	2.827551743
ANKRD5	-0.429375347	1.78279854	-4.600742468	6.63E-06	1.54E-05	2.822482619
SMNDC1	-0.273805938	4.09751417	-4.600189421	6.64E-06	1.55E-05	2.820133788
INSRR	0.072011011	0.06845481	4.600166453	6.64E-06	1.55E-05	2.820036244
USP28	-0.322251719	3.66797188	-4.5998983	6.65E-06	1.55E-05	2.81889747
CCDC22	-0.282314147	5.11655586	-4.599583174	6.66E-06	1.55E-05	2.817559287
ARID3B	-0.27470703	1.73701507	-4.599314686	6.67E-06	1.55E-05	2.816419211
SLC35B4	-0.274105607	2.79775113	-4.598335296	6.70E-06	1.56E-05	2.812260926
TMEM52	0.194910536	0.51376754	4.597480423	6.72E-06	1.56E-05	2.808631931
FYB1	0.616637572	2.21356319	4.597252996	6.73E-06	1.56E-05	2.80766658
IL1RAPL	-0.171703476	0.12896344	-4.596242286	6.76E-06	1.57E-05	2.803376962
ASPDH	-0.257627879	0.63949336	-4.596138524	6.76E-06	1.57E-05	2.802936624
NMUR1	-0.222844443	0.6027104	-4.595753217	6.77E-06	1.57E-05	2.801301559
ICAM3	0.45237129	0.9987641	4.595510261	6.78E-06	1.58E-05	2.800270623
DLL1	-0.525432062	3.36662039	-4.594728748	6.80E-06	1.58E-05	2.79695475
GOLT1B	-0.307517231	4.654386	-4.594285113	6.82E-06	1.58E-05	2.795072666
CCL28	-0.414204646	3.94062057	-4.593754164	6.83E-06	1.59E-05	2.792820362
ZNF821	-0.322511806	2.62018958	-4.591766024	6.89E-06	1.60E-05	2.784388567
PPP4R3I	-0.331312005	4.95944012	-4.591566445	6.90E-06	1.60E-05	2.783542315
TDRP	-0.362803341	5.51822553	-4.591218295	6.91E-06	1.60E-05	2.782066167
MGP	-0.596496509	7.85344315	-4.590304859	6.94E-06	1.61E-05	2.778193669
BIN2	0.525865454	2.03390983	4.589947984	6.95E-06	1.61E-05	2.776680882
CDC27	-0.358942997	5.3338653	-4.589916733	6.95E-06	1.61E-05	2.776548415
ZC3H7B	-0.462776782	5.60385681	-4.588234845	7.00E-06	1.63E-05	2.769420295
WWC1	-0.36216201	4.96503607	-4.587936117	7.01E-06	1.63E-05	2.768154472
TRPM7	-0.356630681	3.47207197	-4.587392664	7.03E-06	1.63E-05	2.765851826
VAV1	0.546795959	2.08697696	4.586603282	7.05E-06	1.64E-05	2.762507582
MBOAT1	0.52576244	3.64486266	4.586490534	7.06E-06	1.64E-05	2.762029958
FXR1	-0.26370545	4.98169311	-4.586305452	7.06E-06	1.64E-05	2.761245938
TMCC2	-0.395642299	2.11861963	-4.586065657	7.07E-06	1.64E-05	2.760230191
FBXO7	-0.36195996	6.1741535	-4.584905082	7.11E-06	1.65E-05	2.755314745
COPB1	-0.374938779	6.35468433	-4.584615082	7.12E-06	1.65E-05	2.75408666
CTSS	0.589274914	6.24356561	4.583922651	7.14E-06	1.65E-05	2.751154627
NOL9	-0.275087999	3.18312211	-4.583834237	7.14E-06	1.65E-05	2.750780274

RPL37A	-0.343991202	8.34866569	-4.583727672	7.14E-06	1.66E-05	2.750329077
ZNF33B	-0.313972907	3.99224692	-4.583537892	7.15E-06	1.66E-05	2.749525568
B3GNTL1	-0.200067203	1.66066879	-4.58223525	7.19E-06	1.67E-05	2.744011066
ADHFE1	-0.330123015	2.76370413	-4.582215937	7.19E-06	1.67E-05	2.743929319
GJD3	-0.274357547	0.3252665	-4.581983183	7.20E-06	1.67E-05	2.742944143
KCNJ14	-0.231789959	1.10462808	-4.581150077	7.23E-06	1.67E-05	2.7394182
SLC9A9	-0.380833837	1.97024653	-4.578555787	7.31E-06	1.69E-05	2.728441908
EYA3	-0.310334794	3.9530012	-4.577024716	7.36E-06	1.70E-05	2.721966508
ASPHD2	0.485567021	2.07864535	4.576909606	7.36E-06	1.70E-05	2.721479747
COG6	-0.354325887	3.57851977	-4.576787991	7.37E-06	1.70E-05	2.720965487
LANCL1	-0.36054413	4.67651893	-4.575550694	7.41E-06	1.71E-05	2.715734124
GBX2	0.311852201	0.31524644	4.574394398	7.44E-06	1.72E-05	2.710846324
CYTH2	-0.295851976	5.24077197	-4.573545068	7.47E-06	1.73E-05	2.707256774
STPG2	-0.079583848	0.23008191	-4.573195169	7.48E-06	1.73E-05	2.705778149
CLRN3	0.239463077	0.48675377	4.572648886	7.50E-06	1.73E-05	2.703469824
FAM111A	0.434671421	4.53843737	4.571737102	7.53E-06	1.74E-05	2.699617591
HP1BP3	-0.347035571	6.21418775	-4.570453163	7.57E-06	1.75E-05	2.694194134
NR2E1	-0.249013129	0.15949307	-4.570115709	7.59E-06	1.75E-05	2.692768917
CTSK	0.715013435	4.79258036	4.569892104	7.59E-06	1.76E-05	2.69182458
MAP1LC3	-0.270997755	6.0050754	-4.568750614	7.63E-06	1.76E-05	2.687004424
KRT78	-0.457362494	0.66594485	-4.568743159	7.63E-06	1.76E-05	2.686972951
TEF	-0.477230589	5.77401135	-4.56837866	7.64E-06	1.77E-05	2.685434003
ENTPD2	0.775978979	2.0423092	4.567969366	7.66E-06	1.77E-05	2.683706047
NFKBIA	-0.416436549	6.87402955	-4.566693851	7.70E-06	1.78E-05	2.678321937
FST	0.456331123	0.96189769	4.566628101	7.70E-06	1.78E-05	2.678044433
NPM2	-0.510762703	4.9033696	-4.565960006	7.73E-06	1.78E-05	2.675224863
MYH15	-0.096545096	0.1184402	-4.565946123	7.73E-06	1.78E-05	2.675166275
PCP4L1	-0.688213125	1.49994382	-4.565786501	7.73E-06	1.79E-05	2.674492675
SEPTIN7	-0.303057519	5.15954778	-4.565243224	7.75E-06	1.79E-05	2.672200209
EMP3	0.554812375	6.25270706	4.564817832	7.77E-06	1.79E-05	2.670405345
CHMP4A	-0.281377929	2.51398218	-4.563440853	7.81E-06	1.80E-05	2.6645964
MFSD4A	0.459433759	1.92331488	4.56338435	7.81E-06	1.80E-05	2.664358067
PCBP1	-0.43495565	8.04876235	-4.563073877	7.83E-06	1.81E-05	2.663048521
MS4A15	0.254560622	0.24695753	4.562259532	7.85E-06	1.81E-05	2.659614049
CDKN2A	-0.346151872	4.49479847	-4.562237487	7.85E-06	1.81E-05	2.659521078
TUBE1	-0.35769499	2.80939483	-4.562092201	7.86E-06	1.81E-05	2.658908398
C10orf82	-0.57966968	1.50127358	-4.562048393	7.86E-06	1.81E-05	2.658723658
ZNF618	-0.385758536	3.19516245	-4.56157894	7.88E-06	1.82E-05	2.656744071
PCMTD2	-0.358071596	4.93420565	-4.561199188	7.89E-06	1.82E-05	2.655142862
PDE11A	-0.296120986	0.62291453	-4.560795688	7.90E-06	1.82E-05	2.653441642

TMPRSS	0.099637862	0.1066341	4.559698443	7.94E-06	1.83E-05	2.648816132
PSMD2	-0.311519448	6.46599876	-4.559562383	7.95E-06	1.83E-05	2.648242627
MFAP2	0.874546104	3.49443195	4.559420794	7.95E-06	1.83E-05	2.647645835
ERGIC1	-0.330972798	5.62527945	-4.556968086	8.04E-06	1.85E-05	2.637310231
CARNMT	-0.335002129	4.30974172	-4.555859032	8.08E-06	1.86E-05	2.632638278
CABP1	-0.168070517	0.4333603	-4.554958689	8.11E-06	1.87E-05	2.628846241
MMP10	0.274709706	0.30412864	4.55421948	8.14E-06	1.87E-05	2.625733337
ZC3H12A	0.495075414	2.82722423	4.553632931	8.16E-06	1.88E-05	2.623263613
FAM131C	-0.276703744	0.96949032	-4.553191572	8.17E-06	1.88E-05	2.621405401
TDRD3	-0.230394056	3.51187511	-4.552995762	8.18E-06	1.88E-05	2.620581053
RBM10	-0.256694582	6.04243601	-4.55245818	8.20E-06	1.89E-05	2.618318012
ZNF200	-0.221260409	2.00920701	-4.552066607	8.21E-06	1.89E-05	2.616669766
NHSL2	0.530001999	2.0986099	4.552042413	8.21E-06	1.89E-05	2.616567931
SNX14	-0.302393347	5.04358327	-4.552040843	8.21E-06	1.89E-05	2.616561322
DCAF6	-0.31144618	4.95827371	-4.551329377	8.24E-06	1.90E-05	2.613566876
ZNF502	-0.319961085	3.18501262	-4.550572117	8.27E-06	1.90E-05	2.610380122
LRRC8C	-0.324563139	2.27616544	-4.550283126	8.28E-06	1.90E-05	2.609164089
NIPA2	-0.450631821	4.53055498	-4.550040775	8.29E-06	1.91E-05	2.608144362
FLOT1	-0.248277985	7.73348258	-4.54964843	8.30E-06	1.91E-05	2.606493612
ALKBH8	-0.285739473	3.02162411	-4.548477	8.34E-06	1.92E-05	2.601565662
NAT14	-0.465109594	5.1016973	-4.547109913	8.39E-06	1.93E-05	2.595815985
KIF26A	-0.348451274	1.47599368	-4.546440799	8.42E-06	1.93E-05	2.593002373
FAM210B	-0.247995437	5.88984115	-4.546057947	8.43E-06	1.94E-05	2.591392642
SIGIRR	-0.357997192	5.03205749	-4.545192821	8.47E-06	1.94E-05	2.587755584
CBLN1	1.116140847	3.13144099	4.545087768	8.47E-06	1.95E-05	2.587313973
KLHDC4	-0.2428197	3.26818157	-4.544975445	8.47E-06	1.95E-05	2.586841812
NAPSA	0.870180946	2.38077377	4.544001553	8.51E-06	1.95E-05	2.582748361
MAMDC4	-0.396294239	2.8005578	-4.543657729	8.52E-06	1.96E-05	2.581303384
CCDC17	-0.290503909	1.04785299	-4.542816082	8.55E-06	1.96E-05	2.577766613
SLC41A2	-0.385836581	3.62324891	-4.542166459	8.58E-06	1.97E-05	2.575037145
LAIR1	0.514253767	2.17615148	4.542138824	8.58E-06	1.97E-05	2.574921038
BID	0.491579765	4.33893431	4.541887187	8.59E-06	1.97E-05	2.573863853
SERINC1	-0.409645727	8.2408568	-4.541708913	8.60E-06	1.97E-05	2.57311491
SLC30A6	-0.352357938	4.19055853	-4.541687554	8.60E-06	1.97E-05	2.573025183
MUC3A	-0.248280188	0.63025749	-4.541376018	8.61E-06	1.97E-05	2.571716465
AZU1	0.358210078	0.46728043	4.541212066	8.62E-06	1.98E-05	2.571027752
TMEM10	-0.292495556	3.32910331	-4.540690949	8.63E-06	1.98E-05	2.568838852
C2orf49	-0.309771566	3.75119116	-4.540555442	8.64E-06	1.98E-05	2.568269702
TENT5C	-0.491762595	4.46741596	-4.539457286	8.68E-06	1.99E-05	2.563657817
RPL9	-0.3283833	8.68295226	-4.539325383	8.69E-06	1.99E-05	2.563103935

BICRA	-0.267352929	2.78779176	-4.538911047	8.70E-06	1.99E-05	2.561364152
FBXW2	-0.318695659	4.20019853	-4.538489321	8.72E-06	2.00E-05	2.559593478
RELL2	-0.304194594	1.62667562	-4.538209487	8.73E-06	2.00E-05	2.558418636
UBP1	-0.313736606	4.51743718	-4.5377729	8.75E-06	2.00E-05	2.556585813
SP6	-0.442234737	1.07538673	-4.537460459	8.76E-06	2.01E-05	2.555274253
ZW10	-0.300220525	3.7830815	-4.536192354	8.81E-06	2.02E-05	2.549951812
WDR91	-0.290675797	2.9843696	-4.536079881	8.81E-06	2.02E-05	2.549479805
JCHAIN	1.659125638	4.65271583	4.535516593	8.83E-06	2.02E-05	2.547116051
PRPF18	-0.287334654	4.48826707	-4.534835034	8.86E-06	2.03E-05	2.544256328
USP10	-0.325903321	5.30611758	-4.534805428	8.86E-06	2.03E-05	2.544132111
ELOVL7	0.547195279	4.59446393	4.534491651	8.87E-06	2.03E-05	2.542815677
UGCG	0.366837845	4.16325081	4.532228564	8.96E-06	2.05E-05	2.533323313
F10	-0.371674796	1.1771959	-4.532123847	8.97E-06	2.05E-05	2.532884185
ZNF250	-0.304237796	3.27099536	-4.531685295	8.98E-06	2.06E-05	2.531045209
BICDL1	-0.47143523	3.58840749	-4.531367302	9.00E-06	2.06E-05	2.529711871
CIDEB	-0.151391697	0.46226326	-4.531254279	9.00E-06	2.06E-05	2.529237985
ACSM1	-0.333724576	1.48165776	-4.531252313	9.00E-06	2.06E-05	2.52922974
CCNY	-0.284205056	5.64184911	-4.531053286	9.01E-06	2.06E-05	2.528395285
SRGAP3	0.353303028	2.11023225	4.530467491	9.03E-06	2.06E-05	2.525939404
NUCKS1	-0.305346466	7.5356156	-4.529768359	9.06E-06	2.07E-05	2.52300873
FRS3	-0.283310487	3.38173885	-4.528999191	9.09E-06	2.08E-05	2.519784915
ATP6V1(	-0.302189112	5.39531539	-4.528855807	9.09E-06	2.08E-05	2.519184
MAK	-0.196941717	0.71532035	-4.528618464	9.10E-06	2.08E-05	2.518189345
CUL4A	-0.303540018	4.90476001	-4.528525405	9.11E-06	2.08E-05	2.517799369
FTO	-0.321220437	3.65683133	-4.528334142	9.12E-06	2.08E-05	2.51699787
LTBP2	0.56729183	6.59485604	4.52715843	9.16E-06	2.09E-05	2.512071623
FOXA3	0.310990828	0.39664165	4.526434117	9.19E-06	2.10E-05	2.509037285
SPPL2A	-0.31483293	4.36316467	-4.526348581	9.20E-06	2.10E-05	2.50867898
ARMC1	-0.252293758	4.609368	-4.524917959	9.25E-06	2.11E-05	2.502687043
STAM	0.440084678	4.91001661	4.524612353	9.27E-06	2.12E-05	2.501407268
VIPAS39	-0.2550384	4.43083075	-4.524070045	9.29E-06	2.12E-05	2.499136444
F2RL3	-0.727682878	3.41358264	-4.523321176	9.32E-06	2.13E-05	2.496001066
TAP1	0.55658148	5.28479956	4.522785425	9.34E-06	2.13E-05	2.493758243
EXOC2	-0.308479255	4.51815353	-4.522016227	9.37E-06	2.14E-05	2.490538536
DUS3L	-0.27235656	4.06561855	-4.520792733	9.42E-06	2.15E-05	2.485418197
ZNHIT6	-0.286213584	3.5203093	-4.520758742	9.42E-06	2.15E-05	2.485275961
CCDC69	-0.539492138	3.64526964	-4.520427569	9.44E-06	2.15E-05	2.48389021
TRIM37	-0.348857187	3.91680392	-4.519060203	9.49E-06	2.17E-05	2.478169555
TNFRSF:	0.447458252	2.19569314	4.518905758	9.50E-06	2.17E-05	2.477523495
RUNDC3	-0.226640695	1.02647042	-4.518789615	9.50E-06	2.17E-05	2.477037667

TRIM47	0.375994343	5.16006064	4.518662096	9.51E-06	2.17E-05	2.476504269
LRRFIP1	-0.295291409	4.80014634	-4.517678985	9.55E-06	2.18E-05	2.472392441
MKRN1	-0.260631316	6.00391547	-4.516774819	9.59E-06	2.19E-05	2.468611474
LANCL3	-0.205058435	0.46598001	-4.516623291	9.59E-06	2.19E-05	2.46797789
SF1	-0.296421427	6.79968535	-4.516419904	9.60E-06	2.19E-05	2.467127496
ZNF793	-0.280750748	1.85414755	-4.516359994	9.61E-06	2.19E-05	2.466877005
ENTPD6	-0.24997049	5.01750479	-4.516306964	9.61E-06	2.19E-05	2.466655286
CASC3	-0.331839228	5.72356132	-4.515931101	9.62E-06	2.19E-05	2.465083863
ZNF585E	-0.268374981	3.2105468	-4.515618809	9.64E-06	2.19E-05	2.463778303
GET4	-0.20972143	1.49036084	-4.515500559	9.64E-06	2.20E-05	2.463283968
ATXN7L1	-0.248249732	1.98880261	-4.514707142	9.68E-06	2.20E-05	2.459967448
LATS2	-0.417188009	4.36228066	-4.514159426	9.70E-06	2.21E-05	2.45767826
TXK	0.270559881	0.92293836	4.513860528	9.71E-06	2.21E-05	2.456429113
OGG1	-0.297591707	3.29216818	-4.513762942	9.72E-06	2.21E-05	2.456021296
RGS3	-0.34087807	4.03067652	-4.5132436	9.74E-06	2.22E-05	2.453851081
OLFM4	-0.383897878	0.39158432	-4.511821285	9.80E-06	2.23E-05	2.447908621
ASPG	-0.247108701	0.476451	-4.511716325	9.80E-06	2.23E-05	2.447470162
APOL2	0.423273616	4.94408142	4.511299457	9.82E-06	2.23E-05	2.445728817
GLS2	0.281392523	1.03297361	4.511174745	9.83E-06	2.23E-05	2.445207896
ANAPC4	-0.256056982	3.39352649	-4.510409414	9.86E-06	2.24E-05	2.44201138
EFTUD2	-0.259941386	4.63540017	-4.510166552	9.87E-06	2.24E-05	2.440997126
QTRT2	-0.294072789	2.91603305	-4.509892028	9.88E-06	2.25E-05	2.439850704
DYSF	-0.444719023	4.27115288	-4.509701776	9.89E-06	2.25E-05	2.439056238
ST13	-0.349856879	7.60598839	-4.50861799	9.94E-06	2.26E-05	2.434531047
SPP1	1.094543623	4.91197946	4.508606321	9.94E-06	2.26E-05	2.434482329
SMG7	-0.260166487	5.04387428	-4.508451209	9.94E-06	2.26E-05	2.433834758
LRRC56	-0.463602973	2.65129221	-4.508377515	9.95E-06	2.26E-05	2.433527103
KCNJ16	-0.564913454	7.68850619	-4.508309601	9.95E-06	2.26E-05	2.433243582
UTP23	-0.322132065	3.45836451	-4.507681392	9.98E-06	2.27E-05	2.430621164
PKDCC	-0.435269214	1.55287419	-4.507672648	9.98E-06	2.27E-05	2.430584664
TMEM88	0.337068215	0.48017494	4.507489531	9.99E-06	2.27E-05	2.429820315
SH2D2A	0.487742681	1.34700991	4.506941455	1.00E-05	2.27E-05	2.427532743
WNT2	0.267866978	0.32050256	4.506676339	1.00E-05	2.27E-05	2.426426278
BTNL3	-0.103646518	0.11519204	-4.505016066	1.01E-05	2.29E-05	2.419498392
TMEM25	-0.308312826	4.36195172	-4.504485487	1.01E-05	2.30E-05	2.417284882
KIF3B	-0.32180412	5.12629306	-4.503560744	1.02E-05	2.30E-05	2.413427498
TMEM16	-0.29796823	2.50507507	-4.503286006	1.02E-05	2.31E-05	2.412281616
WDR7	-0.28466156	2.74705529	-4.503021155	1.02E-05	2.31E-05	2.411177022
EIF3G	-0.304949702	7.00705621	-4.501153578	1.03E-05	2.33E-05	2.403389654
GRAMD2	-0.543826549	2.01114495	-4.499648961	1.03E-05	2.34E-05	2.397117745

GLB1L2	-0.474139999	4.38265727	-4.499243647	1.04E-05	2.35E-05	2.395428524
TWSG1	-0.420975867	5.6137259	-4.498090595	1.04E-05	2.36E-05	2.390623673
CCR2	0.530246705	1.54988979	4.497330436	1.04E-05	2.37E-05	2.387456613
NECTIN2	-0.341040326	6.5283304	-4.49685923	1.05E-05	2.37E-05	2.38549365
CHIT1	1.000706481	2.5212812	4.494269395	1.06E-05	2.40E-05	2.374707974
RANGRF	-0.344410858	4.61049509	-4.49341821	1.06E-05	2.41E-05	2.37116427
LAMP1	-0.316389327	7.75354882	-4.49282388	1.06E-05	2.41E-05	2.368690262
NCAPH2	-0.269386012	5.22771732	-4.491194634	1.07E-05	2.43E-05	2.361909649
C5orf22	-0.279263788	3.82778816	-4.491090146	1.07E-05	2.43E-05	2.361474862
SEC23IP	-0.337608512	4.23875549	-4.491066671	1.07E-05	2.43E-05	2.361377181
PTCH2	-0.312494858	1.211255	-4.490767496	1.07E-05	2.43E-05	2.360132332
SERTAD	-0.30462618	5.48613611	-4.489998986	1.08E-05	2.44E-05	2.356934929
CDK1	-0.336721143	2.32417295	-4.4896916	1.08E-05	2.44E-05	2.35565617
GPR158	-0.28863969	0.3835639	-4.488785221	1.08E-05	2.45E-05	2.351885982
JPH3	0.319800018	0.44369079	4.488275122	1.09E-05	2.46E-05	2.349764451
RASGRF	0.201918196	0.76366598	4.487422204	1.09E-05	2.47E-05	2.346217575
PFKP	-0.420021106	6.43096203	-4.486450044	1.09E-05	2.48E-05	2.342175532
ZFP69B	0.337880598	1.64505147	4.486386444	1.09E-05	2.48E-05	2.34191112
NCOA7	0.583689935	4.0302985	4.486109443	1.10E-05	2.48E-05	2.340759559
EHMT1	-0.302007083	3.55091481	-4.485159173	1.10E-05	2.49E-05	2.336809507
TBL3	-0.23213094	4.4093423	-4.484471443	1.10E-05	2.50E-05	2.33395122
CCDC12	-0.262397464	2.07440059	-4.483988334	1.11E-05	2.50E-05	2.331943584
PIP4P1	-0.26045357	4.85287111	-4.483481798	1.11E-05	2.51E-05	2.329838795
TMEM13	-0.816792745	2.28890655	-4.483087617	1.11E-05	2.51E-05	2.328201009
HOXD1	0.378352759	0.47923847	4.479899857	1.13E-05	2.54E-05	2.31496068
FOXN3	0.35070625	3.26998118	4.479122268	1.13E-05	2.55E-05	2.311732192
SLC35C2	-0.263160028	5.07808428	-4.478155074	1.13E-05	2.56E-05	2.307717144
GPR107	-0.383332806	5.59415806	-4.477274282	1.14E-05	2.57E-05	2.304061419
FUS	-0.322171252	6.39246349	-4.476638853	1.14E-05	2.58E-05	2.301424454
CORO1A	0.675653803	4.14989977	4.476282632	1.14E-05	2.58E-05	2.299946313
PDK4	-0.994352157	7.03930962	-4.47558699	1.15E-05	2.59E-05	2.29706003
CHD7	-0.324608781	2.31761028	-4.475250959	1.15E-05	2.59E-05	2.295665946
MIF	-0.369809795	6.81841145	-4.474932903	1.15E-05	2.60E-05	2.294346515
VIM	-0.352416889	10.3069124	-4.474557002	1.15E-05	2.60E-05	2.292787221
RPL29	-0.284898462	9.65919611	-4.474279549	1.15E-05	2.60E-05	2.291636378
MOB1B	-0.307351191	3.86356486	-4.474105281	1.15E-05	2.61E-05	2.290913565
P2RY4	0.150617225	0.30790139	4.474038296	1.15E-05	2.61E-05	2.290635739
GCNT3	-0.461535029	0.95206679	-4.473958966	1.16E-05	2.61E-05	2.29030671
SIN3A	-0.333468403	4.4223066	-4.473866383	1.16E-05	2.61E-05	2.289922726
CLHC1	-0.301155916	1.90397263	-4.473857445	1.16E-05	2.61E-05	2.289885655

NRP2	0.695225251	4.73021989	4.473307269	1.16E-05	2.61E-05	2.287603953
AQP3	0.577071456	1.76431809	4.472408418	1.16E-05	2.62E-05	2.283876729
GMFG	-0.447737055	4.84785229	-4.471961143	1.17E-05	2.63E-05	2.282022274
NXT2	-0.273385818	3.3284538	-4.471904882	1.17E-05	2.63E-05	2.281789021
C11orf45	-0.264315645	1.68558839	-4.471315851	1.17E-05	2.63E-05	2.279347093
TRUB1	-0.373934029	4.26597413	-4.471151788	1.17E-05	2.64E-05	2.278666993
GPR182	-0.137074518	0.20647666	-4.470912994	1.17E-05	2.64E-05	2.277677144
ANPEP	0.577158859	2.61588278	4.470598118	1.17E-05	2.64E-05	2.276371985
SEL1L	-0.42468576	5.2728482	-4.470149766	1.17E-05	2.65E-05	2.274513709
CCSAP	0.274559131	2.22226804	4.470061378	1.18E-05	2.65E-05	2.274147389
ATF6B	-0.328697663	6.6058582	-4.469896505	1.18E-05	2.65E-05	2.273464093
USP51	-0.345191774	2.92353659	-4.468502312	1.18E-05	2.66E-05	2.2676869
ZNF777	-0.252082527	4.47792984	-4.467649923	1.19E-05	2.67E-05	2.264155571
PCNP	-0.33040093	7.03292316	-4.466840262	1.19E-05	2.68E-05	2.260801787
NCDN	-0.352461553	5.18721127	-4.46635764	1.19E-05	2.69E-05	2.258802915
BCL2L2-I	-0.111476917	0.33635841	-4.465068378	1.20E-05	2.70E-05	2.253464094
EYA1	-0.120535064	0.16984191	-4.46499284	1.20E-05	2.70E-05	2.253151332
COL4A2	-0.485911802	6.99742304	-4.464943806	1.20E-05	2.70E-05	2.252948312
CIAO1	-0.286901054	5.78748519	-4.464834479	1.20E-05	2.70E-05	2.252495662
APEX2	-0.230193672	4.49240081	-4.464827424	1.20E-05	2.70E-05	2.252466454
ERI2	-0.276802	2.31173837	-4.464569461	1.20E-05	2.71E-05	2.251398436
FKBP7	-0.328644339	3.1972807	-4.463948084	1.21E-05	2.71E-05	2.24882604
ZNF853	-0.372378636	4.14579288	-4.4627536	1.21E-05	2.73E-05	2.24388193
FERMT3	0.588581275	3.57402435	4.462404339	1.21E-05	2.73E-05	2.242436511
TOB1	-0.543646831	5.96023402	-4.461616331	1.22E-05	2.74E-05	2.239175692
MOCS3	-0.256181175	2.97678995	-4.460791239	1.22E-05	2.75E-05	2.235761942
XKRX	0.81271591	2.50101464	4.460778336	1.22E-05	2.75E-05	2.235708563
TOMM20	-0.319231347	8.03009422	-4.45906177	1.23E-05	2.77E-05	2.228608164
ZNF101	-0.23466804	2.38037286	-4.458432337	1.24E-05	2.78E-05	2.226005167
VGLL3	0.318551847	0.69918484	4.457085861	1.24E-05	2.79E-05	2.220437916
COL6A3	0.730549408	3.83422478	4.455613139	1.25E-05	2.81E-05	2.214350328
KCNH1	0.069251269	0.13077577	4.455522812	1.25E-05	2.81E-05	2.213977011
G0S2	1.007584872	4.82365102	4.455233692	1.25E-05	2.81E-05	2.212782139
MAF	-0.37993138	5.08866082	-4.454548908	1.26E-05	2.82E-05	2.209952332
LIN52	-0.22844	3.13790735	-4.454348465	1.26E-05	2.82E-05	2.209124089
TP63	0.518105859	0.88839448	4.453768645	1.26E-05	2.83E-05	2.206728424
MAP3K8	-0.337732225	2.67206653	-4.453762888	1.26E-05	2.83E-05	2.206704636
RAB39B	-0.436931725	2.36657596	-4.452834153	1.27E-05	2.84E-05	2.2028679
PIK3C3	-0.277860312	3.05299531	-4.452730259	1.27E-05	2.84E-05	2.202438743
SHC3	0.483325907	1.86137052	4.452679983	1.27E-05	2.84E-05	2.202231067

CCR6	0.082445402	0.10963575	4.45045388	1.28E-05	2.87E-05	2.193037782
DHCR7	-0.391256221	4.95198146	-4.450065057	1.28E-05	2.87E-05	2.191432438
GNLY	0.818200008	2.86114891	4.44951152	1.28E-05	2.88E-05	2.189147239
FAM83A	0.600488154	1.16721261	4.448578314	1.29E-05	2.89E-05	2.185295184
CAPN8	0.336093323	0.61448565	4.447991494	1.29E-05	2.90E-05	2.182873282
TRIL	-0.456368594	3.53510754	-4.447429895	1.30E-05	2.91E-05	2.18055573
PSG4	0.283466117	0.21895269	4.447298629	1.30E-05	2.91E-05	2.18001407
RPGR	-0.278479624	2.15807451	-4.447117299	1.30E-05	2.91E-05	2.179265848
ANXA3	0.755834222	5.13353523	4.446230676	1.30E-05	2.92E-05	2.175607744
CCDC88	0.466221786	2.12394472	4.445821891	1.31E-05	2.92E-05	2.173921358
PLIN1	-0.353678249	0.77009301	-4.44549657	1.31E-05	2.93E-05	2.172579384
VCX3B	-0.084278978	0.13638968	-4.445146834	1.31E-05	2.93E-05	2.171136791
WEE2	-0.062183191	0.13551128	-4.444647147	1.31E-05	2.94E-05	2.169075843
AGPS	-0.313037888	3.73559831	-4.443263962	1.32E-05	2.96E-05	2.163371973
ZNF431	0.465322305	3.34935992	4.442441555	1.32E-05	2.97E-05	2.159981319
SPAG1	-0.320285086	2.18103331	-4.442371369	1.32E-05	2.97E-05	2.159691975
ZNF772	-0.359858812	3.26562302	-4.442132908	1.33E-05	2.97E-05	2.158708951
REV1	-0.362474873	3.68402375	-4.439661093	1.34E-05	3.00E-05	2.14852188
SLC6A9	-0.408950134	2.20642356	-4.439268226	1.34E-05	3.00E-05	2.146903209
C2CD2L	-0.186981073	2.64109559	-4.438808233	1.35E-05	3.01E-05	2.145008124
CKS1B	-0.334631826	3.66970933	-4.438565176	1.35E-05	3.01E-05	2.14400684
ANKRD2	0.636689783	1.70497955	4.438214307	1.35E-05	3.02E-05	2.142561509
AP1M1	-0.191062536	3.30689074	-4.438027429	1.35E-05	3.02E-05	2.141791743
PCBP4	-0.343237393	4.49799815	-4.436602752	1.36E-05	3.04E-05	2.135924289
ZIM2	-0.063981171	0.11399378	-4.436171065	1.36E-05	3.04E-05	2.134146729
AP3M1	-0.357054605	4.52521776	-4.435813455	1.36E-05	3.05E-05	2.132674307
MIEF1	-0.329360047	3.9598345	-4.435405741	1.37E-05	3.05E-05	2.130995712
NSUN7	-0.3573691	3.05537928	-4.435286235	1.37E-05	3.05E-05	2.130503721
POGLUT	-0.258281056	2.82454181	-4.433791705	1.37E-05	3.07E-05	2.124351882
UBE2A	-0.3038853	5.28674756	-4.432852149	1.38E-05	3.08E-05	2.120485356
NUDT3	-0.234100717	3.77840549	-4.432232847	1.38E-05	3.09E-05	2.117937145
AMTN	0.24886673	0.29146622	4.431827023	1.39E-05	3.10E-05	2.116267487
CYP4X1	-0.385421238	2.08930553	-4.431734296	1.39E-05	3.10E-05	2.115886006
MASP1	-0.133544969	0.16379188	-4.431379617	1.39E-05	3.10E-05	2.114426906
AGA	-0.328913509	5.26227294	-4.42986756	1.40E-05	3.12E-05	2.10820763
FGR	0.480582694	3.06606729	4.428884445	1.40E-05	3.14E-05	2.104164933
S100A8	0.66067368	2.76666489	4.428831618	1.40E-05	3.14E-05	2.103947724
OVOL2	-0.345797578	3.47708114	-4.428630895	1.41E-05	3.14E-05	2.103122426
TMEM67	-0.310138325	2.39320624	-4.427699447	1.41E-05	3.15E-05	2.099293089
ZSCAN3	-0.20744256	1.17460559	-4.427599793	1.41E-05	3.15E-05	2.098883435

NFRKB	-0.322356538	4.29141514	-4.42696657	1.42E-05	3.16E-05	2.096280599
CNOT8	-0.299897783	5.60878795	-4.426629109	1.42E-05	3.16E-05	2.094893605
ATP6V0(	-0.359983729	6.24304074	-4.426465307	1.42E-05	3.17E-05	2.094220398
MTHFD2	-0.275216838	2.66571917	-4.426136495	1.42E-05	3.17E-05	2.092869088
LIG3	-0.263989765	2.82011009	-4.426045053	1.42E-05	3.17E-05	2.092493303
SORBS3	-0.298612218	5.6605012	-4.425910536	1.42E-05	3.17E-05	2.091940517
DOCK1	-0.41394684	5.13048725	-4.425602654	1.42E-05	3.18E-05	2.090675353
TRMT6	-0.242925053	3.6575349	-4.42514752	1.43E-05	3.18E-05	2.088805233
B4GALT2	-0.257569659	4.82303539	-4.423597304	1.44E-05	3.20E-05	2.082436719
MUS81	-0.245758097	4.24905397	-4.423013958	1.44E-05	3.21E-05	2.08004074
CORIN	0.184543981	0.34267777	4.422392148	1.44E-05	3.22E-05	2.077487083
UBL3	-0.402529811	6.26155947	-4.422354029	1.44E-05	3.22E-05	2.077330545
IKBKB	-0.288043399	3.84574826	-4.421983242	1.45E-05	3.22E-05	2.075807947
TNFRSF:	0.482698239	5.03409426	4.419701522	1.46E-05	3.25E-05	2.066440719
CSNK1D	-0.287134629	5.74240136	-4.418212667	1.47E-05	3.27E-05	2.060330707
ETNK1	-0.344050065	4.54506019	-4.418001695	1.47E-05	3.28E-05	2.059465056
FAM199X	-0.346752325	4.87196516	-4.417871346	1.47E-05	3.28E-05	2.05893023
LIMD1	-0.395587229	3.75842918	-4.41764331	1.47E-05	3.28E-05	2.057994624
MT3	-0.532724966	0.87358696	-4.41715899	1.48E-05	3.29E-05	2.056007656
RNF182	-0.347905591	1.27673231	-4.417007895	1.48E-05	3.29E-05	2.055387812
MARVEL	0.450274079	4.42816704	4.416133517	1.48E-05	3.30E-05	2.051801172
TMIE	-0.348990869	1.9708051	-4.416032954	1.48E-05	3.30E-05	2.05138871
CLEC4A	0.420704673	2.38956424	4.415830583	1.48E-05	3.30E-05	2.050558698
CAPNS1	-0.234237922	8.44415266	-4.41458803	1.49E-05	3.32E-05	2.045463177
OGFOD1	-0.235208312	4.29284397	-4.414540447	1.49E-05	3.32E-05	2.045268073
SYNJ2BP	-0.124642852	0.51455844	-4.413143157	1.50E-05	3.34E-05	2.039539519
MICALL1	-0.345728646	4.27255418	-4.412743109	1.50E-05	3.35E-05	2.037899708
SLC41A1	-0.402824495	5.69435882	-4.41263141	1.51E-05	3.35E-05	2.03744187
DCAF12	-0.318962811	4.9148333	-4.411852644	1.51E-05	3.36E-05	2.034250109
FAM163A	-0.337805482	0.34762874	-4.41169595	1.51E-05	3.36E-05	2.033607962
ASB7	-0.259447176	3.46237111	-4.410824297	1.52E-05	3.37E-05	2.030036189
SHKBP1	-0.251242001	5.72706587	-4.410613177	1.52E-05	3.38E-05	2.029171175
GSPT2	-0.36694938	3.75373636	-4.410233272	1.52E-05	3.38E-05	2.02761469
SYTL2	0.378754723	1.93912759	4.408762261	1.53E-05	3.40E-05	2.021588995
TBCB	-0.250546858	5.70109871	-4.408558411	1.53E-05	3.40E-05	2.020754098
RBAK	-0.288719751	2.890318	-4.407583129	1.54E-05	3.42E-05	2.016760166
VAMP7	-0.311021534	5.45222193	-4.406904036	1.54E-05	3.43E-05	2.01397962
POLR2B	-0.370209442	5.25675924	-4.406854674	1.54E-05	3.43E-05	2.013777522
TMEM17	0.72609911	3.43597972	4.406727397	1.54E-05	3.43E-05	2.013256434
GINS3	-0.197593449	1.77235354	-4.40662996	1.54E-05	3.43E-05	2.012857521

SLC44A3	-0.356869038	3.09619226	-4.40520731	1.55E-05	3.45E-05	2.007033989
ZNF253	-0.348023898	3.70756933	-4.403814606	1.56E-05	3.47E-05	2.001334601
HAUS8	-0.20006791	1.87013112	-4.403440193	1.57E-05	3.48E-05	1.999802646
UBE2G1	-0.311255619	4.54907587	-4.403416625	1.57E-05	3.48E-05	1.99970622
FOXJ3	-0.32349153	4.54990905	-4.403081758	1.57E-05	3.48E-05	1.998336174
GNA11	-0.323960129	5.60576685	-4.402638614	1.57E-05	3.49E-05	1.996523271
FAAP100	-0.24880864	4.71613496	-4.402538418	1.57E-05	3.49E-05	1.996113391
AP1G1	-0.315806908	4.80282054	-4.402501122	1.57E-05	3.49E-05	1.995960822
TFEC	0.339704634	0.88039391	4.401508872	1.58E-05	3.50E-05	1.991902204
ATF7	-0.228526568	2.94906156	-4.401183618	1.58E-05	3.51E-05	1.990571982
ITK	0.421860338	0.83869823	4.400405751	1.59E-05	3.52E-05	1.987391007
CLCN3	-0.371624417	4.87857609	-4.400269855	1.59E-05	3.52E-05	1.98683533
IL37	0.390041794	0.34956572	4.399361852	1.59E-05	3.53E-05	1.983122894
ADRB3	0.124494366	0.15485943	4.398964663	1.60E-05	3.54E-05	1.981499166
ANO4	-0.147720999	0.18573584	-4.398729367	1.60E-05	3.54E-05	1.980537325
MGAT5B	-0.160374573	0.3234587	-4.398669531	1.60E-05	3.54E-05	1.980292733
PPP4R2	-0.28182817	4.96361798	-4.397944237	1.60E-05	3.55E-05	1.977328192
UBC	-0.275417475	8.72435016	-4.397720976	1.60E-05	3.55E-05	1.976415726
ETAA1	-0.267153782	3.73584039	-4.396946285	1.61E-05	3.57E-05	1.97324988
DGCR2	-0.343161824	6.71913111	-4.395951396	1.62E-05	3.58E-05	1.96918488
CD3E	0.788825514	2.85914994	4.395659027	1.62E-05	3.59E-05	1.967990441
AKT1	-0.279661963	5.38992279	-4.395451463	1.62E-05	3.59E-05	1.96714251
KRTAP5-	0.175046753	0.43389228	4.394704385	1.63E-05	3.60E-05	1.964090854
KHDRBS	-0.279755603	7.11421811	-4.394602955	1.63E-05	3.60E-05	1.963676566
CLEC11A	-0.444272976	4.25666178	-4.393749361	1.63E-05	3.61E-05	1.960190428
FETUB	-0.134454354	0.12447232	-4.393453633	1.63E-05	3.62E-05	1.958982793
TGDS	-0.335546652	4.12744687	-4.391981209	1.64E-05	3.64E-05	1.952971024
SPHK1	0.463745422	3.64044717	4.391755498	1.65E-05	3.64E-05	1.95204962
CLVS1	-0.096023118	0.16280108	-4.391693102	1.65E-05	3.64E-05	1.951794913
VPS26A	-0.295391543	5.67120937	-4.391272666	1.65E-05	3.65E-05	1.950078729
CSKMT	-0.27082879	2.27049693	-4.39111546	1.65E-05	3.65E-05	1.949437062
CAMLG	-0.251717375	6.08544975	-4.390289826	1.66E-05	3.66E-05	1.946067406
SCUBE2	-0.493296039	1.63700984	-4.390111271	1.66E-05	3.67E-05	1.945338742
PLAG1	0.661071868	2.88501904	4.388187052	1.67E-05	3.70E-05	1.937487826
QKI	-0.328266982	3.42080141	-4.388126647	1.67E-05	3.70E-05	1.937241418
PLA2G7	-0.656438011	3.99241345	-4.38769726	1.68E-05	3.70E-05	1.935489918
CCDC14	-0.228934486	1.7758478	-4.387672484	1.68E-05	3.70E-05	1.935388861
AFAP1L2	-0.501634052	6.67490083	-4.387169561	1.68E-05	3.71E-05	1.933337603
RPL13A	-0.302970351	11.0976513	-4.385877602	1.69E-05	3.73E-05	1.92806905
PRSS12	0.476839134	0.95125866	4.385376245	1.69E-05	3.74E-05	1.926024899

TPPP3	-0.598185583	3.95478561	-4.384520147	1.70E-05	3.75E-05	1.922534845
EIF3F	-0.251928383	5.71404697	-4.384188191	1.70E-05	3.75E-05	1.921181722
KIAA089!	-0.252395425	1.77930639	-4.383770119	1.70E-05	3.76E-05	1.919477692
ZNF823	-0.251144361	2.54188088	-4.38346516	1.71E-05	3.77E-05	1.918234791
LPIN3	-0.375464076	3.76001581	-4.382510594	1.71E-05	3.78E-05	1.914344809
ZNF627	-0.3507335	4.1603979	-4.382405582	1.71E-05	3.78E-05	1.913916916
RBM43	-0.405538649	4.03006249	-4.381142783	1.72E-05	3.80E-05	1.908772074
HOMEZ	-0.216719622	3.61741608	-4.380748197	1.73E-05	3.81E-05	1.907164729
TNFRSF:	0.798646315	7.44694688	4.380580402	1.73E-05	3.81E-05	1.906481252
FAN1	-0.385491007	3.10734191	-4.380302102	1.73E-05	3.81E-05	1.905347713
ARHGEF	-0.335025332	2.98376106	-4.379153388	1.74E-05	3.83E-05	1.900669547
ARG2	-0.681836989	4.97379136	-4.378903857	1.74E-05	3.84E-05	1.899653466
RSU1	-0.296975018	5.49099143	-4.378812219	1.74E-05	3.84E-05	1.899280331
MARK1	-0.363301953	2.54487895	-4.378516982	1.74E-05	3.84E-05	1.898078222
SPAST	-0.346608496	3.93691241	-4.378449706	1.74E-05	3.84E-05	1.897804306
CNTD2	-0.70831809	3.17499393	-4.378312729	1.74E-05	3.84E-05	1.897246613
STAT4	0.524566303	1.95508856	4.376233291	1.76E-05	3.88E-05	1.888782138
DPF1	-0.066144963	0.15289887	-4.375957819	1.76E-05	3.88E-05	1.887661072
KCTD14	-0.394330044	4.26064567	-4.375608472	1.76E-05	3.89E-05	1.88623945
INSL5	-0.122498673	0.19900431	-4.374906612	1.77E-05	3.90E-05	1.883383621
TMEM15	0.367170727	1.02198113	4.374693499	1.77E-05	3.90E-05	1.882516553
CPE	-0.704801923	8.1585733	-4.373379143	1.78E-05	3.92E-05	1.877169791
C3orf52	-0.504942905	3.00971381	-4.371759697	1.79E-05	3.95E-05	1.870583835
ZNF135	-0.243599234	2.65444023	-4.371284021	1.80E-05	3.96E-05	1.868649757
ZNF24	-0.292666767	5.08341581	-4.370129297	1.81E-05	3.98E-05	1.863955452
HLA-DPE	0.761746419	7.4356803	4.369466722	1.81E-05	3.99E-05	1.861262363
COMMD:	-0.232692176	4.1044899	-4.368824198	1.82E-05	4.00E-05	1.858651112
TTC4	-0.186152193	1.6399998	-4.366898189	1.83E-05	4.03E-05	1.850825684
FHDC1	-0.468475499	3.98741519	-4.365493031	1.84E-05	4.05E-05	1.845118366
GCHFR	-0.327881827	3.3910617	-4.365465762	1.84E-05	4.05E-05	1.845007621
DNAJC1:	-0.361289953	3.7211174	-4.364633386	1.85E-05	4.07E-05	1.84162753
CCL16	-0.105564099	0.25045921	-4.364569135	1.85E-05	4.07E-05	1.841366646
THUMPD	-0.265028367	4.40945341	-4.363402231	1.86E-05	4.09E-05	1.836629103
ZC3H12E	-0.283027214	1.10166979	-4.362710497	1.86E-05	4.10E-05	1.833821231
ALDH16/	-0.281189216	4.2941566	-4.362215751	1.87E-05	4.11E-05	1.831813204
SLC5A7	-0.361825813	0.29503383	-4.360951655	1.88E-05	4.13E-05	1.826683503
BRAT1	-0.259403206	4.65611931	-4.360896518	1.88E-05	4.13E-05	1.82645979
GSTP1	-0.367234291	9.7289909	-4.360846986	1.88E-05	4.13E-05	1.826258816
FTCD	-0.228266869	0.36987524	-4.359851367	1.89E-05	4.15E-05	1.822219579
ZNF624	-0.264856823	1.87907283	-4.35931163	1.89E-05	4.15E-05	1.820030195

SGSH	-0.297448216	4.70812885	-4.358493312	1.90E-05	4.17E-05	1.81671122
ARL6IP1	-0.294619955	7.07959295	-4.358009862	1.90E-05	4.18E-05	1.814750669
TPSB2	0.628025666	1.28804782	4.358003457	1.90E-05	4.18E-05	1.814724698
GSAP	-0.284931271	3.23263576	-4.356398388	1.91E-05	4.20E-05	1.808216967
PPP1R18	0.377544278	4.8113623	4.35575148	1.92E-05	4.22E-05	1.805594673
IGFL4	0.135326057	0.19183858	4.354929071	1.93E-05	4.23E-05	1.802261451
MAX	-0.22584318	5.74112412	-4.354848428	1.93E-05	4.23E-05	1.801934635
CYB5R3	-0.284865419	6.48184898	-4.353362051	1.94E-05	4.26E-05	1.795911831
FCGR2B	0.404535672	1.266114	4.352811637	1.94E-05	4.27E-05	1.793681999
ZNF70	-0.248461309	2.10862965	-4.352164923	1.95E-05	4.28E-05	1.791062348
FKBP1B	-0.378498042	2.59794937	-4.351593297	1.95E-05	4.29E-05	1.788747137
DUSP6	0.777142735	7.56023567	4.350735832	1.96E-05	4.30E-05	1.78527471
PPP1R3(	-0.212544525	0.55426716	-4.350494712	1.96E-05	4.31E-05	1.784298366
STRN3	-0.324532665	4.12855527	-4.349902433	1.97E-05	4.32E-05	1.781900304
SAMD4A	0.516522175	3.54943757	4.349708796	1.97E-05	4.32E-05	1.781116354
YME1L1	-0.352747019	5.81093459	-4.348948018	1.98E-05	4.33E-05	1.778036589
CELSR3	0.183243977	0.81176472	4.348696073	1.98E-05	4.34E-05	1.777016774
CWF19L	-0.245063003	4.06797141	-4.348644412	1.98E-05	4.34E-05	1.776807672
SMAD5	-0.334340811	4.16587105	-4.348572994	1.98E-05	4.34E-05	1.776518598
KLHL6	0.417921388	1.14203878	4.348567638	1.98E-05	4.34E-05	1.776496919
TMEM59	0.85543905	1.98347453	4.348338752	1.98E-05	4.34E-05	1.775570512
MTMR14	-0.182867881	4.51568177	-4.346965218	1.99E-05	4.37E-05	1.770012055
FXYD6	-0.494682222	3.87981794	-4.346796002	1.99E-05	4.37E-05	1.769327373
BUB1	0.342472843	1.29480508	4.345975144	2.00E-05	4.38E-05	1.766006335
TREM2	0.738512155	4.22360153	4.345903843	2.00E-05	4.38E-05	1.765717889
SIGLEC1	0.437944832	1.16003516	4.344289992	2.02E-05	4.41E-05	1.75919022
PI4K2A	0.314486278	5.33575432	4.343964943	2.02E-05	4.42E-05	1.757875723
IL18	0.704637177	3.22882051	4.343850725	2.02E-05	4.42E-05	1.757413843
RASSF4	-0.368336265	5.13627747	-4.343523287	2.02E-05	4.43E-05	1.756089799
SP140	0.414331212	0.91326699	4.34256949	2.03E-05	4.44E-05	1.752233473
DLGAP2	-0.197244679	0.28439691	-4.342517391	2.03E-05	4.44E-05	1.752022852
RTF1	-0.256598333	5.25139221	-4.342377804	2.03E-05	4.45E-05	1.751458551
HPN	0.940316247	5.80467699	4.342319498	2.03E-05	4.45E-05	1.751222843
CD300LE	0.215665603	0.6359598	4.342071166	2.03E-05	4.45E-05	1.750218972
TACR1	0.175770286	0.44969093	4.341283729	2.04E-05	4.46E-05	1.747036127
ARHGEF	-0.442898002	1.740581	-4.341133547	2.04E-05	4.47E-05	1.746429142
SEPSEC	-0.276397678	2.92051911	-4.340965118	2.04E-05	4.47E-05	1.745748434
FIG4	-0.22241164	4.48343023	-4.340219049	2.05E-05	4.48E-05	1.742733445
AHCYL1	-0.329056225	6.90932632	-4.339808988	2.05E-05	4.49E-05	1.741076513
GPR37L1	-0.311342951	0.35028812	-4.339568122	2.06E-05	4.49E-05	1.740103309

SCAMP2	-0.239602728	6.16006325	-4.339473437	2.06E-05	4.50E-05	1.739720753
KIAA121	-0.306044127	4.07434395	-4.338046674	2.07E-05	4.52E-05	1.733957071
WDR17	-0.341917632	1.34150587	-4.337709219	2.07E-05	4.53E-05	1.732594096
CLEC6A	0.128686357	0.15852489	4.337468877	2.07E-05	4.53E-05	1.731623417
SECTM1	0.555427566	2.10205037	4.336555675	2.08E-05	4.55E-05	1.727935645
UTP3	-0.26382357	5.64970009	-4.335977999	2.09E-05	4.56E-05	1.725603167
PDE4D	-0.499381909	2.37293492	-4.335805784	2.09E-05	4.56E-05	1.724907871
APPL2	-0.323389013	4.05959539	-4.335408152	2.09E-05	4.57E-05	1.723302566
GP6	-0.153748976	0.37565242	-4.334783643	2.10E-05	4.58E-05	1.72078158
PPM1E	-0.112655256	0.32482369	-4.334673709	2.10E-05	4.58E-05	1.720337836
TTC22	-0.246323502	2.99165032	-4.334610395	2.10E-05	4.58E-05	1.720082278
MYH10	0.580332664	5.80719253	4.334447052	2.10E-05	4.59E-05	1.719422979
IMPAD1	-0.325701163	5.54204693	-4.333977847	2.11E-05	4.59E-05	1.717529248
SIRPG	0.564885853	1.1836474	4.333476353	2.11E-05	4.60E-05	1.715505396
ZNF488	-0.087376565	0.14843184	-4.33315843	2.11E-05	4.61E-05	1.714222475
LGALS1	0.6512062	8.41342284	4.332676863	2.12E-05	4.62E-05	1.712279355
GOLPH3	-0.314000855	6.60682789	-4.330056155	2.14E-05	4.67E-05	1.701708068
SRSF10	-0.311395717	4.75985967	-4.328861274	2.15E-05	4.69E-05	1.696890047
SURF2	-0.315024413	5.90779721	-4.328756422	2.15E-05	4.69E-05	1.696467318
CASKIN1	-0.311945453	1.14026981	-4.328494788	2.15E-05	4.70E-05	1.695412525
ZC3H12(	-0.354927472	2.87123421	-4.327526178	2.16E-05	4.72E-05	1.691508008
BOK	-0.485699175	4.44212592	-4.326905098	2.17E-05	4.73E-05	1.689004796
FCAR	0.107671348	0.14483772	4.326304929	2.17E-05	4.74E-05	1.686586162
GPR142	-0.206916135	0.19086458	-4.3262354	2.18E-05	4.74E-05	1.686305984
LGALS12	0.114345233	0.2315338	4.325789987	2.18E-05	4.75E-05	1.684511216
HTR2A	0.04804243	0.07163885	4.325648424	2.18E-05	4.75E-05	1.68394083
CDC20	-0.451601503	2.75415275	-4.32526071	2.18E-05	4.76E-05	1.68237873
IRX1	-0.151943555	0.35053886	-4.32498455	2.19E-05	4.76E-05	1.681266156
NUP133	-0.314693049	4.59106206	-4.324550641	2.19E-05	4.77E-05	1.679518177
NUTM2D	-0.121468648	0.55438814	-4.324368127	2.19E-05	4.77E-05	1.678782972
PARVG	0.414126712	1.72903204	4.324001065	2.20E-05	4.78E-05	1.677304456
CD33	0.272607511	0.98707185	4.323355791	2.20E-05	4.79E-05	1.674705571
VSTM5	-0.128818792	0.29699843	-4.322262804	2.21E-05	4.82E-05	1.670304251
CLEC4F	0.264690354	0.53190566	4.32205061	2.21E-05	4.82E-05	1.669449885
CCR7	0.581502442	1.73352628	4.321096922	2.22E-05	4.84E-05	1.665610453
NFASC	-0.228571089	0.78509893	-4.320696528	2.23E-05	4.85E-05	1.663998734
TRAF5	0.31663204	1.47803367	4.320337707	2.23E-05	4.85E-05	1.662554471
CASK	-0.35083882	3.18158321	-4.32022919	2.23E-05	4.85E-05	1.662117707
PTPRN	0.086353587	0.09452874	4.318543656	2.25E-05	4.89E-05	1.655334919
SEPTIN5	-0.471595921	3.36395233	-4.317834587	2.25E-05	4.90E-05	1.652482226

RYK	-0.338949635	4.82055027	-4.317396235	2.26E-05	4.91E-05	1.650718872
ARMC3	-0.146514321	0.09341427	-4.317236489	2.26E-05	4.91E-05	1.650076298
SPAG8	-0.342724075	2.39130642	-4.316383927	2.27E-05	4.93E-05	1.646647258
TRA2A	-0.301545327	5.85889929	-4.315316794	2.28E-05	4.95E-05	1.642356026
SH3RF2	-0.443409328	2.78969085	-4.315304639	2.28E-05	4.95E-05	1.642307154
DDX46	-0.318948106	4.06042352	-4.31485978	2.28E-05	4.96E-05	1.64051853
RSRC2	-0.295063736	4.74421514	-4.314811518	2.28E-05	4.96E-05	1.640324495
ZDHHC1	-0.269488109	4.64683796	-4.314754507	2.28E-05	4.96E-05	1.640095284
CYTIP	0.63581622	2.33422621	4.313270251	2.30E-05	4.99E-05	1.634128885
CD28	0.368528948	0.79657178	4.313194731	2.30E-05	4.99E-05	1.633825358
RPS18	-0.257019857	10.6564596	-4.312243821	2.31E-05	5.01E-05	1.630003886
PCDHAC	0.349108491	0.91913686	4.312042324	2.31E-05	5.02E-05	1.629194215
PTGIS	0.540036375	1.45844802	4.311495598	2.31E-05	5.03E-05	1.626997476
SLC4A7	0.371606677	2.1201898	4.310898202	2.32E-05	5.04E-05	1.624597421
ZNF726	-0.172219114	0.88958221	-4.310503509	2.32E-05	5.05E-05	1.62301189
INTS2	-0.30175433	2.35321416	-4.310088692	2.33E-05	5.05E-05	1.621345655
XRCC4	-0.24882937	2.83129148	-4.309172343	2.34E-05	5.07E-05	1.617665355
MATN3	0.397098402	1.17023158	4.307892586	2.35E-05	5.10E-05	1.612526646
NFXL1	-0.339855896	3.10391251	-4.307072141	2.36E-05	5.12E-05	1.60923294
TAF9B	-0.269634185	5.04155109	-4.306715082	2.36E-05	5.12E-05	1.607799684
ADAMTS	-0.318443701	1.60810883	-4.306481765	2.36E-05	5.13E-05	1.606863191
RSPH3	-0.305766537	3.89202149	-4.30638002	2.37E-05	5.13E-05	1.606454819
PRKCD	-0.266893026	7.04090958	-4.304906985	2.38E-05	5.16E-05	1.600543453
ZNF12	-0.305077501	4.15769351	-4.304627693	2.38E-05	5.17E-05	1.599422837
ZSWIM1	-0.228358797	3.8720656	-4.304524326	2.38E-05	5.17E-05	1.599008105
MROH7-"	-0.086511238	0.161197	-4.30441177	2.39E-05	5.17E-05	1.598556521
GPR61	-0.368088833	1.10365133	-4.304411666	2.39E-05	5.17E-05	1.598556105
CENPV	-0.400076514	3.33704861	-4.303748128	2.39E-05	5.18E-05	1.595894128
PSG5	0.092803885	0.08072868	4.302849307	2.40E-05	5.20E-05	1.592288811
TNFAIP8	0.542104957	2.82698708	4.302653301	2.40E-05	5.21E-05	1.591502683
CDH26	-0.108984618	0.37947839	-4.302608803	2.40E-05	5.21E-05	1.591324217
CD300C	-0.419964626	2.26465841	-4.302336326	2.41E-05	5.21E-05	1.590231452
TMEM43	0.461158012	6.88853746	4.301222799	2.42E-05	5.23E-05	1.585766276
CCNJ	-0.329630046	2.7579952	-4.301200501	2.42E-05	5.23E-05	1.585676871
IQSEC1	-0.242636364	4.64439196	-4.300768079	2.42E-05	5.24E-05	1.583943164
ABHD16/	-0.304700151	2.75645839	-4.299802236	2.43E-05	5.26E-05	1.580071357
SNX6	-0.321812086	5.23689898	-4.299794531	2.43E-05	5.26E-05	1.580040473
ID2	-0.408045035	8.05409765	-4.299015884	2.44E-05	5.28E-05	1.576919634
MAP3K5	0.577035938	5.1394242	4.298672791	2.44E-05	5.29E-05	1.575544665
GBP5	0.606167605	1.44937021	4.298627464	2.44E-05	5.29E-05	1.575363022

C6orf120	-0.32517303	3.95320619	-4.298524983	2.44E-05	5.29E-05	1.574952342
CAPNS2	-0.147342091	0.39933637	-4.297949859	2.45E-05	5.30E-05	1.572647775
CLIP2	0.374917647	2.85428105	4.297395529	2.46E-05	5.31E-05	1.570426779
EIF4ENIF	-0.283143149	4.12035213	-4.296762999	2.46E-05	5.33E-05	1.567892771
PMS1	-0.268285871	3.07211237	-4.29644315	2.47E-05	5.33E-05	1.566611533
EGR1	-0.800627805	8.77581037	-4.296421333	2.47E-05	5.33E-05	1.566524142
TMA16	-0.240055801	3.63959103	-4.294936702	2.48E-05	5.37E-05	1.560578163
CCDC10:	-0.240713137	1.79157361	-4.29454564	2.49E-05	5.37E-05	1.559012249
BLVRA	-0.244740657	6.02065897	-4.294295581	2.49E-05	5.38E-05	1.55801101
DENND1	-0.265306856	3.70603622	-4.294161143	2.49E-05	5.38E-05	1.557472743
SYCP2L	-0.19218407	0.50486847	-4.293339971	2.50E-05	5.40E-05	1.554185209
PLEK	0.636089517	3.20857922	4.292897831	2.50E-05	5.41E-05	1.552415344
C5orf15	-0.339144748	6.61748044	-4.292660775	2.51E-05	5.41E-05	1.551466482
ADAM19	0.423805357	1.66678283	4.292559526	2.51E-05	5.41E-05	1.551061231
SPIN3	-0.290220027	2.26457426	-4.292434233	2.51E-05	5.42E-05	1.550559749
TUBB4B	-0.331727305	8.66401572	-4.292029018	2.51E-05	5.43E-05	1.548937983
NOS3	-0.374408668	3.01224146	-4.291197686	2.52E-05	5.44E-05	1.545611207
ARHGAP	0.522661334	1.94952427	4.291031815	2.52E-05	5.45E-05	1.544947503
ESPNL	-0.587877153	1.57523246	-4.290632084	2.53E-05	5.46E-05	1.543348135
CASS4	0.303235457	0.76348655	4.288750478	2.55E-05	5.50E-05	1.535821355
MEF2B	-0.193866572	0.96382939	-4.288003123	2.56E-05	5.51E-05	1.532832586
KIF21B	0.288027723	0.76203557	4.287964821	2.56E-05	5.51E-05	1.532679423
TGFBR3	-0.391614026	2.7197051	-4.287327716	2.56E-05	5.53E-05	1.530131934
PSME1	-0.282133586	8.24953152	-4.287191946	2.56E-05	5.53E-05	1.529589094
PPM1B	-0.299855778	4.18924454	-4.287140202	2.56E-05	5.53E-05	1.529382214
NYAP2	0.295444344	0.23952154	4.286372446	2.57E-05	5.55E-05	1.526312864
NIFK	-0.268566723	5.18879795	-4.285235912	2.59E-05	5.57E-05	1.521770079
KCNE1	0.066522229	0.11010948	4.285113521	2.59E-05	5.58E-05	1.521280937
RPL27A	-0.298768792	8.33578436	-4.284028095	2.60E-05	5.60E-05	1.516943517
DCAF1	-0.331599287	4.2178287	-4.283765699	2.60E-05	5.61E-05	1.515895108
SIK2	-0.324039359	4.15775524	-4.283401804	2.61E-05	5.61E-05	1.514441255
NSA2	-0.265313063	5.80633563	-4.28324745	2.61E-05	5.62E-05	1.513824604
COL11A2	-0.676358246	1.71959608	-4.283235136	2.61E-05	5.62E-05	1.51377541
C1orf162	0.458145318	3.30383185	4.282684461	2.61E-05	5.63E-05	1.511575598
SUDS3	-0.266506209	5.15426853	-4.282142644	2.62E-05	5.64E-05	1.509411408
KCND2	0.129624773	0.17632637	4.281451358	2.63E-05	5.66E-05	1.506650542
NECAP1	-0.282353098	4.62022696	-4.280314262	2.64E-05	5.68E-05	1.502110029
MTMR2	-0.26370066	3.7906485	-4.280096176	2.64E-05	5.69E-05	1.501239314
NAP1L3	-0.50389473	3.28306304	-4.279572235	2.65E-05	5.70E-05	1.499147621
C16orf54	0.513419462	1.38336374	4.27956977	2.65E-05	5.70E-05	1.499137778

BTBD1	-0.325506695	5.87977215	-4.279564502	2.65E-05	5.70E-05	1.499116751
DOK3	0.404950053	1.77393009	4.279220058	2.65E-05	5.71E-05	1.497741772
AZI2	-0.356516592	3.12465383	-4.27920687	2.65E-05	5.71E-05	1.497689129
NOTCH2	0.537725728	4.87393033	4.278987694	2.65E-05	5.71E-05	1.496814259
LHCGR	-0.231747106	0.24499439	-4.278797402	2.66E-05	5.71E-05	1.496054713
DPH7	-0.269324041	3.83342937	-4.278675267	2.66E-05	5.72E-05	1.49556723
RAB3GA	-0.307976487	5.13955607	-4.278654315	2.66E-05	5.72E-05	1.495483607
DUSP16	-0.335813847	4.85433633	-4.278646337	2.66E-05	5.72E-05	1.495451763
ZFP92	-0.225402384	0.8128612	-4.278411653	2.66E-05	5.72E-05	1.494515096
CMTM1	0.34071283	1.43412967	4.278191003	2.66E-05	5.73E-05	1.493634487
RCOR2	-0.401055569	1.36419878	-4.276514957	2.68E-05	5.76E-05	1.486946682
MBLAC2	-0.295398929	2.43596386	-4.276247936	2.68E-05	5.77E-05	1.485881416
NOP14	-0.292586537	5.35005853	-4.275879168	2.69E-05	5.78E-05	1.484410336
NOA1	-0.284151367	4.67156766	-4.275425967	2.69E-05	5.79E-05	1.482602584
CCR5	0.573341731	1.94641809	4.274782372	2.70E-05	5.80E-05	1.480035662
ZNF594	-0.32303917	2.51252996	-4.274730927	2.70E-05	5.80E-05	1.479830493
CDC42SI	-0.326468817	5.67966166	-4.274599846	2.70E-05	5.81E-05	1.479307736
FASN	-0.387856748	4.39636985	-4.274152614	2.71E-05	5.82E-05	1.477524255
LRP8	-0.611100959	3.59560776	-4.273888332	2.71E-05	5.82E-05	1.476470423
ZYX	0.4132867	6.81461471	4.273819636	2.71E-05	5.82E-05	1.476196505
C2orf69	-0.269741015	3.2997701	-4.273415106	2.72E-05	5.83E-05	1.474583557
TNNI2	0.363661879	0.88380928	4.273344734	2.72E-05	5.83E-05	1.474302983
HLA-DM/	0.598616223	6.15440107	4.273015873	2.72E-05	5.84E-05	1.472991862
NASP	-0.225084035	4.74542497	-4.272601931	2.73E-05	5.85E-05	1.47134166
RABGAP	-0.338103593	4.93212915	-4.272165712	2.73E-05	5.86E-05	1.4696028
GPR21	-0.338103593	4.93212915	-4.272165712	2.73E-05	5.86E-05	1.4696028
PIK3R6	0.277927348	1.07140136	4.271493615	2.74E-05	5.88E-05	1.466923983
NBEAL2	0.354008945	3.43822617	4.271390634	2.74E-05	5.88E-05	1.466513555
MED24	-0.238371357	4.8622775	-4.271337139	2.74E-05	5.88E-05	1.466300358
GLUL	-0.397171231	7.76580926	-4.271300846	2.74E-05	5.88E-05	1.466155718
ZNF256	-0.224679298	2.99403063	-4.271019031	2.74E-05	5.88E-05	1.465032621
ANTXR1	-0.528927552	4.9221114	-4.270735673	2.75E-05	5.89E-05	1.463903444
PAXIP1	-0.24647932	2.24399357	-4.270001662	2.76E-05	5.91E-05	1.460978717
MORN5	-0.199087725	0.18394857	-4.269758326	2.76E-05	5.91E-05	1.460009219
DAPK3	-0.24141241	4.93924739	-4.269554236	2.76E-05	5.92E-05	1.459196124
ARSF	-0.373713626	0.27106591	-4.26938019	2.76E-05	5.92E-05	1.458502752
ABHD14/	-0.150671057	0.5800595	-4.269375684	2.76E-05	5.92E-05	1.458484801
NUDCD1	-0.286983944	3.4802507	-4.269347808	2.76E-05	5.92E-05	1.45837375
SOCS4	-0.290034543	3.3240652	-4.269240805	2.76E-05	5.92E-05	1.457947479
PRSS35	-0.285642327	0.63143821	-4.268218629	2.78E-05	5.95E-05	1.453875891

VASH2	0.148009892	0.39830236	4.266930681	2.79E-05	5.98E-05	1.448746867
ATRIP	-0.083529305	0.21112895	-4.266822873	2.79E-05	5.98E-05	1.448317605
RIC3	-0.374205695	3.21477088	-4.266380787	2.80E-05	5.99E-05	1.446557422
VARS2	-0.286146495	3.81351055	-4.263987651	2.83E-05	6.05E-05	1.437031814
MYBPC2	0.429042819	0.84195671	4.263452691	2.83E-05	6.06E-05	1.434903098
SKOR1	-0.118740807	0.38886896	-4.261203343	2.86E-05	6.12E-05	1.425955017
PTGER2	0.377807172	1.10417533	4.260642928	2.87E-05	6.13E-05	1.423726279
PXK	-0.406858395	4.97093384	-4.260512118	2.87E-05	6.14E-05	1.423206095
RSL1D1	-0.254139864	6.07015276	-4.260370179	2.87E-05	6.14E-05	1.422641667
MYL12A	-0.268103408	7.93976207	-4.260177057	2.87E-05	6.14E-05	1.421873731
FANCL	-0.299964881	4.59644091	-4.2599041	2.87E-05	6.15E-05	1.420788395
FITM1	-0.287098764	1.26537162	-4.259676505	2.88E-05	6.15E-05	1.419883469
CXADR	-0.353745452	4.9838561	-4.259234582	2.88E-05	6.16E-05	1.418126492
TRIM29	0.754113321	1.7787144	4.258898627	2.89E-05	6.17E-05	1.416790927
CATSPE	-0.092444327	0.23656176	-4.258654649	2.89E-05	6.18E-05	1.415821064
ZNF506	-0.275808293	3.26996664	-4.258014157	2.90E-05	6.19E-05	1.413275209
RGS4	0.75082768	1.71410584	4.257534952	2.90E-05	6.20E-05	1.41137066
SAA2	0.391278072	0.45787615	4.257364104	2.91E-05	6.21E-05	1.410691688
TFAM	-0.315510735	4.50996061	-4.256784653	2.91E-05	6.22E-05	1.408389051
TRAM1	-0.332221823	7.3927294	-4.255524839	2.93E-05	6.25E-05	1.403383708
RBBP5	-0.270082219	4.38182152	-4.255213436	2.93E-05	6.26E-05	1.402146675
CLTRN	-0.377119435	2.6762695	-4.254677651	2.94E-05	6.28E-05	1.400018483
LARP6	-0.418350341	4.31313838	-4.254595041	2.94E-05	6.28E-05	1.39969037
ASMTL	-0.269905685	5.69943904	-4.254459209	2.94E-05	6.28E-05	1.399150879
NOC4L	-0.242847302	4.98948721	-4.253845825	2.95E-05	6.29E-05	1.396714848
SLC6A12	0.536047526	3.20362942	4.253431108	2.95E-05	6.30E-05	1.395067988
ANXA1	0.744605704	9.00163729	4.252743968	2.96E-05	6.32E-05	1.392339632
APBB1	-0.32769995	4.54484165	-4.251672601	2.98E-05	6.35E-05	1.388086429
TMEM17	-0.286019105	4.00422512	-4.25142712	2.98E-05	6.36E-05	1.387112029
DACT1	-0.513085017	2.64134664	-4.250256852	2.99E-05	6.39E-05	1.382467494
SLC25A5	-0.065893392	0.11345987	-4.249768042	3.00E-05	6.40E-05	1.380527846
F2RL2	0.373140472	0.82393013	4.249342715	3.00E-05	6.41E-05	1.378840262
MFGE8	0.659366605	6.35362122	4.248814144	3.01E-05	6.42E-05	1.376743234
TMEFF2	-0.087615161	0.17716315	-4.248655179	3.01E-05	6.43E-05	1.376112611
TMEM17	-0.293171798	2.92673762	-4.248141547	3.02E-05	6.44E-05	1.374075135
KCNS3	0.475168856	2.93456925	4.248052239	3.02E-05	6.44E-05	1.373720891
RNF125	0.341586733	2.10102235	4.247431743	3.03E-05	6.45E-05	1.371259837
NEURL4	-0.260595574	3.77830184	-4.247426695	3.03E-05	6.45E-05	1.371239818
ZFYVE1	-0.279311252	4.48112539	-4.247412185	3.03E-05	6.45E-05	1.371182272
BORCS5	-0.216753153	2.50779171	-4.24707526	3.03E-05	6.46E-05	1.369846075

DMRTA1	-0.291460542	2.06629704	-4.2469533	3.03E-05	6.47E-05	1.369362418
LRATD1	0.506372326	3.22164018	4.24578991	3.05E-05	6.50E-05	1.364749396
UCHL1	-0.593731671	3.1060928	-4.245602525	3.05E-05	6.50E-05	1.364006489
CHRNA1	-0.19642894	0.81609548	-4.245502864	3.05E-05	6.50E-05	1.363611382
TRAF3IP	-0.23977689	3.98867428	-4.245391631	3.05E-05	6.50E-05	1.363170412
FUT10	-0.279093049	2.18504681	-4.244751535	3.06E-05	6.52E-05	1.360633006
PRKAB2	-0.311776789	2.9534166	-4.24460097	3.06E-05	6.52E-05	1.360036198
MVB12B	-0.319703758	3.59500567	-4.244371499	3.07E-05	6.53E-05	1.359126659
TMBIM1	-0.335336296	7.49292542	-4.242975443	3.08E-05	6.57E-05	1.353594134
ANKHD1	-0.161935323	1.32294091	-4.242206259	3.09E-05	6.59E-05	1.350546559
ARL10	0.259192997	1.31296508	4.241108494	3.11E-05	6.62E-05	1.346197943
ADCK5	-0.284303176	3.13746368	-4.240519901	3.12E-05	6.63E-05	1.343866731
TCTN2	-0.310993975	3.80997977	-4.24045587	3.12E-05	6.63E-05	1.343613144
INA	0.235111728	0.428381	4.239984919	3.12E-05	6.64E-05	1.341748099
BMT2	-0.273868971	3.35066624	-4.239502591	3.13E-05	6.66E-05	1.339838184
ZNF808	-0.233108099	2.70179098	-4.23940551	3.13E-05	6.66E-05	1.339453788
CSTF2	-0.28170263	4.11655932	-4.239349048	3.13E-05	6.66E-05	1.339230229
COL4A1	-0.514948738	6.78969328	-4.238242443	3.15E-05	6.69E-05	1.334849162
RGS9BP	-0.101445224	0.25785188	-4.237873832	3.15E-05	6.70E-05	1.333390048
GCC1	-0.324511554	3.76858271	-4.235110857	3.19E-05	6.78E-05	1.322456555
TTLL11	-0.240338128	1.83354343	-4.234992488	3.19E-05	6.78E-05	1.321988289
OCSTAM	0.090410443	0.10167703	4.234905278	3.19E-05	6.78E-05	1.321643296
CLTA	-0.279896689	7.94890375	-4.234841147	3.19E-05	6.78E-05	1.321389608
CDKN1B	-0.322469661	6.13563989	-4.234230617	3.20E-05	6.80E-05	1.318974618
TRPC4AI	-0.241717406	6.53906492	-4.234187117	3.20E-05	6.80E-05	1.318802565
PPP1R3I	-0.305141661	3.28691454	-4.233066148	3.21E-05	6.83E-05	1.314369332
CD79A	1.25874823	2.50557745	4.232952325	3.22E-05	6.83E-05	1.313919241
DARS2	-0.314735969	3.69652145	-4.23175407	3.23E-05	6.86E-05	1.309181598
MEMO1	-0.17298135	1.47881642	-4.231728421	3.23E-05	6.86E-05	1.309080198
ARHGAF	0.419862006	3.45321293	4.230302483	3.25E-05	6.90E-05	1.303443894
BRIX1	-0.229907756	3.93703611	-4.230124834	3.25E-05	6.91E-05	1.302741813
SLA2	0.465214374	1.21322693	4.229464422	3.26E-05	6.93E-05	1.300132059
KRT17	1.089493935	2.67649492	4.228511007	3.28E-05	6.95E-05	1.296365064
SNRPB	-0.212044238	8.01995094	-4.228050483	3.28E-05	6.97E-05	1.29454577
EVI5	-0.320668721	3.37929558	-4.227671702	3.29E-05	6.98E-05	1.293049533
ACLY	-0.322414488	5.87813902	-4.226339332	3.31E-05	7.01E-05	1.287787409
ZNF189	-0.31018189	4.37503561	-4.226260871	3.31E-05	7.01E-05	1.28747758
ECPAS	-0.252270531	5.32355675	-4.224428512	3.33E-05	7.07E-05	1.280243248
ARHGAF	-0.252949498	2.19599542	-4.223079986	3.35E-05	7.11E-05	1.27492088
ZG16	-0.034860506	0.04869599	-4.221967271	3.37E-05	7.14E-05	1.270530323

TAF1C	-0.297134895	4.25910252	-4.221950091	3.37E-05	7.14E-05	1.270462539
PLOD2	-0.390665732	6.4874123	-4.22174636	3.37E-05	7.14E-05	1.26965877
SMPDL3.	-0.379364818	4.42586318	-4.221551628	3.37E-05	7.15E-05	1.268890534
HIST3H2	0.857402556	3.13705225	4.221262275	3.38E-05	7.16E-05	1.267749064
BCLAF1	-0.307912225	5.33987296	-4.221093001	3.38E-05	7.16E-05	1.267081328
PARP2	-0.244882451	3.83842796	-4.220814158	3.38E-05	7.17E-05	1.265981425
MCM9	-0.25729891	2.19930619	-4.220311535	3.39E-05	7.18E-05	1.26399897
SMIM24	0.723107338	1.11051275	4.219654117	3.40E-05	7.20E-05	1.261406286
ENTPD5	-0.292506588	2.49093438	-4.219607336	3.40E-05	7.20E-05	1.261221806
SLC22A5	-0.259712535	2.53572638	-4.219301611	3.40E-05	7.21E-05	1.260016236
BCAR1	-0.268571205	4.70174236	-4.218734457	3.41E-05	7.22E-05	1.257779974
RPL4	-0.262013312	9.73341485	-4.218649202	3.41E-05	7.23E-05	1.257443838
ZNF644	-0.322059271	3.98226143	-4.216980731	3.44E-05	7.28E-05	1.25086676
DOCK5	-0.405157219	3.09038963	-4.216513467	3.44E-05	7.29E-05	1.24902522
FABP6	-0.293107473	0.41737051	-4.216476723	3.44E-05	7.29E-05	1.248880416
ZSWIM6	-0.323258144	3.2983448	-4.216256817	3.45E-05	7.29E-05	1.24801381
MSANTD	-0.121015615	0.49154676	-4.215963364	3.45E-05	7.30E-05	1.24685743
RBBP4	-0.270932488	5.60547879	-4.215867235	3.45E-05	7.30E-05	1.246478641
IFT122	-0.304283493	3.81496033	-4.214638042	3.47E-05	7.34E-05	1.241635749
DPP8	-0.296119457	3.78782707	-4.214068586	3.48E-05	7.36E-05	1.239392564
PLP2	0.510583536	7.18465556	4.213457771	3.49E-05	7.37E-05	1.236986757
RGS18	0.305911409	0.99354927	4.213425229	3.49E-05	7.37E-05	1.236858594
USP14	-0.279110044	4.95002085	-4.212128671	3.51E-05	7.41E-05	1.231752898
CPLX1	-0.306542172	1.39871571	-4.211903794	3.51E-05	7.42E-05	1.230867497
HNRNPC	-0.26864068	7.14955446	-4.211762706	3.51E-05	7.42E-05	1.230312018
CD40LG	0.412792416	0.85046868	4.211756668	3.51E-05	7.42E-05	1.230288247
HSBP1L1	-0.322996632	4.75789623	-4.211638224	3.51E-05	7.42E-05	1.229821929
BBS10	-0.333571755	3.85106597	-4.210903369	3.52E-05	7.45E-05	1.226929058
FAF1	-0.245814799	4.86069796	-4.209745087	3.54E-05	7.48E-05	1.222370188
FZD5	-0.443589691	3.79256716	-4.20918668	3.55E-05	7.50E-05	1.220172747
PLD6	-0.264747958	2.31841282	-4.208485086	3.56E-05	7.52E-05	1.217412203
NUDT19	-0.21257874	3.65761186	-4.207759667	3.57E-05	7.54E-05	1.214558331
RFC1	-0.325933473	4.74302292	-4.207657284	3.57E-05	7.54E-05	1.214155582
GPR180	-0.272626472	2.58443053	-4.206727137	3.59E-05	7.57E-05	1.210496996
ZNF714	0.567971913	2.85847102	4.206636614	3.59E-05	7.57E-05	1.210140976
LAMA1	0.580464109	2.33889334	4.205856921	3.60E-05	7.60E-05	1.207074777
GALNT1(	-0.444619174	1.63870799	-4.205176525	3.61E-05	7.62E-05	1.204399471
HLA-DQA	1.024828113	4.52039208	4.205169584	3.61E-05	7.62E-05	1.204372184
MAP3K9	-0.308707134	2.48982565	-4.205022728	3.61E-05	7.62E-05	1.203794798
DNAJC1	-0.257845627	3.4777377	-4.205018878	3.61E-05	7.62E-05	1.20377966

ANKDD1	-0.332898183	2.34333987	-4.202932937	3.64E-05	7.68E-05	1.195580403
TTC27	-0.260762948	4.15971735	-4.201732682	3.66E-05	7.72E-05	1.190864133
SLC2A7	-0.052857182	0.06891661	-4.201559633	3.66E-05	7.73E-05	1.190184253
BTN2A1	-0.317396553	4.25022786	-4.200834612	3.67E-05	7.75E-05	1.187336035
ZNF74	-0.267207195	2.72185224	-4.198297376	3.71E-05	7.83E-05	1.177371968
GPATCH	-0.253597641	2.62321982	-4.19816647	3.71E-05	7.83E-05	1.176858025
ETHE1	0.49789516	6.14748768	4.197796555	3.72E-05	7.84E-05	1.175405796
RNF220	-0.20038307	4.43697315	-4.197712878	3.72E-05	7.84E-05	1.175077308
TSC22D3	-0.490116291	6.82819126	-4.197207429	3.73E-05	7.86E-05	1.173093209
HNRNPH	-0.347237871	6.17514762	-4.194723319	3.77E-05	7.94E-05	1.163345054
METAP1	-0.267709349	4.56956765	-4.193540084	3.79E-05	7.98E-05	1.158703567
DDX18	-0.322537209	4.66768376	-4.192086842	3.81E-05	8.02E-05	1.153004484
TAS1R1	-0.214587094	0.25029767	-4.192056467	3.81E-05	8.02E-05	1.15288538
UPF2	-0.303480841	4.33896589	-4.191793881	3.81E-05	8.03E-05	1.151855804
SNRK	-0.332396764	4.6372059	-4.190586034	3.83E-05	8.07E-05	1.147120666
PRR14	-0.209497343	4.88985724	-4.190456149	3.83E-05	8.07E-05	1.146611547
DISP1	-0.287186827	3.66912903	-4.190101588	3.84E-05	8.09E-05	1.145221818
NOS1AP	0.228090869	0.60180757	4.189247386	3.85E-05	8.11E-05	1.141874134
MMRN1	-0.510336703	1.54140598	-4.189152457	3.86E-05	8.12E-05	1.141502138
SF3B4	-0.233061284	6.80324077	-4.189096423	3.86E-05	8.12E-05	1.141282561
IL17RB	-0.25824527	2.48930231	-4.188333934	3.87E-05	8.14E-05	1.138294905
ITGB7	0.536166796	2.0112322	4.187397469	3.88E-05	8.17E-05	1.134626204
AP2M1	-0.245780442	7.72386872	-4.187285811	3.89E-05	8.17E-05	1.13418882
TLR8	0.348598817	0.84267894	4.186610694	3.90E-05	8.20E-05	1.131544485
TMEM33	-0.275726555	4.69324011	-4.186455999	3.90E-05	8.20E-05	1.130938619
DICER1	-0.342647715	3.84095385	-4.186374681	3.90E-05	8.20E-05	1.130620143
SLC46A1	-0.251478142	2.10708589	-4.186044922	3.91E-05	8.21E-05	1.129328722
BNIP3L	-0.292290879	5.87817135	-4.185890944	3.91E-05	8.22E-05	1.128725737
PRSS23	0.738802534	5.49314378	4.185408691	3.92E-05	8.23E-05	1.12683733
SOBP	-0.469627668	2.75345672	-4.184228108	3.93E-05	8.27E-05	1.122215209
TM7SF3	-0.418634085	5.7629638	-4.183647927	3.94E-05	8.29E-05	1.119944145
FAT3	0.033946438	0.03922284	4.18277265	3.96E-05	8.32E-05	1.116518477
PYGO1	-0.340377456	2.89746316	-4.182500105	3.96E-05	8.33E-05	1.115451918
PNMA5	-0.113008311	0.11720443	-4.182493813	3.96E-05	8.33E-05	1.115427297
GOLGA2	-0.292077934	5.62562039	-4.181391955	3.98E-05	8.36E-05	1.111115974
SFTA3	-0.54514905	6.06860888	-4.181382905	3.98E-05	8.36E-05	1.111080568
RBMX2	-0.21618071	4.66687686	-4.180940605	3.99E-05	8.38E-05	1.109350227
SERPIN(	0.605312471	6.84584933	4.179891524	4.01E-05	8.41E-05	1.105246715
ARHGDI/	-0.224356168	7.53724003	-4.1793257	4.01E-05	8.43E-05	1.103033851
CD3G	0.477411493	1.13889345	4.179219832	4.02E-05	8.43E-05	1.102619846

GCLM	-0.413883395	4.44094621	-4.178549787	4.03E-05	8.45E-05	1.099999779
PLPPR2	-0.343377908	4.75450732	-4.178476501	4.03E-05	8.46E-05	1.099713236
FOSB	-1.184081972	5.77855804	-4.178190019	4.03E-05	8.46E-05	1.098593139
CCAR1	-0.268427899	4.92830999	-4.177947571	4.04E-05	8.47E-05	1.097645259
EXOG	-0.232068466	2.3693989	-4.177610053	4.04E-05	8.48E-05	1.096325775
MFSD2A	-0.766541662	3.9523236	-4.177499316	4.05E-05	8.49E-05	1.095892883
PGM3	-0.294940433	3.97151427	-4.176824758	4.06E-05	8.51E-05	1.093256118
PGM2L1	0.440540409	2.96150028	4.175676701	4.08E-05	8.55E-05	1.088769363
ZBTB24	-0.257934526	3.02192879	-4.17551085	4.08E-05	8.55E-05	1.088121283
EMC1	-0.296158247	4.16513332	-4.175391092	4.08E-05	8.56E-05	1.08765333
PEX26	-0.246464928	3.17603474	-4.175312786	4.08E-05	8.56E-05	1.087347357
ZSCAN2	-0.206623773	2.17382299	-4.174935472	4.09E-05	8.57E-05	1.085873112
HACD1	0.478946723	3.2189908	4.174560538	4.09E-05	8.58E-05	1.084408281
TLE2	-0.348754732	6.10021647	-4.17390948	4.11E-05	8.60E-05	1.081864933
LSM14A	-0.280158997	6.80633224	-4.173873908	4.11E-05	8.60E-05	1.081725979
ARHGAF	-0.353528581	4.97036204	-4.173847205	4.11E-05	8.60E-05	1.081621672
NMBR	-0.102960133	0.21585121	-4.173207156	4.12E-05	8.63E-05	1.079121698
CCDC50	-0.27650258	4.55421809	-4.17056592	4.16E-05	8.72E-05	1.06880881
GRIPAP1	-0.238476203	4.86602683	-4.168840523	4.19E-05	8.78E-05	1.062074952
SHOX2	0.180811313	0.3979411	4.16826008	4.20E-05	8.80E-05	1.059810152
TRIM17	0.332312355	1.45163064	4.168106645	4.20E-05	8.80E-05	1.059211522
ARHGEF	-0.255716892	3.56177559	-4.167822324	4.21E-05	8.81E-05	1.05810228
BRICD5	-0.474897545	3.20055607	-4.166537392	4.23E-05	8.86E-05	1.05309011
ABCD3	-0.334942903	4.63014371	-4.165577691	4.25E-05	8.89E-05	1.049347459
CEP112	-0.290219039	2.60640331	-4.165455403	4.25E-05	8.90E-05	1.048870614
CEP170E	0.362493072	5.37323292	4.165026475	4.26E-05	8.91E-05	1.047198162
PIAS4	-0.214461578	4.09531535	-4.164741267	4.26E-05	8.92E-05	1.046086178
METTL1	-0.297608064	4.35150849	-4.164215948	4.27E-05	8.94E-05	1.044038211
YOD1	-0.292384823	3.06991285	-4.164183435	4.27E-05	8.94E-05	1.043911466
NPY1R	0.501131756	2.36029915	4.162804728	4.30E-05	8.99E-05	1.038537659
MS4A2	0.142135502	0.20967705	4.161476973	4.32E-05	9.04E-05	1.033363915
VPS4B	-0.319411326	5.29234298	-4.161439265	4.32E-05	9.04E-05	1.033217005
PLEKHA	-0.30479938	4.3381845	-4.160682906	4.33E-05	9.06E-05	1.030270436
MAGEE1	-0.293735354	2.8906084	-4.159228713	4.36E-05	9.12E-05	1.02460661
AKAP12	-0.629078101	3.8624581	-4.159078967	4.36E-05	9.12E-05	1.024023472
PFN1	-0.240912439	9.36648768	-4.158910368	4.37E-05	9.13E-05	1.02336694
BASP1	0.699955221	6.108724	4.158119574	4.38E-05	9.15E-05	1.020287867
STX4	-0.206164711	5.15344656	-4.15805751	4.38E-05	9.15E-05	1.020046234
TXN	-0.268289929	7.86721273	-4.157634125	4.39E-05	9.17E-05	1.018397957
DRD1	-0.144911411	0.13238893	-4.15680325	4.40E-05	9.20E-05	1.0151637

SFT2D2	-0.269560831	4.00091137	-4.156703691	4.41E-05	9.20E-05	1.014776198
HNRNPD	-0.287003926	6.830491	-4.156453496	4.41E-05	9.21E-05	1.013802422
SPATA4(	0.113905936	0.16605721	4.156032685	4.42E-05	9.22E-05	1.012164714
PGBD1	-0.274699014	1.92107499	-4.156014126	4.42E-05	9.22E-05	1.012092489
TCIRG1	0.404581312	5.60042783	4.155303104	4.43E-05	9.25E-05	1.009325682
CLEC1B	0.04263586	0.06354871	4.154223624	4.45E-05	9.29E-05	1.005125879
KIAA023	-0.338892422	4.51097339	-4.153959142	4.46E-05	9.30E-05	1.004097038
CTNND1	-0.376284503	6.77272156	-4.153911148	4.46E-05	9.30E-05	1.003910345
TOB2	-0.380697011	6.55510099	-4.15333871	4.47E-05	9.32E-05	1.001683759
ZNF423	-0.313720205	1.69071301	-4.152948967	4.47E-05	9.33E-05	1.000167949
PHKG1	-0.179130898	0.91169241	-4.152591465	4.48E-05	9.35E-05	0.998777642
KRBOX1	-0.466114443	1.10648125	-4.151851171	4.49E-05	9.37E-05	0.995899009
TEDC1	-0.261173305	2.23082445	-4.151423401	4.50E-05	9.39E-05	0.994235828
PLAT	-0.558487469	4.03033499	-4.151418009	4.50E-05	9.39E-05	0.994214866
SRPK2	-0.305248166	3.93239033	-4.151342829	4.50E-05	9.39E-05	0.993922581
UBE2W	-0.261473392	3.58644489	-4.151182557	4.51E-05	9.40E-05	0.99329949
RUBCN	0.302759378	3.77961685	4.150675127	4.52E-05	9.41E-05	0.991326884
IDE	-0.290057037	4.20787585	-4.15062523	4.52E-05	9.41E-05	0.991132922
SLC25A2	-0.245162233	3.58704812	-4.150599748	4.52E-05	9.41E-05	0.99103387
CSGALN	0.401880841	4.21150458	4.150243128	4.52E-05	9.43E-05	0.989647679
SPEG	-0.305720698	1.04572279	-4.149409353	4.54E-05	9.46E-05	0.986407176
GNS	-0.314764844	6.70004797	-4.148992427	4.55E-05	9.47E-05	0.984786988
PKNOX1	-0.330220394	3.82213424	-4.148888006	4.55E-05	9.48E-05	0.984381227
IDH3A	-0.387742446	2.77360156	-4.148816246	4.55E-05	9.48E-05	0.984102389
YIPF2	-0.274241223	6.4456161	-4.148154084	4.56E-05	9.50E-05	0.981529594
RTL9	-0.20141463	0.32793288	-4.148058087	4.56E-05	9.50E-05	0.981156631
SCARF2	-0.392094423	2.98648193	-4.147960427	4.57E-05	9.51E-05	0.980777217
AFMID	-0.262233379	3.80361229	-4.147758128	4.57E-05	9.51E-05	0.9799913
PIGZ	-0.350555746	1.88476023	-4.14733356	4.58E-05	9.53E-05	0.978341989
TMEM63	0.299886576	3.26379276	4.14559109	4.61E-05	9.60E-05	0.971574603
CNR2	-0.224640215	0.37950155	-4.145457494	4.61E-05	9.60E-05	0.971055847
ADAMTS	0.128984218	0.36245406	4.145266455	4.62E-05	9.61E-05	0.970314063
IL31RA	0.074257783	0.07555053	4.144680181	4.63E-05	9.63E-05	0.968037816
1-Mar	0.277616308	1.2676277	4.144401361	4.63E-05	9.64E-05	0.966955374
ADAM21	-0.103028377	0.26859069	-4.144256014	4.64E-05	9.64E-05	0.96639113
XYLB	0.139758157	0.6666878	4.143433488	4.65E-05	9.67E-05	0.963198377
FAM13B	-0.328663978	3.87775968	-4.142761619	4.66E-05	9.70E-05	0.96059083
EPOP	0.442225904	2.03826047	4.142214748	4.67E-05	9.72E-05	0.958468675
LRFN4	0.319580013	2.54415993	4.140986685	4.70E-05	9.77E-05	0.953704024
MYL4	-0.850110067	4.08761998	-4.140372729	4.71E-05	9.79E-05	0.951322456

TSPO2	0.101103057	0.2220205	4.140239647	4.71E-05	9.80E-05	0.950806264
PSMC3IF	-0.233050814	1.99659482	-4.140035078	4.72E-05	9.80E-05	0.95001282
NME6	-0.226918197	3.28472366	-4.139475141	4.73E-05	9.82E-05	0.947841214
UBA3	-0.261927008	4.8289536	-4.138362203	4.75E-05	9.87E-05	0.943525674
USP22	-0.320363504	6.59288975	-4.138333396	4.75E-05	9.87E-05	0.943413984
EXO5	-0.228141799	2.51919303	-4.137845424	4.76E-05	9.89E-05	0.941522148
CELF2	0.475591217	3.23243424	4.137512842	4.77E-05	9.90E-05	0.940232861
CASQ2	-0.423112197	1.58162987	-4.13732567	4.77E-05	9.90E-05	0.939507308
MSN	0.386174436	8.69042412	4.137027088	4.77E-05	9.91E-05	0.938349948
VPS36	-0.299421465	4.74606348	-4.136636313	4.78E-05	9.93E-05	0.936835343
BCHE	-0.692688809	1.10992192	-4.13662897	4.78E-05	9.93E-05	0.936806883
INTS11	-0.24069956	5.14796704	-4.136494731	4.79E-05	9.93E-05	0.936286615
RNF103	-0.245076626	3.80449481	-4.136340724	4.79E-05	9.94E-05	0.935689753
TCF7	0.514392085	3.00576438	4.135347057	4.81E-05	9.98E-05	0.931839197
HERC1	-0.337318378	3.77657715	-4.134845065	4.82E-05	1.00E-04	0.929894237
RNF175	0.239958649	0.70497228	4.134088224	4.83E-05	0.0001	0.926962259
DHFR	-0.299063821	2.55394343	-4.133763143	4.84E-05	0.0001	0.925703053
ZNF776	-0.309860656	3.19370929	-4.133732271	4.84E-05	0.0001	0.92558347
ZNF37A	-0.284650344	3.07194137	-4.133344068	4.85E-05	0.0001	0.924079881
DOCK2	0.440835389	1.56541191	4.13323817	4.85E-05	0.0001	0.923669738
PROSER	0.391820135	3.44250065	4.132962608	4.85E-05	0.0001	0.922602527
PLAA	-0.26402311	4.32862383	-4.132859618	4.86E-05	0.0001	0.92220368
FILIP1	-0.386677293	1.70491988	-4.132707097	4.86E-05	0.0001	0.92161303
GGCT	0.489804694	6.7407635	4.132529575	4.86E-05	0.0001	0.920925584
FAM86B2	-0.175427074	0.55958282	-4.131621555	4.88E-05	0.0001	0.917409722
UPB1	-0.069576395	0.13031502	-4.131515959	4.88E-05	0.0001	0.917000898
PRPF40B	-0.303816636	2.56630892	-4.131066429	4.89E-05	0.0001	0.915260603
FLI1	-0.329991322	2.88856009	-4.128504552	4.94E-05	0.0001	0.905345804
TTLL12	-0.290897763	4.09955029	-4.127904911	4.96E-05	0.0001	0.903025892
KMO	0.211545751	0.53036984	4.127784344	4.96E-05	0.0001	0.902559473
MYL12B	-0.26889267	9.87969347	-4.127393694	4.97E-05	0.0001	0.90104831
UBE2Z	-0.279095693	6.27329581	-4.127319633	4.97E-05	0.0001	0.900761832
GPALPP	-0.247960642	2.94023491	-4.127105188	4.97E-05	0.0001	0.899932351
DKK2	0.374121247	1.21754865	4.127055321	4.97E-05	0.0001	0.899739472
IPO4	-0.19988714	2.06587057	-4.125560086	5.00E-05	0.0001	0.89395698
PLPP4	0.482816682	0.94740027	4.124428527	5.03E-05	0.0001	0.88958214
SLC25A5	-0.234086258	2.82358025	-4.124391274	5.03E-05	0.0001	0.889438132
POLE3	-0.272251429	5.7861719	-4.122706986	5.06E-05	0.0001	0.882928329
ARMH4	-0.29490765	1.47152397	-4.120756135	5.10E-05	0.00011	0.875391162
CCDC3	-0.498978883	3.82532618	-4.120392642	5.11E-05	0.00011	0.873987142

FAM104A	-0.273563029	3.87621083	-4.119943929	5.12E-05	0.00011	0.872254102
HMCN2	-0.326938966	0.6664622	-4.119777132	5.12E-05	0.00011	0.871609933
TST	-0.315662219	5.47417364	-4.119596661	5.13E-05	0.00011	0.870912979
DENND2	-0.195539537	1.28487074	-4.119594309	5.13E-05	0.00011	0.870903897
XPOT	-0.411891056	5.15039576	-4.119262773	5.13E-05	0.00011	0.869623626
FAM229A	-0.361495469	1.96994809	-4.118535305	5.15E-05	0.00011	0.866814724
TBC1D10	-0.21468996	4.83617204	-4.11705326	5.18E-05	0.00011	0.86109359
CLTC	-0.358642146	6.74665581	-4.116729547	5.19E-05	0.00011	0.859844202
WIPF3	-0.418086184	2.51197648	-4.116553297	5.19E-05	0.00011	0.859163992
ZFP36L1	0.53828708	8.52122771	4.115616408	5.21E-05	0.00011	0.855548637
FAM155A	-0.394148923	0.94354689	-4.11528722	5.22E-05	0.00011	0.854278504
MRNIP	-0.315733773	3.2458912	-4.115138013	5.22E-05	0.00011	0.853702837
SLC18A3	0.509301462	0.59663692	4.115038482	5.22E-05	0.00011	0.853318838
GART	-0.290787857	4.0449598	-4.115034717	5.22E-05	0.00011	0.853304312
LURAP1	-0.392972474	3.39846199	-4.113448385	5.26E-05	0.00011	0.847185228
SNCG	0.628770472	4.83468958	4.112329112	5.28E-05	0.00011	0.842869005
GPR17	-0.189238839	0.4886459	-4.112284098	5.28E-05	0.00011	0.842695441
ZNF136	-0.278095076	3.32505045	-4.112276421	5.28E-05	0.00011	0.842665842
DENND3	-0.237223477	2.76769113	-4.111963685	5.29E-05	0.00011	0.841460042
SGIP1	-0.539662472	1.52114372	-4.111308244	5.30E-05	0.00011	0.83893316
MAPKAP	-0.20125912	2.96830278	-4.111089074	5.31E-05	0.00011	0.838088286
COL14A1	-0.690529102	5.94009617	-4.11066751	5.32E-05	0.00011	0.836463316
SLC2A13	0.351582347	1.95123572	4.110182427	5.33E-05	0.00011	0.834593684
METTL26	-0.300649327	5.95148125	-4.110002683	5.33E-05	0.00011	0.833900956
UBA1	-0.251369449	7.77040944	-4.109670439	5.34E-05	0.00011	0.832620568
ZSWIM4	0.314367821	3.61650053	4.109485369	5.34E-05	0.00011	0.831907388
RAD21	-0.336585679	6.3087131	-4.109396747	5.34E-05	0.00011	0.83156589
MPL	-0.149918944	0.54725461	-4.109092781	5.35E-05	0.00011	0.830394623
TCF15	-0.320954585	0.86542904	-4.108484508	5.36E-05	0.00011	0.828051006
ZNF366	-0.405942107	1.55723156	-4.108476396	5.36E-05	0.00011	0.828019754
KCNJ6	0.04031986	0.03480373	4.106543532	5.41E-05	0.00011	0.820574643
GPR68	0.463982307	1.63593769	4.105875874	5.42E-05	0.00011	0.818003635
NAB2	0.600063965	6.7962216	4.105324168	5.43E-05	0.00011	0.815879413
BRD9	-0.218851411	3.50001911	-4.104798999	5.44E-05	0.00011	0.813857594
NEK6	0.539331028	3.32650643	4.104605523	5.45E-05	0.00011	0.813112797
LSS	-0.23914777	3.99927738	-4.104455967	5.45E-05	0.00011	0.812537097
ZNF746	-0.259726342	3.19291234	-4.104140829	5.46E-05	0.00011	0.811324064
NTNG1	0.04548122	0.05951882	4.104135637	5.46E-05	0.00011	0.811304076
HNRNPL	-0.272479001	7.1293775	-4.103928815	5.46E-05	0.00011	0.810508021
CCN5	0.54879738	0.96831148	4.103438654	5.47E-05	0.00011	0.808621532

LSMEM1	-0.256775503	1.16977094	-4.101755671	5.51E-05	0.00011	0.802145721
LACTB	-0.21755745	3.4002202	-4.101402716	5.52E-05	0.00011	0.800787911
SESTD1	0.293594077	2.20007043	4.101262557	5.52E-05	0.00011	0.800248749
PMS2	-0.308710379	3.06130143	-4.099970411	5.55E-05	0.00011	0.79527891
SPICE1	-0.290116388	2.59367128	-4.099753985	5.56E-05	0.00011	0.794446629
PGM1	-0.361684164	5.40056609	-4.098958661	5.57E-05	0.00011	0.791388487
VSTM2L	0.739841672	3.75706989	4.098918104	5.58E-05	0.00011	0.791232553
CSDE1	-0.305397838	8.27820832	-4.0984767	5.59E-05	0.00011	0.789535525
ZYG11A	-0.363028374	1.58017075	-4.097655335	5.60E-05	0.00012	0.786378121
BMP2	-0.534993071	4.2067215	-4.096913892	5.62E-05	0.00012	0.783528421
SAP130	-0.271508898	4.21780578	-4.096874415	5.62E-05	0.00012	0.783376706
IFITM3	-0.421410385	8.41413568	-4.096518529	5.63E-05	0.00012	0.782009047
CYSLTR:	0.931007208	3.38648134	4.09435984	5.68E-05	0.00012	0.773715506
VAMP3	-0.269585578	6.92269158	-4.093629656	5.70E-05	0.00012	0.770911056
KCTD9	-0.334368507	3.82859931	-4.093529396	5.70E-05	0.00012	0.77052602
ZNF561	-0.272522578	3.83716898	-4.092756984	5.72E-05	0.00012	0.767559921
AZIN1	-0.321321124	5.89059624	-4.0923639	5.73E-05	0.00012	0.76605065
GTF2F2	-0.209514184	4.51679718	-4.091289713	5.75E-05	0.00012	0.761926887
NCK2	-0.321207132	6.22256294	-4.090154326	5.78E-05	0.00012	0.757569208
CCNYL1	-0.332474992	3.86114895	-4.089962453	5.78E-05	0.00012	0.756832894
ZNF397	-0.251957147	3.53606104	-4.089709308	5.79E-05	0.00012	0.755861495
TLR3	-0.350013413	2.34662119	-4.089022723	5.80E-05	0.00012	0.753227113
CCDC34	-0.26110822	3.10321129	-4.08826668	5.82E-05	0.00012	0.75032667
PRDM6	-0.245908157	0.89797166	-4.08824285	5.82E-05	0.00012	0.750235256
ALPK2	0.129343265	0.1337199	4.086359598	5.87E-05	0.00012	0.743012537
SPART	-0.308219676	5.03502793	-4.085362762	5.89E-05	0.00012	0.739190614
SLFN5	0.409308545	3.90760438	4.085265216	5.89E-05	0.00012	0.738816661
PDGFRA	0.489535694	1.29677498	4.085066941	5.90E-05	0.00012	0.738056578
NT5DC2	-0.390576192	5.00164226	-4.084046931	5.92E-05	0.00012	0.7341469
FAM177/	-0.29730719	4.789472	-4.081058203	5.99E-05	0.00012	0.722696097
SLC36A4	-0.229857221	2.27549231	-4.080007093	6.02E-05	0.00012	0.718670697
MMGT1	-0.406591964	4.96755543	-4.079636832	6.03E-05	0.00012	0.717252936
USP18	-0.393371063	3.09379524	-4.07913266	6.04E-05	0.00012	0.715322601
CXorf21	0.414325187	1.58064135	4.079063496	6.04E-05	0.00012	0.715057807
RBM22	-0.236862193	5.39261607	-4.078395112	6.06E-05	0.00012	0.712499112
MCF2L2	0.080041487	0.20092427	4.078165175	6.06E-05	0.00012	0.711618957
PLXNB3	-0.170580686	0.48179102	-4.076387582	6.11E-05	0.00013	0.704816136
ASH2L	-0.246132468	4.49111004	-4.076308695	6.11E-05	0.00013	0.704514295
WDPCP	-0.163400875	1.16816606	-4.075535382	6.13E-05	0.00013	0.701555701
NR2E3	-0.105139556	0.33606906	-4.075180028	6.14E-05	0.00013	0.700196328

ACP2	-0.232682336	5.35302676	-4.074350171	6.16E-05	0.00013	0.697022197
VPS11	-0.2288707	5.33833326	-4.074192733	6.16E-05	0.00013	0.696420073
TCF7L2	-0.33501644	4.12858586	-4.073787665	6.17E-05	0.00013	0.694870981
TNNI3K	-0.064729618	0.13555519	-4.073671886	6.17E-05	0.00013	0.694428233
B4GALN⁻	-0.517846526	0.9575098	-4.073447613	6.18E-05	0.00013	0.69357063
RS1	-0.138141323	0.42821248	-4.07283585	6.20E-05	0.00013	0.691231507
PDPR	-0.332728187	3.70112429	-4.072691001	6.20E-05	0.00013	0.690677712
DEPDC4	-0.131707862	0.71202758	-4.071857056	6.22E-05	0.00013	0.687489652
GNG8	0.366731254	0.67419037	4.071664475	6.22E-05	0.00013	0.686753524
TNFAIP6	0.463578592	1.62648743	4.070653893	6.25E-05	0.00013	0.682891134
MRPL9	-0.250907429	6.1572222	-4.070633738	6.25E-05	0.00013	0.682814114
PLA2G2/	0.882102687	1.64194652	4.069982375	6.27E-05	0.00013	0.680325096
SOX7	-0.373843776	2.23055059	-4.069756602	6.27E-05	0.00013	0.679462446
GSDMA	0.46884465	1.24785444	4.069354036	6.28E-05	0.00013	0.677924394
BMP5	-0.198824446	0.29460721	-4.067432148	6.33E-05	0.00013	0.670583424
POLR2M	-0.313060595	4.2727292	-4.067050031	6.34E-05	0.00013	0.669124228
FAM222/	-0.269086954	1.47669217	-4.066302365	6.36E-05	0.00013	0.666269453
ADPRHL	-0.219497122	6.43194592	-4.064398795	6.41E-05	0.00013	0.659003231
ALX4	-0.152923446	0.16945446	-4.062758143	6.45E-05	0.00013	0.652743005
PGR	0.397396275	0.93244961	4.060468558	6.51E-05	0.00013	0.64401036
RRP12	-0.224981887	3.6445121	-4.060320636	6.52E-05	0.00013	0.643446322
ZNF551	-0.282202135	2.6462598	-4.058878457	6.55E-05	0.00013	0.637948144
ARAP3	-0.349440252	3.6444323	-4.058158153	6.57E-05	0.00013	0.635202692
IMPA1	-0.319362891	4.20872674	-4.057502112	6.59E-05	0.00013	0.632702549
AZIN2	-0.348463213	2.48009148	-4.057098929	6.60E-05	0.00013	0.631166217
UTS2B	-0.043451717	0.10993225	-4.056918066	6.61E-05	0.00013	0.630477078
ZNF638	-0.285259165	4.83958618	-4.056539643	6.62E-05	0.00014	0.629035268
CEP152	-0.220415696	1.23244237	-4.054660324	6.67E-05	0.00014	0.621876734
GAK	-0.1889457	4.42132911	-4.054366085	6.67E-05	0.00014	0.620756211
SYNJ1	-0.318736444	2.95172446	-4.054046988	6.68E-05	0.00014	0.619541101
LLGL1	0.416239183	4.05985728	4.053666027	6.69E-05	0.00014	0.618090528
CYP2D6	-0.347897015	1.30606025	-4.05316758	6.71E-05	0.00014	0.616192785
MTA2	-0.235560697	5.60899912	-4.051940566	6.74E-05	0.00014	0.611522038
FKBP14	-0.259021537	2.67932251	-4.051119571	6.76E-05	0.00014	0.608397533
BRINP2	0.466635289	0.92436586	4.050438545	6.78E-05	0.00014	0.605806141
CYP26A1	0.236384018	0.33615164	4.049883555	6.79E-05	0.00014	0.603694615
ENOPH1	-0.254341348	6.18040716	-4.047875824	6.85E-05	0.00014	0.596058084
TNNI1	0.335575141	1.43186054	4.04565463	6.91E-05	0.00014	0.587613514
THRSP	-0.777560773	5.17501987	-4.04501134	6.93E-05	0.00014	0.585168606
RPS28	-0.345041347	8.71904189	-4.044135	6.95E-05	0.00014	0.581838508

POGLUT	-0.248658707	2.38649389	-4.044037555	6.96E-05	0.00014	0.581468257
CCDC14	-0.065065989	0.16680265	-4.043874396	6.96E-05	0.00014	0.580848337
UNK	-0.258034643	4.36791143	-4.043494328	6.97E-05	0.00014	0.579404354
TMOD2	-0.330224836	2.08404454	-4.043376979	6.97E-05	0.00014	0.578958537
OSM	0.471885168	1.4147987	4.043375386	6.97E-05	0.00014	0.578952487
INTS4	-0.214313998	3.50280938	-4.043235488	6.98E-05	0.00014	0.578421021
TRIM50	-0.374441096	0.4365372	-4.043187379	6.98E-05	0.00014	0.57823826
S100Z	0.087090431	0.22091172	4.041837003	7.02E-05	0.00014	0.573109123
GGT2	0.501753426	1.863512	4.041022938	7.04E-05	0.00014	0.570017787
MYOZ1	-0.538559608	2.99723184	-4.040745096	7.05E-05	0.00014	0.568962836
NRDC	-0.195797795	5.58747237	-4.039793723	7.08E-05	0.00014	0.565350997
DNAJC1:	-0.34144093	4.25451748	-4.039016968	7.10E-05	0.00014	0.56240264
SHOC2	-0.268192057	5.23432901	-4.03895366	7.10E-05	0.00014	0.562162364
SLC30A2	0.78254373	5.39405955	4.038676636	7.11E-05	0.00014	0.561110988
REPS2	-0.344179587	1.99358182	-4.0385609	7.11E-05	0.00014	0.560671761
KCND3	-0.242941775	0.50060809	-4.038469723	7.11E-05	0.00014	0.560325746
PTGER1	0.279799353	0.67261313	4.038019316	7.13E-05	0.00014	0.558616551
SALL1	-0.572259464	5.37364483	-4.038015287	7.13E-05	0.00014	0.558601263
BRWD1	-0.288239581	3.45763333	-4.037860225	7.13E-05	0.00015	0.558012877
RNF216	-0.258801705	4.67463226	-4.037723682	7.13E-05	0.00015	0.557494778
ZNF233	-0.3216917	1.70209971	-4.037680443	7.14E-05	0.00015	0.557330716
SLC19A3	0.133870767	0.17917664	4.037668016	7.14E-05	0.00015	0.557283566
SPRED3	0.27635413	1.37328551	4.037101328	7.15E-05	0.00015	0.555133514
TMEM17	0.512138877	5.87587019	4.03661256	7.17E-05	0.00015	0.553279311
DDX39A	-0.223774177	4.66125463	-4.036381703	7.17E-05	0.00015	0.552403595
ACTL10	-0.292072051	2.28407369	-4.035909844	7.19E-05	0.00015	0.550613815
HSF2	-0.228825071	3.78907983	-4.035852742	7.19E-05	0.00015	0.550397238
P2RX1	0.292118041	0.59493913	4.034800908	7.22E-05	0.00015	0.546408304
RINT1	-0.234948543	3.57497877	-4.034092395	7.24E-05	0.00015	0.543721883
ZNF473	-0.219894761	2.47412322	-4.03348714	7.26E-05	0.00015	0.541427306
PCDHGA	-0.403412174	1.64667246	-4.033460165	7.26E-05	0.00015	0.541325047
ZNF394	-0.187785002	4.23810546	-4.032746855	7.28E-05	0.00015	0.538621231
HSPA6	0.577936914	1.82816836	4.03106581	7.33E-05	0.00015	0.532250856
SNX31	-0.147521766	0.2481824	-4.029897919	7.36E-05	0.00015	0.527826469
IL18R1	0.586157184	1.85829992	4.029464193	7.37E-05	0.00015	0.52618365
TMEM12	-0.242667884	5.75785823	-4.029357213	7.38E-05	0.00015	0.525778467
STIMATE	-0.164418264	1.36010774	-4.029321668	7.38E-05	0.00015	0.525643842
BEND7	-0.348804986	3.89609759	-4.028712748	7.40E-05	0.00015	0.523337776
LRRC41	-0.273260163	5.17350282	-4.028195794	7.41E-05	0.00015	0.521380238
PABPN1	-0.287830204	6.34967381	-4.028025676	7.42E-05	0.00015	0.520736108

THY1	-0.473697451	5.337405	-4.027812535	7.42E-05	0.00015	0.519929105
RPS24	-0.269370765	9.26812622	-4.027715652	7.43E-05	0.00015	0.519562296
LILRB4	0.524098381	2.140065	4.025734184	7.48E-05	0.00015	0.512061955
SREK1	-0.29567267	3.75290697	-4.024916392	7.51E-05	0.00015	0.508967358
TYROBP	0.593324239	6.18878398	4.024039695	7.54E-05	0.00015	0.505650474
CLEC10A	0.568671236	1.8907811	4.023467028	7.55E-05	0.00015	0.5034842
DYNC2H	-0.336889073	2.43471865	-4.023452294	7.55E-05	0.00015	0.503428469
DCC	0.081611705	0.07553308	4.023101634	7.56E-05	0.00015	0.502102134
TGOLN2	-0.412963303	7.08309446	-4.022882909	7.57E-05	0.00015	0.501274884
IGF2BP2	0.793389233	4.69461937	4.022846164	7.57E-05	0.00015	0.501135912
POLM	-0.267189242	4.04987929	-4.022559847	7.58E-05	0.00015	0.500053084
ZNF316	-0.245343159	5.04622564	-4.021661336	7.61E-05	0.00015	0.496655432
RHOQ	-0.249556321	5.36464274	-4.019765972	7.67E-05	0.00016	0.489490448
PCYT1A	-0.238370553	4.27093175	-4.01938244	7.68E-05	0.00016	0.488040957
AMPD1	0.3390393	0.38639854	4.019327436	7.68E-05	0.00016	0.487833089
TFDP2	-0.276901547	4.18585505	-4.018546782	7.70E-05	0.00016	0.484883155
UBXN1	-0.253540957	7.51643719	-4.018399046	7.71E-05	0.00016	0.484324946
DCAF4L1	-0.051397894	0.14830259	-4.018254553	7.71E-05	0.00016	0.48377901
LYL1	-0.342640113	2.33054756	-4.01766576	7.73E-05	0.00016	0.481554559
UBALD2	-0.252659466	6.15872682	-4.0168842	7.75E-05	0.00016	0.478602278
FBXL8	-0.273191078	2.66510208	-4.016672178	7.76E-05	0.00016	0.477801467
NETO1	0.468988963	0.69746356	4.0164541	7.77E-05	0.00016	0.476977824
ROBO3	-0.348191563	1.34847075	-4.016432676	7.77E-05	0.00016	0.476896911
ENDOD1	0.508878218	5.88200227	4.015979121	7.78E-05	0.00016	0.475184047
LETM2	-0.119109778	0.72091911	-4.015953863	7.78E-05	0.00016	0.475088662
CHD8	-0.298008921	4.497063	-4.015106204	7.81E-05	0.00016	0.471887926
TCF20	-0.330512794	4.6549123	-4.014781004	7.82E-05	0.00016	0.470660134
TMPRSS	0.644461446	3.19226665	4.013090459	7.87E-05	0.00016	0.464278915
MFSD12	-0.230183951	4.27390366	-4.011622567	7.92E-05	0.00016	0.458740052
SCLT1	-0.166949767	1.78168914	-4.010947199	7.94E-05	0.00016	0.456192254
PARP14	0.425794021	5.35626891	4.010833769	7.94E-05	0.00016	0.455764383
LONP1	-0.268275841	6.03298646	-4.010785279	7.94E-05	0.00016	0.455581472
S1PR2	0.408500815	3.27443403	4.009948622	7.97E-05	0.00016	0.452425859
ZNF461	-0.245718248	2.41162544	-4.009927796	7.97E-05	0.00016	0.452347317
PRSS2	1.153761563	2.61236649	4.009295623	7.99E-05	0.00016	0.449963348
SBK1	-0.346419887	3.37001042	-4.00865953	8.01E-05	0.00016	0.447564931
ZNF791	-0.233591498	4.20793496	-4.00743445	8.05E-05	0.00016	0.442946656
SGCD	-0.722939591	2.22142967	-4.005971983	8.10E-05	0.00016	0.43743512
SLAMF9	0.177209709	0.21151479	4.005384775	8.12E-05	0.00016	0.435222631
MTHFD1	-0.248203165	4.28777594	-4.005339832	8.12E-05	0.00016	0.435053308

RPSA	-0.297109925	8.47258723	-4.005268986	8.12E-05	0.00016	0.434786395
C5orf58	0.079876393	0.17061094	4.004832319	8.14E-05	0.00016	0.433141345
MASTL	-0.235326806	2.95373304	-4.004412789	8.15E-05	0.00016	0.431561003
MDK	0.775291332	7.13415654	4.003884718	8.17E-05	0.00016	0.429572003
SLAMF6	0.601695247	1.55817985	4.002880186	8.20E-05	0.00017	0.425789031
RNF214	-0.272496845	3.50443255	-4.002840593	8.20E-05	0.00017	0.425639945
GALNT7	0.555576136	5.10809953	4.002519089	8.21E-05	0.00017	0.424429376
SGSM2	-0.282531205	4.46952698	-4.002408182	8.21E-05	0.00017	0.424011796
RAP2B	0.295012891	4.24113568	4.001803408	8.23E-05	0.00017	0.421734914
RFLNA	0.24791566	0.83129984	4.001309913	8.25E-05	0.00017	0.419877207
SFRP4	0.958656895	2.99466783	4.000092476	8.29E-05	0.00017	0.415295163
KIF1C	-0.267927242	6.33684684	-3.99972539	8.30E-05	0.00017	0.413913813
RPL38	-0.264233886	7.95623681	-3.999648404	8.31E-05	0.00017	0.413624126
PTK2	-0.270001194	4.60333435	-3.999199018	8.32E-05	0.00017	0.411933255
PPIG	-0.28365733	5.1139665	-3.999057069	8.32E-05	0.00017	0.411399193
STARD5	-0.221327472	1.04455892	-3.998938072	8.33E-05	0.00017	0.410951493
WFDC2	-0.619469894	8.19303651	-3.998855758	8.33E-05	0.00017	0.410641812
SLC22A1	-0.503581299	5.5978618	-3.99834805	8.35E-05	0.00017	0.408731847
ACSL6	-0.165237743	0.24052599	-3.997263309	8.38E-05	0.00017	0.404651834
CRK	-0.25400551	6.11210409	-3.997127123	8.39E-05	0.00017	0.404139668
ZBTB47	-0.319833246	4.9453806	-3.996848136	8.40E-05	0.00017	0.40309051
DIDO1	-0.278600178	4.18355575	-3.996658608	8.40E-05	0.00017	0.402377809
IGFN1	1.166895621	2.4168805	3.996598795	8.41E-05	0.00017	0.402152894
CD9	-0.379931843	8.62355914	-3.996537272	8.41E-05	0.00017	0.401921551
GADD45	-0.603706251	6.76743597	-3.996181159	8.42E-05	0.00017	0.400582539
DMRT3	-0.827857479	3.30268033	-3.996011559	8.43E-05	0.00017	0.399944869
SETX	-0.381008856	4.94498089	-3.995825416	8.43E-05	0.00017	0.399245024
LRP1	0.451537325	4.41265684	3.995548126	8.44E-05	0.00017	0.398202547
CD99	-0.305540657	6.38658044	-3.995513373	8.44E-05	0.00017	0.398071896
TEX22	-0.168441341	0.61757786	-3.995459613	8.44E-05	0.00017	0.397869792
LMX1B	-0.325394088	0.33537893	-3.994533424	8.48E-05	0.00017	0.394388299
PZP	-0.050128774	0.14773328	-3.994394703	8.48E-05	0.00017	0.393866917
FOSL1	0.684262931	2.90417268	3.994303985	8.48E-05	0.00017	0.393525959
ADGRL1	-0.314161985	5.03471476	-3.993494533	8.51E-05	0.00017	0.390484012
ZNF324E	-0.204080059	2.24333883	-3.993216834	8.52E-05	0.00017	0.389440535
RPS6KA	-0.140217872	0.6593983	-3.991959884	8.56E-05	0.00017	0.384718239
SIDT2	-0.255263598	4.39202152	-3.990880508	8.60E-05	0.00017	0.380664127
RNF212E	-0.141151038	0.57734027	-3.990683936	8.61E-05	0.00017	0.379925913
LNPK	-0.265974844	3.20073214	-3.99050505	8.61E-05	0.00017	0.379254141
NCAPD3	-0.28998713	3.05321223	-3.989842117	8.63E-05	0.00017	0.376764868

TBX5	-0.278140663	0.32788578	-3.98920183	8.66E-05	0.00017	0.374360973
DRP2	0.056772131	0.06641515	3.989193111	8.66E-05	0.00017	0.374328241
TFE3	-0.291682065	6.21872716	-3.988637386	8.68E-05	0.00017	0.372242109
DHRS3	0.61556275	8.35676525	3.988548928	8.68E-05	0.00017	0.37191007
FAM217E	-0.272488573	3.53925225	-3.988255268	8.69E-05	0.00017	0.370807823
MAPKBP	-0.339946778	3.19418705	-3.987647322	8.71E-05	0.00017	0.368526146
WIPF1	0.343997093	3.92416458	3.986842078	8.74E-05	0.00018	0.36550446
REEP6	-0.447385828	4.25442602	-3.986080361	8.76E-05	0.00018	0.362646605
LAX1	0.518502316	0.84628941	3.984368628	8.82E-05	0.00018	0.356226184
C17orf11	-0.378486003	2.13069495	-3.983855786	8.84E-05	0.00018	0.354303074
AMBP	-0.250361793	0.35146274	-3.983782967	8.84E-05	0.00018	0.354030027
KANK2	0.366349469	5.68938397	3.982505499	8.89E-05	0.00018	0.349240667
MBIP	-0.393939847	6.40601431	-3.980529221	8.96E-05	0.00018	0.341834071
TBXAS1	0.388200506	1.99974001	3.980331749	8.97E-05	0.00018	0.341094175
WASHC1	-0.342154224	3.38225789	-3.979918377	8.98E-05	0.00018	0.339545436
SPATC1	0.094232898	0.19114189	3.979718709	8.99E-05	0.00018	0.338797412
ARCN1	-0.302093573	6.96417816	-3.979717241	8.99E-05	0.00018	0.338791913
C17orf10	-0.2652145	3.24036535	-3.979515576	9.00E-05	0.00018	0.338036444
CASC4	-0.350867843	6.30107189	-3.977527815	9.07E-05	0.00018	0.33059176
CTPS2	-0.248832886	3.59583604	-3.976757126	9.09E-05	0.00018	0.327706214
SLC7A5	0.580664976	3.03840458	3.976063329	9.12E-05	0.00018	0.325108982
ZNF786	-0.257422072	3.20254414	-3.976017852	9.12E-05	0.00018	0.324938752
CD69	0.592370005	2.08748498	3.975895003	9.13E-05	0.00018	0.324478913
C11orf88	-0.099772697	0.14907083	-3.975809844	9.13E-05	0.00018	0.324160158
SUCNR1	-0.495904014	2.06017755	-3.973806998	9.20E-05	0.00018	0.316665162
DDAH1	-0.325603831	5.97494626	-3.973629851	9.21E-05	0.00018	0.316002407
ELOA2	-0.042977219	0.04088469	-3.973595189	9.21E-05	0.00018	0.315872732
SLC22A1	-0.284332755	4.59309071	-3.97327801	9.22E-05	0.00018	0.314686156
SASH3	0.571445757	3.03650381	3.973030908	9.23E-05	0.00018	0.313761797
LPP	-0.293128601	2.77823188	-3.972977031	9.23E-05	0.00018	0.313560259
BIVM-ER	-0.235969961	3.89599155	-3.972575927	9.25E-05	0.00019	0.312059936
ERCC5	-0.235969961	3.89599155	-3.972575927	9.25E-05	0.00019	0.312059936
ITGA4	0.429517837	1.43534435	3.972553145	9.25E-05	0.00019	0.311974728
LHFPL2	-0.346178131	3.86840955	-3.971356699	9.29E-05	0.00019	0.307500259
CCNI	-0.342434453	8.90980125	-3.971312764	9.29E-05	0.00019	0.307335973
ZFP14	-0.251919583	2.89371471	-3.971141918	9.30E-05	0.00019	0.306697144
CCL19	1.204868705	2.6618207	3.971022425	9.30E-05	0.00019	0.306250353
DNMT3A	-0.267293378	2.95623378	-3.970724723	9.31E-05	0.00019	0.305137275
DZIP1	0.396211689	1.31184734	3.970493225	9.32E-05	0.00019	0.30427178
ATP10A	0.461351144	2.0254799	3.970147397	9.34E-05	0.00019	0.302978921

INTS5	-0.241195647	4.93860296	-3.969198823	9.37E-05	0.00019	0.299433244
LIN54	-0.292686532	3.41032697	-3.969168891	9.37E-05	0.00019	0.299321374
FOXD4L:	-0.080693788	0.16276767	-3.969056467	9.38E-05	0.00019	0.298901198
BPIFB2	-0.100668258	0.10584101	-3.969010866	9.38E-05	0.00019	0.298730769
SLC35A5	-0.324886762	4.92729356	-3.968586224	9.39E-05	0.00019	0.29714381
DCUN1D	-0.202220726	2.44233137	-3.9678284	9.42E-05	0.00019	0.29431206
MLNR	-0.229965639	0.29675704	-3.967747651	9.42E-05	0.00019	0.294010357
RASSF5	0.35758938	3.96819631	3.967611882	9.43E-05	0.00019	0.293503091
TAGLN2	0.404629096	8.53334939	3.967404112	9.44E-05	0.00019	0.292726841
ARL15	-0.297024798	4.16707999	-3.966902815	9.46E-05	0.00019	0.290854093
MSC	0.45306563	1.48418053	3.966511087	9.47E-05	0.00019	0.289390819
ZNF131	-0.22816238	3.06110873	-3.965967992	9.49E-05	0.00019	0.287362338
CD7	0.591684412	1.98857487	3.964704381	9.54E-05	0.00019	0.282643644
RPH3AL	-0.276378922	4.11273942	-3.964626125	9.54E-05	0.00019	0.282351455
RNASEK	-0.200839367	3.7264327	-3.963145216	9.60E-05	0.00019	0.2768231
ZNF668	-0.20536933	1.6925228	-3.962925401	9.61E-05	0.00019	0.276002666
STON1-C	-0.082375998	0.11096418	-3.962437124	9.62E-05	0.00019	0.274180377
ZNF385B	0.173249356	0.24234621	3.962093373	9.64E-05	0.00019	0.272897589
PDK2	-0.369677258	4.73088326	-3.961024737	9.68E-05	0.00019	0.26891035
LARP1	-0.294252865	6.52474214	-3.96040212	9.70E-05	0.00019	0.266587711
HIST2H3	-0.176723475	0.44314392	-3.960310224	9.71E-05	0.00019	0.266244926
IL12RB1	0.354217133	1.13850914	3.959369619	9.74E-05	0.00019	0.262736739
C20orf19	-0.301210934	3.8133668	-3.958944107	9.76E-05	0.00019	0.261149942
ZNF419	-0.218590422	2.42323426	-3.958815935	9.76E-05	0.00019	0.260671999
NDC1	-0.269264917	3.44824059	-3.958761015	9.76E-05	0.00019	0.26046721
SLCO1C	-0.22337041	0.78764692	-3.958742247	9.77E-05	0.00019	0.260397228
CCDC11:	-0.282285242	2.27071871	-3.958660648	9.77E-05	0.00019	0.260092966
S100A9	0.737987453	4.75348209	3.958462076	9.78E-05	0.00019	0.259352557
BTBD16	-0.079839966	0.14663116	-3.957606382	9.81E-05	0.0002	0.256162351
LAMC2	0.465805721	6.44212263	3.956821321	9.84E-05	0.0002	0.253236014
MLIP	-0.179840602	0.44180488	-3.956562993	9.85E-05	0.0002	0.252273198
TRIM6	-0.438034332	2.53844269	-3.956213069	9.86E-05	0.0002	0.250969087
HMSD	-0.199933025	0.49529992	-3.955954274	9.87E-05	0.0002	0.250004665
WFDC3	-0.205072155	0.75223547	-3.95578819	9.88E-05	0.0002	0.249385768
CSF1R	0.470140998	3.96246042	3.954648267	9.92E-05	0.0002	0.245138571
SPRED1	-0.376737867	5.30464712	-3.954544355	9.93E-05	0.0002	0.244751459
IZUMO4	-0.318380577	1.38196624	-3.95424222	9.94E-05	0.0002	0.243625954
ZBTB18	-0.229953342	4.36122133	-3.954104735	9.95E-05	0.0002	0.243113822
SPATA1:	-0.272774204	2.13147435	-3.953566359	9.97E-05	0.0002	0.241108526
SH3GLB:	-0.232962393	5.49911668	-3.952897044	9.99E-05	0.0002	0.238615857

LY6D	0.448743231	0.57612781	3.952754964	1.00E-04	0.0002	0.238086768
GPT	-0.457610118	1.93987552	-3.952470481	0.0001	0.0002	0.237027438
SLAMF7	0.758873098	1.90297824	3.951990692	0.0001	0.0002	0.235241005
AMDHD1	-0.202092727	0.59572197	-3.951460078	0.0001	0.0002	0.233265551
IFRD1	-0.304608606	3.91885778	-3.951099006	0.000101	0.0002	0.23192143
ARHGAF	-0.134289112	0.20530439	-3.950531421	0.000101	0.0002	0.229808768
STRADB	0.394794748	4.67743428	3.950358828	0.000101	0.0002	0.229166394
PAQR5	-0.2921024	0.8537522	-3.950327895	0.000101	0.0002	0.22905127
CDV3	-0.337519234	6.87276213	-3.950310876	0.000101	0.0002	0.228987927
SRSF4	-0.239082809	6.45236438	-3.949533312	0.000101	0.0002	0.226094259
KRT79	0.071395751	0.08688611	3.948710376	0.000102	0.0002	0.223032293
MED28	-0.226225787	4.10079039	-3.947992273	0.000102	0.0002	0.220360847
CSAG1	-0.551064585	0.5597642	-3.947548738	0.000102	0.0002	0.218711046
BOC	-0.547621635	3.45802917	-3.947455686	0.000102	0.0002	0.218364946
DACH2	-0.286412477	0.48682574	-3.947364434	0.000102	0.0002	0.218025546
HLA-F	0.484511253	5.25236453	3.947327007	0.000102	0.0002	0.217886344
GLOD5	-0.4304955	2.55037291	-3.946700144	0.000102	0.0002	0.215555024
FCN1	0.490915538	1.38443477	3.946652149	0.000102	0.0002	0.215376542
CBLB	-0.288257505	3.02229922	-3.946464973	0.000102	0.0002	0.214680501
RAET1E	0.343707249	1.04254185	3.946027428	0.000103	0.0002	0.213053537
SNRPA	-0.268571259	6.45016311	-3.945794736	0.000103	0.0002	0.212188365
PRAM1	0.287557171	1.56717394	3.945579104	0.000103	0.0002	0.211386662
NLE1	-0.23581539	3.32505561	-3.945470956	0.000103	0.0002	0.210984591
HINT3	-0.29460855	5.22860881	-3.945160689	0.000103	0.0002	0.209831135
ZNF439	-0.257126991	2.51117669	-3.944312656	0.000103	0.00021	0.206678888
TAFA4	-0.067883579	0.08111064	-3.944291526	0.000103	0.00021	0.206600353
TNS2	-0.275463033	5.61021915	-3.943764781	0.000104	0.00021	0.204642684
TMEM11	0.333969867	3.21569878	3.943204472	0.000104	0.00021	0.202560529
TEX9	-0.264068055	2.39195804	-3.942731283	0.000104	0.00021	0.200802322
PRSS21	0.200304984	0.26476757	3.941755254	0.000104	0.00021	0.197176325
PISD	-0.24686563	4.60771165	-3.941462351	0.000105	0.00021	0.196088332
SCIMP	0.336354144	1.16757913	3.940695774	0.000105	0.00021	0.193241205
PPM1N	0.310313901	0.95380301	3.939598604	0.000105	0.00021	0.189167081
DESI1	-0.201879175	4.31335781	-3.9394066	0.000105	0.00021	0.188454214
PRR3	-0.263211996	3.72679936	-3.938976289	0.000106	0.00021	0.186856682
PRPF6	-0.239250787	7.34830978	-3.938769169	0.000106	0.00021	0.186087804
NFKB2	0.285113917	5.06682902	3.938237164	0.000106	0.00021	0.18411304
SOX8	0.374028306	1.46017838	3.937751492	0.000106	0.00021	0.182310464
TLR6	0.232455679	0.72838847	3.936574869	0.000107	0.00021	0.177944241
BLOC1S(	-0.292827801	4.94922569	-3.932154161	0.000108	0.00021	0.161550149

CDH1	-0.388090088	8.33725045	-3.931755668	0.000109	0.00022	0.160073151
SNAI1	-0.557063465	3.41060048	-3.931427109	0.000109	0.00022	0.158855458
MKI67	0.357431107	1.34577962	3.931221037	0.000109	0.00022	0.158091769
RHOH	0.411915238	0.99303765	3.930936382	0.000109	0.00022	0.157036912
TSPAN4	-0.279402025	5.5339963	-3.930601731	0.000109	0.00022	0.155796871
AFF2	0.319494815	0.81508173	3.930564087	0.000109	0.00022	0.155657389
GPR171	0.416557294	1.03763518	3.930114009	0.000109	0.00022	0.153989799
ZNF277	-0.228973726	4.21991703	-3.929356548	0.00011	0.00022	0.1511837
QRFP	-0.155666235	0.70712671	-3.929145779	0.00011	0.00022	0.150402967
JARID2	-0.251925635	3.12939058	-3.927647283	0.00011	0.00022	0.144853298
CNNM1	-0.122688056	0.1452562	-3.9273532	0.00011	0.00022	0.143764384
PNMA3	-0.378674791	1.22801138	-3.9262337	0.000111	0.00022	0.139619822
PLCG1	-0.28965116	4.75543688	-3.92566215	0.000111	0.00022	0.13750426
PGLS	-0.253337648	5.98836385	-3.925358723	0.000111	0.00022	0.136381253
INF2	0.485032147	5.13703988	3.925220626	0.000111	0.00022	0.135870169
TPSD1	0.232675003	0.27429934	3.925035447	0.000111	0.00022	0.135184866
EPM2AIF	-0.290286468	5.03917553	-3.923972309	0.000112	0.00022	0.131250999
DSTYK	-0.302106187	3.92271815	-3.92372492	0.000112	0.00022	0.130335735
IARS2	-0.331895472	5.97291341	-3.923485126	0.000112	0.00022	0.129448619
RGS20	-0.302225892	0.95194952	-3.922972329	0.000112	0.00022	0.127551692
PIP4K2C	-0.296740252	5.61879016	-3.921992603	0.000113	0.00022	0.123928124
SMOC1	-0.677962999	2.03480089	-3.921680834	0.000113	0.00022	0.122775198
TPPP2	-0.045764746	0.05576079	-3.921494715	0.000113	0.00022	0.122086968
EDC3	-0.254183609	4.30885256	-3.921471573	0.000113	0.00022	0.122001394
ACHE	-0.351508086	1.16724241	-3.921421664	0.000113	0.00022	0.121816849
ZNF620	-0.231245569	2.23977127	-3.921087688	0.000113	0.00022	0.120581963
RDH5	0.597483838	2.1465527	3.920749551	0.000113	0.00022	0.119331786
DNAJC5I	0.229222964	0.49817573	3.920411369	0.000114	0.00022	0.118081542
PCNA	-0.263815247	6.94586268	-3.920369127	0.000114	0.00022	0.117925381
ANGPT1	-0.468454966	1.79199762	-3.919416018	0.000114	0.00023	0.114402321
USF1	-0.220203694	5.87936433	-3.918664708	0.000114	0.00023	0.111625722
DCAF5	-0.246829108	4.64948164	-3.918273322	0.000114	0.00023	0.110179472
C22orf15	-0.096606735	0.17335937	-3.916856318	0.000115	0.00023	0.104944435
PLCD1	0.341355944	4.64505302	3.916672375	0.000115	0.00023	0.10426499
ATOH7	-0.135331028	0.24994238	-3.916427981	0.000115	0.00023	0.103362297
ACCS	-0.349336235	3.33427209	-3.915825343	0.000116	0.00023	0.10113661
CDC42EI	0.437090733	6.27159847	3.915530715	0.000116	0.00023	0.10004859
POMZP3	-0.376349463	3.69271082	-3.914906318	0.000116	0.00023	0.097743018
PI15	0.191783731	0.23965965	3.914093858	0.000116	0.00023	0.094743511
MRPL38	-0.24316054	2.76230692	-3.913904224	0.000116	0.00023	0.094043486

MRI1	-0.303139217	4.03581663	-3.913836931	0.000116	0.00023	0.093795081
PRKACA	-0.237395953	5.86752522	-3.91352478	0.000117	0.00023	0.092642872
OSER1	-0.234453193	5.74688365	-3.913475851	0.000117	0.00023	0.092462274
ARPP19	-0.271815516	6.16465565	-3.913190929	0.000117	0.00023	0.091410652
FBXO43	-0.07749067	0.22445189	-3.912860794	0.000117	0.00023	0.090192241
ANKRD4	-0.270110853	4.67114292	-3.912575585	0.000117	0.00023	0.089139709
RHBDD1	-0.249755525	3.92229159	-3.912400719	0.000117	0.00023	0.088494421
ZFP69	-0.188505028	2.23725645	-3.912212114	0.000117	0.00023	0.087798459
PIK3CB	-0.340375624	4.9703498	-3.911519015	0.000118	0.00023	0.085241147
RAB6C	-0.088251755	0.15771318	-3.91127663	0.000118	0.00023	0.084346919
TJP3	-0.259826617	4.68581474	-3.91067639	0.000118	0.00023	0.082132674
NF2	-0.2253936	4.68573776	-3.910379264	0.000118	0.00023	0.081036707
MYLK2	-0.239044995	0.53995854	-3.910270913	0.000118	0.00023	0.08063707
SNAP23	-0.288782064	4.62600938	-3.909465769	0.000118	0.00023	0.077667694
TRIM62	-0.211826746	2.88867465	-3.908928907	0.000119	0.00023	0.075688043
GDI1	-0.190097722	6.15737155	-3.908834955	0.000119	0.00023	0.075341626
RTL8B	-0.26837765	4.53737123	-3.904496512	0.000121	0.00024	0.059353048
PUM3	-0.218497508	4.43217255	-3.904476383	0.000121	0.00024	0.059278903
PRRC1	-0.293491203	4.61099675	-3.904018858	0.000121	0.00024	0.057593702
PPM1D	-0.261859563	3.40545876	-3.903852887	0.000121	0.00024	0.056982424
MIOS	-0.230679779	3.20364812	-3.903066899	0.000121	0.00024	0.054087909
OBSL1	-0.364925355	5.73714372	-3.902787618	0.000122	0.00024	0.053059543
TRIM25	-0.27802732	4.36690895	-3.902742326	0.000122	0.00024	0.052892773
CRTAC1	-0.61733084	2.87287211	-3.902678318	0.000122	0.00024	0.052657094
CAPRIN2	-0.265884517	2.73611534	-3.90262117	0.000122	0.00024	0.052446678
CPNE3	-0.321425099	5.38946758	-3.902306114	0.000122	0.00024	0.051286701
ELANE	0.33803693	0.45337195	3.902027404	0.000122	0.00024	0.050260613
FBXO32	0.41316648	3.97336419	3.901941314	0.000122	0.00024	0.049943683
GEMIN5	-0.26931401	3.64557887	-3.900815185	0.000123	0.00024	0.045798513
SH2B3	-0.317291319	4.2559147	-3.90038916	0.000123	0.00024	0.044230635
SLC5A1	-0.264143638	0.22421886	-3.900011711	0.000123	0.00024	0.042841655
HMGA1	0.534677795	6.83140076	3.89994229	0.000123	0.00024	0.042586206
DPF2	-0.251607179	4.62156713	-3.899763426	0.000123	0.00024	0.041928054
AKR1B15	-0.329650674	0.31721795	-3.899740446	0.000123	0.00024	0.041843497
NOTCH1	-0.345084086	3.64677073	-3.89956478	0.000123	0.00024	0.041197141
LTBR	-0.21366871	5.72150703	-3.898927346	0.000123	0.00024	0.038851948
8-Mar	-0.284067429	4.30289474	-3.898721798	0.000124	0.00024	0.038095784
SETD2	-0.305933762	4.81476031	-3.897730819	0.000124	0.00024	0.034450705
KRT16	0.421594009	0.54486153	3.897168361	0.000124	0.00024	0.032382203
ZNF524	-0.267439506	5.71066271	-3.894886583	0.000125	0.00025	0.023993433

HCRTR1	-0.105220875	0.34229202	-3.894183404	0.000126	0.00025	0.021409129
RAB35	-0.254546104	4.61843043	-3.894047916	0.000126	0.00025	0.020911237
OPA1	-0.28264046	4.33484586	-3.893794236	0.000126	0.00025	0.019979049
FCRL5	0.38064487	0.48768273	3.892790903	0.000126	0.00025	0.016292676
ZBTB8A	-0.226762447	2.73674251	-3.892612632	0.000127	0.00025	0.015637772
NEK4	-0.34568961	3.49035408	-3.89170331	0.000127	0.00025	0.012297672
KIF12	-0.543846207	4.04687058	-3.891604082	0.000127	0.00025	0.011933232
GTF2H3	-0.306483968	4.23075263	-3.891179073	0.000127	0.00025	0.010372372
CAPN10	-0.235258331	3.1781604	-3.891114552	0.000127	0.00025	0.010135427
ZBTB6	-0.297239008	3.30825943	-3.890808475	0.000127	0.00025	0.009011459
FZD2	-0.414563265	2.9822191	-3.890738451	0.000127	0.00025	0.00875433
SNX21	-0.226282356	3.69812486	-3.890695766	0.000127	0.00025	0.008597594
SERINC5	0.409824523	3.67234006	3.890000869	0.000128	0.00025	0.006046165
LDLRAD3	0.316287845	2.67073611	3.889488527	0.000128	0.00025	0.004165277
SFTA2	0.651353323	2.6081655	3.888335601	0.000129	0.00025	-6.65E-05
ZNF75D	-0.353669983	3.71618413	-3.888328453	0.000129	0.00025	-9.27E-05
FNDC4	0.712021263	4.7734372	3.888191438	0.000129	0.00025	-0.00059555
WDR46	-0.255817744	5.66977606	-3.887989207	0.000129	0.00025	-0.00133769
CRAMP1	-0.256777941	2.79739712	-3.887910875	0.000129	0.00025	-0.00162514
RNF148	-0.076263509	0.18821529	-3.887832428	0.000129	0.00025	-0.00191301
BCL11A	-0.272470527	1.16769643	-3.887802266	0.000129	0.00025	-0.00202369
UHRF1	0.294281381	1.29623002	3.887772285	0.000129	0.00025	-0.00213371
RASSF3	-0.333116158	4.18039877	-3.88701729	0.000129	0.00025	-0.00490393
AOPEP	-0.350879324	3.72283932	-3.886830651	0.000129	0.00025	-0.00558866
NRCAM	0.5366086	4.2846029	3.886289799	0.00013	0.00025	-0.00757278
DBF4B	-0.258569657	2.09213647	-3.886077899	0.00013	0.00025	-0.00835006
SMARCA	-0.305708956	5.50793684	-3.885697957	0.00013	0.00025	-0.00974366
RIPK2	0.252061304	3.803419	3.885523227	0.00013	0.00025	-0.01038451
SLC26A8	-0.318320941	0.77403158	-3.885403346	0.00013	0.00025	-0.01082419
ARHGEF	-0.261286917	4.15936169	-3.885304333	0.00013	0.00025	-0.01118731
PCDH17	-0.370962693	2.76272834	-3.885214919	0.00013	0.00025	-0.01151523
KRT13	0.407767458	0.7485588	3.885155885	0.00013	0.00025	-0.01173173
ZBP1	0.377143094	0.69625426	3.884687549	0.00013	0.00026	-0.01344917
BECN1	-0.358607412	4.49232304	-3.884341171	0.000131	0.00026	-0.01471926
KRT83	-0.60597936	2.40705552	-3.88408859	0.000131	0.00026	-0.01564535
XG	0.250011778	0.55584273	3.882707765	0.000132	0.00026	-0.02070722
CENPN	-0.214920153	1.93379888	-3.882231484	0.000132	0.00026	-0.02245282
PDE3B	0.175794751	0.41030033	3.881099601	0.000132	0.00026	-0.02660048
CREB1	-0.292880744	3.83619823	-3.880645697	0.000133	0.00026	-0.02826345
RNF41	-0.262150426	4.69511877	-3.880333626	0.000133	0.00026	-0.02940669

MAP4K1	0.500502287	2.30233928	3.880231766	0.000133	0.00026	-0.02977983
ZNF707	-0.224363609	2.70539097	-3.879759928	0.000133	0.00026	-0.03150816
DDI2	-0.246437823	3.31358705	-3.879564778	0.000133	0.00026	-0.03222294
TMEM20	0.577872425	1.77520453	3.876530458	0.000135	0.00026	-0.04333263
HSD17B:	-0.279632096	4.79063748	-3.875204586	0.000135	0.00026	-0.04818468
CARD17	0.152347298	0.239548	3.874829511	0.000136	0.00026	-0.04955701
ZNF275	-0.331587208	3.34305851	-3.873936194	0.000136	0.00027	-0.05282501
MANBA	-0.271608234	4.31742997	-3.871860296	0.000137	0.00027	-0.06041662
CRISPLD	0.494233258	2.90993711	3.871642797	0.000137	0.00027	-0.06121181
CSPP1	-0.235853553	2.40974641	-3.87163923	0.000137	0.00027	-0.06122485
IL22RA2	0.222714491	0.28048894	3.87077534	0.000138	0.00027	-0.06438289
PPP1R1:	0.346358783	4.47551588	3.870432081	0.000138	0.00027	-0.06563754
SLC7A1C	-0.272964033	0.45201437	-3.870168666	0.000138	0.00027	-0.06660028
LGALS2	0.546146334	1.94629053	3.869626599	0.000138	0.00027	-0.06858127
NOCT	-0.262777898	1.81097946	-3.868578009	0.000139	0.00027	-0.07241265
LAT2	0.39772652	2.47495063	3.868410886	0.000139	0.00027	-0.0730232
DDB2	0.297353447	4.18994654	3.868140339	0.000139	0.00027	-0.07401155
SRSF12	-0.19824962	1.58408044	-3.867222727	0.00014	0.00027	-0.07736326
ECD	-0.250455168	4.80313914	-3.866738759	0.00014	0.00027	-0.07913074
CALHM2	-0.329498125	3.59939333	-3.865194965	0.000141	0.00027	-0.08476745
ZNF146	-0.303484347	5.71753452	-3.864231872	0.000141	0.00028	-0.08828289
APH1B	-0.301778475	4.60751233	-3.863494588	0.000142	0.00028	-0.09097356
ROR2	-0.60242283	2.32036566	-3.86336061	0.000142	0.00028	-0.09146246
MS4A4E	0.201755571	0.60224031	3.862874654	0.000142	0.00028	-0.09323562
TRMT2A	-0.252304689	4.76366879	-3.862180386	0.000142	0.00028	-0.09576853
EDEM3	-0.315739587	3.88521057	-3.861917099	0.000143	0.00028	-0.09672898
AP4M1	-0.229764774	3.91295847	-3.861868177	0.000143	0.00028	-0.09690743
DHX15	-0.307350132	4.96528305	-3.861526342	0.000143	0.00028	-0.09815432
RPL35	-0.301527835	9.6954323	-3.861144917	0.000143	0.00028	-0.09954549
KCTD11	-0.266134359	5.17319228	-3.86054006	0.000143	0.00028	-0.10175135
RECQL4	-0.252923054	2.85657727	-3.859648871	0.000144	0.00028	-0.10500086
CBFA2T:	-0.254081931	3.70752733	-3.859636369	0.000144	0.00028	-0.10504645
DHX33	-0.216995229	3.29521768	-3.859318753	0.000144	0.00028	-0.1062044
PRRX2	0.489187394	2.33868038	3.859270352	0.000144	0.00028	-0.10638085
ELF2	-0.256835697	3.62521188	-3.859125612	0.000144	0.00028	-0.1069085
SP110	0.223305387	1.74730428	3.858240992	0.000145	0.00028	-0.11013302
CEP72	-0.222059916	1.85436234	-3.858125239	0.000145	0.00028	-0.1105549
BCL2L15	-0.068585618	0.20067688	-3.85732189	0.000145	0.00028	-0.11348253
C12orf66	-0.222620209	2.59175346	-3.857091403	0.000145	0.00028	-0.11432239
TRAM2	-0.395254464	4.04043961	-3.856868052	0.000145	0.00028	-0.1151362

GCA	-0.250897695	4.1486297	-3.856071876	0.000146	0.00028	-0.11803685
MME	0.402946109	0.74738172	3.855094414	0.000146	0.00028	-0.12159724
PTPRZ1	0.058872584	0.0717137	3.85479083	0.000147	0.00028	-0.12270287
SLC7A11	-0.50204453	1.82631967	-3.854561855	0.000147	0.00029	-0.12353673
STBD1	0.240414288	1.65949426	3.854533924	0.000147	0.00029	-0.12363845
HCST	0.522703619	3.6806127	3.854283273	0.000147	0.00029	-0.12455119
PPBP	0.542000782	0.98412243	3.854127978	0.000147	0.00029	-0.12511667
ZDHHC1	-0.152565479	0.46279213	-3.854095701	0.000147	0.00029	-0.1252342
F8A1	-0.353197546	4.23788677	-3.853681583	0.000147	0.00029	-0.12674203
CD3D	0.740719632	2.888877	3.853480499	0.000147	0.00029	-0.12747413
SLC7A9	-0.165920343	0.58782961	-3.852982808	0.000148	0.00029	-0.12928598
KRT71	-0.116147101	0.13601448	-3.85179972	0.000148	0.00029	-0.13359218
BORA	-0.201828469	1.44525008	-3.851395437	0.000148	0.00029	-0.13506342
SPAG9	-0.35548609	4.89057246	-3.85112182	0.000149	0.00029	-0.13605907
TSPAN6	-0.327835345	5.2293864	-3.850779328	0.000149	0.00029	-0.13730525
LRP5	-0.346006861	5.45900416	-3.850663684	0.000149	0.00029	-0.13772601
KPNA4	-0.229302925	4.73628335	-3.850146914	0.000149	0.00029	-0.13960609
IL3RA	-0.285075203	4.54569033	-3.849333381	0.00015	0.00029	-0.14256537
GRK1	-0.349464202	1.24210985	-3.848651479	0.00015	0.00029	-0.1450454
STX7	-0.223716719	3.77161569	-3.846870605	0.000151	0.00029	-0.15152049
KLF3	-0.331584336	5.05898957	-3.846779518	0.000151	0.00029	-0.1518516
KRT2	-0.079167999	0.06570918	-3.846674318	0.000151	0.00029	-0.152234
CENPC	-0.282257108	3.30793168	-3.846086851	0.000152	0.00029	-0.1543693
ILRUN	-0.245620969	6.74913382	-3.845845298	0.000152	0.00029	-0.15524719
KLF8	0.325355784	2.04006313	3.845093687	0.000152	0.0003	-0.15797853
USP50	-0.053464961	0.14447702	-3.844538483	0.000152	0.0003	-0.15999582
APOL6	0.438008491	4.34829839	3.844111557	0.000153	0.0003	-0.16154685
DPY19L3	-0.311110432	3.49516862	-3.843854573	0.000153	0.0003	-0.1624804
ADAM33	-0.321577038	1.01738086	-3.842151052	0.000154	0.0003	-0.16866742
SLC35F3	-0.555941027	1.76192817	-3.841868227	0.000154	0.0003	-0.16969437
ZNF302	-0.274067547	4.25182521	-3.838905832	0.000156	0.0003	-0.18044697
ZNF214	-0.280074789	2.40006125	-3.838159216	0.000156	0.0003	-0.18315579
SOCS7	-0.321852045	3.38831351	-3.837246012	0.000157	0.0003	-0.18646838
INPP5D	0.43775353	2.99565476	3.836766496	0.000157	0.0003	-0.18820752
SLC37A1	-0.231087169	4.02083588	-3.835980802	0.000158	0.00031	-0.19105669
ZNF257	-0.191722732	1.18971168	-3.835377586	0.000158	0.00031	-0.19324379
BRD8	-0.269070142	4.59050969	-3.834751356	0.000158	0.00031	-0.19551401
MDC1	-0.279297552	3.84634628	-3.834304902	0.000159	0.00031	-0.1971323
LSM12	-0.24657034	3.29972004	-3.834261465	0.000159	0.00031	-0.19728974
PTX4	-0.063288011	0.09157056	-3.834190384	0.000159	0.00031	-0.19754738

PFDN2	-0.229904677	7.44667003	-3.833904691	0.000159	0.00031	-0.19858283
VSX1	-0.21088417	0.28375076	-3.833574696	0.000159	0.00031	-0.19977876
PHLPP2	-0.289435989	2.39188555	-3.8327818	0.000159	0.00031	-0.20265192
SPRY3	-0.126763778	0.84714525	-3.832748924	0.00016	0.00031	-0.20277105
SOX13	-0.265088781	3.47211049	-3.831247654	0.00016	0.00031	-0.2082096
SIGLEC1	0.233379485	0.44026192	3.830843885	0.000161	0.00031	-0.20967198
HHATL	-0.871633595	5.33732259	-3.830755555	0.000161	0.00031	-0.20999188
SMAP1	-0.221078899	3.39922483	-3.830664708	0.000161	0.00031	-0.21032088
ACSM6	-0.245145784	0.53013621	-3.8306149	0.000161	0.00031	-0.21050126
ROM1	-0.300855875	2.919816	-3.830408442	0.000161	0.00031	-0.21124892
ZNF239	-0.264115806	2.59349873	-3.830123619	0.000161	0.00031	-0.21228032
SCX	-0.632087961	6.41196354	-3.829651919	0.000161	0.00031	-0.21398827
CSNK1A	-0.028970893	0.04640295	-3.829637813	0.000161	0.00031	-0.21403935
RABL2A	-0.263857156	2.48247584	-3.82948847	0.000162	0.00031	-0.21458005
WFDC8	-0.28662234	0.23624488	-3.829146691	0.000162	0.00031	-0.21581742
ARID1B	-0.304036733	4.31866878	-3.827446019	0.000163	0.00031	-0.22197303
SIX2	-0.356034044	0.64925784	-3.826156703	0.000164	0.00032	-0.2266381
DAZAP1	-0.179016264	4.79339509	-3.826113364	0.000164	0.00032	-0.22679489
FAM227A	-0.209402374	0.97897699	-3.825403412	0.000164	0.00032	-0.22936305
HSD17B1	-0.556648753	5.80947709	-3.82513238	0.000164	0.00032	-0.23034336
CDKL1	-0.211114983	0.79963813	-3.824996007	0.000164	0.00032	-0.2308366
RGS1	0.758695332	4.14783768	3.824975355	0.000164	0.00032	-0.23091129
DHX38	-0.256874417	4.63961223	-3.824254113	0.000165	0.00032	-0.2335196
CFI	0.678263933	5.70962688	3.824167812	0.000165	0.00032	-0.23383167
ASAP2	0.594660865	3.69873409	3.824041873	0.000165	0.00032	-0.23428706
GRIN3A	-0.074384855	0.27862791	-3.823945207	0.000165	0.00032	-0.23463659
BCAM	-0.437439869	7.51209191	-3.823836043	0.000165	0.00032	-0.23503131
ENO1	-0.241517063	8.9453128	-3.820912388	0.000167	0.00032	-0.24559892
GPR108	-0.268374932	6.53212913	-3.820673871	0.000167	0.00032	-0.24646073
ALG10	-0.209815427	1.60925698	-3.819896022	0.000168	0.00032	-0.24927091
ADGRG6	-0.296408212	1.11044705	-3.819564348	0.000168	0.00032	-0.25046902
TNS3	-0.413932616	4.42404983	-3.81909347	0.000168	0.00032	-0.25216981
ARID1A	-0.361003536	5.40126587	-3.818948172	0.000168	0.00032	-0.25269458
ZKSCAN	-0.21258712	2.30223211	-3.817721599	0.000169	0.00033	-0.25712387
TEX10	-0.22842788	3.46415151	-3.817327177	0.000169	0.00033	-0.25854791
FNBP1L	-0.356605907	5.54469322	-3.817033394	0.000169	0.00033	-0.25960851
FAM228B	-0.205234466	2.62740471	-3.816794456	0.00017	0.00033	-0.26047105
COL1A2	0.755205286	6.38206622	3.816384627	0.00017	0.00033	-0.26195039
SEC31A	-0.275789569	6.09583603	-3.815941033	0.00017	0.00033	-0.26355145
AQR	-0.254634184	3.6204349	-3.815793149	0.00017	0.00033	-0.26408516

WNT3A	-0.255568751	0.20025853	-3.815409504	0.00017	0.00033	-0.26546967
SRXN1	-0.293396981	1.34801787	-3.815056899	0.000171	0.00033	-0.26674204
KLRC1	0.242802504	0.60425703	3.814144659	0.000171	0.00033	-0.27003337
POC5	-0.185222315	3.05488025	-3.813189788	0.000172	0.00033	-0.27347776
TMEM62	-0.201839328	3.94581656	-3.812870573	0.000172	0.00033	-0.27462906
TET1	-0.161726671	0.78536067	-3.812215028	0.000173	0.00033	-0.2769931
ADGRF2	0.191502681	0.21543762	3.8116434	0.000173	0.00033	-0.27905422
ZSCAN9	-0.21940516	3.29253466	-3.810039156	0.000174	0.00034	-0.28483719
RAB36	-0.395944952	4.7846911	-3.808589229	0.000175	0.00034	-0.29006201
CMTM3	0.405626482	5.13767548	3.806834128	0.000176	0.00034	-0.29638415
CD63	-0.247733683	10.2804333	-3.80647027	0.000176	0.00034	-0.2976945
TBC1D9I	-0.255659412	5.47002245	-3.806420073	0.000176	0.00034	-0.29787526
MTRR	-0.283574317	3.51711759	-3.805714182	0.000177	0.00034	-0.300417
HERPUD	-0.231013082	4.84355644	-3.804988151	0.000177	0.00034	-0.30303083
C1QTNF	0.326798916	2.40799175	3.803866494	0.000178	0.00034	-0.3070681
MTMR12	-0.423578157	4.59064178	-3.803032348	0.000179	0.00034	-0.31006982
DNAJC1	-0.369147411	3.92619308	-3.802675942	0.000179	0.00034	-0.31135218
SMAGP	-0.274035999	2.64689827	-3.802477198	0.000179	0.00034	-0.31206723
MMP25	0.204920993	0.90307941	3.801938287	0.000179	0.00035	-0.31400596
FAM120	-0.255622067	6.29703566	-3.801804751	0.00018	0.00035	-0.31448632
FAM71D	-0.04360536	0.14665606	-3.801509614	0.00018	0.00035	-0.31554794
USP31	0.225062392	1.84975809	3.801259039	0.00018	0.00035	-0.31644921
GOLGA8	-0.17799982	0.76520925	-3.801239511	0.00018	0.00035	-0.31651944
NCAPD2	-0.278149187	4.53937533	-3.801130923	0.00018	0.00035	-0.31691
PUS10	-0.252626233	2.61611814	-3.799528889	0.000181	0.00035	-0.32267078
CXCL13	1.032470214	1.89573292	3.799513913	0.000181	0.00035	-0.32272462
ZNF766	-0.222337499	3.2451006	-3.798696836	0.000182	0.00035	-0.32566192
PSD3	0.519917222	4.14452473	3.798466241	0.000182	0.00035	-0.32649078
TMEM10	-0.252785306	4.02600702	-3.798126696	0.000182	0.00035	-0.32771117
MPHOSI	-0.265586516	5.61501914	-3.798071537	0.000182	0.00035	-0.32790941
PLPP5	-0.298244067	4.91177188	-3.797694511	0.000182	0.00035	-0.32926438
EYA2	-0.514485394	3.89133694	-3.797438586	0.000183	0.00035	-0.33018407
INSIG2	-0.248914467	4.54707253	-3.797354884	0.000183	0.00035	-0.33048485
CELF4	0.388481904	1.44948027	3.79680994	0.000183	0.00035	-0.33244293
PNOC	0.410511146	0.6193453	3.796428021	0.000183	0.00035	-0.33381508
INVS	-0.246133913	2.47135341	-3.795509831	0.000184	0.00035	-0.33711343
EHD3	-0.307327938	2.72772159	-3.7943956	0.000185	0.00035	-0.34111505
TAFA3	-0.309942706	0.84587557	-3.793762638	0.000185	0.00036	-0.34338779
IL23R	0.035899161	0.04394928	3.793678061	0.000185	0.00036	-0.34369145
VAV2	-0.298440413	5.07136857	-3.792090295	0.000186	0.00036	-0.34939093

TAF3	-0.204900783	3.22638001	-3.791452424	0.000187	0.00036	-0.35168005
LHFPL6	-0.435985955	5.45675039	-3.791276199	0.000187	0.00036	-0.35231241
DUSP26	-0.275374059	0.84931337	-3.790761476	0.000187	0.00036	-0.35415926
TEX29	-0.13764942	0.4862066	-3.790242113	0.000188	0.00036	-0.35602253
SCN3A	-0.177173626	0.36698734	-3.790140555	0.000188	0.00036	-0.35638685
GGA1	-0.22791182	4.60625575	-3.789769499	0.000188	0.00036	-0.35771789
HERC3	-0.351849611	3.71569794	-3.788892553	0.000189	0.00036	-0.36086316
MELTF	0.473639022	2.97900322	3.78888586	0.000189	0.00036	-0.36088716
POSTN	0.839194874	4.50943883	3.788877393	0.000189	0.00036	-0.36091752
SLC35F4	-0.180724873	0.23584264	-3.788608233	0.000189	0.00036	-0.36188277
CPVL	-0.522656788	7.24567111	-3.788396071	0.000189	0.00036	-0.36264356
ZXDC	-0.254741979	3.60305128	-3.788065075	0.000189	0.00036	-0.36383041
TMEM65	-0.281778254	3.29424867	-3.787384474	0.00019	0.00036	-0.36627054
MYO7B	-0.174315494	0.72009609	-3.787336796	0.00019	0.00036	-0.36644147
L3HYPDI	-0.281628845	2.56073809	-3.787140261	0.00019	0.00036	-0.36714602
WNK2	-0.242406135	0.74120255	-3.786339688	0.00019	0.00036	-0.37001561
CTDNEP	-0.18279685	6.88606135	-3.786277239	0.000191	0.00036	-0.37023944
SCNN1B	-0.524110857	2.46395927	-3.784381307	0.000192	0.00037	-0.37703301
CDC40	-0.326027641	3.46101032	-3.784306305	0.000192	0.00037	-0.3773017
FNDC8	-0.065436709	0.16397593	-3.782757326	0.000193	0.00037	-0.3828497
GABRR2	-0.12901113	0.37966864	-3.782571103	0.000193	0.00037	-0.38351656
KIAA035!	-0.280551276	4.2640091	-3.78246617	0.000193	0.00037	-0.38389231
SCG2	0.287072054	0.84659611	3.782339121	0.000193	0.00037	-0.38434724
C8A	-0.377201894	0.47317723	-3.782067657	0.000194	0.00037	-0.38531924
C1orf226	0.441503666	2.55494971	3.781519763	0.000194	0.00037	-0.38728084
LY86	0.486581315	3.08344962	3.781508795	0.000194	0.00037	-0.3873201
GDAP2	-0.198438995	2.61117577	-3.781227751	0.000194	0.00037	-0.38832621
NAPG	-0.255751979	4.24688439	-3.781211295	0.000194	0.00037	-0.38838511
IP6K1	-0.242599658	5.04201617	-3.780229316	0.000195	0.00037	-0.39189995
CCDC15	-0.141924264	0.93344645	-3.77878686	0.000196	0.00037	-0.39706151
GPR63	-0.141617333	0.54028132	-3.778630721	0.000196	0.00038	-0.39762011
CYTH1	-0.25884012	4.78999349	-3.778479511	0.000196	0.00038	-0.39816107
TRIM74	-0.153920854	0.89052712	-3.778404813	0.000196	0.00038	-0.39842829
CRIP2	-0.303701817	7.10517438	-3.778313968	0.000196	0.00038	-0.39875328
KRTAP2-	0.395520113	0.64097001	3.77821473	0.000196	0.00038	-0.39910828
RNF38	-0.336981208	4.86541363	-3.7781614	0.000197	0.00038	-0.39929905
PRPF4	-0.240464142	4.42732061	-3.777839973	0.000197	0.00038	-0.40044881
LEPR	0.380835906	1.90203759	3.777504337	0.000197	0.00038	-0.40164929
MISP3	-0.322740182	3.84948805	-3.77639442	0.000198	0.00038	-0.40561852
ENO2	-0.345438705	5.37369271	-3.776199937	0.000198	0.00038	-0.40631391

KIAA089!	-0.388514791	3.23981104	-3.776095162	0.000198	0.00038	-0.40668853
SNX13	-0.284109908	3.45313815	-3.775796799	0.000198	0.00038	-0.40775526
CARD9	0.246435794	1.03637369	3.774061332	0.0002	0.00038	-0.41395858
PTPN13	-0.46559246	4.57954376	-3.773568968	0.0002	0.00038	-0.41571804
VCPKMT	-0.231978979	3.45381364	-3.773419835	0.0002	0.00038	-0.41625092
XRN2	-0.331454854	6.14537103	-3.773355837	0.0002	0.00038	-0.41647959
NSD1	-0.303002769	4.10192905	-3.773162245	0.0002	0.00038	-0.4171713
IL18RAP	0.164547439	0.466055	3.773121427	0.0002	0.00038	-0.41731714
MARCKS	-0.371383722	5.64572612	-3.773008549	0.0002	0.00038	-0.41772044
ITGB5	-0.376538424	5.74906343	-3.772881497	0.0002	0.00038	-0.41817436
H2AFY	-0.192510046	4.83390139	-3.772582106	0.000201	0.00038	-0.41924396
CILP	-0.699307062	1.53488611	-3.772457396	0.000201	0.00038	-0.41968947
ACTR8	-0.240114579	4.14470864	-3.772038243	0.000201	0.00038	-0.42118676
PPP1R14	-0.281629461	6.32560566	-3.771403585	0.000202	0.00038	-0.42345358
FEZ2	-0.261860661	3.79323745	-3.770419629	0.000202	0.00039	-0.42696731
ARPC2	-0.257677554	6.32060333	-3.769991349	0.000203	0.00039	-0.42849646
SRCIN1	0.434443171	2.59157127	3.768866182	0.000204	0.00039	-0.43251305
MBD5	-0.226012194	2.18419689	-3.768180623	0.000204	0.00039	-0.43495982
KCNE5	0.164718467	0.33014342	3.767885196	0.000204	0.00039	-0.43601408
ARSE	0.235814615	0.66389053	3.767507161	0.000205	0.00039	-0.43736303
AKR7A3	-0.295201317	0.98908758	-3.76664344	0.000205	0.00039	-0.44044459
CKLF-CM	0.169406801	0.75755598	3.766238972	0.000206	0.00039	-0.44188743
C3orf38	-0.226561161	4.02470827	-3.766067794	0.000206	0.00039	-0.44249802
RBBP6	-0.260815299	4.27510112	-3.765279126	0.000206	0.00039	-0.44531088
UBE2B	-0.221768706	5.86881097	-3.765205332	0.000206	0.00039	-0.44557405
RBM39	-0.27257213	6.12399277	-3.764851559	0.000207	0.00039	-0.44683562
SLBP	-0.225383076	6.01967285	-3.764719621	0.000207	0.00039	-0.44730609
BRCA1	-0.312936388	2.00965168	-3.764716045	0.000207	0.00039	-0.44731884
ITGA5	-0.32605429	4.50723675	-3.764543463	0.000207	0.00039	-0.44793422
LTC4S	-0.123703233	0.35976459	-3.764409378	0.000207	0.00039	-0.44841231
ASB14	-0.102963733	0.70078447	-3.763801359	0.000208	0.0004	-0.45058006
SLC9A7	-0.26194721	3.88638451	-3.763643811	0.000208	0.0004	-0.45114171
TBC1D22	-0.23632146	4.55884227	-3.762663848	0.000208	0.0004	-0.45463475
ZNF564	-0.137484157	0.7590006	-3.761003901	0.00021	0.0004	-0.46054971
MYT1L	-0.023911516	0.01942408	-3.760890042	0.00021	0.0004	-0.46095534
NFKBIZ	0.572103958	4.16195208	3.760530142	0.00021	0.0004	-0.46223744
DUSP4	0.53373395	3.94104367	3.760334784	0.00021	0.0004	-0.46293334
C1QTNF	0.386750085	1.58130392	3.760206795	0.00021	0.0004	-0.46338924
GPR25	0.226242331	0.344016	3.759479808	0.000211	0.0004	-0.46597851
CMTR1	-0.24323572	4.75464582	-3.759191326	0.000211	0.0004	-0.46700586

RANBP2	-0.315359027	4.97842525	-3.759142898	0.000211	0.0004	-0.46717831
PPP2R2(	-0.34597888	0.29864256	-3.758985195	0.000211	0.0004	-0.46773989
CLIC5	0.560463269	2.90997181	3.758275556	0.000212	0.0004	-0.47026665
ATG12	-0.189863872	3.54576129	-3.758274024	0.000212	0.0004	-0.47027211
RNF213	0.309982713	4.14548436	3.758261295	0.000212	0.0004	-0.47031743
MPZL2	0.552174137	6.92868848	3.7580173	0.000212	0.0004	-0.4711861
SRP72	-0.281546635	5.79227557	-3.757745242	0.000212	0.0004	-0.47215463
PYCR1	-0.526475135	2.84778089	-3.757428372	0.000213	0.0004	-0.4732826
FAM209E	-0.125392119	0.38609802	-3.755998436	0.000214	0.00041	-0.47837174
IRAK4	-0.22083225	3.87637281	-3.755633484	0.000214	0.00041	-0.47967033
CNNM3	-0.267472531	4.34650804	-3.755292504	0.000214	0.00041	-0.48088351
DIMT1	-0.211190724	4.44740788	-3.755037806	0.000215	0.00041	-0.48178965
ZFR	-0.32486747	5.30620977	-3.754694393	0.000215	0.00041	-0.48301132
ADGRG2	0.493339568	1.64827353	3.754505299	0.000215	0.00041	-0.48368396
HSH2D	0.402349312	2.95805649	3.754166577	0.000215	0.00041	-0.48488879
PANX1	-0.273950414	4.20268707	-3.754116285	0.000215	0.00041	-0.48506767
GLS	0.361581969	5.23472124	3.754085382	0.000215	0.00041	-0.48517759
TFR2	0.108304527	0.32985778	3.753344004	0.000216	0.00041	-0.48781426
DZIP1L	-0.270938553	1.2203285	-3.75280306	0.000216	0.00041	-0.48973781
PDXP	-0.171439163	0.6769077	-3.750019056	0.000219	0.00041	-0.49963356
CPA3	0.495254805	1.16623699	3.74998913	0.000219	0.00041	-0.4997399
SYT17	-0.277286781	2.89285655	-3.749711012	0.000219	0.00042	-0.5007281
TH	0.146559041	0.29962546	3.748615142	0.00022	0.00042	-0.50462128
XRCC1	-0.219169343	5.56881932	-3.747907814	0.00022	0.00042	-0.50713359
MIGA1	-0.287332947	3.42021522	-3.747088643	0.000221	0.00042	-0.51004262
MYOCD	0.194067849	0.52892214	3.746402899	0.000222	0.00042	-0.51247738
NDRG4	-0.227900051	1.18884135	-3.745362349	0.000223	0.00042	-0.51617113
FKBP11	-0.434272868	3.3170527	-3.745078507	0.000223	0.00042	-0.51717856
PEX5L	-0.046826297	0.11524139	-3.74449535	0.000223	0.00042	-0.51924811
LIMS1	-0.27264617	4.09640157	-3.743634937	0.000224	0.00042	-0.52230109
PTPRF	0.493076254	6.39014085	3.743396637	0.000224	0.00042	-0.52314653
ASS1	-0.347766133	7.71805961	-3.742548519	0.000225	0.00043	-0.52615509
SENP6	-0.282599104	4.62852895	-3.742367197	0.000225	0.00043	-0.52679823
LBP	0.244610859	0.2400122	3.7422288	0.000225	0.00043	-0.52728909
ZNF614	-0.301850747	3.05700802	-3.742227679	0.000225	0.00043	-0.52729306
CSMD1	-0.445219148	0.73234587	-3.742009708	0.000225	0.00043	-0.52806612
ZNRF3	0.06256727	0.15376211	3.741273343	0.000226	0.00043	-0.53067743
SLC37A2	0.328874152	1.59751115	3.738387373	0.000228	0.00043	-0.54090724
ZNF510	-0.268378271	2.77048963	-3.73800281	0.000229	0.00043	-0.54226986
MOSPD1	-0.199329409	3.87544754	-3.73765408	0.000229	0.00043	-0.5435054

SCGB1D	-0.25406115	0.46743884	-3.73663244	0.00023	0.00044	-0.54712445
MMP24C	-0.202330996	5.17522373	-3.735545218	0.000231	0.00044	-0.55097484
BCL2L1	0.341089675	6.87969791	3.735120416	0.000231	0.00044	-0.552479
SNAPC4	-0.250693136	3.55965549	-3.735068197	0.000231	0.00044	-0.55266389
TRIM7	-0.40544833	2.21059325	-3.734400578	0.000232	0.00044	-0.55502749
FBXO38	-0.257917412	3.75545438	-3.7343367	0.000232	0.00044	-0.55525362
RNF128	-0.371358691	6.34189834	-3.734009529	0.000232	0.00044	-0.55641176
CPSF6	-0.257449014	4.85467025	-3.733345794	0.000233	0.00044	-0.55876101
ZSCAN4	0.226481064	0.48983949	3.733152032	0.000233	0.00044	-0.55944675
MED26	-0.177192811	2.47136839	-3.73282838	0.000233	0.00044	-0.56059211
IFNE	0.541090976	1.16737188	3.732448668	0.000234	0.00044	-0.56193574
TEP1	0.299974412	3.54711347	3.731361198	0.000235	0.00044	-0.56578315
KANK4	-0.400537108	0.65828361	-3.731350001	0.000235	0.00044	-0.56582276
LGR5	0.175667444	0.17252275	3.730158316	0.000236	0.00045	-0.5700377
CLDN14	0.077302652	0.11586053	3.729381065	0.000236	0.00045	-0.57278616
LEFTY2	-0.296321554	0.23862219	-3.728777231	0.000237	0.00045	-0.57492103
CCDC18	-0.11805663	0.39864898	-3.725586369	0.00024	0.00045	-0.5861973
COLCA2	-0.430314534	2.7679806	-3.72557706	0.00024	0.00045	-0.58623018
ST6GALI	-0.316529219	4.58276558	-3.725314269	0.00024	0.00045	-0.58715848
FXYD5	0.488844931	6.42023901	3.723069011	0.000242	0.00046	-0.59508734
ADGRG1	-0.363265457	7.89636012	-3.72300151	0.000242	0.00046	-0.59532564
OFD1	-0.235119851	4.87138026	-3.722722297	0.000242	0.00046	-0.59631134
TRIM14	0.455041958	3.68655208	3.722462538	0.000243	0.00046	-0.59722829
RAB14	-0.285940968	6.61470217	-3.722411968	0.000243	0.00046	-0.5974068
LY6K	-0.117839163	0.22555611	-3.72178932	0.000243	0.00046	-0.5996045
CEACAM	0.057043606	0.10125663	3.721280249	0.000244	0.00046	-0.60140107
C5AR2	-0.274334448	1.27195375	-3.720487454	0.000244	0.00046	-0.6041985
ANKRD1	-0.291416305	3.62879684	-3.720196598	0.000245	0.00046	-0.60522467
DDX42	-0.244031109	5.40079359	-3.719464905	0.000245	0.00046	-0.60780584
FLT3	0.219344342	0.38090473	3.719326335	0.000245	0.00046	-0.60829462
ACTN1	0.385212244	4.91361282	3.719219486	0.000246	0.00046	-0.6086715
MED17	-0.254423831	3.48319671	-3.719155555	0.000246	0.00046	-0.60889699
ZFX	-0.285173393	3.61471932	-3.71775838	0.000247	0.00047	-0.61382413
MZB1	1.065957251	2.14726697	3.717582529	0.000247	0.00047	-0.61444416
MRTFB	-0.304848354	3.21390577	-3.717311497	0.000247	0.00047	-0.61539972
GCN1	-0.2897616	5.11424134	-3.71698474	0.000248	0.00047	-0.61655166
DUSP13	0.360244334	0.78760274	3.715469759	0.000249	0.00047	-0.62189138
TC2N	-0.390266289	3.30111665	-3.713757154	0.000251	0.00047	-0.62792528
SH3BP4	-0.329619083	4.102588	-3.712847794	0.000251	0.00047	-0.63112815
ZNF133	-0.226292311	3.44067483	-3.71199805	0.000252	0.00048	-0.63412041

GPR45	-0.083732365	0.15804916	-3.711885868	0.000252	0.00048	-0.6345154
C20orf20	-0.170455369	0.53174189	-3.711327155	0.000253	0.00048	-0.63648245
MBD4	-0.229641064	5.05686585	-3.711105058	0.000253	0.00048	-0.6372643
G2E3	-0.241658584	2.1866199	-3.710819486	0.000253	0.00048	-0.63826955
TSHZ3	-0.387103501	3.28521268	-3.709385881	0.000255	0.00048	-0.64331497
OXCT1	-0.389961167	4.73239292	-3.709176594	0.000255	0.00048	-0.64405139
LEXM	-0.047161233	0.08999962	-3.707699324	0.000256	0.00048	-0.64924839
TRIM32	-0.275754837	3.7204961	-3.706683839	0.000257	0.00048	-0.65281976
HERC2	-0.274773711	3.62806698	-3.7064426	0.000258	0.00048	-0.65366805
TIAM1	0.623580684	3.55171445	3.704675509	0.000259	0.00049	-0.6598803
COL5A3	-0.442088805	2.94246628	-3.704140593	0.00026	0.00049	-0.66176028
GALNT2	-0.296010675	5.0958503	-3.703791797	0.00026	0.00049	-0.66298601
FCER1G	0.55620433	5.43821584	3.703488286	0.00026	0.00049	-0.66405251
ZNF326	-0.240069218	3.02006616	-3.70344089	0.00026	0.00049	-0.66421905
ATP6V0A	-0.255532581	4.9235885	-3.70306956	0.000261	0.00049	-0.66552374
LRP2BP	-0.282839089	1.87934622	-3.702733189	0.000261	0.00049	-0.6667055
ELAVL4	0.043617205	0.09253682	3.701608684	0.000262	0.00049	-0.67065548
PLEKHA:	-0.21201718	3.00344556	-3.699841529	0.000264	0.0005	-0.67686067
CREB3L2	-0.378757692	6.32417683	-3.699174917	0.000265	0.0005	-0.67920073
C4orf50	-0.054329409	0.09256098	-3.699138928	0.000265	0.0005	-0.67932705
YY1AP1	-0.232734573	4.79552836	-3.699093923	0.000265	0.0005	-0.67948502
UBASH3	0.351754058	0.85418186	3.698846042	0.000265	0.0005	-0.68035506
ANKAR	-0.196299549	1.42961848	-3.697984152	0.000266	0.0005	-0.68337981
ACVR2A	-0.259370772	2.92144575	-3.697079765	0.000267	0.0005	-0.68655301
CD22	0.760656929	3.30444279	3.696033825	0.000268	0.0005	-0.69022202
TMEM12	0.131747422	0.49928074	3.695996729	0.000268	0.0005	-0.69035213
HIST1H2	-0.219060248	0.43622138	-3.6955433	0.000268	0.0005	-0.69194238
PRTN3	0.30674439	0.91034392	3.695008237	0.000269	0.00051	-0.69381872
GJB4	0.398645688	0.80003636	3.69350104	0.00027	0.00051	-0.69910279
SOWAH(	-0.362078481	3.80242429	-3.691488608	0.000272	0.00051	-0.70615513
ATP6V1(	-0.263404969	1.43403053	-3.69095892	0.000273	0.00051	-0.70801079
FANCD2	0.210563937	1.73650476	3.69076955	0.000273	0.00051	-0.70867415
TMEM24	-0.355740194	6.12836987	-3.689547329	0.000274	0.00052	-0.71295485
C17orf49	-0.193193975	1.68996981	-3.688631581	0.000275	0.00052	-0.71616133
ZNF48	-0.208001143	3.7047467	-3.688207428	0.000276	0.00052	-0.71764626
TTC33	-0.300023551	3.97334068	-3.688197087	0.000276	0.00052	-0.71768245
MORN3	-0.230710792	1.43302985	-3.687979485	0.000276	0.00052	-0.7184442
HEATR5	-0.287927393	2.695459	-3.687858967	0.000276	0.00052	-0.71886607
ZNF311	-0.245423585	2.23357598	-3.687039406	0.000277	0.00052	-0.72173461
SLC6A1(	-0.237107953	0.71985708	-3.686625698	0.000277	0.00052	-0.7231824

C9orf135	-0.194012591	0.19773212	-3.685885178	0.000278	0.00052	-0.72577353
CTNND2	-0.31891207	0.41307125	-3.685661058	0.000278	0.00052	-0.72655765
SH3GL3	-0.122264054	0.15935103	-3.683938469	0.00028	0.00053	-0.73258296
WIPF2	-0.234050708	4.54167151	-3.683880233	0.00028	0.00053	-0.73278662
GLI3	-0.342075359	2.33349231	-3.683333937	0.000281	0.00053	-0.73469691
CGGBP1	-0.249718555	5.51087711	-3.683116648	0.000281	0.00053	-0.73545666
TCTE1	-0.062014946	0.09887326	-3.683024819	0.000281	0.00053	-0.73577772
CUBN	-0.339403884	2.07822857	-3.683008096	0.000281	0.00053	-0.73583619
PRSS36	-0.151183587	1.1524355	-3.68293411	0.000281	0.00053	-0.73609487
PLP1	-0.568792233	0.73071914	-3.682197598	0.000282	0.00053	-0.73866965
APLF	-0.142495549	1.11487537	-3.681995769	0.000282	0.00053	-0.73937515
ZNF398	-0.208318261	2.99210729	-3.681887012	0.000282	0.00053	-0.7397553
PCDHGA	-0.158010622	0.49916657	-3.681879539	0.000282	0.00053	-0.73978142
IGFBP5	0.837509802	8.79013124	3.681364772	0.000283	0.00053	-0.74158059
KCNJ1	-0.438296586	0.63568443	-3.680633967	0.000284	0.00053	-0.74413444
CCL24	0.321394084	0.60563633	3.679633244	0.000285	0.00053	-0.74763081
FBXO41	0.373106376	2.06816849	3.679207702	0.000285	0.00053	-0.74911733
PNN	-0.27205049	5.91228536	-3.678802915	0.000286	0.00053	-0.7505312
STX2	-0.272697013	3.12958145	-3.678542151	0.000286	0.00054	-0.75144195
LKAAEAI	-0.333961941	0.68327563	-3.677321267	0.000287	0.00054	-0.75570523
SLC16A5	-0.49207589	3.40456995	-3.677157403	0.000287	0.00054	-0.75627734
INHBB	0.697862996	6.25927809	3.676712189	0.000288	0.00054	-0.75783163
MAPK1IF	-0.236652967	5.22015262	-3.676390859	0.000288	0.00054	-0.75895332
CEP68	-0.262377228	3.33626042	-3.67515375	0.000289	0.00054	-0.76327098
ABCA7	-0.276229838	3.52684359	-3.673975649	0.000291	0.00054	-0.76738148
MEI1	0.413487074	0.95145019	3.673471315	0.000291	0.00055	-0.76914078
LIPI	-0.524028609	0.64573446	-3.672484263	0.000292	0.00055	-0.77258335
PPM1A	-0.246981619	3.9919767	-3.671855469	0.000293	0.00055	-0.77477598
FRMD8	-0.252886025	4.82408918	-3.670924691	0.000294	0.00055	-0.77802102
NFATC1	-0.292579062	2.14737283	-3.67071441	0.000294	0.00055	-0.77875404
CCDC85I	-0.472573556	5.59721507	-3.670320903	0.000295	0.00055	-0.78012566
AHNAK	0.444959926	6.31120853	3.670255765	0.000295	0.00055	-0.78035269
NUTM2E	-0.072304364	0.1166648	-3.670194618	0.000295	0.00055	-0.78056582
EAF1	-0.27868633	4.0882365	-3.670167913	0.000295	0.00055	-0.78065889
SCN3B	-0.31089552	1.16680659	-3.670114238	0.000295	0.00055	-0.78084597
SLC10A5	-0.18004322	1.15636995	-3.669793153	0.000295	0.00055	-0.78196499
CCT8	-0.203831285	6.99473814	-3.669453277	0.000296	0.00055	-0.78314942
GNL3	-0.214899387	5.35640484	-3.668777521	0.000296	0.00055	-0.78550404
ATP13A5	-0.125705684	0.40282431	-3.668369253	0.000297	0.00055	-0.78692644
ABL2	0.257134301	2.88562997	3.667042462	0.000298	0.00056	-0.79154795

MXD1	-0.287718297	3.02917837	-3.666912612	0.000298	0.00056	-0.79200017
ALDH3A:	-0.284703574	0.76068646	-3.666722182	0.000299	0.00056	-0.79266333
CYBRD1	-0.386529384	7.66360356	-3.666557308	0.000299	0.00056	-0.79323748
TMEM17	0.672500447	5.03344032	3.666387963	0.000299	0.00056	-0.79382717
DMRTA2	-0.029881807	0.04135689	-3.66619548	0.000299	0.00056	-0.7944974
WDR88	-0.215061349	1.242017	-3.664666432	0.000301	0.00056	-0.79982044
CLEC12E	0.044400463	0.07640252	3.664186791	0.000302	0.00056	-0.8014898
ZNF574	-0.19735406	3.74828532	-3.664065519	0.000302	0.00056	-0.80191185
TAB2	-0.279857387	4.6281726	-3.663614468	0.000302	0.00056	-0.80348147
CRYAB	-0.553638325	6.8901247	-3.663574065	0.000302	0.00056	-0.80362206
ITGAL	0.500789081	2.308979	3.663450388	0.000302	0.00056	-0.80405241
PTPN20	-0.311221597	0.45512763	-3.662995459	0.000303	0.00057	-0.80563529
CCDC82	-0.261831581	3.41980082	-3.662071524	0.000304	0.00057	-0.80884948
PIK3C2A	-0.347463403	4.60485582	-3.661707875	0.000304	0.00057	-0.81011435
HMGCLL	-0.108700445	0.19697654	-3.661208407	0.000305	0.00057	-0.81185144
IKZF5	-0.255605291	3.80668784	-3.66068777	0.000305	0.00057	-0.81366193
CDO1	0.746418784	2.87886663	3.660150302	0.000306	0.00057	-0.81553071
RERE	-0.303920705	5.51382744	-3.659905485	0.000306	0.00057	-0.81638185
PRPF38E	-0.264441227	4.7146515	-3.659880542	0.000306	0.00057	-0.81646857
GPRIN1	0.298244403	1.41172713	3.658764286	0.000308	0.00057	-0.82034874
STXBP6	-0.581838933	4.70148643	-3.658327518	0.000308	0.00057	-0.82186668
RPS6KB:	-0.22132989	3.5934396	-3.658301855	0.000308	0.00057	-0.82195587
DENND5	-0.267747593	4.19763421	-3.657520003	0.000309	0.00058	-0.82467269
PIAS1	-0.297130166	3.72386054	-3.657170595	0.000309	0.00058	-0.82588667
SLC9C1	-0.208050035	0.57504759	-3.655938681	0.000311	0.00058	-0.83016596
CENPB	-0.182829194	6.94461139	-3.655794258	0.000311	0.00058	-0.83066755
EFCAB8	-0.064990879	0.16010489	-3.655450908	0.000311	0.00058	-0.83185997
TBC1D1(	-0.291645483	3.51304822	-3.65406959	0.000313	0.00058	-0.83665613
CORO1C	0.306409531	4.77690458	3.653884653	0.000313	0.00058	-0.83729814
PRUNE2	0.26309373	0.82321233	3.653695332	0.000313	0.00058	-0.83795533
ZNF474	0.342157704	0.83264121	3.65352879	0.000314	0.00058	-0.83853343
EXOC8	-0.250532664	3.64648771	-3.652760614	0.000315	0.00059	-0.84119959
CEACAM	0.398206781	1.3941227	3.652317664	0.000315	0.00059	-0.84273675
SPSB3	-0.18412095	1.51883797	-3.651243369	0.000316	0.00059	-0.84646413
ZBTB21	-0.304722913	2.88363775	-3.650784597	0.000317	0.00059	-0.84805559
TMEM12	-0.290398475	2.2889819	-3.649973575	0.000318	0.00059	-0.85086855
QRICH1	-0.236515237	5.21875755	-3.647733932	0.00032	0.0006	-0.85863362
CYBA	-0.376131264	7.01035676	-3.646838453	0.000322	0.0006	-0.86173714
ZNF345	-0.193808519	1.91547645	-3.646372624	0.000322	0.0006	-0.86335132
NRG4	-0.131827464	0.29422491	-3.644841147	0.000324	0.0006	-0.86865686

RETREG	-0.24472176	6.63113422	-3.643449098	0.000326	0.00061	-0.87347763
C11orf96	-0.535248568	3.63854114	-3.643430048	0.000326	0.00061	-0.87354359
RREB1	-0.261182428	3.66863845	-3.641499161	0.000328	0.00061	-0.88022761
FBRSL1	-0.212895963	3.92671887	-3.640427663	0.000329	0.00061	-0.88393537
DNAJC9	-0.233752058	3.26577231	-3.640417397	0.000329	0.00061	-0.88397089
LRRC20	-0.231302021	3.7348794	-3.640215726	0.000329	0.00061	-0.88466863
SLC6A6	0.465468144	4.35428967	3.640134764	0.00033	0.00061	-0.88494873
ZNF25	-0.237438951	3.32022159	-3.639318112	0.000331	0.00061	-0.88777377
RBP4	0.432345238	1.21580785	3.639179548	0.000331	0.00061	-0.88825304
RABEP1	-0.232638883	4.86660251	-3.637794723	0.000332	0.00062	-0.89304208
TUBB8	-0.171493105	0.43914116	-3.637726738	0.000333	0.00062	-0.89327715
SLCO5A1	0.114889411	0.14013049	3.637147427	0.000333	0.00062	-0.89528002
FEN1	-0.246381942	4.01711173	-3.637096694	0.000333	0.00062	-0.89545541
PRR29	-0.484739975	3.53105563	-3.63623931	0.000334	0.00062	-0.8984191
DHX16	-0.22329414	5.03005887	-3.635368638	0.000335	0.00062	-0.90142808
SLITRK4	0.328954126	1.51453041	3.634138318	0.000337	0.00063	-0.90567887
VPS8	-0.21140075	3.32964271	-3.634076179	0.000337	0.00063	-0.90589353
SUPT3H	-0.208056646	3.09674118	-3.633959598	0.000337	0.00063	-0.90629625
LRIG2	-0.236873324	2.95457779	-3.633776033	0.000337	0.00063	-0.90693033
NKAPL	-0.324199081	1.37146319	-3.63218838	0.000339	0.00063	-0.91241331
KIAA121	0.202271448	0.36999122	3.631852878	0.00034	0.00063	-0.91357169
TSPAN1	-0.350573408	6.41753521	-3.631743464	0.00034	0.00063	-0.91394944
CACFD1	-0.261445611	5.75129158	-3.63026866	0.000342	0.00063	-0.9190402
SPTSSA	-0.26367654	5.68843104	-3.63020946	0.000342	0.00063	-0.9192445
PPFIBP2	-0.273704979	4.71151802	-3.630082655	0.000342	0.00063	-0.91968212
DMWD	-0.185846652	4.14987695	-3.629715622	0.000343	0.00064	-0.9209487
CD200R1	0.224023424	0.71607765	3.629466066	0.000343	0.00064	-0.92180982
KLHL7	-0.227273787	3.44673112	-3.628729889	0.000344	0.00064	-0.92434977
STOX1	-0.359239827	2.27931004	-3.628324157	0.000344	0.00064	-0.92574942
MMS22L	0.131115737	0.85426014	3.627543844	0.000345	0.00064	-0.92844086
MAL	-0.51570428	5.94637208	-3.627219751	0.000346	0.00064	-0.92955856
HNRNPA	-0.198170791	7.1067031	-3.626947365	0.000346	0.00064	-0.93049787
PROC	0.305702151	1.03093722	3.626574423	0.000346	0.00064	-0.93178384
S100A3	0.319122587	1.43908254	3.626383772	0.000347	0.00064	-0.93244119
GOLGA4	-0.366951857	5.29994192	-3.62626151	0.000347	0.00064	-0.93286273
INCA1	-0.278511327	3.49787275	-3.62597445	0.000347	0.00064	-0.93385241
TAF6	-0.313333779	4.85696855	-3.625968411	0.000347	0.00064	-0.93387323
BAZ1A	0.275190165	3.64178503	3.625871987	0.000347	0.00064	-0.93420564
WWP2	-0.240991226	4.56375524	-3.625030503	0.000348	0.00065	-0.93710629
HNRNPU	-0.141161197	0.52774318	-3.62473144	0.000349	0.00065	-0.93813703

RSAD2	0.402114745	2.83232628	3.624141687	0.00035	0.00065	-0.94016942
NINJ2	0.323199951	1.41508923	3.624134465	0.00035	0.00065	-0.94019431
UBXN2A	-0.219779779	3.15573802	-3.62385696	0.00035	0.00065	-0.94115054
RFX3	-0.259484389	2.03482112	-3.623577543	0.00035	0.00065	-0.94211329
HNRNPL	-0.262395651	4.55079559	-3.623274226	0.000351	0.00065	-0.94315831
AMPD2	-0.248219942	3.91814676	-3.623265982	0.000351	0.00065	-0.94318671
IDO2	-0.122167319	0.22600331	-3.622612075	0.000352	0.00065	-0.94543936
ZC3HAV	-0.36025166	2.65744434	-3.622559952	0.000352	0.00065	-0.9456189
HECTD4	-0.310151614	3.27018313	-3.621728751	0.000353	0.00065	-0.94848174
ZNF483	-0.262642652	1.60649106	-3.621728707	0.000353	0.00065	-0.94848189
POLR2A	-0.350526216	6.59776155	-3.621285019	0.000353	0.00065	-0.95000981
MAML3	-0.310171592	3.53024379	-3.62101927	0.000354	0.00065	-0.95092488
ASB16	-0.223056195	1.38830957	-3.62083267	0.000354	0.00065	-0.95156737
MCM3	-0.236451695	5.87319249	-3.619860988	0.000355	0.00066	-0.95491256
RAB3B	0.063427397	0.09263632	3.619649789	0.000355	0.00066	-0.95563954
WDR1	-0.250657992	7.14163989	-3.619359878	0.000356	0.00066	-0.9566374
RIMBP3	-0.11785167	0.38945237	-3.619287953	0.000356	0.00066	-0.95688495
IFNAR2	0.275115867	2.30358919	3.619287291	0.000356	0.00066	-0.95688723
FPR1	0.477460392	2.15748155	3.619142517	0.000356	0.00066	-0.9573855
PLSCR4	-0.370911897	3.89963328	-3.618830861	0.000356	0.00066	-0.95845807
HMGB3	-0.310831154	4.16421938	-3.618649891	0.000357	0.00066	-0.95908084
AREG	0.677589724	2.20377518	3.618111413	0.000357	0.00066	-0.96093374
KIAA154	0.224297555	0.36128887	3.61689585	0.000359	0.00066	-0.96511556
TMEM25	-0.210751119	4.93270251	-3.616569517	0.000359	0.00066	-0.96623801
PLEKHH	-0.292887348	4.53078602	-3.61619398	0.00036	0.00066	-0.96752958
TBC1D7	-0.151371903	2.12348462	-3.616071204	0.00036	0.00067	-0.96795182
USP54	-0.373175622	5.05959991	-3.616003605	0.00036	0.00067	-0.96818429
UBE2T	-0.271311823	3.31045627	-3.615779917	0.000361	0.00067	-0.96895352
ZNF550	-0.278380341	2.62586953	-3.615360061	0.000361	0.00067	-0.97039723
ITPR3	0.525751406	6.12136417	3.615285741	0.000361	0.00067	-0.97065276
ODC1	-0.425656794	7.0805661	-3.615157613	0.000361	0.00067	-0.9710933
LOXHD1	-0.070233875	0.1240508	-3.61495917	0.000362	0.00067	-0.97177558
SLC25A4	-0.24579119	3.25176818	-3.614462895	0.000362	0.00067	-0.97348169
SET	-0.255246164	7.63626758	-3.613773275	0.000363	0.00067	-0.97585215
INO80	-0.266380303	4.04305641	-3.613128652	0.000364	0.00067	-0.97806756
VWA5B2	-0.278651742	0.91118848	-3.612142788	0.000365	0.00067	-0.98145504
IGF2BP3	-0.375967251	0.40727784	-3.611707894	0.000366	0.00067	-0.9829491
SOGA3	0.063981449	0.19955023	3.610569376	0.000367	0.00068	-0.98685965
RGL2	-0.269423284	5.9114514	-3.60979835	0.000369	0.00068	-0.98950732
PPM1J	-0.324416775	2.37800717	-3.609742162	0.000369	0.00068	-0.98970024

RSPRY1	-0.262552886	4.60114418	-3.608096005	0.000371	0.00068	-0.99535128
HSP90AI	-0.278053585	10.1329732	-3.607703068	0.000371	0.00068	-0.99669984
SLC51B	-0.202742244	0.70456119	-3.607197593	0.000372	0.00069	-0.99843443
GPSM2	-0.186769793	1.18317564	-3.606809412	0.000373	0.00069	-0.99976637
TMSB4X	0.477962378	10.4444238	3.606275829	0.000373	0.00069	-1.00159699
BCKDK	-0.203052366	5.2934542	-3.606198039	0.000373	0.00069	-1.00186386
FGF17	-0.276889338	0.86038081	-3.604728934	0.000375	0.00069	-1.00690274
CCDC19	-0.27459728	3.18584126	-3.604423236	0.000376	0.00069	-1.00795102
AMBRA1	-0.236954064	4.30096341	-3.603858408	0.000377	0.00069	-1.00988768
FAM160I	-0.212705404	4.64188498	-3.60352149	0.000377	0.00069	-1.01104276
APPL1	-0.274983776	4.50840852	-3.603521191	0.000377	0.00069	-1.01104379
CEACAM	0.308353877	1.16459321	3.602987969	0.000378	0.0007	-1.01287167
DIPK1B	-0.430283013	4.1019409	-3.602937032	0.000378	0.0007	-1.01304627
FCN2	-0.088599789	0.1034869	-3.602903449	0.000378	0.0007	-1.01316138
OR52N4	0.180117434	0.47272434	3.602181564	0.000379	0.0007	-1.01563557
WDR43	-0.252237888	4.48833486	-3.601517885	0.00038	0.0007	-1.01790986
TEKT1	-0.112041436	0.11218501	-3.601069434	0.00038	0.0007	-1.01944639
ARHGEF	-0.357143078	2.49139686	-3.600864989	0.000381	0.0007	-1.02014683
CCDC66	-0.222991289	2.57541788	-3.599884376	0.000382	0.0007	-1.02350594
TPSAB1	0.505844726	1.28477882	3.599531345	0.000383	0.0007	-1.02471506
PPP4C	-0.206861239	6.59492551	-3.599443844	0.000383	0.0007	-1.02501473
YES1	-0.274411247	4.83476493	-3.599117899	0.000383	0.0007	-1.02613096
CCDC11	-0.217413497	2.46102437	-3.599083964	0.000383	0.0007	-1.02624716
EGR2	-0.724019605	4.93087956	-3.598486637	0.000384	0.00071	-1.02829251
CR1	0.141780762	0.22482178	3.598143076	0.000385	0.00071	-1.02946878
MCOLN2	0.287524138	1.09603517	3.596975937	0.000386	0.00071	-1.03346402
ANKRD1	-0.27354964	4.12149546	-3.5952989	0.000389	0.00071	-1.03920264
PTCHD4	0.528333654	3.29495169	3.595155283	0.000389	0.00071	-1.03969397
EXD1	-0.027437595	0.04965535	-3.594919219	0.000389	0.00072	-1.04050153
CYP39A1	-0.430822687	3.09776586	-3.594737713	0.000389	0.00072	-1.04112242
SMG8	-0.3201477	3.50596439	-3.594669585	0.00039	0.00072	-1.04135546
SOX30	-0.131979357	0.35683015	-3.593632381	0.000391	0.00072	-1.04490288
MAN1A2	-0.311592569	4.64048722	-3.593486501	0.000391	0.00072	-1.04540174
ADIPOR1	-0.187614509	6.62095237	-3.593455211	0.000391	0.00072	-1.04550874
FAM102A	-0.282776223	5.45664348	-3.592357419	0.000393	0.00072	-1.04926221
ZXDA	-0.230431094	2.04130988	-3.59123204	0.000394	0.00072	-1.05310892
RFC4	-0.186031067	3.13082175	-3.590764129	0.000395	0.00073	-1.05470799
TCN1	0.313837175	0.49333357	3.590303902	0.000396	0.00073	-1.05628062
ADAT1	-0.246726107	3.73805518	-3.589886573	0.000396	0.00073	-1.0577065
HS3ST1	0.276138007	1.14524932	3.589499368	0.000397	0.00073	-1.05902933

SLC30A8	-0.288167752	0.33200325	-3.588904111	0.000398	0.00073	-1.06106268
LMO1	-0.161191469	0.33675245	-3.588012179	0.000399	0.00073	-1.06410888
POU2AF	0.556239677	1.45622509	3.587949712	0.000399	0.00073	-1.06432219
EN2	-0.046104656	0.04760459	-3.587628976	0.0004	0.00073	-1.06541741
PADI2	0.303185519	1.43355589	3.587450544	0.0004	0.00073	-1.06602666
TBX4	-0.027756815	0.04441372	-3.587350989	0.0004	0.00073	-1.06636658
WDR82	-0.273509933	6.30543141	-3.586907684	0.000401	0.00074	-1.06788009
GULP1	0.371421917	1.37654593	3.586513147	0.000401	0.00074	-1.06922695
NPM3	-0.288181369	5.33254608	-3.585984269	0.000402	0.00074	-1.07103221
ZSCAN1(	-0.200725565	3.66989006	-3.585842033	0.000402	0.00074	-1.07151767
HIST1H2	-0.286261996	0.86216741	-3.585684972	0.000402	0.00074	-1.07205372
ANLN	0.277236167	1.16495561	3.584518201	0.000404	0.00074	-1.0760352
MIF4GD	-0.266609068	5.02513929	-3.583284171	0.000406	0.00074	-1.08024492
DCUN1D	0.312022492	3.23805051	3.582913911	0.000407	0.00075	-1.08150775
VSIR	-0.267598862	4.96934888	-3.581831688	0.000408	0.00075	-1.08519818
PCDHB1	-0.338606418	1.46121419	-3.581458023	0.000409	0.00075	-1.08647216
TAFA5	-0.40228772	1.95825199	-3.581354112	0.000409	0.00075	-1.08682641
IZUMO1	-0.068770104	0.22580347	-3.581173959	0.000409	0.00075	-1.08744057
WT1	0.109422047	0.12996877	3.580638473	0.00041	0.00075	-1.08926592
PRH1	-0.125331636	0.81242701	-3.58050628	0.00041	0.00075	-1.0897165
RRS1	-0.212081286	4.85476976	-3.579376281	0.000412	0.00075	-1.09356749
MS4A6A	0.447625921	3.16588827	3.579237245	0.000412	0.00075	-1.09404124
SLC35D3	0.088779554	0.09406577	3.578869889	0.000413	0.00076	-1.09529289
ZSCAN3(	-0.257447623	3.2048026	-3.5781475	0.000414	0.00076	-1.09775386
EPPIN	-0.163916601	0.12294683	-3.578087839	0.000414	0.00076	-1.09795709
MT1A	-0.517663898	2.17523736	-3.577537808	0.000415	0.00076	-1.09983056
MSL2	-0.248878326	4.43689144	-3.577067101	0.000415	0.00076	-1.10143364
RBMS1	-0.23669608	3.71102207	-3.576801691	0.000416	0.00076	-1.10233746
MPST	-0.242625823	3.92421987	-3.57403021	0.00042	0.00077	-1.11177173
WDR74	-0.201810881	4.243103	-3.573063617	0.000421	0.00077	-1.11506051
C21orf62	-0.045633512	0.15356185	-3.57275542	0.000422	0.00077	-1.11610896
SKIL	0.298378231	4.46818912	3.572402089	0.000422	0.00077	-1.11731085
PJVK	-0.250171195	1.56513277	-3.572157809	0.000423	0.00077	-1.11814174
OSGEPL	-0.195313037	3.00907065	-3.570858319	0.000425	0.00078	-1.1225609
ACTRT3	-0.276350631	1.96376844	-3.570529181	0.000425	0.00078	-1.12367996
KIAA075	-0.258440221	3.51971803	-3.570305685	0.000426	0.00078	-1.12443979
SNX30	0.284856156	2.56950678	3.569658124	0.000427	0.00078	-1.1266411
CNTROB	-0.215302002	4.09337511	-3.569022575	0.000428	0.00078	-1.12880121
SHB	-0.228468588	4.20128087	-3.567032689	0.000431	0.00079	-1.13556223
TMX4	-0.309391919	5.50147462	-3.566410841	0.000432	0.00079	-1.13767437

A3GALT2	-0.090261866	0.17665051	-3.566135302	0.000432	0.00079	-1.13861015
ANKRD3	-0.077734105	0.25872568	-3.565902213	0.000433	0.00079	-1.13940171
GPR62	-0.204467701	0.49411143	-3.565477097	0.000433	0.00079	-1.14084527
IRF2	-0.243220639	5.20730286	-3.565093659	0.000434	0.00079	-1.14214717
SLC4A1	-0.11643063	0.12888548	-3.563645902	0.000436	0.0008	-1.14706163
IL7	0.248989537	1.02026237	3.56364532	0.000436	0.0008	-1.14706361
RRH	-0.076338397	0.28728302	-3.562833441	0.000437	0.0008	-1.14981877
SP140L	0.252473114	2.2608041	3.562162437	0.000438	0.0008	-1.15209543
KCTD16	-0.1746734	0.21812974	-3.561024336	0.00044	0.0008	-1.15595603
CYP2C8	-0.328673832	1.18621948	-3.560303098	0.000441	0.00081	-1.158402
ZAP70	0.408680837	1.38456149	3.560163764	0.000442	0.00081	-1.15887448
MINDY2	-0.297869999	3.60684032	-3.559607857	0.000443	0.00081	-1.16075938
ZNF341	-0.174340922	2.19460053	-3.558915412	0.000444	0.00081	-1.16310686
IQUB	-0.247111951	1.54060407	-3.557635303	0.000446	0.00081	-1.16744551
VPS35L	-0.187676121	4.4549747	-3.555851704	0.000449	0.00082	-1.17348827
CDC42BI	0.35299971	4.76337858	3.555704297	0.000449	0.00082	-1.17398756
TEX30	-0.214468006	3.00216128	-3.554635263	0.000451	0.00082	-1.17760795
SART3	-0.251129936	4.62025883	-3.554561159	0.000451	0.00082	-1.17785888
ZNF207	-0.214594107	4.48795108	-3.55364254	0.000452	0.00082	-1.18096903
SLC11A1	0.296748241	1.69945362	3.553608161	0.000452	0.00082	-1.18108541
SLC25A5	-0.117519841	1.04984958	-3.553304303	0.000453	0.00083	-1.18211401
PPP1R1A	-0.334364331	0.8971049	-3.553047908	0.000453	0.00083	-1.18298188
TGIF1	0.331784143	4.7730771	3.551685727	0.000455	0.00083	-1.18759175
ADD2	-0.297702489	0.58568547	-3.551236679	0.000456	0.00083	-1.18911106
C14orf18	-0.135342823	0.12632762	-3.55112677	0.000456	0.00083	-1.1894829
DCAF10	-0.229420033	3.84454085	-3.550427331	0.000458	0.00083	-1.19184897
NXPE4	0.116834029	0.11394137	3.550228314	0.000458	0.00083	-1.19252213
SLC35E4	-0.175130193	1.37690629	-3.549698284	0.000459	0.00084	-1.19431475
KLHL34	0.137695233	0.17368824	3.549466917	0.000459	0.00084	-1.19509719
GALR3	-0.076743427	0.12205432	-3.549214337	0.00046	0.00084	-1.1959513
UNC5D	-0.310735755	0.4526059	-3.549196161	0.00046	0.00084	-1.19601277
SLC16A1	-0.556656931	4.024091	-3.549011098	0.00046	0.00084	-1.19663853
RPUSD4	-0.206957955	3.80872194	-3.547144552	0.000463	0.00084	-1.20294836
INPP5E	-0.209841983	4.02792829	-3.54691889	0.000463	0.00084	-1.203711
PLK3	0.416115348	4.68949021	3.546379007	0.000464	0.00085	-1.20553541
BBS7	-0.201587846	2.8440525	-3.545754142	0.000465	0.00085	-1.20764667
IL1R2	0.37892824	1.04538901	3.54543812	0.000466	0.00085	-1.2087143
POLR1A	-0.246848999	3.3273508	-3.545143018	0.000466	0.00085	-1.20971117
EFEMP2	-0.404692483	6.05933068	-3.543965687	0.000468	0.00085	-1.21368754
FRRS1	-0.286392594	1.62618098	-3.543936178	0.000468	0.00085	-1.21378719

TBC1D5	-0.273429772	4.39181411	-3.543820636	0.000469	0.00085	-1.21417736
CDC5L	-0.258436947	4.55733377	-3.543751436	0.000469	0.00085	-1.21441103
ZNF846	-0.199688544	2.483311	-3.543373341	0.000469	0.00085	-1.2156877
RNASEH	-0.219969582	4.50951236	-3.542529596	0.000471	0.00086	-1.21853624
MIER3	-0.268524771	3.73505135	-3.542116474	0.000471	0.00086	-1.21993074
TENT5D	-0.038400476	0.04337794	-3.542033173	0.000472	0.00086	-1.2202119
RNF123	-0.284048258	3.53178211	-3.541443482	0.000473	0.00086	-1.22220212
SYK	-0.263187536	5.72796819	-3.540764374	0.000474	0.00086	-1.22449374
TTC21B	-0.215651835	2.65576363	-3.540546157	0.000474	0.00086	-1.22523002
CHRNB4	-0.19469067	0.40920711	-3.540108672	0.000475	0.00086	-1.226706
LRRC25	0.368509553	2.23555817	3.539207478	0.000476	0.00087	-1.22974593
XRCC5	-0.252778566	7.39124309	-3.53916277	0.000477	0.00087	-1.22989672
SGK2	0.231854365	0.89020394	3.538513271	0.000478	0.00087	-1.23208716
ZNF827	-0.269163653	3.50464361	-3.5383854	0.000478	0.00087	-1.23251836
ANKRD3	-0.07655889	0.07834738	-3.538364512	0.000478	0.00087	-1.2325888
NME8	-0.316134009	0.58255441	-3.537024182	0.00048	0.00087	-1.23710774
ATP5MG	-0.055094754	0.11908146	-3.537007718	0.00048	0.00087	-1.23716324
SYNPO	0.354803743	5.13893958	3.536383989	0.000481	0.00087	-1.23926562
DDX60	0.418018773	3.92065452	3.536332776	0.000481	0.00087	-1.23943822
EFCAB1	-0.291968338	6.27252051	-3.536323454	0.000481	0.00087	-1.23946964
PTBP1	-0.186128419	6.71910723	-3.536022338	0.000482	0.00088	-1.24048447
PRMT2	-0.201396981	5.11318811	-3.534828241	0.000484	0.00088	-1.24450804
FAM161	-0.249457384	2.42360185	-3.531286059	0.00049	0.00089	-1.25643638
HAS1	0.390102218	0.45291533	3.531233944	0.00049	0.00089	-1.2566118
CEP95	-0.260888223	3.60021557	-3.530647682	0.000491	0.00089	-1.25858497
PAM16	-0.202097705	3.23331029	-3.529929996	0.000493	0.00089	-1.26100007
PHF21A	-0.233253646	3.76718775	-3.529726287	0.000493	0.00089	-1.26168549
CPTP	-0.206179688	5.48773268	-3.529036064	0.000494	0.0009	-1.26400763
GSN	0.457983899	8.19114227	3.527697371	0.000497	0.0009	-1.26851027
MFSD6	-0.28478271	5.83864165	-3.527229009	0.000497	0.0009	-1.27008522
STUM	-0.209680908	0.37896627	-3.526940465	0.000498	0.0009	-1.2710554
PCNX4	-0.232298682	2.82420515	-3.526362593	0.000499	0.00091	-1.27299819
AP5Z1	-0.187729486	3.92404262	-3.525473293	0.000501	0.00091	-1.27598743
ZSCAN5	-0.031224206	0.06446589	-3.525301546	0.000501	0.00091	-1.27656465
DAPL1	-0.567477482	6.1213567	-3.524984015	0.000502	0.00091	-1.27763177
ZNF182	-0.22998486	3.48420023	-3.524523316	0.000502	0.00091	-1.27917987
WNT9B	0.099332793	0.2652869	3.523595888	0.000504	0.00091	-1.28229578
ZNF575	-0.158661118	2.01107544	-3.522700698	0.000506	0.00092	-1.28530268
TAGAP	0.419308964	1.91322342	3.522490648	0.000506	0.00092	-1.28600812
C5AR1	0.410075519	2.74514209	3.522117561	0.000507	0.00092	-1.28726103

ECE1	0.389378818	7.66588174	3.521478799	0.000508	0.00092	-1.28940585
ADAMTS	0.200406531	1.09561345	3.519841809	0.000511	0.00093	-1.29490089
ZC3H14	-0.24826614	3.16962082	-3.519783945	0.000511	0.00093	-1.29509508
CAGE1	-0.030150523	0.06317499	-3.519219587	0.000512	0.00093	-1.29698895
LURAP1I	0.561155031	3.46059354	3.518990365	0.000512	0.00093	-1.29775809
CDH8	-0.086692021	0.16937176	-3.518945988	0.000513	0.00093	-1.29790699
ASIC1	-0.237497145	0.81856796	-3.518868363	0.000513	0.00093	-1.29816744
CHIC1	-0.234682626	3.25614657	-3.517528658	0.000515	0.00093	-1.3026617
GORAB	-0.251288943	3.07418239	-3.51727613	0.000516	0.00093	-1.30350867
TAF5L	-0.2348965	3.52578598	-3.516538776	0.000517	0.00094	-1.30598142
VWCE	0.270977368	1.07654415	3.515838344	0.000518	0.00094	-1.30832992
ANKRD3	-0.022202834	0.02384742	-3.515583934	0.000519	0.00094	-1.30918283
IL11	0.162255556	0.25002588	3.515165481	0.00052	0.00094	-1.31058558
SGMS1	0.35847913	4.84892617	3.514618239	0.000521	0.00094	-1.31241982
RANBP3	-0.200280759	5.01066369	-3.513746334	0.000522	0.00094	-1.31534174
APOOL	-0.292664532	3.78561562	-3.513416078	0.000523	0.00095	-1.31644832
FYTTD1	-0.228247472	4.82648471	-3.511886434	0.000526	0.00095	-1.32157241
TMSB10	0.444373194	12.0301923	3.511160846	0.000527	0.00095	-1.32400232
ZNF140	-0.253882219	3.41450058	-3.509283631	0.000531	0.00096	-1.33028679
RAPGEF	-0.33226567	5.20063728	-3.508805972	0.000532	0.00096	-1.33188539
CASZ1	-0.234922184	1.10130951	-3.508609869	0.000532	0.00096	-1.33254164
C6orf163	-0.113494771	0.50440328	-3.508544888	0.000532	0.00096	-1.33275908
FAIM2	0.234067203	0.49812304	3.507188621	0.000535	0.00097	-1.33729677
MAP2K4	-0.23770863	4.55455212	-3.506560363	0.000536	0.00097	-1.33939821
TBL1XR1	-0.261444846	5.64937337	-3.504563572	0.00054	0.00098	-1.34607492
WDSUB1	-0.296874589	4.96183842	-3.503787553	0.000541	0.00098	-1.34866878
FAM193A	-0.2115746	4.19759363	-3.50277179	0.000543	0.00098	-1.35206321
VIRMA	-0.220901207	3.88643037	-3.502460925	0.000544	0.00098	-1.35310186
STRIP1	-0.226186986	3.75544469	-3.501936721	0.000545	0.00098	-1.35485312
CST7	0.570185713	3.14195874	3.500937903	0.000547	0.00099	-1.35818933
CYS1	-0.975467663	4.00217524	-3.500605278	0.000547	0.00099	-1.35930015
ZNF157	-0.138802955	0.55116019	-3.500563565	0.000547	0.00099	-1.35943945
ZMAT3	0.372764947	4.79231646	3.500119852	0.000548	0.00099	-1.36092109
HRCT1	-0.396047499	2.6012705	-3.500030872	0.000548	0.00099	-1.3612182
NR1H2	-0.177471276	6.5702324	-3.499282564	0.00055	0.00099	-1.3637165
CXCL9	0.971142391	3.69362754	3.498934557	0.000551	0.00099	-1.36487819
MFAP5	0.593653218	1.44346511	3.497312863	0.000554	0.001	-1.37029024
MAP4K3	-0.304570775	4.28021111	-3.496665666	0.000555	0.001	-1.37244948
CABLES1	-0.208895641	3.48112367	-3.496397418	0.000556	0.001	-1.37334433
RPL22L1	-0.2590312	4.45189999	-3.496298135	0.000556	0.001	-1.37367551

RIPK3	-0.368594506	3.56786824	-3.495998001	0.000556	0.001	-1.37467663
GJA3	-0.343430028	0.64135523	-3.495941206	0.000557	0.001	-1.37486606
TIGD4	-0.2014244	1.14143385	-3.495615181	0.000557	0.00101	-1.37595344
SREK1IF	-0.176241666	3.58045234	-3.495458742	0.000558	0.00101	-1.37647516
TBC1D1	-0.232527011	5.22661675	-3.494996101	0.000558	0.00101	-1.37801797
PLA2G4(	-0.305110945	2.38215611	-3.492900293	0.000563	0.00101	-1.38500467
CEP57	-0.226635043	4.06388097	-3.492884353	0.000563	0.00101	-1.3850578
CCDC85	-0.239287896	2.70419958	-3.492691654	0.000563	0.00102	-1.3857
STAB2	-0.103054308	0.15060717	-3.492568716	0.000563	0.00102	-1.38610969
CALML6	-0.093141051	0.23678165	-3.491927855	0.000565	0.00102	-1.38824516
SMR3B	-0.557762482	0.51919153	-3.491494813	0.000565	0.00102	-1.38968793
RHPN2	-0.300648869	2.79805568	-3.491464774	0.000566	0.00102	-1.38978801
CITED1	1.074056485	7.2504957	3.489892001	0.000569	0.00102	-1.39502664
HIST1H2	-0.297994079	1.67491498	-3.48973055	0.000569	0.00103	-1.39556428
PCNX3	-0.223198825	4.82119536	-3.489081243	0.00057	0.00103	-1.39772629
DGCR8	-0.215347321	3.80201813	-3.488567052	0.000571	0.00103	-1.39943814
LBH	-0.334705723	7.21694396	-3.488542783	0.000571	0.00103	-1.39951894
MAFG	-0.298363874	4.25336705	-3.487992224	0.000573	0.00103	-1.4013516
ABHD13	-0.251089881	3.66516114	-3.48792348	0.000573	0.00103	-1.40158041
COL12A1	0.508146602	2.43907101	3.487223804	0.000574	0.00103	-1.40390903
ICK	-0.24888062	3.28747234	-3.486911524	0.000575	0.00103	-1.4049482
PIP5KL1	0.432857034	2.11153313	3.486540689	0.000576	0.00104	-1.40618211
FBXO27	-0.287290079	1.72003085	-3.486339613	0.000576	0.00104	-1.40685112
LPA	-0.066869595	0.06021763	-3.486200319	0.000576	0.00104	-1.40731455
TSR1	-0.230079257	3.99205968	-3.485945595	0.000577	0.00104	-1.40816198
ANKRD1	0.238720884	1.84021045	3.485150907	0.000578	0.00104	-1.4108054
CDH17	0.088127464	0.21670879	3.484873347	0.000579	0.00104	-1.41172854
MFSD14I	-0.248524064	5.73298616	-3.484528463	0.00058	0.00104	-1.4128755
SOS1	-0.268614547	4.09439296	-3.484181974	0.00058	0.00104	-1.4140277
FKBP10	-0.38624838	5.95621378	-3.484002141	0.000581	0.00104	-1.41462566
ANGPTL:	-0.108805682	0.41202053	-3.48382079	0.000581	0.00105	-1.41522865
LRRC49	-0.193768592	1.49693919	-3.483763409	0.000581	0.00105	-1.41541943
MAP2K3	-0.214147421	5.3148895	-3.482572122	0.000584	0.00105	-1.41937963
SAP30L	-0.236023105	4.04324104	-3.482355415	0.000584	0.00105	-1.4200999
PELI2	-0.249953582	1.92992552	-3.482351987	0.000584	0.00105	-1.4201113
PLPP2	0.553523834	3.19584915	3.481929263	0.000585	0.00105	-1.42151618
LPCAT3	-0.396984848	3.18186097	-3.481384237	0.000586	0.00105	-1.42332731
DGKB	-0.040704495	0.09725356	-3.481358326	0.000586	0.00105	-1.4234134
SFPQ	-0.229122367	6.53777441	-3.480194602	0.000589	0.00106	-1.42727956
RARG	0.327841599	4.9819014	3.479862801	0.000589	0.00106	-1.42838167

TMEM22	-0.294332073	2.42261917	-3.479232646	0.000591	0.00106	-1.43047452
KLF12	0.297758005	1.99342327	3.47904282	0.000591	0.00106	-1.4311049
DNAH12	-0.042583451	0.0940082	-3.478011704	0.000593	0.00107	-1.43452849
ABCC9	-0.299502614	1.49412629	-3.477848494	0.000594	0.00107	-1.43507031
ARHGEF	-0.12331089	0.65648572	-3.477514023	0.000594	0.00107	-1.4361806
KLC1	-0.203867139	2.6333938	-3.47736145	0.000595	0.00107	-1.43668704
CYSLTR:	-0.223997756	1.23378399	-3.477205501	0.000595	0.00107	-1.43720467
CTBP1	-0.176382343	5.73296351	-3.476489948	0.000597	0.00107	-1.43957946
RABIF	-0.191468512	3.82125081	-3.475996443	0.000598	0.00107	-1.44121706
LRRC71	-0.067899892	0.13629064	-3.475671996	0.000598	0.00107	-1.44229355
PJA2	-0.331486594	6.74794472	-3.47564174	0.000598	0.00107	-1.44239394
GJA4	-0.489528638	6.34775583	-3.475362491	0.000599	0.00107	-1.44332039
TREML1	0.206165852	0.68446466	3.474775851	0.0006	0.00108	-1.44526644
RGS11	0.355212089	1.47932904	3.473902373	0.000602	0.00108	-1.44816345
MAT2B	-0.279227174	6.06244345	-3.473839197	0.000602	0.00108	-1.44837296
TNFSF1:	-0.371306266	4.79060362	-3.473377294	0.000603	0.00108	-1.44990464
KIAA121	0.442997896	6.16197797	3.47273614	0.000605	0.00108	-1.4520304
NLRP3	0.341042612	1.5175993	3.472540797	0.000605	0.00109	-1.452678
DCTN4	-0.213074127	5.24273792	-3.472421261	0.000605	0.00109	-1.45307426
FGFR1O	-0.222286369	4.42232669	-3.472383835	0.000605	0.00109	-1.45319833
YEATS2	-0.2613832	3.65594688	-3.472340502	0.000605	0.00109	-1.45334198
FZD10	0.240261966	0.81709329	3.471791496	0.000607	0.00109	-1.45516175
RPL8	-0.226786633	10.840045	-3.471711998	0.000607	0.00109	-1.45542524
NOL8	-0.207202041	3.53225077	-3.470435175	0.00061	0.00109	-1.45965638
ACBD3	-0.315937479	5.70232348	-3.46885546	0.000613	0.0011	-1.4648893
TRAP1	-0.260256487	4.41117495	-3.467972604	0.000615	0.0011	-1.46781288
USP12	-0.250177421	4.22771367	-3.465592545	0.00062	0.00111	-1.47569106
ZFP30	-0.219178468	2.34401951	-3.464931283	0.000622	0.00111	-1.47787902
XPO5	-0.23456134	4.19719036	-3.464701721	0.000622	0.00111	-1.4786385
GALR1	-0.056183963	0.07951556	-3.462288132	0.000627	0.00112	-1.48662078
RNASE3	0.080448227	0.15495081	3.46139357	0.000629	0.00113	-1.48957801
DDIAS	0.114491662	0.54247513	3.460798688	0.000631	0.00113	-1.49154418
KLHL15	-0.289549687	2.97801306	-3.460546508	0.000631	0.00113	-1.49237758
HSF1	-0.179072273	5.81070591	-3.459256612	0.000634	0.00114	-1.49663952
PARVB	-0.254370199	2.62410183	-3.458727684	0.000635	0.00114	-1.49838674
NEDD8-M	-0.081693461	0.27352278	-3.458675677	0.000636	0.00114	-1.49855852
PTPN2	-0.192904787	3.94149316	-3.457749634	0.000638	0.00114	-1.5016169
MSH3	-0.240573146	3.81746298	-3.457678866	0.000638	0.00114	-1.50185059
GNE	-0.271901986	3.4189628	-3.457152305	0.000639	0.00114	-1.50358926
FAM20B	-0.234265443	5.00925604	-3.456941019	0.000639	0.00114	-1.50428685

MTRNR2	0.146752463	0.38174298	3.456828086	0.00064	0.00114	-1.50465969
ST20-MT	-0.114628705	0.40107034	-3.456676662	0.00064	0.00115	-1.5051596
PVR	-0.236740045	4.47154552	-3.456641193	0.00064	0.00115	-1.50527669
PUM2	-0.24873527	6.13963292	-3.456265507	0.000641	0.00115	-1.50651687
RELT	0.239936356	1.81071272	3.455765931	0.000642	0.00115	-1.50816583
GPRIN2	-0.340488467	2.91400747	-3.455764324	0.000642	0.00115	-1.50817114
GTF3C3	-0.222692642	3.63567227	-3.455677493	0.000642	0.00115	-1.50845772
ARHGEF	-0.283244026	4.40120596	-3.455129698	0.000644	0.00115	-1.51026555
H3F3C	-0.147199251	0.8667016	-3.454915248	0.000644	0.00115	-1.5109732
CLASP2	-0.230185948	2.90381388	-3.454509606	0.000645	0.00115	-1.51231165
IRAK2	0.388917309	3.4480877	3.454421641	0.000645	0.00115	-1.51260188
GYG1	-0.215523259	4.92787157	-3.454327805	0.000645	0.00115	-1.51291148
BZW1	-0.254571197	4.7503028	-3.454237135	0.000646	0.00115	-1.51321061
INAFM1	-0.298330413	4.93768371	-3.453973989	0.000646	0.00115	-1.51407875
CDAN1	-0.207447933	3.27027748	-3.453895684	0.000646	0.00115	-1.51433707
PLEKHF:	-0.252131193	4.06668557	-3.453817729	0.000647	0.00115	-1.51459423
GDPD2	-0.063241442	0.21215815	-3.453252573	0.000648	0.00116	-1.51645844
TAS1R3	0.377807888	1.47869715	3.452844181	0.000649	0.00116	-1.51780537
WDR6	-0.275948511	6.89423872	-3.452355586	0.00065	0.00116	-1.51941664
SLC9A8	-0.240430456	3.85699862	-3.452229426	0.00065	0.00116	-1.51983265
PRPF4B	-0.268082753	4.71359428	-3.4505143	0.000654	0.00117	-1.52548687
ACSL5	0.454005641	3.96832246	3.449848922	0.000656	0.00117	-1.52767972
MEIOC	-0.035410194	0.1321608	-3.449626199	0.000656	0.00117	-1.52841366
CYSRT1	0.422244247	3.14300471	3.449283956	0.000657	0.00117	-1.52954135
DOCK9	0.43356779	5.60770651	3.449045603	0.000658	0.00117	-1.53032667
ATN1	-0.240053519	7.27041277	-3.447568596	0.000661	0.00118	-1.53519195
SH2D1A	0.413074218	1.06833717	3.445976453	0.000665	0.00119	-1.54043437
PPP1R1:	-0.188398269	4.58201326	-3.445604173	0.000666	0.00119	-1.54165985
FBN3	0.534719486	2.70271862	3.445213701	0.000667	0.00119	-1.54294509
API5	-0.234629312	5.93439378	-3.444832586	0.000667	0.00119	-1.5441994
SERTAD	-0.318118006	5.41078758	-3.444738116	0.000668	0.00119	-1.5445103
MUC4	0.184069401	0.30528868	3.444631929	0.000668	0.00119	-1.54485975
TMEM31	-0.153632994	0.46530743	-3.444276551	0.000669	0.00119	-1.54602917
ZSCAN5	-0.034676184	0.07311788	-3.443632214	0.00067	0.0012	-1.54814919
DDX3X	-0.312721049	6.53036349	-3.443425193	0.000671	0.0012	-1.54883026
CREG2	0.046056007	0.11036224	3.442573952	0.000673	0.0012	-1.55163033
PARP4	0.398980069	5.80788748	3.441814761	0.000675	0.0012	-1.55412708
SYCE2	-0.157347894	1.04520656	-3.441736268	0.000675	0.0012	-1.55438519
HLA-A	0.39040971	10.3698177	3.441263109	0.000676	0.0012	-1.55594098
UNC13C	-0.174211627	0.33961199	-3.440722559	0.000677	0.00121	-1.55771812

IDUA	-0.310145479	3.50851799	-3.440384337	0.000678	0.00121	-1.55882994
PCP2	-0.408346462	2.36500308	-3.440230236	0.000678	0.00121	-1.55933648
KDM6A	-0.326646888	3.78076757	-3.43987498	0.000679	0.00121	-1.56050415
CLN8	-0.242268035	3.19775232	-3.439854008	0.000679	0.00121	-1.56057308
RPS5	-0.262099137	8.61223412	-3.438706562	0.000682	0.00121	-1.56434377
RBM15B	-0.203709348	5.13894226	-3.43789741	0.000684	0.00122	-1.56700208
IL6R	0.361295643	2.11362011	3.437138773	0.000686	0.00122	-1.56949392
APOC2	0.109425655	0.25825183	3.436996337	0.000686	0.00122	-1.56996171
CRTAP	-0.20842713	6.65351899	-3.436696798	0.000687	0.00122	-1.57094541
TSPYL4	-0.32340163	4.93500067	-3.43631398	0.000688	0.00122	-1.57220248
GNB1	-0.205190445	7.25768091	-3.433887038	0.000694	0.00123	-1.58016897
TMEM26	-0.237897438	3.3663718	-3.433875037	0.000694	0.00123	-1.58020835
KCNB1	-0.191925613	0.26863436	-3.433840697	0.000694	0.00123	-1.58032103
VPS37A	-0.212392096	4.02525127	-3.433645521	0.000694	0.00124	-1.58096147
NUDT21	-0.275943097	5.3860029	-3.433252971	0.000695	0.00124	-1.58224946
ENGASE	-0.278231466	3.56054908	-3.431664712	0.000699	0.00124	-1.58745929
ARRDC5	0.121553074	0.26863432	3.431146395	0.0007	0.00125	-1.58915901
TMEM13	0.285698094	3.31449614	3.430254764	0.000703	0.00125	-1.59208239
NPFF	-0.138142303	0.60522557	-3.429894107	0.000704	0.00125	-1.59326467
RAP1B	-0.189202568	2.76110373	-3.42900329	0.000706	0.00125	-1.59618441
SLC38A1	-0.284598284	1.0950741	-3.4287434	0.000706	0.00126	-1.59703609
SNAPC3	-0.198732309	3.87410634	-3.428517044	0.000707	0.00126	-1.59777783
SLC9A6	-0.220582448	3.89514845	-3.427753798	0.000709	0.00126	-1.60027857
NR2C1	-0.231954686	3.41876108	-3.427662723	0.000709	0.00126	-1.60057694
ZNF665	-0.157524679	1.47280452	-3.426186991	0.000713	0.00127	-1.60541055
SP5	-0.160405979	0.22350543	-3.425774501	0.000714	0.00127	-1.60676127
AK1	-0.325467454	5.45307969	-3.425774025	0.000714	0.00127	-1.60676283
GLRX3	-0.190559239	4.60106583	-3.425629655	0.000714	0.00127	-1.60723555
LILRB2	0.309934335	1.45272309	3.425570815	0.000714	0.00127	-1.6074282
ACKR1	0.627492971	3.37643175	3.425457786	0.000715	0.00127	-1.60779828
GAPVD1	-0.245669833	3.68588406	-3.424723436	0.000717	0.00127	-1.61020239
KLHL9	-0.260106243	4.69775708	-3.424529658	0.000717	0.00127	-1.6108367
DPP7	-0.24499141	7.7803882	-3.424318863	0.000718	0.00127	-1.61152668
TNIP3	0.113426656	0.21742622	3.422646167	0.000722	0.00128	-1.6170004
ALKBH6	-0.19628257	1.49837071	-3.422424212	0.000722	0.00128	-1.61772654
POC1A	-0.218906765	2.49561193	-3.422129356	0.000723	0.00128	-1.61869111
KRBA1	-0.202574868	2.88479017	-3.421942698	0.000724	0.00128	-1.6193017
SFR1	-0.185967652	3.69240605	-3.421936908	0.000724	0.00128	-1.61932064
NR4A1	-0.716863414	5.31829129	-3.421538035	0.000725	0.00129	-1.6206253
ASPN	0.592016349	3.63168592	3.421245682	0.000725	0.00129	-1.62158146

SAMHD1	0.364668679	5.04104941	3.420677156	0.000727	0.00129	-1.62344065
TSHZ1	-0.254545701	3.53094224	-3.420362946	0.000728	0.00129	-1.62446806
IL21R	0.351367955	1.21364292	3.419865473	0.000729	0.00129	-1.62609453
ANKRD2	-0.024155024	0.03723525	-3.419436635	0.00073	0.00129	-1.62749642
TMEM91	-0.337510405	3.12669608	-3.418549073	0.000732	0.0013	-1.6303974
SLFN12L	0.168315336	0.49039713	3.417745407	0.000734	0.0013	-1.63302357
STAP1	0.338415335	0.6350249	3.417729512	0.000734	0.0013	-1.6330755
CDA	-0.387783559	3.89594685	-3.417535037	0.000735	0.0013	-1.63371091
WASL	-0.26358597	5.85975858	-3.416948077	0.000736	0.00131	-1.63562848
ASB2	0.243841435	0.76246639	3.416907673	0.000736	0.00131	-1.63576047
TRIOBP	-0.186489898	3.39619864	-3.416801101	0.000737	0.00131	-1.6361086
CYP8B1	-0.036332491	0.07767042	-3.415952328	0.000739	0.00131	-1.63888086
SH3PXD:	0.292272192	3.29481885	3.415247445	0.000741	0.00131	-1.64118267
ERCC4	-0.216968844	2.01522289	-3.415030007	0.000741	0.00131	-1.64189263
ADAM9	-0.391929928	5.30354335	-3.414463441	0.000743	0.00132	-1.64374234
ERVMER	-0.201773688	0.46790147	-3.414284373	0.000743	0.00132	-1.64432689
FAM71E:	-0.252661327	3.41580267	-3.413126985	0.000746	0.00132	-1.64810445
PIM1	0.377226043	4.37675035	3.412974321	0.000747	0.00132	-1.64860264
PON1	0.047873907	0.09203529	3.412606278	0.000748	0.00132	-1.64980359
PCGF2	-0.23218273	5.9138706	-3.412567585	0.000748	0.00132	-1.64992984
TNFRSF:	0.277235886	5.36424128	3.412190919	0.000749	0.00133	-1.65115879
PALM3	0.74440133	4.69229234	3.41169241	0.00075	0.00133	-1.6527851
RPS3	-0.243697028	8.89952872	-3.411304803	0.000751	0.00133	-1.65404945
PCLAF	-0.302415577	2.43463555	-3.411263362	0.000751	0.00133	-1.65418462
ATP1B1	-0.308926074	9.22477933	-3.410894488	0.000752	0.00133	-1.65538773
ORC3	-0.20555008	4.49325214	-3.410874491	0.000752	0.00133	-1.65545294
MMP8	0.051480153	0.04944409	3.410484042	0.000753	0.00133	-1.65672629
CD180	0.351702895	1.08861633	3.410399073	0.000753	0.00133	-1.65700337
TOR1AIF	-0.280078024	4.95724486	-3.410152755	0.000754	0.00133	-1.65780658
VSIG2	-0.558567818	6.25348937	-3.41004471	0.000754	0.00133	-1.65815889
RGS9	-0.252491598	0.88603821	-3.409535946	0.000756	0.00134	-1.65981769
CRYBB2	-0.239960228	0.90610998	-3.409437925	0.000756	0.00134	-1.66013725
CPNE7	0.212504259	0.50944427	3.409129586	0.000757	0.00134	-1.66114244
PIK3IP1	-0.286871883	6.39896597	-3.407871544	0.00076	0.00134	-1.66524281
NEU3	-0.203652986	2.62039269	-3.40719294	0.000762	0.00135	-1.66745402
HDAC5	-0.207197594	5.36627806	-3.40716399	0.000762	0.00135	-1.66754835
ANG	0.384175082	3.12454589	3.406900595	0.000763	0.00135	-1.6684065
SIGLEC1	0.375228801	1.62342007	3.406411429	0.000764	0.00135	-1.67000006
POU2F2	0.320427862	1.15818645	3.405414849	0.000767	0.00135	-1.67324599
ARRDC2	0.341525665	5.58631665	3.404860216	0.000768	0.00136	-1.67505209

DUSP3	-0.222016597	5.71519349	-3.40478272	0.000768	0.00136	-1.67530442
TMEM22	-0.287050846	1.62701241	-3.404492274	0.000769	0.00136	-1.6762501
TMEM40	0.186174056	0.27854989	3.403853748	0.000771	0.00136	-1.67832885
NHS	-0.285434128	1.17647501	-3.403614024	0.000772	0.00136	-1.67910919
RBM26	-0.220726087	4.10021753	-3.402698743	0.000774	0.00137	-1.68208812
C1QL4	-0.5673438	2.55629257	-3.402245955	0.000775	0.00137	-1.68356152
GPR157	-0.257186452	2.95247484	-3.402238546	0.000775	0.00137	-1.68358562
UTP18	-0.252385042	5.46244328	-3.402073654	0.000776	0.00137	-1.68412215
NUDT11	0.576617903	2.05153615	3.401711017	0.000777	0.00137	-1.68530201
OBP2A	0.25360217	0.297076	3.401443345	0.000777	0.00137	-1.68617282
ARHGAF	-0.27697078	3.61592866	-3.401333929	0.000778	0.00137	-1.68652876
C8orf34	0.054230018	0.14125648	3.401332188	0.000778	0.00137	-1.68653442
KIF24	-0.182580982	1.52759221	-3.400863656	0.000779	0.00137	-1.68805849
GTPBP3	-0.180760109	3.09470357	-3.400530334	0.00078	0.00138	-1.68914263
BCO1	-0.096348427	0.12379854	-3.39973631	0.000782	0.00138	-1.69172481
HDAC9	-0.389285931	1.93871975	-3.399503202	0.000783	0.00138	-1.69248277
NECAP2	-0.196289081	5.24683018	-3.397905176	0.000787	0.00139	-1.69767759
LIPN	0.06929533	0.10167552	3.397396221	0.000789	0.00139	-1.69933161
APOBEC	0.330190205	1.93322287	3.397382952	0.000789	0.00139	-1.69937473
ARMCX2	-0.331764353	6.98241077	-3.396185659	0.000792	0.0014	-1.70326485
FCRL2	0.221978043	0.27123673	3.396159394	0.000792	0.0014	-1.70335017
USPL1	-0.235152091	3.59953748	-3.395757671	0.000793	0.0014	-1.70465511
ANKRD3	0.101936	0.45267046	3.394899603	0.000795	0.0014	-1.70744197
VAT1L	0.607063218	2.1241148	3.394150765	0.000798	0.00141	-1.70987353
KPNB1	-0.207466281	6.2210157	-3.394067804	0.000798	0.00141	-1.71014288
PIP4P2	-0.270185795	3.01324624	-3.393899111	0.000798	0.00141	-1.71069057
HIST1H4	-0.180472803	0.36488429	-3.393839273	0.000798	0.00141	-1.71088484
LAG3	0.466212162	1.57686469	3.393529086	0.000799	0.00141	-1.71189182
RBMS3	-0.277970855	4.1049588	-3.393473238	0.000799	0.00141	-1.71207312
TTC29	-0.105832715	0.09443605	-3.39333394	0.0008	0.00141	-1.7125253
SCAF1	-0.198554686	5.94282154	-3.392848459	0.000801	0.00141	-1.7141011
FSD1L	-0.189613418	1.47841213	-3.392521451	0.000802	0.00141	-1.71516241
MID1IP1	-0.275774154	4.70761389	-3.391357121	0.000805	0.00142	-1.7189405
PCGF6	-0.166650154	2.69488025	-3.390894823	0.000807	0.00142	-1.72044026
SLC52A1	-0.306012018	0.94484712	-3.390623688	0.000807	0.00142	-1.72131977
UTP14A	-0.192209577	4.04240405	-3.390466058	0.000808	0.00142	-1.72183107
TMEM30	-0.424077182	6.66965378	-3.389601802	0.00081	0.00143	-1.72463401
ATP8B4	0.135637478	0.63597597	3.389437731	0.000811	0.00143	-1.72516606
TRAF3IP	0.329189955	1.07266507	3.388272289	0.000814	0.00143	-1.72894461
PNMA8B	-0.32306633	1.48005577	-3.387880459	0.000815	0.00143	-1.73021471

ATR	-0.218648125	2.89875474	-3.386880854	0.000818	0.00144	-1.73345431
CLU	0.761720672	10.7478948	3.386682867	0.000819	0.00144	-1.73409586
FAM171/	-0.492077984	3.54459761	-3.386673325	0.000819	0.00144	-1.73412677
MTCP1	-0.14511156	1.07278854	-3.386147499	0.00082	0.00144	-1.73583046
SLC39A4	-0.386605322	3.71550116	-3.38420646	0.000826	0.00145	-1.74211738
LRRC3C	-0.070129591	0.13600285	-3.384127715	0.000826	0.00145	-1.74237236
CXCR2	0.12215382	0.36481782	3.384002479	0.000826	0.00145	-1.74277787
RFK	-0.265751641	4.84706759	-3.383630595	0.000827	0.00145	-1.74398194
TMEM30	-0.284010109	7.18245777	-3.382912759	0.000829	0.00146	-1.74630578
CYP4F12	-0.13214383	0.27809894	-3.382853902	0.00083	0.00146	-1.74649629
HECTD1	-0.300029046	5.20012497	-3.381479749	0.000834	0.00147	-1.75094348
TEDC2	-0.228716229	1.68714981	-3.38125642	0.000834	0.00147	-1.75166609
ATP2C1	-0.263555059	5.08656957	-3.381048189	0.000835	0.00147	-1.7523398
CBX5	-0.24876716	5.04418489	-3.37996549	0.000838	0.00147	-1.75584218
ACOX1	-0.218716784	4.96402871	-3.379815755	0.000838	0.00147	-1.75632647
NR5A2	-0.222383705	0.8620114	-3.379425831	0.00084	0.00147	-1.75758752
ATP8A2	-0.207916823	0.52953219	-3.378771171	0.000842	0.00148	-1.75970445
ATP2B4	0.323934467	5.08510337	3.37866185	0.000842	0.00148	-1.76005792
ACOT7	0.296167916	3.51659465	3.378596375	0.000842	0.00148	-1.76026961
SF3A1	-0.296146664	6.11552519	-3.378568685	0.000842	0.00148	-1.76035914
CPZ	0.291678306	0.92953765	3.378426579	0.000843	0.00148	-1.76081858
SOAT2	0.12958953	0.2979694	3.377714338	0.000845	0.00148	-1.76312106
DGKA	0.291825639	2.45520211	3.37653772	0.000848	0.00149	-1.76692378
ZNF891	-0.136673832	0.96230488	-3.376245943	0.000849	0.00149	-1.76786658
SIT1	0.473365763	1.60778871	3.374478603	0.000854	0.0015	-1.77357573
LZTR1	-0.187330695	4.067143	-3.374340644	0.000855	0.0015	-1.77402127
HTR1D	0.179026668	0.39265378	3.37431261	0.000855	0.0015	-1.7741118
TDRD7	-0.214201354	3.83349609	-3.374311784	0.000855	0.0015	-1.77411447
GDF1	0.287918874	1.37831881	3.374287176	0.000855	0.0015	-1.77419394
CERS1	0.287918874	1.37831881	3.374287176	0.000855	0.0015	-1.77419394
RBMXL1	-0.248709076	3.36723399	-3.374272906	0.000855	0.0015	-1.77424002
SDR9C7	-0.056589062	0.08397569	-3.373584342	0.000857	0.0015	-1.77646347
AMN	-0.155083501	0.37660578	-3.37257853	0.00086	0.00151	-1.77971059
PKN3	-0.221183197	3.42423464	-3.372272507	0.000861	0.00151	-1.78069837
IL10	0.178327974	0.45213586	3.372153605	0.000861	0.00151	-1.78108214
SULF1	0.606173786	4.47850509	3.371772921	0.000862	0.00151	-1.78231075
ZNF708	-0.238131974	2.5856001	-3.370942588	0.000865	0.00152	-1.78499011
VSIG4	0.456730186	3.68624831	3.370420631	0.000866	0.00152	-1.78667409
CLPX	-0.235883587	4.87611703	-3.369326754	0.00087	0.00152	-1.79020244
PLAAT4	0.521861998	6.17201713	3.368992314	0.000871	0.00153	-1.79128099

PAFAH1I	-0.226728541	5.63409959	-3.36897123	0.000871	0.00153	-1.79134898
KRAS	-0.217586771	3.79127098	-3.368384347	0.000872	0.00153	-1.79324139
ARSA	-0.267744278	5.84368498	-3.368148588	0.000873	0.00153	-1.79400151
ARHGAF	0.257882039	2.31837832	3.368084394	0.000873	0.00153	-1.79420847
WNT5B	0.356963505	1.95184396	3.367862142	0.000874	0.00153	-1.79492499
SIK1B	0.46946984	1.9450851	3.366717917	0.000878	0.00154	-1.79861316
EHMT2	-0.206158911	4.91554503	-3.366451855	0.000878	0.00154	-1.79947059
ANO7	-0.167720141	1.18242186	-3.364775312	0.000883	0.00155	-1.80487211
HLA-DRE	0.688384932	7.30950808	3.364356947	0.000885	0.00155	-1.80621963
RGL4	0.177988884	0.78425218	3.363970845	0.000886	0.00155	-1.80746309
LRRC29	-0.235608243	3.48405006	-3.363867062	0.000886	0.00155	-1.80779731
DNAJB14	-0.251531357	3.54465683	-3.363068598	0.000889	0.00156	-1.81036832
SLC38A3	-0.494363409	1.64887821	-3.362893922	0.000889	0.00156	-1.81093069
TRAT1	0.305798885	0.5921622	3.362366373	0.000891	0.00156	-1.81262898
SCNN1G	-0.523648223	3.50633963	-3.362243326	0.000891	0.00156	-1.81302506
PSMB9	0.486943112	4.72493601	3.362220136	0.000891	0.00156	-1.81309971
PCSK6	-0.132770956	0.30342196	-3.362059524	0.000892	0.00156	-1.81361668
UBR2	-0.22624758	4.37631149	-3.361820744	0.000893	0.00156	-1.81438522
UBXN2B	-0.268231969	4.3165395	-3.361670127	0.000893	0.00156	-1.81486997
MYO6	0.304620842	5.05301539	3.360583401	0.000896	0.00157	-1.81836694
CPN2	0.040725901	0.05811922	3.360383131	0.000897	0.00157	-1.81901127
TANC2	0.237094704	1.98083134	3.35918711	0.000901	0.00157	-1.82285853
DACT3	-0.25418692	1.43446538	-3.35794242	0.000905	0.00158	-1.82686102
VCAM1	0.424247917	2.46620958	3.357616536	0.000906	0.00158	-1.82790872
THEMIS	0.24722003	0.63014123	3.357338352	0.000907	0.00158	-1.828803
NUP85	-0.180718935	4.02989556	-3.357096484	0.000907	0.00159	-1.82958048
FCRLA	0.424411181	0.62410193	3.357027096	0.000907	0.00159	-1.82980351
DCBLD2	0.314894922	4.89995682	3.356108757	0.00091	0.00159	-1.83275496
INSYN1	-0.233441455	0.58407131	-3.355570093	0.000912	0.00159	-1.83448582
GAS2L3	0.15318642	0.8684207	3.355022497	0.000914	0.0016	-1.83624513
IMPG1	0.104124651	0.36336073	3.353980696	0.000917	0.0016	-1.83959149
FCRL6	0.24891087	0.88322854	3.353884213	0.000917	0.0016	-1.83990135
DOCK3	0.355352705	4.35683973	3.352804276	0.000921	0.00161	-1.84336911
ZNF98	-0.177759126	0.83543487	-3.3524952	0.000922	0.00161	-1.84436138
MSH6	-0.233779028	3.80208816	-3.352131071	0.000923	0.00161	-1.8455303
CNTD1	-0.122948521	0.77416315	-3.351833908	0.000924	0.00161	-1.84648415
MEF2D	-0.226435989	5.27673408	-3.351097046	0.000926	0.00162	-1.84884906
ULBP3	-0.465549231	3.0748957	-3.349724703	0.000931	0.00162	-1.85325222
FADS6	-0.09465344	0.09540216	-3.349665144	0.000931	0.00162	-1.85344328
ZNF562	-0.178220372	2.65231825	-3.349278549	0.000932	0.00163	-1.85468335

GFRA2	-0.565109032	3.77307642	-3.349019515	0.000933	0.00163	-1.85551417
SMCO3	-0.297662708	0.64690587	-3.34893692	0.000933	0.00163	-1.85577908
FAM172A	-0.245293543	4.21104254	-3.348466442	0.000935	0.00163	-1.8572879
LGALS3E	-0.339845796	8.86844393	-3.347564665	0.000938	0.00164	-1.86017936
WNK4	-0.082637403	0.21137319	-3.346953938	0.00094	0.00164	-1.86213718
CLDN11	0.305153425	1.17668426	3.346951268	0.00094	0.00164	-1.86214574
EXOC4	-0.228839055	4.76034234	-3.346741312	0.00094	0.00164	-1.86281873
INCENP	-0.32915194	3.40498227	-3.346172438	0.000942	0.00164	-1.86464199
ZNF761	-0.213618793	3.00563113	-3.345934427	0.000943	0.00164	-1.86540474
HSP90A/	-0.275217816	8.51105485	-3.3458959	0.000943	0.00164	-1.8655282
POLD3	-0.211117395	3.30902843	-3.344984668	0.000946	0.00165	-1.86844791
AEBP1	0.5228636	5.91008911	3.344431152	0.000948	0.00165	-1.8702211
HIST1H2	-0.413124223	2.5027641	-3.343637395	0.00095	0.00166	-1.87276343
LGI4	-0.291012483	1.5142004	-3.342932552	0.000953	0.00166	-1.87502052
SERPINE	0.085180404	0.10009139	3.342826427	0.000953	0.00166	-1.87536032
ZNF205	-0.208410479	4.59845709	-3.342781169	0.000953	0.00166	-1.87550522
TUBA1B	-0.353335747	6.3842673	-3.342186908	0.000955	0.00166	-1.87740778
RPS20	-0.235189276	9.73198742	-3.342096436	0.000956	0.00166	-1.8776974
CARD14	-0.155638699	0.44550707	-3.341914144	0.000956	0.00167	-1.87828094
IL24	0.318124245	1.45611144	3.341825314	0.000956	0.00167	-1.87856529
MLXIP	-0.262002891	4.88541674	-3.341514171	0.000957	0.00167	-1.8795612
PLPPR4	0.223302232	0.91825536	3.341431925	0.000958	0.00167	-1.87982444
NCOA4	-0.290718167	7.02939073	-3.340563392	0.000961	0.00167	-1.88260396
CLEC16A	0.239822117	3.87553875	3.340357764	0.000961	0.00167	-1.88326192
MAP1LC3	-0.145893501	1.70768028	-3.340201135	0.000962	0.00167	-1.88376308
APC	-0.264772738	2.78043218	-3.339084242	0.000966	0.00168	-1.88733608
WHAMM	-0.197123719	3.73675858	-3.338324366	0.000968	0.00168	-1.88976633
ZNF276	-0.199697606	2.85347516	-3.338129067	0.000969	0.00169	-1.89039086
SIRPD	0.033331044	0.04972375	3.337910393	0.000969	0.00169	-1.8910901
ZMYM4	-0.234166584	4.64726846	-3.337733455	0.00097	0.00169	-1.89165585
ACSS3	-0.31703077	3.06070297	-3.337704387	0.00097	0.00169	-1.89174879
PLA2G6	-0.369587584	3.44594576	-3.337216364	0.000972	0.00169	-1.89330907
GPRASP	-0.360745589	4.44823593	-3.336567401	0.000974	0.00169	-1.89538357
CDRT1	-0.104663496	0.13485929	-3.336099863	0.000976	0.0017	-1.89687789
PREX1	-0.265615581	4.46419663	-3.335754488	0.000977	0.0017	-1.89798164
KATNBL1	-0.182651988	2.92115845	-3.335492437	0.000978	0.0017	-1.89881903
SLX4	-0.175809724	1.90388128	-3.335200147	0.000979	0.0017	-1.89975298
OR2A1	-0.08610086	0.18111147	-3.334664512	0.00098	0.0017	-1.90146429
EFEMP1	-0.579527717	5.25764483	-3.334301351	0.000982	0.00171	-1.90262441
SNX18	-0.300682709	5.11093078	-3.333942923	0.000983	0.00171	-1.90376931

PBX2	-0.252923738	5.81298895	-3.33191015	0.00099	0.00172	-1.91026027
GPR161	0.290523106	1.92233351	3.331890865	0.00099	0.00172	-1.91032183
ZNF567	-0.20767173	2.3990042	-3.33169318	0.00099	0.00172	-1.91095288
CD163	0.49433347	3.29843473	3.331605001	0.000991	0.00172	-1.91123434
GRTP1	-0.293771556	2.12871491	-3.33085717	0.000993	0.00173	-1.91362117
CMTR2	-0.275848509	3.65320461	-3.329475799	0.000998	0.00173	-1.91802876
RUNX3	0.432076241	1.84155093	3.329424971	0.000998	0.00173	-1.91819091
RENBP	0.496479806	4.07055176	3.329067034	0.000999	0.00174	-1.9193327
CBR3	0.420433774	3.36302838	3.327521031	0.001005	0.00174	-1.92426304
SLC39A1	-0.198146554	0.20053458	-3.327246328	0.001006	0.00175	-1.92513887
PSMB8	0.344190254	6.73043002	3.327132106	0.001006	0.00175	-1.92550302
GTDC1	-0.187191191	2.5389903	-3.327115463	0.001006	0.00175	-1.92555608
ZNF7	-0.183796477	2.71447043	-3.326452115	0.001008	0.00175	-1.92767068
MSH4	0.066455997	0.15704865	3.326345734	0.001009	0.00175	-1.92800976
RBM24	-0.210536265	0.54458409	-3.326231022	0.001009	0.00175	-1.92837539
MFSD14	-0.217129462	2.51081078	-3.325920333	0.00101	0.00175	-1.92936559
ZNF426	-0.204749705	3.22693974	-3.325741892	0.001011	0.00175	-1.92993427
CARMIL2	0.266129745	0.64547739	3.32557679	0.001011	0.00175	-1.93046041
PMEPA1	0.30344285	4.99818538	3.325391683	0.001012	0.00176	-1.93105028
DCAF7	-0.261200294	5.73130368	-3.325120386	0.001013	0.00176	-1.93191475
FIGN	0.159479355	0.69348814	3.324902468	0.001014	0.00176	-1.93260908
TOP2B	-0.284507471	6.49582795	-3.324561514	0.001015	0.00176	-1.93369534
GSTT2B	-0.440699394	2.51091912	-3.323720181	0.001018	0.00177	-1.93637536
ZNF184	-0.240703413	3.27339891	-3.322960166	0.001021	0.00177	-1.93879581
SMIM10L	-0.258346671	1.78496655	-3.322521394	0.001022	0.00177	-1.94019295
EMX1	-0.086938639	0.06835357	-3.322343034	0.001023	0.00177	-1.94076084
AFF1	-0.344615053	6.32058083	-3.320565834	0.001029	0.00178	-1.9464178
ZNF443	-0.183748511	2.23334039	-3.320471427	0.001029	0.00178	-1.94671823
ZNF557	-0.183369016	2.37628609	-3.319766639	0.001032	0.00179	-1.9489608
RPGRIP1	-0.180847127	1.66835274	-3.319685464	0.001032	0.00179	-1.94921906
MLPH	0.479356259	4.38878341	3.319222704	0.001034	0.00179	-1.95069125
ZC3H11A	-0.254663243	5.21068301	-3.318774507	0.001035	0.00179	-1.95211693
GSDME	-0.288619502	3.09806778	-3.318266542	0.001037	0.0018	-1.95373251
OMD	0.52350256	1.40863828	3.317321324	0.001041	0.0018	-1.95673818
STX1A	-0.156882538	1.66083262	-3.317161133	0.001041	0.0018	-1.95724748
CTNNA3	-0.054451304	0.10095311	-3.316624864	0.001043	0.00181	-1.95895232
KLF16	0.262835914	3.81730541	3.315728069	0.001046	0.00181	-1.96180273
IKBIP	-0.261246982	4.06014699	-3.3153061	0.001048	0.00181	-1.96314369
EFHD1	-0.400662865	4.10566561	-3.314590039	0.00105	0.00182	-1.96541887
RASA3	-0.284668603	4.66951396	-3.314379155	0.001051	0.00182	-1.96608884

MTMR6	-0.265813024	4.26310935	-3.314378368	0.001051	0.00182	-1.96609134
CDH23	0.114285205	0.51746016	3.314259544	0.001052	0.00182	-1.96646882
RASSF6	-0.399254709	1.03812242	-3.313769375	0.001053	0.00182	-1.96802586
ZNF692	-0.297778509	3.975256	-3.313721887	0.001054	0.00182	-1.9681767
SWT1	-0.190678997	2.40623829	-3.313559894	0.001054	0.00182	-1.96869122
LIX1	-0.673785422	1.89153241	-3.313460268	0.001054	0.00182	-1.96900764
TMEM53	-0.179230132	3.44232942	-3.313428549	0.001055	0.00182	-1.96910838
NRSN1	-0.285205451	0.44811044	-3.31266557	0.001057	0.00183	-1.97153136
RNF126	-0.192731991	4.37261471	-3.31208624	0.001059	0.00183	-1.97337079
ARHGAF	0.225578036	2.78375583	3.311730807	0.001061	0.00183	-1.97449918
MAP3K1	0.355554942	5.49557863	3.311616011	0.001061	0.00183	-1.9748636
ABHD18	-0.190942496	2.67803519	-3.311357848	0.001062	0.00184	-1.97568309
GALR2	0.185845674	0.56582912	3.310409999	0.001066	0.00184	-1.97869136
JAKMIP2	0.067037086	0.14298491	3.310404404	0.001066	0.00184	-1.97870911
GTF2A1	-0.306420661	5.10295939	-3.310287002	0.001066	0.00184	-1.97908167
STARD3	-0.211090577	5.04227279	-3.310178966	0.001066	0.00184	-1.97942449
RASA2	0.265323777	3.23666647	3.309486005	0.001069	0.00185	-1.98162315
SSTR1	-0.13659874	0.41568944	-3.308820803	0.001071	0.00185	-1.98373335
LDOC1	-0.292967513	7.79377025	-3.308741174	0.001072	0.00185	-1.98398593
SARM1	-0.194465299	1.7461469	-3.307965914	0.001074	0.00186	-1.98644471
LRRC9	-0.039467512	0.04826085	-3.30736491	0.001077	0.00186	-1.98835047
BPIFB1	0.222933991	0.28898991	3.306619317	0.001079	0.00186	-1.99071427
CALR3	-0.036859752	0.07028895	-3.306412119	0.00108	0.00187	-1.99137107
PAH	-0.116319697	0.24777682	-3.305576519	0.001083	0.00187	-1.99401949
SEC61A2	-0.197895186	2.489974	-3.305575246	0.001083	0.00187	-1.99402353
FGFR1	-0.3404305	3.49894991	-3.305309723	0.001084	0.00187	-1.99486497
FAM43A	0.481176686	5.05347745	3.30517326	0.001085	0.00187	-1.9952974
EPHA8	-0.050797297	0.05045109	-3.304948402	0.001086	0.00187	-1.99600989
MMP3	0.220344183	0.23199941	3.304558713	0.001087	0.00188	-1.99724458
MCM3AF	-0.235898845	4.43192251	-3.304436453	0.001088	0.00188	-1.99763192
NT5DC3	0.291091059	2.07962028	3.304262033	0.001088	0.00188	-1.99818449
MTG1	-0.207775039	2.37287199	-3.303785241	0.00109	0.00188	-1.99969485
EXOSC8	-0.192387554	3.96486731	-3.30341701	0.001091	0.00188	-2.00086117
FAM83E	0.255481238	0.34994047	3.303338657	0.001092	0.00188	-2.00110933
ZNF577	-0.250124918	2.81489647	-3.302413058	0.001095	0.00189	-2.00404046
CPNE8	-0.245906222	2.66260325	-3.302095377	0.001096	0.00189	-2.0050463
VWA2	0.293790371	1.43610049	3.301992915	0.001097	0.00189	-2.0053707
FAM133A	-0.144122324	0.34888108	-3.300791942	0.001101	0.0019	-2.0091723
VWA8	-0.246628214	3.46638327	-3.300257672	0.001103	0.0019	-2.01086309
ZNF335	-0.214649427	3.63878352	-3.300246055	0.001103	0.0019	-2.01089985

NFKB1	-0.2850998	4.76378383	-3.297850739	0.001112	0.00192	-2.01847712
SLC25A3	-0.279710276	2.93325887	-3.297747904	0.001113	0.00192	-2.01880231
C2orf50	-0.077784166	0.24400893	-3.297468182	0.001114	0.00192	-2.01968682
INPP5K	-0.2268894	5.68181056	-3.297185956	0.001115	0.00192	-2.02057918
PSEN1	-0.202393729	4.81817505	-3.295887102	0.00112	0.00193	-2.02468506
COTL1	-0.269780267	5.32481618	-3.295642616	0.001121	0.00193	-2.02545775
KLF5	0.465436214	5.7814681	3.295144933	0.001123	0.00193	-2.0270305
SLC13A3	-0.356591767	1.65527659	-3.294697984	0.001124	0.00194	-2.02844275
PPP2R2I	-0.193540816	0.67972254	-3.293538259	0.001129	0.00194	-2.03210635
FARP2	-0.186178179	1.7207408	-3.292444132	0.001133	0.00195	-2.03556164
PITPNM1	-0.28121269	4.8249937	-3.292118986	0.001134	0.00195	-2.03658826
LVRN	0.028403234	0.07136892	3.291404415	0.001137	0.00196	-2.03884413
EFNB2	-0.341195778	4.75810205	-3.290940972	0.001139	0.00196	-2.04030696
TAF1B	-0.244452584	3.50650609	-3.290692807	0.00114	0.00196	-2.04109019
IGF2	0.642858316	2.73240475	3.290585438	0.00114	0.00196	-2.04142905
CD300E	0.237909624	0.80640004	3.288926793	0.001147	0.00197	-2.04666238
RELL1	0.494703423	3.14921091	3.288592209	0.001148	0.00198	-2.04771776
ACER3	-0.194497216	2.95307193	-3.287638444	0.001152	0.00198	-2.05072569
NME2	-0.248118257	5.72197312	-3.285555339	0.00116	0.002	-2.05729247
AFAP1	0.334067112	4.1381718	3.285330679	0.001161	0.002	-2.05800046
WAPL	-0.241475012	4.59363588	-3.285079673	0.001162	0.002	-2.05879143
RUFY1	-0.161797195	5.22065988	-3.28492094	0.001162	0.002	-2.0592916
MZT1	-0.218253912	4.41974141	-3.284794073	0.001163	0.002	-2.05969134
BBS2	-0.217179602	4.35759143	-3.283314613	0.001169	0.00201	-2.06435187
GPRC5C	-0.23916192	6.04119874	-3.282231536	0.001173	0.00202	-2.06776252
USP45	-0.177177278	2.09795629	-3.281951468	0.001174	0.00202	-2.06864429
ZNF91	-0.240164042	3.02621287	-3.281058973	0.001178	0.00202	-2.07145379
NFKBIE	0.271708954	4.12271892	3.280954517	0.001178	0.00203	-2.07178256
LEFTY1	-0.0510361	0.11701248	-3.280757354	0.001179	0.00203	-2.0724031
NBPF3	-0.271782763	2.02506245	-3.280561848	0.00118	0.00203	-2.07301839
TTC17	-0.248841488	4.64771006	-3.280524095	0.00118	0.00203	-2.0731372
SMTNL2	-0.203978272	0.42568066	-3.28040887	0.00118	0.00203	-2.07349982
CFD	-0.420440288	4.33066399	-3.279597353	0.001184	0.00203	-2.07605333
DDX6	-0.252223752	5.30938232	-3.279521918	0.001184	0.00203	-2.07629066
RASL10A	-0.162629545	0.69755224	-3.278938005	0.001186	0.00204	-2.0781276
HMOX1	0.439508958	4.87603689	3.278867228	0.001187	0.00204	-2.07835023
P2RY13	0.341740378	1.93803247	3.278285024	0.001189	0.00204	-2.08018146
RMC1	-0.152904565	4.23360624	-3.278215892	0.001189	0.00204	-2.08039888
VPS33A	-0.154721741	3.05190598	-3.277286304	0.001193	0.00205	-2.08332207
ZNF552	-0.175770856	2.23922335	-3.276287082	0.001197	0.00206	-2.08646338

NANOS1	-0.376223352	3.54208642	-3.275679235	0.0012	0.00206	-2.08837387
SPHKAP	-0.186377486	0.14650171	-3.274461005	0.001205	0.00207	-2.09220184
SMPD3	-0.167127112	0.82942137	-3.2737451	0.001207	0.00207	-2.09445077
DEPDC5	-0.200688977	2.64254481	-3.273665557	0.001208	0.00207	-2.09470062
COPA	-0.257039958	6.90943757	-3.273054739	0.00121	0.00208	-2.09661904
STK36	-0.261121906	3.60794863	-3.272781823	0.001211	0.00208	-2.09747609
BTN3A1	0.320402991	3.74821538	3.272556591	0.001212	0.00208	-2.09818334
DGKG	-0.22381729	0.69291152	-3.271608754	0.001216	0.00209	-2.10115918
MED16	-0.193739723	5.51986712	-3.271499753	0.001217	0.00209	-2.10150135
HIST1H4	-0.27019573	1.22965266	-3.270152386	0.001222	0.0021	-2.10573005
BRIP1	0.13202798	0.59949638	3.269852367	0.001223	0.0021	-2.10667144
SCAPER	-0.281047003	3.12300127	-3.269520735	0.001225	0.0021	-2.10771193
ZNF548	-0.194321418	3.07869981	-3.268887891	0.001228	0.0021	-2.10969721
FNDC10	-0.364565112	5.07405555	-3.267281245	0.001234	0.00212	-2.11473577
ATXN7L3	-0.209294736	5.27855736	-3.267262126	0.001234	0.00212	-2.11479572
BCL2L11	-0.289282022	4.01527104	-3.267238202	0.001234	0.00212	-2.11487073
TTLL5	-0.178353555	2.87488016	-3.266736552	0.001236	0.00212	-2.11644346
ORM1	-0.348765535	0.84984057	-3.266723045	0.001237	0.00212	-2.1164858
GPR174	0.310936218	0.69433104	3.266712086	0.001237	0.00212	-2.11652015
TIFAB	0.127123756	0.19746202	3.266696742	0.001237	0.00212	-2.11656825
NOS1	-0.183516857	0.17204207	-3.266340406	0.001238	0.00212	-2.11768526
MITF	-0.262845271	3.32287216	-3.266259944	0.001238	0.00212	-2.11793747
EPHB2	-0.29297722	2.97549343	-3.265970276	0.00124	0.00212	-2.11884538
KLHL41	-0.263239961	0.86029486	-3.265941212	0.00124	0.00212	-2.11893648
RELB	0.280298001	4.22646073	3.265451357	0.001242	0.00213	-2.12047167
LONP2	-0.236027634	5.39710739	-3.26413463	0.001247	0.00214	-2.1245972
CHEK1	-0.165855715	2.07763318	-3.263827245	0.001249	0.00214	-2.12556007
CDK2AP	-0.254160943	5.3401158	-3.262255637	0.001255	0.00215	-2.13048176
PHLDA2	0.528270025	5.37989173	3.260442724	0.001263	0.00216	-2.13615641
FAM186A	-0.045980432	0.18891325	-3.260227181	0.001264	0.00216	-2.1368309
GSDMC	-0.307438535	0.69489856	-3.260027213	0.001265	0.00216	-2.13745661
CD4	0.41784594	4.43609446	3.258880839	0.00127	0.00217	-2.14104299
SPRY2	-0.344075751	4.47470103	-3.257989423	0.001274	0.00218	-2.14383095
SNTB1	-0.296104024	5.70776291	-3.257787349	0.001275	0.00218	-2.14446285
PABPC4L	0.274271606	1.99101883	3.257765445	0.001275	0.00218	-2.14453134
GLG1	-0.240080175	5.86172797	-3.257453511	0.001276	0.00218	-2.14550671
BACH1	0.295963811	3.82255121	3.256937517	0.001278	0.00219	-2.14711994
DTX1	-0.319915153	3.13067576	-3.256263249	0.001281	0.00219	-2.14922766
ARMCX4	-0.250803093	2.39827612	-3.256228407	0.001281	0.00219	-2.14933657
ABHD11	-0.188865227	6.33367653	-3.256057902	0.001282	0.00219	-2.14986948

LMX1A	-0.034583386	0.0472803	-3.255531553	0.001284	0.0022	-2.15151444
ITGA6	-0.292418457	5.21522919	-3.254795701	0.001287	0.0022	-2.15381374
CYTH4	0.366619213	2.07792658	3.253668929	0.001292	0.00221	-2.1573336
ECEL1	-0.446203899	1.46251124	-3.253653999	0.001292	0.00221	-2.15738023
FADS3	0.228154474	2.9908129	3.253563455	0.001293	0.00221	-2.15766303
PPHLN1	-0.167978653	3.49735717	-3.253443789	0.001293	0.00221	-2.15803677
THOC1	-0.217726159	3.16063006	-3.253283858	0.001294	0.00221	-2.15853625
PCDH7	-0.507241905	2.77658038	-3.253131561	0.001295	0.00221	-2.15901186
CRTAM	0.303825537	0.83287654	3.252822877	0.001296	0.00221	-2.1599758
ALPL	-0.399000987	4.04847376	-3.251347409	0.001303	0.00223	-2.16458214
SC5D	-0.251357637	4.44006295	-3.251135626	0.001304	0.00223	-2.16524316
CD93	-0.359891115	5.09049244	-3.250559252	0.001306	0.00223	-2.16704193
ADA	-0.238818578	2.59105165	-3.250364921	0.001307	0.00223	-2.16764835
CYP2R1	-0.193086174	2.47530028	-3.249486951	0.001311	0.00224	-2.17038765
MTDH	-0.285043633	5.78477618	-3.249178945	0.001312	0.00224	-2.17134848
TESPA1	0.273488284	0.89045629	3.248316251	0.001316	0.00225	-2.17403922
AP2B1	-0.239288963	6.85641402	-3.24804716	0.001317	0.00225	-2.17487838
LAP3	-0.306313767	5.85336749	-3.247416214	0.00132	0.00225	-2.17684574
BCOR	-0.305280222	4.12008166	-3.247369072	0.00132	0.00225	-2.17699272
B4GALT5	0.35367798	5.16091298	3.247071556	0.001322	0.00226	-2.17792027
ZXDB	-0.223069857	2.96553136	-3.244599949	0.001333	0.00227	-2.18562285
FNDC1	-0.664823345	5.87132271	-3.244105893	0.001335	0.00228	-2.18716189
PCDHA1	-0.156589884	0.23083976	-3.244065348	0.001335	0.00228	-2.18728818
CAMKK1	-0.280347254	3.58225755	-3.244017608	0.001335	0.00228	-2.18743689
EHBP1	0.305973529	3.98829477	3.242923232	0.00134	0.00229	-2.19084514
MTMR9	-0.191705373	2.70171857	-3.242527083	0.001342	0.00229	-2.19207862
PPP3CB	-0.203292467	5.08924414	-3.241874708	0.001345	0.00229	-2.1941096
NEUROG	-0.14003778	0.1319179	-3.239707642	0.001355	0.00231	-2.20085344
SEMA6B	-0.322451542	3.20507891	-3.239523408	0.001356	0.00231	-2.20142658
TXLNB	0.162079601	0.33418544	3.23902822	0.001358	0.00231	-2.20296692
FMO3	0.237314993	1.0584336	3.237603495	0.001364	0.00233	-2.2073975
ARHGAP	0.320006501	3.26822004	3.237585956	0.001364	0.00233	-2.20745203
IRF2BPL	-0.26504991	5.78857553	-3.236945046	0.001367	0.00233	-2.20944452
EPB41	-0.30261452	3.71206059	-3.236729304	0.001368	0.00233	-2.21011515
FLVCR1	-0.176443058	2.44572611	-3.236156413	0.001371	0.00234	-2.21189576
BCL2L13	-0.233311469	4.59331054	-3.235687217	0.001373	0.00234	-2.21335386
MSH5	0.141841567	0.69854912	3.235097712	0.001376	0.00234	-2.21518557
TBRG1	-0.20336641	3.72718416	-3.233693073	0.001382	0.00235	-2.21954881
SEC14L1	-0.269999644	4.8965869	-3.233677751	0.001382	0.00235	-2.2195964
HOOK1	-0.302084224	4.23445947	-3.233471581	0.001383	0.00236	-2.22023667

PLPP7	-0.165445742	0.99807367	-3.233279808	0.001384	0.00236	-2.22083221
ELMO2	-0.221667916	4.88331041	-3.233054154	0.001385	0.00236	-2.22153291
RNASE1(	0.11182298	0.28172844	3.232991309	0.001386	0.00236	-2.22172805
PATL2	0.189998477	0.79618557	3.232609576	0.001387	0.00236	-2.2229133
TCTE3	-0.250052237	1.95156638	-3.232570856	0.001388	0.00236	-2.22303351
ATF6	-0.265873861	4.75862763	-3.232207438	0.001389	0.00236	-2.22416176
CYP1A2	-0.360529124	1.46137227	-3.231206114	0.001394	0.00237	-2.22726979
C17orf67	-0.144482901	1.23749155	-3.230776737	0.001396	0.00238	-2.22860228
IQSEC2	-0.153400731	3.29339213	-3.230069942	0.001399	0.00238	-2.23079532
MICU1	-0.15599419	5.28106872	-3.229971651	0.0014	0.00238	-2.23110026
ESRP1	-0.279141509	5.56449784	-3.229910075	0.0014	0.00238	-2.23129129
YLPM1	-0.260500964	4.07595347	-3.227900247	0.00141	0.0024	-2.23752467
PUM1	-0.242114588	5.66262365	-3.227009236	0.001414	0.0024	-2.24028696
ATP9B	-0.203845186	2.73897303	-3.225857047	0.001419	0.00241	-2.24385789
SRSF5	-0.278898218	6.91225483	-3.225408598	0.001421	0.00242	-2.24524744
KANSL1	-0.240088211	3.85979496	-3.225058527	0.001423	0.00242	-2.24633203
DRC7	-0.071941507	0.10112563	-3.224699327	0.001425	0.00242	-2.24744479
MCOLN3	0.340271299	1.78677636	3.223908971	0.001429	0.00243	-2.24989282
SULF2	0.373333038	5.20006621	3.223765901	0.001429	0.00243	-2.2503359
KISS1	0.268194828	0.61578009	3.223075184	0.001433	0.00243	-2.25247477
KLHL32	0.252820521	1.11392049	3.222862867	0.001434	0.00244	-2.25313214
OGFRL1	0.275604119	3.31056985	3.222733302	0.001434	0.00244	-2.25353329
FAT1	-0.424887353	3.71242493	-3.222169753	0.001437	0.00244	-2.25527788
MTRNR2	0.114817764	0.29455998	3.222047135	0.001438	0.00244	-2.25565744
CCZ1	-0.176329634	1.79246846	-3.22180237	0.001439	0.00244	-2.25641505
SLC6A1	-0.291471304	1.00894966	-3.219677285	0.001449	0.00246	-2.26299054
EIF3L	-0.41964821	6.94404283	-3.219165419	0.001451	0.00247	-2.26457377
SNX7	-0.27274315	4.49427909	-3.218225692	0.001456	0.00247	-2.26747979
HDAC7	-0.211465637	5.19387057	-3.21741119	0.00146	0.00248	-2.26999793
FRMD6	0.287378308	2.06808595	3.216782313	0.001463	0.00248	-2.27194179
EIF3H	-0.202143284	6.6319938	-3.216143941	0.001466	0.00249	-2.27391463
CEP44	-0.198243676	2.77683671	-3.215913642	0.001467	0.00249	-2.27462627
DVL1	0.236436847	6.51512118	3.215637205	0.001469	0.00249	-2.27548041
TRAIP	-0.183691249	1.80398154	-3.214891456	0.001472	0.0025	-2.2777843
BMX	-0.314049486	1.33479159	-3.214714161	0.001473	0.0025	-2.27833196
TMEM26	-0.19510055	3.3153133	-3.213185158	0.001481	0.00251	-2.28305384
LUZP1	-0.264189436	3.88120835	-3.212140159	0.001486	0.00252	-2.28627982
ANKRD3	0.150669281	0.77282049	3.21166513	0.001488	0.00253	-2.28774594
TRIM44	-0.237007827	4.4651146	-3.211451045	0.001489	0.00253	-2.28840663
PURA	-0.256473969	3.87765465	-3.211288291	0.00149	0.00253	-2.28890888

PLEKHG	0.24881021	3.07068595	3.209610868	0.001499	0.00254	-2.2940839
NAA15	-0.210018583	3.40011163	-3.208690537	0.001503	0.00255	-2.29692216
KRTCAP	-0.349693165	4.99421451	-3.208577059	0.001504	0.00255	-2.29727207
PRKAA1	-0.255091855	4.84632508	-3.208347708	0.001505	0.00255	-2.29797924
ABCC1	-0.296073154	4.75208559	-3.208016915	0.001507	0.00255	-2.2989991
SOWAHI	0.407515216	3.26371115	3.207821223	0.001508	0.00256	-2.29960239
TCP11L1	0.196152522	1.88289282	3.207748639	0.001508	0.00256	-2.29982615
ZNF365	0.155620816	0.60651876	3.2069677	0.001512	0.00256	-2.30223329
C1S	0.708199696	4.04301122	3.205488835	0.001519	0.00258	-2.30679021
STK10	-0.231244042	3.21933886	-3.205390969	0.00152	0.00258	-2.3070917
BRAF	-0.213722725	3.75209277	-3.205047575	0.001522	0.00258	-2.30814952
RNF122	-0.284361619	2.99386784	-3.204605411	0.001524	0.00258	-2.30951143
ERC1	-0.281454712	3.90068861	-3.203674294	0.001529	0.00259	-2.31237882
CSNK1G	-0.21159982	3.81069137	-3.203475424	0.00153	0.00259	-2.31299114
ZNF765	-0.143274125	1.44496013	-3.202305365	0.001536	0.0026	-2.31659305
CD58	0.240347948	4.42928323	3.202212995	0.001536	0.0026	-2.31687735
XCR1	0.225735713	0.38865168	3.201979293	0.001537	0.0026	-2.31759662
ZRANB2	-0.275049423	5.59259623	-3.200991485	0.001542	0.00261	-2.32063626
LDLRAD	-0.377513442	2.47569339	-3.200759452	0.001544	0.00261	-2.32135013
NREP	-0.294880466	3.01749987	-3.200744925	0.001544	0.00261	-2.32139483
TMEM13	-0.267968772	1.12532821	-3.200624703	0.001544	0.00261	-2.32176468
GFPT2	0.413531694	1.92174037	3.199944634	0.001548	0.00262	-2.32385664
GAB2	0.319492188	3.71975506	3.198891656	0.001553	0.00263	-2.32709489
DDHD2	-0.214707111	3.48424192	-3.19879017	0.001554	0.00263	-2.32740694
C1QL1	0.637601522	2.40750596	3.197846438	0.001559	0.00264	-2.3303083
RCN1	-0.212805995	4.94814869	-3.196545474	0.001565	0.00265	-2.33430662
AARS2	-0.179846065	3.76510553	-3.195640702	0.00157	0.00266	-2.33708641
ZNF778	-0.201689508	2.40697247	-3.195545826	0.001571	0.00266	-2.33737787
TOPORS	-0.240951379	4.48537134	-3.195425285	0.001571	0.00266	-2.33774815
DYRK1B	-0.202661048	5.02109265	-3.195053714	0.001573	0.00266	-2.33888947
B3GNT5	0.248663289	1.94610039	3.195045129	0.001573	0.00266	-2.33891584
WDR19	-0.244444632	3.80378067	-3.194930636	0.001574	0.00266	-2.33926749
CHD1L	-0.234261567	4.49905477	-3.194659811	0.001575	0.00266	-2.34009926
RNF166	-0.177519387	3.3482964	-3.194365769	0.001577	0.00267	-2.34100225
ARRB1	-0.205681074	2.68237864	-3.194300368	0.001577	0.00267	-2.34120308
MCC	-0.301182183	2.52487408	-3.193866151	0.001579	0.00267	-2.34253639
NSD3	-0.219152777	3.886429	-3.19356746	0.001581	0.00267	-2.34345345
EMSY	-0.204409832	2.32983525	-3.193333434	0.001582	0.00267	-2.34417191
CNTN1	0.201705686	0.35169632	3.193234553	0.001583	0.00267	-2.34447547
TMEM21	-0.820903359	2.54922193	-3.192930902	0.001584	0.00268	-2.34540758

WDR35	-0.245120076	3.31691363	-3.192158311	0.001588	0.00268	-2.34777884
C12orf29	-0.174030462	2.96552547	-3.191727323	0.001591	0.00269	-2.34910141
SOCS1	-0.403685013	4.40328575	-3.191468181	0.001592	0.00269	-2.34989656
IARS	-0.334758203	5.29378743	-3.191286761	0.001593	0.00269	-2.35045319
LEMD3	-0.23655572	4.01737111	-3.191250286	0.001593	0.00269	-2.3505651
PTP4A3	0.301190991	5.6679525	3.190668881	0.001596	0.0027	-2.35234874
GFAP	-0.279107183	0.69477131	-3.190433684	0.001598	0.0027	-2.3530702
NEURL2	-0.181780869	1.68204918	-3.189822077	0.001601	0.0027	-2.35494604
COL6A5	0.091833448	0.07897661	3.188593497	0.001607	0.00271	-2.35871319
KPNA6	-0.222979767	5.27941089	-3.188222026	0.001609	0.00272	-2.35985196
TNFRSF1	-0.369221466	2.02483964	-3.187191969	0.001615	0.00273	-2.36300901
GNL2	-0.162362992	4.70381251	-3.186701107	0.001618	0.00273	-2.36451314
NEGR1	-0.255097507	0.71611375	-3.18577651	0.001623	0.00274	-2.36734576
CREB3L3	0.02907285	0.04568167	3.185588982	0.001624	0.00274	-2.36792018
ZNF573	-0.171906357	1.16277522	-3.18555726	0.001624	0.00274	-2.36801734
DHX36	-0.245532959	3.90556799	-3.185494809	0.001624	0.00274	-2.36820863
PDGFA	0.225553141	3.43531679	3.184975495	0.001627	0.00274	-2.36979915
KBTBD4	-0.306524595	3.22980136	-3.183717181	0.001634	0.00276	-2.37365204
POLR2J3	0.256989675	1.38797939	3.182969055	0.001638	0.00276	-2.3759421
TACC3	-0.282818527	2.50139568	-3.182661587	0.001639	0.00276	-2.37688313
CHP1	-0.237196956	6.65107259	-3.18058247	0.001651	0.00278	-2.38324424
SLAIN1	-0.185872368	3.00420036	-3.180452841	0.001651	0.00278	-2.38364072
ZNF66	-0.139222456	1.04658481	-3.179323834	0.001658	0.00279	-2.38709321
UCN2	0.100830593	0.12016667	3.178249829	0.001664	0.0028	-2.39037645
LRRC52	0.270604828	0.86096247	3.177322977	0.001669	0.00281	-2.39320902
UBAP1	-0.214877998	5.96348758	-3.176233272	0.001675	0.00282	-2.39653831
ZNF407	-0.214080002	2.30229103	-3.175606794	0.001678	0.00283	-2.39845187
FRMD4A	-0.207384897	1.74930774	-3.175286598	0.00168	0.00283	-2.39942976
STAT2	0.234493782	5.42001101	3.175215551	0.00168	0.00283	-2.39964673
ABCC3	0.59354161	4.29974218	3.174436194	0.001685	0.00284	-2.40202648
KCNMB4	-0.31576113	2.26778253	-3.174093259	0.001687	0.00284	-2.40307346
FBP1	-0.36007702	6.15067343	-3.174058172	0.001687	0.00284	-2.40318058
NELL1	-0.037022896	0.03622687	-3.173613012	0.001689	0.00284	-2.40453947
CLASRP	-0.213137291	4.41793416	-3.173544187	0.00169	0.00285	-2.40474955
VWA5A	0.276604051	4.08321588	3.173282301	0.001691	0.00285	-2.40554889
PSMD5	-0.248857022	4.54871795	-3.173082724	0.001692	0.00285	-2.40615801
CNMD	-0.781980218	1.73798775	-3.172772067	0.001694	0.00285	-2.40710607
NBPF1	-0.234328011	2.12771385	-3.172327832	0.001697	0.00286	-2.40846164
FBXL14	-0.311851812	4.52405155	-3.171798339	0.0017	0.00286	-2.41007714
ZNF80	0.080141845	0.13934561	3.171720949	0.0017	0.00286	-2.41031324

KALRN	-0.243013071	1.88208311	-3.171648173	0.0017	0.00286	-2.41053526
CBWD3	-0.054704613	0.23399075	-3.170961336	0.001704	0.00287	-2.41263035
ZCCHC8	-0.201207959	3.52434706	-3.170031375	0.00171	0.00288	-2.4154664
PLA2G4/	0.321261449	1.90591603	3.169101502	0.001715	0.00288	-2.41830141
HIST2H2	-0.171823247	0.67707517	-3.168852139	0.001716	0.00289	-2.41906154
TENT5A	0.377280791	3.95963207	3.168294684	0.001719	0.00289	-2.42076063
ZNF585A	-0.191023047	2.06480136	-3.168152579	0.00172	0.00289	-2.42119371
TRIQK	-0.228358501	4.030917	-3.168144506	0.00172	0.00289	-2.42121831
XPR1	0.368693035	5.22840514	3.167805456	0.001722	0.0029	-2.42225153
PLCB4	-0.507900632	4.3129948	-3.167765614	0.001722	0.0029	-2.42237294
ULK1	-0.196083992	4.49360169	-3.166971755	0.001727	0.0029	-2.42479172
PGLYRP	-0.059254119	0.10855914	-3.166361922	0.00173	0.00291	-2.42664941
C21orf91	0.231343375	3.01729035	3.166339311	0.001731	0.00291	-2.42671828
SUSD4	0.417897051	2.41117847	3.166327931	0.001731	0.00291	-2.42675295
LYVE1	-0.342996711	1.61576171	-3.166324858	0.001731	0.00291	-2.42676231
ABCD1	-0.197378027	3.43705208	-3.166286465	0.001731	0.00291	-2.42687925
CNTNAP	0.288050356	2.02660203	3.166081601	0.001732	0.00291	-2.42750322
RBM6	-0.234161867	5.00858916	-3.165696326	0.001734	0.00291	-2.4286766
EAF2	-0.278088143	1.96618197	-3.165329764	0.001736	0.00292	-2.42979286
KLRB1	0.403857967	1.9550479	3.1643096	0.001742	0.00293	-2.43289884
MFSD8	-0.205769979	3.38878973	-3.162851005	0.001751	0.00294	-2.43733806
SEMA3B	0.456288401	3.62140227	3.162618391	0.001752	0.00294	-2.43804585
POLR2D	-0.184067328	4.01550726	-3.162540641	0.001752	0.00294	-2.43828241
NEFL	-0.32901168	0.41256986	-3.16208201	0.001755	0.00295	-2.43967773
CLECL1	0.234194576	0.74280693	3.161657752	0.001758	0.00295	-2.44096831
BTBD7	-0.252044661	3.78553946	-3.161531773	0.001758	0.00295	-2.4413515
HRNR	0.089674161	0.23669142	3.161487709	0.001759	0.00295	-2.44148553
TPSG1	-0.100644242	0.23968019	-3.161151745	0.00176	0.00295	-2.44250736
NRG3	-0.131749354	0.38233993	-3.161132555	0.001761	0.00295	-2.44256572
RCE1	-0.196048748	3.62742993	-3.160816133	0.001762	0.00296	-2.44352802
SUZ12	-0.219369038	4.30238752	-3.159395179	0.001771	0.00297	-2.44784828
ZNF554	-0.165016307	2.59112649	-3.159043391	0.001773	0.00297	-2.44891758
CCHCR1	-0.260418219	4.15004659	-3.158562161	0.001776	0.00298	-2.45038016
VKORC1	-0.238817228	4.58673061	-3.15819342	0.001778	0.00298	-2.45150071
EMILIN2	0.440652799	4.33174502	3.15809132	0.001778	0.00298	-2.45181096
MYL3	-0.278820685	0.56078833	-3.158061119	0.001779	0.00298	-2.45190272
TMEM13	-0.340282395	2.54386488	-3.157826203	0.00178	0.00298	-2.45261651
POLE	0.159745769	2.08345037	3.15619307	0.00179	0.003	-2.45757739
KRT6A	0.476338893	0.80718161	3.155953321	0.001791	0.003	-2.45830546
ESR2	-0.066709794	0.31662947	-3.155903288	0.001791	0.003	-2.4584574

DUSP8	0.35585067	3.01491761	3.155703505	0.001792	0.003	-2.45906405
PASK	-0.646942309	3.26456855	-3.155083097	0.001796	0.00301	-2.46094774
RNF146	-0.301386826	5.79380126	-3.155019004	0.001796	0.00301	-2.46114232
MS4A7	0.334377075	2.99676048	3.154742625	0.001798	0.00301	-2.46198134
KYAT3	-0.222888661	5.50141201	-3.154039837	0.001802	0.00302	-2.46411452
PIGM	-0.208532573	3.90138769	-3.15334421	0.001806	0.00303	-2.46622554
RCOR3	-0.236993519	4.47329403	-3.153074948	0.001808	0.00303	-2.46704255
ZNF526	-0.171737558	3.21252457	-3.152594091	0.001811	0.00303	-2.46850144
ZNF713	-0.184713501	1.43379945	-3.151690989	0.001816	0.00304	-2.47124083
DNM2	-0.173463757	4.89195856	-3.151637036	0.001817	0.00304	-2.47140446
IFT81	-0.232854267	3.36976802	-3.151364668	0.001818	0.00304	-2.47223048
FBXL13	-0.13534135	0.88439635	-3.151044974	0.00182	0.00305	-2.47319995
TRIP11	-0.276732527	4.08701129	-3.150690688	0.001822	0.00305	-2.47427421
AJAP1	0.304370771	0.96688568	3.149519986	0.001829	0.00306	-2.47782319
PNPLA1	-0.056122409	0.10494951	-3.149300073	0.001831	0.00306	-2.47848972
ICE1	-0.248666706	3.83458732	-3.148665168	0.001834	0.00307	-2.4804138
ZNF619	-0.208633335	2.20906052	-3.148543974	0.001835	0.00307	-2.48078104
DUSP11	-0.18704338	4.20965904	-3.148516263	0.001835	0.00307	-2.480865
CCSER2	-0.213734774	4.40920839	-3.14831647	0.001837	0.00307	-2.48147038
LIMD2	0.409093124	2.70688794	3.14810787	0.001838	0.00307	-2.4821024
C15orf39	-0.231171282	4.73870416	-3.147995572	0.001839	0.00308	-2.48244262
F5	0.600097962	1.50881707	3.147906349	0.001839	0.00308	-2.48271293
KCNMB2	-0.116867034	0.17751974	-3.147435207	0.001842	0.00308	-2.48414018
JPH4	0.089422178	0.38627721	3.147314196	0.001843	0.00308	-2.48450673
FAS	0.371547304	4.47266681	3.146979147	0.001845	0.00308	-2.48552155
DNM1	0.234244181	1.00890036	3.146263979	0.001849	0.00309	-2.48768736
FAM160A	-0.218026065	4.79891685	-3.146092133	0.00185	0.00309	-2.48820771
RASGEF	0.359146332	1.45025519	3.145540958	0.001853	0.0031	-2.48987649
FAM156A	-0.032647106	0.12043495	-3.145398267	0.001854	0.0031	-2.49030847
ERICH3	0.161707849	0.18538213	3.145215735	0.001855	0.0031	-2.49086104
MLLT11	-0.303687801	2.8169776	-3.144895701	0.001857	0.0031	-2.49182978
ZNF774	-0.175772563	1.69396766	-3.144503065	0.00186	0.00311	-2.49301817
OPTN	0.225444104	5.17985303	3.144236995	0.001861	0.00311	-2.4938234
ARHGEF	-0.260783207	5.12450512	-3.143697572	0.001865	0.00312	-2.49545571
PILRA	0.304232175	2.8190584	3.143506155	0.001866	0.00312	-2.49603488
MSANTD	-0.262260747	3.62307847	-3.143042866	0.001869	0.00312	-2.49743653
TWISTNI	-0.185583057	3.90952083	-3.14296972	0.001869	0.00312	-2.49765781
DPH1	-0.229517917	3.57105529	-3.142738893	0.001871	0.00312	-2.49835607
ZNF175	-0.220382228	2.68165831	-3.142008706	0.001875	0.00313	-2.50056461
TMEM23	-0.233719019	4.09922021	-3.141933661	0.001876	0.00313	-2.50079156

ALDH3B2	0.182576729	0.380062	3.141419593	0.001879	0.00314	-2.5023461
WIZ	-0.177427772	4.09691489	-3.139915734	0.001888	0.00315	-2.50689241
ALKAL2	0.428843702	1.54157069	3.138895522	0.001894	0.00316	-2.50997546
ACMSD	-0.09871741	0.31669329	-3.138437587	0.001897	0.00317	-2.51135902
CWF19L1	-0.214827753	3.95496068	-3.138339135	0.001898	0.00317	-2.51165645
SYTL1	0.400015292	4.92951158	3.138220735	0.001899	0.00317	-2.51201414
ILF3	-0.205542315	6.14223375	-3.137777749	0.001901	0.00317	-2.51335227
ATAT1	-0.240587197	3.0638237	-3.135480369	0.001916	0.0032	-2.5202892
NEK3	-0.181723896	2.50840992	-3.134518131	0.001922	0.00321	-2.52319328
CLIC4	-0.303897993	5.80346941	-3.134076403	0.001925	0.00321	-2.52452616
HAUS3	-0.166382513	2.59419853	-3.133625163	0.001927	0.00321	-2.52588756
PRDM10	-0.2093119	2.42710403	-3.133150372	0.00193	0.00322	-2.52731982
CPEB4	-0.312416063	4.07549295	-3.132958152	0.001932	0.00322	-2.52789961
HNMT	0.34792884	2.78636248	3.132856927	0.001932	0.00322	-2.52820493
GOLGA6	-0.076620271	0.09942979	-3.131903246	0.001938	0.00323	-2.53108095
DUSP7	-0.225205611	3.47872376	-3.131544587	0.001941	0.00323	-2.53216235
BBC3	0.285205024	3.84990439	3.130903251	0.001945	0.00324	-2.53409577
AGO3	-0.192696615	2.21340125	-3.130872932	0.001945	0.00324	-2.53418717
CAST	0.26633138	5.98718809	3.130629009	0.001946	0.00324	-2.53492242
IL1RAPL	0.092790548	0.12496905	3.13002185	0.00195	0.00325	-2.53675232
HOXD9	0.394114025	1.58298174	3.129318668	0.001955	0.00326	-2.53887121
UBD	0.594000562	1.38225572	3.127639054	0.001966	0.00327	-2.5439306
EIF3D	-0.225677841	7.2796784	-3.127600503	0.001966	0.00327	-2.54404669
SYNGR4	-0.12367038	0.36405932	-3.127307162	0.001968	0.00328	-2.54493004
MPZL1	-0.248742062	4.65411474	-3.127170851	0.001969	0.00328	-2.54534049
INMT-MII	0.100779683	0.12047932	3.126832961	0.001971	0.00328	-2.54635785
FHL3	0.294729732	4.19005493	3.126672706	0.001972	0.00328	-2.54684032
NUTM2A	-0.072133529	0.30206973	-3.126487501	0.001973	0.00329	-2.54739789
APOB	0.040110865	0.04473493	3.126443745	0.001973	0.00329	-2.54752961
NOP53	-0.239370085	7.75360093	-3.126402348	0.001974	0.00329	-2.54765423
RPTOR	-0.180689156	3.65557566	-3.123996895	0.001989	0.00331	-2.55489298
CORO2A	0.399228061	3.46537678	3.123821392	0.00199	0.00331	-2.55542092
ZNF804A	-0.153979991	0.31919527	-3.123225439	0.001994	0.00332	-2.55721344
POLR3A	-0.174653011	3.17133638	-3.123103651	0.001995	0.00332	-2.55757971
COPG2	-0.234236701	5.28670833	-3.122613779	0.001998	0.00332	-2.55905287
C18orf25	-0.214165893	3.60299888	-3.122388239	0.002	0.00333	-2.55973104
JMJD7	-0.079057604	0.30077495	-3.122270838	0.002	0.00333	-2.56008404
ZNF831	0.175059723	0.35631383	3.122184145	0.002001	0.00333	-2.56034469
RBCK1	-0.164155318	5.99572265	-3.121907445	0.002003	0.00333	-2.56117659
USP53	0.230368715	2.42606638	3.12179388	0.002004	0.00333	-2.561518

USP38	-0.224971805	3.88869949	-3.121685551	0.002004	0.00333	-2.56184366
PLA2G2I	0.467334523	0.95885814	3.12133967	0.002007	0.00334	-2.56288339
FYN	0.290302484	5.84067612	3.120579439	0.002012	0.00334	-2.56516828
B3GNT7	0.508864772	3.7196768	3.12056831	0.002012	0.00334	-2.56520173
ZNF829	-0.153921309	1.54951696	-3.12034325	0.002013	0.00335	-2.56587805
HDAC6	-0.190273182	4.08442415	-3.120209185	0.002014	0.00335	-2.5662809
RNF44	-0.217171823	4.89026302	-3.120035997	0.002015	0.00335	-2.56680129
ALS2	-0.197269028	2.80593755	-3.11928576	0.00202	0.00336	-2.56905528
RPL32	-0.211392226	9.1342726	-3.118925432	0.002022	0.00336	-2.57013766
NID2	-0.508686882	5.12573991	-3.118453481	0.002026	0.00337	-2.57155516
TBC1D3	-0.192548468	1.65353854	-3.118244936	0.002027	0.00337	-2.57218147
CTPS1	-0.227049696	3.01104148	-3.118179343	0.002027	0.00337	-2.57237845
LRPPRC	-0.232709219	5.01254864	-3.117424243	0.002032	0.00338	-2.57464579
TRPV4	-0.229736713	1.54554496	-3.117379896	0.002033	0.00338	-2.57477894
ADD1	-0.216666931	5.83216718	-3.117230829	0.002034	0.00338	-2.57522647
RPL18	-0.231735513	9.0596062	-3.116905558	0.002036	0.00338	-2.57620296
PARP15	0.293227173	0.94829459	3.116534029	0.002038	0.00338	-2.57731819
VOPP1	-0.199830961	4.07541974	-3.115981388	0.002042	0.00339	-2.57897685
STK26	0.314409882	2.87214557	3.114541324	0.002052	0.00341	-2.58329767
BTG2	-0.384477327	8.03794946	-3.113721193	0.002057	0.00341	-2.5857576
SLC6A1	-0.035008458	0.03191414	-3.113386794	0.002059	0.00342	-2.58676043
ZNF260	-0.216953413	3.70949261	-3.113257512	0.00206	0.00342	-2.58714811
C1QTNF	-0.238457971	1.23951713	-3.113099339	0.002061	0.00342	-2.58762241
DNAJC1	-0.215472696	2.90048391	-3.11265392	0.002064	0.00342	-2.58895791
FGFBP1	0.735305678	2.16440685	3.112604365	0.002065	0.00343	-2.58910648
LMAN1	-0.29170707	6.46756234	-3.112551169	0.002065	0.00343	-2.58926597
OR2A42	-0.057027652	0.11009926	-3.112224895	0.002067	0.00343	-2.5902441
SMC1B	-0.068748112	0.27771223	-3.111992168	0.002069	0.00343	-2.59094173
ALG1L	-0.375731345	2.23100578	-3.111401314	0.002073	0.00344	-2.59271267
UGDH	-0.282815222	4.76473873	-3.11082697	0.002077	0.00344	-2.59443383
TMEM26	-0.237020933	0.70636227	-3.11063807	0.002078	0.00345	-2.59499985
PLAC1	-0.081936241	0.11831974	-3.110185542	0.002081	0.00345	-2.59635567
ZNF816	-0.198492148	2.75583266	-3.109748965	0.002084	0.00345	-2.59766353
ASIC3	-0.197912168	0.83340151	-3.109459707	0.002086	0.00346	-2.59852997
ZNF566	-0.18748964	2.35940003	-3.10912179	0.002088	0.00346	-2.59954207
FPGT-TN	-0.100676897	0.34219321	-3.107929949	0.002096	0.00347	-2.60311095
CYP4F2	0.077009772	0.20696697	3.107694044	0.002098	0.00348	-2.6038172
CACNA1	-0.136001186	0.26414152	-3.107397477	0.0021	0.00348	-2.60470499
TLR10	0.259933339	0.68484527	3.106230944	0.002108	0.00349	-2.6081963
SLC43A2	-0.22025335	3.70181228	-3.106130969	0.002109	0.00349	-2.60849546

ZNF451	-0.176781758	2.95014681	-3.105248957	0.002115	0.0035	-2.61113435
FYB2	-0.275688727	0.62477463	-3.104643359	0.002119	0.00351	-2.61294583
KCNK10	-0.089601571	0.14278808	-3.103783611	0.002125	0.00352	-2.61551697
MMP14	0.388829512	6.38321803	3.103663597	0.002126	0.00352	-2.61587583
TRIM73	-0.089545547	0.27495026	-3.103629184	0.002126	0.00352	-2.61597873
SYNC	-0.291393909	1.98769076	-3.103620699	0.002126	0.00352	-2.6160041
PIEZO1	-0.223495676	4.55225554	-3.10330091	0.002128	0.00352	-2.61696024
PGAM4	-0.057105467	0.19471641	-3.103155357	0.002129	0.00352	-2.61739539
SLK	-0.250127088	4.97313091	-3.102672478	0.002133	0.00353	-2.61883892
UBR1	-0.261063293	3.46229971	-3.101961682	0.002138	0.00354	-2.6209634
RABL2B	-0.218927386	3.30605979	-3.101916783	0.002138	0.00354	-2.62109759
GPR37	0.246694607	0.55718363	3.101843456	0.002138	0.00354	-2.62131672
MAP3K1!	-0.058481843	0.17386902	-3.101181173	0.002143	0.00355	-2.62329573
ZNF732	0.113132366	0.66670325	3.101022483	0.002144	0.00355	-2.62376987
N4BP2	-0.266167052	2.43954403	-3.100575641	0.002147	0.00355	-2.62510482
NT5C1B	-0.050564111	0.2013297	-3.100004318	0.002151	0.00356	-2.6268114
NELL2	0.874235091	5.05178713	3.096773321	0.002174	0.0036	-2.63645713
GRIK3	0.278240579	0.72731076	3.095779912	0.002181	0.00361	-2.63942095
QPCT	0.676153268	5.62557548	3.095762957	0.002181	0.00361	-2.63947152
MOXD1	0.411796336	1.68609352	3.095296761	0.002184	0.00361	-2.6408621
NCF1	0.305879016	1.16417628	3.094812306	0.002188	0.00362	-2.64230694
TTC16	-0.077659615	0.27097212	-3.093668132	0.002196	0.00363	-2.64571848
KLC2	-0.177909434	3.97974499	-3.093526153	0.002197	0.00363	-2.64614173
FAM131E	0.098577972	0.39666643	3.092530128	0.002204	0.00364	-2.64911046
ZNF347	-0.189963598	2.46602701	-3.092459399	0.002205	0.00364	-2.64932124
POU5F1I	-0.141895817	0.69223781	-3.091973756	0.002208	0.00365	-2.65076839
NBPF12	-0.22873248	2.29467066	-3.089914817	0.002223	0.00367	-2.65690138
ZNF234	-0.188276798	2.38061096	-3.089907829	0.002223	0.00367	-2.65692219
IFI16	0.343846199	4.82265315	3.089706037	0.002224	0.00367	-2.65752307
CHST11	-0.335517536	3.63243539	-3.089481325	0.002226	0.00368	-2.65819215
POU6F1	-0.174476811	2.96842008	-3.089424464	0.002226	0.00368	-2.65836145
DIAPH3	0.113221634	0.46464814	3.08930496	0.002227	0.00368	-2.65871725
OSGIN2	0.253163257	4.4226877	3.088776316	0.002231	0.00368	-2.66029103
ESYT2	-0.262258148	5.69617502	-3.088168425	0.002235	0.00369	-2.66210042
SLC35F1	-0.307162842	0.8631832	-3.08811106	0.002236	0.00369	-2.66227115
ZNF229	-0.249462118	2.81540286	-3.087771392	0.002238	0.00369	-2.66328201
GNG2	-0.250836274	2.38581493	-3.087537253	0.00224	0.0037	-2.66397876
TBC1D9	0.317379936	3.96344654	3.086348303	0.002249	0.00371	-2.66751605
SPTLC2	-0.234439544	4.58802192	-3.085272576	0.002257	0.00372	-2.67071539
OIT3	-0.374255534	1.7380868	-3.085271612	0.002257	0.00372	-2.67071826

ADH1C	-0.121243167	0.27200649	-3.084482802	0.002262	0.00373	-2.67306362
ACD	-0.203361548	4.07959417	-3.084338711	0.002263	0.00373	-2.67349199
FLRT3	0.563402371	3.91284242	3.083720878	0.002268	0.00374	-2.67532852
SCAF8	-0.247398932	4.59274581	-3.083337935	0.002271	0.00375	-2.67646666
SLC38A4	0.21324845	0.46997197	3.083229369	0.002272	0.00375	-2.6767893
TLNRD1	-0.195530567	3.59764095	-3.082949979	0.002274	0.00375	-2.67761956
PDE9A	0.313957867	4.08936207	3.081941511	0.002281	0.00376	-2.68061584
ITLN2	-0.079626725	0.10357815	-3.081435939	0.002285	0.00377	-2.68211761
UBN2	-0.212016908	2.68410419	-3.081221096	0.002286	0.00377	-2.68275572
RALBP1	-0.257140137	5.35926415	-3.081068467	0.002287	0.00377	-2.68320902
TTL	0.185871938	2.71627533	3.080914097	0.002289	0.00377	-2.68366747
SCRT1	-0.032563718	0.07911438	-3.080749745	0.00229	0.00377	-2.68415554
FCMR	0.399113734	2.4963365	3.080027784	0.002295	0.00378	-2.68629924
KIAA010	-0.236613779	5.65799391	-3.079981752	0.002296	0.00378	-2.6864359
PRX	-0.276911376	3.4518094	-3.07971217	0.002298	0.00379	-2.68723624
HIST1H2	-0.119256128	0.2179536	-3.079663326	0.002298	0.00379	-2.68738124
KPNA3	-0.237297907	4.83636336	-3.077844679	0.002311	0.00381	-2.69277864
CHMP4C	-0.264406979	5.29743849	-3.077551997	0.002314	0.00381	-2.69364699
FGF9	0.170622024	0.43091481	3.077198642	0.002316	0.00382	-2.69469525
DEFB124	-0.104516066	0.21983873	-3.076115333	0.002324	0.00383	-2.69790827
ADRA2A	0.434766035	1.7460859	3.075486962	0.002329	0.00384	-2.6997715
ARID5B	0.390799223	3.13789399	3.07544584	0.002329	0.00384	-2.69989342
SLC25A2	-0.125519053	0.85155415	-3.075393736	0.00233	0.00384	-2.7000479
LINC006	-0.086698038	0.18779542	-3.075188603	0.002331	0.00384	-2.70065606
UBE4B	-0.214637938	4.57548305	-3.0750692	0.002332	0.00384	-2.70101004
SLC30A1	-0.311398182	4.94250824	-3.07473731	0.002335	0.00384	-2.70199388
TXNIP	-0.355381178	8.97678207	-3.074471496	0.002337	0.00385	-2.70278178
FBXW8	-0.268615708	3.69703168	-3.074203584	0.002339	0.00385	-2.70357584
INHBE	-0.217011497	0.29142927	-3.074032351	0.00234	0.00385	-2.70408331
RAPGEF	-0.23587041	5.18354563	-3.072690209	0.00235	0.00387	-2.70806006
SPRN	-0.217387696	2.27026239	-3.072673982	0.00235	0.00387	-2.70810813
RNF2	-0.192451389	3.98569295	-3.071911142	0.002356	0.00388	-2.71036768
TIMM23E	-0.217822852	2.57208401	-3.071683613	0.002358	0.00388	-2.71104153
GBP7	-0.049796651	0.1488296	-3.071622297	0.002358	0.00388	-2.71122312
ENTHD1	0.05672792	0.08022872	3.071611026	0.002359	0.00388	-2.71125649
FCGR1A	0.318615206	1.48420865	3.071290654	0.002361	0.00388	-2.7122052
MON2	-0.229083901	3.3306734	-3.070846486	0.002364	0.00389	-2.71352035
CYP3A5	-0.059182074	0.34564227	-3.070713544	0.002365	0.00389	-2.71391395
UBR4	-0.280001595	4.21417885	-3.070635765	0.002366	0.00389	-2.71414422
SMPDL3	-0.269660732	5.5978605	-3.06951816	0.002375	0.0039	-2.71745239

WASHC5	-0.265354249	4.84877954	-3.06948683	0.002375	0.0039	-2.71754511
RNF133	-0.055894165	0.15742941	-3.069266012	0.002376	0.00391	-2.7181986
SRRM5	-0.111146811	0.78490052	-3.069179891	0.002377	0.00391	-2.71845346
GABBR2	0.60324804	4.24659086	3.069135497	0.002377	0.00391	-2.71858483
OAS3	0.369046436	3.27040294	3.069072401	0.002378	0.00391	-2.71877154
VWA3A	-0.162148947	0.21692577	-3.06900201	0.002378	0.00391	-2.71897984
SMN2	-0.300292166	1.86508652	-3.068961296	0.002379	0.00391	-2.71910031
IFIT3	0.379267234	4.774552	3.068786241	0.00238	0.00391	-2.71961829
LRFN5	-0.139588022	0.21841367	-3.067994814	0.002386	0.00392	-2.72195976
CTSA	0.190953105	6.09315834	3.067894025	0.002387	0.00392	-2.7222579
NAA50	-0.231121854	5.36423257	-3.067609909	0.002389	0.00392	-2.72309831
OXSR1	-0.220176648	4.56741878	-3.067093628	0.002393	0.00393	-2.72462526
WASF1	0.320585789	2.44985829	3.066319516	0.002399	0.00394	-2.72691433
CDKL5	-0.211429903	1.9639218	-3.063758653	0.002419	0.00397	-2.73448305
ANKRD4	-0.204287575	0.59059004	-3.062964993	0.002425	0.00398	-2.73682755
HTRA4	0.230637067	0.70528352	3.062513978	0.002429	0.00399	-2.73815961
ZNF286A	-0.149492162	1.55608058	-3.062128297	0.002432	0.00399	-2.73929856
RTN4RL2	-0.503555827	4.45790374	-3.061962648	0.002433	0.00399	-2.7397877
AIM2	0.329688497	0.7696886	3.061934595	0.002433	0.00399	-2.73987053
PABPC1	0.231159926	8.73631401	3.06182945	0.002434	0.00399	-2.74018099
CLPSL2	-0.291467246	0.8655276	-3.061787159	0.002434	0.00399	-2.74030586
EPS15	-0.190974435	5.09780012	-3.061642825	0.002436	0.00399	-2.74073202
MTCL1	-0.094293337	0.32929205	-3.061106695	0.00244	0.004	-2.74231481
PMCH	0.150354285	0.23011374	3.060214417	0.002447	0.00401	-2.74494846
MAN2C1	-0.246017117	4.712448	-3.059866367	0.00245	0.00402	-2.74597558
RBP5	-0.356856434	2.6328376	-3.059693135	0.002451	0.00402	-2.74648676
BCORL1	-0.23932072	3.40520071	-3.059539678	0.002452	0.00402	-2.74693956
PHRF1	-0.195326141	4.69845536	-3.05879645	0.002458	0.00403	-2.74913228
SBNO2	0.210280618	4.70765889	3.058731063	0.002459	0.00403	-2.74932517
PHLDA3	0.29682567	6.09186065	3.058068031	0.002464	0.00404	-2.75128084
SLC28A2	-0.072001445	0.23792344	-3.057867854	0.002465	0.00404	-2.7518712
HIPK1	-0.311337182	4.92887516	-3.056407001	0.002477	0.00406	-2.75617846
SOWAHD	0.215375768	0.8943291	3.056396002	0.002477	0.00406	-2.75621088
MTF1	-0.221433001	2.87759928	-3.056005616	0.00248	0.00406	-2.75736159
COL21A1	-0.302990998	1.48275303	-3.055777508	0.002482	0.00407	-2.7580339
TMEM74	-0.087034979	0.11459747	-3.055527639	0.002484	0.00407	-2.75877029
TM9SF4	-0.209235728	5.69257272	-3.054974749	0.002488	0.00408	-2.76039953
EIF5A2	-0.1687174	1.46405871	-3.05483416	0.00249	0.00408	-2.76081377
AMIGO2	0.512388519	4.2000448	3.054680486	0.002491	0.00408	-2.76126654
MAVS	-0.189381627	4.96427685	-3.052823631	0.002506	0.0041	-2.76673576

NCOA6	-0.214532171	4.25589079	-3.052638073	0.002507	0.0041	-2.76728213
IL12A	-0.149845861	0.37664969	-3.052239803	0.00251	0.00411	-2.76845473
NXF5	-0.034447113	0.07728724	-3.051667061	0.002515	0.00412	-2.77014077
RANBP1	-0.238614687	1.08816602	-3.051308472	0.002518	0.00412	-2.77119624
RHBG	-0.04648581	0.08200493	-3.051034626	0.00252	0.00412	-2.7720022
RTL6	-0.315227364	4.23750022	-3.050959323	0.002521	0.00412	-2.77222381
APLNR	-0.407710042	4.882803	-3.050586595	0.002524	0.00413	-2.77332066
TMEM15	-0.230122082	5.29498258	-3.050420364	0.002525	0.00413	-2.77380979
GEN1	-0.142430216	1.33321245	-3.050085047	0.002528	0.00414	-2.77479639
IFT80	-0.204986264	3.25718163	-3.049792616	0.00253	0.00414	-2.77565672
ZNF208	-0.256248312	1.60740241	-3.048376883	0.002542	0.00416	-2.77982072
OXT	-0.191736286	0.63031473	-3.047778749	0.002547	0.00416	-2.78157944
ADPRHL	-0.195156477	0.70916402	-3.047576817	0.002548	0.00417	-2.78217311
RRP1	-0.18172997	4.27845376	-3.047196168	0.002551	0.00417	-2.78329211
MFSD4B	-0.162002937	1.75244234	-3.046904334	0.002554	0.00418	-2.78414992
KISS1R	0.236489671	0.8630327	3.046861221	0.002554	0.00418	-2.78427664
SLC25A3	-0.19053155	3.7956366	-3.046358746	0.002558	0.00418	-2.78575343
TMPRSS	0.412432699	2.50983109	3.046179507	0.00256	0.00418	-2.78628016
OASL	0.350422735	2.02522627	3.045168071	0.002568	0.0042	-2.78925193
HSF4	-0.376157326	2.75085779	-3.044861578	0.002571	0.0042	-2.79015228
POU4F3	-0.047888369	0.14381946	-3.044637727	0.002572	0.0042	-2.79080981
FZR1	-0.171320065	4.80366006	-3.043601976	0.002581	0.00422	-2.79385158
TNNC1	-0.477344496	3.81275912	-3.042989673	0.002586	0.00422	-2.79564933
GIGYF2	-0.234008498	4.25111051	-3.041956441	0.002595	0.00424	-2.79868218
ZNF493	-0.238587552	2.53146617	-3.041884255	0.002595	0.00424	-2.79889403
AZGP1	0.150145459	0.20051567	3.041227316	0.002601	0.00425	-2.8008218
KRTCAP	-0.200225334	4.27734405	-3.041117813	0.002602	0.00425	-2.8011431
ZNF610	-0.196637094	2.70321116	-3.040672007	0.002605	0.00425	-2.80245105
CCDC88	-0.233454247	2.32045509	-3.039536329	0.002615	0.00427	-2.80578219
HAUS2	-0.221506741	3.67348518	-3.038888411	0.00262	0.00428	-2.80768213
DCN	0.69498334	3.42711582	3.038570847	0.002623	0.00428	-2.80861321
CASP5	0.082364675	0.19749511	3.03853277	0.002623	0.00428	-2.80872485
AMMECF	-0.202244852	3.9697511	-3.037463721	0.002632	0.0043	-2.81185854
ZACN	-0.088966508	0.40205462	-3.037379722	0.002633	0.0043	-2.81210472
GRN	-0.228106947	8.3131437	-3.036984545	0.002636	0.0043	-2.81326281
SLC12A8	-0.324110744	5.22037757	-3.035939696	0.002645	0.00432	-2.81632412
TNFSF12	-0.186399465	5.60117631	-3.035647586	0.002648	0.00432	-2.8171798
RFC5	-0.213681301	4.0643839	-3.035146077	0.002652	0.00433	-2.81864869
SOX11	0.236245286	0.43270909	3.033505353	0.002666	0.00435	-2.82345272
NPM1	-0.20389417	8.60786688	-3.033479025	0.002666	0.00435	-2.82352979

MAP9	-0.261519332	3.0772713	-3.032872207	0.002671	0.00436	-2.82530592
ECRG4	-0.634496974	6.91439416	-3.03211714	0.002678	0.00437	-2.82751551
FOXD4	-0.120927716	0.44095138	-3.030197355	0.002694	0.00439	-2.83313116
REN	0.435105546	1.7360679	3.030132551	0.002695	0.00439	-2.83332066
STN1	-0.158063719	5.57049224	-3.029323023	0.002702	0.0044	-2.83568761
KCNK5	-0.32257331	5.3728422	-3.02864122	0.002708	0.00441	-2.83768065
MGA	-0.232266715	2.9896272	-3.028201855	0.002711	0.00442	-2.83896478
EXOC6B	0.259962118	3.58436105	3.02818013	0.002712	0.00442	-2.83902827
KLRC4	0.108628216	0.20124948	3.027918075	0.002714	0.00442	-2.83979409
SLC25A3	-0.255613121	4.40284164	-3.027075833	0.002721	0.00443	-2.842255
CDK5R1	0.187138827	1.08219562	3.02656822	0.002726	0.00444	-2.84373787
ME2	-0.185289926	3.27069121	-3.026051204	0.00273	0.00445	-2.84524796
BAG3	0.313588121	6.27405634	3.025972729	0.002731	0.00445	-2.84547715
ANO1	-0.298981217	4.78123244	-3.025547246	0.002734	0.00445	-2.84671969
MPIG6B	0.189203414	0.80920358	3.025221485	0.002737	0.00446	-2.8476709
SEMA5B	-0.29273961	1.49424421	-3.024986644	0.002739	0.00446	-2.84835657
PPWD1	-0.206056015	4.22523498	-3.02481689	0.002741	0.00446	-2.84885217
MELK	0.226193561	1.19814276	3.024722018	0.002742	0.00446	-2.84912914
TMEM86	-0.251726744	1.97273953	-3.023925401	0.002749	0.00447	-2.85145447
S1PR1	-0.289532006	5.26521868	-3.023735719	0.00275	0.00448	-2.85200807
NCKIPSD	-0.18191242	4.71973481	-3.023599964	0.002751	0.00448	-2.85240426
UBFD1	-0.229966603	4.87961748	-3.02238922	0.002762	0.0045	-2.85593699
GUCA2A	-0.10693687	0.1716129	-3.021917464	0.002766	0.0045	-2.85731313
CEP78	-0.16291792	1.97192542	-3.02162418	0.002769	0.00451	-2.85816855
GSE1	-0.241187341	5.07259537	-3.020674923	0.002777	0.00452	-2.86093674
PCNT	-0.209082349	3.36412842	-3.02059636	0.002778	0.00452	-2.8611658
RNF6	-0.22088187	4.37220366	-3.020022029	0.002783	0.00453	-2.86284021
COL28A1	-0.175857803	0.51412419	-3.019947296	0.002784	0.00453	-2.86305806
IL34	-0.35620332	3.64214162	-3.019549124	0.002787	0.00453	-2.86421869
WDR47	-0.233417053	3.06552968	-3.017272797	0.002807	0.00457	-2.87085122
ATP13A3	-0.282287021	5.38992817	-3.017272347	0.002807	0.00457	-2.87085253
ZNF784	-0.194205304	3.91317812	-3.016471764	0.002815	0.00458	-2.87318407
SRMS	-0.20179434	0.99330757	-3.016238302	0.002817	0.00458	-2.87386388
C11orf86	0.122041804	0.19463592	3.015659866	0.002822	0.00459	-2.87554799
MIDN	0.305637049	5.94153759	3.014908439	0.002829	0.0046	-2.87773531
KLF1	-0.046242012	0.14273373	-3.014115043	0.002836	0.00461	-2.88004424
DOC2A	-0.066254674	0.14489583	-3.014095577	0.002836	0.00461	-2.88010088
OR7A5	-0.053028285	0.15071541	-3.013702168	0.00284	0.00461	-2.88124556
PLCB3	-0.147166413	4.28584934	-3.013392047	0.002842	0.00462	-2.8821478
RPS15	-0.230075825	8.47048388	-3.013352607	0.002843	0.00462	-2.88226254

THRAP3	-0.224454816	6.78142359	-3.01281281	0.002848	0.00463	-2.88383276
AGRP	0.169212084	0.35487695	3.012803832	0.002848	0.00463	-2.88385887
ST18	0.02047239	0.04011677	3.012780121	0.002848	0.00463	-2.88392784
LGALS9	0.334125815	3.52566223	3.011892041	0.002856	0.00464	-2.88651059
TXLNA	-0.178155965	5.99316287	-3.011632858	0.002858	0.00464	-2.88726422
CDYL2	-0.187101432	1.70371885	-3.011495212	0.00286	0.00464	-2.88766443
VGLL4	-0.357967945	4.24581092	-3.011265733	0.002862	0.00465	-2.88833161
HELZ	-0.224588809	3.06789963	-3.010808525	0.002866	0.00465	-2.88966075
PDK3	0.239103476	2.47568581	3.009163552	0.002881	0.00468	-2.89444124
DNAJC1:	-0.161526143	0.67054507	-3.008867947	0.002884	0.00468	-2.89530004
MYRF	0.412646311	0.96710924	3.008850278	0.002884	0.00468	-2.89535137
TMEM18	-0.237040202	5.83595196	-3.008114419	0.00289	0.00469	-2.89748888
ZNF223	-0.14792717	1.34284135	-3.007399232	0.002897	0.0047	-2.89956586
HSPA2	-0.254469402	2.15179623	-3.006463586	0.002906	0.00471	-2.90228239
DMRTB1	-0.224063372	0.24894397	-3.00426495	0.002926	0.00475	-2.90866276
PCDH11	-0.048312478	0.05167325	-3.003141302	0.002936	0.00476	-2.91192187
SLC35E1	-0.227860462	4.85977158	-3.002850416	0.002939	0.00477	-2.91276539
PDP2	-0.224064192	3.59482773	-3.002334536	0.002944	0.00478	-2.91426117
FAM89B	0.240947772	5.26767504	3.00194387	0.002948	0.00478	-2.91539373
FANCI	0.20336804	2.05379013	3.001816001	0.002949	0.00478	-2.9157644
CTCFL	-0.039988699	0.03647233	-3.001073693	0.002956	0.00479	-2.91791594
PTTG1IP	-0.205664777	8.35747477	-3.000943406	0.002957	0.00479	-2.91829351
TFPI2	0.354091311	1.0575505	3.000716327	0.002959	0.0048	-2.91895156
POLA2	-0.151410187	3.15544644	-2.999870753	0.002967	0.00481	-2.92140153
PRSS37	0.051782691	0.15510298	2.99964065	0.002969	0.00481	-2.92206812
CD6	0.360237896	1.58130665	2.998803756	0.002977	0.00483	-2.92449213
ZNF518A	-0.265308143	3.1219875	-2.99562507	0.003007	0.00487	-2.93369326
TNNT2	0.071943293	0.16406318	2.995456127	0.003009	0.00488	-2.93418203
ZNF449	-0.192879825	2.24863827	-2.99505903	0.003013	0.00488	-2.93533078
INPP4A	0.233951821	3.25690519	2.995028813	0.003013	0.00488	-2.93541819
RALGAP	-0.203619636	2.39190142	-2.994919728	0.003014	0.00488	-2.93573373
ERMAP	-0.255455334	4.26187661	-2.993916657	0.003024	0.0049	-2.93863473
SOX21	-0.040332425	0.06178771	-2.992432703	0.003038	0.00492	-2.94292483
LUC7L3	-0.240937641	5.55450628	-2.992046284	0.003042	0.00493	-2.94404164
NCBP3	-0.195470518	3.42343214	-2.991809542	0.003044	0.00493	-2.9447258
SSX3	-0.092739086	0.16674162	-2.991337963	0.003048	0.00494	-2.94608845
DMXL1	-0.256499944	3.6450059	-2.991231419	0.003049	0.00494	-2.94639628
MSH2	-0.245455151	4.58016849	-2.99115484	0.00305	0.00494	-2.94661754
P2RY14	-0.245769703	1.46252607	-2.990784108	0.003054	0.00494	-2.94768859
CST1	0.58402666	0.87446641	2.99041248	0.003057	0.00495	-2.9487621

PLN	-0.372619848	2.43132509	-2.990030133	0.003061	0.00495	-2.94986645
PARG	-0.210333562	3.38738744	-2.989845313	0.003063	0.00496	-2.95040022
HSPA1A	-0.356538168	5.75620198	-2.988999049	0.003071	0.00497	-2.95284391
ZNF683	0.430278433	1.75289366	2.988464337	0.003076	0.00498	-2.95438762
KCNK9	-0.046278116	0.04966694	-2.987719171	0.003083	0.00499	-2.95653848
CIT	-0.232996238	1.39867976	-2.98709604	0.00309	0.005	-2.95833671
C6orf62	-0.18529327	7.2568551	-2.9867211	0.003093	0.005	-2.95941854
SLC16A1	-0.193749374	1.9429358	-2.986231974	0.003098	0.00501	-2.96082965
CYP2F1	0.021916422	0.03492248	2.984734928	0.003113	0.00503	-2.96514724
FYCO1	-0.248924128	4.91784746	-2.984501134	0.003115	0.00504	-2.96582133
LONRF1	-0.237063914	3.63813627	-2.984328499	0.003117	0.00504	-2.96631906
PCGF5	-0.230332205	4.33878292	-2.984004049	0.00312	0.00504	-2.96725441
SNX22	0.541845272	6.50326359	2.983430489	0.003125	0.00505	-2.96890768
RNF115	-0.180717924	4.16318091	-2.9833794	0.003126	0.00505	-2.96905493
RARRES	0.605506225	4.34581966	2.98309299	0.003129	0.00506	-2.96988038
NELFA	-0.197432599	4.66133386	-2.98305907	0.003129	0.00506	-2.96997813
SPIN2A	-0.043761416	0.12132762	-2.982987922	0.00313	0.00506	-2.97018317
SLC27A1	-0.189417128	4.45070677	-2.981917621	0.00314	0.00507	-2.97326707
SSX7	-0.044665468	0.07958183	-2.981840257	0.003141	0.00508	-2.97348994
NPLOC4	-0.16378123	4.84316825	-2.98100132	0.00315	0.00509	-2.97590642
ESCO1	-0.207756103	3.87646096	-2.980763527	0.003152	0.00509	-2.97659125
GRID2IP	0.132908822	1.01311355	2.979922573	0.00316	0.0051	-2.97901272
MFSD13A	-0.209715152	3.48125834	-2.979161465	0.003168	0.00512	-2.98120374
MUCL3	0.067301683	0.2503261	2.978993337	0.00317	0.00512	-2.98168766
ITCH	-0.23544301	4.70737656	-2.977887515	0.003181	0.00514	-2.98486991
UBQLN1	-0.228143952	5.62005813	-2.977155095	0.003188	0.00515	-2.986977
MAP4	0.222060023	5.64821711	2.976322173	0.003196	0.00516	-2.98937265
FBXO34	-0.235889798	5.47762618	-2.974785793	0.003212	0.00518	-2.99378992
SAMD9L	0.302287809	3.57013759	2.974218339	0.003218	0.00519	-2.99542088
RAB43	-0.226827003	1.49485372	-2.973969266	0.00322	0.0052	-2.99613667
SERPINF	0.504300122	4.55545326	2.97345125	0.003225	0.00521	-2.99762517
CCDC15	-0.19818017	1.0554757	-2.970987454	0.00325	0.00525	-3.00470147
CD48	0.447209175	2.38457734	2.969853092	0.003262	0.00526	-3.00795765
LRRC8B	-0.21164043	2.96429742	-2.969783836	0.003263	0.00526	-3.00815641
SPTY2D	-0.24469951	3.94988774	-2.969543517	0.003265	0.00527	-3.00884608
OGFR	-0.180717151	5.26329268	-2.969218603	0.003269	0.00527	-3.00977844
NONO	-0.20569567	7.49019459	-2.969062798	0.00327	0.00527	-3.0102255
NDST1	-0.252444502	5.5300164	-2.967596155	0.003285	0.0053	-3.01443272
CHORDC	-0.198103456	2.70672078	-2.967310149	0.003288	0.0053	-3.01525293
SNIP1	-0.187345741	3.51730037	-2.967146503	0.00329	0.0053	-3.01572221

ARL2BP	-0.282710663	2.78699827	-2.966485778	0.003297	0.00532	-3.01761667
BACE2	-0.222535748	3.8534883	-2.965573606	0.003306	0.00533	-3.02023144
C5orf51	-0.205948292	4.31165978	-2.964461252	0.003318	0.00535	-3.02341902
IL20RB	-0.171339023	0.66451236	-2.963448083	0.003328	0.00536	-3.02632141
ZNF254	-0.234608307	3.51360851	-2.963209919	0.003331	0.00537	-3.02700353
NKAIN4	0.468065682	2.68072082	2.963117276	0.003332	0.00537	-3.02726885
CDC34	-0.181409853	6.28483346	-2.963004408	0.003333	0.00537	-3.02759209
ZSCAN2!	-0.208730361	3.12916593	-2.961300837	0.003351	0.0054	-3.03246948
MARK4	-0.142095584	4.14882271	-2.960737563	0.003357	0.00541	-3.03408157
SCD5	-0.335969026	6.60280581	-2.960190821	0.003363	0.00542	-3.03564608
KIAA051:	-0.173692605	2.24147109	-2.959550993	0.003369	0.00543	-3.03747661
SWAP70	-0.261596265	5.12030741	-2.958351487	0.003382	0.00545	-3.04090738
ZIK1	-0.225873396	2.6881285	-2.957209953	0.003394	0.00547	-3.04417113
AIF1	0.402182426	4.65763902	2.95675144	0.003399	0.00547	-3.04548173
KLHL26	-0.215102743	4.06714398	-2.95649175	0.003402	0.00548	-3.04622393
UFL1	-0.23213002	5.11431097	-2.956215856	0.003405	0.00548	-3.04701238
TLE3	-0.228665666	4.43094043	-2.954425101	0.003424	0.00551	-3.05212833
TLK1	-0.189968958	4.3808754	-2.954302958	0.003425	0.00551	-3.05247718
PIK3CG	0.249390748	1.20034519	2.954301949	0.003425	0.00551	-3.05248006
FBXL6	-0.214111601	3.36190274	-2.954018651	0.003428	0.00552	-3.05328911
LZTS2	0.231637353	5.9605213	2.953695538	0.003432	0.00552	-3.05421177
AGTRAP	-0.184196494	6.16386835	-2.95352312	0.003434	0.00552	-3.05470408
TPT1	-0.19564794	10.6463216	-2.952667273	0.003443	0.00554	-3.0571474
LIPM	0.147240176	0.35021388	2.951834026	0.003452	0.00555	-3.05952557
ADAMDE	0.442260506	1.12756307	2.951552258	0.003455	0.00556	-3.06032963
FUT4	-0.154168616	1.74134195	-2.951490786	0.003456	0.00556	-3.06050503
RAB39A	0.089860188	0.2480202	2.951249317	0.003458	0.00556	-3.06119401
ACTL6A	-0.207281856	4.69699457	-2.950957963	0.003461	0.00557	-3.06202527
MPZL3	0.300802539	4.65419705	2.950556634	0.003466	0.00557	-3.06317016
ZFPM2	-0.297389252	1.51674143	-2.950542616	0.003466	0.00557	-3.06321014
RPL3	-0.226157577	10.679806	-2.950451505	0.003467	0.00557	-3.06347004
FBXW11	-0.234290664	4.88772899	-2.950361887	0.003468	0.00557	-3.06372567
ZBTB7B	-0.198656799	5.46317217	-2.95020277	0.00347	0.00558	-3.06417952
NDUFAF	-0.149943068	3.33697759	-2.950010138	0.003472	0.00558	-3.06472894
MICAL1	0.289021338	3.37950952	2.949541404	0.003477	0.00559	-3.0660657
SLC38A!	-0.145312217	2.13554231	-2.949121947	0.003481	0.00559	-3.06726177
JADE1	-0.24531326	5.1916391	-2.948955366	0.003483	0.0056	-3.06773673
ZFYVE1(	-0.195321595	3.3064753	-2.948461865	0.003488	0.0056	-3.06914365
ZNF529	-0.165547147	2.82837897	-2.948407459	0.003489	0.00561	-3.06929874
ABO	-0.318228828	4.82237358	-2.948008571	0.003493	0.00561	-3.07043575

GPR162	-0.351753882	1.95438402	-2.946560094	0.003509	0.00564	-3.07456335
RBMX	-0.198202294	6.50119023	-2.946416493	0.003511	0.00564	-3.07497245
ZNF654	-0.20910778	3.28249583	-2.945667357	0.003519	0.00565	-3.07710636
CHI3L2	0.446624804	4.17356386	2.94528809	0.003523	0.00566	-3.07818651
C3orf80	0.193741435	0.83150927	2.944606101	0.003531	0.00567	-3.08012847
BRINP3	-0.140534423	0.35978633	-2.944209905	0.003535	0.00568	-3.08125645
UBE3B	-0.181061038	4.05768149	-2.943991922	0.003538	0.00568	-3.08187699
EFL1	-0.204610034	4.02975554	-2.943892366	0.003539	0.00568	-3.08216038
PLEKHG	-0.233354382	3.69904383	-2.942058487	0.003559	0.00571	-3.08737909
EIF4A2	-0.225128442	8.0432942	-2.941762301	0.003562	0.00572	-3.08822167
MALRD1	-0.109373302	0.2638753	-2.941677427	0.003563	0.00572	-3.0884631
CALU	-0.287332844	6.57243885	-2.941653452	0.003563	0.00572	-3.0885313
DPY19L1	0.295548085	4.05095238	2.941084302	0.00357	0.00573	-3.09015011
SLC22A4	0.212860994	1.65708894	2.940277934	0.003579	0.00574	-3.09244314
TUBA4A	0.321107852	4.90804395	2.938510158	0.003599	0.00577	-3.09746802
RANBP9	-0.187868183	5.40098419	-2.938426262	0.0036	0.00577	-3.09770642
ZNF675	-0.264254159	2.35826408	-2.938095123	0.003603	0.00578	-3.09864734
AGBL2	-0.145849801	0.78742521	-2.938026693	0.003604	0.00578	-3.09884177
SLC45A3	0.19990857	1.17617341	2.937289111	0.003612	0.00579	-3.10093718
DNAJB7	-0.062567088	0.23029274	-2.93477664	0.003641	0.00584	-3.10807121
BTN3A2	0.320095108	3.94306103	2.934574433	0.003643	0.00584	-3.10864511
ERICH5	-0.368461761	1.63086276	-2.934475799	0.003644	0.00584	-3.10892504
TNKS1BI	-0.222523489	6.27273683	-2.934473099	0.003644	0.00584	-3.10893271
EIF3E	-0.194084179	7.11267824	-2.934472682	0.003644	0.00584	-3.10893389
BIRC6	-0.272152281	3.98659673	-2.933817923	0.003652	0.00585	-3.11079193
PRPF39	-0.218509559	3.87268667	-2.933541906	0.003655	0.00586	-3.11157508
KLRC2	0.105949425	0.2302971	2.932513364	0.003666	0.00587	-3.11449277
C19orf48	-0.244288467	5.56020462	-2.931494778	0.003678	0.00589	-3.11738128
BTLA	0.241863367	0.5578194	2.931441321	0.003679	0.00589	-3.11753285
INTS3	-0.270918327	3.82693236	-2.930680764	0.003687	0.00591	-3.11968899
FAM166A	-0.094509719	0.21504695	-2.930611567	0.003688	0.00591	-3.11988514
ANKRD1	-0.189895333	3.90777406	-2.930196777	0.003693	0.00591	-3.1210608
PIEZO2	-0.272603671	1.52877806	-2.929915911	0.003696	0.00592	-3.12185679
STXBP2	-0.179050722	5.11796887	-2.929675305	0.003699	0.00592	-3.12253862
FAM219B	-0.243486147	3.41457764	-2.928892241	0.003708	0.00594	-3.12475731
IRGQ	-0.164878069	3.4920798	-2.928804794	0.003709	0.00594	-3.12500504
LY96	0.379902496	3.67018351	2.928593343	0.003711	0.00594	-3.12560405
EGFLAM	-0.266466469	2.07458151	-2.928384042	0.003714	0.00594	-3.12619692
C4orf48	-0.528691015	4.86992671	-2.926812591	0.003732	0.00597	-3.13064698
RBM15	-0.144300522	2.7172089	-2.926582161	0.003735	0.00598	-3.13129934

IL6	0.538911064	1.55815724	2.92624443	0.003739	0.00598	-3.13225537
TRPS1	-0.19868655	2.52619505	-2.926072191	0.003741	0.00598	-3.13274289
GFOD1	-0.200381082	1.78184349	-2.925284566	0.00375	0.006	-3.13497194
GPRC5D	-0.16830876	1.12996813	-2.925012827	0.003753	0.006	-3.13574085
ATG2B	-0.217365435	3.14516308	-2.924845849	0.003755	0.00601	-3.1362133
EMILIN1	0.4313518	3.51761982	2.923843557	0.003767	0.00602	-3.13904866
MSRB3	-0.329712764	3.17792976	-2.920856584	0.003802	0.00608	-3.14749306
HAAO	-0.267526292	2.99290555	-2.920280703	0.003809	0.00609	-3.14912019
SPIRE1	0.344905123	3.05491233	2.92013914	0.00381	0.00609	-3.14952012
FAM170A	-0.047001811	0.10464753	-2.919965764	0.003812	0.00609	-3.15000991
SMC1A	-0.25026711	4.36916634	-2.919884652	0.003813	0.0061	-3.15023904
KCNS2	0.034027306	0.06416263	2.918976482	0.003824	0.00611	-3.1528041
RBM7	-0.207702055	3.15828074	-2.918702283	0.003827	0.00612	-3.15357841
DLGAP4	0.198037044	5.35356523	2.918678134	0.003828	0.00612	-3.15364661
UPF1	-0.192655214	5.86487255	-2.917822885	0.003838	0.00613	-3.15606128
IPPK	-0.193441731	1.49118642	-2.917580805	0.003841	0.00614	-3.15674464
PLCE1	-0.364505269	3.24088863	-2.91716145	0.003846	0.00614	-3.1579283
PHF21B	-0.238028754	0.83768877	-2.916780567	0.00385	0.00615	-3.15900323
XPO4	-0.209892256	3.07085533	-2.916733491	0.003851	0.00615	-3.15913608
TIPARP	0.442192895	5.41523914	2.915250929	0.003869	0.00618	-3.16331887
BTBD2	-0.173386605	5.58996619	-2.914803441	0.003874	0.00619	-3.16458098
CCDC68	-0.203704209	1.13604332	-2.914242068	0.003881	0.0062	-3.16616405
SLC38A7	-0.148137144	3.51059103	-2.913957302	0.003884	0.0062	-3.16696698
CXCR6	0.374943619	1.55057174	2.912441034	0.003902	0.00623	-3.17124102
SMAD1	-0.280982455	2.74368204	-2.912063639	0.003907	0.00624	-3.17230449
BNIP2	-0.202339209	4.00909316	-2.910867652	0.003921	0.00626	-3.17567386
LRMP	-0.323525295	2.09004271	-2.910752488	0.003923	0.00626	-3.17599823
POLK	-0.231367294	3.5169869	-2.910338922	0.003928	0.00627	-3.17716299
MFSD14A	-0.253083525	5.57795418	-2.909429339	0.003939	0.00629	-3.17972419
GPR82	0.14204347	0.5274531	2.908365287	0.003952	0.00631	-3.18271939
APLP1	-0.331433099	1.98861481	-2.907342727	0.003964	0.00633	-3.18559682
NDST2	-0.10601158	1.17887738	-2.906964437	0.003969	0.00633	-3.18666107
FIBCD1	0.483332171	1.4184184	2.906834723	0.003971	0.00633	-3.18702597
SHANK1	-0.131048351	0.29032195	-2.905012547	0.003993	0.00637	-3.1921503
ABCA4	0.166044705	0.84203862	2.904613364	0.003998	0.00638	-3.19327248
PLB1	-0.164411347	0.97758979	-2.904589202	0.003998	0.00638	-3.1933404
RALGAP	-0.305188982	4.47617211	-2.904428917	0.004	0.00638	-3.19379095
SNX10	0.319367739	2.63758725	2.903306659	0.004014	0.0064	-3.19694488
CSNK2B	-0.174804951	5.97857882	-2.903083528	0.004017	0.0064	-3.19757182
SDAD1	-0.29049532	4.66939415	-2.902660629	0.004022	0.00641	-3.19875993

SNRNP2	-0.22000189	5.86133709	-2.902414832	0.004025	0.00642	-3.1994504
NLRP12	0.061358084	0.22723384	2.901979675	0.004031	0.00642	-3.20067269
OPRD1	-0.052105504	0.12406081	-2.901847678	0.004032	0.00643	-3.20104341
BCL9L	0.351903243	3.28555009	2.901348859	0.004039	0.00644	-3.20244423
ERO1A	0.178240688	4.62083212	2.900976692	0.004043	0.00644	-3.20348924
TSPAN8	-0.266957674	0.59001713	-2.900864631	0.004045	0.00644	-3.20380387
FHOD1	0.26130379	5.05465856	2.900679729	0.004047	0.00645	-3.20432299
GYPE	-0.236771984	0.66227692	-2.899200841	0.004065	0.00648	-3.20847391
GOLGB1	-0.230661157	5.53838115	-2.899015839	0.004068	0.00648	-3.20899303
ULK3	-0.220580239	5.16772407	-2.89857558	0.004073	0.00649	-3.21022828
EEF1A1	-0.253603915	11.6416596	-2.898562408	0.004073	0.00649	-3.21026524
MMAA	-0.122902317	2.28152051	-2.897062409	0.004092	0.00652	-3.21447252
NKRF	-0.160090523	3.28430619	-2.896089194	0.004105	0.00654	-3.21720116
DCAF16	-0.200354956	4.33990202	-2.894867419	0.00412	0.00656	-3.22062547
LYPLA2	-0.235628445	6.86902904	-2.894704515	0.004122	0.00656	-3.22108195
TRPM2	0.235623046	1.48756932	2.894527275	0.004124	0.00657	-3.22157857
CADM3	0.607517118	1.85367057	2.893587333	0.004136	0.00658	-3.22421177
ADCY10	-0.038052583	0.10452204	-2.893251782	0.004141	0.00659	-3.22515161
RNGTT	-0.189976571	3.3916422	-2.892694184	0.004148	0.0066	-3.22671314
MAG	0.571018844	2.53864878	2.892384887	0.004152	0.00661	-3.2275792
COL5A2	0.416518368	4.08439138	2.892243935	0.004154	0.00661	-3.22797384
ZNF71	-0.132468926	2.23472317	-2.892207602	0.004154	0.00661	-3.22807557
HELLS	0.191890815	1.49035569	2.891432598	0.004164	0.00662	-3.23024514
SETDB1	-0.189453738	3.99593598	-2.889996237	0.004182	0.00665	-3.23426468
CRABP2	0.742886558	6.82382003	2.88991464	0.004183	0.00665	-3.23449297
FAT4	-0.287951397	2.14783424	-2.889831741	0.004185	0.00665	-3.23472489
SLC47A2	-0.237034988	0.53791973	-2.889732464	0.004186	0.00666	-3.23500262
PANK3	-0.250716893	3.99650276	-2.889470437	0.004189	0.00666	-3.23573562
LRRC39	-0.214709646	1.30910685	-2.888577763	0.004201	0.00668	-3.23823233
TAZ	-0.180262937	3.94462787	-2.888329792	0.004204	0.00668	-3.23892575
SPATA18	-0.464449682	2.69243603	-2.887591595	0.004214	0.0067	-3.24098969
MEX3D	0.277151863	4.33916581	2.887527603	0.004214	0.0067	-3.24116859
CLIC1	-0.20971322	8.89335967	-2.887407676	0.004216	0.0067	-3.24150384
PSME2	-0.213440909	6.34905579	-2.887379553	0.004216	0.0067	-3.24158245
CEP131	-0.170264872	3.70500425	-2.88699879	0.004221	0.00671	-3.24264677
RFPL3	-0.029909419	0.07184682	-2.886425162	0.004229	0.00672	-3.24424995
TEC	0.193476618	2.52724036	2.885941899	0.004235	0.00673	-3.24560033
TEX35	-0.020942551	0.03245772	-2.885859894	0.004236	0.00673	-3.24582946
ARSD	-0.210624909	5.62197838	-2.884883745	0.004249	0.00675	-3.24855641
MBD6	-0.250034222	5.60034162	-2.88405895	0.00426	0.00677	-3.25085986

GABRQ	-0.20525118	0.37030122	-2.882878841	0.004275	0.00679	-3.25415455
BCR	-0.26640544	4.9385235	-2.88266552	0.004278	0.00679	-3.25474997
TRMT44	-0.204236741	3.04827128	-2.881695325	0.004291	0.00681	-3.25745748
LNX2	-0.250780389	3.82086626	-2.881311168	0.004296	0.00682	-3.25852931
C1R	0.546886634	4.53628415	2.879694705	0.004317	0.00685	-3.26303788
REEP2	0.345142018	2.03073061	2.879536805	0.004319	0.00686	-3.26347816
TMEM72	-0.245452378	0.68617207	-2.878904208	0.004328	0.00687	-3.26524184
PROSER	-0.190645249	3.67824054	-2.877641149	0.004344	0.00689	-3.26876215
RAD1	-0.188983961	3.55210215	-2.876880422	0.004354	0.00691	-3.27088171
RNF138	-0.200712365	3.86896293	-2.876699329	0.004357	0.00691	-3.27138619
HLA-C	0.363193958	9.89685386	2.875979644	0.004367	0.00693	-3.27339079
TRIM11	-0.141477273	3.1612259	-2.875832815	0.004369	0.00693	-3.27379971
SSX5	-0.212092927	0.57931256	-2.875572224	0.004372	0.00694	-3.2745254
NMI	-0.221409868	3.90787445	-2.874533953	0.004386	0.00696	-3.27741617
PCSK9	0.074430582	0.1096458	2.87446937	0.004387	0.00696	-3.27759595
SLC2A10	-0.295367858	4.97449697	-2.874416323	0.004388	0.00696	-3.27774362
SLC38A1	-0.316911113	4.85954763	-2.874236486	0.00439	0.00696	-3.2782442
PNP	0.510478392	5.16630033	2.873884856	0.004395	0.00697	-3.2792229
FOXO4	-0.209880632	4.60247983	-2.873854266	0.004395	0.00697	-3.27930804
PCDHB5	-0.533050941	2.49595435	-2.87340592	0.004401	0.00698	-3.28055575
FOXS1	-0.328260922	2.75230161	-2.873327488	0.004402	0.00698	-3.280774
MAP6D1	-0.15101117	1.36317523	-2.8731119	0.004405	0.00698	-3.28137389
JRKL	-0.226265347	3.80577122	-2.873050047	0.004406	0.00698	-3.28154599
CROCC2	-0.058551618	0.07208088	-2.872041442	0.00442	0.007	-3.28435186
LCAT	-0.281678477	3.4355816	-2.87132419	0.004429	0.00702	-3.28634665
ANXA5	-0.221500304	8.56549159	-2.87127982	0.00443	0.00702	-3.28647004
EPHA2	0.355455551	5.58252867	2.871096659	0.004432	0.00702	-3.28697936
PRKX	-0.330515918	4.80537256	-2.870645885	0.004439	0.00703	-3.2882327
MSLN	0.398445123	0.9294092	2.870486193	0.004441	0.00703	-3.28867666
LY9	0.234024908	0.54151748	2.868957252	0.004462	0.00707	-3.29292616
RGMB	-0.192300683	3.06109122	-2.868809057	0.004464	0.00707	-3.29333794
VAT1	0.209765114	7.6477091	2.868501109	0.004468	0.00707	-3.29419354
KCNIP2	-0.167497033	1.03564338	-2.867760359	0.004478	0.00709	-3.2962513
ABCA3	-0.241343453	5.44156666	-2.86758535	0.00448	0.00709	-3.29673738
ADAM10	-0.239466302	4.41995575	-2.865623165	0.004507	0.00714	-3.30218548
ITGB3BF	-0.188043124	2.89825276	-2.865171825	0.004514	0.00714	-3.30343815
TBC1D2:	-0.162207418	4.76867764	-2.864918086	0.004517	0.00715	-3.30414231
RETREG	-0.169448021	5.68572277	-2.864838147	0.004518	0.00715	-3.30436414
CXCL10	0.637738014	3.15409112	2.86466263	0.004521	0.00715	-3.30485117
RPGRIP:	-0.062104436	0.37481854	-2.864505884	0.004523	0.00716	-3.30528609

TP53BP1	-0.295697509	3.83425584	-2.863303285	0.004539	0.00718	-3.30862222
SLC8A1	0.128622678	0.69402466	2.861634452	0.004563	0.00722	-3.31324954
SYCP2	-0.156634252	0.81932462	-2.861232448	0.004568	0.00723	-3.31436383
MMP19	0.303590362	1.74033726	2.859405003	0.004594	0.00727	-3.31942738
ASCL2	-0.255141311	1.2282945	-2.859146397	0.004597	0.00727	-3.32014369
CACNA1	-0.234698331	1.54980955	-2.859106679	0.004598	0.00727	-3.3202537
PTGDR2	-0.052435209	0.13996785	-2.857110558	0.004626	0.00732	-3.32578061
CCNE1	-0.156059415	2.05576946	-2.857031102	0.004627	0.00732	-3.32600054
SAV1	-0.193684814	5.25469225	-2.856892136	0.004629	0.00732	-3.32638516
CACNB3	-0.22142047	3.48331973	-2.856745406	0.004631	0.00732	-3.32679126
SETD1A	-0.159148545	4.34739276	-2.856679351	0.004632	0.00732	-3.32697407
FEM1B	-0.203243805	4.71205541	-2.855275606	0.004652	0.00735	-3.33085808
ZNF718	-0.251681614	2.62871293	-2.854917213	0.004657	0.00736	-3.33184943
NPC1L1	0.02091559	0.04354778	2.853275486	0.00468	0.0074	-3.3363891
TYW5	-0.144619843	1.72418832	-2.853177328	0.004682	0.0074	-3.33666045
TIRAP	-0.189697367	2.66967852	-2.852024898	0.004698	0.00742	-3.33984556
SGCB	-0.300830129	4.62538553	-2.851333562	0.004708	0.00744	-3.34175571
ZFP91	-0.243845109	5.31058733	-2.850491237	0.00472	0.00746	-3.34408245
UNC5B	0.349939393	4.42945628	2.850407628	0.004722	0.00746	-3.34431337
FRZB	-0.375474283	3.58797847	-2.849647954	0.004733	0.00747	-3.34641119
PHF12	-0.18285729	3.97485663	-2.849259303	0.004738	0.00748	-3.34748424
RAB2B	-0.181735412	4.42144677	-2.84831752	0.004752	0.0075	-3.3500839
CD209	0.232881918	1.06459119	2.847945876	0.004757	0.00751	-3.35110954
ZMYM2	-0.222883637	4.14755852	-2.847175691	0.004768	0.00753	-3.35323467
IFI35	-0.244671048	5.36160058	-2.846829994	0.004773	0.00754	-3.35418835
NEO1	-0.240514944	4.3999497	-2.846779391	0.004774	0.00754	-3.35432795
PRR15	0.938784591	5.84505424	2.846090206	0.004784	0.00755	-3.35622887
ZNF354C	-0.209979551	2.38104168	-2.845716764	0.004789	0.00756	-3.35725872
IFI6	-0.399776658	8.89312502	-2.845694144	0.00479	0.00756	-3.3573211
ATP6AP1	-0.151860192	1.79404327	-2.844693874	0.004804	0.00758	-3.36007893
EML3	-0.205384274	4.91315244	-2.843681816	0.004819	0.0076	-3.36286833
VHL	-0.262403455	4.71251523	-2.841756067	0.004848	0.00765	-3.36817345
NUDT14	-0.202665287	5.83791923	-2.840921418	0.00486	0.00767	-3.37047173
GZMK	0.512729104	1.71543094	2.840365398	0.004868	0.00768	-3.37200242
OSBPL1	-0.205097321	3.8309373	-2.839748195	0.004877	0.00769	-3.37370122
NUMBL	0.267326511	3.02710148	2.839366536	0.004883	0.0077	-3.37475153
DCAF12I	-0.139134629	0.2540879	-2.838569634	0.004895	0.00772	-3.37694415
CCDC38	-0.0259086	0.07465069	-2.83856116	0.004895	0.00772	-3.37696746
ITPRIPL2	0.342303818	3.92470095	2.838358841	0.004898	0.00772	-3.37752403
GRAPL	-0.049232826	0.10228365	-2.838011423	0.004903	0.00773	-3.37847968

IL32	0.412576684	3.83662673	2.837853133	0.004905	0.00773	-3.37891505
GEMIN4	-0.204987346	3.86941943	-2.837846756	0.004905	0.00773	-3.37893259
WDHD1	0.182750306	1.79104131	2.836656717	0.004923	0.00776	-3.38220503
SERPINF	0.266201866	1.5740136	2.836636476	0.004923	0.00776	-3.38226068
HK2	-0.346045767	5.4549269	-2.836438287	0.004926	0.00776	-3.38280554
MICALL2	0.235786902	3.19455974	2.836142109	0.004931	0.00777	-3.38361974
LILRB1	0.254794351	1.39050717	2.835249854	0.004944	0.00779	-3.38607205
UBE2V1	-0.172797768	2.30298034	-2.834502248	0.004955	0.00781	-3.38812625
APOBEC	0.279950723	1.91975464	2.834407315	0.004957	0.00781	-3.38838706
VNN1	0.18949131	0.76305065	2.834395845	0.004957	0.00781	-3.38841857
CHIA	-0.260775781	1.09240949	-2.834046311	0.004962	0.00781	-3.38937877
ATP13A2	-0.180651566	4.83005796	-2.833966619	0.004964	0.00782	-3.38959768
NR6A1	-0.236799799	2.53434789	-2.833412392	0.004972	0.00783	-3.39111993
MAP3K7	-0.190867027	1.93813313	-2.833376172	0.004972	0.00783	-3.39121941
HMGN4	-0.304551906	6.99055217	-2.833328235	0.004973	0.00783	-3.39135106
ENPP1	-0.460254387	4.38561789	-2.833002358	0.004978	0.00784	-3.39224596
MYO9B	0.189291248	4.22111791	2.831157211	0.005006	0.00788	-3.39731118
UBASH3	-0.228309563	1.48936814	-2.83084595	0.005011	0.00789	-3.39816533
KRT8	-0.238536125	8.94893558	-2.829960521	0.005024	0.00791	-3.40059462
TSEN54	-0.162584676	4.63275282	-2.829391102	0.005033	0.00792	-3.40215652
SIGLEC1	-0.134168148	0.47346533	-2.828679998	0.005044	0.00794	-3.40410664
APOBEC	0.206019875	0.78496213	2.827937087	0.005055	0.00795	-3.40614349
MAMDC2	-0.331894697	2.01430571	-2.825642752	0.00509	0.00801	-3.41243075
LRRC58	-0.230887945	4.22180703	-2.825450983	0.005093	0.00801	-3.41295604
BAG4	-0.184122735	3.86265863	-2.825093997	0.005099	0.00802	-3.41393381
HSD3B7	0.2493268	3.53409881	2.824753437	0.005104	0.00803	-3.41486649
ZNF304	-0.204468137	3.44146188	-2.824661321	0.005105	0.00803	-3.41511874
RAB18	-0.223477189	5.79108211	-2.824550681	0.005107	0.00803	-3.41542171
TGS1	-0.205190274	3.8696191	-2.824155605	0.005113	0.00804	-3.41650348
NKX6-2	-0.018414494	0.02679843	-2.823486938	0.005124	0.00805	-3.41833404
SH3D19	-0.23722249	4.77879534	-2.822256498	0.005143	0.00808	-3.42170148
MAT1A	-0.224921901	0.56175454	-2.821818271	0.005149	0.00809	-3.42290047
LITAF	-0.231665355	6.94928642	-2.821815761	0.00515	0.00809	-3.42290734
ARHGAP	-0.324415306	2.12839595	-2.821455416	0.005155	0.0081	-3.42389312
CERS5	-0.180088134	4.48196945	-2.820726031	0.005166	0.00812	-3.42588809
NTRK2	-0.345683336	6.08393182	-2.818646138	0.005199	0.00817	-3.43157425
FREM3	0.103589217	0.10250899	2.817508008	0.005217	0.0082	-3.43468408
MED13	0.325403838	4.96217033	2.816583479	0.005232	0.00822	-3.4372094
LRRN3	-0.53728516	3.23487389	-2.816069373	0.00524	0.00823	-3.43861332
PI16	-0.548249792	1.9756553	-2.814643073	0.005262	0.00826	-3.44250701

RDH10	-0.254915392	3.26379269	-2.814173903	0.00527	0.00828	-3.44378741
ATG7	-0.143469451	3.09514514	-2.813902311	0.005274	0.00828	-3.44452851
CXCL11	0.5013682	1.55783636	2.81381693	0.005275	0.00828	-3.44476147
LFNG	0.251304441	2.94559465	2.812722638	0.005293	0.00831	-3.44774673
SLC23A2	-0.264399011	4.93881321	-2.811333786	0.005315	0.00834	-3.45153398
KIF23	0.174030191	0.91252407	2.811110814	0.005319	0.00835	-3.45214183
CENPL	-0.12487012	1.53740411	-2.810816474	0.005323	0.00835	-3.45294418
PORCN	-0.258096571	4.51441276	-2.810808671	0.005323	0.00835	-3.45296545
TIGD2	-0.191391222	3.38147307	-2.810590412	0.005327	0.00836	-3.45356036
CHERP	-0.189770845	4.48463401	-2.810505808	0.005328	0.00836	-3.45379095
IRF4	0.457510213	1.66873033	2.809689808	0.005341	0.00838	-3.45601466
RBM25	-0.225946281	4.63785252	-2.808195687	0.005365	0.00842	-3.46008476
TMEM15	-0.13656417	0.3841545	-2.807993362	0.005369	0.00842	-3.46063575
MTMR3	-0.16392842	2.22927543	-2.807825659	0.005371	0.00843	-3.46109243
H2AFJ	-0.225871604	5.60625223	-2.807806365	0.005372	0.00843	-3.46114497
TRPC3	-0.062205527	0.17335093	-2.807691306	0.005374	0.00843	-3.46145828
IFNG	0.244776373	0.55040104	2.80726243	0.005381	0.00844	-3.46262599
HTRA3	-0.514300999	3.41450847	-2.807088448	0.005383	0.00844	-3.46309966
MYORG	-0.214018856	2.02221998	-2.807080776	0.005384	0.00844	-3.46312054
ZNF660	-0.186076827	1.69012721	-2.807003997	0.005385	0.00844	-3.46332956
SERPINE	0.274296953	3.08253633	2.806037439	0.0054	0.00847	-3.46596041
TRABD2	0.291624795	1.54494413	2.805534914	0.005409	0.00848	-3.46732788
LDLRAD	-0.148774805	1.19253657	-2.805235283	0.005414	0.00848	-3.46814313
GUCY2C	-0.126968452	0.71119561	-2.804468979	0.005426	0.0085	-3.47022774
UBTD1	-0.185749123	5.78721302	-2.803158202	0.005447	0.00854	-3.47379227
ZDHHC5	-0.181072624	6.4122038	-2.802330669	0.005461	0.00856	-3.47604186
TBC1D1	-0.185622683	4.3084089	-2.802306005	0.005461	0.00856	-3.4761089
ALG11	-0.191541043	1.62688794	-2.802136104	0.005464	0.00856	-3.47657068
DPP10	-0.257018354	0.58548492	-2.800996682	0.005483	0.00859	-3.4796669
PIP5K1C	-0.151816328	5.19668852	-2.799846634	0.005502	0.00862	-3.48279078
ADAMTS	0.185691702	0.86083286	2.799078733	0.005515	0.00864	-3.48487597
PLIN3	-0.221203906	6.12178322	-2.798994851	0.005516	0.00864	-3.48510371
HEXB	-0.201529336	6.41342669	-2.798610593	0.005522	0.00865	-3.4861469
SMIM1	-0.2973292	5.10957895	-2.798310887	0.005527	0.00865	-3.48696046
WBP11	-0.196749337	5.49988252	-2.798099696	0.005531	0.00866	-3.4875337
CDCA4	0.185942815	3.81395505	2.797839081	0.005535	0.00866	-3.48824103
GPR12	0.062134419	0.06500147	2.796346734	0.00556	0.0087	-3.49229017
IRF8	0.372145769	2.0991919	2.79612689	0.005564	0.00871	-3.49288649
DDAH2	-0.237040911	7.3539378	-2.795741132	0.00557	0.00872	-3.49393275
THAP2	-0.142500019	2.08119199	-2.795069675	0.005581	0.00873	-3.49575357

MUC22	0.019999282	0.02439389	2.794207026	0.005596	0.00876	-3.49809224
ARL17A	-0.089676718	0.49319339	-2.793821796	0.005602	0.00876	-3.49913639
APBB1IP	0.347353327	2.29749294	2.792892198	0.005618	0.00879	-3.50165547
SUPT6H	-0.166281658	5.25257119	-2.792860372	0.005619	0.00879	-3.5017417
HDGFL2	-0.188911472	5.56450459	-2.792393694	0.005626	0.0088	-3.50300603
CBLN4	0.421659587	1.49545258	2.792383754	0.005627	0.0088	-3.50303295
TMEM51	0.220864309	4.13916383	2.791910669	0.005635	0.00881	-3.50431443
TTC7B	-0.162649083	1.73104482	-2.791663666	0.005639	0.00882	-3.50498342
FBXL4	-0.187451591	3.71257952	-2.791242471	0.005646	0.00883	-3.50612407
SLC26A1	-0.206254747	4.6368241	-2.790585387	0.005657	0.00884	-3.50790321
SOCS6	-0.205256949	3.66031239	-2.788557101	0.005691	0.0089	-3.51339258
RAPGEF	0.286514167	3.18885464	2.78850347	0.005692	0.0089	-3.51353768
EZR	-0.252932242	8.60628271	-2.787132207	0.005716	0.00893	-3.51724669
P3H3	0.322937958	5.1063997	2.787093856	0.005716	0.00893	-3.51735039
EFNA4	-0.237256929	4.71619897	-2.786787702	0.005722	0.00894	-3.51817824
TRIM15	0.025779695	0.03793768	2.786072496	0.005734	0.00896	-3.52011182
ABCC4	-0.367307449	2.08189029	-2.7859117	0.005737	0.00896	-3.52054648
MPEG1	0.337561451	3.09411974	2.785299802	0.005747	0.00898	-3.52220031
STEAP3	-0.27541432	4.03267993	-2.785211075	0.005749	0.00898	-3.52244009
MADCAM	-0.192397422	0.66882468	-2.785129806	0.00575	0.00898	-3.52265971
SKIV2L	-0.160130637	4.93946722	-2.784887882	0.005754	0.00899	-3.52331345
UBAP1L	-0.208143819	1.44443336	-2.78477113	0.005756	0.00899	-3.52362892
MINAR1	0.084981503	0.53615124	2.784623866	0.005759	0.00899	-3.52402683
NRBP1	-0.158866163	5.93198552	-2.78356526	0.005777	0.00902	-3.52688655
HIST1H2	-0.089944024	0.19494014	-2.783441777	0.005779	0.00902	-3.52722007
HBA2	-0.533198371	4.32906364	-2.78264607	0.005793	0.00904	-3.52936883
P4HA3	-0.246929134	1.88733523	-2.782343159	0.005798	0.00905	-3.53018668
GRIN2A	0.151522427	0.46114165	2.782315385	0.005799	0.00905	-3.53026166
RHNO1	-0.173320899	5.31950122	-2.781514494	0.005813	0.00907	-3.5324236
MS4A4A	0.330896155	2.74433126	2.781208927	0.005818	0.00908	-3.5332483
TRIM59	0.181433744	1.53528662	2.780692239	0.005827	0.00909	-3.53464261
PLCG2	-0.210159698	2.32222052	-2.779615534	0.005846	0.00912	-3.53754735
ASTN2	-0.199680656	1.2805579	-2.77871671	0.005861	0.00914	-3.5399714
NHLRC2	-0.186055362	3.62526434	-2.778404313	0.005867	0.00915	-3.54081373
SMIM17	0.129493469	0.64140878	2.777389496	0.005885	0.00918	-3.54354942
NCKAP5	-0.176707422	3.52424956	-2.77691224	0.005893	0.00919	-3.54483566
PRRC2C	0.218386498	4.86546291	2.776862524	0.005894	0.00919	-3.54496964
MANEA	-0.256260478	3.359788	-2.776337278	0.005903	0.00921	-3.54638495
WFIKKN1	-0.125753836	0.28207137	-2.776076229	0.005908	0.00921	-3.54708827
PPEF1	0.143079829	0.51332422	2.776039923	0.005908	0.00921	-3.54718608

OR2B6	-0.110579405	0.28127852	-2.775645924	0.005915	0.00922	-3.54824746
SAMD9	0.285722383	2.64042545	2.775569906	0.005917	0.00922	-3.54845223
DNAJC24	-0.173037909	1.94046249	-2.775047314	0.005926	0.00924	-3.54985977
CCDC148	0.275071498	2.02303456	2.775033039	0.005926	0.00924	-3.54989821
CD19	0.374863075	0.72938176	2.77414969	0.005942	0.00926	-3.55227683
ADAMTS	0.221561454	1.06164337	2.77403155	0.005944	0.00926	-3.5525949
CD276	0.259937453	5.08669118	2.773757397	0.005949	0.00927	-3.55333294
CD320	-0.235824593	6.59417132	-2.772836102	0.005965	0.00929	-3.55581265
PYHIN1	0.251939621	0.78753112	2.772295684	0.005975	0.00931	-3.55726685
CLEC4E	-0.311061853	1.44349837	-2.772238334	0.005976	0.00931	-3.55742116
SLFN12	-0.222658553	2.81018046	-2.772087305	0.005979	0.00931	-3.5578275
NFKBID	0.23781367	2.63414434	2.77202725	0.00598	0.00931	-3.55798908
LAT	0.090987314	0.38034203	2.771747396	0.005985	0.00932	-3.55874195
TEPSIN	-0.196012683	2.87656583	-2.771189181	0.005995	0.00933	-3.56024348
EXOC1	-0.164480232	4.50223439	-2.770842137	0.006001	0.00934	-3.56117685
NLGN1	-0.076811334	0.15139961	-2.769726528	0.006021	0.00937	-3.56417649
PKN2	-0.233866607	4.5052557	-2.769368495	0.006027	0.00938	-3.56513893
DTX3L	0.216964258	5.07084241	2.767749897	0.006056	0.00943	-3.56948845
IFI27L2	-0.295155054	4.92332397	-2.767228273	0.006066	0.00944	-3.57088966
IFNAR1	-0.182906647	5.5638516	-2.767101706	0.006068	0.00944	-3.57122961
SCLY	-0.083555879	0.71861644	-2.765554073	0.006096	0.00949	-3.57538528
PRMT8	0.488918191	1.04578557	2.765476176	0.006097	0.00949	-3.57559439
XPNPEP	0.143055544	0.34505457	2.764744824	0.006111	0.00951	-3.57755738
HORMAD	0.053671843	0.15012053	2.763745528	0.006129	0.00953	-3.58023877
EDRF1	-0.181148045	2.65637625	-2.762978105	0.006143	0.00956	-3.58229736
ACADL	-0.339521264	3.70412412	-2.7625919	0.00615	0.00957	-3.58333314
CHKB-CP	0.196918724	1.06216809	2.761653405	0.006167	0.00959	-3.58584955
RAI14	0.275057341	3.59679606	2.761597778	0.006168	0.00959	-3.58599868
LRFN2	-0.182285017	0.25403642	-2.761517678	0.00617	0.00959	-3.58621342
ZDHHC1	-0.156213421	2.77676521	-2.759773001	0.006202	0.00964	-3.59088912
VPS53	-0.151456935	3.20174442	-2.759618507	0.006205	0.00965	-3.59130303
APBA1	-0.17467913	1.0391073	-2.759268828	0.006211	0.00965	-3.59223978
TRPV1	0.04684432	0.1414698	2.759225928	0.006212	0.00966	-3.59235469
DENND4	-0.22054704	3.39917996	-2.758478172	0.006226	0.00968	-3.59435744
ATP6V1A	-0.237444157	6.34707733	-2.757998042	0.006234	0.00969	-3.59564312
HNRNPA	-0.194908357	8.24618555	-2.757545818	0.006243	0.0097	-3.59685388
IL13RA2	-0.225546835	0.87690348	-2.756926474	0.006254	0.00972	-3.59851178
SMYD5	-0.151012738	4.88188498	-2.756890062	0.006255	0.00972	-3.59860924
CCNT1	-0.207540732	3.84364219	-2.755783804	0.006276	0.00975	-3.60156964
MMP23B	0.044776105	0.10567757	2.754504058	0.006299	0.00979	-3.60499289

PROK1	-0.158049775	0.39944343	-2.754377099	0.006302	0.00979	-3.60533242
LDHC	-0.214062061	0.38689673	-2.754192899	0.006305	0.00979	-3.605825
APOD	0.576252608	3.57072954	2.754090724	0.006307	0.00979	-3.60609822
HRH4	0.034646354	0.08221065	2.753499375	0.006318	0.00981	-3.60767932
NLRC4	0.160324405	1.25005085	2.752794628	0.006331	0.00983	-3.6095632
NUP43	-0.144425318	4.5430533	-2.752748703	0.006332	0.00983	-3.60968595
DAGLA	0.222452205	2.14874407	2.752497545	0.006337	0.00984	-3.6103572
KLHL36	-0.193847688	4.62897983	-2.752132863	0.006344	0.00985	-3.61133176
PRDM4	-0.190307939	4.39033623	-2.751972868	0.006347	0.00985	-3.61175929
SCML4	0.137968739	0.33595745	2.751446493	0.006357	0.00987	-3.61316566
SENP5	-0.144320344	3.70189662	-2.750304441	0.006378	0.0099	-3.61621614
POU5F1	-0.061855751	0.38759822	-2.750089253	0.006382	0.0099	-3.61679078
FURIN	-0.226268183	7.15060865	-2.749532611	0.006393	0.00992	-3.61827705
ANKRD6	0.325860674	2.9251188	2.74910361	0.006401	0.00993	-3.61942232
NPB	-0.018511527	0.03311966	-2.749053742	0.006402	0.00993	-3.61955544
ZNF625	-0.091647253	0.51814905	-2.748701557	0.006409	0.00994	-3.6204955
FAM122(	-0.096132602	1.19712347	-2.748116003	0.00642	0.00996	-3.62205822
LARP4	-0.19832305	4.02926526	-2.748096273	0.00642	0.00996	-3.62211087
CCL3L1	-0.381927232	1.55759107	-2.747826315	0.006425	0.00996	-3.62283122
SPARC	-0.275827236	9.33515435	-2.747602157	0.00643	0.00997	-3.62342931
TLR2	0.291793117	3.35365543	2.747539915	0.006431	0.00997	-3.62359537
SEC23A	0.253163437	4.03350913	2.747390295	0.006434	0.00997	-3.62399454
YTHDF3	-0.210981122	4.92675442	-2.746854475	0.006444	0.00999	-3.6254239
KCNH3	-0.319695165	2.02094624	-2.746657074	0.006448	0.00999	-3.62595043
TTC31	-0.157136522	3.74981112	-2.746595587	0.006449	0.00999	-3.62611442
OPN5	0.022752742	0.05007645	2.746111743	0.006458	0.01001	-3.62740479
KCNMB1	0.172512783	1.24785233	2.746075838	0.006459	0.01001	-3.62750054
GIMAP2	-0.276552868	3.00733556	-2.745965458	0.006461	0.01001	-3.62779488
KRT1	0.078873822	0.14146224	2.745485928	0.00647	0.01002	-3.62907346
KIAA114	-0.181479755	4.19286063	-2.745200981	0.006475	0.01003	-3.62983313
STEAP2	-0.293875635	1.62696492	-2.74494423	0.00648	0.01004	-3.63051756
USP9X	-0.227424796	4.98560136	-2.744748819	0.006484	0.01004	-3.63103843
STAU2	-0.172674317	3.49673602	-2.743977924	0.006499	0.01006	-3.63309293
ZNF251	-0.219525563	4.12348121	-2.742795279	0.006522	0.0101	-3.63624372
PDPN	0.320508037	1.49267885	2.742592958	0.006526	0.0101	-3.63678262
POLRMT	-0.14868782	4.80401013	-2.742543464	0.006527	0.0101	-3.63691444
RPRD2	-0.233565205	5.05246546	-2.741757221	0.006542	0.01013	-3.63900825
KIAA132	-0.135148587	1.49533213	-2.740600296	0.006564	0.01016	-3.64208818
CASP6	-0.179375865	4.5470587	-2.739411419	0.006587	0.0102	-3.6452519
SNX8	0.183300176	4.63395825	2.739291756	0.00659	0.0102	-3.64557026

TRIM31	-0.122711276	0.47387582	-2.738744078	0.0066	0.01021	-3.64702719
NRP1	-0.267129503	4.82177016	-2.738608682	0.006603	0.01022	-3.64738733
DENND5	-0.228780016	2.01575554	-2.737723119	0.00662	0.01024	-3.6497424
HDAC10	-0.174856715	2.11367096	-2.737631026	0.006622	0.01024	-3.64998728
ATXN1L	-0.245217899	4.04924222	-2.737606853	0.006622	0.01024	-3.65005155
PRR7	-0.271420925	2.11552877	-2.737468907	0.006625	0.01025	-3.65041833
CBX2	-0.170932669	1.56217643	-2.737392029	0.006627	0.01025	-3.65062272
CA5B	-0.184127798	2.21285697	-2.737141785	0.006632	0.01026	-3.65128802
SERPINE	-0.037870894	0.1522161	-2.737004739	0.006634	0.01026	-3.65165234
7-Mar	-0.205613151	5.21627293	-2.73679338	0.006638	0.01026	-3.65221419
PEAK1	0.206334782	2.32388374	2.736444176	0.006645	0.01027	-3.65314237
NAAA	-0.227288823	3.62931067	-2.736225631	0.00665	0.01028	-3.6537232
TMEM68	-0.15001029	3.08190704	-2.735600549	0.006662	0.0103	-3.65538427
EPSTI1	0.339030113	2.88909463	2.735084982	0.006672	0.01031	-3.65675404
NHLRC3	-0.194639871	4.12258448	-2.734398005	0.006685	0.01033	-3.65857884
RAMP1	0.602703967	3.24538902	2.734024575	0.006693	0.01034	-3.65957059
NSMF	-0.254397014	4.5704308	-2.733928733	0.006695	0.01035	-3.6598251
HAS2	0.408266767	1.19532471	2.732870019	0.006716	0.01038	-3.66263604
FCRL1	0.179300818	0.24988375	2.732544589	0.006722	0.01039	-3.66349986
MLKL	-0.178003717	2.34996614	-2.73198013	0.006733	0.0104	-3.66499794
FEM1A	-0.09895191	0.84390652	-2.731837299	0.006736	0.01041	-3.66537697
KLF13	-0.267219732	5.29852023	-2.731808301	0.006737	0.01041	-3.66545392
TBC1D8I	-0.218981737	2.52861037	-2.731731888	0.006738	0.01041	-3.66565668
SLC16A9	-0.385650007	5.11119777	-2.731129744	0.00675	0.01042	-3.66725432
TMED7-T	-0.049612499	0.24887246	-2.73092272	0.006754	0.01043	-3.66780352
ZNF10	-0.232707943	3.67964921	-2.728731299	0.006798	0.0105	-3.67361467
PRPF40A	-0.203007795	4.839606	-2.72782758	0.006816	0.01052	-3.67600985
MYO1D	0.310140765	6.32360308	2.727194077	0.006829	0.01054	-3.67768841
MOB3A	-0.168550642	5.11846892	-2.726624833	0.00684	0.01056	-3.67919639
GRK3	-0.207359794	2.91381908	-2.725945667	0.006854	0.01058	-3.68099518
CLDN2	0.568986722	2.45235115	2.725719428	0.006859	0.01058	-3.68159429
SLC35A3	-0.2522209	4.38169022	-2.72474521	0.006878	0.01061	-3.68417359
TUSC3	0.502235981	4.78745235	2.724322187	0.006887	0.01063	-3.6852933
NFIL3	-0.328400865	4.50040166	-2.723573164	0.006902	0.01065	-3.6872755
CHAF1B	0.199084718	2.26902546	2.723132826	0.006911	0.01066	-3.68844057
MYO3A	0.158319683	0.83195214	2.722947451	0.006915	0.01067	-3.68893098
ZNF468	-0.254301352	3.46326959	-2.722945678	0.006915	0.01067	-3.68893568
DHX8	-0.171251807	4.06821696	-2.722203178	0.00693	0.01069	-3.69089968
SEC24D	-0.28626211	4.28573692	-2.721706644	0.00694	0.0107	-3.69221279
SYN2	-0.136441655	0.24142893	-2.72133365	0.006948	0.01071	-3.69319905

GPRC5A	0.270262816	7.05552887	2.720503627	0.006965	0.01074	-3.69539331
KCNMA1	0.162951184	0.8107125	2.720376487	0.006967	0.01074	-3.69572936
TIMM29	-0.140250348	4.57931753	-2.720196832	0.006971	0.01075	-3.69620419
CNTNAP	-0.257844243	0.42071609	-2.719249384	0.00699	0.01078	-3.69870783
C3AR1	0.368590267	3.22174448	2.717044166	0.007036	0.01084	-3.70453196
KLHL42	-0.200489631	3.44535304	-2.716995462	0.007037	0.01085	-3.70466054
CD83	-0.265668239	4.54112085	-2.716705744	0.007043	0.01085	-3.70542536
C7	0.680975445	3.06143361	2.716483889	0.007047	0.01086	-3.70601098
DSE	0.197838824	2.05291032	2.716267767	0.007052	0.01087	-3.70658142
SLCO3A1	-0.172146337	3.94043096	-2.715838959	0.007061	0.01088	-3.70771311
AMPH	0.096053569	0.36229106	2.715379866	0.00707	0.01089	-3.70892453
IGLL1	0.230390486	0.38877313	2.714704952	0.007084	0.01091	-3.7107051
SMG5	-0.173467408	5.9606553	-2.712961433	0.007121	0.01097	-3.71530296
FMNL2	0.327874525	5.34979898	2.712626651	0.007128	0.01098	-3.71618549
SERPINE	0.284355193	3.47488175	2.712221973	0.007136	0.01099	-3.71725215
IFNGR2	-0.19294578	6.86651431	-2.71174241	0.007146	0.011	-3.718516
MYH14	0.227601554	6.67170643	2.711599097	0.007149	0.01101	-3.71889365
DRD4	-0.225082379	1.20697522	-2.710866407	0.007164	0.01103	-3.72082408
ZNF410	-0.098502566	0.93246136	-2.710149206	0.007179	0.01105	-3.72271323
FCGR3A	0.427467885	4.45940724	2.709648171	0.00719	0.01107	-3.72403271
FBXO17	-0.145061921	0.80514044	-2.708585575	0.007212	0.0111	-3.7268303
LY6G6F-	-0.07367765	0.12078965	-2.708131045	0.007222	0.01111	-3.72802666
PHF10	-0.190452993	5.49498517	-2.707834449	0.007228	0.01112	-3.72880722
RAD18	0.172679968	2.74169169	2.707674327	0.007232	0.01113	-3.72922859
CEMIP	0.3536595	1.68545339	2.707421781	0.007237	0.01113	-3.72989312
SSH2	-0.183400252	3.03256073	-2.707226449	0.007241	0.01114	-3.73040707
HES7	-0.17240167	0.63323268	-2.707113477	0.007244	0.01114	-3.7307043
CERKL	0.279679544	1.663886	2.706891021	0.007248	0.01115	-3.73128955
FBLN2	0.402352162	4.26662975	2.706865958	0.007249	0.01115	-3.73135548
PHACTR	-0.252668859	3.90423761	-2.705370934	0.007281	0.0112	-3.73528744
IRF1	0.295731791	3.87840614	2.705300216	0.007282	0.0112	-3.73547338
ZFAND5	-0.196154753	6.26047705	-2.704837478	0.007292	0.01121	-3.73668995
RNF217	-0.206461456	2.80719888	-2.704161693	0.007306	0.01123	-3.73846629
GPHN	-0.210175506	3.76822867	-2.703109497	0.007329	0.01127	-3.7412312
MEFV	0.071304041	0.2701779	2.702171184	0.007349	0.0113	-3.743696
RXRG	0.799922693	5.5475222	2.700595388	0.007383	0.01135	-3.74783354
SESN3	-0.300885518	4.25331015	-2.700422945	0.007387	0.01135	-3.74828619
PURG	-0.033379437	0.11662153	-2.699132911	0.007415	0.01139	-3.75167151
CUZD1	0.116299204	0.76878091	2.698887946	0.00742	0.0114	-3.75231418
FOXD2	-0.090291611	0.59157457	-2.698227356	0.007434	0.01142	-3.75404697

CYB5R2	-0.159188596	1.65710175	-2.697488078	0.00745	0.01145	-3.75598569
ST8SIA5	-0.101144352	0.15359538	-2.697428356	0.007452	0.01145	-3.75614228
BBS1	-0.133101215	1.36426429	-2.697402363	0.007452	0.01145	-3.75621044
GPR137I	-0.224097239	5.07482267	-2.697302199	0.007454	0.01145	-3.75647307
CYP2U1	-0.198080823	2.16875925	-2.696962414	0.007462	0.01146	-3.75736391
ZNF865	-0.157155843	4.38718208	-2.696945051	0.007462	0.01146	-3.75740943
SERTAD	0.245360254	4.56961113	2.696766034	0.007466	0.01146	-3.75787873
RNASE6	0.389379424	3.84769232	2.696566673	0.00747	0.01147	-3.75840132
LYSMD4	-0.137987565	2.08740233	-2.695665759	0.00749	0.0115	-3.76076249
RNASE1	-0.056848795	0.15169784	-2.694550096	0.007514	0.01154	-3.76368544
METTL1(	-0.173016745	4.12474166	-2.693788883	0.007531	0.01156	-3.7656791
CCM2	0.181504868	4.5201727	2.693580424	0.007536	0.01157	-3.76622498
ADGRB2	-0.313941577	3.15528853	-2.693372545	0.00754	0.01157	-3.7667693
PPIL3	-0.20764799	4.28458248	-2.692460273	0.00756	0.0116	-3.76915754
SAMD3	0.116554843	0.37971823	2.692212848	0.007566	0.01161	-3.76980515
KLHL35	-0.167242696	1.00015622	-2.691193837	0.007588	0.01164	-3.77247169
NET1	0.291648337	5.48654843	2.690987372	0.007593	0.01165	-3.77301186
GATA3	0.352745499	3.20033934	2.690718885	0.007599	0.01166	-3.77371422
ORAI3	-0.189374912	5.08370832	-2.689911719	0.007617	0.01168	-3.77582539
TRPA1	0.016992109	0.02304478	2.689452423	0.007627	0.0117	-3.77702642
AP1S3	-0.275412443	3.25431495	-2.688936575	0.007638	0.01171	-3.7783751
CARD19	-0.171537163	4.38761508	-2.687664287	0.007667	0.01176	-3.78170044
PDGFRB	-0.338090783	4.93338342	-2.686470399	0.007693	0.0118	-3.78481953
UBE2H	-0.183200576	6.33970146	-2.685778315	0.007709	0.01182	-3.78662702
SLC22A1	0.033707503	0.05932335	2.685689528	0.007711	0.01182	-3.78685887
FMN2	-0.16497381	0.24382558	-2.685668109	0.007711	0.01182	-3.7869148
ZNF534	-0.128431428	0.31996879	-2.685222854	0.007721	0.01183	-3.78807739
AGAP1	-0.223570983	3.33714869	-2.684690957	0.007733	0.01185	-3.78946596
CES1	-0.571523814	2.44616283	-2.683048714	0.00777	0.01191	-3.79375156
CFAP10(	-0.040625495	0.08572197	-2.682913568	0.007773	0.01191	-3.79410413
PLA2G4I	-0.032488378	0.02634344	-2.682759691	0.007777	0.01192	-3.79450554
PM20D1	-0.180947313	0.51028045	-2.682523942	0.007782	0.01192	-3.79512048
DNAH3	-0.020646459	0.04571536	-2.681500368	0.007805	0.01196	-3.79778986
PLXNB1	0.237050375	5.7599551	2.681395577	0.007808	0.01196	-3.79806309
FAM118/	-0.234546687	2.89806119	-2.68027548	0.007833	0.012	-3.80098297
CPB2	-0.125695694	0.32396295	-2.680172848	0.007835	0.012	-3.80125045
IL10RA	0.318053922	2.83143866	2.679692156	0.007846	0.01202	-3.80250313
RADX	-0.17931094	1.62265958	-2.679382938	0.007853	0.01203	-3.80330883
CLEC4G	0.099280513	0.23642847	2.678277305	0.007879	0.01206	-3.80618897
PPM1M	-0.205260372	4.5549086	-2.676555327	0.007918	0.01212	-3.81067244

CEP162	-0.171649653	2.81307329	-2.675709926	0.007938	0.01215	-3.81287259
FAM122E	-0.187039106	4.64948303	-2.675635626	0.007939	0.01215	-3.81306592
RELN	-0.146109352	0.29441444	-2.675582325	0.007941	0.01215	-3.81320461
ARG1	-0.063415231	0.32632126	-2.674851443	0.007957	0.01218	-3.81510611
TBC1D1(	-0.214300119	3.53097441	-2.674707812	0.007961	0.01218	-3.81547973
XRCC2	0.113069099	0.77466748	2.674456973	0.007967	0.01219	-3.81613218
TTBK2	-0.141272528	2.085291	-2.674228789	0.007972	0.0122	-3.81672565
BST2	-0.383041813	6.08933095	-2.67421501	0.007972	0.0122	-3.81676148
NOP9	-0.178953336	4.32217865	-2.674120784	0.007974	0.0122	-3.81700654
ZNF195	-0.14816422	3.44531521	-2.673913645	0.007979	0.0122	-3.81754521
TUBA3E	-0.083224796	0.10828434	-2.673105088	0.007998	0.01223	-3.81964752
GAREM1	-0.256608595	1.97066139	-2.672840459	0.008004	0.01224	-3.82033545
IDH1	-0.235839695	5.46441424	-2.672427369	0.008014	0.01225	-3.82140918
LMBRD1	-0.245508826	7.33491692	-2.672425111	0.008014	0.01225	-3.82141505
ZNF143	-0.165501054	3.11667835	-2.672342907	0.008016	0.01225	-3.8216287
FUT7	0.215053669	0.77659209	2.671197982	0.008042	0.01229	-3.82460376
SLC11A2	-0.199858028	4.99596439	-2.671018351	0.008046	0.0123	-3.82507042
MYB	0.133306321	0.6074817	2.671011716	0.008046	0.0123	-3.82508766
LPO	-0.083236621	0.08968406	-2.670749734	0.008053	0.01231	-3.8257682
S100A13	-0.262529242	8.19073429	-2.670260932	0.008064	0.01232	-3.82703777
GPR173	0.301803208	1.54758576	2.669722321	0.008077	0.01234	-3.82843646
USP1	-0.22468625	4.67218152	-2.669312068	0.008086	0.01235	-3.82950164
B3GNT2	-0.211605021	5.14101019	-2.669081286	0.008092	0.01236	-3.83010078
PPP1R2I	-0.087084698	0.35933895	-2.66879075	0.008098	0.01237	-3.83085498
ZNF318	-0.219979025	3.51154791	-2.668459225	0.008106	0.01238	-3.83171548
ANKRD2	-0.03193434	0.07652076	-2.667782375	0.008122	0.01241	-3.83347199
SCAMP1	-0.213732154	5.38616837	-2.66730566	0.008133	0.01242	-3.83470887
POLD1	0.193015232	4.00824546	2.665990265	0.008164	0.01247	-3.8381207
SLC16A1	0.41517965	2.03238934	2.665758865	0.00817	0.01247	-3.83872073
AMDHD2	-0.172395994	3.51069448	-2.665756845	0.00817	0.01247	-3.83872597
GMEB1	-0.162569991	3.19421379	-2.665138812	0.008184	0.01249	-3.84032832
RALA	-0.149840639	5.09822464	-2.664394962	0.008202	0.01252	-3.84225641
CSRNP3	-0.272211403	1.95954554	-2.663720141	0.008218	0.01254	-3.84400513
ASPM	0.133805329	0.52099673	2.663562729	0.008222	0.01255	-3.84441298
COL22A1	0.13278974	0.52514666	2.662387467	0.00825	0.01259	-3.84745737
PNPLA7	-0.230921789	3.13207374	-2.662052061	0.008258	0.0126	-3.84832597
CYFIP1	-0.194140021	5.14052852	-2.661494557	0.008271	0.01262	-3.8497695
SPDEF	0.117246069	0.34262012	2.661269743	0.008276	0.01263	-3.85035153
FERMT2	-0.21421603	4.31294878	-2.661251183	0.008277	0.01263	-3.85039958
ASGR2	0.12207275	0.48285759	2.660438223	0.008296	0.01266	-3.85250386

PPP1R32	-0.16629514	1.84754071	-2.659926052	0.008309	0.01267	-3.85382927
CHRNA9	-0.158902389	0.36180303	-2.659158208	0.008327	0.0127	-3.85581586
RO60	-0.233085023	3.26119274	-2.65851445	0.008343	0.01272	-3.85748099
ACIN1	-0.180427635	5.89094818	-2.657407905	0.008369	0.01276	-3.86034227
MTUS2	-0.247497397	0.64707848	-2.657042626	0.008378	0.01277	-3.86128655
TNFRSF1	0.487826695	1.17570361	2.656786556	0.008384	0.01278	-3.86194844
LAMA3	0.190045823	0.72214134	2.656010654	0.008403	0.01281	-3.86395363
CXCR3	0.368217461	2.22540035	2.652726867	0.008483	0.01293	-3.87243391
NAA25	-0.173033165	2.75855401	-2.652645926	0.008485	0.01293	-3.87264281
RSPO3	0.206246997	0.72910568	2.651395021	0.008516	0.01298	-3.87587053
PIP4K2A	0.207440273	4.38677928	2.65134058	0.008517	0.01298	-3.87601097
SSTR3	0.306688418	1.09403119	2.651286098	0.008518	0.01298	-3.87615151
TUBGCP	-0.167186463	3.47481099	-2.651077221	0.008523	0.01299	-3.87669032
TKTL1	-0.025867105	0.05714105	-2.650932554	0.008527	0.01299	-3.87706347
RFX5	-0.198301292	5.26690904	-2.650789071	0.00853	0.01299	-3.87743354
HPDL	-0.313421143	1.46997963	-2.650491962	0.008538	0.013	-3.8781998
GDPD5	-0.140266977	1.28531801	-2.650231477	0.008544	0.01301	-3.87887153
ATL1	-0.191968198	1.93432626	-2.648797808	0.00858	0.01307	-3.88256753
CAP1	-0.17972704	7.84188314	-2.648606344	0.008584	0.01307	-3.88306098
NMT2	-0.153453877	2.86852598	-2.648401702	0.008589	0.01308	-3.88358836
SOD2	0.2597603	4.8093965	2.647830896	0.008603	0.0131	-3.88505916
KRT4	-0.084075595	0.10399125	-2.647601163	0.008609	0.01311	-3.88565103
WDR36	-0.183874079	3.62692233	-2.647446588	0.008613	0.01311	-3.88604924
ACER2	0.36754252	2.33592295	2.646623419	0.008633	0.01314	-3.88816949
SCN11A	-0.044210503	0.16846191	-2.645756208	0.008655	0.01317	-3.8904025
CCN2	-0.514947686	8.23087793	-2.645749802	0.008655	0.01317	-3.89041899
CEBPB	-0.202988104	6.43050906	-2.645690323	0.008657	0.01317	-3.89057212
GRM7	0.02394503	0.03859594	2.645429388	0.008663	0.01318	-3.89124386
ISL2	0.063260571	0.09255828	2.64538551	0.008664	0.01318	-3.89135681
WARS	0.382986212	7.11147768	2.645268008	0.008667	0.01319	-3.89165928
EIF4G1	-0.199290196	6.90743969	-2.645074637	0.008672	0.01319	-3.89215702
TUBGCP	-0.146355389	2.20108322	-2.644578391	0.008684	0.01321	-3.8934342
ITPRID2	-0.260029553	5.29693005	-2.644456019	0.008687	0.01321	-3.89374911
PHF6	-0.194117472	3.6288834	-2.644111404	0.008696	0.01322	-3.89463586
DERA	-0.176572417	5.52060442	-2.642109014	0.008746	0.0133	-3.89978621
BARX1	-0.321027724	0.72872519	-2.64089695	0.008777	0.01334	-3.90290196
UBE3C	-0.208037128	4.26161014	-2.640776781	0.00878	0.01335	-3.9032108
SENP1	-0.166232082	3.06581892	-2.64019675	0.008794	0.01337	-3.90470129
KIAA167	-0.300391641	4.35430958	-2.639450056	0.008813	0.0134	-3.9066196
ZSCAN2	-0.161746841	2.26439196	-2.639297925	0.008817	0.0134	-3.90701038

CLSPN	0.150939795	0.75392188	2.637814464	0.008855	0.01346	-3.91081976
CAPN9	-0.200020671	0.39502585	-2.636776211	0.008881	0.0135	-3.91348469
AVPI1	0.219407415	4.99331703	2.634450128	0.008941	0.01359	-3.91945153
HTR4	0.023786883	0.03429366	2.634179884	0.008947	0.01359	-3.92014444
HMGXB3	0.1577143	4.76821751	2.634081269	0.00895	0.0136	-3.92039727
FBXL18	-0.16861844	1.5823686	-2.634052201	0.008951	0.0136	-3.92047179
ABI2	-0.216245265	3.76298538	-2.633627452	0.008962	0.01361	-3.92156066
ARL17B	-0.114231481	0.5807835	-2.632943832	0.008979	0.01364	-3.92331281
TNRC6B	-0.183098871	2.95381587	-2.632478424	0.008991	0.01366	-3.92450543
DRD2	0.036679676	0.06081255	2.632334114	0.008995	0.01366	-3.92487518
TARBP1	-0.201103638	3.53526338	-2.632257306	0.008997	0.01366	-3.92507197
RAB7B	0.363766728	3.41207502	2.63153604	0.009015	0.01369	-3.92691969
MEGF11	-0.220711355	0.30429935	-2.631279741	0.009022	0.0137	-3.92757615
FAM163B	0.09221182	0.18154715	2.631279257	0.009022	0.0137	-3.92757739
GPR19	0.096039482	0.46827352	2.63103376	0.009028	0.0137	-3.92820613
IGFBP3	-0.456394821	5.5522048	-2.630909914	0.009032	0.01371	-3.92852329
GLRX	-0.217950935	3.07984101	-2.630735195	0.009036	0.01371	-3.9289707
TRIP12	-0.211485332	5.31199019	-2.629844535	0.009059	0.01375	-3.93125105
ARHGAP	0.238907705	3.95777279	2.629225393	0.009075	0.01377	-3.9328358
MRVI1	-0.221669818	2.26321794	-2.629139326	0.009078	0.01377	-3.93305606
MYO18A	-0.241531169	3.45614553	-2.628409849	0.009097	0.0138	-3.93492271
CMTM5	-0.033771594	0.0867703	-2.627712486	0.009115	0.01383	-3.93670672
IFI44	-0.268987703	4.596022	-2.627190983	0.009128	0.01385	-3.93804056
PKD2L2	-0.035246159	0.14857798	-2.626897371	0.009136	0.01386	-3.93879141
PRPF8	-0.197184473	6.31696529	-2.626721232	0.009141	0.01386	-3.93924181
SELP	0.353313012	2.92454467	2.626458286	0.009147	0.01387	-3.93991413
IZUMO1R	-0.119566373	0.31024469	-2.626409263	0.009149	0.01387	-3.94003947
CAMK4	0.112106455	0.40080687	2.625988618	0.00916	0.01389	-3.94111485
C5orf24	-0.227393171	5.19218098	-2.622459843	0.009253	0.01403	-3.95012978
LRRC69	-0.138804401	0.75953048	-2.620900172	0.009294	0.01409	-3.95411059
MTOR	-0.191549437	3.95468085	-2.62058036	0.009302	0.0141	-3.95492659
TIGAR	-0.213693361	3.11542597	-2.620336349	0.009309	0.01411	-3.95554911
GABRB1	-0.058272045	0.10384959	-2.620165646	0.009313	0.01411	-3.95598458
KIF14	0.086453902	0.3295118	2.618607603	0.009355	0.01418	-3.95995794
GLIPR1	0.227366113	2.52798089	2.618323662	0.009362	0.01419	-3.96068182
CST4	0.533594156	1.63922263	2.618062644	0.009369	0.0142	-3.96134718
KCNIP1	-0.150888453	0.22347154	-2.616887737	0.009401	0.01424	-3.96434139
MYO3B	0.215548157	2.00743184	2.616688407	0.009406	0.01425	-3.96484925
ADGRG5	0.25039871	1.11220269	2.616345067	0.009415	0.01426	-3.96572394
ZBTB37	-0.156825337	1.81356664	-2.616293615	0.009417	0.01426	-3.96585501

TPRN	-0.164263742	3.31280925	-2.61625904	0.009418	0.01426	-3.96594308
PAK5	-0.035445296	0.07027906	-2.615804274	0.00943	0.01428	-3.96710143
PFN4	-0.161566068	1.9947451	-2.61441479	0.009467	0.01433	-3.97063945
TK2	-0.161121797	4.76850332	-2.613762532	0.009485	0.01436	-3.97229967
M1AP	-0.340564159	2.28143067	-2.61364422	0.009488	0.01436	-3.97260077
SPTLC1	-0.301470069	5.53173425	-2.613230661	0.009499	0.01438	-3.97365317
MAK16	-0.154562973	3.62575766	-2.61300878	0.009505	0.01439	-3.97421773
CHGB	0.604633436	2.08298253	2.612085137	0.00953	0.01442	-3.9765674
ECT2L	0.112865173	0.62769825	2.611718515	0.00954	0.01444	-3.97749984
C17orf10	0.325377046	2.77192009	2.611061651	0.009558	0.01446	-3.97917015
HPCAL4	0.519220166	2.43498689	2.610935417	0.009562	0.01447	-3.97949109
MRAP2	-0.128595208	0.30983112	-2.609671274	0.009596	0.01452	-3.98270435
ZNF549	-0.149519243	2.15243586	-2.609460647	0.009602	0.01453	-3.98323959
CCDC57	-0.191728154	2.89027333	-2.60862402	0.009625	0.01456	-3.98536519
PHF7	-0.140697034	2.21340087	-2.608163454	0.009637	0.01458	-3.98653506
SIPA1L3	-0.209727027	3.9540136	-2.608118144	0.009639	0.01458	-3.98665015
PLCB1	-0.163897342	1.13022643	-2.606493603	0.009683	0.01464	-3.99077498
ADGRF5	-0.34320501	5.36166226	-2.606216515	0.009691	0.01465	-3.99147829
SEMA6C	-0.184198604	1.85927074	-2.605585951	0.009708	0.01468	-3.99307852
A2ML1	-0.040126951	0.08097668	-2.605432704	0.009713	0.01468	-3.99346737
TTC24	0.094221028	0.18324947	2.605311684	0.009716	0.01469	-3.99377443
SIGLEC1	0.245085201	1.73574301	2.60500567	0.009724	0.0147	-3.99455082
FUT9	0.132900189	0.16155428	2.604990253	0.009725	0.0147	-3.99458993
SFTPA2	0.49016526	1.0666687	2.604539797	0.009737	0.01472	-3.99573262
ZNF687	-0.21286403	4.51921057	-2.603892917	0.009755	0.01474	-3.99737325
SP4	-0.217565789	3.40319628	-2.602910707	0.009782	0.01478	-3.99986362
MDGA1	-0.1366155	0.77299457	-2.602820312	0.009785	0.01478	-4.00009277
CD47	0.264380509	6.00457148	2.602585083	0.009791	0.01479	-4.00068904
MCTP2	0.244164994	3.4881859	2.60123044	0.009829	0.01485	-4.00412183
ZNF598	-0.158362716	4.07669934	-2.601065563	0.009834	0.01485	-4.00453952
MTR	-0.208682391	4.39991142	-2.600695517	0.009844	0.01487	-4.0054769
CPAMD8	0.63643382	3.52436216	2.600501563	0.009849	0.01487	-4.00596817
MOB3C	-0.172364534	3.76654064	-2.600236348	0.009857	0.01488	-4.00663987
LRRC1	-0.213681866	4.4041615	-2.59995144	0.009865	0.0149	-4.00736138
PBLD	-0.156971595	2.02184875	-2.599264726	0.009884	0.01492	-4.00910011
NADSYN	-0.138796386	3.44153505	-2.599141425	0.009888	0.01493	-4.00941226
FAM124E	-0.195324459	1.2720819	-2.598963799	0.009893	0.01493	-4.00986191
C11orf16	-0.048779851	0.15753437	-2.598042669	0.009918	0.01497	-4.01219325
PER2	-0.249460773	3.70189385	-2.597800083	0.009925	0.01498	-4.01280709
SLC25A1	-0.065301398	0.59168811	-2.597725445	0.009927	0.01498	-4.01299594

CALB2	0.289839661	0.73962158	2.597071972	0.009946	0.01501	-4.01464918
ARHGAF	-0.250342843	2.36216187	-2.597039959	0.009947	0.01501	-4.01473016
CBFA2T3	-0.20137084	1.5247725	-2.596875593	0.009951	0.01501	-4.01514593
OLFML2I	0.344813254	3.78859983	2.596849662	0.009952	0.01501	-4.01521152
DMC1	-0.077668457	0.47465766	-2.596731532	0.009955	0.01502	-4.01551032
AP1AR	-0.183082321	3.87816472	-2.596618877	0.009959	0.01502	-4.01579525
C19orf71	-0.260874121	3.06993212	-2.596583275	0.00996	0.01502	-4.01588529
PWWP2/	-0.153769465	3.11109685	-2.596403774	0.009965	0.01503	-4.01633927
ULK2	-0.16457069	3.0492583	-2.59596954	0.009977	0.01504	-4.01743736
LCP2	0.277305942	2.81765007	2.595545963	0.009989	0.01506	-4.01850834
NFATC3	-0.232435801	2.61568592	-2.595355263	0.009994	0.01507	-4.01899045
CAPN12	0.16938945	0.80876854	2.594087665	0.01003	0.01512	-4.02219425
ORAI2	-0.19397413	4.94395544	-2.593386635	0.01005	0.01515	-4.02396544
SETBP1	-0.234964008	2.33053863	-2.592874371	0.010065	0.01517	-4.0252594
ACAP2	-0.216542722	3.94393965	-2.592855157	0.010065	0.01517	-4.02530793
GPX2	-0.265929446	0.50208229	-2.5928252	0.010066	0.01517	-4.0253836
UBE2J1	-0.173532374	5.90843865	-2.592117753	0.010086	0.0152	-4.02717017
CACNG8	-0.044735246	0.1371267	-2.591593412	0.010101	0.01522	-4.02849403
LIPE	-0.152930436	2.38125925	-2.591580377	0.010102	0.01522	-4.02852694
FASLG	0.23472758	0.81145538	2.591536258	0.010103	0.01522	-4.02863831
ZC3HAV	-0.188504754	4.27772261	-2.591333115	0.010109	0.01523	-4.02915113
SASH1	-0.275843654	5.02623858	-2.591223948	0.010112	0.01523	-4.0294267
GPHA2	-0.077063003	0.15413731	-2.591205906	0.010112	0.01523	-4.02947224
PTH2R	0.024918477	0.07458782	2.590850279	0.010123	0.01524	-4.03036986
XIRP1	0.18501358	0.1949439	2.590757799	0.010125	0.01525	-4.03060326
TWF1	-0.213347394	5.81049607	-2.590491186	0.010133	0.01526	-4.0312761
CACNG1	0.089771317	0.10108497	2.589813238	0.010152	0.01528	-4.03298673
SOX6	-0.174028283	1.41321619	-2.589569339	0.010159	0.01529	-4.03360203
USP41	-0.111028844	0.11843386	-2.589540081	0.01016	0.01529	-4.03367584
PCDH1	0.270111669	4.87918486	2.589498847	0.010161	0.01529	-4.03377986
CP	0.349795186	1.27214233	2.589260492	0.010168	0.0153	-4.03438112
PLTP	0.360797358	5.13084298	2.588574046	0.010188	0.01533	-4.0361124
SASS6	-0.127575609	1.84868898	-2.587938712	0.010206	0.01536	-4.03771438
GNGT2	-0.182274827	1.91656738	-2.587759698	0.010211	0.01536	-4.03816569
TMEM14	-0.167057628	0.49926424	-2.587700952	0.010213	0.01537	-4.03831379
TTC37	-0.22264646	4.77012147	-2.587375214	0.010223	0.01538	-4.03913491
OGA	-0.186270287	6.17036556	-2.587098022	0.010231	0.01539	-4.03983359
APBB2	-0.221717753	4.33266136	-2.586729546	0.010241	0.0154	-4.04076224
PRDM7	-0.019577522	0.03472589	-2.586508903	0.010248	0.01541	-4.04131825
VPS37C	-0.139909409	4.31324384	-2.585257999	0.010284	0.01546	-4.04446964

DBP	-0.249760555	4.90130623	-2.584608307	0.010303	0.01549	-4.04610582
SP7	-0.134477382	0.34627337	-2.58431509	0.010311	0.0155	-4.04684413
ZNF317	-0.141287384	4.16912265	-2.584134418	0.010317	0.01551	-4.04729902
NOX5	-0.312422004	0.81928875	-2.582793023	0.010356	0.01557	-4.05067538
TUBB	-0.171612083	8.37787758	-2.582260018	0.010371	0.01559	-4.05201652
DNAH1	0.143987252	1.58983012	2.58219619	0.010373	0.01559	-4.0521771
P2RY12	0.242927758	1.30811043	2.581763856	0.010386	0.01561	-4.05326472
AKAP6	-0.173129421	1.69881303	-2.581749189	0.010386	0.01561	-4.05330161
BMP1	0.244364618	5.04400589	2.581676844	0.010388	0.01561	-4.05348359
NFX1	-0.165794102	4.66519905	-2.581664837	0.010389	0.01561	-4.05351379
MANSC4	0.164657616	0.32542189	2.581649131	0.010389	0.01561	-4.0535533
HAUS4	-0.194208165	4.66758254	-2.581528759	0.010393	0.01561	-4.05385607
SLC25A1	-0.141750576	3.20558359	-2.58143499	0.010396	0.01561	-4.05409192
AQP1	-0.308674627	7.19768621	-2.581097622	0.010405	0.01563	-4.05494041
TESK1	-0.147064134	5.00742649	-2.580918437	0.010411	0.01563	-4.05539102
PPP3CA	-0.17240501	4.9272339	-2.580009537	0.010437	0.01567	-4.05767624
SLC16A3	0.317037793	3.80601429	2.579978049	0.010438	0.01567	-4.0577554
ZNF678	-0.169218137	2.16332812	-2.579359708	0.010457	0.0157	-4.05930962
ZSWIM5	-0.30688366	3.74860355	-2.579026536	0.010466	0.01571	-4.06014692
KIRREL1	0.247982098	3.67572598	2.578806386	0.010473	0.01572	-4.06070012
ZNF90	0.349969295	2.65353277	2.578669684	0.010477	0.01573	-4.06104361
GABRA5	-0.088363577	0.15871057	-2.578077739	0.010494	0.01575	-4.06253078
ZNF267	0.197978274	2.72597736	2.57807114	0.010495	0.01575	-4.06254735
RSPH10I	-0.01991563	0.05208748	-2.576699172	0.010535	0.01581	-4.06599295
CTDSPL	-0.176765874	3.37894038	-2.576411635	0.010544	0.01582	-4.06671486
AREL1	-0.173523016	4.52230626	-2.574963727	0.010587	0.01588	-4.0703489
VXN	-0.092681955	0.41426744	-2.574872439	0.01059	0.01589	-4.07057796
RBBP9	-0.248637587	5.90844745	-2.574444092	0.010602	0.0159	-4.07165264
FANCA	0.145733503	1.19773592	2.573754972	0.010623	0.01593	-4.07338122
GAPT	0.205706941	1.1960597	2.573413325	0.010633	0.01595	-4.07423804
PPTC7	-0.200769207	4.14983194	-2.573349923	0.010635	0.01595	-4.07439704
XCL1	0.24509347	0.89086582	2.572609786	0.010657	0.01598	-4.07625282
PTX3	-0.201169202	1.25797729	-2.572471819	0.010661	0.01599	-4.0765987
ZNF558	-0.176311591	2.93219431	-2.570391437	0.010724	0.01608	-4.08181197
POU1F1	-0.053763673	0.24648898	-2.57037857	0.010724	0.01608	-4.0818442
TEX11	0.022269828	0.03767507	2.569400283	0.010754	0.01612	-4.08429431
AMOTL2	-0.255003599	5.45389211	-2.568343372	0.010786	0.01617	-4.08694034
RB1	-0.209111255	4.77790172	-2.567973965	0.010797	0.01618	-4.08786493
FCRL3	0.208835862	0.43067584	2.567832274	0.010802	0.01619	-4.08821953
FAM90A1	-0.270682249	1.23885416	-2.567792293	0.010803	0.01619	-4.08831959

MYBPC3	0.031460915	0.09083286	2.567333294	0.010817	0.01621	-4.08946816
MYRFL	-0.084124652	0.25269389	-2.566739877	0.010835	0.01623	-4.0909528
SGK3	0.24290195	3.8353546	2.56661671	0.010838	0.01624	-4.09126091
FLII	0.188592442	6.32318918	2.565813321	0.010863	0.01627	-4.09327026
CDK11A	-0.145908583	2.0557277	-2.564624636	0.010899	0.01632	-4.09624217
FAM149E	-0.149562727	3.60496176	-2.564580824	0.010901	0.01633	-4.09635168
ADAT3	-0.195473358	1.66672526	-2.564042119	0.010917	0.01635	-4.09769808
LINC022	-0.150856835	0.5156932	-2.563677863	0.010928	0.01636	-4.09860833
CRHR1	-0.150856835	0.5156932	-2.563677863	0.010928	0.01636	-4.09860833
RNFT2	-0.137399231	1.66356654	-2.563532458	0.010933	0.01637	-4.09897165
SHPK	-0.146433203	2.52988503	-2.563515048	0.010933	0.01637	-4.09901515
URB2	-0.173866179	2.33223281	-2.562938341	0.010951	0.01639	-4.10045595
FGF20	0.250961821	0.94064376	2.56288032	0.010953	0.01639	-4.10060089
RSPH14	-0.13759876	1.05057314	-2.562630835	0.01096	0.0164	-4.10122408
DGKQ	-0.156219465	3.69325109	-2.562629148	0.010961	0.0164	-4.10122829
GPSM3	0.309370096	4.21642705	2.562252641	0.010972	0.01642	-4.10216865
ZNF430	0.29597165	3.34990819	2.561954664	0.010981	0.01643	-4.10291279
NLRP2B	-0.02018649	0.05350033	-2.561268472	0.011002	0.01646	-4.10462609
CSPG5	-0.153897336	0.96824472	-2.560334732	0.011031	0.0165	-4.10695678
P2RX5	0.194649811	0.39411295	2.559734967	0.01105	0.01653	-4.10845342
CPM	-0.269812519	3.33921205	-2.558949869	0.011074	0.01656	-4.11041203
HMCES	-0.160791671	6.00370664	-2.55884873	0.011077	0.01657	-4.1106643
NXNL1	-0.065291562	0.15507266	-2.558119471	0.0111	0.0166	-4.11248303
ZNF165	-0.167829139	2.96721622	-2.557971256	0.011105	0.0166	-4.1128526
ITPK1	-0.177318761	5.33778144	-2.557603658	0.011116	0.01662	-4.11376913
ZNF655	-0.185619388	4.25416416	-2.556111123	0.011163	0.01669	-4.11748917
ATF1	-0.231869984	4.88180601	-2.556049295	0.011165	0.01669	-4.11764323
SIPA1L1	0.214667956	4.29089897	2.555740124	0.011174	0.0167	-4.11841354
NRAS	-0.187443631	4.73396302	-2.555078283	0.011195	0.01673	-4.12006224
CSE1L	-0.150288937	5.65121419	-2.55358474	0.011242	0.0168	-4.1237813
CD247	0.307093349	1.56175036	2.553332046	0.01125	0.01681	-4.12441032
EDEM1	0.256992487	5.15377746	2.553055997	0.011259	0.01682	-4.12509742
DOK1	-0.187425481	4.02157424	-2.552050797	0.01129	0.01687	-4.1275988
DNM3	0.150455385	1.24950281	2.550862473	0.011328	0.01692	-4.13055467
FBLN1	0.508008329	3.44310964	2.550839999	0.011329	0.01692	-4.13061056
ESYT3	-0.28933166	2.90982068	-2.55073363	0.011332	0.01693	-4.13087508
ALG1L2	-0.104826252	0.53168081	-2.550597747	0.011336	0.01693	-4.13121298
TBC1D2	-0.149887372	3.91408974	-2.550120487	0.011351	0.01695	-4.13239964
IGBP1	-0.179473304	7.64151888	-2.549330732	0.011376	0.01699	-4.13436283
CFAP97I	-0.01802644	0.04361812	-2.54854961	0.011401	0.01702	-4.136304

MAMSTF	-0.124498029	1.50132504	-2.548457905	0.011404	0.01703	-4.13653185
LEPROT	-0.159873446	4.81404299	-2.548300002	0.011409	0.01703	-4.13692418
NISCH	-0.193554776	5.34422424	-2.548220593	0.011412	0.01704	-4.13712146
GPR65	0.211796098	1.14501574	2.548150145	0.011414	0.01704	-4.13729649
STRIP2	0.148706315	0.95525263	2.548049463	0.011417	0.01704	-4.13754661
USP6NL	0.179338385	2.59041843	2.546072382	0.01148	0.01713	-4.1424564
ZNF385C	-0.164873191	1.03280004	-2.545957101	0.011484	0.01714	-4.14274258
SYCP3	0.088853816	0.52613187	2.545041689	0.011514	0.01718	-4.14501454
CREBRF	-0.195434023	4.07273825	-2.543828636	0.011553	0.01724	-4.14802403
RBM11	-0.176929709	2.13551469	-2.543607183	0.01156	0.01724	-4.14857329
KLK12	0.434930904	1.13779543	2.543599014	0.01156	0.01724	-4.14859355
GJA5	-0.264834888	3.3722196	-2.54330766	0.011569	0.01726	-4.14931611
TMCO3	-0.203919778	2.59896724	-2.543241305	0.011572	0.01726	-4.14948066
USH1G	-0.03909637	0.05305779	-2.543114882	0.011576	0.01726	-4.14979416
RCC2	0.181112577	6.15208009	2.54236961	0.0116	0.0173	-4.15164195
NCKAP5	-0.094601704	0.49164689	-2.541548318	0.011626	0.01734	-4.15367763
PTGES	0.299114603	2.13530669	2.54069422	0.011654	0.01738	-4.15579395
ZNF211	-0.184589551	3.88968909	-2.540666009	0.011655	0.01738	-4.15586384
LYG2	-0.06176462	0.25677657	-2.539815415	0.011683	0.01742	-4.15797079
SLC3A2	-0.188890179	6.74446839	-2.539116873	0.011705	0.01745	-4.1597006
CGAS	0.173036727	2.0536667	2.538566814	0.011723	0.01747	-4.1610624
ASB13	-0.141770602	3.60962674	-2.538388506	0.011729	0.01748	-4.16150379
TSNARE	-0.174582304	3.74078305	-2.537948282	0.011744	0.0175	-4.16259339
GOLT1A	0.47411402	4.41868146	2.537804998	0.011748	0.0175	-4.162948
CLOCK	-0.19642268	3.58997099	-2.537782281	0.011749	0.0175	-4.16300421
CNP	-0.156774496	5.39250745	-2.537106873	0.011771	0.01754	-4.16467547
STAC3	0.190543562	2.52894945	2.536720119	0.011784	0.01755	-4.16563228
E2F3	-0.198133473	4.01899197	-2.53603494	0.011806	0.01759	-4.16732704
CDC20B	-0.053411009	0.10642699	-2.535540661	0.011823	0.01761	-4.16854934
BHLHE22	0.177469789	0.37854296	2.534792348	0.011847	0.01764	-4.17039942
TENT4A	-0.163538047	3.85874215	-2.534606538	0.011853	0.01765	-4.17085872
HSF2BP	-0.119752677	1.00279382	-2.534087644	0.01187	0.01767	-4.1721412
ERCC6L2	-0.199026024	2.3947343	-2.533594501	0.011887	0.0177	-4.17335981
RASAL2	0.198630026	2.69470696	2.533334754	0.011895	0.01771	-4.17400158
TMEM24	-0.147194368	1.79919312	-2.532362652	0.011928	0.01776	-4.17640284
JPH1	0.277033428	2.7139	2.531396464	0.01196	0.0178	-4.17878863
SESN2	-0.263663513	3.91982936	-2.530348379	0.011995	0.01785	-4.18137567
DNASE1	-0.340075431	2.47645597	-2.528910988	0.012043	0.01792	-4.18492199
SNAI3	-0.162288889	2.17211084	-2.527228684	0.012099	0.018	-4.18907012
ABHD4	-0.18821132	5.27253324	-2.526957224	0.012108	0.01802	-4.18973923

SPSB1	0.253477798	4.07621673	2.525324905	0.012163	0.0181	-4.19376119
C1QB	0.426108382	6.3896712	2.525289677	0.012164	0.0181	-4.19384796
PDCD6IF	-0.17658208	5.69388613	-2.52471628	0.012184	0.01812	-4.19526019
TAC4	-0.108517262	0.57830015	-2.524560539	0.012189	0.01813	-4.19564371
NSUN3	-0.141553178	2.45451662	-2.524383474	0.012195	0.01814	-4.19607971
RBM5	-0.19952155	4.85321726	-2.523342781	0.01223	0.01819	-4.19864174
SENP7	-0.211062375	3.60462527	-2.523087709	0.012239	0.0182	-4.19926953
LRRC17	-0.264919913	2.58510926	-2.52296832	0.012243	0.0182	-4.19956336
SLC30A1	-0.020969669	0.03138578	-2.522942339	0.012244	0.0182	-4.19962729
ADAMTS	-0.316393328	3.34852867	-2.522035854	0.012275	0.01825	-4.20185776
NUP160	-0.197272434	4.11670688	-2.521372807	0.012297	0.01828	-4.20348875
MAST3	0.202189494	3.50319709	2.520990691	0.01231	0.0183	-4.20442851
ANKLE2	0.170717283	3.94070652	2.520988817	0.01231	0.0183	-4.20443312
POLR2F	-0.038556554	0.17503094	-2.520317988	0.012333	0.01833	-4.2060826
PSIP1	-0.178635368	5.0127408	-2.520185456	0.012338	0.01833	-4.20640843
ASTL	0.143383121	0.24714489	2.5190988	0.012375	0.01839	-4.20907935
CCL5	0.492930601	4.68449367	2.518392456	0.012399	0.01842	-4.2108149
FKBP8	-0.156968516	8.5067149	-2.518272813	0.012403	0.01843	-4.21110883
XPO1	-0.179692695	5.12899704	-2.517801631	0.012419	0.01845	-4.21226626
SELENO	-0.327369863	4.42102795	-2.51779438	0.01242	0.01845	-4.21228407
ZNF283	-0.12296156	1.6038427	-2.517789387	0.01242	0.01845	-4.21229633
TEKT5	-0.058987901	0.19837385	-2.517137476	0.012442	0.01848	-4.21389736
OCLM	0.118710913	0.59236397	2.516726957	0.012456	0.0185	-4.21490535
ZNF418	-0.171729771	2.57689421	-2.516466672	0.012465	0.01851	-4.21554438
CLIP1	-0.225260221	4.89754724	-2.516116595	0.012477	0.01853	-4.21640375
DCLRE1(	-0.132276975	2.34664903	-2.515870732	0.012486	0.01854	-4.21700723
SCN9A	0.135272333	0.37441915	2.515219773	0.012508	0.01857	-4.21860477
STRN4	-0.138576971	5.14880791	-2.515087346	0.012513	0.01857	-4.21892971
SPG11	-0.205756215	4.83009906	-2.514713124	0.012526	0.01859	-4.21984788
SH3BGR	-0.257756675	7.07558919	-2.513726847	0.01256	0.01864	-4.2222671
SLC44A1	-0.210509547	5.71032232	-2.513645642	0.012563	0.01864	-4.22246625
FAM92B	0.13952056	0.24614589	2.513472598	0.012569	0.01865	-4.22289061
SPIN1	-0.22473757	6.15156544	-2.513034619	0.012584	0.01867	-4.22396453
ETNK2	0.351146256	4.6773364	2.512389201	0.012607	0.0187	-4.22554677
CHP2	-0.039851247	0.06177339	-2.512372773	0.012607	0.0187	-4.22558703
PDS5B	-0.188537395	3.15853228	-2.512224984	0.012612	0.01871	-4.22594928
TNFSF8	0.248084721	1.04254936	2.512182571	0.012614	0.01871	-4.22605324
RCN3	0.297511476	3.83475047	2.512077999	0.012618	0.01871	-4.22630954
IER3	0.409144678	6.05345168	2.511937877	0.012623	0.01872	-4.22665295
DKK3	-0.277940542	4.26128314	-2.510876828	0.01266	0.01877	-4.22925282

ETV4	0.435394951	4.921852	2.509470757	0.012709	0.01884	-4.23269648
RHOV	0.376477419	2.41613569	2.507546449	0.012777	0.01894	-4.2374064
GCKR	0.128889818	0.45955819	2.507339885	0.012784	0.01895	-4.23791178
PLEKHG	0.276269306	3.76472609	2.5070374	0.012795	0.01896	-4.23865176
CFAP70	-0.242658496	2.35327895	-2.507027183	0.012795	0.01896	-4.23867676
NEDD4	-0.219349425	2.65261269	-2.506690213	0.012807	0.01898	-4.239501
ADNP	-0.199698631	5.47362722	-2.506649843	0.012808	0.01898	-4.23959974
CTBS	-0.193176122	3.69238604	-2.506587978	0.01281	0.01898	-4.23975106
EXOC5	-0.1779096	3.64194955	-2.505318698	0.012855	0.01905	-4.24285472
ERAP2	0.45903108	3.90008602	2.504603115	0.012881	0.01908	-4.24460382
TPCN1	-0.203548058	5.63736323	-2.504327328	0.012891	0.01909	-4.24527779
TUBA1A	0.276955182	6.0703573	2.503787355	0.01291	0.01912	-4.24659719
MYD88	0.211389386	4.66523407	2.502304841	0.012963	0.0192	-4.25021825
BNIPL	0.421132482	3.84948569	2.499830389	0.013051	0.01933	-4.25625759
ZBTB4	-0.173933314	6.99143236	-2.49953904	0.013062	0.01934	-4.25696831
FANCG	-0.144951554	3.42453828	-2.499085632	0.013078	0.01936	-4.25807419
PBRM1	-0.216572306	4.27187634	-2.498872331	0.013086	0.01937	-4.25859438
MAB21L1	-0.069012869	0.27716319	-2.498871352	0.013086	0.01937	-4.25859676
ZNF507	-0.178240173	3.12598067	-2.49874133	0.01309	0.01938	-4.25891383
TES	-0.210467066	6.31843716	-2.49845508	0.013101	0.01939	-4.25961182
PRDM12	-0.061675257	0.08063671	-2.497374925	0.01314	0.01945	-4.26224496
USP42	-0.160695236	2.84196423	-2.497305102	0.013142	0.01945	-4.26241514
TNFRSF	0.160431341	0.31918555	2.496935736	0.013156	0.01947	-4.26331529
TINAGL1	-0.237522514	5.98060747	-2.49611687	0.013185	0.01951	-4.26531042
C1QC	0.401181214	6.3815591	2.495947373	0.013191	0.01952	-4.26572331
PBX1	-0.248538097	4.26590562	-2.495856019	0.013195	0.01952	-4.26594584
LY6G6F	-0.07994727	0.25900731	-2.495747899	0.013199	0.01952	-4.2662092
KAZN	-0.237727855	2.72948969	-2.495473445	0.013209	0.01954	-4.26687766
ANKRD3	0.096078547	0.57309301	2.495426793	0.01321	0.01954	-4.26699128
CHAMP1	-0.231542781	4.10723707	-2.495354545	0.013213	0.01954	-4.26716723
ADGRE1	0.1163191	0.44470361	2.495046738	0.013224	0.01955	-4.26791681
TAF2	-0.211144152	3.42880178	-2.494067105	0.01326	0.01961	-4.27030185
MORC2	-0.147666638	4.15878334	-2.493674772	0.013274	0.01963	-4.27125678
PROM2	-0.306544199	5.3164766	-2.493121092	0.013294	0.01965	-4.27260419
TRPV2	0.239050183	3.0474113	2.492798806	0.013306	0.01967	-4.27338836
IRGM	0.027579948	0.08999051	2.492328094	0.013323	0.01969	-4.27453349
BTC	-0.212601306	3.81833298	-2.492290774	0.013325	0.01969	-4.27462427
ZNF202	-0.145750551	3.03337582	-2.491717562	0.013346	0.01972	-4.27601847
M6PR	-0.308521052	5.78474317	-2.491498848	0.013354	0.01973	-4.27655036
ABCG1	-0.242092139	3.71410538	-2.489225913	0.013437	0.01985	-4.28207524

CISH	-0.259274812	5.04544644	-2.489174891	0.013439	0.01986	-4.28219921
GPR141	-0.23219116	0.7298523	-2.48890254	0.013449	0.01987	-4.28286088
ZFP90	-0.145443189	3.61777504	-2.488888255	0.01345	0.01987	-4.28289559
ZNF154	-0.231883783	2.96443862	-2.488842372	0.013452	0.01987	-4.28300705
ITIH1	0.039359622	0.05277297	2.487670234	0.013495	0.01993	-4.28585391
PVRIG	0.07081836	0.2738629	2.487619249	0.013497	0.01993	-4.28597771
NDRG1	-0.196662397	5.27102067	-2.486251973	0.013547	0.02001	-4.28929683
LAIR2	0.181694886	0.58159882	2.486053876	0.013555	0.02001	-4.28977757
GNB4	0.260043459	3.11258448	2.485789323	0.013565	0.02003	-4.29041953
OSBP2	-0.167074447	2.6865633	-2.485163101	0.013588	0.02006	-4.29193886
TRIM9	-0.059622804	0.24957312	-2.483829575	0.013638	0.02013	-4.29517303
ASNS	-0.24434131	3.97036863	-2.483445625	0.013652	0.02015	-4.29610391
HSPA13	-0.231205041	4.5303086	-2.482237863	0.013697	0.02022	-4.2990312
TMIGD2	0.161405122	0.49920865	2.481545092	0.013723	0.02025	-4.30070968
NFE4	-0.045552575	0.14272782	-2.480715839	0.013755	0.0203	-4.30271825
HS3ST3I	-0.167164216	0.97191759	-2.480566485	0.01376	0.0203	-4.30307994
PVALEF	-0.026935096	0.05296083	-2.480334067	0.013769	0.02031	-4.30364274
SPAG4	0.303745229	2.54947306	2.479565	0.013798	0.02036	-4.30550468
HOMER2	-0.20405687	3.60988212	-2.479518626	0.0138	0.02036	-4.30561694
TMEM15	-0.160697107	4.46056024	-2.478695568	0.013831	0.0204	-4.30760895
SCN8A	0.125911885	0.52503805	2.477577172	0.013873	0.02046	-4.31031475
CLCN5	-0.1422169	1.70657011	-2.47722931	0.013886	0.02048	-4.31115611
PELP1	-0.135568583	5.27760529	-2.477070538	0.013892	0.02049	-4.3115401
CC2D2B	-0.229928702	1.02724561	-2.476275685	0.013923	0.02053	-4.31346204
RNF111	-0.212149614	3.68299204	-2.475432805	0.013955	0.02057	-4.31549948
MID2	-0.235497914	3.40383091	-2.474675104	0.013984	0.02062	-4.31733046
PCNX1	-0.19854314	3.57310692	-2.474606367	0.013986	0.02062	-4.31749653
ARFGAP	-0.149687586	5.06797163	-2.474430656	0.013993	0.02063	-4.31792105
KLHL20	-0.215561172	3.62322085	-2.473079992	0.014045	0.0207	-4.32118328
SMIM22	-0.413659515	1.09643884	-2.472894751	0.014052	0.02071	-4.32163056
TMEM98	0.337439782	6.08753608	2.472804451	0.014055	0.02071	-4.32184858
CCDC54	-0.030905191	0.1073516	-2.472018406	0.014086	0.02075	-4.32374612
GGT1	-0.238539518	4.08626747	-2.47140113	0.014109	0.02079	-4.32523584
GJA1	-0.356101638	7.42653969	-2.471021051	0.014124	0.02081	-4.32615293
CSRNP1	-0.294329606	5.76623382	-2.470629935	0.014139	0.02083	-4.32709652
IPP	-0.203481632	4.06521123	-2.470294178	0.014152	0.02085	-4.32790644
NARF	-0.145556246	3.77234011	-2.469728372	0.014174	0.02088	-4.32927105
GRAP2	0.162247904	0.69443448	2.4692309	0.014193	0.0209	-4.3304706
PFKFB3	0.303359694	3.7032491	2.467948663	0.014243	0.02097	-4.3335614
MSTN	-0.131769264	0.5328515	-2.467888465	0.014245	0.02098	-4.33370647

AKR1B1	-0.296259419	5.54151257	-2.467074753	0.014277	0.02102	-4.33566706
CFAP30(	-0.279814393	2.11052152	-2.466619913	0.014295	0.02104	-4.3367627
SCMH1	-0.147059082	5.21116695	-2.466379662	0.014304	0.02106	-4.33734135
MPND	-0.148688529	5.23413842	-2.466345801	0.014305	0.02106	-4.3374229
ADGRL3	0.14630988	0.26228129	2.466029519	0.014318	0.02107	-4.33818458
IL19	-0.064499897	0.15255011	-2.465973784	0.01432	0.02107	-4.33831879
NCLN	-0.142717522	5.26785944	-2.465615365	0.014334	0.02109	-4.33918182
N4BP2L1	0.206323325	3.34223147	2.465562447	0.014336	0.02109	-4.33930923
SLC17A7	-0.088748491	0.38133621	-2.465545527	0.014336	0.02109	-4.33934997
SCAMP4	-0.180630198	5.56694824	-2.464916843	0.014361	0.02113	-4.34086343
IGF2R	0.261258896	5.61266959	2.464746582	0.014368	0.02114	-4.34127324
TIMP2	0.24381261	6.53664491	2.464691585	0.01437	0.02114	-4.34140561
ACKR4	0.117085803	0.57350416	2.463974863	0.014398	0.02118	-4.34313041
ZNF33A	-0.172952147	4.30309877	-2.463909803	0.0144	0.02118	-4.34328695
PDP1	0.207918266	4.6529915	2.463766577	0.014406	0.02118	-4.34363156
TLL1	-0.131799192	0.65637933	-2.463518354	0.014416	0.0212	-4.34422875
ANKRD5	-0.219150347	3.32527945	-2.462234929	0.014466	0.02127	-4.34731559
FAM71F2	-0.11474122	0.69419706	-2.462010201	0.014475	0.02128	-4.34785593
SPIB	0.22521994	0.5122129	2.46196963	0.014477	0.02128	-4.34795348
PAQR8	-0.223621549	2.48970094	-2.461912584	0.014479	0.02128	-4.34809063
NGF	-0.204542592	1.33429107	-2.461643051	0.01449	0.0213	-4.34873862
LGALS4	-0.158696999	0.74604718	-2.460819612	0.014522	0.02134	-4.35071786
UNKL	-0.209887598	2.60493915	-2.460185427	0.014547	0.02138	-4.35224177
MGLL	0.212696933	3.25663094	2.460171184	0.014548	0.02138	-4.35227599
RORA	-0.208236998	2.58406585	-2.459408701	0.014578	0.02142	-4.35410769
GBP3	0.414795267	5.02559097	2.459258705	0.014584	0.02143	-4.35446796
CNGA4	-0.075405017	0.29703462	-2.458266724	0.014623	0.02148	-4.35685002
CMTM2	-0.105096388	0.67708543	-2.454832344	0.01476	0.02168	-4.36508999
CNIH3	-0.174824852	2.39347817	-2.45446145	0.014775	0.0217	-4.3659792
DCT	-0.074163941	0.26559848	-2.45406617	0.014791	0.02172	-4.36692674
VPS13C	0.229575801	3.87241395	2.452561232	0.014852	0.02181	-4.37053294
ECM2	-0.25282308	2.49215829	-2.452529046	0.014853	0.02181	-4.37061004
LINC006	0.127737645	0.79421818	2.452421439	0.014857	0.02181	-4.37086781
TRPM8	0.062955235	0.08550943	2.4522333	0.014865	0.02182	-4.37131847
GRIK1	0.041688297	0.15859311	2.451085803	0.014911	0.02189	-4.37406638
CEND1	-0.14965198	0.61861262	-2.450584831	0.014932	0.02192	-4.37526568
TASOR	-0.187510982	4.58196584	-2.450527026	0.014934	0.02192	-4.37540404
PPARA	-0.197616246	2.97045543	-2.450058641	0.014953	0.02194	-4.37652509
ABCC11	-0.32832089	0.81088995	-2.450044163	0.014954	0.02194	-4.37655974
CFLAR	0.189797801	4.46950516	2.449505546	0.014975	0.02197	-4.37784862

GZMA	0.439766876	3.12743161	2.449387589	0.01498	0.02198	-4.37813085
CNOT6	-0.177093541	4.09867981	-2.449257312	0.014985	0.02198	-4.37844254
CENPE	0.105620286	0.59891194	2.44794689	0.015039	0.02206	-4.38157688
SPERT	-0.099292891	0.11067585	-2.447861004	0.015042	0.02206	-4.38178225
TRAPPC	-0.199256001	4.03915699	-2.447771407	0.015046	0.02207	-4.38199649
PDZD2	-0.164249887	1.20254646	-2.44742106	0.01506	0.02209	-4.38283414
TAS2R43	-0.035558677	0.08834623	-2.447174813	0.01507	0.0221	-4.38342283
PPAN	-0.142902276	2.63899449	-2.446240374	0.015108	0.02215	-4.38565622
LRCH1	-0.167861427	3.00668777	-2.445109792	0.015155	0.02222	-4.38835732
CLDND1	-0.158699896	4.69524641	-2.444358177	0.015186	0.02226	-4.39015237
GLRB	0.265698953	2.40730588	2.442523164	0.015261	0.02237	-4.39453262
GRAMD2	0.318777926	5.76853611	2.441456013	0.015305	0.02244	-4.39707852
SUPT16H	-0.169975282	5.48878717	-2.440908961	0.015328	0.02247	-4.3983832
COLGAL	0.155401822	4.62272769	2.44088758	0.015329	0.02247	-4.39843419
PPP1R12	-0.192563152	3.93893263	-2.440597486	0.015341	0.02248	-4.39912592
BAX	-0.182742394	6.14863613	-2.440522424	0.015344	0.02248	-4.3993049
H2AFX	-0.244511541	5.75752577	-2.439871741	0.015371	0.02252	-4.40085614
SH2D3A	0.209515922	3.59698614	2.4398045	0.015374	0.02252	-4.40101642
FBXL16	0.353569724	4.49710795	2.438529366	0.015427	0.0226	-4.40405514
SUCO	-0.183025823	4.3544461	-2.438416103	0.015432	0.0226	-4.40432498
ZCCHC1	1.017115594	7.33976862	2.438094821	0.015445	0.02262	-4.40509034
C3orf49	-0.05292364	0.27543919	-2.437964066	0.015451	0.02263	-4.4054018
KIAA1109	-0.238033838	3.7697305	-2.436987848	0.015491	0.02269	-4.40772664
RSPH9	-0.140095423	1.21973344	-2.436793101	0.0155	0.0227	-4.40819033
RANBP6	-0.180867944	4.38067061	-2.436594799	0.015508	0.02271	-4.40866243
CNTNAP	-0.03887334	0.0931985	-2.435956478	0.015535	0.02274	-4.41018187
INTS7	-0.164870089	3.61876953	-2.435330539	0.015561	0.02278	-4.41167146
GPR1	0.176425791	0.41975512	2.434678338	0.015588	0.02282	-4.41322316
STK32B	-0.09323074	0.54428837	-2.434029457	0.015616	0.02286	-4.41476657
SUMO4	0.072469571	0.33138552	2.43324669	0.015649	0.0229	-4.41662792
NT5C2	-0.324589374	4.92326624	-2.432226091	0.015692	0.02296	-4.41905394
EPC2	-0.210353115	4.30792732	-2.432086936	0.015698	0.02297	-4.41938465
DIPK1C	0.090187752	0.24335995	2.432044117	0.0157	0.02297	-4.41948641
IPO9	-0.161714658	4.35228527	-2.431953657	0.015703	0.02297	-4.41970137
BCLAF3	-0.142323809	2.6501088	-2.431229067	0.015734	0.02302	-4.42142299
GALNT1	-0.194283659	5.31892476	-2.429823836	0.015794	0.0231	-4.42476042
SVIL	0.266285136	5.49871526	2.429471536	0.015809	0.02312	-4.42559684
EPRS	-0.217888976	5.98633494	-2.429453973	0.01581	0.02312	-4.42563853
RBM17	-0.145598804	5.22445099	-2.429153507	0.015822	0.02314	-4.4263518
WNK3	-0.14153068	0.89627793	-2.428596532	0.015846	0.02317	-4.42767376

ZNF728	-0.251836582	0.94511476	-2.428543684	0.015848	0.02317	-4.42779918
KCNJ5	0.346360533	2.03345656	2.427928806	0.015875	0.02321	-4.4292582
FFAR1	0.147714055	0.3277022	2.42752112	0.015892	0.02323	-4.43022538
NCR3	0.212652927	0.75253591	2.427513646	0.015892	0.02323	-4.43024312
PRODH	0.236979457	1.39316644	2.426542239	0.015934	0.02329	-4.43254704
C1QA	0.378513679	6.82292831	2.426240993	0.015947	0.02331	-4.43326133
BFAR	-0.153232783	5.44534388	-2.425888488	0.015962	0.02333	-4.43409706
ZDHHC9	-0.196336598	4.51614469	-2.42558483	0.015975	0.02334	-4.4348169
CHD5	0.094538019	0.29521049	2.425276211	0.015988	0.02336	-4.4355484
INO80B	-0.188664058	3.27604033	-2.425103	0.015996	0.02337	-4.43595891
STX1B	0.177425782	0.9334846	2.42483379	0.016007	0.02339	-4.43659689
CACNA2	-0.101297761	0.80177205	-2.423906609	0.016047	0.02344	-4.43879362
CPSF4L	-0.038721262	0.14378933	-2.423595546	0.016061	0.02346	-4.43953043
PDZD3	-0.043114959	0.09036357	-2.423490741	0.016065	0.02346	-4.43977866
ZBTB1	-0.212515523	4.21467846	-2.422580013	0.016105	0.02352	-4.44193527
SMARCA	-0.204743235	4.00849968	-2.42183506	0.016137	0.02356	-4.44369875
LCOR	-0.191675826	2.80189791	-2.420973076	0.016175	0.02362	-4.44573862
UNC5A	-0.072986883	0.22643583	-2.420786767	0.016183	0.02363	-4.44617943
ABCD2	0.09549971	0.33109323	2.420423349	0.016198	0.02365	-4.44703918
CDK13	-0.164773283	4.32817307	-2.420304608	0.016204	0.02365	-4.44732007
PIP4K2B	-0.162159923	5.56167681	-2.420255833	0.016206	0.02365	-4.44743544
FKTN	-0.183781713	3.05013265	-2.42013829	0.016211	0.02366	-4.44771347
EME2	-0.191137958	2.17744515	-2.419606122	0.016234	0.02369	-4.44897208
CRP	0.041984702	0.05186406	2.419221888	0.016251	0.02371	-4.44988065
UBA2	-0.15854181	5.69943545	-2.419179645	0.016253	0.02371	-4.44998053
SPINT1	0.271040327	7.55226405	2.418800011	0.016269	0.02374	-4.45087808
PRR5-AF	0.045874479	0.21786122	2.418457075	0.016284	0.02376	-4.45168874
BMPR1B	0.064951293	0.1730717	2.418003809	0.016304	0.02378	-4.45276005
HEPACA	-0.025753771	0.04784936	-2.417648832	0.01632	0.0238	-4.45359891
ABHD12	-0.141687533	7.16668209	-2.41714988	0.016341	0.02383	-4.45477781
GALNT4	0.058834541	0.26399006	2.416269057	0.01638	0.02389	-4.4568584
RNF31	-0.209965892	3.41724077	-2.41550239	0.016414	0.02394	-4.45866876
NIPBL	-0.185299211	4.26303796	-2.41476522	0.016446	0.02398	-4.46040895
SLC4A2	-0.200645986	4.84734403	-2.413125711	0.016519	0.02408	-4.46427741
AHCTF1	-0.181293331	3.87259588	-2.412789266	0.016534	0.0241	-4.46507095
STIMATE	-0.059627175	0.30100413	-2.41210888	0.016564	0.02415	-4.46667539
GPS2	-0.184187291	2.18513632	-2.412076662	0.016565	0.02415	-4.46675135
ACTR3	-0.16583417	5.02722371	-2.411514488	0.01659	0.02418	-4.46807669
BAIAP2L	-0.179618123	1.39078247	-2.410871719	0.016619	0.02422	-4.46959166
SORBS1	-0.206228461	1.80955228	-2.410604277	0.016631	0.02424	-4.4702219

TRIM65	-0.146409977	4.66673603	-2.410468962	0.016637	0.02424	-4.47054074
STPG3	-0.231605165	1.24020516	-2.409819069	0.016666	0.02428	-4.47207187
REC8	0.328160101	2.52905957	2.409614739	0.016675	0.02429	-4.47255319
P3H1	-0.151239376	3.67280395	-2.408957208	0.016704	0.02433	-4.47410179
IKBKE	-0.208770126	4.40507882	-2.408766358	0.016713	0.02434	-4.4745512
ELL2	-0.220078869	4.099337	-2.408533515	0.016723	0.02436	-4.47509944
ZNF681	-0.135578428	1.61674396	-2.407806771	0.016756	0.0244	-4.47681029
ZNF622	-0.176288813	6.02054832	-2.407413776	0.016774	0.02443	-4.47773525
RAB42	0.189806953	1.30852975	2.406970465	0.016793	0.02445	-4.47877846
LYPD1	0.27404762	1.08553428	2.406953251	0.016794	0.02445	-4.47881896
LRRC75/	-0.244707862	3.76727628	-2.406807677	0.016801	0.02446	-4.47916149
RAP1GD	-0.164878676	4.30554613	-2.406000243	0.016837	0.02451	-4.48106096
ARIH1	-0.154604372	3.27239191	-2.405872451	0.016843	0.02452	-4.48136153
PCOLCE	0.32950362	4.51825213	2.404702411	0.016896	0.02459	-4.48411281
PSORS1	0.218613837	1.49644236	2.40468841	0.016896	0.02459	-4.48414572
NBPF11	-0.186736112	2.40058499	-2.404532957	0.016903	0.0246	-4.48451116
ARRB2	0.181491306	3.89133887	2.404031138	0.016926	0.02463	-4.48569069
SEMA3A	0.189909056	1.05244181	2.403999012	0.016927	0.02463	-4.48576619
HCN3	-0.141785488	1.83193691	-2.403836222	0.016935	0.02464	-4.48614877
CYP1A1	-0.115807491	0.14906032	-2.403511715	0.01695	0.02466	-4.48691134
TUBB8P:	-0.098228866	0.37578342	-2.403331895	0.016958	0.02467	-4.48733386
P2RY2	0.240602702	3.95534283	2.402667686	0.016988	0.02471	-4.48889429
GLI2	0.135087563	0.50875272	2.402305758	0.017004	0.02473	-4.48974439
RAD54B	-0.054061127	0.3944525	-2.402138241	0.017012	0.02474	-4.49013782
YJEFN3	-0.267041814	1.67388198	-2.402001824	0.017018	0.02475	-4.49045818
MYBL2	0.25933227	1.80986461	2.401194363	0.017055	0.0248	-4.49235409
MAN2A1	-0.211824	3.46524189	-2.400992794	0.017064	0.02481	-4.49282728
CLCF1	0.329014466	4.31664673	2.400912149	0.017068	0.02481	-4.49301659
G3BP1	-0.160080141	4.73644327	-2.400408278	0.017091	0.02484	-4.49419923
MUC13	0.029538127	0.06345717	2.400301519	0.017096	0.02485	-4.49444977
RPS19	-0.187192438	8.94148932	-2.399496011	0.017132	0.0249	-4.49633982
PHF13	0.174416301	4.59413642	2.399302818	0.017141	0.02491	-4.49679304
TPM2	-0.295552204	4.93686201	-2.397614161	0.017219	0.02502	-4.50075303
GRM2	0.050267016	0.20914943	2.397458819	0.017226	0.02503	-4.50111718
NSG1	0.284674693	0.82830088	2.3964038	0.017274	0.0251	-4.50358976
ATP6V0I	0.231404477	0.91316031	2.3963703	0.017276	0.0251	-4.50366825
B3GALT4	-0.213690676	4.36019488	-2.396360972	0.017276	0.0251	-4.50369011
KDM5C	-0.173062438	5.47110903	-2.395506981	0.017316	0.02515	-4.50569075
SPINK7	0.057071501	0.1397093	2.395463576	0.017318	0.02515	-4.50579242
FAM90A:	-0.01750527	0.03385289	-2.395178186	0.017331	0.02517	-4.50646083

KCNK15	0.359498082	3.26723419	2.395147796	0.017332	0.02517	-4.50653201
THSD4	0.281425187	3.54503058	2.394782246	0.017349	0.02519	-4.50738805
SYTL4	-0.23541917	2.64615175	-2.394642067	0.017356	0.0252	-4.50771629
CAVIN4	-0.13019051	0.64619572	-2.394614182	0.017357	0.0252	-4.50778158
TMEM45	-0.297107176	3.95593836	-2.394388833	0.017367	0.02521	-4.5083092
C12orf56	0.121017491	0.40030621	2.393456475	0.017411	0.02527	-4.51049168
SUPT5H	-0.137520398	6.0687823	-2.393221286	0.017421	0.02529	-4.51104208
MED13L	-0.206163011	3.63882407	-2.392593403	0.017451	0.02532	-4.51251125
PIGR	0.301604127	1.33280571	2.392574294	0.017452	0.02532	-4.51255595
COL9A2	-0.328863198	3.24142683	-2.392002286	0.017478	0.02536	-4.51389405
EFNA2	0.059055547	0.14011665	2.391907251	0.017483	0.02537	-4.51411633
SOX15	-0.176287806	1.32092156	-2.390397857	0.017553	0.02547	-4.51764563
TET3	0.205598623	2.66666841	2.389837475	0.017579	0.0255	-4.51895539
HBA1	-0.170542317	0.50075592	-2.389366201	0.017601	0.02553	-4.52005665
HIF1AN	-0.137395447	3.43549493	-2.389155893	0.017611	0.02554	-4.52054803
RHOBTB	-0.169753489	5.47092591	-2.388563708	0.017639	0.02558	-4.52193142
PRIM1	-0.168707981	2.74766676	-2.388476597	0.017643	0.02559	-4.52213489
TYW1B	-0.233786223	1.91512283	-2.387864817	0.017672	0.02562	-4.52356367
ZNF700	-0.152368723	3.0457178	-2.387838891	0.017673	0.02562	-4.52362421
IL13	0.018635977	0.03037467	2.387826493	0.017673	0.02562	-4.52365316
CACHD1	-0.278895634	3.38151352	-2.387697482	0.01768	0.02563	-4.52395441
OR2L13	-0.143632398	0.3122875	-2.387542041	0.017687	0.02564	-4.52431735
GMFB	-0.177622945	4.18898738	-2.387093233	0.017708	0.02567	-4.52536516
CDC42SI	-0.18688141	6.11332787	-2.386543961	0.017734	0.0257	-4.52664725
SLITRK6	0.238897328	0.39401598	2.386264592	0.017747	0.02572	-4.52729924
ETS2	-0.191887152	5.72964589	-2.385821919	0.017768	0.02575	-4.52833219
NAT10	-0.126545921	4.73636073	-2.384743854	0.017819	0.02582	-4.53084704
NUP93	-0.163041887	3.81914093	-2.384743153	0.017819	0.02582	-4.53084867
ABCE1	-0.162781875	4.44908924	-2.384667697	0.017823	0.02582	-4.53102465
DOCK10	-0.260745128	2.36800048	-2.38466204	0.017823	0.02582	-4.53103784
ADAM23	0.1314151	0.3473772	2.383633763	0.017872	0.02589	-4.53343545
ITGA9	0.331374039	5.78971714	2.383004439	0.017901	0.02593	-4.53490234
IQGAP1	0.253828574	6.49785289	2.382615508	0.01792	0.02595	-4.53580872
SLC24A1	-0.175699691	2.72902157	-2.382305922	0.017935	0.02597	-4.53653008
MAP1S	0.158334499	4.86100291	2.381317374	0.017982	0.02604	-4.5388329
CTNS	-0.149999262	4.0928001	-2.381194678	0.017988	0.02604	-4.53911865
ELOVL5	-0.21804797	5.69793063	-2.380816708	0.018006	0.02607	-4.53999884
CPS1	-0.191477395	0.73943885	-2.379911381	0.018049	0.02613	-4.54210657
ASCL5	0.160072589	0.73042226	2.379543377	0.018067	0.02615	-4.54296311
CEACAM	-0.212196572	1.64593964	-2.379381936	0.018074	0.02616	-4.54333883

HYPK	-0.097105285	0.8442846	-2.379141771	0.018086	0.02617	-4.54389772
GFI1	0.180790455	0.89643369	2.377249697	0.018177	0.0263	-4.54829889
IL1B	0.283093802	1.55066698	2.375832416	0.018245	0.0264	-4.55159344
ZNF432	-0.157471934	3.18972469	-2.375740211	0.01825	0.0264	-4.55180771
ZZEF1	-0.172825542	3.56177211	-2.375477941	0.018262	0.02642	-4.55241715
YTHDC2	-0.167411218	3.13102596	-2.374534675	0.018308	0.02648	-4.55460847
FNBP1	0.188618331	5.0097121	2.374306389	0.018319	0.0265	-4.55513868
SPACA6	-0.26081202	2.98928912	-2.374109226	0.018329	0.02651	-4.55559657
DDX60L	0.168512235	2.3929953	2.373484565	0.018359	0.02655	-4.55704702
TAL2	-0.053886143	0.17897063	-2.373230615	0.018372	0.02657	-4.55763659
HCAR3	0.077776333	0.20517394	2.373171128	0.018374	0.02657	-4.55777469
PLD5	-0.088488096	0.0908173	-2.372508371	0.018407	0.02661	-4.55931301
ZNF441	-0.194612451	3.24057534	-2.371095357	0.018476	0.02671	-4.56259138
REM2	-0.136393376	0.79358452	-2.370818297	0.018489	0.02673	-4.56323397
LTA4H	-0.147994178	5.65527718	-2.370289186	0.018515	0.02676	-4.56446095
ZDHHC2	-0.17414867	3.28028085	-2.370265203	0.018516	0.02676	-4.56451656
CLSTN3	-0.209431652	4.78783354	-2.369781435	0.01854	0.02679	-4.56563816
SMG9	-0.144575397	3.72650479	-2.369427131	0.018557	0.02682	-4.56645946
WBP2NL	-0.13198041	0.81411684	-2.369165443	0.01857	0.02683	-4.56706599
STON2	-0.229386857	3.06668824	-2.369085336	0.018574	0.02684	-4.56725165
MARF1	-0.178502958	4.67938549	-2.368976047	0.01858	0.02684	-4.56750493
DNAJB5	-0.265318967	3.84515802	-2.368890527	0.018584	0.02685	-4.56770312
CAMSAF	0.200247457	3.45806707	2.368810604	0.018588	0.02685	-4.56788833
RUBCNL	0.257194724	1.9997159	2.367964119	0.018629	0.02691	-4.56984958
KRT81	-0.196861004	1.55760002	-2.367300606	0.018662	0.02695	-4.57138642
HOXB8	-0.080216257	0.12211965	-2.366830692	0.018685	0.02698	-4.57247459
CTNNA1	-0.185071345	7.27622634	-2.366268203	0.018713	0.02702	-4.57377687
TNFRSF	0.257158065	0.79928091	2.365078635	0.018772	0.0271	-4.57652999
MAP3K2	-0.180418252	1.93666324	-2.364830212	0.018784	0.02712	-4.57710477
MGAT5	-0.204727389	4.60720598	-2.364005035	0.018825	0.02718	-4.57901358
CPSF7	-0.170657396	5.5380768	-2.363284328	0.018861	0.02722	-4.5806802
SORCS1	-0.38415457	1.29697062	-2.363267952	0.018862	0.02722	-4.58071807
RFX1	-0.156670451	4.17857405	-2.36254982	0.018898	0.02727	-4.58237824
ITPKB	-0.18707558	4.40080456	-2.361620667	0.018944	0.02734	-4.58452554
HES6	-0.407448189	4.10160753	-2.361195252	0.018965	0.02736	-4.58550841
RAD50	-0.183995363	4.27448035	-2.361169815	0.018967	0.02736	-4.58556718
AEBP2	-0.173627362	4.41322583	-2.361161518	0.018967	0.02736	-4.58558635
MYO9A	-0.190330622	2.92046099	-2.360956556	0.018977	0.02738	-4.58605982
VNN2	0.206324363	0.92831381	2.360905258	0.01898	0.02738	-4.58617832
CA8	-0.177006402	0.36485967	-2.360226488	0.019014	0.02743	-4.58774601

CCDC13(	-0.095791582	0.65761485	-2.359538851	0.019048	0.02747	-4.58933374
DAAM1	-0.144168205	3.1038266	-2.359435574	0.019053	0.02748	-4.58957216
DNAJC5(	-0.112495465	0.22773282	-2.359351742	0.019058	0.02748	-4.58976569
FCHSD1	0.234030004	3.47071685	2.357742364	0.019139	0.0276	-4.59347967
FIBIN	-0.449256703	2.52665471	-2.357468674	0.019152	0.02761	-4.59411103
MIPOL1	-0.113703511	1.07749431	-2.357177917	0.019167	0.02763	-4.59478168
CLGN	0.24598672	0.66011758	2.356146635	0.019219	0.02771	-4.59715976
PPIP5K1	-0.131283413	3.05704629	-2.355915262	0.019231	0.02772	-4.59769316
THAP10	-0.156710045	2.79756727	-2.355593513	0.019247	0.02774	-4.59843482
CCDC93	-0.178946748	3.97205438	-2.355374958	0.019258	0.02775	-4.59893855
IRF9	0.212762257	2.13198402	2.354977575	0.019279	0.02778	-4.59985434
NIPAL2	0.200633834	3.5703405	2.354849493	0.019285	0.02779	-4.60014948
MEX3C	0.217971977	4.29586288	2.353901331	0.019333	0.02786	-4.60233386
RHCG	-0.086622675	0.13677585	-2.353400818	0.019359	0.02789	-4.60348661
AKAP13	0.227902273	4.96653902	2.352999237	0.019379	0.02792	-4.60441133
CAMK2B	-0.22189801	4.19437084	-2.352910126	0.019384	0.02792	-4.60461651
ASTN1	-0.189640459	0.30249584	-2.351955601	0.019432	0.02799	-4.60681381
YIPF7	-0.043506428	0.19536365	-2.351465675	0.019457	0.02802	-4.60794128
CAV2	0.327853818	6.2045313	2.350841789	0.019489	0.02807	-4.60937671
MTM1	-0.214357379	4.2361724	-2.350808955	0.019491	0.02807	-4.60945224
B3GALT:	-0.041182326	0.13543604	-2.350042436	0.01953	0.02812	-4.61121531
CD248	-0.296460015	4.63843896	-2.349852306	0.01954	0.02813	-4.61165255
DIP2B	-0.184013737	3.47310981	-2.349277841	0.01957	0.02817	-4.61297341
TNPO2	-0.135967187	5.28012837	-2.348836485	0.019592	0.0282	-4.61398801
VPREB3	-0.311854848	1.48662252	-2.347822281	0.019645	0.02828	-4.61631879
ARHGAF	-0.156672634	1.46566023	-2.347615213	0.019655	0.02829	-4.61679455
MMP21	-0.060438056	0.16093625	-2.347513153	0.019661	0.02829	-4.61702902
CCDC19(	-0.064783498	0.0906979	-2.347232668	0.019675	0.02831	-4.61767336
NCAPH	0.16642083	1.16664838	2.347054742	0.019684	0.02832	-4.61808206
CHAD	-0.253858029	1.63775838	-2.346704994	0.019702	0.02835	-4.61888536
TMEM21	-0.090863071	0.07137522	-2.34619303	0.019729	0.02838	-4.62006102
SOGA1	-0.261293722	4.34953235	-2.346018666	0.019738	0.02839	-4.62046137
ITPRIP	-0.212808616	4.71910117	-2.34463846	0.01981	0.02849	-4.62362939
CLEC2L	-0.106990381	0.20414345	-2.34411932	0.019837	0.02853	-4.62482053
CR1L	0.052352892	0.14217566	2.343845811	0.019851	0.02855	-4.62544798
LRRC40	-0.169889069	3.58554224	-2.343713534	0.019858	0.02855	-4.6257514
PPP2R3I	-0.153323919	2.32245946	-2.343374383	0.019875	0.02858	-4.6265293
RUFY2	-0.150451989	2.74391266	-2.343023188	0.019894	0.0286	-4.6273347
CYLD	-0.165371222	4.12071171	-2.34209694	0.019942	0.02867	-4.62945834
FBXL7	-0.185470057	2.5912698	-2.342054932	0.019944	0.02867	-4.62955464

GBP6	0.151442812	0.61502868	2.341440008	0.019976	0.02871	-4.63096402
APELA	-0.148714205	0.17576093	-2.341164242	0.019991	0.02873	-4.63159595
SCGB3A	-0.470203553	2.38803378	-2.340828347	0.020008	0.02875	-4.63236558
OXTR	0.125190313	0.82976176	2.340629035	0.020019	0.02877	-4.6328222
NLRP11	-0.039448219	0.05360929	-2.340368145	0.020033	0.02878	-4.63341985
SETD9	-0.179301399	3.10709845	-2.340200021	0.020041	0.02879	-4.63380495
GTF2IRD	0.185673906	4.92753311	2.339986958	0.020053	0.02881	-4.63429296
SIPA1L2	0.252097313	4.35079227	2.339417138	0.020083	0.02885	-4.63559788
RGS22	-0.055261613	0.15533801	-2.339083571	0.0201	0.02887	-4.63636162
TCAP	-0.20452732	1.50229361	-2.33905669	0.020102	0.02887	-4.63642316
F2R	-0.290726117	4.95888572	-2.339026217	0.020103	0.02887	-4.63649293
CRKL	-0.173218855	5.20196165	-2.338898054	0.02011	0.02888	-4.63678634
ITIH3	-0.091731959	0.37583824	-2.338726089	0.020119	0.02889	-4.63718
MAN2A2	-0.224080875	4.30334241	-2.338222146	0.020146	0.02892	-4.63833348
FFAR3	-0.044846195	0.09752498	-2.338085402	0.020153	0.02893	-4.63864643
AQP6	-0.143162172	0.37441543	-2.337757839	0.02017	0.02896	-4.63939602
HOXC8	0.128701902	0.1900336	2.336796346	0.020221	0.02903	-4.6415957
RBFOX2	-0.206806297	4.72614787	-2.33632835	0.020246	0.02906	-4.64266606
C1orf198	-0.219928381	5.28147606	-2.335685325	0.02028	0.0291	-4.64413639
FBLN5	-0.27253967	3.88493244	-2.335658357	0.020281	0.0291	-4.64419805
CCL21	-0.80948599	3.3845882	-2.33490213	0.020321	0.02916	-4.64592671
ERP27	0.443760529	4.2710194	2.33476801	0.020328	0.02917	-4.64623324
SLC25A4	-0.174034766	0.82332908	-2.334383824	0.020349	0.02919	-4.6471112
ZNF792	-0.145944558	2.1299436	-2.334270001	0.020355	0.0292	-4.64737129
C1QTNF	0.288115329	4.62595223	2.334230451	0.020357	0.0292	-4.64746166
ETV3	-0.189468128	3.58280298	-2.334131789	0.020362	0.02921	-4.64768709
PGBD5	-0.266602742	2.55791684	-2.333668195	0.020387	0.02924	-4.64874622
VPS35	-0.149673148	5.17285892	-2.333582964	0.020392	0.02924	-4.64894092
ZNF721	-0.143511076	3.47434707	-2.332787323	0.020434	0.0293	-4.65075811
BRD1	-0.173604495	4.36473532	-2.33197108	0.020478	0.02936	-4.65262175
TGFA	0.359887163	5.36294102	2.331882384	0.020482	0.02937	-4.65282422
MESP2	-0.160564727	0.6293462	-2.330790069	0.020541	0.02945	-4.65531711
PKD1L3	0.02452545	0.10856459	2.33068704	0.020546	0.02945	-4.65555218
GLYATL1	0.035842845	0.08697005	2.330582604	0.020552	0.02946	-4.65579045
OCLN	-0.312683552	4.16614917	-2.33040049	0.020562	0.02947	-4.65620593
ZBED2	-0.391110642	7.11364103	-2.330211798	0.020572	0.02948	-4.65663639
RASA1	0.285587628	5.39047938	2.330193188	0.020573	0.02948	-4.65667884
PMM2	-0.149993235	1.86805385	-2.329508275	0.02061	0.02953	-4.65824099
ARHGAP	-0.20442398	5.08705227	-2.329035294	0.020635	0.02956	-4.65931952
RAB27A	0.340195157	6.04996746	2.329029307	0.020636	0.02956	-4.65933317

WLS	0.237416918	4.77871344	2.32876662	0.02065	0.02958	-4.65993207
CARD10	-0.171494503	3.04472375	-2.32789838	0.020697	0.02965	-4.66191113
CENPI	0.07346998	0.46670908	2.326287877	0.020784	0.02977	-4.66558022
ARL9	-0.211879316	1.45881072	-2.325998123	0.020799	0.02979	-4.66624008
TAS2R3	0.038324247	0.11386218	2.325081157	0.020849	0.02986	-4.6683278
NPAS3	0.190306968	2.14175077	2.325067188	0.02085	0.02986	-4.6683596
OR7C1	-0.047709752	0.16673441	-2.324327436	0.02089	0.02991	-4.67004326
ARGLU1	-0.231584386	5.52110223	-2.323968805	0.02091	0.02994	-4.67085931
OTUD6B	-0.156213399	2.95462729	-2.323453887	0.020938	0.02997	-4.67203077
OPRL1	-0.158205808	1.46357598	-2.323164918	0.020954	0.02999	-4.67268808
TRAF7	-0.160950168	5.61346313	-2.321554852	0.021042	0.03012	-4.67634902
TDG	-0.15155835	3.21233429	-2.321272804	0.021057	0.03014	-4.67699009
TTC28	-0.163345948	3.37490707	-2.320886634	0.021078	0.03016	-4.67786769
BCL2L14	0.062957967	0.15470015	2.319699479	0.021144	0.03025	-4.68056472
RIN2	-0.204218505	5.12615752	-2.319679714	0.021145	0.03025	-4.68060961
LRP4	0.519832356	5.45546389	2.318780713	0.021194	0.03032	-4.6826511
MEIS3	0.166635397	1.39352912	2.31867676	0.0212	0.03033	-4.68288712
CLK2	-0.16822738	5.05276526	-2.318366392	0.021217	0.03035	-4.68359171
EXPH5	0.166306383	1.5789226	2.317700854	0.021254	0.0304	-4.68510229
IGFLR1	0.199539911	1.50134897	2.317367536	0.021272	0.03042	-4.68585868
CREBZF	-0.214905388	4.74384649	-2.317186871	0.021282	0.03044	-4.68626861
MBOAT4	-0.055951818	0.27588825	-2.317065527	0.021289	0.03044	-4.68654392
LOXL3	0.153375672	1.53991747	2.316724325	0.021308	0.03047	-4.68731799
RBM12	-0.188982669	4.81738052	-2.316126987	0.021341	0.03051	-4.68867288
OR56B4	-0.02942818	0.09110711	-2.31582672	0.021358	0.03053	-4.68935382
PNPLA3	0.064970042	0.18926103	2.315426818	0.02138	0.03056	-4.69026059
EPB41L2	-0.205742151	3.4882732	-2.315200485	0.021392	0.03058	-4.69077372
SGCE	-0.303327673	4.67570861	-2.314990681	0.021404	0.03059	-4.69124934
CYP4Z1	0.084122813	0.22131799	2.314842649	0.021412	0.0306	-4.6915849
TMEM20	0.046297185	0.15633613	2.31319172	0.021504	0.03073	-4.69532582
KCNG2	-0.043805228	0.08784426	-2.313084575	0.02151	0.03074	-4.69556852
LCK	0.326263353	2.34244238	2.31296452	0.021517	0.03074	-4.69584044
TPP1	0.177755475	6.86536309	2.312716511	0.021531	0.03076	-4.69640215
POLQ	0.055986892	0.28254585	2.311970975	0.021573	0.03082	-4.69809032
MSH5-SA	-0.10488664	0.68417356	-2.311321471	0.021609	0.03087	-4.69956062
RAB21	-0.138147863	4.10311356	-2.310745684	0.021641	0.03091	-4.70086371
ZNF333	-0.135394505	1.94427413	-2.310732119	0.021642	0.03091	-4.70089441
CCL15	-0.116745216	0.17534173	-2.310646568	0.021647	0.03091	-4.701088
LRRC8E	0.088662155	0.48789624	2.310423779	0.02166	0.03093	-4.7015921
PRELP	0.406228799	3.07578056	2.309798718	0.021695	0.03098	-4.70300616

RPS8	-0.164621829	9.97546665	-2.309240994	0.021726	0.03102	-4.70426758
PKDREJ	-0.029504734	0.13740054	-2.308415233	0.021773	0.03108	-4.70613469
MYO1A	-0.059868221	0.23136086	-2.307532322	0.021823	0.03115	-4.70813032
POGLUT	-0.175413775	4.05879141	-2.307319571	0.021835	0.03117	-4.70861108
MBNL2	-0.166077308	5.08472965	-2.306302202	0.021892	0.03124	-4.7109095
KCNN1	-0.150522924	0.29775395	-2.306113975	0.021903	0.03126	-4.71133464
PRRX1	0.312747811	2.8689216	2.305398103	0.021944	0.03131	-4.71295121
RASGRF	-0.195390201	1.54241923	-2.304663385	0.021985	0.03137	-4.71460985
NPAP1	-0.08608743	0.2369341	-2.30438503	0.022001	0.03139	-4.71523811
SHISAL2	-0.020445084	0.04454554	-2.303980471	0.022024	0.03142	-4.71615108
MED15	0.152685665	4.77784032	2.303965215	0.022025	0.03142	-4.7161855
HPS1	-0.124836658	5.5461403	-2.303289017	0.022064	0.03147	-4.71771114
GIPR	0.173436535	1.03923413	2.300766609	0.022208	0.03167	-4.72339838
ABCB7	-0.166320565	4.37985575	-2.299881834	0.022259	0.03174	-4.72539186
KLF11	-0.213150102	3.79087383	-2.298693691	0.022328	0.03184	-4.7280677
LCN8	-0.265360203	0.95788204	-2.298566054	0.022335	0.03185	-4.72835507
THOC3	-0.178412894	3.51136876	-2.298242129	0.022354	0.03187	-4.72908432
MTRNR2	0.214606332	1.33191854	2.297162241	0.022416	0.03196	-4.73151474
DCP1A	-0.167513652	3.79552939	-2.296827783	0.022436	0.03198	-4.73226726
BHLHE4	0.346660623	7.39946274	2.296098327	0.022478	0.03204	-4.73390814
ATXN7L2	-0.150579711	2.67308354	-2.296000841	0.022484	0.03205	-4.73412739
GPD2	-0.201655848	4.55976634	-2.295541959	0.02251	0.03208	-4.73515933
CCDC20	-0.112356336	0.99839548	-2.295284861	0.022525	0.0321	-4.73573741
PCDHGA	-0.039532779	0.16611975	-2.2952743	0.022526	0.0321	-4.73576116
ATP4B	-0.146942991	1.03011256	-2.293712982	0.022617	0.03222	-4.73927039
AFF3	0.177341517	0.53155116	2.293338882	0.022639	0.03225	-4.74011088
GPR18	0.178057803	0.64268548	2.292930617	0.022663	0.03228	-4.74102798
SLC5A12	-0.035549391	0.09637243	-2.291627713	0.022739	0.03239	-4.74395368
DOCK11	0.252804061	4.42465601	2.291509419	0.022746	0.0324	-4.74421924
STX17	-0.202551134	3.20498154	-2.291455411	0.022749	0.0324	-4.74434047
PTPRK	0.216093846	4.03160102	2.291295607	0.022758	0.03241	-4.74469918
KCNA1	-0.065146051	0.09445047	-2.291264532	0.02276	0.03241	-4.74476893
MGAT4A	-0.157831453	3.45478782	-2.291203738	0.022764	0.03241	-4.74490539
INSIG1	-0.163780022	4.26863499	-2.288947188	0.022897	0.0326	-4.74996786
DLGAP5	0.176269605	1.01283162	2.288877536	0.022901	0.0326	-4.75012404
HAPLN1	-0.226922553	1.0612301	-2.288197608	0.022941	0.03266	-4.75164845
CUL9	-0.136779446	3.54160463	-2.287942224	0.022956	0.03267	-4.75222091
BPIFA1	0.066243723	0.14802435	2.287825796	0.022963	0.03268	-4.75248187
FOXL1	0.122106778	0.73778882	2.287499267	0.022982	0.03271	-4.75321368
SQSTM1	-0.168069071	7.50603191	-2.287100823	0.023006	0.03274	-4.75410654

RHOBTB	0.246467129	4.9723031	2.286980644	0.023013	0.03275	-4.75437581
FNDC3A	-0.217498271	5.5001994	-2.286360631	0.02305	0.03279	-4.7557648
CYP4B1	0.43804296	4.23700866	2.285653287	0.023092	0.03285	-4.75734899
DESI2	-0.170443679	4.85211684	-2.285214996	0.023118	0.03289	-4.75833036
CCL26	0.175878094	0.93714091	2.281613298	0.023333	0.03319	-4.76638805
PSD	-0.177176654	1.68676196	-2.281544305	0.023337	0.03319	-4.76654228
KSR2	-0.147792514	1.28439937	-2.281003579	0.023369	0.03324	-4.7677509
RAET1L	0.09827016	0.3255194	2.280777465	0.023383	0.03325	-4.76825622
HLA-DMI	-0.230964288	5.99094746	-2.280283977	0.023413	0.03329	-4.76935891
FAM91A:	-0.170215313	4.45716579	-2.27981019	0.023441	0.03333	-4.77041736
SPATA3:	0.077406252	0.4974758	2.279598806	0.023454	0.03334	-4.77088953
C2CD4D	-0.128985466	1.10412888	-2.279576812	0.023455	0.03334	-4.77093866
MIA	0.110508605	0.29819095	2.27945897	0.023462	0.03335	-4.77120186
CPNE6	-0.10791773	0.13907659	-2.279201748	0.023478	0.03337	-4.77177633
SLFN14	0.019408242	0.04017103	2.279153722	0.023481	0.03337	-4.77188358
COX8C	-0.04816553	0.14051861	-2.278930583	0.023494	0.03339	-4.77238187
VSTM4	-0.392381115	5.63774635	-2.277342212	0.02359	0.03352	-4.77592748
CHST2	0.340227044	5.67706416	2.276730309	0.023627	0.03357	-4.77729275
BCL7C	-0.178189116	5.35944734	-2.27585583	0.02368	0.03364	-4.77924327
FLG2	0.014791071	0.02419881	2.27573597	0.023688	0.03365	-4.77951056
KLRK1	0.095154043	0.27227851	2.274754364	0.023748	0.03373	-4.78169906
KLRC4-K	0.095154043	0.27227851	2.274754364	0.023748	0.03373	-4.78169906
FAM92A	-0.122906222	2.88424915	-2.274224413	0.02378	0.03377	-4.78288021
ZNF682	-0.238352469	2.928579	-2.274158114	0.023784	0.03378	-4.78302796
PCYOX1	-0.140028641	2.30699414	-2.274078694	0.023789	0.03378	-4.78320494
PIK3CA	0.192634863	3.11044589	2.273996	0.023794	0.03379	-4.78338921
AP3S1	-0.144504422	5.53664702	-2.272540813	0.023883	0.03391	-4.78663086
GPR183	0.36871648	4.01753396	2.272252541	0.0239	0.03393	-4.78727279
LDHAL6I	0.027836209	0.08799675	2.271473899	0.023948	0.034	-4.7890063
TIMELES	-0.162078075	3.4612442	-2.27121721	0.023964	0.03402	-4.78957765
NUDT2	-0.173283544	3.84864768	-2.271025606	0.023976	0.03403	-4.79000409
ANKZF1	-0.165435761	3.96447228	-2.270238419	0.024024	0.0341	-4.79175571
ABCC10	-0.137571439	3.31590472	-2.269860174	0.024047	0.03412	-4.79259716
KYNU	0.101511131	0.56117341	2.269847213	0.024048	0.03412	-4.79262599
PHF19	0.169150242	3.20969307	2.26946427	0.024072	0.03415	-4.79347775
ARHGDI	0.080945911	0.20633829	2.269400106	0.024076	0.03416	-4.79362045
FOLR2	0.296215367	3.93679748	2.268562423	0.024127	0.03423	-4.79548313
ARSH	-0.058725725	0.20966167	-2.267458661	0.024196	0.03432	-4.79793647
BMI1	-0.158768878	4.86412894	-2.267363551	0.024201	0.03433	-4.79814781
CTAGE8	-0.09538575	0.40642723	-2.267248079	0.024209	0.03433	-4.79840439

PDGFRL	-0.252650544	5.96101601	-2.266000258	0.024286	0.03444	-4.8011763
EOMES	0.216455486	0.68939258	2.265642556	0.024308	0.03447	-4.80197062
DIS3	-0.151713778	3.74343036	-2.265445488	0.024321	0.03448	-4.80240819
FLG	0.09610028	0.32399194	2.265386965	0.024324	0.03449	-4.80253813
S1PR4	0.231601362	2.28614097	2.265316554	0.024329	0.03449	-4.80269445
LILRA2	0.122019459	0.84850695	2.263603514	0.024435	0.03464	-4.8064963
LRFN3	-0.163294174	3.3102265	-2.263471476	0.024444	0.03465	-4.80678922
H1FNT	-0.058369587	0.18544071	-2.262810069	0.024485	0.0347	-4.8082563
TFPI	-0.171960773	1.70081142	-2.262733604	0.02449	0.03471	-4.80842588
FMR1NB	-0.045268919	0.15356796	-2.262640713	0.024496	0.03471	-4.80863188
FLVCR2	0.193392905	2.82769246	2.262454972	0.024507	0.03473	-4.80904377
PRDM8	0.107429836	0.46706616	2.262008452	0.024535	0.03476	-4.81003382
NAA80	-0.140827532	4.02855535	-2.261763541	0.02455	0.03478	-4.81057678
RPL36A	-0.171736504	5.3765514	-2.261107192	0.024592	0.03484	-4.81203158
AKT3	0.203842299	4.3438789	2.259721789	0.024679	0.03496	-4.81510101
MCM4	0.208924374	4.09101077	2.259527843	0.024691	0.03497	-4.81553056
RPLP0	-0.1716316	9.5700127	-2.259053184	0.024721	0.03501	-4.81658168
ITPKC	0.185894674	5.30017507	2.25883269	0.024735	0.03503	-4.81706989
TMEFF1	-0.087434662	0.35413879	-2.258399733	0.024762	0.03506	-4.8180284
MMP28	-0.240140088	3.4776846	-2.258111732	0.02478	0.03509	-4.81866589
ADPGK	-0.132841127	4.66634035	-2.257262393	0.024834	0.03516	-4.82054546
PHF20	-0.148934108	3.89014422	-2.257157813	0.024841	0.03517	-4.82077685
ATAD3B	-0.173484556	2.7068357	-2.257043547	0.024848	0.03517	-4.82102965
AURKB	0.210454811	1.42628764	2.256862248	0.02486	0.03519	-4.82143074
CEP104	-0.153661759	3.8703825	-2.256799994	0.024863	0.03519	-4.82156845
STK32A	0.352147437	4.42042204	2.256534324	0.02488	0.03521	-4.82215612
C1orf112	0.11805116	1.46729092	2.256419428	0.024888	0.03522	-4.82241025
CNFN	0.294822673	3.44302866	2.256310346	0.024895	0.03522	-4.82265151
TSTD3	-0.057300872	0.28708081	-2.255970153	0.024916	0.03525	-4.82340386
BCL6	0.204294024	4.2195661	2.255627327	0.024938	0.03528	-4.82416192
ZC3H4	-0.143197687	4.6611063	-2.255538239	0.024944	0.03529	-4.82435889
TWIST1	0.240892061	1.65822186	2.255417548	0.024951	0.03529	-4.82462572
RFPL2	-0.051933305	0.13252734	-2.254414984	0.025015	0.03538	-4.82684176
GLIS1	-0.179266351	0.66892575	-2.254108906	0.025035	0.0354	-4.82751812
ZBBX	-0.041166681	0.07439362	-2.254070215	0.025037	0.03541	-4.82760361
NPC2	0.281570118	10.8101586	2.252888236	0.025113	0.03551	-4.83021462
BAG6	-0.124965605	7.24263366	-2.252785105	0.025119	0.03552	-4.83044237
SRSF11	-0.189406773	5.67146793	-2.252263274	0.025153	0.03556	-4.83159464
HECW1	-0.110898164	0.18051777	-2.252256471	0.025153	0.03556	-4.83160966
FOXK2	-0.116579883	4.26681445	-2.251719212	0.025187	0.0356	-4.83279572

GALNT1:	0.107571067	0.19655971	2.251643535	0.025192	0.03561	-4.83296276
CEP164	-0.149522306	3.23474617	-2.251436424	0.025206	0.03562	-4.83341989
XCL2	0.285070079	1.25538326	2.251082991	0.025228	0.03565	-4.83419989
KDM2A	-0.159526627	5.2135829	-2.250824808	0.025245	0.03567	-4.83476961
ATF5	-0.270496691	5.6736442	-2.250104681	0.025291	0.03574	-4.83635833
GLCCI1	-0.270054347	4.91289796	-2.24989533	0.025305	0.03575	-4.83682011
ADRA1A	-0.030368484	0.08188868	-2.249795382	0.025311	0.03576	-4.83704055
ARPIN-A	-0.06457397	0.40790276	-2.24949746	0.02533	0.03578	-4.83769759
AMER2	-0.080110724	0.07040606	-2.2488659	0.025371	0.03584	-4.83909016
ACSM3	-0.167464239	1.24479245	-2.24844462	0.025398	0.03587	-4.84001886
C2CD3	-0.152737167	3.26804682	-2.247818069	0.025439	0.03593	-4.84139976
NR0B2	-0.184736602	0.40369427	-2.247726316	0.025445	0.03593	-4.84160195
TASOR2	-0.197538927	3.86057395	-2.247703396	0.025446	0.03593	-4.84165246
RPH3A	-0.097245968	0.14907698	-2.247424902	0.025464	0.03595	-4.84226611
RBP2	-0.101338822	0.31619446	-2.247095874	0.025485	0.03598	-4.84299101
ATG16L1	-0.157438353	4.21981207	-2.247061727	0.025488	0.03598	-4.84306623
CAPN1	-0.164443499	6.75232246	-2.246813967	0.025504	0.036	-4.84361201
STAT5B	-0.158698009	5.44647245	-2.245934545	0.025561	0.03608	-4.84554878
CYP7A1	-0.015305535	0.04302824	-2.245629067	0.025581	0.0361	-4.84622137
TENT2	-0.149176359	4.85997333	-2.2453894	0.025596	0.03612	-4.846749
MMP1	0.256060104	1.03143445	2.244915203	0.025627	0.03616	-4.84779279
HS3ST3/	0.106380027	0.30168887	2.24407325	0.025682	0.03624	-4.84964554
CFAP73	-0.128332873	0.77985129	-2.244034701	0.025684	0.03624	-4.84973036
MEF2A	-0.201762966	4.3338116	-2.243441193	0.025723	0.03629	-4.85103598
CAMK2A	0.044461433	0.07748504	2.242703883	0.025771	0.03635	-4.85265748
NOC3L	-0.166680613	3.95628166	-2.241264214	0.025866	0.03648	-4.85582214
INTS6	-0.124487191	2.43501217	-2.240044951	0.025946	0.03659	-4.85850077
FOXR1	-0.094815712	0.10322996	-2.239687247	0.025969	0.03662	-4.85928636
ANKIB1	-0.185360994	4.35188493	-2.238629436	0.026039	0.03672	-4.8616088
OSTM1	-0.154930993	4.19628942	-2.238064174	0.026076	0.03677	-4.86284941
GUCY1A	-0.145723155	1.09858578	-2.237943	0.026084	0.03678	-4.86311532
KCNC3	-0.289983684	4.02433743	-2.237522877	0.026112	0.03681	-4.86403715
POMK	0.224003215	1.60167528	2.236409637	0.026186	0.03691	-4.86647899
RNF151	-0.035309055	0.1109532	-2.236240089	0.026197	0.03693	-4.86685078
ARMCX6	-0.244932767	4.79715045	-2.235851221	0.026223	0.03696	-4.8677034
GRHL2	-0.20352719	4.23425478	-2.235125452	0.026271	0.03702	-4.86929433
LRRC31	-0.03391915	0.06443965	-2.234747393	0.026296	0.03706	-4.87012287
TBK1	-0.138737664	4.03035394	-2.23338617	0.026387	0.03718	-4.87310492
SHISAL1	0.121090368	0.43273957	2.232700314	0.026433	0.03724	-4.87460678
SEPTIN1	0.228820356	4.5131384	2.23141764	0.026519	0.03736	-4.87741433

TTC14	-0.230108132	3.77922513	-2.230676574	0.026569	0.03743	-4.87903569
OVCH1	-0.072265467	0.18631957	-2.228853734	0.026691	0.0376	-4.88302162
MFSD6L	-0.30683028	3.99415536	-2.228742452	0.026699	0.0376	-4.88326486
MAP4K5	-0.167647373	3.9572738	-2.227598936	0.026776	0.03771	-4.88576362
ADAP1	-0.169724199	2.88346204	-2.227520159	0.026781	0.03771	-4.88593572
MAEL	-0.090492649	0.21448031	-2.227503381	0.026783	0.03771	-4.88597237
SGPP2	-0.358275822	4.23499741	-2.226612983	0.026843	0.0378	-4.88791709
EPDR1	-0.274218897	5.44769002	-2.22641827	0.026856	0.03781	-4.88834227
CAMP	-0.356168499	1.61064462	-2.225468191	0.026921	0.0379	-4.89041635
RNF168	-0.142782795	3.26155658	-2.224374315	0.026995	0.038	-4.89280329
NRTN	-0.194538518	1.09715935	-2.224201881	0.027007	0.03801	-4.89317945
MYO16	-0.042341123	0.07980611	-2.223302289	0.027068	0.0381	-4.89514145
PPP2R3/	0.164005103	2.55583803	2.222893983	0.027096	0.03813	-4.89603171
TSN	-0.166965765	5.80391516	-2.222626271	0.027114	0.03816	-4.89661534
RNF152	-0.193517632	1.69675796	-2.222528117	0.027121	0.03816	-4.8968293
ME3	0.2106206	3.77840207	2.222089003	0.027151	0.0382	-4.89778641
ZNF155	-0.158597267	1.7868095	-2.221804851	0.02717	0.03822	-4.89840566
NFAM1	-0.261643407	2.81986621	-2.221650451	0.027181	0.03824	-4.89874211
GNG4	-0.12413449	0.51593796	-2.220859663	0.027235	0.03831	-4.90046494
ZNF615	-0.165789926	3.00003292	-2.220384319	0.027268	0.03835	-4.90150026
MAP3K1:	0.155435824	2.32192368	2.219837126	0.027305	0.0384	-4.9026918
SLC5A1(	-0.028442484	0.11732647	-2.219734843	0.027313	0.03841	-4.9029145
SF3B3	-0.158273515	5.21030649	-2.219424445	0.027334	0.03844	-4.90359025
ZWINT	-0.187217041	3.69746838	-2.219303292	0.027342	0.03844	-4.90385399
MRLN	-0.025842145	0.04285416	-2.219030172	0.027361	0.03847	-4.90444847
RSBN1	-0.163470645	3.62647507	-2.218321606	0.02741	0.03853	-4.90599046
NBAS	-0.18831475	4.48146484	-2.217971125	0.027434	0.03856	-4.906753
RPL7A	-0.153251792	10.6856301	-2.217933862	0.027437	0.03856	-4.90683407
EGLN2	-0.155028785	3.58302126	-2.217779862	0.027447	0.03858	-4.90716908
BTBD8	0.14908632	1.00919207	2.217060572	0.027497	0.03864	-4.90873355
TNRC6A	-0.166471603	3.71326993	-2.216630535	0.027527	0.03868	-4.90966865
CHST13	0.096423526	0.49469752	2.216289391	0.02755	0.03871	-4.91041034
SECISBF	-0.203408298	4.75694748	-2.216196093	0.027557	0.03872	-4.91061316
GAREM2	-0.157060425	2.06638277	-2.216018297	0.027569	0.03873	-4.91099965
CASP3	-0.15014475	4.43583392	-2.215671017	0.027593	0.03876	-4.91175447
ZMYM1	-0.160682697	2.65567891	-2.215331351	0.027617	0.03879	-4.91249263
RNF227	0.133357546	2.44197006	2.21384279	0.027721	0.03893	-4.91572629
C19orf38	0.170598593	1.33429865	2.213731695	0.027728	0.03894	-4.91596755
TSHZ2	0.168584917	1.10177832	2.213380856	0.027753	0.03897	-4.91672935
PPM1K	-0.161931389	3.03917018	-2.213309257	0.027758	0.03898	-4.9168848

ISOC1	-0.17314806	5.45181084	-2.213114453	0.027771	0.03899	-4.91730773
RNF112	-0.069007341	0.40927796	-2.213047975	0.027776	0.039	-4.91745205
OR51E2	0.078621395	0.23796981	2.212546702	0.027811	0.03904	-4.91854014
TACR2	-0.095467163	0.50085049	-2.212384054	0.027822	0.03905	-4.91889314
GSTT4	-0.022203957	0.04548381	-2.212370013	0.027823	0.03905	-4.91892361
BMF	-0.277243644	5.05209141	-2.211529088	0.027882	0.03913	-4.92074829
YPEL4	0.253329734	1.59724004	2.211518151	0.027883	0.03913	-4.92077202
C11orf91	0.057512264	0.15844491	2.211017324	0.027918	0.03918	-4.92185842
RPL17-C	-0.021598683	0.06077538	-2.21099164	0.02792	0.03918	-4.92191413
PPP1CB	-0.155434905	7.15003919	-2.210342071	0.027965	0.03924	-4.92332281
ITIH5	-0.308993856	3.46902874	-2.210179065	0.027977	0.03925	-4.92367625
SLITRK3	-0.059326016	0.13028447	-2.209086485	0.028054	0.03935	-4.92604459
PTGER4	0.294535833	2.38314979	2.209043292	0.028057	0.03935	-4.9261382
PTPN11	-0.20702876	5.65603641	-2.208321794	0.028108	0.03942	-4.92770151
PDE1A	-0.281716541	5.06779488	-2.207073475	0.028196	0.03954	-4.93040517
GTF2I	-0.321285803	4.03056214	-2.206679071	0.028224	0.03958	-4.93125908
GAS2L2	-0.184307174	0.65818478	-2.206374611	0.028245	0.03961	-4.93191815
MKRN2C	0.27282272	1.72916741	2.205966432	0.028274	0.03964	-4.93280162
C19orf57	0.110743297	1.19513586	2.20498853	0.028344	0.03974	-4.93491754
TENM3	0.326680561	2.16320913	2.204487816	0.028379	0.03978	-4.93600061
ENHO	-0.157688085	0.97600059	-2.204082162	0.028408	0.03982	-4.93687788
RGL3	-0.230448591	6.1736524	-2.203914808	0.02842	0.03983	-4.93723976
BCL2L12	-0.134637956	3.62456147	-2.203889103	0.028422	0.03983	-4.93729534
MYO19	-0.137688099	2.63839196	-2.203252693	0.028467	0.03989	-4.93867122
SPC24	0.164004571	1.69128969	2.202392475	0.028529	0.03998	-4.94053035
ARHGAF	0.284685091	3.29664629	2.202156395	0.028545	0.04	-4.94104045
NIPAL3	-0.196047936	5.55768961	-2.20048256	0.028665	0.04016	-4.94465562
EYA4	-0.241969949	0.74448769	-2.199362729	0.028746	0.04027	-4.94707277
SLC24A2	0.055511243	0.09676396	2.199272952	0.028752	0.04028	-4.9472665
KLHL28	-0.170211757	2.60914998	-2.199036147	0.028769	0.0403	-4.94777747
DACT2	-0.190627153	0.37285433	-2.197105	0.028909	0.04049	-4.95194247
CPT1B	0.175735463	1.46969593	2.196543408	0.028949	0.04054	-4.95315301
ADNP2	-0.174431242	3.62451925	-2.194106192	0.029126	0.04079	-4.95840314
RHBDF1	0.185783805	4.83981368	2.194081457	0.029128	0.04079	-4.9584564
STAT5A	0.160748266	4.49503566	2.193925119	0.029139	0.0408	-4.95879298
REXO2	-0.150533839	4.5139399	-2.193685402	0.029157	0.04082	-4.95930902
PLEKHA!	0.196241296	3.39481478	2.193133697	0.029197	0.04087	-4.96049648
IFI30	0.110586886	1.03086553	2.193084779	0.0292	0.04087	-4.96060175
GRIA4	-0.026171888	0.04652358	-2.192336607	0.029255	0.04095	-4.96221158
GCSAM	-0.195594753	1.48944687	-2.191903221	0.029287	0.04099	-4.96314384

WNT8B	0.021875929	0.07071011	2.191431631	0.029321	0.04103	-4.96415809
CANT1	-0.182241013	5.2611051	-2.19089405	0.02936	0.04108	-4.96531401
THAP8	-0.117219242	3.84454194	-2.190238818	0.029408	0.04115	-4.96672253
ACTR2	-0.179051968	6.9274249	-2.189416427	0.029469	0.04123	-4.96848981
IL1RL2	0.234554595	3.10635966	2.187485975	0.029611	0.04142	-4.97263575
NSMAF	-0.132711187	4.24421725	-2.187391767	0.029618	0.04143	-4.97283799
PHIP	0.163824805	3.70086407	2.186564556	0.029679	0.04151	-4.9746134
ANKRA2	-0.161992598	4.61878053	-2.18642582	0.029689	0.04152	-4.9749111
CDT1	0.233132235	3.15625699	2.185831324	0.029733	0.04158	-4.97618657
TANC1	0.242114488	3.94383829	2.185543376	0.029755	0.04161	-4.97680423
PRLHR	-0.187607688	0.19232944	-2.185365984	0.029768	0.04162	-4.9771847
ANKRD3	0.096488538	0.19973124	2.185197802	0.02978	0.04164	-4.9775454
TMEM54	-0.180772941	6.81804147	-2.185178424	0.029782	0.04164	-4.97758695
TYK2	-0.126011627	4.89333517	-2.184747704	0.029814	0.04168	-4.97851057
NTN5	-0.168880482	0.89095753	-2.184680722	0.029819	0.04168	-4.97865419
BTBD19	-0.252861381	3.25378975	-2.184668315	0.02982	0.04168	-4.97868079
EVPL	0.253574419	5.44560054	2.184501622	0.029832	0.04169	-4.97903818
CD226	0.103073296	0.42055007	2.184060395	0.029865	0.04173	-4.97998404
OTX1	-0.101353191	0.18334073	-2.183435484	0.029911	0.0418	-4.98132336
RINL	-0.20750967	4.05817043	-2.182916052	0.02995	0.04185	-4.98243633
DGCR6	-0.34007133	3.07601641	-2.182890443	0.029952	0.04185	-4.98249119
TRNAU1.	-0.117334352	3.40122738	-2.182419343	0.029987	0.04189	-4.98350037
SAMD4B	-0.128981895	5.37404493	-2.181741934	0.030038	0.04196	-4.98495113
SHROOI	-0.158580968	3.32819826	-2.180077089	0.030162	0.04213	-4.98851478
HACD2	-0.196177425	4.40291092	-2.179759012	0.030186	0.04216	-4.98919533
EIF1AD	-0.114498643	4.60972021	-2.178481532	0.030282	0.04229	-4.99192765
SCAF4	-0.125546232	4.07070109	-2.17828563	0.030297	0.04231	-4.99234651
TMEM16	-0.256907245	1.98873249	-2.176440845	0.030436	0.0425	-4.99628913
INSM2	0.018944123	0.0308155	2.176345636	0.030443	0.0425	-4.99649252
MAPKAP	0.161376919	6.9733622	2.176034232	0.030467	0.04253	-4.9971577
PLXNA1	0.187363403	3.6669255	2.175995561	0.03047	0.04253	-4.9972403
EDIL3	-0.243933287	3.18497548	-2.174920524	0.030551	0.04264	-4.9995359
TMEM13	0.39116806	1.69301197	2.174631089	0.030573	0.04267	-5.00015376
PLA2G5	0.305186085	1.41185392	2.174244088	0.030602	0.04271	-5.00097978
TRIO	0.17856289	2.75177298	2.174096307	0.030613	0.04272	-5.00129517
VSNL1	0.17315585	0.49826368	2.174086731	0.030614	0.04272	-5.0013156
CRYGS	-0.188644579	1.85063192	-2.173722351	0.030642	0.04275	-5.00209315
CLEC18I	-0.048159623	0.16269651	-2.172878426	0.030706	0.04284	-5.00389352
SLC35C1	-0.141699912	4.24423539	-2.172270996	0.030752	0.0429	-5.00518895
CHRNA5	-0.050705924	0.29371038	-2.171901626	0.030781	0.04294	-5.00597652

LRIF1	-0.135924788	3.79377768	-2.17167077	0.030798	0.04296	-5.00646868
ANK1	0.062331236	0.26401101	2.170289834	0.030904	0.0431	-5.00941164
LPAR4	0.042955709	0.20802168	2.169321917	0.030978	0.0432	-5.01147332
RSF1	-0.173715731	3.65222372	-2.169266923	0.030982	0.0432	-5.01159043
INMT	0.506267338	4.03568379	2.169146734	0.030991	0.04321	-5.01184637
DOT1L	-0.141334477	2.69803933	-2.168428952	0.031047	0.04329	-5.01337457
PCDH18	0.2353552	2.38496212	2.167882954	0.031089	0.04334	-5.01453671
SPATA2:	-0.062401387	0.06463128	-2.167608267	0.03111	0.04337	-5.01512126
MIIP	-0.168287086	5.26636206	-2.166200256	0.031218	0.04352	-5.01811649
CNTN4	0.264219207	2.78832965	2.165775689	0.031251	0.04356	-5.01901929
KCTD19	-0.031125359	0.0748179	-2.16530206	0.031288	0.0436	-5.02002622
YWHAZ	-0.141964177	7.61628471	-2.164865374	0.031321	0.04365	-5.02095442
TBL1X	-0.166601036	3.94105193	-2.16386845	0.031399	0.04375	-5.02307276
CPNE1	-0.149576955	6.6324267	-2.16380673	0.031404	0.04376	-5.02320388
ALK	0.271293015	1.13705958	2.16270971	0.031489	0.04387	-5.02553376
SLC18A1	0.024253151	0.05775178	2.161918054	0.031551	0.04395	-5.02721439
PANX2	-0.216045068	3.21287321	-2.161881441	0.031553	0.04395	-5.02729211
SLC26A1	-0.036012612	0.13567105	-2.160786354	0.031639	0.04407	-5.02961589
LMTK3	0.215791653	3.01295916	2.160669215	0.031648	0.04408	-5.02986439
RIMBP3E	-0.012927286	0.03137042	-2.160160212	0.031688	0.04413	-5.03094405
SYNE3	-0.146598946	1.63391741	-2.158198925	0.031842	0.04434	-5.03510192
TRPC5	0.462565706	1.80630001	2.158041854	0.031854	0.04435	-5.03543474
THEM5	0.354597719	2.25850891	2.156445008	0.03198	0.04453	-5.03881708
SLC25A4	0.169158048	3.0451616	2.155759125	0.032034	0.0446	-5.04026913
GNAQ	-0.164007862	5.64210306	-2.154092232	0.032166	0.04478	-5.04379617
TRIM36	-0.354189575	1.3722522	-2.153432162	0.032218	0.04485	-5.04519211
PFAS	-0.145136551	3.35841817	-2.153404399	0.03222	0.04485	-5.04525082
TEN1-CD	-0.125469726	1.02050967	-2.152081538	0.032325	0.04499	-5.04804718
GINS2	-0.193686304	3.00374911	-2.151830181	0.032345	0.04501	-5.04857833
TMEM97	-0.176679772	2.85687887	-2.150027523	0.032489	0.04521	-5.05238582
ZNF845	-0.143545235	2.34562114	-2.149790346	0.032508	0.04523	-5.05288655
EFNB1	-0.18224912	5.20331397	-2.149434669	0.032537	0.04527	-5.05363735
RIT1	-0.135953069	4.36251342	-2.149098313	0.032564	0.0453	-5.05434725
MMP24	0.140946767	1.06787383	2.148448849	0.032616	0.04537	-5.0557177
BTBD9	-0.124792735	3.37803804	-2.148329203	0.032625	0.04538	-5.05597012
CNOT1	-0.225752741	4.97151738	-2.14789333	0.03266	0.04542	-5.05688959
SAPCD1	0.08141358	0.42329479	2.147764372	0.032671	0.04544	-5.05716159
ZNF470	-0.133764378	2.28485879	-2.146428192	0.032778	0.04558	-5.05997896
XAB2	-0.134648965	5.81356315	-2.146325398	0.032786	0.04559	-5.06019563
EVL	-0.152695314	3.81170683	-2.146279551	0.03279	0.04559	-5.06029227

ZNF93	-0.140188568	2.33404499	-2.145702019	0.032837	0.04565	-5.0615094
ZNF649	-0.173950131	3.38012613	-2.145458188	0.032856	0.04567	-5.06202318
DYNLT3	-0.148262843	4.90021717	-2.145298911	0.032869	0.04569	-5.06235876
SPOCK3	0.155193361	0.26764417	2.144389582	0.032943	0.04579	-5.06427416
ATXN2	-0.117011974	3.28442392	-2.143120271	0.033045	0.04593	-5.06694652
MAN2B1	0.178766515	4.87737096	2.142694388	0.03308	0.04597	-5.06784281
LMTK2	-0.16690172	3.81478725	-2.142686578	0.033081	0.04597	-5.06785925
RRM2B	-0.186095002	5.85783373	-2.14257031	0.03309	0.04598	-5.06810391
IGSF9B	-0.150514916	1.19722368	-2.142352373	0.033108	0.046	-5.06856248
TMEM8A	-0.141808505	6.08299304	-2.142272932	0.033114	0.046	-5.06872962
FTL	-0.165972894	12.3854569	-2.14190618	0.033144	0.04604	-5.06950119
TNRC6C	-0.131675782	2.77620767	-2.141433235	0.033183	0.04609	-5.07049597
GNAO1	0.254280031	2.10616671	2.141343178	0.03319	0.0461	-5.07068537
F7	0.102075158	0.35230387	2.140394574	0.033267	0.0462	-5.07267993
TMEM17	-0.108313379	0.28111159	-2.139232862	0.033362	0.04633	-5.07512142
SGPP1	-0.173750689	4.71401467	-2.139053873	0.033377	0.04634	-5.07549747
PRDM2	-0.175492086	4.12637066	-2.139052108	0.033377	0.04634	-5.07550118
CLCN7	-0.151327818	5.08188015	-2.138088083	0.033456	0.04645	-5.07752606
C4BPB	-0.026406338	0.07744714	-2.137991371	0.033464	0.04645	-5.07772915
TPTE2	0.059881883	0.1719825	2.137842453	0.033476	0.04646	-5.07804185
EQTN	-0.031922794	0.11023849	-2.137833471	0.033477	0.04646	-5.07806071
PRSS1	0.410236833	1.34207181	2.136885353	0.033555	0.04657	-5.0800511
CDC14A	-0.209315297	3.17098857	-2.135610833	0.03366	0.04671	-5.08272536
MYEF2	0.184282331	2.3890791	2.135473535	0.033671	0.04672	-5.08301335
USP3	-0.130108229	3.52573416	-2.134111487	0.033783	0.04687	-5.08586938
ZSWIM8	-0.162455768	4.80009098	-2.134071932	0.033787	0.04688	-5.0859523
MSMB	0.298542938	0.63987772	2.13371599	0.033816	0.04691	-5.08669836
SMIM11A	-0.012676182	0.02983415	-2.13352195	0.033832	0.04693	-5.08710502
TMEM15	0.200832984	0.99105234	2.132985755	0.033876	0.04699	-5.08822857
GRIK2	-0.270815589	1.0947583	-2.132818792	0.03389	0.047	-5.08857837
MOV10	0.133107566	4.42206134	2.132457766	0.03392	0.04704	-5.08933465
FAM180A	0.168377827	0.6173117	2.131330935	0.034014	0.04716	-5.09169437
DVL3	-0.125248874	5.58111487	-2.131327658	0.034014	0.04716	-5.09170123
BHMT	-0.091137627	0.33069402	-2.130614077	0.034074	0.04724	-5.09319494
GPC5	0.196701132	0.70731062	2.130173021	0.03411	0.04729	-5.09411793
COL15A1	-0.278472457	4.99129016	-2.1298606	0.034136	0.04732	-5.09477162
RRAGC	-0.118981619	3.87263631	-2.129723017	0.034148	0.04733	-5.09505947
NUP205	-0.165638991	3.9555514	-2.128729424	0.034231	0.04745	-5.09713765
IHH	-0.156802774	0.11629086	-2.128080392	0.034285	0.04752	-5.09849466
ALDH1A2	-0.113903616	0.42981038	-2.127789425	0.034309	0.04755	-5.09910289

NUP155	-0.142655897	3.01162521	-2.127761465	0.034312	0.04755	-5.09916133
CTTNBP	0.302573053	2.31706637	2.126484682	0.034419	0.04769	-5.10182929
C19orf18	-0.17403841	1.8709001	-2.126301343	0.034434	0.04771	-5.10221227
STAG2	-0.172220234	5.5068304	-2.126071408	0.034454	0.04773	-5.10269254
SETD1B	-0.152580788	4.05815336	-2.124916852	0.034551	0.04786	-5.10510331
KLKB1	-0.155177854	1.06205999	-2.123666155	0.034656	0.048	-5.10771341
RHBDL2	-0.121282364	1.44908512	-2.123272848	0.03469	0.04805	-5.1085339
MICA	-0.153217696	4.81475764	-2.122969257	0.034715	0.04808	-5.10916713
C3orf67	-0.072212401	0.34353964	-2.122194581	0.034781	0.04816	-5.11078255
KCTD20	-0.151469015	4.49719233	-2.122105108	0.034789	0.04817	-5.11096909
TMEM10	-0.308293971	2.73557994	-2.121665384	0.034826	0.04822	-5.11188576
KLHL5	-0.177128455	3.66619638	-2.12094478	0.034887	0.0483	-5.11338755
PCNX2	0.243866819	2.97224923	2.120032746	0.034965	0.0484	-5.1152876
C4B	0.348495411	4.47941324	2.120005761	0.034967	0.0484	-5.1153438
CNIH4	-0.114831833	4.27831038	-2.119820256	0.034983	0.04842	-5.11573017
LCA5	0.245767816	3.63603262	2.119682537	0.034995	0.04843	-5.11601698
PPAT	-0.135995153	2.6257546	-2.118871581	0.035064	0.04852	-5.11770552
ATIC	0.185076916	5.77653026	2.118479516	0.035097	0.04857	-5.11852164
RAB37	-0.145665255	1.00225086	-2.118209835	0.03512	0.04859	-5.11908292
FAM214E	-0.117376605	3.72487857	-2.117988325	0.035139	0.04862	-5.11954389
PHLDB1	-0.182543473	3.3732118	-2.117750484	0.035159	0.04864	-5.12003879
TNFRSF	0.27854212	3.72545662	2.117527559	0.035179	0.04866	-5.12050261
CDC42EI	-0.168412583	5.7111256	-2.116258565	0.035287	0.04881	-5.12314199
ZNF232	-0.153681437	3.24992569	-2.116065944	0.035304	0.04883	-5.12354248
DTNA	-0.238745496	3.25370153	-2.114603049	0.03543	0.049	-5.12658299
CASTOR	0.22733104	2.81118077	2.114296329	0.035456	0.04903	-5.12722022
LGR6	-0.328100106	2.66569358	-2.114163274	0.035468	0.04904	-5.12749663
JAG1	-0.208815655	4.64822474	-2.11411776	0.035472	0.04904	-5.12759117
XKR6	-0.135667986	1.54062855	-2.113896525	0.035491	0.04907	-5.12805071
C6orf15	0.074601019	0.19048488	2.113508534	0.035524	0.04911	-5.12885652
TM4SF19	0.133029077	0.49657257	2.113446578	0.035529	0.04911	-5.12898518
TTLL10	0.078723234	0.34357897	2.113349709	0.035538	0.04912	-5.12918634
EIF4B	-0.174421248	7.67395785	-2.113218607	0.035549	0.04913	-5.12945857
RCL1	-0.116440691	2.89482938	-2.113090712	0.03556	0.04914	-5.12972412
MB21D2	-0.143559967	2.62237763	-2.113025871	0.035566	0.04915	-5.12985875
ATG9B	0.11962528	0.62788184	2.112825703	0.035583	0.04917	-5.13027432
ST5	-0.179071122	4.26210101	-2.11256983	0.035605	0.04919	-5.13080549
SREBF1	0.203840692	5.57953939	2.110656843	0.035771	0.04942	-5.13477473
ROCK2	-0.170637495	3.59055625	-2.110579109	0.035778	0.04942	-5.13493594
MEP1A	0.032557707	0.07859339	2.109868648	0.035839	0.04951	-5.13640913

AWAT1	0.044855791	0.10238757	2.109423534	0.035878	0.04955	-5.13733186
ABCA2	-0.219123422	5.45013118	-2.109253977	0.035893	0.04957	-5.13768331
WNT16	0.093148311	0.18017612	2.108624335	0.035948	0.04964	-5.13898814
TRHDE	0.021181629	0.03643297	2.108091049	0.035994	0.0497	-5.14009301
KLHL17	0.15339187	2.44944122	2.107825798	0.036018	0.04973	-5.14064245
RNF24	0.203623128	4.52634573	2.107142272	0.036077	0.04981	-5.14205801
RAPH1	0.204170791	1.41757245	2.1066148	0.036123	0.04987	-5.14315009
RUFY3	-0.163107366	4.42857058	-2.106490994	0.036134	0.04988	-5.14340638
HMCN1	0.159652016	1.14218194	2.105993115	0.036178	0.04994	-5.14443688
CAVIN1	0.198713197	8.11809823	2.105812394	0.036194	0.04995	-5.14481088
IGSF21	0.180525669	1.25414144	2.103865804	0.036365	0.05019	-5.14883731
ZFP28	-0.112825063	2.3288868	-2.103441797	0.036402	0.05023	-5.14971388
XRRA1	-0.170170836	2.32575801	-2.103105507	0.036432	0.05027	-5.15040898
LMO7	0.221633066	4.9584017	2.101723475	0.036554	0.05043	-5.15326447
PLCXD1	-0.149622254	2.22941161	-2.101627868	0.036562	0.05044	-5.15346195
RBM12B	-0.155174077	2.72268352	-2.101117309	0.036608	0.0505	-5.15451634
ZNF385A	-0.138438305	6.26754504	-2.100114116	0.036697	0.05062	-5.15658738
SFSWAF	-0.117112539	3.65397799	-2.100066958	0.036701	0.05062	-5.15668471
SOCS5	-0.167521864	3.92073427	-2.099727959	0.036731	0.05066	-5.15738432
TDRD5	-0.119678304	0.6785654	-2.099667828	0.036736	0.05066	-5.15750841
ANKRD5	-0.160626362	4.02462085	-2.099360195	0.036764	0.05069	-5.15814318
PKMYT1	0.131910723	1.14526636	2.099202707	0.036778	0.05071	-5.15846811
XRCC3	-0.130496405	2.13745357	-2.09841801	0.036847	0.0508	-5.16008673
PLA2G2F	0.012522473	0.01762744	2.098332292	0.036855	0.05081	-5.16026351
TRIM8	-0.124643521	7.56273757	-2.097472216	0.036932	0.05091	-5.16203687
COL6A2	0.287117895	6.58797928	2.097279041	0.036949	0.05093	-5.16243508
MRAP	-0.103908455	0.1577036	-2.095552212	0.037103	0.05114	-5.16599314
SEMA3F	-0.205972469	5.7927657	-2.095486258	0.037109	0.05114	-5.16612898
WFDC10	0.054945832	0.14424504	2.095231153	0.037132	0.05117	-5.16665436
SMIM13	-0.123056733	2.7259416	-2.095081879	0.037146	0.05118	-5.16696175
CORO7	-0.128650164	2.12211515	-2.094489554	0.037199	0.05125	-5.1681813
NAA16	-0.135841721	2.72256365	-2.094252791	0.03722	0.05128	-5.16866868
SPACA4	0.029937604	0.08216692	2.093950776	0.037247	0.05131	-5.16929031
PGAP1	-0.188073824	2.34236955	-2.093926702	0.037249	0.05131	-5.16933985
PRICKLE	-0.115993552	3.12786395	-2.093742642	0.037266	0.05133	-5.16971865
CCDC14	0.041214691	0.08850303	2.093309827	0.037305	0.05138	-5.17060926
FBXO45	-0.113025385	2.82371951	-2.091639943	0.037455	0.05158	-5.17404374
TEAD3	0.194523589	4.88205845	2.091297974	0.037486	0.05162	-5.17474675
PLEKHS	0.125382602	0.23061234	2.091134168	0.037501	0.05164	-5.17508346
YAP1	-0.219090761	6.30584069	-2.090644272	0.037545	0.05169	-5.1760903

MAB21L3	-0.029138382	0.06882055	-2.089919657	0.037611	0.05178	-5.17757912
MCMBP	-0.139318191	4.84588052	-2.089603192	0.03764	0.05181	-5.17822918
FGD4	-0.154271106	2.68803053	-2.08947825	0.037651	0.05183	-5.1784858
MROH1	-0.140909432	5.12231101	-2.089380792	0.03766	0.05183	-5.17868596
NHLRC4	-0.188356351	1.22415502	-2.088170783	0.03777	0.05198	-5.18117036
CCDC85	-0.249451745	1.75331362	-2.087841385	0.0378	0.05202	-5.18184644
ANGEL1	-0.149982655	3.72544297	-2.086612471	0.037912	0.05217	-5.18436785
TPH1	-0.132377993	0.64376573	-2.084568967	0.038099	0.05242	-5.1885574
JMJD7-P	-0.203536589	1.55113259	-2.084055866	0.038146	0.05248	-5.18960872
BAHD1	-0.171669263	5.48126869	-2.083252453	0.03822	0.05258	-5.19125438
MTFR2	0.083832298	0.71517688	2.0832301	0.038222	0.05258	-5.19130016
HOXA1	0.048321765	0.22766093	2.083011667	0.038242	0.0526	-5.19174748
ZNF84	-0.135200084	3.61958197	-2.081766832	0.038356	0.05275	-5.19429582
MCM5	0.158628519	4.1193608	2.081689955	0.038364	0.05276	-5.19445315
GMIP	-0.128965109	4.05851366	-2.081658403	0.038366	0.05276	-5.19451772
LARP1B	-0.142647092	2.97224315	-2.081302522	0.038399	0.0528	-5.19524595
ACY3	0.158949879	0.72534245	2.081117514	0.038416	0.05282	-5.19562448
CCL2	0.353161692	3.79294861	2.080711	0.038454	0.05287	-5.1964561
GJB5	-0.199900955	1.19531449	-2.080546699	0.038469	0.05288	-5.19679218
PTK2B	0.227153048	4.99727338	2.080172325	0.038504	0.05293	-5.19755785
SIK1	0.257464012	0.81594641	2.080131914	0.038507	0.05293	-5.19764049
CCDC12	-0.110285077	2.7743281	-2.079566668	0.03856	0.053	-5.19879626
NKX3-1	-0.150548275	1.14330334	-2.079210579	0.038593	0.05304	-5.19952421
C4BPA	-0.097391067	0.21954523	-2.078780339	0.038633	0.05309	-5.20040358
ZBTB41	-0.167018513	3.3277004	-2.078492291	0.038659	0.05312	-5.20099223
PANO1	-0.102986911	0.9244518	-2.07803945	0.038701	0.05317	-5.20191748
E2F7	0.064776191	0.40768045	2.077082314	0.03879	0.05329	-5.20387248
C12orf49	0.253456263	6.46415605	2.076836988	0.038813	0.05332	-5.20437343
NFATC4	0.227208027	3.99673554	2.075754217	0.038914	0.05345	-5.20658374
KNL1	0.084002338	0.65762095	2.075651226	0.038924	0.05346	-5.20679392
RPS9	-0.163437029	9.12396678	-2.075158942	0.03897	0.05352	-5.20779843
ARNT	-0.14482442	4.62776772	-2.074111236	0.039068	0.05365	-5.2099355
IL12B	0.072002503	0.15964894	2.073626	0.039113	0.05371	-5.21092492
PINLYP	-0.253448422	2.45317595	-2.072697752	0.0392	0.05382	-5.21281703
FGD2	-0.224407018	1.46652497	-2.072597573	0.03921	0.05383	-5.21302118
SLC26A2	0.189161866	3.14461091	2.071689433	0.039295	0.05394	-5.21487143
KLRG2	-0.31441009	3.15442176	-2.07148516	0.039314	0.05397	-5.2152875
MLLT10	-0.182782913	4.10313796	-2.071013386	0.039359	0.05402	-5.21624829
SIRT6	-0.122367595	4.53392368	-2.070747886	0.039384	0.05405	-5.2167889
SMC3	-0.19059656	5.27098622	-2.069316948	0.039519	0.05423	-5.21970143

TSSK3	-0.104346142	0.64852882	-2.068803015	0.039568	0.0543	-5.22074701
CHM	-0.190370183	3.23880769	-2.067930685	0.03965	0.05441	-5.22252116
ZC3H6	-0.131678482	2.71018113	-2.067598438	0.039682	0.05444	-5.22319669
EPHA3	-0.233150859	2.13776699	-2.06678149	0.039759	0.05455	-5.22485729
SLC2A5	0.16370768	0.70281879	2.066094545	0.039825	0.05463	-5.22625315
IGSF23	-0.019925226	0.0489924	-2.065774846	0.039855	0.05467	-5.22690262
UGGT2	-0.135282422	3.37181559	-2.065398204	0.039891	0.05471	-5.22766764
ZSCAN2(	-0.104497091	1.27857955	-2.064107926	0.040014	0.05488	-5.23028738
APOF	-0.024043319	0.07266542	-2.064059917	0.040019	0.05488	-5.23038483
DDX47	0.12921136	1.32849286	2.062988398	0.040121	0.05502	-5.23255916
ABHD3	0.187543972	4.51067005	2.062871934	0.040133	0.05503	-5.23279542
ARL13B	0.220731156	4.09264771	2.062836184	0.040136	0.05503	-5.23286794
FAM222E	-0.129079265	4.34026203	-2.062673282	0.040152	0.05504	-5.23319839
INTS6L	0.17388349	2.1639384	2.061847765	0.040231	0.05515	-5.23487253
PABPC1I	0.258936891	3.72123811	2.061794512	0.040236	0.05515	-5.23498051
EDC4	-0.19114401	4.04131442	-2.060631456	0.040348	0.0553	-5.23733803
AFDN	0.200974857	4.92468578	2.060415821	0.040368	0.05532	-5.23777498
SRRM1	-0.157108342	4.89305726	-2.060193439	0.04039	0.05535	-5.23822556
DOP1B	0.171337194	3.62219128	2.059815182	0.040426	0.05539	-5.23899185
ARAP2	-0.18740545	3.63185764	-2.058313416	0.040571	0.05559	-5.24203287
GRPR	0.066296804	0.25650589	2.05830453	0.040572	0.05559	-5.24205086
ALS2CL	0.233475726	4.6362951	2.057074293	0.040691	0.05574	-5.24454043
ZNF780E	-0.151668081	2.8562867	-2.056786961	0.040719	0.05578	-5.24512168
PTCH1	-0.167149246	2.04676562	-2.056193347	0.040777	0.05585	-5.24632228
B3GNT8	0.281475352	3.23261166	2.055998465	0.040796	0.05587	-5.24671635
HECTD3	-0.115751295	5.25653746	-2.055202811	0.040873	0.05597	-5.2483249
LINGO1	-0.217387942	2.05654241	-2.055148716	0.040878	0.05598	-5.24843424
NOVA1	-0.289310785	1.47115577	-2.054073834	0.040983	0.05612	-5.25060628
NOL6	-0.128361165	4.50192154	-2.052526769	0.041134	0.05632	-5.25373054
TGM3	0.064981138	0.22242516	2.052509102	0.041136	0.05632	-5.25376621
GABRG3	-0.244611541	0.95349657	-2.052105551	0.041176	0.05637	-5.25458079
SLC22A3	-0.222110056	1.2623213	-2.051968748	0.041189	0.05638	-5.25485689
FAM53C	-0.142966918	3.80901361	-2.051475047	0.041237	0.05644	-5.25585317
JUND	-0.23652713	8.47363704	-2.050491778	0.041334	0.05657	-5.25783669
TCTEX1I	0.053441291	0.29111571	2.050488581	0.041334	0.05657	-5.25784314
HSPB6	-0.296035394	2.7212532	-2.050081924	0.041374	0.05662	-5.25866321
CBS	-0.126597502	0.77743395	-2.049319666	0.041449	0.05671	-5.26019996
SLC12A3	0.03487678	0.081245	2.049099432	0.041471	0.05674	-5.26064386
MIXL1	-0.194727538	0.64720697	-2.047581865	0.041621	0.05694	-5.26370139
GUCD1	-0.117157668	5.44183967	-2.047415608	0.041637	0.05696	-5.26403622

VTN	-0.148057473	0.62628928	-2.04711813	0.041667	0.05699	-5.26463527
ZNF628	-0.122321099	2.79693747	-2.046818689	0.041696	0.05703	-5.26523818
RAB8B	-0.167165225	4.11289732	-2.046669305	0.041711	0.05704	-5.26553892
TGFB3	0.226827915	3.11462626	2.046324599	0.041745	0.05709	-5.26623282
FOXP1	-0.147174751	3.94823281	-2.046277644	0.04175	0.05709	-5.26632733
STX16-N	-0.118630029	0.70761587	-2.046216496	0.041756	0.05709	-5.2664504
FAM180E	-0.220620685	0.633597	-2.045888532	0.041788	0.05713	-5.26711046
TRIM54	-0.312495954	2.01282306	-2.045488994	0.041828	0.05718	-5.26791442
TAF4	-0.106293858	2.39867463	-2.04429314	0.041947	0.05734	-5.27031985
SERAC1	-0.13452615	2.53341296	-2.043889277	0.041987	0.05739	-5.2711319
FSD1	0.056010482	0.16601424	2.043022572	0.042073	0.0575	-5.27287407
ASB12	-0.075518627	0.70928207	-2.042785468	0.042097	0.05753	-5.27335055
MRE11	-0.123910245	3.19828783	-2.042321609	0.042143	0.05759	-5.27428256
METTL7E	0.432819239	6.33040776	2.041887968	0.042187	0.05764	-5.27515367
ACO1	-0.20595761	5.36706012	-2.041685406	0.042207	0.05767	-5.27556052
KLK8	0.142281001	0.34251589	2.041044493	0.042271	0.05775	-5.27684755
LRATD2	0.238605707	4.91718415	2.03964599	0.042411	0.05794	-5.27965454
ZNF800	-0.137272357	3.3622926	-2.038996142	0.042477	0.05802	-5.28095825
EFCAB1	-0.040363539	0.06562836	-2.038699266	0.042507	0.05806	-5.2815537
DDR1	-0.186014159	7.12144543	-2.037998162	0.042577	0.05815	-5.28295958
BICRAL	-0.167287363	4.09031221	-2.037808719	0.042596	0.05817	-5.28333939
CCDC11	-0.040358307	0.26062782	-2.035334431	0.042846	0.05851	-5.28829675
CAPN13	-0.013585699	0.02100125	-2.035269118	0.042853	0.05851	-5.28842753
NBN	-0.160615743	4.61021309	-2.035085098	0.042872	0.05853	-5.28879598
GRM1	-0.06358775	0.16250333	-2.034872452	0.042893	0.05856	-5.2892217
WWC3	-0.160676124	5.04810026	-2.034594057	0.042921	0.05859	-5.289779
TP53I3	-0.190116388	5.06855637	-2.034471868	0.042934	0.0586	-5.29002357
TUBB6	0.216733701	5.33105857	2.032792048	0.043105	0.05883	-5.29338448
WWC2	-0.213883831	3.78019492	-2.032726053	0.043111	0.05884	-5.29351646
RAI2	0.223501258	5.31954165	2.03236706	0.043148	0.05888	-5.29423434
DERL1	-0.154907534	5.25855318	-2.032337843	0.043151	0.05888	-5.29429277
SLC22A1	-0.133561811	1.46003228	-2.032313051	0.043153	0.05888	-5.29434234
BRI3	-0.121402954	5.70581639	-2.032072273	0.043178	0.05891	-5.29482374
REST	-0.169920051	4.19709232	-2.031765065	0.043209	0.05895	-5.29543789
AASS	-0.178886089	2.25025787	-2.030052695	0.043384	0.05918	-5.29885948
TLR5	0.154707133	2.38747751	2.029824493	0.043408	0.05921	-5.29931525
LYPD5	0.128585269	1.37509942	2.029285023	0.043463	0.05928	-5.3003925
JSRP1	0.279498785	1.21703729	2.029059607	0.043486	0.0593	-5.30084255
ILDR2	-0.068550324	0.21046686	-2.028944649	0.043498	0.05932	-5.30107204
CD300A	0.210192392	2.26479825	2.028059785	0.043589	0.05943	-5.30283811

VPS18	-0.124520149	5.34635948	-2.027317121	0.043665	0.05953	-5.30431979
RTL8C	-0.117798402	7.66441348	-2.0271843	0.043679	0.05955	-5.30458473
ZNF142	-0.130407014	3.32181558	-2.027148726	0.043683	0.05955	-5.30465568
PPT2-EG	-0.058995656	0.53468444	-2.026800756	0.043718	0.05959	-5.30534968
ZNF69	-0.123083327	2.23940784	-2.026736624	0.043725	0.0596	-5.30547757
PHEX	-0.539587771	2.62499186	-2.025797646	0.043822	0.05972	-5.30734965
TUBA1C	-0.170889298	4.80917037	-2.02564372	0.043838	0.05974	-5.30765646
ZC3H13	-0.173457517	4.57902402	-2.024785594	0.043927	0.05986	-5.30936647
C9orf16	0.177732629	6.72061278	2.02398844	0.044009	0.05996	-5.31095436
CLDN4	0.244973923	8.0610058	2.023895641	0.044019	0.05997	-5.31113917
PRKCI	-0.18934699	5.11868886	-2.023874865	0.044021	0.05997	-5.31118055
HCLS1	0.234423813	3.89302132	2.022574449	0.044156	0.06015	-5.31376946
ADAMTS	0.253687611	1.95079961	2.021453444	0.044272	0.0603	-5.31599991
ATP2B1	-0.159561181	3.50694597	-2.020743617	0.044346	0.0604	-5.31741162
WDFY3	-0.16839736	3.46196184	-2.019737023	0.044452	0.06054	-5.31941273
RAB15	-0.21018706	4.30255388	-2.019281106	0.044499	0.0606	-5.32031877
HERC6	0.196593729	3.44966863	2.019077887	0.044521	0.06062	-5.32072256
PTBP3	-0.155831682	4.63548916	-2.019055213	0.044523	0.06062	-5.32076761
PRR12	-0.156607135	4.5208134	-2.018806031	0.044549	0.06065	-5.32126267
PAN3	-0.160553118	4.31842864	-2.017268218	0.04471	0.06087	-5.32431661
ABCA6	0.091061145	0.48122267	2.016800444	0.044759	0.06093	-5.32524511
GTSE1	0.122541618	0.89162481	2.016247613	0.044818	0.061	-5.32634218
UTRN	0.200908021	5.29368732	2.015901885	0.044854	0.06105	-5.32702811
CDK16	-0.097369638	5.78904772	-2.015722289	0.044873	0.06107	-5.32738438
TRIB2	0.177156147	4.31662749	2.015705448	0.044875	0.06107	-5.32741779
IL4	0.075159864	0.34127708	2.015641772	0.044881	0.06107	-5.3275441
NPR2	-0.183521798	2.53167808	-2.015095268	0.044939	0.06114	-5.32862801
MINDY4E	-0.053533569	0.09308099	-2.015063792	0.044942	0.06114	-5.32869043
PSD2	0.146473412	0.57259214	2.014609649	0.04499	0.0612	-5.32959093
LHFPL4	0.105551742	0.23106163	2.013731616	0.045083	0.06132	-5.33133137
CLEC17/	0.108801337	0.31475823	2.013634381	0.045093	0.06133	-5.33152406
PIK3AP1	-0.201445917	3.09913051	-2.013248169	0.045134	0.06138	-5.33228935
TNS4	-0.477527848	4.03856754	-2.013135505	0.045146	0.0614	-5.33251257
EIF4A1	0.149281786	1.61475669	2.012696775	0.045193	0.06145	-5.3333817
TCF21	-0.068881744	0.15274018	-2.012496781	0.045214	0.06148	-5.33377783
CA10	0.072432171	0.0921594	2.012293018	0.045236	0.0615	-5.33418138
BIRC2	-0.154227711	5.08460776	-2.012004319	0.045266	0.06154	-5.33475309
RPL13	-0.155914158	8.86341478	-2.011625976	0.045306	0.06159	-5.33550219
NRK	0.257032814	0.89440303	2.01140282	0.04533	0.06162	-5.33594397
CMIP	-0.134628825	3.22325585	-2.011291176	0.045342	0.06163	-5.33616497

DSG2	0.229556663	6.13772501	2.010820314	0.045392	0.06169	-5.33709692
ODF3B	-0.207864583	4.30889243	-2.010287788	0.045449	0.06176	-5.33815066
AGAP2	-0.156140943	1.40076031	-2.010263811	0.045451	0.06176	-5.3381981
MYLK	-0.178267966	2.47003178	-2.009334989	0.04555	0.06189	-5.34003535
FNDC3B	-0.173628813	4.68572916	-2.008904002	0.045596	0.06195	-5.34088758
GUCY2D	0.111807602	1.00822332	2.008597352	0.045629	0.06199	-5.34149384
NEDD1	-0.144746168	3.56105298	-2.007368794	0.045761	0.06216	-5.34392185
CKM	0.175505562	0.25959082	2.007216499	0.045777	0.06218	-5.34422273
ARL6IP4	-0.158862589	1.85649824	-2.006068965	0.0459	0.06234	-5.34648914
ZNF274	-0.154187173	4.00707225	-2.005206698	0.045993	0.06246	-5.34819132
CCDC11	-0.139052086	1.07820321	-2.00372192	0.046153	0.06267	-5.35112072
TEX101	0.144968798	0.19331089	2.003529915	0.046173	0.0627	-5.35149938
SCG3	-0.071074469	0.30865571	-2.003254812	0.046203	0.06273	-5.35204186
RNF165	-0.145468752	1.51698805	-2.003236517	0.046205	0.06273	-5.35207794
RDM1	-0.093159066	0.53422221	-2.003018386	0.046229	0.06276	-5.35250802
ZNF670	-0.11748573	2.09382193	-2.002964304	0.046234	0.06276	-5.35261465
CTNNB1	-0.20602056	7.89726842	-2.002589452	0.046275	0.06281	-5.35335361
SMIM3	0.200244087	5.06376017	2.00199684	0.046339	0.06289	-5.35452158
PRAG1	-0.269029329	5.01704103	-2.001614937	0.046381	0.06294	-5.35527409
VENTX	0.122980277	0.90696382	2.001544226	0.046388	0.06295	-5.3554134
CYFIP2	-0.184590478	4.22731657	-2.000986968	0.046449	0.06303	-5.35651114
TAF1A	-0.145823858	2.22004948	-2.000474426	0.046504	0.0631	-5.35752054
TNF	0.18744758	0.81261731	2.000163531	0.046538	0.06314	-5.35813269
MSI2	-0.160588991	4.08037115	-1.999890378	0.046568	0.06317	-5.35867046
CERS6	-0.195934969	4.02031518	-1.999611628	0.046598	0.06321	-5.35921916
ELOVL3	0.04961958	0.25604339	1.99927514	0.046635	0.06325	-5.35988143
FBXO6	0.131383421	4.59071437	1.998685907	0.046699	0.06333	-5.36104087
TMEM13	-0.132448829	0.20607834	-1.998360075	0.046734	0.06338	-5.36168188
STAB1	-0.177157834	3.58968762	-1.997191272	0.046862	0.06355	-5.36398041
C19orf12	-0.094701734	3.65534627	-1.996264954	0.046963	0.06368	-5.36580116
SAPCD2	-0.139404979	1.63050506	-1.996095629	0.046982	0.0637	-5.36613389
HASPIN	0.081619688	0.4717225	1.995838253	0.04701	0.06373	-5.3666396
TMOD3	0.180551695	4.24291472	1.995792882	0.047015	0.06373	-5.36672874
EML4	-0.158939023	4.97748301	-1.994843888	0.047119	0.06387	-5.36859278
DONSON	0.128161127	3.05694728	1.994369521	0.047171	0.06393	-5.36952422
6-Mar	-0.142317728	5.33725648	-1.993672713	0.047248	0.06403	-5.37089205
ANKRD3	-0.1235615	0.68852167	-1.99300396	0.047322	0.06413	-5.37220437
NID1	0.236839089	4.76266272	1.99183488	0.047451	0.0643	-5.37449747
SRPK3	0.077870036	0.35054169	1.991236117	0.047517	0.06438	-5.37567142
CPA4	0.029112867	0.08364564	1.991112428	0.04753	0.0644	-5.37591388

LPCAT1	-0.14163749	4.41914101	-1.990653837	0.047581	0.06446	-5.37681272
MAP3K1	0.180022642	3.1779027	1.990437744	0.047605	0.06449	-5.37723619
RCOR1	-0.138155776	3.621273	-1.990312039	0.047619	0.0645	-5.37748251
MAML2	0.222270627	3.82315482	1.990272273	0.047624	0.0645	-5.37756043
MDM2	-0.167113416	4.42998286	-1.990239082	0.047627	0.0645	-5.37762546
JMY	-0.157281755	4.18656438	-1.990018253	0.047652	0.06453	-5.37805813
UBE2QL	-0.282377374	3.68226046	-1.98974452	0.047682	0.06456	-5.37859438
GAL3ST	0.044122072	0.13655841	1.988351897	0.047837	0.06477	-5.38132148
HEATR9	0.024825058	0.08967574	1.98766203	0.047914	0.06487	-5.38267172
HEXIM1	-0.152380129	5.64656296	-1.987511614	0.04793	0.06489	-5.38296606
PTGIR	-0.143537683	1.53665955	-1.986652324	0.048026	0.06501	-5.38464715
DCAF12I	-0.147399026	0.58285102	-1.98611226	0.048086	0.06509	-5.38570336
EXOC3L	0.097213258	0.32807198	1.985788697	0.048123	0.06513	-5.38633601
ZNF730	0.092007411	0.5507316	1.985156455	0.048193	0.06522	-5.38757194
UBE2C	0.248527823	2.40429267	1.984944015	0.048217	0.06525	-5.38798714
FAM71F	-0.020375696	0.0359885	-1.984271166	0.048292	0.06534	-5.38930189
ANGEL2	-0.120255118	3.52736892	-1.983079487	0.048426	0.06552	-5.39162938
TNXB	0.197003264	1.80030461	1.982826391	0.048455	0.06555	-5.39212353
PTGER3	-0.106347421	0.68639956	-1.982724856	0.048466	0.06556	-5.39232175
FGF22	-0.027790959	0.13724122	-1.982342244	0.048509	0.06562	-5.39306862
MEGF9	0.268621442	6.14644821	1.981540632	0.048599	0.06573	-5.39463293
UMODL1	-0.016538529	0.02687753	-1.981464765	0.048608	0.06574	-5.39478095
MERTK	-0.214444767	3.88661438	-1.981336949	0.048622	0.06575	-5.39503031
KLHL30	0.202528959	1.39423349	1.980905605	0.048671	0.06581	-5.39587173
CPED1	-0.163796706	1.27580042	-1.980103913	0.048762	0.06593	-5.3974351
GGA2	-0.135986259	6.16806749	-1.979911577	0.048783	0.06596	-5.39781008
CDK7	-0.118086182	5.0536626	-1.97790948	0.04901	0.06626	-5.4017113
TMPRSS	0.07034105	0.5583383	1.977872775	0.049014	0.06626	-5.40178278
FAR2	-0.149305808	1.9456691	-1.977431974	0.049065	0.06632	-5.40264118
TMPRSS	-0.046859291	0.29154136	-1.977169131	0.049094	0.06635	-5.40315295
PTGFR	0.055043969	0.13785888	1.977151632	0.049096	0.06635	-5.40318702
RASA4B	0.024791868	0.10785095	1.977035857	0.04911	0.06637	-5.40341241
ATP8B3	-0.136939458	0.75308027	-1.976879698	0.049127	0.06638	-5.4037164
SEPTIN1	-0.188880163	5.11121052	-1.976280224	0.049196	0.06647	-5.40488319
STAG3	0.110384621	0.83661101	1.97604652	0.049222	0.0665	-5.40533796
GANC	-0.145209083	2.87220124	-1.975533637	0.049281	0.06658	-5.40633582
PSMA8	-0.03507357	0.08129199	-1.975447112	0.049291	0.06658	-5.40650414
GNG12	-0.179747521	6.34193157	-1.975367755	0.0493	0.06659	-5.4066585
NCOA3	-0.149496361	4.25923251	-1.975201899	0.049319	0.06661	-5.40698111
LACTB2	-0.15251251	4.59965732	-1.975196693	0.049319	0.06661	-5.40699124

ARHGAF	0.86803182	4.22967695	1.975133383	0.049326	0.06661	-5.40711437
CAMSAF	-0.154125956	4.49201495	-1.974874684	0.049356	0.06664	-5.4076175
GNAZ	0.187653448	2.62170061	1.974754895	0.04937	0.06666	-5.40785045
EBF1	-0.176822848	2.25422723	-1.973243797	0.049542	0.06689	-5.41078784
RAB12	-0.134526073	5.29016675	-1.97320366	0.049547	0.06689	-5.41086583
MAPK6	-0.178440101	4.45819733	-1.973062568	0.049563	0.0669	-5.41113998
APC2	-0.064636865	0.5995851	-1.97296996	0.049574	0.06691	-5.41131991
NDST3	-0.098726386	0.28230063	-1.972446692	0.049634	0.06699	-5.41233643
CSNK1A	-0.202751238	4.70761314	-1.971927931	0.049693	0.06706	-5.41334394
USP36	-0.12408421	4.10774475	-1.971747558	0.049714	0.06709	-5.41369419
TMEM25	0.250299107	1.3475021	1.970764526	0.049827	0.06723	-5.41560251
PI4KA	-0.174424008	4.50704502	-1.970431024	0.049866	0.06728	-5.41624971
ZNF169	-0.099181514	1.09407024	-1.970366327	0.049873	0.06728	-5.41637525
CA13	-0.15517127	3.62057903	-1.969672311	0.049953	0.06739	-5.41772169
DMD	0.322383281	2.71707179	1.969629762	0.049958	0.06739	-5.41780423
SETD7	0.188150319	3.40532914	1.969591891	0.049962	0.06739	-5.41787768
OAF	-0.169017959	4.67611889	-1.969538648	0.049969	0.06739	-5.41798096
DPP9	-0.096737137	4.28513954	-1.969165208	0.050012	0.06744	-5.41870522
CTAGE1	-0.054431978	0.2356785	-1.969000749	0.050031	0.06746	-5.41902414
CHRNA1	0.10586279	0.27696786	1.967422932	0.050213	0.06771	-5.42208249
PTPRS	-0.185617062	4.79185096	-1.966900424	0.050274	0.06778	-5.42309477
CTRB1	-0.043902368	0.10617729	-1.966108701	0.050366	0.0679	-5.42462811
NCSTN	-0.148834582	6.86340138	-1.966014859	0.050377	0.06791	-5.42480982
NLRC3	-0.132094248	1.85498114	-1.965395985	0.050449	0.068	-5.42600793
U2SURP	-0.145409255	4.52803547	-1.96500393	0.050494	0.06806	-5.42676674
ABCC12	-0.094906715	0.11762305	-1.964734099	0.050526	0.06809	-5.4272889
GNA12	-0.142935085	4.32536959	-1.964319951	0.050574	0.06815	-5.42809021
ARC	-0.3723701	2.10433931	-1.963328506	0.05069	0.0683	-5.43000782
ATG9A	-0.119286883	4.64658088	-1.963140947	0.050711	0.06833	-5.43037048
SYT16	-0.024572345	0.08121207	-1.96277309	0.050754	0.06838	-5.43108167
LHX5	0.02233512	0.05760041	1.96274767	0.050757	0.06838	-5.43113081
SRGAP2	0.114099271	1.55612274	1.962626813	0.050772	0.06839	-5.43136443
MGAT2	-0.17141475	1.81810393	-1.962279612	0.050812	0.06844	-5.43203552
TSPYL2	-0.200751339	4.90256712	-1.961987295	0.050846	0.06848	-5.43260043
STEAP4	0.260241124	3.83810954	1.961731697	0.050876	0.06852	-5.43309432
PALM2-A	-0.028777786	0.12221514	-1.96161035	0.050891	0.06853	-5.43332878
SCYL2	-0.149995452	4.26311451	-1.961383414	0.050917	0.06856	-5.4337672
PPP1R1	-0.282812845	6.93867043	-1.960837302	0.050981	0.06864	-5.43482205
EEF1B2	-0.153596248	8.28811451	-1.960363405	0.051037	0.06871	-5.43573718
PCDHB1	-0.020135305	0.0271608	-1.960061252	0.051072	0.06875	-5.43632055

TEAD1	0.219718633	4.87446511	1.959737966	0.05111	0.0688	-5.43694462
GOLGA6	-0.093119218	0.87074001	-1.959266555	0.051166	0.06887	-5.43785446
MUC12	0.015565229	0.03641475	1.958996857	0.051198	0.06891	-5.43837488
BFSP2	-0.129636763	0.41874134	-1.958863989	0.051213	0.06892	-5.43863125
DHRS2	-0.115745541	0.35284921	-1.958755515	0.051226	0.06893	-5.43884054
BUB1B	-0.156256709	1.55236845	-1.957788972	0.05134	0.06908	-5.44070485
KCND1	0.138252461	1.39384165	1.957599446	0.051362	0.06911	-5.44107032
CYP2A6	0.051068657	0.17842989	1.95743184	0.051382	0.06913	-5.44139348
ZNF320	-0.150244273	3.28466435	-1.956444132	0.051499	0.06928	-5.44329736
PDXK	-0.124762074	5.09814557	-1.955678789	0.05159	0.0694	-5.44477197
DHX37	-0.111010741	3.44467057	-1.955374531	0.051626	0.06944	-5.44535804
KIRREL2	-0.050581507	0.13725327	-1.955177258	0.051649	0.06946	-5.44573799
MTHFD2	0.237291836	3.30266747	1.954549631	0.051724	0.06956	-5.44694654
ADCYAP	0.072375378	0.16726335	1.952914823	0.051918	0.06982	-5.45009275
CHAC1	-0.239884394	1.66221371	-1.952289623	0.051993	0.06991	-5.45129527
SERPINA	-0.155670029	0.32094257	-1.95152882	0.052084	0.07003	-5.45275812
ACTC1	0.150581623	0.42626694	1.950858658	0.052164	0.07013	-5.45404623
KCNH4	0.042661548	0.20156467	1.950161384	0.052247	0.07024	-5.45538599
CRACR2	0.168032265	0.81313531	1.950105349	0.052254	0.07024	-5.45549364
TAP2	0.169547362	3.37169091	1.949686618	0.052304	0.0703	-5.45629795
CSNK2A	-0.07284079	0.45459707	-1.9492037	0.052362	0.07037	-5.45722535
ELF5	0.032403175	0.10578798	1.949033398	0.052383	0.0704	-5.45755235
ANP32E	-0.136862335	6.01282779	-1.948814097	0.052409	0.07043	-5.45797339
TRIM69	0.180651733	4.00868878	1.948230659	0.052479	0.07051	-5.45909332
SELE	-0.330955505	2.23290858	-1.946846978	0.052646	0.07073	-5.46174803
AHSG	-0.072143267	0.14986978	-1.946846413	0.052646	0.07073	-5.46174911
HBE1	-0.072249447	0.3641588	-1.9449816	0.052871	0.07102	-5.46532402
TSPAN1	-0.020742334	0.07752326	-1.944810389	0.052892	0.07105	-5.46565207
SLAIN2	-0.139523246	4.77002611	-1.94471105	0.052904	0.07106	-5.4658424
HBQ1	-0.079901414	0.25679026	-1.944681721	0.052907	0.07106	-5.46589859
CPA6	-0.044384025	0.13245313	-1.944398732	0.052942	0.0711	-5.46644072
CTSF	-0.248026981	5.27634259	-1.944066324	0.052982	0.07114	-5.46707742
PDLIM2	-0.113477309	1.83183046	-1.943838874	0.05301	0.07118	-5.46751302
NECTIN1	0.185374503	2.50676595	1.942885761	0.053125	0.07133	-5.46933785
MOGAT2	-0.019697751	0.03972144	-1.942090821	0.053222	0.07145	-5.47085917
TNFSF1	0.248743331	2.35176109	1.941969478	0.053237	0.07146	-5.47109134
BPI	0.022764707	0.06960944	1.941899722	0.053245	0.07147	-5.4712248
BMPR2	-0.173765047	4.74725179	-1.941708659	0.053269	0.0715	-5.47159032
PHOSPH	-0.117607835	0.76401841	-1.940998318	0.053355	0.07161	-5.47294897
HAND2	-0.128429051	0.52488438	-1.940306042	0.05344	0.07171	-5.4742726

KLHL24	-0.152304148	4.47189443	-1.939491805	0.053539	0.07184	-5.47582884
CAMTA2	-0.10853965	5.00165449	-1.939192232	0.053576	0.07188	-5.47640125
CEP290	-0.132584445	2.78414048	-1.939020797	0.053597	0.0719	-5.47672878
AGBL3	-0.082540069	1.21153855	-1.939004028	0.053599	0.0719	-5.47676081
HUS1B	-0.071142612	0.34307154	-1.938073879	0.053713	0.07205	-5.47853738
FANK1	-0.144057458	3.76678613	-1.937292839	0.053809	0.07217	-5.48002851
C6orf141	0.090857207	0.36184724	1.936115167	0.053953	0.07236	-5.48227577
TNFAIP1	-0.121377016	5.7683411	-1.935303931	0.054053	0.07249	-5.48382302
ZCCHC1	0.154421827	4.78261433	1.934697362	0.054128	0.07259	-5.4849795
HOXA13	-0.045096799	0.07381025	-1.934321715	0.054175	0.07264	-5.48569553
DNAH11	-0.162732052	0.99268454	-1.934269601	0.054181	0.07265	-5.48579486
TSTA3	-0.136069923	5.91601748	-1.933910947	0.054225	0.0727	-5.48647835
PPFIA1	-0.148118605	4.21218872	-1.932787138	0.054364	0.07288	-5.48861923
CACNA1	-0.311157629	2.48777378	-1.932350866	0.054419	0.07295	-5.48945
SDE2	-0.172617226	4.29234544	-1.932309818	0.054424	0.07295	-5.48952816
LRCH3	-0.105519657	2.85998157	-1.932194382	0.054438	0.07296	-5.48974795
RASEF	-0.212556415	4.92815181	-1.931842843	0.054482	0.07301	-5.49041719
PDZD7	0.106867036	0.91021232	1.931801434	0.054487	0.07301	-5.49049601
KNDC1	-0.190371163	2.77955309	-1.930089184	0.0547	0.07329	-5.49375393
SLC4A11	0.310404467	4.57379514	1.929852321	0.054729	0.07333	-5.49420439
RMI1	-0.147973922	3.12479049	-1.929711378	0.054747	0.07335	-5.49447241
POU5F2	0.011762633	0.02900554	1.928876996	0.054851	0.07348	-5.49605868
MBTD1	-0.143174637	3.44871497	-1.928820892	0.054858	0.07348	-5.49616532
DRICH1	0.155742242	1.17216607	1.928795864	0.054861	0.07348	-5.49621289
CPNE9	0.043300497	0.16902429	1.928728139	0.054869	0.07349	-5.49634161
CEACAM	0.083771406	0.13833035	1.928322644	0.05492	0.07355	-5.49711221
ERI1	0.110913297	2.68525564	1.928270938	0.054926	0.07355	-5.49721046
CCNF	-0.094484774	1.51360361	-1.928117619	0.054946	0.07357	-5.49750178
PODN	0.289599099	1.90292755	1.928017594	0.054958	0.07358	-5.49769183
PSCA	-0.094797454	0.1908434	-1.927866918	0.054977	0.0736	-5.49797809
FBXO31	-0.118116687	4.19252591	-1.927826425	0.054982	0.0736	-5.49805501
ATAD5	0.077585113	1.06603421	1.927636781	0.055006	0.07363	-5.49841527
PKD1L1	-0.056045054	0.47148661	-1.926553589	0.055141	0.0738	-5.50047228
CAPRIN1	-0.156039317	6.21427272	-1.926100655	0.055198	0.07387	-5.50133208
HBP1	-0.197354397	4.75921177	-1.92609901	0.055198	0.07387	-5.5013352
SLC9B2	0.11495442	1.44531255	1.925834711	0.055232	0.07391	-5.50183683
PAX5	0.153940789	0.2875559	1.925583573	0.055263	0.07394	-5.50231342
ACSBG1	-0.04445996	0.11297578	-1.925580862	0.055263	0.07394	-5.50231856
PYCARD	0.24077674	4.11998326	1.923876926	0.055478	0.07422	-5.50555052
ATP2B2	-0.057714869	0.06737776	-1.923757018	0.055493	0.07423	-5.50577786

PNMA1	-0.171972173	6.18551585	-1.92341792	0.055536	0.07429	-5.50642068
ANKRD1	-0.040231523	0.09146672	-1.923382786	0.05554	0.07429	-5.50648727
EDNRA	0.195911506	3.16602016	1.923121913	0.055573	0.07432	-5.50698172
DNA2	-0.083384877	1.06635427	-1.92281948	0.055611	0.07437	-5.50755485
ABLIM3	-0.187644213	1.99286911	-1.921840423	0.055735	0.07453	-5.50940964
LTBP4	-0.289498493	6.50855108	-1.921367223	0.055795	0.0746	-5.51030578
PRSS27	-0.084117356	0.75342607	-1.921188007	0.055817	0.07463	-5.51064512
IMPDH1	0.13240028	4.25642098	1.921153273	0.055822	0.07463	-5.51071088
SAMD12	-0.176017258	3.46335929	-1.920850092	0.05586	0.07467	-5.51128486
PRR4	-0.085352798	0.92386619	-1.92084376	0.055861	0.07467	-5.51129685
PSORS1	0.080433691	0.29795337	1.920597492	0.055892	0.0747	-5.51176302
SYNE2	-0.192219733	4.47120011	-1.92051732	0.055902	0.07471	-5.51191476
DTL	0.169460141	1.7205232	1.920317251	0.055928	0.07474	-5.51229342
TLR7	0.162796623	1.10805492	1.920307557	0.055929	0.07474	-5.51231177
PXDC1	-0.122731955	5.84837047	-1.919809281	0.055992	0.07481	-5.51325465
GLDN	0.339145754	1.28437625	1.919417933	0.056042	0.07487	-5.51399503
SEMA4C	0.178711057	4.43996127	1.918915347	0.056106	0.07495	-5.51494564
MYMX	-0.076774559	0.39580642	-1.918704062	0.056133	0.07498	-5.5153452
FRS2	-0.160041672	3.67019121	-1.918466693	0.056163	0.07502	-5.51579404
MED23	-0.147623525	3.92247573	-1.917896513	0.056236	0.07511	-5.51687196
RARA	0.151383082	4.99319206	1.916967814	0.056354	0.07526	-5.51862699
ZNF41	-0.159103397	2.89746279	-1.916710269	0.056387	0.0753	-5.51911355
ERCC6L	0.064121855	0.58190627	1.91643038	0.056423	0.07534	-5.51964225
PCDHB1	-0.172435457	1.20195633	-1.916223827	0.056449	0.07537	-5.52003237
IL27RA	0.213690383	4.16840915	1.915910246	0.056489	0.07542	-5.52062456
ADAMTS	0.16648204	0.61094092	1.91582766	0.0565	0.07543	-5.5207805
LRCH2	0.23914456	2.06223843	1.914861043	0.056624	0.07559	-5.52260526
SSTR2	-0.128256496	1.0626805	-1.914582733	0.056659	0.07563	-5.52313048
PKIB	0.1699507	1.65553431	1.913118639	0.056847	0.07587	-5.52589228
GREB1L	0.199653899	1.86924822	1.912484368	0.056929	0.07598	-5.52708809
B2M	0.253916296	11.0716717	1.912100934	0.056978	0.07604	-5.52781081
NANOS3	-0.18963982	1.97835091	-1.91195273	0.056998	0.07606	-5.52809012
SPC25	-0.125023715	0.98882795	-1.910508023	0.057184	0.0763	-5.53081171
SLC15A3	0.196054807	2.86885507	1.91040266	0.057198	0.07631	-5.53101012
ZNF578	-0.080132842	0.58223939	-1.909956763	0.057255	0.07638	-5.53184967
OSGEP	-0.119498263	4.65848415	-1.909943335	0.057257	0.07638	-5.53187495
FBN2	0.251349853	1.28678191	1.908830067	0.057401	0.07656	-5.5339702
SPESP1	-0.297893967	1.61274469	-1.908264204	0.057475	0.07666	-5.53503474
PLBD1	0.212235265	3.54815376	1.907275562	0.057603	0.07682	-5.53689391
SLC16A4	-0.239477003	4.62882399	-1.907214764	0.057611	0.07683	-5.53700821

RAD52	-0.151964863	3.11871863	-1.905757609	0.057801	0.07707	-5.53974663
TMSB15I	0.013435607	0.06075409	1.905454032	0.05784	0.07712	-5.54031688
CDK19	0.133781661	3.42501963	1.904963428	0.057904	0.0772	-5.54123827
CCNG1	-0.143915202	6.92731307	-1.904263281	0.057996	0.07731	-5.5425528
TRNP1	0.254845085	3.27256653	1.903924811	0.05804	0.07736	-5.54318811
UBE2D1	-0.126850091	3.95156975	-1.903913946	0.058042	0.07736	-5.5432085
RNF103-	-0.022727477	0.11533139	-1.90305955	0.058154	0.07751	-5.5448117
C11orf95	-0.190864062	4.39176803	-1.902978244	0.058164	0.07751	-5.54496423
SLC4A8	0.067430837	0.21121771	1.902399126	0.05824	0.07761	-5.54605046
CYP11A1	-0.134830869	0.45408358	-1.90217936	0.058269	0.07764	-5.54646258
NUDCD3	-0.110663494	4.88900381	-1.901989832	0.058294	0.07767	-5.54681796
STRADA	-0.113361419	2.14840763	-1.90156177	0.05835	0.07774	-5.54762048
CNTLN	-0.095238971	1.47683586	-1.90134315	0.058379	0.07777	-5.54803028
GPC1	-0.150725745	4.37540429	-1.901247449	0.058392	0.07778	-5.54820965
LENEP	-0.051547638	0.19083438	-1.900397961	0.058503	0.07792	-5.54980148
CARF	-0.138856095	2.63529223	-1.900205898	0.058529	0.07795	-5.55016129
SLC18A2	0.087686366	0.27386592	1.8990966	0.058675	0.07814	-5.55223872
DKKL1	0.066555739	0.28771513	1.898984609	0.05869	0.07815	-5.55244838
TESC	-0.422459307	6.23279716	-1.898520272	0.058751	0.07823	-5.55331757
PI3	0.138901756	0.45846654	1.898317781	0.058778	0.07826	-5.55369654
PKP1	0.201681716	1.06936556	1.89743488	0.058895	0.07841	-5.55534849
KDM3B	-0.159629654	5.40076393	-1.896962763	0.058957	0.07848	-5.55623153
KLK2	-0.185493528	0.63874751	-1.896260241	0.059051	0.0786	-5.55754513
NTN1	-0.152396276	0.90641846	-1.895996679	0.059086	0.07864	-5.55803782
TPBGL	-0.10117605	0.53229353	-1.895774128	0.059115	0.07868	-5.5584538
UPP2	0.083520923	0.66289083	1.895582166	0.059141	0.0787	-5.55881257
CDK12	-0.151034079	3.49813918	-1.895450202	0.059158	0.07872	-5.55905918
TICRR	-0.058898672	0.53880479	-1.894931459	0.059227	0.07881	-5.56002843
FBRS	-0.107399748	5.41507038	-1.894869338	0.059235	0.07881	-5.56014449
CALCB	-0.28335982	0.88272669	-1.894547591	0.059278	0.07886	-5.56074552
CCDC14	0.174043518	2.9845951	1.894498559	0.059285	0.07887	-5.5608371
PDCD11	-0.128451985	3.98799767	-1.894404952	0.059297	0.07888	-5.56101193
TTC13	-0.139479814	3.09864233	-1.893916995	0.059362	0.07896	-5.56192319
SCO2	-0.124427188	4.79615127	-1.893217124	0.059456	0.07907	-5.56322978
TPCN2	-0.107868643	2.54668941	-1.893196443	0.059459	0.07907	-5.56326839
ATXN3	-0.079682646	1.83876252	-1.892308319	0.059577	0.07922	-5.56492574
NKAIN2	0.080256129	0.34645343	1.892283394	0.059581	0.07922	-5.56497224
BAZ1B	-0.140360387	5.55118634	-1.892236318	0.059587	0.07922	-5.56506007
SLC22A1	-0.01547424	0.04806689	-1.891163127	0.059731	0.07941	-5.56706171
TNMD	-0.056974826	0.14574415	-1.890980502	0.059755	0.07943	-5.56740222

CTSW	0.307579653	3.07311618	1.890951263	0.059759	0.07943	-5.56745673
PLD3	-0.189562913	7.94483007	-1.89039125	0.059834	0.07953	-5.56850067
APOC1	0.495451367	6.24118328	1.890157127	0.059866	0.07956	-5.56893702
PDCL2	0.1257765	0.43705076	1.890089537	0.059875	0.07957	-5.56906298
KLRF2	0.047630611	0.11529555	1.889840296	0.059908	0.07961	-5.56952743
ZNF888	0.15091347	1.36155665	1.889056553	0.060013	0.07974	-5.57098752
CEP55	0.148394424	1.44602575	1.888810544	0.060047	0.07977	-5.5714457
CENPF	0.153285376	1.14960699	1.888773126	0.060052	0.07977	-5.57151539
GFRA1	-0.175048546	0.682638	-1.888764107	0.060053	0.07977	-5.57153218
ART1	-0.051725923	0.21903145	-1.88865859	0.060067	0.07979	-5.57172868
OTUD1	-0.150309131	5.4578332	-1.888516847	0.060086	0.07981	-5.57199263
MMP2	0.282977986	5.70987396	1.888285678	0.060117	0.07984	-5.57242305
IL9R	0.078283566	0.30978089	1.887921584	0.060166	0.0799	-5.57310088
FAM221E	-0.053920563	0.29606147	-1.8878109	0.060181	0.07991	-5.57330691
ZNF334	-0.176804785	2.61411258	-1.886764473	0.060323	0.08009	-5.5752542
GIT2	-0.131844106	2.73743908	-1.886498661	0.060359	0.08014	-5.57574868
ZFR2	-0.13322173	0.51115973	-1.886312873	0.060384	0.08016	-5.57609426
PURB	-0.122854019	4.83792665	-1.885810837	0.060452	0.08025	-5.5770279
B3GLCT	-0.125253434	3.59054332	-1.885661074	0.060472	0.08027	-5.57730637
EIF5AL1	-0.224816278	1.4538969	-1.885521112	0.060491	0.08029	-5.5775666
CHRM5	-0.05393517	0.10363094	-1.885422639	0.060504	0.0803	-5.57774967
ACTG2	-0.268865476	2.00403258	-1.88532852	0.060517	0.08031	-5.57792465
CCER2	-0.167100554	1.07413948	-1.884613524	0.060614	0.08043	-5.57925359
PRR14L	-0.142111235	3.07520439	-1.884358795	0.060648	0.08047	-5.57972693
RNMT	-0.158601664	4.54264293	-1.884025034	0.060694	0.08052	-5.58034703
ARID2	-0.128133943	3.17989194	-1.883976689	0.0607	0.08053	-5.58043684
C17orf78	-0.02470803	0.12923125	-1.883901054	0.060711	0.08053	-5.58057735
F11R	-0.154440614	6.61070678	-1.883549186	0.060758	0.08059	-5.58123093
NSG2	-0.034746157	0.10245385	-1.883373341	0.060782	0.08062	-5.58155752
SPATA6L	-0.102619176	1.48936014	-1.883007222	0.060832	0.08068	-5.58223739
MDGA2	-0.018987075	0.02552444	-1.882139139	0.06095	0.08083	-5.58384887
ECM1	-0.545033074	5.23068092	-1.881221251	0.061076	0.08099	-5.58555203
NEURL3	0.066892255	0.19961515	1.881152817	0.061085	0.08099	-5.58567898
KHDRBS	0.138718431	1.71411201	1.880908896	0.061118	0.08103	-5.58613142
PADI4	0.035471233	0.096499	1.880352046	0.061194	0.08113	-5.58716411
RXFP4	-0.048547438	0.28742402	-1.879971221	0.061247	0.08119	-5.58787018
AUTS2	0.211686005	3.09795487	1.878718885	0.061418	0.08141	-5.5901911
COL19A1	0.153018831	0.53268375	1.878593447	0.061435	0.08143	-5.59042349
MAP3K2	-0.146792145	4.33489518	-1.878391638	0.061463	0.08146	-5.59079733
AGAP6	-0.161651323	2.57934523	-1.877802483	0.061544	0.08156	-5.59188849

ELL3	0.07801132	0.84687062	1.877035847	0.061649	0.08169	-5.59330787
SIPA1	0.146623387	4.74166207	1.876839595	0.061676	0.08172	-5.59367112
RANGAF	-0.139063854	5.47808121	-1.875825319	0.061816	0.0819	-5.59554793
RBMS2	0.146586894	5.22819045	1.875655568	0.06184	0.08192	-5.59586193
UBXN4	-0.158199267	6.4757991	-1.875289074	0.06189	0.08198	-5.59653979
CX3CL1	0.181206256	5.65543075	1.874642998	0.06198	0.0821	-5.59773443
USB1	-0.11661945	4.76270022	-1.874258215	0.062033	0.08216	-5.59844573
PPA1	-0.148520905	6.245485	-1.874224334	0.062037	0.08216	-5.59850836
HYAL3	-0.127005508	2.19887426	-1.874150049	0.062048	0.08217	-5.59864566
HOXD10	0.115376058	0.28726359	1.87284898	0.062228	0.0824	-5.6010496
NT5C1A	-0.181473025	0.92128246	-1.87268241	0.062251	0.08242	-5.60135725
RHEX	0.273386033	2.83361993	1.872536664	0.062271	0.08244	-5.60162642
MX2	0.164269052	1.49899531	1.872524228	0.062273	0.08244	-5.60164938
FBXL19	-0.128569871	3.89752019	-1.870644984	0.062534	0.08278	-5.60511815
PLA2G1E	-0.048387016	0.20688165	-1.870346328	0.062576	0.08283	-5.60566911
REPS1	-0.111924935	3.62211204	-1.86968272	0.062668	0.08294	-5.60689301
TAGLN	-0.228697545	5.90746791	-1.869248919	0.062729	0.08302	-5.60769285
DAPK1	0.221000299	2.7566151	1.868151535	0.062882	0.08321	-5.6097154
PYGL	0.179677334	3.53537587	1.867777211	0.062935	0.08328	-5.61040504
ADRA1B	-0.240172759	2.78780622	-1.867525358	0.06297	0.08332	-5.61086897
LINGO4	-0.157638277	1.24498063	-1.867395972	0.062988	0.08333	-5.61110728
ROBO2	-0.057599657	0.13888062	-1.866678872	0.063089	0.08346	-5.6124278
DDX11	-0.17468974	2.22706216	-1.866531693	0.063109	0.08348	-5.61269876
MYO15A	-0.057358386	0.43582713	-1.866439528	0.063122	0.08349	-5.61286843
HDC	0.054098724	0.15539838	1.865627588	0.063236	0.08364	-5.61436281
HUWE1	-0.160882661	5.40360776	-1.865547548	0.063247	0.08364	-5.61451009
PDZD8	-0.164403539	3.46804753	-1.86528104	0.063285	0.08369	-5.61500044
ELF1	-0.213321942	5.76925531	-1.864068823	0.063456	0.08391	-5.61722995
ANKRD2	-0.093934721	0.79721673	-1.863285289	0.063566	0.08405	-5.61867027
SON	-0.154481987	5.84879161	-1.862161412	0.063725	0.08425	-5.6207352
FCRLB	0.182769589	1.4898694	1.861986296	0.06375	0.08428	-5.62105684
DNAJC2:	-0.132744921	4.25241526	-1.861789318	0.063778	0.08431	-5.62141859
TRPC5O	0.021157451	0.07616399	1.86120215	0.063861	0.08441	-5.62249672
CBLN2	-0.015011263	0.02761291	-1.860941909	0.063898	0.08445	-5.62297446
CRTC3	-0.128484686	4.18499423	-1.860912046	0.063902	0.08445	-5.62302927
TLCD2	0.164606586	2.57607232	1.86053807	0.063955	0.08451	-5.62371567
CCDC97	-0.122757729	5.10814542	-1.859852072	0.064052	0.08464	-5.62497441
ALG10B	-0.110755297	2.06222078	-1.859098895	0.06416	0.08477	-5.62635589
KBTBD1:	-0.029433989	0.09178179	-1.858095451	0.064302	0.08495	-5.62819557
RTL4	0.415754276	2.47402159	1.85663822	0.06451	0.08522	-5.63086549

LASP1	0.15895522	6.43439836	1.856572569	0.06452	0.08523	-5.63098572
JCAD	-0.206794414	5.26525803	-1.856373944	0.064548	0.08526	-5.63134947
CTRC	-0.126521647	0.34831429	-1.856189179	0.064575	0.08529	-5.6316878
GZMB	0.257353039	1.85801596	1.855860257	0.064622	0.08534	-5.63229002
NXF1	-0.165156526	5.73072779	-1.855690794	0.064646	0.08537	-5.63260025
NAGPA	-0.100210673	3.21017314	-1.855380633	0.06469	0.08542	-5.63316798
BPTF	-0.168511766	3.90379349	-1.855317766	0.064699	0.08542	-5.63328304
ZNF699	0.141735165	1.29142065	1.855279989	0.064705	0.08543	-5.63335218
MAATS1	-0.164497164	2.66327057	-1.854402862	0.06483	0.08558	-5.63495711
SBF2	-0.171831612	3.46040764	-1.854217337	0.064857	0.08561	-5.63529648
OR2C1	-0.039802697	0.21350219	-1.854176885	0.064863	0.08561	-5.63537048
TMEM10	-0.150335753	4.35148656	-1.853932693	0.064898	0.08565	-5.6358171
RFC3	-0.136460288	3.1019549	-1.85390953	0.064901	0.08565	-5.63585947
ZRANB1	-0.184020927	3.21772491	-1.85380004	0.064917	0.08567	-5.6360597
CCDC18	-0.170484376	3.80860784	-1.85360176	0.064946	0.0857	-5.63642229
TNNT3	-0.128836605	0.46057219	-1.852898581	0.065047	0.08582	-5.63770786
OSBPL3	0.156287733	4.03765748	1.852856291	0.065053	0.08582	-5.63778516
SEC24B	-0.146354484	4.72057354	-1.852318463	0.06513	0.08592	-5.6387681
TSPOAP	-0.16597262	2.85985696	-1.85210009	0.065162	0.08595	-5.63916711
TLR1	0.143131771	1.35138736	1.851782348	0.065207	0.08601	-5.63974762
SCN1A	0.031293591	0.04613524	1.851367629	0.065267	0.08608	-5.64050516
TMEM61	0.159582268	1.10026076	1.850107705	0.065449	0.08631	-5.64280555
TSLP	-0.171549848	0.86080574	-1.849775536	0.065497	0.08637	-5.64341178
PPP1R2	-0.126929783	4.5867497	-1.849425475	0.065548	0.08643	-5.64405054
PLK4	0.103072514	1.56422504	1.848815905	0.065636	0.08654	-5.64516256
ARL6IP5	-0.141603418	7.70486085	-1.848477818	0.065685	0.0866	-5.64577916
RGS2	0.279859651	3.72351548	1.848050673	0.065747	0.08667	-5.64655804
MTMR1	-0.127593858	4.03407146	-1.847860956	0.065775	0.0867	-5.64690392
CNNM4	-0.164741262	4.08902086	-1.847635172	0.065808	0.08674	-5.64731551
RGS19	-0.123874904	4.26865862	-1.847308347	0.065855	0.0868	-5.64791121
HEXD	-0.161087109	4.43724211	-1.846447613	0.06598	0.08695	-5.64947956
HSD17B	-0.034558214	0.18204833	-1.846384775	0.06599	0.08696	-5.64959403
SMAP2	-0.154333863	5.25683009	-1.845087618	0.066179	0.0872	-5.65195618
FAM168	-0.141996935	5.20078827	-1.844821525	0.066218	0.08725	-5.65244054
MAB21L	-0.212302702	1.40268373	-1.844614997	0.066248	0.08727	-5.65281642
NAALAD	-0.079101446	0.46097567	-1.84460046	0.06625	0.08727	-5.65284288
TRIP13	0.097551466	1.00253267	1.844040925	0.066332	0.08738	-5.65386104
SPATA2	-0.145206296	4.35654643	-1.842283129	0.066589	0.08771	-5.65705767
TOP2A	0.219762929	2.43678557	1.841717225	0.066672	0.08781	-5.65808615
RIN3	0.125964646	2.61586261	1.84159208	0.066691	0.08783	-5.65831355

GABBR1	0.171823311	2.89037229	1.84102788	0.066774	0.08793	-5.65933857
OTULINL	-0.215005476	3.22473101	-1.840086994	0.066912	0.08811	-5.66104726
BBOX1	-0.297991366	2.79864366	-1.839699292	0.066969	0.08817	-5.66175109
LILRA5	0.131092899	0.93637137	1.839534376	0.066993	0.0882	-5.66205044
RELCH	-0.13006605	3.73643089	-1.838908115	0.067086	0.08831	-5.66318695
UHRF1B	-0.15529542	3.23827685	-1.838346056	0.067169	0.08842	-5.66420663
TNFRSF	-0.197364392	2.83362209	-1.838263484	0.067181	0.08843	-5.6643564
NGFR	0.420910033	3.51922739	1.838104499	0.067205	0.08845	-5.66464476
TTC21A	-0.107744758	1.74846302	-1.837321085	0.067321	0.0886	-5.66606533
GOLGA8	-0.010158485	0.01401186	-1.836348128	0.067465	0.08878	-5.66782878
HTR7	0.058409254	0.29277546	1.83536098	0.067611	0.08896	-5.66961702
INPP5B	-0.132493036	3.07203997	-1.83516857	0.06764	0.089	-5.66996546
ARL14	0.192953456	0.75007763	1.835028253	0.067661	0.08901	-5.67021955
SHOC1	-0.024646074	0.14939883	-1.835026632	0.067661	0.08901	-5.67022248
CCN6	-0.091376561	0.50346615	-1.83497569	0.067669	0.08901	-5.67031472
TSC1	-0.127748616	3.71132038	-1.834939251	0.067674	0.08901	-5.6703807
TMEM26	-0.08836367	0.45488822	-1.83464658	0.067717	0.08906	-5.67091058
AMHR2	-0.067141434	0.14273066	-1.834469199	0.067744	0.08909	-5.67123169
HIST1H3	-0.066004017	0.2128808	-1.834458711	0.067745	0.08909	-5.67125067
FGD6	-0.156274138	3.14857918	-1.834098763	0.067799	0.08915	-5.67190218
MGAT4B	0.171617135	6.44280164	1.832303411	0.068067	0.08949	-5.67514992
MYC	0.261498848	3.97834828	1.832010198	0.068111	0.08955	-5.67568004
GAL3ST2	-0.071181928	0.23334608	-1.831238105	0.068226	0.08968	-5.67707556
NEMF	-0.150879297	3.34835442	-1.831237986	0.068226	0.08968	-5.67707578
NCBP2L	-0.037901374	0.16051908	-1.831118349	0.068244	0.0897	-5.67729196
MS4A1	0.317094829	1.09204762	1.831081239	0.06825	0.0897	-5.67735902
VWA3B	-0.022743853	0.03957187	-1.828512031	0.068635	0.0902	-5.68199823
CMTM6	-0.165679967	6.66510923	-1.826983217	0.068866	0.0905	-5.6847558
KLK15	-0.201235743	0.47812726	-1.82630255	0.068968	0.09062	-5.68598281
VEZF1	-0.156818909	5.48231837	-1.82625688	0.068975	0.09063	-5.68606512
KPNA1	-0.115547522	4.11028295	-1.825964452	0.069019	0.09068	-5.68659212
ST14	0.156763335	7.34550081	1.82547185	0.069094	0.09077	-5.68747968
ZAN	-0.020130081	0.04047683	-1.825281677	0.069123	0.0908	-5.68782227
FCER2	0.184032563	0.42224126	1.824909645	0.069179	0.09087	-5.68849237
ACAP3	-0.113165834	3.29312199	-1.824683614	0.069213	0.0909	-5.68889942
ZDBF2	0.153793829	2.2879244	1.823729378	0.069358	0.09109	-5.69061736
CACUL1	-0.104164283	3.73636288	-1.822903447	0.069483	0.09124	-5.69210359
MTTP	-0.031208775	0.10466725	-1.822592824	0.069531	0.0913	-5.69266238
PITX1	-0.069513814	0.10827093	-1.822553869	0.069536	0.0913	-5.69273245
SH2D7	-0.033881334	0.12264266	-1.822123342	0.069602	0.09138	-5.69350676

CD40	0.27327744	5.35475618	1.822034385	0.069616	0.09139	-5.69366673
CARD16	-0.162512641	2.50738727	-1.821424435	0.069708	0.0915	-5.69476339
ZFP36	-0.320551292	8.22094658	-1.821416463	0.06971	0.0915	-5.69477772
AMPD3	-0.149646472	2.82522377	-1.820755285	0.06981	0.09162	-5.69596607
ADAM20	-0.022317426	0.14757735	-1.819566031	0.069992	0.09186	-5.69810248
ZNF322	-0.122216374	1.97626736	-1.819414181	0.070015	0.09188	-5.69837517
SHISA3	0.281043154	1.67961576	1.818711905	0.070123	0.09201	-5.69963602
PCDHB1	-0.104047102	0.75522557	-1.817433553	0.070319	0.09226	-5.70192993
EBLN2	0.110421465	0.88452637	1.817428796	0.070319	0.09226	-5.70193846
TCF12	-0.152710844	4.27928113	-1.816837674	0.07041	0.09237	-5.70299865
IQSEC3	0.217322163	1.08716285	1.816701441	0.070431	0.09239	-5.70324294
CDK5RA	0.221259606	4.64120106	1.816667793	0.070436	0.09239	-5.70330328
SH3TC2	0.04801447	0.22605507	1.816235238	0.070503	0.09247	-5.70407879
ANGPTL	0.193775773	4.02701239	1.815982184	0.070542	0.09251	-5.7045324
EZHIP	-0.033608525	0.1301522	-1.815849811	0.070562	0.09253	-5.70476966
ART4	-0.126529261	0.60532686	-1.815049348	0.070685	0.09269	-5.70620401
SH2D4A	0.180324468	5.26216197	1.814231977	0.070812	0.09284	-5.70766802
KNOP1	-0.102172445	2.67511039	-1.813221517	0.070968	0.09304	-5.70947699
MKLN1	-0.157249695	3.06181218	-1.812766128	0.071038	0.09313	-5.71029193
TRANK1	0.174834246	3.63939395	1.812405103	0.071094	0.09319	-5.71093785
EIF3A	-0.194264174	6.54851701	-1.81227813	0.071114	0.09321	-5.711165
C7orf57	0.081984944	0.52275869	1.811162905	0.071287	0.09343	-5.71315937
CDKL2	0.209550427	2.27346164	1.810675012	0.071362	0.09352	-5.7140315
MECP2	-0.119268056	3.85050698	-1.810602931	0.071374	0.09353	-5.71416033
RUFY4	0.087163208	0.28176213	1.809935353	0.071477	0.09366	-5.71535324
GPR15	0.063837243	0.15693738	1.809913514	0.071481	0.09366	-5.71539226
HOXB4	0.086985075	0.74227146	1.809877011	0.071486	0.09366	-5.71545747
ATP8A1	0.21476687	5.12723252	1.808524957	0.071697	0.09393	-5.71787209
FOXA1	0.098612677	0.33863475	1.806829532	0.071962	0.09427	-5.72089745
ADORA1	0.267327161	4.51657598	1.805861737	0.072113	0.09446	-5.72262317
ZNF646	-0.108778056	3.27200897	-1.803798846	0.072437	0.09487	-5.72629859
APOA2	-0.150989665	0.36089587	-1.802495035	0.072643	0.09513	-5.72861946
HPGD	-0.175426277	1.07282504	-1.802427382	0.072653	0.09514	-5.72873984
EFNA5	0.145260818	0.98563112	1.802107635	0.072704	0.0952	-5.72930874
IGF1R	0.197028127	4.70934902	1.802062898	0.072711	0.0952	-5.72938833
CCDC80	0.322209095	4.87102921	1.80177026	0.072757	0.09525	-5.7299089
ZNF581	0.146052273	5.5165074	1.801769742	0.072757	0.09525	-5.72990982
ZBTB44	-0.130788471	3.90905169	-1.800244087	0.072998	0.09556	-5.73262244
VNN3	0.017204669	0.04714111	1.799766111	0.073074	0.09565	-5.73347183
GOLGA8	-0.349442301	4.81821408	-1.799706142	0.073083	0.09565	-5.73357838

SSUH2	-0.078855829	0.45029693	-1.797093453	0.073498	0.09619	-5.7382172
CEPT1	-0.143196704	3.19489169	-1.796560033	0.073583	0.09629	-5.73916348
CDCA3	-0.098942152	1.09624356	-1.795488616	0.073754	0.09651	-5.74106332
LGR4	-0.191811577	4.25190202	-1.79531152	0.073782	0.09654	-5.74137725
SH3YL1	-0.149365882	4.32961985	-1.794657897	0.073887	0.09667	-5.7425356
VSIG10	0.124574187	4.27398372	1.794223755	0.073956	0.09675	-5.74330477
ZNF543	-0.121725074	2.44642996	-1.79406949	0.073981	0.09678	-5.74357803
SMURF2	0.14190037	4.35890385	1.793821172	0.074021	0.09682	-5.74401785
DLGAP3	-0.158733379	0.88810124	-1.79379221	0.074025	0.09682	-5.74406915
TMPO	0.139579296	4.08492795	1.79368469	0.074043	0.09683	-5.74425957
MGST1	-0.232003119	6.69154249	-1.793441206	0.074082	0.09688	-5.74469074
THRB	0.138629475	1.98878526	1.793299728	0.074104	0.0969	-5.74494125
FAHD2B	-0.101595832	1.00481629	-1.793252268	0.074112	0.0969	-5.74502528
CHST6	0.109787714	0.51264508	1.793098994	0.074136	0.09693	-5.74529665
SLC35G5	-0.03595478	0.19648229	-1.792255293	0.074272	0.0971	-5.74678999
FGF7	-0.262087197	1.17081399	-1.790507246	0.074553	0.09746	-5.74988183
COX6B2	0.060418215	0.13978353	1.790298301	0.074587	0.09749	-5.7502512
ANGPTL	0.017314729	0.08185416	1.790148554	0.074611	0.09752	-5.75051589
SLC44A4	-0.285245076	1.59189146	-1.789865273	0.074656	0.09757	-5.75101657
HIPK2	0.173605258	4.55208882	1.789703879	0.074682	0.0976	-5.75130179
NXNL2	0.163247147	1.02434683	1.789650153	0.074691	0.0976	-5.75139673
PTK6	0.080312405	0.74978945	1.789495743	0.074716	0.09762	-5.75166957
PCDHB1	-0.169189291	1.82188579	-1.789124532	0.074776	0.09769	-5.7523254
GCC2	-0.166633225	3.56833763	-1.788002205	0.074957	0.09792	-5.75430746
CRACR2	-0.192446299	3.06732252	-1.787982772	0.07496	0.09792	-5.75434177
RSPO2	-0.070000528	0.09340174	-1.787538081	0.075032	0.09801	-5.75512676
PCDHA7	0.038307731	0.16137726	1.787344516	0.075064	0.09804	-5.7554684
NYNRIN	-0.252588395	4.43208271	-1.786967295	0.075125	0.09811	-5.75613407
WASHC2	-0.113247709	4.19042482	-1.786940668	0.075129	0.09811	-5.75618105
KPNA2	-0.141784786	4.69646437	-1.786575273	0.075188	0.09818	-5.75682571
SLC44A2	-0.128665399	7.09919714	-1.786546559	0.075193	0.09818	-5.75687637
GDF5	-0.141516259	0.90140224	-1.785954566	0.075289	0.0983	-5.75792052
ING1	-0.096953008	2.93749317	-1.785238735	0.075405	0.09844	-5.75918265
CLASP1	-0.130624133	4.30435014	-1.785139311	0.075422	0.09845	-5.75935792
DR1	-0.116218123	4.00626949	-1.784981714	0.075447	0.09848	-5.75963571
PGGHG	-0.338665221	3.72589817	-1.784830488	0.075472	0.09851	-5.75990224
CDH15	0.093801833	0.38934713	1.784495771	0.075526	0.09857	-5.7604921
S1PR3	0.158928843	2.37044868	1.783315954	0.075719	0.09881	-5.7625704
RP1L1	-0.013193151	0.06404293	-1.782965103	0.075776	0.09888	-5.76318818
LMNB2	-0.132751674	4.67202286	-1.782909186	0.075785	0.09888	-5.76328663

RASSF7	-0.161404051	4.52412126	-1.782874259	0.075791	0.09888	-5.76334812
HOXB5	-0.117580767	0.77579659	-1.782738337	0.075813	0.0989	-5.76358741
HCFC1	-0.137439643	4.71188449	-1.782395597	0.075869	0.09897	-5.76419072
NHSL1	0.1107215	1.58000995	1.782353076	0.075876	0.09897	-5.76426556
CNN1	-0.242942403	2.73108637	-1.782342334	0.075877	0.09897	-5.76428446
MYLPF	0.167447138	1.29472396	1.78215739	0.075908	0.099	-5.76460995
LIG4	-0.155424142	3.51526784	-1.781210081	0.076063	0.09919	-5.76627665
F8A3	-0.013473621	0.04214564	-1.781035999	0.076091	0.09922	-5.76658284
FAM110(	-0.168125991	4.33888107	-1.780910617	0.076112	0.09924	-5.76680335
ZNF648	-0.037667832	0.06773657	-1.780813501	0.076128	0.09925	-5.76697414
NIBAN2	0.171693841	7.94415054	1.780039398	0.076255	0.09941	-5.76833516
MTF2	-0.129547808	3.63634149	-1.779392961	0.076361	0.09954	-5.76947127
CCDC16!	-0.02690884	0.0741303	-1.777831376	0.076618	0.09987	-5.77221411
UTS2R	-0.293802395	1.40678725	-1.77619537	0.076887	0.10021	-5.77508514
SLC10A6	-0.136406918	0.94197333	-1.775362515	0.077025	0.10039	-5.77654573
URB1	-0.141976132	3.41878627	-1.774400382	0.077184	0.10058	-5.7782322
SNX32	-0.065959625	0.56269238	-1.774393895	0.077185	0.10058	-5.77824357
DSCC1	-0.082221304	1.45709236	-1.774110776	0.077232	0.10063	-5.77873966
RNF43	0.147666963	2.02401877	1.77355134	0.077325	0.10075	-5.7797197
RAP1GA	0.157536039	3.73089072	1.773135863	0.077394	0.10082	-5.78044735
ZBTB8B	-0.031848928	0.14850206	-1.773118374	0.077397	0.10082	-5.78047798
OMG	0.038977735	0.27955813	1.772520459	0.077496	0.10095	-5.78152484
TRIM23	-0.123855036	3.59749246	-1.771847837	0.077608	0.10108	-5.7827021
KCNS1	-0.121349352	0.28565129	-1.77062535	0.077812	0.10134	-5.78484064
S100A14	-0.197211111	8.49263077	-1.769729081	0.077962	0.10152	-5.7864076
SNORC	0.116618775	0.96403991	1.769723706	0.077963	0.10152	-5.78641699
ANGPTL	0.298594299	5.37973465	1.769027031	0.078079	0.10167	-5.78763446
NLRP14	-0.031303467	0.14687279	-1.768659996	0.07814	0.10174	-5.78827568
SST	0.180549996	0.3708912	1.768631716	0.078145	0.10174	-5.78832509
PKP3	-0.165473423	4.77230874	-1.768415543	0.078181	0.10178	-5.78870268
RBFOX3	-0.058461825	0.07003029	-1.767655566	0.078309	0.10193	-5.79002979
STK38L	-0.134772074	3.52501501	-1.76713295	0.078396	0.10204	-5.79094209
ITSN2	-0.162851074	4.91650388	-1.766299206	0.078536	0.10221	-5.79239695
PAM	0.193581636	6.78268392	1.765800558	0.07862	0.10232	-5.79326676
ANKDD1	-0.157161862	1.27422765	-1.765680647	0.07864	0.10233	-5.79347589
CD8B	0.275878245	1.80895358	1.765126724	0.078734	0.10245	-5.79444178
ZFP57	0.14929649	0.3759102	1.765005338	0.078754	0.10247	-5.7946534
TPM3	-0.12731188	6.33612315	-1.76443103	0.078851	0.10258	-5.79565445
GK3P	0.013923498	0.03778632	1.76333028	0.079037	0.10282	-5.79757222
PPAN-P2	-0.024753295	0.14159055	-1.763126137	0.079071	0.10286	-5.79792776

NPY	-0.264143097	0.79262684	-1.762903883	0.079109	0.1029	-5.7983148
GPR55	0.14436942	0.58190727	1.761334308	0.079375	0.10323	-5.80104672
SHISA5	-0.130999639	7.00048655	-1.761038692	0.079425	0.10329	-5.80156099
SEMA4G	0.191526471	2.96708967	1.760756704	0.079473	0.10335	-5.80205147
LPAR1	-0.191764348	2.98344139	-1.759280717	0.079723	0.10366	-5.80461751
TRIB3	-0.332691148	3.69990176	-1.75904724	0.079763	0.10371	-5.80502322
EPB42	-0.02796988	0.1223357	-1.758546391	0.079848	0.10381	-5.80589337
COL4A3	0.216536953	3.77948905	1.758276351	0.079894	0.10386	-5.80636242
HIST1H2	-0.137829233	1.36797357	-1.757608289	0.080008	0.104	-5.80752253
PPIP5K2	-0.115714643	2.78485293	-1.757540883	0.08002	0.10401	-5.80763955
GNB1L	-0.101252494	1.53916382	-1.75709626	0.080096	0.1041	-5.80841138
SIGLEC7	0.160707653	1.17755203	1.756309052	0.08023	0.10427	-5.80977745
PLEKHO	-0.157170366	5.85824881	-1.755579006	0.080355	0.10442	-5.81104378
DRGX	0.086035096	0.08250883	1.754389742	0.080559	0.10468	-5.81310557
PRAF2	-0.13383875	6.4303846	-1.754149758	0.0806	0.10472	-5.81352146
ZNF841	0.123872083	2.76703235	1.754107758	0.080608	0.10472	-5.81359424
UBE2E2	0.147631176	4.77436069	1.753890227	0.080645	0.10477	-5.81397116
HTR3A	-0.136392632	0.38674357	-1.753080971	0.080784	0.10494	-5.81537297
DNAH17	-0.063838947	0.65593821	-1.753037113	0.080792	0.10494	-5.81544892
CSNK1G	-0.107053471	5.34778369	-1.752541778	0.080877	0.10503	-5.81630662
ALOX12E	-0.07546929	0.35979712	-1.752522221	0.08088	0.10503	-5.81634048
DISP3	-0.041262295	0.15415248	-1.752506338	0.080883	0.10503	-5.81636798
VPS41	-0.123277814	4.56853458	-1.752419383	0.080898	0.10505	-5.81651852
DAGLB	-0.103700537	3.76742323	-1.750774738	0.081181	0.10541	-5.81936442
NIPSNAF	-0.132680384	2.78081644	-1.750650947	0.081203	0.10543	-5.81957852
KCNJ4	-0.032896003	0.09488472	-1.750409318	0.081245	0.10547	-5.81999639
LSR	0.149025647	7.2868791	1.750180918	0.081284	0.10552	-5.82039132
MXD3	-0.13223162	1.535755	-1.750084866	0.081301	0.10553	-5.8205574
GAB3	0.11502199	2.43194124	1.750023632	0.081311	0.10553	-5.82066327
EFNA3	-0.145517154	2.34855962	-1.749851386	0.081341	0.10556	-5.82096104
RABGGT	-0.097390644	4.69181667	-1.749296459	0.081437	0.10568	-5.82192021
NPHP3	-0.138777748	2.2806589	-1.749287713	0.081439	0.10568	-5.82193532
FBXO21	-0.159808151	5.27895849	-1.748044992	0.081654	0.10595	-5.82408222
CHST5	-0.025318454	0.1319024	-1.748002548	0.081661	0.10595	-5.82415552
DOK5	-0.214858804	2.13994194	-1.747848644	0.081688	0.10597	-5.82442129
DYNC1LI	-0.213966522	4.94884534	-1.747170654	0.081806	0.10612	-5.82559181
SLC13A4	-0.067430104	0.46665445	-1.746644143	0.081897	0.10623	-5.82650051
ZNF26	-0.072348864	1.50176308	-1.746217266	0.081972	0.10632	-5.82723705
TMEM13	-0.133283454	3.8689694	-1.746078812	0.081996	0.10634	-5.8274759
BAG5	-0.128984218	5.42460791	-1.745392804	0.082115	0.10649	-5.82865909

SPAG17	-0.115570832	0.73724881	-1.743343343	0.082473	0.10694	-5.83219119
NACA2	-0.089762217	0.76954682	-1.742983047	0.082536	0.10702	-5.83281172
CYP27C1	0.045399041	0.12988185	1.742923985	0.082547	0.10702	-5.83291343
CDKL4	-0.05953068	0.17037311	-1.741853266	0.082734	0.10726	-5.83475669
XKR9	-0.099181913	0.63431128	-1.741797406	0.082744	0.10726	-5.83485283
KRT3	-0.077226955	0.15816602	-1.741446813	0.082805	0.10733	-5.83545612
PLAGL1	0.118472823	1.40153444	1.741042126	0.082877	0.10742	-5.83615235
OIP5	-0.081315323	0.96843931	-1.740820373	0.082915	0.10746	-5.83653379
WEE1	-0.213810658	4.12446989	-1.739359191	0.083173	0.10778	-5.83904601
MKX	0.284333035	1.61198602	1.738952596	0.083244	0.10787	-5.8397447
CSDC2	0.119684259	0.44896254	1.738591568	0.083308	0.10794	-5.84036496
RPL36	-0.145368615	8.68607913	-1.738560709	0.083313	0.10794	-5.84041797
LIME1	-0.15081708	1.81867332	-1.737894638	0.083431	0.10809	-5.84156195
C1QL3	-0.150243487	1.18657091	-1.737850088	0.083439	0.10809	-5.84163845
LUC7L	-0.166783573	4.51944368	-1.737703921	0.083465	0.10811	-5.84188943
CEACAM	0.054175473	0.08508875	1.737460069	0.083508	0.10816	-5.84230809
PHF24	0.059898932	0.26191254	1.737328425	0.083531	0.10818	-5.84253408
KLK5	0.308734571	1.06013111	1.736285932	0.083715	0.10841	-5.84432314
PITPNM2	-0.112113117	2.02286112	-1.73556538	0.083843	0.10857	-5.84555909
DLG5	-0.222665608	4.356739	-1.735382416	0.083875	0.1086	-5.84587285
PEG10	0.268800603	3.73742707	1.735149612	0.083917	0.10865	-5.84627202
CCND1	0.215202279	8.1201534	1.735068497	0.083931	0.10866	-5.8464111
ESPL1	0.069120806	0.51976595	1.734060964	0.08411	0.10888	-5.84813798
NWD2	-0.124169436	0.16385003	-1.733873025	0.084143	0.10892	-5.84845999
CBX3	-0.114074771	6.34708419	-1.733166559	0.084269	0.10907	-5.84967014
DNPEP	-0.090103651	4.56780356	-1.732662951	0.084359	0.10918	-5.85053251
EGFL8	-0.113123532	1.04905643	-1.732449077	0.084397	0.10922	-5.85089867
TAS2R5	0.118127616	0.96498607	1.731976277	0.084481	0.10932	-5.85170796
PRH2	-0.056958951	0.40562951	-1.731400051	0.084584	0.10945	-5.852694
RPS6KA	-0.100524045	4.67489629	-1.730767935	0.084697	0.10958	-5.8537753
SBSN	-0.247651842	0.69509563	-1.730708091	0.084708	0.10959	-5.85387765
PTCRA	0.09140781	0.46766086	1.730218239	0.084795	0.1097	-5.8547153
ANGPTL	0.06885201	0.61988729	1.729452465	0.084933	0.10986	-5.85602432
RACK1	-0.103367018	9.04544095	-1.729442889	0.084934	0.10986	-5.85604069
ATAD2	0.150231654	3.29942959	1.729231999	0.084972	0.1099	-5.85640108
VAX2	-0.090033035	0.53062981	-1.729175831	0.084982	0.1099	-5.85649706
SLC39A2	-0.065835241	0.12385866	-1.728764718	0.085056	0.10999	-5.85719948
LILRA4	0.1042767	0.39647921	1.728589102	0.085087	0.11002	-5.85749948
CHD1	0.12780306	3.46208852	1.72839229	0.085123	0.11006	-5.85783565
NUTM1	0.012284925	0.02717117	1.728373718	0.085126	0.11006	-5.85786738

CCRL2	-0.116920853	1.54160476	-1.728286947	0.085142	0.11007	-5.85801558
TWIST2	0.143914928	0.84510831	1.728247974	0.085149	0.11007	-5.85808214
GXYLT1	-0.13276038	3.90352542	-1.727871084	0.085216	0.11015	-5.85872575
TBC1D1:	-0.115535348	2.54522517	-1.727658374	0.085254	0.11018	-5.85908893
KIF4A	0.113227411	1.05646798	1.727647321	0.085256	0.11018	-5.8591078
ENTPD7	-0.13113024	2.18606309	-1.727640201	0.085258	0.11018	-5.85911996
VSTM1	0.074484536	0.24115084	1.727486287	0.085285	0.1102	-5.85938272
SUPT20I	-0.117461956	3.65722365	-1.727368987	0.085306	0.11022	-5.85958296
SHFL	0.12190794	4.43525764	1.727071564	0.08536	0.11028	-5.86009063
ZNF445	-0.110763277	2.64195371	-1.726280936	0.085502	0.11045	-5.86143972
ANKMY1	-0.091755951	2.17519145	-1.726270822	0.085504	0.11045	-5.86145698
SNCB	-0.066350952	0.09674152	-1.726168651	0.085523	0.11047	-5.86163127
PPP1R3I	-0.198420861	5.41559551	-1.725508692	0.085642	0.11061	-5.86275686
ACER1	-0.038845335	0.18089381	-1.724735961	0.085781	0.11078	-5.86407426
XAF1	0.165064686	1.56722417	1.724073568	0.085901	0.11093	-5.86520309
SLC15A4	0.126997783	4.25817095	1.723315612	0.086038	0.1111	-5.86649425
MED1	-0.141649236	3.87670373	-1.723169748	0.086064	0.11112	-5.86674266
SNX25	-0.226317178	5.37460005	-1.722252508	0.086231	0.11133	-5.86830429
PIKFYVE	-0.143347727	3.51071664	-1.721520175	0.086363	0.11149	-5.86955052
FAM171A	-0.150241651	3.72070117	-1.720687687	0.086515	0.11168	-5.87096656
RIMS1	-0.01086063	0.03711264	-1.720581245	0.086534	0.1117	-5.87114757
SLC22A1	-0.081048788	0.74723096	-1.720223814	0.086599	0.11177	-5.87175531
MOGAT1	-0.030409707	0.09261933	-1.720218958	0.0866	0.11177	-5.87176356
SEMA5A	0.172130831	2.05750244	1.720162095	0.08661	0.11177	-5.87186024
RAB44	0.022549708	0.1075443	1.719423825	0.086745	0.11194	-5.87311508
SLC36A2	0.016712517	0.02456113	1.71883276	0.086853	0.11207	-5.87411934
TMEM74	-0.161518025	1.93545601	-1.718190759	0.08697	0.11221	-5.87520976
SHMT2	-0.14480082	5.12222196	-1.718003486	0.087004	0.11224	-5.87552777
SCHIP1	-0.03760981	0.24641954	-1.71749693	0.087097	0.11236	-5.87638776
RALGPS	0.152521626	2.86575062	1.717399225	0.087115	0.11237	-5.87655361
HELZ2	0.145280791	3.30311316	1.717312012	0.08713	0.11238	-5.87670164
TTLL6	-0.015380934	0.0307399	-1.717091621	0.087171	0.11243	-5.87707569
FRMD3	-0.351729117	5.75127568	-1.716972989	0.087193	0.11244	-5.87727702
GFRA4	-0.127079322	0.75984288	-1.716571528	0.087266	0.11253	-5.87795821
TRPV5	-0.019904797	0.02994182	-1.716354661	0.087306	0.11257	-5.87832613
EFNB3	-0.180414555	1.45098013	-1.716330552	0.08731	0.11257	-5.87836703
TSPO	-0.164440395	7.16029838	-1.716085592	0.087355	0.11262	-5.87878254
CHRNE	0.122384295	1.13475561	1.715906917	0.087388	0.11265	-5.87908558
ADK	-0.139351533	5.85958852	-1.715380107	0.087484	0.11277	-5.87997889
SFI1	0.107405913	2.98686385	1.714884099	0.087576	0.11288	-5.88081973

CDH20	0.090856739	0.37018903	1.714361233	0.087672	0.11299	-5.88170584
PCDHGC	0.234826284	2.55327145	1.71430877	0.087681	0.113	-5.88179473
SHISA6	-0.298168317	1.60923786	-1.713876834	0.087761	0.11309	-5.88252652
IL1A	0.080234079	0.3459917	1.713560656	0.087819	0.11316	-5.88306208
ADCY3	-0.145196984	2.85283449	-1.712560052	0.088003	0.11339	-5.88475631
S100A16	0.139399068	7.44767481	1.71244974	0.088024	0.1134	-5.88494303
CRYBB1	-0.102929459	0.92200086	-1.712355594	0.088041	0.11342	-5.88510238
GINS1	0.110330464	1.62911248	1.712203051	0.088069	0.11344	-5.88536055
ZNF677	-0.119961394	2.49936131	-1.712187036	0.088072	0.11344	-5.88538765
HHLA2	-0.021092439	0.10045889	-1.711580432	0.088184	0.11358	-5.88641407
MTA1	-0.114107082	4.64736983	-1.711475303	0.088204	0.11359	-5.88659192
METTL27	-0.229296881	3.41766472	-1.71099105	0.088293	0.1137	-5.88741101
GMCL1	-0.129910956	4.11579006	-1.710553813	0.088374	0.1138	-5.88815037
LPIN1	-0.111847189	3.23351502	-1.709981098	0.08848	0.11392	-5.88911856
ZNF880	-0.134624088	3.64607802	-1.709524686	0.088565	0.11402	-5.8898899
ERF	-0.136887349	5.47267601	-1.708868769	0.088686	0.11417	-5.89099805
ZER1	-0.099553274	6.25668869	-1.708487086	0.088757	0.11425	-5.89164271
CLCA2	0.124183673	0.28692622	1.707934904	0.08886	0.11438	-5.89257508
TM4SF20	-0.010977727	0.02941716	-1.707318863	0.088974	0.11452	-5.89361493
MCM7	-0.132944772	5.71850887	-1.706670103	0.089095	0.11466	-5.89470961
POC1B-(	-0.015774236	0.12756354	-1.70651734	0.089123	0.11469	-5.89496732
CHST15	0.19218265	5.08260203	1.706393927	0.089146	0.11471	-5.89517549
MRGPRF	0.154335881	1.38586242	1.706065168	0.089208	0.11478	-5.89572998
PCDHB8	-0.113390012	0.46463639	-1.706030788	0.089214	0.11478	-5.89578796
GFRA3	-0.064420005	0.20837292	-1.705824037	0.089253	0.11482	-5.89613661
KHDC1L	-0.055197606	0.16539626	-1.705695846	0.089277	0.11484	-5.89635276
MEOX1	0.165809148	1.18074665	1.70486342	0.089432	0.11504	-5.89775597
TPR	-0.143151062	5.01079296	-1.704189255	0.089558	0.11519	-5.89889192
BLM	0.080734292	1.07115359	1.703704207	0.089649	0.1153	-5.89970893
LHX6	-0.11647545	1.35110585	-1.703286218	0.089727	0.11539	-5.90041281
GCGR	-0.2266793	0.46271728	-1.701451478	0.090071	0.11582	-5.90350046
CCDC18	-0.155021755	1.19690856	-1.700641918	0.090224	0.11601	-5.90486181
ZNF805	0.122207757	1.66908762	1.700539278	0.090243	0.11603	-5.90503437
FGF18	0.156973712	1.5019549	1.700212242	0.090304	0.1161	-5.9055841
SS18L1	-0.106694025	3.52427742	-1.700172661	0.090312	0.1161	-5.90565063
ENOX2	-0.12137144	3.20631871	-1.700064472	0.090332	0.11611	-5.90583246
FGD3	-0.173979809	2.44352185	-1.699259146	0.090484	0.1163	-5.90718562
FOXK1	0.124406169	3.14796906	1.698942672	0.090544	0.11637	-5.90771721
TSSK4	-0.134009906	1.32229777	-1.698365986	0.090653	0.1165	-5.90868563
RNF19A	-0.136730561	4.26038252	-1.698323098	0.090661	0.1165	-5.90875764

FCGR1B	-0.099171954	0.82733483	-1.697679541	0.090782	0.11665	-5.90983795
CCR10	-0.104342198	0.95133957	-1.697594545	0.090798	0.11665	-5.9099806
ZFHX2	0.092883593	1.4758393	1.697587985	0.0908	0.11665	-5.90999161
ZNF484	-0.11595777	2.19445178	-1.69728077	0.090858	0.11672	-5.91050715
ASCC3	0.156047774	3.60076236	1.696234275	0.091056	0.11696	-5.9122626
OAZ3	-0.110601289	2.03228104	-1.696149549	0.091072	0.11698	-5.91240467
LONRF3	-0.196343764	2.6472157	-1.695536217	0.091188	0.11712	-5.91343297
DPYD	0.201442865	4.04313851	1.695294938	0.091234	0.11717	-5.91383739
CPA5	-0.052205873	0.18082675	-1.694282922	0.091427	0.11741	-5.91553308
HPS5	-0.125907931	3.52279208	-1.693986846	0.091483	0.11747	-5.91602898
POLR3D	-0.111628572	3.77074805	-1.693726762	0.091532	0.11752	-5.91646453
LRRC19	-0.033347157	0.19000429	-1.693352723	0.091604	0.11761	-5.9170908
TRIM67	-0.026170128	0.06235615	-1.692804903	0.091708	0.11773	-5.91800779
CLCN6	-0.119015196	3.39634522	-1.692340803	0.091797	0.11784	-5.91878442
IQCB1	-0.121025909	4.20821602	-1.692068936	0.091849	0.11789	-5.91923927
ENTPD3	-0.230368382	2.85679741	-1.691992051	0.091863	0.1179	-5.91936789
MAGEC3	0.069711437	0.29313264	1.691586884	0.091941	0.11799	-5.9200456
HPSE2	0.123591378	0.64510755	1.691143823	0.092025	0.11809	-5.9207865
UBQLNL	-0.0754849	0.76725429	-1.691126174	0.092029	0.11809	-5.92081601
WHRN	-0.120441238	2.56919652	-1.690875401	0.092077	0.11814	-5.92123528
CHST4	0.040817081	0.15675752	1.690599607	0.092129	0.1182	-5.9216963
ASF1B	0.191132123	3.2103072	1.690334879	0.09218	0.11826	-5.92213876
PLSCR3	0.063025346	0.70215499	1.689136759	0.09241	0.11854	-5.92414042
TMEM21	0.15707471	2.16500962	1.689091353	0.092418	0.11854	-5.92421625
ZC3H12D	0.115889864	1.06139935	1.688890397	0.092457	0.11858	-5.92455184
EGLN3	0.192737334	1.38737249	1.688787295	0.092477	0.1186	-5.924724
CCDC27	-0.073444638	0.44236242	-1.688515084	0.092529	0.11866	-5.92517849
CIART	0.213383763	3.91873515	1.688024131	0.092623	0.11877	-5.92599801
SLC3A1	-0.029500299	0.14725441	-1.686118085	0.09299	0.11923	-5.92917747
OSBPL7	-0.107475058	3.3316941	-1.685437011	0.093122	0.11939	-5.93031271
POC1B	-0.097832286	2.83152428	-1.684872387	0.093231	0.11952	-5.93125351
TAPBP	0.099921626	7.11325014	1.684729655	0.093258	0.11955	-5.93149128
PCYT1B	-0.062164319	0.11965956	-1.68468632	0.093267	0.11955	-5.93156347
SEC24C	-0.241736873	4.53825415	-1.6846046	0.093283	0.11956	-5.9316996
ASXL1	-0.120527053	4.38576152	-1.682875267	0.093617	0.11998	-5.93457871
ZBTB9	-0.015435333	0.0548771	-1.682632542	0.093664	0.12003	-5.93498259
PLA2G4D	-0.04269314	0.06184413	-1.681697791	0.093846	0.12026	-5.9365374
EPHX4	0.203963264	2.94233675	1.680849367	0.094011	0.12046	-5.93794789
KCNA3	0.139330269	0.76281705	1.679413143	0.09429	0.12081	-5.940334
RC3H2	-0.134490917	3.63710553	-1.679243038	0.094324	0.12084	-5.94061648

CASP7	-0.115987829	3.93844324	-1.676850432	0.094791	0.12142	-5.94458668
MYBPHL	0.151631124	0.8114695	1.676845649	0.094792	0.12142	-5.94459461
DEPP1	0.215264133	5.72480902	1.676462548	0.094867	0.12151	-5.9452298
IGFBP2	-0.206843325	4.29202462	-1.676444965	0.094871	0.12151	-5.94525895
CCP110	-0.115347584	2.48594461	-1.675990944	0.09496	0.12161	-5.94601154
CCDC62	0.035225285	0.25713744	1.67530593	0.095094	0.12177	-5.94714663
AHDC1	-0.132340812	5.02231274	-1.675275156	0.0951	0.12177	-5.94719762
CAV1	0.283642847	7.21572517	1.674366853	0.095279	0.12199	-5.94870199
ZFHX3	-0.147024268	3.91000739	-1.673922495	0.095366	0.12209	-5.94943766
MACROD	0.281462483	2.27257948	1.673711084	0.095408	0.12214	-5.9497876
SUGCT	0.163691453	1.91116557	1.673268877	0.095495	0.12224	-5.95051944
ZNF117	0.132216497	2.76335929	1.673145277	0.095519	0.12226	-5.95072395
UCP1	-0.035171708	0.15080213	-1.673089071	0.09553	0.12227	-5.95081695
ARSB	-0.117762677	2.74189268	-1.672316276	0.095683	0.12245	-5.95209531
LRRC3	-0.105486658	0.96635663	-1.671863278	0.095772	0.12256	-5.95284438
TGFBI	0.267759867	4.82943192	1.670883229	0.095966	0.12279	-5.95446431
RLN1	-0.078142758	0.76503998	-1.670882701	0.095966	0.12279	-5.95446519
LRAT	-0.142714801	2.07209352	-1.670777907	0.095987	0.12281	-5.95463835
QSOX2	-0.163283114	4.31170862	-1.670277162	0.096086	0.12292	-5.95546562
FLT3LG	0.104423161	1.68105923	1.668673935	0.096403	0.12332	-5.95811266
CTRB2	-0.028767717	0.07600598	-1.668145158	0.096508	0.12344	-5.95898517
TNFAIP3	0.203678681	3.44396551	1.667951081	0.096547	0.12349	-5.95930533
C4A	0.286352833	4.01150263	1.666087908	0.096918	0.12395	-5.96237714
PDLIM1	0.209467053	8.64537349	1.664414566	0.097252	0.12437	-5.9651331
WNT1	0.048258383	0.17863052	1.664300839	0.097275	0.12439	-5.96532031
RIF1	-0.126286014	3.04923668	-1.664148517	0.097305	0.12442	-5.96557103
C1orf21	-0.156555645	3.00796221	-1.663882789	0.097358	0.12448	-5.96600836
MGAT4C	0.205936878	2.86755912	1.663683525	0.097398	0.12451	-5.96633626
ANKFN1	-0.04349238	0.05200041	-1.663676775	0.0974	0.12451	-5.96634737
HRH2	0.090609076	0.42851324	1.663605362	0.097414	0.12452	-5.96646488
CTF1	0.223402117	4.86394682	1.663392458	0.097457	0.12456	-5.96681516
NXPE3	-0.135286285	2.7698749	-1.663263054	0.097482	0.12459	-5.96702805
UBE2L5	-0.068709127	0.61459556	-1.663179099	0.097499	0.1246	-5.96716616
HIP1R	-0.111271596	4.75571781	-1.662560225	0.097623	0.12475	-5.96818401
ADAMTS	-0.091467952	0.41472922	-1.66249283	0.097637	0.12476	-5.96829483
CEP295N	0.021950748	0.07517508	1.661038145	0.097929	0.12512	-5.97068576
A4GNT	-0.025442474	0.10703601	-1.660776422	0.097982	0.12518	-5.97111571
ZNF486	0.288619391	5.55504092	1.660360806	0.098065	0.12528	-5.97179834
ZNF532	0.145528689	4.28369875	1.659864489	0.098165	0.12539	-5.9726133
OR13C5	-0.025645829	0.09561037	-1.658694315	0.098401	0.12569	-5.97453379

NMD3	-0.111873647	4.6091628	-1.658641077	0.098412	0.12569	-5.97462114
KDM5B	0.142750448	4.03287954	1.658384488	0.098464	0.12575	-5.97504206
TMF1	-0.149038394	4.64485235	-1.658197863	0.098501	0.12579	-5.97534817
MSS51	0.103424548	1.26944531	1.657987864	0.098544	0.12583	-5.97569258
ATP11A	0.26890467	5.20177571	1.65793794	0.098554	0.12583	-5.97577445
SCRN1	0.181943116	4.90955467	1.657910501	0.098559	0.12583	-5.97581945
C10orf95	0.18953476	2.99210096	1.657875858	0.098566	0.12583	-5.97587626
MAP3K1:	-0.098811381	4.79541568	-1.657657084	0.098611	0.12588	-5.97623499
IPO5	-0.135656663	5.4642485	-1.657474819	0.098647	0.12591	-5.97653382
NAALAD	-0.119979002	1.47666403	-1.657384874	0.098666	0.12593	-5.97668128
CDCA7	-0.136494049	1.23320351	-1.656729137	0.098798	0.12609	-5.97775607
COL20A:	-0.01223548	0.02761691	-1.656399179	0.098865	0.12616	-5.97829673
EFCAB1(	0.063638294	0.64863305	1.655932416	0.09896	0.12628	-5.97906138
PCSK1N	0.480038515	4.12106303	1.655529888	0.099041	0.12636	-5.97972062
TTLL3	0.163314985	2.64003895	1.655527996	0.099042	0.12636	-5.97972372
RAP2A	0.139346275	4.24659736	1.65535945	0.099076	0.1264	-5.97999971
PKD1	-0.105081112	2.86251004	-1.654908063	0.099168	0.1265	-5.98073872
RAD54L	-0.088427521	1.16734032	-1.654811554	0.099187	0.12652	-5.9808967
HOXC10	0.114163082	0.15871794	1.654649252	0.09922	0.12655	-5.98116235
EPOR	-0.229830756	5.62907153	-1.654520361	0.099246	0.12657	-5.98137331
ESRP2	-0.145388032	4.41536925	-1.654110162	0.09933	0.12667	-5.98204455
CAPZA1	-0.111024917	6.38302655	-1.653731086	0.099407	0.12676	-5.98266473
FOXH1	-0.031064267	0.12783326	-1.653457027	0.099463	0.12682	-5.98311301
NR1I2	-0.039796515	0.31492862	-1.653388497	0.099476	0.12683	-5.98322509
BTBD10	-0.118802388	4.42787759	-1.652172935	0.099724	0.12714	-5.98521243
CREBBP	-0.132308356	4.48968496	-1.652115874	0.099736	0.12714	-5.98530568
CA6	0.024108083	0.06343847	1.651801565	0.0998	0.12721	-5.9858193
PDE6A	0.020114797	0.07210986	1.651367709	0.099889	0.12732	-5.98652811
TAS2R1(	-0.056689936	0.34999108	-1.651149998	0.099933	0.12736	-5.98688373
ACSBG2	0.019141926	0.10516811	1.649981255	0.100172	0.12766	-5.98879201
TSKU	-0.198314233	5.40261667	-1.649908939	0.100187	0.12767	-5.98891005
CARD6	-0.122246073	3.45704943	-1.649861517	0.100197	0.12767	-5.98898745
CENPK	0.106263251	1.26234124	1.649647303	0.10024	0.12772	-5.98933704
ATP6V1E	-0.056399762	0.31120811	-1.649308656	0.10031	0.1278	-5.98988961
NRBP2	0.157083565	4.473735	1.648806623	0.100413	0.12792	-5.99070858
PPP4R1	-0.129598388	4.85848254	-1.648650825	0.100445	0.12795	-5.99096269
HOXA5	-0.082441647	0.61691006	-1.648315978	0.100513	0.12803	-5.99150874
CYB5RL	-0.078954452	1.07675809	-1.648127398	0.100552	0.12807	-5.99181622
ADAM28	0.175326906	1.2501387	1.646888412	0.100807	0.12838	-5.99383553
ATP6V0A	-0.070145911	0.11944507	-1.646844289	0.100816	0.12838	-5.99390741

RAB8A	-0.078021171	4.11618532	-1.645592279	0.101074	0.1287	-5.99594638
FBXO2	0.23624606	5.0452645	1.645578443	0.101077	0.1287	-5.9959689
SRRM3	-0.145958879	1.6948981	-1.645391357	0.101115	0.12874	-5.99627345
GDPD3	-0.127695528	1.80099266	-1.645201486	0.101154	0.12878	-5.9965825
EEF1G	-0.101366195	0.81774517	-1.644956398	0.101205	0.12883	-5.99698137
ATP1A2	-0.152815352	1.08658027	-1.644733014	0.101251	0.12888	-5.99734486
PCDH20	0.021242613	0.0524623	1.644660482	0.101266	0.12889	-5.99746288
STXBP5	-0.125392661	2.80281716	-1.644163266	0.101369	0.12901	-5.99827175
KIF2C	-0.127797791	1.8682361	-1.643736497	0.101457	0.12911	-5.99896584
LCORL	-0.091283351	1.9547358	-1.643499552	0.101506	0.12917	-5.99935112
EPHA1	-0.15417456	3.28815526	-1.643060541	0.101597	0.12927	-6.00006482
CAVIN3	-0.177636375	3.94303715	-1.641194008	0.101984	0.12975	-6.00309718
GABRE	0.206140453	1.93338428	1.641190323	0.101985	0.12975	-6.00310316
TMX3	-0.121199779	4.23115156	-1.640781674	0.10207	0.12984	-6.0037666
TRAF1	0.14683661	3.08430564	1.640681398	0.102091	0.12986	-6.00392937
SNCAIP	0.121474731	0.66759449	1.640456412	0.102137	0.12991	-6.00429454
ZNF99	-0.032105032	0.16964619	-1.638628533	0.102518	0.13038	-6.00725953
ZNF106	-0.149670888	4.17422913	-1.638026371	0.102644	0.13053	-6.00823559
DPY19L4	-0.140944736	4.27225568	-1.637354317	0.102784	0.1307	-6.00932451
SMAD3	-0.160105523	5.12369574	-1.637092513	0.102839	0.13076	-6.00974859
GATD3A	-0.200023957	1.05662632	-1.63675406	0.10291	0.13084	-6.01029674
CGN	0.200485479	3.98969072	1.636241074	0.103017	0.13097	-6.01112733
ASIC4	0.013487123	0.02366952	1.636197627	0.103026	0.13097	-6.01119767
CCDC19	0.029743473	0.09677311	1.635164516	0.103242	0.13124	-6.0128696
USP35	-0.118282802	3.12437181	-1.634721388	0.103335	0.13135	-6.01358642
PROCR	-0.159523188	4.49321972	-1.634364258	0.10341	0.13143	-6.01416399
LMCD1	0.138447688	2.12480944	1.634088078	0.103468	0.13149	-6.01461056
CMTM8	-0.149144569	5.43489264	-1.633955028	0.103496	0.13152	-6.01482566
CATSPE	-0.018971769	0.03950199	-1.633871972	0.103514	0.13153	-6.01495994
CPLANE	0.15308443	2.90834441	1.633723337	0.103545	0.13156	-6.01520021
HBG2	-0.040210333	0.1765865	-1.6331616	0.103663	0.1317	-6.01610808
FBXO11	-0.173649885	3.59417438	-1.632105029	0.103886	0.13198	-6.01781486
CACNA1	-0.081196655	0.42829391	-1.631782556	0.103954	0.13205	-6.01833556
BEND4	0.023305248	0.04552316	1.631702481	0.103971	0.13206	-6.01846485
DSG3	0.040429105	0.06828255	1.631176686	0.104082	0.13219	-6.01931361
PIP5K1A	-0.13913272	4.85825565	-1.630840604	0.104153	0.13227	-6.01985598
TTK	-0.083267491	0.93647093	-1.630657369	0.104191	0.13231	-6.02015165
TUFT1	-0.126167113	3.62951554	-1.630343278	0.104258	0.13239	-6.02065838
CLK4	-0.128834731	3.45548269	-1.630288343	0.104269	0.13239	-6.020747
EIF4EBP	-0.118515027	5.90500967	-1.630224406	0.104283	0.1324	-6.02085013

PRSS53	0.055603277	0.46984898	1.629780791	0.104377	0.13251	-6.02156562
STK4	0.099434624	3.55208984	1.629272476	0.104484	0.13264	-6.02238522
ADGRA2	-0.179902274	3.1779539	-1.628525254	0.104642	0.13282	-6.02358957
TBX19	-0.090601551	1.56135959	-1.628523592	0.104643	0.13282	-6.02359225
SIX1	-0.108620383	0.83640603	-1.62773578	0.10481	0.13302	-6.02486144
ZNF670-:	-0.013513139	0.06538328	-1.626935682	0.10498	0.13323	-6.02614981
NTN4	0.169695055	3.30021924	1.626524971	0.105067	0.13333	-6.02681092
NCAPG	0.102855761	0.8601369	1.626119341	0.105154	0.13343	-6.0274637
IFFO1	-0.132656097	2.21839121	-1.625541649	0.105277	0.13357	-6.02839309
CEBPD	-0.240394795	6.53446593	-1.625167412	0.105356	0.13366	-6.02899499
DDR2	-0.191774162	2.47795172	-1.624809142	0.105433	0.13375	-6.02957108
DDX17	-0.148941913	7.23649953	-1.624302953	0.105541	0.13388	-6.03038482
CLDN8	-0.268542686	4.25682476	-1.624160165	0.105571	0.1339	-6.03061432
ADCY6	-0.131654552	4.07130439	-1.624142163	0.105575	0.1339	-6.03064325
CCNI2	0.093416108	1.07509191	1.624040262	0.105597	0.13392	-6.03080701
CHPT1	-0.172729833	5.75990788	-1.622836012	0.105854	0.13423	-6.03274162
WFDC13	-0.026910294	0.07676105	-1.622500455	0.105926	0.13432	-6.03328044
NFYA	-0.130771524	4.82950245	-1.621825595	0.106071	0.13449	-6.03436376
PAPPA2	-0.056914867	0.25358777	-1.621088242	0.106229	0.13468	-6.03554689
SLC39A8	-0.146034706	3.73761007	-1.62096491	0.106255	0.1347	-6.03574473
LMF1	-0.112581279	3.21701818	-1.620768329	0.106297	0.13475	-6.03606005
ACAD11	-0.050346096	0.51001002	-1.620657444	0.106321	0.13477	-6.03623789
DOCK6	-0.116249745	4.35915766	-1.620346676	0.106388	0.13484	-6.03673625
CASP1	0.156363789	2.68691853	1.61950305	0.106569	0.13506	-6.03808865
SLC6A11	-0.031583304	0.05407177	-1.619257642	0.106622	0.13512	-6.03848193
TXLNG	-0.118715684	4.23321554	-1.61914922	0.106645	0.13513	-6.03865566
NHLH2	0.016706062	0.02777379	1.619107793	0.106654	0.13513	-6.03872204
ARL8B	-0.109432713	6.7233041	-1.619084213	0.106659	0.13513	-6.03875982
PTGS1	0.149056231	2.25399682	1.618311813	0.106826	0.13534	-6.03999712
PRG4	0.118536807	0.42484347	1.617201964	0.107065	0.13563	-6.04177397
PCM1	-0.12324371	5.10852406	-1.615832226	0.107361	0.13599	-6.04396524
RAB40AI	-0.019015824	0.07926288	-1.615246485	0.107488	0.13614	-6.04490174
HIST1H1	-0.046282593	0.1881886	-1.61515889	0.107507	0.13616	-6.04504176
SLC46A2	0.024235262	0.11350632	1.614072061	0.107743	0.13645	-6.04677844
NLGN4X	0.077348407	0.57023843	1.613434833	0.107881	0.13661	-6.04779615
SLC29A3	-0.114937462	3.64892303	-1.61265933	0.10805	0.13681	-6.04903418
DYNC1I2	-0.129241492	5.64742548	-1.610939229	0.108424	0.13728	-6.05177808
DACH1	-0.053152341	0.34027029	-1.610636664	0.10849	0.13735	-6.05226044
AMER1	-0.103087065	1.68977333	-1.609029024	0.108841	0.13779	-6.05482188
EGFR	0.209838842	4.63389265	1.608789208	0.108894	0.13784	-6.05520376

ZNF331	-0.144587434	3.61971568	-1.608651499	0.108924	0.13787	-6.05542303
CMTM7	0.141809518	4.35263901	1.603940059	0.109959	0.13917	-6.06291357
MMS19	-0.096212265	5.01023266	-1.603818911	0.109986	0.13919	-6.0631059
ANKRD6	-0.073123524	0.72169401	-1.603790674	0.109992	0.13919	-6.06315072
BHLHA1!	-0.204870907	1.04459231	-1.603594916	0.110035	0.13924	-6.06346146
EPS8	0.219412394	5.68364597	1.603503874	0.110055	0.13925	-6.06360596
ANKRD2	-0.098041396	2.0139753	-1.60296966	0.110173	0.13939	-6.0644537
ROCK1	0.148680727	3.84099645	1.602887744	0.110191	0.1394	-6.06458367
PCDH19	-0.043458577	0.2402052	-1.601711129	0.110452	0.13972	-6.06644976
PTPRG	-0.179181406	4.41286809	-1.60135949	0.110529	0.13981	-6.0670072
C1orf159	-0.096222678	2.23607887	-1.601305259	0.110541	0.13981	-6.06709316
CLDN18	-0.024243544	0.11594023	-1.601113398	0.110584	0.13986	-6.06739724
ZNF354E	-0.109666353	2.73344627	-1.600007917	0.110829	0.14015	-6.06914866
TSPAN3:	0.100811657	1.12353534	1.599962673	0.110839	0.14015	-6.06922031
RIPPLY3	-0.098508523	0.91911942	-1.59994459	0.110843	0.14015	-6.06924895
TNNT1	0.254292098	0.55497441	1.599625727	0.110914	0.14023	-6.06975389
TK1	0.168274503	3.98207801	1.598822436	0.111092	0.14045	-6.0710255
BROX	-0.108969064	4.94910287	-1.598730877	0.111113	0.14046	-6.0711704
PRRT3	-0.138875306	3.47829409	-1.598495227	0.111165	0.14052	-6.07154329
KDM3A	-0.117601127	3.75325629	-1.597866069	0.111305	0.14068	-6.07253861
ABCG4	0.102282259	1.05182348	1.596169946	0.111683	0.14115	-6.07521995
PITX2	0.024445694	0.03688381	1.593627149	0.112252	0.14186	-6.07923454
TUBB2B	-0.151010956	0.73251663	-1.593422414	0.112298	0.1419	-6.0795575
HIGD1C	-0.059107963	0.37599005	-1.593401501	0.112303	0.1419	-6.07959049
POLR1E	-0.109518724	4.65838059	-1.592038916	0.112609	0.14228	-6.08173886
TOP3A	-0.084447209	3.15710524	-1.591753762	0.112673	0.14235	-6.08218824
N4BP2L2	-0.109174588	3.99251618	-1.590880771	0.112869	0.14258	-6.08356349
TICAM1	0.101660672	4.69102929	1.590879639	0.11287	0.14258	-6.08356527
UHRF2	-0.105618909	3.25959545	-1.590713369	0.112907	0.14261	-6.08382712
PPIAL4A	-0.029966806	0.14953104	-1.590241328	0.113013	0.14274	-6.08457035
BHMG1	-0.01127216	0.03178768	-1.589744259	0.113126	0.14287	-6.08535276
GCNT1	0.184250678	4.72947907	1.589332767	0.113218	0.14298	-6.08600029
A4GALT	-0.159470488	3.93383512	-1.588962645	0.113302	0.14307	-6.08658257
EVA1C	0.130313388	3.886337	1.588458908	0.113416	0.1432	-6.08737485
KRT86	-0.124225092	2.680234	-1.587408043	0.113654	0.14349	-6.08902686
ABCB9	-0.080065786	1.50086598	-1.587261295	0.113687	0.14352	-6.08925747
PCDHA4	0.046678192	0.15561642	1.586955519	0.113756	0.1436	-6.08973792
TWF2	-0.120243454	6.0727866	-1.586831513	0.113784	0.14363	-6.08993274
MPP3	-0.121010694	1.80989675	-1.585842272	0.114008	0.1439	-6.09148634
EMB	0.200185948	5.84922731	1.585490564	0.114088	0.14399	-6.09203846

DCST1	0.054102867	0.43138378	1.584839557	0.114236	0.14416	-6.09306013
NUP98	-0.127463253	5.02996651	-1.584824294	0.11424	0.14416	-6.09308408
IL27	0.038792579	0.19753044	1.583892148	0.114452	0.14441	-6.09454622
EFHD2	-0.191930948	7.93207117	-1.583870901	0.114457	0.14441	-6.09457954
INO80D	-0.108539942	2.80980843	-1.583593206	0.11452	0.14448	-6.09501495
SCRG1	0.044036478	0.17210069	1.58349877	0.114541	0.14449	-6.09516301
SZT2	-0.104038003	3.32245777	-1.583473312	0.114547	0.14449	-6.09520292
SLC9A4	-0.025924857	0.06280543	-1.582632341	0.114739	0.14472	-6.09652098
EXD3	-0.119814302	3.14306755	-1.579508916	0.115453	0.14561	-6.10141034
NUB1	-0.09489077	5.74183933	-1.579287712	0.115504	0.14567	-6.10175625
PROCA1	0.08171099	0.89060525	1.57832793	0.115724	0.14593	-6.10325657
ENTPD8	0.199220059	1.82279778	1.577689796	0.115871	0.14611	-6.1042536
CSMD3	-0.018527096	0.02064896	-1.577331768	0.115953	0.1462	-6.10481282
EFCAB1:	-0.207012907	2.76564762	-1.577135087	0.115998	0.14625	-6.10511997
GTPBP2	-0.110449898	5.09038725	-1.576661893	0.116107	0.14637	-6.10585878
CELA2A	-0.016692862	0.06051119	-1.576086443	0.11624	0.14653	-6.10675696
TNFSF1(	-0.159865488	4.14357484	-1.576007293	0.116258	0.14653	-6.10688047
ZMYM5	-0.12125871	3.90895973	-1.575992158	0.116261	0.14653	-6.10690409
DNAJB1	-0.146134651	6.30572547	-1.575299334	0.116421	0.14672	-6.10798497
HVCN1	-0.11575024	2.20035004	-1.575172927	0.11645	0.14674	-6.10818213
TMEM82	-0.046309342	0.08163954	-1.575165425	0.116452	0.14674	-6.10819383
ZFAT	-0.111792652	2.31365553	-1.574046946	0.116711	0.14706	-6.10993766
PARP10	-0.111617486	4.97426893	-1.573547546	0.116826	0.14719	-6.11071589
CATSPE	0.098937798	1.48226725	1.572633816	0.117038	0.14745	-6.11213915
TRIM56	-0.110102779	4.84698106	-1.572536224	0.11706	0.14746	-6.11229111
DAB2	-0.136011357	3.67205367	-1.57198706	0.117188	0.14761	-6.11314607
KCNG1	-0.069965365	0.20162685	-1.571601857	0.117277	0.14772	-6.11374559
ATP7A	-0.124024581	2.90710106	-1.57080707	0.117462	0.14794	-6.11498212
SGPL1	0.128158996	5.37067463	1.570584318	0.117513	0.14799	-6.11532857
ZNF319	-0.139972858	4.88226684	-1.569731145	0.117712	0.14823	-6.11665508
RNF224	0.158812363	1.84788721	1.569640828	0.117733	0.14825	-6.11679546
UPK1A	-0.046725239	0.1011321	-1.569377281	0.117794	0.14831	-6.11720505
TEPP	0.265346102	2.84929039	1.569333077	0.117804	0.14831	-6.11727374
SLC8B1	0.094420171	3.58720842	1.568223268	0.118063	0.14863	-6.11899778
AKR1B1(	-0.193867039	0.3159323	-1.567996147	0.118116	0.14868	-6.11935045
UAP1	-0.132043196	4.97132304	-1.567796622	0.118163	0.14873	-6.11966023
U2AF1L5	-0.037208552	0.18513229	-1.567026358	0.118343	0.14895	-6.12085577
PTPN3	-0.119327051	3.70300841	-1.566065774	0.118567	0.14921	-6.12234591
HCN2	-0.154790458	0.58347934	-1.566043457	0.118573	0.14921	-6.12238051
PACSIN:	-0.145063732	5.35007405	-1.56579036	0.118632	0.14928	-6.12277298

DSC1	0.011677586	0.02617293	1.565376616	0.118729	0.14939	-6.12341443
FUT6	0.033786733	0.05030839	1.563867462	0.119083	0.14982	-6.12575273
IMPDH2	-0.113488621	7.35452022	-1.563812635	0.119096	0.14983	-6.12583764
SYNRG	-0.109155975	3.67560606	-1.563329669	0.119209	0.14996	-6.12658546
WDR75	-0.086786653	4.103601	-1.563060757	0.119273	0.15003	-6.12700174
GPR78	0.023581986	0.09984419	1.562616034	0.119377	0.15015	-6.12769003
RRP9	-0.094698079	5.45538128	-1.56172849	0.119586	0.1504	-6.12906308
TNFRSF!	0.120539844	0.95435843	1.561586523	0.11962	0.15043	-6.12928264
ZDHHC2	-0.158998349	5.54171797	-1.560576435	0.119858	0.15072	-6.13084421
ZBTB42	-0.138154597	4.59759281	-1.560384212	0.119903	0.15077	-6.13114127
DTHD1	0.049611372	0.15743991	1.560246647	0.119936	0.1508	-6.13135384
MINK1	-0.115076341	4.91592859	-1.559890652	0.12002	0.15089	-6.13190384
PROX2	-0.044008298	0.33093022	-1.557605719	0.120561	0.15156	-6.1354311
KRBA2	-0.075393711	0.73515122	-1.557494691	0.120587	0.15158	-6.13560237
INSM1	0.027725261	0.07689387	1.55611669	0.120915	0.15197	-6.13772699
ANKRD1	-0.132319887	4.4403405	-1.556084254	0.120922	0.15197	-6.13777698
ABLIM1	0.168454397	5.96045988	1.556068706	0.120926	0.15197	-6.13780094
FOXG1	0.036233929	0.09631862	1.555729584	0.121007	0.15206	-6.13832351
C12orf42	0.017264025	0.03733911	1.553658648	0.1215	0.15266	-6.14151226
GMNC	-0.056406203	0.09910607	-1.553657439	0.1215	0.15266	-6.14151412
C8B	-0.075655024	0.21264055	-1.553579332	0.121519	0.15267	-6.1416343
GDF3	0.056949703	0.42742675	1.55344433	0.121551	0.1527	-6.14184202
PLAC8	0.148289457	1.32045896	1.552982763	0.121662	0.15283	-6.14255206
CABYR	0.116142301	0.67870174	1.552070331	0.12188	0.15309	-6.14395507
AGTPBP	-0.103585638	3.10679806	-1.551676828	0.121974	0.1532	-6.14455989
HMG20A	-0.151713381	3.35720581	-1.551533105	0.122008	0.15323	-6.14478076
ARPC1B	-0.113094997	6.63334409	-1.55146548	0.122024	0.15324	-6.14488468
CHRNA3	-0.032120841	0.12806502	-1.551172661	0.122095	0.15331	-6.14533459
FAM169/	-0.180349711	1.91296816	-1.551140317	0.122102	0.15331	-6.14538428
TVP23A	-0.061394663	0.62400281	-1.550072393	0.122358	0.15363	-6.14702439
RRM1	-0.127790182	5.00109279	-1.547716024	0.122925	0.15432	-6.15063936
APOL3	-0.166383686	3.35415873	-1.547633899	0.122945	0.15434	-6.15076525
TOP1	-0.140490946	6.46357295	-1.547560072	0.122962	0.15435	-6.15087842
UAP1L1	-0.118460266	3.48647153	-1.547506191	0.122975	0.15435	-6.15096101
ROS1	0.044537473	0.07283448	1.547385469	0.123005	0.15438	-6.15114604
KAT6B	-0.134127578	3.67627481	-1.547330764	0.123018	0.15438	-6.15122988
MAU2	-0.107016787	4.05192364	-1.547120215	0.123068	0.15444	-6.15155255
EPB41L3	-0.158623421	2.12818898	-1.546833979	0.123138	0.15451	-6.15199114
C11orf21	0.101491546	0.77927696	1.546805285	0.123144	0.15451	-6.1520351
RAET1G	0.169169199	2.12330824	1.546490028	0.12322	0.15459	-6.15251806

MX1	0.167532194	4.73880036	1.545785534	0.123391	0.15479	-6.15359695
FILIP1L	-0.177670076	3.27168635	-1.545685587	0.123415	0.15481	-6.15374998
HSPB2-C	-0.012816567	0.04804092	-1.545226957	0.123526	0.15494	-6.15445204
PIGL	-0.110285741	2.74993199	-1.544462988	0.12371	0.15516	-6.15562106
FGF10	0.069333291	0.16665077	1.544137018	0.123789	0.15525	-6.15611968
CHL1	0.177252853	0.6009806	1.544018971	0.123818	0.15527	-6.15630023
PCDHGE	-0.146290767	1.5693198	-1.542919355	0.124084	0.1556	-6.15798138
ADAMTS	0.183541491	2.40408277	1.542681262	0.124142	0.15565	-6.15834524
HYAL2	-0.12043684	5.99936744	-1.542677243	0.124143	0.15565	-6.15835138
ANKRD5	0.050702577	0.26910587	1.540820573	0.124595	0.1562	-6.16118685
HERC4	-0.089045811	2.96184035	-1.539529252	0.124909	0.15658	-6.16315695
ACVR1	0.17112938	6.24900235	1.539315413	0.124961	0.15664	-6.16348304
TDRD1	-0.040093957	0.06530313	-1.539264529	0.124974	0.15664	-6.16356063
TMC2	-0.01780989	0.08442478	-1.538559802	0.125146	0.15685	-6.16463493
JPT1	0.111189811	4.54990121	1.538056577	0.125269	0.15699	-6.16540176
ABI1	-0.108770382	5.30782726	-1.537901858	0.125307	0.15702	-6.16563748
LAMB1	-0.19245722	6.34537148	-1.537543831	0.125394	0.15712	-6.16618285
PLAAT5	0.058025806	0.34444182	1.536278091	0.125704	0.1575	-6.16810991
SPNS3	0.078176125	0.67697972	1.535602369	0.12587	0.1577	-6.16913804
CASQ1	0.081882928	0.32751604	1.534564713	0.126125	0.158	-6.17071601
PCDHA1	-0.15937161	1.29808122	-1.534185118	0.126218	0.15811	-6.17129299
SYNJ2	0.114217971	3.2946665	1.533281643	0.12644	0.15837	-6.17266572
GATA1	0.029905757	0.18876678	1.532932026	0.126526	0.15847	-6.17319671
TNKS2	-0.137164037	5.38654086	-1.532444558	0.126647	0.15861	-6.17393687
SLX1B	0.013543574	0.04512306	1.531948812	0.126769	0.15875	-6.17468936
SMC6	-0.098307844	2.80458558	-1.531761785	0.126815	0.1588	-6.17497318
CHEK2	0.096457638	2.64295978	1.53132243	0.126923	0.15892	-6.1756398
CCNA2	0.123716751	1.72625516	1.53112172	0.126973	0.15897	-6.17594426
LDHAL6/	-0.068912991	0.2277326	-1.531103959	0.126977	0.15897	-6.1759712
GLYATL:	-0.0411111	0.09299655	-1.531044836	0.126992	0.15897	-6.17606088
PTGFRN	0.133138333	4.58760319	1.530771634	0.12706	0.15904	-6.17647523
NPIPB3	0.068857423	0.59438805	1.529245865	0.127437	0.15951	-6.17878794
RCSD1	-0.154917295	2.55229438	-1.528998808	0.127498	0.15957	-6.1791622
SPATA1	-0.045013143	0.49859687	-1.528521463	0.127617	0.15971	-6.17988517
DEFB134	-0.042095334	0.11443135	-1.5284543	0.127633	0.15972	-6.17998687
GPRIN3	0.115485262	1.44533383	1.528019282	0.127741	0.15984	-6.18064551
GOLGA3	-0.098466894	4.70720227	-1.526494937	0.12812	0.1603	-6.18295197
DMTF1	-0.13894723	4.16054822	-1.52530429	0.128417	0.16066	-6.18475195
TUBB4A	-0.106246767	0.38353386	-1.524446376	0.128631	0.16092	-6.18604806
C2orf91	0.015974649	0.06050603	1.523820075	0.128787	0.1611	-6.18699381

COLQ	-0.111028083	1.47812082	-1.523207816	0.12894	0.16128	-6.18791798
ETS1	-0.164056418	5.60416164	-1.52286054	0.129027	0.16138	-6.18844201
RIPK4	-0.143150172	4.31924965	-1.522212918	0.129189	0.16157	-6.18941895
DNAI1	-0.063831669	0.339093	-1.522037762	0.129233	0.16161	-6.1896831
C1QTNF	-0.010237265	0.03583225	-1.521232959	0.129434	0.16185	-6.19089643
KRT6B	0.108103675	0.21026451	1.520746738	0.129556	0.16199	-6.19162917
PLA2G12	-0.301548499	2.05941412	-1.520413364	0.12964	0.16208	-6.19213142
OSBPL8	0.137210142	3.89750693	1.520239301	0.129684	0.16213	-6.19239362
ABL1	-0.123188866	6.06524858	-1.519954203	0.129756	0.1622	-6.19282302
ERLIN1	-0.135009036	4.95835626	-1.519859106	0.129779	0.16222	-6.19296623
SLC45A2	-0.08112484	0.22421575	-1.519810277	0.129792	0.16223	-6.19303976
TBPL2	-0.009975146	0.03263868	-1.519756476	0.129805	0.16223	-6.19312077
VWA7	-0.175417011	3.04605753	-1.519336939	0.129911	0.16235	-6.19375242
SNX1	-0.155388957	6.99612749	-1.518230102	0.130189	0.16269	-6.19541803
C5orf38	-0.329822714	2.42951486	-1.517798712	0.130298	0.1628	-6.19606689
VRK2	-0.105359871	3.61897665	-1.517796263	0.130299	0.1628	-6.19607057
TCL1A	0.185232911	0.47827096	1.516302865	0.130676	0.16326	-6.19831538
ATP9A	-0.146728446	5.63597083	-1.51498196	0.13101	0.16366	-6.2002991
ELK3	0.152035264	5.64900048	1.513947586	0.131272	0.16398	-6.20185132
HSPA1B	-0.168429583	6.51738488	-1.513849992	0.131296	0.164	-6.20199772
PLSCR2	-0.038971533	0.39390846	-1.513615252	0.131356	0.16406	-6.20234981
FABP5	-0.169407792	2.73744357	-1.513285326	0.13144	0.16415	-6.20284459
HHIPL1	-0.080988629	0.8589537	-1.512935128	0.131529	0.16425	-6.20336965
WNT3	0.162405308	2.63042424	1.512139353	0.131731	0.16449	-6.20456233
NR1D2	-0.159797937	5.56388418	-1.511838191	0.131807	0.16457	-6.20501354
FAM78A	0.134264468	1.83309974	1.51176823	0.131825	0.16458	-6.20511834
SPTB	0.057300374	0.40845652	1.511679855	0.131848	0.1646	-6.20525073
RLN2	-0.09547838	1.01973013	-1.511520615	0.131888	0.16464	-6.20548924
C1orf146	-0.048513066	0.31835584	-1.511207616	0.131968	0.16473	-6.205958
PLA2G10	0.129469859	0.53884451	1.511170574	0.131977	0.16473	-6.20601347
TTC7A	-0.110766548	4.9777361	-1.511103844	0.131994	0.16474	-6.20611339
FAM135I	-0.029365966	0.03122201	-1.510795047	0.132073	0.16482	-6.20657573
SERPINE	-0.114803692	6.37414752	-1.510499574	0.132148	0.1649	-6.20701803
NAIP	-0.061009029	0.30073044	-1.509948342	0.132289	0.16507	-6.20784295
STARD6	-0.013956421	0.06134056	-1.509886366	0.132305	0.16507	-6.20793568
FLRT2	-0.101193418	0.92468197	-1.509813484	0.132323	0.16509	-6.20804472
FGF14	0.049014637	0.2098233	1.509619071	0.132373	0.16513	-6.20833557
PRSS3	0.068256805	0.39288454	1.509483119	0.132408	0.16517	-6.20853893
TMEM44	-0.118884754	3.30454011	-1.509438137	0.132419	0.16517	-6.20860622
CYP3A43	0.012715846	0.04059196	1.507969709	0.132794	0.16562	-6.21080157

CHRD	-0.096234763	1.16599221	-1.507857007	0.132823	0.16565	-6.21096997
KREMEN	0.06960367	0.54213257	1.507624201	0.132883	0.16571	-6.21131781
F2RL1	0.186180326	4.29282962	1.507394161	0.132942	0.16577	-6.21166146
CASD1	-0.124131423	4.52224867	-1.50733678	0.132956	0.16578	-6.21174717
GRAMD1	0.0958248	5.24840835	1.506415843	0.133192	0.16606	-6.21312236
CIP2A	-0.079974634	1.34117812	-1.506192273	0.13325	0.16612	-6.21345608
CYP24A1	0.352161565	2.1217499	1.506052218	0.133286	0.16615	-6.21366512
NKX6-1	-0.321098572	1.93300723	-1.505154355	0.133516	0.16642	-6.21500475
KLK3	0.20018111	0.5608832	1.505128398	0.133523	0.16642	-6.21504346
EIF2AK2	-0.121341421	4.03147215	-1.503599546	0.133916	0.1669	-6.21732269
HOXD3	0.02195376	0.14935217	1.503315265	0.133989	0.16698	-6.21774624
ZNF711	-0.136125251	2.49774819	-1.503135076	0.134036	0.16702	-6.21801467
MMP17	0.159299113	0.81837522	1.503099536	0.134045	0.16702	-6.21806761
DUSP9	0.045662175	0.08166049	1.502736599	0.134138	0.16713	-6.21860817
ZNF496	-0.09755607	3.87119372	-1.501081169	0.134566	0.16765	-6.22107214
SLIT1	-0.408093282	3.52248111	-1.501051478	0.134574	0.16765	-6.22111631
IL17RA	-0.091732832	3.34726708	-1.500979266	0.134592	0.16766	-6.22122373
AMER3	-0.018659239	0.04351878	-1.500485535	0.13472	0.1678	-6.22195804
PDZRN4	0.356398319	1.91517956	1.500231577	0.134786	0.16787	-6.22233566
NUP153	-0.129215635	4.180661	-1.499719009	0.134918	0.16803	-6.22309761
SPRY1	-0.201746723	6.1052357	-1.499218624	0.135048	0.16818	-6.2238412
KLHL18	-0.108254306	3.6509345	-1.498901638	0.13513	0.16827	-6.22431213
NFAT5	0.199098943	3.25667708	1.497909096	0.135388	0.16857	-6.22578606
MDFI	0.246637009	3.13957915	1.497804168	0.135415	0.1686	-6.22594182
ARRDC3	-0.170563798	7.08084876	-1.497735867	0.135433	0.16861	-6.22604321
UNC13A	0.046017089	0.22672087	1.497658953	0.135453	0.16862	-6.22615737
SLFN13	0.115621451	3.28546018	1.497617901	0.135463	0.16862	-6.2262183
ANKRD1	0.09773908	0.27640045	1.495633872	0.13598	0.16925	-6.22916116
VSIG8	-0.082665073	0.61773842	-1.495131966	0.136111	0.1694	-6.22990502
JAG2	-0.123379154	3.94300366	-1.49473326	0.136215	0.16952	-6.23049575
OTUD3	0.103126202	2.82675297	1.494333148	0.136319	0.16963	-6.23108841
PHF2	-0.124087733	5.19170776	-1.493450816	0.13655	0.16991	-6.2323948
CNN3	0.130418448	7.5621591	1.493188661	0.136618	0.16998	-6.23278281
TBX18	0.057450588	0.38463443	1.492341593	0.13684	0.17024	-6.23403606
JAKMIP1	-0.096512339	0.68600293	-1.492283847	0.136855	0.17025	-6.23412147
L3MBTL1	-0.112373047	1.79218657	-1.49149207	0.137062	0.1705	-6.23529223
ATG16L2	-0.134786105	2.96247719	-1.490852064	0.13723	0.17069	-6.23623814
CTSV	-0.103684923	1.17268344	-1.490788565	0.137247	0.1707	-6.23633197
MTSS2	0.104488439	3.18095136	1.490731668	0.137262	0.17071	-6.23641604
PTPDC1	0.116407157	2.17514681	1.490280654	0.13738	0.17084	-6.23708233

CLSTN2	-0.099991917	0.48191268	-1.489776236	0.137513	0.17099	-6.23782727
BAHCC1	-0.121156548	2.97280773	-1.489502509	0.137585	0.17107	-6.23823142
ZFP37	-0.117919058	1.9341348	-1.489424017	0.137606	0.17108	-6.2383473
AGO1	-0.089862659	3.20234835	-1.489402684	0.137611	0.17108	-6.23837879
DMP1	0.078819014	0.24396817	1.489335083	0.137629	0.17109	-6.23847859
SATB1	-0.139192811	3.94037938	-1.489049077	0.137704	0.17117	-6.23890074
TMCC1	-0.10664619	3.84601721	-1.48842597	0.137868	0.17136	-6.23982019
HDX	-0.073938565	1.46193469	-1.488016964	0.137976	0.17148	-6.24042351
DOC2B	-0.11229019	1.51502196	-1.487839993	0.138023	0.17153	-6.24068451
FAM110E	0.151430798	3.8498557	1.487700229	0.13806	0.17156	-6.24089061
UHRF1B	-0.118751113	3.22225015	-1.487477041	0.138118	0.17162	-6.2412197
PID1	-0.130950281	1.07622273	-1.487417516	0.138134	0.17163	-6.24130746
GBF1	-0.114765227	5.69844014	-1.486198483	0.138456	0.17202	-6.24310396
RIMS3	-0.114648715	1.43370173	-1.485185412	0.138724	0.17234	-6.24459583
ZMYM3	-0.115649168	4.79648416	-1.483632362	0.139136	0.17283	-6.24688095
PDLIM7	0.141310579	4.89580872	1.48326546	0.139233	0.17294	-6.24742046
SRRM4	-0.100004724	0.22092032	-1.482690909	0.139385	0.17312	-6.24826504
TMEM26	-0.056723114	0.74604788	-1.482142591	0.139531	0.17329	-6.24907075
IGFALS	-0.216743825	2.03666901	-1.481712658	0.139646	0.17342	-6.24970231
CALML4	-0.054616495	0.74087425	-1.48024806	0.140036	0.17389	-6.25185239
TXNDC2	0.012499099	0.06134839	1.479113699	0.140338	0.17425	-6.25351623
TUT4	-0.123467032	3.08234359	-1.478883695	0.1404	0.17431	-6.25385344
TEX38	-0.056990477	0.54198898	-1.478849494	0.140409	0.17431	-6.25390358
ADAMTS	0.012054568	0.03179866	1.478223369	0.140576	0.17451	-6.25482126
PARPBP	-0.059435841	0.91181044	-1.478132836	0.1406	0.17453	-6.25495392
KIR2DL4	-0.078566472	0.39986758	-1.478012456	0.140633	0.17455	-6.2551303
COL26A1	0.122387514	0.83191203	1.477463122	0.14078	0.17472	-6.255935
CGREF1	0.082960758	0.47943515	1.476810006	0.140955	0.17492	-6.25689135
AIFM3	-0.066055329	0.70676812	-1.476797728	0.140958	0.17492	-6.25690933
SLC36A1	0.085561714	2.08624559	1.476688869	0.140987	0.17494	-6.25706869
LRRK2	0.378436659	5.11028273	1.476627339	0.141004	0.17495	-6.25715876
SEC16B	-0.019983709	0.12797013	-1.475498019	0.141306	0.17531	-6.25881122
HOPX	-0.164501351	6.47812995	-1.474603827	0.141547	0.1756	-6.26011876
SEPTIN6	0.125733679	3.42078533	1.473500348	0.141844	0.17595	-6.26173126
SLC39A6	-0.162643102	5.92908901	-1.473255914	0.141909	0.17602	-6.26208828
ZNF280C	-0.087821107	1.82241868	-1.472650765	0.142073	0.17621	-6.26297193
AP3B2	-0.131886615	1.72792045	-1.472506723	0.142111	0.17625	-6.26318221
IGSF5	-0.06652212	0.33810934	-1.472036214	0.142238	0.17639	-6.26386894
HTR6	0.01147379	0.03584112	1.471888235	0.142278	0.17642	-6.26408487
SRGAP2	0.055252727	0.64213634	1.471870833	0.142283	0.17642	-6.26411027

SUFU	0.094939295	2.7212758	1.471521676	0.142377	0.17652	-6.26461968
WDR90	-0.108458213	3.14932567	-1.470244372	0.142722	0.17694	-6.26648224
RLIM	-0.108615644	4.46790311	-1.470072238	0.142769	0.17698	-6.26673312
KDSR	-0.092850587	4.49481546	-1.469994488	0.14279	0.177	-6.26684643
RYBP	-0.101881597	4.46830773	-1.469587461	0.1429	0.17712	-6.26743952
HGFAC	0.051809979	0.19647134	1.469329702	0.14297	0.17719	-6.26781503
LCN6	0.339467149	2.11156494	1.469310103	0.142975	0.17719	-6.26784358
ZNF579	-0.1105789	4.14449621	-1.469013416	0.143056	0.17728	-6.26827571
FABP1	-0.031158128	0.09005816	-1.46884889	0.1431	0.17732	-6.2685153
BCL11B	-0.131692602	1.07382153	-1.468256013	0.143261	0.1775	-6.26937849
TRIB1	-0.137516973	7.83820919	-1.467840282	0.143374	0.17763	-6.26998355
FAM83D	-0.102268423	1.0523162	-1.467641046	0.143428	0.17769	-6.27027347
APOBEC	0.152386075	3.11782708	1.466208411	0.143818	0.17815	-6.272357
FAM76B	-0.11176602	2.92353647	-1.466070412	0.143855	0.17819	-6.27255759
FAM187E	-0.027834676	0.13349594	-1.464805347	0.1442	0.1786	-6.27439559
NOTCH2	0.03591332	0.25959142	1.464440805	0.144299	0.1787	-6.27492493
NOTCH2	0.03591332	0.25959142	1.464440805	0.144299	0.1787	-6.27492493
OPN4	-0.075852686	0.33157019	-1.463520577	0.144551	0.179	-6.27626061
CHRNA2	-0.057914336	0.08096688	-1.462378868	0.144863	0.17937	-6.27791662
PON3	-0.180026007	1.87408816	-1.462080154	0.144945	0.17946	-6.27834968
CNKSR3	-0.076491508	0.97142989	-1.461813308	0.145018	0.17954	-6.27873647
PIAS3	0.140039487	4.24584124	1.461306011	0.145157	0.1797	-6.27947159
ESYT1	0.119885086	6.10108372	1.460921391	0.145262	0.17981	-6.28002878
HEATR5I	-0.117405426	3.78381117	-1.460377071	0.145412	0.17998	-6.28081707
RFX7	-0.102886006	2.76721806	-1.460290534	0.145435	0.18	-6.28094237
ZNF367	-0.128766881	2.19201795	-1.46017431	0.145467	0.18002	-6.28111064
RASSF8	-0.122399214	3.04212732	-1.460163959	0.14547	0.18002	-6.28112563
PTPN14	0.140022404	2.92860408	1.458531978	0.145919	0.18056	-6.28348702
SINHCAF	0.133299809	4.23052488	1.458298582	0.145983	0.18063	-6.28382452
IQCN	-0.16941447	2.33755962	-1.458176445	0.146017	0.18065	-6.28400111
ZNF667	-0.109756044	2.71790533	-1.456735033	0.146414	0.18113	-6.2860841
WDR27	-0.128852657	2.21282265	-1.456145941	0.146577	0.18132	-6.28693482
TSC22D4	-0.091916676	5.656695	-1.455485846	0.146759	0.18153	-6.28788767
ARHGAP	-0.112601103	4.74221318	-1.455284886	0.146814	0.18159	-6.28817767
TMEM63	0.133236769	5.0914005	1.4549577	0.146905	0.18169	-6.28864974
RORB	0.053733452	0.27503561	1.454304188	0.147086	0.1819	-6.28959233
CARS2	-0.069364626	3.65314907	-1.45410424	0.147141	0.18195	-6.28988065
RAB32	0.139537767	4.72233463	1.452719684	0.147525	0.18241	-6.29187601
TNR	0.016623122	0.05212055	1.452659939	0.147542	0.18242	-6.29196207
ANXA8	0.082061856	0.17785474	1.452238192	0.147659	0.18255	-6.29256948

RPA4	0.017643549	0.05293461	1.450449798	0.148156	0.18315	-6.29514323
CAMSAF	-0.109230991	4.56923677	-1.449775291	0.148344	0.18337	-6.29611313
GPATCH	-0.087975539	2.15994775	-1.449508592	0.148418	0.18345	-6.29649651
GLP2R	-0.034606523	0.07833773	-1.449299363	0.148477	0.18351	-6.29679722
TMEM13	-0.086034918	0.5887542	-1.448852835	0.148601	0.18365	-6.29743885
USP15	-0.100799414	2.87873393	-1.448289503	0.148759	0.18383	-6.29824805
DCK	-0.124363804	4.23903994	-1.447825106	0.148888	0.18398	-6.29891489
HIF1A	0.158478022	6.776774	1.446755871	0.149188	0.18434	-6.30044944
PDZK1	-0.064580138	0.41955977	-1.446259694	0.149327	0.18449	-6.30116117
SEL1L2	-0.018498028	0.03647453	-1.446195655	0.149344	0.1845	-6.30125301
FAM78B	-0.052427089	0.82082619	-1.445836306	0.149445	0.18461	-6.3017683
SUN1	-0.109887068	4.82292748	-1.445682364	0.149488	0.18465	-6.30198901
CMYA5	0.159190694	2.89577458	1.445314741	0.149591	0.18477	-6.30251597
ANGPTL	0.062099359	0.11774557	1.444238688	0.149894	0.18513	-6.30405767
TEN1	-0.028844479	0.29087788	-1.443329704	0.150149	0.18543	-6.30535913
HSPG2	-0.223157775	5.32474283	-1.442024075	0.150517	0.18587	-6.30722707
KCNK7	0.085616643	0.69381545	1.440848828	0.150848	0.18627	-6.30890706
STON1	-0.117215591	1.64318594	-1.440567132	0.150928	0.18635	-6.30930954
ENTPD4	-0.09977383	4.12187213	-1.439959633	0.1511	0.18655	-6.31017725
ZNF224	-0.088978911	2.56269345	-1.439338305	0.151275	0.18675	-6.31106434
PCDHGE	0.193043336	2.42584406	1.439096482	0.151344	0.18682	-6.3114095
GNB3	0.131795589	1.45232978	1.438766494	0.151437	0.18692	-6.3118804
TMEM11	0.036290845	0.11522086	1.438735036	0.151446	0.18692	-6.31192529
CNKSR1	-0.102812491	4.92571277	-1.438591564	0.151487	0.18696	-6.31212999
ACOT12	0.072243932	0.24285312	1.438502325	0.151512	0.18698	-6.31225731
POM121(	-0.094254378	3.73071861	-1.437349167	0.151839	0.18737	-6.31390177
MACF1	0.142403198	4.69331237	1.437254739	0.151866	0.18737	-6.31403637
CASP10	-0.115134306	3.21714637	-1.437247527	0.151868	0.18737	-6.31404665
ZNF358	-0.108974093	6.60604291	-1.436809912	0.151992	0.18751	-6.31467033
TOR1AIF	-0.094522305	3.64889437	-1.436453941	0.152093	0.18762	-6.31517751
FZD1	-0.176924265	6.40746791	-1.436431509	0.152099	0.18762	-6.31520947
PLXNB2	0.149534203	8.01322874	1.435617249	0.152331	0.18789	-6.31636914
KIAA031	0.07318409	0.2827295	1.435529866	0.152356	0.18791	-6.31649355
GPSM1	0.135777511	3.48972881	1.435008753	0.152504	0.18808	-6.31723533
C20orf20	-0.065504887	0.4181216	-1.434702075	0.152591	0.18817	-6.31767175
RNF17	-0.048339777	0.07202584	-1.4344981	0.15265	0.18823	-6.31796197
OGT	0.147776919	5.65756919	1.432750175	0.153148	0.18883	-6.32044726
SMC4	0.147393309	2.87249789	1.431998176	0.153363	0.18908	-6.32151558
TDRD10	0.151398525	2.86346152	1.431808518	0.153417	0.18913	-6.32178492
PYDC1	0.093779284	0.37396054	1.431456227	0.153518	0.18924	-6.32228515

HOXA2	-0.046381086	0.39711713	-1.431356878	0.153547	0.18927	-6.32242619
ZNF114	0.136754629	3.2753158	1.431315934	0.153558	0.18927	-6.32248432
ACTN3	0.015416165	0.05871533	1.431169477	0.1536	0.1893	-6.32269222
NTRK3	-0.138715634	0.88988066	-1.430909538	0.153675	0.18938	-6.32306116
KCNJ15	-0.167695742	6.29287003	-1.430636655	0.153753	0.18946	-6.3234484
CSNK2A	-0.071391767	5.01054157	-1.430604072	0.153762	0.18946	-6.32349463
FAM72A	0.02165404	0.17672372	1.429222123	0.154158	0.18994	-6.32545455
IL5RA	-0.03063154	0.12105105	-1.42884212	0.154268	0.19006	-6.32599316
OSBPL1(	0.137752775	2.21440167	1.428557822	0.154349	0.19015	-6.32639602
DGKZ	-0.079921639	3.70140506	-1.428363237	0.154405	0.1902	-6.32667171
PPP1R1(	-0.148689737	2.30707379	-1.428249918	0.154438	0.19023	-6.32683225
FUCA1	-0.117144217	7.9399963	-1.426920057	0.15482	0.19068	-6.32871528
FAM3C	-0.133905252	6.26029446	-1.426894392	0.154828	0.19068	-6.3287516
FLNB	-0.103665833	6.14116654	-1.426791308	0.154857	0.1907	-6.32889749
POLA1	-0.092664806	2.80782679	-1.426159208	0.15504	0.19091	-6.32979183
VASP	0.10152909	6.18452962	1.42519532	0.155318	0.19124	-6.33115487
SHC4	-0.113171432	0.75164751	-1.424978758	0.15538	0.1913	-6.33146098
MAD2L1	-0.08211778	1.57266934	-1.42482166	0.155426	0.19135	-6.33168301
C1orf167	-0.010044314	0.0502408	-1.42477019	0.155441	0.19135	-6.33175575
CD244	0.089247045	0.71447931	1.424368524	0.155557	0.19148	-6.33232331
PAX2	-0.088356063	0.10659995	-1.424022492	0.155657	0.19159	-6.33281214
GNB5	-0.101508245	3.57288701	-1.423853402	0.155706	0.19162	-6.33305096
PLRG1	0.107383327	5.44600413	1.423843006	0.155709	0.19162	-6.33306564
SRPK1	-0.097027263	3.67059154	-1.423518755	0.155802	0.19173	-6.33352353
UBE2F-S	-0.01283817	0.08193398	-1.423439168	0.155825	0.19174	-6.3336359
RSPH10I	-0.00833374	0.02419884	-1.423363949	0.155847	0.19175	-6.3337421
NUDT10	0.10076099	0.66911601	1.423231445	0.155886	0.19179	-6.33392917
ZBED6	0.181872343	1.68455929	1.422926324	0.155974	0.19188	-6.33435987
KIF3C	-0.142579751	2.51462364	-1.422851781	0.155995	0.19189	-6.33446507
NIBAN3	0.077157702	0.27488524	1.422599771	0.156069	0.19197	-6.33482072
SHCBP1	-0.010725065	0.03181979	-1.420712679	0.156616	0.19263	-6.33748184
SLITRK2	-0.06708212	0.1510564	-1.420476408	0.156685	0.1927	-6.33781478
LGALS9(	0.019280178	0.04074943	1.420419053	0.156701	0.19271	-6.33789559
FKBP6	-0.014925524	0.05407952	-1.420056274	0.156807	0.19282	-6.33840667
LINGO3	0.051937136	0.30308389	1.41956871	0.156949	0.19298	-6.33909334
ITIH4	-0.033681938	0.24643817	-1.419148506	0.157071	0.19312	-6.33968496
MYMK	-0.064215823	0.14660958	-1.417447343	0.157567	0.19372	-6.34207831
ZNF528	-0.108215762	3.10982177	-1.416575633	0.157822	0.19401	-6.34330362
ZNF724	0.079794191	0.74914838	1.41630497	0.157901	0.19409	-6.34368392
TMCO6	-0.089808154	3.41088468	-1.416295428	0.157904	0.19409	-6.34369733

AVIL	0.116759652	1.94928901	1.414975866	0.15829	0.19455	-6.34555038
BCL9	0.147795237	4.73427295	1.413587768	0.158697	0.19503	-6.34749784
TDP1	0.077366992	2.12309735	1.413558049	0.158705	0.19503	-6.34753951
WSB1	0.171551478	5.06692792	1.413511482	0.158719	0.19503	-6.34760481
MIP	-0.016314224	0.08698972	-1.412615742	0.158982	0.19534	-6.34886044
SH3PXD:	0.132205867	3.75511194	1.412590899	0.15899	0.19534	-6.34889526
CD46	-0.116439549	6.00621163	-1.411735852	0.159241	0.19563	-6.35009309
RBM44	-0.026773279	0.2074168	-1.411433964	0.15933	0.19573	-6.35051584
BMP4	0.228963725	2.84439955	1.409901382	0.159782	0.19627	-6.3526606
GJB7	-0.017419724	0.04741873	-1.409853133	0.159796	0.19627	-6.35272808
HS3ST5	-0.014278805	0.04001788	-1.409761606	0.159823	0.19629	-6.35285609
CALHM1	0.03419461	0.10215103	1.409435098	0.159919	0.19639	-6.35331268
GIPC1	-0.093333888	6.45653288	-1.408823589	0.1601	0.1966	-6.35416753
RIMBP3(	-0.020157985	0.12162735	-1.4087504	0.160121	0.19661	-6.35426982
ZNF266	-0.105030588	4.0676897	-1.408402625	0.160224	0.19673	-6.3547558
AKNAD1	-0.023778405	0.04005228	-1.407831112	0.160393	0.19692	-6.35555418
MAPK8IF	-0.083330847	0.88088636	-1.406150404	0.160891	0.19752	-6.35790019
ZNF264	-0.097511949	3.3758743	-1.406003468	0.160935	0.19755	-6.35810516
ZNF519	-0.046375069	0.66425086	-1.405606801	0.161052	0.19768	-6.35865839
LRRC10I	0.1136102	1.0339347	1.40445158	0.161395	0.19809	-6.36026869
LCMT1	-0.080757359	5.64466403	-1.40432678	0.161433	0.19812	-6.36044258
KANSL1I	-0.117923422	3.15394971	-1.404147427	0.161486	0.19817	-6.36069245
C3orf20	-0.020104248	0.12766529	-1.403424979	0.161701	0.19842	-6.36169861
PRDM1	0.203601732	5.30928374	1.402949259	0.161843	0.19858	-6.36236088
KIRREL3	-0.07384333	0.13367475	-1.402463745	0.161987	0.19875	-6.36303655
NPIPA5	-0.149774236	1.66894684	-1.402197223	0.162067	0.19883	-6.36340736
NDP	-0.12168928	0.60353522	-1.402096111	0.162097	0.19885	-6.36354802
C10orf10	-0.016709858	0.07421128	-1.401937277	0.162144	0.1989	-6.36376896
KIR2DL1	0.028665535	0.10600933	1.401700344	0.162215	0.19897	-6.36409848
HIVEP2	0.11581415	3.77436887	1.401511435	0.162271	0.19902	-6.36436118
DENND6	-0.110222878	3.60072254	-1.401005589	0.162422	0.19919	-6.36506443
SMIM29	0.098571954	5.74794001	1.400956019	0.162437	0.1992	-6.36513333
NPBWR1	0.052164189	0.13794153	1.400669307	0.162523	0.19929	-6.36553181
E2F2	0.080498615	0.89735755	1.399283957	0.162937	0.19976	-6.36745606
TEAD2	-0.140966327	3.85962218	-1.399251453	0.162947	0.19976	-6.36750118
ABCA13	-0.13517366	0.89775288	-1.399250934	0.162947	0.19976	-6.3675019
KLF14	-0.012629567	0.03463394	-1.399215116	0.162958	0.19976	-6.36755163
SGO2	-0.063720127	1.14235213	-1.397344929	0.163519	0.20042	-6.37014621
CKLF	0.133889537	4.57001894	1.397340486	0.16352	0.20042	-6.37015237
TAC1	-0.070441888	0.1780732	-1.397045353	0.163609	0.20052	-6.37056151

SCRIB	0.096980146	5.34962085	1.396981652	0.163628	0.20053	-6.37064981
PPRC1	0.102943832	4.41580759	1.396808274	0.16368	0.20058	-6.37089011
HSFX1	-0.015300747	0.07362307	-1.396199536	0.163863	0.20079	-6.37173358
KLHL33	-0.025131754	0.1309438	-1.395991338	0.163925	0.20085	-6.37202197
CCDC18	0.040432919	0.1120799	1.395949382	0.163938	0.20085	-6.37208009
FBXO28	-0.102633955	4.2644194	-1.395757608	0.163996	0.20091	-6.37234569
STPG4	0.011430431	0.05608417	1.395669023	0.164022	0.20092	-6.37246836
KRT36	0.021774295	0.0574597	1.39530974	0.164131	0.20104	-6.37296583
STARD9	0.077793368	1.14626587	1.394805506	0.164282	0.20121	-6.37366379
CYP4F3	-0.090425015	0.10259322	-1.393773145	0.164594	0.20158	-6.375092
EXOSC1	-0.071438379	4.78145865	-1.393575448	0.164653	0.20164	-6.37536538
GAL3ST4	0.15538271	2.10738282	1.392964231	0.164838	0.20185	-6.37621035
AMY2B	0.145952492	2.34365438	1.39235413	0.165022	0.20206	-6.37705342
PELI3	0.082347128	3.39373929	1.392123388	0.165092	0.20213	-6.37737218
KAT2A	-0.120729681	5.21840719	-1.392002435	0.165129	0.20216	-6.37753924
DCP2	-0.079957589	3.10414914	-1.391255769	0.165355	0.20241	-6.37857027
SEC24A	-0.122995864	3.94979367	-1.391249887	0.165356	0.20241	-6.37857839
ZDHHC2	-0.088097588	0.15208143	-1.390817883	0.165487	0.20256	-6.37917467
KCNK12	-0.026208269	0.15101866	-1.390298561	0.165645	0.20273	-6.37989122
WNT5A	0.191806981	3.29870346	1.389974878	0.165743	0.20284	-6.38033771
AXIN1	-0.080650396	4.44200152	-1.389863781	0.165777	0.20287	-6.38049093
CXCR5	0.031547371	0.07371974	1.389326149	0.16594	0.20305	-6.38123224
PROKR1	-0.031184017	0.0332966	-1.389105639	0.166007	0.20312	-6.38153621
MYZAP	-0.07915054	0.89166348	-1.388740587	0.166118	0.20324	-6.38203933
BGN	0.19636807	8.14320388	1.387297753	0.166556	0.20376	-6.38402656
INTS1	-0.081797606	5.35073326	-1.387058688	0.166629	0.20384	-6.38435563
NUP214	-0.080430136	3.92349299	-1.386008149	0.166949	0.20421	-6.38580103
HS3ST4	0.067393704	0.15780899	1.385061557	0.167238	0.20455	-6.38710248
CBFB	0.105654328	5.22476635	1.384996749	0.167258	0.20456	-6.38719155
VASH1	-0.119897614	3.4027679	-1.384933636	0.167277	0.20457	-6.38727829
PABPC4	0.089161002	5.95441144	1.384846916	0.167304	0.20459	-6.38739746
THEG	0.084035192	0.1951886	1.384198405	0.167502	0.20482	-6.38828845
RHBDL1	-0.097083318	1.21892887	-1.383988883	0.167566	0.20488	-6.38857622
CRX	0.009614165	0.02825744	1.383691335	0.167657	0.20498	-6.38898483
DDX43	-0.164505187	0.80096119	-1.383457351	0.167729	0.20505	-6.38930608
S100PBF	-0.08818237	3.12853356	-1.383325093	0.167769	0.20508	-6.38948764
ARHGEF	0.117422705	5.27664357	1.382721394	0.167954	0.20529	-6.39031617
HS6ST2	-0.336850292	2.06953515	-1.382561009	0.168003	0.20534	-6.39053623
CTSD	0.112922856	9.5447951	1.382180234	0.16812	0.20547	-6.39105858
ARID5A	0.13643672	3.81237325	1.381770689	0.168246	0.2056	-6.39162023

NAALAD	0.079936436	1.33982126	1.381752324	0.168251	0.2056	-6.39164541
NKAIN1	-0.10009399	0.17447343	-1.381189974	0.168424	0.20579	-6.39241634
GPLD1	-0.118484681	1.42727924	-1.380641799	0.168592	0.20599	-6.39316754
P2RX6	0.036299841	0.10951621	1.380496824	0.168637	0.20603	-6.39336616
BSN	0.051344731	0.55342278	1.380000547	0.168789	0.2062	-6.39404592
CPEB2	-0.111951309	3.08502179	-1.379800371	0.168851	0.20626	-6.39432004
IL1R1	0.148456551	4.99749949	1.379583674	0.168918	0.20632	-6.39461673
SIDT1	-0.115228614	0.76765764	-1.379307171	0.169003	0.20641	-6.39499525
SMIM10L	-0.247947745	4.87059137	-1.37909026	0.16907	0.20648	-6.39529213
TENM1	0.313783706	3.61350087	1.378794171	0.169161	0.20658	-6.39569731
GASK1A	-0.108268511	0.98394307	-1.378721562	0.169183	0.20659	-6.39579666
DSP	0.191100309	7.04867042	1.37799027	0.169409	0.20685	-6.39679697
PPP1R1!	-0.167640267	6.24393314	-1.377855063	0.16945	0.20688	-6.39698186
ABHD2	-0.161924761	4.20719611	-1.377592714	0.169531	0.20697	-6.39734055
LOXL4	-0.23658211	3.71225309	-1.377351333	0.169606	0.20704	-6.39767052
CEP76	-0.06822325	1.8833002	-1.377293328	0.169624	0.20705	-6.39774981
APLP2	-0.15579344	10.0908345	-1.376382122	0.169905	0.20737	-6.39899486
SLC7A1	0.154085158	3.52991502	1.376379245	0.169906	0.20737	-6.39899879
NCKAP1	-0.122463349	4.86100947	-1.376185659	0.169966	0.20742	-6.3992632
FXYD4	-0.044438493	0.13184564	-1.375914463	0.17005	0.20751	-6.39963355
ZNF676	-0.149964763	2.21915775	-1.37575606	0.170099	0.20756	-6.39984983
KLK13	0.165129662	1.6055982	1.375202242	0.17027	0.20775	-6.40060582
OR5K2	-0.024571896	0.13026876	-1.375040397	0.17032	0.2078	-6.40082669
RRM2	0.140917781	1.66797477	1.374132821	0.170601	0.20813	-6.40206479
ST6GALI	0.060939455	0.55504421	1.373991049	0.170645	0.20816	-6.40225812
INTU	-0.102792852	2.15154551	-1.373250539	0.170875	0.20843	-6.40326761
VAMP4	-0.102458805	4.28860416	-1.37293713	0.170972	0.20853	-6.4036947
GRM8	-0.050027636	0.36418484	-1.372813053	0.171011	0.20856	-6.40386375
MRAS	-0.101069535	4.51243127	-1.372794654	0.171017	0.20856	-6.40388882
NKX6-3	-0.032325098	0.06798068	-1.372594325	0.171079	0.20862	-6.40416174
CDC6	-0.104046175	1.65459633	-1.372493766	0.17111	0.20864	-6.40429872
UBE2L6	0.130619267	6.23073762	1.372206052	0.171199	0.20874	-6.40469058
PBK	-0.119269443	1.19175738	-1.371892593	0.171297	0.20884	-6.40511742
IRF3	-0.104359073	5.78959751	-1.37178302	0.171331	0.20887	-6.4052666
MAP4K4	0.10598281	4.76328543	1.371679086	0.171363	0.20889	-6.40540809
RPRD1A	-0.109289697	4.83604597	-1.370725884	0.17166	0.20924	-6.40670528
KIF5B	-0.100785127	6.31058712	-1.370493534	0.171732	0.20931	-6.40702134
REV3L	-0.120308489	2.9387659	-1.369543275	0.172029	0.20966	-6.40831342
CYP2A7	0.011064334	0.04283101	1.369385078	0.172078	0.2097	-6.40852844
CXCL12	-0.203099329	4.18171815	-1.368656054	0.172306	0.20996	-6.40951899

PRR16	-0.080773054	1.06221697	-1.367211927	0.172757	0.2105	-6.41147965
ENTPD1	-0.195382973	5.44022531	-1.366717065	0.172912	0.21067	-6.41215104
BBX	-0.123365847	4.41364394	-1.366520656	0.172974	0.21073	-6.41241745
CHST3	-0.123623777	3.47980373	-1.366351925	0.173026	0.21078	-6.41264628
RAB31	0.144303448	5.23025975	1.365258763	0.173369	0.21118	-6.41412816
ACP7	0.022146006	0.06697361	1.365170253	0.173397	0.2112	-6.4142481
TBC1D2:	-0.097395917	4.31808088	-1.364981589	0.173456	0.21125	-6.41450371
BST1	-0.090783345	1.66268003	-1.364971877	0.173459	0.21125	-6.41451687
MYOZ2	0.052571398	0.09363679	1.364932855	0.173472	0.21125	-6.41456974
GRM6	-0.037732458	0.30649774	-1.364493417	0.17361	0.2114	-6.41516497
ABCC5	-0.084338944	1.88426696	-1.363958912	0.173777	0.21159	-6.41588871
ZNF384	-0.118877561	3.75713529	-1.363742407	0.173846	0.21166	-6.41618179
OPCML	0.208359547	0.76225492	1.363571302	0.173899	0.21171	-6.41641338
DHX32	-0.086014796	4.88513544	-1.363149078	0.174032	0.21185	-6.41698473
ERFE	-0.311713406	4.48659095	-1.362736581	0.174162	0.212	-6.41754276
MYO5A	0.109262485	3.20387476	1.36203131	0.174384	0.21225	-6.41849645
PRR26	-0.01207009	0.06438816	-1.361485219	0.174556	0.21245	-6.41923457
CTXND1	-0.305110792	3.11047916	-1.361299014	0.174615	0.2125	-6.41948618
EDAR	-0.067227858	0.14469628	-1.361193585	0.174648	0.21253	-6.41962863
RAD21L1	-0.01677381	0.06354191	-1.360698308	0.174805	0.2127	-6.42029767
CCDC89	-0.0874292	0.5020528	-1.360652265	0.174819	0.21271	-6.42035986
NAV1	0.131716422	3.01368394	1.360453517	0.174882	0.21277	-6.42062826
SLC7A7	0.138925396	2.87328115	1.359597531	0.175153	0.21308	-6.42178379
CDR1	0.011414844	0.04257292	1.359390744	0.175218	0.21314	-6.42206283
NRAP	0.080163366	0.05840488	1.358400811	0.175531	0.2135	-6.42339808
GDNF	0.046066238	0.14034796	1.358390842	0.175534	0.2135	-6.42341153
ATP6V1E	0.098032906	5.58922015	1.358097589	0.175627	0.2136	-6.42380689
LTBP3	0.159424132	6.70689269	1.357289494	0.175884	0.21389	-6.42489592
LAMP3	0.257073531	3.26890097	1.356970631	0.175985	0.214	-6.42532547
RAD51AI	-0.01688364	0.04926394	-1.356563508	0.176114	0.21414	-6.42587376
PLK5	-0.039958962	0.0587794	-1.356157427	0.176243	0.21428	-6.42642049
GH1	-0.036844264	0.13207824	-1.355915673	0.17632	0.21436	-6.4267459
SLC16A8	-0.087301909	0.96420082	-1.355293968	0.176517	0.21459	-6.42758247
SPDYE4	-0.018741106	0.09065442	-1.3542978	0.176835	0.21496	-6.42892214
HIST3H2	0.082946	0.50326976	1.35339688	0.177122	0.21529	-6.43013288
OVGP1	-0.071350359	1.07177334	-1.352936844	0.177268	0.21545	-6.43075081
PTAR1	-0.106210225	3.16813304	-1.352793139	0.177314	0.21549	-6.4309438
SERPIN(	-0.046387888	0.27926464	-1.351784659	0.177636	0.21587	-6.43229755
PF4	-0.071840522	0.3333796	-1.351423672	0.177752	0.21599	-6.43278189
FAM9B	0.007811312	0.01366081	1.350850446	0.177935	0.2162	-6.43355073

LRRK1	0.107437294	1.91352423	1.350047424	0.178192	0.2165	-6.43462723
WNT2B	-0.066355811	0.59308181	-1.349814216	0.178267	0.21657	-6.43493974
TNRC18	-0.108434852	5.00375789	-1.349579878	0.178342	0.21665	-6.43525372
GDAP1L:	-0.057621263	0.22320566	-1.34933979	0.178419	0.21673	-6.43557534
VIPR2	-0.041077654	0.10858801	-1.349184359	0.178469	0.21677	-6.43578352
IGSF10	-0.056203087	0.25903806	-1.348809406	0.178589	0.2169	-6.43628564
PPFIA2	0.018072791	0.12705709	1.348625419	0.178648	0.21696	-6.43653198
C8orf89	-0.079598803	0.64479839	-1.348211464	0.178781	0.2171	-6.43708609
MGAM	0.014056538	0.11112192	1.347699447	0.178945	0.21729	-6.43777123
ZNF460	0.090115367	1.11105673	1.347502043	0.179009	0.21735	-6.43803532
DNASE2	0.066764012	0.61710582	1.347423936	0.179034	0.21737	-6.4381398
IL15	-0.107071851	2.65736423	-1.347233915	0.179095	0.21742	-6.43839395
CXCR1	-0.052334846	0.21130015	-1.346970992	0.17918	0.21751	-6.43874556
NPFFR1	0.10833552	0.66234128	1.346862341	0.179215	0.21754	-6.43889084
NCOR1	-0.099682856	4.64918715	-1.34643601	0.179352	0.21769	-6.43946078
CDH19	0.040453808	0.10060249	1.345089897	0.179786	0.2182	-6.44125916
GLUD1	-0.09714663	6.91837512	-1.344424248	0.18	0.21844	-6.4421478
SAMD1	0.083923589	5.85326184	1.342075461	0.18076	0.21935	-6.44527996
PXT1	-0.01411894	0.10496025	-1.34178682	0.180853	0.21945	-6.4456645
MAP3K7	-0.090834922	4.4398891	-1.341717875	0.180875	0.21946	-6.44575634
ALDOB	0.086346562	0.27046038	1.341389499	0.180982	0.21957	-6.44619369
KIAA004(	-0.093731336	4.49569976	-1.34132929	0.181001	0.21958	-6.44627387
NEIL1	-0.122365969	2.82283956	-1.340861565	0.181153	0.21975	-6.4468966
LMNA	0.12901771	7.7693589	1.340694477	0.181207	0.21978	-6.44711902
KCNA7	0.016124068	0.03344495	1.340693492	0.181207	0.21978	-6.44712033
MCOLN1	-0.065076774	3.92230542	-1.340349616	0.181319	0.2199	-6.44757798
LTN1	-0.111570563	3.39164555	-1.340254298	0.18135	0.21992	-6.44770481
MIER1	-0.092099039	3.90845889	-1.340240198	0.181355	0.21992	-6.44772357
USP37	0.125985269	1.48356033	1.339312621	0.181656	0.22026	-6.44895736
CHKB	-0.120972864	2.5694285	-1.339065898	0.181736	0.22035	-6.44928539
FSTL1	0.137152355	5.40401623	1.338243981	0.182003	0.22065	-6.45037774
HOXB9	-0.03239736	0.06784817	-1.338124508	0.182042	0.22069	-6.45053647
CALCR	0.026408272	0.13092754	1.338068667	0.18206	0.22069	-6.45061065
ANXA10	0.013882767	0.05921194	1.337379022	0.182285	0.22095	-6.45152658
SRD5A1	-0.069356446	1.95915201	-1.337119298	0.18237	0.22104	-6.4518714
FAM221A	-0.08625093	1.85171006	-1.336896777	0.182442	0.22111	-6.45216678
LPAR6	-0.097302768	4.63317182	-1.336650305	0.182522	0.22119	-6.45249389
STX11	-0.173184613	3.21335996	-1.336516963	0.182566	0.22123	-6.45267084
NUP107	-0.082987845	3.22587304	-1.336457901	0.182585	0.22123	-6.45274921
CD5L	-0.077172079	0.19109489	-1.336021597	0.182728	0.22139	-6.45332803

SULT1A3	-0.013531729	0.07390432	-1.335491046	0.182901	0.22158	-6.45403164
CEP120	-0.092503528	3.63880163	-1.335288688	0.182967	0.22165	-6.45429993
HTR1F	-0.057552312	0.53371314	-1.333963605	0.1834	0.22216	-6.45605578
ATXN1	-0.098450764	3.21377426	-1.333836818	0.183442	0.22219	-6.45622369
PRR32	-0.0952622	0.40146585	-1.332688872	0.183818	0.22263	-6.45774328
BEST2	-0.009535854	0.03373499	-1.331165594	0.184318	0.22322	-6.45975772
PRRG1	0.144166772	3.5940222	1.330876623	0.184413	0.22332	-6.46013961
PLCH2	0.169149196	1.63673527	1.330651583	0.184487	0.22339	-6.46043695
PLSCR1	0.123224405	4.42365848	1.330235165	0.184624	0.22354	-6.46098704
MEGF10	0.060791837	0.20641809	1.329899145	0.184734	0.22366	-6.46143079
PPFIBP1	-0.118888702	4.23657793	-1.329828002	0.184758	0.22367	-6.46152473
SLC5A9	-0.059386197	0.46861733	-1.329159995	0.184978	0.22392	-6.46240653
WDR97	0.07663194	0.81147279	1.328735051	0.185118	0.22407	-6.46296726
HAVCR1	0.016955164	0.05655322	1.328706652	0.185127	0.22407	-6.46300472
APOBEC	-0.099543909	0.82499978	-1.328416517	0.185223	0.22417	-6.46338745
MCF2	-0.054781782	0.30055534	-1.327831996	0.185415	0.22439	-6.46415827
ALDOC	0.198120929	2.41603145	1.327295314	0.185592	0.22459	-6.4648657
PCDHGC	0.07671585	0.40112214	1.32544905	0.186203	0.22531	-6.46729723
OR13J1	0.020679275	0.09245912	1.324879796	0.186391	0.22552	-6.46804627
RNF169	0.134273242	2.35698162	1.324826029	0.186409	0.22553	-6.468117
DAB2IP	0.134736804	5.47273173	1.324226739	0.186608	0.22575	-6.46890518
SLC2A14	0.018191336	0.0805526	1.324074356	0.186658	0.2258	-6.46910553
ERCC6	0.078938752	2.15347487	1.322533089	0.18717	0.2264	-6.47113075
ESR1	0.210258961	1.90580165	1.322383595	0.18722	0.22644	-6.47132706
B3GAT1	0.189946741	3.42394246	1.320479603	0.187853	0.22719	-6.4738254
CELSR2	0.136313117	5.11054249	1.320428535	0.18787	0.2272	-6.47389236
BDNF	0.126925057	1.12062598	1.320305963	0.187911	0.22723	-6.47405307
ESF1	-0.10200418	4.01431781	-1.320210014	0.187943	0.22725	-6.47417886
ARHGAP	-0.077633624	4.09012677	-1.320166165	0.187958	0.22726	-6.47423634
GLYATL2	0.025023796	0.09994049	1.319729271	0.188103	0.22742	-6.47480898
GRK7	-0.010293774	0.05149934	-1.319609556	0.188143	0.22745	-6.47496586
MYH7B	-0.078412206	1.10503804	-1.319243446	0.188265	0.22758	-6.47544553
DEGS1	-0.105498123	7.15932785	-1.318810812	0.18841	0.22774	-6.4760122
SHISA9	0.089678055	0.318361	1.318290655	0.188584	0.22793	-6.47669326
PPP1R14	-0.107689225	1.32725922	-1.316243661	0.189269	0.22874	-6.47937089
PNLIPRP	-0.007770667	0.02404701	-1.316022271	0.189343	0.22882	-6.47966024
GJA9	-0.012897993	0.06480666	-1.315779747	0.189424	0.2289	-6.47997716
GALNT3	0.166651781	4.87351959	1.315266807	0.189596	0.22907	-6.48064725
HMGB2	-0.111719936	6.08757169	-1.315252137	0.189601	0.22907	-6.48066641
PCDHB6	-0.066823983	0.67775648	-1.31519675	0.18962	0.22907	-6.48073875

CCK	-0.026593785	0.07317136	-1.315169764	0.189629	0.22907	-6.48077399
PTPN23	-0.080511305	5.40130104	-1.315147866	0.189636	0.22907	-6.48080259
RBM19	-0.073017476	4.09261102	-1.315111759	0.189648	0.22907	-6.48084975
PLIN5	-0.128979643	1.88840657	-1.314570116	0.18983	0.22927	-6.48155697
PTPRM	-0.147488108	6.0391139	-1.314542644	0.189839	0.22927	-6.48159284
TAS2R31	-0.043900021	0.40223493	-1.314325058	0.189912	0.22934	-6.48187685
SMG1	-0.116872689	3.45337782	-1.313872036	0.190064	0.22951	-6.48246803
RAB3IL1	0.107910952	2.65336542	1.31351471	0.190185	0.22963	-6.4829342
DIRAS3	0.272068668	4.67630545	1.313492561	0.190192	0.22963	-6.48296309
NPIPB12	0.029712827	0.15516825	1.313332129	0.190246	0.22968	-6.48317234
RALGAP	-0.097623495	4.43289336	-1.313016125	0.190352	0.22979	-6.48358443
LMO4	-0.107910274	5.18676722	-1.312708085	0.190456	0.2299	-6.48398604
FBXO40	0.023241357	0.02215424	1.312528926	0.190516	0.22996	-6.48421958
PWP2	-0.079053416	0.55657345	-1.311738827	0.190782	0.23026	-6.48524912
SPOPL	-0.115222788	3.56761673	-1.311628114	0.19082	0.23029	-6.48539334
RPS10-N	0.022009428	0.10843355	1.311557857	0.190843	0.2303	-6.48548485
SREBF2	-0.112240315	5.37477002	-1.310474621	0.191209	0.23073	-6.48689517
LLGL2	-0.073104152	4.74552906	-1.310108891	0.191332	0.23086	-6.48737108
TRIM45	-0.124016852	2.29993176	-1.309728856	0.191461	0.231	-6.48786546
F13A1	0.191034022	3.51669282	1.30871997	0.191802	0.23139	-6.48917721
ENPP2	0.199939535	2.95990617	1.30805346	0.192027	0.23165	-6.49004326
POU2F1	-0.073635005	2.68280177	-1.308019755	0.192039	0.23165	-6.49008705
ESCO2	0.037511965	0.33386697	1.307591078	0.192184	0.2318	-6.49064382
EXOC7	-0.09584185	4.59112537	-1.307403091	0.192248	0.23186	-6.49088792
ELMO3	-0.134373852	5.93884217	-1.307265055	0.192295	0.2319	-6.49106714
RTL5	-0.132134492	5.22641179	-1.306549221	0.192537	0.23218	-6.49199624
KLRD1	0.058477393	0.36544998	1.306505475	0.192552	0.23218	-6.492053
C17orf64	-0.023024837	0.12961128	-1.306424566	0.19258	0.2322	-6.49215798
NAV2	0.169411598	4.90323603	1.306351687	0.192604	0.23221	-6.49225254
HNRNPU	-0.08462692	6.95200747	-1.305932392	0.192747	0.23237	-6.49279644
GALNT15	-0.139060354	2.15370499	-1.304673221	0.193175	0.23287	-6.49442877
RNASE7	0.037967937	0.17980053	1.303444691	0.193593	0.23334	-6.49601989
ZNF592	-0.100271371	4.48049672	-1.303430856	0.193598	0.23334	-6.4960378
ZNF281	-0.101380215	3.10564789	-1.303328554	0.193633	0.23337	-6.49617023
PRDM15	-0.059923632	1.57778109	-1.301912954	0.194115	0.23392	-6.49800163
KLK1	0.292562279	1.4328199	1.301911345	0.194116	0.23392	-6.49800371
ST3GAL4	-0.134663914	3.29081602	-1.301825984	0.194145	0.23394	-6.49811408
DLC1	0.147810424	4.2906121	1.301591723	0.194225	0.23401	-6.49841694
LRRC37A	-0.03455348	0.33278776	-1.301579141	0.194229	0.23401	-6.49843321
HJURP	0.088607105	0.87136509	1.300712365	0.194526	0.23435	-6.49955333

GDA	0.012060749	0.02583101	1.300023667	0.194761	0.23461	-6.5004428
HIST2H2	-0.051231235	0.23809507	-1.299914188	0.194799	0.23464	-6.50058415
DCLRE1I	-0.082663546	2.5952612	-1.298911884	0.195142	0.23504	-6.50187771
CCDC74.	0.135070048	2.99890755	1.298458639	0.195297	0.23521	-6.50246233
CDHR4	-0.031847993	0.05979924	-1.298371921	0.195327	0.23523	-6.50257417
TCP10L2	-0.022140566	0.06831691	-1.298224934	0.195377	0.23527	-6.5027637
MSL1	-0.09201594	4.83593943	-1.296876001	0.195841	0.23581	-6.50450216
KLHL25	-0.081656432	2.11861269	-1.296563576	0.195948	0.23592	-6.50490454
C1orf216	-0.105107056	3.90963074	-1.296437495	0.195991	0.23596	-6.5050669
NUP188	0.091001664	4.63509834	1.296313133	0.196034	0.23599	-6.50522703
NRXN3	0.081308715	0.40386779	1.296289781	0.196042	0.23599	-6.5052571
CEP83	-0.080212299	2.78724943	-1.295767584	0.196222	0.23619	-6.5059293
TRAK2	0.133036779	5.95981856	1.294896911	0.196521	0.23653	-6.5070495
LY75-CD	0.009795145	0.04726833	1.294660561	0.196603	0.23661	-6.50735345
RNASE4	0.086517965	1.10316854	1.294346953	0.196711	0.23673	-6.50775668
PKD2	-0.120339435	4.15207169	-1.293515532	0.196998	0.23705	-6.50882524
MATN1	-0.113656364	0.31835795	-1.293206114	0.197105	0.23717	-6.50922273
NUMA1	-0.104500037	6.02139454	-1.292690212	0.197283	0.23736	-6.50988528
MTBP	0.038923099	0.73842557	1.291061762	0.197846	0.23802	-6.5119749
SPINK2	0.164591151	2.60982213	1.289723572	0.19831	0.23857	-6.51369011
CT55	-0.014009923	0.06739242	-1.289165794	0.198503	0.23878	-6.51440452
RTP4	0.127441361	3.16646782	1.28895649	0.198576	0.23885	-6.51467253
DVL2	-0.086417632	4.52751497	-1.288749239	0.198648	0.23892	-6.51493786
CHN2	-0.146410511	2.76312414	-1.288082403	0.198879	0.23917	-6.51579127
ROBO1	0.122200312	2.46200043	1.288065555	0.198885	0.23917	-6.51581283
PLXDC2	0.117151372	3.90146913	1.287991824	0.198911	0.23919	-6.51590716
PMP22	-0.168484345	5.76296845	-1.287527592	0.199072	0.23936	-6.51650099
STYX	-0.073505034	3.44568007	-1.287448453	0.1991	0.23938	-6.5166022
SALL3	-0.087330176	0.12475449	-1.287197127	0.199187	0.23947	-6.51692358
KIR3DL2	0.033271715	0.1027762	1.286775304	0.199334	0.23963	-6.51746284
FOLH1	-0.121248396	1.61068366	-1.286737526	0.199347	0.23963	-6.51751113
CCL4	0.205033276	2.62307268	1.286332131	0.199488	0.23978	-6.51802921
TBX1	-0.107661933	0.89500497	-1.286255181	0.199515	0.23978	-6.51812753
SNX16	0.10173067	3.4738436	1.286251232	0.199516	0.23978	-6.51813257
ORC6	0.065787377	1.07665906	1.28609848	0.19957	0.23983	-6.51832773
NRIP3	0.108458079	1.45018395	1.285739588	0.199695	0.23996	-6.51878616
GOLGA6	-0.034846755	0.28987194	-1.285697339	0.199709	0.23996	-6.51884012
PARP9	0.120748089	4.48235166	1.285216821	0.199877	0.24014	-6.5194537
CD59	-0.104921978	8.18279833	-1.284469988	0.200138	0.24043	-6.52040688
NEFH	-0.117471415	0.83104774	-1.284443393	0.200147	0.24043	-6.52044081

NRGN	-0.158314588	4.74341649	-1.283904187	0.200335	0.24064	-6.52112865
FAM43B	0.101112397	0.88207283	1.28369604	0.200408	0.24071	-6.5213941
TMPPE	0.076383453	1.12180172	1.28302861	0.200641	0.24098	-6.52224497
PCDHA1	0.123667021	0.83088383	1.281297155	0.201248	0.24169	-6.52445028
PKHD1	0.025963879	0.04220838	1.280913682	0.201382	0.24183	-6.5249383
MAP1B	-0.129050193	3.00562728	-1.280825886	0.201413	0.24185	-6.52505001
MNX1	-0.065805666	0.04846028	-1.280324027	0.201589	0.24205	-6.52568843
S100A1	0.180266896	8.24563464	1.280223251	0.201624	0.24207	-6.5258166
RBM33	-0.082434509	3.29872864	-1.279291271	0.201952	0.24245	-6.52700144
C4orf51	-0.028664576	0.09928818	-1.279060328	0.202033	0.24253	-6.52729491
SCAF11	-0.102785501	4.39187913	-1.278249735	0.202318	0.24285	-6.52832455
ESRRB	-0.047384358	0.1485916	-1.277725916	0.202503	0.24305	-6.52898958
EIF3C	-0.111342372	2.21215673	-1.277703826	0.20251	0.24305	-6.52901762
SSH1	0.098336974	3.60580417	1.276793676	0.202831	0.24342	-6.53017247
PCP4	-0.223184636	6.76981071	-1.27633836	0.202992	0.24359	-6.53074989
ZNF736	-0.086567741	2.37448328	-1.276100045	0.203076	0.24368	-6.53105204
SLC6A7	0.016609964	0.07925396	1.276028372	0.203101	0.24369	-6.5311429
ERAP1	0.120983763	4.11160414	1.274446454	0.20366	0.24434	-6.533147
IMPG2	0.014366526	0.13440039	1.273265408	0.204078	0.24483	-6.53464164
SPATA2!	-0.100102549	1.74755491	-1.273206907	0.204099	0.24483	-6.53471564
SPATC1l	-0.152615511	2.92373536	-1.272829819	0.204232	0.24498	-6.53519255
ACTG1	-0.081692589	11.9332689	-1.272682593	0.204284	0.24502	-6.53537871
TLE4	-0.132426784	2.02719447	-1.272456733	0.204364	0.2451	-6.53566425
MCTP1	0.086118941	1.33134683	1.27221213	0.204451	0.24519	-6.53597344
ANKH	0.141968819	4.68679809	1.271605651	0.204666	0.24543	-6.5367398
GPR31	0.02409298	0.07659657	1.27132323	0.204767	0.24553	-6.53709655
VN1R1	-0.085039112	1.44273154	-1.270283962	0.205136	0.24596	-6.53840866
MFN1	-0.086354319	4.05718426	-1.270132299	0.20519	0.246	-6.53860004
LRP12	0.102752391	2.3630749	1.269662629	0.205357	0.24619	-6.5391926
UBAP2	-0.095179194	3.41397853	-1.26895946	0.205607	0.24647	-6.54007934
STAG1	-0.140263595	3.35781483	-1.268828532	0.205654	0.24651	-6.5402444
TPM4	0.101413256	5.95945892	1.267748431	0.206039	0.24695	-6.5416054
RNF208	-0.11618335	5.35225221	-1.267336037	0.206186	0.24711	-6.54212474
ZP3	-0.122790312	1.8680253	-1.26721071	0.206231	0.24715	-6.54228253
DERL3	-0.231472017	3.77241364	-1.2660267	0.206653	0.24764	-6.54377252
CDKN1C	-0.119959547	3.57832924	-1.265421783	0.20687	0.24788	-6.54453324
HNF1B	0.087283603	0.23828532	1.262951947	0.207755	0.24892	-6.54763545
ZDHHC1	-0.094629433	4.70388346	-1.262603473	0.20788	0.24905	-6.54807266
NOMO2	-0.13264422	2.25096284	-1.261596635	0.208241	0.24946	-6.54933523
VWA5B1	-0.084829868	0.49436312	-1.261577098	0.208248	0.24946	-6.54935972

TNK2	-0.088102316	3.48066934	-1.260867963	0.208503	0.24975	-6.55024836
MDN1	-0.108179162	3.41424049	-1.260226609	0.208734	0.25001	-6.55105164
HNF4G	0.008719122	0.02185499	1.259524127	0.208987	0.25029	-6.55193101
WDR54	-0.112001066	3.84182689	-1.259448928	0.209014	0.25031	-6.55202512
AQP2	-0.039201019	0.13804262	-1.258515041	0.209351	0.25069	-6.55319335
LZTS3	-0.093860563	4.67387465	-1.257032922	0.209886	0.25132	-6.55504562
PTPN5	0.055917649	0.44419225	1.255973632	0.21027	0.25176	-6.55636814
TRIM72	0.025697479	0.06228528	1.255895081	0.210298	0.25177	-6.55646617
CSK	-0.07558516	5.15109942	-1.255180371	0.210557	0.25206	-6.55735781
DENND1	-0.079234682	2.63068892	-1.255137695	0.210572	0.25207	-6.55741103
ZNF750	-0.271863022	3.39814738	-1.254492528	0.210806	0.25233	-6.55821546
SUSD2	-0.21318218	4.24263388	-1.254264923	0.210889	0.25241	-6.55849915
RNASEH	-0.090105984	3.29798251	-1.254153862	0.210929	0.25243	-6.55863756
TECPR2	-0.081044518	3.80504358	-1.254129501	0.210938	0.25243	-6.55866791
KCNAB3	-0.068525886	0.67910144	-1.253389993	0.211206	0.25274	-6.5595892
DSCAML	-0.127047778	1.04905687	-1.252848936	0.211403	0.25294	-6.56026291
KCNA5	-0.115497111	1.21255349	-1.252844502	0.211404	0.25294	-6.56026843
SPTSSB	0.041094241	0.26090233	1.252707294	0.211454	0.25298	-6.56043923
SKA3	0.068825287	0.79565973	1.252427311	0.211556	0.25308	-6.56078771
PAOX	-0.12001225	3.64252833	-1.252277936	0.21161	0.25313	-6.5609736
ANXA11	-0.06775364	6.07974762	-1.252217033	0.211633	0.25314	-6.56104938
HOXA7	0.032207624	0.09403773	1.252074906	0.211684	0.25318	-6.56122622
MLANA	-0.030467816	0.39467786	-1.251068945	0.21205	0.2536	-6.56247729
ISLR	0.204486827	7.33607836	1.250306291	0.212328	0.25392	-6.56342511
ABCA10	-0.053098468	0.49976064	-1.250200068	0.212367	0.25395	-6.56355708
NRM	-0.095432944	4.11413667	-1.248995605	0.212807	0.25445	-6.56505269
ADTRP	0.202310804	1.92075841	1.248723993	0.212906	0.25455	-6.56538976
KL	0.141385501	2.25171707	1.248551931	0.212969	0.25461	-6.56560325
TNFRSF	0.101734506	6.79991015	1.248437742	0.21301	0.25464	-6.56574492
NFIB	-0.108667683	4.74144862	-1.248157708	0.213113	0.25475	-6.56609228
C1QTNF	0.124012902	1.41009973	1.247637165	0.213303	0.25496	-6.56673778
FGL1	0.018514965	0.07336359	1.24745731	0.213369	0.25502	-6.56696075
PCDHB7	-0.077335891	0.84074516	-1.247332775	0.213414	0.25505	-6.56711512
ZDHHC1	-0.069303109	4.9245332	-1.246867787	0.213585	0.25524	-6.56769137
BEST4	-0.110671743	1.53431265	-1.246212463	0.213825	0.25551	-6.56850313
UBL4B	0.017691159	0.06111768	1.244247506	0.214545	0.25635	-6.57093464
GNPDA1	-0.073791861	5.85592505	-1.244173204	0.214573	0.25637	-6.57102651
LRRN1	-0.169399419	3.27044112	-1.243563395	0.214797	0.25662	-6.57178029
SSX1	0.250036172	2.69937638	1.243235075	0.214918	0.25674	-6.57218597
TDRD12	0.082545421	0.20403128	1.242698906	0.215115	0.25696	-6.57284826

WSCD1	-0.075572911	1.38285142	-1.242614427	0.215146	0.25698	-6.57295258
ATOH8	-0.162730576	5.24075373	-1.24257439	0.215161	0.25698	-6.57300202
THOC2	-0.087707621	4.02851981	-1.242200284	0.215298	0.25712	-6.57346391
FAM160A	0.129449836	2.94621893	1.240341346	0.215984	0.25792	-6.575757
ODAPH	0.019059252	0.09375884	1.240082643	0.216079	0.25802	-6.57607585
NPIPB11	0.070889124	0.4603172	1.239737918	0.216207	0.25815	-6.57650062
JMJD1C	-0.114260733	4.05441056	-1.239517201	0.216288	0.25822	-6.57677253
GINS4	-0.067771298	1.15602432	-1.239498523	0.216295	0.25822	-6.57679554
WDR76	0.099056298	2.21537085	1.239476244	0.216303	0.25822	-6.57682298
KANK1	0.125286662	4.1013876	1.238779185	0.216561	0.2585	-6.57768137
ATXN2L	0.088463453	5.54968683	1.238586734	0.216632	0.25856	-6.57791828
PICALM	-0.085176451	6.15981704	-1.238559854	0.216642	0.25856	-6.57795137
ACR	-0.043941605	0.53595192	-1.238401528	0.216701	0.25862	-6.57814624
PAQR3	-0.071958467	1.6859064	-1.23743971	0.217057	0.25902	-6.57932952
CDC7	-0.078948794	1.89636967	-1.237207064	0.217143	0.25911	-6.5796156
CHTF18	0.093278214	2.51960167	1.236006627	0.217588	0.25962	-6.5810909
GLP1R	-0.15590844	0.80935394	-1.235557716	0.217755	0.2598	-6.58164223
NPAS1	0.093768045	1.2603605	1.235384131	0.217819	0.25986	-6.58185537
SMIM14	-0.110108629	6.25789073	-1.235135528	0.217911	0.25995	-6.58216057
MTHFR	-0.093222202	4.80836305	-1.234888283	0.218003	0.26004	-6.58246403
FAM102B	0.115418064	2.58465336	1.233858809	0.218386	0.26048	-6.58372697
CTAGE1	-0.006615868	0.01443707	-1.233736216	0.218432	0.26052	-6.58387729
ADAM11	-0.063149846	0.45030475	-1.232241023	0.218989	0.26116	-6.58570952
TRIM35	-0.076235987	3.92871581	-1.231784957	0.219159	0.26135	-6.58626796
BTAF1	0.127089586	3.75736442	1.231178832	0.219385	0.2616	-6.58700981
ORC1	0.068887188	1.08600745	1.230748432	0.219546	0.26177	-6.58753638
LHX4	-0.050704155	0.51079265	-1.230449863	0.219657	0.26189	-6.58790155
CYP19A1	-0.037007896	0.11753759	-1.230161639	0.219765	0.262	-6.58825398
KIF21A	-0.111374425	2.75328432	-1.22961625	0.219969	0.26222	-6.58892065
AP1G2	-0.109829011	3.67470945	-1.229547121	0.219995	0.26223	-6.58900513
HIST1H3	-0.046424566	0.24850795	-1.229391712	0.220053	0.26228	-6.58919503
SHPRH	-0.092559302	1.38488539	-1.228344346	0.220445	0.26273	-6.59047425
GLIS2	0.097840316	4.84511827	1.22791715	0.220605	0.26291	-6.5909957
SEZ6	-0.009784623	0.05073146	-1.227101066	0.220911	0.26325	-6.59199135
CTDSP2	-0.097457776	6.52322364	-1.226199365	0.221249	0.26364	-6.5930907
IL20RA	-0.082992638	0.74553204	-1.226145674	0.22127	0.26364	-6.59315613
EEF1A2	-0.269513516	2.01517469	-1.225890005	0.221366	0.26374	-6.59346768
ASH1L	-0.130329331	4.2599139	-1.225690275	0.221441	0.26381	-6.59371102
FPGS	-0.07880403	5.64683407	-1.225503933	0.221511	0.26387	-6.59393802
PACSIN1	-0.090495015	1.02320761	-1.225479478	0.22152	0.26387	-6.5939678

TAS2R19	-0.037995883	0.41920189	-1.225414444	0.221544	0.26388	-6.59404702
CLDN15	-0.083992627	2.76034847	-1.224839473	0.221761	0.26412	-6.59474715
SGO1	0.044675191	0.49512931	1.223571628	0.222238	0.26467	-6.59628985
RP2	-0.100304374	3.53547479	-1.223275314	0.22235	0.26478	-6.59665018
TMEM25	-0.075403767	0.33063548	-1.223087527	0.22242	0.26485	-6.59687848
DPYSL2	-0.112837615	4.32002844	-1.222403724	0.222678	0.26513	-6.59770955
SLFNL1	-0.025890803	0.2263513	-1.221640221	0.222967	0.26546	-6.59863693
DAB1	0.021400752	0.09666154	1.220674645	0.223331	0.26588	-6.59980894
SPATA9	0.065198627	1.04234507	1.220328142	0.223462	0.26601	-6.6002293
HOXA10	0.049910681	0.15815139	1.220066791	0.223561	0.26611	-6.60054628
KRT7	0.1452889	9.53748686	1.219367494	0.223826	0.26641	-6.60139409
FBXW12	0.02378362	0.19543246	1.219043995	0.223949	0.26654	-6.60178613
OTOA	-0.028925649	0.13831577	-1.218999532	0.223965	0.26654	-6.60184001
THSD7A	-0.12582806	1.56432068	-1.218824036	0.224032	0.2666	-6.60205264
C17orf98	-0.043805328	0.22100447	-1.218466591	0.224167	0.26674	-6.60248562
PLEKHA1	-0.137819185	3.02124474	-1.217596963	0.224497	0.26711	-6.6035385
TMEM27	0.096915442	2.25774328	1.217461401	0.224549	0.26716	-6.60370256
SMTNL1	0.084268528	0.57892073	1.217410718	0.224568	0.26716	-6.60376389
IGFBP4	-0.106776409	9.89374107	-1.217221883	0.22464	0.26723	-6.60399239
CYP4A22	0.008744479	0.02689741	1.215983814	0.22511	0.26777	-6.60548961
RRAGD	-0.159981573	4.97151432	-1.215039301	0.22547	0.26818	-6.60663081
OPN3	0.126709102	2.67643004	1.214964617	0.225498	0.26819	-6.60672101
PAPSS1	0.105065325	5.81753335	1.214545571	0.225658	0.26836	-6.60722701
NUP58	-0.092522565	3.51479719	-1.214296532	0.225752	0.26846	-6.60752764
RFWD3	-0.081199019	3.58249083	-1.214017364	0.225859	0.26856	-6.60786457
SPATS2L	0.124947337	5.18537013	1.213830207	0.22593	0.26863	-6.60809041
BRSK1	-0.15824501	1.44126724	-1.213545162	0.226039	0.26874	-6.60843431
AP5B1	0.091397691	3.86965647	1.213140044	0.226194	0.26891	-6.60892293
GRIP1	-0.125984342	1.84617745	-1.212901381	0.226285	0.26899	-6.60921071
TLR9	0.011841853	0.06602521	1.212252713	0.226532	0.26926	-6.60999259
NPTX1	0.066647799	0.18303199	1.212225953	0.226543	0.26926	-6.61002483
ASCL4	0.012050226	0.04367442	1.211427258	0.226848	0.26961	-6.61098696
ERVV-1	0.074374732	0.21155356	1.210695397	0.227128	0.26992	-6.61186803
POU6F2	-0.017719696	0.04966131	-1.209937984	0.227418	0.27025	-6.61277931
USP13	-0.098149053	4.18157802	-1.209701619	0.227508	0.27034	-6.61306357
CLDN34	-0.019160328	0.10708607	-1.205675492	0.229055	0.27215	-6.61789717
NBPF9	0.105592346	2.26924029	1.205515743	0.229116	0.27221	-6.61808863
HNRNPF	-0.08736399	7.24592078	-1.205308143	0.229196	0.27228	-6.6183374
HAL	-0.015167042	0.12854902	-1.205046889	0.229297	0.27238	-6.61865041
SLC28A1	-0.021468083	0.10574462	-1.204607771	0.229466	0.27257	-6.61917636

TRIM33	-0.085571644	4.10073532	-1.204177513	0.229632	0.27274	-6.61969152
CHODL	-0.056984264	0.62766509	-1.203798285	0.229779	0.2729	-6.62014543
SMCHD1	0.095141072	3.47690797	1.203683656	0.229823	0.27293	-6.6202826
AQP8	0.070421287	0.32255044	1.20364271	0.229839	0.27293	-6.6203316
RFTN1	-0.11872559	5.88987136	-1.203352686	0.229951	0.27303	-6.62067859
TP53BP2	0.121407756	5.44006005	1.203340573	0.229955	0.27303	-6.62069308
PCDH15	0.007935077	0.01799859	1.20246513	0.230293	0.27342	-6.62173999
EIF3CL	-0.027438361	0.36141013	-1.202287682	0.230362	0.27348	-6.6219521
ERN1	-0.113666237	3.21958061	-1.202043548	0.230456	0.27357	-6.62224387
PLA2G4E	-0.061034059	0.49238599	-1.202000998	0.230473	0.27357	-6.62229471
SORCS2	0.233455368	3.61538154	1.201413139	0.2307	0.27382	-6.62299702
CCDC16	-0.027575482	0.23446773	-1.201009922	0.230856	0.27399	-6.62347854
PNISR	-0.122167621	5.01703293	-1.200689872	0.23098	0.27412	-6.62386062
CHD6	-0.106660126	3.96866339	-1.199717292	0.231357	0.27454	-6.62502111
PPEF2	0.006865757	0.0184173	1.199171886	0.231569	0.27478	-6.62567148
RHPN1	-0.105763489	5.51348775	-1.199108748	0.231593	0.27479	-6.62574675
AMZ1	-0.034288857	0.20099553	-1.198313592	0.231902	0.27513	-6.62669437
NLRP7	0.032460826	0.12672217	1.197944091	0.232046	0.27528	-6.6271345
DDX21	-0.116953605	4.96118387	-1.197267885	0.232309	0.27558	-6.62793963
SEMA4D	-0.097463491	3.16809892	-1.196820644	0.232483	0.27576	-6.62847189
MRPL14	-0.102603792	7.03688065	-1.196120046	0.232755	0.27605	-6.62930527
PLBD2	0.080696718	5.693817	1.196108133	0.23276	0.27605	-6.62931944
CRTC1	-0.112416514	4.57111185	-1.195941101	0.232825	0.27611	-6.62951805
TIGD3	-0.041052325	0.67027656	-1.195300627	0.233075	0.27639	-6.63027938
YY2	0.062253208	1.00155607	1.19508309	0.23316	0.27647	-6.63053788
CD2AP	-0.090498839	4.67710722	-1.194938285	0.233216	0.27652	-6.63070992
ARL13A	-0.043069965	0.45607898	-1.194034539	0.233569	0.27692	-6.63178319
SPRED2	-0.111716568	5.15673158	-1.193809335	0.233657	0.277	-6.63205051
NUSAP1	0.123006467	2.43750605	1.193212231	0.23389	0.27726	-6.63275905
IRX5	-0.053881351	0.37669155	-1.193036856	0.233959	0.27732	-6.63296709
OLFM1	-0.10993493	1.50108084	-1.192695489	0.234092	0.27746	-6.63337194
LEF1	-0.154483587	2.98767981	-1.192366433	0.234221	0.27759	-6.63376209
NACC2	-0.08014695	3.9646131	-1.191904989	0.234401	0.27779	-6.63430903
APOBEC	-0.097865817	5.67603751	-1.190779762	0.234842	0.27829	-6.63564185
SMIM2	-0.03917628	0.2771805	-1.190656343	0.23489	0.27833	-6.63578796
SLC12A6	-0.102734257	3.44431388	-1.190286464	0.235035	0.27848	-6.63622576
SEZ6L	0.072314528	0.23532325	1.189830899	0.235214	0.27867	-6.6367648
PRNP	-0.121329352	7.60052031	-1.189489563	0.235348	0.27881	-6.63716855
AARD	-0.067639221	0.41109497	-1.187984808	0.235939	0.27949	-6.63894706
NPAS2	0.086747541	1.55716847	1.187819675	0.236004	0.27955	-6.6391421

CPLX2	-0.085402001	0.14652198	-1.186862247	0.236381	0.27998	-6.6402724
UNC93B1	0.101081747	4.99201061	1.186447878	0.236544	0.28015	-6.6407613
KHDC3L	-0.021903643	0.08853206	-1.185729627	0.236828	0.28047	-6.64160835
PHKA2	-0.081221176	3.66940941	-1.185481832	0.236925	0.28056	-6.64190047
ARHGDII	-0.12179353	8.06715416	-1.185335918	0.236983	0.28061	-6.64207245
CA9	-0.101523595	0.34513267	-1.1845224	0.237304	0.28097	-6.64303092
C1RL	0.105452747	4.48726999	1.184428215	0.237341	0.281	-6.64314185
MCM8	-0.063447707	1.76104078	-1.184197165	0.237432	0.28108	-6.64341393
GSTA1	-0.125606902	0.47769283	-1.183865259	0.237564	0.28122	-6.64380468
TAS2R13	0.010202224	0.04319814	1.183466013	0.237721	0.28137	-6.64427457
ETV5	-0.144939901	5.38302419	-1.183456953	0.237725	0.28137	-6.64428523
CHD9	-0.099159508	3.49109075	-1.182552386	0.238083	0.28178	-6.64534926
MAP2K7	-0.068704362	4.74536039	-1.182506754	0.238101	0.28178	-6.64540292
CEMIP2	0.111739254	3.69185073	1.182405939	0.238141	0.2818	-6.64552145
WNT10B	0.070256308	0.49662313	1.181944217	0.238324	0.28198	-6.64606419
KIF18B	0.069524208	0.71823827	1.181937688	0.238326	0.28198	-6.64607187
ALPK3	-0.106850076	2.41615542	-1.181594095	0.238462	0.28213	-6.64647562
RGS7BP	-0.050082742	0.20682308	-1.181146295	0.23864	0.28232	-6.64700164
ANXA8L1	-0.043379201	0.19642495	-1.1810539	0.238676	0.28234	-6.64711015
ATRX	-0.098291721	3.71676812	-1.180822546	0.238768	0.28243	-6.64738182
PRSS45P	-0.121750769	1.51157621	-1.180723566	0.238807	0.28246	-6.64749803
SLC23A1	-0.03427644	0.34613623	-1.180509116	0.238893	0.28254	-6.64774978
ZNF454	-0.084784736	1.72110101	-1.179949271	0.239115	0.28278	-6.6484068
SEMA4B	-0.107841775	5.61538965	-1.179731504	0.239201	0.28286	-6.64866227
GNA13	-0.094884391	4.85227139	-1.179637044	0.239239	0.28289	-6.64877308
RIPPLY2	-0.031720097	0.15946569	-1.179573093	0.239264	0.2829	-6.64884809
PCDHB9	-0.073242643	0.62567054	-1.179101148	0.239452	0.28309	-6.64940153
ZC2HC1A	-0.124859019	4.36306963	-1.179069524	0.239464	0.28309	-6.64943861
PTTG2	-0.026502513	0.17967769	-1.178748727	0.239592	0.28323	-6.64981467
PLGLB2	-0.011997969	0.06444508	-1.178577069	0.23966	0.28329	-6.65001585
DEPDC7	0.053560559	0.59281261	1.178358297	0.239747	0.28337	-6.65027222
ERBB2	-0.118822363	6.39960293	-1.177491245	0.240092	0.28376	-6.65128779
GRIN2D	-0.074049334	1.01287336	-1.177147572	0.240229	0.2839	-6.65169013
BTG3	0.090232059	5.82521947	1.17590899	0.240723	0.28446	-6.65313917
AMIGO3	-0.010216378	0.05789613	-1.175468799	0.240898	0.28465	-6.6536538
MAST2	0.075396546	4.35234178	1.174910695	0.241121	0.28489	-6.654306
TTYH3	-0.106790123	4.8125892	-1.174739522	0.24119	0.28496	-6.65450597
TANK	-0.094401642	4.87211868	-1.174684139	0.241212	0.28496	-6.65457067
ELK4	0.083342558	3.3318635	1.174338512	0.24135	0.2851	-6.65497434
KLHL29	-0.060354768	0.73172019	-1.173964575	0.241499	0.28526	-6.65541094

ATP13A4	0.170273276	5.66479963	1.172897162	0.241926	0.28575	-6.65665648
C6orf58	-0.041040282	0.55175599	-1.172317625	0.242158	0.286	-6.65733226
SLC9A3	-0.083617584	0.80267344	-1.172038533	0.24227	0.28611	-6.65765758
PTPRT	-0.07249647	0.13668456	-1.170072134	0.243059	0.28702	-6.65994752
POM121	-0.10882728	3.09775819	-1.170017771	0.243081	0.28703	-6.66001078
SLC39A1	-0.064221683	5.18954541	-1.168939967	0.243514	0.28752	-6.66126425
KNG1	-0.02511605	0.10148431	-1.168795195	0.243572	0.28757	-6.66143253
ARMH1	-0.071507143	0.96265231	-1.168640753	0.243635	0.28762	-6.66161203
TMEM15	0.045151118	0.25815889	1.168468494	0.243704	0.28769	-6.6618122
EPHA7	-0.03160228	0.06209415	-1.167922477	0.243924	0.28791	-6.66244652
CTNNAL	-0.128033525	7.02485719	-1.167900792	0.243932	0.28791	-6.66247171
ZNF471	-0.082955178	2.65810953	-1.167690654	0.244017	0.28799	-6.66271575
TRMT11	-0.092354996	4.22779614	-1.166620161	0.244448	0.28848	-6.66395827
SLN	0.092836477	0.21825971	1.165235235	0.245007	0.28912	-6.66556408
PHF3	-0.102280715	4.85166164	-1.165091924	0.245065	0.28916	-6.66573014
PDIA2	0.030821663	0.15492567	1.165067313	0.245075	0.28916	-6.66575866
GZMM	0.156220948	1.81791396	1.165031325	0.24509	0.28916	-6.66580036
SCN7A	0.07056093	0.31562669	1.164759092	0.2452	0.28927	-6.66611573
FAM193E	-0.096741167	3.88586374	-1.164603041	0.245263	0.28932	-6.66629648
CYP2J2	-0.061603474	0.7402846	-1.164155216	0.245444	0.28952	-6.66681505
LPAR3	0.079101409	0.35769058	1.163843448	0.24557	0.28965	-6.66717595
KRT31	-0.018495971	0.0677346	-1.163683788	0.245635	0.2897	-6.66736073
C9orf50	-0.106065937	1.09134328	-1.163624709	0.245659	0.2897	-6.6674291
TTLL4	-0.057727519	2.15686765	-1.163596749	0.24567	0.2897	-6.66746146
IRF6	-0.093463186	5.5566504	-1.163024723	0.245901	0.28996	-6.66812325
RTN4R	0.12279071	3.58501912	1.162770813	0.246004	0.29006	-6.6684169
GABRP	0.02823827	0.20587455	1.162292604	0.246198	0.29027	-6.66896978
LRBA	-0.103555445	4.57023207	-1.162092967	0.246279	0.29034	-6.66920052
VPS13A	-0.085118772	2.64504956	-1.161597079	0.24648	0.29056	-6.66977351
KRT5	0.170487164	0.76371807	1.161469863	0.246532	0.2906	-6.66992046
GPR20	-0.061514473	0.47276164	-1.159952083	0.247148	0.2913	-6.67167253
THOC5	0.083244829	3.31718532	1.159169892	0.247466	0.29166	-6.67257457
VWDE	0.131390825	1.83527964	1.158541961	0.247722	0.29194	-6.67329828
NNAT	0.079654541	0.74961998	1.158207877	0.247858	0.29208	-6.67368317
YPEL1	0.082810247	1.74946588	1.158083175	0.247908	0.29211	-6.6738268
ANKRD4	0.084513933	1.72081133	1.1580682	0.247915	0.29211	-6.67384405
SMC5	-0.099479497	4.20852415	-1.157668594	0.248077	0.29226	-6.67430422
SKAP2	0.104616953	3.38199374	1.157656715	0.248082	0.29226	-6.6743179
JUP	-0.097768216	7.79445385	-1.157250738	0.248248	0.29243	-6.67478524
KLF10	-0.125117347	5.30481566	-1.157217553	0.248261	0.29243	-6.67482343

NOMO3	-0.099119247	1.1290272	-1.157153899	0.248287	0.29244	-6.67489669
ARHGEF	0.102392208	3.06408946	1.155539108	0.248946	0.29318	-6.67675381
PTPN1	-0.081909711	4.99011922	-1.155529242	0.24895	0.29318	-6.67676514
SLC2A6	0.087783969	1.70037931	1.155401311	0.249002	0.29323	-6.67691216
GRID2	-0.013737764	0.02762063	-1.154983635	0.249173	0.29341	-6.67739204
B4GALN'	-0.053453714	0.55478118	-1.154250594	0.249473	0.29374	-6.67823384
GUCY1B	0.116126432	3.57561233	1.154136773	0.249519	0.29377	-6.6783645
ITPRID1	0.006894333	0.01447915	1.153906073	0.249614	0.29386	-6.67862929
TP53TG3	-0.019346549	0.07974766	-1.153847045	0.249638	0.29387	-6.67869703
C2CD4C	0.196755877	2.38113835	1.153222177	0.249894	0.29415	-6.67941394
GZMH	0.178772315	2.49300148	1.152749412	0.250088	0.29436	-6.67995609
WDR87	-0.052409843	0.2331532	-1.151792197	0.25048	0.2948	-6.68105311
MTRNR2	0.03460722	0.22963205	1.151080199	0.250772	0.29513	-6.68186851
SLC25A3	-0.008761328	0.03801588	-1.150761117	0.250903	0.29526	-6.68223377
CRYBA4	-0.053826226	0.25599905	-1.150698384	0.250929	0.29527	-6.68230557
NOS2	-0.088833232	1.01540078	-1.150552717	0.250989	0.29532	-6.68247228
PAK2	0.075728899	5.36205339	1.150154355	0.251153	0.29549	-6.68292807
DCDC2B	-0.055651079	0.61168238	-1.149367932	0.251476	0.29585	-6.68382741
OR51B5	-0.016747843	0.12798361	-1.148995358	0.251629	0.29601	-6.68425326
MAPK1	-0.110467279	5.50616981	-1.148406467	0.251872	0.29627	-6.68492609
VDR	0.1141915	3.20235933	1.148335839	0.251901	0.29627	-6.68500677
ACVR1C	0.046962436	0.45988673	1.148333741	0.251902	0.29627	-6.68500916
CXXC5	-0.092083998	6.30329909	-1.148024592	0.252029	0.2964	-6.68536222
EPS8L1	-0.116385107	5.172106	-1.147844129	0.252103	0.29647	-6.68556827
CAPS	-0.174309784	5.0686226	-1.147670042	0.252175	0.29653	-6.68576701
FAM47E-	0.021506516	0.17310663	1.147422764	0.252277	0.29663	-6.68604925
SYT2	-0.060963548	0.31197967	-1.147337282	0.252312	0.29665	-6.68614681
IL15RA	0.085270642	2.82581149	1.146914379	0.252487	0.29683	-6.68662933
PTPRH	0.040718375	0.26461395	1.146707474	0.252572	0.29691	-6.68686534
LYPD3	0.105590611	1.63008894	1.1450003	0.253277	0.29772	-6.68881107
KIFC1	0.099154463	2.06115698	1.144548776	0.253464	0.29792	-6.68932521
TAC3	-0.01784986	0.06965814	-1.144458519	0.253502	0.29794	-6.68942796
HPS4	-0.077488846	3.50340409	-1.144121558	0.253641	0.29809	-6.68981149
ZDHHC8	-0.076303904	4.44622173	-1.143869822	0.253745	0.29819	-6.69009794
KAZALD:	-0.093488779	2.24987248	-1.143778832	0.253783	0.29821	-6.69020146
MEIS1	-0.051617537	0.71104022	-1.143613875	0.253851	0.29827	-6.69038912
XPC	-0.108195176	5.4729779	-1.140833427	0.255004	0.29961	-6.69354816
NOTUM	-0.085867068	0.21118595	-1.140741943	0.255042	0.29963	-6.69365197
FGF1	-0.119325243	3.07799051	-1.139525404	0.255548	0.30019	-6.69503165
DIAPH1	0.091571853	6.14295483	1.139514491	0.255553	0.30019	-6.69504402

TBX15	-0.098192363	1.15188454	-1.139263047	0.255657	0.30029	-6.695329
NAV3	0.044465875	0.31795405	1.139179457	0.255692	0.30031	-6.69542372
RHOF	-0.075652994	1.63651368	-1.138879518	0.255817	0.30044	-6.69576356
RTN2	0.110617007	2.45943355	1.138785804	0.255856	0.30046	-6.69586972
TGFB1I1	0.091381758	4.37237231	1.137878807	0.256234	0.30089	-6.69689674
SLC25A2	-0.107956007	3.94220195	-1.137779235	0.256275	0.3009	-6.69700944
MECOM	0.122354596	2.40521864	1.137772213	0.256278	0.3009	-6.69701739
KIAA058	-0.06457197	2.45397691	-1.137652102	0.256329	0.30093	-6.69715332
E2F1	0.118905532	3.56307574	1.137229497	0.256505	0.30112	-6.69763148
ITGA7	-0.122691564	3.84376418	-1.135061509	0.25741	0.30216	-6.70008169
ADGRB1	-0.078868288	0.48644682	-1.134760278	0.257536	0.30229	-6.70042176
ZNF597	-0.073827658	2.38467369	-1.134519138	0.257637	0.30239	-6.70069394
ARAP1	0.088784376	5.96846885	1.134381389	0.257695	0.30243	-6.70084939
NR2C2	0.118629941	3.69397922	1.133925882	0.257885	0.30264	-6.7013633
LYPD6	-0.025755049	0.09334891	-1.13375111	0.257959	0.3027	-6.70156042
ITSN1	-0.070114162	2.31596098	-1.133287848	0.258153	0.30291	-6.70208279
KBTBD2	-0.074685161	4.97474314	-1.132818568	0.258349	0.30312	-6.70261173
RSPO1	0.153086723	0.48670136	1.132728944	0.258387	0.30314	-6.70271273
PAQR7	-0.090759408	4.45322587	-1.131663705	0.258834	0.30364	-6.70391249
ZBTB38	-0.103585307	4.21109297	-1.131362288	0.25896	0.30377	-6.70425177
TBX21	0.092684699	0.90675062	1.131181711	0.259036	0.30384	-6.70445499
CCDC78	-0.087001782	0.98151321	-1.129743501	0.259641	0.30453	-6.70607237
CHRDL1	-0.211327876	1.64854825	-1.129542318	0.259725	0.30461	-6.70629846
N4BP3	0.085202749	2.75614448	1.12946012	0.25976	0.30462	-6.70639082
HEATR1	0.09867384	3.18703351	1.129104237	0.25991	0.30478	-6.70679062
KIF20B	0.083336796	1.96066933	1.128838603	0.260022	0.30489	-6.70708896
RTN4	-0.085330439	6.53721188	-1.128235689	0.260276	0.30517	-6.70776584
HS3ST2	-0.121471886	0.99071304	-1.127242356	0.260695	0.30564	-6.70888026
C14orf17	-0.034642263	0.26893611	-1.126931625	0.260826	0.30577	-6.70922867
SRGN	-0.133413663	6.76003537	-1.126199877	0.261135	0.30611	-6.71004877
CFAP97	-0.102977785	4.68410348	-1.125978319	0.261228	0.3062	-6.71029697
EMP1	0.152223279	6.10475257	1.125022412	0.261632	0.30665	-6.71136729
KLHL40	0.046278523	0.06629335	1.124448612	0.261875	0.30691	-6.71200934
SLC27A2	-0.125930059	3.64688192	-1.123547283	0.262257	0.30734	-6.71301722
MYOZ3	0.036089284	0.25962402	1.12349148	0.26228	0.30735	-6.71307959
NEFM	0.026472105	0.08886209	1.122993865	0.262491	0.30757	-6.71363566
RABGAP	-0.072983536	2.87795677	-1.122464605	0.262716	0.30781	-6.71422683
GADL1	0.010024986	0.02299147	1.122250583	0.262806	0.3079	-6.7144658
CTAGE9	-0.022093616	0.14128625	-1.122032529	0.262899	0.30799	-6.71470923
LYG1	-0.075945307	1.47561196	-1.120143755	0.263701	0.30891	-6.71681587

SLC10A1	0.018367586	0.14441346	1.119878123	0.263814	0.30902	-6.71711186
NPL	-0.106329263	2.39731546	-1.117307546	0.264909	0.31028	-6.71997263
SNX15	-0.035346798	0.57535892	-1.117060833	0.265015	0.31038	-6.72024685
CELF3	-0.007685759	0.02288391	-1.116577169	0.265221	0.3106	-6.72078426
RMI2	0.107615493	3.41935612	1.115759338	0.26557	0.31099	-6.72169247
C19orf67	-0.049566028	0.40124538	-1.115564431	0.265654	0.31106	-6.72190881
CHD2	-0.088560787	4.55634627	-1.115032757	0.265881	0.31131	-6.72249878
EPG5	0.086941893	3.62854741	1.114993874	0.265898	0.31131	-6.72254192
FAM3B	-0.156974423	1.67606793	-1.114442645	0.266134	0.31156	-6.72315327
ANXA2R	-0.075563514	1.45894422	-1.113159359	0.266683	0.31218	-6.72457536
CCNB2	-0.094749408	2.04466778	-1.11154334	0.267376	0.31297	-6.72636387
IFNA1	0.047252722	0.17055871	1.111505543	0.267393	0.31297	-6.72640567
DUSP23	-0.090715903	6.9800854	-1.11125074	0.267502	0.31308	-6.72668743
RPLP2	-0.087232183	9.86231611	-1.110969694	0.267623	0.3132	-6.72699813
PROZ	-0.022912802	0.17929806	-1.110789145	0.2677	0.31327	-6.72719769
AMY2A	0.023808132	0.0993821	1.110741235	0.267721	0.31327	-6.72725064
HMGXB4	0.085929605	3.45715225	1.110365421	0.267883	0.31344	-6.7276659
NUP210L	-0.069723156	0.34557249	-1.108100761	0.268857	0.31455	-6.73016533
PRKAR1	-0.084583627	4.11259584	-1.107944711	0.268925	0.31461	-6.73033737
NEIL3	-0.044148508	0.34741856	-1.107750154	0.269009	0.31469	-6.73055183
MIER2	-0.069476796	4.25425486	-1.1074445	0.26914	0.31482	-6.73088868
KLB	-0.010517636	0.10128716	-1.107229757	0.269233	0.31491	-6.73112528
HBEGF	0.199948465	4.85987024	1.107189467	0.26925	0.31491	-6.73116966
TUBAL3	0.024730758	0.08798076	1.107146277	0.269269	0.31491	-6.73121724
DOCK4	-0.091506854	2.09473275	-1.107097034	0.26929	0.31491	-6.73127149
SLC7A3	0.01097957	0.04568939	1.106655428	0.269481	0.31511	-6.73175785
SSBP4	-0.099863394	5.67498224	-1.106056762	0.269739	0.31539	-6.73241688
MYOC	-0.157944695	0.70816146	-1.104746953	0.270306	0.31603	-6.73385753
LAMP2	-0.095672262	6.83895596	-1.104412796	0.27045	0.31618	-6.73422479
CRTC2	-0.071505229	5.58863159	-1.1035418	0.270827	0.31659	-6.73518157
DBNDD1	0.079665392	3.60783716	1.103507898	0.270842	0.31659	-6.73521879
CUL7	-0.083430424	5.41997415	-1.103025961	0.271051	0.31681	-6.73574786
PTPN6	-0.064636957	5.02021656	-1.102376529	0.271332	0.31712	-6.73646043
DNAH8	-0.008361278	0.03850885	-1.101216694	0.271836	0.31769	-6.737732
TGM5	0.011241974	0.057243	1.101073662	0.271898	0.31774	-6.73788872
FCGR3B	0.05590573	0.33293036	1.100733506	0.272046	0.31789	-6.73826135
CCDC12	0.086054162	3.74031348	1.100296435	0.272236	0.31807	-6.73873997
TAF7L	-0.032883765	0.11355679	-1.100294125	0.272237	0.31807	-6.7387425
GBGT1	0.069486712	2.96907701	1.10003583	0.272349	0.31818	-6.73902527
ZNF83	0.094487092	4.16383076	1.099789891	0.272456	0.31828	-6.73929444

SV2A	-0.173583456	3.6437902	-1.099745129	0.272475	0.31828	-6.73934343
HEYL	-0.133518061	3.66251677	-1.098545323	0.272998	0.31885	-6.74065568
DGKD	-0.10307897	3.72170824	-1.09853482	0.273002	0.31885	-6.74066716
HERC5	0.101321979	2.93536208	1.098437813	0.273044	0.31888	-6.7407732
PLD2	-0.077570805	4.1708473	-1.097551804	0.273431	0.31931	-6.74174124
PCSK7	-0.064820344	1.65639061	-1.097480742	0.273462	0.31932	-6.74181885
KLF6	0.181187851	6.44333506	1.097094849	0.27363	0.3195	-6.7422402
EVC2	-0.07787928	2.64047167	-1.095506897	0.274323	0.32029	-6.74397253
ZNF177	-0.011184184	0.06482214	-1.094728502	0.274664	0.32066	-6.74482079
SV2C	-0.025046419	0.08675859	-1.094653601	0.274696	0.32068	-6.74490238
CDK9	-0.074325505	6.17490857	-1.094485072	0.27477	0.32074	-6.74508595
TMTC4	-0.080949005	3.0649506	-1.093716395	0.275107	0.32108	-6.74592284
WDR64	-0.00992212	0.08119284	-1.093693813	0.275116	0.32108	-6.74594742
ARFGEF	-0.108839766	1.93346111	-1.093683679	0.275121	0.32108	-6.74595845
MCM6	0.090983142	4.04831119	1.092904747	0.275462	0.32146	-6.7468059
RAB6D	-0.021470763	0.15671944	-1.092316801	0.27572	0.32173	-6.74744516
PLA2G2(	0.024765608	0.17221426	1.092269539	0.275741	0.32173	-6.74749653
SERPINE	0.067900934	0.25866037	1.092254944	0.275747	0.32173	-6.74751239
KMT2A	0.090206834	3.73743341	1.091353348	0.276143	0.32217	-6.74849197
STEAP1I	0.027842842	0.25310084	1.091238267	0.276193	0.3222	-6.74861694
C2orf92	-0.07747827	1.31080148	-1.090448551	0.27654	0.32259	-6.74947421
HCAR1	0.15463028	1.83438227	1.089932562	0.276767	0.32281	-6.75003401
MBNL3	-0.082242314	2.69299107	-1.089915286	0.276774	0.32281	-6.75005274
PNMT	0.157606439	1.89251495	1.089552585	0.276934	0.32298	-6.75044607
CKS2	-0.121734234	5.91559258	-1.088452162	0.277418	0.32352	-6.75163863
NBPF19	-0.042525332	0.90868822	-1.088088065	0.277579	0.32369	-6.75203295
AOAH	0.121510267	1.70567723	1.087645797	0.277774	0.32389	-6.75251175
GNRHR	-0.02580757	0.27119101	-1.086014359	0.278493	0.32471	-6.75427628
GM2A	0.083764542	5.1216254	1.085635946	0.27866	0.32488	-6.75468519
RCC1	0.078542354	4.16865612	1.085491178	0.278724	0.32493	-6.75484159
HOXB6	-0.065869974	0.86995665	-1.084909939	0.278981	0.32521	-6.75546931
PRAP1	-0.075856248	0.25156866	-1.084425309	0.279196	0.32542	-6.75599245
WNT8A	0.013167763	0.06183643	1.08441894	0.279198	0.32542	-6.75599932
RIC1	-0.080671242	3.21697088	-1.084250295	0.279273	0.32548	-6.75618131
ADAM17	-0.113431258	3.49769407	-1.083704158	0.279515	0.32574	-6.75677047
SLC5A11	-0.015750094	0.13559966	-1.083593137	0.279564	0.32578	-6.7568902
CNGA3	-0.052805897	0.3546862	-1.082329466	0.280124	0.32641	-6.75825214
CNEP1R	-0.061807475	2.80831975	-1.082241182	0.280163	0.32643	-6.75834723
UCN	0.134293392	2.7578541	1.081727793	0.280391	0.32667	-6.75890005
ALOX12	-0.059853488	1.18498063	-1.081245318	0.280605	0.3269	-6.75941935

CCDC17	-0.050394081	1.01932736	-1.080988756	0.280719	0.32701	-6.75969539
NF1	-0.089304296	3.73935071	-1.08069911	0.280847	0.32714	-6.76000696
CALCA	-0.194542221	0.76760364	-1.080159168	0.281087	0.32738	-6.76058754
CHN1	-0.094566205	2.79055124	-1.080146093	0.281093	0.32738	-6.7606016
CCDC71	0.089384586	4.21234975	1.078976528	0.281613	0.32796	-6.7618582
SNX27	0.078767999	4.46246538	1.078880563	0.281656	0.32799	-6.76196125
ARID4B	-0.084676212	4.09516524	-1.078788113	0.281697	0.32801	-6.76206051
GOLGA8	-0.201847087	4.97894634	-1.078700355	0.281736	0.32804	-6.76215473
AGAP5	-0.025122144	0.28562101	-1.078060139	0.282021	0.32835	-6.76284184
CDHR2	-0.012022606	0.11115894	-1.07781091	0.282132	0.32845	-6.76310921
SEPTIN8	0.097681298	4.93467093	1.077282576	0.282367	0.3287	-6.76367581
CASP4	0.09995719	2.65400876	1.077198872	0.282405	0.32871	-6.76376556
IL17RE	-0.108031803	3.44290852	-1.077185764	0.28241	0.32871	-6.76377961
CRH	-0.03569102	0.11215466	-1.076832101	0.282568	0.32887	-6.7641587
LGALS8	0.065519634	3.91704339	1.076569199	0.282685	0.32898	-6.76444042
SLC26A1	0.078229867	1.65918025	1.076046568	0.282919	0.32923	-6.76500027
RTN1	-0.097653617	1.53704833	-1.076009066	0.282935	0.32923	-6.76504043
SOAT1	-0.075011835	3.87764441	-1.075936956	0.282968	0.32923	-6.76511765
OR1L8	-0.02568561	0.23050116	-1.075923449	0.282974	0.32923	-6.76513212
PLXNA2	-0.108039393	4.01293248	-1.075313989	0.283246	0.32952	-6.76578456
KCNJ12	0.108786315	1.69903985	1.075253335	0.283273	0.32953	-6.76584947
SERTAD	0.097937874	2.00483618	1.074706957	0.283517	0.32979	-6.76643404
OLFML2	-0.126334393	5.57374797	-1.074591262	0.283569	0.32983	-6.76655778
DSEL	-0.041615632	0.67732119	-1.073964534	0.283849	0.33012	-6.76722788
DKK1	-0.099944188	0.73539775	-1.073948673	0.283856	0.33012	-6.76724484
FAM183	-0.109696289	0.39848805	-1.073913835	0.283872	0.33012	-6.76728207
MICU3	0.092391007	2.60343975	1.073794959	0.283925	0.33016	-6.76740913
TRPM1	-0.006423376	0.02042378	-1.073194492	0.284194	0.33045	-6.76805068
PEAK3	0.051541722	0.58459211	1.072882339	0.284333	0.33059	-6.76838406
OTOF	0.014347923	0.08283114	1.072732357	0.284401	0.33064	-6.7685442
IFI44L	-0.142604769	2.51293563	-1.069949754	0.285649	0.33207	-6.77151131
PPDPF	-0.082537086	9.52515587	-1.069816357	0.285709	0.33212	-6.77165336
SIAH2	0.079868306	4.31313102	1.069515639	0.285844	0.33225	-6.77197352
SP100	0.074582327	4.04089026	1.069330777	0.285927	0.33232	-6.77217029
COLGAL	0.13653913	2.62216805	1.069264177	0.285957	0.33234	-6.77224118
IGFBP1	-0.050486463	0.25371178	-1.069154132	0.286006	0.33237	-6.77235829
ADRB2	0.077803626	1.06070274	1.068612721	0.28625	0.33263	-6.77293428
SRM	-0.061337276	5.83498576	-1.067247243	0.286865	0.33332	-6.77438571
LCNL1	-0.035697145	0.10482903	-1.066598317	0.287157	0.33364	-6.77507484
USF3	-0.081144007	2.78501792	-1.066431918	0.287232	0.3337	-6.77525148

UBQLN4	0.085817654	4.74917902	1.066247161	0.287316	0.33378	-6.77544757
PROK2	0.019119278	0.09057487	1.066070547	0.287395	0.33385	-6.77563499
PNMA2	-0.152089343	1.49241286	-1.064216033	0.288233	0.3348	-6.77760114
ADGRB3	0.089246188	0.8768243	1.063542477	0.288537	0.33513	-6.7783144
LIMK1	0.080354406	5.0574721	1.063312506	0.288641	0.33523	-6.77855782
LRP11	-0.091298615	5.97457624	-1.063268166	0.288661	0.33523	-6.77860475
CD44	-0.128927303	7.11497183	-1.062886235	0.288834	0.3354	-6.77900889
PSAP	-0.092037328	10.7560169	-1.062653501	0.28894	0.3355	-6.77925509
SEH1L	-0.062675901	3.43608606	-1.06171583	0.289364	0.33597	-6.78024647
STMND1	-0.012422615	0.03218475	-1.059587448	0.29033	0.33707	-6.78249353
CREB3L1	0.135945404	2.39971569	1.05938337	0.290423	0.33716	-6.78270876
HPCA	-0.092984554	1.24901045	-1.059151565	0.290528	0.33726	-6.78295317
SVOPL	-0.107080204	1.16537589	-1.059097144	0.290553	0.33726	-6.78301054
ANO6	0.124560294	4.66562343	1.057722373	0.291178	0.33796	-6.78445893
APOBEC	0.10844214	1.98550312	1.057458792	0.291298	0.33805	-6.78473642
ARRDC1	0.065679576	5.29472345	1.057432439	0.29131	0.33805	-6.78476415
ITPRIPL1	0.052168823	0.79824711	1.057422107	0.291315	0.33805	-6.78477503
MUSTN1	-0.025744059	0.19906263	-1.057371223	0.291338	0.33806	-6.78482859
RTL10	-0.096296602	3.8780147	-1.056199479	0.291872	0.33865	-6.7860612
IL16	0.104241655	1.63120416	1.054725357	0.292544	0.33941	-6.78760998
XIAP	-0.077095665	4.94724164	-1.054242423	0.292765	0.33964	-6.7881169
CROCC	-0.079902713	3.11880198	-1.05402822	0.292863	0.33973	-6.78834167
HEY2	0.140856806	4.17140235	1.053016451	0.293325	0.34025	-6.78940274
VIT	-0.079462335	0.33939791	-1.052451079	0.293584	0.34052	-6.78999522
U2AF1	0.033561627	0.3301449	1.051973237	0.293803	0.34075	-6.79049573
INSR	-0.088417716	5.35628609	-1.051794199	0.293885	0.34083	-6.7906832
TSPAN2	-0.083163893	1.46795434	-1.051195418	0.294159	0.34112	-6.79130996
CELSR1	0.142176518	3.60691494	1.050779132	0.29435	0.34132	-6.79174549
AGER	0.199123443	5.07597068	1.050097834	0.294663	0.34166	-6.79245791
LIMA1	0.098311762	5.15086902	1.049724315	0.294834	0.34183	-6.7928483
TYRO3	-0.101990126	2.16900872	-1.049596736	0.294893	0.34188	-6.79298161
HEATR6	-0.064221109	3.30847386	-1.04936748	0.294998	0.34197	-6.79322112
MEAF6	-0.081406498	6.382612	-1.049327055	0.295016	0.34197	-6.79326335
SRC	0.080119691	4.50108799	1.049223676	0.295064	0.34201	-6.79337134
FAM153B	0.057403783	0.44658994	1.049135809	0.295104	0.34203	-6.79346311
POGZ	-0.085015965	4.95524092	-1.047411125	0.295897	0.34293	-6.7952629
RBL2	-0.11490035	5.04943658	-1.047148849	0.296018	0.34304	-6.79553634
FFAR2	-0.055399943	0.4492229	-1.047057205	0.29606	0.34307	-6.79563187
GSTM1	-0.275746951	2.45659564	-1.046301372	0.296408	0.34345	-6.79641942
PIPOX	0.037863564	0.65030687	1.045246015	0.296894	0.34399	-6.79751814

CBSL	-0.067576124	0.45622763	-1.044895021	0.297056	0.34414	-6.7978833
CALY	-0.065783169	0.2532626	-1.044870102	0.297068	0.34414	-6.79790923
BTBD18	-0.011118915	0.11088478	-1.043819388	0.297553	0.34466	-6.79900162
CSF3R	0.083027913	1.50112174	1.043805617	0.297559	0.34466	-6.79901593
OBP2B	0.15249498	0.44171816	1.042840283	0.298005	0.34516	-6.80001859
COL13A1	-0.161738057	2.85179436	-1.041558229	0.298598	0.34582	-6.80134878
FLNC	0.108443621	1.03430032	1.041498188	0.298626	0.34583	-6.80141104
SYNPO2	0.096149145	2.14425971	1.041292663	0.298721	0.34591	-6.80162412
NRN1L	0.017523107	0.0901309	1.041123658	0.2988	0.34598	-6.80179931
GFI1B	0.008303056	0.03596437	1.040535865	0.299072	0.34627	-6.80240838
ZNF710	-0.094428476	3.84577243	-1.040485841	0.299095	0.34628	-6.8024602
SLC22A1	-0.104517464	1.42827042	-1.040335528	0.299165	0.34633	-6.80261589
ATP11C	0.08084327	2.61734948	1.039369418	0.299613	0.34683	-6.80361604
CBLC	-0.116262575	1.17708975	-1.038631371	0.299955	0.3472	-6.80437947
MBOAT7	-0.07528758	5.76160642	-1.038190944	0.30016	0.34741	-6.80483479
TSTD2	-0.111912967	2.60750426	-1.038010908	0.300244	0.34749	-6.80502086
ABI3BP	-0.171778017	2.74638481	-1.036875484	0.300771	0.34807	-6.80619359
VANGL2	-0.102480485	4.2627092	-1.03647101	0.30096	0.34827	-6.80661105
ALAS2	-0.034793336	0.1795647	-1.034702418	0.301783	0.3492	-6.80843451
STS	-0.086469717	3.28651139	-1.034142475	0.302045	0.34948	-6.80901118
RHEBL1	-0.056658578	1.37746521	-1.033815855	0.302197	0.34963	-6.80934741
NPTX2	0.12058803	1.32867301	1.033683635	0.302259	0.34968	-6.80948349
NPHP4	-0.09108107	2.82687887	-1.033160183	0.302503	0.34994	-6.81002207
MYH9	0.090022463	8.10030186	1.0326721	0.302731	0.35018	-6.81052401
PALD1	-0.093565415	3.2809134	-1.032146436	0.302977	0.35044	-6.81106433
ISG20	-0.110945392	3.13007995	-1.031836976	0.303121	0.35058	-6.81138229
IRX3	0.164773662	3.48479112	1.031744573	0.303165	0.35061	-6.81147721
TMTC3	-0.073023956	3.32053281	-1.03093907	0.303542	0.35102	-6.81230433
ZNF705E	-0.009469347	0.08131584	-1.03083406	0.303591	0.35105	-6.81241211
CCDC18	-0.102554583	3.00412855	-1.030738784	0.303635	0.35106	-6.81250989
NTRK1	0.066037915	0.16858864	1.030722686	0.303643	0.35106	-6.81252641
SLC52A3	-0.075989274	1.24479849	-1.028890439	0.304501	0.35203	-6.81440505
NPIPB5	0.058606399	0.5864233	1.028691075	0.304595	0.35212	-6.81460926
PRR5	-0.072763007	2.62127912	-1.028507969	0.304681	0.35219	-6.81479678
CDX1	0.02769802	0.2066755	1.027781899	0.305022	0.35256	-6.81554004
OLFML1	-0.100634177	2.22235682	-1.027639404	0.305089	0.35261	-6.81568585
STAC2	0.074680669	0.34153198	1.027583546	0.305115	0.35262	-6.815743
MAPK13	0.112043075	5.69997916	1.026883818	0.305444	0.35298	-6.81645867
ADAMTS	0.140288742	2.01690286	1.026177922	0.305776	0.35334	-6.81718015
PHYHIPL	-0.029339532	0.1272813	-1.024927771	0.306364	0.35399	-6.8184567

RASAL3	0.103883092	2.48853762	1.024782421	0.306432	0.35405	-6.81860502
PM20D2	-0.091053994	4.21824463	-1.024712295	0.306465	0.35406	-6.81867657
USP6	-0.057610539	0.48310687	-1.024606306	0.306515	0.35409	-6.81878471
DUSP18	-0.04583011	1.77931241	-1.023890951	0.306852	0.35446	-6.81951425
GPR39	0.084837524	1.46439354	1.022507164	0.307505	0.35519	-6.82092404
CKAP2	-0.081408852	3.13128275	-1.020451015	0.308477	0.35629	-6.82301533
PML	0.061817854	4.71820785	1.01977001	0.308799	0.35664	-6.82370706
BMP6	-0.1023666	2.06488411	-1.019220122	0.30906	0.35691	-6.82426527
DTX2	0.061695939	3.14290964	1.017295918	0.309972	0.35794	-6.82621624
DPYSL4	0.146495323	1.28049052	1.016548832	0.310327	0.35831	-6.82697274
CAND1	0.093337379	4.92088439	1.016494044	0.310353	0.35831	-6.8270282
CPT1C	0.112595918	1.32946585	1.016488603	0.310356	0.35831	-6.8270337
BCL10	-0.073933537	4.20760962	-1.014928747	0.311097	0.35914	-6.82861133
NHEJ1	-0.025783308	0.48221989	-1.01469076	0.31121	0.35925	-6.82885182
MTMR7	0.109211356	2.14218731	1.012980896	0.312025	0.36016	-6.830578
PAFAH1I	0.08695439	5.33185931	1.01238789	0.312308	0.36047	-6.831176
OSR2	0.100706807	0.75311342	1.012296654	0.312351	0.36049	-6.83126797
KLRF1	-0.130061126	1.53131179	-1.011869213	0.312555	0.3607	-6.83169875
POLG	-0.066036227	4.18774297	-1.011436222	0.312762	0.36091	-6.83213494
DDX58	0.08853352	3.64543619	1.011404428	0.312777	0.36091	-6.83216696
GRIN3B	0.063262767	0.62327034	1.011307879	0.312823	0.36094	-6.83226419
BAZ2A	0.086046169	4.92130054	1.010927964	0.313005	0.36112	-6.83264672
ADRA2C	-0.160604575	2.10186985	-1.010252321	0.313327	0.36145	-6.83332664
EIF4E3	-0.082979296	4.05623786	-1.010240889	0.313333	0.36145	-6.83333814
FTH1	-0.110172451	9.86037878	-1.01010463	0.313398	0.3615	-6.83347521
SAMD10	-0.057214178	3.46485737	-1.009990659	0.313453	0.36154	-6.83358984
PTGDR	-0.049610137	0.55901222	-1.009877912	0.313507	0.36158	-6.83370323
CADPS	-0.05685792	0.11974905	-1.008988494	0.313932	0.36205	-6.83459727
GSG1L	-0.06800204	0.2552801	-1.008562286	0.314136	0.36226	-6.83502541
KRT12	-0.024700506	0.07371193	-1.008347908	0.314239	0.36235	-6.8352407
OLIG1	0.023523721	0.1294275	1.007849647	0.314477	0.3626	-6.83574089
MYOM3	0.052986678	0.65178933	1.007491859	0.314649	0.36277	-6.83609992
AGO2	0.076006229	3.08961639	1.007223781	0.314777	0.3629	-6.83636884
BICD2	-0.068941818	4.21676304	-1.006896524	0.314934	0.36305	-6.83669703
RSKR	-0.065977212	1.11847073	-1.006619818	0.315067	0.36318	-6.83697444
MAPKAP	-0.05728465	6.70543246	-1.006477658	0.315135	0.36324	-6.83711693
OR2A7	-0.089309752	1.61096407	-1.005518	0.315596	0.36374	-6.83807832
BEST1	0.043824448	0.98670737	1.005371397	0.315667	0.3638	-6.83822511
SLC40A1	-0.098931713	6.82851951	-1.004287899	0.316187	0.36437	-6.83930931
GLT1D1	0.155170408	3.40723136	1.004013338	0.316319	0.3645	-6.83958386

SCARB2	-0.085090231	7.5679185	-1.003797787	0.316423	0.36458	-6.83979936
ACRV1	-0.016826493	0.17751077	-1.003773037	0.316435	0.36458	-6.8398241
STK17A	0.08594922	3.47755481	1.003120598	0.316749	0.36492	-6.84047607
TAF1	-0.088348249	3.85672922	-1.002968717	0.316822	0.36498	-6.84062778
RTEL1-T	-0.055590645	0.88599055	-1.002307467	0.317141	0.36532	-6.84128802
CRB3	-0.071309505	5.45021883	-1.001836408	0.317368	0.36556	-6.8417581
VMP1	0.095091011	5.953109	1.001257628	0.317647	0.36586	-6.84233538
SHC1	-0.076477244	6.25823353	-1.000081478	0.318215	0.36649	-6.84350746
TMEM20	-0.057537666	1.02889774	-0.999867244	0.318318	0.36658	-6.84372081
CDC42BI	-0.079550781	3.35342768	-0.999810916	0.318345	0.36659	-6.84377689
PAK3	-0.114246584	1.12424798	-0.999433792	0.318528	0.36677	-6.84415232
TUBB1	-0.020190181	0.17696688	-0.999211955	0.318635	0.36687	-6.84437309
DEFB131	0.074006498	0.84320064	0.999110285	0.318684	0.3669	-6.84447426
SEPTIN9	0.082803044	6.0120419	0.998393082	0.319031	0.36728	-6.84518762
SULT4A1	0.084200535	0.42357809	0.997825083	0.319306	0.36757	-6.84575222
BPGM	-0.06368068	4.73100259	-0.997498421	0.319464	0.36772	-6.84607678
ALPK1	-0.062051121	2.56995358	-0.996592658	0.319903	0.3682	-6.84697616
CCNT2	-0.078200712	3.79070698	-0.996396626	0.319998	0.36829	-6.8471707
DBF4	0.049715741	1.9960537	0.996000903	0.320189	0.36849	-6.84756331
LBX2	-0.05957825	0.89349154	-0.995828628	0.320273	0.36856	-6.84773418
ACTA2	-0.112749572	6.63767918	-0.995339896	0.32051	0.3688	-6.84821876
POLR3C	-0.062812377	4.70134735	-0.995306955	0.320526	0.3688	-6.84825141
RYR3	-0.051951099	0.32060726	-0.995141523	0.320606	0.36886	-6.84841538
ZMAT1	-0.119369238	4.02829487	-0.994865483	0.32074	0.36899	-6.84868892
ERBIN	-0.078790966	4.96315993	-0.994704253	0.320819	0.36906	-6.84884866
PRIM2	-0.05276089	2.46098379	-0.994655285	0.320842	0.36906	-6.84889716
UCP2	0.137184929	7.73883396	0.994348953	0.320991	0.36921	-6.84920057
MUC19	0.005698177	0.01300149	0.993984112	0.321168	0.36937	-6.8495618
MSL3	-0.058351617	3.52648229	-0.993974837	0.321173	0.36937	-6.84957098
SLC2A12	-0.053279996	0.24835074	-0.993663267	0.321324	0.36952	-6.84987937
PHETA1	-0.091279666	4.39131658	-0.992444397	0.321917	0.37017	-6.85108484
MFAP3	-0.119881028	2.79304677	-0.992092669	0.322088	0.37034	-6.85143243
ARMCX3	0.1579476	7.27463493	0.992017616	0.322125	0.37036	-6.85150659
MAGEB1	-0.01832737	0.09823238	-0.991570277	0.322343	0.37059	-6.85194845
PKLR	0.009341499	0.04424286	0.99127789	0.322485	0.37072	-6.85223716
CALHM6	0.151399602	2.44277282	0.991120387	0.322562	0.37079	-6.85239264
HAUS5	0.055677166	3.5797001	0.990273761	0.322975	0.37124	-6.85322799
MYO7A	-0.100941014	1.45153996	-0.989444753	0.323379	0.37167	-6.85404527
ZG16B	0.035451071	0.31587835	0.989408889	0.323397	0.37167	-6.85408061
NEDD9	0.128666012	4.57797742	0.989375284	0.323413	0.37167	-6.85411373

DPT	-0.241412702	2.4776631	-0.989319748	0.32344	0.37167	-6.85416845
IGSF8	-0.077352232	6.69189025	-0.989089237	0.323553	0.37178	-6.85439555
CYP27A1	0.12126848	4.24966646	0.988963131	0.323614	0.37182	-6.85451977
LMO3	0.146883358	6.31970488	0.988098424	0.324037	0.37228	-6.85537111
KCNK13	-0.136804711	3.00608499	-0.987603503	0.324279	0.37253	-6.85585805
FAM9C	-0.00553697	0.01599176	-0.986835303	0.324654	0.37294	-6.85661338
MOB1A	-0.070824314	5.84754395	-0.986230332	0.32495	0.37326	-6.8572078
SLC29A1	-0.08486005	5.75415204	-0.986109488	0.32501	0.37329	-6.85732649
B3GNT4	-0.0297701	0.43502404	-0.9860845	0.325022	0.37329	-6.85735104
HTT	0.068001191	4.36942861	0.985769639	0.325176	0.37344	-6.85766022
HOXC6	0.056231438	0.32066398	0.985459249	0.325328	0.37359	-6.85796492
SERINC2	0.082880821	7.08619538	0.984377959	0.325858	0.37417	-6.85902563
ZNF300	0.106852568	3.12679336	0.983615075	0.326233	0.37458	-6.8597733
MTHFD1	0.084637328	4.2205308	0.982199564	0.326928	0.37535	-6.86115906
PRELID3	0.107120369	2.71977227	0.982004229	0.327024	0.37543	-6.86135014
CENPA	-0.061490798	0.74147427	-0.981914302	0.327068	0.37546	-6.86143809
REXO5	-0.04683083	1.41463398	-0.981227045	0.327406	0.37582	-6.86211
PDS5A	-0.070083306	4.95707147	-0.980605587	0.327712	0.37612	-6.86271717
AGR2	-0.25020886	4.99868186	-0.980604493	0.327713	0.37612	-6.86271824
C1orf61	0.006464666	0.02403835	0.979751248	0.328133	0.37656	-6.86355125
B4GALT6	-0.111917141	2.35461813	-0.979746384	0.328135	0.37656	-6.863556
ST3GAL5	-0.119758301	6.70887245	-0.97908414	0.328462	0.37691	-6.86420204
USP4	-0.064616404	4.94353202	-0.978859849	0.328573	0.37701	-6.86442074
PHYHD1	-0.153701746	4.68305574	-0.978530587	0.328735	0.37717	-6.86474171
ARHGEF	-0.06457119	4.69124395	-0.978383214	0.328808	0.37723	-6.86488534
USP25	0.077853434	3.90496065	0.978039406	0.328977	0.3774	-6.86522032
CD96	0.101062659	1.91384202	0.977816304	0.329088	0.3775	-6.86543764
TDRKH	0.088819335	3.82672966	0.977044009	0.329469	0.37791	-6.86618952
TMEM15	-0.00922494	0.05208548	-0.976592436	0.329692	0.37814	-6.86662889
THEGL	-0.016289577	0.07155632	-0.976182723	0.329895	0.37834	-6.86702735
NTF3	0.063264532	0.4282808	0.976119776	0.329926	0.37836	-6.86708855
DPYSL5	-0.040572763	0.0345625	-0.975586624	0.33019	0.37863	-6.86760678
OGN	0.16224124	1.49623933	0.975102403	0.330429	0.37888	-6.8680772
RHOU	-0.105969832	5.92384079	-0.974443539	0.330755	0.37923	-6.86871692
EPS8L2	-0.102985843	5.72335761	-0.973883353	0.331033	0.37952	-6.86926049
PCDHGA	-0.091065165	0.93595204	-0.973255088	0.331344	0.37985	-6.86986975
RASL11B	-0.1118704	1.06761124	-0.972482499	0.331728	0.38027	-6.87061844
PDLIM5	-0.072382715	4.47212421	-0.971683394	0.332124	0.38068	-6.8713922
NEMP1	0.076836762	3.44652232	0.971673408	0.332129	0.38068	-6.87140186
RGPD2	-0.019684973	0.11496886	-0.971076843	0.332426	0.38099	-6.87197909

WDR86	-0.139233703	5.12763078	-0.970817497	0.332555	0.38111	-6.87222992
NLRP6	0.033779245	0.18948015	0.970606037	0.33266	0.38121	-6.87243438
KIF3A	-0.071667507	3.22836056	-0.970557773	0.332684	0.38121	-6.87248104
ZNF124	-0.052388837	1.51557424	-0.970383296	0.332771	0.38128	-6.87264971
CSTB	-0.062850302	7.74727808	-0.969637808	0.333141	0.38168	-6.87337002
GPR34	0.109657501	2.45674679	0.969166589	0.333376	0.38192	-6.87382505
SKI	-0.075867786	5.46031766	-0.968417767	0.333749	0.38233	-6.87454768
TAOK1	-0.079521917	3.94854036	-0.965990868	0.33496	0.38369	-6.8768859
ARF6	0.074767574	6.52396953	0.965298846	0.335305	0.38406	-6.87755156
WDTC1	-0.067645	6.09728136	-0.964634834	0.335637	0.38441	-6.87818984
PCDHA6	0.04262134	0.26049801	0.964586926	0.335661	0.38441	-6.87823588
CDKN1A	0.122621764	7.95497906	0.963601984	0.336154	0.38495	-6.8791818
ASIC2	0.015168244	0.06217919	0.96335715	0.336277	0.38506	-6.87941678
FOXRED	-0.082589864	3.07437929	-0.963327775	0.336291	0.38506	-6.87944497
MIS18BF	0.076432575	2.89453562	0.96292968	0.336491	0.38526	-6.87982692
ADAMTS	0.113918421	1.99602178	0.962563221	0.336674	0.38544	-6.88017837
AGAP4	-0.053924777	1.18391074	-0.96224475	0.336834	0.3856	-6.88048369
RBAK-RE	-0.017422524	0.13721271	-0.961764605	0.337075	0.38583	-6.88094382
HIPK3	-0.079509853	5.51194944	-0.96174551	0.337084	0.38583	-6.88096212
SOHLH2	-0.040997417	0.30203825	-0.961448873	0.337233	0.38598	-6.88124627
SLC17A3	-0.01258989	0.04062074	-0.96074647	0.337585	0.38636	-6.88191876
SLC1A4	-0.075185204	3.21689327	-0.960537905	0.33769	0.38645	-6.88211835
MAN1A1	0.113602242	3.91786509	0.960287956	0.337816	0.38657	-6.88235749
DLX1	-0.050859695	0.6364103	-0.959591871	0.338165	0.38694	-6.88302314
BMP3	0.025972119	0.13880617	0.957678095	0.339128	0.38802	-6.88485076
C2orf66	-0.028449827	0.2421826	-0.957364776	0.339286	0.38817	-6.88514963
PXDNL	-0.062620029	0.70035696	-0.957062071	0.339438	0.38832	-6.88543828
PLK1	-0.063714983	1.28338708	-0.955910121	0.340019	0.38896	-6.88653593
HSPE1-M	-0.016012611	0.11134406	-0.955452884	0.340249	0.38919	-6.88697125
ANKRD6	-0.005476744	0.01518131	-0.955405577	0.340273	0.3892	-6.88701627
NUFIP2	-0.067811024	4.60371024	-0.954440547	0.34076	0.38973	-6.88793432
MAP3K3	0.071558079	4.02417829	0.953942367	0.341012	0.38999	-6.88840788
CRYGN	0.172277373	4.85681986	0.953021546	0.341477	0.39049	-6.88928256
ALMS1	-0.068841219	2.76608761	-0.952918596	0.341529	0.39053	-6.8893803
CBARP	0.067141473	1.24223529	0.952822215	0.341578	0.39056	-6.88947179
ELN	-0.12767073	3.52677716	-0.952718748	0.34163	0.39059	-6.88957
SLC35G6	-0.018717314	0.10388411	-0.952353213	0.341815	0.39077	-6.88991687
ZBTB20	0.046083217	0.79622127	0.951936864	0.342026	0.39099	-6.89031181
CLDN12	-0.072198491	4.03773371	-0.951666372	0.342163	0.39112	-6.89056829
C6orf132	-0.091745843	4.26767882	-0.951500751	0.342247	0.39119	-6.8907253

ANKRD6	-0.078685053	2.65837181	-0.951372306	0.342312	0.39124	-6.89084705
MED14	-0.075935569	4.16757346	-0.950883791	0.342559	0.39149	-6.89130995
VARS	-0.059478713	5.14624968	-0.95067374	0.342666	0.39159	-6.89150891
SEC14L2	0.102876472	3.73497374	0.950486216	0.342761	0.39167	-6.8916865
PRKAR2	0.077778844	1.96642876	0.949845937	0.343086	0.39202	-6.89229259
TTPA	-0.010389633	0.06312845	-0.949521036	0.34325	0.39218	-6.89259999
PPFIA4	0.054418244	0.71139454	0.948618316	0.343709	0.39267	-6.89345353
FSCN3	-0.009458734	0.07271911	-0.948384198	0.343828	0.39278	-6.89367476
KIAA040	0.031762352	0.20918221	0.948135522	0.343954	0.3929	-6.89390969
TSPAN1	0.090737281	1.12633684	0.94799546	0.344025	0.39296	-6.89404199
ARHGAF	0.126792596	0.75313717	0.947510166	0.344272	0.39321	-6.89450021
CAMKV	-0.024871641	0.11071445	-0.947330177	0.344363	0.39329	-6.8946701
CYP3A7	-0.016378649	0.2565273	-0.947034823	0.344513	0.39343	-6.89494881
CHAF1A	-0.062471435	3.23900639	-0.946819118	0.344623	0.39353	-6.89515231
PC	0.113857014	4.7648555	0.94654578	0.344762	0.39367	-6.89541011
TAF1L	0.005695747	0.02286256	0.946075964	0.345001	0.39391	-6.89585305
SLC16A1	-0.088561474	2.80109444	-0.945623741	0.345231	0.39414	-6.8962792
CSAD	-0.105840767	3.55574242	-0.94558443	0.345251	0.39414	-6.89631623
ACSL4	0.090069435	4.67488107	0.945346293	0.345373	0.39425	-6.89654054
COX6A2	0.060478346	0.19041949	0.945317574	0.345387	0.39425	-6.89656759
CPXM2	0.124060035	2.39460988	0.944851407	0.345625	0.39449	-6.89700653
OTOGL	-0.028788913	0.13393801	-0.944206416	0.345954	0.39484	-6.89761348
SLC26A6	-0.058764682	2.09660052	-0.942670172	0.346738	0.39571	-6.89905748
GRIA2	0.030266536	0.08776747	0.94260523	0.346771	0.39572	-6.89911847
TICAM2	-0.010073615	0.10151764	-0.941900313	0.347131	0.39608	-6.89978023
ADAMTS	0.083170787	2.364066	0.941898082	0.347132	0.39608	-6.89978232
CHDH	-0.07495254	2.14012644	-0.94179742	0.347183	0.39611	-6.89987678
SCIN	-0.100563771	0.97650354	-0.941037507	0.347572	0.39653	-6.90058954
CD82	-0.098704101	5.18026897	-0.940689138	0.34775	0.3967	-6.9009161
USP43	-0.077369153	2.07904335	-0.940376425	0.34791	0.39686	-6.90120914
ZNRF2	0.086306027	4.76653329	0.939807032	0.348202	0.39717	-6.90174245
LACC1	0.072964456	2.65792089	0.939002993	0.348614	0.39761	-6.902495
SLC43A3	0.167803606	4.38866843	0.938692051	0.348773	0.39776	-6.90278586
KCNQ2	-0.007255559	0.02449212	-0.938276595	0.348986	0.39798	-6.90317433
PHC1	-0.073233722	2.78988795	-0.938021058	0.349117	0.3981	-6.90341319
JUNB	-0.125234576	8.70315221	-0.937914588	0.349172	0.39814	-6.90351269
NEURL1I	-0.090875866	3.45459708	-0.93741884	0.349426	0.3984	-6.90397583
IKZF4	0.083419564	3.77323911	0.937286936	0.349494	0.39845	-6.90409902
RAI1	0.094142377	4.51090709	0.936348054	0.349976	0.39897	-6.90497538
RPL39L	0.103569585	3.17421184	0.936071348	0.350118	0.39911	-6.90523349

ERVV-2	0.059866959	0.22400124	0.935969551	0.35017	0.39914	-6.90532842
LRRC37A	-0.056535892	1.19765639	-0.935587046	0.350367	0.39934	-6.90568506
SERPINH	-0.067546413	6.41378513	-0.935095777	0.350619	0.3996	-6.90614289
ZKSCAN	-0.074306976	3.90814791	-0.933936363	0.351216	0.40025	-6.90722245
CCDC10	-0.00501914	0.0200248	-0.932971696	0.351713	0.40079	-6.90811966
FANCD2	-0.008061807	0.05093291	-0.932848933	0.351776	0.40084	-6.90823377
CKAP2L	0.046229299	0.58832167	0.932508372	0.351952	0.40101	-6.90855026
CDK18	-0.055450145	3.9710222	-0.931703945	0.352367	0.40146	-6.90929736
KATNAL1	0.058713117	2.29933714	0.931277013	0.352587	0.40166	-6.90969361
RGPD4	-0.005727223	0.02461736	-0.931270471	0.35259	0.40166	-6.90969968
ARL11	0.092951779	1.26884886	0.930807598	0.352829	0.4019	-6.91012907
TMEM11	0.152865951	2.6839548	0.930635728	0.352918	0.40198	-6.91028846
FANCE	0.075721767	3.43591713	0.930345936	0.353068	0.40212	-6.91055714
ZNF783	-0.056525363	2.81157571	-0.930264776	0.35311	0.40214	-6.91063237
ARX	-0.138934936	0.8952129	-0.930181962	0.353152	0.40216	-6.91070913
DAO	0.007414706	0.03370605	0.930140505	0.353174	0.40216	-6.91074755
MKNK1	-0.052396408	3.05527025	-0.929872558	0.353312	0.40229	-6.91099585
TNFSF12	0.010671782	0.08070037	0.928780859	0.353877	0.40291	-6.91200675
EZH2	0.073875621	2.00684488	0.928593005	0.353974	0.40299	-6.91218058
HRG	-0.014848077	0.06395766	-0.928522636	0.35401	0.403	-6.91224568
DHH	-0.050080426	0.80910946	-0.928370758	0.354089	0.40307	-6.91238619
KDM7A	0.06881614	2.69657038	0.928250783	0.354151	0.4031	-6.91249716
MUC5B	0.022254921	0.04657894	0.928218805	0.354168	0.4031	-6.91252674
CX3CR1	-0.112026471	2.37514475	-0.927974781	0.354294	0.40322	-6.91275241
ZNF462	-0.077483845	3.31185917	-0.927368954	0.354608	0.40355	-6.9133124
PAXBP1	-0.080200926	3.66179196	-0.927118073	0.354738	0.40367	-6.9135442
ELP2	-0.056230785	4.3944505	-0.927043318	0.354776	0.40369	-6.91361325
C2orf72	0.032835424	0.19582553	0.925210534	0.355727	0.40474	-6.91530458
TCF23	-0.011919733	0.06192154	-0.92477825	0.355951	0.40497	-6.91570302
CCDC84	-0.075341975	3.31653761	-0.924331817	0.356183	0.40521	-6.9161143
SFMBT2	0.080325114	2.1559665	0.923972857	0.35637	0.40539	-6.91644486
MYH6	-0.029336336	0.08546551	-0.923811725	0.356454	0.40546	-6.9165932
NRXN1	0.009617885	0.0208279	0.922426579	0.357174	0.40623	-6.91786731
RRAS	0.101687827	7.6696161	0.92238287	0.357197	0.40623	-6.91790749
RPL10L	-0.014708073	0.09655617	-0.922365115	0.357206	0.40623	-6.91792381
FAM107B	0.107106995	3.38573382	0.92176125	0.35752	0.40656	-6.91847864
IFIT1	0.108224475	4.14254769	0.921418037	0.357699	0.40674	-6.91879382
TMEM27	0.007800995	0.03790736	0.921290698	0.357765	0.40679	-6.91891073
OLFML3	-0.145131582	4.2056951	-0.920598536	0.358126	0.40717	-6.91954592
PRLR	-0.034532306	0.1824672	-0.919892723	0.358494	0.40756	-6.92019315

APAF1	0.085194684	2.59663443	0.919742806	0.358572	0.40762	-6.92033056
EDN1	0.10881154	2.35945809	0.918389746	0.359279	0.4084	-6.92156973
NPDC1	0.098702802	6.51353915	0.918231803	0.359361	0.40847	-6.92171426
RGS12	-0.056293873	3.00527343	-0.918047984	0.359457	0.40855	-6.92188244
ANOS1	0.09752782	4.29829772	0.917960053	0.359503	0.40857	-6.92196288
FRYL	-0.063227547	2.77898225	-0.917624394	0.359678	0.40874	-6.92226986
BOD1L1	-0.080665381	3.3885097	-0.917352738	0.359821	0.40888	-6.92251823
GREB1	-0.110180666	0.76258393	-0.916154415	0.360447	0.40956	-6.92361295
MYOF	0.102033102	6.08805878	0.915773413	0.360647	0.40976	-6.92396071
DCHS1	-0.082491246	2.60412007	-0.915596156	0.36074	0.40984	-6.92412245
PRRT2	0.089009223	1.51061924	0.913330414	0.361927	0.41116	-6.92618717
CADM1	-0.111643702	5.64131261	-0.912593872	0.362314	0.41157	-6.92685726
RBPMS	-0.065176971	5.58874067	-0.912383238	0.362425	0.41167	-6.9270488
PGPEP1	-0.058152432	4.89281175	-0.911800651	0.362731	0.41199	-6.92757833
CR2	0.137606199	0.883139	0.910760009	0.363278	0.41259	-6.92852336
RAB3GA	-0.065921671	3.75862769	-0.91057602	0.363375	0.41267	-6.92869033
CYP51A:	-0.0789968	1.85068256	-0.910494092	0.363418	0.41269	-6.92876467
PLAGL2	-0.081514046	3.60170553	-0.910119571	0.363615	0.41289	-6.92910442
BARD1	0.065384333	2.07603585	0.908902976	0.364255	0.41359	-6.93020711
CAPN11	-0.043952993	0.73399388	-0.908603604	0.364413	0.41373	-6.93047823
C12orf54	-0.044173564	0.66881753	-0.908561858	0.364435	0.41373	-6.93051603
CCDC73	-0.015865546	0.15751948	-0.908454858	0.364492	0.41377	-6.9306129
DDIT4	-0.111957143	6.21436944	-0.907735995	0.364871	0.41417	-6.93126345
AGPAT2	-0.072276809	7.32695542	-0.907314012	0.365093	0.4144	-6.93164509
ZNF292	0.074899687	3.18467836	0.907174752	0.365167	0.41443	-6.931771
ELK1	0.060711143	4.92835564	0.907169171	0.36517	0.41443	-6.93177605
GDF6	-0.163008393	0.62580777	-0.906553703	0.365495	0.41477	-6.93233228
RRAD	0.189489059	3.8400589	0.906484031	0.365531	0.41478	-6.93239522
CLSTN1	-0.069004598	7.06632326	-0.905265416	0.366175	0.41548	-6.93349535
ADCY2	-0.033019097	0.15057223	-0.905238554	0.366189	0.41548	-6.93351958
10-Mar	-0.036747142	0.34218367	-0.904688884	0.36648	0.41578	-6.93401531
PCDHGA	-0.108713434	1.56633282	-0.904459068	0.366602	0.41589	-6.93422249
SARDH	-0.087353354	1.73716818	-0.904120734	0.366781	0.41603	-6.9345274
MYOT	-0.052613312	0.26854517	-0.904085112	0.3668	0.41603	-6.93455949
AHSP	-0.029271406	0.23869679	-0.904080366	0.366802	0.41603	-6.93456377
UBXN10	-0.100371745	3.74865293	-0.903412294	0.367156	0.4164	-6.93516547
GPX4	-0.062751159	8.93539041	-0.902644708	0.367562	0.41684	-6.93585627
ATP1A3	-0.07631624	0.38961411	-0.902514056	0.367632	0.41689	-6.93597379
PLEKHH	0.075159912	1.4305103	0.902438745	0.367671	0.41691	-6.93604152
DMBT1	0.046979501	0.20883331	0.90217203	0.367813	0.41704	-6.93628136

HNRNPC	-0.008384174	0.05376804	-0.901614061	0.368109	0.41735	-6.93678288
ZNF559-Z	-0.041262127	0.67381613	-0.901544593	0.368145	0.41736	-6.9368453
OTUD4	0.075063504	3.77321133	0.901368966	0.368239	0.41744	-6.93700308
SYNPO2	0.027567626	0.09718184	0.900073823	0.368926	0.41819	-6.93816569
RHBDL3	-0.025020686	0.28821477	-0.89933093	0.369321	0.41861	-6.93883181
NBPF26	0.06776176	1.1913269	0.89872342	0.369644	0.41895	-6.93937614
CACNA1	0.026663364	0.03893393	0.8984278	0.369801	0.4191	-6.93964088
MLC1	-0.034259688	0.24645189	-0.898296862	0.369871	0.41915	-6.93975811
FBXL20	-0.0571493	3.11242471	-0.897314953	0.370393	0.41971	-6.9406367
MYL2	0.073574952	0.13636902	0.897033668	0.370543	0.41985	-6.94088821
CARMIL1	-0.074435185	3.41498395	-0.896794509	0.37067	0.41997	-6.941102
CBLN3	-0.079988112	2.85431916	-0.896646565	0.370749	0.42003	-6.94123422
NODAL	0.032379951	0.15064106	0.895744973	0.37123	0.42055	-6.94203951
CCDC88	0.081525568	2.20612258	0.895448717	0.371388	0.4207	-6.94230394
SEC14L6	0.085506006	1.03876391	0.893264976	0.372554	0.42199	-6.94425045
ATP2A1	-0.050237828	0.76830653	-0.893113843	0.372634	0.42205	-6.94438499
GCSAML	-0.01280634	0.07405001	-0.892668652	0.372872	0.42229	-6.94478117
MYL9	-0.088093154	8.2798148	-0.892599157	0.372909	0.42231	-6.944843
CALD1	0.07915488	6.15388807	0.892420762	0.373005	0.42239	-6.94500169
HSPB9	-0.045700818	0.49851059	-0.891962738	0.37325	0.42264	-6.94540897
MPP4	-0.00721547	0.06231601	-0.89130361	0.373603	0.42299	-6.94599472
ZNF556	-0.006620058	0.05086932	-0.891283822	0.373613	0.42299	-6.9460123
IL18BP	-0.075380068	2.8876366	-0.89091917	0.373808	0.42319	-6.94633616
KCNF1	-0.044953742	0.37178683	-0.890769966	0.373888	0.42325	-6.94646863
REXO1	-0.051600644	4.01961274	-0.890409647	0.374081	0.42344	-6.94678846
TCEAL6	-0.062115935	0.27381688	-0.890156816	0.374217	0.42356	-6.94701281
UBQLN2	-0.081212652	6.00779918	-0.890014164	0.374293	0.42362	-6.94713936
FRAS1	0.121611218	2.28917428	0.889741194	0.374439	0.42376	-6.94738147
NOL4L	0.068792263	3.82892492	0.889110923	0.374777	0.42411	-6.94794019
PCSK2	0.302910077	6.04949462	0.889069462	0.3748	0.42411	-6.94797693
ATP6V0A	-0.049510583	2.6111829	-0.888212231	0.37526	0.4246	-6.94873618
POM121	-0.006066855	0.02700557	-0.88721535	0.375795	0.42517	-6.94961821
RAB3D	-0.071218967	3.99781927	-0.887176382	0.375816	0.42517	-6.94965267
TET2	-0.063136396	2.22645056	-0.886451518	0.376206	0.42559	-6.95029337
TMEM23	0.064932345	2.93405468	0.88601675	0.376439	0.42582	-6.95067741
GJC2	-0.108161521	1.87254153	-0.885901089	0.376502	0.42586	-6.95077954
CIZ1	-0.058490272	5.81196203	-0.885659334	0.376632	0.42598	-6.95099298
RASSF2	0.089632465	2.76644155	0.88548745	0.376724	0.42606	-6.9511447
KBTBD8	0.052677342	0.90977224	0.885027453	0.376972	0.42631	-6.95155057
CEP128	-0.052287634	1.575325	-0.88425047	0.37739	0.42676	-6.95223567

NCR1	0.043540184	0.27599461	0.883968254	0.377542	0.4269	-6.95248436
ICOSLG	0.039426836	0.37691077	0.883928727	0.377563	0.4269	-6.95251919
MFAP4	0.198849858	3.39497686	0.883588764	0.377747	0.42707	-6.95281865
RBPJL	0.010182552	0.04283812	0.882729098	0.37821	0.42757	-6.95357539
USP32	-0.076199741	3.68769858	-0.882612461	0.378273	0.42761	-6.95367801
HIST1H1	0.069172807	0.62323087	0.881184129	0.379044	0.42845	-6.95493354
SUSD1	-0.089916704	3.4869466	-0.880747634	0.37928	0.42869	-6.95531683
MST1	-0.100400939	3.24666243	-0.879929732	0.379722	0.42916	-6.95603452
SETSIP	-0.021867034	0.26302032	-0.879888849	0.379744	0.42916	-6.95607038
RB1CC1	-0.065646092	4.47133222	-0.879609106	0.379895	0.4293	-6.95631568
PCDHGA	-0.085363389	1.28369178	-0.879283659	0.380071	0.42947	-6.95660097
CSF3	0.096490646	0.42775055	0.879024761	0.380211	0.4296	-6.95682784
ABTB1	0.065227529	3.80109477	0.878248672	0.380632	0.43005	-6.95750754
CYP2E1	0.040408871	0.57929313	0.877488631	0.381043	0.43049	-6.9581726
PRKDC	-0.086125043	4.46536524	-0.876995663	0.381311	0.43076	-6.95860365
ZDHHC2	0.087724074	5.39677353	0.876169847	0.381758	0.43124	-6.95932522
STX16	-0.067377294	5.23794454	-0.874802288	0.382501	0.43205	-6.96051864
RHO	-0.006557948	0.03850901	-0.874608541	0.382606	0.43213	-6.96068757
VGF	-0.13611077	0.81197855	-0.874565089	0.38263	0.43213	-6.96072545
MYO1E	0.066029514	3.94506188	0.874478281	0.382677	0.43216	-6.96080112
EML5	-0.035660514	0.40790972	-0.872886109	0.383542	0.43311	-6.96218772
SHF	0.100302668	3.42865612	0.871544495	0.384273	0.43389	-6.96335416
IGSF1	0.286049956	5.9667114	0.871514099	0.384289	0.43389	-6.96338057
SCNN1D	0.084660816	1.8064917	0.871475978	0.38431	0.43389	-6.96341368
RET	0.136334121	0.94114659	0.871232293	0.384443	0.434	-6.96362534
GRIP2	-0.02621738	0.30479616	-0.87119323	0.384464	0.434	-6.96365927
NTF4	-0.085613663	1.03663764	-0.871141448	0.384492	0.43401	-6.96370423
GOLIM4	-0.083008036	4.96398032	-0.870471946	0.384857	0.43439	-6.96428538
MIEF2	-0.063076377	4.93587997	-0.870060834	0.385082	0.43461	-6.96464202
PIP5K1B	0.075756608	1.1442878	0.869882254	0.385179	0.43469	-6.96479689
HAUS6	0.05142704	2.41426363	0.86864945	0.385852	0.43542	-6.96586512
IL33	-0.106066465	2.70889957	-0.868031125	0.38619	0.43578	-6.96640034
CEP192	-0.071479966	3.02583569	-0.867480835	0.38649	0.43609	-6.96687635
ADORA2	-0.0135803	0.14364452	-0.86729308	0.386593	0.43617	-6.96703869
STAMBP	0.052759109	1.03695215	0.866833601	0.386844	0.43643	-6.96743583
SACS	-0.050635703	1.30099578	-0.866387982	0.387088	0.43667	-6.96782079
CACNB1	0.106753637	2.97188581	0.866094123	0.387249	0.4368	-6.96807454
TRAPPC	-0.085514325	2.60319012	-0.866090076	0.387251	0.4368	-6.96807803
UPK3BL2	0.1231384	1.03544328	0.865391157	0.387634	0.43717	-6.96868121
UPK3BL1	0.1231384	1.03544328	0.865391157	0.387634	0.43717	-6.96868121

CDC25C	-0.039053708	0.45480832	-0.86465165	0.388039	0.4376	-6.96931889
XPO7	-0.057772887	5.45223579	-0.863955675	0.38842	0.438	-6.96991853
ZBTB7C	-0.06155591	5.05200223	-0.863913061	0.388444	0.438	-6.96995523
EIF2S3	-0.059459076	7.03751119	-0.863554706	0.38864	0.43819	-6.97026378
NMRK2	0.037579985	0.17380944	0.862562147	0.389185	0.43878	-6.97111774
GALNT1:	-0.093334661	5.2956316	-0.861614885	0.389705	0.43933	-6.97193181
ALOX15	0.015736653	0.10150636	0.8613115	0.389872	0.43949	-6.97219235
ABRA	0.031273951	0.05105488	0.860559582	0.390285	0.43993	-6.97283768
CPEB1	0.075380516	1.38259834	0.860500921	0.390317	0.43994	-6.97288801
MARVEL	0.054537318	2.92735762	0.857782809	0.391814	0.44159	-6.975216
ZNF609	-0.066117987	3.65288764	-0.856148388	0.392716	0.44258	-6.97661231
SUSD3	0.110058188	3.35452849	0.855964939	0.392817	0.44267	-6.97676887
TRIM71	-0.005684646	0.01538747	-0.855769323	0.392925	0.44276	-6.97693578
SLC15A5	-0.00608131	0.03226726	-0.855712066	0.392957	0.44276	-6.97698462
SLC16A7	-0.049929941	0.68792548	-0.85532097	0.393173	0.44298	-6.97731818
PLXDC1	0.080840587	2.24224948	0.854889285	0.393411	0.4432	-6.97768618
FAM83H	-0.064059565	5.59255816	-0.854873304	0.39342	0.4432	-6.9776998
S100A12	0.052472497	0.47712026	0.854120437	0.393836	0.44364	-6.97834114
ENPP6	0.017845814	0.15349761	0.852878061	0.394523	0.44438	-6.97939826
WDR44	-0.076875973	3.69167818	-0.851165406	0.395472	0.44542	-6.98085302
LAMC1	-0.082037661	6.6817424	-0.850557687	0.395809	0.44577	-6.98136852
TMEM87	-0.06829886	4.75209612	-0.850439464	0.395875	0.44582	-6.98146877
LRRC14I	0.013389362	0.0803228	0.850260053	0.395974	0.4459	-6.98162086
PRKCB	-0.107821651	1.48278079	-0.850027293	0.396103	0.44601	-6.98181814
TSNAX-D	-0.005222049	0.02912728	-0.849523584	0.396383	0.4463	-6.98224488
ITGB1BF	-0.032876493	0.62943343	-0.849299945	0.396507	0.44641	-6.98243426
LPXN	0.064771034	4.14995045	0.848571492	0.396912	0.44681	-6.98305079
GRIN1	0.033421795	0.23270935	0.84856398	0.396916	0.44681	-6.98305715
RBM41	-0.064290777	2.38960745	-0.848250843	0.39709	0.44698	-6.98332201
ADAMTS	-0.044710917	0.50487691	-0.847996029	0.397232	0.44711	-6.98353747
NXF3	-0.035481727	0.16248257	-0.847615281	0.397443	0.44731	-6.98385929
NTS	-0.059319409	0.39879404	-0.847409278	0.397558	0.44741	-6.98403335
NACC1	-0.04972134	5.75456875	-0.846710836	0.397946	0.44782	-6.98462319
UBR5	-0.073023633	4.34430596	-0.846533494	0.398045	0.4479	-6.98477287
CNN2	0.10329522	7.04338584	0.846345954	0.39815	0.44799	-6.98493114
SERPINE	0.15829728	4.83273545	0.846041017	0.398319	0.44815	-6.98518839
KIAA093	0.05762393	3.51194784	0.845426368	0.398662	0.44851	-6.98570665
SPDYE1	0.014504645	0.21966452	0.844725529	0.399052	0.44892	-6.98629713
NECAB2	-0.054385143	0.23403611	-0.844029752	0.39944	0.44932	-6.98688286
ZNF608	0.100999513	3.73945904	0.843457828	0.399759	0.44965	-6.98736397

CBL	0.069602735	3.34749221	0.843384556	0.3998	0.44967	-6.98742559
HBD	0.026361286	0.12437304	0.842276161	0.400419	0.45032	-6.98835697
PDCD1	0.124333156	1.69848482	0.842248764	0.400434	0.45032	-6.98837998
CUL4B	-0.05228249	4.80101329	-0.841491667	0.400857	0.45077	-6.98901545
BRD3	-0.075721588	3.95127391	-0.841310892	0.400958	0.45085	-6.9891671
ZNF662	-0.071004211	2.41723172	-0.839749759	0.401832	0.4518	-6.99047537
TRABD2I	0.080859954	0.89818663	0.839238341	0.402118	0.45207	-6.99090342
HABP2	0.009625719	0.03768964	0.839226263	0.402125	0.45207	-6.99091353
ITPKA	-0.072850064	1.15458888	-0.839007838	0.402247	0.45218	-6.99109627
ICA1L	-0.064498211	2.4047047	-0.838866165	0.402326	0.45224	-6.99121477
FAM228/	-0.05070852	0.42818164	-0.838786469	0.402371	0.45226	-6.99128142
CNDP1	-0.046148691	0.30392926	-0.838620138	0.402464	0.45234	-6.99142051
DPYS	0.011452569	0.08100902	0.838327469	0.402628	0.45249	-6.99166518
HAO2	-0.013953231	0.08595339	-0.837781444	0.402934	0.4528	-6.99212142
TBC1D1	-0.104393641	2.98783167	-0.837313392	0.403197	0.45307	-6.99251227
OR10AD	-0.030002332	0.32451592	-0.837201569	0.403259	0.45311	-6.99260562
DLX5	0.098623168	1.4624322	0.835099473	0.40444	0.45441	-6.9943581
SCART1	0.048355669	0.78174125	0.834925274	0.404538	0.45449	-6.99450313
UBE2I	-0.052992696	5.43230739	-0.834734967	0.404645	0.45458	-6.99466154
PRND	-0.063802452	0.42358243	-0.834446425	0.404807	0.45473	-6.99490164
RSRP1	-0.098219052	4.76665343	-0.834378491	0.404845	0.45474	-6.99495816
RPLP1	-0.06207901	9.67733167	-0.834056737	0.405026	0.45491	-6.99522578
GCNT7	-0.017551351	0.21860913	-0.833540647	0.405316	0.45521	-6.99565483
PLK2	-0.09117091	4.50773628	-0.833271162	0.405468	0.45535	-6.99587877
KMT2C	-0.071997079	3.99609765	-0.832626968	0.405831	0.45573	-6.99641377
SPDYA	0.031380645	0.59404347	0.832576568	0.405859	0.45573	-6.99645561
KMT5C	-0.056760158	2.46699615	-0.831483509	0.406475	0.45639	-6.99736241
CCBE1	-0.026013237	0.13978547	-0.831422874	0.406509	0.4564	-6.99741268
SPON2	0.082084836	1.88843109	0.831328439	0.406562	0.45643	-6.99749096
HOXB2	-0.071231297	1.80820013	-0.830250426	0.40717	0.45706	-6.99838395
FMC1-LU	-0.008354105	0.06409971	-0.830234319	0.407179	0.45706	-6.99839728
SPINK9	-0.037700887	0.35294424	-0.830103927	0.407253	0.45711	-6.99850521
SLC13A5	-0.037236316	0.12479386	-0.829898858	0.407369	0.45721	-6.99867492
HIVEP1	0.078357646	2.93614176	0.828910066	0.407927	0.45781	-6.99949265
RTBDN	0.046437321	0.26164331	0.828805752	0.407986	0.45784	-6.99957886
CHST1	0.116742937	4.89600849	0.828375233	0.408229	0.45808	-6.99993455
FGF13	0.062524132	0.67199574	0.828335108	0.408252	0.45808	-6.99996769
HEG1	-0.095556891	6.38884706	-0.82754841	0.408696	0.45855	-7.00061714
DEF8	-0.058105791	4.87307111	-0.827434408	0.408761	0.45859	-7.0007112
ZNF121	-0.043469435	3.28235525	-0.826945186	0.409038	0.45887	-7.00111471

CACNA1	-0.047237295	0.98459156	-0.826757281	0.409144	0.45896	-7.00126963
SV2B	-0.02018104	0.14754738	-0.82621416	0.409451	0.45928	-7.00171721
ZNF611	-0.051564075	1.93927252	-0.826147649	0.409489	0.45929	-7.001772
IGF1	-0.067523883	0.59777479	-0.825465354	0.409875	0.45969	-7.00233382
CLEC9A	-0.048588839	0.61834692	-0.825347467	0.409942	0.45973	-7.00243084
ZBTB43	0.063685825	3.34593713	0.825120945	0.41007	0.45985	-7.00261724
RGS13	0.033725683	0.14000016	0.824643214	0.410341	0.46012	-7.00301018
CNTRL	0.065849714	3.00677612	0.823945797	0.410737	0.46053	-7.0035834
SRFBP1	-0.069089603	3.42336392	-0.823428892	0.41103	0.46083	-7.00400794
A1CF	-0.004600634	0.01480232	-0.82184442	0.41193	0.46181	-7.00530765
TLR4	-0.075593056	2.60013646	-0.821494837	0.412128	0.462	-7.00559407
TP73	0.046804605	0.79835781	0.821455635	0.41215	0.462	-7.00562619
TYMP	0.108578083	4.73901622	0.821098899	0.412353	0.46219	-7.00591833
PRAME	-0.025809906	0.08026648	-0.820699815	0.41258	0.46242	-7.006245
CYP4F11	-0.153755863	2.02484391	-0.820212207	0.412858	0.4627	-7.00664392
DDA1	-0.040308172	4.68913035	-0.81910786	0.413486	0.46337	-7.00754653
SIGLEC5	0.020079804	0.24705545	0.818658278	0.413742	0.46361	-7.00791364
PLIN2	0.080431686	4.27785969	0.818642371	0.413751	0.46361	-7.00792663
NCOR2	0.059937465	5.04724041	0.8185173	0.413822	0.46366	-7.00802872
ANGPTL	-0.034116366	0.29968369	-0.818329733	0.413929	0.46375	-7.00818179
C2orf16	-0.042784458	1.29555964	-0.817864848	0.414194	0.46401	-7.00856103
TDO2	0.07647459	0.65480939	0.817140102	0.414608	0.46444	-7.00915184
NCAPG2	-0.056186887	2.33235448	-0.816824264	0.414788	0.46462	-7.00940914
FREM1	-0.039631997	0.20204546	-0.81665059	0.414887	0.4647	-7.00955058
KLHL2	0.066468997	5.11317649	0.816096532	0.415203	0.46502	-7.01000162
AP4E1	-0.05595388	2.64892213	-0.816044339	0.415233	0.46502	-7.0100441
SHISA7	0.005828854	0.03551694	0.815991801	0.415263	0.46503	-7.01008685
MPP7	-0.090025123	4.5301214	-0.81564262	0.415462	0.46522	-7.01037091
IL13RA1	0.071077174	6.45762738	0.815550091	0.415515	0.46525	-7.01044617
CERCAM	-0.097975041	4.67361452	-0.815061613	0.415794	0.46553	-7.0108433
ZC3H11B	-0.008315514	0.08101747	-0.814885019	0.415895	0.46561	-7.01098682
SLC5A3	-0.104771608	5.76456682	-0.814816906	0.415934	0.46562	-7.01104216
SLC34A3	-0.060221839	0.68905687	-0.814733181	0.415982	0.46565	-7.01111019
PDK1	0.050497327	1.61054163	0.814158	0.41631	0.46598	-7.01157732
LUZP2	0.014784979	0.07168293	0.8140911	0.416349	0.466	-7.01163163
ZFHX4	0.026512712	0.25293249	0.81389031	0.416464	0.46609	-7.01179462
PCDHA2	0.023634169	0.16336668	0.813345128	0.416775	0.46641	-7.01223694
CNGA1	0.062747762	0.96095743	0.812713173	0.417137	0.46679	-7.0127493
GALNT6	0.084099688	1.88225631	0.812366501	0.417335	0.46698	-7.01303019
GRK2	-0.047032795	5.09532691	-0.811837487	0.417638	0.46728	-7.0134586

LSM11	0.037778844	1.77671267	0.811515104	0.417823	0.46746	-7.01371954
PHLDB3	-0.063896299	2.69209542	-0.811127383	0.418045	0.46768	-7.01403323
NDEL1	-0.05935613	3.90905798	-0.810637394	0.418326	0.46796	-7.01442944
SLC35E2	-0.07620547	4.09569176	-0.810498919	0.418406	0.46802	-7.01454137
YWHAG	-0.058831859	6.29068743	-0.810341666	0.418496	0.46809	-7.01466846
SMG6	-0.090077361	3.13762096	-0.809941861	0.418725	0.46832	-7.01499146
NPAS4	-0.007673679	0.04044674	-0.809140314	0.419185	0.4688	-7.01563854
CYB5R4	-0.043990207	1.96022346	-0.80872282	0.419425	0.46904	-7.01597532
ELOVL4	-0.071704995	1.7159912	-0.808611185	0.419489	0.46908	-7.01606535
MTUS1	0.088025724	4.81136309	0.8082918	0.419672	0.46925	-7.01632284
TRPV3	0.03649498	0.48735375	0.807773838	0.41997	0.46955	-7.01674021
GOLGA6	0.011704611	0.10851602	0.807724642	0.419998	0.46955	-7.01677983
FBXW7	-0.055574947	3.28474184	-0.807467038	0.420146	0.46969	-7.0169873
CC2D1B	0.039202533	4.54962624	0.807236141	0.420279	0.46981	-7.0171732
CHPF2	-0.060945876	5.77939528	-0.806997335	0.420416	0.46993	-7.01736541
ARL6IP6	0.0688284	3.56652147	0.806533746	0.420683	0.47019	-7.01773838
LRP10	-0.075167586	6.50565764	-0.805811602	0.421099	0.47063	-7.01831895
RPL14	-0.05264184	7.63198245	-0.804513559	0.421846	0.47143	-7.0193612
TMPRSS	0.007419259	0.05803122	0.803955272	0.422168	0.47176	-7.01980896
BEND3	-0.051592423	1.85189435	-0.80388158	0.422211	0.47178	-7.01986804
FOXP4	-0.072779568	6.26691106	-0.803721988	0.422303	0.47185	-7.01999597
TIAF1	-0.030389654	0.64197349	-0.802592441	0.422954	0.47255	-7.02090069
INSYN2A	0.030470657	0.37775602	0.799184496	0.424924	0.47472	-7.02362262
AMY1B	0.010404233	0.05703111	0.799063068	0.424994	0.47476	-7.0237194
BTN3A3	0.079117964	3.44552054	0.798979885	0.425042	0.47479	-7.02378568
CCL3	0.119931435	2.79945144	0.798590332	0.425268	0.47501	-7.024096
LRRC66	-0.047479775	0.36847186	-0.797049862	0.42616	0.47594	-7.02532169
NCR3LG	-0.063008496	1.05629725	-0.797042735	0.426164	0.47594	-7.02532736
S100P	-0.044334109	0.42438587	-0.795745569	0.426917	0.47675	-7.02635762
ARPC4-T	0.021200371	0.43221349	0.795277662	0.427188	0.47703	-7.02672885
GATAD2	-0.056215141	4.29545243	-0.795009483	0.427344	0.47717	-7.02694151
TMEM35	-0.052844503	0.87015408	-0.794545776	0.427613	0.47744	-7.02730906
SAMD11	0.159264951	3.07484009	0.794450718	0.427668	0.47744	-7.02738439
SPTAN1	-0.069053791	6.49964075	-0.794437166	0.427676	0.47744	-7.02739512
SPDYE5	0.020169552	0.36548244	0.794226667	0.427798	0.47755	-7.02756188
CDK3	-0.047747231	0.88353809	-0.793076821	0.428467	0.47825	-7.02847201
PTHLH	-0.059864965	1.01921406	-0.793052913	0.428481	0.47825	-7.02849092
EIF4EBP	-0.073792431	5.20703249	-0.792731475	0.428668	0.47843	-7.0287451
MPDZ	-0.074933457	3.23514933	-0.792544908	0.428776	0.47852	-7.02889259
MATR3	0.039401117	1.05829477	0.79230797	0.428914	0.47864	-7.02907984

ADH1B	-0.093643203	0.7876937	-0.79217554	0.428991	0.47869	-7.02918447
RPS2	0.06156965	9.6411337	0.790122385	0.430187	0.47999	-7.03080449
NDNF	-0.047654556	0.53082512	-0.790080145	0.430212	0.47999	-7.03083778
NPIPB4	0.03800404	0.61386237	0.789893099	0.430321	0.48008	-7.03098515
TAF1D	0.052206806	4.03912	0.788633841	0.431055	0.48087	-7.03197639
LTB4R2	-0.047076098	1.01743798	-0.788437928	0.431169	0.48096	-7.03213047
DIO3	0.044163327	0.31945955	0.78803011	0.431408	0.4812	-7.03245107
MYH11	-0.111234927	2.53044788	-0.787934918	0.431463	0.48123	-7.03252589
NUP62	-0.051124867	4.70687554	-0.787396424	0.431778	0.48153	-7.03294892
KRTAP5-	0.012821885	0.1021986	0.787381722	0.431786	0.48153	-7.03296046
ANO9	-0.069792494	2.25149812	-0.787108084	0.431946	0.48167	-7.03317531
EVX1	-0.011291631	0.09726957	-0.786028926	0.432577	0.48234	-7.0340219
LRRC74I	-0.007460418	0.02083615	-0.785941819	0.432628	0.48237	-7.03409019
TSKS	0.018090588	0.22639695	0.784740718	0.433331	0.48312	-7.03503098
FABP7	-0.01849536	0.06389275	-0.780777412	0.435654	0.48568	-7.03812518
ACOXL	-0.03670953	0.70524673	-0.779601481	0.436345	0.48642	-7.03904024
PRSS57	0.048508944	0.55978951	0.777538846	0.437558	0.48774	-7.04064198
RPL12	-0.061565909	9.94020363	-0.777218304	0.437747	0.48792	-7.04089052
SLC24A4	0.01068885	0.13269019	0.777013907	0.437867	0.48802	-7.04104895
GSDMB	0.098588017	2.927481	0.776631663	0.438092	0.48824	-7.04134512
MC1R	-0.069549273	2.13807058	-0.775973839	0.43848	0.48864	-7.04185448
OPRK1	-0.012326059	0.04244668	-0.775576354	0.438714	0.48887	-7.04216204
BAZ2B	-0.055793405	2.47337827	-0.774448938	0.439379	0.48958	-7.04303356
PILRB	-0.076399222	1.58539155	-0.774200134	0.439526	0.48971	-7.04322572
GCOM1	0.00855219	0.05707124	0.773332657	0.440038	0.49025	-7.04389523
ZNF28	-0.068481026	3.14644758	-0.77296583	0.440255	0.49045	-7.04417811
SBK3	-0.025254213	0.23597958	-0.771008547	0.441412	0.49171	-7.04568525
TBX10	-0.025481187	0.13489919	-0.769361206	0.442388	0.49277	-7.04695079
HYAL4	0.018010407	0.04623577	0.76765085	0.443401	0.49386	-7.04826188
CHRM4	-0.016142996	0.23283752	-0.766362459	0.444166	0.49468	-7.0492476
LONRF2	0.135669129	4.86223536	0.766191337	0.444268	0.49476	-7.04937839
TLDC2	-0.077878142	1.77726861	-0.76510856	0.444911	0.49545	-7.05020534
C2CD6	-0.034762394	0.35389708	-0.764471414	0.44529	0.49584	-7.0506914
WDFY1	0.061644088	5.15965402	0.763364836	0.445948	0.49654	-7.05153462
ANKRD3	0.040170454	0.63739734	0.762261019	0.446605	0.49723	-7.05237452
TF	0.027120967	0.21301516	0.761663527	0.446961	0.4976	-7.05282865
C9orf131	-0.007005464	0.05963186	-0.760878261	0.447429	0.49809	-7.05342497
FZD7	0.090083249	3.65618713	0.758778941	0.448682	0.49945	-7.05501614
KIF11	0.056867225	2.20214177	0.758187604	0.449035	0.49981	-7.05546355
MBTPS2	-0.061530077	3.10798835	-0.757590519	0.449392	0.50017	-7.05591496

AP2A1	-0.047670807	5.02987339	-0.756884693	0.449814	0.50061	-7.05644812
DEFB1	-0.130282987	1.87607804	-0.756644043	0.449958	0.50074	-7.05662979
PALLD	-0.086897254	5.55355847	-0.756075593	0.450298	0.50108	-7.05705868
SAT1	-0.06901887	9.89238816	-0.755627298	0.450567	0.50135	-7.05739669
TMEM26	0.009519342	0.09758569	0.754419361	0.45129	0.50212	-7.05830648
GAPDHS	-0.032812045	0.08918862	-0.75400861	0.451536	0.50236	-7.05861552
FCHSD2	-0.050533935	4.65751505	-0.752861717	0.452224	0.50309	-7.05947752
SYT4	-0.01204947	0.06982042	-0.752674789	0.452336	0.50319	-7.05961789
GRIN2B	-0.005901319	0.01283649	-0.752621417	0.452368	0.50319	-7.05965796
PRPS2	0.060837111	5.27038483	0.752269344	0.45258	0.50339	-7.05992223
PROM1	0.088455528	0.73401493	0.75218584	0.45263	0.50341	-7.05998489
HOXA11	0.038679761	0.15638225	0.75186474	0.452822	0.50359	-7.06022578
ALOXE3	0.030537631	0.44281958	0.751483537	0.453051	0.50379	-7.06051162
CEL	0.037284397	0.64594264	0.751474913	0.453057	0.50379	-7.06051809
COCH	-0.037480585	0.49356068	-0.750253972	0.45379	0.50457	-7.06143263
DAG1	-0.065768647	5.81747906	-0.749818515	0.454052	0.50483	-7.06175844
DHX58	-0.079513201	3.50027491	-0.749705689	0.45412	0.50487	-7.06184283
NKG7	0.128342216	3.55070611	0.749362034	0.454326	0.50507	-7.06209979
ZNF701	-0.041081759	2.14554511	-0.748668965	0.454743	0.5055	-7.06261766
AFF4	-0.073381749	5.09742101	-0.748539218	0.454822	0.50555	-7.06271455
PATL1	0.058155367	4.8334454	0.748312957	0.454958	0.50567	-7.06288348
ZNFX1	0.058214683	4.84524117	0.747967716	0.455166	0.50587	-7.06314115
SYT6	-0.013142911	0.12954647	-0.747479655	0.45546	0.50616	-7.06350521
DNAJB13	-0.02988673	0.214911	-0.746346222	0.456143	0.50689	-7.06434975
NIPAL1	0.08149444	1.97171536	0.746106567	0.456287	0.50702	-7.06452816
CLDN7	-0.076440865	7.79064518	-0.746025798	0.456336	0.50704	-7.06458827
CNR1	0.079732165	0.75055169	0.745893015	0.456416	0.50707	-7.06468708
SYNGR2	-0.047705701	6.80081578	-0.745869658	0.45643	0.50707	-7.06470446
DOP1A	-0.067983489	3.3563647	-0.745416443	0.456703	0.50735	-7.06504159
HAGHL	-0.078440808	1.80564171	-0.745285726	0.456782	0.5074	-7.06513879
NBPF15	0.078359752	2.95758559	0.745240732	0.456809	0.5074	-7.06517224
ZBTB7A	-0.040658926	4.98419863	-0.745122749	0.45688	0.50744	-7.06525995
KCNH7	-0.009255282	0.05545277	-0.744957803	0.45698	0.50752	-7.06538255
HPX	0.028680285	0.42523331	0.744790726	0.457081	0.5076	-7.0655067
ULBP1	-0.100113039	1.5106478	-0.743191624	0.458047	0.50864	-7.06669358
C12orf76	-0.04436096	3.20845809	-0.74310994	0.458096	0.50866	-7.06675414
ZNF107	-0.06442757	2.48041853	-0.7430071	0.458158	0.50869	-7.06683037
SMCO1	0.012781073	0.04821792	0.742908276	0.458218	0.50873	-7.06690362
RNF207	0.065291538	2.29108878	0.742127323	0.45869	0.50919	-7.06748211
REC114	-0.013444889	0.1188983	-0.742118992	0.458695	0.50919	-7.06748828

CADM2	-0.036969806	0.19439967	-0.742013413	0.458759	0.50923	-7.06756644
PPARGC	-0.020682094	0.4080291	-0.741367964	0.459149	0.50963	-7.06804403
FBN1	-0.124001973	4.21811655	-0.741119375	0.4593	0.50976	-7.06822786
GLB1L3	-0.09388533	0.79897898	-0.741050863	0.459341	0.50977	-7.06827851
KIF20A	0.067906995	1.43217442	0.740885638	0.459441	0.50985	-7.06840065
MAML1	-0.055282081	3.85134977	-0.740429617	0.459717	0.51013	-7.0687376
PARP6	-0.050384211	4.24509653	-0.740232202	0.459837	0.51022	-7.06888341
KCNH8	0.027325613	0.25418462	0.739979263	0.45999	0.51036	-7.06907017
ZNF669	-0.057418997	2.77952228	-0.73980238	0.460097	0.51045	-7.06920073
ZNF770	-0.121119202	4.57795716	-0.739300449	0.460401	0.51075	-7.06957106
AKAP9	-0.074160898	3.80849878	-0.738837276	0.460682	0.51103	-7.06991257
ADAP2	-0.056510933	2.97178784	-0.738580518	0.460838	0.51117	-7.0701018
ISM2	0.035417186	0.39244091	0.738478959	0.460899	0.5112	-7.07017662
SPTA1	-0.005184888	0.03322188	-0.737914619	0.461242	0.51154	-7.07059224
UVSSA	0.059725802	2.26773738	0.737835558	0.46129	0.51154	-7.07065044
CRIPAK	0.059725802	2.26773738	0.737835558	0.46129	0.51154	-7.07065044
SMURF1	0.052529887	3.58251313	0.737479882	0.461505	0.51174	-7.07091219
TP53	-0.048413424	5.33351757	-0.737117377	0.461725	0.51195	-7.07117884
CDR2L	-0.060265117	3.10257763	-0.737072054	0.461753	0.51195	-7.07121216
AGRN	0.073945285	6.97268781	0.735347165	0.462801	0.51308	-7.07247907
ATXN7	-0.051060285	3.24470852	-0.734848856	0.463104	0.51335	-7.07284452
LOXL2	-0.076031844	3.29903014	-0.73484359	0.463107	0.51335	-7.07284838
VMO1	0.074914083	2.09535059	0.734389147	0.463383	0.51362	-7.07318145
STIL	0.032163203	1.02309646	0.734039836	0.463596	0.51382	-7.07343732
BACE1	-0.074792622	4.41888285	-0.732814615	0.464341	0.51462	-7.07433384
TEX48	-0.014461003	0.101734	-0.732625419	0.464457	0.51471	-7.07447215
PPIAL4C	-0.01201997	0.09615127	-0.731825073	0.464944	0.51522	-7.07505682
KIF1B	-0.058155635	3.81367853	-0.731648447	0.465052	0.5153	-7.07518577
PCDHGC	0.026244701	0.28512414	0.731606074	0.465078	0.5153	-7.0752167
PNPLA6	0.04903401	5.01278666	0.730949494	0.465478	0.51571	-7.07569573
DCDC2	0.083403501	4.06215689	0.730512582	0.465744	0.51597	-7.07601426
ARMC9	-0.048441693	2.84292886	-0.729835174	0.466158	0.51637	-7.07650774
MCM10	0.034437352	0.70900516	0.729815241	0.46617	0.51637	-7.07652225
KIR2DL3	0.012168859	0.07781037	0.729211327	0.466538	0.51675	-7.0769618
ZNF385D	0.034784446	0.39086266	0.729009282	0.466662	0.51685	-7.07710878
DOCK8	-0.067142035	2.41495303	-0.728797636	0.466791	0.51696	-7.07726269
TMPRSS	-0.010475563	0.0960211	-0.728454091	0.467001	0.51716	-7.07751243
CEP85L	-0.037984056	1.29601755	-0.728363225	0.467056	0.51719	-7.07757847
WASHC2	-0.041939659	4.47135672	-0.728216916	0.467146	0.51725	-7.07768478
TTYH1	0.014357179	0.15884257	0.727234924	0.467746	0.51788	-7.07839776

PRB3	0.03209742	0.2778824	0.727172929	0.467784	0.51789	-7.07844274
NLN	0.059186925	3.07502375	0.726919216	0.467939	0.51803	-7.07862678
KDM5A	-0.056238021	3.72002097	-0.726298436	0.468319	0.51842	-7.07907682
GAL	0.023871929	0.19018445	0.725048628	0.469084	0.51923	-7.0799817
XRN1	0.054506524	3.27149928	0.724720281	0.469285	0.51942	-7.08021917
C12orf75	0.078873681	2.24009808	0.72424406	0.469577	0.51971	-7.0805634
RCVRN	-0.009498687	0.05619501	-0.723917056	0.469777	0.51989	-7.08079964
VPS13B	-0.062174667	2.69158598	-0.722092912	0.470896	0.5211	-7.08211552
ANTXR2	-0.06101418	2.07891409	-0.721723857	0.471122	0.52131	-7.08238134
ATP8B2	0.078330616	3.86366207	0.720921414	0.471615	0.52183	-7.08295885
PLAAT3	0.05850679	6.49592041	0.720852487	0.471657	0.52184	-7.08300843
RALGDS	-0.053064109	4.78971717	-0.720545501	0.471846	0.52201	-7.08322918
POTEF	0.004770501	0.03295384	0.719970372	0.472199	0.52237	-7.08364249
CC2D1A	-0.03662713	5.05828585	-0.71953437	0.472468	0.52263	-7.08395559
TRPC1	0.060605081	2.64296844	0.718841477	0.472894	0.52303	-7.0844528
SMARCC	-0.05793378	5.3191743	-0.718808222	0.472914	0.52303	-7.08447665
UPK3B	-0.060910056	0.6867272	-0.718806269	0.472915	0.52303	-7.08447805
GABRR1	-0.006388567	0.0422951	-0.718611784	0.473035	0.52312	-7.08461751
CTRL	-0.005421687	0.04773261	-0.718521779	0.47309	0.52315	-7.08468204
FCAMR	0.048411784	0.19539606	0.718155335	0.473316	0.52337	-7.08494468
CHRNB2	-0.011191716	0.07925427	-0.717799593	0.473535	0.52357	-7.08519953
TSPAN1	-0.144797921	7.36029319	-0.717325479	0.473827	0.52386	-7.08553897
FGFR3	0.103947561	2.18448584	0.716840535	0.474125	0.52415	-7.08588594
SUSD6	0.06984403	5.91738332	0.716800293	0.47415	0.52415	-7.08591472
MTRNR2	-0.018037805	0.25017057	-0.716713399	0.474204	0.52418	-7.08597687
ARID4A	-0.057084243	3.59717628	-0.716002521	0.474642	0.52463	-7.08648498
ORM2	-0.04455796	0.37737824	-0.715945724	0.474677	0.52463	-7.08652555
SHC2	-0.072175216	4.72017865	-0.715757556	0.474793	0.52473	-7.08665995
PAQR4	0.065326828	3.02464655	0.715694836	0.474832	0.52473	-7.08670474
PCDHB2	0.101136513	1.68655579	0.715072903	0.475215	0.52512	-7.08714867
PKN1	-0.039189842	6.50075889	-0.714654897	0.475473	0.52537	-7.08744683
SLC9C2	-0.011514466	0.07496483	-0.714367611	0.47565	0.52554	-7.08765164
SEC14L4	0.03553942	0.29508865	0.714291233	0.475697	0.52555	-7.08770608
ZNF417	-0.044519484	2.41211062	-0.714101695	0.475814	0.52565	-7.08784115
LCT	-0.00407633	0.01968913	-0.713430326	0.476229	0.52607	-7.08831929
SIMC1	0.036884957	2.46291774	0.713350314	0.476278	0.52609	-7.08837624
ZMIZ1	-0.068733787	5.62518242	-0.713156033	0.476398	0.52619	-7.08851451
LENG8	0.085559793	5.99072381	0.712376724	0.47688	0.52669	-7.08906874
AVPR1B	-0.030491073	0.43741798	-0.711863185	0.477197	0.527	-7.08943364
USP24	0.04811563	3.82137862	0.711775822	0.477251	0.52703	-7.08949569

CRIP1	-0.083065557	2.69179758	-0.711336144	0.477523	0.5273	-7.08980786
NDFIP2	-0.057124544	4.42960722	-0.709985193	0.478359	0.52818	-7.09076582
SYMPK	-0.037904747	4.98518605	-0.709424835	0.478706	0.52853	-7.09116263
TP53TG3	-0.004291166	0.01739447	-0.709038635	0.478945	0.52873	-7.09143594
TP53TG3	-0.004291166	0.01739447	-0.709038635	0.478945	0.52873	-7.09143594
CCDC15!	0.006891127	0.05441261	0.708864529	0.479053	0.52881	-7.0915591
PCDHGE	-0.097790907	2.5339493	-0.708621688	0.479203	0.52894	-7.09173084
GNPNAT	-0.066923293	3.34721923	-0.708578442	0.47923	0.52894	-7.09176141
PATJ	-0.062611182	4.50995937	-0.708015711	0.479579	0.52929	-7.09215912
ACSM2A	-0.005881582	0.02808787	-0.707855446	0.479678	0.52937	-7.09227233
TJP1	-0.059685846	5.10476115	-0.707771789	0.47973	0.52937	-7.09233142
ST8SIA1	-0.046861914	0.52272052	-0.707753272	0.479742	0.52937	-7.0923445
ZNF404	-0.065075798	2.75401484	-0.706010788	0.480823	0.53053	-7.09357355
SYCE1	-0.045456519	0.18776497	-0.705797433	0.480955	0.53061	-7.09372383
SDC1	-0.077143174	7.02949995	-0.705784902	0.480963	0.53061	-7.09373265
PER1	-0.089477194	5.92301756	-0.705145742	0.48136	0.53101	-7.09418258
GAS1	-0.095544694	2.06319122	-0.704971365	0.481468	0.5311	-7.09430526
ANKHD1	0.027667278	1.20399205	0.704689546	0.481643	0.53126	-7.09450347
FGL2	0.095937079	3.57273749	0.704276705	0.4819	0.53151	-7.09479368
EEF2	-0.053304675	11.0606968	-0.703193867	0.482573	0.53221	-7.09555406
TBC1D2E	0.058206055	3.84746687	0.702517848	0.482994	0.53264	-7.09602818
KLHL21	-0.055716173	4.70440049	-0.702352806	0.483097	0.53272	-7.09614387
ZDHHC1	0.00966222	0.09855783	0.701735151	0.483481	0.53309	-7.09657656
LMBR1L	0.059331993	3.99127315	0.701718069	0.483492	0.53309	-7.09658852
DLEC1	0.067051617	1.1998236	0.699273636	0.485015	0.53473	-7.09829717
PCDHGA	-0.066076521	0.87153237	-0.699111078	0.485117	0.53481	-7.09841058
RLF	-0.05300604	3.33199841	-0.698877072	0.485263	0.53494	-7.0985738
HTR1B	0.030177518	0.34775273	0.697630883	0.486041	0.53576	-7.09944211
SMIM18	0.009182262	0.09236909	0.697397417	0.486186	0.53589	-7.09960461
VCL	-0.065942273	5.80319352	-0.697266973	0.486268	0.53594	-7.09969538
NOM1	-0.037095473	2.77107998	-0.697215124	0.4863	0.53594	-7.09973145
PHACTR	0.047317509	1.24507493	0.697164464	0.486332	0.53594	-7.0997667
SMC2	-0.059909804	3.32208468	-0.696965145	0.486456	0.53604	-7.09990534
FOXN1	-0.031060477	0.34378996	-0.69655078	0.486715	0.53629	-7.10019344
CLCA4	-0.004859208	0.02723216	-0.696475255	0.486763	0.53631	-7.10024593
PGPEP1	-0.008706541	0.07851418	-0.6963372	0.486849	0.53637	-7.10034187
EMX2	0.038964503	0.25689767	0.696085935	0.487006	0.53651	-7.10051644
BRWD3	-0.05095408	2.23987529	-0.695256671	0.487525	0.53702	-7.10109211
ACP4	-0.021944486	0.25197747	-0.695238035	0.487536	0.53702	-7.10110504
MORC3	0.056902865	4.05878791	0.694875288	0.487763	0.53724	-7.10135664

PKP4	0.058990353	5.75602229	0.693576389	0.488576	0.5381	-7.10225647
RTKN	0.052255722	4.70531845	0.693512425	0.488616	0.53811	-7.10230074
CHFR	-0.032275687	2.15592131	-0.693324003	0.488734	0.5382	-7.10243112
PCDHGE	0.065365022	1.23534624	0.692964638	0.488959	0.53842	-7.10267969
IFIH1	0.062583386	3.93310131	0.692643406	0.489161	0.5386	-7.10290177
TMEM41	0.046774995	3.67519669	0.692073352	0.489518	0.53896	-7.10329562
STAT3	0.056439743	6.1238077	0.691946126	0.489598	0.53902	-7.10338348
IRAK3	-0.046459768	1.46487696	-0.691648977	0.489784	0.53919	-7.10358862
GHRL	-0.036285615	0.98410825	-0.691191269	0.490071	0.53947	-7.10390443
ASB4	0.025500801	0.36545045	0.690912459	0.490246	0.53962	-7.1040967
GCH1	0.04920191	2.69516309	0.690645887	0.490413	0.53977	-7.10428045
CHSY1	-0.060062131	4.06933971	-0.690585712	0.490451	0.53978	-7.10432192
NOXA1	-0.072955112	5.14900186	-0.690273284	0.490647	0.53996	-7.10453718
ANKRD1	0.054193635	5.42360002	0.689194749	0.491324	0.54067	-7.10527954
ST8SIA6	-0.03658694	0.52533193	-0.689107292	0.491379	0.5407	-7.10533968
CXCR4	0.104354705	5.35529884	0.689046892	0.491417	0.5407	-7.10538122
CDC45	0.053447477	1.59555274	0.688713997	0.491626	0.5409	-7.10561007
STRN	0.049973104	3.28657224	0.688067744	0.492033	0.54131	-7.10605402
OAS1	0.085217627	3.21715448	0.687805943	0.492197	0.54146	-7.10623375
CTSG	0.052895026	0.52491089	0.687166458	0.492599	0.54186	-7.10667247
DPEP1	0.058587793	0.68079844	0.687009945	0.492698	0.54194	-7.10677979
TRARG1	0.019215882	0.0881329	0.686636789	0.492933	0.54216	-7.10703555
CLMP	0.085051004	1.58176621	0.686428716	0.493064	0.54227	-7.10717811
NTNG2	-0.055378184	0.6220715	-0.686322522	0.49313	0.54231	-7.10725085
ZNF296	-0.045471613	1.51733131	-0.685745405	0.493494	0.54267	-7.10764595
ACOT9	0.044256931	4.33799931	0.685341258	0.493748	0.54291	-7.10792244
USP9Y	0.135782222	0.93306886	0.685194283	0.493841	0.54298	-7.10802295
ARHGAF	0.053801409	1.51230584	0.684866785	0.494047	0.54317	-7.10824683
PLEKHA	-0.034805138	1.8996381	-0.684464694	0.4943	0.54342	-7.10852157
STRC	0.008942734	0.07766546	0.681717863	0.496033	0.54529	-7.11039406
DLG4	0.075168161	4.16839265	0.681486748	0.496179	0.54541	-7.11055127
ZBTB32	0.032908119	0.61375002	0.681017255	0.496476	0.5457	-7.11087046
CFP	-0.04586097	1.09976431	-0.680787159	0.496621	0.54583	-7.11102682
KCNJ3	-0.038582994	0.14053293	-0.680064095	0.497078	0.54627	-7.11151781
FSTL5	-0.005211322	0.0271555	-0.680052587	0.497085	0.54627	-7.11152562
ZNF492	0.01379172	0.23378444	0.679381397	0.49751	0.5467	-7.11198091
CASP8AI	0.050641093	2.72171044	0.679054157	0.497716	0.54689	-7.11220273
PAX9	-0.113041216	1.71360667	-0.678525855	0.498051	0.54719	-7.11256061
LCTL	0.012981414	0.1555188	0.678522486	0.498053	0.54719	-7.11256289
LHX8	-0.027637957	0.11395463	-0.678166803	0.498278	0.5474	-7.11280368

KIAA082!	-0.030320175	0.89997318	-0.677331544	0.498807	0.54794	-7.11336863
CIDEC	0.031806028	0.13583212	0.67634393	0.499432	0.5486	-7.11403574
LRRC63	0.009583611	0.09914516	0.676125371	0.499571	0.54871	-7.11418324
SOWAH/	0.086214314	2.32203109	0.675792924	0.499782	0.54891	-7.11440751
SIRT1	-0.056928435	4.16881254	-0.675722354	0.499826	0.54892	-7.1144551
NEDD4L	-0.047572477	3.81183088	-0.673796525	0.501048	0.55023	-7.11575197
LGI1	0.004628471	0.02272838	0.673555324	0.501201	0.55036	-7.11591414
PDE6G	0.062480693	1.05289748	0.673423573	0.501285	0.55041	-7.1160027
SETD5	-0.058306744	4.81449775	-0.673388886	0.501307	0.55041	-7.11602601
CELF5	0.034459036	0.31544911	0.672719797	0.501732	0.55084	-7.11647544
MALL	-0.111338784	2.91352005	-0.672639695	0.501783	0.55086	-7.11652922
RAP2C	-0.045497133	4.08875423	-0.671739686	0.502355	0.55145	-7.11713299
TCERG1	-0.048209567	3.68158927	-0.671473936	0.502524	0.5516	-7.11731112
SCN2A	0.004081405	0.03287937	0.671168275	0.502718	0.55178	-7.11751591
NAGA	-0.045268737	5.29102952	-0.67103864	0.5028	0.55183	-7.11760273
IDO1	0.122045069	1.99353192	0.670378989	0.50322	0.55226	-7.11804429
POTEJ	0.003972275	0.02443396	0.669191815	0.503976	0.55305	-7.11883787
CDCA2	0.028535943	0.56682174	0.668706561	0.504285	0.55335	-7.11916184
MYO5B	-0.063421003	4.88778716	-0.668632551	0.504332	0.55337	-7.11921123
ARHGAF	-0.059250999	2.3262807	-0.667815493	0.504852	0.5539	-7.11975614
KIAA152	-0.05634897	6.60598412	-0.66744745	0.505087	0.55413	-7.12000137
UTP20	-0.047580602	2.865615	-0.667256741	0.505209	0.55419	-7.12012839
RBM38	-0.05147852	4.50859146	-0.667255764	0.505209	0.55419	-7.12012904
ZAR1	-0.009582575	0.08124944	-0.666382203	0.505766	0.55476	-7.12071041
THOC6	-0.045680543	5.30009477	-0.666303134	0.505817	0.55478	-7.12076299
SLC23A3	0.029638201	0.67620349	0.665880587	0.506086	0.55504	-7.12104389
SIAH3	-0.010934083	0.03532242	-0.665481397	0.506341	0.55529	-7.12130911
LDB1	-0.065212631	6.42061575	-0.664387641	0.50704	0.55602	-7.12203497
VSIG10L	0.048166125	1.50413114	0.663070206	0.507882	0.5569	-7.12290768
TMEM13	0.077954518	1.22402675	0.661383588	0.508961	0.55805	-7.12402245
C1orf100	0.016742239	0.31929982	0.660257819	0.509682	0.5588	-7.12476494
OR51E1	-0.033771576	0.72570488	-0.659711453	0.510032	0.55915	-7.12512484
SLC25A1	0.039719332	2.445987	0.659076346	0.510439	0.55956	-7.12554282
FAM131/	-0.03886616	2.91709948	-0.658505641	0.510805	0.55993	-7.12591808
NDOR1	-0.043519935	3.7217086	-0.658400963	0.510872	0.55996	-7.12598687
PARP12	0.045547204	4.12152612	0.658343306	0.510909	0.55997	-7.12602476
LRRC37/	-0.053913704	2.48387385	-0.658276624	0.510951	0.55998	-7.12606857
GIT1	-0.031815377	5.47115279	-0.657640092	0.51136	0.56039	-7.12648658
TMEM15	0.064534359	1.45051013	0.657326395	0.511561	0.56057	-7.12669244
FCHO2	0.058058561	5.01741236	0.657275859	0.511594	0.56057	-7.12672559

PHETA2	0.053894432	3.38056761	0.656346438	0.51219	0.56119	-7.12733488
ANO8	0.051935911	2.91914328	0.656277929	0.512234	0.5612	-7.12737976
FSIP2	0.03574258	0.39959383	0.654992054	0.51306	0.56207	-7.12822122
C14orf13	-0.048000401	1.16555819	-0.654471377	0.513395	0.5624	-7.12856147
TRHR	-0.049171686	0.40248727	-0.653986254	0.513707	0.56271	-7.12887825
LMNB1	0.064238531	3.18701309	0.653869096	0.513783	0.56276	-7.12895472
SLC17A5	0.08285855	6.84097878	0.652877566	0.514421	0.5634	-7.12960133
CCDC19	0.01079085	0.1171705	0.652854065	0.514436	0.5634	-7.12961665
VCPIP1	-0.039986132	3.22712144	-0.651379447	0.515385	0.5644	-7.13057646
MSANTD	0.007856428	0.10088938	0.651278113	0.515451	0.56444	-7.13064234
GNL3L	-0.072036187	3.01465358	-0.651199844	0.515501	0.56446	-7.13069321
HOXB3	0.033052433	0.85275793	0.651145679	0.515536	0.56446	-7.13072842
CLDN20	-0.02518481	0.41659368	-0.651093651	0.51557	0.56446	-7.13076223
CCL4L2	-0.104349946	2.07151998	-0.650957958	0.515657	0.56452	-7.1308504
AMT	0.072149794	2.92992292	0.649507387	0.516592	0.5655	-7.13179184
KDM5D	0.155982458	1.14714289	0.649147613	0.516824	0.56572	-7.13202502
TPX2	0.067978577	2.2900738	0.647511988	0.51788	0.56684	-7.13308346
OAS2	-0.073271293	3.4719287	-0.647144099	0.518118	0.56706	-7.13332117
KRT23	0.014408063	0.08392361	0.647056688	0.518175	0.56709	-7.13337762
KIF15	-0.034418033	0.80572179	-0.647000453	0.518211	0.56709	-7.13341394
CHRNA6	0.030146732	0.367484	0.645823378	0.518972	0.56789	-7.1341734
HGS	0.050226313	4.7032193	0.645284614	0.51932	0.56821	-7.13452056
DNER	-0.027791688	0.15638199	-0.645271012	0.519329	0.56821	-7.13452932
NR3C2	-0.062444926	4.24575013	-0.644787411	0.519642	0.56851	-7.13484068
PARD3	0.055198424	4.45108215	0.643144428	0.520706	0.56964	-7.13589676
SUN3	0.008330868	0.11800138	0.643079084	0.520748	0.56965	-7.1359387
TAF4B	0.048773941	1.81059079	0.642657355	0.521021	0.56991	-7.13620932
DDX3Y	0.180305048	1.35439165	0.642219636	0.521305	0.57018	-7.13649001
KMT2E	0.056506065	4.8007626	0.642107386	0.521378	0.57023	-7.13656196
NAT8L	-0.130791496	3.85896135	-0.641064027	0.522054	0.57093	-7.13723014
ZNF727	0.07274373	1.52413618	0.640247334	0.522584	0.57147	-7.1377524
RUNDC3	-0.061821799	2.05274297	-0.639609401	0.522998	0.57189	-7.13815989
FABP3	0.10717724	3.96315319	0.639070833	0.523348	0.57223	-7.13850359
SYT7	0.092466046	2.81107007	0.638769106	0.523544	0.57241	-7.13869602
FAM120	-0.046299345	3.00815821	-0.63817921	0.523927	0.57278	-7.13907197
NOTCH3	0.067006386	5.17296395	0.638109369	0.523972	0.57278	-7.13911646
PLS3	0.058227535	7.41941807	0.638091897	0.523984	0.57278	-7.13912759
NEK7	-0.053705514	4.62442403	-0.637807719	0.524168	0.57295	-7.13930855
ADARB2	-0.034471117	0.15148549	-0.63537284	0.525752	0.57464	-7.14085577
SEMA3E	-0.097355788	1.46864892	-0.635148993	0.525898	0.57476	-7.14099772

RASD1	0.116650326	6.70415284	0.634615853	0.526245	0.57508	-7.14133559
ULBP2	0.081201043	2.97593965	0.634595807	0.526258	0.57508	-7.14134829
RAD54L2	-0.040138718	3.11118125	-0.633889656	0.526718	0.57552	-7.14179535
EVC	0.074680376	3.4655444	0.633872914	0.526729	0.57552	-7.14180595
CCKBR	0.052955802	0.68931303	0.633201926	0.527167	0.57596	-7.14223028
SPDYE2	0.011435714	0.15720233	0.633111726	0.527225	0.57599	-7.14228729
TEX45	-0.013166054	0.11915715	-0.632790564	0.527435	0.57618	-7.14249021
TTLL2	-0.003932192	0.02357708	-0.632639943	0.527533	0.57623	-7.14258534
RAB11FI	-0.044884824	4.27483537	-0.632622805	0.527544	0.57623	-7.14259616
WASF2	-0.046427652	6.32627997	-0.632057409	0.527913	0.57659	-7.14295304
ZNF337	0.042842756	1.49120395	0.631411044	0.528335	0.57702	-7.14336065
IER5	0.055253348	4.64590024	0.63073759	0.528774	0.57746	-7.14378489
GLUD2	-0.045762707	1.3530046	-0.630561658	0.528889	0.57753	-7.14389564
SRPX	-0.088485123	3.00066737	-0.630541953	0.528902	0.57753	-7.14390805
CCDC15	0.008817072	0.18353353	0.62934559	0.529684	0.57834	-7.14466035
GHRHR	-0.009219277	0.04486234	-0.62878832	0.530048	0.5787	-7.14501029
TM6SF2	-0.03078228	0.30931377	-0.628357766	0.530329	0.57897	-7.14528045
IPO7	-0.056014787	6.28639081	-0.627422308	0.530941	0.5796	-7.14586678
DEPDC1	-0.023912906	0.43965494	-0.627212901	0.531078	0.57972	-7.14599791
LZTS1	0.072125627	2.44794447	0.626622285	0.531465	0.58006	-7.14636753
TPRG1	0.060515878	0.88482519	0.626620742	0.531466	0.58006	-7.14636849
FZD6	0.06865375	5.55783254	0.626519152	0.531532	0.5801	-7.14643203
KIAA201	-0.004985801	0.02347236	-0.626449686	0.531578	0.58011	-7.14647548
STK31	-0.011230241	0.15716788	-0.626083359	0.531818	0.58034	-7.14670449
KCNH2	-0.032069933	0.22482145	-0.62587289	0.531955	0.58045	-7.14683601
CAPN3	0.056339047	1.09877424	0.625803673	0.532001	0.58046	-7.14687925
SEC16A	-0.043570432	5.23473898	-0.624289715	0.532993	0.58151	-7.14782389
TRIP10	0.04662144	5.81805902	0.623959597	0.533209	0.5817	-7.14802957
RNF19B	-0.064714816	5.17896588	-0.623813516	0.533305	0.58177	-7.14812054
WNK1	0.052058669	4.85178172	0.623678433	0.533394	0.58183	-7.14820465
OTULIN	-0.031424119	2.6807339	-0.623419851	0.533563	0.58198	-7.14836561
RBM3	-0.03920996	7.17238684	-0.623215202	0.533697	0.58209	-7.14849295
GPRC5B	0.069283766	5.29722032	0.622917472	0.533893	0.58223	-7.14867813
RBBP8	0.055888908	4.5305066	0.622911444	0.533897	0.58223	-7.14868188
GPR132	-0.083769408	2.15215907	-0.622238933	0.534338	0.58267	-7.14909984
VWC2	0.015950742	0.04096309	0.622148275	0.534398	0.5827	-7.14915615
MAP7D1	0.044847493	6.05680623	0.621844141	0.534597	0.58288	-7.14934499
C10orf67	-0.041287536	0.98986424	-0.620964213	0.535175	0.58347	-7.14989084
CLNK	0.025802612	0.34245864	0.620488481	0.535488	0.58378	-7.15018563
TAGLN3	0.087731668	0.81303627	0.62043472	0.535523	0.58378	-7.15021893

PKM	0.051264419	8.03809727	0.620263244	0.535636	0.58386	-7.15032512
SLC12A7	-0.045893062	4.97827532	-0.619982578	0.53582	0.58403	-7.15049887
MLLT6	-0.049293282	5.62822815	-0.618942194	0.536504	0.58473	-7.15114223
NPPA	-0.019997207	0.34865396	-0.618897957	0.536533	0.58473	-7.15116957
TAT	-0.009343862	0.11146664	-0.618492469	0.5368	0.58498	-7.15142001
CCDC18	-0.061784452	1.2606304	-0.618429282	0.536842	0.58498	-7.15145902
NDE1	0.038017797	2.39039865	0.61838441	0.536871	0.58498	-7.15148672
FANCM	-0.028123008	1.29229106	-0.61791121	0.537183	0.58529	-7.15177873
CACNA1	-0.04097764	0.35739687	-0.617527235	0.537435	0.58552	-7.15201551
GAS6	0.049054606	7.05636582	0.617330091	0.537565	0.58563	-7.15213702
CYBC1	0.039438659	4.62558969	0.617242687	0.537623	0.58565	-7.15219088
ZNF860	0.063749141	1.68937123	0.617072756	0.537735	0.58574	-7.15229558
AMOTL1	-0.057249996	3.83841398	-0.616911894	0.537841	0.58582	-7.15239466
PRR22	0.052351577	2.36521054	0.616051154	0.538408	0.5864	-7.15292438
LRCOL1	0.065368454	0.83201376	0.615837015	0.538549	0.58651	-7.15305606
EZH1	-0.040508561	4.77771936	-0.615156329	0.538997	0.58696	-7.1534743
SRL	-0.094739154	3.81661795	-0.614990775	0.539107	0.58704	-7.15357596
TAX1BP3	0.046806242	5.93845312	0.614759008	0.539259	0.58715	-7.15371822
CRIM1	-0.070583345	5.20905621	-0.614737216	0.539274	0.58715	-7.1537316
DUSP27	-0.017418179	0.17476275	-0.614610623	0.539357	0.5872	-7.15380928
BTNL2	-0.031441054	0.2091074	-0.614455341	0.53946	0.58728	-7.15390454
IL12RB2	-0.035887472	0.2785222	-0.614202048	0.539627	0.58742	-7.15405988
GAGE10	-0.027999768	0.36356466	-0.613888233	0.539834	0.58761	-7.15425225
BMERB1	0.072540983	4.84784266	0.61362663	0.540006	0.58776	-7.15441254
LILRA1	0.022809453	0.52765387	0.612838403	0.540527	0.58829	-7.15489509
SHCBP1	0.040668625	0.99711782	0.612778983	0.540566	0.58829	-7.15493144
ZNF525	-0.033344554	1.95283435	-0.612585269	0.540694	0.58839	-7.15504992
NMNAT2	-0.11045468	2.87311016	-0.612542775	0.540722	0.58839	-7.15507591
FAM149A	-0.030885999	1.57900272	-0.61242972	0.540797	0.58843	-7.15514504
CEP97	-0.037007332	2.1252076	-0.61190158	0.541145	0.58877	-7.1554678
SLC22A1	-0.006110156	0.05650631	-0.610694877	0.541943	0.58957	-7.15620422
BBS9	-0.043432381	3.88068991	-0.610653902	0.54197	0.58957	-7.1562292
PLS1	-0.063310294	3.04327275	-0.610634747	0.541983	0.58957	-7.15624087
FAM177B	0.021326435	0.34927228	0.610223342	0.542255	0.58983	-7.15649158
CCDC60	0.009747601	0.11947432	0.609506963	0.542729	0.59031	-7.15692775
HUNK	-0.044660328	0.70867418	-0.609189495	0.542939	0.59049	-7.15712087
MGRN1	0.045745229	5.18222627	0.609142882	0.542969	0.59049	-7.15714922
CCDC6	-0.045501135	5.01304013	-0.608612882	0.54332	0.59084	-7.15747139
PHKA1	-0.052702717	3.8442846	-0.608416104	0.543451	0.59094	-7.15759093
LCN10	0.095562155	1.21898644	0.608175552	0.54361	0.59107	-7.15773701

ZNF623	-0.043360651	3.62873234	-0.607197578	0.544258	0.59174	-7.15833032
APOE	0.157281588	8.32898264	0.60715118	0.544288	0.59174	-7.15835845
REEP3	-0.054242362	5.46154405	-0.606724385	0.544571	0.59201	-7.15861705
RTP5	0.016926912	0.10134544	0.606465375	0.544743	0.59215	-7.15877391
GTPBP1	0.043006827	4.7476478	0.605839834	0.545157	0.59257	-7.15915245
ZBTB11	-0.043166113	3.42532151	-0.605016859	0.545703	0.59312	-7.15964988
TUBB2A	-0.065617788	2.77292052	-0.604636612	0.545956	0.59334	-7.15987948
USP34	0.077068539	3.42161264	0.604606848	0.545975	0.59334	-7.15989745
STAT6	0.04296696	6.33221934	0.604342324	0.546151	0.59349	-7.16005708
POF1B	-0.056416525	0.5142715	-0.604153684	0.546276	0.59359	-7.16017088
ADAMTS	-0.107308291	3.85680798	-0.603828526	0.546492	0.59379	-7.16036695
LTB4R	-0.060007962	2.23977045	-0.602995689	0.547045	0.59435	-7.16086867
CCR3	0.047714894	0.66963531	0.602886891	0.547117	0.59439	-7.16093416
PTPRD	0.096020155	1.7090898	0.601102897	0.548303	0.59564	-7.16200635
AGT	0.096964418	3.01587528	0.600746387	0.54854	0.59584	-7.16222024
ABCA9	0.030813379	0.466113	0.600728954	0.548552	0.59584	-7.16223069
KRTAP5-	-0.008056821	0.07681415	-0.600472911	0.548722	0.59598	-7.16238422
NPIPA1	-0.036213108	1.21624485	-0.600063021	0.548995	0.59622	-7.16262986
CBX6	-0.042685248	5.83561602	-0.60004364	0.549007	0.59622	-7.16264147
FSTL4	0.056907097	0.87950404	0.599384608	0.549446	0.59666	-7.16303606
E2F8	0.027678323	0.50380578	0.599218419	0.549557	0.59674	-7.1631355
SLC7A14	0.005944916	0.01526297	0.599013191	0.549693	0.59685	-7.16325825
MYO1B	-0.062028874	4.12478521	-0.598380809	0.550114	0.59727	-7.16363624
ERV3-1	0.062456529	3.0851675	0.597859442	0.550462	0.59759	-7.16394758
PLAAT2	-0.046339493	1.2017276	-0.597829811	0.550482	0.59759	-7.16396526
CNOT6L	-0.043362035	3.92769975	-0.597771628	0.55052	0.59759	-7.16399999
C12orf50	-0.00324776	0.01620184	-0.597397296	0.55077	0.59782	-7.16422332
ACSM4	-0.005140635	0.04371138	-0.597331487	0.550814	0.59783	-7.16426257
ATAD2B	-0.03985889	2.28517573	-0.597260566	0.550861	0.59785	-7.16430486
HES5	-0.028973802	0.48441738	-0.596986377	0.551044	0.59801	-7.16446832
PCDHGE	-0.049140689	0.89186198	-0.596121503	0.55162	0.59859	-7.16498343
PARVA	0.04244267	4.4287762	0.595680799	0.551914	0.59888	-7.16524563
UTP25	0.041786612	2.88570639	0.594192486	0.552908	0.59991	-7.16612966
KEL	-0.023101738	0.33613848	-0.594048705	0.553004	0.59998	-7.16621494
ARFGEF	-0.0636693	5.22497613	-0.592399256	0.554106	0.60114	-7.16719187
CLEC18A	-0.007441096	0.07912157	-0.592090649	0.554312	0.60132	-7.16737435
GGH	-0.053127487	2.36687322	-0.59203978	0.554346	0.60132	-7.16740442
AGAP3	-0.03488668	4.27674042	-0.591770195	0.554526	0.60148	-7.16756373
GPR143	-0.052773127	2.11476563	-0.591651762	0.554605	0.60153	-7.1676337
SMCR8	-0.05369117	3.62861	-0.591145381	0.554944	0.60185	-7.1679327

ZGRF1	-0.027315314	0.96204776	-0.590820458	0.555162	0.60205	-7.16812442
CDYL	-0.047035491	3.99951571	-0.59046855	0.555397	0.60227	-7.16833194
IGLON5	0.023098663	0.12743501	0.590283726	0.555521	0.60236	-7.16844088
CPNE5	0.063797857	2.39032616	0.590116059	0.555633	0.60245	-7.16853968
RTEL1	-0.019456529	0.54258011	-0.589180821	0.556259	0.60305	-7.16909027
AGFG1	-0.042962702	4.28999116	-0.589180089	0.55626	0.60305	-7.1690907
RNF149	-0.03608229	4.71462701	-0.589089381	0.55632	0.60308	-7.16914406
GPR22	-0.004233781	0.03489497	-0.588713485	0.556572	0.60331	-7.16936507
PARD3B	-0.049045039	2.92705528	-0.587520023	0.557372	0.60414	-7.17006586
SERTM2	0.020368213	0.10493192	0.587186771	0.557595	0.60434	-7.17026129
PP2D1	-0.016240768	0.42357513	-0.586987617	0.557729	0.60445	-7.17037803
VPS13D	0.051907376	4.48191128	0.5863742	0.55814	0.60486	-7.17073735
GXYLT2	0.064911358	4.07145953	0.586275955	0.558206	0.60489	-7.17079486
IGF2BP1	-0.025512129	0.07574354	-0.585476975	0.558742	0.60543	-7.17126223
RAD51B	0.025370163	1.58903825	0.58513785	0.55897	0.60564	-7.17146041
ITLN1	0.027272188	0.39214223	0.584797525	0.559198	0.60585	-7.17165918
ISLR2	-0.037171697	0.8046644	-0.584665672	0.559287	0.60591	-7.17173616
PCK1	-0.01432035	0.09729314	-0.58329343	0.560209	0.60687	-7.17253627
PLEKHG	-0.0497595	2.52816632	-0.582726706	0.56059	0.60724	-7.17286617
WBP2	0.033810441	6.91443769	0.582350009	0.560843	0.60748	-7.17308527
SLC12A1	0.006775305	0.03141243	0.582196885	0.560946	0.60755	-7.17317429
SSX2IP	-0.039530557	2.78993089	-0.581392061	0.561487	0.6081	-7.17364181
SCT	-0.075179288	1.31248008	-0.580850868	0.561852	0.60845	-7.17395582
LRRC37I	0.033589432	2.1024316	0.580625497	0.562003	0.60858	-7.1740865
MAGEA8	-0.005177965	0.04883102	-0.580200111	0.56229	0.60885	-7.17433302
SF3B1	-0.046601585	6.52174253	-0.579556163	0.562723	0.60928	-7.17470586
DIAPH2	0.047323978	2.11967112	0.579279119	0.56291	0.60944	-7.17486614
SLC12A2	-0.058398632	3.7551523	-0.578526845	0.563417	0.60995	-7.17530096
ELAVL3	-0.037097267	0.15140553	-0.578020068	0.563758	0.61028	-7.17559357
SIN3B	0.03883288	4.25397582	0.577335494	0.56422	0.61074	-7.17598843
PIM3	-0.044886899	6.07138064	-0.576866183	0.564536	0.61105	-7.17625886
FOXI2	-0.067252139	0.89349344	-0.576160616	0.565012	0.61152	-7.17666501
CRYM	-0.018813147	0.1592865	-0.57598472	0.565131	0.61161	-7.17676618
CENPU	0.050875883	2.40705962	0.575765312	0.565279	0.61173	-7.17689234
ZNF850	-0.019841284	0.78242672	-0.57512339	0.565713	0.61216	-7.17726117
FLACC1	-0.035019147	0.98148475	-0.574900656	0.565863	0.61229	-7.17738905
RGS10	0.065590218	5.29706842	0.574571203	0.566085	0.61249	-7.17757811
ANKFY1	0.054981881	4.16084285	0.574226091	0.566319	0.6127	-7.17777604
C9orf72	0.04721938	3.41016059	0.574165155	0.56636	0.61271	-7.17781098
KMT2B	-0.039273586	3.99926372	-0.573600615	0.566741	0.61308	-7.17813447

AMH	0.042972442	0.63872263	0.573358872	0.566905	0.61322	-7.1782729
KCNV1	0.030531715	0.75685006	0.57313335	0.567057	0.61334	-7.17840198
TXNDC5	-0.060397797	1.81288171	-0.571399543	0.56823	0.61457	-7.17939269
ALDH8A1	0.013591876	0.26335869	0.57062055	0.568757	0.6151	-7.17983684
TULP2	-0.009476883	0.14032338	-0.570062049	0.569135	0.61547	-7.1801549
RASAL1	-0.061356921	4.82842616	-0.569513705	0.569507	0.61584	-7.18046687
NLRP2	-0.124153738	2.07539584	-0.569252526	0.569683	0.61599	-7.18061536
SLC25A2	-0.071079357	3.19713459	-0.569017594	0.569843	0.61612	-7.18074887
GPCPD1	0.045897314	4.81845825	0.568732961	0.570036	0.61629	-7.18091055
ASMT	-0.015759883	0.25346758	-0.568553538	0.570157	0.61638	-7.18101243
HCFC1R	0.046503671	7.07884315	0.567754729	0.570699	0.61693	-7.1814656
PSMB10	0.050291797	5.30011977	0.56754588	0.57084	0.61704	-7.18158398
PABPC3	-0.020487514	0.44733252	-0.567365415	0.570963	0.6171	-7.18168623
MASP2	-0.030651853	0.72282206	-0.567354688	0.57097	0.6171	-7.18169231
PCGF3	-0.041564624	4.2782161	-0.566593827	0.571486	0.61762	-7.18212306
DPEP2	-0.041089202	1.59528002	-0.56625183	0.571718	0.61783	-7.18231649
ATCAY	-0.013802951	0.03125611	-0.565890155	0.571964	0.61805	-7.18252092
CIDEA	0.018663405	0.08658808	0.565855647	0.571987	0.61805	-7.18254042
TMEM27	-0.018913803	0.25357356	-0.565650798	0.572126	0.61816	-7.18265614
EEA1	-0.046120569	3.26361703	-0.56544943	0.572263	0.61827	-7.18276986
STOM	0.052732994	7.2328488	0.565082257	0.572512	0.6185	-7.1829771
NBPF20	-0.010070759	0.38994902	-0.564913563	0.572627	0.61858	-7.18307227
R3HDM4	0.031878044	6.14710845	0.564782779	0.572716	0.61864	-7.18314604
PCDHGA	-0.047365891	1.29054429	-0.56423806	0.573086	0.619	-7.18345308
POLN	-0.015703271	0.58874403	-0.563808225	0.573378	0.61927	-7.18369517
PRRG4	-0.055635509	4.22576664	-0.563458037	0.573616	0.61949	-7.18389225
MYBPC1	0.035684683	0.14675084	0.562589369	0.574206	0.62002	-7.18438062
NTM	-0.088411948	1.87594984	-0.562565511	0.574223	0.62002	-7.18439402
ISYNA1	0.070531906	5.67646186	0.562564263	0.574224	0.62002	-7.18439472
NUP210	-0.041934041	3.58658872	-0.562516724	0.574256	0.62002	-7.18442143
PCDHA8	-0.007202474	0.09763599	-0.562227849	0.574452	0.6202	-7.18458364
PPT1	-0.039003206	7.05583854	-0.562119403	0.574526	0.62024	-7.18464452
SEC14L5	-0.021840861	0.26587621	-0.561828246	0.574724	0.62041	-7.18480791
TENM4	-0.073681561	2.34756496	-0.561280667	0.575097	0.62077	-7.18511495
FKBP5	0.074655637	4.35187938	0.560861905	0.575382	0.62104	-7.18534957
RAD23B	0.057267463	7.3358253	0.560369807	0.575717	0.62136	-7.18562504
SLC49A3	0.064276468	3.75242704	0.559382771	0.576389	0.62205	-7.18617686
NFIC	-0.034698545	6.14699218	-0.559009572	0.576643	0.62228	-7.18638525
LAMA5	-0.05505488	5.67232871	-0.558825726	0.576769	0.62238	-7.18648786
CCDC19	0.00735602	0.05502361	0.558776301	0.576802	0.62238	-7.18651544

SCN2B	-0.076286768	2.04118081	-0.55866618	0.576877	0.62242	-7.18657688
SLC39A5	-0.015951259	0.36783808	-0.557918623	0.577387	0.62293	-7.18699363
ELMSAN	-0.045394266	3.29356659	-0.557326945	0.577791	0.62332	-7.18732309
PRR19	-0.024147794	1.12241767	-0.556808783	0.578144	0.62367	-7.18761133
GALNT8	-0.006396319	0.08065145	-0.556721578	0.578204	0.62369	-7.18765981
F3	-0.097672429	5.12283119	-0.556014252	0.578686	0.62417	-7.18805278
LDHA	0.047623641	7.21167934	0.555457926	0.579066	0.62454	-7.18836151
STMN1	0.053029418	5.07455872	0.55483619	0.579491	0.62496	-7.18870617
CAPG	0.070983302	8.16038964	0.554255062	0.579888	0.62535	-7.18902797
FAM72B	0.009856076	0.16507162	0.554126142	0.579976	0.6254	-7.18909932
ADARB1	-0.038769548	2.68587083	-0.553115587	0.580667	0.62611	-7.18965799
SCG5	0.126757443	5.31651705	0.552600977	0.581018	0.62645	-7.18994209
DNAJC6	0.04181098	1.36189375	0.552308411	0.581219	0.6266	-7.19010349
CENPW	0.043037029	2.67609354	0.552283433	0.581236	0.6266	-7.19011727
AVL9	0.038318554	3.56690395	0.552150197	0.581327	0.62665	-7.19019074
SMIM6	0.024124235	0.25631668	0.552115846	0.58135	0.62665	-7.19020968
HCN1	-0.004992881	0.02976553	-0.55178343	0.581578	0.62685	-7.19039289
GAN	-0.026488702	1.47801253	-0.551268232	0.58193	0.62719	-7.19067664
PTN	-0.077838001	3.27989921	-0.551187784	0.581985	0.62721	-7.19072092
LGALS7	0.04309156	0.55232485	0.551032988	0.582091	0.62725	-7.19080611
LGALS7B	0.04309156	0.55232485	0.551032988	0.582091	0.62725	-7.19080611
TCP11	0.007690486	0.11967479	0.55037957	0.582539	0.62769	-7.19116544
CDH24	0.043445488	3.23710634	0.550150163	0.582696	0.62782	-7.1912915
CEP295	0.036793115	2.13953871	0.54956579	0.583096	0.62821	-7.19161237
CHST8	-0.01668427	0.28125885	-0.548595932	0.58376	0.62888	-7.19214416
ARMC4	0.040098596	0.74968314	0.548548	0.583793	0.62888	-7.19217041
ELL	-0.032699016	3.62211575	-0.54711739	0.584774	0.6299	-7.19295305
ZNF221	0.028479532	0.97294929	0.546966284	0.584878	0.62997	-7.1930356
CRHR2	0.045280297	0.90097449	0.546162161	0.58543	0.6305	-7.19347449
CDC42BI	-0.047155402	5.57124081	-0.546141238	0.585444	0.6305	-7.19348591
ARID3C	-0.015758938	0.18225879	-0.544635892	0.586478	0.63157	-7.19430577
RPS4Y1	0.24293697	2.27319124	0.543687131	0.58713	0.63223	-7.19482133
SH3GLB:	-0.03738667	5.52394705	-0.543147646	0.5875	0.63259	-7.19511409
TRIM38	-0.03714604	3.76336029	-0.542537834	0.58792	0.633	-7.19544467
IFNGR1	-0.043449538	6.734544	-0.54195943	0.588317	0.63339	-7.19575788
ATM	0.041722561	2.89738129	0.541900243	0.588358	0.6334	-7.19578991
GNPTAB	-0.036247645	4.14074425	-0.541571698	0.588584	0.6336	-7.19596765
ITGAV	0.067628875	6.40123585	0.541481085	0.588647	0.63363	-7.19601665
RACGAF	0.040634162	2.30968778	0.540764008	0.58914	0.63408	-7.19640415
CSRNP2	0.040411954	4.36822907	0.540760388	0.589143	0.63408	-7.19640611

CGB7	-0.01236475	0.14850458	-0.540668456	0.589206	0.63411	-7.19645575
SPDYE6	-0.016549912	0.50297377	-0.540171048	0.589548	0.63443	-7.19672419
BCAS1	-0.008786083	0.13323682	-0.539999919	0.589666	0.63452	-7.19681649
FNDC9	0.004785216	0.03575624	0.539184658	0.590228	0.63509	-7.1972558
CAPN15	-0.033027711	3.5537366	-0.537915013	0.591103	0.63599	-7.19793864
TLX2	-0.007877106	0.08743532	-0.53643367	0.592124	0.63705	-7.1987333
RHOG	0.032638571	6.1610556	0.53629702	0.592219	0.63707	-7.1988065
LBR	-0.040122021	3.57023267	-0.536294096	0.592221	0.63707	-7.19880806
LEPROT	-0.043846894	6.18224033	-0.535498413	0.59277	0.63758	-7.19923389
ARHGEF	-0.046548067	2.49288481	-0.535491265	0.592775	0.63758	-7.19923772
CEP170	0.048687657	3.31882708	0.534600098	0.59339	0.6382	-7.19971389
ZGLP1	-0.053864391	1.98811846	-0.534398561	0.593529	0.63831	-7.19982147
KRTAP5-	0.03234143	0.57108765	0.533948975	0.59384	0.63861	-7.2000613
SCML1	0.03994176	2.07837377	0.53380602	0.593939	0.63867	-7.20013752
PRR5L	-0.037966737	1.54899233	-0.533693935	0.594016	0.63872	-7.20019727
ERRFI1	-0.081560093	6.24352841	-0.533514176	0.59414	0.63881	-7.20029306
TROAP	0.034245434	0.89856279	0.533408758	0.594213	0.63885	-7.20034922
RHD	-0.026601401	0.73380025	-0.533102553	0.594425	0.639	-7.20051229
RBM46	-0.022000145	0.12292883	-0.533091309	0.594432	0.639	-7.20051827
MUC6	0.007186703	0.06167174	0.532418208	0.594898	0.63945	-7.20087639
SYNGAP	0.041439667	2.44110966	0.532383316	0.594922	0.63945	-7.20089495
BTG1	-0.03882564	5.63323624	-0.532167835	0.595071	0.63957	-7.20100949
TGM4	0.021978541	0.10364906	0.532066084	0.595141	0.6396	-7.20106356
POLR2J2	-0.017080044	0.21396441	-0.53082091	0.596003	0.64049	-7.20172442
KCNT1	-0.021957952	0.07671535	-0.530650287	0.596121	0.64057	-7.20181486
ZNF215	0.03746284	1.52731613	0.5297903	0.596716	0.64117	-7.20227024
ADGRE5	0.063451143	5.35853092	0.529211556	0.597117	0.64156	-7.20257628
SPATA21	-0.003208736	0.02067823	-0.528971656	0.597283	0.6417	-7.20270304
CILP2	0.063261714	2.5269519	0.528722435	0.597456	0.64184	-7.20283467
CD3EAP	0.025972072	2.11888437	0.527131293	0.598559	0.64299	-7.20367357
DNMT1	0.042955373	4.07221009	0.526242791	0.599175	0.64361	-7.20414092
UNC5C	0.069083986	3.95092577	0.525826132	0.599464	0.64388	-7.20435981
PNLDC1	0.043843585	0.2041495	0.525766781	0.599505	0.64388	-7.20439098
TLX1	0.017894616	0.07738098	0.524916517	0.600095	0.64448	-7.20483708
C1orf115	-0.075503533	6.51936085	-0.524629266	0.600295	0.64465	-7.20498762
SMARCC	0.054885581	4.95211325	0.524139943	0.600634	0.64497	-7.20524388
FAM24B	0.041218665	1.87771787	0.52262549	0.601686	0.64606	-7.2060355
AURKC	0.046365609	1.59488093	0.522264297	0.601938	0.64627	-7.20622396
ATP1B3	-0.04507009	6.44108603	-0.52224404	0.601952	0.64627	-7.20623452
PLEKHM	0.034756167	3.86348429	0.521984863	0.602132	0.64642	-7.20636967

MS4A12	-0.036369681	0.52050066	-0.52181166	0.602252	0.64651	-7.20645995
POTEE	0.002868864	0.01943693	0.52147409	0.602487	0.64672	-7.20663581
MALT1	0.033595472	3.21369193	0.520825472	0.602938	0.64716	-7.2069734
MAST4	0.037379717	3.02825879	0.520573176	0.603114	0.64731	-7.20710461
FUT11	-0.032978563	3.66703172	-0.520465881	0.603188	0.64735	-7.20716038
XKR5	0.011560333	0.20301921	0.520409006	0.603228	0.64735	-7.20718995
SVEP1	0.045076476	0.84161871	0.520117552	0.603431	0.64753	-7.20734138
CELA2B	-0.017760619	0.38169209	-0.518883429	0.60429	0.64841	-7.20798169
LPCAT4	0.038216101	3.88858539	0.518442502	0.604597	0.64869	-7.20821009
TFAP4	0.03379557	2.50265541	0.518156797	0.604796	0.64887	-7.20835799
SOX5	-0.024577812	0.60677354	-0.517853089	0.605008	0.64905	-7.20851511
BLNK	-0.049013375	4.51591372	-0.517682495	0.605126	0.64914	-7.20860332
CMKLR1	0.056612768	2.3971955	0.51744792	0.60529	0.64927	-7.20872458
ARHGAF	-0.037783848	3.56220006	-0.515704065	0.606506	0.65051	-7.20962427
OTUD7B	0.03760274	3.48011767	0.515683217	0.60652	0.65051	-7.20963501
KCTD13	-0.028150163	3.25606104	-0.515126079	0.606909	0.65089	-7.2099218
FAM89A	0.041565759	3.1990515	0.514781957	0.607149	0.6511	-7.21009878
HSPB2	-0.043147093	1.88054175	-0.513060601	0.608351	0.65235	-7.21098231
ACPP	-0.049200001	2.08975777	-0.5126294	0.608652	0.65263	-7.21120318
SLC1A6	-0.003304822	0.01911584	-0.512256751	0.608913	0.65287	-7.2113939
CDHR1	0.02576546	0.24262622	0.51213883	0.608995	0.65289	-7.21145422
CATSPE	-0.007851298	0.11248172	-0.512127029	0.609003	0.65289	-7.21146026
SLC25A4	-0.032733103	0.49415795	-0.511580212	0.609385	0.65325	-7.2117398
ADAM32	-0.00584991	0.13072652	-0.511220471	0.609637	0.65348	-7.21192354
CHGA	0.019224591	0.07767144	0.51031144	0.610273	0.65412	-7.21238727
ONECUT	0.002645545	0.01266856	0.509858223	0.61059	0.65442	-7.21261816
FOXL2	-0.007018842	0.06845305	-0.509757126	0.61066	0.65444	-7.21266964
TCOF1	-0.023680096	3.75536373	-0.509728696	0.61068	0.65444	-7.21268411
SHH	-0.032902737	0.18950115	-0.509550916	0.610805	0.65453	-7.2127746
C5	0.030875957	1.7897481	0.509342038	0.610951	0.65462	-7.21288088
POU3F1	-0.03969072	0.53730782	-0.509313003	0.610971	0.65462	-7.21289565
NPHS1	0.013899935	0.06661248	0.509175729	0.611067	0.65468	-7.21296547
SLC2A1	0.048632097	4.6491945	0.508062465	0.611847	0.65548	-7.213531
SLC25A2	-0.009135194	0.11576769	-0.507986211	0.6119	0.65549	-7.21356969
CENPM	-0.046459075	2.20913337	-0.507555688	0.612202	0.65578	-7.21378803
CHRNG	0.005161874	0.04990222	0.507116332	0.612509	0.65606	-7.21401066
CNTF	-0.029599512	0.62442008	-0.506760722	0.612759	0.65629	-7.21419071
PIDD1	-0.041336323	3.36300786	-0.50619868	0.613152	0.65667	-7.21447502
DDC	-0.007558173	0.06754367	-0.505889589	0.613369	0.65686	-7.21463124
C4orf54	-0.008748105	0.03750522	-0.505809254	0.613425	0.65688	-7.21467183

PACS2	-0.025189316	4.21967444	-0.505478706	0.613657	0.65709	-7.21483877
CYP2C9	0.005314937	0.04276941	0.505334304	0.613758	0.65715	-7.21491166
ANXA13	0.005779	0.03155479	0.505175757	0.61387	0.65723	-7.21499167
CABP7	-0.03778174	0.41527723	-0.504919342	0.614049	0.65738	-7.21512101
ZCCHC2	-0.033113985	2.74063069	-0.504432987	0.614391	0.65771	-7.21536616
KRT14	-0.072552751	1.05824488	-0.503692778	0.61491	0.65822	-7.21573882
CCND2	-0.058293059	7.13399173	-0.503252213	0.615219	0.65851	-7.21596037
SPECC1	-0.003078243	0.03261959	-0.503058286	0.615355	0.65861	-7.21605782
MDM4	-0.037622341	3.42829337	-0.502649166	0.615643	0.65885	-7.2162633
GK5	0.044703572	2.78540221	0.502626237	0.615659	0.65885	-7.21627481
CD79B	-0.067676739	2.55391892	-0.502141456	0.615999	0.65918	-7.21651806
GAD1	0.014520493	0.10638612	0.501142657	0.616701	0.65989	-7.2170185
NAP1L1	-0.03231273	5.95218881	-0.50024913	0.617329	0.66052	-7.21746535
HELB	-0.021405466	1.04519065	-0.500012568	0.617495	0.66065	-7.21758352
PDGFC	-0.053246696	4.70303344	-0.499334751	0.617972	0.66112	-7.2179218
LHX2	-0.066278218	0.30049112	-0.499136259	0.618112	0.66122	-7.21802077
PLEKHM	-0.050432602	1.652652	-0.499063228	0.618163	0.66122	-7.21805718
DIP2A	-0.031326193	3.00020846	-0.499043562	0.618177	0.66122	-7.21806698
FNIP2	0.041817733	3.41113666	0.497497832	0.619265	0.66231	-7.21883626
GSK3B	-0.033482356	4.78787759	-0.497485393	0.619273	0.66231	-7.21884244
HECTD2	0.034674247	2.11503884	0.497288326	0.619412	0.66241	-7.21894034
APBB3	-0.041994035	3.80923776	-0.496845239	0.619724	0.6627	-7.21916032
ADAM15	-0.035970807	5.64254319	-0.496706021	0.619822	0.66277	-7.2192294
TMEM14	-0.037390655	3.94505643	-0.496120407	0.620235	0.66317	-7.21951976
HOXC4	0.034002424	0.54268444	0.495872168	0.62041	0.66331	-7.21964274
CD151	0.047868942	7.81805071	0.495005727	0.62102	0.6639	-7.2200715
HAMP	-0.035471819	0.6799192	-0.494979921	0.621038	0.6639	-7.22008426
BNC2	0.057848643	1.1644338	0.493659493	0.62197	0.66484	-7.2207362
TNFRSF	0.026603882	1.01364861	0.493621223	0.621997	0.66484	-7.22075507
ENAM	0.004552868	0.03903922	0.493457109	0.622112	0.6649	-7.22083598
DNAH14	0.019270466	1.07007635	0.493424344	0.622135	0.6649	-7.22085212
PRC1	0.036084576	2.09392316	0.492389677	0.622866	0.66564	-7.22136152
MAS1	0.002498315	0.00901985	0.492276189	0.622946	0.66569	-7.22141733
HSPA4L	-0.043216261	1.54494372	-0.491711691	0.623344	0.66607	-7.22169473
CDK17	-0.034147516	3.62587161	-0.491620667	0.623408	0.6661	-7.22173943
SLF1	0.021228701	1.72780141	0.491378307	0.62358	0.66624	-7.22185842
GBP1	0.067357213	4.76274321	0.491071182	0.623796	0.66643	-7.22200911
LARGE1	0.037524828	2.60968696	0.490868474	0.62394	0.66654	-7.22210851
ETV1	0.067283056	3.76997142	0.490679566	0.624073	0.66664	-7.22220112
HGSNAT	0.036939227	5.51223466	0.49024599	0.624379	0.66692	-7.22241352

NKTR	0.049257532	3.73250936	0.48970864	0.624759	0.66729	-7.22267651
PIWIL4	0.020735709	0.98419863	0.489443426	0.624947	0.66745	-7.2228062
ANKRD1	-0.034348765	3.7673578	-0.488915318	0.62532	0.6678	-7.22306424
TBC1D2(	-0.002952377	0.02049513	-0.488789664	0.625409	0.66786	-7.22312559
NAPRT	-0.054740584	5.25400449	-0.488378396	0.6257	0.66812	-7.2233263
OXR1	-0.048896773	4.12541458	-0.488282088	0.625768	0.66816	-7.22337328
ZNF587	0.033627838	2.67865537	0.486846263	0.626783	0.6692	-7.22407253
ATP1A4	-0.006021867	0.06078639	-0.48651588	0.627017	0.66941	-7.22423313
CD8B2	0.032437903	0.20480282	0.486053286	0.627345	0.66971	-7.22445783
LGALS9I	0.005704684	0.03460166	0.485974813	0.6274	0.66973	-7.22449592
TMEM24	0.077721587	1.86144666	0.485331237	0.627856	0.67017	-7.22480812
VWC2L	-0.002884745	0.02791209	-0.485229193	0.627928	0.67021	-7.22485758
MAPK7	-0.029031033	3.73855918	-0.485171281	0.627969	0.67021	-7.22488565
ALKAL1	-0.057901002	2.9025544	-0.484956476	0.628122	0.67033	-7.22498973
GAB1	-0.035023583	2.70499488	-0.484816431	0.628221	0.67039	-7.22505755
MEX3B	-0.028224649	1.03276128	-0.484589341	0.628382	0.67052	-7.2251675
WDR55	-0.025454467	3.52696587	-0.484417598	0.628503	0.67061	-7.22525062
CD72	-0.040378146	1.80147913	-0.484072155	0.628748	0.67083	-7.2254177
SDS	-0.052931636	1.39861045	-0.483616359	0.629071	0.67113	-7.22563799
DCST2	0.03448181	1.25653265	0.482970949	0.629529	0.67158	-7.22594956
C14orf39	0.00559347	0.06521615	0.482820475	0.629635	0.67165	-7.22602214
CEP350	-0.038421895	3.40478311	-0.482687832	0.62973	0.67171	-7.2260861
HTR2B	0.037417844	1.08927953	0.481529298	0.630551	0.67254	-7.226644
GJB2	0.063959913	2.54199714	0.48101027	0.63092	0.67289	-7.22689352
FAM72D	-0.006782835	0.12338078	-0.480836227	0.631043	0.67298	-7.22697712
CCND3	-0.03402333	6.18300598	-0.480404858	0.631349	0.67327	-7.22718421
TAS2R5(	-0.005339821	0.0605756	-0.48031055	0.631416	0.6733	-7.22722946
MN1	-0.046568247	1.64575922	-0.480138402	0.631539	0.67338	-7.22731204
RPS6KA:	-0.04798014	4.91465397	-0.47976276	0.631805	0.67363	-7.22749213
C1QTNF	0.036449651	0.75806476	0.479549098	0.631957	0.67375	-7.2275945
MYADM	-0.068273323	6.52560914	-0.479407554	0.632058	0.67381	-7.22766229
FGF16	-0.014501043	0.13866531	-0.479079377	0.632291	0.67402	-7.22781939
CYP3A4	0.006573655	0.0706536	0.47878067	0.632503	0.6742	-7.22796229
PCDHA1	0.021844108	0.38440363	0.478219549	0.632902	0.67458	-7.22823048
TRRAP	-0.040969147	4.43857498	-0.477933497	0.633105	0.67476	-7.22836709
DHCR24	-0.068616847	5.05972034	-0.476350863	0.63423	0.67591	-7.22912139
CDCA5	-0.032378091	1.53479103	-0.476247675	0.634304	0.67595	-7.22917048
HIST1H2	-0.031562774	0.45291434	-0.475721689	0.634678	0.67631	-7.22942057
GLMP	-0.035219378	5.04789653	-0.4755765	0.634781	0.67635	-7.22948955
PCDHGE	-0.041406205	0.79647584	-0.475550633	0.6348	0.67635	-7.22950184

TP53AIP	-0.006898346	0.06767527	-0.474983904	0.635203	0.67674	-7.22977089
GPC6	0.071840284	1.6165311	0.474639081	0.635449	0.67696	-7.22993443
SLCO2B	0.051682097	3.20706521	0.474494579	0.635551	0.67702	-7.23000293
CABP4	0.01954744	0.44325681	0.474290961	0.635696	0.67714	-7.23009942
BRD4	-0.026470658	4.46367003	-0.473991138	0.63591	0.67732	-7.23024142
OBSCN	0.038923404	2.82255425	0.473268015	0.636425	0.67783	-7.23058354
POGK	0.033280695	4.32989369	0.472815676	0.636747	0.67813	-7.23079728
PTCHD1	0.012084704	0.12703027	0.471687564	0.637551	0.67891	-7.23132945
GIGYF1	-0.035074769	5.09518145	-0.47167459	0.63756	0.67891	-7.23133556
SLC38A5	-0.089910344	5.49236372	-0.471357321	0.637787	0.67911	-7.231485
CAMK1G	-0.04113644	0.55260401	-0.470989166	0.638049	0.67934	-7.23165827
TEX15	-0.002580949	0.01080819	-0.470703849	0.638253	0.67952	-7.23179246
CCL8	-0.053771938	1.59074486	-0.47063172	0.638304	0.67953	-7.23182638
TOGARA	-0.006676599	0.11953365	-0.46967159	0.638989	0.68022	-7.23227729
FAM117B	-0.039340308	3.23845109	-0.469466573	0.639135	0.68033	-7.23237346
HOXD4	0.007066667	0.15085802	0.469408075	0.639177	0.68033	-7.23240089
DNAH2	0.011981381	0.20279658	0.469014453	0.639458	0.68059	-7.23258538
KIAA202	-0.036005692	3.80178089	-0.468503863	0.639823	0.68093	-7.23282447
DLX6	-0.038176364	0.31963266	-0.468118865	0.640098	0.68118	-7.23300457
CYP2W1	0.048397272	0.55582279	0.465148312	0.64222	0.6834	-7.23438924
GABRA2	0.00258146	0.01143097	0.46486689	0.642422	0.68357	-7.23451997
LSMEM2	-0.008317789	0.1254013	-0.464654428	0.642574	0.68364	-7.2346186
PACS1	0.031666436	5.82652144	0.464624592	0.642595	0.68364	-7.23463245
SLC1A7	0.013265057	0.17920402	0.464606961	0.642607	0.68364	-7.23464063
C6orf201	-0.006978083	0.17048798	-0.464480705	0.642698	0.68369	-7.23469922
MRGPRE	0.058656941	1.56677143	0.464182629	0.642911	0.68388	-7.23483748
ANKRD2	-0.023462155	0.63176743	-0.463082448	0.643698	0.68467	-7.23534702
TIMD4	-0.046681022	1.16373955	-0.462768343	0.643923	0.68487	-7.23549227
FADS1	-0.028935357	2.22917863	-0.462262433	0.644285	0.68521	-7.23572602
ZNF490	0.02112562	0.77879312	0.46220331	0.644328	0.68521	-7.23575332
PRRC2A	-0.032497917	6.51749614	-0.461856069	0.644576	0.68543	-7.23591358
HEPH	-0.052544947	2.0703967	-0.461549276	0.644796	0.68562	-7.23605508
PLCXD2	0.028290046	0.61676528	0.461352149	0.644937	0.68573	-7.23614595
ZBTB39	-0.033054582	2.43225778	-0.461093005	0.645123	0.68589	-7.23626535
SPNS1	-0.024231598	1.17081752	-0.459976426	0.645923	0.68669	-7.23677903
TMEM23	-0.080002554	4.85158744	-0.459346022	0.646375	0.68713	-7.2370685
SFRP5	0.030497563	0.21020789	0.459236987	0.646453	0.68717	-7.23711852
GATAD2	0.027586314	5.18130572	0.458869137	0.646717	0.68741	-7.23728721
C6	0.018236037	0.18518406	0.458334094	0.647101	0.68777	-7.23753232
KAT6A	0.037672958	3.93018845	0.456389007	0.648497	0.68921	-7.23842101

ADGB	-0.003455571	0.0216128	-0.455328378	0.649259	0.68998	-7.238904
TAS2R3(	0.003814695	0.04017347	0.455088619	0.649431	0.69012	-7.23901303
GNAI2	-0.030728861	7.04377671	-0.454410069	0.649918	0.69059	-7.23932128
DUSP10	-0.045114153	3.34188757	-0.454009986	0.650206	0.69086	-7.23950282
HOXC5	0.009170411	0.04765374	0.45382838	0.650337	0.69095	-7.23958517
PLIN4	0.039015005	0.84514008	0.453305671	0.650712	0.69131	-7.23982201
GLIPR2	0.049200963	4.46648857	0.452809471	0.651069	0.69164	-7.24004658
DES	-0.083364881	1.62666317	-0.452645573	0.651187	0.69173	-7.24012071
SAP25	0.023087836	0.64158417	0.452352098	0.651398	0.69191	-7.24025337
DLEU7	0.01766575	0.42502823	0.452290296	0.651443	0.69191	-7.2402813
CSNK1E	0.033753241	5.49060123	0.451839932	0.651767	0.69221	-7.24048468
CCDC18	-0.0201142	1.12736893	-0.451503217	0.652009	0.69243	-7.24063661
RIOK3	-0.030952165	5.26320414	-0.451023225	0.652354	0.69274	-7.24085299
SLF2	0.027875542	3.50002858	0.450950658	0.652407	0.69274	-7.24088569
TOPBP1	-0.032419732	3.58800559	-0.450921678	0.652427	0.69274	-7.24089874
ZNF503	-0.03298211	3.71291686	-0.450797772	0.652517	0.69279	-7.24095455
MICAL3	-0.03522396	1.61633066	-0.450604178	0.652656	0.6929	-7.24104171
SHROOM	-0.030827444	2.18114545	-0.450175285	0.652965	0.69318	-7.24123469
SYT5	0.018815483	0.18374045	0.449862368	0.65319	0.69338	-7.24137536
TOP3B	-0.006082001	0.19896834	-0.449769425	0.653257	0.6934	-7.24141713
TMEM71	-0.048026245	1.7063672	-0.44911709	0.653727	0.69386	-7.24171002
TRH	-0.017574782	0.11005507	-0.447946278	0.65457	0.69471	-7.24223463
DDX52	0.030422381	3.24008468	0.447842187	0.654646	0.69475	-7.24228121
NPHP3-A	0.002551655	0.02751741	0.447464093	0.654918	0.69499	-7.24245029
ZEB2	-0.033418182	1.84621333	-0.447209951	0.655101	0.69514	-7.24256387
DMXL2	0.034797232	2.62149657	0.446791659	0.655403	0.69542	-7.24275066
TNNC2	-0.058262398	2.12022716	-0.445893473	0.656051	0.69606	-7.24315115
FBXO39	0.011010082	0.19274832	0.445559276	0.656292	0.69628	-7.24329996
GPAT2	-0.042283907	0.84924505	-0.44538087	0.656421	0.69637	-7.24337936
EYS	0.005698776	0.21534967	0.44527438	0.656497	0.69641	-7.24342674
ST8SIA2	-0.036849616	0.61709493	-0.445015645	0.656684	0.69656	-7.2435418
PPP1R9A	0.040201969	3.36876202	0.444860698	0.656796	0.69664	-7.24361067
PUSL1	-0.025546895	3.3996557	-0.444641089	0.656955	0.69676	-7.24370824
CCDC33	0.012652567	0.1838692	0.44454848	0.657021	0.69679	-7.24374938
GPBAR1	-0.018382718	0.60269181	-0.444304379	0.657198	0.69693	-7.24385775
TBC1D3	-0.01266762	0.342953	-0.443700417	0.657634	0.69735	-7.24412565
L1TD1	-0.010214691	0.17903597	-0.443496984	0.657781	0.69746	-7.2442158
CHSY3	-0.027470906	1.3002671	-0.443006671	0.658135	0.69779	-7.24443292
ATL2	-0.042075899	5.97048336	-0.442359913	0.658602	0.69825	-7.24471894
PCSK5	-0.053099349	2.63703532	-0.441449045	0.65926	0.6989	-7.24512106

SAMD8	-0.03260172	3.81716403	-0.441257625	0.659399	0.699	-7.24520546
ASAP1	-0.040091579	3.77082618	-0.441015877	0.659573	0.69915	-7.245312
RNF139	-0.034552671	5.67499957	-0.440586791	0.659884	0.69943	-7.24550096
LY6H	0.029535186	0.41902228	0.440378733	0.660034	0.69955	-7.24559251
MKRN3	0.016840453	0.102388	0.439876243	0.660398	0.69989	-7.24581346
MYH7	0.033955554	0.13969416	0.439693038	0.66053	0.69998	-7.24589395
DDN	-0.011376407	0.10586182	-0.439357337	0.660773	0.7002	-7.24604135
CENPP	0.011974526	0.73173566	0.438603644	0.661318	0.70073	-7.24637189
UPK1B	0.010166718	0.07356208	0.437958075	0.661786	0.70118	-7.24665455
GREM2	0.039298873	0.54483089	0.437868107	0.661851	0.70121	-7.24669391
FOXL2NI	-0.003340393	0.02212433	-0.437307537	0.662257	0.70159	-7.24693897
PDC	-0.006399555	0.1107317	-0.437140786	0.662377	0.70165	-7.24701181
GPATCH	-0.03849511	4.20577655	-0.437127023	0.662387	0.70165	-7.24701782
PDE7A	-0.042368555	3.56876257	-0.436689806	0.662704	0.70191	-7.24720866
ZNF852	-0.016909721	0.87111762	-0.436668883	0.662719	0.70191	-7.24721779
LRIG3	-0.051750902	5.48539673	-0.436214948	0.663048	0.70221	-7.24741571
LY75	0.049983808	3.60592835	0.435954151	0.663237	0.70237	-7.24752933
ITGB1	0.055013484	6.45514227	0.435871847	0.663297	0.70239	-7.24756517
JOSD1	0.036838816	5.13999689	0.435820729	0.663334	0.70239	-7.24758743
ATP11B	-0.041230217	3.93747856	-0.435329557	0.66369	0.70272	-7.24780117
CA1	0.008937832	0.13189179	0.435137927	0.663829	0.70282	-7.24788449
SLC30A7	-0.02866008	3.87464765	-0.434773087	0.664093	0.70306	-7.24804303
COBL	0.051118515	2.53986516	0.434304409	0.664433	0.70337	-7.24824649
HOXA3	-0.017685885	0.57486741	-0.433684023	0.664883	0.70381	-7.24851548
DMRT1	0.041943956	0.74568005	0.432082797	0.666045	0.70497	-7.24920796
S100A5	0.068325164	4.66408001	0.432050754	0.666068	0.70497	-7.24922179
ERAS	0.013190225	0.13526614	0.431976335	0.666122	0.70499	-7.24925391
KCNH6	-0.004986787	0.06716611	-0.431913921	0.666167	0.70499	-7.24928084
VIL1	-0.002705737	0.02223437	-0.431655935	0.666354	0.70514	-7.24939213
N4BP1	-0.025909267	4.45537184	-0.431192619	0.666691	0.70546	-7.24959183
CDKN2D	-0.027216624	3.32250186	-0.43082843	0.666955	0.70569	-7.24974865
SLC26A5	-0.020888875	0.56576137	-0.430663636	0.667075	0.70577	-7.24981956
HPS3	-0.036871899	3.67114706	-0.430392274	0.667272	0.70594	-7.24993628
CAMK2D	-0.036515025	4.74918687	-0.430282841	0.667352	0.70598	-7.24998333
CTSB	0.043921898	11.9346653	0.429640955	0.667818	0.70643	-7.25025906
RAB23	-0.058600173	3.21721462	-0.428575451	0.668592	0.7072	-7.25071584
ZBTB33	-0.038042184	4.76616925	-0.42823656	0.668839	0.7074	-7.25086089
NKX2-8	-0.044051648	2.68354229	-0.428207536	0.66886	0.7074	-7.25087331
PTGDS	0.099333787	3.38286518	0.428005414	0.669007	0.70751	-7.25095976
UBA7	-0.030854316	5.44392574	-0.427172586	0.669612	0.7081	-7.25131553

GGA3	0.033164502	4.21149373	0.427077212	0.669682	0.70813	-7.25135623
SUN2	-0.037503071	5.40589804	-0.426780222	0.669898	0.70831	-7.2514829
SMARCA	-0.02756526	5.59220187	-0.426738606	0.669928	0.70831	-7.25150065
SYT11	0.052569167	2.95101004	0.425378173	0.670918	0.70931	-7.25207973
CERS3	-0.003800217	0.05333403	-0.423950132	0.671958	0.71036	-7.25268561
CCNG2	-0.034037803	4.69685402	-0.423768326	0.67209	0.71046	-7.25276259
DPPA4	0.00393542	0.0356007	0.423660699	0.672169	0.71046	-7.25280816
ISG15	0.06101225	6.51279884	0.423653099	0.672174	0.71046	-7.25281137
DNLZ	0.010085615	0.29150058	0.423363654	0.672385	0.71064	-7.25293384
CDK14	0.038145653	4.12866024	0.42204539	0.673346	0.71161	-7.25349056
IFITM1	0.052833884	6.35133496	0.421569256	0.673693	0.71193	-7.25369122
EXO1	0.018294924	0.68153659	0.421346093	0.673856	0.71206	-7.25378518
DBNDD2	-0.02229976	1.62340184	-0.419323412	0.675331	0.71357	-7.25463461
PRR11	-0.024240448	1.30706601	-0.419212078	0.675412	0.71362	-7.25468125
BRDT	-0.006050533	0.06986788	-0.41904356	0.675535	0.7137	-7.25475181
OVOL1	-0.040013775	1.81883153	-0.418934061	0.675615	0.7137	-7.25479765
NPIPA3	-0.023681071	0.41242659	-0.418923917	0.675623	0.7137	-7.2548019
RAB11FI	0.03440069	3.37257995	0.41801517	0.676286	0.71436	-7.25518183
NKD1	0.02516098	0.45492413	0.417663167	0.676543	0.71459	-7.25532878
HR	-0.045148165	1.50678656	-0.417235466	0.676856	0.71487	-7.25550716
ANKUB1	0.002214011	0.01531351	0.417090277	0.676962	0.71494	-7.25556768
NUF2	0.026335959	0.98180632	0.416938295	0.677073	0.71501	-7.255631
ZWILCH	-0.024744803	2.44837601	-0.416747853	0.677212	0.71512	-7.25571031
PIK3R2	0.021817836	0.89748298	0.415493592	0.678128	0.71604	-7.25623177
ETV6	-0.022507813	4.190808	-0.414987749	0.678498	0.71638	-7.25644162
CPD	-0.042023327	5.26348717	-0.414670199	0.67873	0.71655	-7.25657324
MED12	-0.032176127	4.4695804	-0.414656111	0.678741	0.71655	-7.25657907
RAB34	0.040645286	6.9644545	0.412619377	0.680231	0.71808	-7.25742079
IGSF11	0.026811509	0.41036783	0.412566845	0.680269	0.71808	-7.25744245
ACTA1	-0.040334967	0.58761209	-0.412404104	0.680388	0.71816	-7.25750951
TULP1	-0.010034063	0.20432722	-0.412242322	0.680507	0.71824	-7.25757616
RTTN	-0.019655179	1.63949681	-0.412027336	0.680664	0.71836	-7.25766469
PLEKHO	-0.044516892	3.68673875	-0.411856769	0.680789	0.71845	-7.25773489
TBC1D3I	-0.018776149	0.59656905	-0.411142997	0.681311	0.71895	-7.25802835
AP1S2	-0.027857579	3.60150862	-0.410888877	0.681497	0.7191	-7.2581327
DNHD1	-0.020836728	1.01454461	-0.410464508	0.681808	0.71939	-7.25830683
GBP4	0.057980095	3.88744987	0.410024778	0.68213	0.71968	-7.25848706
LPIN2	0.03493374	4.64212495	0.409844488	0.682262	0.71978	-7.25856091
CTTN	0.029840092	7.01255824	0.408826887	0.683008	0.72052	-7.25897708
HOXA9	0.017114915	0.11065155	0.408750965	0.683064	0.72053	-7.25900809

FGD1	-0.028076162	3.5442779	-0.408680314	0.683116	0.72054	-7.25903694
TTC3	-0.034113375	5.79434223	-0.408402112	0.68332	0.72065	-7.2591505
PPP1R1(	0.047470228	1.05985269	0.408338153	0.683366	0.72065	-7.25917659
MCU	-0.023053489	3.85696311	-0.408329431	0.683373	0.72065	-7.25918015
ANP32D	-0.006038661	0.09632444	-0.408305551	0.68339	0.72065	-7.25918989
RNF222	0.004682371	0.07570506	0.408022584	0.683598	0.72083	-7.25930529
OVOL3	-0.00954403	0.22707296	-0.407248315	0.684166	0.72137	-7.25962063
ZSCAN2:	-0.027938401	0.89539607	-0.407209002	0.684194	0.72137	-7.25963663
CCNK	-0.032273078	1.85149146	-0.406084157	0.68502	0.72219	-7.26009365
CTTNBP.	0.032750702	3.64347411	0.403718245	0.686757	0.72398	-7.26105078
SATB2	0.021241914	0.89559699	0.403629172	0.686822	0.724	-7.26108671
DPEP3	-0.0099932	0.14288161	-0.402346895	0.687764	0.72495	-7.26160301
MED12L	-0.019990298	0.60312059	-0.401627016	0.688293	0.72546	-7.26189214
SH2D1B	-0.014805225	0.30502026	-0.401260873	0.688563	0.7257	-7.262039
KLHDC8/	0.090717163	4.70888565	0.400504122	0.689119	0.72624	-7.26234211
PYGB	0.029460322	7.33863363	0.400407242	0.68919	0.72627	-7.26238087
ZNF621	-0.026677093	3.16389734	-0.400095283	0.68942	0.72647	-7.26250563
IPMK	-0.027997315	2.67007245	-0.399833476	0.689612	0.72663	-7.26261025
RICTOR	-0.029335375	3.0293371	-0.399596752	0.689786	0.72677	-7.2627048
ARHGEF	-0.037374972	5.1205084	-0.399363079	0.689958	0.7269	-7.26279807
RAD9B	-0.010346625	0.51774418	-0.399099977	0.690152	0.72706	-7.26290302
ADAD2	-0.006301133	0.08994891	-0.398907715	0.690293	0.72716	-7.26297966
HIPK4	-0.013308136	0.47701066	-0.398262839	0.690768	0.72762	-7.26323648
FBLIM1	0.03804432	4.01442426	0.398109776	0.690881	0.72769	-7.26329738
PGLYRP	-0.010766377	0.2018086	-0.39789192	0.691041	0.72782	-7.26338402
MTMR8	0.013289136	0.84519574	0.397362683	0.691431	0.72818	-7.26359428
BCL7A	-0.031636701	4.44840531	-0.396156406	0.692319	0.72907	-7.26407248
IL17B	0.009956087	0.21074748	0.39569598	0.692659	0.72938	-7.26425462
STK24	-0.040878939	4.0049975	-0.395085824	0.693108	0.72981	-7.26449567
TTBK1	0.007775669	0.20057134	0.394999725	0.693172	0.72981	-7.26452965
UACA	-0.045044847	5.39568717	-0.394965441	0.693197	0.72981	-7.26454318
PFKFB4	-0.027103429	1.79788504	-0.394643634	0.693434	0.73002	-7.26467013
EGR4	-0.021986154	0.21102537	-0.394214933	0.69375	0.73031	-7.26483908
ARHGAF	0.041094325	4.02958843	0.393922454	0.693966	0.73049	-7.26495424
JADE2	-0.030174186	4.57511558	-0.393177666	0.694515	0.73102	-7.26524711
FXYD2	0.008279401	0.21241332	0.392837497	0.694766	0.73124	-7.26538069
FER	-0.032466802	2.83825548	-0.392523371	0.694998	0.73144	-7.26550394
UTF1	0.007660715	0.09303945	0.392238081	0.695208	0.73161	-7.26561579
LSAMP	0.03554987	0.67219377	0.391985449	0.695395	0.73176	-7.26571477
DDO	0.040911466	2.8165995	0.391928629	0.695437	0.73176	-7.26573702

CATSPE	0.020184367	1.34126947	0.391654464	0.695639	0.73193	-7.26584435
ODF2L	-0.024610546	3.19711922	-0.390481409	0.696505	0.7328	-7.26630272
CKAP5	-0.029234175	4.41334655	-0.390350087	0.696602	0.73285	-7.26635394
KAAG1	-0.020636121	0.75554712	-0.389894348	0.696939	0.73313	-7.26653159
ADORA2	0.032555356	0.98873773	0.389873257	0.696954	0.73313	-7.26653981
SPSB4	0.01369879	0.14218584	0.389669847	0.697104	0.73325	-7.26661903
SKAP1	-0.053878684	1.87351422	-0.389258117	0.697409	0.73352	-7.26677925
RESF1	-0.032087343	4.10040897	-0.388822164	0.697731	0.73381	-7.26694872
TFAP2E	0.024208299	0.88471447	0.387909224	0.698405	0.73448	-7.26730298
LATS1	-0.030838814	3.68038759	-0.387702258	0.698558	0.73459	-7.26738318
RHBDF2	0.023729596	3.52860056	0.386763355	0.699252	0.73528	-7.26774646
TMEM24	-0.003932249	0.06104652	-0.386607627	0.699368	0.73535	-7.26780663
NYAP1	-0.016657058	0.52786873	-0.386423162	0.699504	0.73545	-7.26787787
RBL1	-0.025322458	2.38919605	-0.386365681	0.699547	0.73545	-7.26790006
NPFFR2	-0.046369971	0.83789917	-0.386104382	0.69974	0.73561	-7.2680009
SPATA5	-0.023249441	2.02900091	-0.385982451	0.69983	0.73566	-7.26804794
ZFYVE1	-0.029345799	4.46566705	-0.385636703	0.700086	0.73588	-7.26818122
CD14	-0.040599239	6.20243208	-0.385521623	0.700171	0.73592	-7.26822556
ACVR2B	0.024736077	1.96711035	0.385419571	0.700246	0.73596	-7.26826487
KLHL31	0.029859356	1.1039863	0.384660248	0.700808	0.7365	-7.268557
ABHD17	-0.036510254	4.54519382	-0.384026952	0.701277	0.73695	-7.26880021
FOXJ2	-0.024898646	3.97293449	-0.383911601	0.701362	0.73699	-7.26884447
TUBGCP	-0.030170331	4.50675451	-0.383678055	0.701535	0.73713	-7.26893403
MUC15	0.061805589	7.01187666	0.383312357	0.701806	0.73737	-7.26907416
LAMA2	0.041830081	1.42628186	0.382105429	0.7027	0.73823	-7.26953569
C7orf61	-0.034018403	0.9926873	-0.382093238	0.702709	0.73823	-7.26954035
CLIC6	-0.055050732	4.99114342	-0.381901885	0.702851	0.73833	-7.26961339
DQX1	-0.006602515	0.1257789	-0.381488911	0.703157	0.7386	-7.26977089
GATA6	-0.03617651	3.17336548	-0.380985858	0.703529	0.73895	-7.26996252
PAWR	0.029008601	4.1314511	0.379645699	0.704523	0.73995	-7.27047181
CRB1	-0.005851086	0.14098468	-0.37941765	0.704692	0.74008	-7.27055829
ZNF160	-0.0272966	3.61570597	-0.379240619	0.704823	0.74017	-7.27062539
H6PD	-0.032144194	5.13173985	-0.37899378	0.705006	0.74032	-7.2707189
THPO	0.008516919	0.24309466	0.37834114	0.70549	0.74078	-7.27096584
TMTC2	0.031071354	2.73724245	0.377927818	0.705797	0.74106	-7.27112201
ZBED4	-0.02708266	2.99885056	-0.377462189	0.706143	0.74137	-7.27129774
B3GALT	-0.03059939	0.62897174	-0.376618932	0.706769	0.74198	-7.27161543
CEP250	0.020224573	2.82542974	0.376542019	0.706826	0.742	-7.27164437
PCOLCE	-0.040906111	0.90487181	-0.375739824	0.707421	0.74258	-7.27194588
PER3	-0.041034079	4.36073106	-0.373567648	0.709035	0.74423	-7.27275906

KLF4	0.055350129	4.66890525	0.371168924	0.710818	0.74605	-7.27365159
PHF8	-0.03148686	3.8129177	-0.370794942	0.711097	0.7463	-7.27379022
LRRN4CI	-0.028037695	0.50706713	-0.369884332	0.711774	0.74696	-7.2741272
SHOX	0.002701739	0.02914762	0.369673076	0.711931	0.74708	-7.27420526
CDKAL1	0.026363071	4.05647114	0.368926177	0.712487	0.74762	-7.27448088
ITGAD	0.016231955	0.23870136	0.368865068	0.712533	0.74762	-7.27450341
NFIX	-0.033876537	5.6965466	-0.36844327	0.712847	0.7479	-7.27465879
ZDHHC1	0.027173384	3.62930269	0.368242188	0.712997	0.74801	-7.2747328
SPIN4	-0.026778238	2.2384111	-0.368011217	0.713169	0.74815	-7.27481776
CLK1	-0.037851052	5.88310741	-0.367004668	0.713918	0.74886	-7.2751874
RAD51	0.021052372	1.28718372	0.366982197	0.713935	0.74886	-7.27519564
IFNLR1	-0.036445358	3.55516354	-0.36664242	0.714188	0.74903	-7.27532018
STAP2	-0.028192817	5.4329763	-0.366641087	0.714189	0.74903	-7.27532067
ZNF516	0.029263639	3.48978839	0.365464254	0.715066	0.74991	-7.27575113
ZFYVE26	0.022578699	2.84964424	0.364314259	0.715924	0.75076	-7.27617044
BTN2A2	0.024945525	2.86212023	0.364225349	0.71599	0.75078	-7.2762028
STK35	0.025702178	4.10192453	0.364106891	0.716078	0.75078	-7.27624591
MAD1L1	0.019544083	3.89836267	0.364104013	0.716081	0.75078	-7.27624696
FAM72C	-0.00377464	0.05654507	-0.363720737	0.716366	0.75104	-7.27638633
ARL5B	0.030048162	3.5600163	0.363586673	0.716466	0.7511	-7.27643505
KHDC4	0.029955583	4.26169664	0.363332538	0.716656	0.75125	-7.27652735
KLHL11	0.040595786	1.64126933	0.363151427	0.716791	0.75134	-7.27659309
DYNC1H	-0.024487075	6.23655513	-0.36308502	0.716841	0.75135	-7.27661718
SRD5A2	0.00493837	0.05094893	0.36234772	0.717391	0.75184	-7.27688442
HIP1	0.038919809	5.1413196	0.362343923	0.717394	0.75184	-7.27688579
CDKN3	-0.025096471	1.21925606	-0.361633121	0.717924	0.75235	-7.27714291
SLX1A	0.00261354	0.03226331	0.361227065	0.718227	0.75262	-7.27728956
ARFGEF	-0.032008589	4.60123142	-0.360805006	0.718543	0.7529	-7.27744182
RGPD8	0.014449191	0.62047406	0.360130362	0.719046	0.75334	-7.27768483
SEPTIN3	-0.03802859	1.8357866	-0.360128242	0.719048	0.75334	-7.27768559
NLRP1	-0.03803863	3.58433262	-0.359981636	0.719157	0.75341	-7.27773834
P2RY11	-0.027080913	3.26162068	-0.359314681	0.719656	0.75388	-7.27797803
EPO	-0.00497034	0.08461839	-0.358718874	0.720101	0.7543	-7.27819178
ATF7IP	-0.027961407	4.82307422	-0.357617765	0.720924	0.75511	-7.27858588
TOMM34	-0.027592231	6.31291536	-0.357569338	0.72096	0.75511	-7.27860318
TMEM21	0.01242966	0.33857283	0.357134454	0.721285	0.7554	-7.27875848
TNKS	-0.029094992	3.76791292	-0.357044747	0.721352	0.75542	-7.27879049
CACNA1	0.015492373	0.11608885	0.356479176	0.721775	0.75582	-7.27899212
TP53INP	0.030578151	4.76204728	0.356423998	0.721816	0.75582	-7.27901177
APOBEC	-0.022515197	2.94957543	-0.355877387	0.722225	0.7562	-7.27920631

TTF2	-0.022664184	2.20137587	-0.355430967	0.722559	0.7565	-7.27936496
CRYBG3	-0.061280234	3.46764219	-0.354946454	0.722922	0.75683	-7.27953694
TNFAIP8	-0.032225237	1.52320627	-0.354656579	0.723139	0.75701	-7.27963971
SLC31A2	0.022830152	1.10509378	0.35355929	0.72396	0.75783	-7.28002799
CALCOC	0.029448374	5.45966795	0.35310046	0.724303	0.75814	-7.28018999
CA3	-0.034677806	2.5187222	-0.352787856	0.724538	0.75834	-7.28030025
ARR3	-0.01461946	0.72817512	-0.349915335	0.72669	0.76054	-7.2813088
NOX1	-0.019407304	1.21763903	-0.349637389	0.726898	0.76072	-7.28140595
FOXD1	0.01334334	0.20716321	0.349507619	0.726995	0.76077	-7.28145128
TMC5	0.026118153	1.00587419	0.348552295	0.727712	0.76146	-7.28178449
NCCRP1	0.038339294	1.05727147	0.348508513	0.727745	0.76146	-7.28179974
PCDHGA	-0.045009595	1.72492605	-0.348323071	0.727884	0.76156	-7.2818643
CALB1	-0.006822847	0.1105823	-0.347995675	0.728129	0.76177	-7.28197821
ZNF485	-0.016561209	1.60777474	-0.346971354	0.728898	0.76253	-7.2823339
NEUROD	-0.002285528	0.02892919	-0.346744383	0.729068	0.76266	-7.28241257
SAG	0.001841317	0.01495341	0.346655717	0.729135	0.76268	-7.28244329
LTK	0.030247172	0.8900745	0.346022059	0.72961	0.76313	-7.2826626
RAD51AI	-0.024207242	1.89452017	-0.344785709	0.730538	0.76405	-7.28308934
ZNF705A	-0.0029716	0.02102064	-0.344733295	0.730578	0.76405	-7.2831074
MAGEE2	-0.016359134	0.40814423	-0.343964842	0.731155	0.7646	-7.28337184
ARHGAF	0.03405098	5.41037218	0.343275562	0.731673	0.7651	-7.28360852
RAVER1	-0.035024478	3.72952468	-0.342920838	0.731939	0.76533	-7.28373015
KRTAP5-	-0.020035453	0.47453393	-0.342628996	0.732158	0.76551	-7.28383011
SNX9	-0.024198704	5.79594802	-0.34238032	0.732345	0.76566	-7.28391523
GCNA	-0.030547477	1.7046051	-0.342182009	0.732494	0.76577	-7.28398306
EDA2R	0.037505716	3.68825215	0.342088631	0.732565	0.7658	-7.28401499
ACTN2	0.020378932	0.16800125	0.341662694	0.732885	0.76608	-7.28416051
POU3F2	-0.002177807	0.03468464	-0.341099761	0.733308	0.76648	-7.28435255
DOK4	-0.028216084	4.33805439	-0.34053116	0.733736	0.76688	-7.28454621
WRN	-0.025944418	2.55887811	-0.340382622	0.733847	0.76695	-7.28459674
MYSM1	0.030282212	3.1479591	0.339548326	0.734475	0.76744	-7.28488018
SF3A2	0.02352715	5.97176585	0.339523231	0.734494	0.76744	-7.2848887
ADAMTS	-0.045457007	4.17336104	-0.339515954	0.734499	0.76744	-7.28489117
PALB2	-0.02161748	3.05181397	-0.339513365	0.734501	0.76744	-7.28489205
PCDHA9	-0.003058423	0.05779429	-0.339219445	0.734722	0.76758	-7.28499173
RECQL	0.039882398	3.56670385	0.33921775	0.734724	0.76758	-7.2849923
MYCBP2	-0.031575223	3.66811206	-0.338679716	0.735128	0.76795	-7.28517454
HOXB7	-0.028407653	1.09365228	-0.338555109	0.735222	0.76801	-7.28521671
PLEKHG	0.034962543	3.25175908	0.338351998	0.735375	0.76812	-7.28528541
MAFK	-0.031504576	3.97771185	-0.338270074	0.735437	0.76813	-7.28531311

WDR26	-0.026303058	5.2093409	-0.338204275	0.735486	0.76814	-7.28533535
TOR4A	0.033956487	4.35849343	0.335364828	0.737624	0.77032	-7.28629099
ARHGEF	0.018720612	1.38003892	0.334022389	0.738636	0.77133	-7.28674
FARP1	0.02913222	3.7389268	0.333630527	0.738931	0.77159	-7.28687073
TREML4	-0.006839379	0.05948015	-0.33355424	0.738989	0.77161	-7.28689616
NPAT	0.027657406	3.16951479	0.333417211	0.739092	0.77167	-7.28694183
SNAI2	-0.03702673	2.71577244	-0.333290006	0.739188	0.77172	-7.2869842
CCDC18	0.018339779	0.9658749	0.333134754	0.739305	0.77178	-7.2870359
IL5	0.004463359	0.1113696	0.333091938	0.739337	0.77178	-7.28705016
PRKG2	-0.013103012	0.26640563	-0.332711949	0.739624	0.77203	-7.28717657
ZFC3H1	-0.026574668	3.50907269	-0.332216425	0.739997	0.77238	-7.28734121
TAS2R20	-0.016655526	0.84771868	-0.331272862	0.740709	0.77307	-7.28765403
TSBP1	0.003757025	0.0535304	0.331196692	0.740767	0.77308	-7.28767924
RPS4X	-0.026046631	11.0452959	-0.330837005	0.741038	0.77332	-7.28779822
HTD2	0.00429138	0.11776625	0.330350395	0.741405	0.77365	-7.28795899
LRRC8A	0.028089544	5.78906268	0.330266388	0.741469	0.77367	-7.28798672
PDE3A	0.024840966	1.5352784	0.329936607	0.741717	0.77388	-7.28809551
CLCN1	-0.006822515	0.14386531	-0.329445432	0.742088	0.77422	-7.28825734
IRF7	-0.030965735	4.46275115	-0.329224394	0.742255	0.77435	-7.28833009
DDHD1	0.01917502	1.92292304	0.328922839	0.742483	0.77454	-7.28842926
SIX4	0.026187238	1.66566924	0.328738779	0.742622	0.77464	-7.28848975
MATK	0.029516529	1.5652949	0.327907893	0.743249	0.77524	-7.28876237
EPB41L4	0.030377603	2.99810892	0.327787678	0.74334	0.77526	-7.28880176
BDP1	-0.028385051	3.39358286	-0.327762866	0.743359	0.77526	-7.28880988
HIST1H1	-0.012237036	0.18840555	-0.327393887	0.743637	0.77551	-7.28893068
ARHGAP	-0.018947333	1.09246196	-0.326071316	0.744637	0.7765	-7.28936253
FMR1	-0.025386361	4.88291599	-0.325552591	0.745029	0.77686	-7.28953143
FNBP4	-0.029186662	4.65740306	-0.32547264	0.745089	0.77688	-7.28955743
LEP	0.008344621	0.07740126	0.325161416	0.745324	0.77703	-7.28965862
STX3	0.025965196	4.92327202	0.32515214	0.745331	0.77703	-7.28966163
CD200	-0.032069596	5.00291466	-0.324645931	0.745714	0.77736	-7.28982599
GGTLC3	-0.013617648	0.21513958	-0.3246161	0.745737	0.77736	-7.28983567
SSPN	-0.041689464	4.09959408	-0.324536081	0.745797	0.77737	-7.28986163
OCIAD2	-0.024750226	6.60278498	-0.324478355	0.745841	0.77737	-7.28988035
PHC3	0.027366336	3.13574563	0.324328547	0.745954	0.7774	-7.28992891
SGTB	-0.023979945	2.25678397	-0.324326092	0.745956	0.7774	-7.28992971
CTC1	0.023401193	3.8054651	0.324176255	0.746069	0.77747	-7.28997826
CAPN6	-0.047518553	1.0353008	-0.32386037	0.746308	0.77767	-7.29008055
CD160	0.01029979	0.39326335	0.323489792	0.746588	0.77791	-7.29020042
TJP2	-0.031720355	5.93760304	-0.322156275	0.747597	0.77892	-7.29063064

AK5	-0.012656949	0.25605479	-0.321590345	0.748025	0.77932	-7.29081269
ZKSCAN	-0.022121562	4.37847256	-0.320559383	0.748806	0.78008	-7.29114349
MORC4	0.029014653	3.91970575	0.320490504	0.748858	0.78009	-7.29116556
MCHR1	0.010337101	0.06889636	0.320405408	0.748922	0.78011	-7.29119281
C1orf54	0.021924978	4.43539167	0.320173691	0.749098	0.78024	-7.29126698
MBP	0.027056051	3.49010965	0.32005642	0.749186	0.78026	-7.2913045
HECA	-0.019907851	4.07278633	-0.320026735	0.749209	0.78026	-7.29131399
BICD1	0.026260959	2.9653667	0.319897403	0.749307	0.78032	-7.29135535
ASXL2	-0.028636792	3.41540512	-0.319203502	0.749832	0.78081	-7.29157695
SGCA	-0.025772094	1.02901033	-0.31903932	0.749957	0.7809	-7.29162931
BTN1A1	0.006143277	0.12123547	0.318714548	0.750203	0.78107	-7.29173281
TLN1	-0.024477989	6.6352282	-0.318700172	0.750214	0.78107	-7.29173738
NOTO	0.002592194	0.03713633	0.318053214	0.750704	0.78153	-7.29194323
ZNF236	-0.021907641	2.3896456	-0.317813909	0.750885	0.78167	-7.29201927
CCDC42	-0.009316187	0.22370818	-0.317258976	0.751305	0.78206	-7.29219537
SRCAP	-0.019092103	5.01875103	-0.317073476	0.751446	0.78216	-7.29225417
OR7D2	0.016896877	0.22450986	0.316380284	0.751971	0.78266	-7.29247359
P4HA2	0.031996542	4.82301925	0.315397964	0.752716	0.78335	-7.2927837
IRX2	0.060364275	1.94526526	0.315383574	0.752727	0.78335	-7.29278824
ANKRD3	0.005179737	0.0468818	0.315241641	0.752835	0.78341	-7.29283296
SOX2	-0.004383792	0.08182554	-0.314893642	0.753099	0.78363	-7.29294254
BMPER	0.027626069	0.6129326	0.314839224	0.75314	0.78363	-7.29295966
TSPYL6	-0.002008677	0.02489364	-0.314000714	0.753776	0.78425	-7.29322315
BLOC1S!	-0.009857405	0.37199337	-0.313860613	0.753882	0.78431	-7.2932671
TRPC7	-0.006140898	0.04927008	-0.313128278	0.754438	0.78484	-7.29349655
ENAH	-0.026352632	3.57688853	-0.311750187	0.755484	0.78588	-7.29392686
ZNF8	-0.018743872	2.08218303	-0.310512556	0.756424	0.78681	-7.29431169
KIFC2	-0.028400141	3.04075669	-0.310269479	0.756608	0.78695	-7.2943871
CCNE2	0.016239066	0.9982053	0.310169372	0.756684	0.78698	-7.29441813
ASCL1	0.010642533	0.11996282	0.309873346	0.756909	0.78717	-7.29450985
KIF18A	0.022894289	1.69818668	0.309528254	0.757171	0.78739	-7.29461666
CRLF3	0.019329382	3.10694267	0.309221063	0.757405	0.78759	-7.29471164
GUCY1A	0.032342342	2.84066038	0.308872658	0.757669	0.78781	-7.29481925
SKA1	0.016109487	0.73193094	0.308776895	0.757742	0.78784	-7.29484881
CDRT4	-0.021597979	1.03501296	-0.308618737	0.757862	0.78792	-7.2948976
PRKY	0.027489102	0.43347837	0.307477629	0.75873	0.78877	-7.29524891
WNT6	0.017325308	0.45581571	0.307320743	0.758849	0.78882	-7.29529711
BMP2K	0.021842301	1.76938205	0.307299257	0.758865	0.78882	-7.29530371
CA11	-0.037637794	6.0633219	-0.307078702	0.759033	0.78894	-7.29537142
SH3D21	-0.04662717	3.8824131	-0.306949491	0.759131	0.789	-7.29541107

MYBL1	0.013831451	0.91195493	0.306653745	0.759356	0.78918	-7.29550175
PXN	0.019524994	5.99759261	0.306557957	0.759429	0.78921	-7.2955311
SCN5A	-0.020851565	0.58425253	-0.306148421	0.75974	0.78945	-7.29565649
COL27A1	-0.035202597	1.78438775	-0.306134675	0.759751	0.78945	-7.29566069
DENND2	-0.016945154	4.31980754	-0.305987696	0.759862	0.78951	-7.29570565
JADE3	0.02399557	3.3371318	0.304834122	0.76074	0.79038	-7.29605776
DLX2	-0.006651973	0.1532138	-0.304721829	0.760825	0.79038	-7.29609196
NBPF10	-0.008029504	0.44443306	-0.304698249	0.760843	0.79038	-7.29609915
CHD4	-0.023247662	7.3914873	-0.304644462	0.760884	0.79038	-7.29611552
FEM1C	-0.02065975	3.76436879	-0.303999819	0.761375	0.79084	-7.29631159
TYMS	0.029003879	3.71872882	0.30390282	0.761449	0.79087	-7.29634105
RBP1	0.046897434	3.15417211	0.303744844	0.761569	0.79095	-7.29638902
FMO5	-0.033986275	1.82062813	-0.303395654	0.761835	0.79118	-7.29649496
KIDINS22	-0.028964337	4.72260094	-0.303224924	0.761965	0.79126	-7.29654672
GRID1	0.038545858	4.01987032	0.303165448	0.76201	0.79126	-7.29656474
SLC7A8	-0.034599623	5.75044011	-0.302965309	0.762162	0.79137	-7.29662536
DDX4	0.00196268	0.02027212	0.301768201	0.763074	0.79227	-7.29698712
AIRE	0.005853556	0.05059551	0.301375735	0.763372	0.79253	-7.2971054
TNFRSF1	0.029049211	2.93648193	0.299833643	0.764547	0.7937	-7.2975687
GABRA1	0.003526554	0.05091184	0.299694432	0.764653	0.79376	-7.2976104
ABHD12I	0.006883938	0.19242249	0.299458897	0.764833	0.7939	-7.29768093
CASP2	-0.018027608	3.55424558	-0.299236788	0.765002	0.79403	-7.29774737
CLDN3	-0.032912934	8.72895445	-0.299176175	0.765048	0.79403	-7.2977655
RBMXL2	-0.00362409	0.05290668	-0.297378109	0.766419	0.7954	-7.29830152
PPP1R9I	0.018171148	6.12858006	0.296425313	0.767145	0.79611	-7.29858424
SGSM1	-0.02591702	2.2285708	-0.296203948	0.767314	0.79618	-7.2986498
CYP26C1	0.00870398	0.25820423	0.296203011	0.767315	0.79618	-7.29865008
KHNYN	0.022892668	4.245816	0.295844405	0.767589	0.79642	-7.29875617
ADA2	0.050486439	4.57729131	0.295325326	0.767985	0.79678	-7.29890952
SFTPA1	0.093674999	1.84243667	0.294902003	0.768308	0.79707	-7.29903438
AKAP11	0.027499166	4.16027086	0.294356074	0.768724	0.79745	-7.29919513
CEP135	-0.017405058	1.75497749	-0.293338034	0.769501	0.79821	-7.29949411
BAAT	0.002573814	0.04236303	0.29254333	0.770108	0.79879	-7.29972678
KCTD4	-0.001987965	0.03038623	-0.291957776	0.770555	0.7992	-7.29989782
LNPEP	-0.022593645	3.84262341	-0.291846439	0.77064	0.79924	-7.2999303
KIF26B	-0.024845451	1.50519844	-0.291669461	0.770775	0.79933	-7.29998191
POLH	0.025121306	3.8958935	0.290929693	0.77134	0.79987	-7.30019728
PON2	0.028060554	5.70767981	0.290700315	0.771516	0.8	-7.30026395
C17orf99	0.006311959	0.10704827	0.289557159	0.772389	0.80086	-7.30059543
NTAN1	-0.017668273	3.50491462	-0.28911587	0.772726	0.80116	-7.30072304

BATF2	-0.035928042	2.3401306	-0.288549336	0.77316	0.80156	-7.30088658
TXNDC1(	-0.020221651	2.38237756	-0.288449355	0.773236	0.80159	-7.30091541
COL4A4	0.030309962	3.59023223	0.288218575	0.773412	0.80173	-7.30098192
SLC19A2	-0.027514255	4.0168155	-0.288028827	0.773558	0.80179	-7.30103656
ANKRD2	-0.022090418	4.02108204	-0.288020186	0.773564	0.80179	-7.30103905
ARNT2	-0.036506888	3.9175561	-0.28790083	0.773655	0.80183	-7.3010734
HACE1	-0.018957172	2.10199047	-0.287416352	0.774026	0.80217	-7.30121269
HSD17B2	0.0032339	0.02453194	0.287210627	0.774183	0.80228	-7.30127176
CNIH2	-0.013868492	0.81729308	-0.286278755	0.774896	0.80297	-7.30153883
ASIP	-0.027128812	1.15460198	-0.286043762	0.775076	0.80311	-7.30160604
GTF2H4	0.020446108	2.38785311	0.284793716	0.776032	0.80405	-7.30196263
ANAPC1	-0.017311687	1.8794325	-0.283081097	0.777343	0.80536	-7.30244864
PIF1	-0.012493891	0.51714777	-0.282077597	0.778112	0.80611	-7.30273206
CNGB3	-0.001739164	0.02667485	-0.281265269	0.778734	0.8067	-7.30296075
FANCB	0.006829224	0.40892807	0.281148426	0.778823	0.80674	-7.30299359
EFR3A	-0.026308198	6.02977067	-0.280911969	0.779005	0.80684	-7.30306
PPP1R3(	-0.026900935	1.30755439	-0.28090713	0.779008	0.80684	-7.30306136
SORL1	-0.035423578	3.70750748	-0.280375477	0.779416	0.80721	-7.30321049
CCNL2	0.030498331	5.5005186	0.279062139	0.780422	0.8082	-7.30357766
PCDHB3	-0.014720474	0.59456344	-0.278918369	0.780532	0.80827	-7.30361775
LILRB5	0.015841133	0.6834067	0.278517579	0.78084	0.80852	-7.3037294
APCDD1	0.027847847	2.48684096	0.278470256	0.780876	0.80852	-7.30374257
CRMP1	-0.01805378	2.96073183	-0.277918327	0.781299	0.80891	-7.30389603
PTP4A1	-0.025126697	6.02300149	-0.277635295	0.781516	0.80909	-7.30397461
FAT2	-0.023158668	0.74852184	-0.275243385	0.783351	0.81092	-7.30463549
SLC30A3	-0.047755571	1.09552587	-0.27514893	0.783424	0.81092	-7.30466147
CAD	0.023200121	4.04300214	0.275137709	0.783432	0.81092	-7.30466455
LHB	-0.018610231	0.55397227	-0.274877833	0.783632	0.81108	-7.30473598
RBPJ	-0.014846381	4.7909614	-0.274491229	0.783928	0.81129	-7.30484212
SLC39A1	-0.034730912	5.77069512	-0.274483535	0.783934	0.81129	-7.30484423
GLCE	0.024834387	3.12838393	0.27422125	0.784136	0.81145	-7.30491615
EGR3	-0.048102746	2.64439679	-0.273536698	0.784661	0.81195	-7.30510354
MREG	-0.025823859	3.77869017	-0.272790247	0.785234	0.81249	-7.30530734
EFS	-0.034268456	3.48746797	-0.272581684	0.785394	0.81261	-7.30536418
GPR137(	-0.016100347	0.98936617	-0.272421655	0.785517	0.81268	-7.30540777
TAB3	0.019008088	2.98045161	0.272363376	0.785562	0.81268	-7.30542364
TLCD1	-0.022910865	4.37722661	-0.272074254	0.785784	0.81286	-7.3055023
RRP1B	0.016917852	4.18866667	0.271295672	0.786382	0.81343	-7.30571372
CAPN2	0.021530301	6.4755414	0.27053801	0.786964	0.81398	-7.30591888
BICDL2	-0.027405672	3.70860018	-0.26985656	0.787488	0.81448	-7.30610291

GPR156	-0.005040507	0.15111257	-0.26755615	0.789256	0.81625	-7.30672074
DENND1	-0.022589631	3.90367509	-0.26688685	0.789771	0.81674	-7.3068995
TNFRSF:	-0.028695908	4.42958133	-0.266199486	0.7903	0.81723	-7.30708262
FLCN	0.019544125	3.72395111	0.266107944	0.79037	0.81726	-7.30710697
NTSR1	0.00286498	0.04620371	0.264165105	0.791865	0.81875	-7.30762183
PARD6B	-0.024471297	3.52515488	-0.263903158	0.792067	0.81891	-7.30769096
GPR35	0.012934638	0.71367889	0.263523962	0.792359	0.81916	-7.30779091
MAP3K1!	-0.002838085	0.05672256	-0.262900309	0.792839	0.81961	-7.30795498
MND1	-0.017881284	1.47741265	-0.262000882	0.793531	0.82028	-7.30819092
SLC8A3	0.002687884	0.03078713	0.261672099	0.793784	0.82049	-7.30827697
IZUMO2	0.011883877	0.23208642	0.26105061	0.794263	0.82093	-7.30843932
TMC1	0.004136097	0.09756387	0.260944882	0.794345	0.82097	-7.3084669
CCIN	0.005991998	0.1951483	0.260652023	0.79457	0.82115	-7.30854324
MYO5C	-0.023223114	4.40345098	-0.259568008	0.795405	0.82196	-7.30882507
GLI1	-0.0125939	0.36941694	-0.259287502	0.795622	0.82214	-7.3088978
AICDA	-0.010677194	0.10966657	-0.259123719	0.795748	0.82218	-7.30894024
CHML	-0.020539245	2.62888424	-0.259107144	0.795761	0.82218	-7.30894453
TACC1	0.024743909	4.54618947	0.258772788	0.796018	0.8224	-7.30903106
MAP7D2	-0.017890708	0.56594774	-0.2571068	0.797303	0.82367	-7.30946058
B4GALN'	-0.017595818	0.30009888	-0.257022278	0.797368	0.82369	-7.3094823
GRIA3	0.028503006	0.49825503	0.255181056	0.798788	0.82511	-7.30995361
MAST1	-0.009891726	0.35401431	-0.254139968	0.799591	0.82584	-7.31021861
SRRM2	-0.022118218	6.82712446	-0.254135641	0.799595	0.82584	-7.31021971
KIF2A	-0.025435624	3.29238424	-0.253375588	0.800181	0.82639	-7.31041249
DNAH5	0.017236257	0.60519878	0.252465442	0.800884	0.82707	-7.31064257
KIF27	0.021508473	2.26550029	0.252174309	0.801109	0.82725	-7.310716
MSMO1	-0.021542621	5.55021973	-0.25133951	0.801753	0.82787	-7.31092607
NAF1	-0.01894214	2.95163549	-0.251096382	0.801941	0.82801	-7.31098711
AKAP5	0.013207765	0.98612503	0.250920465	0.802077	0.8281	-7.31103125
SLC4A9	0.010017466	0.20462524	0.250667423	0.802272	0.82821	-7.31109468
ITIH2	0.007617968	0.33397647	0.250652992	0.802283	0.82821	-7.3110983
CIC	0.019798085	5.18219407	0.250010179	0.80278	0.82867	-7.31125914
ASB11	0.003293309	0.03486946	0.249908953	0.802858	0.8287	-7.31128443
PCDHGE	-0.020922564	0.71640426	-0.24984091	0.802911	0.82871	-7.31130142
EPN2	-0.017151221	3.82207088	-0.248682313	0.803806	0.82958	-7.31159008
C5orf46	0.013337346	0.30276011	0.248378591	0.80404	0.82976	-7.31166553
NIN	0.017174884	2.82350663	0.248338564	0.804071	0.82976	-7.31167547
ZNF81	-0.01536146	1.38914066	-0.247950928	0.804371	0.83001	-7.31177161
ACAP1	-0.020303046	2.5882452	-0.247892265	0.804416	0.83001	-7.31178615
FMO2	0.024140969	0.97785038	0.247422342	0.80478	0.83033	-7.31190248

PKD2L1	0.01036171	0.23810532	0.246225123	0.805705	0.83124	-7.31219784
ABRAXA	-0.016312789	2.20969197	-0.246050848	0.80584	0.83133	-7.31224072
SLC22A1	0.002082121	0.04983011	0.24551901	0.806251	0.8317	-7.31237138
ST6GALI	-0.026656931	3.61402401	-0.24538751	0.806353	0.83176	-7.31240364
SYNPR	0.035988405	0.81331748	0.245173314	0.806518	0.83188	-7.31245615
MAPK15	0.037593447	3.32735311	0.244911776	0.806721	0.83203	-7.31252021
TUT7	0.01657016	3.42225412	0.244769026	0.806831	0.8321	-7.31255515
EFNA1	-0.021582636	6.50060931	-0.24460958	0.806955	0.83217	-7.31259415
NUTM2G	-0.003878737	0.18615484	-0.243251299	0.808005	0.83321	-7.31292532
KIAA121(	-0.001843741	0.0163957	-0.242780929	0.808369	0.83353	-7.31303958
NLGN3	-0.010701032	0.47265378	-0.242404199	0.808661	0.83378	-7.31313093
ANKS6	-0.019544232	4.5789771	-0.24077144	0.809925	0.83502	-7.3135252
GSTCD	0.020276131	2.41813944	0.240721204	0.809964	0.83502	-7.31353729
ZNF703	-0.017647632	3.71963411	-0.240439704	0.810182	0.8352	-7.31360499
PIK3R1	-0.02390896	4.35626108	-0.240135198	0.810417	0.83536	-7.31367812
AQP5	-0.061870374	3.67016317	-0.240109064	0.810438	0.83536	-7.31368439
NR4A2	0.039472977	3.4925226	0.239868873	0.810624	0.8355	-7.31374201
CES4A	0.026792706	2.78267203	0.238716566	0.811516	0.83637	-7.31401763
CCNL1	-0.022457512	4.60490192	-0.238477802	0.811701	0.83651	-7.31407457
ABCC2	-0.00423613	0.23240171	-0.237675803	0.812322	0.8371	-7.31426542
ZNF141	0.015928083	2.34277409	0.237519362	0.812444	0.83717	-7.31430258
BGLAP	0.021074355	1.48774261	0.236744185	0.813044	0.83773	-7.31448632
DEF6	0.025389235	4.00357758	0.236694011	0.813083	0.83773	-7.31449819
ISY1-RAI	0.00140636	0.02592416	0.235964634	0.813649	0.83826	-7.31467048
TMEM19	-0.015035883	0.20222466	-0.23587047	0.813722	0.83829	-7.31469269
ECT2	-0.017733573	2.28864337	-0.234189076	0.815025	0.83958	-7.31508769
EIF4EBP	0.022078853	4.48313779	0.233962626	0.815201	0.83971	-7.31514068
SNAPC2	0.017060071	5.01795028	0.233873127	0.81527	0.83973	-7.3151616
C11orf42	-0.008259043	0.35348586	-0.233737044	0.815376	0.83979	-7.31519341
RAB33A	0.017341	1.08953224	0.233427851	0.815616	0.83998	-7.3152656
MAPK8IF	0.022453789	3.80985187	0.231922028	0.816784	0.84113	-7.31561581
ZNF738	-0.014944528	2.09915185	-0.230877345	0.817594	0.84192	-7.31585745
RIPPLY1	0.014210506	0.44043831	0.230675927	0.817751	0.84203	-7.31590391
C10orf11	-0.006752346	0.15494732	-0.230115252	0.818186	0.84242	-7.31603303
OSR1	0.01467044	0.49657176	0.22998516	0.818287	0.84248	-7.31606295
ASAH2	0.007641423	0.42165092	0.229755638	0.818465	0.84258	-7.31611568
PITPNM3	-0.021967646	2.35215815	-0.229623414	0.818568	0.84258	-7.31614604
HP	-0.024085114	0.53248494	-0.229605964	0.818581	0.84258	-7.31615005
SLC22A2	-0.002280742	0.0268414	-0.229573525	0.818606	0.84258	-7.31615749
MYO1C	0.016297489	6.5429474	0.229530613	0.81864	0.84258	-7.31616734

ZNF213	-0.012689983	3.78699497	-0.229398383	0.818742	0.84264	-7.31619767
PLA1A	-0.027156057	2.32476703	-0.229013822	0.819041	0.84289	-7.31628577
GPR85	-0.010910127	0.46158784	-0.228337872	0.819566	0.84338	-7.31644028
LCN12	-0.034160764	6.73606932	-0.227611542	0.82013	0.84391	-7.3166058
PTPN12	0.016461698	5.22709169	0.227047494	0.820568	0.84431	-7.31673397
FIGNL2	0.014843242	0.66779803	0.226856136	0.820717	0.84441	-7.31677738
PCSK1	0.006676877	0.22326729	0.226670042	0.820861	0.84451	-7.31681956
RASA4	-0.007193403	0.46000344	-0.22446748	0.822572	0.84622	-7.31731618
HSF5	0.002495666	0.05925543	0.223534296	0.823298	0.8469	-7.31752512
GRHL1	0.018970021	2.18902908	0.223486289	0.823335	0.8469	-7.31753585
PRF1	0.029044726	2.43975604	0.222956904	0.823747	0.84724	-7.31765397
YPEL2	0.016775434	3.91770015	0.222939287	0.82376	0.84724	-7.3176579
LMOD3	0.006916156	0.15540898	0.222385571	0.824191	0.84763	-7.31778114
L3MBTL3	0.020002054	2.95146659	0.22206455	0.824441	0.84783	-7.31785245
PRRC2B	0.028234717	4.90838081	0.221037875	0.825239	0.8486	-7.31807981
MAD2L2	0.016197865	4.559971	0.219574319	0.826378	0.84972	-7.31840211
PIGA	0.01748126	3.23597588	0.219299835	0.826591	0.84989	-7.31846232
ARHGAP	-0.015906628	6.07063811	-0.218532321	0.827188	0.85045	-7.31863027
RGPD6	-0.001074907	0.01035943	-0.218221767	0.82743	0.85065	-7.31869806
GDPD4	-0.009529071	0.09906939	-0.217026173	0.828361	0.85156	-7.31895815
PCDHA1	0.018325116	0.94662785	0.216574959	0.828712	0.85187	-7.31905593
C9	-0.002470598	0.07044563	-0.21594104	0.829206	0.85232	-7.31919296
POLE2	0.011802712	1.71118422	0.214898317	0.830018	0.8531	-7.3194175
C18orf54	-0.008323003	0.95783455	-0.214641273	0.830218	0.85326	-7.31947268
PEA15	0.014314497	7.80594295	0.214545543	0.830292	0.85328	-7.31949321
IL17REL	0.004209425	0.08635129	0.213058486	0.831451	0.85442	-7.31981102
ENC1	-0.026934491	3.14796638	-0.212773632	0.831673	0.8546	-7.31987165
GEM	-0.030479077	3.30019233	-0.21220072	0.832119	0.85499	-7.31999334
C5orf67	0.022037587	1.75091597	0.212155581	0.832154	0.85499	-7.32000291
TOX	-0.025670495	1.39851621	-0.211751786	0.832469	0.85522	-7.32008846
FUT8	0.017628276	3.66972197	0.211725089	0.83249	0.85522	-7.32009411
SPEN	0.019206864	4.53912827	0.211672512	0.832531	0.85522	-7.32010524
CLDN6	-0.016083516	0.67998193	-0.210704412	0.833285	0.85594	-7.32030961
CCDC15	0.016658094	1.13907578	0.210617285	0.833353	0.85596	-7.32032796
MAP3K2	0.020070751	3.47870604	0.209700082	0.834068	0.85662	-7.32052064
CAPN5	0.02684638	2.82683642	0.209667997	0.834093	0.85662	-7.32052736
TCF19	-0.016205265	3.14970111	-0.209578297	0.834163	0.85664	-7.32054616
ZNF600	-0.009974996	2.02746449	-0.209497695	0.834226	0.85665	-7.32056304
TPBG	-0.01992431	3.38889519	-0.208374422	0.835102	0.8575	-7.32079764
RHOXF1	0.018424282	0.94654481	0.208210828	0.83523	0.85758	-7.3208317

CCL25	-0.011905931	0.50900162	-0.207575231	0.835726	0.85804	-7.32096379
SHD	-0.006141386	0.06098483	-0.207153208	0.836055	0.85832	-7.32105127
GPR27	0.029096007	4.45347924	0.207004211	0.836171	0.85839	-7.32108211
BAIAP2	-0.018573106	4.32212	-0.206787121	0.83634	0.85851	-7.32112701
CENPH	0.013175619	2.52220783	0.206115885	0.836864	0.85898	-7.32126553
NUDT1	0.01534913	3.65977208	0.206061449	0.836907	0.85898	-7.32127674
AMMECF	0.014604341	2.58998929	0.206005961	0.83695	0.85898	-7.32128817
KIF13B	-0.017272871	4.42745983	-0.205012785	0.837725	0.85972	-7.3214922
HIST1H1	0.019189371	0.93363666	0.204685861	0.83798	0.85991	-7.32155914
KCNG3	-0.004292139	0.04491213	-0.204650274	0.838008	0.85991	-7.32156643
PXDN	-0.022682966	3.08863193	-0.20410447	0.838434	0.8603	-7.32167793
MATN4	0.007819822	0.28620315	0.203964426	0.838543	0.86031	-7.3217065
B4GALT:	-0.018691131	7.21715422	-0.20395182	0.838553	0.86031	-7.32170907
SOCS3	0.035526587	5.66443484	0.203755145	0.838707	0.86038	-7.32174914
ITPR2	-0.020936173	3.5624281	-0.203739164	0.838719	0.86038	-7.3217524
CRYBG1	0.021075135	3.39369163	0.202346662	0.839806	0.86144	-7.32203503
TNIK	-0.021643212	4.11767377	-0.202104858	0.839995	0.86158	-7.32208391
MGARP	0.011393267	1.03998712	0.201505441	0.840463	0.86201	-7.32220483
TRIM34	-0.006185223	0.56589127	-0.200143169	0.841527	0.86303	-7.3224783
ENOX1	-0.010147382	0.68962231	-0.200102993	0.841559	0.86303	-7.32248634
ERVFRD	0.001432717	0.04233147	0.199186415	0.842275	0.86371	-7.32266926
BRPF3	-0.019503287	4.74476912	-0.198631902	0.842708	0.86408	-7.32277951
TTC39A	0.019434408	3.08564461	0.198602356	0.842731	0.86408	-7.32278538
RPL3L	-0.007232796	0.1142869	-0.198521432	0.842795	0.86409	-7.32280144
GGT6	0.039315573	2.30524747	0.198442445	0.842856	0.8641	-7.32281711
ANKLE1	0.006321974	0.43216855	0.198337426	0.842938	0.86413	-7.32283794
EEF2K	-0.012545286	3.70894188	-0.198052136	0.843161	0.86431	-7.32289446
HOXC9	-0.013192136	0.39005199	-0.197737346	0.843407	0.86451	-7.32295674
SLC8A2	0.005526447	0.15548832	0.197566161	0.843541	0.86459	-7.32299056
FOXM1	0.014646063	1.48821343	0.196545044	0.844339	0.86532	-7.3231917
PNCK	-0.004303936	0.1142791	-0.196532664	0.844349	0.86532	-7.32319413
LRRC74/	-0.002124352	0.06692581	-0.195683444	0.845013	0.86586	-7.32336061
CPSF1	0.014552672	5.42521055	0.19566719	0.845026	0.86586	-7.32336379
NRROS	0.016225482	2.86718635	0.195628653	0.845056	0.86586	-7.32337132
CDK2	0.013422144	3.89254491	0.195588729	0.845087	0.86586	-7.32337913
ERICH6E	-0.004518432	0.28821549	-0.194960728	0.845578	0.86632	-7.32350172
ERN2	-0.004119842	0.0933217	-0.194530249	0.845915	0.86661	-7.32358552
NPW	0.036365022	1.76044182	0.193928139	0.846386	0.86701	-7.32370243
CASP8	0.014872215	2.95106452	0.193896499	0.846411	0.86701	-7.32370856
PDE4DIF	0.013775507	2.60899926	0.1937175	0.846551	0.8671	-7.32374324

NCEH1	-0.015510469	3.4475348	-0.192531432	0.847478	0.868	-7.32397221
ACTN4	0.013549046	8.03575418	0.192374249	0.847601	0.86806	-7.32400245
HORMAD	-0.013997821	0.28273397	-0.192319715	0.847644	0.86806	-7.32401294
ZNF514	-0.014408687	3.01645227	-0.191882273	0.847986	0.86836	-7.32409694
ZYG11B	0.015575312	4.60473876	0.191440584	0.848332	0.86863	-7.32418156
SNX33	0.01365065	4.55532549	0.191414423	0.848353	0.86863	-7.32418657
TNIP1	0.010523189	6.30499707	0.191157996	0.848553	0.86879	-7.3242356
FBLL1	0.022457733	1.41548718	0.190874762	0.848775	0.86895	-7.32428968
ADAR	0.01544971	6.55617357	0.190817651	0.84882	0.86895	-7.32430058
CALN1	-0.010653135	0.10951555	-0.190043328	0.849426	0.86952	-7.32444798
AGAP9	-0.01627334	1.85139783	-0.189094171	0.850169	0.87023	-7.32462785
BAALC	-0.01992466	1.1715016	-0.188439909	0.850681	0.8707	-7.3247513
AHI1	-0.013729378	2.96162919	-0.188369541	0.850736	0.87071	-7.32476456
KCNQ4	-0.005145883	0.4818629	-0.187479942	0.851433	0.87137	-7.32493168
MTRNR2	-0.054064723	1.44152307	-0.186219687	0.85242	0.87232	-7.32516707
GNRH1	-0.013595411	1.35532351	-0.186159882	0.852467	0.87232	-7.3251782
ACTB	0.010239011	11.5337984	0.185795019	0.852753	0.87251	-7.32524603
CNTN6	-0.033391754	2.67071028	-0.185784786	0.852761	0.87251	-7.32524793
RELA	-0.011101581	5.57035412	-0.18494596	0.853418	0.87313	-7.32540337
LRRC55	-0.006209646	0.30753894	-0.184137809	0.854051	0.87373	-7.32555245
UBA6	-0.0115637	3.1979969	-0.182357312	0.855447	0.87508	-7.32587861
RGPD3	-0.003417422	0.11389846	-0.182319526	0.855476	0.87508	-7.3258855
EXTL1	0.0112774	0.82841955	0.18214779	0.855611	0.87517	-7.32591678
CA7	-0.018027574	1.03991185	-0.18160672	0.856035	0.87553	-7.32601515
RPL36A-	-0.004062067	0.2446491	-0.18156239	0.85607	0.87553	-7.3260232
RFX2	0.014845701	2.05094754	0.181501069	0.856118	0.87553	-7.32603433
PIMREG	0.011321838	0.86655526	0.181382384	0.856211	0.87557	-7.32605586
ELMOD1	-0.00592711	0.11190853	-0.181190058	0.856362	0.87564	-7.32609071
TGFB1	-0.021026968	5.88769859	-0.181161398	0.856384	0.87564	-7.3260959
XYLT1	-0.017160722	2.41155435	-0.180367662	0.857007	0.87622	-7.32623933
BLK	-0.016452473	0.53534673	-0.179089091	0.858009	0.8771	-7.32646905
SEC31B	-0.014690273	1.61279564	-0.179026682	0.858058	0.8771	-7.32648022
RGPD1	0.001829778	0.03753103	0.178978158	0.858096	0.8771	-7.3264889
MYO15B	0.022802067	3.53844416	0.178916841	0.858144	0.8771	-7.32649987
CCDC11	-0.011950618	1.14388245	-0.178894548	0.858162	0.8771	-7.32650385
FAM160E	-0.013281321	4.19313546	-0.178878666	0.858174	0.8771	-7.32650669
MCL1	0.016689174	7.98355689	0.178490558	0.858479	0.87736	-7.32657602
PCDHB1	-0.012058082	1.05480704	-0.177843384	0.858986	0.87783	-7.32669128
ZNF560	-0.019987114	0.71409783	-0.177000225	0.859648	0.87845	-7.32684083
RNPC3	-0.015652865	3.05021415	-0.176608401	0.859956	0.87871	-7.32691008

ANXA4	-0.014377638	4.94124583	-0.175959464	0.860465	0.87918	-7.32702444
BCAN	-0.016100845	0.25785486	-0.175527813	0.860804	0.87943	-7.32710027
FMN1	-0.016002324	1.76784892	-0.175515552	0.860813	0.87943	-7.32710242
FXYD7	-0.00667991	0.36353943	-0.175349675	0.860943	0.87951	-7.32713151
ATP12A	0.010886747	0.24334241	0.175067671	0.861165	0.87964	-7.32718091
AWAT2	-0.005801005	0.24884382	-0.175054674	0.861175	0.87964	-7.32718318
SHISAL2	0.012588693	1.0648876	0.173575342	0.862336	0.88077	-7.32744096
UTS2	0.011341807	0.34073056	0.173441664	0.862441	0.88083	-7.32746415
EP400	-0.012341529	3.42872894	-0.173267406	0.862578	0.88091	-7.32749435
ACAN	-0.014619136	0.76901436	-0.171131963	0.864255	0.88257	-7.32786195
TERB1	0.00286977	0.15781393	0.170802248	0.864514	0.88278	-7.3279183
FAM126A	0.01037847	1.47618633	0.170696627	0.864597	0.88281	-7.32793633
RBFOX1	-0.020214138	0.49809126	-0.170285165	0.86492	0.88309	-7.32800645
TCP11L2	0.015959425	2.01279325	0.17020014	0.864987	0.88311	-7.32802092
PCDHGA	-0.009207095	0.56912193	-0.169461774	0.865567	0.88365	-7.32814628
ALDH1L2	-0.014073491	1.13028515	-0.16938334	0.865629	0.88366	-7.32815956
PRKD2	-0.011880483	5.39390731	-0.168480274	0.866338	0.88431	-7.32831207
PDE6C	0.005184153	0.38430716	0.16843011	0.866378	0.88431	-7.32832052
INPP4B	-0.011673489	1.65410151	-0.16824126	0.866526	0.88441	-7.3283523
PCBP3	0.005159208	0.24595074	0.167779235	0.866889	0.88473	-7.3284299
KLHL38	0.003551449	0.15301992	0.166887247	0.86759	0.88539	-7.3285791
NHLH1	0.002519939	0.13753473	0.166748668	0.867699	0.88545	-7.32860221
C21orf58	0.010220222	1.67716427	0.166661467	0.867768	0.88547	-7.32861675
ZBTB34	0.011752344	2.93100361	0.165951328	0.868326	0.88598	-7.32873481
DNMBP	-0.013281063	3.49007906	-0.165820036	0.868429	0.88603	-7.32875658
NCOA2	-0.01495305	3.94926689	-0.165375472	0.868779	0.88634	-7.32883018
NAB1	-0.014860083	5.45747459	-0.164496356	0.86947	0.88694	-7.32897513
INPP5F	0.015950855	4.17865812	0.164490403	0.869475	0.88694	-7.3289761
A1BG	-0.005149483	0.44093703	-0.164249578	0.869664	0.88708	-7.32901568
KLK4	0.030674683	1.27335434	0.164152939	0.86974	0.8871	-7.32903154
FRG2C	-0.001766539	0.04850083	-0.1639531	0.869897	0.88721	-7.32906431
OMP	0.004437125	0.14702856	0.163434687	0.870305	0.88757	-7.32914915
AKR1D1	-0.002691554	0.11749222	-0.16303081	0.870623	0.88784	-7.32921505
FAM217A	-0.001366549	0.04987404	-0.162840093	0.870773	0.88794	-7.32924611
MPO	0.002819085	0.136871	0.16240404	0.871116	0.88816	-7.329317
LRIT3	-0.003872337	0.24435411	-0.162403767	0.871116	0.88816	-7.32931705
UBXN7	-0.013391469	3.79567805	-0.162366073	0.871146	0.88816	-7.32932317
FAM209A	0.002944092	0.15365472	0.162096293	0.871358	0.88833	-7.32936692
TTC6	-0.000997353	0.01299183	-0.161722359	0.871652	0.88857	-7.32942744
SEMA4F	-0.011080209	2.74661787	-0.161281559	0.871999	0.88885	-7.32949861

PCDH8	-0.012825404	0.94744717	-0.161245643	0.872027	0.88885	-7.3295044
GRAMD1	0.016285308	2.45899321	0.160975399	0.87224	0.88901	-7.32954793
PRKCG	-0.007033312	0.08829587	-0.159942432	0.873052	0.88979	-7.32971362
DDX39B	-0.015917474	4.67105593	-0.159094326	0.87372	0.89041	-7.32984887
PRPH	-0.01040927	0.51792152	-0.158843347	0.873917	0.89056	-7.32988875
ERVW-1	-0.001976384	0.0901031	-0.156741115	0.875572	0.89219	-7.33022036
MFSD2B	0.002635573	0.19791143	0.156313562	0.875909	0.89248	-7.33028726
NPEPPS	-0.012109025	4.62775415	-0.155537438	0.87652	0.89305	-7.33040824
CMA1	0.009803879	0.54816144	0.155378397	0.876645	0.89309	-7.33043296
SSH3	0.012177977	5.65734121	0.155300401	0.876707	0.89309	-7.33044507
MOBP	-0.001671035	0.05425602	-0.155286687	0.876718	0.89309	-7.3304472
TRIM55	-0.011673172	1.16972701	-0.154995871	0.876947	0.89327	-7.3304923
NR4A3	-0.029055858	2.00211331	-0.154193842	0.877578	0.89386	-7.33061625
LRCH4	0.013114484	3.3656257	0.153368014	0.878229	0.89447	-7.33074321
SLC7A2	0.024860304	5.87369683	0.153040784	0.878487	0.89468	-7.33079333
AKIRIN2	0.009995465	5.81995879	0.15294283	0.878564	0.89471	-7.33080831
SCARA5	0.020876411	0.80403029	0.152062261	0.879257	0.89536	-7.33094256
SPOCK1	0.034362961	3.66083368	0.151397115	0.879782	0.89584	-7.33104345
NBEAL1	0.011273396	1.38933318	0.150428549	0.880545	0.89656	-7.33118957
COBLL1	-0.015835046	3.21177661	-0.150294123	0.880651	0.89658	-7.33120978
TRIM21	0.012374328	4.93717913	0.150272051	0.880668	0.89658	-7.33121309
ARHGAF	-0.015206553	3.03134463	-0.149715332	0.881107	0.89697	-7.33129657
ZHX2	0.016113811	5.51269866	0.149553594	0.881234	0.89705	-7.33132077
KDM6B	-0.011707314	4.37733569	-0.148939547	0.881718	0.89749	-7.33141238
CHRND	-0.002936126	0.0522261	-0.148827422	0.881807	0.89752	-7.33142907
STAR	-0.002526121	0.09635083	-0.148642763	0.881952	0.89762	-7.33145653
FAM135A	-0.008516062	1.8584255	-0.148269828	0.882246	0.89786	-7.33151187
ZCCHC1	0.003846924	0.3318399	0.147937196	0.882509	0.89808	-7.33156112
LIG1	0.010489297	3.91726745	0.147870924	0.882561	0.89808	-7.33157092
MFHAS1	-0.012202028	3.78257458	-0.147618123	0.88276	0.89822	-7.33160826
CD38	-0.016773412	1.11258318	-0.147127761	0.883147	0.89856	-7.3316805
CAMK2N	0.016974363	1.48544464	0.146985884	0.883259	0.89862	-7.33170136
ALB	0.014185085	0.60850982	0.146515718	0.883629	0.89895	-7.33177034
TFAP2A	-0.004062583	0.35341132	-0.14629982	0.8838	0.89902	-7.33180194
POTEI	-0.000738953	0.009254	-0.146285661	0.883811	0.89902	-7.33180401
RGPD5	-0.000710417	0.00598943	-0.145930184	0.884091	0.89925	-7.33185593
ERC2	-0.003784544	0.38872733	-0.145260846	0.884619	0.89974	-7.33195335
FAM205A	-0.000897819	0.02660354	-0.144964805	0.884853	0.89992	-7.3319963
DENND4	0.015661822	4.08499557	0.144859762	0.884935	0.89992	-7.33201152
CDRT15	-0.003058449	0.14111985	-0.144827462	0.884961	0.89992	-7.33201619

MGAM2	-0.000858059	0.02151779	-0.144360881	0.885329	0.90024	-7.33208364
ZNF43	0.010009213	3.07260861	0.14427985	0.885393	0.90025	-7.33209533
SYCE1L	-0.019062781	3.34522249	-0.144096203	0.885538	0.90035	-7.3321218
KDM4E	0.004237106	0.17135215	0.143834804	0.885744	0.9005	-7.33215942
MUSK	-0.001791023	0.05894271	-0.143130524	0.8863	0.90101	-7.33226044
ATG2A	-0.009503645	4.31863634	-0.143071767	0.886346	0.90101	-7.33226885
YWHAH	-0.011012731	6.8650804	-0.14272053	0.886623	0.90124	-7.33231902
ARPP21	-0.002024599	0.02156361	-0.142386631	0.886887	0.90145	-7.33236661
RBSN	-0.011710919	4.24513791	-0.142267644	0.88698	0.90149	-7.33238354
C5orf34	-0.007009014	1.36955544	-0.141990665	0.887199	0.90166	-7.33242289
MEP1B	-0.001046451	0.03341248	-0.141437642	0.887635	0.90205	-7.33250124
CD163L1	0.008204933	0.67811082	0.141348261	0.887706	0.90207	-7.33251388
C12orf71	0.003013667	0.16972857	0.141221713	0.887806	0.90211	-7.33253175
ELOVL6	0.013445475	2.14695628	0.140918617	0.888045	0.90225	-7.33257449
GRK6	0.008655759	4.29349275	0.140915053	0.888048	0.90225	-7.332575
MNT	0.00959757	3.44823742	0.140800647	0.888138	0.90229	-7.33259111
SH3GL1	-0.009620785	6.22923276	-0.140288754	0.888542	0.90265	-7.33266303
SEPTIN1	0.013240978	2.57633178	0.139905088	0.888845	0.9029	-7.33271676
TCF3	0.010152329	4.96464048	0.13873777	0.889766	0.90378	-7.33287935
COL17A1	0.028184861	2.63407989	0.138225694	0.890171	0.90414	-7.33295024
AGO4	-0.009390005	3.26624024	-0.137823147	0.890488	0.90441	-7.33300578
KMT2D	0.012281219	3.86153381	0.137526434	0.890723	0.90455	-7.33304662
TESK2	0.010855529	2.94372543	0.137498885	0.890744	0.90455	-7.33305041
ZMIZ2	0.007852065	5.84951158	0.137436615	0.890794	0.90455	-7.33305897
TRMT1	-0.008822768	4.68505975	-0.137337129	0.890872	0.90458	-7.33307263
DCAF15	0.008799141	4.6039443	0.137091168	0.891066	0.90472	-7.33310637
CEBPE	-0.003637824	0.25077322	-0.136249892	0.891731	0.90534	-7.33322131
AIDA	0.01103199	3.7721626	0.135995938	0.891931	0.90549	-7.33325587
KDELR3	0.015683127	5.0244289	0.135818671	0.892071	0.90554	-7.33327996
HS2ST1	-0.011111184	4.37618047	-0.135802194	0.892084	0.90554	-7.3332822
ADM	0.020657583	5.11808636	0.135185647	0.892571	0.90597	-7.33336571
ZPLD1	0.008365426	0.21903018	0.13512909	0.892616	0.90597	-7.33337335
PDE1C	0.01351688	1.36725855	0.135018886	0.892703	0.906	-7.33338823
ATG14	-0.008447762	4.15451494	-0.134359862	0.893223	0.90648	-7.33347697
FNIP1	0.0101115	3.58821028	0.132573143	0.894635	0.90786	-7.33371537
MIB1	-0.012548245	4.46400167	-0.132327662	0.894829	0.908	-7.33374787
BCAR3	-0.013042774	3.8621293	-0.131740135	0.895293	0.90842	-7.33382542
DSCAM	0.000950103	0.03360004	0.131466509	0.895509	0.90858	-7.33386142
KLK14	-0.007302943	0.44999047	-0.130991339	0.895885	0.90891	-7.33392376
SULT1B1	-0.00566537	0.50619053	-0.129870184	0.896771	0.90975	-7.33406995

ZMYND8	-0.009186913	4.26008435	-0.128656641	0.89773	0.91067	-7.33422677
UBE4A	0.010924454	3.8546627	0.127593195	0.898571	0.91147	-7.33436299
KCNV2	-0.001349414	0.07845712	-0.126305277	0.899589	0.91245	-7.33452644
MYLK3	0.003622646	0.08676165	0.12591889	0.899895	0.91261	-7.33457516
CALML3	-0.007909293	0.18810092	-0.125895574	0.899913	0.91261	-7.33457809
ADH6	-0.00122549	0.08971546	-0.125861968	0.89994	0.91261	-7.33458232
DLX3	0.017405858	2.79526243	0.125832407	0.899963	0.91261	-7.33458604
SHISA8	0.003870669	0.13087006	0.125619497	0.900132	0.91272	-7.3346128
ZNF395	-0.009898742	4.81657031	-0.125329838	0.900361	0.9129	-7.33464913
EIF2S3B	0.006289961	0.47962178	0.124375743	0.901115	0.91361	-7.33476822
KLF7	-0.010548355	2.97553944	-0.123810551	0.901562	0.91401	-7.33483834
MYBBP1	-0.007085833	4.77057357	-0.123161652	0.902076	0.91448	-7.33491845
C1QTNF	-0.010908566	0.62710071	-0.12275964	0.902394	0.91474	-7.33496786
FOXO3	0.00938845	4.61430373	0.122465468	0.902626	0.91493	-7.33500392
ZCCHC2	-0.010432833	4.27209061	-0.122184281	0.902849	0.9151	-7.33503831
PENK	0.016233529	0.40912904	0.122042054	0.902961	0.91516	-7.33505567
ANKRD1	0.008150951	5.25976571	0.120490271	0.904189	0.91635	-7.3352438
TNNI3	-0.011246202	0.39162374	-0.119904809	0.904653	0.91676	-7.33531415
FAM153A	0.008445211	0.51715221	0.119708369	0.904808	0.91686	-7.33533768
CRB2	0.007820635	0.36449543	0.11861069	0.905677	0.91769	-7.33546844
MOCOS	-0.009361232	1.27918413	-0.118362204	0.905873	0.91783	-7.33549788
ATF3	-0.028317584	5.40342956	-0.118217851	0.905988	0.91789	-7.33551495
ARPIN	0.00800948	4.24609185	0.118147751	0.906043	0.91789	-7.33552323
CORO7-I	-0.000896943	0.05429753	-0.117302622	0.906712	0.91852	-7.33562269
OSBPL5	0.007579968	3.55093035	0.115703191	0.907978	0.91975	-7.33580898
PLCXD3	-0.008477986	0.73281421	-0.114038141	0.909297	0.92103	-7.3360002
CYP4A11	0.001600049	0.06963108	0.113711624	0.909555	0.92123	-7.33603737
RAB33B	0.007385146	2.97450814	0.1114846	0.911319	0.92296	-7.33628806
DOCK7	0.009448949	2.85426358	0.110519631	0.912084	0.92368	-7.33639514
CNGB1	0.002414103	0.13298522	0.110286165	0.912269	0.92382	-7.33642091
SPAG6	-0.006879974	0.23340377	-0.109927658	0.912553	0.92405	-7.33646037
CDCA8	-0.007196106	1.92277351	-0.10955824	0.912845	0.92429	-7.3365009
RPEL1	0.000884536	0.05181114	0.109340225	0.913018	0.92441	-7.33652476
KCNQ5	0.014449429	2.21606933	0.10855142	0.913643	0.92494	-7.33661067
STX19	-0.005104038	0.48541686	-0.108499211	0.913685	0.92494	-7.33661634
PMFBP1	0.002705569	0.26379961	0.108474563	0.913704	0.92494	-7.33661901
GSTM5	0.007727263	0.66141916	0.108334	0.913816	0.92498	-7.33663424
OVCH2	0.001551173	0.07407342	0.108289291	0.913851	0.92498	-7.33663909
TSC22D1	0.019093312	7.40822323	0.107520134	0.91446	0.92554	-7.33672206
WDR81	-0.007541306	3.72213245	-0.107192349	0.91472	0.92575	-7.33675724

RPL28	0.008211003	8.31782566	0.107014086	0.914862	0.92583	-7.33677633
TPD52L1	0.012482917	5.58440047	0.106418585	0.915334	0.92626	-7.33683986
DGKK	0.001045813	0.0357929	0.104982209	0.916472	0.92732	-7.33699165
RNPEPL	-0.005782236	4.89766056	-0.104947677	0.916499	0.92732	-7.33699527
HSPH1	0.009644266	5.17783251	0.103568425	0.917593	0.92838	-7.33713903
DENND4	-0.005625077	4.03048624	-0.103358134	0.91776	0.92849	-7.33716078
FUOM	-0.012949944	3.63449766	-0.103034362	0.918016	0.92869	-7.33719418
HS3ST6	-0.007650632	0.3994186	-0.10278661	0.918213	0.92884	-7.33721967
CELF6	0.000997307	0.06147609	0.102705681	0.918277	0.92885	-7.33722799
CORO6	0.023417951	4.39612463	0.102064791	0.918785	0.9293	-7.33729359
RDH16	0.00260547	0.34703763	0.101795357	0.918999	0.92947	-7.33732104
TBC1D3(	-0.000905945	0.05273174	-0.101554926	0.919189	0.9296	-7.33734548
CCDC16	-0.000505535	0.01075558	-0.10135455	0.919348	0.92971	-7.33736581
RPS6KA	-0.009568206	6.21774702	-0.101076085	0.919569	0.92988	-7.33739398
ZBTB10	-0.00736228	3.59567865	-0.10092032	0.919693	0.92995	-7.33740971
OXGR1	0.003080346	0.23975898	0.099716395	0.920647	0.93086	-7.33753046
HES4	-0.013134465	3.8417206	-0.098644572	0.921498	0.93166	-7.33763673
PNPLA2	-0.007464803	6.10929136	-0.098478442	0.921629	0.93174	-7.3376531
PHTF2	-0.005967461	2.73160824	-0.098190046	0.921858	0.93191	-7.33768145
WASHC	0.007340628	4.14197651	0.098107946	0.921923	0.93192	-7.33768951
CDK15	0.001464379	0.10244352	0.097052768	0.92276	0.93271	-7.33779245
QSER1	-0.00779383	3.46844467	-0.095612615	0.923903	0.93381	-7.33793115
GPR75	-0.008275011	1.73469248	-0.095157667	0.924264	0.93412	-7.33797453
UGT2B7	0.004766749	0.17246458	0.095089214	0.924318	0.93412	-7.33798104
MCM2	0.008340491	4.23025669	0.095013626	0.924378	0.93413	-7.33798822
SLC1A3	-0.010900196	1.72974494	-0.092899657	0.926056	0.93577	-7.33818679
USH2A	-0.000502426	0.0229973	-0.092748224	0.926176	0.93583	-7.33820085
ATP10B	0.010483944	0.67794267	0.092029222	0.926747	0.93635	-7.33826726
RABGGT	0.005069309	5.13202373	0.091643634	0.927053	0.93661	-7.33830266
NFATC2I	-0.005118928	3.90871063	-0.090403108	0.928038	0.93755	-7.33841555
FAAH2	0.007562954	4.45881429	0.089953551	0.928394	0.93785	-7.33845608
RGL1	-0.008643732	3.40581394	-0.089404813	0.92883	0.93823	-7.33850528
MTAP	-0.005226985	2.8280135	-0.088466013	0.929575	0.93888	-7.33858875
NPIPB15	0.013983705	2.13632594	0.08845717	0.929582	0.93888	-7.33858953
SLC4A1(	-0.002078511	0.07627228	-0.088323421	0.929689	0.93893	-7.33860135
GGN	-0.005991346	1.73067005	-0.088012123	0.929936	0.93913	-7.33862879
AOC1	0.015442604	1.50632746	0.087625631	0.930243	0.93934	-7.33866272
CMPK2	-0.007042296	2.4595289	-0.087602452	0.930261	0.93934	-7.33866475
SHE	0.010871255	4.52233025	0.086983085	0.930753	0.93978	-7.3387188
LPL	-0.015071762	4.20945215	-0.085947703	0.931575	0.94056	-7.33880831

GPR179	-0.000641941	0.04807215	-0.085479851	0.931947	0.94088	-7.3388484
GP1BA	0.006159474	1.12613891	0.084114988	0.933031	0.94186	-7.33896411
SLC47A1	0.009401563	4.04450607	0.084113416	0.933032	0.94186	-7.33896424
EP300	0.008641418	5.14612671	0.084006705	0.933117	0.94189	-7.33897321
PCDHGA	0.008139572	1.12570962	0.083028496	0.933894	0.94262	-7.33905488
COL6A1	0.010702552	6.13378674	0.082949948	0.933956	0.94263	-7.3390614
TMIGD3	0.007825385	1.58948819	0.082648842	0.934195	0.94281	-7.33908632
LRP5L	0.010062691	3.65227471	0.082444374	0.934358	0.94292	-7.3391032
TSC22D2	-0.005575078	3.1435755	-0.080556554	0.935858	0.94438	-7.33925702
RGMA	0.003750429	0.69251851	0.080267716	0.936087	0.94455	-7.33928024
TMTC1	0.006456703	1.50476116	0.080177725	0.936158	0.94457	-7.33928746
FOXC2	0.006632511	1.46774733	0.079867949	0.936405	0.94476	-7.33931224
MRPS25	-0.005329665	5.32582177	-0.078732019	0.937307	0.94561	-7.3394023
PCDH10	-0.003799196	0.27867798	-0.077572733	0.938228	0.94649	-7.33949288
PCDHA5	0.00120104	0.08567696	0.077110996	0.938595	0.94675	-7.33952858
ADSS	0.006505603	5.89311721	0.077110076	0.938596	0.94675	-7.33952865
LY6G5B	0.005651428	1.23314993	0.076217655	0.939305	0.94738	-7.33959705
WIF1	-0.007519928	0.23935755	-0.076177819	0.939337	0.94738	-7.33960008
ABCB4	0.002419777	0.49928144	0.075414897	0.939943	0.94794	-7.3396579
RAPGEF	-0.00610701	2.00304801	-0.074961953	0.940303	0.94824	-7.33969194
DMRT2	-0.001895426	0.15765534	-0.074272734	0.940851	0.94874	-7.33974336
ZNF23	0.002923696	0.63081467	0.073600716	0.941386	0.94922	-7.33979303
LIPA	-0.007419704	5.1797822	-0.073278581	0.941642	0.94942	-7.33981669
ZNF695	-0.001125286	0.1700999	-0.073113839	0.941773	0.94947	-7.33982874
UHMK1	0.005363414	5.00256043	0.073074419	0.941804	0.94947	-7.33983162
GADD45	0.009797451	6.25172567	0.072961622	0.941894	0.94951	-7.33983986
NUDT17	-0.007516409	3.34986517	-0.072737301	0.942072	0.94963	-7.33985619
SHROOM	-0.007919688	3.30998685	-0.072476534	0.942279	0.94978	-7.33987512
SOX9	0.008408487	5.41194745	0.072302685	0.942417	0.94987	-7.3398877
CAPN14	-0.000737765	0.07087768	-0.072102391	0.942577	0.94997	-7.33990216
HGF	-0.004505835	0.81414054	-0.071836898	0.942788	0.95013	-7.33992127
LYPD2	0.002964819	0.17835082	0.071284791	0.943227	0.95045	-7.33996077
PLXND1	0.006727812	5.73308821	0.071222301	0.943276	0.95045	-7.33996522
SLCO4C	0.01170275	2.04463793	0.071216869	0.943281	0.95045	-7.3399656
ANKRD2	0.004504897	0.34687779	0.071010349	0.943445	0.95056	-7.33998029
FAM110A	0.005945656	4.17365135	0.070799352	0.943613	0.95068	-7.33999524
RAB40A	-0.002820436	0.66429294	-0.070597926	0.943773	0.95078	-7.34000948
TMEM89	-0.001229267	0.10196847	-0.069571568	0.944589	0.95155	-7.3400814
FGFRL1	-0.006192135	6.77462806	-0.06939846	0.944727	0.95163	-7.34009342
SLC38A2	-0.005684465	5.55162198	-0.069172498	0.944906	0.95175	-7.34010907

YPEL3	0.005694716	5.74507209	0.068209195	0.945672	0.95247	-7.34017523
NEK2	-0.004811169	0.87748883	-0.068066046	0.945786	0.95253	-7.34018498
B3GAT2	0.001570774	0.33977268	0.067370131	0.94634	0.95303	-7.34023209
PPP4R4	0.002904424	0.37279365	0.066998566	0.946635	0.95327	-7.34025705
MBNL1	-0.004282079	4.91597191	-0.065622599	0.947729	0.95431	-7.34034827
NBPF14	0.003341038	1.36146509	0.064757977	0.948417	0.95494	-7.34040462
WNT7B	-0.006181356	1.61378108	-0.064695678	0.948467	0.95494	-7.34040865
NLRP4	0.001414807	0.05887135	0.062198734	0.950453	0.95688	-7.34056704
TTN	0.002632311	0.38662483	0.061744067	0.950815	0.95719	-7.34059521
HPR	0.000982053	0.06488277	0.061422241	0.951071	0.95739	-7.34061502
R3HDM2	-0.003328488	4.6103397	-0.06127314	0.951189	0.95746	-7.34062417
GARS	0.004342864	6.13134239	0.061117396	0.951313	0.95752	-7.3406337
EPB41L1	-0.004476661	4.90908892	-0.059529219	0.952577	0.95874	-7.34072948
SLC12A5	-0.003845886	0.58672617	-0.059219421	0.952824	0.95893	-7.34074787
LRRD1	-0.000549436	0.07410379	-0.058835334	0.953129	0.95918	-7.34077054
PCDHB1	-0.007935021	1.57331521	-0.058432117	0.95345	0.95945	-7.34079417
NDC80	0.00405982	1.38850281	0.057413686	0.95426	0.9602	-7.34085315
CARM1	0.004366194	4.1447105	0.057234488	0.954403	0.96027	-7.34086342
PRG2	-0.000906425	0.091422	-0.05715781	0.954464	0.96027	-7.3408678
EOGT	0.003686775	3.19865761	0.05711601	0.954497	0.96027	-7.34087019
TULP3	0.004417117	4.46530002	0.056802884	0.954746	0.96047	-7.34088802
PTK7	0.006738156	5.18695525	0.056448292	0.955029	0.96069	-7.3409081
OXER1	-0.004833024	1.47989799	-0.056299842	0.955147	0.96075	-7.34091646
UBAP2L	0.003687519	5.80803621	0.056198621	0.955227	0.96078	-7.34092216
CTDSPL	0.004674489	4.00343183	0.05596995	0.955409	0.96089	-7.34093498
SRGAP1	-0.003569333	2.00728688	-0.055915999	0.955452	0.96089	-7.34093799
SEMA4A	0.006191226	4.73403414	0.055471242	0.955806	0.96119	-7.34096276
FAM83B	-0.006327932	1.96416465	-0.055355305	0.955899	0.96123	-7.34096918
TEX12	-0.000957005	0.17009762	-0.054644338	0.956464	0.96174	-7.34100828
CCN3	0.005507918	3.90826774	0.05360455	0.957292	0.96251	-7.34106454
MPRIP	0.003986774	5.21813547	0.052087148	0.9585	0.96367	-7.34114471
ART3	-0.000713128	0.08012963	-0.051872056	0.958671	0.96379	-7.34115589
ALCAM	0.005591486	6.065273	0.050842101	0.959491	0.96455	-7.34120877
SLC35E2	-0.00334918	1.41272921	-0.049764155	0.960349	0.96536	-7.34126298
HLA-E	0.003894403	9.50070556	0.049442678	0.960605	0.96556	-7.34127893
WDR62	-0.001885166	0.71727826	-0.049132878	0.960852	0.96575	-7.34129419
ABCA1	0.005529287	3.71058662	0.048772672	0.961138	0.96598	-7.34131182
JAK1	-0.004078641	6.30167867	-0.048222484	0.961576	0.96631	-7.3413385
THBS3	-0.003616122	3.88258188	-0.048221731	0.961577	0.96631	-7.34133853
SGCG	0.002877848	0.25770895	0.047777658	0.961931	0.9666	-7.34135984

USP27X	0.003763401	3.15836734	0.0469769	0.962568	0.96719	-7.34139777
PCDHGA	0.005464898	1.50359437	0.046610039	0.96286	0.96742	-7.34141494
EMILIN3	0.005355956	1.0532005	0.045924042	0.963407	0.96791	-7.34144667
GAS7	-0.00704951	2.24847421	-0.045408566	0.963817	0.96827	-7.3414702
KHSRP	-0.002786548	6.48967038	-0.044990295	0.96415	0.96855	-7.3414891
KIR3DL1	-0.000784225	0.08964317	-0.044300462	0.964699	0.96904	-7.34151989
KCNJ9	-0.000389081	0.05723236	-0.044155832	0.964815	0.9691	-7.34152629
MANEAL	0.00425804	3.92419708	0.043555534	0.965293	0.96952	-7.34155261
SLC6A8	-0.004162989	2.66286243	-0.043025112	0.965715	0.96989	-7.34157557
FAM83G	0.003743943	3.79523283	0.042684741	0.965986	0.9701	-7.34159015
4-Mar	-0.002581287	0.47905159	-0.042519648	0.966118	0.97018	-7.34159718
GTSF1	-0.002873442	0.33831836	-0.040841806	0.967454	0.97146	-7.3416671
CADM4	-0.003754425	4.15008394	-0.039528036	0.9685	0.97246	-7.34171988
NFATC2	0.003120908	3.1237213	0.039090833	0.968848	0.97275	-7.34173706
CARNS1	-0.005338095	4.68528155	-0.038756276	0.969115	0.97296	-7.34175008
TOX2	0.003740452	2.65120669	0.037819375	0.969861	0.97365	-7.34178595
IL6ST	-0.003320324	6.50977129	-0.03691639	0.97058	0.97431	-7.34181968
HMMR	-0.002349714	1.11818586	-0.034969069	0.972132	0.97581	-7.34188966
NANOG	0.000192546	0.01930968	0.03476527	0.972294	0.97592	-7.34189676
PCDHA3	0.002133473	0.47780707	0.034682807	0.97236	0.97593	-7.34189962
GPC2	-0.001408137	0.36789063	-0.034527053	0.972484	0.97595	-7.34190501
DYRK2	-0.002442043	3.38541547	-0.03451484	0.972493	0.97595	-7.34190543
C11orf24	-0.001893246	4.41650328	-0.034284288	0.972677	0.97607	-7.34191336
XKR7	0.000778355	0.09641335	0.033777146	0.973081	0.97642	-7.34193062
TMSB4Y	-0.005066682	0.74321691	-0.03325719	0.973495	0.97678	-7.34194804
LYPD6B	-0.000902583	0.08538944	-0.031983684	0.97451	0.97774	-7.34198957
PROSER	0.002294854	2.40551346	0.031607917	0.974809	0.97798	-7.34200152
FXYD1	-0.001772344	0.57299406	-0.031319333	0.975039	0.97815	-7.3420106
WDR49	0.000340513	0.05010334	0.030699136	0.975533	0.97859	-7.34202983
HOXD8	-0.002534779	1.66350276	-0.030587928	0.975622	0.97862	-7.34203323
ACSM2B	0.000231166	0.02162989	0.030338745	0.975821	0.97876	-7.34204082
SLC44A5	0.003735461	0.78287464	0.030129815	0.975987	0.97887	-7.34204714
HHIP	-0.000737016	0.09150872	-0.029813067	0.976239	0.97907	-7.34205663
MAL2	-0.002772582	7.61631803	-0.029480401	0.976504	0.97928	-7.34206649
NAT8	0.00133134	0.25846414	0.029233763	0.976701	0.97941	-7.34207373
GON4L	-0.002120893	3.35640367	-0.028827246	0.977025	0.97968	-7.34208553
GGT5	0.003643741	5.21528887	0.02865111	0.977165	0.97976	-7.34209059
TBC1D3I	-0.000383535	0.09981902	-0.027508441	0.978076	0.98062	-7.34212267
NCOA1	-0.00223099	3.78145201	-0.02728044	0.978257	0.98074	-7.34212891
CLIP4	-0.002650582	5.1488814	-0.026718557	0.978705	0.98113	-7.34214408

TCN2	0.002624365	6.10023982	0.025916354	0.979344	0.98172	-7.34216518
SCAI	-0.001556506	2.0928529	-0.024689202	0.980322	0.98258	-7.34219623
UBN1	-0.001885771	4.22823284	-0.024582916	0.980407	0.98258	-7.34219885
C19orf84	0.00139175	0.35422733	0.02458109	0.980408	0.98258	-7.34219889
FOXN2	0.001866892	2.99056699	0.024551151	0.980432	0.98258	-7.34219963
CRYBA1	0.000381205	0.11918464	0.022424721	0.982127	0.98422	-7.34224956
RAPSN	-0.000909197	0.34194794	-0.022041241	0.982432	0.98446	-7.34225808
EME1	-0.000883035	0.7241638	-0.021669368	0.982729	0.9847	-7.34226621
GPR88	-0.000682549	0.1919734	-0.021351317	0.982982	0.9849	-7.34227305
COL2A1	0.000303168	0.07496207	0.020864237	0.98337	0.98523	-7.34228333
KIAA184	-0.000940815	1.80588452	-0.019668295	0.984323	0.98611	-7.34230756
PRKD3	-0.001624757	3.53193425	-0.019614047	0.984367	0.98611	-7.34230863
TIA1	-0.00203233	5.12280328	-0.019424564	0.984518	0.9862	-7.34231232
NPIPB13	0.000713129	0.15224439	0.019268686	0.984642	0.98627	-7.34231534
KCNK1	-0.0016144	4.31675016	-0.018356028	0.985369	0.98694	-7.3423325
ADM5	-0.001062685	0.90641994	-0.017834731	0.985785	0.9873	-7.34234194
SPINK6	0.000692822	0.28340782	0.017647448	0.985934	0.98739	-7.34234526
AJUBA	0.002055694	4.26875384	0.017167581	0.986316	0.98771	-7.34235361
TCERG1	0.004008491	5.05767119	0.016865578	0.986557	0.9879	-7.34235875
SLC5A2	-0.001015024	0.8192702	-0.016697226	0.986691	0.98797	-7.34236157
BIRC5	-0.001534888	1.44445208	-0.016509422	0.986841	0.98806	-7.34236469
SP1	0.000948628	5.12059002	0.014493902	0.988447	0.98961	-7.34239593
MYO18B	-0.000480984	0.05741888	-0.014069786	0.988785	0.98989	-7.34240198
NR2C2AI	0.000904814	4.82725957	0.013824702	0.988981	0.99003	-7.3424054
ZNF836	-0.000650603	2.52112123	-0.012901476	0.989716	0.99071	-7.34241773
STK39	-0.001024463	3.88923672	-0.012469664	0.990061	0.991	-7.34242321
ARID3A	-0.000788763	2.08750727	-0.011870189	0.990538	0.99142	-7.3424305
FRRS1L	-0.000599624	0.3299358	-0.011492339	0.99084	0.99166	-7.34243492
CDHR5	0.000174464	0.13511477	0.010319536	0.991774	0.99254	-7.3424477
KRT18	-0.000810249	8.96127855	-0.009574762	0.992368	0.99302	-7.34245511
OOEP	0.000745052	0.40359684	0.009570237	0.992372	0.99302	-7.34245515
SIGLEC8	0.000831634	0.7906148	0.009292607	0.992593	0.99317	-7.34245777
ITGB3	0.00150426	4.70189716	0.009225852	0.992646	0.99317	-7.34245839
CD8A	-0.00129282	2.75603391	-0.00846088	0.993256	0.99371	-7.34246515
JAK2	0.000615046	2.5898583	0.008400896	0.993304	0.99371	-7.34246566
FAM114A	0.000576704	4.75767219	0.006914053	0.994489	0.99484	-7.34247704
TAS2R4	-0.00033683	0.76188028	-0.006666909	0.994686	0.99498	-7.34247872
PPP1R27	-0.000227473	0.13002948	-0.00522952	0.995832	0.99607	-7.34248727
PLEKHM	0.000203116	6.10737838	0.003612111	0.997121	0.9973	-7.34249442
UNC45B	7.07E-05	0.10002459	0.002980583	0.997624	0.99774	-7.3424965

SERPINE	-0.000292185	3.56891386	-0.001770678	0.998589	0.99865	-7.34249937
IKZF2	3.40E-05	3.72471777	0.000275177	0.999781	0.99978	-7.3425009
